# Isothermal microcalorimetry for thermal viable count of microorganisms in pure cultures and stabilized formulations

**DOI:** 10.1186/s12866-019-1432-8

**Published:** 2019-03-21

**Authors:** Johanna Nykyri, Anke M. Herrmann, Sebastian Håkansson

**Affiliations:** 1 Department of Molecular Sciences, Swedish University of Agricultural Sciences, P.O. Box 7015, SE-75007 Uppsala, Sweden; 2 Department of Soil and Environment, Swedish University of Agricultural Sciences, P.O. Box 7014, SE-75007 Uppsala, Sweden

**Keywords:** Viable count, Isothermal microcalorimetry, Aerophilic, Microbial products, Plant seed coating, Biological control, Plant protection

## Abstract

**Background:**

Quantification of viable microorganisms is an important step in microbiological research as well as in microbial product formulation to develop biological control products or probiotics. Often, the efficiency of the resulting product is dependent on the microbial cell density and their viability, which may decrease over time. Commonly, the number of viable cells is determined by serial dilution and plating techniques or flow cytometry. In 2017, we developed a mathematical model for isothermal microcalorimetry (IMC) data analysis and showed that the new method allows for a more rapid quantification of viable fresh and freeze-dried anaerobic *Lactobacillus reuteri* cells than traditional viable count methods.

**Results:**

This study developed the new method further by applying it to well-known aerophilic plant-beneficial microbial species (*Pseudomonas brassicacearum*, *Bacillus amyloliquefaciens subsp. plantarum* and *Clonostachys rosea*) used in biological control products. We utilized IMC to quantify viable cells in microbial pure cultures as well as when coated onto wheat seeds. The results from this study confirmed that thermal viable count methods are more rapid and sensitive than traditional viable count techniques. Most interestingly, a thermal viable count method was able to quantify microbes coated on seeds despite the presence of the natural microbiota of the seeds. Our results also showed that, in contrast to plating techniques for which clustered cells skew the results, IMC does not require single cells for accurate viable counts.

**Conclusions:**

Thermal viable count methods are novel methods for the rapid quantification of divergent bacterial and fungal species and enhance the speed, sensitivity, and accuracy of routine viable counts of pure cultures and controlled microbiomes such as plant seed coatings.

**Electronic supplementary material:**

The online version of this article (10.1186/s12866-019-1432-8) contains supplementary material, which is available to authorized users.

## Background

Microbes are present in all kinds of environments, both natural and man-made, and they are utilized in multiple industrial processes or products. When considering microbial applications, it is of utmost interest whether the microbes are viable and how to best quantify them [1–4]. For example, in the case of microbial plant protection products [1] or probiotics [3], viable cell concentrations have dramatic effects on the efficacy of the products when they are applied. In certain products, such as coated seeds, the natural microbiota generate background in viable count processes. The stability of products and long shelf lives are of interest for the entire supply chain, from manufacturers to end-users. To reach these goals more efficiently, new rapid and accurate quality control systems for viable counts or viability assessments would be an advantage.

Currently, viability assessments or viable counts of microbes rely mainly on dilution series and plating techniques, flow cytometry and different microscopy techniques [1, 2, 4]. The method of serial dilutions and plating is still considered the gold standard. The results of flow cytometry and microscopy techniques are often difficult to interpret because cells could respond oddly to the staining process, giving false positives and negatives [1–3]. However, serial dilutions and plating techniques also have limitations: (i) They require a relatively long time span (up to several days) to obtain results; (ii) the microbes need to be cultivable, but 99% of microbes are non-cultivable; (iii) these techniques have poor automation possibilities; and (iv) they do not differentiate whether cells are single or attached together to form a larger unit, thus underestimating the viable count [1–3]. Nucleic acid-based methods, such as quantitative PCR, have been routinely used to quantify and identify microbes. However, these methods do not generally differentiate whether microbes are viable or not, despite the efforts to develop these methods towards viable counting [1–3].

In addition to these traditional methods, isothermal microcalorimetry (IMC) has been proposed to have potential in viable count or viability assessment [1, 2, 5, 6]. IMC measures the amount of heat released from a sample by any physical, biological or chemical process [6]. In microbiology and related fields, IMC has been commonly applied in clinical applications to detect infection or to diagnose diseases, and in environmental sciences, IMC is used to measure metabolic activity in different types of soil samples [6]. The vast majority of IMC applications have utilized IMC methodology for purposes other than quantitative or viable counting. However, IMC has been utilized to estimate soil microbial biomass relative to its microbial activity, focusing on general microbial activity in divergent soils and the carbon cycle for land management [7–10].

A few articles describe findings that have paved the way for fine-tuned quantification of viable microbial cells by IMC. In 2002, Critter and colleagues [11] showed the correlation between viable bacterial and fungal counts and IMC-measured heat production. Bonkat and colleagues in 2012 [12] showed for the first time that there is a link between growth rate and IMC-measured cumulative heat and applied the method to successfully detect common facultative anaerobic urinary tract pathogens in urine. Maskow and colleagues in 2012 [13] took the analysis a step further by presenting a theoretical model to calculate detection times for visual and IMC-based detection of a population originating from a single bacterial cell. They tested the theory by applying it to aerobically and anaerobically growing *Escherichia coli* and aerobically growing *Pseudomonas putida*, finding that the time when the microbial heat production was detected by IMC was a function of *initial cell density*. They also showed how the volume of air in a closed IMC vial should be considered in future applications. Isothermal titration calorimetry has also been used to study the life cycle of *Rhodobacter sphaeroides* [14], and that study in 2013 found a correlation between cell concentration at log phase and the measured thermal power value.

In 2017, we proposed a novel IMC viability assessment method, from sample preparation to statistical analysis, to quantify viable bacterial cells based on the thermal power detection time point for certain cell concentrations of an *initial cell batch* [15]. This novel method developed further the earlier calculations of relations between microbial growth and the thermal power [12, 13]. We applied this novel method to quantify viable formulated and freeze-dried microbes; specifically, we utilized IMC to quantify viable fresh as well as formulated and freeze-dried anaerobic gram-positive *Lactobacillus reuteri* cells [15].

In the current work, our aims were to validate the mathematical model of IMC viability assessment methods [15] and to develop methodology further to quantify a gram-negative bacterium (*Pseudomonas brassicacearum*), gram-positive bacterium (*Bacillus amyloliquefaciens subsp. plantarum*) and fungus (*Clonostachys rosea*) in pure cultures or coated on seeds. We also re-named these methods “thermal viable count methods” to be more precise. These aerophilic plant beneficial microbes are used as biocontrol agents in microbial plant protection products. First, we show how the headspace air volume above culturing media in IMC vials affects the growth of these microbes. Then, we display how initial cell densities affects the detection time of these different microbes by IMC and how thermal viable count methods are applied. Finally, we discuss how the IMC thermal power detection time point is a valid parameter to generate standard curves in thermal viable count methods. To our knowledge, this is the first time that thermal viable count methods are applied to these bacterial and fungal species and the corresponding standard curves are generated; most importantly, it is the first time that they are applied to bacteria coated onto seeds or, generally, to any system other than a pure microbial culture.

## Results

### Headspace air volume in IMC vials affects the microbial thermal power and final cell density

**Fig. 1:**
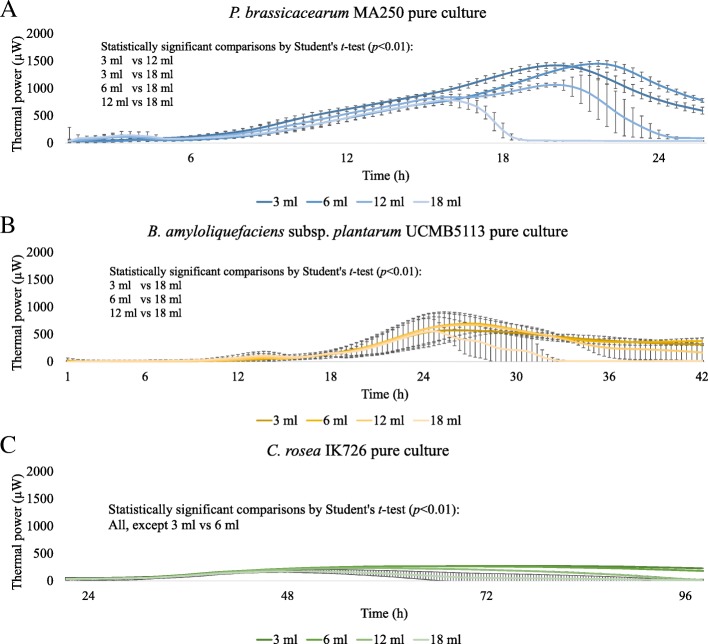
Isothermal microcalorimetry thermal power (μW) curves of microbes in different culturing volumes (3, 6, 12 and 18 ml). **a** *Pseudomonas brassicacearum* MA250 in TSB, 25 °C. **b** *Bacillus amyloliquefaciens subsp. plantarum* UCMB5113 in LB, 25 °C. **c** *Clonostachys rosea* IK726 in PDB, 25 °C. The figures for *Pseudomonas* and *Bacillus* represent the averages and standard deviations of three independent experiments with 3–6 replicates per treatment. The figure for *C. rosea* IK726 represents the averages and standard deviations of six replicates per treatment

To find a protocol to utilize IMC to study aerophilic microbial strains, we first investigated the effect of standard IMC running conditions on microbial growth and thermal power. Standard running conditions in IMC may create an environment where the gas exchange is limited and therefore limits the growth of aerophilic microbes. We measured the thermal power (μW) of *P. brassicacearum* MA250, *B. amyloliquefaciens* subsp. *plantarum* UCMB5113 and *C. rosea* IK726 in different volumes (3 ml, 6 ml, 12 ml and 18 ml) of culturing media (tryptic soy broth (TSB), Luria broth (LB), or potato dextrose broth (PDB), respectively) in 20 ml vials. Different volumes of media created different ratios of culturing media volume to headspace air volume in the capped vials. All the tested microbial strains gave their strongest thermal power output and grew to their highest densities (cfu/ml) or greatest biomass (mg/ml) when there was a 3 ml or 6 ml culture in an IMC vial (Fig. 1a, b, and c, Additional file 1). There were no statistically significant differences between the properties of 3 ml and 6 ml cultures for each microbial strain. When we increased the culturing volume to 12 ml, the thermal power output and cell concentration or biomass were lower for *Pseudomonas* (*p* < 0.01) and *Clonostachys* (*p* < 0.01) than they were in cultures of 3 ml and 6 ml (Fig. 1a and c, Additional file 1). When we increased the culturing volume to 18 ml, the thermal power output and cell concentration or biomass were considerably lower in all of the studied strains (*p* < 0.01) than they were in cultures of 3 ml, 6 ml and 12 ml (Fig. 1a, b, and c, Additional file 1). In conclusion, the culturing volumes had an effect on the thermal power curves and cell density, and in larger volumes, the cultures reached stationary phase earlier.

### Thermal viable count methods are able to quantify microbes in pure cultures and coated on seeds

**Fig. 2:**
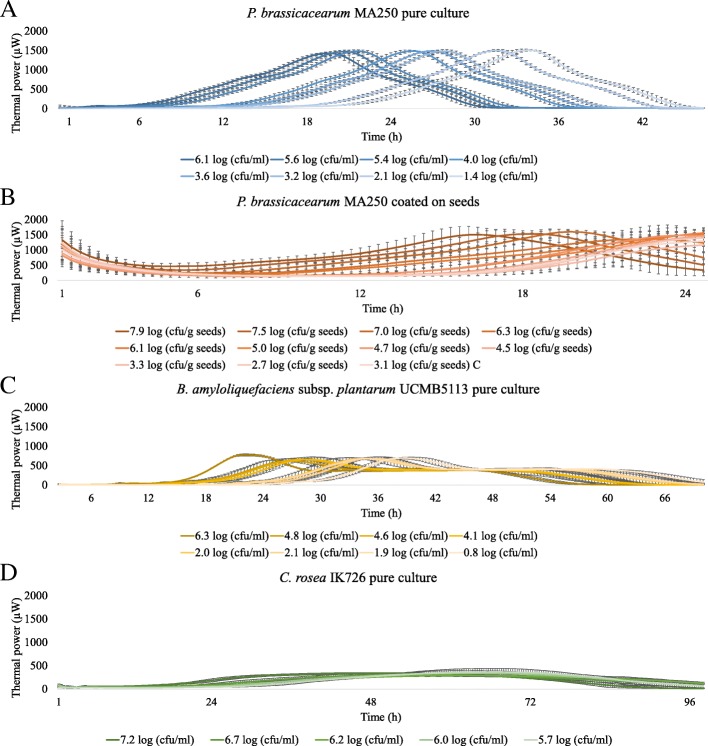
Isothermal microcalorimetry thermal power curves (μW) and respective initial microbial cell densities for each curve (log (cfu/ml)). The pure culture volume was 6 ml, and the seed sample size was 3 g, supplemented with 5.5 ml of media. **a** *Pseudomonas brassicacearum* MA250 pure culture in TSB, 25 °C. **b** *P. brassicacearum* MA250 coated on wheat seeds, 25 °C. C = surface sterilization control (3.1 log (cfu/ml)). **c** *Bacillus amyloliquefaciens subsp. plantarum* UCMB5113 in LB, 25 °C. **d** *Clonostachys rosea* IK726 in PDB, 25 °C. Each strain displayed distinct and repeatable thermal power curves. The figures represent the averages and standard deviations of 2–3 independent experiments with 3–6 replicates each

**Fig. 3:**
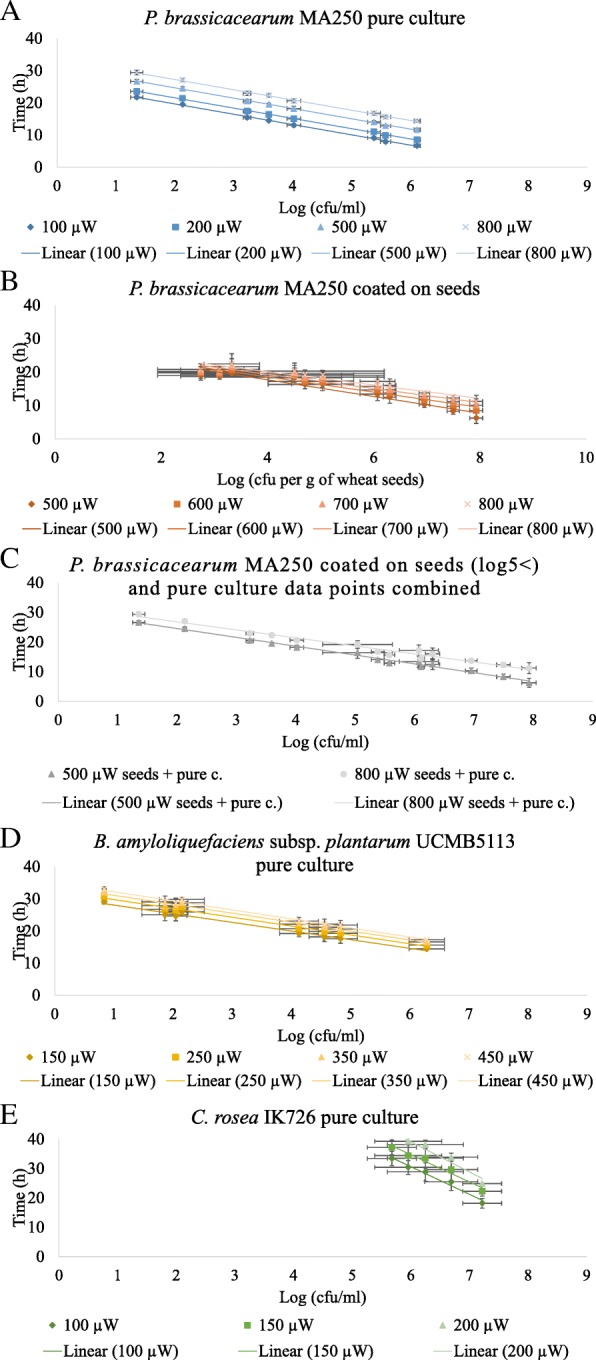
Regression analyses of cell concentrations (log (cfu/ml)) and detection times (h) at certain thermal power values to determine the correlation and resolution of isothermal microcalorimetry thermal power curves for the quantification of microbial densities. **a** The *Pseudomonas brassicacearum* MA250 resolution in pure culture was ~ 0.2–0.4 log (cfu/ml). **b** The *P. brassicacearum* MA250 resolution after seed coating was ~ 0.5–1 log (cfu/g seed). **c** Different thermal power (500 μW and 800 μW) time points of *P. brassicacearum* MA250 pure cultured samples and coated seed samples fitted into the same regression curve despite of the differences in the resolution per treatment (see Fig. 3a and b). **d** The *Bacillus amyloliquefaciens subsp. plantarum* UCMB5113 strain’s resolution in pure culture was ~ 0.6–0.8 log (cfu/ml). **e** The *Clonostachys rosea* IK726 resolution in pure culture was ~ 1–1.5 log (cfu/ml). The figures represent the averages and standard deviations of 2–3 independent experiments with 3–6 replicates each

To study if IMC could be used to quantify viable microbial cells, we studied dilution series of *P. brassicacearum* MA250, *B. amyloliquefaciens* subsp. *plantarum* UCMB5113 spores and *C. rosea* IK726 conidia by measuring the thermal power (μW) of each dilution by IMC. Based on the results with pure cultures, we decided to prepare a dilution series of *P. brassicacearum* MA250 coated onto wheat seeds and measure the thermal power (μW) of coated seed batches by IMC. IMC was able to differentiate initial cell densities of *P. brassicacearum* MA250 in pure cultures with a resolution as low as ~ 0.2–0.4 log (cfu/ml) (Figs. 2). However, in the case of *P*. *brassicacearum* MA250-coated seeds, IMC differentiated initial microbial cell densities with a resolution from ~ 0.5–1 log (cfu/g seed) (Figs. 2b and 3b and c). IMC differentiated pure cultured *B. amyloliquefaciens* subsp. *plantarum* UCMB5113 initial cell densities with an ~ 0.6–0.8 log (cfu/ml) resolution and *C. rosea* IK726 with an ~ 1–1.5 log (cfu/ml) resolution (Figs. 2c and d and 3d and e). Regression analyses showed that the model fits the data well (R^2^ = 0.91–1.00) when detection times were compared at certain thermal power values and cell concentrations (Fig. 3, Table 1).

**Table 1:** Detection time equations for the relationships between initial microbial cell densities (log (cfu/ml)) and detection times (isothermal microcalorimetry thermal power values) for strains *Pseudomonas brassicacearum* MA250, *Bacillus amyloliquefaciens subsp. plantarum* UCMB5113 and *Clonostachys rosea* IK726. In all strains, initial cell densities correlated with thermal power values. The results represent the averages of 2–4 independent experiments

Strain and culturing conditions	Thermal power (μW)	Detection Time Equation (h)	R^2^
*P. brassicacearum* MA250 pure culture	800	-3.12x + 33.49	1.00
	500	-3.15x + 31.02	1.00
	200	-3.12x + 27.86	1.00
	100	-3.16x + 26.06	1.00
*P. brassicacearum* MA250 coated on seeds	800	-2.01x + 28.16	0.91
	700	-2.12x + 27.83	0.92
	600	-2.28x + 27.71	0.94
	500	-2.50x + 27.69	0.93
*P. brassicacearum* MA250 Seeds 5< log (cfu/g seeds) + pure culture combined data	800	-2.71x + 32.26	0.97
	500	-2.99x + 30.55	0.99
*B. amyloliquefaciens* subsp. *plantarum* UCMB5113 pure culture	450	-2.81x + 35.01	0.99
	350	-2.78x + 33.88	0.99
	250	-2.75x + 32.53	0.99
	150	-2.69x + 30.76	0.98
*C. rosea* IK726 pure culture	200	-10.28x + 100.80	0.93
	150	-9.29x + 90.49	0.96
	100	-9.41x + 87.13	0.97

Interestingly, the seed surface sterilization control carrying residual natural microbiota, fitted in the same standard curve as samples with coated seeds when a regression analysis was made with both data sets (Fig. 3b, surface sterilization control is marked with an arrow in the figure). Based on serial dilutions and plating, the density of the natural microbiota on wheat seeds was approximately 2–3 log (cfu/g seed) after surface sterilization. To study if the model also fits the data collected from *P. brassicacearum* MA250 *pure cultures and coated seeds*, we fitted the time point data collected from both treatments’ thermal power values of 500 μW and 800 μW in the same standard curve generated by a regression analysis. To minimize the possible effect of the seeds’ natural microbiota on the quantification of MA250, we excluded data from surface sterilization controls and samples with the lowest MA250 cell density (< 5 log). The model fitted the data collected from *P. brassicacearum* MA250 pure cultures and coated seeds combined together well (Fig. 3c, Table 1). Therefore, these data of pure cultures and coated seeds support the correlation between the detection time point and cell concentration at a specific IMC thermal power value.

### The natural microbiota of seeds affects the thermal viable count method at low cell densities of coating

The natural microbiota of wheat seeds affected the quantification of *P. brassicacearum* MA250 on seeds when the initial MA250 cell concentration on seeds was below ~ 5 log (cfu/g seed), which was reflected by the increased variances in log (cfu/g seeds) in different experiments (Figs. 2b and 3b). *P. brassicacearum* MA250-specific sequence characterized amplified region polymerase chain reaction (SCAR-PCR) primers and 16S rRNA gene sequencing detected the presence of the said strain on wheat seeds at the beginning and at the end of IMC measurements. During the experiments, colony morphology and 16S rRNA gene sequencing were utilized to determine what microbial species were present on coated and surface-sterilized seeds (Additional file 2). The reciprocal best hits by BLASTN suggested that we found other *Pseudomonas* strains and *Pantoea* and *Paenibacillus* strains in samples just before and after IMC runs (Additional file 3). The natural microbiota of wheat seeds was detected mainly in samples whose seed coating cell density was low or in the surface sterilization controls, where the seeds were only surface-sterilized and not coated.

## Discussion

The viable count of microbes is commonly calculated by serial dilution and plating techniques, which are the gold standard [1, 3]. In a previous study [15], we showed how an thermal viable count method is competitive with flow cytometry due to the reliability of IMC and showed that IMC is a faster tool for quantifying anaerobic *L. reuteri* than serial dilutions and plating. Other studies have noted that IMC is a sensitive method that detects the presence of microbes earlier than most known growth or viability measuring methods [5]. In this work, we have shown that thermal viable count methods are also faster methods to quantify aerophilic bacteria and fungi than serial dilutions and plating. To obtain countable colonies by serial dilutions and plating, researchers may have to wait 24–48 h with *Pseudomonas* and *Bacillus,* over 48 h with *Clonostachys* and, generally, 1–7 days, depending on the microbial strain. After 1–2 h of preparations in the thermal viable count methods, a wide variety of cell concentrations of pure cultured *Pseudomonas* was detected within 6–21 h, of *Bacillus* within 18–28 h and of *Clonostachys* within 18–32 h (Figs. 2 and 3). Notably, we also quantified viable *Pseudomonas* cells coated on wheat seeds after 6–11 h and generated a standard curve despite the residual natural microbiota present after the surface sterilization and coating of the wheat seeds, which may skew the viable count results (Figs. 2 and 3). The natural microbiota was approximately 2–3 log (cfu/g seed), causing increased variance when the cell density of the coated *Pseudomonas* decreased below five log (cfu/g seed) (Figs. 2 and 3). However, the model fitted the data well, even when the population was a mixture of coated microbial cells and the residual natural microbiota of the wheat seeds. That the model fitted in all cases could be explained by the fact that the majority of the microbes found from the samples were other *Pseudomonas* (Additional file 3) or had otherwise similar features, thus producing similar thermal power profiles under the conditions used. The data validated our previously justified model for the thermal viable count method (viability assessment) of *L. reuteri* [15]. Using the detection time of certain thermal power values for each initial microbial cell density is, indeed, a suitable method to apply to cases other than the specific *L. reuteri* model.

The resolution or ability of the thermal viable count methods to differentiate cell densities varied with microbial genus and culturing conditions (Figs. 2 and 3). The obtained resolution (~ 2–10-fold changes) of the utilized methods for viable counting is sufficient for most microbiological applications. *Pseudomonas* showed as accurate a resolution as *L. reuteri* in our previous work [15], but *Bacillus* and *Clonostachys* showed more variation between experiments. However, we should emphasize that these standard curves were built based on three independent experiments and that each experiment had slightly different initial cell densities, which contributed to the variance in the standard curves. Therefore, we suggest having better control of the variance of the initial cell density when standard curves of greater accuracy are required.

In addition to other growth requirements, the availability of air should be taken into account when studying aerophilic microbes by IMC [13]. The aerophilic microbial strains in this study have different growth requirements than the anaerobic microbe *L. reuteri*, the subject of our previous study [15–20]. Our results (Fig. 1) showed that the ratio of culturing medium volume to headspace air volume in IMC vials has an effect on the microbial growth during IMC analyses and that this ratio should be considered when planning further IMC experiments.

IMC is known to be a non-specific method that measures all heat produced or consumed in samples [2, 5, 6]. In this work, the surface sterilization of seeds and the culturing conditions in IMC vials could have enriched the *P. brassicacearum* MA250 in the coating as well as certain genera of that natural microbiota that were found, namely, *Pseudomonas*, *Pantoea*, and *Paenibacillus* (Additional file 3). These genera are commonly found in wheat seeds [21, 22]. However, the natural microbiota of wheat seeds could be much more diverse [21–24]. When studying seed coatings, it could also be important to take into account possible seed germination, the heat flow it produces, and its effect on IMC measurements. In this work, we prevented seed germination by immersing the seeds in culturing media. This is most likely explained by the fact that the salinity of media lowered the water potential inhibiting germination. In conclusion, we propose that the specificity of thermal viable count methods could be enhanced by specific sample preparation protocols and using culturing media or substrates enriching the species of interest if the sample is composed of different organisms.

The accuracy of IMC is not dependent on single cells due to its non-specific nature. Our model strains grow differently in pure cultures or if they are coated on seeds, and the thermal viable count methods were applicable in all cases (Fig. 2). In the case of *Pseudomonas*, we did not detect any specific additional grouping of cells within IMC vials (Additional file 4: Figure S1 and S2). *Bacillus* species generate cell chains and grow in clusters in liquid media when not shaken [18, 25]. The *Bacillus* strain in the current study behaved similarly in IMC vials, producing rafts near the medium surface and keeping most of the media clear (Additional file 4: Figure S3). *Clonostachys* grows abundant mycelia and, in some conditions, conidia [19]. In the current study, the *Clonostachys* strain grew a thick layer of hyphae near the medium surface, but no conidium formation was detected under these conditions (Additional file 4: Figure S4). Viable counting of biofilms or biofilm-like structures often requires enhanced protocols, including the disruption of biofilm to utilize dilution series and plating techniques or flow cytometry [26]. IMC has been applied to study biofilms with increasing frequency during the past few years but has not actually been applied to quantify viable cells [27–30]. Therefore, based on our results demonstrating the versatile ability of IMC to quantify the initial viable count of differentially growing microbes, we propose that IMC also has potential to quantify microbes within biofilms and may even quantify selected species within biofilms, similar to the coatings on wheat seeds in this study. However, it should be considered whether a medium, substrate, or species-specific standard curve corresponding to the system of interest should be established.

Thus far, we have discussed the speed, sensitivity and accuracy of IMC measurements as well as how to enhance the specificity of thermal viable count methods. In addition, IMC results can also be followed in real time, IMC-based methods do not require stains or toxic or isotope-labelled chemicals, and downstream applications are optional, similar to standard microbial cultures [2, 5, 6]. Today, high-throughput workflows are becoming mainstream in many biological fields due to the possibility to work, for example, with metagenomics or in a 384-well format with robotics during sample preparation and measurements. We consider the good possibilities to automate the sample preparation process, and IMC equipment is being developed in high-throughput configurations. To our knowledge, a 48-channel system is currently the largest single instrument available for IMC (TA Instruments).

Herein and in our previous work [15], we have proposed and applied a method, thermal viable count, for analysing IMC data and supported it with a workflow for sample preparation and IMC measurements. Thermal viable count methods might overcome and benefit from previously noted challenges related to the non-specificity of IMC measurements [2, 5, 6]. After the preparation of standard curves, the use of IMC equipment and comparison of IMC results to the standard curves would be fast and not require more than standard laboratory skills and brief training. In the simplest IMC procedure, a researcher could add a product or sample of interest into an IMC vial and supplement it with a sufficient amount of substrate to enrich or activate a microbial species of interest. Then, the thermal power produced by IMC would be measured, and the detection time (t) of certain thermal power value (μW) would be noted; these results would be compared to a pre-prepared standard curve to approximate the concentration of viable cells in the sample. Therefore, we propose that these methods could be easily transformed into standard procedures in industry and research and official control laboratories. In future studies, generalized standard curves applicable to different microbial groups or substrates may be developed and commonly available.

## Conclusions

Isothermal microcalorimetry based thermal viable count methods are promising novel methods for the rapid quantification of divergent bacterial and fungal species to enhance routine viable counting processes in academia, industry, and control authorities.

## Methods

### Strains and culturing conditions

*Pseudomonas brassicacearum* MA250 [16], *Bacillus amyloliquefaciens subsp. plantarum* UCMB5113 [17, 18], and *Clonostachys rosea* IK726 [19] were used in this study. *P. brassicacearum* MA250 cells were cultured in tryptic soy broth (TSB) (105,459, Merck, Germany) with shaking at 210 rpm or on TSA (TSB + agar) (105,458, Merck, Germany) at 25 °C. *B. amyloliquefaciens* subsp. *plantarum* UCMB5113 cells were cultured in Luria broth (LB) Miller (L1520, USBiological Life Sciences, USA) with 210 rpm shaking or on LB Lennox plates (L2897, Sigma-Aldrich, USA) at 30 °C, but IMC analyses were performed at 25 °C. *C. rosea* IK726 cells were cultured in potato dextrose broth (PDB) (P6685, Sigma-Aldrich, USA) with 210 rpm shaking or on PDA (PDB + agar) plates (110,130, Merck, Germany) at 25 °C.

### Serial dilutions and plating technique to obtain viable count and biomass measurements

Serial dilutions and plating technique was performed during the study when needed. Samples were diluted up to a computed 10^1^ cfu/ml with 10-fold serial dilutions. From each dilution, 3-8 × 10 μl drops were placed on LB, TSA or PDA plates. The plates were incubated 1–2 days, and log (cfu/ml) was calculated. The biomass (mg/ml) of *C. rosea* IK726 mycelia was weighed wet during the study when needed. Mycelia was collected from the culture and briefly dried against a paper towel to remove dripping water.

### Collection of endospores and conidia

*B. amyloliquefaciens* subsp. *plantarum* UCMB5113 batches were cultured three days in liquid cultures to collect endospores for the experiments. Cells were heat-treated at 75 °C for 10 min to harvest endospores as previously described [31]. The stock concentration of endospores was calculated by serial dilutions and plating viable count analysis. Endospores were stored in LB at 4 °C and used within 3 weeks. *C. rosea* IK726 was grown for 7–14 days to collect conidia for the experiments. One conidium batch was collected out of four full PDA plates by washing the plates with 25 ml of PDB. Conidia and mycelia were filtered through sterilized cotton plugs to harvest conidia [32]. The final volume of a conidium batch was 20 ml. Conidium collection and purity were confirmed by light microscopy (Olympus BH-2, Olympus, Japan) and serial dilutions and plating viable count analysis. Conidium batches were prepared freshly for the experiments.

### Isothermal microcalorimetry

Standard 20 ml IMC vials were capped with matching rubber-metal caps (TA Instruments, USA), and the microbial cultures were cultivated without shaking during analyses. The IMC analyses were performed by an 8-channel TAM Air isothermal microcalorimetry instrument (TA Instruments, USA). Samples were equilibrated 30–60 min at 25 °C prior to IMC measurements in capped IMC vials. All IMC measurements were performed at 25 °C. The IMC data were analysed by TAM Air Assistant v.1.4.2.38 (2011) software (TA Instruments, USA). Data points from every 20 min were exported into Excel for statistical analyses and visualization.

### Sample preparation for isothermal microcalorimetry with microbial pure cultures

*P. brassicacearum* MA250 was grown overnight, pelleted, resuspended, and diluted to the desired cell concentration prior to being pipetted into IMC vials. *B. amyloliquefaciens* subsp. *plantarum* UCMB5113 endospore batches and *C. rosea* IK726 conidium batches were pelleted, resuspended, and diluted prior to their pipetting into IMC vials. To study the effect of headspace air volume in vials on microbial growth, different volumes (3 ml, 6 ml, 12 ml, and 18 ml) of diluted o/n culture of MA250 (~ 10^5^ cfu/ml), diluted spores of UCMB5113 (~ 10^6^ cfu/ml) or diluted conidia of IK726 (~ 10^6^ cfu/ml) were pipetted into separate IMC vials (20 ml). To study the effect of different cell concentrations on the detection time of thermal power, 6 ml of 5–8 different dilutions were pipetted into separate IMC vials. The inert control contained ddH_2_O, and its volume was the same as the respective sample volume. In individual experiments, initial cell concentration (cfu/ml) was calculated by serial dilutions and plating viable count from a stock dilution that was divided into IMC vials. The end cell concentration (cfu/ml) of bacteria was calculated by serial dilutions and plating viable count from 2 to 6 pooled technical replicates. The end biomass (mg/ml) of fungi was weighed from six individual technical replicates. All experiments were repeated independently a minimum of three times with 3–6 replicates, except that volume analyses of *C. rosea* IK726 were performed once with six replicates and measurements with certain cell concentrations (6.3, 2.1 and 0.8 log (cfu/ml)) of *B. amyloliquefaciens* subsp. *plantarum* UCMB5113 were repeated twice with 3–6 replicates.

### Sample preparation for isothermal microcalorimetry with coated wheat seeds

Winter wheat (*Triticum aestivum* L. cv. Norin) seeds (Lantmännen, SE) were coated as previously described [16] with different dilutions (cfu/ml) of *P. brassicacearum* MA250. Seeds (140 g) were surface-sterilized with 400 ml of ¼-diluted bleach supplemented with a few drops of Tween 20 for 15 min with occasional mixing and washed six times with the same volume of ddH_2_O. The germination rate of seeds at the seed bag was determined by individual samples, which were either non-surface-sterilized, surface-sterilized, or coated. For IMC experiments, coated seeds and seed surface sterilization control seeds were weighed (3 g) into IMC vials, which were filled up to 8 ml with TSB media (5.5 ml media). The inert control was 8 ml of ddH_2_O. The initial cfu of *P. brassicacearum* MA250 per g of seeds was calculated from a sample (5.7 g of seeds, filled up to 14 ml with TSB) taken after the coating process and just before seeds were weighed into IMC vials. This sample was incubated and vortexed a few times during IMC sample preparation (1–2 h), and then cfu/g seed was counted by serial dilutions and plating viable count. Experiments with coated seeds were repeated independently three times with three replicates, except for certain concentrations (4.5, 3.3 and 2.7 log (cfu/ml)) whose measurements were repeated independently twice.

### Statistical analyses

To study the linearity of the thermal power detection time point vs cell concentration of the initial cell batch relation at certain thermal power values, regression analyses were performed based on our previously presented mathematical model for this purpose [15]. For regression analyses, thermal power values were chosen based on the individual thermal power curves of each strain. First, a thermal power value was chosen at the point where the IMC measurements were stable and the thermal power curve started to ascend, which was proposed to indicate growth of the bacterial population (*Pseudomonas* = 100 μW, *Pseudomonas* on wheat seeds = 500 μW, *Bacillus* = 150 μW and *Clonostachys* 100 μW). The last thermal power values were chosen near the peak of the thermal power output, after which the thermal power starts to descend again. When required, statistical analyses were performed by Student’s *t*-test for samples with equal variation. All statistical analyses were performed with Microsoft Excel 2016 (Microsoft, USA).

### Strain-specific SCAR-PCR to detect Pseudomonas brassicacearum after being coated on wheat seeds

To confirm the presence of *P. brassicacearum* MA250, we performed MA250 strain-specific SCAR-marker-detecting PCR. Colonies (115 pcs.) were picked from serial dilutions and plating viable count plates before and after IMC experiments for coated and surface-sterilized wheat seeds. Colonies were selected based on their morphology to cover putative MA250 and non-MA250 colonies. Selected colonies represented each independent experiment and seed treatment. Pure cultured MA250 colonies acted as positive controls for SCAR-PCR primers, and *E. coli* DH5α acted as negative controls. *P. brassicacearum* MA250-specific SCAR-PCRs were performed utilizing OPA2–648-forward (TGC CGA GCT GCT AAC CAG ATG CTG G) and OPA2–648-reverse (TGC CGA GCT GAG GGT CGA AGG TCG C) primers [33]. PCRs were run in illustra™ PuReTaq™ Ready-To-Go™ PCR beads (GE Healthcare, UK) supplemented with 1.25 μl of each primer (10 pmol/μl) and 22.5 μl of sterile ddH_2_O. PCRs were run with the following program: 95 °C 2 min, 30x (95 °C 30 s, 65 °C 30 s, and 72 °C 30 s), and 72 °C 5 min. PCR products were analysed by agarose gel electrophoresis.

### 16S rRNA gene PCR and sequencing to identify members of the microbiota on wheat seeds during experiments

Some of the colonies selected for SCAR-PCR were also selected to confirm their identity via 16S rRNA gene PCR. The 16S rRNA gene PCRs were also performed for some of the colonies whose morphology indicated that they represent the natural microbiota of wheat seeds. The 16S rRNA gene PCRs were performed utilizing the following primers: 27F AGA GTT TGA TCM TGG CTC AG and 1492R CGG TTA CCT TGT TAC GAC TT [34]. PCRs were run utilizing illustra™ PuReTaq™ Ready-To-Go™ PCR beads (GE Healthcare, UK) supplemented with 1.25 μl of each primer (10 pmol/μl) and 22.5 μl of sterile ddH_2_O. PCRs were run with the following program: 95 °C 2 min, 30x (95 °C 30 s, 55 °C 30 s and 72 °C 1 min), and 72 °C 7 min. PCR products were purified utilizing illustra™ GFX PCR DNA and Gel Band Purification Kit (GE Healthcare, UK). PCR products were analysed by agarose gel electrophoresis and sequencing. The 16S rRNA gene PCR products of 25 colonies (including one pure cultured MA250 and one *E. coli* DH5α) were submitted for sequencing and sequenced with the same 27F and 1492R primers as PCR (TubeSeq Service, Eurofins Genomics GmbH, DE). Sequencing results were compared to the sequences in the NCBI database by BLASTN suite MegaBLAST to fetch the reciprocal best hits for each colony.

## Additional files

Additional file 1: **Table S1.** Microbial cell densities before (initial) and after (end) isothermal microcalorimetry measurements. (DOCX 29 kb)

Additional file 2: 16S rRNA gene sequences produced in the study in text format. (TXT 49 kb)

Additional file 3: 16S rRNA gene sequences’ BLASTN hits in zipped HTML format. (ZIP 15810 kb)

Additional file 4: **Figure S1.** *Pseudomonas* cultured in IMC vials. **Figure S2.** Coated seeds in IMC vials. **Figure S3.** *Bacillus* cultured in IMC vials. **Figure S4.** *Clonostachys* cultured in IMC vials. (DOCX 1472 kb)

## Abbreviations

Cfu: colony-forming unit
IMC: isothermal microcalorimetry
LB: Luria broth
PCR: polymerase chain reaction
PDA: potato dextrose agar
PDB: potato dextrose broth
SCAR: sequence characterized amplified region
TSA: tryptic soy agar
TSB: tryptic soy broth
