# Supplementary material for: Isothermal microcalorimetry for thermal viable count of microorganisms in pure cultures and stabilized formulations
Source: BMC Microbiol. 2019 Mar 21;19:65. doi: 10.1186/s12866-019-1432-8 (PMC6429831; doi:10.1186/s12866-019-1432-8)
Supplement: Supplementary file 1 — Table S1. Microbial cell densities before (initial) and after (end) isothermal microcalorimetry measurements. (DOCX 29 kb) [file 12866_2019_1432_MOESM1_ESM.docx]

Table S1. Microbial cell densities, calculated by serial dilutions and plating, before (initial) and after (end) isothermal microcalorimetry measurements. In larger culturing volumes, the cultures reached lower end cell concentration (bacterial strains) or lower biomass (fungal strain). Statistically significant difference between end-point samples’ average per bacterial strain is marked with a-j. For *Pseudomonas brassicacearum* MA250 and *Bacillus amyloliquefaciens* subsp. *plantarum* UCMB5113, the table is representing averages and standard deviations of three independent experiments with 3-6 replicates per treatment. For *Clonostachys* *rosea* IK726, the table is representing averages and standard deviations of 6 replicates per treatment.

|  | MA250 | UCMB5113 | IK726 |
| --- | --- | --- | --- |
| Sample | Average log (cfu/ml) | Average log (cfu/ml) | Average log (cfu/ml) |
| Initial | 5.8±0.2 | 3.8±0.4 | 5.9±0.2 |
| End 3 ml | 9.3±0.2^a, b^ | 7.5±0.4^c^ | 95±8 mg/ml^f, g^ |
| End 6 ml | 9.2±0.4 | 6.5±1.1^d^ | 98±14 mg/ml^h, i^ |
| End 12 ml | 8.5±0.3^a^ | 5.5±0.4^e^ | 68±10 mg/ml^f, h, j^ |
| End 18 ml | 8.0±0.3^b^ | 5.1±0.9^c, d, e^ | 37±3 mg/ml^g, i, j^ |
| Statistically different averages within a column | ^a^*p*=0.012, ^b^*p*=0.01, ^c-e^*p*<0.01, ^f^*p*=0.012, ^g^*p*<0.01, ^h^*p*=0.012, ^i^*p<*0.01, ^j^*p*<0.01 | | |
