# Supplementary material for: Isothermal microcalorimetry for thermal viable count of microorganisms in pure cultures and stabilized formulations
Source: BMC Microbiol. 2019 Mar 21;19:65. doi: 10.1186/s12866-019-1432-8 (PMC6429831; doi:10.1186/s12866-019-1432-8)
Supplement: Supplementary file 3 — 16S rRNA gene sequences’ BLASTN hits in zipped HTML format. (ZIP 15810 kb) [file 12866_2019_1432_MOESM3_ESM.zip › Best blastn hits/NCBI Blast_113 pale positive F -- 15..1037 of sequence.html]

NCBI Blast:113 pale positive F -- 15..1037 of sequence


- NCBI Home
- Sign in to NCBI
- Skip to Main Content
- Skip to Navigation
- About NCBI Accesskeys

U.S. National Library of Medicine

NCBI
National Center for Biotechnology Information

- My NCBI
- Sign in to NCBI
- Register
- Sign Out

BLAST ® » blastn suite » RID-A4X421W8014


- Home
- Recent Results
- Saved Strategies
- Help

BLAST Results


Edit and Resubmit
Save Search Strategies
[Sign in above to save your search strategy]

Formatting options 


Download


How to read this page
Blast report description
Questions/comments


|  |  |
| --- | --- |
| Formatting options | |
| Show | Alignment as  HTML Plain text   Old View Reset form to defaults [?]  These options control formatting of alignments in results pages. The default is HTML, but other formats (including plain text) are available. PSSM and PssmWithParameters are representations of Position Specific Scoring Matrices and are only available for PSI-BLAST. The Advanced view option allows the database descriptions to be sorted by various indices in a table. |
| Alignment View | Pairwise Pairwise with dots for identities Query-anchored with dots for identities Query-anchored with letters for identities Flat query-anchored with dots for identities Flat query-anchored with letters for identities [?]  Choose how to view alignments. The default "pairwise" view shows how each subject sequence aligns individually to the query sequence. The "query-anchored" view shows how all subject sequences align to the query sequence. For each view type, you can choose to show "identities" (matching residues) as letters or dots. more... |
| Display | Graphical Overview   Linkout   Sequence Retrieval  NCBI-gi   CDS feature [?]  - Graphical Overview: Graphical Overview: Show graph of similar sequence regions aligned to query.   more... - NCBI-gi: Show NCBI gi identifiers. - CDS feature: Show annotated coding region and translation.   more... |
| Masking | Character:   X for protein, n for nucleotide Lower Case  Color:  Black Grey Red [?]  - Masking Character: Display masked (filtered) sequence regions as lower-case or as specific letters (N for nucleotide, P for protein). - Masking Color: Display masked sequence regions in the given color. |
| Limit results | Descriptions:  10 50 100 Graphical overview:  0 10 50 100  Alignments:  0 10 50 100 Line length:  60 90 120 150 [?]  - Descriptions: Show short descriptions for up to the given number of sequences. - Alignments: Show alignments for up to the given number of sequences, in order of statistical significance. - Line lenghth: Number of letters to show on one line in an alignment. |
|  | Organism Type common name, binomial, taxid, or group name. Only 20 top taxa will be shown.     Exclude    [?]  Show only sequences from the given organism. |
|  | Entrez query:  [?]  Show only those sequences that match the given Entrez query. more... |
|  | Expect Min:  Expect Max:  [?]  Show only sequences with expect values in the given range. more... |
|  | Percent Identity Min:  Percent Identity Max:  [?]  Show only sequences with percent identity values in the given range. |
| Format for | PSI-BLAST with inclusion threshold:  [?]  - Format for PSI-BLAST: The Position-Specific Iterated BLAST (PSI-BLAST) program performs iterative searches with a protein query,   in which sequences found in one round of search are used to build a custom score model for the next round.   more... - Inclusion Threshold: This sets the statistical significance threshold for including a sequence in the model used   by PSI-BLAST to create the PSSM on the next iteration. |

|  |  |  |  |  |  |
| --- | --- | --- | --- | --- | --- |
| Download | | | | | |
| Alignment  Text XML ASN.1 JSON Seq-align Hit Table(text) Hit Table(csv) Multiple-file XML2 Single-file XML2 Multiple-file JSON Single-file JSON SAM | Search Strategies  ASN.1 | PSSM to restart search  PSSM | [?] |

The Download link provides BLAST output that may be used as input to another program.
This includes parseable formats such as the tabular report or XML as well as the Search Strategy files read by the BLAST+ applications.
More details on the parseable (XML, tabular, and ASN.1) reports can be found at
https://www.ncbi.nlm.nih.gov/books/NBK153387/  
  

The following formats are offered under the Alignment section:  
1). "Text". Non-HTML standard BLAST report.  
2). "XML". XML report based upon the DTD at https://www.ncbi.nlm.nih.gov/data\_specs/dtd/NCBI\_BlastOutput.dtd  
3). "ASN.1". Alignment written out in Abstract Syntax Notation 1.  
4). "JSON Seq-align". Alignment written out in JSON.  
4). "Hit Table(text)". The tabular report as text.  
5). "Hit Table(csv)". The tabular report ready for import into spread-sheet programs like Excel.  
6). "XML2". New XML format described at ftp://ftp.ncbi.nlm.nih.gov/blast/documents/NEWXML/xml2.pdf.  
7). "JSON". New JSON format described at ftp://ftp.ncbi.nlm.nih.gov/blast/documents/NEWXML/xml2.pdf.  
8). "SAM". Sequence Alignment Map format.

XML2 and JSON can be downloaded either as one file per query (multiple-file) or one file for all queries (single-file). These formats are listed as Multiple-file XML2 (and JSON) or Single-file XML (and JSON).

The following report is offered under the Search Strategy section:  
1). "ASN.1" Search Strategy. A record of the parameters, query, and database used in the search. This file can be used to start a stand-alone BLAST search, see
https://www.ncbi.nlm.nih.gov/books/NBK1763/#CmdLineAppsManual.I455\_BLAST\_search\_stra


# Job title: 113 pale positive F -- 15..1037 of sequence

Results for:

lcl|Query\_185019 113 pale positive F -- 15..1037 of sequence(1023bp)
[?]

Your BLAST job specified more than one input sequence.
This box lets you choose which input sequence to show BLAST results for.

RID
:   A4X421W8014 (Expires on 03-10 18:48 pm)

Query ID
:   lcl|Query\_185019
:   lcl|Query\_185019

Description
:   113 pale positive F -- 15..1037 of sequence

Molecule type
:   nucleic acid

Query Length
:   1023

Database Name
:   nr

Description
:   Nucleotide collection (nt) See details

Program
:   BLASTN 2.8.0+ Citation

  

Reference 

Zheng Zhang, Scott Schwartz, Lukas Wagner, and Webb Miller (2000), "A greedy algorithm for aligning DNA sequences", J Comput Biol 2000; 7(1-2):203-14.

Reference - database indexing

Aleksandr Morgulis, George Coulouris, Yan Raytselis, Thomas L. Madden, Richa Agarwala, Alejandro A. Schäffer (2008), "Database Indexing for Production MegaBLAST Searches", Bioinformatics 24:1757-1764.

Other reports:
Search Summary

[Taxonomy reports]
[Distance tree of results]
[MSA viewer]

Search Parameters

| Search parameter name | Search parameter value |
| --- | --- |
| Program | blastn |
| Word size | 28 |
| Expect value | 10 |
| Hitlist size | 100 |
| Match/Mismatch scores | 1,-2 |
| Gapcosts | 0,2.5 |
| Low Complexity Filter | Yes |
| Filter string | L;m; |
| Genetic Code | 1 |

Database

| Database parameter name | Database parameter value |
| --- | --- |
| Posted date | Mar 7, 2018 1:58 PM |
| Number of letters | 174,044,644,244 |
| Number of sequences | 46,882,714 |
| Entrez query | Includes:  Excludes:  None |

Karlin-Altschul statistics

| Params | Ungapped | Gapped |
| --- | --- | --- |
| Lambda | 1.33271 | 1.28 |
| K | 0.620991 | 0.46 |
| H | 1.12409 | 0.85 |

Results Statistics

| Results Statistics parameter name | Results Statistics parameter value |
| --- | --- |
| Length adjustment | 35 |
| Effective length of query | 988 |
| Effective length of database | 172403749254 |
| Effective search space | 170334904262952 |
| Effective search space used | 170334904262952 |


## Graphic Summary

### Distribution of the top 128 Blast Hits on 100 subject sequences [?]

The graphic is an overview of the database sequences aligned to the query sequence. These are represented horizontal bars colored coded by score and showing the extent
of the alignment on the query sequence. Separate aligned regions on the same database sequence are connected by a thin grey line.
Mousing over an alignment shows the database sequence title. Clicking an alignment displays a box with more details about the alignment and
link to the sequence alignment itself in the Alignments section of the report.

Mouse over to see the title, click to show alignments

Color key for alignment scores

<40

40-50

50-80

80-200

>=200

Query

1

200

400

600

800

1000

Pseudomonas sp. strain 7.3 16S ribosomal RNA gene, part..

Score:1840 Evalue:0

Accession:KY542120.1

Alignment

Pseudomonas sp. MAR9909 16S ribosomal RNA gene, partial..

Score:1840 Evalue:0

Accession:KU882744.1

Alignment

Pseudomonas brassicacearum subsp. neoaurantiaca strain ..

Score:1840 Evalue:0

Accession:KT997466.1

Alignment

Pseudomonas brassicacearum subsp. neoaurantiaca strain ..

Score:1840 Evalue:0

Accession:KT997442.1

Alignment

Pseudomonas sp. cpRA293 16S ribosomal RNA gene, partial..

Score:1840 Evalue:0

Accession:KJ510220.1

Alignment

Pseudomonas sp. SAM1 16S ribosomal RNA gene, partial se..

Score:1840 Evalue:0

Accession:KM269192.1

Alignment

Pseudomonas thivervalensis strain PE32 16S ribosomal RN..

Score:1840 Evalue:0

Accession:KJ420530.1

Alignment

Pseudomonas thivervalensis strain h-23 16S ribosomal RN..

Score:1840 Evalue:0

Accession:KC139440.1

Alignment

Uncultured Pseudomonas sp. clone QL0ABY37ZD02 16S ribos..

Score:1840 Evalue:0

Accession:JQ712549.1

Alignment

Pseudomonas thivervalensis 16S ribosomal RNA gene, part..

Score:1840 Evalue:0

Accession:JN628032.1

Alignment

Pseudomonas brassicacearum strain Zy-2-1 16S ribosomal ..

Score:1840 Evalue:0

Accession:GU201849.1

Alignment

Pseudomonas brassicacearum isolate MA250 16S ribosomal ..

Score:1840 Evalue:0

Accession:DQ886486.1

Alignment

Pseudomonas putida partial 16S rRNA gene, strain CFBP 4..

Score:1836 Evalue:0

Accession:HF545841.1

Alignment

Pseudomonas brassicacearum strain LBUM300 16S ribosomal..

Score:1834 Evalue:0

Accession:MG461459.1

Alignment

Pseudomonas putida strain 42R-P6 16S ribosomal RNA gene..

Score:1834 Evalue:0

Accession:MF062638.1

Alignment

Pseudomonas fluorescens strain 42R-P4 16S ribosomal RNA..

Score:1834 Evalue:0

Accession:MF062635.1

Alignment

Pseudomonas sp. strain A18 16S ribosomal RNA gene, part..

Score:1834 Evalue:0

Accession:KX859159.1

Alignment

Pseudomonas sp. strain A13 16S ribosomal RNA gene, part..

Score:1834 Evalue:0

Accession:KX859154.1

Alignment

Pseudomonas sp. strain A12 16S ribosomal RNA gene, part..

Score:1834 Evalue:0

Accession:KX859153.1

Alignment

Pseudomonas sp. strain A10 16S ribosomal RNA gene, part..

Score:1834 Evalue:0

Accession:KX859151.1

Alignment

Pseudomonas sp. strain A9 16S ribosomal RNA gene, parti..

Score:1834 Evalue:0

Accession:KX859150.1

Alignment

Pseudomonas sp. strain A8 16S ribosomal RNA gene, parti..

Score:1834 Evalue:0

Accession:KX859149.1

Alignment

Pseudomonas sp. strain A4 16S ribosomal RNA gene, parti..

Score:1834 Evalue:0

Accession:KX859145.1

Alignment

Pseudomonas sp. HA-09 partial 16S rRNA gene, isolate HA..

Score:1834 Evalue:0

Accession:LT844660.1

Alignment

Uncultured Pseudomonas sp. partial 16S rRNA gene, isola..

Score:1834 Evalue:0

Accession:LT718479.1

Alignment

Pseudomonas sp. MAR9910 16S ribosomal RNA gene, partial..

Score:1834 Evalue:0

Accession:KU882739.1

Alignment

Pseudomonas brassicacearum strain BS3663 genome assembl..

Score:1834 Evalue:0

Accession:LT629713.1

Alignment

Pseudomonas brassicacearum strain Delaware 16S ribosoma..

Score:1834 Evalue:0

Accession:KT695846.1

Alignment

Pseudomonas brassicacearum strain Wood1 16S ribosomal R..

Score:1834 Evalue:0

Accession:KT695843.1

Alignment

Pseudomonas brassicacearum strain 93F8 16S ribosomal RN..

Score:1834 Evalue:0

Accession:KT695841.1

Alignment

Pseudomonas brassicacearum strain L13-6-12, complete ge..

Score:1834 Evalue:0

Accession:CP014693.1

Alignment

Pseudomonas brassicacearum subsp. neoaurantiaca partial..

Score:1834 Evalue:0

Accession:LT547829.1

Alignment

Pseudomonas brassicacearum strain PG17 16S ribosomal RN..

Score:1834 Evalue:0

Accession:KU350605.1

Alignment

Pseudomonas brassicacearum strain PG16 16S ribosomal RN..

Score:1834 Evalue:0

Accession:KU350593.1

Alignment

Pseudomonas brassicacearum strain PG14 16S ribosomal RN..

Score:1834 Evalue:0

Accession:KU350592.1

Alignment

Pseudomonas brassicacearum strain LBUM300, complete gen..

Score:1834 Evalue:0

Accession:CP012680.1

Alignment

Pseudomonas fluorescens strain FW300-N2C3, complete gen..

Score:1834 Evalue:0

Accession:CP012831.1

Alignment

Pseudomonas brassicacearum strain SY04(2) 16S ribosomal..

Score:1834 Evalue:0

Accession:KT239461.1

Alignment

Pseudomonas brassicacearum strain KAR22 16S ribosomal R..

Score:1834 Evalue:0

Accession:KR054984.1

Alignment

Pseudomonas brassicacearum strain IHB B 13650 16S ribos..

Score:1834 Evalue:0

Accession:KP762561.1

Alignment

Pseudomonas brassicacearum subsp. neoaurantiaca strain ..

Score:1834 Evalue:0

Accession:KP762555.1

Alignment

Pseudomonas fluorescens strain WCS365 16S ribosomal RNA..

Score:1834 Evalue:0

Accession:KP253039.1

Alignment

Pseudomonas brassicacearum subsp. brassicacearum strain..

Score:1834 Evalue:0

Accession:KP730603.1

Alignment

Pseudomonas brassicacearum subsp. brassicacearum gene f..

Score:1834 Evalue:0

Accession:LC015569.1

Alignment

Pseudomonas sp. DT 5-12 16S ribosomal RNA gene, partial..

Score:1834 Evalue:0

Accession:KM253057.1

Alignment

Pseudomonas sp. DR 2-03 16S ribosomal RNA gene, partial..

Score:1834 Evalue:0

Accession:KM253000.1

Alignment

Pseudomonas fluorescens strain JK15 16S ribosomal RNA g..

Score:1834 Evalue:0

Accession:KF148637.1

Alignment

Pseudomonas fluorescens strain ALEB 7B 16S ribosomal RN..

Score:1834 Evalue:0

Accession:KF460526.1

Alignment

Pseudomonas brassicacearum partial 16S rRNA gene, strai..

Score:1834 Evalue:0

Accession:HF952549.1

Alignment

Pseudomonas brassicacearum partial 16S rRNA gene, strai..

Score:1834 Evalue:0

Accession:HF952537.1

Alignment

Pseudomonas fluorescens strain IBFC2012-45 16S ribosoma..

Score:1834 Evalue:0

Accession:KC246049.1

Alignment

Pseudomonas sp. EA3(2012) 16S ribosomal RNA gene, parti..

Score:1834 Evalue:0

Accession:JX912367.1

Alignment

Pseudomonas brassicacearum strain J12 16S ribosomal RNA..

Score:1834 Evalue:0

Accession:JN605747.1

Alignment

Pseudomonas sp. LB184 partial 16S rRNA gene, isolate LB..

Score:1834 Evalue:0

Accession:FR675976.1

Alignment

Pseudomonas sp. LB183 partial 16S rRNA gene, isolate LB..

Score:1834 Evalue:0

Accession:FR675975.1

Alignment

Pseudomonas sp. SHB3 16S ribosomal RNA gene, partial se..

Score:1834 Evalue:0

Accession:HQ848635.1

Alignment

## Descriptions

, Reading indexes 1-5, displaying indexes 1-5


Load next setPrevious Match

Sequences producing significant alignments:

Show all columns  of the table presenting sequences producing significant alignments 

Select:AllNone
Selected:0

Alignments
Download

FASTA (complete sequence)

FASTA (aligned sequences)

GenBank (complete sequence)

Hit Table (text)

Hit Table (CSV)

Text

XML

ASN.1

Continue
Cancel

GenBank 
Graphics
Distance tree of results
Multiple alignment
Show/hide columns of the table presenting sequences producing significant alignments 

Available columns

Description  
Max Score  
Total Score  
Coverage  
E-value  
IdentN  
Accession  
Restore Defaults
Ok
Cancel

Sequences producing significant alignments:

| Select for downloading or viewing reports | Description | Max score | Total score | Query cover | E value | Ident | Accession |
| --- | --- | --- | --- | --- | --- | --- | --- |
| 1Select seq KY542120.1 | Pseudomonas sp. strain 7.3 16S ribosomal RNA gene, partial sequence | 1840 | 1840 | 98% | 0.0 | 99% | KY542120.1 |
| 2Select seq KU882744.1 | Pseudomonas sp. MAR9909 16S ribosomal RNA gene, partial sequence | 1840 | 1840 | 98% | 0.0 | 99% | KU882744.1 |
| 3Select seq KT997466.1 | Pseudomonas brassicacearum subsp. neoaurantiaca strain MLS-8-1 16S ribosomal RNA gene, partial sequence | 1840 | 1840 | 98% | 0.0 | 99% | KT997466.1 |
| 4Select seq KT997442.1 | Pseudomonas brassicacearum subsp. neoaurantiaca strain MLS-2-8 16S ribosomal RNA gene, partial sequence | 1840 | 1840 | 98% | 0.0 | 99% | KT997442.1 |
| 5Select seq KJ510220.1 | Pseudomonas sp. cpRA293 16S ribosomal RNA gene, partial sequence | 1840 | 1840 | 98% | 0.0 | 99% | KJ510220.1 |
| 6Select seq KM269192.1 | Pseudomonas sp. SAM1 16S ribosomal RNA gene, partial sequence | 1840 | 1840 | 98% | 0.0 | 99% | KM269192.1 |
| 7Select seq KJ420530.1 | Pseudomonas thivervalensis strain PE32 16S ribosomal RNA gene, partial sequence | 1840 | 1840 | 98% | 0.0 | 99% | KJ420530.1 |
| 8Select seq KC139440.1 | Pseudomonas thivervalensis strain h-23 16S ribosomal RNA gene, partial sequence | 1840 | 1840 | 98% | 0.0 | 99% | KC139440.1 |
| 9Select seq JQ712549.1 | Uncultured Pseudomonas sp. clone QL0ABY37ZD02 16S ribosomal RNA gene, partial sequence | 1840 | 1840 | 98% | 0.0 | 99% | JQ712549.1 |
| 10Select seq JN628032.1 | Pseudomonas thivervalensis 16S ribosomal RNA gene, partial sequence | 1840 | 1840 | 98% | 0.0 | 99% | JN628032.1 |
| 11Select seq GU201849.1 | Pseudomonas brassicacearum strain Zy-2-1 16S ribosomal RNA gene, partial sequence | 1840 | 1840 | 98% | 0.0 | 99% | GU201849.1 |
| 12Select seq DQ886486.1 | Pseudomonas brassicacearum isolate MA250 16S ribosomal RNA gene, partial sequence | 1840 | 1840 | 98% | 0.0 | 99% | DQ886486.1 |
| 13Select seq HF545841.1 | Pseudomonas putida partial 16S rRNA gene, strain CFBP 4629 | 1836 | 1836 | 98% | 0.0 | 99% | HF545841.1 |
| 14Select seq MG461459.1 | Pseudomonas brassicacearum strain LBUM300 16S ribosomal RNA gene, partial sequence | 1834 | 1834 | 98% | 0.0 | 99% | MG461459.1 |
| 15Select seq MF062638.1 | Pseudomonas putida strain 42R-P6 16S ribosomal RNA gene, partial sequence | 1834 | 1834 | 98% | 0.0 | 99% | MF062638.1 |
| 16Select seq MF062635.1 | Pseudomonas fluorescens strain 42R-P4 16S ribosomal RNA gene, partial sequence | 1834 | 1834 | 98% | 0.0 | 99% | MF062635.1 |
| 17Select seq KX859159.1 | Pseudomonas sp. strain A18 16S ribosomal RNA gene, partial sequence | 1834 | 1834 | 98% | 0.0 | 99% | KX859159.1 |
| 18Select seq KX859154.1 | Pseudomonas sp. strain A13 16S ribosomal RNA gene, partial sequence | 1834 | 1834 | 98% | 0.0 | 99% | KX859154.1 |
| 19Select seq KX859153.1 | Pseudomonas sp. strain A12 16S ribosomal RNA gene, partial sequence | 1834 | 1834 | 98% | 0.0 | 99% | KX859153.1 |
| 20Select seq KX859151.1 | Pseudomonas sp. strain A10 16S ribosomal RNA gene, partial sequence | 1834 | 1834 | 98% | 0.0 | 99% | KX859151.1 |
| 21Select seq KX859150.1 | Pseudomonas sp. strain A9 16S ribosomal RNA gene, partial sequence | 1834 | 1834 | 98% | 0.0 | 99% | KX859150.1 |
| 22Select seq KX859149.1 | Pseudomonas sp. strain A8 16S ribosomal RNA gene, partial sequence | 1834 | 1834 | 98% | 0.0 | 99% | KX859149.1 |
| 23Select seq KX859145.1 | Pseudomonas sp. strain A4 16S ribosomal RNA gene, partial sequence | 1834 | 1834 | 98% | 0.0 | 99% | KX859145.1 |
| 24Select seq LT844660.1 | Pseudomonas sp. HA-09 partial 16S rRNA gene, isolate HA-09 | 1834 | 1834 | 98% | 0.0 | 99% | LT844660.1 |
| 25Select seq LT718479.1 | Uncultured Pseudomonas sp. partial 16S rRNA gene, isolate VF5 | 1834 | 1834 | 98% | 0.0 | 99% | LT718479.1 |
| 26Select seq KU882739.1 | Pseudomonas sp. MAR9910 16S ribosomal RNA gene, partial sequence | 1834 | 1834 | 98% | 0.0 | 99% | KU882739.1 |
| 27Select seq LT629713.1 | Pseudomonas brassicacearum strain BS3663 genome assembly, chromosome: I | 1834 | 9168 | 98% | 0.0 | 99% | LT629713.1 |
| 28Select seq KT695846.1 | Pseudomonas brassicacearum strain Delaware 16S ribosomal RNA gene, partial sequence | 1834 | 1834 | 98% | 0.0 | 99% | KT695846.1 |
| 29Select seq KT695843.1 | Pseudomonas brassicacearum strain Wood1 16S ribosomal RNA gene, partial sequence | 1834 | 1834 | 98% | 0.0 | 99% | KT695843.1 |
| 30Select seq KT695841.1 | Pseudomonas brassicacearum strain 93F8 16S ribosomal RNA gene, partial sequence | 1834 | 1834 | 98% | 0.0 | 99% | KT695841.1 |
| 31Select seq CP014693.1 | Pseudomonas brassicacearum strain L13-6-12, complete genome | 1834 | 9174 | 98% | 0.0 | 99% | CP014693.1 |
| 32Select seq LT547829.1 | Pseudomonas brassicacearum subsp. neoaurantiaca partial 16S rRNA gene, isolate 3\_C | 1834 | 1834 | 98% | 0.0 | 99% | LT547829.1 |
| 33Select seq KU350605.1 | Pseudomonas brassicacearum strain PG17 16S ribosomal RNA gene, partial sequence | 1834 | 1834 | 98% | 0.0 | 99% | KU350605.1 |
| 34Select seq KU350593.1 | Pseudomonas brassicacearum strain PG16 16S ribosomal RNA gene, partial sequence | 1834 | 1834 | 98% | 0.0 | 99% | KU350593.1 |
| 35Select seq KU350592.1 | Pseudomonas brassicacearum strain PG14 16S ribosomal RNA gene, partial sequence | 1834 | 1834 | 98% | 0.0 | 99% | KU350592.1 |
| 36Select seq CP012680.1 | Pseudomonas brassicacearum strain LBUM300, complete genome | 1834 | 9168 | 98% | 0.0 | 99% | CP012680.1 |
| 37Select seq CP012831.1 | Pseudomonas fluorescens strain FW300-N2C3, complete genome | 1834 | 9174 | 98% | 0.0 | 99% | CP012831.1 |
| 38Select seq KT239461.1 | Pseudomonas brassicacearum strain SY04(2) 16S ribosomal RNA gene, partial sequence | 1834 | 1834 | 98% | 0.0 | 99% | KT239461.1 |
| 39Select seq KR054984.1 | Pseudomonas brassicacearum strain KAR22 16S ribosomal RNA gene, complete sequence | 1834 | 1834 | 98% | 0.0 | 99% | KR054984.1 |
| 40Select seq KP762561.1 | Pseudomonas brassicacearum strain IHB B 13650 16S ribosomal RNA gene, partial sequence | 1834 | 1834 | 98% | 0.0 | 99% | KP762561.1 |
| 41Select seq KP762555.1 | Pseudomonas brassicacearum subsp. neoaurantiaca strain IHB B 13645 16S ribosomal RNA gene, partial sequence | 1834 | 1834 | 98% | 0.0 | 99% | KP762555.1 |
| 42Select seq KP253039.1 | Pseudomonas fluorescens strain WCS365 16S ribosomal RNA gene, partial sequence | 1834 | 1834 | 98% | 0.0 | 99% | KP253039.1 |
| 43Select seq KP730603.1 | Pseudomonas brassicacearum subsp. brassicacearum strain BW0808 16S ribosomal RNA gene, partial sequence | 1834 | 1834 | 98% | 0.0 | 99% | KP730603.1 |
| 44Select seq LC015569.1 | Pseudomonas brassicacearum subsp. brassicacearum gene for 16S ribosomal RNA, partial sequence, strain: AF5 | 1834 | 1834 | 98% | 0.0 | 99% | LC015569.1 |
| 45Select seq KM253057.1 | Pseudomonas sp. DT 5-12 16S ribosomal RNA gene, partial sequence | 1834 | 1834 | 98% | 0.0 | 99% | KM253057.1 |
| 46Select seq KM253000.1 | Pseudomonas sp. DR 2-03 16S ribosomal RNA gene, partial sequence | 1834 | 1834 | 98% | 0.0 | 99% | KM253000.1 |
| 47Select seq KF148637.1 | Pseudomonas fluorescens strain JK15 16S ribosomal RNA gene, partial sequence | 1834 | 1834 | 98% | 0.0 | 99% | KF148637.1 |
| 48Select seq KF460526.1 | Pseudomonas fluorescens strain ALEB 7B 16S ribosomal RNA gene, partial sequence | 1834 | 1834 | 98% | 0.0 | 99% | KF460526.1 |
| 49Select seq HF952549.1 | Pseudomonas brassicacearum partial 16S rRNA gene, strain HMGU196 | 1834 | 1834 | 98% | 0.0 | 99% | HF952549.1 |
| 50Select seq HF952537.1 | Pseudomonas brassicacearum partial 16S rRNA gene, strain HMGU70 | 1834 | 1834 | 98% | 0.0 | 99% | HF952537.1 |
| 51Select seq KC246049.1 | Pseudomonas fluorescens strain IBFC2012-45 16S ribosomal RNA gene, partial sequence | 1834 | 1834 | 98% | 0.0 | 99% | KC246049.1 |
| 52Select seq JX912367.1 | Pseudomonas sp. EA3(2012) 16S ribosomal RNA gene, partial sequence | 1834 | 1834 | 98% | 0.0 | 99% | JX912367.1 |
| 53Select seq JN605747.1 | Pseudomonas brassicacearum strain J12 16S ribosomal RNA gene, partial sequence | 1834 | 1834 | 98% | 0.0 | 99% | JN605747.1 |
| 54Select seq FR675976.1 | Pseudomonas sp. LB184 partial 16S rRNA gene, isolate LB184 | 1834 | 1834 | 98% | 0.0 | 99% | FR675976.1 |
| 55Select seq FR675975.1 | Pseudomonas sp. LB183 partial 16S rRNA gene, isolate LB183 | 1834 | 1834 | 98% | 0.0 | 99% | FR675975.1 |
| 56Select seq HQ848635.1 | Pseudomonas sp. SHB3 16S ribosomal RNA gene, partial sequence | 1834 | 1834 | 98% | 0.0 | 99% | HQ848635.1 |
| 57Select seq JF500978.1 | Uncultured Pseudomonas sp. clone 101 16S ribosomal RNA gene, partial sequence | 1834 | 1834 | 98% | 0.0 | 99% | JF500978.1 |
| 58Select seq JF500932.1 | Uncultured Pseudomonas sp. clone 52 16S ribosomal RNA gene, partial sequence | 1834 | 1834 | 98% | 0.0 | 99% | JF500932.1 |
| 59Select seq CP002585.1 | Pseudomonas brassicacearum subsp. brassicacearum NFM421, complete genome | 1834 | 9168 | 98% | 0.0 | 99% | CP002585.1 |
| 60Select seq FN547411.1 | Pseudomonas sp. G68 partial 16S rRNA gene, strain G68 | 1834 | 1834 | 98% | 0.0 | 99% | FN547411.1 |
| 61Select seq FJ225306.1 | Pseudomonas sp. TAD163 16S ribosomal RNA gene, partial sequence | 1834 | 1834 | 98% | 0.0 | 99% | FJ225306.1 |
| 62Select seq FJ225265.1 | Pseudomonas sp. TAD117 16S ribosomal RNA gene, partial sequence | 1834 | 1834 | 98% | 0.0 | 99% | FJ225265.1 |
| 63Select seq AM900685.1 | Pseudomonas fluorescens partial 16S rRNA gene, strain SCAM BA\_1 | 1834 | 1834 | 98% | 0.0 | 99% | AM900685.1 |
| 64Select seq DQ453838.1 | Pseudomonas sp. P96.25 16S ribosomal RNA gene, partial sequence | 1834 | 1834 | 98% | 0.0 | 99% | DQ453838.1 |
| 65Select seq DQ453837.1 | Pseudomonas sp. K93.3 16S ribosomal RNA gene, partial sequence | 1834 | 1834 | 98% | 0.0 | 99% | DQ453837.1 |
| 66Select seq DQ453836.1 | Pseudomonas sp. C\*1A1 16S ribosomal RNA gene, partial sequence | 1834 | 1834 | 98% | 0.0 | 99% | DQ453836.1 |
| 67Select seq DQ453835.1 | Pseudomonas sp. TM1A3 16S ribosomal RNA gene, partial sequence | 1834 | 1834 | 98% | 0.0 | 99% | DQ453835.1 |
| 68Select seq DQ453820.1 | Pseudomonas sp. K94.37 16S ribosomal RNA gene, complete sequence | 1834 | 1834 | 98% | 0.0 | 99% | DQ453820.1 |
| 69Select seq DQ453818.1 | Pseudomonas sp. K93.2 16S ribosomal RNA gene, complete sequence | 1834 | 1834 | 98% | 0.0 | 99% | DQ453818.1 |
| 70Select seq AY271792.1 | Pseudomonas aurantiaca VKM B-1524 16S ribosomal RNA gene, partial sequence | 1834 | 1834 | 98% | 0.0 | 99% | AY271792.1 |
| 71Select seq AY512624.1 | Pseudomonas sp. A1Y13 16S ribosomal RNA gene, partial sequence | 1834 | 1834 | 98% | 0.0 | 99% | AY512624.1 |
| 72Select seq DQ377772.1 | Pseudomonas kilonensis isolate PD 31 16S ribosomal RNA gene, partial sequence | 1834 | 1834 | 98% | 0.0 | 99% | DQ377772.1 |
| 73Select seq DQ377746.1 | Pseudomonas brassicacearum isolate PD 5 16S ribosomal RNA gene, partial sequence | 1834 | 1834 | 98% | 0.0 | 99% | DQ377746.1 |
| 74Select seq AJ417074.1 | Pseudomonas sp. Q65c-80 16S rRNA gene, strain Q65c-80 | 1834 | 1834 | 98% | 0.0 | 99% | AJ417074.1 |
| 75Select seq AJ417068.1 | Pseudomonas sp. CM1'A2 16S rRNA gene, strain CM1'A2 | 1834 | 1834 | 98% | 0.0 | 99% | AJ417068.1 |
| 76Select seq AJ292381.1 | Pseudomonas brassicacearum 16S rRNA gene, strain 520-1 | 1834 | 1834 | 98% | 0.0 | 99% | AJ292381.1 |
| 77Select seq KR088357.1 | Pseudomonas brassicacearum strain BK23 16S ribosomal RNA gene, partial sequence | 1832 | 1832 | 98% | 0.0 | 99% | KR088357.1 |
| 78Select seq HF952553.1 | Pseudomonas brassicacearum partial 16S rRNA gene, strain HMGU245 | 1832 | 1832 | 98% | 0.0 | 99% | HF952553.1 |
| 79Select seq KX953866.1 | Pseudomonas sp. strain C50T3 16S ribosomal RNA gene, partial sequence | 1831 | 1831 | 98% | 0.0 | 99% | KX953866.1 |
| 80Select seq KY111476.1 | Pseudomonas sp. strain B10 16S ribosomal RNA gene, partial sequence | 1831 | 1831 | 98% | 0.0 | 99% | KY111476.1 |
| 81Select seq KR855698.1 | Pseudomonas brassicacearum strain SM27 16S ribosomal RNA gene, partial sequence | 1831 | 1831 | 98% | 0.0 | 99% | KR855698.1 |
| 82Select seq LN880129.1 | Uncultured Pseudomonas sp. partial 16S rRNA gene, clone W3S26 | 1831 | 1831 | 98% | 0.0 | 99% | LN880129.1 |
| 83Select seq HF584852.1 | Pseudomonas fluorescens partial 16S rRNA gene, isolate BD17-B21 | 1831 | 1831 | 98% | 0.0 | 99% | HF584852.1 |
| 84Select seq JQ977353.1 | Pseudomonas sp. Azb2 16S ribosomal RNA gene, partial sequence | 1831 | 1831 | 98% | 0.0 | 99% | JQ977353.1 |
| 85Select seq JX885768.1 | Pseudomonas fluorescens strain MazP22 16S ribosomal RNA gene, partial sequence | 1831 | 1831 | 98% | 0.0 | 99% | JX885768.1 |
| 86Select seq AB621591.1 | Pseudomonas fluorescens gene for 16S rRNA, partial sequence, strain: MPF18 | 1831 | 1831 | 98% | 0.0 | 99% | AB621591.1 |
| 87Select seq AB602399.1 | Pseudomonas fluorescens gene for 16S ribosomal RNA, partial sequence, strain: MPF3 | 1831 | 1831 | 98% | 0.0 | 99% | AB602399.1 |
| 88Select seq JF500893.1 | Uncultured Pseudomonas sp. clone 7 16S ribosomal RNA gene, partial sequence | 1831 | 1831 | 98% | 0.0 | 99% | JF500893.1 |
| 89Select seq JF500889.1 | Uncultured Pseudomonas sp. clone 3 16S ribosomal RNA gene, partial sequence | 1831 | 1831 | 98% | 0.0 | 99% | JF500889.1 |
| 90Select seq EU373313.1 | Pseudomonas fluorescens strain SSR04 16S ribosomal RNA gene, partial sequence | 1831 | 1831 | 98% | 0.0 | 99% | EU373313.1 |
| 91Select seq DQ377745.1 | Pseudomonas brassicacearum isolate PD 4 16S ribosomal RNA gene, partial sequence | 1831 | 1831 | 98% | 0.0 | 99% | DQ377745.1 |
| 92Select seq CP025542.1 | Pseudomonas fluorescens strain 2P24 chromosome, complete genome | 1829 | 9135 | 98% | 0.0 | 99% | CP025542.1 |
| 93Select seq KX859163.1 | Pseudomonas sp. strain A22 16S ribosomal RNA gene, partial sequence | 1829 | 1829 | 98% | 0.0 | 99% | KX859163.1 |
| 94Select seq KX859162.1 | Pseudomonas sp. strain A21 16S ribosomal RNA gene, partial sequence | 1829 | 1829 | 98% | 0.0 | 99% | KX859162.1 |
| 95Select seq KX758046.1 | Pseudomonas sp. strain Fe13 16S ribosomal RNA gene, partial sequence | 1829 | 1829 | 98% | 0.0 | 99% | KX758046.1 |
| 96Select seq LT629691.1 | Pseudomonas thivervalensis strain BS3779 genome assembly, chromosome: I | 1829 | 9124 | 98% | 0.0 | 99% | LT629691.1 |
| 97Select seq KT580640.1 | Pseudomonas fluorescens strain CanR-1 16S ribosomal RNA gene, partial sequence | 1829 | 1829 | 98% | 0.0 | 99% | KT580640.1 |
| 98Select seq LC015568.1 | Pseudomonas brassicacearum subsp. brassicacearum gene for 16S ribosomal RNA, partial sequence, strain: AF78 | 1829 | 1829 | 98% | 0.0 | 99% | LC015568.1 |
| 99Select seq KM253151.1 | Pseudomonas sp. SR 1-04 16S ribosomal RNA gene, partial sequence | 1829 | 1829 | 98% | 0.0 | 99% | KM253151.1 |
| 100Select seq KJ642348.1 | Pseudomonas sp. EA\_S\_74 16S ribosomal RNA gene, partial sequence | 1829 | 1829 | 98% | 0.0 | 99% | KJ642348.1 |


## Alignments

Loading alignment... for sequences gi|1137473154,gi|1008987913,gi|1018445764,gi|1018445740,gi|673537586 Reading indexes 1-5

Download

FASTA (complete sequence)

FASTA (aligned sequences)

GenBank (complete sequence)

Continue
Cancel

GenBankGraphics

Next
Previous
Descriptions

Pseudomonas sp. strain 7.3 16S ribosomal RNA gene, partial sequence

Sequence ID: KY542120.1Length: 1546Number of Matches: 1

Related Information

Range 1: 60 to 1068GenBankGraphics

Next Match
Previous Match
First Match

Alignment statistics for match #1

| Score | Expect | Identities | Gaps | Strand | Frame |
| --- | --- | --- | --- | --- | --- |
| 1840 bits(996) | 0.0() | 1004/1010(99%) | 1/1010(0%) | Plus/Plus |  |

Features:

```
Query  9     AGTCGAGCGGTAGAGAGGTGCTTGCACCTCTTGAGAGCGGCGGACGGGTGAGTAATGCCT  68
             ||||||||||||||||||||||||||||||||||||||||||||||||||||||||||||
Sbjct  60    AGTCGAGCGGTAGAGAGGTGCTTGCACCTCTTGAGAGCGGCGGACGGGTGAGTAATGCCT  119

Query  69    AGGAATCTGCCTGGTAGTGGGGGATAACGCTCGGAAACGGACGCTAATACCGCATACGTC  128
             ||||||||||||||||||||||||||||||||||||||||||||||||||||||||||||
Sbjct  120   AGGAATCTGCCTGGTAGTGGGGGATAACGCTCGGAAACGGACGCTAATACCGCATACGTC  179

Query  129   CTACGGGAGAAAGCAGGGGACCTTCGGGCCTTGCGCTATCAGATGAGCCTAGGTCGGATT  188
             ||||||||||||||||||||||||||||||||||||||||||||||||||||||||||||
Sbjct  180   CTACGGGAGAAAGCAGGGGACCTTCGGGCCTTGCGCTATCAGATGAGCCTAGGTCGGATT  239

Query  189   AGCTAGTTGGTGGGGTAATGGCTCACCAAGGCGACGATCCGTAACTGGTCTGAGAGGATG  248
             ||||||||||||||||||||||||||||||||||||||||||||||||||||||||||||
Sbjct  240   AGCTAGTTGGTGGGGTAATGGCTCACCAAGGCGACGATCCGTAACTGGTCTGAGAGGATG  299

Query  249   ATCAGTCACACTGGAACTGAGACACGGTCCAGACTCCTACGGGAGGCAGCAGTGGGGAAT  308
             ||||||||||||||||||||||||||||||||||||||||||||||||||||||||||||
Sbjct  300   ATCAGTCACACTGGAACTGAGACACGGTCCAGACTCCTACGGGAGGCAGCAGTGGGGAAT  359

Query  309   ATTGGACAATGGGCGAAAGCCTGATCCAGCCATGCCGCGTGTGTGAAGAAGGTCTTCGGA  368
             ||||||||||||||||||||||||||||||||||||||||||||||||||||||||||||
Sbjct  360   ATTGGACAATGGGCGAAAGCCTGATCCAGCCATGCCGCGTGTGTGAAGAAGGTCTTCGGA  419

Query  369   TTGTAAAGCACTTTAAGTTGGGAGGAAGGGCATTAACCTAATACGTTAGTGTTTTGACGT  428
             ||||||||||||||||||||||||||||||||||||||||||||||||||||||||||||
Sbjct  420   TTGTAAAGCACTTTAAGTTGGGAGGAAGGGCATTAACCTAATACGTTAGTGTTTTGACGT  479

Query  429   TACCGACAGAATAAGCACCGGCTAACTCTGTGCCAGCAGCCGCGGTAATACAGAGGGTGC  488
             ||||||||||||||||||||||||||||||||||||||||||||||||||||||||||||
Sbjct  480   TACCGACAGAATAAGCACCGGCTAACTCTGTGCCAGCAGCCGCGGTAATACAGAGGGTGC  539

Query  489   AAGCGTTAATCGGAATTACTGGGCGTAAAGCGCGCGTAGGTGGTTCGTTAAGTTGGATGT  548
             ||||||||||||||||||||||||||||||||||||||||||||||||||||||||||||
Sbjct  540   AAGCGTTAATCGGAATTACTGGGCGTAAAGCGCGCGTAGGTGGTTCGTTAAGTTGGATGT  599

Query  549   GAAAGCCCCGGGCTCAACCTGGGAACTGCATTCAAAACTGTCGAGCTAGAGTATGGTAGA  608
             ||||||||||||||||||||||||||||||||||||||||||||||||||||||||||||
Sbjct  600   GAAAGCCCCGGGCTCAACCTGGGAACTGCATTCAAAACTGTCGAGCTAGAGTATGGTAGA  659

Query  609   GGGTGGTGGAATTTCCTGTGTAGCGGTGAAATGCGTAGATATAGGAAGGAACACCAGTGG  668
             ||||||||||||||||||||||||||||||||||||||||||||||||||||||||||||
Sbjct  660   GGGTGGTGGAATTTCCTGTGTAGCGGTGAAATGCGTAGATATAGGAAGGAACACCAGTGG  719

Query  669   CGAAGGCGACCACCTGGACTGATACTGACACTGAGGTGCGAAAGCGTGGGGAGCAAACAG  728
             ||||||||||||||||||||||||||||||||||||||||||||||||||||||||||||
Sbjct  720   CGAAGGCGACCACCTGGACTGATACTGACACTGAGGTGCGAAAGCGTGGGGAGCAAACAG  779

Query  729   GATTAGATACCCTGGTAGTCCACGCCGTAAACGATGTCAACTAGCCGTTGGGAGCCTTGA  788
             ||||||||||||||||||||||||||||||||||||||||||||||||||||||||||||
Sbjct  780   GATTAGATACCCTGGTAGTCCACGCCGTAAACGATGTCAACTAGCCGTTGGGAGCCTTGA  839

Query  789   GCTCTTAGTGGCGCAGCTAACGCATTAAGTTGACCGCCTGGGGGAGTACGGCCGCAAGGT  848
             ||||||||||||||||||||||||||||||||||||||| ||||||||||||||||||||
Sbjct  840   GCTCTTAGTGGCGCAGCTAACGCATTAAGTTGACCGCCT-GGGGAGTACGGCCGCAAGGT  898

Query  849   TAAAACTCAAATGAATTGACGGGGGCCCGCACAAGCGNNNGAGCATGTGGTTTAATTCGA  908
             |||||||||||||||||||||||||||||||||||||   ||||||||||||||||||||
Sbjct  899   TAAAACTCAAATGAATTGACGGGGGCCCGCACAAGCGGTGGAGCATGTGGTTTAATTCGA  958

Query  909   AGCAACGCGAAGAACCTTACCAGGCCTTGACATCCAATGAACTTTCCAGANNTGGATTGG  968
             ||||||||||||||||||||||||||||||||||||||||||||||||||  ||||||||
Sbjct  959   AGCAACGCGAAGAACCTTACCAGGCCTTGACATCCAATGAACTTTCCAGAGATGGATTGG  1018

Query  969   TGCCTTCGGGAACATTGAGACAGGTGCTGCATGGCTGTCGTCAGCTCGTG  1018
             ||||||||||||||||||||||||||||||||||||||||||||||||||
Sbjct  1019  TGCCTTCGGGAACATTGAGACAGGTGCTGCATGGCTGTCGTCAGCTCGTG  1068
```

Download

FASTA (complete sequence)

FASTA (aligned sequences)

GenBank (complete sequence)

Continue
Cancel

GenBankGraphics

Next
Previous
Descriptions

Pseudomonas sp. MAR9909 16S ribosomal RNA gene, partial sequence

Sequence ID: KU882744.1Length: 1435Number of Matches: 1

Related Information

Range 1: 5 to 1013GenBankGraphics

Next Match
Previous Match
First Match

Alignment statistics for match #1

| Score | Expect | Identities | Gaps | Strand | Frame |
| --- | --- | --- | --- | --- | --- |
| 1840 bits(996) | 0.0() | 1004/1010(99%) | 1/1010(0%) | Plus/Plus |  |

Features:

```
Query  9     AGTCGAGCGGTAGAGAGGTGCTTGCACCTCTTGAGAGCGGCGGACGGGTGAGTAATGCCT  68
             ||||||||||||||||||||||||||||||||||||||||||||||||||||||||||||
Sbjct  5     AGTCGAGCGGTAGAGAGGTGCTTGCACCTCTTGAGAGCGGCGGACGGGTGAGTAATGCCT  64

Query  69    AGGAATCTGCCTGGTAGTGGGGGATAACGCTCGGAAACGGACGCTAATACCGCATACGTC  128
             ||||||||||||||||||||||||||||||||||||||||||||||||||||||||||||
Sbjct  65    AGGAATCTGCCTGGTAGTGGGGGATAACGCTCGGAAACGGACGCTAATACCGCATACGTC  124

Query  129   CTACGGGAGAAAGCAGGGGACCTTCGGGCCTTGCGCTATCAGATGAGCCTAGGTCGGATT  188
             ||||||||||||||||||||||||||||||||||||||||||||||||||||||||||||
Sbjct  125   CTACGGGAGAAAGCAGGGGACCTTCGGGCCTTGCGCTATCAGATGAGCCTAGGTCGGATT  184

Query  189   AGCTAGTTGGTGGGGTAATGGCTCACCAAGGCGACGATCCGTAACTGGTCTGAGAGGATG  248
             ||||||||||||||||||||||||||||||||||||||||||||||||||||||||||||
Sbjct  185   AGCTAGTTGGTGGGGTAATGGCTCACCAAGGCGACGATCCGTAACTGGTCTGAGAGGATG  244

Query  249   ATCAGTCACACTGGAACTGAGACACGGTCCAGACTCCTACGGGAGGCAGCAGTGGGGAAT  308
             ||||||||||||||||||||||||||||||||||||||||||||||||||||||||||||
Sbjct  245   ATCAGTCACACTGGAACTGAGACACGGTCCAGACTCCTACGGGAGGCAGCAGTGGGGAAT  304

Query  309   ATTGGACAATGGGCGAAAGCCTGATCCAGCCATGCCGCGTGTGTGAAGAAGGTCTTCGGA  368
             ||||||||||||||||||||||||||||||||||||||||||||||||||||||||||||
Sbjct  305   ATTGGACAATGGGCGAAAGCCTGATCCAGCCATGCCGCGTGTGTGAAGAAGGTCTTCGGA  364

Query  369   TTGTAAAGCACTTTAAGTTGGGAGGAAGGGCATTAACCTAATACGTTAGTGTTTTGACGT  428
             ||||||||||||||||||||||||||||||||||||||||||||||||||||||||||||
Sbjct  365   TTGTAAAGCACTTTAAGTTGGGAGGAAGGGCATTAACCTAATACGTTAGTGTTTTGACGT  424

Query  429   TACCGACAGAATAAGCACCGGCTAACTCTGTGCCAGCAGCCGCGGTAATACAGAGGGTGC  488
             ||||||||||||||||||||||||||||||||||||||||||||||||||||||||||||
Sbjct  425   TACCGACAGAATAAGCACCGGCTAACTCTGTGCCAGCAGCCGCGGTAATACAGAGGGTGC  484

Query  489   AAGCGTTAATCGGAATTACTGGGCGTAAAGCGCGCGTAGGTGGTTCGTTAAGTTGGATGT  548
             ||||||||||||||||||||||||||||||||||||||||||||||||||||||||||||
Sbjct  485   AAGCGTTAATCGGAATTACTGGGCGTAAAGCGCGCGTAGGTGGTTCGTTAAGTTGGATGT  544

Query  549   GAAAGCCCCGGGCTCAACCTGGGAACTGCATTCAAAACTGTCGAGCTAGAGTATGGTAGA  608
             ||||||||||||||||||||||||||||||||||||||||||||||||||||||||||||
Sbjct  545   GAAAGCCCCGGGCTCAACCTGGGAACTGCATTCAAAACTGTCGAGCTAGAGTATGGTAGA  604

Query  609   GGGTGGTGGAATTTCCTGTGTAGCGGTGAAATGCGTAGATATAGGAAGGAACACCAGTGG  668
             ||||||||||||||||||||||||||||||||||||||||||||||||||||||||||||
Sbjct  605   GGGTGGTGGAATTTCCTGTGTAGCGGTGAAATGCGTAGATATAGGAAGGAACACCAGTGG  664

Query  669   CGAAGGCGACCACCTGGACTGATACTGACACTGAGGTGCGAAAGCGTGGGGAGCAAACAG  728
             ||||||||||||||||||||||||||||||||||||||||||||||||||||||||||||
Sbjct  665   CGAAGGCGACCACCTGGACTGATACTGACACTGAGGTGCGAAAGCGTGGGGAGCAAACAG  724

Query  729   GATTAGATACCCTGGTAGTCCACGCCGTAAACGATGTCAACTAGCCGTTGGGAGCCTTGA  788
             ||||||||||||||||||||||||||||||||||||||||||||||||||||||||||||
Sbjct  725   GATTAGATACCCTGGTAGTCCACGCCGTAAACGATGTCAACTAGCCGTTGGGAGCCTTGA  784

Query  789   GCTCTTAGTGGCGCAGCTAACGCATTAAGTTGACCGCCTGGGGGAGTACGGCCGCAAGGT  848
             ||||||||||||||||||||||||||||||||||||||| ||||||||||||||||||||
Sbjct  785   GCTCTTAGTGGCGCAGCTAACGCATTAAGTTGACCGCCT-GGGGAGTACGGCCGCAAGGT  843

Query  849   TAAAACTCAAATGAATTGACGGGGGCCCGCACAAGCGNNNGAGCATGTGGTTTAATTCGA  908
             |||||||||||||||||||||||||||||||||||||   ||||||||||||||||||||
Sbjct  844   TAAAACTCAAATGAATTGACGGGGGCCCGCACAAGCGGTGGAGCATGTGGTTTAATTCGA  903

Query  909   AGCAACGCGAAGAACCTTACCAGGCCTTGACATCCAATGAACTTTCCAGANNTGGATTGG  968
             ||||||||||||||||||||||||||||||||||||||||||||||||||  ||||||||
Sbjct  904   AGCAACGCGAAGAACCTTACCAGGCCTTGACATCCAATGAACTTTCCAGAGATGGATTGG  963

Query  969   TGCCTTCGGGAACATTGAGACAGGTGCTGCATGGCTGTCGTCAGCTCGTG  1018
             ||||||||||||||||||||||||||||||||||||||||||||||||||
Sbjct  964   TGCCTTCGGGAACATTGAGACAGGTGCTGCATGGCTGTCGTCAGCTCGTG  1013
```

Download

FASTA (complete sequence)

FASTA (aligned sequences)

GenBank (complete sequence)

Continue
Cancel

GenBankGraphics

Next
Previous
Descriptions

Pseudomonas brassicacearum subsp. neoaurantiaca strain MLS-8-1 16S ribosomal RNA gene, partial sequence

Sequence ID: KT997466.1Length: 1380Number of Matches: 1

Related Information

Range 1: 3 to 1011GenBankGraphics

Next Match
Previous Match
First Match

Alignment statistics for match #1

| Score | Expect | Identities | Gaps | Strand | Frame |
| --- | --- | --- | --- | --- | --- |
| 1840 bits(996) | 0.0() | 1004/1010(99%) | 1/1010(0%) | Plus/Plus |  |

Features:

```
Query  9     AGTCGAGCGGTAGAGAGGTGCTTGCACCTCTTGAGAGCGGCGGACGGGTGAGTAATGCCT  68
             ||||||||||||||||||||||||||||||||||||||||||||||||||||||||||||
Sbjct  3     AGTCGAGCGGTAGAGAGGTGCTTGCACCTCTTGAGAGCGGCGGACGGGTGAGTAATGCCT  62

Query  69    AGGAATCTGCCTGGTAGTGGGGGATAACGCTCGGAAACGGACGCTAATACCGCATACGTC  128
             ||||||||||||||||||||||||||||||||||||||||||||||||||||||||||||
Sbjct  63    AGGAATCTGCCTGGTAGTGGGGGATAACGCTCGGAAACGGACGCTAATACCGCATACGTC  122

Query  129   CTACGGGAGAAAGCAGGGGACCTTCGGGCCTTGCGCTATCAGATGAGCCTAGGTCGGATT  188
             ||||||||||||||||||||||||||||||||||||||||||||||||||||||||||||
Sbjct  123   CTACGGGAGAAAGCAGGGGACCTTCGGGCCTTGCGCTATCAGATGAGCCTAGGTCGGATT  182

Query  189   AGCTAGTTGGTGGGGTAATGGCTCACCAAGGCGACGATCCGTAACTGGTCTGAGAGGATG  248
             ||||||||||||||||||||||||||||||||||||||||||||||||||||||||||||
Sbjct  183   AGCTAGTTGGTGGGGTAATGGCTCACCAAGGCGACGATCCGTAACTGGTCTGAGAGGATG  242

Query  249   ATCAGTCACACTGGAACTGAGACACGGTCCAGACTCCTACGGGAGGCAGCAGTGGGGAAT  308
             ||||||||||||||||||||||||||||||||||||||||||||||||||||||||||||
Sbjct  243   ATCAGTCACACTGGAACTGAGACACGGTCCAGACTCCTACGGGAGGCAGCAGTGGGGAAT  302

Query  309   ATTGGACAATGGGCGAAAGCCTGATCCAGCCATGCCGCGTGTGTGAAGAAGGTCTTCGGA  368
             ||||||||||||||||||||||||||||||||||||||||||||||||||||||||||||
Sbjct  303   ATTGGACAATGGGCGAAAGCCTGATCCAGCCATGCCGCGTGTGTGAAGAAGGTCTTCGGA  362

Query  369   TTGTAAAGCACTTTAAGTTGGGAGGAAGGGCATTAACCTAATACGTTAGTGTTTTGACGT  428
             ||||||||||||||||||||||||||||||||||||||||||||||||||||||||||||
Sbjct  363   TTGTAAAGCACTTTAAGTTGGGAGGAAGGGCATTAACCTAATACGTTAGTGTTTTGACGT  422

Query  429   TACCGACAGAATAAGCACCGGCTAACTCTGTGCCAGCAGCCGCGGTAATACAGAGGGTGC  488
             ||||||||||||||||||||||||||||||||||||||||||||||||||||||||||||
Sbjct  423   TACCGACAGAATAAGCACCGGCTAACTCTGTGCCAGCAGCCGCGGTAATACAGAGGGTGC  482

Query  489   AAGCGTTAATCGGAATTACTGGGCGTAAAGCGCGCGTAGGTGGTTCGTTAAGTTGGATGT  548
             ||||||||||||||||||||||||||||||||||||||||||||||||||||||||||||
Sbjct  483   AAGCGTTAATCGGAATTACTGGGCGTAAAGCGCGCGTAGGTGGTTCGTTAAGTTGGATGT  542

Query  549   GAAAGCCCCGGGCTCAACCTGGGAACTGCATTCAAAACTGTCGAGCTAGAGTATGGTAGA  608
             ||||||||||||||||||||||||||||||||||||||||||||||||||||||||||||
Sbjct  543   GAAAGCCCCGGGCTCAACCTGGGAACTGCATTCAAAACTGTCGAGCTAGAGTATGGTAGA  602

Query  609   GGGTGGTGGAATTTCCTGTGTAGCGGTGAAATGCGTAGATATAGGAAGGAACACCAGTGG  668
             ||||||||||||||||||||||||||||||||||||||||||||||||||||||||||||
Sbjct  603   GGGTGGTGGAATTTCCTGTGTAGCGGTGAAATGCGTAGATATAGGAAGGAACACCAGTGG  662

Query  669   CGAAGGCGACCACCTGGACTGATACTGACACTGAGGTGCGAAAGCGTGGGGAGCAAACAG  728
             ||||||||||||||||||||||||||||||||||||||||||||||||||||||||||||
Sbjct  663   CGAAGGCGACCACCTGGACTGATACTGACACTGAGGTGCGAAAGCGTGGGGAGCAAACAG  722

Query  729   GATTAGATACCCTGGTAGTCCACGCCGTAAACGATGTCAACTAGCCGTTGGGAGCCTTGA  788
             ||||||||||||||||||||||||||||||||||||||||||||||||||||||||||||
Sbjct  723   GATTAGATACCCTGGTAGTCCACGCCGTAAACGATGTCAACTAGCCGTTGGGAGCCTTGA  782

Query  789   GCTCTTAGTGGCGCAGCTAACGCATTAAGTTGACCGCCTGGGGGAGTACGGCCGCAAGGT  848
             ||||||||||||||||||||||||||||||||||||||| ||||||||||||||||||||
Sbjct  783   GCTCTTAGTGGCGCAGCTAACGCATTAAGTTGACCGCCT-GGGGAGTACGGCCGCAAGGT  841

Query  849   TAAAACTCAAATGAATTGACGGGGGCCCGCACAAGCGNNNGAGCATGTGGTTTAATTCGA  908
             |||||||||||||||||||||||||||||||||||||   ||||||||||||||||||||
Sbjct  842   TAAAACTCAAATGAATTGACGGGGGCCCGCACAAGCGGTGGAGCATGTGGTTTAATTCGA  901

Query  909   AGCAACGCGAAGAACCTTACCAGGCCTTGACATCCAATGAACTTTCCAGANNTGGATTGG  968
             ||||||||||||||||||||||||||||||||||||||||||||||||||  ||||||||
Sbjct  902   AGCAACGCGAAGAACCTTACCAGGCCTTGACATCCAATGAACTTTCCAGAGATGGATTGG  961

Query  969   TGCCTTCGGGAACATTGAGACAGGTGCTGCATGGCTGTCGTCAGCTCGTG  1018
             ||||||||||||||||||||||||||||||||||||||||||||||||||
Sbjct  962   TGCCTTCGGGAACATTGAGACAGGTGCTGCATGGCTGTCGTCAGCTCGTG  1011
```

Download

FASTA (complete sequence)

FASTA (aligned sequences)

GenBank (complete sequence)

Continue
Cancel

GenBankGraphics

Next
Previous
Descriptions

Pseudomonas brassicacearum subsp. neoaurantiaca strain MLS-2-8 16S ribosomal RNA gene, partial sequence

Sequence ID: KT997442.1Length: 1368Number of Matches: 1

Related Information

Range 1: 3 to 1011GenBankGraphics

Next Match
Previous Match
First Match

Alignment statistics for match #1

| Score | Expect | Identities | Gaps | Strand | Frame |
| --- | --- | --- | --- | --- | --- |
| 1840 bits(996) | 0.0() | 1004/1010(99%) | 1/1010(0%) | Plus/Plus |  |

Features:

```
Query  9     AGTCGAGCGGTAGAGAGGTGCTTGCACCTCTTGAGAGCGGCGGACGGGTGAGTAATGCCT  68
             ||||||||||||||||||||||||||||||||||||||||||||||||||||||||||||
Sbjct  3     AGTCGAGCGGTAGAGAGGTGCTTGCACCTCTTGAGAGCGGCGGACGGGTGAGTAATGCCT  62

Query  69    AGGAATCTGCCTGGTAGTGGGGGATAACGCTCGGAAACGGACGCTAATACCGCATACGTC  128
             ||||||||||||||||||||||||||||||||||||||||||||||||||||||||||||
Sbjct  63    AGGAATCTGCCTGGTAGTGGGGGATAACGCTCGGAAACGGACGCTAATACCGCATACGTC  122

Query  129   CTACGGGAGAAAGCAGGGGACCTTCGGGCCTTGCGCTATCAGATGAGCCTAGGTCGGATT  188
             ||||||||||||||||||||||||||||||||||||||||||||||||||||||||||||
Sbjct  123   CTACGGGAGAAAGCAGGGGACCTTCGGGCCTTGCGCTATCAGATGAGCCTAGGTCGGATT  182

Query  189   AGCTAGTTGGTGGGGTAATGGCTCACCAAGGCGACGATCCGTAACTGGTCTGAGAGGATG  248
             ||||||||||||||||||||||||||||||||||||||||||||||||||||||||||||
Sbjct  183   AGCTAGTTGGTGGGGTAATGGCTCACCAAGGCGACGATCCGTAACTGGTCTGAGAGGATG  242

Query  249   ATCAGTCACACTGGAACTGAGACACGGTCCAGACTCCTACGGGAGGCAGCAGTGGGGAAT  308
             ||||||||||||||||||||||||||||||||||||||||||||||||||||||||||||
Sbjct  243   ATCAGTCACACTGGAACTGAGACACGGTCCAGACTCCTACGGGAGGCAGCAGTGGGGAAT  302

Query  309   ATTGGACAATGGGCGAAAGCCTGATCCAGCCATGCCGCGTGTGTGAAGAAGGTCTTCGGA  368
             ||||||||||||||||||||||||||||||||||||||||||||||||||||||||||||
Sbjct  303   ATTGGACAATGGGCGAAAGCCTGATCCAGCCATGCCGCGTGTGTGAAGAAGGTCTTCGGA  362

Query  369   TTGTAAAGCACTTTAAGTTGGGAGGAAGGGCATTAACCTAATACGTTAGTGTTTTGACGT  428
             ||||||||||||||||||||||||||||||||||||||||||||||||||||||||||||
Sbjct  363   TTGTAAAGCACTTTAAGTTGGGAGGAAGGGCATTAACCTAATACGTTAGTGTTTTGACGT  422

Query  429   TACCGACAGAATAAGCACCGGCTAACTCTGTGCCAGCAGCCGCGGTAATACAGAGGGTGC  488
             ||||||||||||||||||||||||||||||||||||||||||||||||||||||||||||
Sbjct  423   TACCGACAGAATAAGCACCGGCTAACTCTGTGCCAGCAGCCGCGGTAATACAGAGGGTGC  482

Query  489   AAGCGTTAATCGGAATTACTGGGCGTAAAGCGCGCGTAGGTGGTTCGTTAAGTTGGATGT  548
             ||||||||||||||||||||||||||||||||||||||||||||||||||||||||||||
Sbjct  483   AAGCGTTAATCGGAATTACTGGGCGTAAAGCGCGCGTAGGTGGTTCGTTAAGTTGGATGT  542

Query  549   GAAAGCCCCGGGCTCAACCTGGGAACTGCATTCAAAACTGTCGAGCTAGAGTATGGTAGA  608
             ||||||||||||||||||||||||||||||||||||||||||||||||||||||||||||
Sbjct  543   GAAAGCCCCGGGCTCAACCTGGGAACTGCATTCAAAACTGTCGAGCTAGAGTATGGTAGA  602

Query  609   GGGTGGTGGAATTTCCTGTGTAGCGGTGAAATGCGTAGATATAGGAAGGAACACCAGTGG  668
             ||||||||||||||||||||||||||||||||||||||||||||||||||||||||||||
Sbjct  603   GGGTGGTGGAATTTCCTGTGTAGCGGTGAAATGCGTAGATATAGGAAGGAACACCAGTGG  662

Query  669   CGAAGGCGACCACCTGGACTGATACTGACACTGAGGTGCGAAAGCGTGGGGAGCAAACAG  728
             ||||||||||||||||||||||||||||||||||||||||||||||||||||||||||||
Sbjct  663   CGAAGGCGACCACCTGGACTGATACTGACACTGAGGTGCGAAAGCGTGGGGAGCAAACAG  722

Query  729   GATTAGATACCCTGGTAGTCCACGCCGTAAACGATGTCAACTAGCCGTTGGGAGCCTTGA  788
             ||||||||||||||||||||||||||||||||||||||||||||||||||||||||||||
Sbjct  723   GATTAGATACCCTGGTAGTCCACGCCGTAAACGATGTCAACTAGCCGTTGGGAGCCTTGA  782

Query  789   GCTCTTAGTGGCGCAGCTAACGCATTAAGTTGACCGCCTGGGGGAGTACGGCCGCAAGGT  848
             ||||||||||||||||||||||||||||||||||||||| ||||||||||||||||||||
Sbjct  783   GCTCTTAGTGGCGCAGCTAACGCATTAAGTTGACCGCCT-GGGGAGTACGGCCGCAAGGT  841

Query  849   TAAAACTCAAATGAATTGACGGGGGCCCGCACAAGCGNNNGAGCATGTGGTTTAATTCGA  908
             |||||||||||||||||||||||||||||||||||||   ||||||||||||||||||||
Sbjct  842   TAAAACTCAAATGAATTGACGGGGGCCCGCACAAGCGGTGGAGCATGTGGTTTAATTCGA  901

Query  909   AGCAACGCGAAGAACCTTACCAGGCCTTGACATCCAATGAACTTTCCAGANNTGGATTGG  968
             ||||||||||||||||||||||||||||||||||||||||||||||||||  ||||||||
Sbjct  902   AGCAACGCGAAGAACCTTACCAGGCCTTGACATCCAATGAACTTTCCAGAGATGGATTGG  961

Query  969   TGCCTTCGGGAACATTGAGACAGGTGCTGCATGGCTGTCGTCAGCTCGTG  1018
             ||||||||||||||||||||||||||||||||||||||||||||||||||
Sbjct  962   TGCCTTCGGGAACATTGAGACAGGTGCTGCATGGCTGTCGTCAGCTCGTG  1011
```

Download

FASTA (complete sequence)

FASTA (aligned sequences)

GenBank (complete sequence)

Continue
Cancel

GenBankGraphics

Next
Previous
Descriptions

Pseudomonas sp. cpRA293 16S ribosomal RNA gene, partial sequence

Sequence ID: KJ510220.1Length: 1420Number of Matches: 1

Related Information

Range 1: 19 to 1027GenBankGraphics

Next Match
Previous Match
First Match

Alignment statistics for match #1

| Score | Expect | Identities | Gaps | Strand | Frame |
| --- | --- | --- | --- | --- | --- |
| 1840 bits(996) | 0.0() | 1004/1010(99%) | 1/1010(0%) | Plus/Plus |  |

Features:

```
Query  9     AGTCGAGCGGTAGAGAGGTGCTTGCACCTCTTGAGAGCGGCGGACGGGTGAGTAATGCCT  68
             ||||||||||||||||||||||||||||||||||||||||||||||||||||||||||||
Sbjct  19    AGTCGAGCGGTAGAGAGGTGCTTGCACCTCTTGAGAGCGGCGGACGGGTGAGTAATGCCT  78

Query  69    AGGAATCTGCCTGGTAGTGGGGGATAACGCTCGGAAACGGACGCTAATACCGCATACGTC  128
             ||||||||||||||||||||||||||||||||||||||||||||||||||||||||||||
Sbjct  79    AGGAATCTGCCTGGTAGTGGGGGATAACGCTCGGAAACGGACGCTAATACCGCATACGTC  138

Query  129   CTACGGGAGAAAGCAGGGGACCTTCGGGCCTTGCGCTATCAGATGAGCCTAGGTCGGATT  188
             ||||||||||||||||||||||||||||||||||||||||||||||||||||||||||||
Sbjct  139   CTACGGGAGAAAGCAGGGGACCTTCGGGCCTTGCGCTATCAGATGAGCCTAGGTCGGATT  198

Query  189   AGCTAGTTGGTGGGGTAATGGCTCACCAAGGCGACGATCCGTAACTGGTCTGAGAGGATG  248
             ||||||||||||||||||||||||||||||||||||||||||||||||||||||||||||
Sbjct  199   AGCTAGTTGGTGGGGTAATGGCTCACCAAGGCGACGATCCGTAACTGGTCTGAGAGGATG  258

Query  249   ATCAGTCACACTGGAACTGAGACACGGTCCAGACTCCTACGGGAGGCAGCAGTGGGGAAT  308
             ||||||||||||||||||||||||||||||||||||||||||||||||||||||||||||
Sbjct  259   ATCAGTCACACTGGAACTGAGACACGGTCCAGACTCCTACGGGAGGCAGCAGTGGGGAAT  318

Query  309   ATTGGACAATGGGCGAAAGCCTGATCCAGCCATGCCGCGTGTGTGAAGAAGGTCTTCGGA  368
             ||||||||||||||||||||||||||||||||||||||||||||||||||||||||||||
Sbjct  319   ATTGGACAATGGGCGAAAGCCTGATCCAGCCATGCCGCGTGTGTGAAGAAGGTCTTCGGA  378

Query  369   TTGTAAAGCACTTTAAGTTGGGAGGAAGGGCATTAACCTAATACGTTAGTGTTTTGACGT  428
             ||||||||||||||||||||||||||||||||||||||||||||||||||||||||||||
Sbjct  379   TTGTAAAGCACTTTAAGTTGGGAGGAAGGGCATTAACCTAATACGTTAGTGTTTTGACGT  438

Query  429   TACCGACAGAATAAGCACCGGCTAACTCTGTGCCAGCAGCCGCGGTAATACAGAGGGTGC  488
             ||||||||||||||||||||||||||||||||||||||||||||||||||||||||||||
Sbjct  439   TACCGACAGAATAAGCACCGGCTAACTCTGTGCCAGCAGCCGCGGTAATACAGAGGGTGC  498

Query  489   AAGCGTTAATCGGAATTACTGGGCGTAAAGCGCGCGTAGGTGGTTCGTTAAGTTGGATGT  548
             ||||||||||||||||||||||||||||||||||||||||||||||||||||||||||||
Sbjct  499   AAGCGTTAATCGGAATTACTGGGCGTAAAGCGCGCGTAGGTGGTTCGTTAAGTTGGATGT  558

Query  549   GAAAGCCCCGGGCTCAACCTGGGAACTGCATTCAAAACTGTCGAGCTAGAGTATGGTAGA  608
             ||||||||||||||||||||||||||||||||||||||||||||||||||||||||||||
Sbjct  559   GAAAGCCCCGGGCTCAACCTGGGAACTGCATTCAAAACTGTCGAGCTAGAGTATGGTAGA  618

Query  609   GGGTGGTGGAATTTCCTGTGTAGCGGTGAAATGCGTAGATATAGGAAGGAACACCAGTGG  668
             ||||||||||||||||||||||||||||||||||||||||||||||||||||||||||||
Sbjct  619   GGGTGGTGGAATTTCCTGTGTAGCGGTGAAATGCGTAGATATAGGAAGGAACACCAGTGG  678

Query  669   CGAAGGCGACCACCTGGACTGATACTGACACTGAGGTGCGAAAGCGTGGGGAGCAAACAG  728
             ||||||||||||||||||||||||||||||||||||||||||||||||||||||||||||
Sbjct  679   CGAAGGCGACCACCTGGACTGATACTGACACTGAGGTGCGAAAGCGTGGGGAGCAAACAG  738

Query  729   GATTAGATACCCTGGTAGTCCACGCCGTAAACGATGTCAACTAGCCGTTGGGAGCCTTGA  788
             ||||||||||||||||||||||||||||||||||||||||||||||||||||||||||||
Sbjct  739   GATTAGATACCCTGGTAGTCCACGCCGTAAACGATGTCAACTAGCCGTTGGGAGCCTTGA  798

Query  789   GCTCTTAGTGGCGCAGCTAACGCATTAAGTTGACCGCCTGGGGGAGTACGGCCGCAAGGT  848
             ||||||||||||||||||||||||||||||||||||||| ||||||||||||||||||||
Sbjct  799   GCTCTTAGTGGCGCAGCTAACGCATTAAGTTGACCGCCT-GGGGAGTACGGCCGCAAGGT  857

Query  849   TAAAACTCAAATGAATTGACGGGGGCCCGCACAAGCGNNNGAGCATGTGGTTTAATTCGA  908
             |||||||||||||||||||||||||||||||||||||   ||||||||||||||||||||
Sbjct  858   TAAAACTCAAATGAATTGACGGGGGCCCGCACAAGCGGTGGAGCATGTGGTTTAATTCGA  917

Query  909   AGCAACGCGAAGAACCTTACCAGGCCTTGACATCCAATGAACTTTCCAGANNTGGATTGG  968
             ||||||||||||||||||||||||||||||||||||||||||||||||||  ||||||||
Sbjct  918   AGCAACGCGAAGAACCTTACCAGGCCTTGACATCCAATGAACTTTCCAGAGATGGATTGG  977

Query  969   TGCCTTCGGGAACATTGAGACAGGTGCTGCATGGCTGTCGTCAGCTCGTG  1018
             ||||||||||||||||||||||||||||||||||||||||||||||||||
Sbjct  978   TGCCTTCGGGAACATTGAGACAGGTGCTGCATGGCTGTCGTCAGCTCGTG  1027
```

```

```


BLAST is a registered trademark of the National Library of Medicine

Support center
Mailing list


YouTube

- National Library Of Medicine
- National Institutes Of Health
- U.S. Department of Health & Human Services
- USA.gov

### NCBI


National Center for Biotechnology Information,
 U.S. National Library of Medicine

8600 Rockville Pike,
Bethesda
 MD,
20894
USA

Policies and Guidelines
|
Contact


PreferencesTurn off

External link. Please review our privacy policy.
