# Supplementary material for: Isothermal microcalorimetry for thermal viable count of microorganisms in pure cultures and stabilized formulations
Source: BMC Microbiol. 2019 Mar 21;19:65. doi: 10.1186/s12866-019-1432-8 (PMC6429831; doi:10.1186/s12866-019-1432-8)
Supplement: Supplementary file 3 — 16S rRNA gene sequences’ BLASTN hits in zipped HTML format. (ZIP 15810 kb) [file 12866_2019_1432_MOESM3_ESM.zip › Best blastn hits/NCBI Blast_113 pale positive R -- 11..1069 of sequence.html]

NCBI Blast:113 pale positive R -- 11..1069 of sequence


- NCBI Home
- Sign in to NCBI
- Skip to Main Content
- Skip to Navigation
- About NCBI Accesskeys

U.S. National Library of Medicine

NCBI
National Center for Biotechnology Information

- My NCBI
- Sign in to NCBI
- Register
- Sign Out

BLAST ® » blastn suite » RID-A4X4A61Y015


- Home
- Recent Results
- Saved Strategies
- Help

BLAST Results


Edit and Resubmit
Save Search Strategies
[Sign in above to save your search strategy]

Formatting options 


Download


How to read this page
Blast report description
Questions/comments


|  |  |
| --- | --- |
| Formatting options | |
| Show | Alignment as  HTML Plain text   Old View Reset form to defaults [?]  These options control formatting of alignments in results pages. The default is HTML, but other formats (including plain text) are available. PSSM and PssmWithParameters are representations of Position Specific Scoring Matrices and are only available for PSI-BLAST. The Advanced view option allows the database descriptions to be sorted by various indices in a table. |
| Alignment View | Pairwise Pairwise with dots for identities Query-anchored with dots for identities Query-anchored with letters for identities Flat query-anchored with dots for identities Flat query-anchored with letters for identities [?]  Choose how to view alignments. The default "pairwise" view shows how each subject sequence aligns individually to the query sequence. The "query-anchored" view shows how all subject sequences align to the query sequence. For each view type, you can choose to show "identities" (matching residues) as letters or dots. more... |
| Display | Graphical Overview   Linkout   Sequence Retrieval  NCBI-gi   CDS feature [?]  - Graphical Overview: Graphical Overview: Show graph of similar sequence regions aligned to query.   more... - NCBI-gi: Show NCBI gi identifiers. - CDS feature: Show annotated coding region and translation.   more... |
| Masking | Character:   X for protein, n for nucleotide Lower Case  Color:  Black Grey Red [?]  - Masking Character: Display masked (filtered) sequence regions as lower-case or as specific letters (N for nucleotide, P for protein). - Masking Color: Display masked sequence regions in the given color. |
| Limit results | Descriptions:  10 50 100 Graphical overview:  0 10 50 100  Alignments:  0 10 50 100 Line length:  60 90 120 150 [?]  - Descriptions: Show short descriptions for up to the given number of sequences. - Alignments: Show alignments for up to the given number of sequences, in order of statistical significance. - Line lenghth: Number of letters to show on one line in an alignment. |
|  | Organism Type common name, binomial, taxid, or group name. Only 20 top taxa will be shown.     Exclude    [?]  Show only sequences from the given organism. |
|  | Entrez query:  [?]  Show only those sequences that match the given Entrez query. more... |
|  | Expect Min:  Expect Max:  [?]  Show only sequences with expect values in the given range. more... |
|  | Percent Identity Min:  Percent Identity Max:  [?]  Show only sequences with percent identity values in the given range. |
| Format for | PSI-BLAST with inclusion threshold:  [?]  - Format for PSI-BLAST: The Position-Specific Iterated BLAST (PSI-BLAST) program performs iterative searches with a protein query,   in which sequences found in one round of search are used to build a custom score model for the next round.   more... - Inclusion Threshold: This sets the statistical significance threshold for including a sequence in the model used   by PSI-BLAST to create the PSSM on the next iteration. |

|  |  |  |  |  |  |
| --- | --- | --- | --- | --- | --- |
| Download | | | | | |
| Alignment  Text XML ASN.1 JSON Seq-align Hit Table(text) Hit Table(csv) Multiple-file XML2 Single-file XML2 Multiple-file JSON Single-file JSON SAM | Search Strategies  ASN.1 | PSSM to restart search  PSSM | [?] |

The Download link provides BLAST output that may be used as input to another program.
This includes parseable formats such as the tabular report or XML as well as the Search Strategy files read by the BLAST+ applications.
More details on the parseable (XML, tabular, and ASN.1) reports can be found at
https://www.ncbi.nlm.nih.gov/books/NBK153387/  
  

The following formats are offered under the Alignment section:  
1). "Text". Non-HTML standard BLAST report.  
2). "XML". XML report based upon the DTD at https://www.ncbi.nlm.nih.gov/data\_specs/dtd/NCBI\_BlastOutput.dtd  
3). "ASN.1". Alignment written out in Abstract Syntax Notation 1.  
4). "JSON Seq-align". Alignment written out in JSON.  
4). "Hit Table(text)". The tabular report as text.  
5). "Hit Table(csv)". The tabular report ready for import into spread-sheet programs like Excel.  
6). "XML2". New XML format described at ftp://ftp.ncbi.nlm.nih.gov/blast/documents/NEWXML/xml2.pdf.  
7). "JSON". New JSON format described at ftp://ftp.ncbi.nlm.nih.gov/blast/documents/NEWXML/xml2.pdf.  
8). "SAM". Sequence Alignment Map format.

XML2 and JSON can be downloaded either as one file per query (multiple-file) or one file for all queries (single-file). These formats are listed as Multiple-file XML2 (and JSON) or Single-file XML (and JSON).

The following report is offered under the Search Strategy section:  
1). "ASN.1" Search Strategy. A record of the parameters, query, and database used in the search. This file can be used to start a stand-alone BLAST search, see
https://www.ncbi.nlm.nih.gov/books/NBK1763/#CmdLineAppsManual.I455\_BLAST\_search\_stra


# Job title: 113 pale positive R -- 11..1069 of sequence

Results for:

lcl|Query\_188547 113 pale positive R -- 11..1069 of sequence(1059bp)
[?]

Your BLAST job specified more than one input sequence.
This box lets you choose which input sequence to show BLAST results for.

RID
:   A4X4A61Y015 (Expires on 03-10 18:48 pm)

Query ID
:   lcl|Query\_188547
:   lcl|Query\_188547

Description
:   113 pale positive R -- 11..1069 of sequence

Molecule type
:   nucleic acid

Query Length
:   1059

Database Name
:   nr

Description
:   Nucleotide collection (nt) See details

Program
:   BLASTN 2.8.0+ Citation

  

Reference 

Zheng Zhang, Scott Schwartz, Lukas Wagner, and Webb Miller (2000), "A greedy algorithm for aligning DNA sequences", J Comput Biol 2000; 7(1-2):203-14.

Reference - database indexing

Aleksandr Morgulis, George Coulouris, Yan Raytselis, Thomas L. Madden, Richa Agarwala, Alejandro A. Schäffer (2008), "Database Indexing for Production MegaBLAST Searches", Bioinformatics 24:1757-1764.

Other reports:
Search Summary

[Taxonomy reports]
[Distance tree of results]
[MSA viewer]

Search Parameters

| Search parameter name | Search parameter value |
| --- | --- |
| Program | blastn |
| Word size | 28 |
| Expect value | 10 |
| Hitlist size | 100 |
| Match/Mismatch scores | 1,-2 |
| Gapcosts | 0,2.5 |
| Low Complexity Filter | Yes |
| Filter string | L;m; |
| Genetic Code | 1 |

Database

| Database parameter name | Database parameter value |
| --- | --- |
| Posted date | Mar 7, 2018 1:58 PM |
| Number of letters | 174,044,644,244 |
| Number of sequences | 46,882,714 |
| Entrez query | Includes:  Excludes:  None |

Karlin-Altschul statistics

| Params | Ungapped | Gapped |
| --- | --- | --- |
| Lambda | 1.33271 | 1.28 |
| K | 0.620991 | 0.46 |
| H | 1.12409 | 0.85 |

Results Statistics

| Results Statistics parameter name | Results Statistics parameter value |
| --- | --- |
| Length adjustment | 35 |
| Effective length of query | 1024 |
| Effective length of database | 172403749254 |
| Effective search space | 176541439236096 |
| Effective search space used | 176541439236096 |


## Graphic Summary

### Distribution of the top 130 Blast Hits on 100 subject sequences [?]

The graphic is an overview of the database sequences aligned to the query sequence. These are represented horizontal bars colored coded by score and showing the extent
of the alignment on the query sequence. Separate aligned regions on the same database sequence are connected by a thin grey line.
Mousing over an alignment shows the database sequence title. Clicking an alignment displays a box with more details about the alignment and
link to the sequence alignment itself in the Alignments section of the report.

Mouse over to see the title, click to show alignments

Color key for alignment scores

<40

40-50

50-80

80-200

>=200

Query

1

200

400

600

800

1000

Pseudomonas brassicacearum isolate MA250 16S ribosomal ..

Score:1899 Evalue:0

Accession:DQ886486.1

Alignment

Pseudomonas sp. strain 7.3 16S ribosomal RNA gene, part..

Score:1893 Evalue:0

Accession:KY542120.1

Alignment

Pseudomonas fluorescens strain SBR10 16S ribosomal RNA ..

Score:1893 Evalue:0

Accession:KX018310.1

Alignment

Pseudomonas sp. cpRA293 16S ribosomal RNA gene, partial..

Score:1893 Evalue:0

Accession:KJ510220.1

Alignment

Pseudomonas brassicacearum strain Kr21 16S ribosomal RN..

Score:1893 Evalue:0

Accession:KT215482.1

Alignment

Pseudomonas brassicacearum strain IHB B 13650 16S ribos..

Score:1893 Evalue:0

Accession:KP762561.1

Alignment

Pseudomonas brassicacearum subsp. neoaurantiaca strain ..

Score:1893 Evalue:0

Accession:KP762555.1

Alignment

Pseudomonas sp. 41(2015) 16S ribosomal RNA gene, partia..

Score:1893 Evalue:0

Accession:KR080568.1

Alignment

Pseudomonas sp. SAM1 16S ribosomal RNA gene, partial se..

Score:1893 Evalue:0

Accession:KM269192.1

Alignment

Pseudomonas brassicacearum strain WK-444s 16S ribosomal..

Score:1893 Evalue:0

Accession:KF580861.1

Alignment

Pseudomonas putida partial 16S rRNA gene, strain CFBP 4..

Score:1893 Evalue:0

Accession:HF545841.1

Alignment

Pseudomonas fluorescens gene for 16S rRNA, partial sequ..

Score:1893 Evalue:0

Accession:AB621593.1

Alignment

Pseudomonas thivervalensis 16S ribosomal RNA gene, part..

Score:1893 Evalue:0

Accession:JN628032.1

Alignment

Pseudomonas fluorescens 16S rRNA gene, strain C7R12

Score:1893 Evalue:0

Accession:AM229082.1

Alignment

Pseudomonas sp. HA-09 partial 16S rRNA gene, isolate HA..

Score:1892 Evalue:0

Accession:LT844660.1

Alignment

Pseudomonas brassicacearum subsp. brassicacearum strain..

Score:1892 Evalue:0

Accession:KP730603.1

Alignment

Pseudomonas sp. S-3 16S ribosomal RNA gene, partial seq..

Score:1892 Evalue:0

Accession:KC207086.1

Alignment

Pseudomonas brassicacearum strain LBUM300 16S ribosomal..

Score:1888 Evalue:0

Accession:MG461459.1

Alignment

Pseudomonas putida strain 42R-P6 16S ribosomal RNA gene..

Score:1888 Evalue:0

Accession:MF062638.1

Alignment

Pseudomonas brassicacearum strain Pv10 16S ribosomal RN..

Score:1888 Evalue:0

Accession:MF624719.1

Alignment

Pseudomonas jessenii strain AP3\_16 16S ribosomal RNA ge..

Score:1888 Evalue:0

Accession:MF498772.1

Alignment

Pseudomonas sp. strain A9 16S ribosomal RNA gene, parti..

Score:1888 Evalue:0

Accession:KX859150.1

Alignment

Pseudomonas brassicacearum strain SKUAST-K21 16S riboso..

Score:1888 Evalue:0

Accession:KY612271.1

Alignment

Pseudomonas brassicacearum strain B04 16S ribosomal RNA..

Score:1888 Evalue:0

Accession:KU878092.1

Alignment

Pseudomonas synxantha strain LMG 2190 genome assembly, ..

Score:1888 Evalue:0

Accession:LT629786.1

Alignment

Pseudomonas brassicacearum strain BS3663 genome assembl..

Score:1888 Evalue:0

Accession:LT629713.1

Alignment

Pseudomonas brassicacearum strain TSDS1 16S ribosomal R..

Score:1888 Evalue:0

Accession:KX984045.1

Alignment

Pseudomonas brassicacearum strain Delaware 16S ribosoma..

Score:1888 Evalue:0

Accession:KT695846.1

Alignment

Pseudomonas brassicacearum strain Wood1 16S ribosomal R..

Score:1888 Evalue:0

Accession:KT695843.1

Alignment

Pseudomonas brassicacearum strain 93F8 16S ribosomal RN..

Score:1888 Evalue:0

Accession:KT695841.1

Alignment

Pseudomonas brassicacearum strain L13-6-12, complete ge..

Score:1888 Evalue:0

Accession:CP014693.1

Alignment

Pseudomonas brassicacearum strain KK 5 16S ribosomal RN..

Score:1888 Evalue:0

Accession:KP858915.1

Alignment

Pseudomonas jessenii partial 16S rRNA gene, strain R-52..

Score:1888 Evalue:0

Accession:LN995697.1

Alignment

Pseudomonas brassicacearum strain LBUM300, complete gen..

Score:1888 Evalue:0

Accession:CP012680.1

Alignment

Pseudomonas brassicacearum strain SM27 16S ribosomal RN..

Score:1888 Evalue:0

Accession:KR855698.1

Alignment

Pseudomonas sp. 12(2015) 16S ribosomal RNA gene, partia..

Score:1888 Evalue:0

Accession:KR080562.1

Alignment

Pseudomonas brassicacearum subsp. brassicacearum gene f..

Score:1888 Evalue:0

Accession:LC015570.1

Alignment

Pseudomonas brassicacearum subsp. brassicacearum gene f..

Score:1888 Evalue:0

Accession:LC015569.1

Alignment

Pseudomonas brassicacearum subsp. neoaurantiaca gene fo..

Score:1888 Evalue:0

Accession:LC015567.1

Alignment

Uncultured bacterium clone Untreatedsoil-0day-94 16S ri..

Score:1886 Evalue:0

Accession:MF314815.2

Alignment

Uncultured bacterium clone Untreatedsoil-0day-1 16S rib..

Score:1886 Evalue:0

Accession:MF314725.2

Alignment

Pseudomonas fluorescens strain 2P24 chromosome, complet..

Score:1886 Evalue:0

Accession:CP025542.1

Alignment

Uncultured bacterium clone SPN400-90day-85 16S ribosoma..

Score:1886 Evalue:0

Accession:MF314628.1

Alignment

Uncultured bacterium clone SPN400-90day-76 16S ribosoma..

Score:1886 Evalue:0

Accession:MF314621.1

Alignment

Uncultured bacterium clone SPN400-90day-68 16S ribosoma..

Score:1886 Evalue:0

Accession:MF314613.1

Alignment

Uncultured bacterium clone SPN400-90day-50 16S ribosoma..

Score:1886 Evalue:0

Accession:MF314595.1

Alignment

Uncultured bacterium clone SPN400-90day-46 16S ribosoma..

Score:1886 Evalue:0

Accession:MF314592.1

Alignment

Uncultured bacterium clone SPN400-90day-43 16S ribosoma..

Score:1886 Evalue:0

Accession:MF314589.1

Alignment

Uncultured bacterium clone SPN400-90day-20 16S ribosoma..

Score:1886 Evalue:0

Accession:MF314569.1

Alignment

Uncultured bacterium clone SPN400-90day-6 16S ribosomal..

Score:1886 Evalue:0

Accession:MF314558.1

Alignment

Pseudomonas brassicacearum strain YC-1 16S ribosomal RN..

Score:1886 Evalue:0

Accession:KY753310.1

Alignment

Pseudomonas brassicacearum strain FC-7 16S ribosomal RN..

Score:1886 Evalue:0

Accession:KY649379.1

Alignment

Pseudomonas fluorescens strain PFR1 16S ribosomal RNA g..

Score:1886 Evalue:0

Accession:MF000304.1

Alignment

Pseudomonas syringae strain yangyueP4 16S ribosomal RNA..

Score:1886 Evalue:0

Accession:KU977139.1

Alignment

Pseudomonas sp. RhizorgN 16S ribosomal RNA gene, partia..

Score:1886 Evalue:0

Accession:KT318824.1

Alignment

Pseudomonas sp. RhizorgB 16S ribosomal RNA gene, partia..

Score:1886 Evalue:0

Accession:KT318813.1

Alignment

## Descriptions

, Reading indexes 1-5, displaying indexes 1-5


Load next setPrevious Match

Sequences producing significant alignments:

Show all columns  of the table presenting sequences producing significant alignments 

Select:AllNone
Selected:0

Alignments
Download

FASTA (complete sequence)

FASTA (aligned sequences)

GenBank (complete sequence)

Hit Table (text)

Hit Table (CSV)

Text

XML

ASN.1

Continue
Cancel

GenBank 
Graphics
Distance tree of results
Multiple alignment
Show/hide columns of the table presenting sequences producing significant alignments 

Available columns

Description  
Max Score  
Total Score  
Coverage  
E-value  
IdentN  
Accession  
Restore Defaults
Ok
Cancel

Sequences producing significant alignments:

| Select for downloading or viewing reports | Description | Max score | Total score | Query cover | E value | Ident | Accession |
| --- | --- | --- | --- | --- | --- | --- | --- |
| 1Select seq DQ886486.1 | Pseudomonas brassicacearum isolate MA250 16S ribosomal RNA gene, partial sequence | 1899 | 1899 | 98% | 0.0 | 99% | DQ886486.1 |
| 2Select seq KY542120.1 | Pseudomonas sp. strain 7.3 16S ribosomal RNA gene, partial sequence | 1893 | 1893 | 98% | 0.0 | 99% | KY542120.1 |
| 3Select seq KX018310.1 | Pseudomonas fluorescens strain SBR10 16S ribosomal RNA gene, partial sequence | 1893 | 1893 | 98% | 0.0 | 99% | KX018310.1 |
| 4Select seq KJ510220.1 | Pseudomonas sp. cpRA293 16S ribosomal RNA gene, partial sequence | 1893 | 1893 | 98% | 0.0 | 99% | KJ510220.1 |
| 5Select seq KT215482.1 | Pseudomonas brassicacearum strain Kr21 16S ribosomal RNA gene, partial sequence | 1893 | 1893 | 98% | 0.0 | 99% | KT215482.1 |
| 6Select seq KP762561.1 | Pseudomonas brassicacearum strain IHB B 13650 16S ribosomal RNA gene, partial sequence | 1893 | 1893 | 98% | 0.0 | 99% | KP762561.1 |
| 7Select seq KP762555.1 | Pseudomonas brassicacearum subsp. neoaurantiaca strain IHB B 13645 16S ribosomal RNA gene, partial sequence | 1893 | 1893 | 98% | 0.0 | 99% | KP762555.1 |
| 8Select seq KR080568.1 | Pseudomonas sp. 41(2015) 16S ribosomal RNA gene, partial sequence | 1893 | 1893 | 98% | 0.0 | 99% | KR080568.1 |
| 9Select seq KM269192.1 | Pseudomonas sp. SAM1 16S ribosomal RNA gene, partial sequence | 1893 | 1893 | 98% | 0.0 | 99% | KM269192.1 |
| 10Select seq KF580861.1 | Pseudomonas brassicacearum strain WK-444s 16S ribosomal RNA gene, partial sequence | 1893 | 1893 | 98% | 0.0 | 99% | KF580861.1 |
| 11Select seq HF545841.1 | Pseudomonas putida partial 16S rRNA gene, strain CFBP 4629 | 1893 | 1893 | 98% | 0.0 | 99% | HF545841.1 |
| 12Select seq AB621593.1 | Pseudomonas fluorescens gene for 16S rRNA, partial sequence, strain: MPF29 | 1893 | 1893 | 98% | 0.0 | 99% | AB621593.1 |
| 13Select seq JN628032.1 | Pseudomonas thivervalensis 16S ribosomal RNA gene, partial sequence | 1893 | 1893 | 98% | 0.0 | 99% | JN628032.1 |
| 14Select seq AM229082.1 | Pseudomonas fluorescens 16S rRNA gene, strain C7R12 | 1893 | 1893 | 98% | 0.0 | 99% | AM229082.1 |
| 15Select seq LT844660.1 | Pseudomonas sp. HA-09 partial 16S rRNA gene, isolate HA-09 | 1892 | 1892 | 98% | 0.0 | 99% | LT844660.1 |
| 16Select seq KP730603.1 | Pseudomonas brassicacearum subsp. brassicacearum strain BW0808 16S ribosomal RNA gene, partial sequence | 1892 | 1892 | 98% | 0.0 | 99% | KP730603.1 |
| 17Select seq KC207086.1 | Pseudomonas sp. S-3 16S ribosomal RNA gene, partial sequence | 1892 | 1892 | 97% | 0.0 | 99% | KC207086.1 |
| 18Select seq MG461459.1 | Pseudomonas brassicacearum strain LBUM300 16S ribosomal RNA gene, partial sequence | 1888 | 1888 | 98% | 0.0 | 99% | MG461459.1 |
| 19Select seq MF062638.1 | Pseudomonas putida strain 42R-P6 16S ribosomal RNA gene, partial sequence | 1888 | 1888 | 98% | 0.0 | 99% | MF062638.1 |
| 20Select seq MF624719.1 | Pseudomonas brassicacearum strain Pv10 16S ribosomal RNA gene, partial sequence | 1888 | 1888 | 98% | 0.0 | 99% | MF624719.1 |
| 21Select seq MF498772.1 | Pseudomonas jessenii strain AP3\_16 16S ribosomal RNA gene, partial sequence | 1888 | 1888 | 98% | 0.0 | 99% | MF498772.1 |
| 22Select seq KX859150.1 | Pseudomonas sp. strain A9 16S ribosomal RNA gene, partial sequence | 1888 | 1888 | 98% | 0.0 | 99% | KX859150.1 |
| 23Select seq KY612271.1 | Pseudomonas brassicacearum strain SKUAST-K21 16S ribosomal RNA gene, partial sequence | 1888 | 1888 | 98% | 0.0 | 99% | KY612271.1 |
| 24Select seq KU878092.1 | Pseudomonas brassicacearum strain B04 16S ribosomal RNA gene, partial sequence | 1888 | 1888 | 98% | 0.0 | 99% | KU878092.1 |
| 25Select seq LT629786.1 | Pseudomonas synxantha strain LMG 2190 genome assembly, chromosome: I | 1888 | 12563 | 98% | 0.0 | 99% | LT629786.1 |
| 26Select seq LT629713.1 | Pseudomonas brassicacearum strain BS3663 genome assembly, chromosome: I | 1888 | 9441 | 98% | 0.0 | 99% | LT629713.1 |
| 27Select seq KX984045.1 | Pseudomonas brassicacearum strain TSDS1 16S ribosomal RNA gene, partial sequence | 1888 | 1888 | 98% | 0.0 | 99% | KX984045.1 |
| 28Select seq KT695846.1 | Pseudomonas brassicacearum strain Delaware 16S ribosomal RNA gene, partial sequence | 1888 | 1888 | 98% | 0.0 | 99% | KT695846.1 |
| 29Select seq KT695843.1 | Pseudomonas brassicacearum strain Wood1 16S ribosomal RNA gene, partial sequence | 1888 | 1888 | 98% | 0.0 | 99% | KT695843.1 |
| 30Select seq KT695841.1 | Pseudomonas brassicacearum strain 93F8 16S ribosomal RNA gene, partial sequence | 1888 | 1888 | 98% | 0.0 | 99% | KT695841.1 |
| 31Select seq CP014693.1 | Pseudomonas brassicacearum strain L13-6-12, complete genome | 1888 | 9441 | 98% | 0.0 | 99% | CP014693.1 |
| 32Select seq KP858915.1 | Pseudomonas brassicacearum strain KK 5 16S ribosomal RNA gene, partial sequence | 1888 | 1888 | 98% | 0.0 | 99% | KP858915.1 |
| 33Select seq LN995697.1 | Pseudomonas jessenii partial 16S rRNA gene, strain R-52636 | 1888 | 1888 | 98% | 0.0 | 99% | LN995697.1 |
| 34Select seq CP012680.1 | Pseudomonas brassicacearum strain LBUM300, complete genome | 1888 | 9441 | 98% | 0.0 | 99% | CP012680.1 |
| 35Select seq KR855698.1 | Pseudomonas brassicacearum strain SM27 16S ribosomal RNA gene, partial sequence | 1888 | 1888 | 98% | 0.0 | 99% | KR855698.1 |
| 36Select seq KR080562.1 | Pseudomonas sp. 12(2015) 16S ribosomal RNA gene, partial sequence | 1888 | 1888 | 98% | 0.0 | 99% | KR080562.1 |
| 37Select seq LC015570.1 | Pseudomonas brassicacearum subsp. brassicacearum gene for 16S ribosomal RNA, partial sequence, strain: AF129 | 1888 | 1888 | 98% | 0.0 | 99% | LC015570.1 |
| 38Select seq LC015569.1 | Pseudomonas brassicacearum subsp. brassicacearum gene for 16S ribosomal RNA, partial sequence, strain: AF5 | 1888 | 1888 | 98% | 0.0 | 99% | LC015569.1 |
| 39Select seq LC015567.1 | Pseudomonas brassicacearum subsp. neoaurantiaca gene for 16S ribosomal RNA, partial sequence, strain: AF82 | 1888 | 1888 | 98% | 0.0 | 99% | LC015567.1 |
| 40Select seq MF314815.2 | Uncultured bacterium clone Untreatedsoil-0day-94 16S ribosomal RNA gene, partial sequence | 1886 | 1886 | 97% | 0.0 | 99% | MF314815.2 |
| 41Select seq MF314725.2 | Uncultured bacterium clone Untreatedsoil-0day-1 16S ribosomal RNA gene, partial sequence | 1886 | 1886 | 97% | 0.0 | 99% | MF314725.2 |
| 42Select seq CP025542.1 | Pseudomonas fluorescens strain 2P24 chromosome, complete genome | 1886 | 9421 | 97% | 0.0 | 99% | CP025542.1 |
| 43Select seq MF314628.1 | Uncultured bacterium clone SPN400-90day-85 16S ribosomal RNA gene, partial sequence | 1886 | 1886 | 97% | 0.0 | 99% | MF314628.1 |
| 44Select seq MF314621.1 | Uncultured bacterium clone SPN400-90day-76 16S ribosomal RNA gene, partial sequence | 1886 | 1886 | 97% | 0.0 | 99% | MF314621.1 |
| 45Select seq MF314613.1 | Uncultured bacterium clone SPN400-90day-68 16S ribosomal RNA gene, partial sequence | 1886 | 1886 | 97% | 0.0 | 99% | MF314613.1 |
| 46Select seq MF314595.1 | Uncultured bacterium clone SPN400-90day-50 16S ribosomal RNA gene, partial sequence | 1886 | 1886 | 97% | 0.0 | 99% | MF314595.1 |
| 47Select seq MF314592.1 | Uncultured bacterium clone SPN400-90day-46 16S ribosomal RNA gene, partial sequence | 1886 | 1886 | 97% | 0.0 | 99% | MF314592.1 |
| 48Select seq MF314589.1 | Uncultured bacterium clone SPN400-90day-43 16S ribosomal RNA gene, partial sequence | 1886 | 1886 | 97% | 0.0 | 99% | MF314589.1 |
| 49Select seq MF314569.1 | Uncultured bacterium clone SPN400-90day-20 16S ribosomal RNA gene, partial sequence | 1886 | 1886 | 97% | 0.0 | 99% | MF314569.1 |
| 50Select seq MF314558.1 | Uncultured bacterium clone SPN400-90day-6 16S ribosomal RNA gene, partial sequence | 1886 | 1886 | 97% | 0.0 | 99% | MF314558.1 |
| 51Select seq KY753310.1 | Pseudomonas brassicacearum strain YC-1 16S ribosomal RNA gene, partial sequence | 1886 | 1886 | 97% | 0.0 | 99% | KY753310.1 |
| 52Select seq KY649379.1 | Pseudomonas brassicacearum strain FC-7 16S ribosomal RNA gene, partial sequence | 1886 | 1886 | 97% | 0.0 | 99% | KY649379.1 |
| 53Select seq MF000304.1 | Pseudomonas fluorescens strain PFR1 16S ribosomal RNA gene, partial sequence | 1886 | 1886 | 97% | 0.0 | 99% | MF000304.1 |
| 54Select seq KU977139.1 | Pseudomonas syringae strain yangyueP4 16S ribosomal RNA gene, partial sequence | 1886 | 1886 | 97% | 0.0 | 99% | KU977139.1 |
| 55Select seq KT318824.1 | Pseudomonas sp. RhizorgN 16S ribosomal RNA gene, partial sequence | 1886 | 1886 | 97% | 0.0 | 99% | KT318824.1 |
| 56Select seq KT318813.1 | Pseudomonas sp. RhizorgB 16S ribosomal RNA gene, partial sequence | 1886 | 1886 | 97% | 0.0 | 99% | KT318813.1 |
| 57Select seq CP015225.1 | Pseudomonas fluorescens strain FW300-N2E2 genome | 1886 | 9425 | 97% | 0.0 | 99% | CP015225.1 |
| 58Select seq KJ191010.1 | Pseudomonas sp. B055-40 16S ribosomal RNA gene, partial sequence | 1886 | 1886 | 97% | 0.0 | 99% | KJ191010.1 |
| 59Select seq KT957303.1 | Pseudomonas corrugata strain G1 16S ribosomal RNA gene, partial sequence | 1886 | 1886 | 97% | 0.0 | 99% | KT957303.1 |
| 60Select seq CP012831.1 | Pseudomonas fluorescens strain FW300-N2C3, complete genome | 1886 | 9432 | 97% | 0.0 | 99% | CP012831.1 |
| 61Select seq KR822273.1 | Pseudomonas migulae strain DD290 16S ribosomal RNA gene, partial sequence | 1886 | 1886 | 97% | 0.0 | 99% | KR822273.1 |
| 62Select seq KT321681.1 | Pseudomonas fluorescens 16S ribosomal RNA gene, partial sequence | 1886 | 1886 | 97% | 0.0 | 99% | KT321681.1 |
| 63Select seq KR083019.1 | Pseudomonas brassicacearum strain 13B-23 16S ribosomal RNA gene, partial sequence | 1886 | 1886 | 97% | 0.0 | 99% | KR083019.1 |
| 64Select seq KR061416.1 | Pseudomonas brassicacearum strain 10C-30 16S ribosomal RNA gene, partial sequence | 1886 | 1886 | 97% | 0.0 | 99% | KR061416.1 |
| 65Select seq KR061398.1 | Pseudomonas brassicacearum strain 1Cg-22 16S ribosomal RNA gene, partial sequence | 1886 | 1886 | 97% | 0.0 | 99% | KR061398.1 |
| 66Select seq KP742980.1 | Pseudomonas thivervalensis strain MAH1 16S ribosomal RNA gene, partial sequence | 1886 | 1886 | 97% | 0.0 | 99% | KP742980.1 |
| 67Select seq KR611048.1 | Pseudomonas brassicacearum strain 11B23 16S ribosomal RNA gene, partial sequence | 1886 | 1886 | 97% | 0.0 | 99% | KR611048.1 |
| 68Select seq KR611047.1 | Pseudomonas brassicacearum strain 3B1 16S ribosomal RNA gene, partial sequence | 1886 | 1886 | 97% | 0.0 | 99% | KR611047.1 |
| 69Select seq KR080563.1 | Pseudomonas sp. 15(2015) 16S ribosomal RNA gene, partial sequence | 1886 | 1886 | 97% | 0.0 | 99% | KR080563.1 |
| 70Select seq KM030057.1 | Pseudomonas sp. TY1210 16S ribosomal RNA gene, partial sequence | 1886 | 1886 | 97% | 0.0 | 99% | KM030057.1 |
| 71Select seq KJ420530.1 | Pseudomonas thivervalensis strain PE32 16S ribosomal RNA gene, partial sequence | 1886 | 1886 | 97% | 0.0 | 99% | KJ420530.1 |
| 72Select seq KF148637.1 | Pseudomonas fluorescens strain JK15 16S ribosomal RNA gene, partial sequence | 1886 | 1886 | 97% | 0.0 | 99% | KF148637.1 |
| 73Select seq KF840730.1 | Pseudomonas kilonensis strain JX22 16S ribosomal RNA gene, partial sequence | 1886 | 1886 | 97% | 0.0 | 99% | KF840730.1 |
| 74Select seq KF835389.1 | Pseudomonas sp. SDW-16 16S ribosomal RNA gene, partial sequence | 1886 | 1886 | 97% | 0.0 | 99% | KF835389.1 |
| 75Select seq KF475874.1 | Pseudomonas brassicacearum strain IHB B 821 16S ribosomal RNA gene, partial sequence | 1886 | 1886 | 97% | 0.0 | 99% | KF475874.1 |
| 76Select seq KF312468.1 | Pseudomonas sp. SJ7b 16S ribosomal RNA gene, partial sequence | 1886 | 1886 | 97% | 0.0 | 99% | KF312468.1 |
| 77Select seq KC428747.1 | Pseudomonas sp. X123 16S ribosomal RNA gene, partial sequence | 1886 | 1886 | 97% | 0.0 | 99% | KC428747.1 |
| 78Select seq KC246049.1 | Pseudomonas fluorescens strain IBFC2012-45 16S ribosomal RNA gene, partial sequence | 1886 | 1886 | 97% | 0.0 | 99% | KC246049.1 |
| 79Select seq HQ888871.1 | Pseudomonas fluorescens strain P-72-10 16S ribosomal RNA gene, partial sequence | 1886 | 1886 | 97% | 0.0 | 99% | HQ888871.1 |
| 80Select seq JN216880.1 | Pseudomonas sp. N-128 16S ribosomal RNA gene, partial sequence | 1886 | 1886 | 97% | 0.0 | 99% | JN216880.1 |
| 81Select seq JX077087.1 | Uncultured Pseudomonas sp. clone CGMCG 6071 16S ribosomal RNA gene, partial sequence | 1886 | 1886 | 97% | 0.0 | 99% | JX077087.1 |
| 82Select seq JN399995.1 | Pseudomonas fluorescens strain J2 16S ribosomal RNA gene, partial sequence | 1886 | 1886 | 97% | 0.0 | 99% | JN399995.1 |
| 83Select seq JX082293.1 | Pseudomonas sp. R25(2012) 16S ribosomal RNA gene, partial sequence | 1886 | 1886 | 97% | 0.0 | 99% | JX082293.1 |
| 84Select seq JF901350.1 | Endophytic bacterium 41P-2 16S ribosomal RNA gene, partial sequence | 1886 | 1886 | 97% | 0.0 | 99% | JF901350.1 |
| 85Select seq JQ779065.1 | Bacterium NTL501 16S ribosomal RNA gene, partial sequence | 1886 | 1886 | 97% | 0.0 | 99% | JQ779065.1 |
| 86Select seq JQ779056.1 | Bacterium NTL264 16S ribosomal RNA gene, partial sequence | 1886 | 1886 | 97% | 0.0 | 99% | JQ779056.1 |
| 87Select seq JQ779041.1 | Bacterium NTL206 16S ribosomal RNA gene, partial sequence | 1886 | 1886 | 97% | 0.0 | 99% | JQ779041.1 |
| 88Select seq JQ237663.1 | Pseudomonas sp. hswx161 16S ribosomal RNA gene, partial sequence | 1886 | 1886 | 97% | 0.0 | 99% | JQ237663.1 |
| 89Select seq JN628030.1 | Pseudomonas brassicacearum subsp. neoaurantiaca 16S ribosomal RNA gene, partial sequence | 1886 | 1886 | 97% | 0.0 | 99% | JN628030.1 |
| 90Select seq JN033549.1 | Pseudomonas brassicacearum strain ME BHU2 16S ribosomal RNA gene, partial sequence | 1886 | 1886 | 97% | 0.0 | 99% | JN033549.1 |
| 91Select seq HQ143608.1 | Pseudomonas sp. strain MTQ15 16S ribosomal RNA gene, partial sequence | 1886 | 1886 | 97% | 0.0 | 99% | HQ143608.1 |
| 92Select seq HM854217.1 | Pseudomonas fluorescens strain STAD384 16S ribosomal RNA gene, partial sequence | 1886 | 1886 | 97% | 0.0 | 99% | HM854217.1 |
| 93Select seq HM579795.1 | Pseudomonas sp. HY4(2010) 16S ribosomal RNA gene, partial sequence | 1886 | 1886 | 97% | 0.0 | 99% | HM579795.1 |
| 94Select seq GU201849.1 | Pseudomonas brassicacearum strain Zy-2-1 16S ribosomal RNA gene, partial sequence | 1886 | 1886 | 97% | 0.0 | 99% | GU201849.1 |
| 95Select seq EF540490.1 | Pseudomonas sp. 4\_C7/16\_5 16S ribosomal RNA gene, partial sequence | 1886 | 1886 | 97% | 0.0 | 99% | EF540490.1 |
| 96Select seq DQ453833.1 | Pseudomonas sp. P97.26 16S ribosomal RNA gene, partial sequence | 1886 | 1886 | 97% | 0.0 | 99% | DQ453833.1 |
| 97Select seq DQ431467.1 | Pseudomonas fluorescens strain XG32 16S ribosomal RNA gene, partial sequence | 1886 | 1886 | 97% | 0.0 | 99% | DQ431467.1 |
| 98Select seq AY447045.1 | Pseudomonas fluorescens strain 2P24 16S ribosomal RNA gene, partial sequence | 1886 | 1886 | 97% | 0.0 | 99% | AY447045.1 |
| 99Select seq AB204716.1 | Pseudomonas fluorescens gene for 16S rRNA, partial sequence, strain:LRB3W1 | 1886 | 1886 | 97% | 0.0 | 99% | AB204716.1 |
| 100Select seq KF460526.1 | Pseudomonas fluorescens strain ALEB 7B 16S ribosomal RNA gene, partial sequence | 1884 | 1884 | 97% | 0.0 | 99% | KF460526.1 |


## Alignments

Loading alignment... for sequences gi|115315703,gi|1137473154,gi|1015918342,gi|673537586,gi|858972104 Reading indexes 1-5

Download

FASTA (complete sequence)

FASTA (aligned sequences)

GenBank (complete sequence)

Continue
Cancel

GenBankGraphics

Next
Previous
Descriptions

Pseudomonas brassicacearum isolate MA250 16S ribosomal RNA gene, partial sequence

Sequence ID: DQ886486.1Length: 1515Number of Matches: 1

Related Information

Range 1: 402 to 1443GenBankGraphics

Next Match
Previous Match
First Match

Alignment statistics for match #1

| Score | Expect | Identities | Gaps | Strand | Frame |
| --- | --- | --- | --- | --- | --- |
| 1899 bits(1028) | 0.0() | 1035/1042(99%) | 0/1042(0%) | Plus/Minus |  |

Features:

```
Query  10    CGTCCTNNCGAAGGTTAGACTAGCTACTTCTGGTGCAACCCACTCCCATGGTGTGACGGG  69
             ||||||  ||||||||||||||||||||||||||||||||||||||||||||||||||||
Sbjct  1443  CGTCCTCCCGAAGGTTAGACTAGCTACTTCTGGTGCAACCCACTCCCATGGTGTGACGGG  1384

Query  70    CGGTGTGTACAAGGCCCGGGAACGTATTCACCGCGACATTCTGATTCGCGATTACTAGCG  129
             ||||||||||||||||||||||||||||||||||||||||||||||||||||||||||||
Sbjct  1383  CGGTGTGTACAAGGCCCGGGAACGTATTCACCGCGACATTCTGATTCGCGATTACTAGCG  1324

Query  130   ATTCCGACTTCACGCAGTCGAGTTGCAGACTGCGATCCGGACTACGATCGGTTTTGTGGG  189
             ||||||||||||||||||||||||||||||||||||||||||||||||||||||||||||
Sbjct  1323  ATTCCGACTTCACGCAGTCGAGTTGCAGACTGCGATCCGGACTACGATCGGTTTTGTGGG  1264

Query  190   ATTAGCTCCACCTCGCGGCTTGGCAACCCTCTGTACCGACCATTGTAGCACGTGTGTAGC  249
             ||||||||||||||||||||||||||||||||||||||||||||||||||||||||||||
Sbjct  1263  ATTAGCTCCACCTCGCGGCTTGGCAACCCTCTGTACCGACCATTGTAGCACGTGTGTAGC  1204

Query  250   CCAGGCCGTAAGGGCCATGATGACTTGACGTCATCCCCACCTTCCTCCGGTTTGTCACCG  309
             ||||||||||||||||||||||||||||||||||||||||||||||||||||||||||||
Sbjct  1203  CCAGGCCGTAAGGGCCATGATGACTTGACGTCATCCCCACCTTCCTCCGGTTTGTCACCG  1144

Query  310   GCAGTCTCCTTAGAGTGCCCACCATGACGTGCTGGTAACTAAGGACAAGGGTTGCGCTCG  369
             ||||||||||||||||||||||||||||||||||||||||||||||||||||||||||||
Sbjct  1143  GCAGTCTCCTTAGAGTGCCCACCATGACGTGCTGGTAACTAAGGACAAGGGTTGCGCTCG  1084

Query  370   TTACGGGACTTAACCCAACATCTCACGACACGAGCTGACGACAGCCATGCAGCACCTGTC  429
             ||||||||||||||||||||||||||||||||||||||||||||||||||||||||||||
Sbjct  1083  TTACGGGACTTAACCCAACATCTCACGACACGAGCTGACGACAGCCATGCAGCACCTGTC  1024

Query  430   TCAATNNNCCCGAAGGCACCAATCCATCTCTGGAAAGTTCATTGGATGTCAAGGCCTGGT  489
             |||||   ||||||||||||||||||||||||||||||||||||||||||||||||||||
Sbjct  1023  TCAATGTTCCCGAAGGCACCAATCCATCTCTGGAAAGTTCATTGGATGTCAAGGCCTGGT  964

Query  490   AAGGTTCTTCGCGTTGCTTCGAATTAAACCACATGCTCCACCGCTTGTGCGGGCCCCCGT  549
             ||||||||||||||||||||||||||||||||||||||||||||||||||||||||||||
Sbjct  963   AAGGTTCTTCGCGTTGCTTCGAATTAAACCACATGCTCCACCGCTTGTGCGGGCCCCCGT  904

Query  550   CAATTCATTTGAGTTTTAACCTTGCGGCCGTACTCCCCAGGCGGTCAACTTAATGCGTTA  609
             ||||||||||||||||||||||||||||||||||||||||||||||||||||||||||||
Sbjct  903   CAATTCATTTGAGTTTTAACCTTGCGGCCGTACTCCCCAGGCGGTCAACTTAATGCGTTA  844

Query  610   GCTGCGCCACTAAGAGCTCAAGGCTCCCAACGGCTAGTTGACATCGTTTACGGCGTGGAC  669
             ||||||||||||||||||||||||||||||||||||||||||||||||||||||||||||
Sbjct  843   GCTGCGCCACTAAGAGCTCAAGGCTCCCAACGGCTAGTTGACATCGTTTACGGCGTGGAC  784

Query  670   TACCAGGGTATCTAATCCTGTTTGCTCCCCACGCTTTCGCACCTCAGTGTCAGTATCAGT  729
             ||||||||||||||||||||||||||||||||||||||||||||||||||||||||||||
Sbjct  783   TACCAGGGTATCTAATCCTGTTTGCTCCCCACGCTTTCGCACCTCAGTGTCAGTATCAGT  724

Query  730   CCAGGTGGTCGCCTTCGCCACTGGTGTTCCTTCCTATATCTACGCATTTCACCGCTACAC  789
             ||||||||||||||||||||||||||||||||||||||||||||||||||||||||||||
Sbjct  723   CCAGGTGGTCGCCTTCGCCACTGGTGTTCCTTCCTATATCTACGCATTTCACCGCTACAC  664

Query  790   AGGAAATTCCACCACCCTCTACCATACTCTAGCTCGACAGTTTTGAATGCAGTTCCCAGG  849
             ||||||||||||||||||||||||||||||||||||||||||||||||||||||||||||
Sbjct  663   AGGAAATTCCACCACCCTCTACCATACTCTAGCTCGACAGTTTTGAATGCAGTTCCCAGG  604

Query  850   TTGAGCCCGGGGCTTTCACATCCAACTTAACGAACCACCTACGCGCGCTTTACGCCCAGT  909
             ||||||||||||||||||||||||||||||||||||||||||||||||||||||||||||
Sbjct  603   TTGAGCCCGGGGCTTTCACATCCAACTTAACGAACCACCTACGCGCGCTTTACGCCCAGT  544

Query  910   AATTCCGATTAACGCTTGCACCCTCTGTATTACCGCGGCTGCTGGCACAGAGTTAGCCGG  969
             ||||||||||||||||||||||||||||||||||||||||||||||||||||||||||||
Sbjct  543   AATTCCGATTAACGCTTGCACCCTCTGTATTACCGCGGCTGCTGGCACAGAGTTAGCCGG  484

Query  970   TGCTTATTCTGTCGGTAACGTCAAANCACTAACGTATTAGGTTAATGCCCTTCCTCCCAA  1029
             ||||||||||||||||||||||||| ||||||||||||||||||||||||||||||||||
Sbjct  483   TGCTTATTCTGTCGGTAACGTCAAAACACTAACGTATTAGGTTAATGCCCTTCCTCCCAA  424

Query  1030  CTTAAAGTGCTTTNCAATCCGA  1051
             ||||||||||||| ||||||||
Sbjct  423   CTTAAAGTGCTTTACAATCCGA  402
```

Download

FASTA (complete sequence)

FASTA (aligned sequences)

GenBank (complete sequence)

Continue
Cancel

GenBankGraphics

Next
Previous
Descriptions

Pseudomonas sp. strain 7.3 16S ribosomal RNA gene, partial sequence

Sequence ID: KY542120.1Length: 1546Number of Matches: 1

Related Information

Range 1: 415 to 1456GenBankGraphics

Next Match
Previous Match
First Match

Alignment statistics for match #1

| Score | Expect | Identities | Gaps | Strand | Frame |
| --- | --- | --- | --- | --- | --- |
| 1893 bits(1025) | 0.0() | 1034/1042(99%) | 0/1042(0%) | Plus/Minus |  |

Features:

```
Query  10    CGTCCTNNCGAAGGTTAGACTAGCTACTTCTGGTGCAACCCACTCCCATGGTGTGACGGG  69
             ||||||  ||||||||||||||||||||||||||||||||||||||||||||||||||||
Sbjct  1456  CGTCCTCCCGAAGGTTAGACTAGCTACTTCTGGTGCAACCCACTCCCATGGTGTGACGGG  1397

Query  70    CGGTGTGTACAAGGCCCGGGAACGTATTCACCGCGACATTCTGATTCGCGATTACTAGCG  129
             ||||||||||||||||||||||||||||||||||||||||||||||||||||||||||||
Sbjct  1396  CGGTGTGTACAAGGCCCGGGAACGTATTCACCGCGACATTCTGATTCGCGATTACTAGCG  1337

Query  130   ATTCCGACTTCACGCAGTCGAGTTGCAGACTGCGATCCGGACTACGATCGGTTTTGTGGG  189
             ||||||||||||||||||||||||||||||||||||||||||||||||||||||||||||
Sbjct  1336  ATTCCGACTTCACGCAGTCGAGTTGCAGACTGCGATCCGGACTACGATCGGTTTTGTGGG  1277

Query  190   ATTAGCTCCACCTCGCGGCTTGGCAACCCTCTGTACCGACCATTGTAGCACGTGTGTAGC  249
             ||||||||||||||||||||||||||||||||||||||||||||||||||||||||||||
Sbjct  1276  ATTAGCTCCACCTCGCGGCTTGGCAACCCTCTGTACCGACCATTGTAGCACGTGTGTAGC  1217

Query  250   CCAGGCCGTAAGGGCCATGATGACTTGACGTCATCCCCACCTTCCTCCGGTTTGTCACCG  309
             ||||||||||||||||||||||||||||||||||||||||||||||||||||||||||||
Sbjct  1216  CCAGGCCGTAAGGGCCATGATGACTTGACGTCATCCCCACCTTCCTCCGGTTTGTCACCG  1157

Query  310   GCAGTCTCCTTAGAGTGCCCACCATGACGTGCTGGTAACTAAGGACAAGGGTTGCGCTCG  369
             ||||||||||||||||||||||||| ||||||||||||||||||||||||||||||||||
Sbjct  1156  GCAGTCTCCTTAGAGTGCCCACCATAACGTGCTGGTAACTAAGGACAAGGGTTGCGCTCG  1097

Query  370   TTACGGGACTTAACCCAACATCTCACGACACGAGCTGACGACAGCCATGCAGCACCTGTC  429
             ||||||||||||||||||||||||||||||||||||||||||||||||||||||||||||
Sbjct  1096  TTACGGGACTTAACCCAACATCTCACGACACGAGCTGACGACAGCCATGCAGCACCTGTC  1037

Query  430   TCAATNNNCCCGAAGGCACCAATCCATCTCTGGAAAGTTCATTGGATGTCAAGGCCTGGT  489
             |||||   ||||||||||||||||||||||||||||||||||||||||||||||||||||
Sbjct  1036  TCAATGTTCCCGAAGGCACCAATCCATCTCTGGAAAGTTCATTGGATGTCAAGGCCTGGT  977

Query  490   AAGGTTCTTCGCGTTGCTTCGAATTAAACCACATGCTCCACCGCTTGTGCGGGCCCCCGT  549
             ||||||||||||||||||||||||||||||||||||||||||||||||||||||||||||
Sbjct  976   AAGGTTCTTCGCGTTGCTTCGAATTAAACCACATGCTCCACCGCTTGTGCGGGCCCCCGT  917

Query  550   CAATTCATTTGAGTTTTAACCTTGCGGCCGTACTCCCCAGGCGGTCAACTTAATGCGTTA  609
             ||||||||||||||||||||||||||||||||||||||||||||||||||||||||||||
Sbjct  916   CAATTCATTTGAGTTTTAACCTTGCGGCCGTACTCCCCAGGCGGTCAACTTAATGCGTTA  857

Query  610   GCTGCGCCACTAAGAGCTCAAGGCTCCCAACGGCTAGTTGACATCGTTTACGGCGTGGAC  669
             ||||||||||||||||||||||||||||||||||||||||||||||||||||||||||||
Sbjct  856   GCTGCGCCACTAAGAGCTCAAGGCTCCCAACGGCTAGTTGACATCGTTTACGGCGTGGAC  797

Query  670   TACCAGGGTATCTAATCCTGTTTGCTCCCCACGCTTTCGCACCTCAGTGTCAGTATCAGT  729
             ||||||||||||||||||||||||||||||||||||||||||||||||||||||||||||
Sbjct  796   TACCAGGGTATCTAATCCTGTTTGCTCCCCACGCTTTCGCACCTCAGTGTCAGTATCAGT  737

Query  730   CCAGGTGGTCGCCTTCGCCACTGGTGTTCCTTCCTATATCTACGCATTTCACCGCTACAC  789
             ||||||||||||||||||||||||||||||||||||||||||||||||||||||||||||
Sbjct  736   CCAGGTGGTCGCCTTCGCCACTGGTGTTCCTTCCTATATCTACGCATTTCACCGCTACAC  677

Query  790   AGGAAATTCCACCACCCTCTACCATACTCTAGCTCGACAGTTTTGAATGCAGTTCCCAGG  849
             ||||||||||||||||||||||||||||||||||||||||||||||||||||||||||||
Sbjct  676   AGGAAATTCCACCACCCTCTACCATACTCTAGCTCGACAGTTTTGAATGCAGTTCCCAGG  617

Query  850   TTGAGCCCGGGGCTTTCACATCCAACTTAACGAACCACCTACGCGCGCTTTACGCCCAGT  909
             ||||||||||||||||||||||||||||||||||||||||||||||||||||||||||||
Sbjct  616   TTGAGCCCGGGGCTTTCACATCCAACTTAACGAACCACCTACGCGCGCTTTACGCCCAGT  557

Query  910   AATTCCGATTAACGCTTGCACCCTCTGTATTACCGCGGCTGCTGGCACAGAGTTAGCCGG  969
             ||||||||||||||||||||||||||||||||||||||||||||||||||||||||||||
Sbjct  556   AATTCCGATTAACGCTTGCACCCTCTGTATTACCGCGGCTGCTGGCACAGAGTTAGCCGG  497

Query  970   TGCTTATTCTGTCGGTAACGTCAAANCACTAACGTATTAGGTTAATGCCCTTCCTCCCAA  1029
             ||||||||||||||||||||||||| ||||||||||||||||||||||||||||||||||
Sbjct  496   TGCTTATTCTGTCGGTAACGTCAAAACACTAACGTATTAGGTTAATGCCCTTCCTCCCAA  437

Query  1030  CTTAAAGTGCTTTNCAATCCGA  1051
             ||||||||||||| ||||||||
Sbjct  436   CTTAAAGTGCTTTACAATCCGA  415
```

Download

FASTA (complete sequence)

FASTA (aligned sequences)

GenBank (complete sequence)

Continue
Cancel

GenBankGraphics

Next
Previous
Descriptions

Pseudomonas fluorescens strain SBR10 16S ribosomal RNA gene, partial sequence

Sequence ID: KX018310.1Length: 1486Number of Matches: 1

Related Information

Range 1: 419 to 1460GenBankGraphics

Next Match
Previous Match
First Match

Alignment statistics for match #1

| Score | Expect | Identities | Gaps | Strand | Frame |
| --- | --- | --- | --- | --- | --- |
| 1893 bits(1025) | 0.0() | 1035/1042(99%) | 0/1042(0%) | Plus/Minus |  |

Features:

```
Query  10    CGTCCTNNCGAAGGTTAGACTAGCTACTTCTGGTGCAACCCACTCCCATGGTGTGACGGG  69
             ||||||  ||||||||||||||||||||||||||||||||||||||||||||||||||||
Sbjct  1460  CGTCCTCCCGAAGGTTAGACTAGCTACTTCTGGTGCAACCCACTCCCATGGTGTGACGGG  1401

Query  70    CGGTGTGTACAAGGCCCGGGAACGTATTCACCGCGACATTCTGATTCGCGATTACTAGCG  129
             ||||||||||||||||||||||||||||||||||||||||||||||||||||||||||||
Sbjct  1400  CGGTGTGTACAAGGCCCGGGAACGTATTCACCGCGACATTCTGATTCGCGATTACTAGCG  1341

Query  130   ATTCCGACTTCACGCAGTCGAGTTGCAGACTGCGATCCGGACTACGATCGGTTTTGTGGG  189
             ||||||||||||||||||||||||||||||||||||||||||||||||||||||||||||
Sbjct  1340  ATTCCGACTTCACGCAGTCGAGTTGCAGACTGCGATCCGGACTACGATCGGTTTTGTGGG  1281

Query  190   ATTAGCTCCACCTCGCGGCTTGGCAACCCTCTGTACCGACCATTGTAGCACGTGTGTAGC  249
             ||||||||||||||||||||||||||||||||||||||||||||||||||||||||||||
Sbjct  1280  ATTAGCTCCACCTCGCGGCTTGGCAACCCTCTGTACCGACCATTGTAGCACGTGTGTAGC  1221

Query  250   CCAGGCCGTAAGGGCCATGATGACTTGACGTCATCCCCACCTTCCTCCGGTTTGTCACCG  309
             ||||||||||||||||||||||||||||||||||||||||||||||||||||||||||||
Sbjct  1220  CCAGGCCGTAAGGGCCATGATGACTTGACGTCATCCCCACCTTCCTCCGGTTTGTCACCG  1161

Query  310   GCAGTCTCCTTAGAGTGCCCACCATGACGTGCTGGTAACTAAGGACAAGGGTTGCGCTCG  369
             ||||||||||||||||||||||||| ||||||||||||||||||||||||||||||||||
Sbjct  1160  GCAGTCTCCTTAGAGTGCCCACCATAACGTGCTGGTAACTAAGGACAAGGGTTGCGCTCG  1101

Query  370   TTACGGGACTTAACCCAACATCTCACGACACGAGCTGACGACAGCCATGCAGCACCTGTC  429
             ||||||||||||||||||||||||||||||||||||||||||||||||||||||||||||
Sbjct  1100  TTACGGGACTTAACCCAACATCTCACGACACGAGCTGACGACAGCCATGCAGCACCTGTC  1041

Query  430   TCAATNNNCCCGAAGGCACCAATCCATCTCTGGAAAGTTCATTGGATGTCAAGGCCTGGT  489
             ||||| | ||||||||||||||||||||||||||||||||||||||||||||||||||||
Sbjct  1040  TCAATGNTCCCGAAGGCACCAATCCATCTCTGGAAAGTTCATTGGATGTCAAGGCCTGGT  981

Query  490   AAGGTTCTTCGCGTTGCTTCGAATTAAACCACATGCTCCACCGCTTGTGCGGGCCCCCGT  549
             ||||||||||||||||||||||||||||||||||||||||||||||||||||||||||||
Sbjct  980   AAGGTTCTTCGCGTTGCTTCGAATTAAACCACATGCTCCACCGCTTGTGCGGGCCCCCGT  921

Query  550   CAATTCATTTGAGTTTTAACCTTGCGGCCGTACTCCCCAGGCGGTCAACTTAATGCGTTA  609
             ||||||||||||||||||||||||||||||||||||||||||||||||||||||||||||
Sbjct  920   CAATTCATTTGAGTTTTAACCTTGCGGCCGTACTCCCCAGGCGGTCAACTTAATGCGTTA  861

Query  610   GCTGCGCCACTAAGAGCTCAAGGCTCCCAACGGCTAGTTGACATCGTTTACGGCGTGGAC  669
             ||||||||||||||||||||||||||||||||||||||||||||||||||||||||||||
Sbjct  860   GCTGCGCCACTAAGAGCTCAAGGCTCCCAACGGCTAGTTGACATCGTTTACGGCGTGGAC  801

Query  670   TACCAGGGTATCTAATCCTGTTTGCTCCCCACGCTTTCGCACCTCAGTGTCAGTATCAGT  729
             ||||||||||||||||||||||||||||||||||||||||||||||||||||||||||||
Sbjct  800   TACCAGGGTATCTAATCCTGTTTGCTCCCCACGCTTTCGCACCTCAGTGTCAGTATCAGT  741

Query  730   CCAGGTGGTCGCCTTCGCCACTGGTGTTCCTTCCTATATCTACGCATTTCACCGCTACAC  789
             ||||||||||||||||||||||||||||||||||||||||||||||||||||||||||||
Sbjct  740   CCAGGTGGTCGCCTTCGCCACTGGTGTTCCTTCCTATATCTACGCATTTCACCGCTACAC  681

Query  790   AGGAAATTCCACCACCCTCTACCATACTCTAGCTCGACAGTTTTGAATGCAGTTCCCAGG  849
             ||||||||||||||||||||||||||||||||||||||||||||||||||||||||||||
Sbjct  680   AGGAAATTCCACCACCCTCTACCATACTCTAGCTCGACAGTTTTGAATGCAGTTCCCAGG  621

Query  850   TTGAGCCCGGGGCTTTCACATCCAACTTAACGAACCACCTACGCGCGCTTTACGCCCAGT  909
             ||||||||||||||||||||||||||||||||||||||||||||||||||||||||||||
Sbjct  620   TTGAGCCCGGGGCTTTCACATCCAACTTAACGAACCACCTACGCGCGCTTTACGCCCAGT  561

Query  910   AATTCCGATTAACGCTTGCACCCTCTGTATTACCGCGGCTGCTGGCACAGAGTTAGCCGG  969
             ||||||||||||||||||||||||||||||||||||||||||||||||||||||||||||
Sbjct  560   AATTCCGATTAACGCTTGCACCCTCTGTATTACCGCGGCTGCTGGCACAGAGTTAGCCGG  501

Query  970   TGCTTATTCTGTCGGTAACGTCAAANCACTAACGTATTAGGTTAATGCCCTTCCTCCCAA  1029
             ||||||||||||||||||||||||| ||||||||||||||||||||||||||||||||||
Sbjct  500   TGCTTATTCTGTCGGTAACGTCAAAACACTAACGTATTAGGTTAATGCCCTTCCTCCCAA  441

Query  1030  CTTAAAGTGCTTTNCAATCCGA  1051
             ||||||||||||| ||||||||
Sbjct  440   CTTAAAGTGCTTTACAATCCGA  419
```

Download

FASTA (complete sequence)

FASTA (aligned sequences)

GenBank (complete sequence)

Continue
Cancel

GenBankGraphics

Next
Previous
Descriptions

Pseudomonas sp. cpRA293 16S ribosomal RNA gene, partial sequence

Sequence ID: KJ510220.1Length: 1420Number of Matches: 1

Related Information

Range 1: 374 to 1415GenBankGraphics

Next Match
Previous Match
First Match

Alignment statistics for match #1

| Score | Expect | Identities | Gaps | Strand | Frame |
| --- | --- | --- | --- | --- | --- |
| 1893 bits(1025) | 0.0() | 1034/1042(99%) | 0/1042(0%) | Plus/Minus |  |

Features:

```
Query  10    CGTCCTNNCGAAGGTTAGACTAGCTACTTCTGGTGCAACCCACTCCCATGGTGTGACGGG  69
             ||||||  ||||||||||||||||||||||||||||||||||||||||||||||||||||
Sbjct  1415  CGTCCTCCCGAAGGTTAGACTAGCTACTTCTGGTGCAACCCACTCCCATGGTGTGACGGG  1356

Query  70    CGGTGTGTACAAGGCCCGGGAACGTATTCACCGCGACATTCTGATTCGCGATTACTAGCG  129
             ||||||||||||||||||||||||||||||||||||||||||||||||||||||||||||
Sbjct  1355  CGGTGTGTACAAGGCCCGGGAACGTATTCACCGCGACATTCTGATTCGCGATTACTAGCG  1296

Query  130   ATTCCGACTTCACGCAGTCGAGTTGCAGACTGCGATCCGGACTACGATCGGTTTTGTGGG  189
             ||||||||||||||||||||||||||||||||||||||||||||||||||||||||||||
Sbjct  1295  ATTCCGACTTCACGCAGTCGAGTTGCAGACTGCGATCCGGACTACGATCGGTTTTGTGGG  1236

Query  190   ATTAGCTCCACCTCGCGGCTTGGCAACCCTCTGTACCGACCATTGTAGCACGTGTGTAGC  249
             ||||||||||||||||||||||||||||||||||||||||||||||||||||||||||||
Sbjct  1235  ATTAGCTCCACCTCGCGGCTTGGCAACCCTCTGTACCGACCATTGTAGCACGTGTGTAGC  1176

Query  250   CCAGGCCGTAAGGGCCATGATGACTTGACGTCATCCCCACCTTCCTCCGGTTTGTCACCG  309
             ||||||||||||||||||||||||||||||||||||||||||||||||||||||||||||
Sbjct  1175  CCAGGCCGTAAGGGCCATGATGACTTGACGTCATCCCCACCTTCCTCCGGTTTGTCACCG  1116

Query  310   GCAGTCTCCTTAGAGTGCCCACCATGACGTGCTGGTAACTAAGGACAAGGGTTGCGCTCG  369
             ||||||||||||||||||||||||| ||||||||||||||||||||||||||||||||||
Sbjct  1115  GCAGTCTCCTTAGAGTGCCCACCATAACGTGCTGGTAACTAAGGACAAGGGTTGCGCTCG  1056

Query  370   TTACGGGACTTAACCCAACATCTCACGACACGAGCTGACGACAGCCATGCAGCACCTGTC  429
             ||||||||||||||||||||||||||||||||||||||||||||||||||||||||||||
Sbjct  1055  TTACGGGACTTAACCCAACATCTCACGACACGAGCTGACGACAGCCATGCAGCACCTGTC  996

Query  430   TCAATNNNCCCGAAGGCACCAATCCATCTCTGGAAAGTTCATTGGATGTCAAGGCCTGGT  489
             |||||   ||||||||||||||||||||||||||||||||||||||||||||||||||||
Sbjct  995   TCAATGTTCCCGAAGGCACCAATCCATCTCTGGAAAGTTCATTGGATGTCAAGGCCTGGT  936

Query  490   AAGGTTCTTCGCGTTGCTTCGAATTAAACCACATGCTCCACCGCTTGTGCGGGCCCCCGT  549
             ||||||||||||||||||||||||||||||||||||||||||||||||||||||||||||
Sbjct  935   AAGGTTCTTCGCGTTGCTTCGAATTAAACCACATGCTCCACCGCTTGTGCGGGCCCCCGT  876

Query  550   CAATTCATTTGAGTTTTAACCTTGCGGCCGTACTCCCCAGGCGGTCAACTTAATGCGTTA  609
             ||||||||||||||||||||||||||||||||||||||||||||||||||||||||||||
Sbjct  875   CAATTCATTTGAGTTTTAACCTTGCGGCCGTACTCCCCAGGCGGTCAACTTAATGCGTTA  816

Query  610   GCTGCGCCACTAAGAGCTCAAGGCTCCCAACGGCTAGTTGACATCGTTTACGGCGTGGAC  669
             ||||||||||||||||||||||||||||||||||||||||||||||||||||||||||||
Sbjct  815   GCTGCGCCACTAAGAGCTCAAGGCTCCCAACGGCTAGTTGACATCGTTTACGGCGTGGAC  756

Query  670   TACCAGGGTATCTAATCCTGTTTGCTCCCCACGCTTTCGCACCTCAGTGTCAGTATCAGT  729
             ||||||||||||||||||||||||||||||||||||||||||||||||||||||||||||
Sbjct  755   TACCAGGGTATCTAATCCTGTTTGCTCCCCACGCTTTCGCACCTCAGTGTCAGTATCAGT  696

Query  730   CCAGGTGGTCGCCTTCGCCACTGGTGTTCCTTCCTATATCTACGCATTTCACCGCTACAC  789
             ||||||||||||||||||||||||||||||||||||||||||||||||||||||||||||
Sbjct  695   CCAGGTGGTCGCCTTCGCCACTGGTGTTCCTTCCTATATCTACGCATTTCACCGCTACAC  636

Query  790   AGGAAATTCCACCACCCTCTACCATACTCTAGCTCGACAGTTTTGAATGCAGTTCCCAGG  849
             ||||||||||||||||||||||||||||||||||||||||||||||||||||||||||||
Sbjct  635   AGGAAATTCCACCACCCTCTACCATACTCTAGCTCGACAGTTTTGAATGCAGTTCCCAGG  576

Query  850   TTGAGCCCGGGGCTTTCACATCCAACTTAACGAACCACCTACGCGCGCTTTACGCCCAGT  909
             ||||||||||||||||||||||||||||||||||||||||||||||||||||||||||||
Sbjct  575   TTGAGCCCGGGGCTTTCACATCCAACTTAACGAACCACCTACGCGCGCTTTACGCCCAGT  516

Query  910   AATTCCGATTAACGCTTGCACCCTCTGTATTACCGCGGCTGCTGGCACAGAGTTAGCCGG  969
             ||||||||||||||||||||||||||||||||||||||||||||||||||||||||||||
Sbjct  515   AATTCCGATTAACGCTTGCACCCTCTGTATTACCGCGGCTGCTGGCACAGAGTTAGCCGG  456

Query  970   TGCTTATTCTGTCGGTAACGTCAAANCACTAACGTATTAGGTTAATGCCCTTCCTCCCAA  1029
             ||||||||||||||||||||||||| ||||||||||||||||||||||||||||||||||
Sbjct  455   TGCTTATTCTGTCGGTAACGTCAAAACACTAACGTATTAGGTTAATGCCCTTCCTCCCAA  396

Query  1030  CTTAAAGTGCTTTNCAATCCGA  1051
             ||||||||||||| ||||||||
Sbjct  395   CTTAAAGTGCTTTACAATCCGA  374
```

Download

FASTA (complete sequence)

FASTA (aligned sequences)

GenBank (complete sequence)

Continue
Cancel

GenBankGraphics

Next
Previous
Descriptions

Pseudomonas brassicacearum strain Kr21 16S ribosomal RNA gene, partial sequence

Sequence ID: KT215482.1Length: 1410Number of Matches: 1

Related Information

Range 1: 360 to 1401GenBankGraphics

Next Match
Previous Match
First Match

Alignment statistics for match #1

| Score | Expect | Identities | Gaps | Strand | Frame |
| --- | --- | --- | --- | --- | --- |
| 1893 bits(1025) | 0.0() | 1034/1042(99%) | 0/1042(0%) | Plus/Minus |  |

Features:

```
Query  10    CGTCCTNNCGAAGGTTAGACTAGCTACTTCTGGTGCAACCCACTCCCATGGTGTGACGGG  69
             ||||||  ||||||||||||||||||||||||||||||||||||||||||||||||||||
Sbjct  1401  CGTCCTCCCGAAGGTTAGACTAGCTACTTCTGGTGCAACCCACTCCCATGGTGTGACGGG  1342

Query  70    CGGTGTGTACAAGGCCCGGGAACGTATTCACCGCGACATTCTGATTCGCGATTACTAGCG  129
             ||||||||||||||||||||||||||||||||||||||||||||||||||||||||||||
Sbjct  1341  CGGTGTGTACAAGGCCCGGGAACGTATTCACCGCGACATTCTGATTCGCGATTACTAGCG  1282

Query  130   ATTCCGACTTCACGCAGTCGAGTTGCAGACTGCGATCCGGACTACGATCGGTTTTGTGGG  189
             ||||||||||||||||||||||||||||||||||||||||||||||||||||||||||||
Sbjct  1281  ATTCCGACTTCACGCAGTCGAGTTGCAGACTGCGATCCGGACTACGATCGGTTTTGTGGG  1222

Query  190   ATTAGCTCCACCTCGCGGCTTGGCAACCCTCTGTACCGACCATTGTAGCACGTGTGTAGC  249
             ||||||||||||||||||||||||||||||||||||||||||||||||||||||||||||
Sbjct  1221  ATTAGCTCCACCTCGCGGCTTGGCAACCCTCTGTACCGACCATTGTAGCACGTGTGTAGC  1162

Query  250   CCAGGCCGTAAGGGCCATGATGACTTGACGTCATCCCCACCTTCCTCCGGTTTGTCACCG  309
             ||||||||||||||||||||||||||||||||||||||||||||||||||||||||||||
Sbjct  1161  CCAGGCCGTAAGGGCCATGATGACTTGACGTCATCCCCACCTTCCTCCGGTTTGTCACCG  1102

Query  310   GCAGTCTCCTTAGAGTGCCCACCATGACGTGCTGGTAACTAAGGACAAGGGTTGCGCTCG  369
             ||||||||||||||||||||||||||||||||||||||||||||||||||||||||||||
Sbjct  1101  GCAGTCTCCTTAGAGTGCCCACCATGACGTGCTGGTAACTAAGGACAAGGGTTGCGCTCG  1042

Query  370   TTACGGGACTTAACCCAACATCTCACGACACGAGCTGACGACAGCCATGCAGCACCTGTC  429
             ||||||||||||||||||||||||||||||||||||||||||||||||||||||||||||
Sbjct  1041  TTACGGGACTTAACCCAACATCTCACGACACGAGCTGACGACAGCCATGCAGCACCTGTC  982

Query  430   TCAATNNNCCCGAAGGCACCAATCCATCTCTGGAAAGTTCATTGGATGTCAAGGCCTGGT  489
             |||||   ||||||||||||||||||||||||||||||||||||||||||||||||||||
Sbjct  981   TCAATGTTCCCGAAGGCACCAATCCATCTCTGGAAAGTTCATTGGATGTCAAGGCCTGGT  922

Query  490   AAGGTTCTTCGCGTTGCTTCGAATTAAACCACATGCTCCACCGCTTGTGCGGGCCCCCGT  549
             ||||||||||||||||||||||||||||||||||||||||||||||||||||||||||||
Sbjct  921   AAGGTTCTTCGCGTTGCTTCGAATTAAACCACATGCTCCACCGCTTGTGCGGGCCCCCGT  862

Query  550   CAATTCATTTGAGTTTTAACCTTGCGGCCGTACTCCCCAGGCGGTCAACTTAATGCGTTA  609
             ||||||||||||||||||||||||||||||||||||||||||||||||||||||||||||
Sbjct  861   CAATTCATTTGAGTTTTAACCTTGCGGCCGTACTCCCCAGGCGGTCAACTTAATGCGTTA  802

Query  610   GCTGCGCCACTAAGAGCTCAAGGCTCCCAACGGCTAGTTGACATCGTTTACGGCGTGGAC  669
             ||||||||||||||||||||||||||||||||||||||||||||||||||||||||||||
Sbjct  801   GCTGCGCCACTAAGAGCTCAAGGCTCCCAACGGCTAGTTGACATCGTTTACGGCGTGGAC  742

Query  670   TACCAGGGTATCTAATCCTGTTTGCTCCCCACGCTTTCGCACCTCAGTGTCAGTATCAGT  729
             ||||||||||||||||||||||||||||||||||||||||||||||||||||||||||||
Sbjct  741   TACCAGGGTATCTAATCCTGTTTGCTCCCCACGCTTTCGCACCTCAGTGTCAGTATCAGT  682

Query  730   CCAGGTGGTCGCCTTCGCCACTGGTGTTCCTTCCTATATCTACGCATTTCACCGCTACAC  789
             ||||||||||||||||||||||||||||||||||||||||||||||||||||||||||||
Sbjct  681   CCAGGTGGTCGCCTTCGCCACTGGTGTTCCTTCCTATATCTACGCATTTCACCGCTACAC  622

Query  790   AGGAAATTCCACCACCCTCTACCATACTCTAGCTCGACAGTTTTGAATGCAGTTCCCAGG  849
             ||||||||||||||||||||||||||||||||||||||||||||||||||||||||||||
Sbjct  621   AGGAAATTCCACCACCCTCTACCATACTCTAGCTCGACAGTTTTGAATGCAGTTCCCAGG  562

Query  850   TTGAGCCCGGGGCTTTCACATCCAACTTAACGAACCACCTACGCGCGCTTTACGCCCAGT  909
             |||||||||||| |||||||||||||||||||||||||||||||||||||||||||||||
Sbjct  561   TTGAGCCCGGGGATTTCACATCCAACTTAACGAACCACCTACGCGCGCTTTACGCCCAGT  502

Query  910   AATTCCGATTAACGCTTGCACCCTCTGTATTACCGCGGCTGCTGGCACAGAGTTAGCCGG  969
             ||||||||||||||||||||||||||||||||||||||||||||||||||||||||||||
Sbjct  501   AATTCCGATTAACGCTTGCACCCTCTGTATTACCGCGGCTGCTGGCACAGAGTTAGCCGG  442

Query  970   TGCTTATTCTGTCGGTAACGTCAAANCACTAACGTATTAGGTTAATGCCCTTCCTCCCAA  1029
             ||||||||||||||||||||||||| ||||||||||||||||||||||||||||||||||
Sbjct  441   TGCTTATTCTGTCGGTAACGTCAAAACACTAACGTATTAGGTTAATGCCCTTCCTCCCAA  382

Query  1030  CTTAAAGTGCTTTNCAATCCGA  1051
             ||||||||||||| ||||||||
Sbjct  381   CTTAAAGTGCTTTACAATCCGA  360
```

```

```


BLAST is a registered trademark of the National Library of Medicine

Support center
Mailing list


YouTube

- National Library Of Medicine
- National Institutes Of Health
- U.S. Department of Health & Human Services
- USA.gov

### NCBI


National Center for Biotechnology Information,
 U.S. National Library of Medicine

8600 Rockville Pike,
Bethesda
 MD,
20894
USA

Policies and Guidelines
|
Contact


PreferencesTurn off

External link. Please review our privacy policy.
