# Supplementary material for: Isothermal microcalorimetry for thermal viable count of microorganisms in pure cultures and stabilized formulations
Source: BMC Microbiol. 2019 Mar 21;19:65. doi: 10.1186/s12866-019-1432-8 (PMC6429831; doi:10.1186/s12866-019-1432-8)
Supplement: Supplementary file 3 — 16S rRNA gene sequences’ BLASTN hits in zipped HTML format. (ZIP 15810 kb) [file 12866_2019_1432_MOESM3_ESM.zip › Best blastn hits/NCBI Blast_114 yellowish negative R -- 469..1070 of.html]

NCBI Blast:114 yellowish negative R -- 469..1070 of


- NCBI Home
- Sign in to NCBI
- Skip to Main Content
- Skip to Navigation
- About NCBI Accesskeys

U.S. National Library of Medicine

NCBI
National Center for Biotechnology Information

- My NCBI
- Sign in to NCBI
- Register
- Sign Out

BLAST ® » blastn suite » RID-A4X4TSHY015


- Home
- Recent Results
- Saved Strategies
- Help

BLAST Results


Edit and Resubmit
Save Search Strategies
[Sign in above to save your search strategy]

Formatting options 


Download


How to read this page
Blast report description
Questions/comments


|  |  |
| --- | --- |
| Formatting options | |
| Show | Alignment as  HTML Plain text   Old View Reset form to defaults [?]  These options control formatting of alignments in results pages. The default is HTML, but other formats (including plain text) are available. PSSM and PssmWithParameters are representations of Position Specific Scoring Matrices and are only available for PSI-BLAST. The Advanced view option allows the database descriptions to be sorted by various indices in a table. |
| Alignment View | Pairwise Pairwise with dots for identities Query-anchored with dots for identities Query-anchored with letters for identities Flat query-anchored with dots for identities Flat query-anchored with letters for identities [?]  Choose how to view alignments. The default "pairwise" view shows how each subject sequence aligns individually to the query sequence. The "query-anchored" view shows how all subject sequences align to the query sequence. For each view type, you can choose to show "identities" (matching residues) as letters or dots. more... |
| Display | Graphical Overview   Linkout   Sequence Retrieval  NCBI-gi   CDS feature [?]  - Graphical Overview: Graphical Overview: Show graph of similar sequence regions aligned to query.   more... - NCBI-gi: Show NCBI gi identifiers. - CDS feature: Show annotated coding region and translation.   more... |
| Masking | Character:   X for protein, n for nucleotide Lower Case  Color:  Black Grey Red [?]  - Masking Character: Display masked (filtered) sequence regions as lower-case or as specific letters (N for nucleotide, P for protein). - Masking Color: Display masked sequence regions in the given color. |
| Limit results | Descriptions:  10 50 100 Graphical overview:  0 10 50 100  Alignments:  0 10 50 100 Line length:  60 90 120 150 [?]  - Descriptions: Show short descriptions for up to the given number of sequences. - Alignments: Show alignments for up to the given number of sequences, in order of statistical significance. - Line lenghth: Number of letters to show on one line in an alignment. |
|  | Organism Type common name, binomial, taxid, or group name. Only 20 top taxa will be shown.     Exclude    [?]  Show only sequences from the given organism. |
|  | Entrez query:  [?]  Show only those sequences that match the given Entrez query. more... |
|  | Expect Min:  Expect Max:  [?]  Show only sequences with expect values in the given range. more... |
|  | Percent Identity Min:  Percent Identity Max:  [?]  Show only sequences with percent identity values in the given range. |
| Format for | PSI-BLAST with inclusion threshold:  [?]  - Format for PSI-BLAST: The Position-Specific Iterated BLAST (PSI-BLAST) program performs iterative searches with a protein query,   in which sequences found in one round of search are used to build a custom score model for the next round.   more... - Inclusion Threshold: This sets the statistical significance threshold for including a sequence in the model used   by PSI-BLAST to create the PSSM on the next iteration. |

|  |  |  |  |  |  |
| --- | --- | --- | --- | --- | --- |
| Download | | | | | |
| Alignment  Text XML ASN.1 JSON Seq-align Hit Table(text) Hit Table(csv) Multiple-file XML2 Single-file XML2 Multiple-file JSON Single-file JSON SAM | Search Strategies  ASN.1 | PSSM to restart search  PSSM | [?] |

The Download link provides BLAST output that may be used as input to another program.
This includes parseable formats such as the tabular report or XML as well as the Search Strategy files read by the BLAST+ applications.
More details on the parseable (XML, tabular, and ASN.1) reports can be found at
https://www.ncbi.nlm.nih.gov/books/NBK153387/  
  

The following formats are offered under the Alignment section:  
1). "Text". Non-HTML standard BLAST report.  
2). "XML". XML report based upon the DTD at https://www.ncbi.nlm.nih.gov/data\_specs/dtd/NCBI\_BlastOutput.dtd  
3). "ASN.1". Alignment written out in Abstract Syntax Notation 1.  
4). "JSON Seq-align". Alignment written out in JSON.  
4). "Hit Table(text)". The tabular report as text.  
5). "Hit Table(csv)". The tabular report ready for import into spread-sheet programs like Excel.  
6). "XML2". New XML format described at ftp://ftp.ncbi.nlm.nih.gov/blast/documents/NEWXML/xml2.pdf.  
7). "JSON". New JSON format described at ftp://ftp.ncbi.nlm.nih.gov/blast/documents/NEWXML/xml2.pdf.  
8). "SAM". Sequence Alignment Map format.

XML2 and JSON can be downloaded either as one file per query (multiple-file) or one file for all queries (single-file). These formats are listed as Multiple-file XML2 (and JSON) or Single-file XML (and JSON).

The following report is offered under the Search Strategy section:  
1). "ASN.1" Search Strategy. A record of the parameters, query, and database used in the search. This file can be used to start a stand-alone BLAST search, see
https://www.ncbi.nlm.nih.gov/books/NBK1763/#CmdLineAppsManual.I455\_BLAST\_search\_stra


# Job title: 114 yellowish negative R -- 469..1070 of

Results for:

lcl|Query\_62931 114 yellowish negative R -- 469..1070 of sequence(602bp)
[?]

Your BLAST job specified more than one input sequence.
This box lets you choose which input sequence to show BLAST results for.

RID
:   A4X4TSHY015 (Expires on 03-10 18:48 pm)

Query ID
:   lcl|Query\_62931
:   lcl|Query\_62931

Description
:   114 yellowish negative R -- 469..1070 of sequence

Molecule type
:   nucleic acid

Query Length
:   602

Database Name
:   nr

Description
:   Nucleotide collection (nt) See details

Program
:   BLASTN 2.8.0+ Citation

  

Reference 

Zheng Zhang, Scott Schwartz, Lukas Wagner, and Webb Miller (2000), "A greedy algorithm for aligning DNA sequences", J Comput Biol 2000; 7(1-2):203-14.

Reference - database indexing

Aleksandr Morgulis, George Coulouris, Yan Raytselis, Thomas L. Madden, Richa Agarwala, Alejandro A. Schäffer (2008), "Database Indexing for Production MegaBLAST Searches", Bioinformatics 24:1757-1764.

Other reports:
Search Summary

[Taxonomy reports]
[Distance tree of results]
[MSA viewer]

Search Parameters

| Search parameter name | Search parameter value |
| --- | --- |
| Program | blastn |
| Word size | 28 |
| Expect value | 10 |
| Hitlist size | 100 |
| Match/Mismatch scores | 1,-2 |
| Gapcosts | 0,2.5 |
| Low Complexity Filter | Yes |
| Filter string | L;m; |
| Genetic Code | 1 |

Database

| Database parameter name | Database parameter value |
| --- | --- |
| Posted date | Mar 7, 2018 1:58 PM |
| Number of letters | 174,044,644,244 |
| Number of sequences | 46,882,714 |
| Entrez query | Includes:  Excludes:  None |

Karlin-Altschul statistics

| Params | Ungapped | Gapped |
| --- | --- | --- |
| Lambda | 1.33271 | 1.28 |
| K | 0.620991 | 0.46 |
| H | 1.12409 | 0.85 |

Results Statistics

| Results Statistics parameter name | Results Statistics parameter value |
| --- | --- |
| Length adjustment | 34 |
| Effective length of query | 568 |
| Effective length of database | 172450631968 |
| Effective search space | 97951958957824 |
| Effective search space used | 97951958957824 |


## Graphic Summary

### Distribution of the top 112 Blast Hits on 100 subject sequences [?]

The graphic is an overview of the database sequences aligned to the query sequence. These are represented horizontal bars colored coded by score and showing the extent
of the alignment on the query sequence. Separate aligned regions on the same database sequence are connected by a thin grey line.
Mousing over an alignment shows the database sequence title. Clicking an alignment displays a box with more details about the alignment and
link to the sequence alignment itself in the Alignments section of the report.

Mouse over to see the title, click to show alignments

Color key for alignment scores

<40

40-50

50-80

80-200

>=200

Query

1

100

200

300

400

500

600

Pantoea vagans strain FDAARGOS\_160 chromosome, complete..

Score:1098 Evalue:0

Accession:CP014129.2

Alignment

Pantoea sp. strain AL38 16S ribosomal RNA gene, partial..

Score:1098 Evalue:0

Accession:MG819433.1

Alignment

Pantoea sp. strain AL226 16S ribosomal RNA gene, partia..

Score:1098 Evalue:0

Accession:MG819432.1

Alignment

Pantoea sp. strain AL273 16S ribosomal RNA gene, partia..

Score:1098 Evalue:0

Accession:MG819431.1

Alignment

Pantoea sp. strain AL269 16S ribosomal RNA gene, partia..

Score:1098 Evalue:0

Accession:MG819430.1

Alignment

Pantoea sp. strain AL264 16S ribosomal RNA gene, partia..

Score:1098 Evalue:0

Accession:MG819429.1

Alignment

Pantoea sp. strain AL249 16S ribosomal RNA gene, partia..

Score:1098 Evalue:0

Accession:MG819428.1

Alignment

Pantoea sp. strain AL268 16S ribosomal RNA gene, partia..

Score:1098 Evalue:0

Accession:MG819427.1

Alignment

Pantoea sp. strain AL28 16S ribosomal RNA gene, partial..

Score:1098 Evalue:0

Accession:MG819226.1

Alignment

Pantoea sp. strain AL147 16S ribosomal RNA gene, partia..

Score:1098 Evalue:0

Accession:MG819225.1

Alignment

Pantoea sp. strain AL165 16S ribosomal RNA gene, partia..

Score:1098 Evalue:0

Accession:MG819224.1

Alignment

Pantoea sp. strain AL102 16S ribosomal RNA gene, partia..

Score:1098 Evalue:0

Accession:MG819223.1

Alignment

Pantoea sp. strain AL64 16S ribosomal RNA gene, partial..

Score:1098 Evalue:0

Accession:MG819222.1

Alignment

Pantoea sp. strain AL121 16S ribosomal RNA gene, partia..

Score:1098 Evalue:0

Accession:MG819221.1

Alignment

Pantoea sp. strain AL132 16S ribosomal RNA gene, partia..

Score:1098 Evalue:0

Accession:MG819220.1

Alignment

Pantoea agglomerans strain 62 16S ribosomal RNA gene, p..

Score:1098 Evalue:0

Accession:MF767519.1

Alignment

Pantoea sp. strain 5 16S ribosomal RNA gene, partial se..

Score:1098 Evalue:0

Accession:KY446016.1

Alignment

Uncultured bacterium clone EHB-P0362 #05 16S ribosomal ..

Score:1098 Evalue:0

Accession:KU978258.1

Alignment

Pantoea vagans strain FPBBIH2 16S ribosomal RNA gene, p..

Score:1098 Evalue:0

Accession:KU605690.1

Alignment

Enterobacteriaceae bacterium Ku1409-7-6 gene for 16S rR..

Score:1098 Evalue:0

Accession:LC114113.1

Alignment

Uncultured Pantoea sp. clone GJ\_(2)\_73 16S ribosomal RN..

Score:1098 Evalue:0

Accession:KX603481.1

Alignment

Uncultured Pantoea sp. clone GJ\_(2)\_15 16S ribosomal RN..

Score:1098 Evalue:0

Accession:KX603457.1

Alignment

Bacterium DS4EC-385 16S ribosomal RNA gene, partial seq..

Score:1098 Evalue:0

Accession:KU726462.1

Alignment

Pantoea agglomerans strain TAbd1 16S ribosomal RNA gene..

Score:1098 Evalue:0

Accession:KT184494.1

Alignment

Pantoea agglomerans strain OI-16 16S ribosomal RNA gene..

Score:1098 Evalue:0

Accession:KT184492.1

Alignment

Pantoea agglomerans strain ZN-1 16S ribosomal RNA gene,..

Score:1098 Evalue:0

Accession:KT184491.1

Alignment

Pantoea agglomerans strain VRBG-21 16S ribosomal RNA ge..

Score:1098 Evalue:0

Accession:KR265414.1

Alignment

Pantoea agglomerans strain WSA 16S ribosomal RNA gene, ..

Score:1098 Evalue:0

Accession:KT075208.1

Alignment

Pantoea agglomerans strain SSC 16S ribosomal RNA gene, ..

Score:1098 Evalue:0

Accession:KT075191.1

Alignment

Pantoea agglomerans strain QSD 16S ribosomal RNA gene, ..

Score:1098 Evalue:0

Accession:KT075185.1

Alignment

Pantoea agglomerans strain NSD 16S ribosomal RNA gene, ..

Score:1098 Evalue:0

Accession:KT075169.1

Alignment

Pantoea agglomerans strain NSA 16S ribosomal RNA gene, ..

Score:1098 Evalue:0

Accession:KT075166.1

Alignment

Pantoea agglomerans strain GSC 16S ribosomal RNA gene, ..

Score:1098 Evalue:0

Accession:KT075163.1

Alignment

Enterobacteriaceae bacterium X1/SB86 gene for 16S ribos..

Score:1098 Evalue:0

Accession:LC007911.1

Alignment

Enterobacteriaceae bacterium X1/SB52 gene for 16S ribos..

Score:1098 Evalue:0

Accession:LC007878.1

Alignment

Enterobacteriaceae bacterium X1/SB44 gene for 16S ribos..

Score:1098 Evalue:0

Accession:LC007874.1

Alignment

Enterobacteriaceae bacterium X1/SB18 gene for 16S ribos..

Score:1098 Evalue:0

Accession:LC007863.1

Alignment

Uncultured bacterium clone nbw231h09c1 16S ribosomal RN..

Score:1098 Evalue:0

Accession:KF065296.1

Alignment

Pantoea agglomerans strain CC15 16S ribosomal RNA gene,..

Score:1098 Evalue:0

Accession:KJ016255.1

Alignment

Pantoea agglomerans partial 16S rRNA gene, strain LHR-0..

Score:1098 Evalue:0

Accession:HE716932.1

Alignment

Pantoea agglomerans strain PNG 06-1 16S ribosomal RNA g..

Score:1098 Evalue:0

Accession:KF805975.1

Alignment

Pantoea agglomerans partial 16S rRNA gene, isolate BFDP..

Score:1098 Evalue:0

Accession:HF584997.1

Alignment

Pantoea sp. W1.09-234-1 16S ribosomal RNA gene, complet..

Score:1098 Evalue:0

Accession:JX458430.1

Alignment

Pantoea sp. G2Ec2 16S ribosomal RNA gene, partial seque..

Score:1098 Evalue:0

Accession:KF465938.1

Alignment

Enterobacteriaceae bacterium SAP758.2 16S ribosomal RNA..

Score:1098 Evalue:0

Accession:JX067680.1

Alignment

Pantoea agglomerans strain -Y115 16S ribosomal RNA gene..

Score:1098 Evalue:0

Accession:JX134624.1

Alignment

Pantoea agglomerans strain +Y38 16S ribosomal RNA gene,..

Score:1098 Evalue:0

Accession:JX113251.1

Alignment

Pantoea agglomerans strain Y34 16S ribosomal RNA gene, ..

Score:1098 Evalue:0

Accession:JX113242.1

Alignment

Pantoea sp. SAP71\_2 16S ribosomal RNA gene, partial seq..

Score:1098 Evalue:0

Accession:JN872530.1

Alignment

Pantoea sp. SAP10\_1 16S ribosomal RNA gene, partial seq..

Score:1098 Evalue:0

Accession:JN872524.1

Alignment

Pantoea agglomerans partial 16S rRNA gene, strain LPPA ..

Score:1098 Evalue:0

Accession:HE613777.1

Alignment

Pantoea conspicua strain KNUC9005 16S ribosomal RNA gen..

Score:1098 Evalue:0

Accession:JF505939.1

Alignment

Enterobacter sp. P19-19 16S ribosomal RNA gene, partial..

Score:1098 Evalue:0

Accession:HQ439419.1

Alignment

Pantoea agglomerans isolate PSB27 16S ribosomal RNA gen..

Score:1098 Evalue:0

Accession:HQ242740.1

Alignment

Pantoea agglomerans isolate PSB26 16S ribosomal RNA gen..

Score:1098 Evalue:0

Accession:HQ242739.1

Alignment

Pantoea sp. 3DT5 16S ribosomal RNA gene, partial sequen..

Score:1098 Evalue:0

Accession:HQ849996.1

Alignment

## Descriptions

, Reading indexes 1-5, displaying indexes 1-5


Load next setPrevious Match

Sequences producing significant alignments:

Show all columns  of the table presenting sequences producing significant alignments 

Select:AllNone
Selected:0

Alignments
Download

FASTA (complete sequence)

FASTA (aligned sequences)

GenBank (complete sequence)

Hit Table (text)

Hit Table (CSV)

Text

XML

ASN.1

Continue
Cancel

GenBank 
Graphics
Distance tree of results
Multiple alignment
Show/hide columns of the table presenting sequences producing significant alignments 

Available columns

Description  
Max Score  
Total Score  
Coverage  
E-value  
IdentN  
Accession  
Restore Defaults
Ok
Cancel

Sequences producing significant alignments:

| Select for downloading or viewing reports | Description | Max score | Total score | Query cover | E value | Ident | Accession |
| --- | --- | --- | --- | --- | --- | --- | --- |
| 1Select seq CP014129.2 | Pantoea vagans strain FDAARGOS\_160 chromosome, complete genome | 1098 | 7619 | 99% | 0.0 | 99% | CP014129.2 |
| 2Select seq MG819433.1 | Pantoea sp. strain AL38 16S ribosomal RNA gene, partial sequence | 1098 | 1098 | 99% | 0.0 | 99% | MG819433.1 |
| 3Select seq MG819432.1 | Pantoea sp. strain AL226 16S ribosomal RNA gene, partial sequence | 1098 | 1098 | 99% | 0.0 | 99% | MG819432.1 |
| 4Select seq MG819431.1 | Pantoea sp. strain AL273 16S ribosomal RNA gene, partial sequence | 1098 | 1098 | 99% | 0.0 | 99% | MG819431.1 |
| 5Select seq MG819430.1 | Pantoea sp. strain AL269 16S ribosomal RNA gene, partial sequence | 1098 | 1098 | 99% | 0.0 | 99% | MG819430.1 |
| 6Select seq MG819429.1 | Pantoea sp. strain AL264 16S ribosomal RNA gene, partial sequence | 1098 | 1098 | 99% | 0.0 | 99% | MG819429.1 |
| 7Select seq MG819428.1 | Pantoea sp. strain AL249 16S ribosomal RNA gene, partial sequence | 1098 | 1098 | 99% | 0.0 | 99% | MG819428.1 |
| 8Select seq MG819427.1 | Pantoea sp. strain AL268 16S ribosomal RNA gene, partial sequence | 1098 | 1098 | 99% | 0.0 | 99% | MG819427.1 |
| 9Select seq MG819226.1 | Pantoea sp. strain AL28 16S ribosomal RNA gene, partial sequence | 1098 | 1098 | 99% | 0.0 | 99% | MG819226.1 |
| 10Select seq MG819225.1 | Pantoea sp. strain AL147 16S ribosomal RNA gene, partial sequence | 1098 | 1098 | 99% | 0.0 | 99% | MG819225.1 |
| 11Select seq MG819224.1 | Pantoea sp. strain AL165 16S ribosomal RNA gene, partial sequence | 1098 | 1098 | 99% | 0.0 | 99% | MG819224.1 |
| 12Select seq MG819223.1 | Pantoea sp. strain AL102 16S ribosomal RNA gene, partial sequence | 1098 | 1098 | 99% | 0.0 | 99% | MG819223.1 |
| 13Select seq MG819222.1 | Pantoea sp. strain AL64 16S ribosomal RNA gene, partial sequence | 1098 | 1098 | 99% | 0.0 | 99% | MG819222.1 |
| 14Select seq MG819221.1 | Pantoea sp. strain AL121 16S ribosomal RNA gene, partial sequence | 1098 | 1098 | 99% | 0.0 | 99% | MG819221.1 |
| 15Select seq MG819220.1 | Pantoea sp. strain AL132 16S ribosomal RNA gene, partial sequence | 1098 | 1098 | 99% | 0.0 | 99% | MG819220.1 |
| 16Select seq MF767519.1 | Pantoea agglomerans strain 62 16S ribosomal RNA gene, partial sequence | 1098 | 1098 | 99% | 0.0 | 99% | MF767519.1 |
| 17Select seq KY446016.1 | Pantoea sp. strain 5 16S ribosomal RNA gene, partial sequence | 1098 | 1098 | 99% | 0.0 | 99% | KY446016.1 |
| 18Select seq KU978258.1 | Uncultured bacterium clone EHB-P0362 #05 16S ribosomal RNA gene, partial sequence | 1098 | 1098 | 99% | 0.0 | 99% | KU978258.1 |
| 19Select seq KU605690.1 | Pantoea vagans strain FPBBIH2 16S ribosomal RNA gene, partial sequence | 1098 | 1098 | 99% | 0.0 | 99% | KU605690.1 |
| 20Select seq LC114113.1 | Enterobacteriaceae bacterium Ku1409-7-6 gene for 16S rRNA, partial sequence | 1098 | 1098 | 99% | 0.0 | 99% | LC114113.1 |
| 21Select seq KX603481.1 | Uncultured Pantoea sp. clone GJ\_(2)\_73 16S ribosomal RNA gene, partial sequence | 1098 | 1098 | 99% | 0.0 | 99% | KX603481.1 |
| 22Select seq KX603457.1 | Uncultured Pantoea sp. clone GJ\_(2)\_15 16S ribosomal RNA gene, partial sequence | 1098 | 1098 | 99% | 0.0 | 99% | KX603457.1 |
| 23Select seq KU726462.1 | Bacterium DS4EC-385 16S ribosomal RNA gene, partial sequence | 1098 | 1098 | 99% | 0.0 | 99% | KU726462.1 |
| 24Select seq KT184494.1 | Pantoea agglomerans strain TAbd1 16S ribosomal RNA gene, partial sequence | 1098 | 1098 | 99% | 0.0 | 99% | KT184494.1 |
| 25Select seq KT184492.1 | Pantoea agglomerans strain OI-16 16S ribosomal RNA gene, partial sequence | 1098 | 1098 | 99% | 0.0 | 99% | KT184492.1 |
| 26Select seq KT184491.1 | Pantoea agglomerans strain ZN-1 16S ribosomal RNA gene, partial sequence | 1098 | 1098 | 99% | 0.0 | 99% | KT184491.1 |
| 27Select seq KR265414.1 | Pantoea agglomerans strain VRBG-21 16S ribosomal RNA gene, partial sequence | 1098 | 1098 | 99% | 0.0 | 99% | KR265414.1 |
| 28Select seq KT075208.1 | Pantoea agglomerans strain WSA 16S ribosomal RNA gene, partial sequence | 1098 | 1098 | 99% | 0.0 | 99% | KT075208.1 |
| 29Select seq KT075191.1 | Pantoea agglomerans strain SSC 16S ribosomal RNA gene, partial sequence | 1098 | 1098 | 99% | 0.0 | 99% | KT075191.1 |
| 30Select seq KT075185.1 | Pantoea agglomerans strain QSD 16S ribosomal RNA gene, partial sequence | 1098 | 1098 | 99% | 0.0 | 99% | KT075185.1 |
| 31Select seq KT075169.1 | Pantoea agglomerans strain NSD 16S ribosomal RNA gene, partial sequence | 1098 | 1098 | 99% | 0.0 | 99% | KT075169.1 |
| 32Select seq KT075166.1 | Pantoea agglomerans strain NSA 16S ribosomal RNA gene, partial sequence | 1098 | 1098 | 99% | 0.0 | 99% | KT075166.1 |
| 33Select seq KT075163.1 | Pantoea agglomerans strain GSC 16S ribosomal RNA gene, partial sequence | 1098 | 1098 | 99% | 0.0 | 99% | KT075163.1 |
| 34Select seq LC007911.1 | Enterobacteriaceae bacterium X1/SB86 gene for 16S ribosomal RNA, partial sequence, strain: X1/SB86 | 1098 | 1098 | 99% | 0.0 | 99% | LC007911.1 |
| 35Select seq LC007878.1 | Enterobacteriaceae bacterium X1/SB52 gene for 16S ribosomal RNA, partial sequence, strain: X1/SB52 | 1098 | 1098 | 99% | 0.0 | 99% | LC007878.1 |
| 36Select seq LC007874.1 | Enterobacteriaceae bacterium X1/SB44 gene for 16S ribosomal RNA, partial sequence, strain: X1/SB44 | 1098 | 1098 | 99% | 0.0 | 99% | LC007874.1 |
| 37Select seq LC007863.1 | Enterobacteriaceae bacterium X1/SB18 gene for 16S ribosomal RNA, partial sequence, strain: X1/SB18 | 1098 | 1098 | 99% | 0.0 | 99% | LC007863.1 |
| 38Select seq KF065296.1 | Uncultured bacterium clone nbw231h09c1 16S ribosomal RNA gene, partial sequence | 1098 | 1098 | 99% | 0.0 | 99% | KF065296.1 |
| 39Select seq KJ016255.1 | Pantoea agglomerans strain CC15 16S ribosomal RNA gene, partial sequence | 1098 | 1098 | 99% | 0.0 | 99% | KJ016255.1 |
| 40Select seq HE716932.1 | Pantoea agglomerans partial 16S rRNA gene, strain LHR-06 | 1098 | 1098 | 99% | 0.0 | 99% | HE716932.1 |
| 41Select seq KF805975.1 | Pantoea agglomerans strain PNG 06-1 16S ribosomal RNA gene, partial sequence | 1098 | 1098 | 99% | 0.0 | 99% | KF805975.1 |
| 42Select seq HF584997.1 | Pantoea agglomerans partial 16S rRNA gene, isolate BFDP-S04 | 1098 | 1098 | 99% | 0.0 | 99% | HF584997.1 |
| 43Select seq JX458430.1 | Pantoea sp. W1.09-234-1 16S ribosomal RNA gene, complete sequence | 1098 | 1098 | 99% | 0.0 | 99% | JX458430.1 |
| 44Select seq KF465938.1 | Pantoea sp. G2Ec2 16S ribosomal RNA gene, partial sequence | 1098 | 1098 | 99% | 0.0 | 99% | KF465938.1 |
| 45Select seq JX067680.1 | Enterobacteriaceae bacterium SAP758.2 16S ribosomal RNA gene, partial sequence | 1098 | 1098 | 99% | 0.0 | 99% | JX067680.1 |
| 46Select seq JX134624.1 | Pantoea agglomerans strain -Y115 16S ribosomal RNA gene, partial sequence | 1098 | 1098 | 99% | 0.0 | 99% | JX134624.1 |
| 47Select seq JX113251.1 | Pantoea agglomerans strain +Y38 16S ribosomal RNA gene, partial sequence | 1098 | 1098 | 99% | 0.0 | 99% | JX113251.1 |
| 48Select seq JX113242.1 | Pantoea agglomerans strain Y34 16S ribosomal RNA gene, partial sequence | 1098 | 1098 | 99% | 0.0 | 99% | JX113242.1 |
| 49Select seq JN872530.1 | Pantoea sp. SAP71\_2 16S ribosomal RNA gene, partial sequence | 1098 | 1098 | 99% | 0.0 | 99% | JN872530.1 |
| 50Select seq JN872524.1 | Pantoea sp. SAP10\_1 16S ribosomal RNA gene, partial sequence | 1098 | 1098 | 99% | 0.0 | 99% | JN872524.1 |
| 51Select seq HE613777.1 | Pantoea agglomerans partial 16S rRNA gene, strain LPPA 1486 | 1098 | 1098 | 99% | 0.0 | 99% | HE613777.1 |
| 52Select seq JF505939.1 | Pantoea conspicua strain KNUC9005 16S ribosomal RNA gene, partial sequence | 1098 | 1098 | 99% | 0.0 | 99% | JF505939.1 |
| 53Select seq HQ439419.1 | Enterobacter sp. P19-19 16S ribosomal RNA gene, partial sequence | 1098 | 1098 | 99% | 0.0 | 99% | HQ439419.1 |
| 54Select seq HQ242740.1 | Pantoea agglomerans isolate PSB27 16S ribosomal RNA gene, partial sequence | 1098 | 1098 | 99% | 0.0 | 99% | HQ242740.1 |
| 55Select seq HQ242739.1 | Pantoea agglomerans isolate PSB26 16S ribosomal RNA gene, partial sequence | 1098 | 1098 | 99% | 0.0 | 99% | HQ242739.1 |
| 56Select seq HQ849996.1 | Pantoea sp. 3DT5 16S ribosomal RNA gene, partial sequence | 1098 | 1098 | 99% | 0.0 | 99% | HQ849996.1 |
| 57Select seq CP002206.1 | Pantoea vagans C9-1, complete genome | 1098 | 7621 | 99% | 0.0 | 99% | CP002206.1 |
| 58Select seq HM130689.1 | Pantoea agglomerans strain 1.2244 16S ribosomal RNA gene, partial sequence | 1098 | 1098 | 99% | 0.0 | 99% | HM130689.1 |
| 59Select seq HM306352.1 | Uncultured bacterium clone ncd864h02c1 16S ribosomal RNA gene, partial sequence | 1098 | 1098 | 99% | 0.0 | 99% | HM306352.1 |
| 60Select seq HM306298.1 | Uncultured bacterium clone ncd864d07c1 16S ribosomal RNA gene, partial sequence | 1098 | 1098 | 99% | 0.0 | 99% | HM306298.1 |
| 61Select seq HM306134.1 | Uncultured bacterium clone ncd861f07c1 16S ribosomal RNA gene, partial sequence | 1098 | 1098 | 99% | 0.0 | 99% | HM306134.1 |
| 62Select seq FN814227.1 | Uncultured bacterium partial 16S rRNA gene, clone 8B2-C9 | 1098 | 1098 | 99% | 0.0 | 99% | FN814227.1 |
| 63Select seq FN814199.1 | Uncultured bacterium partial 16S rRNA gene, clone 8B1-H10 | 1098 | 1098 | 99% | 0.0 | 99% | FN814199.1 |
| 64Select seq FN814191.1 | Uncultured bacterium partial 16S rRNA gene, clone 8B1-G7 | 1098 | 1098 | 99% | 0.0 | 99% | FN814191.1 |
| 65Select seq FN814138.1 | Uncultured bacterium partial 16S rRNA gene, clone 8B1-A3 | 1098 | 1098 | 99% | 0.0 | 99% | FN814138.1 |
| 66Select seq FN814047.1 | Uncultured bacterium partial 16S rRNA gene, clone 26B2-F4 | 1098 | 1098 | 99% | 0.0 | 99% | FN814047.1 |
| 67Select seq FN813970.1 | Uncultured bacterium partial 16S rRNA gene, clone 26B1-C8 | 1098 | 1098 | 99% | 0.0 | 99% | FN813970.1 |
| 68Select seq FN813924.1 | Uncultured bacterium partial 16S rRNA gene, clone 2.00E+07 | 1098 | 1098 | 99% | 0.0 | 99% | FN813924.1 |
| 69Select seq FN421868.1 | Uncultured bacterium partial 16S rRNA gene, clone 7\_G04 | 1098 | 1098 | 99% | 0.0 | 99% | FN421868.1 |
| 70Select seq FJ756348.1 | Pantoea agglomerans strain NZ 16S ribosomal RNA gene, partial sequence | 1098 | 1098 | 99% | 0.0 | 99% | FJ756348.1 |
| 71Select seq FJ611844.1 | Pantoea agglomerans strain P6WAL 16S ribosomal RNA gene, partial sequence | 1098 | 1098 | 99% | 0.0 | 99% | FJ611844.1 |
| 72Select seq FJ357836.1 | Pantoea sp. SB547 16S ribosomal RNA gene, partial sequence | 1098 | 1098 | 99% | 0.0 | 99% | FJ357836.1 |
| 73Select seq FJ357826.1 | Pantoea agglomerans strain PA18 16S ribosomal RNA gene, partial sequence | 1098 | 1098 | 99% | 0.0 | 99% | FJ357826.1 |
| 74Select seq FJ357811.1 | Pantoea agglomerans strain BBPE277471 16S ribosomal RNA gene, partial sequence | 1098 | 1098 | 99% | 0.0 | 99% | FJ357811.1 |
| 75Select seq EU130700.1 | Pantoea agglomerans strain PGHL23-15 16S ribosomal RNA gene, partial sequence | 1098 | 1098 | 99% | 0.0 | 99% | EU130700.1 |
| 76Select seq EU130699.1 | Pantoea agglomerans strain PGHL14-11 16S ribosomal RNA gene, partial sequence | 1098 | 1098 | 99% | 0.0 | 99% | EU130699.1 |
| 77Select seq AB244440.1 | Pantoea sp. An4-1 gene for 16S rRNA, partial sequence, strain: An4-1 | 1098 | 1098 | 99% | 0.0 | 99% | AB244440.1 |
| 78Select seq EF050810.1 | Pantoea agglomerans strain PGHL6 16S ribosomal RNA gene, partial sequence | 1098 | 1098 | 99% | 0.0 | 99% | EF050810.1 |
| 79Select seq EF050806.1 | Pantoea agglomerans strain PGHLT4 16S ribosomal RNA gene, partial sequence | 1098 | 1098 | 99% | 0.0 | 99% | EF050806.1 |
| 80Select seq DQ068762.1 | Pantoea sp. Co9941 16S ribosomal RNA gene, partial sequence | 1098 | 1098 | 99% | 0.0 | 99% | DQ068762.1 |
| 81Select seq DQ307453.1 | Pantoea agglomerans strain MM2 16S ribosomal RNA gene, partial sequence | 1098 | 1098 | 99% | 0.0 | 99% | DQ307453.1 |
| 82Select seq AM050142.1 | Pantoea agglomerans partial 16S rRNA gene | 1098 | 1098 | 99% | 0.0 | 99% | AM050142.1 |
| 83Select seq AM050140.1 | Pantoea agglomerans partial 16S rRNA gene | 1098 | 1098 | 99% | 0.0 | 99% | AM050140.1 |
| 84Select seq AM050139.1 | Pantoea agglomerans partial 16S rRNA gene | 1098 | 1098 | 99% | 0.0 | 99% | AM050139.1 |
| 85Select seq AB004759.1 | Uncultured bacterium gene for 16S ribosomal RNA, partial sequence, Bacterium T | 1098 | 1098 | 99% | 0.0 | 99% | AB004759.1 |
| 86Select seq AB004757.2 | Pantoea agglomerans gene for 16S ribosomal RNA, partial sequence | 1098 | 1098 | 99% | 0.0 | 99% | AB004757.2 |
| 87Select seq AF130946.1 | Enterobacter agglomerans strain A81 16S ribosomal RNA, partial sequence | 1098 | 1098 | 99% | 0.0 | 99% | AF130946.1 |
| 88Select seq KC283045.1 | Uncultured Pantoea sp. clone WCFC2 16S ribosomal RNA gene, partial sequence | 1094 | 1094 | 99% | 0.0 | 99% | KC283045.1 |
| 89Select seq FJ357814.1 | Pantoea agglomerans strain CUETM8553 16S ribosomal RNA gene, partial sequence | 1094 | 1094 | 99% | 0.0 | 99% | FJ357814.1 |
| 90Select seq AF130925.1 | Enterobacter agglomerans strain A55 16S ribosomal RNA, partial sequence | 1094 | 1094 | 99% | 0.0 | 99% | AF130925.1 |
| 91Select seq MG607395.1 | Pantoea vagans strain R5R 16S ribosomal RNA gene, partial sequence | 1092 | 1092 | 99% | 0.0 | 99% | MG607395.1 |
| 92Select seq KY604954.1 | Pantoea agglomerans strain Z-C 16S ribosomal RNA gene, partial sequence | 1092 | 1092 | 99% | 0.0 | 99% | KY604954.1 |
| 93Select seq MF407401.1 | Pantoea agglomerans strain BE 16S ribosimal RNA gene, partial sequence | 1092 | 1092 | 99% | 0.0 | 99% | MF407401.1 |
| 94Select seq KY883992.1 | Pantoea sp. strain DE006 16S ribosomal RNA gene, partial sequence | 1092 | 1092 | 99% | 0.0 | 99% | KY883992.1 |
| 95Select seq KU978278.1 | Uncultured bacterium clone EHB-P0445 #05 16S ribosomal RNA gene, partial sequence | 1092 | 1092 | 99% | 0.0 | 99% | KU978278.1 |
| 96Select seq KT184493.1 | Pantoea vagans strain TD19 16S ribosomal RNA gene, partial sequence | 1092 | 1092 | 99% | 0.0 | 99% | KT184493.1 |
| 97Select seq KT075186.1 | Pantoea agglomerans strain QSE 16S ribosomal RNA gene, partial sequence | 1092 | 1092 | 99% | 0.0 | 99% | KT075186.1 |
| 98Select seq KP279978.1 | Curtobacterium plantarum strain NS1 16S ribosomal RNA gene, partial sequence | 1092 | 1092 | 99% | 0.0 | 99% | KP279978.1 |
| 99Select seq KP279964.1 | Curtobacterium plantarum strain KS1 16S ribosomal RNA gene, partial sequence | 1092 | 1092 | 99% | 0.0 | 99% | KP279964.1 |
| 100Select seq KP736169.1 | Pantoea agglomerans strain YJ3 16S ribosomal RNA gene, complete sequence | 1088 | 1088 | 98% | 0.0 | 99% | KP736169.1 |


## Alignments

Loading alignment... for sequences gi|1344462721,gi|1332982595,gi|1332982594,gi|1332982593,gi|1332982592 Reading indexes 1-5

Download

FASTA (complete sequence)

FASTA (aligned sequences)

GenBank (complete sequence)

Continue
Cancel

GenBankGraphics

Sort by:

E value
 Score
Percent identity
Query start position
Subject start position

Next
Previous
Descriptions

Pantoea vagans strain FDAARGOS\_160 chromosome, complete genome

Sequence ID: CP014129.2Length: 4050121Number of Matches: 7

Related Information

Range 1: 50224 to 50819GenBankGraphics

Next Match
Previous Match
First Match

Alignment statistics for match #1

| Score | Expect | Identities | Gaps | Strand | Frame |
| --- | --- | --- | --- | --- | --- |
| 1098 bits(594) | 0.0() | 595/596(99%) | 0/596(0%) | Plus/Plus |  |

Features:

```
Query  5      TTCCGTGGATGTCAAGAGTAGGTAAGGTTCTTCGCGTTGCATCGAATTAAACCACATGCT  64
              ||||||||||||||||||||||||||||||||||||||||||||||||||||||||||||
Sbjct  50224  TTCCGTGGATGTCAAGAGTAGGTAAGGTTCTTCGCGTTGCATCGAATTAAACCACATGCT  50283

Query  65     CCACCGCTTGTGCGGGCCCCCGTCAATTCATTTGAGTTTTAACCTTGCGGCCGTACTCCC  124
              ||||||||||||||||||||||||||||||||||||||||||||||||||||||||||||
Sbjct  50284  CCACCGCTTGTGCGGGCCCCCGTCAATTCATTTGAGTTTTAACCTTGCGGCCGTACTCCC  50343

Query  125    CAGGCGGTCGACTTAACGCGTTAGCTCCGGAAGCCACTCCTCAAGGGAACAACCTCCAAG  184
              ||||||||||||||||||||||||||||||||||||||||||||||||||||||||||||
Sbjct  50344  CAGGCGGTCGACTTAACGCGTTAGCTCCGGAAGCCACTCCTCAAGGGAACAACCTCCAAG  50403

Query  185    TCGACATCGTTTACGGCGTGGACTACCAGGGTATCTAATCCTGTTTGCTCCCCACGCTTT  244
              ||||||||||||||||||||||||||||||||||||||||||||||||||||||||||||
Sbjct  50404  TCGACATCGTTTACGGCGTGGACTACCAGGGTATCTAATCCTGTTTGCTCCCCACGCTTT  50463

Query  245    CGCACCTGAGCGTCAGTCTTTGTCCAGGGGGCCGCCTTCGCCACCGGTATTCCTCCAGAT  304
              ||||||||||||||||||||||||||||||||||||||||||||||||||||||||||||
Sbjct  50464  CGCACCTGAGCGTCAGTCTTTGTCCAGGGGGCCGCCTTCGCCACCGGTATTCCTCCAGAT  50523

Query  305    CTCTACGCATTTCACCGCTACACCTGGAATTCTACCCCCCTCTACAAGACTCAAGCCTGC  364
              ||||||||||||||||||||||||||||||||||||||||||||||||||||||||||||
Sbjct  50524  CTCTACGCATTTCACCGCTACACCTGGAATTCTACCCCCCTCTACAAGACTCAAGCCTGC  50583

Query  365    CAGTTTCAAATGCAGTTCCCAGGTTAAGCCCGGGGATTTCACATCTGACTTAACAGACCG  424
              ||||||||||||||||||||||||||||||||||||||||||||||||||||||||||||
Sbjct  50584  CAGTTTCAAATGCAGTTCCCAGGTTAAGCCCGGGGATTTCACATCTGACTTAACAGACCG  50643

Query  425    CCTGCGTGCGCTTTACGCCCAGTAATTCCGATTAACGCTTGCACCCTCCGTATTACCGCG  484
              ||||||||||||||||||||||||||||||||||||||||||||||||||||||||||||
Sbjct  50644  CCTGCGTGCGCTTTACGCCCAGTAATTCCGATTAACGCTTGCACCCTCCGTATTACCGCG  50703

Query  485    GCTGCTGGCACGGAGTTAGCCGGTGCTTCTTCTGCGGGTAACGTCAATCGACGCGGTTAT  544
              ||||||||||||||||||||||||||||||||||||||||||||||||||||||||||||
Sbjct  50704  GCTGCTGGCACGGAGTTAGCCGGTGCTTCTTCTGCGGGTAACGTCAATCGACGCGGTTAT  50763

Query  545    TAACCNCATCGCCTTCCTCCCCGCTGAAAGTACTTTACAACCCGAAGGCCTTCTTC  600
              ||||| ||||||||||||||||||||||||||||||||||||||||||||||||||
Sbjct  50764  TAACCGCATCGCCTTCCTCCCCGCTGAAAGTACTTTACAACCCGAAGGCCTTCTTC  50819
```

Range 2: 171492 to 172087GenBankGraphics

Next Match
Previous Match
First Match

Alignment statistics for match #2

| Score | Expect | Identities | Gaps | Strand | Frame |
| --- | --- | --- | --- | --- | --- |
| 1086 bits(588) | 0.0() | 593/596(99%) | 0/596(0%) | Plus/Plus |  |

Features:

```
Query  5       TTCCGTGGATGTCAAGAGTAGGTAAGGTTCTTCGCGTTGCATCGAATTAAACCACATGCT  64
               ||||||||||||||||||||||||||||||||||||||||||||||||||||||||||||
Sbjct  171492  TTCCGTGGATGTCAAGAGTAGGTAAGGTTCTTCGCGTTGCATCGAATTAAACCACATGCT  171551

Query  65      CCACCGCTTGTGCGGGCCCCCGTCAATTCATTTGAGTTTTAACCTTGCGGCCGTACTCCC  124
               ||||||||||||||||||||||||||||||||||||||||||||||||||||||||||||
Sbjct  171552  CCACCGCTTGTGCGGGCCCCCGTCAATTCATTTGAGTTTTAACCTTGCGGCCGTACTCCC  171611

Query  125     CAGGCGGTCGACTTAACGCGTTAGCTCCGGAAGCCACTCCTCAAGGGAACAACCTCCAAG  184
               ||||||||||||||||||||||||||||||||||||||||||||||||||||||||||||
Sbjct  171612  CAGGCGGTCGACTTAACGCGTTAGCTCCGGAAGCCACTCCTCAAGGGAACAACCTCCAAG  171671

Query  185     TCGACATCGTTTACGGCGTGGACTACCAGGGTATCTAATCCTGTTTGCTCCCCACGCTTT  244
               ||||||||||||||||||||||||||||||||||||||||||||||||||||||||||||
Sbjct  171672  TCGACATCGTTTACGGCGTGGACTACCAGGGTATCTAATCCTGTTTGCTCCCCACGCTTT  171731

Query  245     CGCACCTGAGCGTCAGTCTTTGTCCAGGGGGCCGCCTTCGCCACCGGTATTCCTCCAGAT  304
               ||||||||||||||||||||||||||||||||||||||||||||||||||||||||||||
Sbjct  171732  CGCACCTGAGCGTCAGTCTTTGTCCAGGGGGCCGCCTTCGCCACCGGTATTCCTCCAGAT  171791

Query  305     CTCTACGCATTTCACCGCTACACCTGGAATTCTACCCCCCTCTACAAGACTCAAGCCTGC  364
               ||||||||||||||||||||||||||||||||||||||||||||||||||||||||||||
Sbjct  171792  CTCTACGCATTTCACCGCTACACCTGGAATTCTACCCCCCTCTACAAGACTCAAGCCTGC  171851

Query  365     CAGTTTCAAATGCAGTTCCCAGGTTAAGCCCGGGGATTTCACATCTGACTTAACAGACCG  424
               ||||||||||||||||||||||||||||||||||||||||||||||||||||||||||||
Sbjct  171852  CAGTTTCAAATGCAGTTCCCAGGTTAAGCCCGGGGATTTCACATCTGACTTAACAGACCG  171911

Query  425     CCTGCGTGCGCTTTACGCCCAGTAATTCCGATTAACGCTTGCACCCTCCGTATTACCGCG  484
               ||||||||||||||||||||||||||||||||||||||||||||||||||||||||||||
Sbjct  171912  CCTGCGTGCGCTTTACGCCCAGTAATTCCGATTAACGCTTGCACCCTCCGTATTACCGCG  171971

Query  485     GCTGCTGGCACGGAGTTAGCCGGTGCTTCTTCTGCGGGTAACGTCAATCGACGCGGTTAT  544
               |||||||||||||||||||||||||||||||||||||||||||||||||| |||||||||
Sbjct  171972  GCTGCTGGCACGGAGTTAGCCGGTGCTTCTTCTGCGGGTAACGTCAATCGGCGCGGTTAT  172031

Query  545     TAACCNCATCGCCTTCCTCCCCGCTGAAAGTACTTTACAACCCGAAGGCCTTCTTC  600
               ||||| || |||||||||||||||||||||||||||||||||||||||||||||||
Sbjct  172032  TAACCGCACCGCCTTCCTCCCCGCTGAAAGTACTTTACAACCCGAAGGCCTTCTTC  172087
```

Range 3: 410916 to 411511GenBankGraphics

Next Match
Previous Match
First Match

Alignment statistics for match #3

| Score | Expect | Identities | Gaps | Strand | Frame |
| --- | --- | --- | --- | --- | --- |
| 1086 bits(588) | 0.0() | 593/596(99%) | 0/596(0%) | Plus/Minus |  |

Features:

```
Query  5       TTCCGTGGATGTCAAGAGTAGGTAAGGTTCTTCGCGTTGCATCGAATTAAACCACATGCT  64
               ||||||||||||||||||||||||||||||||||||||||||||||||||||||||||||
Sbjct  411511  TTCCGTGGATGTCAAGAGTAGGTAAGGTTCTTCGCGTTGCATCGAATTAAACCACATGCT  411452

Query  65      CCACCGCTTGTGCGGGCCCCCGTCAATTCATTTGAGTTTTAACCTTGCGGCCGTACTCCC  124
               ||||||||||||||||||||||||||||||||||||||||||||||||||||||||||||
Sbjct  411451  CCACCGCTTGTGCGGGCCCCCGTCAATTCATTTGAGTTTTAACCTTGCGGCCGTACTCCC  411392

Query  125     CAGGCGGTCGACTTAACGCGTTAGCTCCGGAAGCCACTCCTCAAGGGAACAACCTCCAAG  184
               ||||||||||||||||||||||||||||||||||||||||||||||||||||||||||||
Sbjct  411391  CAGGCGGTCGACTTAACGCGTTAGCTCCGGAAGCCACTCCTCAAGGGAACAACCTCCAAG  411332

Query  185     TCGACATCGTTTACGGCGTGGACTACCAGGGTATCTAATCCTGTTTGCTCCCCACGCTTT  244
               ||||||||||||||||||||||||||||||||||||||||||||||||||||||||||||
Sbjct  411331  TCGACATCGTTTACGGCGTGGACTACCAGGGTATCTAATCCTGTTTGCTCCCCACGCTTT  411272

Query  245     CGCACCTGAGCGTCAGTCTTTGTCCAGGGGGCCGCCTTCGCCACCGGTATTCCTCCAGAT  304
               ||||||||||||||||||||||||||||||||||||||||||||||||||||||||||||
Sbjct  411271  CGCACCTGAGCGTCAGTCTTTGTCCAGGGGGCCGCCTTCGCCACCGGTATTCCTCCAGAT  411212

Query  305     CTCTACGCATTTCACCGCTACACCTGGAATTCTACCCCCCTCTACAAGACTCAAGCCTGC  364
               ||||||||||||||||||||||||||||||||||||||||||||||||||||||||||||
Sbjct  411211  CTCTACGCATTTCACCGCTACACCTGGAATTCTACCCCCCTCTACAAGACTCAAGCCTGC  411152

Query  365     CAGTTTCAAATGCAGTTCCCAGGTTAAGCCCGGGGATTTCACATCTGACTTAACAGACCG  424
               ||||||||||||||||||||||||||||||||||||||||||||||||||||||||||||
Sbjct  411151  CAGTTTCAAATGCAGTTCCCAGGTTAAGCCCGGGGATTTCACATCTGACTTAACAGACCG  411092

Query  425     CCTGCGTGCGCTTTACGCCCAGTAATTCCGATTAACGCTTGCACCCTCCGTATTACCGCG  484
               ||||||||||||||||||||||||||||||||||||||||||||||||||||||||||||
Sbjct  411091  CCTGCGTGCGCTTTACGCCCAGTAATTCCGATTAACGCTTGCACCCTCCGTATTACCGCG  411032

Query  485     GCTGCTGGCACGGAGTTAGCCGGTGCTTCTTCTGCGGGTAACGTCAATCGACGCGGTTAT  544
               |||||||||||||||||||||||||||||||||||||||||||||||||| |||||||||
Sbjct  411031  GCTGCTGGCACGGAGTTAGCCGGTGCTTCTTCTGCGGGTAACGTCAATCGGCGCGGTTAT  410972

Query  545     TAACCNCATCGCCTTCCTCCCCGCTGAAAGTACTTTACAACCCGAAGGCCTTCTTC  600
               ||||| || |||||||||||||||||||||||||||||||||||||||||||||||
Sbjct  410971  TAACCGCACCGCCTTCCTCCCCGCTGAAAGTACTTTACAACCCGAAGGCCTTCTTC  410916
```

Range 4: 447625 to 448220GenBankGraphics

Next Match
Previous Match
First Match

Alignment statistics for match #4

| Score | Expect | Identities | Gaps | Strand | Frame |
| --- | --- | --- | --- | --- | --- |
| 1086 bits(588) | 0.0() | 593/596(99%) | 0/596(0%) | Plus/Minus |  |

Features:

```
Query  5       TTCCGTGGATGTCAAGAGTAGGTAAGGTTCTTCGCGTTGCATCGAATTAAACCACATGCT  64
               ||||||||||||||||||||||||||||||||||||||||||||||||||||||||||||
Sbjct  448220  TTCCGTGGATGTCAAGAGTAGGTAAGGTTCTTCGCGTTGCATCGAATTAAACCACATGCT  448161

Query  65      CCACCGCTTGTGCGGGCCCCCGTCAATTCATTTGAGTTTTAACCTTGCGGCCGTACTCCC  124
               ||||||||||||||||||||||||||||||||||||||||||||||||||||||||||||
Sbjct  448160  CCACCGCTTGTGCGGGCCCCCGTCAATTCATTTGAGTTTTAACCTTGCGGCCGTACTCCC  448101

Query  125     CAGGCGGTCGACTTAACGCGTTAGCTCCGGAAGCCACTCCTCAAGGGAACAACCTCCAAG  184
               ||||||||||||||||||||||||||||||||||||||||||||||||||||||||||||
Sbjct  448100  CAGGCGGTCGACTTAACGCGTTAGCTCCGGAAGCCACTCCTCAAGGGAACAACCTCCAAG  448041

Query  185     TCGACATCGTTTACGGCGTGGACTACCAGGGTATCTAATCCTGTTTGCTCCCCACGCTTT  244
               ||||||||||||||||||||||||||||||||||||||||||||||||||||||||||||
Sbjct  448040  TCGACATCGTTTACGGCGTGGACTACCAGGGTATCTAATCCTGTTTGCTCCCCACGCTTT  447981

Query  245     CGCACCTGAGCGTCAGTCTTTGTCCAGGGGGCCGCCTTCGCCACCGGTATTCCTCCAGAT  304
               ||||||||||||||||||||||||||||||||||||||||||||||||||||||||||||
Sbjct  447980  CGCACCTGAGCGTCAGTCTTTGTCCAGGGGGCCGCCTTCGCCACCGGTATTCCTCCAGAT  447921

Query  305     CTCTACGCATTTCACCGCTACACCTGGAATTCTACCCCCCTCTACAAGACTCAAGCCTGC  364
               ||||||||||||||||||||||||||||||||||||||||||||||||||||||||||||
Sbjct  447920  CTCTACGCATTTCACCGCTACACCTGGAATTCTACCCCCCTCTACAAGACTCAAGCCTGC  447861

Query  365     CAGTTTCAAATGCAGTTCCCAGGTTAAGCCCGGGGATTTCACATCTGACTTAACAGACCG  424
               ||||||||||||||||||||||||||||||||||||||||||||||||||||||||||||
Sbjct  447860  CAGTTTCAAATGCAGTTCCCAGGTTAAGCCCGGGGATTTCACATCTGACTTAACAGACCG  447801

Query  425     CCTGCGTGCGCTTTACGCCCAGTAATTCCGATTAACGCTTGCACCCTCCGTATTACCGCG  484
               ||||||||||||||||||||||||||||||||||||||||||||||||||||||||||||
Sbjct  447800  CCTGCGTGCGCTTTACGCCCAGTAATTCCGATTAACGCTTGCACCCTCCGTATTACCGCG  447741

Query  485     GCTGCTGGCACGGAGTTAGCCGGTGCTTCTTCTGCGGGTAACGTCAATCGACGCGGTTAT  544
               |||||||||||||||||||||||||||||||||||||||||||||||||| |||||||||
Sbjct  447740  GCTGCTGGCACGGAGTTAGCCGGTGCTTCTTCTGCGGGTAACGTCAATCGGCGCGGTTAT  447681

Query  545     TAACCNCATCGCCTTCCTCCCCGCTGAAAGTACTTTACAACCCGAAGGCCTTCTTC  600
               ||||| || |||||||||||||||||||||||||||||||||||||||||||||||
Sbjct  447680  TAACCGCACCGCCTTCCTCCCCGCTGAAAGTACTTTACAACCCGAAGGCCTTCTTC  447625
```

Range 5: 1038242 to 1038837GenBankGraphics

Next Match
Previous Match
First Match

Alignment statistics for match #5

| Score | Expect | Identities | Gaps | Strand | Frame |
| --- | --- | --- | --- | --- | --- |
| 1086 bits(588) | 0.0() | 593/596(99%) | 0/596(0%) | Plus/Minus |  |

Features:

```
Query  5        TTCCGTGGATGTCAAGAGTAGGTAAGGTTCTTCGCGTTGCATCGAATTAAACCACATGCT  64
                ||||||||||||||||||||||||||||||||||||||||||||||||||||||||||||
Sbjct  1038837  TTCCGTGGATGTCAAGAGTAGGTAAGGTTCTTCGCGTTGCATCGAATTAAACCACATGCT  1038778

Query  65       CCACCGCTTGTGCGGGCCCCCGTCAATTCATTTGAGTTTTAACCTTGCGGCCGTACTCCC  124
                ||||||||||||||||||||||||||||||||||||||||||||||||||||||||||||
Sbjct  1038777  CCACCGCTTGTGCGGGCCCCCGTCAATTCATTTGAGTTTTAACCTTGCGGCCGTACTCCC  1038718

Query  125      CAGGCGGTCGACTTAACGCGTTAGCTCCGGAAGCCACTCCTCAAGGGAACAACCTCCAAG  184
                ||||||||||||||||||||||||||||||||||||||||||||||||||||||||||||
Sbjct  1038717  CAGGCGGTCGACTTAACGCGTTAGCTCCGGAAGCCACTCCTCAAGGGAACAACCTCCAAG  1038658

Query  185      TCGACATCGTTTACGGCGTGGACTACCAGGGTATCTAATCCTGTTTGCTCCCCACGCTTT  244
                ||||||||||||||||||||||||||||||||||||||||||||||||||||||||||||
Sbjct  1038657  TCGACATCGTTTACGGCGTGGACTACCAGGGTATCTAATCCTGTTTGCTCCCCACGCTTT  1038598

Query  245      CGCACCTGAGCGTCAGTCTTTGTCCAGGGGGCCGCCTTCGCCACCGGTATTCCTCCAGAT  304
                ||||||||||||||||||||||||||||||||||||||||||||||||||||||||||||
Sbjct  1038597  CGCACCTGAGCGTCAGTCTTTGTCCAGGGGGCCGCCTTCGCCACCGGTATTCCTCCAGAT  1038538

Query  305      CTCTACGCATTTCACCGCTACACCTGGAATTCTACCCCCCTCTACAAGACTCAAGCCTGC  364
                ||||||||||||||||||||||||||||||||||||||||||||||||||||||||||||
Sbjct  1038537  CTCTACGCATTTCACCGCTACACCTGGAATTCTACCCCCCTCTACAAGACTCAAGCCTGC  1038478

Query  365      CAGTTTCAAATGCAGTTCCCAGGTTAAGCCCGGGGATTTCACATCTGACTTAACAGACCG  424
                ||||||||||||||||||||||||||||||||||||||||||||||||||||||||||||
Sbjct  1038477  CAGTTTCAAATGCAGTTCCCAGGTTAAGCCCGGGGATTTCACATCTGACTTAACAGACCG  1038418

Query  425      CCTGCGTGCGCTTTACGCCCAGTAATTCCGATTAACGCTTGCACCCTCCGTATTACCGCG  484
                ||||||||||||||||||||||||||||||||||||||||||||||||||||||||||||
Sbjct  1038417  CCTGCGTGCGCTTTACGCCCAGTAATTCCGATTAACGCTTGCACCCTCCGTATTACCGCG  1038358

Query  485      GCTGCTGGCACGGAGTTAGCCGGTGCTTCTTCTGCGGGTAACGTCAATCGACGCGGTTAT  544
                |||||||||||||||||||||||||||||||||||||||||||||||||| |||||||||
Sbjct  1038357  GCTGCTGGCACGGAGTTAGCCGGTGCTTCTTCTGCGGGTAACGTCAATCGGCGCGGTTAT  1038298

Query  545      TAACCNCATCGCCTTCCTCCCCGCTGAAAGTACTTTACAACCCGAAGGCCTTCTTC  600
                ||||| || |||||||||||||||||||||||||||||||||||||||||||||||
Sbjct  1038297  TAACCGCACCGCCTTCCTCCCCGCTGAAAGTACTTTACAACCCGAAGGCCTTCTTC  1038242
```

Range 6: 3350844 to 3351439GenBankGraphics

Next Match
Previous Match
First Match

Alignment statistics for match #6

| Score | Expect | Identities | Gaps | Strand | Frame |
| --- | --- | --- | --- | --- | --- |
| 1086 bits(588) | 0.0() | 593/596(99%) | 0/596(0%) | Plus/Plus |  |

Features:

```
Query  5        TTCCGTGGATGTCAAGAGTAGGTAAGGTTCTTCGCGTTGCATCGAATTAAACCACATGCT  64
                ||||||||||||||||||||||||||||||||||||||||||||||||||||||||||||
Sbjct  3350844  TTCCGTGGATGTCAAGAGTAGGTAAGGTTCTTCGCGTTGCATCGAATTAAACCACATGCT  3350903

Query  65       CCACCGCTTGTGCGGGCCCCCGTCAATTCATTTGAGTTTTAACCTTGCGGCCGTACTCCC  124
                ||||||||||||||||||||||||||||||||||||||||||||||||||||||||||||
Sbjct  3350904  CCACCGCTTGTGCGGGCCCCCGTCAATTCATTTGAGTTTTAACCTTGCGGCCGTACTCCC  3350963

Query  125      CAGGCGGTCGACTTAACGCGTTAGCTCCGGAAGCCACTCCTCAAGGGAACAACCTCCAAG  184
                ||||||||||||||||||||||||||||||||||||||||||||||||||||||||||||
Sbjct  3350964  CAGGCGGTCGACTTAACGCGTTAGCTCCGGAAGCCACTCCTCAAGGGAACAACCTCCAAG  3351023

Query  185      TCGACATCGTTTACGGCGTGGACTACCAGGGTATCTAATCCTGTTTGCTCCCCACGCTTT  244
                ||||||||||||||||||||||||||||||||||||||||||||||||||||||||||||
Sbjct  3351024  TCGACATCGTTTACGGCGTGGACTACCAGGGTATCTAATCCTGTTTGCTCCCCACGCTTT  3351083

Query  245      CGCACCTGAGCGTCAGTCTTTGTCCAGGGGGCCGCCTTCGCCACCGGTATTCCTCCAGAT  304
                ||||||||||||||||||||||||||||||||||||||||||||||||||||||||||||
Sbjct  3351084  CGCACCTGAGCGTCAGTCTTTGTCCAGGGGGCCGCCTTCGCCACCGGTATTCCTCCAGAT  3351143

Query  305      CTCTACGCATTTCACCGCTACACCTGGAATTCTACCCCCCTCTACAAGACTCAAGCCTGC  364
                ||||||||||||||||||||||||||||||||||||||||||||||||||||||||||||
Sbjct  3351144  CTCTACGCATTTCACCGCTACACCTGGAATTCTACCCCCCTCTACAAGACTCAAGCCTGC  3351203

Query  365      CAGTTTCAAATGCAGTTCCCAGGTTAAGCCCGGGGATTTCACATCTGACTTAACAGACCG  424
                ||||||||||||||||||||||||||||||||||||||||||||||||||||||||||||
Sbjct  3351204  CAGTTTCAAATGCAGTTCCCAGGTTAAGCCCGGGGATTTCACATCTGACTTAACAGACCG  3351263

Query  425      CCTGCGTGCGCTTTACGCCCAGTAATTCCGATTAACGCTTGCACCCTCCGTATTACCGCG  484
                ||||||||||||||||||||||||||||||||||||||||||||||||||||||||||||
Sbjct  3351264  CCTGCGTGCGCTTTACGCCCAGTAATTCCGATTAACGCTTGCACCCTCCGTATTACCGCG  3351323

Query  485      GCTGCTGGCACGGAGTTAGCCGGTGCTTCTTCTGCGGGTAACGTCAATCGACGCGGTTAT  544
                |||||||||||||||||||||||||||||||||||||||||||||||||| |||||||||
Sbjct  3351324  GCTGCTGGCACGGAGTTAGCCGGTGCTTCTTCTGCGGGTAACGTCAATCGGCGCGGTTAT  3351383

Query  545      TAACCNCATCGCCTTCCTCCCCGCTGAAAGTACTTTACAACCCGAAGGCCTTCTTC  600
                ||||| || |||||||||||||||||||||||||||||||||||||||||||||||
Sbjct  3351384  TAACCGCACCGCCTTCCTCCCCGCTGAAAGTACTTTACAACCCGAAGGCCTTCTTC  3351439
```

Range 7: 3833601 to 3834196GenBankGraphics

Next Match
Previous Match
First Match

Alignment statistics for match #7

| Score | Expect | Identities | Gaps | Strand | Frame |
| --- | --- | --- | --- | --- | --- |
| 1086 bits(588) | 0.0() | 593/596(99%) | 0/596(0%) | Plus/Plus |  |

Features:

```
Query  5        TTCCGTGGATGTCAAGAGTAGGTAAGGTTCTTCGCGTTGCATCGAATTAAACCACATGCT  64
                ||||||||||||||||||||||||||||||||||||||||||||||||||||||||||||
Sbjct  3833601  TTCCGTGGATGTCAAGAGTAGGTAAGGTTCTTCGCGTTGCATCGAATTAAACCACATGCT  3833660

Query  65       CCACCGCTTGTGCGGGCCCCCGTCAATTCATTTGAGTTTTAACCTTGCGGCCGTACTCCC  124
                ||||||||||||||||||||||||||||||||||||||||||||||||||||||||||||
Sbjct  3833661  CCACCGCTTGTGCGGGCCCCCGTCAATTCATTTGAGTTTTAACCTTGCGGCCGTACTCCC  3833720

Query  125      CAGGCGGTCGACTTAACGCGTTAGCTCCGGAAGCCACTCCTCAAGGGAACAACCTCCAAG  184
                ||||||||||||||||||||||||||||||||||||||||||||||||||||||||||||
Sbjct  3833721  CAGGCGGTCGACTTAACGCGTTAGCTCCGGAAGCCACTCCTCAAGGGAACAACCTCCAAG  3833780

Query  185      TCGACATCGTTTACGGCGTGGACTACCAGGGTATCTAATCCTGTTTGCTCCCCACGCTTT  244
                ||||||||||||||||||||||||||||||||||||||||||||||||||||||||||||
Sbjct  3833781  TCGACATCGTTTACGGCGTGGACTACCAGGGTATCTAATCCTGTTTGCTCCCCACGCTTT  3833840

Query  245      CGCACCTGAGCGTCAGTCTTTGTCCAGGGGGCCGCCTTCGCCACCGGTATTCCTCCAGAT  304
                ||||||||||||||||||||||||||||||||||||||||||||||||||||||||||||
Sbjct  3833841  CGCACCTGAGCGTCAGTCTTTGTCCAGGGGGCCGCCTTCGCCACCGGTATTCCTCCAGAT  3833900

Query  305      CTCTACGCATTTCACCGCTACACCTGGAATTCTACCCCCCTCTACAAGACTCAAGCCTGC  364
                ||||||||||||||||||||||||||||||||||||||||||||||||||||||||||||
Sbjct  3833901  CTCTACGCATTTCACCGCTACACCTGGAATTCTACCCCCCTCTACAAGACTCAAGCCTGC  3833960

Query  365      CAGTTTCAAATGCAGTTCCCAGGTTAAGCCCGGGGATTTCACATCTGACTTAACAGACCG  424
                ||||||||||||||||||||||||||||||||||||||||||||||||||||||||||||
Sbjct  3833961  CAGTTTCAAATGCAGTTCCCAGGTTAAGCCCGGGGATTTCACATCTGACTTAACAGACCG  3834020

Query  425      CCTGCGTGCGCTTTACGCCCAGTAATTCCGATTAACGCTTGCACCCTCCGTATTACCGCG  484
                ||||||||||||||||||||||||||||||||||||||||||||||||||||||||||||
Sbjct  3834021  CCTGCGTGCGCTTTACGCCCAGTAATTCCGATTAACGCTTGCACCCTCCGTATTACCGCG  3834080

Query  485      GCTGCTGGCACGGAGTTAGCCGGTGCTTCTTCTGCGGGTAACGTCAATCGACGCGGTTAT  544
                |||||||||||||||||||||||||||||||||||||||||||||||||| |||||||||
Sbjct  3834081  GCTGCTGGCACGGAGTTAGCCGGTGCTTCTTCTGCGGGTAACGTCAATCGGCGCGGTTAT  3834140

Query  545      TAACCNCATCGCCTTCCTCCCCGCTGAAAGTACTTTACAACCCGAAGGCCTTCTTC  600
                ||||| || |||||||||||||||||||||||||||||||||||||||||||||||
Sbjct  3834141  TAACCGCACCGCCTTCCTCCCCGCTGAAAGTACTTTACAACCCGAAGGCCTTCTTC  3834196
```

Download

FASTA (complete sequence)

FASTA (aligned sequences)

GenBank (complete sequence)

Continue
Cancel

GenBankGraphics

Next
Previous
Descriptions

Pantoea sp. strain AL38 16S ribosomal RNA gene, partial sequence

Sequence ID: MG819433.1Length: 1417Number of Matches: 1

Related Information

Range 1: 360 to 955GenBankGraphics

Next Match
Previous Match
First Match

Alignment statistics for match #1

| Score | Expect | Identities | Gaps | Strand | Frame |
| --- | --- | --- | --- | --- | --- |
| 1098 bits(594) | 0.0() | 595/596(99%) | 0/596(0%) | Plus/Minus |  |

Features:

```
Query  5    TTCCGTGGATGTCAAGAGTAGGTAAGGTTCTTCGCGTTGCATCGAATTAAACCACATGCT  64
            ||||||||||||||||||||||||||||||||||||||||||||||||||||||||||||
Sbjct  955  TTCCGTGGATGTCAAGAGTAGGTAAGGTTCTTCGCGTTGCATCGAATTAAACCACATGCT  896

Query  65   CCACCGCTTGTGCGGGCCCCCGTCAATTCATTTGAGTTTTAACCTTGCGGCCGTACTCCC  124
            ||||||||||||||||||||||||||||||||||||||||||||||||||||||||||||
Sbjct  895  CCACCGCTTGTGCGGGCCCCCGTCAATTCATTTGAGTTTTAACCTTGCGGCCGTACTCCC  836

Query  125  CAGGCGGTCGACTTAACGCGTTAGCTCCGGAAGCCACTCCTCAAGGGAACAACCTCCAAG  184
            ||||||||||||||||||||||||||||||||||||||||||||||||||||||||||||
Sbjct  835  CAGGCGGTCGACTTAACGCGTTAGCTCCGGAAGCCACTCCTCAAGGGAACAACCTCCAAG  776

Query  185  TCGACATCGTTTACGGCGTGGACTACCAGGGTATCTAATCCTGTTTGCTCCCCACGCTTT  244
            ||||||||||||||||||||||||||||||||||||||||||||||||||||||||||||
Sbjct  775  TCGACATCGTTTACGGCGTGGACTACCAGGGTATCTAATCCTGTTTGCTCCCCACGCTTT  716

Query  245  CGCACCTGAGCGTCAGTCTTTGTCCAGGGGGCCGCCTTCGCCACCGGTATTCCTCCAGAT  304
            ||||||||||||||||||||||||||||||||||||||||||||||||||||||||||||
Sbjct  715  CGCACCTGAGCGTCAGTCTTTGTCCAGGGGGCCGCCTTCGCCACCGGTATTCCTCCAGAT  656

Query  305  CTCTACGCATTTCACCGCTACACCTGGAATTCTACCCCCCTCTACAAGACTCAAGCCTGC  364
            ||||||||||||||||||||||||||||||||||||||||||||||||||||||||||||
Sbjct  655  CTCTACGCATTTCACCGCTACACCTGGAATTCTACCCCCCTCTACAAGACTCAAGCCTGC  596

Query  365  CAGTTTCAAATGCAGTTCCCAGGTTAAGCCCGGGGATTTCACATCTGACTTAACAGACCG  424
            ||||||||||||||||||||||||||||||||||||||||||||||||||||||||||||
Sbjct  595  CAGTTTCAAATGCAGTTCCCAGGTTAAGCCCGGGGATTTCACATCTGACTTAACAGACCG  536

Query  425  CCTGCGTGCGCTTTACGCCCAGTAATTCCGATTAACGCTTGCACCCTCCGTATTACCGCG  484
            ||||||||||||||||||||||||||||||||||||||||||||||||||||||||||||
Sbjct  535  CCTGCGTGCGCTTTACGCCCAGTAATTCCGATTAACGCTTGCACCCTCCGTATTACCGCG  476

Query  485  GCTGCTGGCACGGAGTTAGCCGGTGCTTCTTCTGCGGGTAACGTCAATCGACGCGGTTAT  544
            ||||||||||||||||||||||||||||||||||||||||||||||||||||||||||||
Sbjct  475  GCTGCTGGCACGGAGTTAGCCGGTGCTTCTTCTGCGGGTAACGTCAATCGACGCGGTTAT  416

Query  545  TAACCNCATCGCCTTCCTCCCCGCTGAAAGTACTTTACAACCCGAAGGCCTTCTTC  600
            ||||| ||||||||||||||||||||||||||||||||||||||||||||||||||
Sbjct  415  TAACCGCATCGCCTTCCTCCCCGCTGAAAGTACTTTACAACCCGAAGGCCTTCTTC  360
```

Download

FASTA (complete sequence)

FASTA (aligned sequences)

GenBank (complete sequence)

Continue
Cancel

GenBankGraphics

Next
Previous
Descriptions

Pantoea sp. strain AL226 16S ribosomal RNA gene, partial sequence

Sequence ID: MG819432.1Length: 1417Number of Matches: 1

Related Information

Range 1: 360 to 955GenBankGraphics

Next Match
Previous Match
First Match

Alignment statistics for match #1

| Score | Expect | Identities | Gaps | Strand | Frame |
| --- | --- | --- | --- | --- | --- |
| 1098 bits(594) | 0.0() | 595/596(99%) | 0/596(0%) | Plus/Minus |  |

Features:

```
Query  5    TTCCGTGGATGTCAAGAGTAGGTAAGGTTCTTCGCGTTGCATCGAATTAAACCACATGCT  64
            ||||||||||||||||||||||||||||||||||||||||||||||||||||||||||||
Sbjct  955  TTCCGTGGATGTCAAGAGTAGGTAAGGTTCTTCGCGTTGCATCGAATTAAACCACATGCT  896

Query  65   CCACCGCTTGTGCGGGCCCCCGTCAATTCATTTGAGTTTTAACCTTGCGGCCGTACTCCC  124
            ||||||||||||||||||||||||||||||||||||||||||||||||||||||||||||
Sbjct  895  CCACCGCTTGTGCGGGCCCCCGTCAATTCATTTGAGTTTTAACCTTGCGGCCGTACTCCC  836

Query  125  CAGGCGGTCGACTTAACGCGTTAGCTCCGGAAGCCACTCCTCAAGGGAACAACCTCCAAG  184
            ||||||||||||||||||||||||||||||||||||||||||||||||||||||||||||
Sbjct  835  CAGGCGGTCGACTTAACGCGTTAGCTCCGGAAGCCACTCCTCAAGGGAACAACCTCCAAG  776

Query  185  TCGACATCGTTTACGGCGTGGACTACCAGGGTATCTAATCCTGTTTGCTCCCCACGCTTT  244
            ||||||||||||||||||||||||||||||||||||||||||||||||||||||||||||
Sbjct  775  TCGACATCGTTTACGGCGTGGACTACCAGGGTATCTAATCCTGTTTGCTCCCCACGCTTT  716

Query  245  CGCACCTGAGCGTCAGTCTTTGTCCAGGGGGCCGCCTTCGCCACCGGTATTCCTCCAGAT  304
            ||||||||||||||||||||||||||||||||||||||||||||||||||||||||||||
Sbjct  715  CGCACCTGAGCGTCAGTCTTTGTCCAGGGGGCCGCCTTCGCCACCGGTATTCCTCCAGAT  656

Query  305  CTCTACGCATTTCACCGCTACACCTGGAATTCTACCCCCCTCTACAAGACTCAAGCCTGC  364
            ||||||||||||||||||||||||||||||||||||||||||||||||||||||||||||
Sbjct  655  CTCTACGCATTTCACCGCTACACCTGGAATTCTACCCCCCTCTACAAGACTCAAGCCTGC  596

Query  365  CAGTTTCAAATGCAGTTCCCAGGTTAAGCCCGGGGATTTCACATCTGACTTAACAGACCG  424
            ||||||||||||||||||||||||||||||||||||||||||||||||||||||||||||
Sbjct  595  CAGTTTCAAATGCAGTTCCCAGGTTAAGCCCGGGGATTTCACATCTGACTTAACAGACCG  536

Query  425  CCTGCGTGCGCTTTACGCCCAGTAATTCCGATTAACGCTTGCACCCTCCGTATTACCGCG  484
            ||||||||||||||||||||||||||||||||||||||||||||||||||||||||||||
Sbjct  535  CCTGCGTGCGCTTTACGCCCAGTAATTCCGATTAACGCTTGCACCCTCCGTATTACCGCG  476

Query  485  GCTGCTGGCACGGAGTTAGCCGGTGCTTCTTCTGCGGGTAACGTCAATCGACGCGGTTAT  544
            ||||||||||||||||||||||||||||||||||||||||||||||||||||||||||||
Sbjct  475  GCTGCTGGCACGGAGTTAGCCGGTGCTTCTTCTGCGGGTAACGTCAATCGACGCGGTTAT  416

Query  545  TAACCNCATCGCCTTCCTCCCCGCTGAAAGTACTTTACAACCCGAAGGCCTTCTTC  600
            ||||| ||||||||||||||||||||||||||||||||||||||||||||||||||
Sbjct  415  TAACCGCATCGCCTTCCTCCCCGCTGAAAGTACTTTACAACCCGAAGGCCTTCTTC  360
```

Download

FASTA (complete sequence)

FASTA (aligned sequences)

GenBank (complete sequence)

Continue
Cancel

GenBankGraphics

Next
Previous
Descriptions

Pantoea sp. strain AL273 16S ribosomal RNA gene, partial sequence

Sequence ID: MG819431.1Length: 1417Number of Matches: 1

Related Information

Range 1: 360 to 955GenBankGraphics

Next Match
Previous Match
First Match

Alignment statistics for match #1

| Score | Expect | Identities | Gaps | Strand | Frame |
| --- | --- | --- | --- | --- | --- |
| 1098 bits(594) | 0.0() | 595/596(99%) | 0/596(0%) | Plus/Minus |  |

Features:

```
Query  5    TTCCGTGGATGTCAAGAGTAGGTAAGGTTCTTCGCGTTGCATCGAATTAAACCACATGCT  64
            ||||||||||||||||||||||||||||||||||||||||||||||||||||||||||||
Sbjct  955  TTCCGTGGATGTCAAGAGTAGGTAAGGTTCTTCGCGTTGCATCGAATTAAACCACATGCT  896

Query  65   CCACCGCTTGTGCGGGCCCCCGTCAATTCATTTGAGTTTTAACCTTGCGGCCGTACTCCC  124
            ||||||||||||||||||||||||||||||||||||||||||||||||||||||||||||
Sbjct  895  CCACCGCTTGTGCGGGCCCCCGTCAATTCATTTGAGTTTTAACCTTGCGGCCGTACTCCC  836

Query  125  CAGGCGGTCGACTTAACGCGTTAGCTCCGGAAGCCACTCCTCAAGGGAACAACCTCCAAG  184
            ||||||||||||||||||||||||||||||||||||||||||||||||||||||||||||
Sbjct  835  CAGGCGGTCGACTTAACGCGTTAGCTCCGGAAGCCACTCCTCAAGGGAACAACCTCCAAG  776

Query  185  TCGACATCGTTTACGGCGTGGACTACCAGGGTATCTAATCCTGTTTGCTCCCCACGCTTT  244
            ||||||||||||||||||||||||||||||||||||||||||||||||||||||||||||
Sbjct  775  TCGACATCGTTTACGGCGTGGACTACCAGGGTATCTAATCCTGTTTGCTCCCCACGCTTT  716

Query  245  CGCACCTGAGCGTCAGTCTTTGTCCAGGGGGCCGCCTTCGCCACCGGTATTCCTCCAGAT  304
            ||||||||||||||||||||||||||||||||||||||||||||||||||||||||||||
Sbjct  715  CGCACCTGAGCGTCAGTCTTTGTCCAGGGGGCCGCCTTCGCCACCGGTATTCCTCCAGAT  656

Query  305  CTCTACGCATTTCACCGCTACACCTGGAATTCTACCCCCCTCTACAAGACTCAAGCCTGC  364
            ||||||||||||||||||||||||||||||||||||||||||||||||||||||||||||
Sbjct  655  CTCTACGCATTTCACCGCTACACCTGGAATTCTACCCCCCTCTACAAGACTCAAGCCTGC  596

Query  365  CAGTTTCAAATGCAGTTCCCAGGTTAAGCCCGGGGATTTCACATCTGACTTAACAGACCG  424
            ||||||||||||||||||||||||||||||||||||||||||||||||||||||||||||
Sbjct  595  CAGTTTCAAATGCAGTTCCCAGGTTAAGCCCGGGGATTTCACATCTGACTTAACAGACCG  536

Query  425  CCTGCGTGCGCTTTACGCCCAGTAATTCCGATTAACGCTTGCACCCTCCGTATTACCGCG  484
            ||||||||||||||||||||||||||||||||||||||||||||||||||||||||||||
Sbjct  535  CCTGCGTGCGCTTTACGCCCAGTAATTCCGATTAACGCTTGCACCCTCCGTATTACCGCG  476

Query  485  GCTGCTGGCACGGAGTTAGCCGGTGCTTCTTCTGCGGGTAACGTCAATCGACGCGGTTAT  544
            ||||||||||||||||||||||||||||||||||||||||||||||||||||||||||||
Sbjct  475  GCTGCTGGCACGGAGTTAGCCGGTGCTTCTTCTGCGGGTAACGTCAATCGACGCGGTTAT  416

Query  545  TAACCNCATCGCCTTCCTCCCCGCTGAAAGTACTTTACAACCCGAAGGCCTTCTTC  600
            ||||| ||||||||||||||||||||||||||||||||||||||||||||||||||
Sbjct  415  TAACCGCATCGCCTTCCTCCCCGCTGAAAGTACTTTACAACCCGAAGGCCTTCTTC  360
```

Download

FASTA (complete sequence)

FASTA (aligned sequences)

GenBank (complete sequence)

Continue
Cancel

GenBankGraphics

Next
Previous
Descriptions

Pantoea sp. strain AL269 16S ribosomal RNA gene, partial sequence

Sequence ID: MG819430.1Length: 1417Number of Matches: 1

Related Information

Range 1: 360 to 955GenBankGraphics

Next Match
Previous Match
First Match

Alignment statistics for match #1

| Score | Expect | Identities | Gaps | Strand | Frame |
| --- | --- | --- | --- | --- | --- |
| 1098 bits(594) | 0.0() | 595/596(99%) | 0/596(0%) | Plus/Minus |  |

Features:

```
Query  5    TTCCGTGGATGTCAAGAGTAGGTAAGGTTCTTCGCGTTGCATCGAATTAAACCACATGCT  64
            ||||||||||||||||||||||||||||||||||||||||||||||||||||||||||||
Sbjct  955  TTCCGTGGATGTCAAGAGTAGGTAAGGTTCTTCGCGTTGCATCGAATTAAACCACATGCT  896

Query  65   CCACCGCTTGTGCGGGCCCCCGTCAATTCATTTGAGTTTTAACCTTGCGGCCGTACTCCC  124
            ||||||||||||||||||||||||||||||||||||||||||||||||||||||||||||
Sbjct  895  CCACCGCTTGTGCGGGCCCCCGTCAATTCATTTGAGTTTTAACCTTGCGGCCGTACTCCC  836

Query  125  CAGGCGGTCGACTTAACGCGTTAGCTCCGGAAGCCACTCCTCAAGGGAACAACCTCCAAG  184
            ||||||||||||||||||||||||||||||||||||||||||||||||||||||||||||
Sbjct  835  CAGGCGGTCGACTTAACGCGTTAGCTCCGGAAGCCACTCCTCAAGGGAACAACCTCCAAG  776

Query  185  TCGACATCGTTTACGGCGTGGACTACCAGGGTATCTAATCCTGTTTGCTCCCCACGCTTT  244
            ||||||||||||||||||||||||||||||||||||||||||||||||||||||||||||
Sbjct  775  TCGACATCGTTTACGGCGTGGACTACCAGGGTATCTAATCCTGTTTGCTCCCCACGCTTT  716

Query  245  CGCACCTGAGCGTCAGTCTTTGTCCAGGGGGCCGCCTTCGCCACCGGTATTCCTCCAGAT  304
            ||||||||||||||||||||||||||||||||||||||||||||||||||||||||||||
Sbjct  715  CGCACCTGAGCGTCAGTCTTTGTCCAGGGGGCCGCCTTCGCCACCGGTATTCCTCCAGAT  656

Query  305  CTCTACGCATTTCACCGCTACACCTGGAATTCTACCCCCCTCTACAAGACTCAAGCCTGC  364
            ||||||||||||||||||||||||||||||||||||||||||||||||||||||||||||
Sbjct  655  CTCTACGCATTTCACCGCTACACCTGGAATTCTACCCCCCTCTACAAGACTCAAGCCTGC  596

Query  365  CAGTTTCAAATGCAGTTCCCAGGTTAAGCCCGGGGATTTCACATCTGACTTAACAGACCG  424
            ||||||||||||||||||||||||||||||||||||||||||||||||||||||||||||
Sbjct  595  CAGTTTCAAATGCAGTTCCCAGGTTAAGCCCGGGGATTTCACATCTGACTTAACAGACCG  536

Query  425  CCTGCGTGCGCTTTACGCCCAGTAATTCCGATTAACGCTTGCACCCTCCGTATTACCGCG  484
            ||||||||||||||||||||||||||||||||||||||||||||||||||||||||||||
Sbjct  535  CCTGCGTGCGCTTTACGCCCAGTAATTCCGATTAACGCTTGCACCCTCCGTATTACCGCG  476

Query  485  GCTGCTGGCACGGAGTTAGCCGGTGCTTCTTCTGCGGGTAACGTCAATCGACGCGGTTAT  544
            ||||||||||||||||||||||||||||||||||||||||||||||||||||||||||||
Sbjct  475  GCTGCTGGCACGGAGTTAGCCGGTGCTTCTTCTGCGGGTAACGTCAATCGACGCGGTTAT  416

Query  545  TAACCNCATCGCCTTCCTCCCCGCTGAAAGTACTTTACAACCCGAAGGCCTTCTTC  600
            ||||| ||||||||||||||||||||||||||||||||||||||||||||||||||
Sbjct  415  TAACCGCATCGCCTTCCTCCCCGCTGAAAGTACTTTACAACCCGAAGGCCTTCTTC  360
```

```

```


BLAST is a registered trademark of the National Library of Medicine

Support center
Mailing list


YouTube

- National Library Of Medicine
- National Institutes Of Health
- U.S. Department of Health & Human Services
- USA.gov

### NCBI


National Center for Biotechnology Information,
 U.S. National Library of Medicine

8600 Rockville Pike,
Bethesda
 MD,
20894
USA

Policies and Guidelines
|
Contact


PreferencesTurn off

External link. Please review our privacy policy.
