# Supplementary material for: Isothermal microcalorimetry for thermal viable count of microorganisms in pure cultures and stabilized formulations
Source: BMC Microbiol. 2019 Mar 21;19:65. doi: 10.1186/s12866-019-1432-8 (PMC6429831; doi:10.1186/s12866-019-1432-8)
Supplement: Supplementary file 3 — 16S rRNA gene sequences’ BLASTN hits in zipped HTML format. (ZIP 15810 kb) [file 12866_2019_1432_MOESM3_ESM.zip › Best blastn hits/NCBI Blast_38 pinkish lower band F -- 17..1039 of.html]

NCBI Blast:38 pinkish lower band F -- 17..1039 of


- NCBI Home
- Sign in to NCBI
- Skip to Main Content
- Skip to Navigation
- About NCBI Accesskeys

U.S. National Library of Medicine

NCBI
National Center for Biotechnology Information

- My NCBI
- Sign in to NCBI
- Register
- Sign Out

BLAST ® » blastn suite » RID-A4W4RNJW014


- Home
- Recent Results
- Saved Strategies
- Help

BLAST Results


Edit and Resubmit
Save Search Strategies
[Sign in above to save your search strategy]

Formatting options 


Download


How to read this page
Blast report description
Questions/comments


|  |  |
| --- | --- |
| Formatting options | |
| Show | Alignment as  HTML Plain text   Old View Reset form to defaults [?]  These options control formatting of alignments in results pages. The default is HTML, but other formats (including plain text) are available. PSSM and PssmWithParameters are representations of Position Specific Scoring Matrices and are only available for PSI-BLAST. The Advanced view option allows the database descriptions to be sorted by various indices in a table. |
| Alignment View | Pairwise Pairwise with dots for identities Query-anchored with dots for identities Query-anchored with letters for identities Flat query-anchored with dots for identities Flat query-anchored with letters for identities [?]  Choose how to view alignments. The default "pairwise" view shows how each subject sequence aligns individually to the query sequence. The "query-anchored" view shows how all subject sequences align to the query sequence. For each view type, you can choose to show "identities" (matching residues) as letters or dots. more... |
| Display | Graphical Overview   Linkout   Sequence Retrieval  NCBI-gi   CDS feature [?]  - Graphical Overview: Graphical Overview: Show graph of similar sequence regions aligned to query.   more... - NCBI-gi: Show NCBI gi identifiers. - CDS feature: Show annotated coding region and translation.   more... |
| Masking | Character:   X for protein, n for nucleotide Lower Case  Color:  Black Grey Red [?]  - Masking Character: Display masked (filtered) sequence regions as lower-case or as specific letters (N for nucleotide, P for protein). - Masking Color: Display masked sequence regions in the given color. |
| Limit results | Descriptions:  10 50 100 Graphical overview:  0 10 50 100  Alignments:  0 10 50 100 Line length:  60 90 120 150 [?]  - Descriptions: Show short descriptions for up to the given number of sequences. - Alignments: Show alignments for up to the given number of sequences, in order of statistical significance. - Line lenghth: Number of letters to show on one line in an alignment. |
|  | Organism Type common name, binomial, taxid, or group name. Only 20 top taxa will be shown.     Exclude    [?]  Show only sequences from the given organism. |
|  | Entrez query:  [?]  Show only those sequences that match the given Entrez query. more... |
|  | Expect Min:  Expect Max:  [?]  Show only sequences with expect values in the given range. more... |
|  | Percent Identity Min:  Percent Identity Max:  [?]  Show only sequences with percent identity values in the given range. |
| Format for | PSI-BLAST with inclusion threshold:  [?]  - Format for PSI-BLAST: The Position-Specific Iterated BLAST (PSI-BLAST) program performs iterative searches with a protein query,   in which sequences found in one round of search are used to build a custom score model for the next round.   more... - Inclusion Threshold: This sets the statistical significance threshold for including a sequence in the model used   by PSI-BLAST to create the PSSM on the next iteration. |

|  |  |  |  |  |  |
| --- | --- | --- | --- | --- | --- |
| Download | | | | | |
| Alignment  Text XML ASN.1 JSON Seq-align Hit Table(text) Hit Table(csv) Multiple-file XML2 Single-file XML2 Multiple-file JSON Single-file JSON SAM | Search Strategies  ASN.1 | PSSM to restart search  PSSM | [?] |

The Download link provides BLAST output that may be used as input to another program.
This includes parseable formats such as the tabular report or XML as well as the Search Strategy files read by the BLAST+ applications.
More details on the parseable (XML, tabular, and ASN.1) reports can be found at
https://www.ncbi.nlm.nih.gov/books/NBK153387/  
  

The following formats are offered under the Alignment section:  
1). "Text". Non-HTML standard BLAST report.  
2). "XML". XML report based upon the DTD at https://www.ncbi.nlm.nih.gov/data\_specs/dtd/NCBI\_BlastOutput.dtd  
3). "ASN.1". Alignment written out in Abstract Syntax Notation 1.  
4). "JSON Seq-align". Alignment written out in JSON.  
4). "Hit Table(text)". The tabular report as text.  
5). "Hit Table(csv)". The tabular report ready for import into spread-sheet programs like Excel.  
6). "XML2". New XML format described at ftp://ftp.ncbi.nlm.nih.gov/blast/documents/NEWXML/xml2.pdf.  
7). "JSON". New JSON format described at ftp://ftp.ncbi.nlm.nih.gov/blast/documents/NEWXML/xml2.pdf.  
8). "SAM". Sequence Alignment Map format.

XML2 and JSON can be downloaded either as one file per query (multiple-file) or one file for all queries (single-file). These formats are listed as Multiple-file XML2 (and JSON) or Single-file XML (and JSON).

The following report is offered under the Search Strategy section:  
1). "ASN.1" Search Strategy. A record of the parameters, query, and database used in the search. This file can be used to start a stand-alone BLAST search, see
https://www.ncbi.nlm.nih.gov/books/NBK1763/#CmdLineAppsManual.I455\_BLAST\_search\_stra


# Job title: 38 pinkish lower band F -- 17..1039 of

Results for:

lcl|Query\_161107 38 pinkish lower band F -- 17..1039 of sequence(1023bp)
[?]

Your BLAST job specified more than one input sequence.
This box lets you choose which input sequence to show BLAST results for.

RID
:   A4W4RNJW014 (Expires on 03-10 18:31 pm)

Query ID
:   lcl|Query\_161107
:   lcl|Query\_161107

Description
:   38 pinkish lower band F -- 17..1039 of sequence

Molecule type
:   nucleic acid

Query Length
:   1023

Database Name
:   nr

Description
:   Nucleotide collection (nt) See details

Program
:   BLASTN 2.8.0+ Citation

  

Reference 

Zheng Zhang, Scott Schwartz, Lukas Wagner, and Webb Miller (2000), "A greedy algorithm for aligning DNA sequences", J Comput Biol 2000; 7(1-2):203-14.

Reference - database indexing

Aleksandr Morgulis, George Coulouris, Yan Raytselis, Thomas L. Madden, Richa Agarwala, Alejandro A. Schäffer (2008), "Database Indexing for Production MegaBLAST Searches", Bioinformatics 24:1757-1764.

Other reports:
Search Summary

[Taxonomy reports]
[Distance tree of results]
[MSA viewer]

Search Parameters

| Search parameter name | Search parameter value |
| --- | --- |
| Program | blastn |
| Word size | 28 |
| Expect value | 10 |
| Hitlist size | 100 |
| Match/Mismatch scores | 1,-2 |
| Gapcosts | 0,2.5 |
| Low Complexity Filter | Yes |
| Filter string | L;m; |
| Genetic Code | 1 |

Database

| Database parameter name | Database parameter value |
| --- | --- |
| Posted date | Mar 7, 2018 1:58 PM |
| Number of letters | 174,044,644,244 |
| Number of sequences | 46,882,714 |
| Entrez query | Includes:  Excludes:  None |

Karlin-Altschul statistics

| Params | Ungapped | Gapped |
| --- | --- | --- |
| Lambda | 1.33271 | 1.28 |
| K | 0.620991 | 0.46 |
| H | 1.12409 | 0.85 |

Results Statistics

| Results Statistics parameter name | Results Statistics parameter value |
| --- | --- |
| Length adjustment | 35 |
| Effective length of query | 988 |
| Effective length of database | 172403749254 |
| Effective search space | 170334904262952 |
| Effective search space used | 170334904262952 |


## Graphic Summary

### Distribution of the top 128 Blast Hits on 100 subject sequences [?]

The graphic is an overview of the database sequences aligned to the query sequence. These are represented horizontal bars colored coded by score and showing the extent
of the alignment on the query sequence. Separate aligned regions on the same database sequence are connected by a thin grey line.
Mousing over an alignment shows the database sequence title. Clicking an alignment displays a box with more details about the alignment and
link to the sequence alignment itself in the Alignments section of the report.

Mouse over to see the title, click to show alignments

Color key for alignment scores

<40

40-50

50-80

80-200

>=200

Query

1

200

400

600

800

1000

Pseudomonas sp. SAM1 16S ribosomal RNA gene, partial se..

Score:1866 Evalue:0

Accession:KM269192.1

Alignment

Pseudomonas thivervalensis strain h-23 16S ribosomal RN..

Score:1866 Evalue:0

Accession:KC139440.1

Alignment

Pseudomonas sp. strain 7.3 16S ribosomal RNA gene, part..

Score:1862 Evalue:0

Accession:KY542120.1

Alignment

Pseudomonas sp. MAR9909 16S ribosomal RNA gene, partial..

Score:1862 Evalue:0

Accession:KU882744.1

Alignment

Pseudomonas brassicacearum subsp. neoaurantiaca strain ..

Score:1862 Evalue:0

Accession:KT997466.1

Alignment

Pseudomonas brassicacearum subsp. neoaurantiaca strain ..

Score:1862 Evalue:0

Accession:KT997442.1

Alignment

Pseudomonas sp. cpRA293 16S ribosomal RNA gene, partial..

Score:1862 Evalue:0

Accession:KJ510220.1

Alignment

Pseudomonas thivervalensis strain PE32 16S ribosomal RN..

Score:1862 Evalue:0

Accession:KJ420530.1

Alignment

Pseudomonas putida partial 16S rRNA gene, strain CFBP 4..

Score:1862 Evalue:0

Accession:HF545841.1

Alignment

Uncultured Pseudomonas sp. clone QL0ABY37ZD02 16S ribos..

Score:1862 Evalue:0

Accession:JQ712549.1

Alignment

Pseudomonas thivervalensis 16S ribosomal RNA gene, part..

Score:1862 Evalue:0

Accession:JN628032.1

Alignment

Pseudomonas brassicacearum strain Zy-2-1 16S ribosomal ..

Score:1862 Evalue:0

Accession:GU201849.1

Alignment

Pseudomonas brassicacearum isolate MA250 16S ribosomal ..

Score:1862 Evalue:0

Accession:DQ886486.1

Alignment

Pseudomonas putida strain 42R-P6 16S ribosomal RNA gene..

Score:1860 Evalue:0

Accession:MF062638.1

Alignment

Pseudomonas fluorescens strain 42R-P4 16S ribosomal RNA..

Score:1860 Evalue:0

Accession:MF062635.1

Alignment

Pseudomonas brassicacearum strain PG14 16S ribosomal RN..

Score:1860 Evalue:0

Accession:KU350592.1

Alignment

Pseudomonas brassicacearum strain SY04(2) 16S ribosomal..

Score:1860 Evalue:0

Accession:KT239461.1

Alignment

Pseudomonas sp. TAD163 16S ribosomal RNA gene, partial ..

Score:1860 Evalue:0

Accession:FJ225306.1

Alignment

Pseudomonas sp. TAD117 16S ribosomal RNA gene, partial ..

Score:1860 Evalue:0

Accession:FJ225265.1

Alignment

Pseudomonas fluorescens partial 16S rRNA gene, strain S..

Score:1860 Evalue:0

Accession:AM900685.1

Alignment

Pseudomonas kilonensis isolate PD 31 16S ribosomal RNA ..

Score:1860 Evalue:0

Accession:DQ377772.1

Alignment

Pseudomonas brassicacearum isolate PD 5 16S ribosomal R..

Score:1860 Evalue:0

Accession:DQ377746.1

Alignment

Pseudomonas brassicacearum strain LBUM300 16S ribosomal..

Score:1857 Evalue:0

Accession:MG461459.1

Alignment

Pseudomonas sp. strain A18 16S ribosomal RNA gene, part..

Score:1857 Evalue:0

Accession:KX859159.1

Alignment

Pseudomonas sp. strain A13 16S ribosomal RNA gene, part..

Score:1857 Evalue:0

Accession:KX859154.1

Alignment

Pseudomonas sp. strain A12 16S ribosomal RNA gene, part..

Score:1857 Evalue:0

Accession:KX859153.1

Alignment

Pseudomonas sp. strain A10 16S ribosomal RNA gene, part..

Score:1857 Evalue:0

Accession:KX859151.1

Alignment

Pseudomonas sp. strain A9 16S ribosomal RNA gene, parti..

Score:1857 Evalue:0

Accession:KX859150.1

Alignment

Pseudomonas sp. strain A8 16S ribosomal RNA gene, parti..

Score:1857 Evalue:0

Accession:KX859149.1

Alignment

Pseudomonas sp. strain A4 16S ribosomal RNA gene, parti..

Score:1857 Evalue:0

Accession:KX859145.1

Alignment

Pseudomonas sp. HA-09 partial 16S rRNA gene, isolate HA..

Score:1857 Evalue:0

Accession:LT844660.1

Alignment

Uncultured Pseudomonas sp. partial 16S rRNA gene, isola..

Score:1857 Evalue:0

Accession:LT718479.1

Alignment

Pseudomonas brassicacearum strain BS3663 genome assembl..

Score:1857 Evalue:0

Accession:LT629713.1

Alignment

Pseudomonas brassicacearum strain Delaware 16S ribosoma..

Score:1857 Evalue:0

Accession:KT695846.1

Alignment

Pseudomonas brassicacearum strain Wood1 16S ribosomal R..

Score:1857 Evalue:0

Accession:KT695843.1

Alignment

Pseudomonas brassicacearum strain 93F8 16S ribosomal RN..

Score:1857 Evalue:0

Accession:KT695841.1

Alignment

Pseudomonas brassicacearum strain L13-6-12, complete ge..

Score:1857 Evalue:0

Accession:CP014693.1

Alignment

Pseudomonas brassicacearum subsp. neoaurantiaca partial..

Score:1857 Evalue:0

Accession:LT547829.1

Alignment

Pseudomonas brassicacearum strain PG17 16S ribosomal RN..

Score:1857 Evalue:0

Accession:KU350605.1

Alignment

Pseudomonas brassicacearum strain PG16 16S ribosomal RN..

Score:1857 Evalue:0

Accession:KU350593.1

Alignment

Pseudomonas brassicacearum strain LBUM300, complete gen..

Score:1857 Evalue:0

Accession:CP012680.1

Alignment

Pseudomonas fluorescens strain FW300-N2C3, complete gen..

Score:1857 Evalue:0

Accession:CP012831.1

Alignment

Pseudomonas brassicacearum strain KAR22 16S ribosomal R..

Score:1857 Evalue:0

Accession:KR054984.1

Alignment

Pseudomonas brassicacearum strain IHB B 13650 16S ribos..

Score:1857 Evalue:0

Accession:KP762561.1

Alignment

Pseudomonas brassicacearum subsp. neoaurantiaca strain ..

Score:1857 Evalue:0

Accession:KP762555.1

Alignment

Pseudomonas fluorescens strain WCS365 16S ribosomal RNA..

Score:1857 Evalue:0

Accession:KP253039.1

Alignment

Pseudomonas brassicacearum subsp. brassicacearum gene f..

Score:1857 Evalue:0

Accession:LC015569.1

Alignment

Pseudomonas sp. DT 5-12 16S ribosomal RNA gene, partial..

Score:1857 Evalue:0

Accession:KM253057.1

Alignment

Pseudomonas sp. DR 2-03 16S ribosomal RNA gene, partial..

Score:1857 Evalue:0

Accession:KM253000.1

Alignment

Pseudomonas fluorescens strain JK15 16S ribosomal RNA g..

Score:1857 Evalue:0

Accession:KF148637.1

Alignment

Pseudomonas fluorescens partial 16S rRNA gene, isolate ..

Score:1857 Evalue:0

Accession:HF584852.1

Alignment

Pseudomonas brassicacearum partial 16S rRNA gene, strai..

Score:1857 Evalue:0

Accession:HF952549.1

Alignment

Pseudomonas brassicacearum partial 16S rRNA gene, strai..

Score:1857 Evalue:0

Accession:HF952537.1

Alignment

Pseudomonas fluorescens strain IBFC2012-45 16S ribosoma..

Score:1857 Evalue:0

Accession:KC246049.1

Alignment

Pseudomonas sp. EA3(2012) 16S ribosomal RNA gene, parti..

Score:1857 Evalue:0

Accession:JX912367.1

Alignment

Pseudomonas brassicacearum strain J12 16S ribosomal RNA..

Score:1857 Evalue:0

Accession:JN605747.1

Alignment

## Descriptions

, Reading indexes 1-5, displaying indexes 1-5


Load next setPrevious Match

Sequences producing significant alignments:

Show all columns  of the table presenting sequences producing significant alignments 

Select:AllNone
Selected:0

Alignments
Download

FASTA (complete sequence)

FASTA (aligned sequences)

GenBank (complete sequence)

Hit Table (text)

Hit Table (CSV)

Text

XML

ASN.1

Continue
Cancel

GenBank 
Graphics
Distance tree of results
Multiple alignment
Show/hide columns of the table presenting sequences producing significant alignments 

Available columns

Description  
Max Score  
Total Score  
Coverage  
E-value  
IdentN  
Accession  
Restore Defaults
Ok
Cancel

Sequences producing significant alignments:

| Select for downloading or viewing reports | Description | Max score | Total score | Query cover | E value | Ident | Accession |
| --- | --- | --- | --- | --- | --- | --- | --- |
| 1Select seq KM269192.1 | Pseudomonas sp. SAM1 16S ribosomal RNA gene, partial sequence | 1866 | 1866 | 99% | 0.0 | 99% | KM269192.1 |
| 2Select seq KC139440.1 | Pseudomonas thivervalensis strain h-23 16S ribosomal RNA gene, partial sequence | 1866 | 1866 | 99% | 0.0 | 99% | KC139440.1 |
| 3Select seq KY542120.1 | Pseudomonas sp. strain 7.3 16S ribosomal RNA gene, partial sequence | 1862 | 1862 | 99% | 0.0 | 99% | KY542120.1 |
| 4Select seq KU882744.1 | Pseudomonas sp. MAR9909 16S ribosomal RNA gene, partial sequence | 1862 | 1862 | 99% | 0.0 | 99% | KU882744.1 |
| 5Select seq KT997466.1 | Pseudomonas brassicacearum subsp. neoaurantiaca strain MLS-8-1 16S ribosomal RNA gene, partial sequence | 1862 | 1862 | 98% | 0.0 | 99% | KT997466.1 |
| 6Select seq KT997442.1 | Pseudomonas brassicacearum subsp. neoaurantiaca strain MLS-2-8 16S ribosomal RNA gene, partial sequence | 1862 | 1862 | 98% | 0.0 | 99% | KT997442.1 |
| 7Select seq KJ510220.1 | Pseudomonas sp. cpRA293 16S ribosomal RNA gene, partial sequence | 1862 | 1862 | 99% | 0.0 | 99% | KJ510220.1 |
| 8Select seq KJ420530.1 | Pseudomonas thivervalensis strain PE32 16S ribosomal RNA gene, partial sequence | 1862 | 1862 | 99% | 0.0 | 99% | KJ420530.1 |
| 9Select seq HF545841.1 | Pseudomonas putida partial 16S rRNA gene, strain CFBP 4629 | 1862 | 1862 | 99% | 0.0 | 99% | HF545841.1 |
| 10Select seq JQ712549.1 | Uncultured Pseudomonas sp. clone QL0ABY37ZD02 16S ribosomal RNA gene, partial sequence | 1862 | 1862 | 99% | 0.0 | 99% | JQ712549.1 |
| 11Select seq JN628032.1 | Pseudomonas thivervalensis 16S ribosomal RNA gene, partial sequence | 1862 | 1862 | 99% | 0.0 | 99% | JN628032.1 |
| 12Select seq GU201849.1 | Pseudomonas brassicacearum strain Zy-2-1 16S ribosomal RNA gene, partial sequence | 1862 | 1862 | 99% | 0.0 | 99% | GU201849.1 |
| 13Select seq DQ886486.1 | Pseudomonas brassicacearum isolate MA250 16S ribosomal RNA gene, partial sequence | 1862 | 1862 | 99% | 0.0 | 99% | DQ886486.1 |
| 14Select seq MF062638.1 | Pseudomonas putida strain 42R-P6 16S ribosomal RNA gene, partial sequence | 1860 | 1860 | 99% | 0.0 | 99% | MF062638.1 |
| 15Select seq MF062635.1 | Pseudomonas fluorescens strain 42R-P4 16S ribosomal RNA gene, partial sequence | 1860 | 1860 | 99% | 0.0 | 99% | MF062635.1 |
| 16Select seq KU350592.1 | Pseudomonas brassicacearum strain PG14 16S ribosomal RNA gene, partial sequence | 1860 | 1860 | 99% | 0.0 | 99% | KU350592.1 |
| 17Select seq KT239461.1 | Pseudomonas brassicacearum strain SY04(2) 16S ribosomal RNA gene, partial sequence | 1860 | 1860 | 99% | 0.0 | 99% | KT239461.1 |
| 18Select seq FJ225306.1 | Pseudomonas sp. TAD163 16S ribosomal RNA gene, partial sequence | 1860 | 1860 | 99% | 0.0 | 99% | FJ225306.1 |
| 19Select seq FJ225265.1 | Pseudomonas sp. TAD117 16S ribosomal RNA gene, partial sequence | 1860 | 1860 | 99% | 0.0 | 99% | FJ225265.1 |
| 20Select seq AM900685.1 | Pseudomonas fluorescens partial 16S rRNA gene, strain SCAM BA\_1 | 1860 | 1860 | 99% | 0.0 | 99% | AM900685.1 |
| 21Select seq DQ377772.1 | Pseudomonas kilonensis isolate PD 31 16S ribosomal RNA gene, partial sequence | 1860 | 1860 | 99% | 0.0 | 99% | DQ377772.1 |
| 22Select seq DQ377746.1 | Pseudomonas brassicacearum isolate PD 5 16S ribosomal RNA gene, partial sequence | 1860 | 1860 | 99% | 0.0 | 99% | DQ377746.1 |
| 23Select seq MG461459.1 | Pseudomonas brassicacearum strain LBUM300 16S ribosomal RNA gene, partial sequence | 1857 | 1857 | 99% | 0.0 | 99% | MG461459.1 |
| 24Select seq KX859159.1 | Pseudomonas sp. strain A18 16S ribosomal RNA gene, partial sequence | 1857 | 1857 | 99% | 0.0 | 99% | KX859159.1 |
| 25Select seq KX859154.1 | Pseudomonas sp. strain A13 16S ribosomal RNA gene, partial sequence | 1857 | 1857 | 99% | 0.0 | 99% | KX859154.1 |
| 26Select seq KX859153.1 | Pseudomonas sp. strain A12 16S ribosomal RNA gene, partial sequence | 1857 | 1857 | 99% | 0.0 | 99% | KX859153.1 |
| 27Select seq KX859151.1 | Pseudomonas sp. strain A10 16S ribosomal RNA gene, partial sequence | 1857 | 1857 | 99% | 0.0 | 99% | KX859151.1 |
| 28Select seq KX859150.1 | Pseudomonas sp. strain A9 16S ribosomal RNA gene, partial sequence | 1857 | 1857 | 99% | 0.0 | 99% | KX859150.1 |
| 29Select seq KX859149.1 | Pseudomonas sp. strain A8 16S ribosomal RNA gene, partial sequence | 1857 | 1857 | 99% | 0.0 | 99% | KX859149.1 |
| 30Select seq KX859145.1 | Pseudomonas sp. strain A4 16S ribosomal RNA gene, partial sequence | 1857 | 1857 | 99% | 0.0 | 99% | KX859145.1 |
| 31Select seq LT844660.1 | Pseudomonas sp. HA-09 partial 16S rRNA gene, isolate HA-09 | 1857 | 1857 | 99% | 0.0 | 99% | LT844660.1 |
| 32Select seq LT718479.1 | Uncultured Pseudomonas sp. partial 16S rRNA gene, isolate VF5 | 1857 | 1857 | 99% | 0.0 | 99% | LT718479.1 |
| 33Select seq LT629713.1 | Pseudomonas brassicacearum strain BS3663 genome assembly, chromosome: I | 1857 | 9279 | 99% | 0.0 | 99% | LT629713.1 |
| 34Select seq KT695846.1 | Pseudomonas brassicacearum strain Delaware 16S ribosomal RNA gene, partial sequence | 1857 | 1857 | 99% | 0.0 | 99% | KT695846.1 |
| 35Select seq KT695843.1 | Pseudomonas brassicacearum strain Wood1 16S ribosomal RNA gene, partial sequence | 1857 | 1857 | 99% | 0.0 | 99% | KT695843.1 |
| 36Select seq KT695841.1 | Pseudomonas brassicacearum strain 93F8 16S ribosomal RNA gene, partial sequence | 1857 | 1857 | 99% | 0.0 | 99% | KT695841.1 |
| 37Select seq CP014693.1 | Pseudomonas brassicacearum strain L13-6-12, complete genome | 1857 | 9285 | 99% | 0.0 | 99% | CP014693.1 |
| 38Select seq LT547829.1 | Pseudomonas brassicacearum subsp. neoaurantiaca partial 16S rRNA gene, isolate 3\_C | 1857 | 1857 | 99% | 0.0 | 99% | LT547829.1 |
| 39Select seq KU350605.1 | Pseudomonas brassicacearum strain PG17 16S ribosomal RNA gene, partial sequence | 1857 | 1857 | 99% | 0.0 | 99% | KU350605.1 |
| 40Select seq KU350593.1 | Pseudomonas brassicacearum strain PG16 16S ribosomal RNA gene, partial sequence | 1857 | 1857 | 99% | 0.0 | 99% | KU350593.1 |
| 41Select seq CP012680.1 | Pseudomonas brassicacearum strain LBUM300, complete genome | 1857 | 9279 | 99% | 0.0 | 99% | CP012680.1 |
| 42Select seq CP012831.1 | Pseudomonas fluorescens strain FW300-N2C3, complete genome | 1857 | 9285 | 99% | 0.0 | 99% | CP012831.1 |
| 43Select seq KR054984.1 | Pseudomonas brassicacearum strain KAR22 16S ribosomal RNA gene, complete sequence | 1857 | 1857 | 99% | 0.0 | 99% | KR054984.1 |
| 44Select seq KP762561.1 | Pseudomonas brassicacearum strain IHB B 13650 16S ribosomal RNA gene, partial sequence | 1857 | 1857 | 99% | 0.0 | 99% | KP762561.1 |
| 45Select seq KP762555.1 | Pseudomonas brassicacearum subsp. neoaurantiaca strain IHB B 13645 16S ribosomal RNA gene, partial sequence | 1857 | 1857 | 99% | 0.0 | 99% | KP762555.1 |
| 46Select seq KP253039.1 | Pseudomonas fluorescens strain WCS365 16S ribosomal RNA gene, partial sequence | 1857 | 1857 | 99% | 0.0 | 99% | KP253039.1 |
| 47Select seq LC015569.1 | Pseudomonas brassicacearum subsp. brassicacearum gene for 16S ribosomal RNA, partial sequence, strain: AF5 | 1857 | 1857 | 99% | 0.0 | 99% | LC015569.1 |
| 48Select seq KM253057.1 | Pseudomonas sp. DT 5-12 16S ribosomal RNA gene, partial sequence | 1857 | 1857 | 99% | 0.0 | 99% | KM253057.1 |
| 49Select seq KM253000.1 | Pseudomonas sp. DR 2-03 16S ribosomal RNA gene, partial sequence | 1857 | 1857 | 99% | 0.0 | 99% | KM253000.1 |
| 50Select seq KF148637.1 | Pseudomonas fluorescens strain JK15 16S ribosomal RNA gene, partial sequence | 1857 | 1857 | 99% | 0.0 | 99% | KF148637.1 |
| 51Select seq HF584852.1 | Pseudomonas fluorescens partial 16S rRNA gene, isolate BD17-B21 | 1857 | 1857 | 99% | 0.0 | 99% | HF584852.1 |
| 52Select seq HF952549.1 | Pseudomonas brassicacearum partial 16S rRNA gene, strain HMGU196 | 1857 | 1857 | 99% | 0.0 | 99% | HF952549.1 |
| 53Select seq HF952537.1 | Pseudomonas brassicacearum partial 16S rRNA gene, strain HMGU70 | 1857 | 1857 | 99% | 0.0 | 99% | HF952537.1 |
| 54Select seq KC246049.1 | Pseudomonas fluorescens strain IBFC2012-45 16S ribosomal RNA gene, partial sequence | 1857 | 1857 | 99% | 0.0 | 99% | KC246049.1 |
| 55Select seq JX912367.1 | Pseudomonas sp. EA3(2012) 16S ribosomal RNA gene, partial sequence | 1857 | 1857 | 99% | 0.0 | 99% | JX912367.1 |
| 56Select seq JN605747.1 | Pseudomonas brassicacearum strain J12 16S ribosomal RNA gene, partial sequence | 1857 | 1857 | 99% | 0.0 | 99% | JN605747.1 |
| 57Select seq FR675976.1 | Pseudomonas sp. LB184 partial 16S rRNA gene, isolate LB184 | 1857 | 1857 | 99% | 0.0 | 99% | FR675976.1 |
| 58Select seq FR675975.1 | Pseudomonas sp. LB183 partial 16S rRNA gene, isolate LB183 | 1857 | 1857 | 99% | 0.0 | 99% | FR675975.1 |
| 59Select seq HQ848635.1 | Pseudomonas sp. SHB3 16S ribosomal RNA gene, partial sequence | 1857 | 1857 | 99% | 0.0 | 99% | HQ848635.1 |
| 60Select seq JF500978.1 | Uncultured Pseudomonas sp. clone 101 16S ribosomal RNA gene, partial sequence | 1857 | 1857 | 99% | 0.0 | 99% | JF500978.1 |
| 61Select seq JF500932.1 | Uncultured Pseudomonas sp. clone 52 16S ribosomal RNA gene, partial sequence | 1857 | 1857 | 99% | 0.0 | 99% | JF500932.1 |
| 62Select seq CP002585.1 | Pseudomonas brassicacearum subsp. brassicacearum NFM421, complete genome | 1857 | 9279 | 99% | 0.0 | 99% | CP002585.1 |
| 63Select seq FN547411.1 | Pseudomonas sp. G68 partial 16S rRNA gene, strain G68 | 1857 | 1857 | 99% | 0.0 | 99% | FN547411.1 |
| 64Select seq DQ453838.1 | Pseudomonas sp. P96.25 16S ribosomal RNA gene, partial sequence | 1857 | 1857 | 99% | 0.0 | 99% | DQ453838.1 |
| 65Select seq DQ453837.1 | Pseudomonas sp. K93.3 16S ribosomal RNA gene, partial sequence | 1857 | 1857 | 99% | 0.0 | 99% | DQ453837.1 |
| 66Select seq DQ453836.1 | Pseudomonas sp. C\*1A1 16S ribosomal RNA gene, partial sequence | 1857 | 1857 | 99% | 0.0 | 99% | DQ453836.1 |
| 67Select seq DQ453835.1 | Pseudomonas sp. TM1A3 16S ribosomal RNA gene, partial sequence | 1857 | 1857 | 99% | 0.0 | 99% | DQ453835.1 |
| 68Select seq DQ453820.1 | Pseudomonas sp. K94.37 16S ribosomal RNA gene, complete sequence | 1857 | 1857 | 99% | 0.0 | 99% | DQ453820.1 |
| 69Select seq DQ453818.1 | Pseudomonas sp. K93.2 16S ribosomal RNA gene, complete sequence | 1857 | 1857 | 99% | 0.0 | 99% | DQ453818.1 |
| 70Select seq AY271792.1 | Pseudomonas aurantiaca VKM B-1524 16S ribosomal RNA gene, partial sequence | 1857 | 1857 | 99% | 0.0 | 99% | AY271792.1 |
| 71Select seq AY512624.1 | Pseudomonas sp. A1Y13 16S ribosomal RNA gene, partial sequence | 1857 | 1857 | 99% | 0.0 | 99% | AY512624.1 |
| 72Select seq AJ417074.1 | Pseudomonas sp. Q65c-80 16S rRNA gene, strain Q65c-80 | 1857 | 1857 | 99% | 0.0 | 99% | AJ417074.1 |
| 73Select seq AJ417068.1 | Pseudomonas sp. CM1'A2 16S rRNA gene, strain CM1'A2 | 1857 | 1857 | 99% | 0.0 | 99% | AJ417068.1 |
| 74Select seq AJ292381.1 | Pseudomonas brassicacearum 16S rRNA gene, strain 520-1 | 1857 | 1857 | 99% | 0.0 | 99% | AJ292381.1 |
| 75Select seq KU882739.1 | Pseudomonas sp. MAR9910 16S ribosomal RNA gene, partial sequence | 1855 | 1855 | 99% | 0.0 | 99% | KU882739.1 |
| 76Select seq KP730603.1 | Pseudomonas brassicacearum subsp. brassicacearum strain BW0808 16S ribosomal RNA gene, partial sequence | 1855 | 1855 | 98% | 0.0 | 99% | KP730603.1 |
| 77Select seq KM253151.1 | Pseudomonas sp. SR 1-04 16S ribosomal RNA gene, partial sequence | 1855 | 1855 | 99% | 0.0 | 99% | KM253151.1 |
| 78Select seq KJ642302.1 | Pseudomonas sp. EA\_S\_63 16S ribosomal RNA gene, partial sequence | 1855 | 1855 | 99% | 0.0 | 99% | KJ642302.1 |
| 79Select seq KJ642253.1 | Pseudomonas sp. EA\_R\_70 16S ribosomal RNA gene, partial sequence | 1855 | 1855 | 99% | 0.0 | 99% | KJ642253.1 |
| 80Select seq KJ642247.1 | Pseudomonas sp. EA\_R\_84 16S ribosomal RNA gene, partial sequence | 1855 | 1855 | 99% | 0.0 | 99% | KJ642247.1 |
| 81Select seq KF460526.1 | Pseudomonas fluorescens strain ALEB 7B 16S ribosomal RNA gene, partial sequence | 1855 | 1855 | 98% | 0.0 | 99% | KF460526.1 |
| 82Select seq KX953866.1 | Pseudomonas sp. strain C50T3 16S ribosomal RNA gene, partial sequence | 1853 | 1853 | 99% | 0.0 | 99% | KX953866.1 |
| 83Select seq KR088357.1 | Pseudomonas brassicacearum strain BK23 16S ribosomal RNA gene, partial sequence | 1853 | 1853 | 98% | 0.0 | 99% | KR088357.1 |
| 84Select seq HF952553.1 | Pseudomonas brassicacearum partial 16S rRNA gene, strain HMGU245 | 1853 | 1853 | 98% | 0.0 | 99% | HF952553.1 |
| 85Select seq CP025542.1 | Pseudomonas fluorescens strain 2P24 chromosome, complete genome | 1851 | 9246 | 99% | 0.0 | 99% | CP025542.1 |
| 86Select seq KX859163.1 | Pseudomonas sp. strain A22 16S ribosomal RNA gene, partial sequence | 1851 | 1851 | 99% | 0.0 | 99% | KX859163.1 |
| 87Select seq KX859162.1 | Pseudomonas sp. strain A21 16S ribosomal RNA gene, partial sequence | 1851 | 1851 | 99% | 0.0 | 99% | KX859162.1 |
| 88Select seq KY753310.1 | Pseudomonas brassicacearum strain YC-1 16S ribosomal RNA gene, partial sequence | 1851 | 1851 | 99% | 0.0 | 99% | KY753310.1 |
| 89Select seq KY111476.1 | Pseudomonas sp. strain B10 16S ribosomal RNA gene, partial sequence | 1851 | 1851 | 99% | 0.0 | 99% | KY111476.1 |
| 90Select seq LT629691.1 | Pseudomonas thivervalensis strain BS3779 genome assembly, chromosome: I | 1851 | 9235 | 99% | 0.0 | 99% | LT629691.1 |
| 91Select seq KR855698.1 | Pseudomonas brassicacearum strain SM27 16S ribosomal RNA gene, partial sequence | 1851 | 1851 | 99% | 0.0 | 99% | KR855698.1 |
| 92Select seq LN880129.1 | Uncultured Pseudomonas sp. partial 16S rRNA gene, clone W3S26 | 1851 | 1851 | 99% | 0.0 | 99% | LN880129.1 |
| 93Select seq KT580640.1 | Pseudomonas fluorescens strain CanR-1 16S ribosomal RNA gene, partial sequence | 1851 | 1851 | 99% | 0.0 | 99% | KT580640.1 |
| 94Select seq KM187303.1 | Pseudomonas sp. CC6M 16S ribosomal RNA gene, partial sequence | 1851 | 1851 | 99% | 0.0 | 99% | KM187303.1 |
| 95Select seq LC015568.1 | Pseudomonas brassicacearum subsp. brassicacearum gene for 16S ribosomal RNA, partial sequence, strain: AF78 | 1851 | 1851 | 99% | 0.0 | 99% | LC015568.1 |
| 96Select seq KJ642348.1 | Pseudomonas sp. EA\_S\_74 16S ribosomal RNA gene, partial sequence | 1851 | 1851 | 99% | 0.0 | 99% | KJ642348.1 |
| 97Select seq KJ642346.1 | Pseudomonas sp. EA\_S\_77 16S ribosomal RNA gene, partial sequence | 1851 | 1851 | 99% | 0.0 | 99% | KJ642346.1 |
| 98Select seq KJ642309.1 | Pseudomonas sp. EA\_S\_27 16S ribosomal RNA gene, partial sequence | 1851 | 1851 | 99% | 0.0 | 99% | KJ642309.1 |
| 99Select seq KJ642307.1 | Pseudomonas sp. EA\_S\_71 16S ribosomal RNA gene, partial sequence | 1851 | 1851 | 99% | 0.0 | 99% | KJ642307.1 |
| 100Select seq KJ642306.1 | Pseudomonas sp. EA\_S\_7 16S ribosomal RNA gene, partial sequence | 1851 | 1851 | 99% | 0.0 | 99% | KJ642306.1 |


## Alignments

Loading alignment... for sequences gi|719245556,gi|444438253,gi|1137473154,gi|1008987913,gi|1018445764 Reading indexes 1-5

Download

FASTA (complete sequence)

FASTA (aligned sequences)

GenBank (complete sequence)

Continue
Cancel

GenBankGraphics

Next
Previous
Descriptions

Pseudomonas sp. SAM1 16S ribosomal RNA gene, partial sequence

Sequence ID: KM269192.1Length: 1439Number of Matches: 1

Related Information

Range 1: 21 to 1033GenBankGraphics

Next Match
Previous Match
First Match

Alignment statistics for match #1

| Score | Expect | Identities | Gaps | Strand | Frame |
| --- | --- | --- | --- | --- | --- |
| 1866 bits(1010) | 0.0() | 1013/1014(99%) | 1/1014(0%) | Plus/Plus |  |

Features:

```
Query  7     TGCAGTCGAGCGGTAGAGAGGTGCTTGCACCTCTTGAGAGCGGCGGACGGGTGAGTAATG  66
             ||||||||||||||||||||||||||||||||||||||||||||||||||||||||||||
Sbjct  21    TGCAGTCGAGCGGTAGAGAGGTGCTTGCACCTCTTGAGAGCGGCGGACGGGTGAGTAATG  80

Query  67    CCTAGGAATCTGCCTGGTAGTGGGGGATAACGCTCGGAAACGGACGCTAATACCGCATAC  126
             ||||||||||||||||||||||||||||||||||||||||||||||||||||||||||||
Sbjct  81    CCTAGGAATCTGCCTGGTAGTGGGGGATAACGCTCGGAAACGGACGCTAATACCGCATAC  140

Query  127   GTCCTACGGGAGAAAGCAGGGGACCTTCGGGCCTTGCGCTATCAGATGAGCCTAGGTCGG  186
             ||||||||||||||||||||||||||||||||||||||||||||||||||||||||||||
Sbjct  141   GTCCTACGGGAGAAAGCAGGGGACCTTCGGGCCTTGCGCTATCAGATGAGCCTAGGTCGG  200

Query  187   ATTAGCTAGTTGGTGGGGTAATGGCTCACCAAGGCGACGATCCGTAACTGGTCTGAGAGG  246
             ||||||||||||||||||||||||||||||||||||||||||||||||||||||||||||
Sbjct  201   ATTAGCTAGTTGGTGGGGTAATGGCTCACCAAGGCGACGATCCGTAACTGGTCTGAGAGG  260

Query  247   ATGATCAGTCACACTGGAACTGAGACACGGTCCAGACTCCTACGGGAGGCAGCAGTGGGG  306
             ||||||||||||||||||||||||||||||||||||||||||||||||||||||||||||
Sbjct  261   ATGATCAGTCACACTGGAACTGAGACACGGTCCAGACTCCTACGGGAGGCAGCAGTGGGG  320

Query  307   AATATTGGACAATGGGCGAAAGCCTGATCCAGCCATGCCGCGTGTGTGAAGAAGGTCTTC  366
             ||||||||||||||||||||||||||||||||||||||||||||||||||||||||||||
Sbjct  321   AATATTGGACAATGGGCGAAAGCCTGATCCAGCCATGCCGCGTGTGTGAAGAAGGTCTTC  380

Query  367   GGATTGTAAAGCACTTTAAGTTGGGAGGAAGGGCATTAACCTAATACGTTAGTGTTTTGA  426
             ||||||||||||||||||||||||||||||||||||||||||||||||||||||||||||
Sbjct  381   GGATTGTAAAGCACTTTAAGTTGGGAGGAAGGGCATTAACCTAATACGTTAGTGTTTTGA  440

Query  427   CGTTACCGACAGAATAAGCACCGGCTAACTCTGTGCCAGCAGCCGCGGTAATACAGAGGG  486
             ||||||||||||||||||||||||||||||||||||||||||||||||||||||||||||
Sbjct  441   CGTTACCGACAGAATAAGCACCGGCTAACTCTGTGCCAGCAGCCGCGGTAATACAGAGGG  500

Query  487   TGCAAGCGTTAATCGGAATTACTGGGCGTAAAGCGCGCGTAGGTGGTTCGTTAAGTTGGA  546
             ||||||||||||||||||||||||||||||||||||||||||||||||||||||||||||
Sbjct  501   TGCAAGCGTTAATCGGAATTACTGGGCGTAAAGCGCGCGTAGGTGGTTCGTTAAGTTGGA  560

Query  547   TGTGAAAGCCCCGGGCTCAACCTGGGAACTGCATTCAAAACTGTCGAGCTAGAGTATGGT  606
             ||||||||||||||||||||||||||||||||||||||||||||||||||||||||||||
Sbjct  561   TGTGAAAGCCCCGGGCTCAACCTGGGAACTGCATTCAAAACTGTCGAGCTAGAGTATGGT  620

Query  607   AGAGGGTGGTGGAATTTCCTGTGTAGCGGTGAAATGCGTAGATATAGGAAGGAACACCAG  666
             ||||||||||||||||||||||||||||||||||||||||||||||||||||||||||||
Sbjct  621   AGAGGGTGGTGGAATTTCCTGTGTAGCGGTGAAATGCGTAGATATAGGAAGGAACACCAG  680

Query  667   TGGCGAAGGCGACCACCTGGACTGATACTGACACTGAGGTGCGAAAGCGTGGGGAGCAAA  726
             ||||||||||||||||||||||||||||||||||||||||||||||||||||||||||||
Sbjct  681   TGGCGAAGGCGACCACCTGGACTGATACTGACACTGAGGTGCGAAAGCGTGGGGAGCAAA  740

Query  727   CAGGATTAGATACCCTGGTAGTCCACGCCGTAAACGATGTCAACTAGCCGTTGGGAGCCT  786
             ||||||||||||||||||||||||||||||||||||||||||||||||||||||||||||
Sbjct  741   CAGGATTAGATACCCTGGTAGTCCACGCCGTAAACGATGTCAACTAGCCGTTGGGAGCCT  800

Query  787   TGAGCTCTTAGTGGCGCAGCTAACGCATTAAGTTGACCGCCTGGGGGAGTACGGCCGCAA  846
             |||||||||||||||||||||||||||||||||||||||||| |||||||||||||||||
Sbjct  801   TGAGCTCTTAGTGGCGCAGCTAACGCATTAAGTTGACCGCCT-GGGGAGTACGGCCGCAA  859

Query  847   GGTTAAAACTCAAATGAATTGACGGGGGCCCGCACAAGCGGTGGAGCATGTGGTTTAATT  906
             ||||||||||||||||||||||||||||||||||||||||||||||||||||||||||||
Sbjct  860   GGTTAAAACTCAAATGAATTGACGGGGGCCCGCACAAGCGGTGGAGCATGTGGTTTAATT  919

Query  907   CGAAGCAACGCGAAGAACCTTACCAGGCCTTGACATCCAATGAACTTTCCAGAGATGGAT  966
             ||||||||||||||||||||||||||||||||||||||||||||||||||||||||||||
Sbjct  920   CGAAGCAACGCGAAGAACCTTACCAGGCCTTGACATCCAATGAACTTTCCAGAGATGGAT  979

Query  967   TGGTGCCTTCGGGAACATTGAGACAGGTGCTGCATGGCTGTCGTCAGCTCGTGT  1020
             ||||||||||||||||||||||||||||||||||||||||||||||||||||||
Sbjct  980   TGGTGCCTTCGGGAACATTGAGACAGGTGCTGCATGGCTGTCGTCAGCTCGTGT  1033
```

Download

FASTA (complete sequence)

FASTA (aligned sequences)

GenBank (complete sequence)

Continue
Cancel

GenBankGraphics

Next
Previous
Descriptions

Pseudomonas thivervalensis strain h-23 16S ribosomal RNA gene, partial sequence

Sequence ID: KC139440.1Length: 1415Number of Matches: 1

Related Information

Range 1: 12 to 1024GenBankGraphics

Next Match
Previous Match
First Match

Alignment statistics for match #1

| Score | Expect | Identities | Gaps | Strand | Frame |
| --- | --- | --- | --- | --- | --- |
| 1866 bits(1010) | 0.0() | 1013/1014(99%) | 1/1014(0%) | Plus/Plus |  |

Features:

```
Query  7     TGCAGTCGAGCGGTAGAGAGGTGCTTGCACCTCTTGAGAGCGGCGGACGGGTGAGTAATG  66
             ||||||||||||||||||||||||||||||||||||||||||||||||||||||||||||
Sbjct  12    TGCAGTCGAGCGGTAGAGAGGTGCTTGCACCTCTTGAGAGCGGCGGACGGGTGAGTAATG  71

Query  67    CCTAGGAATCTGCCTGGTAGTGGGGGATAACGCTCGGAAACGGACGCTAATACCGCATAC  126
             ||||||||||||||||||||||||||||||||||||||||||||||||||||||||||||
Sbjct  72    CCTAGGAATCTGCCTGGTAGTGGGGGATAACGCTCGGAAACGGACGCTAATACCGCATAC  131

Query  127   GTCCTACGGGAGAAAGCAGGGGACCTTCGGGCCTTGCGCTATCAGATGAGCCTAGGTCGG  186
             ||||||||||||||||||||||||||||||||||||||||||||||||||||||||||||
Sbjct  132   GTCCTACGGGAGAAAGCAGGGGACCTTCGGGCCTTGCGCTATCAGATGAGCCTAGGTCGG  191

Query  187   ATTAGCTAGTTGGTGGGGTAATGGCTCACCAAGGCGACGATCCGTAACTGGTCTGAGAGG  246
             ||||||||||||||||||||||||||||||||||||||||||||||||||||||||||||
Sbjct  192   ATTAGCTAGTTGGTGGGGTAATGGCTCACCAAGGCGACGATCCGTAACTGGTCTGAGAGG  251

Query  247   ATGATCAGTCACACTGGAACTGAGACACGGTCCAGACTCCTACGGGAGGCAGCAGTGGGG  306
             ||||||||||||||||||||||||||||||||||||||||||||||||||||||||||||
Sbjct  252   ATGATCAGTCACACTGGAACTGAGACACGGTCCAGACTCCTACGGGAGGCAGCAGTGGGG  311

Query  307   AATATTGGACAATGGGCGAAAGCCTGATCCAGCCATGCCGCGTGTGTGAAGAAGGTCTTC  366
             ||||||||||||||||||||||||||||||||||||||||||||||||||||||||||||
Sbjct  312   AATATTGGACAATGGGCGAAAGCCTGATCCAGCCATGCCGCGTGTGTGAAGAAGGTCTTC  371

Query  367   GGATTGTAAAGCACTTTAAGTTGGGAGGAAGGGCATTAACCTAATACGTTAGTGTTTTGA  426
             ||||||||||||||||||||||||||||||||||||||||||||||||||||||||||||
Sbjct  372   GGATTGTAAAGCACTTTAAGTTGGGAGGAAGGGCATTAACCTAATACGTTAGTGTTTTGA  431

Query  427   CGTTACCGACAGAATAAGCACCGGCTAACTCTGTGCCAGCAGCCGCGGTAATACAGAGGG  486
             ||||||||||||||||||||||||||||||||||||||||||||||||||||||||||||
Sbjct  432   CGTTACCGACAGAATAAGCACCGGCTAACTCTGTGCCAGCAGCCGCGGTAATACAGAGGG  491

Query  487   TGCAAGCGTTAATCGGAATTACTGGGCGTAAAGCGCGCGTAGGTGGTTCGTTAAGTTGGA  546
             ||||||||||||||||||||||||||||||||||||||||||||||||||||||||||||
Sbjct  492   TGCAAGCGTTAATCGGAATTACTGGGCGTAAAGCGCGCGTAGGTGGTTCGTTAAGTTGGA  551

Query  547   TGTGAAAGCCCCGGGCTCAACCTGGGAACTGCATTCAAAACTGTCGAGCTAGAGTATGGT  606
             ||||||||||||||||||||||||||||||||||||||||||||||||||||||||||||
Sbjct  552   TGTGAAAGCCCCGGGCTCAACCTGGGAACTGCATTCAAAACTGTCGAGCTAGAGTATGGT  611

Query  607   AGAGGGTGGTGGAATTTCCTGTGTAGCGGTGAAATGCGTAGATATAGGAAGGAACACCAG  666
             ||||||||||||||||||||||||||||||||||||||||||||||||||||||||||||
Sbjct  612   AGAGGGTGGTGGAATTTCCTGTGTAGCGGTGAAATGCGTAGATATAGGAAGGAACACCAG  671

Query  667   TGGCGAAGGCGACCACCTGGACTGATACTGACACTGAGGTGCGAAAGCGTGGGGAGCAAA  726
             ||||||||||||||||||||||||||||||||||||||||||||||||||||||||||||
Sbjct  672   TGGCGAAGGCGACCACCTGGACTGATACTGACACTGAGGTGCGAAAGCGTGGGGAGCAAA  731

Query  727   CAGGATTAGATACCCTGGTAGTCCACGCCGTAAACGATGTCAACTAGCCGTTGGGAGCCT  786
             ||||||||||||||||||||||||||||||||||||||||||||||||||||||||||||
Sbjct  732   CAGGATTAGATACCCTGGTAGTCCACGCCGTAAACGATGTCAACTAGCCGTTGGGAGCCT  791

Query  787   TGAGCTCTTAGTGGCGCAGCTAACGCATTAAGTTGACCGCCTGGGGGAGTACGGCCGCAA  846
             |||||||||||||||||||||||||||||||||||||||||| |||||||||||||||||
Sbjct  792   TGAGCTCTTAGTGGCGCAGCTAACGCATTAAGTTGACCGCCT-GGGGAGTACGGCCGCAA  850

Query  847   GGTTAAAACTCAAATGAATTGACGGGGGCCCGCACAAGCGGTGGAGCATGTGGTTTAATT  906
             ||||||||||||||||||||||||||||||||||||||||||||||||||||||||||||
Sbjct  851   GGTTAAAACTCAAATGAATTGACGGGGGCCCGCACAAGCGGTGGAGCATGTGGTTTAATT  910

Query  907   CGAAGCAACGCGAAGAACCTTACCAGGCCTTGACATCCAATGAACTTTCCAGAGATGGAT  966
             ||||||||||||||||||||||||||||||||||||||||||||||||||||||||||||
Sbjct  911   CGAAGCAACGCGAAGAACCTTACCAGGCCTTGACATCCAATGAACTTTCCAGAGATGGAT  970

Query  967   TGGTGCCTTCGGGAACATTGAGACAGGTGCTGCATGGCTGTCGTCAGCTCGTGT  1020
             ||||||||||||||||||||||||||||||||||||||||||||||||||||||
Sbjct  971   TGGTGCCTTCGGGAACATTGAGACAGGTGCTGCATGGCTGTCGTCAGCTCGTGT  1024
```

Download

FASTA (complete sequence)

FASTA (aligned sequences)

GenBank (complete sequence)

Continue
Cancel

GenBankGraphics

Next
Previous
Descriptions

Pseudomonas sp. strain 7.3 16S ribosomal RNA gene, partial sequence

Sequence ID: KY542120.1Length: 1546Number of Matches: 1

Related Information

Range 1: 56 to 1069GenBankGraphics

Next Match
Previous Match
First Match

Alignment statistics for match #1

| Score | Expect | Identities | Gaps | Strand | Frame |
| --- | --- | --- | --- | --- | --- |
| 1862 bits(1008) | 0.0() | 1013/1015(99%) | 2/1015(0%) | Plus/Plus |  |

Features:

```
Query  7     TGC-AGTCGAGCGGTAGAGAGGTGCTTGCACCTCTTGAGAGCGGCGGACGGGTGAGTAAT  65
             ||| ||||||||||||||||||||||||||||||||||||||||||||||||||||||||
Sbjct  56    TGCAAGTCGAGCGGTAGAGAGGTGCTTGCACCTCTTGAGAGCGGCGGACGGGTGAGTAAT  115

Query  66    GCCTAGGAATCTGCCTGGTAGTGGGGGATAACGCTCGGAAACGGACGCTAATACCGCATA  125
             ||||||||||||||||||||||||||||||||||||||||||||||||||||||||||||
Sbjct  116   GCCTAGGAATCTGCCTGGTAGTGGGGGATAACGCTCGGAAACGGACGCTAATACCGCATA  175

Query  126   CGTCCTACGGGAGAAAGCAGGGGACCTTCGGGCCTTGCGCTATCAGATGAGCCTAGGTCG  185
             ||||||||||||||||||||||||||||||||||||||||||||||||||||||||||||
Sbjct  176   CGTCCTACGGGAGAAAGCAGGGGACCTTCGGGCCTTGCGCTATCAGATGAGCCTAGGTCG  235

Query  186   GATTAGCTAGTTGGTGGGGTAATGGCTCACCAAGGCGACGATCCGTAACTGGTCTGAGAG  245
             ||||||||||||||||||||||||||||||||||||||||||||||||||||||||||||
Sbjct  236   GATTAGCTAGTTGGTGGGGTAATGGCTCACCAAGGCGACGATCCGTAACTGGTCTGAGAG  295

Query  246   GATGATCAGTCACACTGGAACTGAGACACGGTCCAGACTCCTACGGGAGGCAGCAGTGGG  305
             ||||||||||||||||||||||||||||||||||||||||||||||||||||||||||||
Sbjct  296   GATGATCAGTCACACTGGAACTGAGACACGGTCCAGACTCCTACGGGAGGCAGCAGTGGG  355

Query  306   GAATATTGGACAATGGGCGAAAGCCTGATCCAGCCATGCCGCGTGTGTGAAGAAGGTCTT  365
             ||||||||||||||||||||||||||||||||||||||||||||||||||||||||||||
Sbjct  356   GAATATTGGACAATGGGCGAAAGCCTGATCCAGCCATGCCGCGTGTGTGAAGAAGGTCTT  415

Query  366   CGGATTGTAAAGCACTTTAAGTTGGGAGGAAGGGCATTAACCTAATACGTTAGTGTTTTG  425
             ||||||||||||||||||||||||||||||||||||||||||||||||||||||||||||
Sbjct  416   CGGATTGTAAAGCACTTTAAGTTGGGAGGAAGGGCATTAACCTAATACGTTAGTGTTTTG  475

Query  426   ACGTTACCGACAGAATAAGCACCGGCTAACTCTGTGCCAGCAGCCGCGGTAATACAGAGG  485
             ||||||||||||||||||||||||||||||||||||||||||||||||||||||||||||
Sbjct  476   ACGTTACCGACAGAATAAGCACCGGCTAACTCTGTGCCAGCAGCCGCGGTAATACAGAGG  535

Query  486   GTGCAAGCGTTAATCGGAATTACTGGGCGTAAAGCGCGCGTAGGTGGTTCGTTAAGTTGG  545
             ||||||||||||||||||||||||||||||||||||||||||||||||||||||||||||
Sbjct  536   GTGCAAGCGTTAATCGGAATTACTGGGCGTAAAGCGCGCGTAGGTGGTTCGTTAAGTTGG  595

Query  546   ATGTGAAAGCCCCGGGCTCAACCTGGGAACTGCATTCAAAACTGTCGAGCTAGAGTATGG  605
             ||||||||||||||||||||||||||||||||||||||||||||||||||||||||||||
Sbjct  596   ATGTGAAAGCCCCGGGCTCAACCTGGGAACTGCATTCAAAACTGTCGAGCTAGAGTATGG  655

Query  606   TAGAGGGTGGTGGAATTTCCTGTGTAGCGGTGAAATGCGTAGATATAGGAAGGAACACCA  665
             ||||||||||||||||||||||||||||||||||||||||||||||||||||||||||||
Sbjct  656   TAGAGGGTGGTGGAATTTCCTGTGTAGCGGTGAAATGCGTAGATATAGGAAGGAACACCA  715

Query  666   GTGGCGAAGGCGACCACCTGGACTGATACTGACACTGAGGTGCGAAAGCGTGGGGAGCAA  725
             ||||||||||||||||||||||||||||||||||||||||||||||||||||||||||||
Sbjct  716   GTGGCGAAGGCGACCACCTGGACTGATACTGACACTGAGGTGCGAAAGCGTGGGGAGCAA  775

Query  726   ACAGGATTAGATACCCTGGTAGTCCACGCCGTAAACGATGTCAACTAGCCGTTGGGAGCC  785
             ||||||||||||||||||||||||||||||||||||||||||||||||||||||||||||
Sbjct  776   ACAGGATTAGATACCCTGGTAGTCCACGCCGTAAACGATGTCAACTAGCCGTTGGGAGCC  835

Query  786   TTGAGCTCTTAGTGGCGCAGCTAACGCATTAAGTTGACCGCCTGGGGGAGTACGGCCGCA  845
             ||||||||||||||||||||||||||||||||||||||||||| ||||||||||||||||
Sbjct  836   TTGAGCTCTTAGTGGCGCAGCTAACGCATTAAGTTGACCGCCT-GGGGAGTACGGCCGCA  894

Query  846   AGGTTAAAACTCAAATGAATTGACGGGGGCCCGCACAAGCGGTGGAGCATGTGGTTTAAT  905
             ||||||||||||||||||||||||||||||||||||||||||||||||||||||||||||
Sbjct  895   AGGTTAAAACTCAAATGAATTGACGGGGGCCCGCACAAGCGGTGGAGCATGTGGTTTAAT  954

Query  906   TCGAAGCAACGCGAAGAACCTTACCAGGCCTTGACATCCAATGAACTTTCCAGAGATGGA  965
             ||||||||||||||||||||||||||||||||||||||||||||||||||||||||||||
Sbjct  955   TCGAAGCAACGCGAAGAACCTTACCAGGCCTTGACATCCAATGAACTTTCCAGAGATGGA  1014

Query  966   TTGGTGCCTTCGGGAACATTGAGACAGGTGCTGCATGGCTGTCGTCAGCTCGTGT  1020
             |||||||||||||||||||||||||||||||||||||||||||||||||||||||
Sbjct  1015  TTGGTGCCTTCGGGAACATTGAGACAGGTGCTGCATGGCTGTCGTCAGCTCGTGT  1069
```

Download

FASTA (complete sequence)

FASTA (aligned sequences)

GenBank (complete sequence)

Continue
Cancel

GenBankGraphics

Next
Previous
Descriptions

Pseudomonas sp. MAR9909 16S ribosomal RNA gene, partial sequence

Sequence ID: KU882744.1Length: 1435Number of Matches: 1

Related Information

Range 1: 1 to 1014GenBankGraphics

Next Match
Previous Match
First Match

Alignment statistics for match #1

| Score | Expect | Identities | Gaps | Strand | Frame |
| --- | --- | --- | --- | --- | --- |
| 1862 bits(1008) | 0.0() | 1013/1015(99%) | 2/1015(0%) | Plus/Plus |  |

Features:

```
Query  7     TGC-AGTCGAGCGGTAGAGAGGTGCTTGCACCTCTTGAGAGCGGCGGACGGGTGAGTAAT  65
             ||| ||||||||||||||||||||||||||||||||||||||||||||||||||||||||
Sbjct  1     TGCAAGTCGAGCGGTAGAGAGGTGCTTGCACCTCTTGAGAGCGGCGGACGGGTGAGTAAT  60

Query  66    GCCTAGGAATCTGCCTGGTAGTGGGGGATAACGCTCGGAAACGGACGCTAATACCGCATA  125
             ||||||||||||||||||||||||||||||||||||||||||||||||||||||||||||
Sbjct  61    GCCTAGGAATCTGCCTGGTAGTGGGGGATAACGCTCGGAAACGGACGCTAATACCGCATA  120

Query  126   CGTCCTACGGGAGAAAGCAGGGGACCTTCGGGCCTTGCGCTATCAGATGAGCCTAGGTCG  185
             ||||||||||||||||||||||||||||||||||||||||||||||||||||||||||||
Sbjct  121   CGTCCTACGGGAGAAAGCAGGGGACCTTCGGGCCTTGCGCTATCAGATGAGCCTAGGTCG  180

Query  186   GATTAGCTAGTTGGTGGGGTAATGGCTCACCAAGGCGACGATCCGTAACTGGTCTGAGAG  245
             ||||||||||||||||||||||||||||||||||||||||||||||||||||||||||||
Sbjct  181   GATTAGCTAGTTGGTGGGGTAATGGCTCACCAAGGCGACGATCCGTAACTGGTCTGAGAG  240

Query  246   GATGATCAGTCACACTGGAACTGAGACACGGTCCAGACTCCTACGGGAGGCAGCAGTGGG  305
             ||||||||||||||||||||||||||||||||||||||||||||||||||||||||||||
Sbjct  241   GATGATCAGTCACACTGGAACTGAGACACGGTCCAGACTCCTACGGGAGGCAGCAGTGGG  300

Query  306   GAATATTGGACAATGGGCGAAAGCCTGATCCAGCCATGCCGCGTGTGTGAAGAAGGTCTT  365
             ||||||||||||||||||||||||||||||||||||||||||||||||||||||||||||
Sbjct  301   GAATATTGGACAATGGGCGAAAGCCTGATCCAGCCATGCCGCGTGTGTGAAGAAGGTCTT  360

Query  366   CGGATTGTAAAGCACTTTAAGTTGGGAGGAAGGGCATTAACCTAATACGTTAGTGTTTTG  425
             ||||||||||||||||||||||||||||||||||||||||||||||||||||||||||||
Sbjct  361   CGGATTGTAAAGCACTTTAAGTTGGGAGGAAGGGCATTAACCTAATACGTTAGTGTTTTG  420

Query  426   ACGTTACCGACAGAATAAGCACCGGCTAACTCTGTGCCAGCAGCCGCGGTAATACAGAGG  485
             ||||||||||||||||||||||||||||||||||||||||||||||||||||||||||||
Sbjct  421   ACGTTACCGACAGAATAAGCACCGGCTAACTCTGTGCCAGCAGCCGCGGTAATACAGAGG  480

Query  486   GTGCAAGCGTTAATCGGAATTACTGGGCGTAAAGCGCGCGTAGGTGGTTCGTTAAGTTGG  545
             ||||||||||||||||||||||||||||||||||||||||||||||||||||||||||||
Sbjct  481   GTGCAAGCGTTAATCGGAATTACTGGGCGTAAAGCGCGCGTAGGTGGTTCGTTAAGTTGG  540

Query  546   ATGTGAAAGCCCCGGGCTCAACCTGGGAACTGCATTCAAAACTGTCGAGCTAGAGTATGG  605
             ||||||||||||||||||||||||||||||||||||||||||||||||||||||||||||
Sbjct  541   ATGTGAAAGCCCCGGGCTCAACCTGGGAACTGCATTCAAAACTGTCGAGCTAGAGTATGG  600

Query  606   TAGAGGGTGGTGGAATTTCCTGTGTAGCGGTGAAATGCGTAGATATAGGAAGGAACACCA  665
             ||||||||||||||||||||||||||||||||||||||||||||||||||||||||||||
Sbjct  601   TAGAGGGTGGTGGAATTTCCTGTGTAGCGGTGAAATGCGTAGATATAGGAAGGAACACCA  660

Query  666   GTGGCGAAGGCGACCACCTGGACTGATACTGACACTGAGGTGCGAAAGCGTGGGGAGCAA  725
             ||||||||||||||||||||||||||||||||||||||||||||||||||||||||||||
Sbjct  661   GTGGCGAAGGCGACCACCTGGACTGATACTGACACTGAGGTGCGAAAGCGTGGGGAGCAA  720

Query  726   ACAGGATTAGATACCCTGGTAGTCCACGCCGTAAACGATGTCAACTAGCCGTTGGGAGCC  785
             ||||||||||||||||||||||||||||||||||||||||||||||||||||||||||||
Sbjct  721   ACAGGATTAGATACCCTGGTAGTCCACGCCGTAAACGATGTCAACTAGCCGTTGGGAGCC  780

Query  786   TTGAGCTCTTAGTGGCGCAGCTAACGCATTAAGTTGACCGCCTGGGGGAGTACGGCCGCA  845
             ||||||||||||||||||||||||||||||||||||||||||| ||||||||||||||||
Sbjct  781   TTGAGCTCTTAGTGGCGCAGCTAACGCATTAAGTTGACCGCCT-GGGGAGTACGGCCGCA  839

Query  846   AGGTTAAAACTCAAATGAATTGACGGGGGCCCGCACAAGCGGTGGAGCATGTGGTTTAAT  905
             ||||||||||||||||||||||||||||||||||||||||||||||||||||||||||||
Sbjct  840   AGGTTAAAACTCAAATGAATTGACGGGGGCCCGCACAAGCGGTGGAGCATGTGGTTTAAT  899

Query  906   TCGAAGCAACGCGAAGAACCTTACCAGGCCTTGACATCCAATGAACTTTCCAGAGATGGA  965
             ||||||||||||||||||||||||||||||||||||||||||||||||||||||||||||
Sbjct  900   TCGAAGCAACGCGAAGAACCTTACCAGGCCTTGACATCCAATGAACTTTCCAGAGATGGA  959

Query  966   TTGGTGCCTTCGGGAACATTGAGACAGGTGCTGCATGGCTGTCGTCAGCTCGTGT  1020
             |||||||||||||||||||||||||||||||||||||||||||||||||||||||
Sbjct  960   TTGGTGCCTTCGGGAACATTGAGACAGGTGCTGCATGGCTGTCGTCAGCTCGTGT  1014
```

Download

FASTA (complete sequence)

FASTA (aligned sequences)

GenBank (complete sequence)

Continue
Cancel

GenBankGraphics

Next
Previous
Descriptions

Pseudomonas brassicacearum subsp. neoaurantiaca strain MLS-8-1 16S ribosomal RNA gene, partial sequence

Sequence ID: KT997466.1Length: 1380Number of Matches: 1

Related Information

Range 1: 2 to 1012GenBankGraphics

Next Match
Previous Match
First Match

Alignment statistics for match #1

| Score | Expect | Identities | Gaps | Strand | Frame |
| --- | --- | --- | --- | --- | --- |
| 1862 bits(1008) | 0.0() | 1011/1012(99%) | 1/1012(0%) | Plus/Plus |  |

Features:

```
Query  9     CAGTCGAGCGGTAGAGAGGTGCTTGCACCTCTTGAGAGCGGCGGACGGGTGAGTAATGCC  68
             ||||||||||||||||||||||||||||||||||||||||||||||||||||||||||||
Sbjct  2     CAGTCGAGCGGTAGAGAGGTGCTTGCACCTCTTGAGAGCGGCGGACGGGTGAGTAATGCC  61

Query  69    TAGGAATCTGCCTGGTAGTGGGGGATAACGCTCGGAAACGGACGCTAATACCGCATACGT  128
             ||||||||||||||||||||||||||||||||||||||||||||||||||||||||||||
Sbjct  62    TAGGAATCTGCCTGGTAGTGGGGGATAACGCTCGGAAACGGACGCTAATACCGCATACGT  121

Query  129   CCTACGGGAGAAAGCAGGGGACCTTCGGGCCTTGCGCTATCAGATGAGCCTAGGTCGGAT  188
             ||||||||||||||||||||||||||||||||||||||||||||||||||||||||||||
Sbjct  122   CCTACGGGAGAAAGCAGGGGACCTTCGGGCCTTGCGCTATCAGATGAGCCTAGGTCGGAT  181

Query  189   TAGCTAGTTGGTGGGGTAATGGCTCACCAAGGCGACGATCCGTAACTGGTCTGAGAGGAT  248
             ||||||||||||||||||||||||||||||||||||||||||||||||||||||||||||
Sbjct  182   TAGCTAGTTGGTGGGGTAATGGCTCACCAAGGCGACGATCCGTAACTGGTCTGAGAGGAT  241

Query  249   GATCAGTCACACTGGAACTGAGACACGGTCCAGACTCCTACGGGAGGCAGCAGTGGGGAA  308
             ||||||||||||||||||||||||||||||||||||||||||||||||||||||||||||
Sbjct  242   GATCAGTCACACTGGAACTGAGACACGGTCCAGACTCCTACGGGAGGCAGCAGTGGGGAA  301

Query  309   TATTGGACAATGGGCGAAAGCCTGATCCAGCCATGCCGCGTGTGTGAAGAAGGTCTTCGG  368
             ||||||||||||||||||||||||||||||||||||||||||||||||||||||||||||
Sbjct  302   TATTGGACAATGGGCGAAAGCCTGATCCAGCCATGCCGCGTGTGTGAAGAAGGTCTTCGG  361

Query  369   ATTGTAAAGCACTTTAAGTTGGGAGGAAGGGCATTAACCTAATACGTTAGTGTTTTGACG  428
             ||||||||||||||||||||||||||||||||||||||||||||||||||||||||||||
Sbjct  362   ATTGTAAAGCACTTTAAGTTGGGAGGAAGGGCATTAACCTAATACGTTAGTGTTTTGACG  421

Query  429   TTACCGACAGAATAAGCACCGGCTAACTCTGTGCCAGCAGCCGCGGTAATACAGAGGGTG  488
             ||||||||||||||||||||||||||||||||||||||||||||||||||||||||||||
Sbjct  422   TTACCGACAGAATAAGCACCGGCTAACTCTGTGCCAGCAGCCGCGGTAATACAGAGGGTG  481

Query  489   CAAGCGTTAATCGGAATTACTGGGCGTAAAGCGCGCGTAGGTGGTTCGTTAAGTTGGATG  548
             ||||||||||||||||||||||||||||||||||||||||||||||||||||||||||||
Sbjct  482   CAAGCGTTAATCGGAATTACTGGGCGTAAAGCGCGCGTAGGTGGTTCGTTAAGTTGGATG  541

Query  549   TGAAAGCCCCGGGCTCAACCTGGGAACTGCATTCAAAACTGTCGAGCTAGAGTATGGTAG  608
             ||||||||||||||||||||||||||||||||||||||||||||||||||||||||||||
Sbjct  542   TGAAAGCCCCGGGCTCAACCTGGGAACTGCATTCAAAACTGTCGAGCTAGAGTATGGTAG  601

Query  609   AGGGTGGTGGAATTTCCTGTGTAGCGGTGAAATGCGTAGATATAGGAAGGAACACCAGTG  668
             ||||||||||||||||||||||||||||||||||||||||||||||||||||||||||||
Sbjct  602   AGGGTGGTGGAATTTCCTGTGTAGCGGTGAAATGCGTAGATATAGGAAGGAACACCAGTG  661

Query  669   GCGAAGGCGACCACCTGGACTGATACTGACACTGAGGTGCGAAAGCGTGGGGAGCAAACA  728
             ||||||||||||||||||||||||||||||||||||||||||||||||||||||||||||
Sbjct  662   GCGAAGGCGACCACCTGGACTGATACTGACACTGAGGTGCGAAAGCGTGGGGAGCAAACA  721

Query  729   GGATTAGATACCCTGGTAGTCCACGCCGTAAACGATGTCAACTAGCCGTTGGGAGCCTTG  788
             ||||||||||||||||||||||||||||||||||||||||||||||||||||||||||||
Sbjct  722   GGATTAGATACCCTGGTAGTCCACGCCGTAAACGATGTCAACTAGCCGTTGGGAGCCTTG  781

Query  789   AGCTCTTAGTGGCGCAGCTAACGCATTAAGTTGACCGCCTGGGGGAGTACGGCCGCAAGG  848
             |||||||||||||||||||||||||||||||||||||||| |||||||||||||||||||
Sbjct  782   AGCTCTTAGTGGCGCAGCTAACGCATTAAGTTGACCGCCT-GGGGAGTACGGCCGCAAGG  840

Query  849   TTAAAACTCAAATGAATTGACGGGGGCCCGCACAAGCGGTGGAGCATGTGGTTTAATTCG  908
             ||||||||||||||||||||||||||||||||||||||||||||||||||||||||||||
Sbjct  841   TTAAAACTCAAATGAATTGACGGGGGCCCGCACAAGCGGTGGAGCATGTGGTTTAATTCG  900

Query  909   AAGCAACGCGAAGAACCTTACCAGGCCTTGACATCCAATGAACTTTCCAGAGATGGATTG  968
             ||||||||||||||||||||||||||||||||||||||||||||||||||||||||||||
Sbjct  901   AAGCAACGCGAAGAACCTTACCAGGCCTTGACATCCAATGAACTTTCCAGAGATGGATTG  960

Query  969   GTGCCTTCGGGAACATTGAGACAGGTGCTGCATGGCTGTCGTCAGCTCGTGT  1020
             ||||||||||||||||||||||||||||||||||||||||||||||||||||
Sbjct  961   GTGCCTTCGGGAACATTGAGACAGGTGCTGCATGGCTGTCGTCAGCTCGTGT  1012
```

```

```


BLAST is a registered trademark of the National Library of Medicine

Support center
Mailing list


YouTube

- National Library Of Medicine
- National Institutes Of Health
- U.S. Department of Health & Human Services
- USA.gov

### NCBI


National Center for Biotechnology Information,
 U.S. National Library of Medicine

8600 Rockville Pike,
Bethesda
 MD,
20894
USA

Policies and Guidelines
|
Contact


PreferencesTurn off

External link. Please review our privacy policy.
