# Supplementary material for: Isothermal microcalorimetry for thermal viable count of microorganisms in pure cultures and stabilized formulations
Source: BMC Microbiol. 2019 Mar 21;19:65. doi: 10.1186/s12866-019-1432-8 (PMC6429831; doi:10.1186/s12866-019-1432-8)
Supplement: Supplementary file 3 — 16S rRNA gene sequences’ BLASTN hits in zipped HTML format. (ZIP 15810 kb) [file 12866_2019_1432_MOESM3_ESM.zip › Best blastn hits/NCBI Blast_38 pinkish lower band R -- 20..1068 of.html]

NCBI Blast:38 pinkish lower band R -- 20..1068 of


- NCBI Home
- Sign in to NCBI
- Skip to Main Content
- Skip to Navigation
- About NCBI Accesskeys

U.S. National Library of Medicine

NCBI
National Center for Biotechnology Information

- My NCBI
- Sign in to NCBI
- Register
- Sign Out

BLAST ® » blastn suite » RID-A4W50R0N014


- Home
- Recent Results
- Saved Strategies
- Help

BLAST Results


Edit and Resubmit
Save Search Strategies
[Sign in above to save your search strategy]

Formatting options 


Download


How to read this page
Blast report description
Questions/comments


|  |  |
| --- | --- |
| Formatting options | |
| Show | Alignment as  HTML Plain text   Old View Reset form to defaults [?]  These options control formatting of alignments in results pages. The default is HTML, but other formats (including plain text) are available. PSSM and PssmWithParameters are representations of Position Specific Scoring Matrices and are only available for PSI-BLAST. The Advanced view option allows the database descriptions to be sorted by various indices in a table. |
| Alignment View | Pairwise Pairwise with dots for identities Query-anchored with dots for identities Query-anchored with letters for identities Flat query-anchored with dots for identities Flat query-anchored with letters for identities [?]  Choose how to view alignments. The default "pairwise" view shows how each subject sequence aligns individually to the query sequence. The "query-anchored" view shows how all subject sequences align to the query sequence. For each view type, you can choose to show "identities" (matching residues) as letters or dots. more... |
| Display | Graphical Overview   Linkout   Sequence Retrieval  NCBI-gi   CDS feature [?]  - Graphical Overview: Graphical Overview: Show graph of similar sequence regions aligned to query.   more... - NCBI-gi: Show NCBI gi identifiers. - CDS feature: Show annotated coding region and translation.   more... |
| Masking | Character:   X for protein, n for nucleotide Lower Case  Color:  Black Grey Red [?]  - Masking Character: Display masked (filtered) sequence regions as lower-case or as specific letters (N for nucleotide, P for protein). - Masking Color: Display masked sequence regions in the given color. |
| Limit results | Descriptions:  10 50 100 Graphical overview:  0 10 50 100  Alignments:  0 10 50 100 Line length:  60 90 120 150 [?]  - Descriptions: Show short descriptions for up to the given number of sequences. - Alignments: Show alignments for up to the given number of sequences, in order of statistical significance. - Line lenghth: Number of letters to show on one line in an alignment. |
|  | Organism Type common name, binomial, taxid, or group name. Only 20 top taxa will be shown.     Exclude    [?]  Show only sequences from the given organism. |
|  | Entrez query:  [?]  Show only those sequences that match the given Entrez query. more... |
|  | Expect Min:  Expect Max:  [?]  Show only sequences with expect values in the given range. more... |
|  | Percent Identity Min:  Percent Identity Max:  [?]  Show only sequences with percent identity values in the given range. |
| Format for | PSI-BLAST with inclusion threshold:  [?]  - Format for PSI-BLAST: The Position-Specific Iterated BLAST (PSI-BLAST) program performs iterative searches with a protein query,   in which sequences found in one round of search are used to build a custom score model for the next round.   more... - Inclusion Threshold: This sets the statistical significance threshold for including a sequence in the model used   by PSI-BLAST to create the PSSM on the next iteration. |

|  |  |  |  |  |  |
| --- | --- | --- | --- | --- | --- |
| Download | | | | | |
| Alignment  Text XML ASN.1 JSON Seq-align Hit Table(text) Hit Table(csv) Multiple-file XML2 Single-file XML2 Multiple-file JSON Single-file JSON SAM | Search Strategies  ASN.1 | PSSM to restart search  PSSM | [?] |

The Download link provides BLAST output that may be used as input to another program.
This includes parseable formats such as the tabular report or XML as well as the Search Strategy files read by the BLAST+ applications.
More details on the parseable (XML, tabular, and ASN.1) reports can be found at
https://www.ncbi.nlm.nih.gov/books/NBK153387/  
  

The following formats are offered under the Alignment section:  
1). "Text". Non-HTML standard BLAST report.  
2). "XML". XML report based upon the DTD at https://www.ncbi.nlm.nih.gov/data\_specs/dtd/NCBI\_BlastOutput.dtd  
3). "ASN.1". Alignment written out in Abstract Syntax Notation 1.  
4). "JSON Seq-align". Alignment written out in JSON.  
4). "Hit Table(text)". The tabular report as text.  
5). "Hit Table(csv)". The tabular report ready for import into spread-sheet programs like Excel.  
6). "XML2". New XML format described at ftp://ftp.ncbi.nlm.nih.gov/blast/documents/NEWXML/xml2.pdf.  
7). "JSON". New JSON format described at ftp://ftp.ncbi.nlm.nih.gov/blast/documents/NEWXML/xml2.pdf.  
8). "SAM". Sequence Alignment Map format.

XML2 and JSON can be downloaded either as one file per query (multiple-file) or one file for all queries (single-file). These formats are listed as Multiple-file XML2 (and JSON) or Single-file XML (and JSON).

The following report is offered under the Search Strategy section:  
1). "ASN.1" Search Strategy. A record of the parameters, query, and database used in the search. This file can be used to start a stand-alone BLAST search, see
https://www.ncbi.nlm.nih.gov/books/NBK1763/#CmdLineAppsManual.I455\_BLAST\_search\_stra


# Job title: 38 pinkish lower band R -- 20..1068 of

Results for:

lcl|Query\_163895 38 pinkish lower band R -- 20..1068 of sequence(1049bp)
[?]

Your BLAST job specified more than one input sequence.
This box lets you choose which input sequence to show BLAST results for.

RID
:   A4W50R0N014 (Expires on 03-10 18:31 pm)

Query ID
:   lcl|Query\_163895
:   lcl|Query\_163895

Description
:   38 pinkish lower band R -- 20..1068 of sequence

Molecule type
:   nucleic acid

Query Length
:   1049

Database Name
:   nr

Description
:   Nucleotide collection (nt) See details

Program
:   BLASTN 2.8.0+ Citation

  

Reference 

Zheng Zhang, Scott Schwartz, Lukas Wagner, and Webb Miller (2000), "A greedy algorithm for aligning DNA sequences", J Comput Biol 2000; 7(1-2):203-14.

Reference - database indexing

Aleksandr Morgulis, George Coulouris, Yan Raytselis, Thomas L. Madden, Richa Agarwala, Alejandro A. Schäffer (2008), "Database Indexing for Production MegaBLAST Searches", Bioinformatics 24:1757-1764.

Other reports:
Search Summary

[Taxonomy reports]
[Distance tree of results]
[MSA viewer]

Search Parameters

| Search parameter name | Search parameter value |
| --- | --- |
| Program | blastn |
| Word size | 28 |
| Expect value | 10 |
| Hitlist size | 100 |
| Match/Mismatch scores | 1,-2 |
| Gapcosts | 0,2.5 |
| Low Complexity Filter | Yes |
| Filter string | L;m; |
| Genetic Code | 1 |

Database

| Database parameter name | Database parameter value |
| --- | --- |
| Posted date | Mar 7, 2018 1:58 PM |
| Number of letters | 174,044,644,244 |
| Number of sequences | 46,882,714 |
| Entrez query | Includes:  Excludes:  None |

Karlin-Altschul statistics

| Params | Ungapped | Gapped |
| --- | --- | --- |
| Lambda | 1.33271 | 1.28 |
| K | 0.620991 | 0.46 |
| H | 1.12409 | 0.85 |

Results Statistics

| Results Statistics parameter name | Results Statistics parameter value |
| --- | --- |
| Length adjustment | 35 |
| Effective length of query | 1014 |
| Effective length of database | 172403749254 |
| Effective search space | 174817401743556 |
| Effective search space used | 174817401743556 |


## Graphic Summary

### Distribution of the top 126 Blast Hits on 100 subject sequences [?]

The graphic is an overview of the database sequences aligned to the query sequence. These are represented horizontal bars colored coded by score and showing the extent
of the alignment on the query sequence. Separate aligned regions on the same database sequence are connected by a thin grey line.
Mousing over an alignment shows the database sequence title. Clicking an alignment displays a box with more details about the alignment and
link to the sequence alignment itself in the Alignments section of the report.

Mouse over to see the title, click to show alignments

Color key for alignment scores

<40

40-50

50-80

80-200

>=200

Query

1

200

400

600

800

1000

Pseudomonas brassicacearum isolate MA250 16S ribosomal ..

Score:1908 Evalue:0

Accession:DQ886486.1

Alignment

Uncultured bacterium clone Untreatedsoil-0day-94 16S ri..

Score:1903 Evalue:0

Accession:MF314815.2

Alignment

Uncultured bacterium clone Untreatedsoil-0day-1 16S rib..

Score:1903 Evalue:0

Accession:MF314725.2

Alignment

Uncultured bacterium clone SPN400-90day-85 16S ribosoma..

Score:1903 Evalue:0

Accession:MF314628.1

Alignment

Uncultured bacterium clone SPN400-90day-76 16S ribosoma..

Score:1903 Evalue:0

Accession:MF314621.1

Alignment

Uncultured bacterium clone SPN400-90day-50 16S ribosoma..

Score:1903 Evalue:0

Accession:MF314595.1

Alignment

Uncultured bacterium clone SPN400-90day-46 16S ribosoma..

Score:1903 Evalue:0

Accession:MF314592.1

Alignment

Uncultured bacterium clone SPN400-90day-43 16S ribosoma..

Score:1903 Evalue:0

Accession:MF314589.1

Alignment

Uncultured bacterium clone SPN400-90day-20 16S ribosoma..

Score:1903 Evalue:0

Accession:MF314569.1

Alignment

Uncultured bacterium clone SPN400-90day-6 16S ribosomal..

Score:1903 Evalue:0

Accession:MF314558.1

Alignment

Pseudomonas sp. HA-09 partial 16S rRNA gene, isolate HA..

Score:1903 Evalue:0

Accession:LT844660.1

Alignment

Pseudomonas brassicacearum strain YC-1 16S ribosomal RN..

Score:1903 Evalue:0

Accession:KY753310.1

Alignment

Pseudomonas brassicacearum strain FC-7 16S ribosomal RN..

Score:1903 Evalue:0

Accession:KY649379.1

Alignment

Pseudomonas fluorescens strain PFR1 16S ribosomal RNA g..

Score:1903 Evalue:0

Accession:MF000304.1

Alignment

Pseudomonas sp. strain 7.3 16S ribosomal RNA gene, part..

Score:1903 Evalue:0

Accession:KY542120.1

Alignment

Pseudomonas syringae strain yangyueP4 16S ribosomal RNA..

Score:1903 Evalue:0

Accession:KU977139.1

Alignment

Pseudomonas sp. RhizorgN 16S ribosomal RNA gene, partia..

Score:1903 Evalue:0

Accession:KT318824.1

Alignment

Pseudomonas sp. RhizorgB 16S ribosomal RNA gene, partia..

Score:1903 Evalue:0

Accession:KT318813.1

Alignment

Pseudomonas fluorescens strain FW300-N2E2 genome

Score:1903 Evalue:0

Accession:CP015225.1

Alignment

Pseudomonas sp. B055-40 16S ribosomal RNA gene, partial..

Score:1903 Evalue:0

Accession:KJ191010.1

Alignment

Pseudomonas sp. cpRA293 16S ribosomal RNA gene, partial..

Score:1903 Evalue:0

Accession:KJ510220.1

Alignment

Pseudomonas corrugata strain G1 16S ribosomal RNA gene,..

Score:1903 Evalue:0

Accession:KT957303.1

Alignment

Pseudomonas fluorescens strain FW300-N2C3, complete gen..

Score:1903 Evalue:0

Accession:CP012831.1

Alignment

Pseudomonas migulae strain DD290 16S ribosomal RNA gene..

Score:1903 Evalue:0

Accession:KR822273.1

Alignment

Pseudomonas fluorescens 16S ribosomal RNA gene, partial..

Score:1903 Evalue:0

Accession:KT321681.1

Alignment

Pseudomonas brassicacearum strain Kr21 16S ribosomal RN..

Score:1903 Evalue:0

Accession:KT215482.1

Alignment

Pseudomonas brassicacearum strain IHB B 13650 16S ribos..

Score:1903 Evalue:0

Accession:KP762561.1

Alignment

Pseudomonas brassicacearum subsp. neoaurantiaca strain ..

Score:1903 Evalue:0

Accession:KP762555.1

Alignment

Pseudomonas brassicacearum strain 13B-23 16S ribosomal ..

Score:1903 Evalue:0

Accession:KR083019.1

Alignment

Pseudomonas brassicacearum strain 10C-30 16S ribosomal ..

Score:1903 Evalue:0

Accession:KR061416.1

Alignment

Pseudomonas brassicacearum strain 1Cg-22 16S ribosomal ..

Score:1903 Evalue:0

Accession:KR061398.1

Alignment

Pseudomonas thivervalensis strain MAH1 16S ribosomal RN..

Score:1903 Evalue:0

Accession:KP742980.1

Alignment

Pseudomonas brassicacearum strain 11B23 16S ribosomal R..

Score:1903 Evalue:0

Accession:KR611048.1

Alignment

Pseudomonas brassicacearum strain 3B1 16S ribosomal RNA..

Score:1903 Evalue:0

Accession:KR611047.1

Alignment

Pseudomonas sp. 41(2015) 16S ribosomal RNA gene, partia..

Score:1903 Evalue:0

Accession:KR080568.1

Alignment

Pseudomonas sp. 15(2015) 16S ribosomal RNA gene, partia..

Score:1903 Evalue:0

Accession:KR080563.1

Alignment

Pseudomonas brassicacearum subsp. brassicacearum strain..

Score:1903 Evalue:0

Accession:KP730603.1

Alignment

Pseudomonas sp. SAM1 16S ribosomal RNA gene, partial se..

Score:1903 Evalue:0

Accession:KM269192.1

Alignment

Pseudomonas sp. TY1210 16S ribosomal RNA gene, partial ..

Score:1903 Evalue:0

Accession:KM030057.1

Alignment

Pseudomonas thivervalensis strain PE32 16S ribosomal RN..

Score:1903 Evalue:0

Accession:KJ420530.1

Alignment

Pseudomonas fluorescens strain JK15 16S ribosomal RNA g..

Score:1903 Evalue:0

Accession:KF148637.1

Alignment

Pseudomonas brassicacearum strain WK-444s 16S ribosomal..

Score:1903 Evalue:0

Accession:KF580861.1

Alignment

Pseudomonas brassicacearum strain IHB B 821 16S ribosom..

Score:1903 Evalue:0

Accession:KF475874.1

Alignment

Pseudomonas putida partial 16S rRNA gene, strain CFBP 4..

Score:1903 Evalue:0

Accession:HF545841.1

Alignment

Pseudomonas sp. S-3 16S ribosomal RNA gene, partial seq..

Score:1903 Evalue:0

Accession:KC207086.1

Alignment

Pseudomonas fluorescens strain IBFC2012-45 16S ribosoma..

Score:1903 Evalue:0

Accession:KC246049.1

Alignment

Pseudomonas sp. N-128 16S ribosomal RNA gene, partial s..

Score:1903 Evalue:0

Accession:JN216880.1

Alignment

Uncultured Pseudomonas sp. clone CGMCG 6071 16S ribosom..

Score:1903 Evalue:0

Accession:JX077087.1

Alignment

Endophytic bacterium 41P-2 16S ribosomal RNA gene, part..

Score:1903 Evalue:0

Accession:JF901350.1

Alignment

Bacterium NTL501 16S ribosomal RNA gene, partial sequen..

Score:1903 Evalue:0

Accession:JQ779065.1

Alignment

Bacterium NTL264 16S ribosomal RNA gene, partial sequen..

Score:1903 Evalue:0

Accession:JQ779056.1

Alignment

Bacterium NTL206 16S ribosomal RNA gene, partial sequen..

Score:1903 Evalue:0

Accession:JQ779041.1

Alignment

Pseudomonas sp. hswx161 16S ribosomal RNA gene, partial..

Score:1903 Evalue:0

Accession:JQ237663.1

Alignment

Pseudomonas thivervalensis 16S ribosomal RNA gene, part..

Score:1903 Evalue:0

Accession:JN628032.1

Alignment

Pseudomonas brassicacearum subsp. neoaurantiaca 16S rib..

Score:1903 Evalue:0

Accession:JN628030.1

Alignment

Pseudomonas brassicacearum strain ME BHU2 16S ribosomal..

Score:1903 Evalue:0

Accession:JN033549.1

Alignment

## Descriptions

, Reading indexes 1-5, displaying indexes 1-5


Load next setPrevious Match

Sequences producing significant alignments:

Show all columns  of the table presenting sequences producing significant alignments 

Select:AllNone
Selected:0

Alignments
Download

FASTA (complete sequence)

FASTA (aligned sequences)

GenBank (complete sequence)

Hit Table (text)

Hit Table (CSV)

Text

XML

ASN.1

Continue
Cancel

GenBank 
Graphics
Distance tree of results
Multiple alignment
Show/hide columns of the table presenting sequences producing significant alignments 

Available columns

Description  
Max Score  
Total Score  
Coverage  
E-value  
IdentN  
Accession  
Restore Defaults
Ok
Cancel

Sequences producing significant alignments:

| Select for downloading or viewing reports | Description | Max score | Total score | Query cover | E value | Ident | Accession |
| --- | --- | --- | --- | --- | --- | --- | --- |
| 1Select seq DQ886486.1 | Pseudomonas brassicacearum isolate MA250 16S ribosomal RNA gene, partial sequence | 1908 | 1908 | 98% | 0.0 | 100% | DQ886486.1 |
| 2Select seq MF314815.2 | Uncultured bacterium clone Untreatedsoil-0day-94 16S ribosomal RNA gene, partial sequence | 1903 | 1903 | 98% | 0.0 | 99% | MF314815.2 |
| 3Select seq MF314725.2 | Uncultured bacterium clone Untreatedsoil-0day-1 16S ribosomal RNA gene, partial sequence | 1903 | 1903 | 98% | 0.0 | 99% | MF314725.2 |
| 4Select seq MF314628.1 | Uncultured bacterium clone SPN400-90day-85 16S ribosomal RNA gene, partial sequence | 1903 | 1903 | 98% | 0.0 | 99% | MF314628.1 |
| 5Select seq MF314621.1 | Uncultured bacterium clone SPN400-90day-76 16S ribosomal RNA gene, partial sequence | 1903 | 1903 | 98% | 0.0 | 99% | MF314621.1 |
| 6Select seq MF314595.1 | Uncultured bacterium clone SPN400-90day-50 16S ribosomal RNA gene, partial sequence | 1903 | 1903 | 98% | 0.0 | 99% | MF314595.1 |
| 7Select seq MF314592.1 | Uncultured bacterium clone SPN400-90day-46 16S ribosomal RNA gene, partial sequence | 1903 | 1903 | 98% | 0.0 | 99% | MF314592.1 |
| 8Select seq MF314589.1 | Uncultured bacterium clone SPN400-90day-43 16S ribosomal RNA gene, partial sequence | 1903 | 1903 | 98% | 0.0 | 99% | MF314589.1 |
| 9Select seq MF314569.1 | Uncultured bacterium clone SPN400-90day-20 16S ribosomal RNA gene, partial sequence | 1903 | 1903 | 98% | 0.0 | 99% | MF314569.1 |
| 10Select seq MF314558.1 | Uncultured bacterium clone SPN400-90day-6 16S ribosomal RNA gene, partial sequence | 1903 | 1903 | 98% | 0.0 | 99% | MF314558.1 |
| 11Select seq LT844660.1 | Pseudomonas sp. HA-09 partial 16S rRNA gene, isolate HA-09 | 1903 | 1903 | 98% | 0.0 | 99% | LT844660.1 |
| 12Select seq KY753310.1 | Pseudomonas brassicacearum strain YC-1 16S ribosomal RNA gene, partial sequence | 1903 | 1903 | 98% | 0.0 | 99% | KY753310.1 |
| 13Select seq KY649379.1 | Pseudomonas brassicacearum strain FC-7 16S ribosomal RNA gene, partial sequence | 1903 | 1903 | 98% | 0.0 | 99% | KY649379.1 |
| 14Select seq MF000304.1 | Pseudomonas fluorescens strain PFR1 16S ribosomal RNA gene, partial sequence | 1903 | 1903 | 98% | 0.0 | 99% | MF000304.1 |
| 15Select seq KY542120.1 | Pseudomonas sp. strain 7.3 16S ribosomal RNA gene, partial sequence | 1903 | 1903 | 98% | 0.0 | 99% | KY542120.1 |
| 16Select seq KU977139.1 | Pseudomonas syringae strain yangyueP4 16S ribosomal RNA gene, partial sequence | 1903 | 1903 | 98% | 0.0 | 99% | KU977139.1 |
| 17Select seq KT318824.1 | Pseudomonas sp. RhizorgN 16S ribosomal RNA gene, partial sequence | 1903 | 1903 | 98% | 0.0 | 99% | KT318824.1 |
| 18Select seq KT318813.1 | Pseudomonas sp. RhizorgB 16S ribosomal RNA gene, partial sequence | 1903 | 1903 | 98% | 0.0 | 99% | KT318813.1 |
| 19Select seq CP015225.1 | Pseudomonas fluorescens strain FW300-N2E2 genome | 1903 | 9497 | 98% | 0.0 | 99% | CP015225.1 |
| 20Select seq KJ191010.1 | Pseudomonas sp. B055-40 16S ribosomal RNA gene, partial sequence | 1903 | 1903 | 98% | 0.0 | 99% | KJ191010.1 |
| 21Select seq KJ510220.1 | Pseudomonas sp. cpRA293 16S ribosomal RNA gene, partial sequence | 1903 | 1903 | 98% | 0.0 | 99% | KJ510220.1 |
| 22Select seq KT957303.1 | Pseudomonas corrugata strain G1 16S ribosomal RNA gene, partial sequence | 1903 | 1903 | 98% | 0.0 | 99% | KT957303.1 |
| 23Select seq CP012831.1 | Pseudomonas fluorescens strain FW300-N2C3, complete genome | 1903 | 9515 | 98% | 0.0 | 99% | CP012831.1 |
| 24Select seq KR822273.1 | Pseudomonas migulae strain DD290 16S ribosomal RNA gene, partial sequence | 1903 | 1903 | 98% | 0.0 | 99% | KR822273.1 |
| 25Select seq KT321681.1 | Pseudomonas fluorescens 16S ribosomal RNA gene, partial sequence | 1903 | 1903 | 98% | 0.0 | 99% | KT321681.1 |
| 26Select seq KT215482.1 | Pseudomonas brassicacearum strain Kr21 16S ribosomal RNA gene, partial sequence | 1903 | 1903 | 98% | 0.0 | 99% | KT215482.1 |
| 27Select seq KP762561.1 | Pseudomonas brassicacearum strain IHB B 13650 16S ribosomal RNA gene, partial sequence | 1903 | 1903 | 98% | 0.0 | 99% | KP762561.1 |
| 28Select seq KP762555.1 | Pseudomonas brassicacearum subsp. neoaurantiaca strain IHB B 13645 16S ribosomal RNA gene, partial sequence | 1903 | 1903 | 98% | 0.0 | 99% | KP762555.1 |
| 29Select seq KR083019.1 | Pseudomonas brassicacearum strain 13B-23 16S ribosomal RNA gene, partial sequence | 1903 | 1903 | 98% | 0.0 | 99% | KR083019.1 |
| 30Select seq KR061416.1 | Pseudomonas brassicacearum strain 10C-30 16S ribosomal RNA gene, partial sequence | 1903 | 1903 | 98% | 0.0 | 99% | KR061416.1 |
| 31Select seq KR061398.1 | Pseudomonas brassicacearum strain 1Cg-22 16S ribosomal RNA gene, partial sequence | 1903 | 1903 | 98% | 0.0 | 99% | KR061398.1 |
| 32Select seq KP742980.1 | Pseudomonas thivervalensis strain MAH1 16S ribosomal RNA gene, partial sequence | 1903 | 1903 | 98% | 0.0 | 99% | KP742980.1 |
| 33Select seq KR611048.1 | Pseudomonas brassicacearum strain 11B23 16S ribosomal RNA gene, partial sequence | 1903 | 1903 | 98% | 0.0 | 99% | KR611048.1 |
| 34Select seq KR611047.1 | Pseudomonas brassicacearum strain 3B1 16S ribosomal RNA gene, partial sequence | 1903 | 1903 | 98% | 0.0 | 99% | KR611047.1 |
| 35Select seq KR080568.1 | Pseudomonas sp. 41(2015) 16S ribosomal RNA gene, partial sequence | 1903 | 1903 | 98% | 0.0 | 99% | KR080568.1 |
| 36Select seq KR080563.1 | Pseudomonas sp. 15(2015) 16S ribosomal RNA gene, partial sequence | 1903 | 1903 | 98% | 0.0 | 99% | KR080563.1 |
| 37Select seq KP730603.1 | Pseudomonas brassicacearum subsp. brassicacearum strain BW0808 16S ribosomal RNA gene, partial sequence | 1903 | 1903 | 98% | 0.0 | 99% | KP730603.1 |
| 38Select seq KM269192.1 | Pseudomonas sp. SAM1 16S ribosomal RNA gene, partial sequence | 1903 | 1903 | 98% | 0.0 | 99% | KM269192.1 |
| 39Select seq KM030057.1 | Pseudomonas sp. TY1210 16S ribosomal RNA gene, partial sequence | 1903 | 1903 | 98% | 0.0 | 99% | KM030057.1 |
| 40Select seq KJ420530.1 | Pseudomonas thivervalensis strain PE32 16S ribosomal RNA gene, partial sequence | 1903 | 1903 | 98% | 0.0 | 99% | KJ420530.1 |
| 41Select seq KF148637.1 | Pseudomonas fluorescens strain JK15 16S ribosomal RNA gene, partial sequence | 1903 | 1903 | 98% | 0.0 | 99% | KF148637.1 |
| 42Select seq KF580861.1 | Pseudomonas brassicacearum strain WK-444s 16S ribosomal RNA gene, partial sequence | 1903 | 1903 | 98% | 0.0 | 99% | KF580861.1 |
| 43Select seq KF475874.1 | Pseudomonas brassicacearum strain IHB B 821 16S ribosomal RNA gene, partial sequence | 1903 | 1903 | 98% | 0.0 | 99% | KF475874.1 |
| 44Select seq HF545841.1 | Pseudomonas putida partial 16S rRNA gene, strain CFBP 4629 | 1903 | 1903 | 98% | 0.0 | 99% | HF545841.1 |
| 45Select seq KC207086.1 | Pseudomonas sp. S-3 16S ribosomal RNA gene, partial sequence | 1903 | 1903 | 98% | 0.0 | 99% | KC207086.1 |
| 46Select seq KC246049.1 | Pseudomonas fluorescens strain IBFC2012-45 16S ribosomal RNA gene, partial sequence | 1903 | 1903 | 98% | 0.0 | 99% | KC246049.1 |
| 47Select seq JN216880.1 | Pseudomonas sp. N-128 16S ribosomal RNA gene, partial sequence | 1903 | 1903 | 98% | 0.0 | 99% | JN216880.1 |
| 48Select seq JX077087.1 | Uncultured Pseudomonas sp. clone CGMCG 6071 16S ribosomal RNA gene, partial sequence | 1903 | 1903 | 98% | 0.0 | 99% | JX077087.1 |
| 49Select seq JF901350.1 | Endophytic bacterium 41P-2 16S ribosomal RNA gene, partial sequence | 1903 | 1903 | 98% | 0.0 | 99% | JF901350.1 |
| 50Select seq JQ779065.1 | Bacterium NTL501 16S ribosomal RNA gene, partial sequence | 1903 | 1903 | 98% | 0.0 | 99% | JQ779065.1 |
| 51Select seq JQ779056.1 | Bacterium NTL264 16S ribosomal RNA gene, partial sequence | 1903 | 1903 | 98% | 0.0 | 99% | JQ779056.1 |
| 52Select seq JQ779041.1 | Bacterium NTL206 16S ribosomal RNA gene, partial sequence | 1903 | 1903 | 98% | 0.0 | 99% | JQ779041.1 |
| 53Select seq JQ237663.1 | Pseudomonas sp. hswx161 16S ribosomal RNA gene, partial sequence | 1903 | 1903 | 98% | 0.0 | 99% | JQ237663.1 |
| 54Select seq JN628032.1 | Pseudomonas thivervalensis 16S ribosomal RNA gene, partial sequence | 1903 | 1903 | 98% | 0.0 | 99% | JN628032.1 |
| 55Select seq JN628030.1 | Pseudomonas brassicacearum subsp. neoaurantiaca 16S ribosomal RNA gene, partial sequence | 1903 | 1903 | 98% | 0.0 | 99% | JN628030.1 |
| 56Select seq JN033549.1 | Pseudomonas brassicacearum strain ME BHU2 16S ribosomal RNA gene, partial sequence | 1903 | 1903 | 98% | 0.0 | 99% | JN033549.1 |
| 57Select seq HQ143608.1 | Pseudomonas sp. strain MTQ15 16S ribosomal RNA gene, partial sequence | 1903 | 1903 | 98% | 0.0 | 99% | HQ143608.1 |
| 58Select seq HM579795.1 | Pseudomonas sp. HY4(2010) 16S ribosomal RNA gene, partial sequence | 1903 | 1903 | 98% | 0.0 | 99% | HM579795.1 |
| 59Select seq GU201849.1 | Pseudomonas brassicacearum strain Zy-2-1 16S ribosomal RNA gene, partial sequence | 1903 | 1903 | 98% | 0.0 | 99% | GU201849.1 |
| 60Select seq EF540490.1 | Pseudomonas sp. 4\_C7/16\_5 16S ribosomal RNA gene, partial sequence | 1903 | 1903 | 98% | 0.0 | 99% | EF540490.1 |
| 61Select seq DQ453833.1 | Pseudomonas sp. P97.26 16S ribosomal RNA gene, partial sequence | 1903 | 1903 | 98% | 0.0 | 99% | DQ453833.1 |
| 62Select seq DQ431467.1 | Pseudomonas fluorescens strain XG32 16S ribosomal RNA gene, partial sequence | 1903 | 1903 | 98% | 0.0 | 99% | DQ431467.1 |
| 63Select seq KF460526.1 | Pseudomonas fluorescens strain ALEB 7B 16S ribosomal RNA gene, partial sequence | 1901 | 1901 | 98% | 0.0 | 99% | KF460526.1 |
| 64Select seq DQ898298.1 | Pseudomonas sp. MTR-61 16S ribosomal RNA gene, partial sequence | 1901 | 1901 | 98% | 0.0 | 99% | DQ898298.1 |
| 65Select seq LT718479.1 | Uncultured Pseudomonas sp. partial 16S rRNA gene, isolate VF5 | 1899 | 1899 | 98% | 0.0 | 99% | LT718479.1 |
| 66Select seq LT629931.1 | Pseudomonas sp. 1A partial 16S rRNA gene, strain 1A | 1899 | 1899 | 98% | 0.0 | 99% | LT629931.1 |
| 67Select seq KC139435.1 | Pseudomonas brassicacearum subsp. neoaurantiaca strain h-16 16S ribosomal RNA gene, partial sequence | 1899 | 1899 | 98% | 0.0 | 99% | KC139435.1 |
| 68Select seq AB621593.1 | Pseudomonas fluorescens gene for 16S rRNA, partial sequence, strain: MPF29 | 1899 | 1899 | 98% | 0.0 | 99% | AB621593.1 |
| 69Select seq AM229082.1 | Pseudomonas fluorescens 16S rRNA gene, strain C7R12 | 1899 | 1899 | 98% | 0.0 | 99% | AM229082.1 |
| 70Select seq MF314711.2 | Uncultured bacterium clone SPN2000-90day-84 16S ribosomal RNA gene, partial sequence | 1897 | 1897 | 98% | 0.0 | 99% | MF314711.2 |
| 71Select seq CP025542.1 | Pseudomonas fluorescens strain 2P24 chromosome, complete genome | 1897 | 9477 | 98% | 0.0 | 99% | CP025542.1 |
| 72Select seq MG461480.1 | Pseudomonas fluorescens strain LBUM770 16S ribosomal RNA gene, partial sequence | 1897 | 1897 | 98% | 0.0 | 99% | MG461480.1 |
| 73Select seq MG461459.1 | Pseudomonas brassicacearum strain LBUM300 16S ribosomal RNA gene, partial sequence | 1897 | 1897 | 98% | 0.0 | 99% | MG461459.1 |
| 74Select seq MG266327.1 | Pseudomonas sp. strain KF-8 16S ribosomal RNA gene, partial sequence | 1897 | 1897 | 98% | 0.0 | 99% | MG266327.1 |
| 75Select seq MF062638.1 | Pseudomonas putida strain 42R-P6 16S ribosomal RNA gene, partial sequence | 1897 | 1897 | 98% | 0.0 | 99% | MF062638.1 |
| 76Select seq MF314613.1 | Uncultured bacterium clone SPN400-90day-68 16S ribosomal RNA gene, partial sequence | 1897 | 1897 | 98% | 0.0 | 99% | MF314613.1 |
| 77Select seq MF314601.1 | Uncultured bacterium clone SPN400-90day-56 16S ribosomal RNA gene, partial sequence | 1897 | 1897 | 98% | 0.0 | 99% | MF314601.1 |
| 78Select seq MF314557.1 | Uncultured bacterium clone SPN400-90day-5 16S ribosomal RNA gene, partial sequence | 1897 | 1897 | 98% | 0.0 | 99% | MF314557.1 |
| 79Select seq MF314553.1 | Uncultured bacterium clone SPN400-90day-1 16S ribosomal RNA gene, partial sequence | 1897 | 1897 | 98% | 0.0 | 99% | MF314553.1 |
| 80Select seq MF314478.1 | Uncultured bacterium clone SPN0-90day-18 16S ribosomal RNA gene, partial sequence | 1897 | 1897 | 98% | 0.0 | 99% | MF314478.1 |
| 81Select seq MF314475.1 | Uncultured bacterium clone SPN0-90day-15 16S ribosomal RNA gene, partial sequence | 1897 | 1897 | 98% | 0.0 | 99% | MF314475.1 |
| 82Select seq MF624719.1 | Pseudomonas brassicacearum strain Pv10 16S ribosomal RNA gene, partial sequence | 1897 | 1897 | 98% | 0.0 | 99% | MF624719.1 |
| 83Select seq MF498772.1 | Pseudomonas jessenii strain AP3\_16 16S ribosomal RNA gene, partial sequence | 1897 | 1897 | 98% | 0.0 | 99% | MF498772.1 |
| 84Select seq KX859150.1 | Pseudomonas sp. strain A9 16S ribosomal RNA gene, partial sequence | 1897 | 1897 | 98% | 0.0 | 99% | KX859150.1 |
| 85Select seq KY347907.1 | Pseudomonas sp. strain YP2 16S ribosomal RNA gene, partial sequence | 1897 | 1897 | 98% | 0.0 | 99% | KY347907.1 |
| 86Select seq MF445217.1 | Pseudomonas sp. strain nenu\_DS-R08 16S ribosomal RNA gene, partial sequence | 1897 | 1897 | 98% | 0.0 | 99% | MF445217.1 |
| 87Select seq KY111476.1 | Pseudomonas sp. strain B10 16S ribosomal RNA gene, partial sequence | 1897 | 1897 | 98% | 0.0 | 99% | KY111476.1 |
| 88Select seq KY122024.1 | Pseudomonas sp. strain SX2 16S ribosomal RNA gene, partial sequence | 1897 | 1897 | 98% | 0.0 | 99% | KY122024.1 |
| 89Select seq KY962733.1 | Pseudomonas sp. strain Mong-1 16S ribosomal RNA gene, partial sequence | 1897 | 1897 | 98% | 0.0 | 99% | KY962733.1 |
| 90Select seq KY907019.1 | Pseudomonas sp. strain MSM-10-1 16S ribosomal RNA gene, partial sequence | 1897 | 1897 | 98% | 0.0 | 99% | KY907019.1 |
| 91Select seq KY612271.1 | Pseudomonas brassicacearum strain SKUAST-K21 16S ribosomal RNA gene, partial sequence | 1897 | 1897 | 98% | 0.0 | 99% | KY612271.1 |
| 92Select seq LT707062.1 | Pseudomonas sp. A214 genome assembly, chromosome: I | 1897 | 9471 | 98% | 0.0 | 99% | LT707062.1 |
| 93Select seq KU878092.1 | Pseudomonas brassicacearum strain B04 16S ribosomal RNA gene, partial sequence | 1897 | 1897 | 98% | 0.0 | 99% | KU878092.1 |
| 94Select seq LT629786.1 | Pseudomonas synxantha strain LMG 2190 genome assembly, chromosome: I | 1897 | 12635 | 98% | 0.0 | 99% | LT629786.1 |
| 95Select seq LT629713.1 | Pseudomonas brassicacearum strain BS3663 genome assembly, chromosome: I | 1897 | 9488 | 98% | 0.0 | 99% | LT629713.1 |
| 96Select seq KX984045.1 | Pseudomonas brassicacearum strain TSDS1 16S ribosomal RNA gene, partial sequence | 1897 | 1897 | 98% | 0.0 | 99% | KX984045.1 |
| 97Select seq KX984039.1 | Pseudomonas brassicacearum strain PNS2 16S ribosomal RNA gene, partial sequence | 1897 | 1897 | 98% | 0.0 | 99% | KX984039.1 |
| 98Select seq KX901786.1 | Pseudomonas fluorescens strain BSW-2 16S ribosomal RNA gene, partial sequence | 1897 | 1897 | 98% | 0.0 | 99% | KX901786.1 |
| 99Select seq KX901785.1 | Pseudomonas fluorescens strain BSW-1 16S ribosomal RNA gene, partial sequence | 1897 | 1897 | 98% | 0.0 | 99% | KX901785.1 |
| 100Select seq LN995697.1 | Pseudomonas jessenii partial 16S rRNA gene, strain R-52636 | 1897 | 1897 | 98% | 0.0 | 99% | LN995697.1 |


## Alignments

Loading alignment... for sequences gi|115315703,gi|1321449613,gi|1321449529,gi|1229861468,gi|1229861461 Reading indexes 1-5

Download

FASTA (complete sequence)

FASTA (aligned sequences)

GenBank (complete sequence)

Continue
Cancel

GenBankGraphics

Next
Previous
Descriptions

Pseudomonas brassicacearum isolate MA250 16S ribosomal RNA gene, partial sequence

Sequence ID: DQ886486.1Length: 1515Number of Matches: 1

Related Information

Range 1: 403 to 1435GenBankGraphics

Next Match
Previous Match
First Match

Alignment statistics for match #1

| Score | Expect | Identities | Gaps | Strand | Frame |
| --- | --- | --- | --- | --- | --- |
| 1908 bits(1033) | 0.0() | 1033/1033(100%) | 0/1033(0%) | Plus/Minus |  |

Features:

```
Query  10    CGAAGGTTAGACTAGCTACTTCTGGTGCAACCCACTCCCATGGTGTGACGGGCGGTGTGT  69
             ||||||||||||||||||||||||||||||||||||||||||||||||||||||||||||
Sbjct  1435  CGAAGGTTAGACTAGCTACTTCTGGTGCAACCCACTCCCATGGTGTGACGGGCGGTGTGT  1376

Query  70    ACAAGGCCCGGGAACGTATTCACCGCGACATTCTGATTCGCGATTACTAGCGATTCCGAC  129
             ||||||||||||||||||||||||||||||||||||||||||||||||||||||||||||
Sbjct  1375  ACAAGGCCCGGGAACGTATTCACCGCGACATTCTGATTCGCGATTACTAGCGATTCCGAC  1316

Query  130   TTCACGCAGTCGAGTTGCAGACTGCGATCCGGACTACGATCGGTTTTGTGGGATTAGCTC  189
             ||||||||||||||||||||||||||||||||||||||||||||||||||||||||||||
Sbjct  1315  TTCACGCAGTCGAGTTGCAGACTGCGATCCGGACTACGATCGGTTTTGTGGGATTAGCTC  1256

Query  190   CACCTCGCGGCTTGGCAACCCTCTGTACCGACCATTGTAGCACGTGTGTAGCCCAGGCCG  249
             ||||||||||||||||||||||||||||||||||||||||||||||||||||||||||||
Sbjct  1255  CACCTCGCGGCTTGGCAACCCTCTGTACCGACCATTGTAGCACGTGTGTAGCCCAGGCCG  1196

Query  250   TAAGGGCCATGATGACTTGACGTCATCCCCACCTTCCTCCGGTTTGTCACCGGCAGTCTC  309
             ||||||||||||||||||||||||||||||||||||||||||||||||||||||||||||
Sbjct  1195  TAAGGGCCATGATGACTTGACGTCATCCCCACCTTCCTCCGGTTTGTCACCGGCAGTCTC  1136

Query  310   CTTAGAGTGCCCACCATGACGTGCTGGTAACTAAGGACAAGGGTTGCGCTCGTTACGGGA  369
             ||||||||||||||||||||||||||||||||||||||||||||||||||||||||||||
Sbjct  1135  CTTAGAGTGCCCACCATGACGTGCTGGTAACTAAGGACAAGGGTTGCGCTCGTTACGGGA  1076

Query  370   CTTAACCCAACATCTCACGACACGAGCTGACGACAGCCATGCAGCACCTGTCTCAATGTT  429
             ||||||||||||||||||||||||||||||||||||||||||||||||||||||||||||
Sbjct  1075  CTTAACCCAACATCTCACGACACGAGCTGACGACAGCCATGCAGCACCTGTCTCAATGTT  1016

Query  430   CCCGAAGGCACCAATCCATCTCTGGAAAGTTCATTGGATGTCAAGGCCTGGTAAGGTTCT  489
             ||||||||||||||||||||||||||||||||||||||||||||||||||||||||||||
Sbjct  1015  CCCGAAGGCACCAATCCATCTCTGGAAAGTTCATTGGATGTCAAGGCCTGGTAAGGTTCT  956

Query  490   TCGCGTTGCTTCGAATTAAACCACATGCTCCACCGCTTGTGCGGGCCCCCGTCAATTCAT  549
             ||||||||||||||||||||||||||||||||||||||||||||||||||||||||||||
Sbjct  955   TCGCGTTGCTTCGAATTAAACCACATGCTCCACCGCTTGTGCGGGCCCCCGTCAATTCAT  896

Query  550   TTGAGTTTTAACCTTGCGGCCGTACTCCCCAGGCGGTCAACTTAATGCGTTAGCTGCGCC  609
             ||||||||||||||||||||||||||||||||||||||||||||||||||||||||||||
Sbjct  895   TTGAGTTTTAACCTTGCGGCCGTACTCCCCAGGCGGTCAACTTAATGCGTTAGCTGCGCC  836

Query  610   ACTAAGAGCTCAAGGCTCCCAACGGCTAGTTGACATCGTTTACGGCGTGGACTACCAGGG  669
             ||||||||||||||||||||||||||||||||||||||||||||||||||||||||||||
Sbjct  835   ACTAAGAGCTCAAGGCTCCCAACGGCTAGTTGACATCGTTTACGGCGTGGACTACCAGGG  776

Query  670   TATCTAATCCTGTTTGCTCCCCACGCTTTCGCACCTCAGTGTCAGTATCAGTCCAGGTGG  729
             ||||||||||||||||||||||||||||||||||||||||||||||||||||||||||||
Sbjct  775   TATCTAATCCTGTTTGCTCCCCACGCTTTCGCACCTCAGTGTCAGTATCAGTCCAGGTGG  716

Query  730   TCGCCTTCGCCACTGGTGTTCCTTCCTATATCTACGCATTTCACCGCTACACAGGAAATT  789
             ||||||||||||||||||||||||||||||||||||||||||||||||||||||||||||
Sbjct  715   TCGCCTTCGCCACTGGTGTTCCTTCCTATATCTACGCATTTCACCGCTACACAGGAAATT  656

Query  790   CCACCACCCTCTACCATACTCTAGCTCGACAGTTTTGAATGCAGTTCCCAGGTTGAGCCC  849
             ||||||||||||||||||||||||||||||||||||||||||||||||||||||||||||
Sbjct  655   CCACCACCCTCTACCATACTCTAGCTCGACAGTTTTGAATGCAGTTCCCAGGTTGAGCCC  596

Query  850   GGGGCTTTCACATCCAACTTAACGAACCACCTACGCGCGCTTTACGCCCAGTAATTCCGA  909
             ||||||||||||||||||||||||||||||||||||||||||||||||||||||||||||
Sbjct  595   GGGGCTTTCACATCCAACTTAACGAACCACCTACGCGCGCTTTACGCCCAGTAATTCCGA  536

Query  910   TTAACGCTTGCACCCTCTGTATTACCGCGGCTGCTGGCACAGAGTTAGCCGGTGCTTATT  969
             ||||||||||||||||||||||||||||||||||||||||||||||||||||||||||||
Sbjct  535   TTAACGCTTGCACCCTCTGTATTACCGCGGCTGCTGGCACAGAGTTAGCCGGTGCTTATT  476

Query  970   CTGTCGGTAACGTCAAAACACTAACGTATTAGGTTAATGCCCTTCCTCCCAACTTAAAGT  1029
             ||||||||||||||||||||||||||||||||||||||||||||||||||||||||||||
Sbjct  475   CTGTCGGTAACGTCAAAACACTAACGTATTAGGTTAATGCCCTTCCTCCCAACTTAAAGT  416

Query  1030  GCTTTACAATCCG  1042
             |||||||||||||
Sbjct  415   GCTTTACAATCCG  403
```

Download

FASTA (complete sequence)

FASTA (aligned sequences)

GenBank (complete sequence)

Continue
Cancel

GenBankGraphics

Next
Previous
Descriptions

Uncultured bacterium clone Untreatedsoil-0day-94 16S ribosomal RNA gene, partial sequence

Sequence ID: MF314815.2Length: 1515Number of Matches: 1

Related Information

Range 1: 410 to 1442GenBankGraphics

Next Match
Previous Match
First Match

Alignment statistics for match #1

| Score | Expect | Identities | Gaps | Strand | Frame |
| --- | --- | --- | --- | --- | --- |
| 1903 bits(1030) | 0.0() | 1032/1033(99%) | 0/1033(0%) | Plus/Minus |  |

Features:

```
Query  10    CGAAGGTTAGACTAGCTACTTCTGGTGCAACCCACTCCCATGGTGTGACGGGCGGTGTGT  69
             ||||||||||||||||||||||||||||||||||||||||||||||||||||||||||||
Sbjct  1442  CGAAGGTTAGACTAGCTACTTCTGGTGCAACCCACTCCCATGGTGTGACGGGCGGTGTGT  1383

Query  70    ACAAGGCCCGGGAACGTATTCACCGCGACATTCTGATTCGCGATTACTAGCGATTCCGAC  129
             ||||||||||||||||||||||||||||||||||||||||||||||||||||||||||||
Sbjct  1382  ACAAGGCCCGGGAACGTATTCACCGCGACATTCTGATTCGCGATTACTAGCGATTCCGAC  1323

Query  130   TTCACGCAGTCGAGTTGCAGACTGCGATCCGGACTACGATCGGTTTTGTGGGATTAGCTC  189
             ||||||||||||||||||||||||||||||||||||||||||||||||||||||||||||
Sbjct  1322  TTCACGCAGTCGAGTTGCAGACTGCGATCCGGACTACGATCGGTTTTGTGGGATTAGCTC  1263

Query  190   CACCTCGCGGCTTGGCAACCCTCTGTACCGACCATTGTAGCACGTGTGTAGCCCAGGCCG  249
             ||||||||||||||||||||||||||||||||||||||||||||||||||||||||||||
Sbjct  1262  CACCTCGCGGCTTGGCAACCCTCTGTACCGACCATTGTAGCACGTGTGTAGCCCAGGCCG  1203

Query  250   TAAGGGCCATGATGACTTGACGTCATCCCCACCTTCCTCCGGTTTGTCACCGGCAGTCTC  309
             ||||||||||||||||||||||||||||||||||||||||||||||||||||||||||||
Sbjct  1202  TAAGGGCCATGATGACTTGACGTCATCCCCACCTTCCTCCGGTTTGTCACCGGCAGTCTC  1143

Query  310   CTTAGAGTGCCCACCATGACGTGCTGGTAACTAAGGACAAGGGTTGCGCTCGTTACGGGA  369
             ||||||||||||||||||||||||||||||||||||||||||||||||||||||||||||
Sbjct  1142  CTTAGAGTGCCCACCATGACGTGCTGGTAACTAAGGACAAGGGTTGCGCTCGTTACGGGA  1083

Query  370   CTTAACCCAACATCTCACGACACGAGCTGACGACAGCCATGCAGCACCTGTCTCAATGTT  429
             ||||||||||||||||||||||||||||||||||||||||||||||||||||||||||||
Sbjct  1082  CTTAACCCAACATCTCACGACACGAGCTGACGACAGCCATGCAGCACCTGTCTCAATGTT  1023

Query  430   CCCGAAGGCACCAATCCATCTCTGGAAAGTTCATTGGATGTCAAGGCCTGGTAAGGTTCT  489
             ||||||||||||||||||||||||||||||||||||||||||||||||||||||||||||
Sbjct  1022  CCCGAAGGCACCAATCCATCTCTGGAAAGTTCATTGGATGTCAAGGCCTGGTAAGGTTCT  963

Query  490   TCGCGTTGCTTCGAATTAAACCACATGCTCCACCGCTTGTGCGGGCCCCCGTCAATTCAT  549
             ||||||||||||||||||||||||||||||||||||||||||||||||||||||||||||
Sbjct  962   TCGCGTTGCTTCGAATTAAACCACATGCTCCACCGCTTGTGCGGGCCCCCGTCAATTCAT  903

Query  550   TTGAGTTTTAACCTTGCGGCCGTACTCCCCAGGCGGTCAACTTAATGCGTTAGCTGCGCC  609
             ||||||||||||||||||||||||||||||||||||||||||||||||||||||||||||
Sbjct  902   TTGAGTTTTAACCTTGCGGCCGTACTCCCCAGGCGGTCAACTTAATGCGTTAGCTGCGCC  843

Query  610   ACTAAGAGCTCAAGGCTCCCAACGGCTAGTTGACATCGTTTACGGCGTGGACTACCAGGG  669
             ||||||||||||||||||||||||||||||||||||||||||||||||||||||||||||
Sbjct  842   ACTAAGAGCTCAAGGCTCCCAACGGCTAGTTGACATCGTTTACGGCGTGGACTACCAGGG  783

Query  670   TATCTAATCCTGTTTGCTCCCCACGCTTTCGCACCTCAGTGTCAGTATCAGTCCAGGTGG  729
             ||||||||||||||||||||||||||||||||||||||||||||||||||||||||||||
Sbjct  782   TATCTAATCCTGTTTGCTCCCCACGCTTTCGCACCTCAGTGTCAGTATCAGTCCAGGTGG  723

Query  730   TCGCCTTCGCCACTGGTGTTCCTTCCTATATCTACGCATTTCACCGCTACACAGGAAATT  789
             ||||||||||||||||||||||||||||||||||||||||||||||||||||||||||||
Sbjct  722   TCGCCTTCGCCACTGGTGTTCCTTCCTATATCTACGCATTTCACCGCTACACAGGAAATT  663

Query  790   CCACCACCCTCTACCATACTCTAGCTCGACAGTTTTGAATGCAGTTCCCAGGTTGAGCCC  849
             ||||||||||||||||||||||||||||||||||||||||||||||||||||||||||||
Sbjct  662   CCACCACCCTCTACCATACTCTAGCTCGACAGTTTTGAATGCAGTTCCCAGGTTGAGCCC  603

Query  850   GGGGCTTTCACATCCAACTTAACGAACCACCTACGCGCGCTTTACGCCCAGTAATTCCGA  909
             |||| |||||||||||||||||||||||||||||||||||||||||||||||||||||||
Sbjct  602   GGGGATTTCACATCCAACTTAACGAACCACCTACGCGCGCTTTACGCCCAGTAATTCCGA  543

Query  910   TTAACGCTTGCACCCTCTGTATTACCGCGGCTGCTGGCACAGAGTTAGCCGGTGCTTATT  969
             ||||||||||||||||||||||||||||||||||||||||||||||||||||||||||||
Sbjct  542   TTAACGCTTGCACCCTCTGTATTACCGCGGCTGCTGGCACAGAGTTAGCCGGTGCTTATT  483

Query  970   CTGTCGGTAACGTCAAAACACTAACGTATTAGGTTAATGCCCTTCCTCCCAACTTAAAGT  1029
             ||||||||||||||||||||||||||||||||||||||||||||||||||||||||||||
Sbjct  482   CTGTCGGTAACGTCAAAACACTAACGTATTAGGTTAATGCCCTTCCTCCCAACTTAAAGT  423

Query  1030  GCTTTACAATCCG  1042
             |||||||||||||
Sbjct  422   GCTTTACAATCCG  410
```

Download

FASTA (complete sequence)

FASTA (aligned sequences)

GenBank (complete sequence)

Continue
Cancel

GenBankGraphics

Next
Previous
Descriptions

Uncultured bacterium clone Untreatedsoil-0day-1 16S ribosomal RNA gene, partial sequence

Sequence ID: MF314725.2Length: 1502Number of Matches: 1

Related Information

Range 1: 410 to 1442GenBankGraphics

Next Match
Previous Match
First Match

Alignment statistics for match #1

| Score | Expect | Identities | Gaps | Strand | Frame |
| --- | --- | --- | --- | --- | --- |
| 1903 bits(1030) | 0.0() | 1032/1033(99%) | 0/1033(0%) | Plus/Minus |  |

Features:

```
Query  10    CGAAGGTTAGACTAGCTACTTCTGGTGCAACCCACTCCCATGGTGTGACGGGCGGTGTGT  69
             ||||||||||||||||||||||||||||||||||||||||||||||||||||||||||||
Sbjct  1442  CGAAGGTTAGACTAGCTACTTCTGGTGCAACCCACTCCCATGGTGTGACGGGCGGTGTGT  1383

Query  70    ACAAGGCCCGGGAACGTATTCACCGCGACATTCTGATTCGCGATTACTAGCGATTCCGAC  129
             ||||||||||||||||||||||||||||||||||||||||||||||||||||||||||||
Sbjct  1382  ACAAGGCCCGGGAACGTATTCACCGCGACATTCTGATTCGCGATTACTAGCGATTCCGAC  1323

Query  130   TTCACGCAGTCGAGTTGCAGACTGCGATCCGGACTACGATCGGTTTTGTGGGATTAGCTC  189
             ||||||||||||||||||||||||||||||||||||||||||||||||||||||||||||
Sbjct  1322  TTCACGCAGTCGAGTTGCAGACTGCGATCCGGACTACGATCGGTTTTGTGGGATTAGCTC  1263

Query  190   CACCTCGCGGCTTGGCAACCCTCTGTACCGACCATTGTAGCACGTGTGTAGCCCAGGCCG  249
             ||||||||||||||||||||||||||||||||||||||||||||||||||||||||||||
Sbjct  1262  CACCTCGCGGCTTGGCAACCCTCTGTACCGACCATTGTAGCACGTGTGTAGCCCAGGCCG  1203

Query  250   TAAGGGCCATGATGACTTGACGTCATCCCCACCTTCCTCCGGTTTGTCACCGGCAGTCTC  309
             ||||||||||||||||||||||||||||||||||||||||||||||||||||||||||||
Sbjct  1202  TAAGGGCCATGATGACTTGACGTCATCCCCACCTTCCTCCGGTTTGTCACCGGCAGTCTC  1143

Query  310   CTTAGAGTGCCCACCATGACGTGCTGGTAACTAAGGACAAGGGTTGCGCTCGTTACGGGA  369
             ||||||||||||||||||||||||||||||||||||||||||||||||||||||||||||
Sbjct  1142  CTTAGAGTGCCCACCATGACGTGCTGGTAACTAAGGACAAGGGTTGCGCTCGTTACGGGA  1083

Query  370   CTTAACCCAACATCTCACGACACGAGCTGACGACAGCCATGCAGCACCTGTCTCAATGTT  429
             ||||||||||||||||||||||||||||||||||||||||||||||||||||||||||||
Sbjct  1082  CTTAACCCAACATCTCACGACACGAGCTGACGACAGCCATGCAGCACCTGTCTCAATGTT  1023

Query  430   CCCGAAGGCACCAATCCATCTCTGGAAAGTTCATTGGATGTCAAGGCCTGGTAAGGTTCT  489
             ||||||||||||||||||||||||||||||||||||||||||||||||||||||||||||
Sbjct  1022  CCCGAAGGCACCAATCCATCTCTGGAAAGTTCATTGGATGTCAAGGCCTGGTAAGGTTCT  963

Query  490   TCGCGTTGCTTCGAATTAAACCACATGCTCCACCGCTTGTGCGGGCCCCCGTCAATTCAT  549
             ||||||||||||||||||||||||||||||||||||||||||||||||||||||||||||
Sbjct  962   TCGCGTTGCTTCGAATTAAACCACATGCTCCACCGCTTGTGCGGGCCCCCGTCAATTCAT  903

Query  550   TTGAGTTTTAACCTTGCGGCCGTACTCCCCAGGCGGTCAACTTAATGCGTTAGCTGCGCC  609
             ||||||||||||||||||||||||||||||||||||||||||||||||||||||||||||
Sbjct  902   TTGAGTTTTAACCTTGCGGCCGTACTCCCCAGGCGGTCAACTTAATGCGTTAGCTGCGCC  843

Query  610   ACTAAGAGCTCAAGGCTCCCAACGGCTAGTTGACATCGTTTACGGCGTGGACTACCAGGG  669
             ||||||||||||||||||||||||||||||||||||||||||||||||||||||||||||
Sbjct  842   ACTAAGAGCTCAAGGCTCCCAACGGCTAGTTGACATCGTTTACGGCGTGGACTACCAGGG  783

Query  670   TATCTAATCCTGTTTGCTCCCCACGCTTTCGCACCTCAGTGTCAGTATCAGTCCAGGTGG  729
             ||||||||||||||||||||||||||||||||||||||||||||||||||||||||||||
Sbjct  782   TATCTAATCCTGTTTGCTCCCCACGCTTTCGCACCTCAGTGTCAGTATCAGTCCAGGTGG  723

Query  730   TCGCCTTCGCCACTGGTGTTCCTTCCTATATCTACGCATTTCACCGCTACACAGGAAATT  789
             ||||||||||||||||||||||||||||||||||||||||||||||||||||||||||||
Sbjct  722   TCGCCTTCGCCACTGGTGTTCCTTCCTATATCTACGCATTTCACCGCTACACAGGAAATT  663

Query  790   CCACCACCCTCTACCATACTCTAGCTCGACAGTTTTGAATGCAGTTCCCAGGTTGAGCCC  849
             ||||||||||||||||||||||||||||||||||||||||||||||||||||||||||||
Sbjct  662   CCACCACCCTCTACCATACTCTAGCTCGACAGTTTTGAATGCAGTTCCCAGGTTGAGCCC  603

Query  850   GGGGCTTTCACATCCAACTTAACGAACCACCTACGCGCGCTTTACGCCCAGTAATTCCGA  909
             |||| |||||||||||||||||||||||||||||||||||||||||||||||||||||||
Sbjct  602   GGGGATTTCACATCCAACTTAACGAACCACCTACGCGCGCTTTACGCCCAGTAATTCCGA  543

Query  910   TTAACGCTTGCACCCTCTGTATTACCGCGGCTGCTGGCACAGAGTTAGCCGGTGCTTATT  969
             ||||||||||||||||||||||||||||||||||||||||||||||||||||||||||||
Sbjct  542   TTAACGCTTGCACCCTCTGTATTACCGCGGCTGCTGGCACAGAGTTAGCCGGTGCTTATT  483

Query  970   CTGTCGGTAACGTCAAAACACTAACGTATTAGGTTAATGCCCTTCCTCCCAACTTAAAGT  1029
             ||||||||||||||||||||||||||||||||||||||||||||||||||||||||||||
Sbjct  482   CTGTCGGTAACGTCAAAACACTAACGTATTAGGTTAATGCCCTTCCTCCCAACTTAAAGT  423

Query  1030  GCTTTACAATCCG  1042
             |||||||||||||
Sbjct  422   GCTTTACAATCCG  410
```

Download

FASTA (complete sequence)

FASTA (aligned sequences)

GenBank (complete sequence)

Continue
Cancel

GenBankGraphics

Next
Previous
Descriptions

Uncultured bacterium clone SPN400-90day-85 16S ribosomal RNA gene, partial sequence

Sequence ID: MF314628.1Length: 1502Number of Matches: 1

Related Information

Range 1: 410 to 1442GenBankGraphics

Next Match
Previous Match
First Match

Alignment statistics for match #1

| Score | Expect | Identities | Gaps | Strand | Frame |
| --- | --- | --- | --- | --- | --- |
| 1903 bits(1030) | 0.0() | 1032/1033(99%) | 0/1033(0%) | Plus/Minus |  |

Features:

```
Query  10    CGAAGGTTAGACTAGCTACTTCTGGTGCAACCCACTCCCATGGTGTGACGGGCGGTGTGT  69
             ||||||||||||||||||||||||||||||||||||||||||||||||||||||||||||
Sbjct  1442  CGAAGGTTAGACTAGCTACTTCTGGTGCAACCCACTCCCATGGTGTGACGGGCGGTGTGT  1383

Query  70    ACAAGGCCCGGGAACGTATTCACCGCGACATTCTGATTCGCGATTACTAGCGATTCCGAC  129
             ||||||||||||||||||||||||||||||||||||||||||||||||||||||||||||
Sbjct  1382  ACAAGGCCCGGGAACGTATTCACCGCGACATTCTGATTCGCGATTACTAGCGATTCCGAC  1323

Query  130   TTCACGCAGTCGAGTTGCAGACTGCGATCCGGACTACGATCGGTTTTGTGGGATTAGCTC  189
             ||||||||||||||||||||||||||||||||||||||||||||||||||||||||||||
Sbjct  1322  TTCACGCAGTCGAGTTGCAGACTGCGATCCGGACTACGATCGGTTTTGTGGGATTAGCTC  1263

Query  190   CACCTCGCGGCTTGGCAACCCTCTGTACCGACCATTGTAGCACGTGTGTAGCCCAGGCCG  249
             ||||||||||||||||||||||||||||||||||||||||||||||||||||||||||||
Sbjct  1262  CACCTCGCGGCTTGGCAACCCTCTGTACCGACCATTGTAGCACGTGTGTAGCCCAGGCCG  1203

Query  250   TAAGGGCCATGATGACTTGACGTCATCCCCACCTTCCTCCGGTTTGTCACCGGCAGTCTC  309
             ||||||||||||||||||||||||||||||||||||||||||||||||||||||||||||
Sbjct  1202  TAAGGGCCATGATGACTTGACGTCATCCCCACCTTCCTCCGGTTTGTCACCGGCAGTCTC  1143

Query  310   CTTAGAGTGCCCACCATGACGTGCTGGTAACTAAGGACAAGGGTTGCGCTCGTTACGGGA  369
             ||||||||||||||||||||||||||||||||||||||||||||||||||||||||||||
Sbjct  1142  CTTAGAGTGCCCACCATGACGTGCTGGTAACTAAGGACAAGGGTTGCGCTCGTTACGGGA  1083

Query  370   CTTAACCCAACATCTCACGACACGAGCTGACGACAGCCATGCAGCACCTGTCTCAATGTT  429
             ||||||||||||||||||||||||||||||||||||||||||||||||||||||||||||
Sbjct  1082  CTTAACCCAACATCTCACGACACGAGCTGACGACAGCCATGCAGCACCTGTCTCAATGTT  1023

Query  430   CCCGAAGGCACCAATCCATCTCTGGAAAGTTCATTGGATGTCAAGGCCTGGTAAGGTTCT  489
             ||||||||||||||||||||||||||||||||||||||||||||||||||||||||||||
Sbjct  1022  CCCGAAGGCACCAATCCATCTCTGGAAAGTTCATTGGATGTCAAGGCCTGGTAAGGTTCT  963

Query  490   TCGCGTTGCTTCGAATTAAACCACATGCTCCACCGCTTGTGCGGGCCCCCGTCAATTCAT  549
             ||||||||||||||||||||||||||||||||||||||||||||||||||||||||||||
Sbjct  962   TCGCGTTGCTTCGAATTAAACCACATGCTCCACCGCTTGTGCGGGCCCCCGTCAATTCAT  903

Query  550   TTGAGTTTTAACCTTGCGGCCGTACTCCCCAGGCGGTCAACTTAATGCGTTAGCTGCGCC  609
             ||||||||||||||||||||||||||||||||||||||||||||||||||||||||||||
Sbjct  902   TTGAGTTTTAACCTTGCGGCCGTACTCCCCAGGCGGTCAACTTAATGCGTTAGCTGCGCC  843

Query  610   ACTAAGAGCTCAAGGCTCCCAACGGCTAGTTGACATCGTTTACGGCGTGGACTACCAGGG  669
             ||||||||||||||||||||||||||||||||||||||||||||||||||||||||||||
Sbjct  842   ACTAAGAGCTCAAGGCTCCCAACGGCTAGTTGACATCGTTTACGGCGTGGACTACCAGGG  783

Query  670   TATCTAATCCTGTTTGCTCCCCACGCTTTCGCACCTCAGTGTCAGTATCAGTCCAGGTGG  729
             ||||||||||||||||||||||||||||||||||||||||||||||||||||||||||||
Sbjct  782   TATCTAATCCTGTTTGCTCCCCACGCTTTCGCACCTCAGTGTCAGTATCAGTCCAGGTGG  723

Query  730   TCGCCTTCGCCACTGGTGTTCCTTCCTATATCTACGCATTTCACCGCTACACAGGAAATT  789
             ||||||||||||||||||||||||||||||||||||||||||||||||||||||||||||
Sbjct  722   TCGCCTTCGCCACTGGTGTTCCTTCCTATATCTACGCATTTCACCGCTACACAGGAAATT  663

Query  790   CCACCACCCTCTACCATACTCTAGCTCGACAGTTTTGAATGCAGTTCCCAGGTTGAGCCC  849
             ||||||||||||||||||||||||||||||||||||||||||||||||||||||||||||
Sbjct  662   CCACCACCCTCTACCATACTCTAGCTCGACAGTTTTGAATGCAGTTCCCAGGTTGAGCCC  603

Query  850   GGGGCTTTCACATCCAACTTAACGAACCACCTACGCGCGCTTTACGCCCAGTAATTCCGA  909
             |||| |||||||||||||||||||||||||||||||||||||||||||||||||||||||
Sbjct  602   GGGGATTTCACATCCAACTTAACGAACCACCTACGCGCGCTTTACGCCCAGTAATTCCGA  543

Query  910   TTAACGCTTGCACCCTCTGTATTACCGCGGCTGCTGGCACAGAGTTAGCCGGTGCTTATT  969
             ||||||||||||||||||||||||||||||||||||||||||||||||||||||||||||
Sbjct  542   TTAACGCTTGCACCCTCTGTATTACCGCGGCTGCTGGCACAGAGTTAGCCGGTGCTTATT  483

Query  970   CTGTCGGTAACGTCAAAACACTAACGTATTAGGTTAATGCCCTTCCTCCCAACTTAAAGT  1029
             ||||||||||||||||||||||||||||||||||||||||||||||||||||||||||||
Sbjct  482   CTGTCGGTAACGTCAAAACACTAACGTATTAGGTTAATGCCCTTCCTCCCAACTTAAAGT  423

Query  1030  GCTTTACAATCCG  1042
             |||||||||||||
Sbjct  422   GCTTTACAATCCG  410
```

Download

FASTA (complete sequence)

FASTA (aligned sequences)

GenBank (complete sequence)

Continue
Cancel

GenBankGraphics

Next
Previous
Descriptions

Uncultured bacterium clone SPN400-90day-76 16S ribosomal RNA gene, partial sequence

Sequence ID: MF314621.1Length: 1502Number of Matches: 1

Related Information

Range 1: 410 to 1442GenBankGraphics

Next Match
Previous Match
First Match

Alignment statistics for match #1

| Score | Expect | Identities | Gaps | Strand | Frame |
| --- | --- | --- | --- | --- | --- |
| 1903 bits(1030) | 0.0() | 1032/1033(99%) | 0/1033(0%) | Plus/Minus |  |

Features:

```
Query  10    CGAAGGTTAGACTAGCTACTTCTGGTGCAACCCACTCCCATGGTGTGACGGGCGGTGTGT  69
             ||||||||||||||||||||||||||||||||||||||||||||||||||||||||||||
Sbjct  1442  CGAAGGTTAGACTAGCTACTTCTGGTGCAACCCACTCCCATGGTGTGACGGGCGGTGTGT  1383

Query  70    ACAAGGCCCGGGAACGTATTCACCGCGACATTCTGATTCGCGATTACTAGCGATTCCGAC  129
             ||||||||||||||||||||||||||||||||||||||||||||||||||||||||||||
Sbjct  1382  ACAAGGCCCGGGAACGTATTCACCGCGACATTCTGATTCGCGATTACTAGCGATTCCGAC  1323

Query  130   TTCACGCAGTCGAGTTGCAGACTGCGATCCGGACTACGATCGGTTTTGTGGGATTAGCTC  189
             ||||||||||||||||||||||||||||||||||||||||||||||||||||||||||||
Sbjct  1322  TTCACGCAGTCGAGTTGCAGACTGCGATCCGGACTACGATCGGTTTTGTGGGATTAGCTC  1263

Query  190   CACCTCGCGGCTTGGCAACCCTCTGTACCGACCATTGTAGCACGTGTGTAGCCCAGGCCG  249
             ||||||||||||||||||||||||||||||||||||||||||||||||||||||||||||
Sbjct  1262  CACCTCGCGGCTTGGCAACCCTCTGTACCGACCATTGTAGCACGTGTGTAGCCCAGGCCG  1203

Query  250   TAAGGGCCATGATGACTTGACGTCATCCCCACCTTCCTCCGGTTTGTCACCGGCAGTCTC  309
             ||||||||||||||||||||||||||||||||||||||||||||||||||||||||||||
Sbjct  1202  TAAGGGCCATGATGACTTGACGTCATCCCCACCTTCCTCCGGTTTGTCACCGGCAGTCTC  1143

Query  310   CTTAGAGTGCCCACCATGACGTGCTGGTAACTAAGGACAAGGGTTGCGCTCGTTACGGGA  369
             ||||||||||||||||||||||||||||||||||||||||||||||||||||||||||||
Sbjct  1142  CTTAGAGTGCCCACCATGACGTGCTGGTAACTAAGGACAAGGGTTGCGCTCGTTACGGGA  1083

Query  370   CTTAACCCAACATCTCACGACACGAGCTGACGACAGCCATGCAGCACCTGTCTCAATGTT  429
             ||||||||||||||||||||||||||||||||||||||||||||||||||||||||||||
Sbjct  1082  CTTAACCCAACATCTCACGACACGAGCTGACGACAGCCATGCAGCACCTGTCTCAATGTT  1023

Query  430   CCCGAAGGCACCAATCCATCTCTGGAAAGTTCATTGGATGTCAAGGCCTGGTAAGGTTCT  489
             ||||||||||||||||||||||||||||||||||||||||||||||||||||||||||||
Sbjct  1022  CCCGAAGGCACCAATCCATCTCTGGAAAGTTCATTGGATGTCAAGGCCTGGTAAGGTTCT  963

Query  490   TCGCGTTGCTTCGAATTAAACCACATGCTCCACCGCTTGTGCGGGCCCCCGTCAATTCAT  549
             ||||||||||||||||||||||||||||||||||||||||||||||||||||||||||||
Sbjct  962   TCGCGTTGCTTCGAATTAAACCACATGCTCCACCGCTTGTGCGGGCCCCCGTCAATTCAT  903

Query  550   TTGAGTTTTAACCTTGCGGCCGTACTCCCCAGGCGGTCAACTTAATGCGTTAGCTGCGCC  609
             ||||||||||||||||||||||||||||||||||||||||||||||||||||||||||||
Sbjct  902   TTGAGTTTTAACCTTGCGGCCGTACTCCCCAGGCGGTCAACTTAATGCGTTAGCTGCGCC  843

Query  610   ACTAAGAGCTCAAGGCTCCCAACGGCTAGTTGACATCGTTTACGGCGTGGACTACCAGGG  669
             ||||||||||||||||||||||||||||||||||||||||||||||||||||||||||||
Sbjct  842   ACTAAGAGCTCAAGGCTCCCAACGGCTAGTTGACATCGTTTACGGCGTGGACTACCAGGG  783

Query  670   TATCTAATCCTGTTTGCTCCCCACGCTTTCGCACCTCAGTGTCAGTATCAGTCCAGGTGG  729
             ||||||||||||||||||||||||||||||||||||||||||||||||||||||||||||
Sbjct  782   TATCTAATCCTGTTTGCTCCCCACGCTTTCGCACCTCAGTGTCAGTATCAGTCCAGGTGG  723

Query  730   TCGCCTTCGCCACTGGTGTTCCTTCCTATATCTACGCATTTCACCGCTACACAGGAAATT  789
             ||||||||||||||||||||||||||||||||||||||||||||||||||||||||||||
Sbjct  722   TCGCCTTCGCCACTGGTGTTCCTTCCTATATCTACGCATTTCACCGCTACACAGGAAATT  663

Query  790   CCACCACCCTCTACCATACTCTAGCTCGACAGTTTTGAATGCAGTTCCCAGGTTGAGCCC  849
             ||||||||||||||||||||||||||||||||||||||||||||||||||||||||||||
Sbjct  662   CCACCACCCTCTACCATACTCTAGCTCGACAGTTTTGAATGCAGTTCCCAGGTTGAGCCC  603

Query  850   GGGGCTTTCACATCCAACTTAACGAACCACCTACGCGCGCTTTACGCCCAGTAATTCCGA  909
             |||| |||||||||||||||||||||||||||||||||||||||||||||||||||||||
Sbjct  602   GGGGATTTCACATCCAACTTAACGAACCACCTACGCGCGCTTTACGCCCAGTAATTCCGA  543

Query  910   TTAACGCTTGCACCCTCTGTATTACCGCGGCTGCTGGCACAGAGTTAGCCGGTGCTTATT  969
             ||||||||||||||||||||||||||||||||||||||||||||||||||||||||||||
Sbjct  542   TTAACGCTTGCACCCTCTGTATTACCGCGGCTGCTGGCACAGAGTTAGCCGGTGCTTATT  483

Query  970   CTGTCGGTAACGTCAAAACACTAACGTATTAGGTTAATGCCCTTCCTCCCAACTTAAAGT  1029
             ||||||||||||||||||||||||||||||||||||||||||||||||||||||||||||
Sbjct  482   CTGTCGGTAACGTCAAAACACTAACGTATTAGGTTAATGCCCTTCCTCCCAACTTAAAGT  423

Query  1030  GCTTTACAATCCG  1042
             |||||||||||||
Sbjct  422   GCTTTACAATCCG  410
```

```

```


BLAST is a registered trademark of the National Library of Medicine

Support center
Mailing list


YouTube

- National Library Of Medicine
- National Institutes Of Health
- U.S. Department of Health & Human Services
- USA.gov

### NCBI


National Center for Biotechnology Information,
 U.S. National Library of Medicine

8600 Rockville Pike,
Bethesda
 MD,
20894
USA

Policies and Guidelines
|
Contact


PreferencesTurn off

External link. Please review our privacy policy.
