# Supplementary material for: Isothermal microcalorimetry for thermal viable count of microorganisms in pure cultures and stabilized formulations
Source: BMC Microbiol. 2019 Mar 21;19:65. doi: 10.1186/s12866-019-1432-8 (PMC6429831; doi:10.1186/s12866-019-1432-8)
Supplement: Supplementary file 3 — 16S rRNA gene sequences’ BLASTN hits in zipped HTML format. (ZIP 15810 kb) [file 12866_2019_1432_MOESM3_ESM.zip › Best blastn hits/NCBI Blast_39 pale upper band R -- 9..1066 of sequence.html]

NCBI Blast:39 pale upper band R -- 9..1066 of sequence


- NCBI Home
- Sign in to NCBI
- Skip to Main Content
- Skip to Navigation
- About NCBI Accesskeys

U.S. National Library of Medicine

NCBI
National Center for Biotechnology Information

- My NCBI
- Sign in to NCBI
- Register
- Sign Out

BLAST ® » blastn suite » RID-A4W5R2JG015


- Home
- Recent Results
- Saved Strategies
- Help

BLAST Results


Edit and Resubmit
Save Search Strategies
[Sign in above to save your search strategy]

Formatting options 


Download


How to read this page
Blast report description
Questions/comments


|  |  |
| --- | --- |
| Formatting options | |
| Show | Alignment as  HTML Plain text   Old View Reset form to defaults [?]  These options control formatting of alignments in results pages. The default is HTML, but other formats (including plain text) are available. PSSM and PssmWithParameters are representations of Position Specific Scoring Matrices and are only available for PSI-BLAST. The Advanced view option allows the database descriptions to be sorted by various indices in a table. |
| Alignment View | Pairwise Pairwise with dots for identities Query-anchored with dots for identities Query-anchored with letters for identities Flat query-anchored with dots for identities Flat query-anchored with letters for identities [?]  Choose how to view alignments. The default "pairwise" view shows how each subject sequence aligns individually to the query sequence. The "query-anchored" view shows how all subject sequences align to the query sequence. For each view type, you can choose to show "identities" (matching residues) as letters or dots. more... |
| Display | Graphical Overview   Linkout   Sequence Retrieval  NCBI-gi   CDS feature [?]  - Graphical Overview: Graphical Overview: Show graph of similar sequence regions aligned to query.   more... - NCBI-gi: Show NCBI gi identifiers. - CDS feature: Show annotated coding region and translation.   more... |
| Masking | Character:   X for protein, n for nucleotide Lower Case  Color:  Black Grey Red [?]  - Masking Character: Display masked (filtered) sequence regions as lower-case or as specific letters (N for nucleotide, P for protein). - Masking Color: Display masked sequence regions in the given color. |
| Limit results | Descriptions:  10 50 100 Graphical overview:  0 10 50 100  Alignments:  0 10 50 100 Line length:  60 90 120 150 [?]  - Descriptions: Show short descriptions for up to the given number of sequences. - Alignments: Show alignments for up to the given number of sequences, in order of statistical significance. - Line lenghth: Number of letters to show on one line in an alignment. |
|  | Organism Type common name, binomial, taxid, or group name. Only 20 top taxa will be shown.     Exclude    [?]  Show only sequences from the given organism. |
|  | Entrez query:  [?]  Show only those sequences that match the given Entrez query. more... |
|  | Expect Min:  Expect Max:  [?]  Show only sequences with expect values in the given range. more... |
|  | Percent Identity Min:  Percent Identity Max:  [?]  Show only sequences with percent identity values in the given range. |
| Format for | PSI-BLAST with inclusion threshold:  [?]  - Format for PSI-BLAST: The Position-Specific Iterated BLAST (PSI-BLAST) program performs iterative searches with a protein query,   in which sequences found in one round of search are used to build a custom score model for the next round.   more... - Inclusion Threshold: This sets the statistical significance threshold for including a sequence in the model used   by PSI-BLAST to create the PSSM on the next iteration. |

|  |  |  |  |  |  |
| --- | --- | --- | --- | --- | --- |
| Download | | | | | |
| Alignment  Text XML ASN.1 JSON Seq-align Hit Table(text) Hit Table(csv) Multiple-file XML2 Single-file XML2 Multiple-file JSON Single-file JSON SAM | Search Strategies  ASN.1 | PSSM to restart search  PSSM | [?] |

The Download link provides BLAST output that may be used as input to another program.
This includes parseable formats such as the tabular report or XML as well as the Search Strategy files read by the BLAST+ applications.
More details on the parseable (XML, tabular, and ASN.1) reports can be found at
https://www.ncbi.nlm.nih.gov/books/NBK153387/  
  

The following formats are offered under the Alignment section:  
1). "Text". Non-HTML standard BLAST report.  
2). "XML". XML report based upon the DTD at https://www.ncbi.nlm.nih.gov/data\_specs/dtd/NCBI\_BlastOutput.dtd  
3). "ASN.1". Alignment written out in Abstract Syntax Notation 1.  
4). "JSON Seq-align". Alignment written out in JSON.  
4). "Hit Table(text)". The tabular report as text.  
5). "Hit Table(csv)". The tabular report ready for import into spread-sheet programs like Excel.  
6). "XML2". New XML format described at ftp://ftp.ncbi.nlm.nih.gov/blast/documents/NEWXML/xml2.pdf.  
7). "JSON". New JSON format described at ftp://ftp.ncbi.nlm.nih.gov/blast/documents/NEWXML/xml2.pdf.  
8). "SAM". Sequence Alignment Map format.

XML2 and JSON can be downloaded either as one file per query (multiple-file) or one file for all queries (single-file). These formats are listed as Multiple-file XML2 (and JSON) or Single-file XML (and JSON).

The following report is offered under the Search Strategy section:  
1). "ASN.1" Search Strategy. A record of the parameters, query, and database used in the search. This file can be used to start a stand-alone BLAST search, see
https://www.ncbi.nlm.nih.gov/books/NBK1763/#CmdLineAppsManual.I455\_BLAST\_search\_stra


# Job title: 39 pale upper band R -- 9..1066 of sequence

Results for:

lcl|Query\_53945 39 pale upper band R -- 9..1066 of sequence(1058bp)
[?]

Your BLAST job specified more than one input sequence.
This box lets you choose which input sequence to show BLAST results for.

RID
:   A4W5R2JG015 (Expires on 03-10 18:32 pm)

Query ID
:   lcl|Query\_53945
:   lcl|Query\_53945

Description
:   39 pale upper band R -- 9..1066 of sequence

Molecule type
:   nucleic acid

Query Length
:   1058

Database Name
:   nr

Description
:   Nucleotide collection (nt) See details

Program
:   BLASTN 2.8.0+ Citation

  

Reference 

Zheng Zhang, Scott Schwartz, Lukas Wagner, and Webb Miller (2000), "A greedy algorithm for aligning DNA sequences", J Comput Biol 2000; 7(1-2):203-14.

Reference - database indexing

Aleksandr Morgulis, George Coulouris, Yan Raytselis, Thomas L. Madden, Richa Agarwala, Alejandro A. Schäffer (2008), "Database Indexing for Production MegaBLAST Searches", Bioinformatics 24:1757-1764.

Other reports:
Search Summary

[Taxonomy reports]
[Distance tree of results]
[MSA viewer]

Search Parameters

| Search parameter name | Search parameter value |
| --- | --- |
| Program | blastn |
| Word size | 28 |
| Expect value | 10 |
| Hitlist size | 100 |
| Match/Mismatch scores | 1,-2 |
| Gapcosts | 0,2.5 |
| Low Complexity Filter | Yes |
| Filter string | L;m; |
| Genetic Code | 1 |

Database

| Database parameter name | Database parameter value |
| --- | --- |
| Posted date | Mar 7, 2018 1:58 PM |
| Number of letters | 174,044,644,244 |
| Number of sequences | 46,882,714 |
| Entrez query | Includes:  Excludes:  None |

Karlin-Altschul statistics

| Params | Ungapped | Gapped |
| --- | --- | --- |
| Lambda | 1.33271 | 1.28 |
| K | 0.620991 | 0.46 |
| H | 1.12409 | 0.85 |

Results Statistics

| Results Statistics parameter name | Results Statistics parameter value |
| --- | --- |
| Length adjustment | 35 |
| Effective length of query | 1023 |
| Effective length of database | 172403749254 |
| Effective search space | 176369035486842 |
| Effective search space used | 176369035486842 |


## Graphic Summary

### Distribution of the top 134 Blast Hits on 100 subject sequences [?]

The graphic is an overview of the database sequences aligned to the query sequence. These are represented horizontal bars colored coded by score and showing the extent
of the alignment on the query sequence. Separate aligned regions on the same database sequence are connected by a thin grey line.
Mousing over an alignment shows the database sequence title. Clicking an alignment displays a box with more details about the alignment and
link to the sequence alignment itself in the Alignments section of the report.

Mouse over to see the title, click to show alignments

Color key for alignment scores

<40

40-50

50-80

80-200

>=200

Query

1

200

400

600

800

1000

Pseudomonas brassicacearum isolate MA250 16S ribosomal ..

Score:1910 Evalue:0

Accession:DQ886486.1

Alignment

Pseudomonas putida partial 16S rRNA gene, strain CFBP 4..

Score:1908 Evalue:0

Accession:HF545841.1

Alignment

Pseudomonas sp. S-3 16S ribosomal RNA gene, partial seq..

Score:1908 Evalue:0

Accession:KC207086.1

Alignment

Pseudomonas sp. strain 7.3 16S ribosomal RNA gene, part..

Score:1905 Evalue:0

Accession:KY542120.1

Alignment

Pseudomonas fluorescens strain SBR10 16S ribosomal RNA ..

Score:1905 Evalue:0

Accession:KX018310.1

Alignment

Pseudomonas sp. cpRA293 16S ribosomal RNA gene, partial..

Score:1905 Evalue:0

Accession:KJ510220.1

Alignment

Pseudomonas brassicacearum strain Kr21 16S ribosomal RN..

Score:1905 Evalue:0

Accession:KT215482.1

Alignment

Pseudomonas brassicacearum strain IHB B 13650 16S ribos..

Score:1905 Evalue:0

Accession:KP762561.1

Alignment

Pseudomonas brassicacearum subsp. neoaurantiaca strain ..

Score:1905 Evalue:0

Accession:KP762555.1

Alignment

Pseudomonas sp. 41(2015) 16S ribosomal RNA gene, partia..

Score:1905 Evalue:0

Accession:KR080568.1

Alignment

Pseudomonas sp. SAM1 16S ribosomal RNA gene, partial se..

Score:1905 Evalue:0

Accession:KM269192.1

Alignment

Pseudomonas brassicacearum strain WK-444s 16S ribosomal..

Score:1905 Evalue:0

Accession:KF580861.1

Alignment

Pseudomonas fluorescens gene for 16S rRNA, partial sequ..

Score:1905 Evalue:0

Accession:AB621593.1

Alignment

Pseudomonas thivervalensis 16S ribosomal RNA gene, part..

Score:1905 Evalue:0

Accession:JN628032.1

Alignment

Pseudomonas fluorescens 16S rRNA gene, strain C7R12

Score:1905 Evalue:0

Accession:AM229082.1

Alignment

Pseudomonas brassicacearum strain YC-1 16S ribosomal RN..

Score:1903 Evalue:0

Accession:KY753310.1

Alignment

Pseudomonas brassicacearum strain FC-7 16S ribosomal RN..

Score:1903 Evalue:0

Accession:KY649379.1

Alignment

Pseudomonas brassicacearum strain 13B-23 16S ribosomal ..

Score:1903 Evalue:0

Accession:KR083019.1

Alignment

Pseudomonas brassicacearum strain 3B1 16S ribosomal RNA..

Score:1903 Evalue:0

Accession:KR611047.1

Alignment

Uncultured Pseudomonas sp. clone CGMCG 6071 16S ribosom..

Score:1903 Evalue:0

Accession:JX077087.1

Alignment

Pseudomonas fluorescens strain J2 16S ribosomal RNA gen..

Score:1901 Evalue:0

Accession:JN399995.1

Alignment

Pseudomonas brassicacearum strain J12 16S ribosomal RNA..

Score:1901 Evalue:0

Accession:JN605747.1

Alignment

Uncultured bacterium clone Untreatedsoil-0day-94 16S ri..

Score:1899 Evalue:0

Accession:MF314815.2

Alignment

Uncultured bacterium clone Untreatedsoil-0day-1 16S rib..

Score:1899 Evalue:0

Accession:MF314725.2

Alignment

Pseudomonas fluorescens strain 2P24 chromosome, complet..

Score:1899 Evalue:0

Accession:CP025542.1

Alignment

Pseudomonas brassicacearum strain LBUM300 16S ribosomal..

Score:1899 Evalue:0

Accession:MG461459.1

Alignment

Pseudomonas putida strain 42R-P6 16S ribosomal RNA gene..

Score:1899 Evalue:0

Accession:MF062638.1

Alignment

Uncultured bacterium clone SPN400-90day-85 16S ribosoma..

Score:1899 Evalue:0

Accession:MF314628.1

Alignment

Uncultured bacterium clone SPN400-90day-76 16S ribosoma..

Score:1899 Evalue:0

Accession:MF314621.1

Alignment

Uncultured bacterium clone SPN400-90day-50 16S ribosoma..

Score:1899 Evalue:0

Accession:MF314595.1

Alignment

Uncultured bacterium clone SPN400-90day-46 16S ribosoma..

Score:1899 Evalue:0

Accession:MF314592.1

Alignment

Uncultured bacterium clone SPN400-90day-43 16S ribosoma..

Score:1899 Evalue:0

Accession:MF314589.1

Alignment

Uncultured bacterium clone SPN400-90day-20 16S ribosoma..

Score:1899 Evalue:0

Accession:MF314569.1

Alignment

Uncultured bacterium clone SPN400-90day-6 16S ribosomal..

Score:1899 Evalue:0

Accession:MF314558.1

Alignment

Pseudomonas brassicacearum strain Pv10 16S ribosomal RN..

Score:1899 Evalue:0

Accession:MF624719.1

Alignment

Pseudomonas jessenii strain AP3\_16 16S ribosomal RNA ge..

Score:1899 Evalue:0

Accession:MF498772.1

Alignment

Pseudomonas sp. strain A9 16S ribosomal RNA gene, parti..

Score:1899 Evalue:0

Accession:KX859150.1

Alignment

Pseudomonas sp. HA-09 partial 16S rRNA gene, isolate HA..

Score:1899 Evalue:0

Accession:LT844660.1

Alignment

Pseudomonas fluorescens strain PFR1 16S ribosomal RNA g..

Score:1899 Evalue:0

Accession:MF000304.1

Alignment

Pseudomonas brassicacearum strain SKUAST-K21 16S riboso..

Score:1899 Evalue:0

Accession:KY612271.1

Alignment

Pseudomonas syringae strain yangyueP4 16S ribosomal RNA..

Score:1899 Evalue:0

Accession:KU977139.1

Alignment

Pseudomonas brassicacearum strain B04 16S ribosomal RNA..

Score:1899 Evalue:0

Accession:KU878092.1

Alignment

Pseudomonas synxantha strain LMG 2190 genome assembly, ..

Score:1899 Evalue:0

Accession:LT629786.1

Alignment

Pseudomonas brassicacearum strain BS3663 genome assembl..

Score:1899 Evalue:0

Accession:LT629713.1

Alignment

Pseudomonas brassicacearum strain TSDS1 16S ribosomal R..

Score:1899 Evalue:0

Accession:KX984045.1

Alignment

Pseudomonas brassicacearum strain Delaware 16S ribosoma..

Score:1899 Evalue:0

Accession:KT695846.1

Alignment

Pseudomonas brassicacearum strain Wood1 16S ribosomal R..

Score:1899 Evalue:0

Accession:KT695843.1

Alignment

Pseudomonas brassicacearum strain 93F8 16S ribosomal RN..

Score:1899 Evalue:0

Accession:KT695841.1

Alignment

Pseudomonas brassicacearum strain L13-6-12, complete ge..

Score:1899 Evalue:0

Accession:CP014693.1

Alignment

Pseudomonas brassicacearum strain KK 5 16S ribosomal RN..

Score:1899 Evalue:0

Accession:KP858915.1

Alignment

Pseudomonas fluorescens strain FW300-N2E2 genome

Score:1899 Evalue:0

Accession:CP015225.1

Alignment

Pseudomonas jessenii partial 16S rRNA gene, strain R-52..

Score:1899 Evalue:0

Accession:LN995697.1

Alignment

Pseudomonas brassicacearum strain LBUM300, complete gen..

Score:1899 Evalue:0

Accession:CP012680.1

Alignment

Pseudomonas corrugata strain G1 16S ribosomal RNA gene,..

Score:1899 Evalue:0

Accession:KT957303.1

Alignment

Pseudomonas brassicacearum strain SM27 16S ribosomal RN..

Score:1899 Evalue:0

Accession:KR855698.1

Alignment

Pseudomonas fluorescens strain FW300-N2C3, complete gen..

Score:1899 Evalue:0

Accession:CP012831.1

Alignment

## Descriptions

, Reading indexes 1-5, displaying indexes 1-5


Load next setPrevious Match

Sequences producing significant alignments:

Show all columns  of the table presenting sequences producing significant alignments 

Select:AllNone
Selected:0

Alignments
Download

FASTA (complete sequence)

FASTA (aligned sequences)

GenBank (complete sequence)

Hit Table (text)

Hit Table (CSV)

Text

XML

ASN.1

Continue
Cancel

GenBank 
Graphics
Distance tree of results
Multiple alignment
Show/hide columns of the table presenting sequences producing significant alignments 

Available columns

Description  
Max Score  
Total Score  
Coverage  
E-value  
IdentN  
Accession  
Restore Defaults
Ok
Cancel

Sequences producing significant alignments:

| Select for downloading or viewing reports | Description | Max score | Total score | Query cover | E value | Ident | Accession |
| --- | --- | --- | --- | --- | --- | --- | --- |
| 1Select seq DQ886486.1 | Pseudomonas brassicacearum isolate MA250 16S ribosomal RNA gene, partial sequence | 1910 | 1910 | 98% | 0.0 | 99% | DQ886486.1 |
| 2Select seq HF545841.1 | Pseudomonas putida partial 16S rRNA gene, strain CFBP 4629 | 1908 | 1908 | 99% | 0.0 | 99% | HF545841.1 |
| 3Select seq KC207086.1 | Pseudomonas sp. S-3 16S ribosomal RNA gene, partial sequence | 1908 | 1908 | 99% | 0.0 | 99% | KC207086.1 |
| 4Select seq KY542120.1 | Pseudomonas sp. strain 7.3 16S ribosomal RNA gene, partial sequence | 1905 | 1905 | 98% | 0.0 | 99% | KY542120.1 |
| 5Select seq KX018310.1 | Pseudomonas fluorescens strain SBR10 16S ribosomal RNA gene, partial sequence | 1905 | 1905 | 98% | 0.0 | 99% | KX018310.1 |
| 6Select seq KJ510220.1 | Pseudomonas sp. cpRA293 16S ribosomal RNA gene, partial sequence | 1905 | 1905 | 98% | 0.0 | 99% | KJ510220.1 |
| 7Select seq KT215482.1 | Pseudomonas brassicacearum strain Kr21 16S ribosomal RNA gene, partial sequence | 1905 | 1905 | 98% | 0.0 | 99% | KT215482.1 |
| 8Select seq KP762561.1 | Pseudomonas brassicacearum strain IHB B 13650 16S ribosomal RNA gene, partial sequence | 1905 | 1905 | 98% | 0.0 | 99% | KP762561.1 |
| 9Select seq KP762555.1 | Pseudomonas brassicacearum subsp. neoaurantiaca strain IHB B 13645 16S ribosomal RNA gene, partial sequence | 1905 | 1905 | 98% | 0.0 | 99% | KP762555.1 |
| 10Select seq KR080568.1 | Pseudomonas sp. 41(2015) 16S ribosomal RNA gene, partial sequence | 1905 | 1905 | 98% | 0.0 | 99% | KR080568.1 |
| 11Select seq KM269192.1 | Pseudomonas sp. SAM1 16S ribosomal RNA gene, partial sequence | 1905 | 1905 | 98% | 0.0 | 99% | KM269192.1 |
| 12Select seq KF580861.1 | Pseudomonas brassicacearum strain WK-444s 16S ribosomal RNA gene, partial sequence | 1905 | 1905 | 98% | 0.0 | 99% | KF580861.1 |
| 13Select seq AB621593.1 | Pseudomonas fluorescens gene for 16S rRNA, partial sequence, strain: MPF29 | 1905 | 1905 | 98% | 0.0 | 99% | AB621593.1 |
| 14Select seq JN628032.1 | Pseudomonas thivervalensis 16S ribosomal RNA gene, partial sequence | 1905 | 1905 | 98% | 0.0 | 99% | JN628032.1 |
| 15Select seq AM229082.1 | Pseudomonas fluorescens 16S rRNA gene, strain C7R12 | 1905 | 1905 | 98% | 0.0 | 99% | AM229082.1 |
| 16Select seq KY753310.1 | Pseudomonas brassicacearum strain YC-1 16S ribosomal RNA gene, partial sequence | 1903 | 1903 | 99% | 0.0 | 99% | KY753310.1 |
| 17Select seq KY649379.1 | Pseudomonas brassicacearum strain FC-7 16S ribosomal RNA gene, partial sequence | 1903 | 1903 | 99% | 0.0 | 99% | KY649379.1 |
| 18Select seq KR083019.1 | Pseudomonas brassicacearum strain 13B-23 16S ribosomal RNA gene, partial sequence | 1903 | 1903 | 99% | 0.0 | 99% | KR083019.1 |
| 19Select seq KR611047.1 | Pseudomonas brassicacearum strain 3B1 16S ribosomal RNA gene, partial sequence | 1903 | 1903 | 99% | 0.0 | 99% | KR611047.1 |
| 20Select seq JX077087.1 | Uncultured Pseudomonas sp. clone CGMCG 6071 16S ribosomal RNA gene, partial sequence | 1903 | 1903 | 99% | 0.0 | 99% | JX077087.1 |
| 21Select seq JN399995.1 | Pseudomonas fluorescens strain J2 16S ribosomal RNA gene, partial sequence | 1901 | 1901 | 99% | 0.0 | 99% | JN399995.1 |
| 22Select seq JN605747.1 | Pseudomonas brassicacearum strain J12 16S ribosomal RNA gene, partial sequence | 1901 | 1901 | 98% | 0.0 | 99% | JN605747.1 |
| 23Select seq MF314815.2 | Uncultured bacterium clone Untreatedsoil-0day-94 16S ribosomal RNA gene, partial sequence | 1899 | 1899 | 98% | 0.0 | 99% | MF314815.2 |
| 24Select seq MF314725.2 | Uncultured bacterium clone Untreatedsoil-0day-1 16S ribosomal RNA gene, partial sequence | 1899 | 1899 | 98% | 0.0 | 99% | MF314725.2 |
| 25Select seq CP025542.1 | Pseudomonas fluorescens strain 2P24 chromosome, complete genome | 1899 | 9486 | 98% | 0.0 | 99% | CP025542.1 |
| 26Select seq MG461459.1 | Pseudomonas brassicacearum strain LBUM300 16S ribosomal RNA gene, partial sequence | 1899 | 1899 | 98% | 0.0 | 99% | MG461459.1 |
| 27Select seq MF062638.1 | Pseudomonas putida strain 42R-P6 16S ribosomal RNA gene, partial sequence | 1899 | 1899 | 98% | 0.0 | 99% | MF062638.1 |
| 28Select seq MF314628.1 | Uncultured bacterium clone SPN400-90day-85 16S ribosomal RNA gene, partial sequence | 1899 | 1899 | 98% | 0.0 | 99% | MF314628.1 |
| 29Select seq MF314621.1 | Uncultured bacterium clone SPN400-90day-76 16S ribosomal RNA gene, partial sequence | 1899 | 1899 | 98% | 0.0 | 99% | MF314621.1 |
| 30Select seq MF314595.1 | Uncultured bacterium clone SPN400-90day-50 16S ribosomal RNA gene, partial sequence | 1899 | 1899 | 98% | 0.0 | 99% | MF314595.1 |
| 31Select seq MF314592.1 | Uncultured bacterium clone SPN400-90day-46 16S ribosomal RNA gene, partial sequence | 1899 | 1899 | 98% | 0.0 | 99% | MF314592.1 |
| 32Select seq MF314589.1 | Uncultured bacterium clone SPN400-90day-43 16S ribosomal RNA gene, partial sequence | 1899 | 1899 | 98% | 0.0 | 99% | MF314589.1 |
| 33Select seq MF314569.1 | Uncultured bacterium clone SPN400-90day-20 16S ribosomal RNA gene, partial sequence | 1899 | 1899 | 98% | 0.0 | 99% | MF314569.1 |
| 34Select seq MF314558.1 | Uncultured bacterium clone SPN400-90day-6 16S ribosomal RNA gene, partial sequence | 1899 | 1899 | 98% | 0.0 | 99% | MF314558.1 |
| 35Select seq MF624719.1 | Pseudomonas brassicacearum strain Pv10 16S ribosomal RNA gene, partial sequence | 1899 | 1899 | 98% | 0.0 | 99% | MF624719.1 |
| 36Select seq MF498772.1 | Pseudomonas jessenii strain AP3\_16 16S ribosomal RNA gene, partial sequence | 1899 | 1899 | 98% | 0.0 | 99% | MF498772.1 |
| 37Select seq KX859150.1 | Pseudomonas sp. strain A9 16S ribosomal RNA gene, partial sequence | 1899 | 1899 | 98% | 0.0 | 99% | KX859150.1 |
| 38Select seq LT844660.1 | Pseudomonas sp. HA-09 partial 16S rRNA gene, isolate HA-09 | 1899 | 1899 | 98% | 0.0 | 99% | LT844660.1 |
| 39Select seq MF000304.1 | Pseudomonas fluorescens strain PFR1 16S ribosomal RNA gene, partial sequence | 1899 | 1899 | 98% | 0.0 | 99% | MF000304.1 |
| 40Select seq KY612271.1 | Pseudomonas brassicacearum strain SKUAST-K21 16S ribosomal RNA gene, partial sequence | 1899 | 1899 | 98% | 0.0 | 99% | KY612271.1 |
| 41Select seq KU977139.1 | Pseudomonas syringae strain yangyueP4 16S ribosomal RNA gene, partial sequence | 1899 | 1899 | 98% | 0.0 | 99% | KU977139.1 |
| 42Select seq KU878092.1 | Pseudomonas brassicacearum strain B04 16S ribosomal RNA gene, partial sequence | 1899 | 1899 | 98% | 0.0 | 99% | KU878092.1 |
| 43Select seq LT629786.1 | Pseudomonas synxantha strain LMG 2190 genome assembly, chromosome: I | 1899 | 12642 | 98% | 0.0 | 99% | LT629786.1 |
| 44Select seq LT629713.1 | Pseudomonas brassicacearum strain BS3663 genome assembly, chromosome: I | 1899 | 9497 | 98% | 0.0 | 99% | LT629713.1 |
| 45Select seq KX984045.1 | Pseudomonas brassicacearum strain TSDS1 16S ribosomal RNA gene, partial sequence | 1899 | 1899 | 98% | 0.0 | 99% | KX984045.1 |
| 46Select seq KT695846.1 | Pseudomonas brassicacearum strain Delaware 16S ribosomal RNA gene, partial sequence | 1899 | 1899 | 98% | 0.0 | 99% | KT695846.1 |
| 47Select seq KT695843.1 | Pseudomonas brassicacearum strain Wood1 16S ribosomal RNA gene, partial sequence | 1899 | 1899 | 98% | 0.0 | 99% | KT695843.1 |
| 48Select seq KT695841.1 | Pseudomonas brassicacearum strain 93F8 16S ribosomal RNA gene, partial sequence | 1899 | 1899 | 98% | 0.0 | 99% | KT695841.1 |
| 49Select seq CP014693.1 | Pseudomonas brassicacearum strain L13-6-12, complete genome | 1899 | 9497 | 98% | 0.0 | 99% | CP014693.1 |
| 50Select seq KP858915.1 | Pseudomonas brassicacearum strain KK 5 16S ribosomal RNA gene, partial sequence | 1899 | 1899 | 98% | 0.0 | 99% | KP858915.1 |
| 51Select seq CP015225.1 | Pseudomonas fluorescens strain FW300-N2E2 genome | 1899 | 9489 | 98% | 0.0 | 99% | CP015225.1 |
| 52Select seq LN995697.1 | Pseudomonas jessenii partial 16S rRNA gene, strain R-52636 | 1899 | 1899 | 98% | 0.0 | 99% | LN995697.1 |
| 53Select seq CP012680.1 | Pseudomonas brassicacearum strain LBUM300, complete genome | 1899 | 9497 | 98% | 0.0 | 99% | CP012680.1 |
| 54Select seq KT957303.1 | Pseudomonas corrugata strain G1 16S ribosomal RNA gene, partial sequence | 1899 | 1899 | 98% | 0.0 | 99% | KT957303.1 |
| 55Select seq KR855698.1 | Pseudomonas brassicacearum strain SM27 16S ribosomal RNA gene, partial sequence | 1899 | 1899 | 98% | 0.0 | 99% | KR855698.1 |
| 56Select seq CP012831.1 | Pseudomonas fluorescens strain FW300-N2C3, complete genome | 1899 | 9497 | 98% | 0.0 | 99% | CP012831.1 |
| 57Select seq KR822273.1 | Pseudomonas migulae strain DD290 16S ribosomal RNA gene, partial sequence | 1899 | 1899 | 98% | 0.0 | 99% | KR822273.1 |
| 58Select seq KT321681.1 | Pseudomonas fluorescens 16S ribosomal RNA gene, partial sequence | 1899 | 1899 | 98% | 0.0 | 99% | KT321681.1 |
| 59Select seq KP742980.1 | Pseudomonas thivervalensis strain MAH1 16S ribosomal RNA gene, partial sequence | 1899 | 1899 | 98% | 0.0 | 99% | KP742980.1 |
| 60Select seq KR611048.1 | Pseudomonas brassicacearum strain 11B23 16S ribosomal RNA gene, partial sequence | 1899 | 1899 | 98% | 0.0 | 99% | KR611048.1 |
| 61Select seq KR080563.1 | Pseudomonas sp. 15(2015) 16S ribosomal RNA gene, partial sequence | 1899 | 1899 | 98% | 0.0 | 99% | KR080563.1 |
| 62Select seq KR080562.1 | Pseudomonas sp. 12(2015) 16S ribosomal RNA gene, partial sequence | 1899 | 1899 | 98% | 0.0 | 99% | KR080562.1 |
| 63Select seq KP730603.1 | Pseudomonas brassicacearum subsp. brassicacearum strain BW0808 16S ribosomal RNA gene, partial sequence | 1899 | 1899 | 98% | 0.0 | 99% | KP730603.1 |
| 64Select seq LC015570.1 | Pseudomonas brassicacearum subsp. brassicacearum gene for 16S ribosomal RNA, partial sequence, strain: AF129 | 1899 | 1899 | 98% | 0.0 | 99% | LC015570.1 |
| 65Select seq LC015569.1 | Pseudomonas brassicacearum subsp. brassicacearum gene for 16S ribosomal RNA, partial sequence, strain: AF5 | 1899 | 1899 | 98% | 0.0 | 99% | LC015569.1 |
| 66Select seq LC015567.1 | Pseudomonas brassicacearum subsp. neoaurantiaca gene for 16S ribosomal RNA, partial sequence, strain: AF82 | 1899 | 1899 | 98% | 0.0 | 99% | LC015567.1 |
| 67Select seq KM030057.1 | Pseudomonas sp. TY1210 16S ribosomal RNA gene, partial sequence | 1899 | 1899 | 98% | 0.0 | 99% | KM030057.1 |
| 68Select seq KJ420530.1 | Pseudomonas thivervalensis strain PE32 16S ribosomal RNA gene, partial sequence | 1899 | 1899 | 98% | 0.0 | 99% | KJ420530.1 |
| 69Select seq KF148637.1 | Pseudomonas fluorescens strain JK15 16S ribosomal RNA gene, partial sequence | 1899 | 1899 | 98% | 0.0 | 99% | KF148637.1 |
| 70Select seq KF840730.1 | Pseudomonas kilonensis strain JX22 16S ribosomal RNA gene, partial sequence | 1899 | 1899 | 98% | 0.0 | 99% | KF840730.1 |
| 71Select seq KF835389.1 | Pseudomonas sp. SDW-16 16S ribosomal RNA gene, partial sequence | 1899 | 1899 | 98% | 0.0 | 99% | KF835389.1 |
| 72Select seq KF475874.1 | Pseudomonas brassicacearum strain IHB B 821 16S ribosomal RNA gene, partial sequence | 1899 | 1899 | 98% | 0.0 | 99% | KF475874.1 |
| 73Select seq KF360067.1 | Pseudomonas sp. BSP5 16S ribosomal RNA gene, partial sequence | 1899 | 1899 | 98% | 0.0 | 99% | KF360067.1 |
| 74Select seq KF312468.1 | Pseudomonas sp. SJ7b 16S ribosomal RNA gene, partial sequence | 1899 | 1899 | 98% | 0.0 | 99% | KF312468.1 |
| 75Select seq KC428747.1 | Pseudomonas sp. X123 16S ribosomal RNA gene, partial sequence | 1899 | 1899 | 98% | 0.0 | 99% | KC428747.1 |
| 76Select seq KC246049.1 | Pseudomonas fluorescens strain IBFC2012-45 16S ribosomal RNA gene, partial sequence | 1899 | 1899 | 98% | 0.0 | 99% | KC246049.1 |
| 77Select seq HQ888871.1 | Pseudomonas fluorescens strain P-72-10 16S ribosomal RNA gene, partial sequence | 1899 | 1899 | 98% | 0.0 | 99% | HQ888871.1 |
| 78Select seq JN216880.1 | Pseudomonas sp. N-128 16S ribosomal RNA gene, partial sequence | 1899 | 1899 | 98% | 0.0 | 99% | JN216880.1 |
| 79Select seq JF901350.1 | Endophytic bacterium 41P-2 16S ribosomal RNA gene, partial sequence | 1899 | 1899 | 98% | 0.0 | 99% | JF901350.1 |
| 80Select seq JQ779065.1 | Bacterium NTL501 16S ribosomal RNA gene, partial sequence | 1899 | 1899 | 98% | 0.0 | 99% | JQ779065.1 |
| 81Select seq JQ779056.1 | Bacterium NTL264 16S ribosomal RNA gene, partial sequence | 1899 | 1899 | 98% | 0.0 | 99% | JQ779056.1 |
| 82Select seq JQ779041.1 | Bacterium NTL206 16S ribosomal RNA gene, partial sequence | 1899 | 1899 | 98% | 0.0 | 99% | JQ779041.1 |
| 83Select seq JN628030.1 | Pseudomonas brassicacearum subsp. neoaurantiaca 16S ribosomal RNA gene, partial sequence | 1899 | 1899 | 98% | 0.0 | 99% | JN628030.1 |
| 84Select seq JN033549.1 | Pseudomonas brassicacearum strain ME BHU2 16S ribosomal RNA gene, partial sequence | 1899 | 1899 | 98% | 0.0 | 99% | JN033549.1 |
| 85Select seq FR675976.1 | Pseudomonas sp. LB184 partial 16S rRNA gene, isolate LB184 | 1899 | 1899 | 98% | 0.0 | 99% | FR675976.1 |
| 86Select seq JF500978.1 | Uncultured Pseudomonas sp. clone 101 16S ribosomal RNA gene, partial sequence | 1899 | 1899 | 98% | 0.0 | 99% | JF500978.1 |
| 87Select seq JF500937.1 | Uncultured Pseudomonas sp. clone 57 16S ribosomal RNA gene, partial sequence | 1899 | 1899 | 98% | 0.0 | 99% | JF500937.1 |
| 88Select seq JF500932.1 | Uncultured Pseudomonas sp. clone 52 16S ribosomal RNA gene, partial sequence | 1899 | 1899 | 98% | 0.0 | 99% | JF500932.1 |
| 89Select seq JF500908.1 | Uncultured Pseudomonas sp. clone 24 16S ribosomal RNA gene, partial sequence | 1899 | 1899 | 98% | 0.0 | 99% | JF500908.1 |
| 90Select seq CP002585.1 | Pseudomonas brassicacearum subsp. brassicacearum NFM421, complete genome | 1899 | 9491 | 98% | 0.0 | 99% | CP002585.1 |
| 91Select seq HM854217.1 | Pseudomonas fluorescens strain STAD384 16S ribosomal RNA gene, partial sequence | 1899 | 1899 | 98% | 0.0 | 99% | HM854217.1 |
| 92Select seq HM579795.1 | Pseudomonas sp. HY4(2010) 16S ribosomal RNA gene, partial sequence | 1899 | 1899 | 98% | 0.0 | 99% | HM579795.1 |
| 93Select seq GU201849.1 | Pseudomonas brassicacearum strain Zy-2-1 16S ribosomal RNA gene, partial sequence | 1899 | 1899 | 98% | 0.0 | 99% | GU201849.1 |
| 94Select seq EU193055.1 | Uncultured Pseudomonas sp. clone Plot21-C05 16S ribosomal RNA gene, partial sequence | 1899 | 1899 | 98% | 0.0 | 99% | EU193055.1 |
| 95Select seq NR\_116299.1 | Pseudomonas brassicacearum subsp. neoaurantiaca strain CIP 109457 16S ribosomal RNA gene, partial sequence | 1899 | 1899 | 98% | 0.0 | 99% | NR\_116299.1 |
| 96Select seq EF540490.1 | Pseudomonas sp. 4\_C7/16\_5 16S ribosomal RNA gene, partial sequence | 1899 | 1899 | 98% | 0.0 | 99% | EF540490.1 |
| 97Select seq EF044357.1 | Pseudomonas sp. K94.38 16S ribosomal RNA gene, partial sequence | 1899 | 1899 | 98% | 0.0 | 99% | EF044357.1 |
| 98Select seq EF044353.1 | Pseudomonas sp. C6-11 16S ribosomal RNA gene, partial sequence | 1899 | 1899 | 98% | 0.0 | 99% | EF044353.1 |
| 99Select seq DQ453838.1 | Pseudomonas sp. P96.25 16S ribosomal RNA gene, partial sequence | 1899 | 1899 | 98% | 0.0 | 99% | DQ453838.1 |
| 100Select seq DQ453837.1 | Pseudomonas sp. K93.3 16S ribosomal RNA gene, partial sequence | 1899 | 1899 | 98% | 0.0 | 99% | DQ453837.1 |


## Alignments

Loading alignment... for sequences gi|115315703,gi|515781775,gi|442571814,gi|1137473154,gi|1015918342 Reading indexes 1-5

Download

FASTA (complete sequence)

FASTA (aligned sequences)

GenBank (complete sequence)

Continue
Cancel

GenBankGraphics

Next
Previous
Descriptions

Pseudomonas brassicacearum isolate MA250 16S ribosomal RNA gene, partial sequence

Sequence ID: DQ886486.1Length: 1515Number of Matches: 1

Related Information

Range 1: 402 to 1445GenBankGraphics

Next Match
Previous Match
First Match

Alignment statistics for match #1

| Score | Expect | Identities | Gaps | Strand | Frame |
| --- | --- | --- | --- | --- | --- |
| 1910 bits(1034) | 0.0() | 1039/1044(99%) | 0/1044(0%) | Plus/Minus |  |

Features:

```
Query  9     ACCGTCCTNNCGAAGGTTAGACTAGCTACTTCTGGTGCAACCCACTCCCATGGTGTGACG  68
             ||||||||  ||||||||||||||||||||||||||||||||||||||||||||||||||
Sbjct  1445  ACCGTCCTCCCGAAGGTTAGACTAGCTACTTCTGGTGCAACCCACTCCCATGGTGTGACG  1386

Query  69    GGCGGTGTGTACAAGGCCCGGGAACGTATTCACCGCGACATTCTGATTCGCGATTACTAG  128
             ||||||||||||||||||||||||||||||||||||||||||||||||||||||||||||
Sbjct  1385  GGCGGTGTGTACAAGGCCCGGGAACGTATTCACCGCGACATTCTGATTCGCGATTACTAG  1326

Query  129   CGATTCCGACTTCACGCAGTCGAGTTGCAGACTGCGATCCGGACTACGATCGGTTTTGTG  188
             ||||||||||||||||||||||||||||||||||||||||||||||||||||||||||||
Sbjct  1325  CGATTCCGACTTCACGCAGTCGAGTTGCAGACTGCGATCCGGACTACGATCGGTTTTGTG  1266

Query  189   GGATTAGCTCCACCTCGCGGCTTGGCAACCCTCTGTACCGACCATTGTAGCACGTGTGTA  248
             ||||||||||||||||||||||||||||||||||||||||||||||||||||||||||||
Sbjct  1265  GGATTAGCTCCACCTCGCGGCTTGGCAACCCTCTGTACCGACCATTGTAGCACGTGTGTA  1206

Query  249   GCCCAGGCCGTAAGGGCCATGATGACTTGACGTCATCCCCACCTTCCTCCGGTTTGTCAC  308
             ||||||||||||||||||||||||||||||||||||||||||||||||||||||||||||
Sbjct  1205  GCCCAGGCCGTAAGGGCCATGATGACTTGACGTCATCCCCACCTTCCTCCGGTTTGTCAC  1146

Query  309   CGGCAGTCTCCTTAGAGTGCCCACCATGACGTGCTGGTAACTAAGGACAAGGGTTGCGCT  368
             ||||||||||||||||||||||||||||||||||||||||||||||||||||||||||||
Sbjct  1145  CGGCAGTCTCCTTAGAGTGCCCACCATGACGTGCTGGTAACTAAGGACAAGGGTTGCGCT  1086

Query  369   CGTTACGGGACTTAACCCAACATCTCACGACACGAGCTGACGACAGCCATGCAGCACCTG  428
             ||||||||||||||||||||||||||||||||||||||||||||||||||||||||||||
Sbjct  1085  CGTTACGGGACTTAACCCAACATCTCACGACACGAGCTGACGACAGCCATGCAGCACCTG  1026

Query  429   TCTCAATNNNCCCGAAGGCACCAATCCATCTCTGGAAAGTTCATTGGATGTCAAGGCCTG  488
             |||||||   ||||||||||||||||||||||||||||||||||||||||||||||||||
Sbjct  1025  TCTCAATGTTCCCGAAGGCACCAATCCATCTCTGGAAAGTTCATTGGATGTCAAGGCCTG  966

Query  489   GTAAGGTTCTTCGCGTTGCTTCGAATTAAACCACATGCTCCACCGCTTGTGCGGGCCCCC  548
             ||||||||||||||||||||||||||||||||||||||||||||||||||||||||||||
Sbjct  965   GTAAGGTTCTTCGCGTTGCTTCGAATTAAACCACATGCTCCACCGCTTGTGCGGGCCCCC  906

Query  549   GTCAATTCATTTGAGTTTTAACCTTGCGGCCGTACTCCCCAGGCGGTCAACTTAATGCGT  608
             ||||||||||||||||||||||||||||||||||||||||||||||||||||||||||||
Sbjct  905   GTCAATTCATTTGAGTTTTAACCTTGCGGCCGTACTCCCCAGGCGGTCAACTTAATGCGT  846

Query  609   TAGCTGCGCCACTAAGAGCTCAAGGCTCCCAACGGCTAGTTGACATCGTTTACGGCGTGG  668
             ||||||||||||||||||||||||||||||||||||||||||||||||||||||||||||
Sbjct  845   TAGCTGCGCCACTAAGAGCTCAAGGCTCCCAACGGCTAGTTGACATCGTTTACGGCGTGG  786

Query  669   ACTACCAGGGTATCTAATCCTGTTTGCTCCCCACGCTTTCGCACCTCAGTGTCAGTATCA  728
             ||||||||||||||||||||||||||||||||||||||||||||||||||||||||||||
Sbjct  785   ACTACCAGGGTATCTAATCCTGTTTGCTCCCCACGCTTTCGCACCTCAGTGTCAGTATCA  726

Query  729   GTCCAGGTGGTCGCCTTCGCCACTGGTGTTCCTTCCTATATCTACGCATTTCACCGCTAC  788
             ||||||||||||||||||||||||||||||||||||||||||||||||||||||||||||
Sbjct  725   GTCCAGGTGGTCGCCTTCGCCACTGGTGTTCCTTCCTATATCTACGCATTTCACCGCTAC  666

Query  789   ACAGGAAATTCCACCACCCTCTACCATACTCTAGCTCGACAGTTTTGAATGCAGTTCCCA  848
             ||||||||||||||||||||||||||||||||||||||||||||||||||||||||||||
Sbjct  665   ACAGGAAATTCCACCACCCTCTACCATACTCTAGCTCGACAGTTTTGAATGCAGTTCCCA  606

Query  849   GGTTGAGCCCGGGGCTTTCACATCCAACTTAACGAACCACCTACGCGCGCTTTACGCCCA  908
             ||||||||||||||||||||||||||||||||||||||||||||||||||||||||||||
Sbjct  605   GGTTGAGCCCGGGGCTTTCACATCCAACTTAACGAACCACCTACGCGCGCTTTACGCCCA  546

Query  909   GTAATTCCGATTAACGCTTGCACCCTCTGTATTACCGCGGCTGCTGGCACAGAGTTAGCC  968
             ||||||||||||||||||||||||||||||||||||||||||||||||||||||||||||
Sbjct  545   GTAATTCCGATTAACGCTTGCACCCTCTGTATTACCGCGGCTGCTGGCACAGAGTTAGCC  486

Query  969   GGTGCTTATTCTGTCGGTAACGTCAAAACACTAACGTATTAGGTTAATGCCCTTCCTCCC  1028
             ||||||||||||||||||||||||||||||||||||||||||||||||||||||||||||
Sbjct  485   GGTGCTTATTCTGTCGGTAACGTCAAAACACTAACGTATTAGGTTAATGCCCTTCCTCCC  426

Query  1029  AACTTAAAGTGCTTTACAATCCGA  1052
             ||||||||||||||||||||||||
Sbjct  425   AACTTAAAGTGCTTTACAATCCGA  402
```

Download

FASTA (complete sequence)

FASTA (aligned sequences)

GenBank (complete sequence)

Continue
Cancel

GenBankGraphics

Next
Previous
Descriptions

Pseudomonas putida partial 16S rRNA gene, strain CFBP 4629

Sequence ID: HF545841.1Length: 1418Number of Matches: 1

Related Information

Range 1: 367 to 1414GenBankGraphics

Next Match
Previous Match
First Match

Alignment statistics for match #1

| Score | Expect | Identities | Gaps | Strand | Frame |
| --- | --- | --- | --- | --- | --- |
| 1908 bits(1033) | 0.0() | 1041/1048(99%) | 0/1048(0%) | Plus/Minus |  |

Features:

```
Query  5     TGGNACCGTCCTNNCGAAGGTTAGACTAGCTACTTCTGGTGCAACCCACTCCCATGGTGT  64
             ||| ||||||||  ||||||||||||||||||||||||||||||||||||||||||||||
Sbjct  1414  TGGTACCGTCCTCCCGAAGGTTAGACTAGCTACTTCTGGTGCAACCCACTCCCATGGTGT  1355

Query  65    GACGGGCGGTGTGTACAAGGCCCGGGAACGTATTCACCGCGACATTCTGATTCGCGATTA  124
             ||||||||||||||||||||||||||||||||||||||||||||||||||||||||||||
Sbjct  1354  GACGGGCGGTGTGTACAAGGCCCGGGAACGTATTCACCGCGACATTCTGATTCGCGATTA  1295

Query  125   CTAGCGATTCCGACTTCACGCAGTCGAGTTGCAGACTGCGATCCGGACTACGATCGGTTT  184
             ||||||||||||||||||||||||||||||||||||||||||||||||||||||||||||
Sbjct  1294  CTAGCGATTCCGACTTCACGCAGTCGAGTTGCAGACTGCGATCCGGACTACGATCGGTTT  1235

Query  185   TGTGGGATTAGCTCCACCTCGCGGCTTGGCAACCCTCTGTACCGACCATTGTAGCACGTG  244
             ||||||||||||||||||||||||||||||||||||||||||||||||||||||||||||
Sbjct  1234  TGTGGGATTAGCTCCACCTCGCGGCTTGGCAACCCTCTGTACCGACCATTGTAGCACGTG  1175

Query  245   TGTAGCCCAGGCCGTAAGGGCCATGATGACTTGACGTCATCCCCACCTTCCTCCGGTTTG  304
             ||||||||||||||||||||||||||||||||||||||||||||||||||||||||||||
Sbjct  1174  TGTAGCCCAGGCCGTAAGGGCCATGATGACTTGACGTCATCCCCACCTTCCTCCGGTTTG  1115

Query  305   TCACCGGCAGTCTCCTTAGAGTGCCCACCATGACGTGCTGGTAACTAAGGACAAGGGTTG  364
             ||||||||||||||||||||||||||||||| ||||||||||||||||||||||||||||
Sbjct  1114  TCACCGGCAGTCTCCTTAGAGTGCCCACCATAACGTGCTGGTAACTAAGGACAAGGGTTG  1055

Query  365   CGCTCGTTACGGGACTTAACCCAACATCTCACGACACGAGCTGACGACAGCCATGCAGCA  424
             ||||||||||||||||||||||||||||||||||||||||||||||||||||||||||||
Sbjct  1054  CGCTCGTTACGGGACTTAACCCAACATCTCACGACACGAGCTGACGACAGCCATGCAGCA  995

Query  425   CCTGTCTCAATNNNCCCGAAGGCACCAATCCATCTCTGGAAAGTTCATTGGATGTCAAGG  484
             |||||||||||   ||||||||||||||||||||||||||||||||||||||||||||||
Sbjct  994   CCTGTCTCAATGTTCCCGAAGGCACCAATCCATCTCTGGAAAGTTCATTGGATGTCAAGG  935

Query  485   CCTGGTAAGGTTCTTCGCGTTGCTTCGAATTAAACCACATGCTCCACCGCTTGTGCGGGC  544
             ||||||||||||||||||||||||||||||||||||||||||||||||||||||||||||
Sbjct  934   CCTGGTAAGGTTCTTCGCGTTGCTTCGAATTAAACCACATGCTCCACCGCTTGTGCGGGC  875

Query  545   CCCCGTCAATTCATTTGAGTTTTAACCTTGCGGCCGTACTCCCCAGGCGGTCAACTTAAT  604
             ||||||||||||||||||||||||||||||||||||||||||||||||||||||||||||
Sbjct  874   CCCCGTCAATTCATTTGAGTTTTAACCTTGCGGCCGTACTCCCCAGGCGGTCAACTTAAT  815

Query  605   GCGTTAGCTGCGCCACTAAGAGCTCAAGGCTCCCAACGGCTAGTTGACATCGTTTACGGC  664
             ||||||||||||||||||||||||||||||||||||||||||||||||||||||||||||
Sbjct  814   GCGTTAGCTGCGCCACTAAGAGCTCAAGGCTCCCAACGGCTAGTTGACATCGTTTACGGC  755

Query  665   GTGGACTACCAGGGTATCTAATCCTGTTTGCTCCCCACGCTTTCGCACCTCAGTGTCAGT  724
             ||||||||||||||||||||||||||||||||||||||||||||||||||||||||||||
Sbjct  754   GTGGACTACCAGGGTATCTAATCCTGTTTGCTCCCCACGCTTTCGCACCTCAGTGTCAGT  695

Query  725   ATCAGTCCAGGTGGTCGCCTTCGCCACTGGTGTTCCTTCCTATATCTACGCATTTCACCG  784
             ||||||||||||||||||||||||||||||||||||||||||||||||||||||||||||
Sbjct  694   ATCAGTCCAGGTGGTCGCCTTCGCCACTGGTGTTCCTTCCTATATCTACGCATTTCACCG  635

Query  785   CTACACAGGAAATTCCACCACCCTCTACCATACTCTAGCTCGACAGTTTTGAATGCAGTT  844
             ||||||||||||||||||||||||||||||||||||||||||||||||||||||||||||
Sbjct  634   CTACACAGGAAATTCCACCACCCTCTACCATACTCTAGCTCGACAGTTTTGAATGCAGTT  575

Query  845   CCCAGGTTGAGCCCGGGGCTTTCACATCCAACTTAACGAACCACCTACGCGCGCTTTACG  904
             ||||||||||||||||||||||||||||||||||||||||||||||||||||||||||||
Sbjct  574   CCCAGGTTGAGCCCGGGGCTTTCACATCCAACTTAACGAACCACCTACGCGCGCTTTACG  515

Query  905   CCCAGTAATTCCGATTAACGCTTGCACCCTCTGTATTACCGCGGCTGCTGGCACAGAGTT  964
             ||||||||||||||||||||||||||||||||||||||||||||||||||||||||||||
Sbjct  514   CCCAGTAATTCCGATTAACGCTTGCACCCTCTGTATTACCGCGGCTGCTGGCACAGAGTT  455

Query  965   AGCCGGTGCTTATTCTGTCGGTAACGTCAAAACACTAACGTATTAGGTTAATGCCCTTCC  1024
             ||||||||||||||||||||||||||||||||||||||||||||||||||||||||||||
Sbjct  454   AGCCGGTGCTTATTCTGTCGGTAACGTCAAAACACTAACGTATTAGGTTAATGCCCTTCC  395

Query  1025  TCCCAACTTAAAGTGCTTTACAATCCGA  1052
             ||||||||||||||||||||||||||||
Sbjct  394   TCCCAACTTAAAGTGCTTTACAATCCGA  367
```

Download

FASTA (complete sequence)

FASTA (aligned sequences)

GenBank (complete sequence)

Continue
Cancel

GenBankGraphics

Next
Previous
Descriptions

Pseudomonas sp. S-3 16S ribosomal RNA gene, partial sequence

Sequence ID: KC207086.1Length: 1444Number of Matches: 1

Related Information

Range 1: 383 to 1430GenBankGraphics

Next Match
Previous Match
First Match

Alignment statistics for match #1

| Score | Expect | Identities | Gaps | Strand | Frame |
| --- | --- | --- | --- | --- | --- |
| 1908 bits(1033) | 0.0() | 1041/1048(99%) | 0/1048(0%) | Plus/Minus |  |

Features:

```
Query  5     TGGNACCGTCCTNNCGAAGGTTAGACTAGCTACTTCTGGTGCAACCCACTCCCATGGTGT  64
             ||| |||||||   ||||||||||||||||||||||||||||||||||||||||||||||
Sbjct  1430  TGGTACCGTCCCCCCGAAGGTTAGACTAGCTACTTCTGGTGCAACCCACTCCCATGGTGT  1371

Query  65    GACGGGCGGTGTGTACAAGGCCCGGGAACGTATTCACCGCGACATTCTGATTCGCGATTA  124
             ||||||||||||||||||||||||||||||||||||||||||||||||||||||||||||
Sbjct  1370  GACGGGCGGTGTGTACAAGGCCCGGGAACGTATTCACCGCGACATTCTGATTCGCGATTA  1311

Query  125   CTAGCGATTCCGACTTCACGCAGTCGAGTTGCAGACTGCGATCCGGACTACGATCGGTTT  184
             ||||||||||||||||||||||||||||||||||||||||||||||||||||||||||||
Sbjct  1310  CTAGCGATTCCGACTTCACGCAGTCGAGTTGCAGACTGCGATCCGGACTACGATCGGTTT  1251

Query  185   TGTGGGATTAGCTCCACCTCGCGGCTTGGCAACCCTCTGTACCGACCATTGTAGCACGTG  244
             ||||||||||||||||||||||||||||||||||||||||||||||||||||||||||||
Sbjct  1250  TGTGGGATTAGCTCCACCTCGCGGCTTGGCAACCCTCTGTACCGACCATTGTAGCACGTG  1191

Query  245   TGTAGCCCAGGCCGTAAGGGCCATGATGACTTGACGTCATCCCCACCTTCCTCCGGTTTG  304
             ||||||||||||||||||||||||||||||||||||||||||||||||||||||||||||
Sbjct  1190  TGTAGCCCAGGCCGTAAGGGCCATGATGACTTGACGTCATCCCCACCTTCCTCCGGTTTG  1131

Query  305   TCACCGGCAGTCTCCTTAGAGTGCCCACCATGACGTGCTGGTAACTAAGGACAAGGGTTG  364
             ||||||||||||||||||||||||||||||||||||||||||||||||||||||||||||
Sbjct  1130  TCACCGGCAGTCTCCTTAGAGTGCCCACCATGACGTGCTGGTAACTAAGGACAAGGGTTG  1071

Query  365   CGCTCGTTACGGGACTTAACCCAACATCTCACGACACGAGCTGACGACAGCCATGCAGCA  424
             ||||||||||||||||||||||||||||||||||||||||||||||||||||||||||||
Sbjct  1070  CGCTCGTTACGGGACTTAACCCAACATCTCACGACACGAGCTGACGACAGCCATGCAGCA  1011

Query  425   CCTGTCTCAATNNNCCCGAAGGCACCAATCCATCTCTGGAAAGTTCATTGGATGTCAAGG  484
             |||||||||||   ||||||||||||||||||||||||||||||||||||||||||||||
Sbjct  1010  CCTGTCTCAATGCTCCCGAAGGCACCAATCCATCTCTGGAAAGTTCATTGGATGTCAAGG  951

Query  485   CCTGGTAAGGTTCTTCGCGTTGCTTCGAATTAAACCACATGCTCCACCGCTTGTGCGGGC  544
             ||||||||||||||||||||||||||||||||||||||||||||||||||||||||||||
Sbjct  950   CCTGGTAAGGTTCTTCGCGTTGCTTCGAATTAAACCACATGCTCCACCGCTTGTGCGGGC  891

Query  545   CCCCGTCAATTCATTTGAGTTTTAACCTTGCGGCCGTACTCCCCAGGCGGTCAACTTAAT  604
             ||||||||||||||||||||||||||||||||||||||||||||||||||||||||||||
Sbjct  890   CCCCGTCAATTCATTTGAGTTTTAACCTTGCGGCCGTACTCCCCAGGCGGTCAACTTAAT  831

Query  605   GCGTTAGCTGCGCCACTAAGAGCTCAAGGCTCCCAACGGCTAGTTGACATCGTTTACGGC  664
             ||||||||||||||||||||||||||||||||||||||||||||||||||||||||||||
Sbjct  830   GCGTTAGCTGCGCCACTAAGAGCTCAAGGCTCCCAACGGCTAGTTGACATCGTTTACGGC  771

Query  665   GTGGACTACCAGGGTATCTAATCCTGTTTGCTCCCCACGCTTTCGCACCTCAGTGTCAGT  724
             ||||||||||||||||||||||||||||||||||||||||||||||||||||||||||||
Sbjct  770   GTGGACTACCAGGGTATCTAATCCTGTTTGCTCCCCACGCTTTCGCACCTCAGTGTCAGT  711

Query  725   ATCAGTCCAGGTGGTCGCCTTCGCCACTGGTGTTCCTTCCTATATCTACGCATTTCACCG  784
             ||||||||||||||||||||||||||||||||||||||||||||||||||||||||||||
Sbjct  710   ATCAGTCCAGGTGGTCGCCTTCGCCACTGGTGTTCCTTCCTATATCTACGCATTTCACCG  651

Query  785   CTACACAGGAAATTCCACCACCCTCTACCATACTCTAGCTCGACAGTTTTGAATGCAGTT  844
             ||||||||||||||||||||||||||||||||||||||||||||||||||||||||||||
Sbjct  650   CTACACAGGAAATTCCACCACCCTCTACCATACTCTAGCTCGACAGTTTTGAATGCAGTT  591

Query  845   CCCAGGTTGAGCCCGGGGCTTTCACATCCAACTTAACGAACCACCTACGCGCGCTTTACG  904
             ||||||||||||||||||||||||||||||||||||||||||||||||||||||||||||
Sbjct  590   CCCAGGTTGAGCCCGGGGCTTTCACATCCAACTTAACGAACCACCTACGCGCGCTTTACG  531

Query  905   CCCAGTAATTCCGATTAACGCTTGCACCCTCTGTATTACCGCGGCTGCTGGCACAGAGTT  964
             ||||||||||||||||||||||||||||||||||||||||||||||||||||||||||||
Sbjct  530   CCCAGTAATTCCGATTAACGCTTGCACCCTCTGTATTACCGCGGCTGCTGGCACAGAGTT  471

Query  965   AGCCGGTGCTTATTCTGTCGGTAACGTCAAAACACTAACGTATTAGGTTAATGCCCTTCC  1024
             ||||||||||||||||||||||||||||||||||||||||||||||||||||||||||||
Sbjct  470   AGCCGGTGCTTATTCTGTCGGTAACGTCAAAACACTAACGTATTAGGTTAATGCCCTTCC  411

Query  1025  TCCCAACTTAAAGTGCTTTACAATCCGA  1052
             ||||||||||||||||||||||||||||
Sbjct  410   TCCCAACTTAAAGTGCTTTACAATCCGA  383
```

Download

FASTA (complete sequence)

FASTA (aligned sequences)

GenBank (complete sequence)

Continue
Cancel

GenBankGraphics

Next
Previous
Descriptions

Pseudomonas sp. strain 7.3 16S ribosomal RNA gene, partial sequence

Sequence ID: KY542120.1Length: 1546Number of Matches: 1

Related Information

Range 1: 415 to 1458GenBankGraphics

Next Match
Previous Match
First Match

Alignment statistics for match #1

| Score | Expect | Identities | Gaps | Strand | Frame |
| --- | --- | --- | --- | --- | --- |
| 1905 bits(1031) | 0.0() | 1038/1044(99%) | 0/1044(0%) | Plus/Minus |  |

Features:

```
Query  9     ACCGTCCTNNCGAAGGTTAGACTAGCTACTTCTGGTGCAACCCACTCCCATGGTGTGACG  68
             ||||||||  ||||||||||||||||||||||||||||||||||||||||||||||||||
Sbjct  1458  ACCGTCCTCCCGAAGGTTAGACTAGCTACTTCTGGTGCAACCCACTCCCATGGTGTGACG  1399

Query  69    GGCGGTGTGTACAAGGCCCGGGAACGTATTCACCGCGACATTCTGATTCGCGATTACTAG  128
             ||||||||||||||||||||||||||||||||||||||||||||||||||||||||||||
Sbjct  1398  GGCGGTGTGTACAAGGCCCGGGAACGTATTCACCGCGACATTCTGATTCGCGATTACTAG  1339

Query  129   CGATTCCGACTTCACGCAGTCGAGTTGCAGACTGCGATCCGGACTACGATCGGTTTTGTG  188
             ||||||||||||||||||||||||||||||||||||||||||||||||||||||||||||
Sbjct  1338  CGATTCCGACTTCACGCAGTCGAGTTGCAGACTGCGATCCGGACTACGATCGGTTTTGTG  1279

Query  189   GGATTAGCTCCACCTCGCGGCTTGGCAACCCTCTGTACCGACCATTGTAGCACGTGTGTA  248
             ||||||||||||||||||||||||||||||||||||||||||||||||||||||||||||
Sbjct  1278  GGATTAGCTCCACCTCGCGGCTTGGCAACCCTCTGTACCGACCATTGTAGCACGTGTGTA  1219

Query  249   GCCCAGGCCGTAAGGGCCATGATGACTTGACGTCATCCCCACCTTCCTCCGGTTTGTCAC  308
             ||||||||||||||||||||||||||||||||||||||||||||||||||||||||||||
Sbjct  1218  GCCCAGGCCGTAAGGGCCATGATGACTTGACGTCATCCCCACCTTCCTCCGGTTTGTCAC  1159

Query  309   CGGCAGTCTCCTTAGAGTGCCCACCATGACGTGCTGGTAACTAAGGACAAGGGTTGCGCT  368
             ||||||||||||||||||||||||||| ||||||||||||||||||||||||||||||||
Sbjct  1158  CGGCAGTCTCCTTAGAGTGCCCACCATAACGTGCTGGTAACTAAGGACAAGGGTTGCGCT  1099

Query  369   CGTTACGGGACTTAACCCAACATCTCACGACACGAGCTGACGACAGCCATGCAGCACCTG  428
             ||||||||||||||||||||||||||||||||||||||||||||||||||||||||||||
Sbjct  1098  CGTTACGGGACTTAACCCAACATCTCACGACACGAGCTGACGACAGCCATGCAGCACCTG  1039

Query  429   TCTCAATNNNCCCGAAGGCACCAATCCATCTCTGGAAAGTTCATTGGATGTCAAGGCCTG  488
             |||||||   ||||||||||||||||||||||||||||||||||||||||||||||||||
Sbjct  1038  TCTCAATGTTCCCGAAGGCACCAATCCATCTCTGGAAAGTTCATTGGATGTCAAGGCCTG  979

Query  489   GTAAGGTTCTTCGCGTTGCTTCGAATTAAACCACATGCTCCACCGCTTGTGCGGGCCCCC  548
             ||||||||||||||||||||||||||||||||||||||||||||||||||||||||||||
Sbjct  978   GTAAGGTTCTTCGCGTTGCTTCGAATTAAACCACATGCTCCACCGCTTGTGCGGGCCCCC  919

Query  549   GTCAATTCATTTGAGTTTTAACCTTGCGGCCGTACTCCCCAGGCGGTCAACTTAATGCGT  608
             ||||||||||||||||||||||||||||||||||||||||||||||||||||||||||||
Sbjct  918   GTCAATTCATTTGAGTTTTAACCTTGCGGCCGTACTCCCCAGGCGGTCAACTTAATGCGT  859

Query  609   TAGCTGCGCCACTAAGAGCTCAAGGCTCCCAACGGCTAGTTGACATCGTTTACGGCGTGG  668
             ||||||||||||||||||||||||||||||||||||||||||||||||||||||||||||
Sbjct  858   TAGCTGCGCCACTAAGAGCTCAAGGCTCCCAACGGCTAGTTGACATCGTTTACGGCGTGG  799

Query  669   ACTACCAGGGTATCTAATCCTGTTTGCTCCCCACGCTTTCGCACCTCAGTGTCAGTATCA  728
             ||||||||||||||||||||||||||||||||||||||||||||||||||||||||||||
Sbjct  798   ACTACCAGGGTATCTAATCCTGTTTGCTCCCCACGCTTTCGCACCTCAGTGTCAGTATCA  739

Query  729   GTCCAGGTGGTCGCCTTCGCCACTGGTGTTCCTTCCTATATCTACGCATTTCACCGCTAC  788
             ||||||||||||||||||||||||||||||||||||||||||||||||||||||||||||
Sbjct  738   GTCCAGGTGGTCGCCTTCGCCACTGGTGTTCCTTCCTATATCTACGCATTTCACCGCTAC  679

Query  789   ACAGGAAATTCCACCACCCTCTACCATACTCTAGCTCGACAGTTTTGAATGCAGTTCCCA  848
             ||||||||||||||||||||||||||||||||||||||||||||||||||||||||||||
Sbjct  678   ACAGGAAATTCCACCACCCTCTACCATACTCTAGCTCGACAGTTTTGAATGCAGTTCCCA  619

Query  849   GGTTGAGCCCGGGGCTTTCACATCCAACTTAACGAACCACCTACGCGCGCTTTACGCCCA  908
             ||||||||||||||||||||||||||||||||||||||||||||||||||||||||||||
Sbjct  618   GGTTGAGCCCGGGGCTTTCACATCCAACTTAACGAACCACCTACGCGCGCTTTACGCCCA  559

Query  909   GTAATTCCGATTAACGCTTGCACCCTCTGTATTACCGCGGCTGCTGGCACAGAGTTAGCC  968
             ||||||||||||||||||||||||||||||||||||||||||||||||||||||||||||
Sbjct  558   GTAATTCCGATTAACGCTTGCACCCTCTGTATTACCGCGGCTGCTGGCACAGAGTTAGCC  499

Query  969   GGTGCTTATTCTGTCGGTAACGTCAAAACACTAACGTATTAGGTTAATGCCCTTCCTCCC  1028
             ||||||||||||||||||||||||||||||||||||||||||||||||||||||||||||
Sbjct  498   GGTGCTTATTCTGTCGGTAACGTCAAAACACTAACGTATTAGGTTAATGCCCTTCCTCCC  439

Query  1029  AACTTAAAGTGCTTTACAATCCGA  1052
             ||||||||||||||||||||||||
Sbjct  438   AACTTAAAGTGCTTTACAATCCGA  415
```

Download

FASTA (complete sequence)

FASTA (aligned sequences)

GenBank (complete sequence)

Continue
Cancel

GenBankGraphics

Next
Previous
Descriptions

Pseudomonas fluorescens strain SBR10 16S ribosomal RNA gene, partial sequence

Sequence ID: KX018310.1Length: 1486Number of Matches: 1

Related Information

Range 1: 419 to 1462GenBankGraphics

Next Match
Previous Match
First Match

Alignment statistics for match #1

| Score | Expect | Identities | Gaps | Strand | Frame |
| --- | --- | --- | --- | --- | --- |
| 1905 bits(1031) | 0.0() | 1039/1044(99%) | 0/1044(0%) | Plus/Minus |  |

Features:

```
Query  9     ACCGTCCTNNCGAAGGTTAGACTAGCTACTTCTGGTGCAACCCACTCCCATGGTGTGACG  68
             ||||||||  ||||||||||||||||||||||||||||||||||||||||||||||||||
Sbjct  1462  ACCGTCCTCCCGAAGGTTAGACTAGCTACTTCTGGTGCAACCCACTCCCATGGTGTGACG  1403

Query  69    GGCGGTGTGTACAAGGCCCGGGAACGTATTCACCGCGACATTCTGATTCGCGATTACTAG  128
             ||||||||||||||||||||||||||||||||||||||||||||||||||||||||||||
Sbjct  1402  GGCGGTGTGTACAAGGCCCGGGAACGTATTCACCGCGACATTCTGATTCGCGATTACTAG  1343

Query  129   CGATTCCGACTTCACGCAGTCGAGTTGCAGACTGCGATCCGGACTACGATCGGTTTTGTG  188
             ||||||||||||||||||||||||||||||||||||||||||||||||||||||||||||
Sbjct  1342  CGATTCCGACTTCACGCAGTCGAGTTGCAGACTGCGATCCGGACTACGATCGGTTTTGTG  1283

Query  189   GGATTAGCTCCACCTCGCGGCTTGGCAACCCTCTGTACCGACCATTGTAGCACGTGTGTA  248
             ||||||||||||||||||||||||||||||||||||||||||||||||||||||||||||
Sbjct  1282  GGATTAGCTCCACCTCGCGGCTTGGCAACCCTCTGTACCGACCATTGTAGCACGTGTGTA  1223

Query  249   GCCCAGGCCGTAAGGGCCATGATGACTTGACGTCATCCCCACCTTCCTCCGGTTTGTCAC  308
             ||||||||||||||||||||||||||||||||||||||||||||||||||||||||||||
Sbjct  1222  GCCCAGGCCGTAAGGGCCATGATGACTTGACGTCATCCCCACCTTCCTCCGGTTTGTCAC  1163

Query  309   CGGCAGTCTCCTTAGAGTGCCCACCATGACGTGCTGGTAACTAAGGACAAGGGTTGCGCT  368
             ||||||||||||||||||||||||||| ||||||||||||||||||||||||||||||||
Sbjct  1162  CGGCAGTCTCCTTAGAGTGCCCACCATAACGTGCTGGTAACTAAGGACAAGGGTTGCGCT  1103

Query  369   CGTTACGGGACTTAACCCAACATCTCACGACACGAGCTGACGACAGCCATGCAGCACCTG  428
             ||||||||||||||||||||||||||||||||||||||||||||||||||||||||||||
Sbjct  1102  CGTTACGGGACTTAACCCAACATCTCACGACACGAGCTGACGACAGCCATGCAGCACCTG  1043

Query  429   TCTCAATNNNCCCGAAGGCACCAATCCATCTCTGGAAAGTTCATTGGATGTCAAGGCCTG  488
             ||||||| | ||||||||||||||||||||||||||||||||||||||||||||||||||
Sbjct  1042  TCTCAATGNTCCCGAAGGCACCAATCCATCTCTGGAAAGTTCATTGGATGTCAAGGCCTG  983

Query  489   GTAAGGTTCTTCGCGTTGCTTCGAATTAAACCACATGCTCCACCGCTTGTGCGGGCCCCC  548
             ||||||||||||||||||||||||||||||||||||||||||||||||||||||||||||
Sbjct  982   GTAAGGTTCTTCGCGTTGCTTCGAATTAAACCACATGCTCCACCGCTTGTGCGGGCCCCC  923

Query  549   GTCAATTCATTTGAGTTTTAACCTTGCGGCCGTACTCCCCAGGCGGTCAACTTAATGCGT  608
             ||||||||||||||||||||||||||||||||||||||||||||||||||||||||||||
Sbjct  922   GTCAATTCATTTGAGTTTTAACCTTGCGGCCGTACTCCCCAGGCGGTCAACTTAATGCGT  863

Query  609   TAGCTGCGCCACTAAGAGCTCAAGGCTCCCAACGGCTAGTTGACATCGTTTACGGCGTGG  668
             ||||||||||||||||||||||||||||||||||||||||||||||||||||||||||||
Sbjct  862   TAGCTGCGCCACTAAGAGCTCAAGGCTCCCAACGGCTAGTTGACATCGTTTACGGCGTGG  803

Query  669   ACTACCAGGGTATCTAATCCTGTTTGCTCCCCACGCTTTCGCACCTCAGTGTCAGTATCA  728
             ||||||||||||||||||||||||||||||||||||||||||||||||||||||||||||
Sbjct  802   ACTACCAGGGTATCTAATCCTGTTTGCTCCCCACGCTTTCGCACCTCAGTGTCAGTATCA  743

Query  729   GTCCAGGTGGTCGCCTTCGCCACTGGTGTTCCTTCCTATATCTACGCATTTCACCGCTAC  788
             ||||||||||||||||||||||||||||||||||||||||||||||||||||||||||||
Sbjct  742   GTCCAGGTGGTCGCCTTCGCCACTGGTGTTCCTTCCTATATCTACGCATTTCACCGCTAC  683

Query  789   ACAGGAAATTCCACCACCCTCTACCATACTCTAGCTCGACAGTTTTGAATGCAGTTCCCA  848
             ||||||||||||||||||||||||||||||||||||||||||||||||||||||||||||
Sbjct  682   ACAGGAAATTCCACCACCCTCTACCATACTCTAGCTCGACAGTTTTGAATGCAGTTCCCA  623

Query  849   GGTTGAGCCCGGGGCTTTCACATCCAACTTAACGAACCACCTACGCGCGCTTTACGCCCA  908
             ||||||||||||||||||||||||||||||||||||||||||||||||||||||||||||
Sbjct  622   GGTTGAGCCCGGGGCTTTCACATCCAACTTAACGAACCACCTACGCGCGCTTTACGCCCA  563

Query  909   GTAATTCCGATTAACGCTTGCACCCTCTGTATTACCGCGGCTGCTGGCACAGAGTTAGCC  968
             ||||||||||||||||||||||||||||||||||||||||||||||||||||||||||||
Sbjct  562   GTAATTCCGATTAACGCTTGCACCCTCTGTATTACCGCGGCTGCTGGCACAGAGTTAGCC  503

Query  969   GGTGCTTATTCTGTCGGTAACGTCAAAACACTAACGTATTAGGTTAATGCCCTTCCTCCC  1028
             ||||||||||||||||||||||||||||||||||||||||||||||||||||||||||||
Sbjct  502   GGTGCTTATTCTGTCGGTAACGTCAAAACACTAACGTATTAGGTTAATGCCCTTCCTCCC  443

Query  1029  AACTTAAAGTGCTTTACAATCCGA  1052
             ||||||||||||||||||||||||
Sbjct  442   AACTTAAAGTGCTTTACAATCCGA  419
```

```

```


BLAST is a registered trademark of the National Library of Medicine

Support center
Mailing list


YouTube

- National Library Of Medicine
- National Institutes Of Health
- U.S. Department of Health & Human Services
- USA.gov

### NCBI


National Center for Biotechnology Information,
 U.S. National Library of Medicine

8600 Rockville Pike,
Bethesda
 MD,
20894
USA

Policies and Guidelines
|
Contact


PreferencesTurn off

External link. Please review our privacy policy.
