# Supplementary material for: Isothermal microcalorimetry for thermal viable count of microorganisms in pure cultures and stabilized formulations
Source: BMC Microbiol. 2019 Mar 21;19:65. doi: 10.1186/s12866-019-1432-8 (PMC6429831; doi:10.1186/s12866-019-1432-8)
Supplement: Supplementary file 3 — 16S rRNA gene sequences’ BLASTN hits in zipped HTML format. (ZIP 15810 kb) [file 12866_2019_1432_MOESM3_ESM.zip › Best blastn hits/NCBI Blast_45 small negative F -- 12..1036 of sequence.html]

NCBI Blast:45 small negative F -- 12..1036 of sequence


- NCBI Home
- Sign in to NCBI
- Skip to Main Content
- Skip to Navigation
- About NCBI Accesskeys

U.S. National Library of Medicine

NCBI
National Center for Biotechnology Information

- My NCBI
- Sign in to NCBI
- Register
- Sign Out

BLAST ® » blastn suite » RID-A4W61CRA014


- Home
- Recent Results
- Saved Strategies
- Help

BLAST Results


Edit and Resubmit
Save Search Strategies
[Sign in above to save your search strategy]

Formatting options 


Download


How to read this page
Blast report description
Questions/comments


|  |  |
| --- | --- |
| Formatting options | |
| Show | Alignment as  HTML Plain text   Old View Reset form to defaults [?]  These options control formatting of alignments in results pages. The default is HTML, but other formats (including plain text) are available. PSSM and PssmWithParameters are representations of Position Specific Scoring Matrices and are only available for PSI-BLAST. The Advanced view option allows the database descriptions to be sorted by various indices in a table. |
| Alignment View | Pairwise Pairwise with dots for identities Query-anchored with dots for identities Query-anchored with letters for identities Flat query-anchored with dots for identities Flat query-anchored with letters for identities [?]  Choose how to view alignments. The default "pairwise" view shows how each subject sequence aligns individually to the query sequence. The "query-anchored" view shows how all subject sequences align to the query sequence. For each view type, you can choose to show "identities" (matching residues) as letters or dots. more... |
| Display | Graphical Overview   Linkout   Sequence Retrieval  NCBI-gi   CDS feature [?]  - Graphical Overview: Graphical Overview: Show graph of similar sequence regions aligned to query.   more... - NCBI-gi: Show NCBI gi identifiers. - CDS feature: Show annotated coding region and translation.   more... |
| Masking | Character:   X for protein, n for nucleotide Lower Case  Color:  Black Grey Red [?]  - Masking Character: Display masked (filtered) sequence regions as lower-case or as specific letters (N for nucleotide, P for protein). - Masking Color: Display masked sequence regions in the given color. |
| Limit results | Descriptions:  10 50 100 Graphical overview:  0 10 50 100  Alignments:  0 10 50 100 Line length:  60 90 120 150 [?]  - Descriptions: Show short descriptions for up to the given number of sequences. - Alignments: Show alignments for up to the given number of sequences, in order of statistical significance. - Line lenghth: Number of letters to show on one line in an alignment. |
|  | Organism Type common name, binomial, taxid, or group name. Only 20 top taxa will be shown.     Exclude    [?]  Show only sequences from the given organism. |
|  | Entrez query:  [?]  Show only those sequences that match the given Entrez query. more... |
|  | Expect Min:  Expect Max:  [?]  Show only sequences with expect values in the given range. more... |
|  | Percent Identity Min:  Percent Identity Max:  [?]  Show only sequences with percent identity values in the given range. |
| Format for | PSI-BLAST with inclusion threshold:  [?]  - Format for PSI-BLAST: The Position-Specific Iterated BLAST (PSI-BLAST) program performs iterative searches with a protein query,   in which sequences found in one round of search are used to build a custom score model for the next round.   more... - Inclusion Threshold: This sets the statistical significance threshold for including a sequence in the model used   by PSI-BLAST to create the PSSM on the next iteration. |

|  |  |  |  |  |  |
| --- | --- | --- | --- | --- | --- |
| Download | | | | | |
| Alignment  Text XML ASN.1 JSON Seq-align Hit Table(text) Hit Table(csv) Multiple-file XML2 Single-file XML2 Multiple-file JSON Single-file JSON SAM | Search Strategies  ASN.1 | PSSM to restart search  PSSM | [?] |

The Download link provides BLAST output that may be used as input to another program.
This includes parseable formats such as the tabular report or XML as well as the Search Strategy files read by the BLAST+ applications.
More details on the parseable (XML, tabular, and ASN.1) reports can be found at
https://www.ncbi.nlm.nih.gov/books/NBK153387/  
  

The following formats are offered under the Alignment section:  
1). "Text". Non-HTML standard BLAST report.  
2). "XML". XML report based upon the DTD at https://www.ncbi.nlm.nih.gov/data\_specs/dtd/NCBI\_BlastOutput.dtd  
3). "ASN.1". Alignment written out in Abstract Syntax Notation 1.  
4). "JSON Seq-align". Alignment written out in JSON.  
4). "Hit Table(text)". The tabular report as text.  
5). "Hit Table(csv)". The tabular report ready for import into spread-sheet programs like Excel.  
6). "XML2". New XML format described at ftp://ftp.ncbi.nlm.nih.gov/blast/documents/NEWXML/xml2.pdf.  
7). "JSON". New JSON format described at ftp://ftp.ncbi.nlm.nih.gov/blast/documents/NEWXML/xml2.pdf.  
8). "SAM". Sequence Alignment Map format.

XML2 and JSON can be downloaded either as one file per query (multiple-file) or one file for all queries (single-file). These formats are listed as Multiple-file XML2 (and JSON) or Single-file XML (and JSON).

The following report is offered under the Search Strategy section:  
1). "ASN.1" Search Strategy. A record of the parameters, query, and database used in the search. This file can be used to start a stand-alone BLAST search, see
https://www.ncbi.nlm.nih.gov/books/NBK1763/#CmdLineAppsManual.I455\_BLAST\_search\_stra


# Job title: 45 small negative F -- 12..1036 of sequence

Results for:

lcl|Query\_56797 45 small negative F -- 12..1036 of sequence(1025bp)
[?]

Your BLAST job specified more than one input sequence.
This box lets you choose which input sequence to show BLAST results for.

RID
:   A4W61CRA014 (Expires on 03-10 18:32 pm)

Query ID
:   lcl|Query\_56797
:   lcl|Query\_56797

Description
:   45 small negative F -- 12..1036 of sequence

Molecule type
:   nucleic acid

Query Length
:   1025

Database Name
:   nr

Description
:   Nucleotide collection (nt) See details

Program
:   BLASTN 2.8.0+ Citation

  

Reference 

Zheng Zhang, Scott Schwartz, Lukas Wagner, and Webb Miller (2000), "A greedy algorithm for aligning DNA sequences", J Comput Biol 2000; 7(1-2):203-14.

Reference - database indexing

Aleksandr Morgulis, George Coulouris, Yan Raytselis, Thomas L. Madden, Richa Agarwala, Alejandro A. Schäffer (2008), "Database Indexing for Production MegaBLAST Searches", Bioinformatics 24:1757-1764.

Other reports:
Search Summary

[Taxonomy reports]
[Distance tree of results]
[MSA viewer]

Search Parameters

| Search parameter name | Search parameter value |
| --- | --- |
| Program | blastn |
| Word size | 28 |
| Expect value | 10 |
| Hitlist size | 100 |
| Match/Mismatch scores | 1,-2 |
| Gapcosts | 0,2.5 |
| Low Complexity Filter | Yes |
| Filter string | L;m; |
| Genetic Code | 1 |

Database

| Database parameter name | Database parameter value |
| --- | --- |
| Posted date | Mar 3, 2018 9:32 PM |
| Number of letters | 171,598,911,005 |
| Number of sequences | 46,870,701 |
| Entrez query | Includes:  Excludes:  None |

Karlin-Altschul statistics

| Params | Ungapped | Gapped |
| --- | --- | --- |
| Lambda | 1.33271 | 1.28 |
| K | 0.620991 | 0.46 |
| H | 1.12409 | 0.85 |

Results Statistics

| Results Statistics parameter name | Results Statistics parameter value |
| --- | --- |
| Length adjustment | 35 |
| Effective length of query | 990 |
| Effective length of database | 169958436470 |
| Effective search space | 168258852105300 |
| Effective search space used | 168258852105300 |


## Graphic Summary

### Distribution of the top 100 Blast Hits on 100 subject sequences [?]

The graphic is an overview of the database sequences aligned to the query sequence. These are represented horizontal bars colored coded by score and showing the extent
of the alignment on the query sequence. Separate aligned regions on the same database sequence are connected by a thin grey line.
Mousing over an alignment shows the database sequence title. Clicking an alignment displays a box with more details about the alignment and
link to the sequence alignment itself in the Alignments section of the report.

Mouse over to see the title, click to show alignments

Color key for alignment scores

<40

40-50

50-80

80-200

>=200

Query

1

200

400

600

800

1000

Pseudomonas graminis strain A3 16S ribosomal RNA gene, ..

Score:1875 Evalue:0

Accession:KU587965.1

Alignment

Pseudomonas sp. PDD-59b-17 16S ribosomal RNA gene, comp..

Score:1875 Evalue:0

Accession:KR922154.1

Alignment

Pseudomonas graminis partial 16S rRNA gene, strain DSM ..

Score:1875 Evalue:0

Accession:LN551925.1

Alignment

Pseudomonas graminis strain SR12 16S ribosomal RNA gene..

Score:1875 Evalue:0

Accession:KJ529045.1

Alignment

Pseudomonas sp. TA\_GU partial 16S rRNA gene, strain TA\_..

Score:1875 Evalue:0

Accession:HG942149.1

Alignment

Pseudomonas graminis strain R5SpM3P2C1 16S ribosomal RN..

Score:1875 Evalue:0

Accession:KF147092.1

Alignment

Pseudomonas sp. Bza15 16S ribosomal RNA gene, partial s..

Score:1875 Evalue:0

Accession:JQ977250.1

Alignment

Pseudomonas graminis strain SL11 16S ribosomal RNA gene..

Score:1873 Evalue:0

Accession:KJ529030.1

Alignment

Bacterium enrichment culture clone SaB5 16S ribosomal R..

Score:1871 Evalue:0

Accession:JQ727450.1

Alignment

Pseudomonas graminis strain AN1 16S ribosomal RNA gene,..

Score:1869 Evalue:0

Accession:MG561813.1

Alignment

Pseudomonas sp. strain MSM-2-10-15 16S ribosomal RNA ge..

Score:1869 Evalue:0

Accession:KY907017.1

Alignment

Pseudomonas sp. CCUG 63225 partial 16S rRNA gene, strai..

Score:1869 Evalue:0

Accession:LT601010.1

Alignment

Pseudomonas graminis 16S ribosomal RNA gene, partial se..

Score:1869 Evalue:0

Accession:KU523561.1

Alignment

Pseudomonas sp. PDD-59b-53 16S ribosomal RNA gene, comp..

Score:1869 Evalue:0

Accession:KR922175.1

Alignment

Pseudomonas graminis strain PDD-59b-25 16S ribosomal RN..

Score:1869 Evalue:0

Accession:KR922157.1

Alignment

Pseudomonas sp. HP7D 16S ribosomal RNA gene, partial se..

Score:1869 Evalue:0

Accession:KM187516.1

Alignment

Pseudomonas sp. HP3I 16S ribosomal RNA gene, partial se..

Score:1869 Evalue:0

Accession:KM187449.1

Alignment

Pseudomonas graminis strain EB25 16S ribosomal RNA gene..

Score:1869 Evalue:0

Accession:KP209394.1

Alignment

Pseudomonas sp. PAMC26590 16S ribosomal RNA gene, parti..

Score:1869 Evalue:0

Accession:KJ606806.1

Alignment

Bacterium 3t 16S ribosomal RNA gene, partial sequence

Score:1869 Evalue:0

Accession:JX042421.1

Alignment

Pseudomonas sp. R1SsM3P3C4 16S ribosomal RNA gene, part..

Score:1869 Evalue:0

Accession:KF147102.1

Alignment

Pseudomonas sp. R1SpM3P3C2 16S ribosomal RNA gene, part..

Score:1869 Evalue:0

Accession:KF147101.1

Alignment

Pseudomonas sp. R1SsM3P2C4 16S ribosomal RNA gene, part..

Score:1869 Evalue:0

Accession:KF147100.1

Alignment

Pseudomonas sp. R1SpM3P2C4 16S ribosomal RNA gene, part..

Score:1869 Evalue:0

Accession:KF147099.1

Alignment

Pseudomonas sp. R5SsM3P3C9 16S ribosomal RNA gene, part..

Score:1869 Evalue:0

Accession:KF147098.1

Alignment

Pseudomonas sp. R2SpM3P2C5 16S ribosomal RNA gene, part..

Score:1869 Evalue:0

Accession:KF147097.1

Alignment

Pseudomonas graminis strain R2SsM3P1C4 16S ribosomal RN..

Score:1869 Evalue:0

Accession:KF147094.1

Alignment

Pseudomonas graminis strain R2SsM3P1C3 16S ribosomal RN..

Score:1869 Evalue:0

Accession:KF147093.1

Alignment

Pseudomonas graminis strain R5SsM3P2C7 16S ribosomal RN..

Score:1869 Evalue:0

Accession:KF147091.1

Alignment

Pseudomonas graminis strain R1SpM3P3C4 16S ribosomal RN..

Score:1869 Evalue:0

Accession:KF147090.1

Alignment

Pseudomonas graminis strain R1SpM3P1C6 16S ribosomal RN..

Score:1869 Evalue:0

Accession:KF147089.1

Alignment

Pseudomonas sp. Axa8 16S ribosomal RNA gene, partial se..

Score:1869 Evalue:0

Accession:JQ977394.1

Alignment

Pseudomonas sp. E9 partial 16S rRNA gene, isolate E9

Score:1869 Evalue:0

Accession:HE652089.1

Alignment

Pseudomonas graminis isolate PDD-32b-60 16S ribosomal R..

Score:1869 Evalue:0

Accession:HQ256858.1

Alignment

Bacterium OC34(2011) 16S ribosomal RNA gene, partial se..

Score:1869 Evalue:0

Accession:HQ179006.1

Alignment

Uncultured bacterium clone nby238d11c1 16S ribosomal RN..

Score:1869 Evalue:0

Accession:HM811719.1

Alignment

Pseudomonas graminis strain RMPP6 16S ribosomal RNA gen..

Score:1869 Evalue:0

Accession:GU396281.1

Alignment

Uncultured bacterium partial 16S rRNA gene, clone 3\_E03

Score:1869 Evalue:0

Accession:FN421687.1

Alignment

Pseudomonas sp. Enf53 16S ribosomal RNA gene, partial s..

Score:1869 Evalue:0

Accession:DQ339614.1

Alignment

Pseudomonas sp. Enf30 16S ribosomal RNA gene, partial s..

Score:1869 Evalue:0

Accession:DQ339606.1

Alignment

Pseudomonas sp. Enf34 16S ribosomal RNA gene, partial s..

Score:1869 Evalue:0

Accession:DQ339600.1

Alignment

Pseudomonas graminis strain DSM 11363 16S ribosomal RNA..

Score:1869 Evalue:0

Accession:NR\_026395.1

Alignment

Pseudomonas sp. HP6G 16S ribosomal RNA gene, partial se..

Score:1868 Evalue:0

Accession:KM187506.1

Alignment

Pseudomonas sp. E06 16S ribosomal RNA gene, partial seq..

Score:1868 Evalue:0

Accession:JQ977048.1

Alignment

Pseudomonas graminis strain CMN4 16S ribosomal RNA gene..

Score:1864 Evalue:0

Accession:KY075949.1

Alignment

Pseudomonas graminis strain IHBB 9249 16S ribosomal RNA..

Score:1864 Evalue:0

Accession:KU921557.1

Alignment

Uncultured bacterium clone HL201309-60 16S ribosomal RN..

Score:1864 Evalue:0

Accession:KU515434.1

Alignment

Pseudomonas graminis strain PDD-58b-20 16S ribosomal RN..

Score:1864 Evalue:0

Accession:KR922133.1

Alignment

Pseudomonas graminis strain AP10-7B 16S ribosomal RNA g..

Score:1864 Evalue:0

Accession:KM891559.1

Alignment

Uncultured bacterium clone nbw115f12c1 16S ribosomal RN..

Score:1864 Evalue:0

Accession:KF064821.1

Alignment

Pseudomonas graminis strain PE24 16S ribosomal RNA gene..

Score:1864 Evalue:0

Accession:KJ127243.1

Alignment

Pseudomonas graminis isolate PDD-32b-55 16S ribosomal R..

Score:1864 Evalue:0

Accession:HQ256853.1

Alignment

Uncultured bacterium clone nby240d06c1 16S ribosomal RN..

Score:1864 Evalue:0

Accession:HM811884.1

Alignment

Uncultured bacterium clone nby239e02c1 16S ribosomal RN..

Score:1864 Evalue:0

Accession:HM811833.1

Alignment

Uncultured bacterium clone nby239f01c1 16S ribosomal RN..

Score:1864 Evalue:0

Accession:HM811814.1

Alignment

Uncultured bacterium clone nby237e07c1 16S ribosomal RN..

Score:1864 Evalue:0

Accession:HM811667.1

Alignment

## Descriptions

, Reading indexes 1-5, displaying indexes 1-5


Load next setPrevious Match

Sequences producing significant alignments:

Show all columns  of the table presenting sequences producing significant alignments 

Select:AllNone
Selected:0

Alignments
Download

FASTA (complete sequence)

FASTA (aligned sequences)

GenBank (complete sequence)

Hit Table (text)

Hit Table (CSV)

Text

XML

ASN.1

Continue
Cancel

GenBank 
Graphics
Distance tree of results
Multiple alignment
Show/hide columns of the table presenting sequences producing significant alignments 

Available columns

Description  
Max Score  
Total Score  
Coverage  
E-value  
IdentN  
Accession  
Restore Defaults
Ok
Cancel

Sequences producing significant alignments:

| Select for downloading or viewing reports | Description | Max score | Total score | Query cover | E value | Ident | Accession |
| --- | --- | --- | --- | --- | --- | --- | --- |
| 1Select seq KU587965.1 | Pseudomonas graminis strain A3 16S ribosomal RNA gene, partial sequence | 1875 | 1875 | 99% | 0.0 | 99% | KU587965.1 |
| 2Select seq KR922154.1 | Pseudomonas sp. PDD-59b-17 16S ribosomal RNA gene, complete sequence | 1875 | 1875 | 99% | 0.0 | 99% | KR922154.1 |
| 3Select seq LN551925.1 | Pseudomonas graminis partial 16S rRNA gene, strain DSM 11363 | 1875 | 1875 | 99% | 0.0 | 99% | LN551925.1 |
| 4Select seq KJ529045.1 | Pseudomonas graminis strain SR12 16S ribosomal RNA gene, partial sequence | 1875 | 1875 | 99% | 0.0 | 99% | KJ529045.1 |
| 5Select seq HG942149.1 | Pseudomonas sp. TA\_GU partial 16S rRNA gene, strain TA\_GU | 1875 | 1875 | 99% | 0.0 | 99% | HG942149.1 |
| 6Select seq KF147092.1 | Pseudomonas graminis strain R5SpM3P2C1 16S ribosomal RNA gene, partial sequence | 1875 | 1875 | 99% | 0.0 | 99% | KF147092.1 |
| 7Select seq JQ977250.1 | Pseudomonas sp. Bza15 16S ribosomal RNA gene, partial sequence | 1875 | 1875 | 99% | 0.0 | 99% | JQ977250.1 |
| 8Select seq KJ529030.1 | Pseudomonas graminis strain SL11 16S ribosomal RNA gene, partial sequence | 1873 | 1873 | 99% | 0.0 | 99% | KJ529030.1 |
| 9Select seq JQ727450.1 | Bacterium enrichment culture clone SaB5 16S ribosomal RNA gene, partial sequence | 1871 | 1871 | 99% | 0.0 | 99% | JQ727450.1 |
| 10Select seq MG561813.1 | Pseudomonas graminis strain AN1 16S ribosomal RNA gene, partial sequence | 1869 | 1869 | 99% | 0.0 | 99% | MG561813.1 |
| 11Select seq KY907017.1 | Pseudomonas sp. strain MSM-2-10-15 16S ribosomal RNA gene, partial sequence | 1869 | 1869 | 99% | 0.0 | 99% | KY907017.1 |
| 12Select seq LT601010.1 | Pseudomonas sp. CCUG 63225 partial 16S rRNA gene, strain CCUG 63225 | 1869 | 1869 | 99% | 0.0 | 99% | LT601010.1 |
| 13Select seq KU523561.1 | Pseudomonas graminis 16S ribosomal RNA gene, partial sequence | 1869 | 1869 | 99% | 0.0 | 99% | KU523561.1 |
| 14Select seq KR922175.1 | Pseudomonas sp. PDD-59b-53 16S ribosomal RNA gene, complete sequence | 1869 | 1869 | 99% | 0.0 | 99% | KR922175.1 |
| 15Select seq KR922157.1 | Pseudomonas graminis strain PDD-59b-25 16S ribosomal RNA gene, complete sequence | 1869 | 1869 | 99% | 0.0 | 99% | KR922157.1 |
| 16Select seq KM187516.1 | Pseudomonas sp. HP7D 16S ribosomal RNA gene, partial sequence | 1869 | 1869 | 98% | 0.0 | 99% | KM187516.1 |
| 17Select seq KM187449.1 | Pseudomonas sp. HP3I 16S ribosomal RNA gene, partial sequence | 1869 | 1869 | 99% | 0.0 | 99% | KM187449.1 |
| 18Select seq KP209394.1 | Pseudomonas graminis strain EB25 16S ribosomal RNA gene, partial sequence | 1869 | 1869 | 99% | 0.0 | 99% | KP209394.1 |
| 19Select seq KJ606806.1 | Pseudomonas sp. PAMC26590 16S ribosomal RNA gene, partial sequence | 1869 | 1869 | 99% | 0.0 | 99% | KJ606806.1 |
| 20Select seq JX042421.1 | Bacterium 3t 16S ribosomal RNA gene, partial sequence | 1869 | 1869 | 99% | 0.0 | 99% | JX042421.1 |
| 21Select seq KF147102.1 | Pseudomonas sp. R1SsM3P3C4 16S ribosomal RNA gene, partial sequence | 1869 | 1869 | 98% | 0.0 | 99% | KF147102.1 |
| 22Select seq KF147101.1 | Pseudomonas sp. R1SpM3P3C2 16S ribosomal RNA gene, partial sequence | 1869 | 1869 | 98% | 0.0 | 99% | KF147101.1 |
| 23Select seq KF147100.1 | Pseudomonas sp. R1SsM3P2C4 16S ribosomal RNA gene, partial sequence | 1869 | 1869 | 98% | 0.0 | 99% | KF147100.1 |
| 24Select seq KF147099.1 | Pseudomonas sp. R1SpM3P2C4 16S ribosomal RNA gene, partial sequence | 1869 | 1869 | 98% | 0.0 | 99% | KF147099.1 |
| 25Select seq KF147098.1 | Pseudomonas sp. R5SsM3P3C9 16S ribosomal RNA gene, partial sequence | 1869 | 1869 | 98% | 0.0 | 99% | KF147098.1 |
| 26Select seq KF147097.1 | Pseudomonas sp. R2SpM3P2C5 16S ribosomal RNA gene, partial sequence | 1869 | 1869 | 98% | 0.0 | 99% | KF147097.1 |
| 27Select seq KF147094.1 | Pseudomonas graminis strain R2SsM3P1C4 16S ribosomal RNA gene, partial sequence | 1869 | 1869 | 98% | 0.0 | 99% | KF147094.1 |
| 28Select seq KF147093.1 | Pseudomonas graminis strain R2SsM3P1C3 16S ribosomal RNA gene, partial sequence | 1869 | 1869 | 98% | 0.0 | 99% | KF147093.1 |
| 29Select seq KF147091.1 | Pseudomonas graminis strain R5SsM3P2C7 16S ribosomal RNA gene, partial sequence | 1869 | 1869 | 99% | 0.0 | 99% | KF147091.1 |
| 30Select seq KF147090.1 | Pseudomonas graminis strain R1SpM3P3C4 16S ribosomal RNA gene, partial sequence | 1869 | 1869 | 98% | 0.0 | 99% | KF147090.1 |
| 31Select seq KF147089.1 | Pseudomonas graminis strain R1SpM3P1C6 16S ribosomal RNA gene, partial sequence | 1869 | 1869 | 98% | 0.0 | 99% | KF147089.1 |
| 32Select seq JQ977394.1 | Pseudomonas sp. Axa8 16S ribosomal RNA gene, partial sequence | 1869 | 1869 | 99% | 0.0 | 99% | JQ977394.1 |
| 33Select seq HE652089.1 | Pseudomonas sp. E9 partial 16S rRNA gene, isolate E9 | 1869 | 1869 | 99% | 0.0 | 99% | HE652089.1 |
| 34Select seq HQ256858.1 | Pseudomonas graminis isolate PDD-32b-60 16S ribosomal RNA gene, partial sequence | 1869 | 1869 | 99% | 0.0 | 99% | HQ256858.1 |
| 35Select seq HQ179006.1 | Bacterium OC34(2011) 16S ribosomal RNA gene, partial sequence | 1869 | 1869 | 99% | 0.0 | 99% | HQ179006.1 |
| 36Select seq HM811719.1 | Uncultured bacterium clone nby238d11c1 16S ribosomal RNA gene, partial sequence | 1869 | 1869 | 99% | 0.0 | 99% | HM811719.1 |
| 37Select seq GU396281.1 | Pseudomonas graminis strain RMPP6 16S ribosomal RNA gene, partial sequence | 1869 | 1869 | 99% | 0.0 | 99% | GU396281.1 |
| 38Select seq FN421687.1 | Uncultured bacterium partial 16S rRNA gene, clone 3\_E03 | 1869 | 1869 | 99% | 0.0 | 99% | FN421687.1 |
| 39Select seq DQ339614.1 | Pseudomonas sp. Enf53 16S ribosomal RNA gene, partial sequence | 1869 | 1869 | 99% | 0.0 | 99% | DQ339614.1 |
| 40Select seq DQ339606.1 | Pseudomonas sp. Enf30 16S ribosomal RNA gene, partial sequence | 1869 | 1869 | 99% | 0.0 | 99% | DQ339606.1 |
| 41Select seq DQ339600.1 | Pseudomonas sp. Enf34 16S ribosomal RNA gene, partial sequence | 1869 | 1869 | 99% | 0.0 | 99% | DQ339600.1 |
| 42Select seq NR\_026395.1 | Pseudomonas graminis strain DSM 11363 16S ribosomal RNA, partial sequence | 1869 | 1869 | 99% | 0.0 | 99% | NR\_026395.1 |
| 43Select seq KM187506.1 | Pseudomonas sp. HP6G 16S ribosomal RNA gene, partial sequence | 1868 | 1868 | 99% | 0.0 | 99% | KM187506.1 |
| 44Select seq JQ977048.1 | Pseudomonas sp. E06 16S ribosomal RNA gene, partial sequence | 1868 | 1868 | 99% | 0.0 | 99% | JQ977048.1 |
| 45Select seq KY075949.1 | Pseudomonas graminis strain CMN4 16S ribosomal RNA gene, partial sequence | 1864 | 1864 | 99% | 0.0 | 99% | KY075949.1 |
| 46Select seq KU921557.1 | Pseudomonas graminis strain IHBB 9249 16S ribosomal RNA gene, partial sequence | 1864 | 1864 | 99% | 0.0 | 99% | KU921557.1 |
| 47Select seq KU515434.1 | Uncultured bacterium clone HL201309-60 16S ribosomal RNA gene, partial sequence | 1864 | 1864 | 99% | 0.0 | 99% | KU515434.1 |
| 48Select seq KR922133.1 | Pseudomonas graminis strain PDD-58b-20 16S ribosomal RNA gene, complete sequence | 1864 | 1864 | 99% | 0.0 | 99% | KR922133.1 |
| 49Select seq KM891559.1 | Pseudomonas graminis strain AP10-7B 16S ribosomal RNA gene, partial sequence | 1864 | 1864 | 99% | 0.0 | 99% | KM891559.1 |
| 50Select seq KF064821.1 | Uncultured bacterium clone nbw115f12c1 16S ribosomal RNA gene, partial sequence | 1864 | 1864 | 99% | 0.0 | 99% | KF064821.1 |
| 51Select seq KJ127243.1 | Pseudomonas graminis strain PE24 16S ribosomal RNA gene, partial sequence | 1864 | 1864 | 99% | 0.0 | 99% | KJ127243.1 |
| 52Select seq HQ256853.1 | Pseudomonas graminis isolate PDD-32b-55 16S ribosomal RNA gene, partial sequence | 1864 | 1864 | 99% | 0.0 | 99% | HQ256853.1 |
| 53Select seq HM811884.1 | Uncultured bacterium clone nby240d06c1 16S ribosomal RNA gene, partial sequence | 1864 | 1864 | 99% | 0.0 | 99% | HM811884.1 |
| 54Select seq HM811833.1 | Uncultured bacterium clone nby239e02c1 16S ribosomal RNA gene, partial sequence | 1864 | 1864 | 99% | 0.0 | 99% | HM811833.1 |
| 55Select seq HM811814.1 | Uncultured bacterium clone nby239f01c1 16S ribosomal RNA gene, partial sequence | 1864 | 1864 | 99% | 0.0 | 99% | HM811814.1 |
| 56Select seq HM811667.1 | Uncultured bacterium clone nby237e07c1 16S ribosomal RNA gene, partial sequence | 1864 | 1864 | 99% | 0.0 | 99% | HM811667.1 |
| 57Select seq HM807832.1 | Uncultured bacterium clone nby240a10c1 16S ribosomal RNA gene, partial sequence | 1864 | 1864 | 99% | 0.0 | 99% | HM807832.1 |
| 58Select seq GQ008235.1 | Uncultured bacterium clone nbw115a10c1 16S ribosomal RNA gene, partial sequence | 1864 | 1864 | 99% | 0.0 | 99% | GQ008235.1 |
| 59Select seq DQ339611.1 | Pseudomonas sp. Enf26 16S ribosomal RNA gene, partial sequence | 1864 | 1864 | 99% | 0.0 | 99% | DQ339611.1 |
| 60Select seq DQ339586.1 | Pseudomonas sp. Enf5 16S ribosomal RNA gene, partial sequence | 1864 | 1864 | 98% | 0.0 | 99% | DQ339586.1 |
| 61Select seq KR922063.1 | Pseudomonas sp. PDD-48b-10 16S ribosomal RNA gene, complete sequence | 1862 | 1862 | 99% | 0.0 | 99% | KR922063.1 |
| 62Select seq LN852371.1 | Pseudomonas graminis partial 16S rRNA gene, isolate PclHEPES2 | 1860 | 1860 | 98% | 0.0 | 99% | LN852371.1 |
| 63Select seq KT752941.1 | Uncultured Pseudomonas sp. clone Pool25 16S ribosomal RNA gene, partial sequence | 1860 | 1860 | 98% | 0.0 | 99% | KT752941.1 |
| 64Select seq KT369928.1 | Pseudomonas graminis strain YF04-3(1) 16S ribosomal RNA gene, partial sequence | 1860 | 1860 | 98% | 0.0 | 99% | KT369928.1 |
| 65Select seq KM187192.1 | Pseudomonas sp. CC15J 16S ribosomal RNA gene, partial sequence | 1860 | 1860 | 98% | 0.0 | 99% | KM187192.1 |
| 66Select seq KF147086.1 | Pseudomonas graminis strain R2SpM3P2C3 16S ribosomal RNA gene, partial sequence | 1860 | 1860 | 98% | 0.0 | 99% | KF147086.1 |
| 67Select seq HQ327116.1 | Pseudomonas graminis strain TP-Snow-C7 16S ribosomal RNA gene, partial sequence | 1860 | 1860 | 99% | 0.0 | 99% | HQ327116.1 |
| 68Select seq KF101357.1 | Uncultured bacterium clone ncd2649h03c1 16S ribosomal RNA gene, partial sequence | 1858 | 1858 | 99% | 0.0 | 99% | KF101357.1 |
| 69Select seq KF147083.1 | Pseudomonas graminis strain R2SsM3P1C6 16S ribosomal RNA gene, partial sequence | 1858 | 1858 | 98% | 0.0 | 99% | KF147083.1 |
| 70Select seq KF387661.1 | Pseudomonas graminis strain BDb2 16S ribosomal RNA gene, partial sequence | 1858 | 1858 | 98% | 0.0 | 99% | KF387661.1 |
| 71Select seq JQ977026.1 | Pseudomonas sp. A19(2013) 16S ribosomal RNA gene, partial sequence | 1858 | 1858 | 98% | 0.0 | 99% | JQ977026.1 |
| 72Select seq HM811683.1 | Uncultured bacterium clone nby237c06c1 16S ribosomal RNA gene, partial sequence | 1858 | 1858 | 99% | 0.0 | 99% | HM811683.1 |
| 73Select seq AM697261.1 | Uncultured bacterium partial 16S rRNA gene, isolate BF0001C102 | 1858 | 1858 | 99% | 0.0 | 99% | AM697261.1 |
| 74Select seq JQ727539.1 | Bacterium enrichment culture clone IcE7 16S ribosomal RNA gene, partial sequence | 1857 | 1857 | 98% | 0.0 | 99% | JQ727539.1 |
| 75Select seq FN814019.1 | Uncultured bacterium partial 16S rRNA gene, clone 26B2-B6 | 1857 | 1857 | 98% | 0.0 | 99% | FN814019.1 |
| 76Select seq FJ999944.1 | Pseudomonas graminis strain EQH15 16S ribosomal RNA gene, partial sequence | 1855 | 1855 | 98% | 0.0 | 99% | FJ999944.1 |
| 77Select seq KF071224.1 | Uncultured bacterium clone ncd272a04c1 16S ribosomal RNA gene, partial sequence | 1853 | 1853 | 99% | 0.0 | 99% | KF071224.1 |
| 78Select seq JQ727533.1 | Bacterium enrichment culture clone IcD11 16S ribosomal RNA gene, partial sequence | 1853 | 1853 | 98% | 0.0 | 99% | JQ727533.1 |
| 79Select seq HM811877.1 | Uncultured bacterium clone nby240g05c1 16S ribosomal RNA gene, partial sequence | 1853 | 1853 | 99% | 0.0 | 99% | HM811877.1 |
| 80Select seq KF147081.1 | Pseudomonas graminis strain R2SpM3P1C2 16S ribosomal RNA gene, partial sequence | 1845 | 1845 | 99% | 0.0 | 99% | KF147081.1 |
| 81Select seq KF147078.1 | Pseudomonas graminis strain R3ScM3P1C4 16S ribosomal RNA gene, partial sequence | 1845 | 1845 | 99% | 0.0 | 99% | KF147078.1 |
| 82Select seq AF511514.1 | Pseudomonas graminis isolate HhSaOsb 16S ribosomal RNA gene, partial sequence | 1845 | 1845 | 99% | 0.0 | 99% | AF511514.1 |
| 83Select seq HM266979.1 | Uncultured bacterium clone ncd223f09c1 16S ribosomal RNA gene, partial sequence | 1844 | 1844 | 99% | 0.0 | 99% | HM266979.1 |
| 84Select seq GQ107306.1 | Uncultured bacterium clone nbw547c03c1 16S ribosomal RNA gene, partial sequence | 1844 | 1844 | 99% | 0.0 | 99% | GQ107306.1 |
| 85Select seq KT991041.1 | Pseudomonas sp. 2EK4 16S ribosomal RNA gene, partial sequence | 1842 | 1842 | 97% | 0.0 | 99% | KT991041.1 |
| 86Select seq JQ727563.1 | Bacterium enrichment culture clone IcH4 16S ribosomal RNA gene, partial sequence | 1842 | 1842 | 97% | 0.0 | 99% | JQ727563.1 |
| 87Select seq JQ727559.1 | Bacterium enrichment culture clone IcG8 16S ribosomal RNA gene, partial sequence | 1842 | 1842 | 97% | 0.0 | 99% | JQ727559.1 |
| 88Select seq EU153018.1 | Uncultured bacterium clone G22-10 16S ribosomal RNA gene, partial sequence | 1842 | 1842 | 99% | 0.0 | 99% | EU153018.1 |
| 89Select seq KF147074.1 | Pseudomonas graminis strain R5SsM3P3C8 16S ribosomal RNA gene, partial sequence | 1840 | 1840 | 99% | 0.0 | 99% | KF147074.1 |
| 90Select seq JX827229.1 | Pseudomonas graminis strain Tibetlhz-52 16S ribosomal RNA gene, partial sequence | 1840 | 1840 | 97% | 0.0 | 99% | JX827229.1 |
| 91Select seq JQ727517.1 | Bacterium enrichment culture clone IcC5 16S ribosomal RNA gene, partial sequence | 1840 | 1840 | 97% | 0.0 | 99% | JQ727517.1 |
| 92Select seq HQ256863.1 | Pseudomonas graminis isolate PDD-32b-66 16S ribosomal RNA gene, partial sequence | 1840 | 1840 | 97% | 0.0 | 99% | HQ256863.1 |
| 93Select seq DQ512786.1 | Pseudomonas graminis strain PDD-13b-3 16S ribosomal RNA gene, partial sequence | 1840 | 1840 | 98% | 0.0 | 99% | DQ512786.1 |
| 94Select seq AF511513.1 | Pseudomonas graminis isolate SaU5 16S ribosomal RNA gene, partial sequence | 1840 | 1840 | 99% | 0.0 | 99% | AF511513.1 |
| 95Select seq KR922249.1 | Pseudomonas graminis strain PDD-66b-5 16S ribosomal RNA gene, complete sequence | 1836 | 1836 | 97% | 0.0 | 99% | KR922249.1 |
| 96Select seq KP324943.1 | Pseudomonas sp. EB24 16S ribosomal RNA gene, partial sequence | 1836 | 1836 | 99% | 0.0 | 99% | KP324943.1 |
| 97Select seq KF147079.1 | Pseudomonas graminis strain R2SsM3P1C5 16S ribosomal RNA gene, partial sequence | 1836 | 1836 | 99% | 0.0 | 99% | KF147079.1 |
| 98Select seq JF706541.1 | Pseudomonas graminis strain PDD-38b-9 16S ribosomal RNA gene, partial sequence | 1836 | 1836 | 97% | 0.0 | 99% | JF706541.1 |
| 99Select seq DQ059301.1 | Pseudomonas graminis strain VH15022005 16S ribosomal RNA gene, partial sequence | 1836 | 1836 | 99% | 0.0 | 99% | DQ059301.1 |
| 100Select seq AY866408.1 | Pseudomonas rhizosphaerae strain OW-2 16S ribosomal RNA gene, partial sequence | 1836 | 1836 | 99% | 0.0 | 99% | AY866408.1 |


## Alignments

Loading alignment... for sequences gi|1047300877,gi|939107655,gi|696533438,gi|632794966,gi|631782784 Reading indexes 1-5

Download

FASTA (complete sequence)

FASTA (aligned sequences)

GenBank (complete sequence)

Continue
Cancel

GenBankGraphics

Next
Previous
Descriptions

Pseudomonas graminis strain A3 16S ribosomal RNA gene, partial sequence

Sequence ID: KU587965.1Length: 1391Number of Matches: 1

Related Information

Range 1: 1 to 1017GenBankGraphics

Next Match
Previous Match
First Match

Alignment statistics for match #1

| Score | Expect | Identities | Gaps | Strand | Frame |
| --- | --- | --- | --- | --- | --- |
| 1875 bits(1015) | 0.0() | 1016/1017(99%) | 0/1017(0%) | Plus/Plus |  |

Features:

```
Query  7     TGCAGTCGAGCGGATGAAGAGAGCTTGCTNTCTGATTCAGCGGCGGACGGGTGAGTAATG  66
             ||||||||||||||||||||||||||||| ||||||||||||||||||||||||||||||
Sbjct  1     TGCAGTCGAGCGGATGAAGAGAGCTTGCTCTCTGATTCAGCGGCGGACGGGTGAGTAATG  60

Query  67    CCTAGGAATCTGCCTGGTAGTGGGGGACAACGTCTCGAAAGGGACGCTAATACCGCATAC  126
             ||||||||||||||||||||||||||||||||||||||||||||||||||||||||||||
Sbjct  61    CCTAGGAATCTGCCTGGTAGTGGGGGACAACGTCTCGAAAGGGACGCTAATACCGCATAC  120

Query  127   GTCCTACGGGAGAAAGCAGGGGACCTTCGGGCCTTGCGCTATCAGATGAGCCTAGGTCGG  186
             ||||||||||||||||||||||||||||||||||||||||||||||||||||||||||||
Sbjct  121   GTCCTACGGGAGAAAGCAGGGGACCTTCGGGCCTTGCGCTATCAGATGAGCCTAGGTCGG  180

Query  187   ATTAGCTAGTTGGTGAGGTAATGGCTCACCAAGGCGACGATCCGTAACTGGTCTGAGAGG  246
             ||||||||||||||||||||||||||||||||||||||||||||||||||||||||||||
Sbjct  181   ATTAGCTAGTTGGTGAGGTAATGGCTCACCAAGGCGACGATCCGTAACTGGTCTGAGAGG  240

Query  247   ATGATCAGTCACACTGGAACTGAGACACGGTCCAGACTCCTACGGGAGGCAGCAGTGGGG  306
             ||||||||||||||||||||||||||||||||||||||||||||||||||||||||||||
Sbjct  241   ATGATCAGTCACACTGGAACTGAGACACGGTCCAGACTCCTACGGGAGGCAGCAGTGGGG  300

Query  307   AATATTGGACAATGGGCGAAAGCCTGATCCAGCCATGCCGCGTGTGTGAAGAAGGTCTTC  366
             ||||||||||||||||||||||||||||||||||||||||||||||||||||||||||||
Sbjct  301   AATATTGGACAATGGGCGAAAGCCTGATCCAGCCATGCCGCGTGTGTGAAGAAGGTCTTC  360

Query  367   GGATTGTAAAGCACTTTAAGTTGGGAGGAAGGGCAGTAAGCGAATACCTTGCTGTTTTGA  426
             ||||||||||||||||||||||||||||||||||||||||||||||||||||||||||||
Sbjct  361   GGATTGTAAAGCACTTTAAGTTGGGAGGAAGGGCAGTAAGCGAATACCTTGCTGTTTTGA  420

Query  427   CGTTACCGACAGAATAAGCACCGGCTAACTCTGTGCCAGCAGCCGCGGTAATACAGAGGG  486
             ||||||||||||||||||||||||||||||||||||||||||||||||||||||||||||
Sbjct  421   CGTTACCGACAGAATAAGCACCGGCTAACTCTGTGCCAGCAGCCGCGGTAATACAGAGGG  480

Query  487   TGCAAGCGTTAATCGGAATTACTGGGCGTAAAGCGCGCGTAGGTGGTTTGTTAAGTTGAA  546
             ||||||||||||||||||||||||||||||||||||||||||||||||||||||||||||
Sbjct  481   TGCAAGCGTTAATCGGAATTACTGGGCGTAAAGCGCGCGTAGGTGGTTTGTTAAGTTGAA  540

Query  547   TGTGAAATCCCCGGGCTCAACCTGGGAACTGCATCCAAAACTGGCAAGCTAGAGTAGGGC  606
             ||||||||||||||||||||||||||||||||||||||||||||||||||||||||||||
Sbjct  541   TGTGAAATCCCCGGGCTCAACCTGGGAACTGCATCCAAAACTGGCAAGCTAGAGTAGGGC  600

Query  607   AGAGGGTGGTGGAATTTCCTGTGTAGCGGTGAAATGCGTAGATATAGGAAGGAACACCAG  666
             ||||||||||||||||||||||||||||||||||||||||||||||||||||||||||||
Sbjct  601   AGAGGGTGGTGGAATTTCCTGTGTAGCGGTGAAATGCGTAGATATAGGAAGGAACACCAG  660

Query  667   TGGCGAAGGCGACCACCTGGGCTCATACTGACACTGAGGTGCGAAAGCGTGGGGAGCAAA  726
             ||||||||||||||||||||||||||||||||||||||||||||||||||||||||||||
Sbjct  661   TGGCGAAGGCGACCACCTGGGCTCATACTGACACTGAGGTGCGAAAGCGTGGGGAGCAAA  720

Query  727   CAGGATTAGATACCCTGGTAGTCCACGCCGTAAACGATGTCAACTAGCCGTTGGAAGCCT  786
             ||||||||||||||||||||||||||||||||||||||||||||||||||||||||||||
Sbjct  721   CAGGATTAGATACCCTGGTAGTCCACGCCGTAAACGATGTCAACTAGCCGTTGGAAGCCT  780

Query  787   TGAGCTTTTAGTGGCGCAGCTAACGCATTAAGTTGACCGCCTGGGGAGTACGGCCGCAAG  846
             ||||||||||||||||||||||||||||||||||||||||||||||||||||||||||||
Sbjct  781   TGAGCTTTTAGTGGCGCAGCTAACGCATTAAGTTGACCGCCTGGGGAGTACGGCCGCAAG  840

Query  847   GTTAAAACTCAAATGAATTGACGGGGGCCCGCACAAGCGGTGGAGCATGTGGTTTAATTC  906
             ||||||||||||||||||||||||||||||||||||||||||||||||||||||||||||
Sbjct  841   GTTAAAACTCAAATGAATTGACGGGGGCCCGCACAAGCGGTGGAGCATGTGGTTTAATTC  900

Query  907   GAAGCAACGCGAAGAACCTTACCAGGCCTTGACATCCAATGAACTTTCCAGAGATGGATT  966
             ||||||||||||||||||||||||||||||||||||||||||||||||||||||||||||
Sbjct  901   GAAGCAACGCGAAGAACCTTACCAGGCCTTGACATCCAATGAACTTTCCAGAGATGGATT  960

Query  967   GGTGCCTTCGGGAACATTGAGACAGGTGCTGCATGGCTGTCGTCAGCTCGTGTCGTG  1023
             |||||||||||||||||||||||||||||||||||||||||||||||||||||||||
Sbjct  961   GGTGCCTTCGGGAACATTGAGACAGGTGCTGCATGGCTGTCGTCAGCTCGTGTCGTG  1017
```

Download

FASTA (complete sequence)

FASTA (aligned sequences)

GenBank (complete sequence)

Continue
Cancel

GenBankGraphics

Next
Previous
Descriptions

Pseudomonas sp. PDD-59b-17 16S ribosomal RNA gene, complete sequence

Sequence ID: KR922154.1Length: 1416Number of Matches: 1

Related Information

Range 1: 8 to 1024GenBankGraphics

Next Match
Previous Match
First Match

Alignment statistics for match #1

| Score | Expect | Identities | Gaps | Strand | Frame |
| --- | --- | --- | --- | --- | --- |
| 1875 bits(1015) | 0.0() | 1016/1017(99%) | 0/1017(0%) | Plus/Plus |  |

Features:

```
Query  7     TGCAGTCGAGCGGATGAAGAGAGCTTGCTNTCTGATTCAGCGGCGGACGGGTGAGTAATG  66
             ||||||||||||||||||||||||||||| ||||||||||||||||||||||||||||||
Sbjct  8     TGCAGTCGAGCGGATGAAGAGAGCTTGCTCTCTGATTCAGCGGCGGACGGGTGAGTAATG  67

Query  67    CCTAGGAATCTGCCTGGTAGTGGGGGACAACGTCTCGAAAGGGACGCTAATACCGCATAC  126
             ||||||||||||||||||||||||||||||||||||||||||||||||||||||||||||
Sbjct  68    CCTAGGAATCTGCCTGGTAGTGGGGGACAACGTCTCGAAAGGGACGCTAATACCGCATAC  127

Query  127   GTCCTACGGGAGAAAGCAGGGGACCTTCGGGCCTTGCGCTATCAGATGAGCCTAGGTCGG  186
             ||||||||||||||||||||||||||||||||||||||||||||||||||||||||||||
Sbjct  128   GTCCTACGGGAGAAAGCAGGGGACCTTCGGGCCTTGCGCTATCAGATGAGCCTAGGTCGG  187

Query  187   ATTAGCTAGTTGGTGAGGTAATGGCTCACCAAGGCGACGATCCGTAACTGGTCTGAGAGG  246
             ||||||||||||||||||||||||||||||||||||||||||||||||||||||||||||
Sbjct  188   ATTAGCTAGTTGGTGAGGTAATGGCTCACCAAGGCGACGATCCGTAACTGGTCTGAGAGG  247

Query  247   ATGATCAGTCACACTGGAACTGAGACACGGTCCAGACTCCTACGGGAGGCAGCAGTGGGG  306
             ||||||||||||||||||||||||||||||||||||||||||||||||||||||||||||
Sbjct  248   ATGATCAGTCACACTGGAACTGAGACACGGTCCAGACTCCTACGGGAGGCAGCAGTGGGG  307

Query  307   AATATTGGACAATGGGCGAAAGCCTGATCCAGCCATGCCGCGTGTGTGAAGAAGGTCTTC  366
             ||||||||||||||||||||||||||||||||||||||||||||||||||||||||||||
Sbjct  308   AATATTGGACAATGGGCGAAAGCCTGATCCAGCCATGCCGCGTGTGTGAAGAAGGTCTTC  367

Query  367   GGATTGTAAAGCACTTTAAGTTGGGAGGAAGGGCAGTAAGCGAATACCTTGCTGTTTTGA  426
             ||||||||||||||||||||||||||||||||||||||||||||||||||||||||||||
Sbjct  368   GGATTGTAAAGCACTTTAAGTTGGGAGGAAGGGCAGTAAGCGAATACCTTGCTGTTTTGA  427

Query  427   CGTTACCGACAGAATAAGCACCGGCTAACTCTGTGCCAGCAGCCGCGGTAATACAGAGGG  486
             ||||||||||||||||||||||||||||||||||||||||||||||||||||||||||||
Sbjct  428   CGTTACCGACAGAATAAGCACCGGCTAACTCTGTGCCAGCAGCCGCGGTAATACAGAGGG  487

Query  487   TGCAAGCGTTAATCGGAATTACTGGGCGTAAAGCGCGCGTAGGTGGTTTGTTAAGTTGAA  546
             ||||||||||||||||||||||||||||||||||||||||||||||||||||||||||||
Sbjct  488   TGCAAGCGTTAATCGGAATTACTGGGCGTAAAGCGCGCGTAGGTGGTTTGTTAAGTTGAA  547

Query  547   TGTGAAATCCCCGGGCTCAACCTGGGAACTGCATCCAAAACTGGCAAGCTAGAGTAGGGC  606
             ||||||||||||||||||||||||||||||||||||||||||||||||||||||||||||
Sbjct  548   TGTGAAATCCCCGGGCTCAACCTGGGAACTGCATCCAAAACTGGCAAGCTAGAGTAGGGC  607

Query  607   AGAGGGTGGTGGAATTTCCTGTGTAGCGGTGAAATGCGTAGATATAGGAAGGAACACCAG  666
             ||||||||||||||||||||||||||||||||||||||||||||||||||||||||||||
Sbjct  608   AGAGGGTGGTGGAATTTCCTGTGTAGCGGTGAAATGCGTAGATATAGGAAGGAACACCAG  667

Query  667   TGGCGAAGGCGACCACCTGGGCTCATACTGACACTGAGGTGCGAAAGCGTGGGGAGCAAA  726
             ||||||||||||||||||||||||||||||||||||||||||||||||||||||||||||
Sbjct  668   TGGCGAAGGCGACCACCTGGGCTCATACTGACACTGAGGTGCGAAAGCGTGGGGAGCAAA  727

Query  727   CAGGATTAGATACCCTGGTAGTCCACGCCGTAAACGATGTCAACTAGCCGTTGGAAGCCT  786
             ||||||||||||||||||||||||||||||||||||||||||||||||||||||||||||
Sbjct  728   CAGGATTAGATACCCTGGTAGTCCACGCCGTAAACGATGTCAACTAGCCGTTGGAAGCCT  787

Query  787   TGAGCTTTTAGTGGCGCAGCTAACGCATTAAGTTGACCGCCTGGGGAGTACGGCCGCAAG  846
             ||||||||||||||||||||||||||||||||||||||||||||||||||||||||||||
Sbjct  788   TGAGCTTTTAGTGGCGCAGCTAACGCATTAAGTTGACCGCCTGGGGAGTACGGCCGCAAG  847

Query  847   GTTAAAACTCAAATGAATTGACGGGGGCCCGCACAAGCGGTGGAGCATGTGGTTTAATTC  906
             ||||||||||||||||||||||||||||||||||||||||||||||||||||||||||||
Sbjct  848   GTTAAAACTCAAATGAATTGACGGGGGCCCGCACAAGCGGTGGAGCATGTGGTTTAATTC  907

Query  907   GAAGCAACGCGAAGAACCTTACCAGGCCTTGACATCCAATGAACTTTCCAGAGATGGATT  966
             ||||||||||||||||||||||||||||||||||||||||||||||||||||||||||||
Sbjct  908   GAAGCAACGCGAAGAACCTTACCAGGCCTTGACATCCAATGAACTTTCCAGAGATGGATT  967

Query  967   GGTGCCTTCGGGAACATTGAGACAGGTGCTGCATGGCTGTCGTCAGCTCGTGTCGTG  1023
             |||||||||||||||||||||||||||||||||||||||||||||||||||||||||
Sbjct  968   GGTGCCTTCGGGAACATTGAGACAGGTGCTGCATGGCTGTCGTCAGCTCGTGTCGTG  1024
```

Download

FASTA (complete sequence)

FASTA (aligned sequences)

GenBank (complete sequence)

Continue
Cancel

GenBankGraphics

Next
Previous
Descriptions

Pseudomonas graminis partial 16S rRNA gene, strain DSM 11363

Sequence ID: LN551925.1Length: 1315Number of Matches: 1

Related Information

Range 1: 6 to 1022GenBankGraphics

Next Match
Previous Match
First Match

Alignment statistics for match #1

| Score | Expect | Identities | Gaps | Strand | Frame |
| --- | --- | --- | --- | --- | --- |
| 1875 bits(1015) | 0.0() | 1016/1017(99%) | 0/1017(0%) | Plus/Plus |  |

Features:

```
Query  7     TGCAGTCGAGCGGATGAAGAGAGCTTGCTNTCTGATTCAGCGGCGGACGGGTGAGTAATG  66
             ||||||||||||||||||||||||||||| ||||||||||||||||||||||||||||||
Sbjct  6     TGCAGTCGAGCGGATGAAGAGAGCTTGCTCTCTGATTCAGCGGCGGACGGGTGAGTAATG  65

Query  67    CCTAGGAATCTGCCTGGTAGTGGGGGACAACGTCTCGAAAGGGACGCTAATACCGCATAC  126
             ||||||||||||||||||||||||||||||||||||||||||||||||||||||||||||
Sbjct  66    CCTAGGAATCTGCCTGGTAGTGGGGGACAACGTCTCGAAAGGGACGCTAATACCGCATAC  125

Query  127   GTCCTACGGGAGAAAGCAGGGGACCTTCGGGCCTTGCGCTATCAGATGAGCCTAGGTCGG  186
             ||||||||||||||||||||||||||||||||||||||||||||||||||||||||||||
Sbjct  126   GTCCTACGGGAGAAAGCAGGGGACCTTCGGGCCTTGCGCTATCAGATGAGCCTAGGTCGG  185

Query  187   ATTAGCTAGTTGGTGAGGTAATGGCTCACCAAGGCGACGATCCGTAACTGGTCTGAGAGG  246
             ||||||||||||||||||||||||||||||||||||||||||||||||||||||||||||
Sbjct  186   ATTAGCTAGTTGGTGAGGTAATGGCTCACCAAGGCGACGATCCGTAACTGGTCTGAGAGG  245

Query  247   ATGATCAGTCACACTGGAACTGAGACACGGTCCAGACTCCTACGGGAGGCAGCAGTGGGG  306
             ||||||||||||||||||||||||||||||||||||||||||||||||||||||||||||
Sbjct  246   ATGATCAGTCACACTGGAACTGAGACACGGTCCAGACTCCTACGGGAGGCAGCAGTGGGG  305

Query  307   AATATTGGACAATGGGCGAAAGCCTGATCCAGCCATGCCGCGTGTGTGAAGAAGGTCTTC  366
             ||||||||||||||||||||||||||||||||||||||||||||||||||||||||||||
Sbjct  306   AATATTGGACAATGGGCGAAAGCCTGATCCAGCCATGCCGCGTGTGTGAAGAAGGTCTTC  365

Query  367   GGATTGTAAAGCACTTTAAGTTGGGAGGAAGGGCAGTAAGCGAATACCTTGCTGTTTTGA  426
             ||||||||||||||||||||||||||||||||||||||||||||||||||||||||||||
Sbjct  366   GGATTGTAAAGCACTTTAAGTTGGGAGGAAGGGCAGTAAGCGAATACCTTGCTGTTTTGA  425

Query  427   CGTTACCGACAGAATAAGCACCGGCTAACTCTGTGCCAGCAGCCGCGGTAATACAGAGGG  486
             ||||||||||||||||||||||||||||||||||||||||||||||||||||||||||||
Sbjct  426   CGTTACCGACAGAATAAGCACCGGCTAACTCTGTGCCAGCAGCCGCGGTAATACAGAGGG  485

Query  487   TGCAAGCGTTAATCGGAATTACTGGGCGTAAAGCGCGCGTAGGTGGTTTGTTAAGTTGAA  546
             ||||||||||||||||||||||||||||||||||||||||||||||||||||||||||||
Sbjct  486   TGCAAGCGTTAATCGGAATTACTGGGCGTAAAGCGCGCGTAGGTGGTTTGTTAAGTTGAA  545

Query  547   TGTGAAATCCCCGGGCTCAACCTGGGAACTGCATCCAAAACTGGCAAGCTAGAGTAGGGC  606
             ||||||||||||||||||||||||||||||||||||||||||||||||||||||||||||
Sbjct  546   TGTGAAATCCCCGGGCTCAACCTGGGAACTGCATCCAAAACTGGCAAGCTAGAGTAGGGC  605

Query  607   AGAGGGTGGTGGAATTTCCTGTGTAGCGGTGAAATGCGTAGATATAGGAAGGAACACCAG  666
             ||||||||||||||||||||||||||||||||||||||||||||||||||||||||||||
Sbjct  606   AGAGGGTGGTGGAATTTCCTGTGTAGCGGTGAAATGCGTAGATATAGGAAGGAACACCAG  665

Query  667   TGGCGAAGGCGACCACCTGGGCTCATACTGACACTGAGGTGCGAAAGCGTGGGGAGCAAA  726
             ||||||||||||||||||||||||||||||||||||||||||||||||||||||||||||
Sbjct  666   TGGCGAAGGCGACCACCTGGGCTCATACTGACACTGAGGTGCGAAAGCGTGGGGAGCAAA  725

Query  727   CAGGATTAGATACCCTGGTAGTCCACGCCGTAAACGATGTCAACTAGCCGTTGGAAGCCT  786
             ||||||||||||||||||||||||||||||||||||||||||||||||||||||||||||
Sbjct  726   CAGGATTAGATACCCTGGTAGTCCACGCCGTAAACGATGTCAACTAGCCGTTGGAAGCCT  785

Query  787   TGAGCTTTTAGTGGCGCAGCTAACGCATTAAGTTGACCGCCTGGGGAGTACGGCCGCAAG  846
             ||||||||||||||||||||||||||||||||||||||||||||||||||||||||||||
Sbjct  786   TGAGCTTTTAGTGGCGCAGCTAACGCATTAAGTTGACCGCCTGGGGAGTACGGCCGCAAG  845

Query  847   GTTAAAACTCAAATGAATTGACGGGGGCCCGCACAAGCGGTGGAGCATGTGGTTTAATTC  906
             ||||||||||||||||||||||||||||||||||||||||||||||||||||||||||||
Sbjct  846   GTTAAAACTCAAATGAATTGACGGGGGCCCGCACAAGCGGTGGAGCATGTGGTTTAATTC  905

Query  907   GAAGCAACGCGAAGAACCTTACCAGGCCTTGACATCCAATGAACTTTCCAGAGATGGATT  966
             ||||||||||||||||||||||||||||||||||||||||||||||||||||||||||||
Sbjct  906   GAAGCAACGCGAAGAACCTTACCAGGCCTTGACATCCAATGAACTTTCCAGAGATGGATT  965

Query  967   GGTGCCTTCGGGAACATTGAGACAGGTGCTGCATGGCTGTCGTCAGCTCGTGTCGTG  1023
             |||||||||||||||||||||||||||||||||||||||||||||||||||||||||
Sbjct  966   GGTGCCTTCGGGAACATTGAGACAGGTGCTGCATGGCTGTCGTCAGCTCGTGTCGTG  1022
```

Download

FASTA (complete sequence)

FASTA (aligned sequences)

GenBank (complete sequence)

Continue
Cancel

GenBankGraphics

Next
Previous
Descriptions

Pseudomonas graminis strain SR12 16S ribosomal RNA gene, partial sequence

Sequence ID: KJ529045.1Length: 1068Number of Matches: 1

Related Information

Range 1: 1 to 1017GenBankGraphics

Next Match
Previous Match
First Match

Alignment statistics for match #1

| Score | Expect | Identities | Gaps | Strand | Frame |
| --- | --- | --- | --- | --- | --- |
| 1875 bits(1015) | 0.0() | 1016/1017(99%) | 0/1017(0%) | Plus/Plus |  |

Features:

```
Query  7     TGCAGTCGAGCGGATGAAGAGAGCTTGCTNTCTGATTCAGCGGCGGACGGGTGAGTAATG  66
             ||||||||||||||||||||||||||||| ||||||||||||||||||||||||||||||
Sbjct  1     TGCAGTCGAGCGGATGAAGAGAGCTTGCTCTCTGATTCAGCGGCGGACGGGTGAGTAATG  60

Query  67    CCTAGGAATCTGCCTGGTAGTGGGGGACAACGTCTCGAAAGGGACGCTAATACCGCATAC  126
             ||||||||||||||||||||||||||||||||||||||||||||||||||||||||||||
Sbjct  61    CCTAGGAATCTGCCTGGTAGTGGGGGACAACGTCTCGAAAGGGACGCTAATACCGCATAC  120

Query  127   GTCCTACGGGAGAAAGCAGGGGACCTTCGGGCCTTGCGCTATCAGATGAGCCTAGGTCGG  186
             ||||||||||||||||||||||||||||||||||||||||||||||||||||||||||||
Sbjct  121   GTCCTACGGGAGAAAGCAGGGGACCTTCGGGCCTTGCGCTATCAGATGAGCCTAGGTCGG  180

Query  187   ATTAGCTAGTTGGTGAGGTAATGGCTCACCAAGGCGACGATCCGTAACTGGTCTGAGAGG  246
             ||||||||||||||||||||||||||||||||||||||||||||||||||||||||||||
Sbjct  181   ATTAGCTAGTTGGTGAGGTAATGGCTCACCAAGGCGACGATCCGTAACTGGTCTGAGAGG  240

Query  247   ATGATCAGTCACACTGGAACTGAGACACGGTCCAGACTCCTACGGGAGGCAGCAGTGGGG  306
             ||||||||||||||||||||||||||||||||||||||||||||||||||||||||||||
Sbjct  241   ATGATCAGTCACACTGGAACTGAGACACGGTCCAGACTCCTACGGGAGGCAGCAGTGGGG  300

Query  307   AATATTGGACAATGGGCGAAAGCCTGATCCAGCCATGCCGCGTGTGTGAAGAAGGTCTTC  366
             ||||||||||||||||||||||||||||||||||||||||||||||||||||||||||||
Sbjct  301   AATATTGGACAATGGGCGAAAGCCTGATCCAGCCATGCCGCGTGTGTGAAGAAGGTCTTC  360

Query  367   GGATTGTAAAGCACTTTAAGTTGGGAGGAAGGGCAGTAAGCGAATACCTTGCTGTTTTGA  426
             ||||||||||||||||||||||||||||||||||||||||||||||||||||||||||||
Sbjct  361   GGATTGTAAAGCACTTTAAGTTGGGAGGAAGGGCAGTAAGCGAATACCTTGCTGTTTTGA  420

Query  427   CGTTACCGACAGAATAAGCACCGGCTAACTCTGTGCCAGCAGCCGCGGTAATACAGAGGG  486
             ||||||||||||||||||||||||||||||||||||||||||||||||||||||||||||
Sbjct  421   CGTTACCGACAGAATAAGCACCGGCTAACTCTGTGCCAGCAGCCGCGGTAATACAGAGGG  480

Query  487   TGCAAGCGTTAATCGGAATTACTGGGCGTAAAGCGCGCGTAGGTGGTTTGTTAAGTTGAA  546
             ||||||||||||||||||||||||||||||||||||||||||||||||||||||||||||
Sbjct  481   TGCAAGCGTTAATCGGAATTACTGGGCGTAAAGCGCGCGTAGGTGGTTTGTTAAGTTGAA  540

Query  547   TGTGAAATCCCCGGGCTCAACCTGGGAACTGCATCCAAAACTGGCAAGCTAGAGTAGGGC  606
             ||||||||||||||||||||||||||||||||||||||||||||||||||||||||||||
Sbjct  541   TGTGAAATCCCCGGGCTCAACCTGGGAACTGCATCCAAAACTGGCAAGCTAGAGTAGGGC  600

Query  607   AGAGGGTGGTGGAATTTCCTGTGTAGCGGTGAAATGCGTAGATATAGGAAGGAACACCAG  666
             ||||||||||||||||||||||||||||||||||||||||||||||||||||||||||||
Sbjct  601   AGAGGGTGGTGGAATTTCCTGTGTAGCGGTGAAATGCGTAGATATAGGAAGGAACACCAG  660

Query  667   TGGCGAAGGCGACCACCTGGGCTCATACTGACACTGAGGTGCGAAAGCGTGGGGAGCAAA  726
             ||||||||||||||||||||||||||||||||||||||||||||||||||||||||||||
Sbjct  661   TGGCGAAGGCGACCACCTGGGCTCATACTGACACTGAGGTGCGAAAGCGTGGGGAGCAAA  720

Query  727   CAGGATTAGATACCCTGGTAGTCCACGCCGTAAACGATGTCAACTAGCCGTTGGAAGCCT  786
             ||||||||||||||||||||||||||||||||||||||||||||||||||||||||||||
Sbjct  721   CAGGATTAGATACCCTGGTAGTCCACGCCGTAAACGATGTCAACTAGCCGTTGGAAGCCT  780

Query  787   TGAGCTTTTAGTGGCGCAGCTAACGCATTAAGTTGACCGCCTGGGGAGTACGGCCGCAAG  846
             ||||||||||||||||||||||||||||||||||||||||||||||||||||||||||||
Sbjct  781   TGAGCTTTTAGTGGCGCAGCTAACGCATTAAGTTGACCGCCTGGGGAGTACGGCCGCAAG  840

Query  847   GTTAAAACTCAAATGAATTGACGGGGGCCCGCACAAGCGGTGGAGCATGTGGTTTAATTC  906
             ||||||||||||||||||||||||||||||||||||||||||||||||||||||||||||
Sbjct  841   GTTAAAACTCAAATGAATTGACGGGGGCCCGCACAAGCGGTGGAGCATGTGGTTTAATTC  900

Query  907   GAAGCAACGCGAAGAACCTTACCAGGCCTTGACATCCAATGAACTTTCCAGAGATGGATT  966
             ||||||||||||||||||||||||||||||||||||||||||||||||||||||||||||
Sbjct  901   GAAGCAACGCGAAGAACCTTACCAGGCCTTGACATCCAATGAACTTTCCAGAGATGGATT  960

Query  967   GGTGCCTTCGGGAACATTGAGACAGGTGCTGCATGGCTGTCGTCAGCTCGTGTCGTG  1023
             |||||||||||||||||||||||||||||||||||||||||||||||||||||||||
Sbjct  961   GGTGCCTTCGGGAACATTGAGACAGGTGCTGCATGGCTGTCGTCAGCTCGTGTCGTG  1017
```

Download

FASTA (complete sequence)

FASTA (aligned sequences)

GenBank (complete sequence)

Continue
Cancel

GenBankGraphics

Next
Previous
Descriptions

Pseudomonas sp. TA\_GU partial 16S rRNA gene, strain TA\_GU

Sequence ID: HG942149.1Length: 1300Number of Matches: 1

Related Information

Range 1: 1 to 1017GenBankGraphics

Next Match
Previous Match
First Match

Alignment statistics for match #1

| Score | Expect | Identities | Gaps | Strand | Frame |
| --- | --- | --- | --- | --- | --- |
| 1875 bits(1015) | 0.0() | 1016/1017(99%) | 0/1017(0%) | Plus/Plus |  |

Features:

```
Query  7     TGCAGTCGAGCGGATGAAGAGAGCTTGCTNTCTGATTCAGCGGCGGACGGGTGAGTAATG  66
             ||||||||||||||||||||||||||||| ||||||||||||||||||||||||||||||
Sbjct  1     TGCAGTCGAGCGGATGAAGAGAGCTTGCTCTCTGATTCAGCGGCGGACGGGTGAGTAATG  60

Query  67    CCTAGGAATCTGCCTGGTAGTGGGGGACAACGTCTCGAAAGGGACGCTAATACCGCATAC  126
             ||||||||||||||||||||||||||||||||||||||||||||||||||||||||||||
Sbjct  61    CCTAGGAATCTGCCTGGTAGTGGGGGACAACGTCTCGAAAGGGACGCTAATACCGCATAC  120

Query  127   GTCCTACGGGAGAAAGCAGGGGACCTTCGGGCCTTGCGCTATCAGATGAGCCTAGGTCGG  186
             ||||||||||||||||||||||||||||||||||||||||||||||||||||||||||||
Sbjct  121   GTCCTACGGGAGAAAGCAGGGGACCTTCGGGCCTTGCGCTATCAGATGAGCCTAGGTCGG  180

Query  187   ATTAGCTAGTTGGTGAGGTAATGGCTCACCAAGGCGACGATCCGTAACTGGTCTGAGAGG  246
             ||||||||||||||||||||||||||||||||||||||||||||||||||||||||||||
Sbjct  181   ATTAGCTAGTTGGTGAGGTAATGGCTCACCAAGGCGACGATCCGTAACTGGTCTGAGAGG  240

Query  247   ATGATCAGTCACACTGGAACTGAGACACGGTCCAGACTCCTACGGGAGGCAGCAGTGGGG  306
             ||||||||||||||||||||||||||||||||||||||||||||||||||||||||||||
Sbjct  241   ATGATCAGTCACACTGGAACTGAGACACGGTCCAGACTCCTACGGGAGGCAGCAGTGGGG  300

Query  307   AATATTGGACAATGGGCGAAAGCCTGATCCAGCCATGCCGCGTGTGTGAAGAAGGTCTTC  366
             ||||||||||||||||||||||||||||||||||||||||||||||||||||||||||||
Sbjct  301   AATATTGGACAATGGGCGAAAGCCTGATCCAGCCATGCCGCGTGTGTGAAGAAGGTCTTC  360

Query  367   GGATTGTAAAGCACTTTAAGTTGGGAGGAAGGGCAGTAAGCGAATACCTTGCTGTTTTGA  426
             ||||||||||||||||||||||||||||||||||||||||||||||||||||||||||||
Sbjct  361   GGATTGTAAAGCACTTTAAGTTGGGAGGAAGGGCAGTAAGCGAATACCTTGCTGTTTTGA  420

Query  427   CGTTACCGACAGAATAAGCACCGGCTAACTCTGTGCCAGCAGCCGCGGTAATACAGAGGG  486
             ||||||||||||||||||||||||||||||||||||||||||||||||||||||||||||
Sbjct  421   CGTTACCGACAGAATAAGCACCGGCTAACTCTGTGCCAGCAGCCGCGGTAATACAGAGGG  480

Query  487   TGCAAGCGTTAATCGGAATTACTGGGCGTAAAGCGCGCGTAGGTGGTTTGTTAAGTTGAA  546
             ||||||||||||||||||||||||||||||||||||||||||||||||||||||||||||
Sbjct  481   TGCAAGCGTTAATCGGAATTACTGGGCGTAAAGCGCGCGTAGGTGGTTTGTTAAGTTGAA  540

Query  547   TGTGAAATCCCCGGGCTCAACCTGGGAACTGCATCCAAAACTGGCAAGCTAGAGTAGGGC  606
             ||||||||||||||||||||||||||||||||||||||||||||||||||||||||||||
Sbjct  541   TGTGAAATCCCCGGGCTCAACCTGGGAACTGCATCCAAAACTGGCAAGCTAGAGTAGGGC  600

Query  607   AGAGGGTGGTGGAATTTCCTGTGTAGCGGTGAAATGCGTAGATATAGGAAGGAACACCAG  666
             ||||||||||||||||||||||||||||||||||||||||||||||||||||||||||||
Sbjct  601   AGAGGGTGGTGGAATTTCCTGTGTAGCGGTGAAATGCGTAGATATAGGAAGGAACACCAG  660

Query  667   TGGCGAAGGCGACCACCTGGGCTCATACTGACACTGAGGTGCGAAAGCGTGGGGAGCAAA  726
             ||||||||||||||||||||||||||||||||||||||||||||||||||||||||||||
Sbjct  661   TGGCGAAGGCGACCACCTGGGCTCATACTGACACTGAGGTGCGAAAGCGTGGGGAGCAAA  720

Query  727   CAGGATTAGATACCCTGGTAGTCCACGCCGTAAACGATGTCAACTAGCCGTTGGAAGCCT  786
             ||||||||||||||||||||||||||||||||||||||||||||||||||||||||||||
Sbjct  721   CAGGATTAGATACCCTGGTAGTCCACGCCGTAAACGATGTCAACTAGCCGTTGGAAGCCT  780

Query  787   TGAGCTTTTAGTGGCGCAGCTAACGCATTAAGTTGACCGCCTGGGGAGTACGGCCGCAAG  846
             ||||||||||||||||||||||||||||||||||||||||||||||||||||||||||||
Sbjct  781   TGAGCTTTTAGTGGCGCAGCTAACGCATTAAGTTGACCGCCTGGGGAGTACGGCCGCAAG  840

Query  847   GTTAAAACTCAAATGAATTGACGGGGGCCCGCACAAGCGGTGGAGCATGTGGTTTAATTC  906
             ||||||||||||||||||||||||||||||||||||||||||||||||||||||||||||
Sbjct  841   GTTAAAACTCAAATGAATTGACGGGGGCCCGCACAAGCGGTGGAGCATGTGGTTTAATTC  900

Query  907   GAAGCAACGCGAAGAACCTTACCAGGCCTTGACATCCAATGAACTTTCCAGAGATGGATT  966
             ||||||||||||||||||||||||||||||||||||||||||||||||||||||||||||
Sbjct  901   GAAGCAACGCGAAGAACCTTACCAGGCCTTGACATCCAATGAACTTTCCAGAGATGGATT  960

Query  967   GGTGCCTTCGGGAACATTGAGACAGGTGCTGCATGGCTGTCGTCAGCTCGTGTCGTG  1023
             |||||||||||||||||||||||||||||||||||||||||||||||||||||||||
Sbjct  961   GGTGCCTTCGGGAACATTGAGACAGGTGCTGCATGGCTGTCGTCAGCTCGTGTCGTG  1017
```

```

```


BLAST is a registered trademark of the National Library of Medicine

Support center
Mailing list


YouTube

- National Library Of Medicine
- National Institutes Of Health
- U.S. Department of Health & Human Services
- USA.gov

### NCBI


National Center for Biotechnology Information,
 U.S. National Library of Medicine

8600 Rockville Pike,
Bethesda
 MD,
20894
USA

Policies and Guidelines
|
Contact


PreferencesTurn off

External link. Please review our privacy policy.
