# Supplementary material for: Isothermal microcalorimetry for thermal viable count of microorganisms in pure cultures and stabilized formulations
Source: BMC Microbiol. 2019 Mar 21;19:65. doi: 10.1186/s12866-019-1432-8 (PMC6429831; doi:10.1186/s12866-019-1432-8)
Supplement: Supplementary file 4 — Figure S1. Pseudomonas cultured in IMC vials. Figure S2. Coated seeds in IMC vials. Figure S3. Bacillus cultured in IMC vials. Figure S4. Clonostachys cultured in IMC vials. (DOCX 1472 kb) [file 12866_2019_1432_MOESM4_ESM.docx]

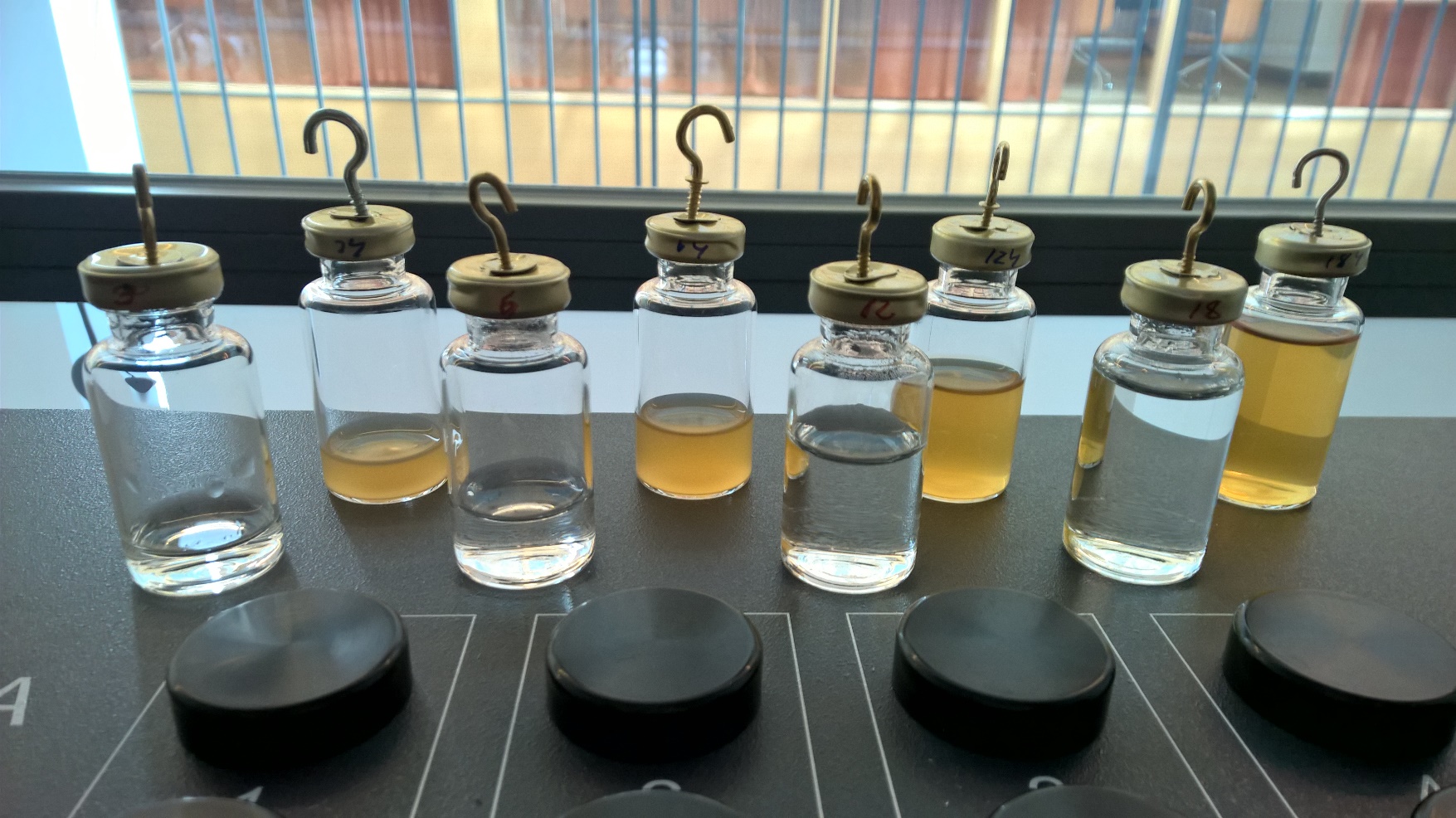


Figure S1. Different volumes of *Pseudomonas brassicacearum* MA250 cultures after 28 h IMC measurements in 25°C and respective inert controls (ddH_2_O).


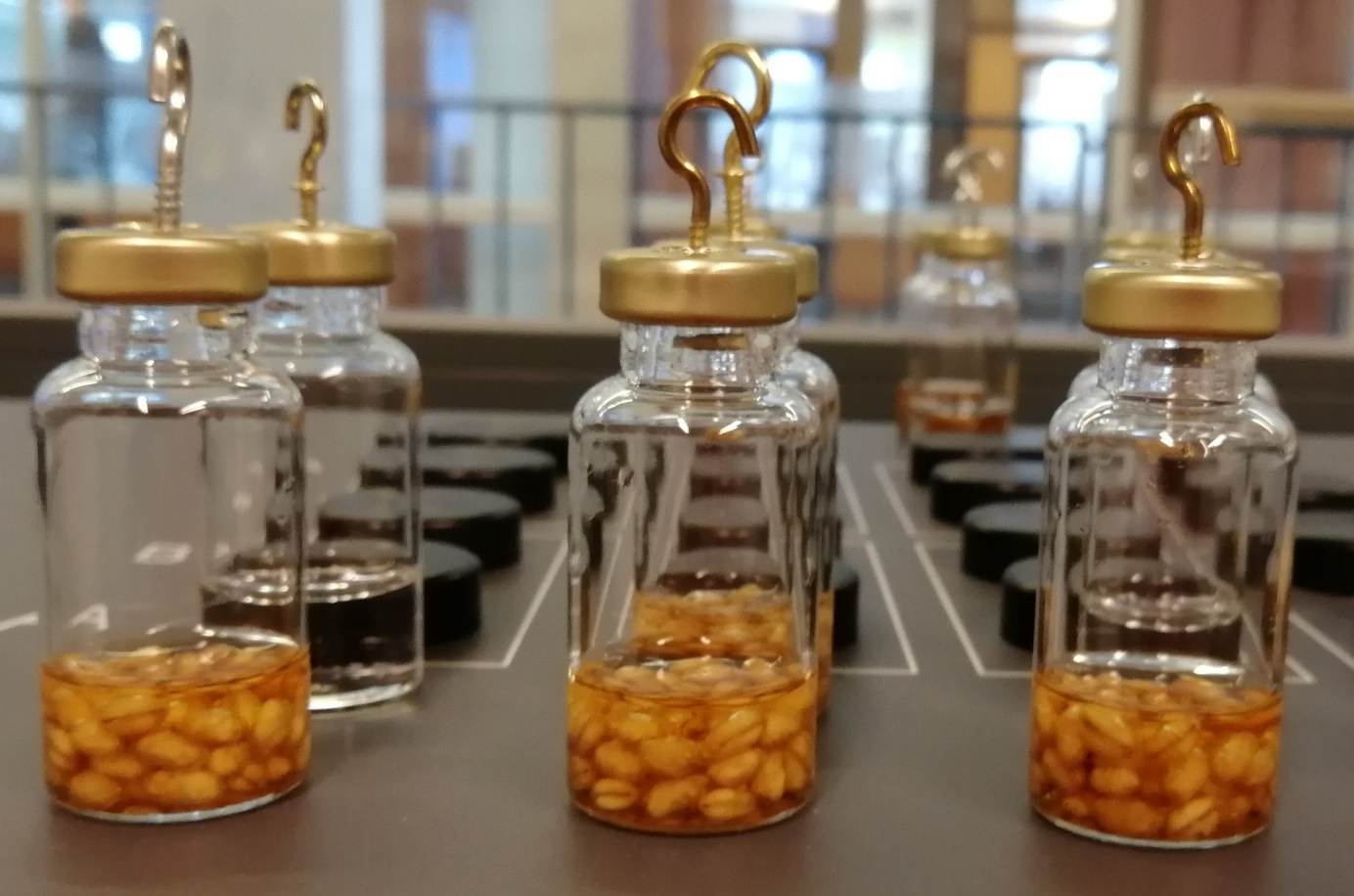


Figure S2. Coated wheat seeds in IMC vials.


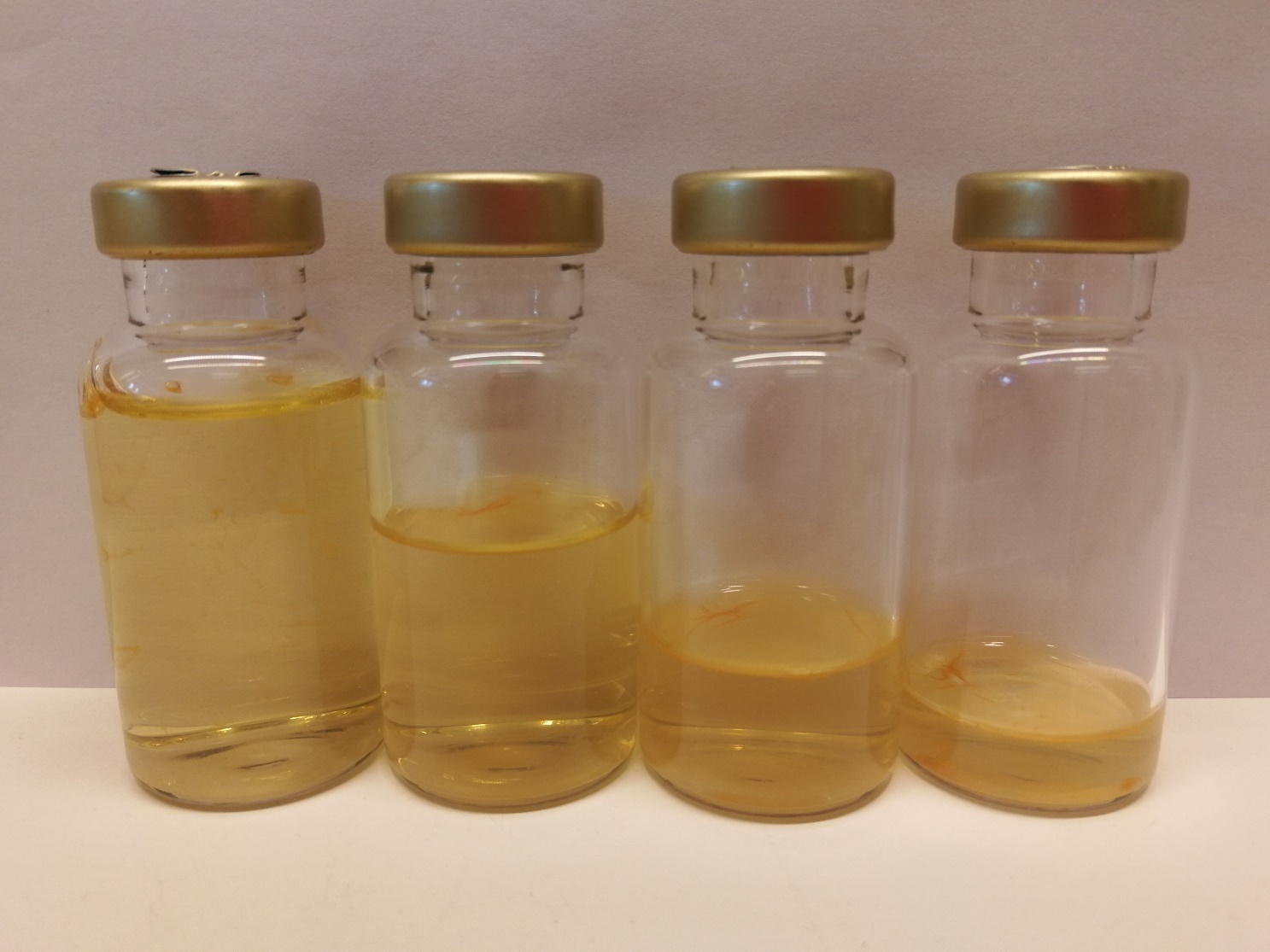


Figure S3. Different volumes of *Bacillus amyloliquefaciens* subsp. *plantarum* UCMB5113 cultures after 42 h IMC measurements in 25°C. *Bacillus* could be seen as orange rafts at the surface.


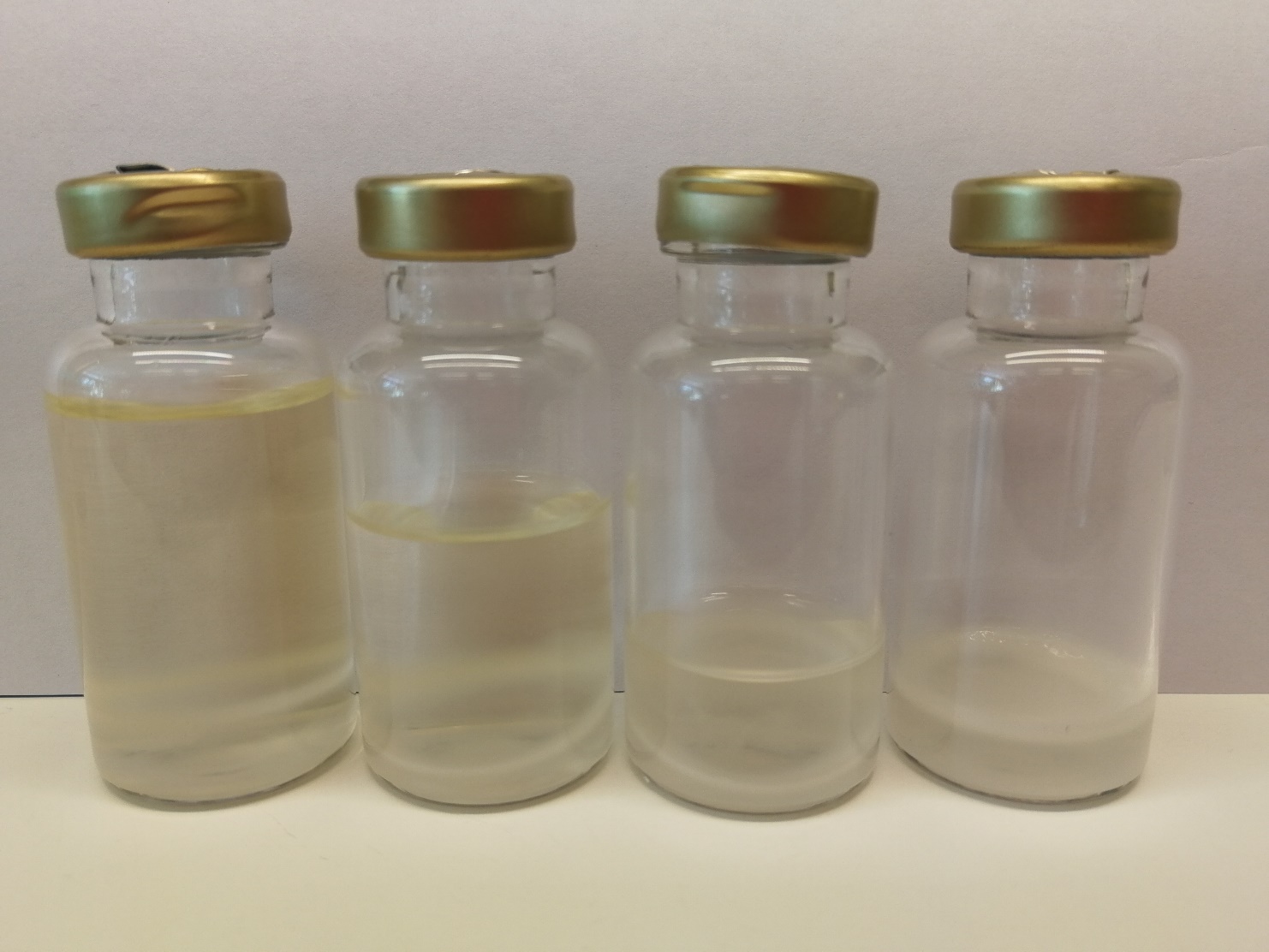


Figure S4. Different volumes of *Clonostachys rosea* IK726 cultures after 100 h IMC measurements in 25°C. *Clonostachys* could be seen as white rings within vials. Rings generated to the surfaces (1^st^ vial on right) and dropped to the bottom after handling the vials.
