# Supplementary material for: Isothermal microcalorimetry for thermal viable count of microorganisms in pure cultures and stabilized formulations
Source: BMC Microbiol. 2019 Mar 21;19:65. doi: 10.1186/s12866-019-1432-8 (PMC6429831; doi:10.1186/s12866-019-1432-8)
Supplement: Supplementary file 3 — 16S rRNA gene sequences’ BLASTN hits in zipped HTML format. (ZIP 15810 kb) [file 12866_2019_1432_MOESM3_ESM.zip › Best blastn hits/NCBI Blast_45 small negative R -- 9..879 of sequence.html]

NCBI Blast:45 small negative R -- 9..879 of sequence


- NCBI Home
- Sign in to NCBI
- Skip to Main Content
- Skip to Navigation
- About NCBI Accesskeys

U.S. National Library of Medicine

NCBI
National Center for Biotechnology Information

- My NCBI
- Sign in to NCBI
- Register
- Sign Out

BLAST ^®^ » blastn suite » RID-A4W6UU4G014


- Home
- Recent Results
- Saved Strategies
- Help

BLAST Results


Edit and Resubmit
Save Search Strategies
[Sign in above to save your search strategy]

Formatting options 


Download


How to read this page
Blast report description
Questions/comments


|  |  |
| --- | --- |
| Formatting options | |
| Show | Alignment as  HTML Plain text   Old View Reset form to defaults [?]  These options control formatting of alignments in results pages. The default is HTML, but other formats (including plain text) are available. PSSM and PssmWithParameters are representations of Position Specific Scoring Matrices and are only available for PSI-BLAST. The Advanced view option allows the database descriptions to be sorted by various indices in a table. |
| Alignment View | Pairwise Pairwise with dots for identities Query-anchored with dots for identities Query-anchored with letters for identities Flat query-anchored with dots for identities Flat query-anchored with letters for identities [?]  Choose how to view alignments. The default "pairwise" view shows how each subject sequence aligns individually to the query sequence. The "query-anchored" view shows how all subject sequences align to the query sequence. For each view type, you can choose to show "identities" (matching residues) as letters or dots. more... |
| Display | Graphical Overview   Linkout   Sequence Retrieval  NCBI-gi   CDS feature [?]  - Graphical Overview: Graphical Overview: Show graph of similar sequence regions aligned to query.   more... - NCBI-gi: Show NCBI gi identifiers. - CDS feature: Show annotated coding region and translation.   more... |
| Masking | Character:   X for protein, n for nucleotide Lower Case  Color:  Black Grey Red [?]  - Masking Character: Display masked (filtered) sequence regions as lower-case or as specific letters (N for nucleotide, P for protein). - Masking Color: Display masked sequence regions in the given color. |
| Limit results | Descriptions:  10 50 100 Graphical overview:  0 10 50 100  Alignments:  0 10 50 100 Line length:  60 90 120 150 [?]  - Descriptions: Show short descriptions for up to the given number of sequences. - Alignments: Show alignments for up to the given number of sequences, in order of statistical significance. - Line lenghth: Number of letters to show on one line in an alignment. |
|  | Organism Type common name, binomial, taxid, or group name. Only 20 top taxa will be shown.     Exclude   [?]  Show only sequences from the given organism. |
|  | Entrez query:  [?]  Show only those sequences that match the given Entrez query. more... |
|  | Expect Min:  Expect Max:  [?]  Show only sequences with expect values in the given range. more... |
|  | Percent Identity Min:  Percent Identity Max:  [?]  Show only sequences with percent identity values in the given range. |
| Format for | PSI-BLAST with inclusion threshold:  [?]  - Format for PSI-BLAST: The Position-Specific Iterated BLAST (PSI-BLAST) program performs iterative searches with a protein query,   in which sequences found in one round of search are used to build a custom score model for the next round.   more... - Inclusion Threshold: This sets the statistical significance threshold for including a sequence in the model used   by PSI-BLAST to create the PSSM on the next iteration. |

|  |  |  |  |  |  |
| --- | --- | --- | --- | --- | --- |
| Download | | | | | |
| Alignment  Text XML ASN.1 JSON Seq-align Hit Table(text) Hit Table(csv) Multiple-file XML2 Single-file XML2 Multiple-file JSON Single-file JSON SAM | Search Strategies  ASN.1 | PSSM to restart search  PSSM | [?] |  |  |

The Download link provides BLAST output that may be used as input to another program.
This includes parseable formats such as the tabular report or XML as well as the Search Strategy files read by the BLAST+ applications.
More details on the parseable (XML, tabular, and ASN.1) reports can be found at
https://www.ncbi.nlm.nih.gov/books/NBK153387/  
  

The following formats are offered under the Alignment section:  
1). "Text". Non-HTML standard BLAST report.  
2). "XML". XML report based upon the DTD at https://www.ncbi.nlm.nih.gov/data\_specs/dtd/NCBI\_BlastOutput.dtd  
3). "ASN.1". Alignment written out in Abstract Syntax Notation 1.  
4). "JSON Seq-align". Alignment written out in JSON.  
4). "Hit Table(text)". The tabular report as text.  
5). "Hit Table(csv)". The tabular report ready for import into spread-sheet programs like Excel.  
6). "XML2". New XML format described at ftp://ftp.ncbi.nlm.nih.gov/blast/documents/NEWXML/xml2.pdf.  
7). "JSON". New JSON format described at ftp://ftp.ncbi.nlm.nih.gov/blast/documents/NEWXML/xml2.pdf.  
8). "SAM". Sequence Alignment Map format.

XML2 and JSON can be downloaded either as one file per query (multiple-file) or one file for all queries (single-file). These formats are listed as Multiple-file XML2 (and JSON) or Single-file XML (and JSON).

The following report is offered under the Search Strategy section:  
1). "ASN.1" Search Strategy. A record of the parameters, query, and database used in the search. This file can be used to start a stand-alone BLAST search, see
https://www.ncbi.nlm.nih.gov/books/NBK1763/#CmdLineAppsManual.I455\_BLAST\_search\_stra


# Job title: 45 small negative R -- 9..879 of sequence

Results for:

lcl|Query\_182559 45 small negative R -- 9..879 of sequence(871bp)
[?]

Your BLAST job specified more than one input sequence.
This box lets you choose which input sequence to show BLAST results for.

RID
:   A4W6UU4G014 (Expires on 03-10 18:32 pm)

Query ID
:   lcl|Query\_182559
:   lcl|Query\_182559

Description
:   45 small negative R -- 9..879 of sequence

Molecule type
:   nucleic acid

Query Length
:   871

Database Name
:   nr

Description
:   Nucleotide collection (nt) See details

Program
:   BLASTN 2.8.0+ Citation

  

Reference 

Zheng Zhang, Scott Schwartz, Lukas Wagner, and Webb Miller (2000), "A greedy algorithm for aligning DNA sequences", J Comput Biol 2000; 7(1-2):203-14.

Reference - database indexing

Aleksandr Morgulis, George Coulouris, Yan Raytselis, Thomas L. Madden, Richa Agarwala, Alejandro A. Schäffer (2008), "Database Indexing for Production MegaBLAST Searches", Bioinformatics 24:1757-1764.

Other reports:
Search Summary

[Taxonomy reports]
[Distance tree of results]
[MSA viewer]

Search Parameters

| Search parameter name | Search parameter value |
| --- | --- |
| Program | blastn |
| Word size | 28 |
| Expect value | 10 |
| Hitlist size | 100 |
| Match/Mismatch scores | 1,-2 |
| Gapcosts | 0,2.5 |
| Low Complexity Filter | Yes |
| Filter string | L;m; |
| Genetic Code | 1 |

Database

| Database parameter name | Database parameter value |
| --- | --- |
| Posted date | Mar 3, 2018 9:32 PM |
| Number of letters | 171,598,911,005 |
| Number of sequences | 46,870,701 |
| Entrez query | Includes:  Excludes:  None |

Karlin-Altschul statistics

| Params | Ungapped | Gapped |
| --- | --- | --- |
| Lambda | 1.33271 | 1.28 |
| K | 0.620991 | 0.46 |
| H | 1.12409 | 0.85 |

Results Statistics

| Results Statistics parameter name | Results Statistics parameter value |
| --- | --- |
| Length adjustment | 35 |
| Effective length of query | 836 |
| Effective length of database | 169958436470 |
| Effective search space | 142085252888920 |
| Effective search space used | 142085252888920 |


## Graphic Summary

### Distribution of the top 105 Blast Hits on 100 subject sequences [?]

The graphic is an overview of the database sequences aligned to the query sequence. These are represented horizontal bars colored coded by score and showing the extent
of the alignment on the query sequence. Separate aligned regions on the same database sequence are connected by a thin grey line.
Mousing over an alignment shows the database sequence title. Clicking an alignment displays a box with more details about the alignment and
link to the sequence alignment itself in the Alignments section of the report.

Mouse over to see the title, click to show alignments

Color key for alignment scores

<40

40-50

50-80

80-200

>=200

Query

1

150

300

450

600

750

Pseudomonas sp. strain CF24P20 16S ribosomal RNA gene, ..

Score:1568 Evalue:0

Accession:MG860023.1

Alignment

Pseudomonas sp. strain MSM-2-10-15 16S ribosomal RNA ge..

Score:1568 Evalue:0

Accession:KY907017.1

Alignment

Pseudomonas sp. strain BAV3487 16S ribosomal RNA gene, ..

Score:1568 Evalue:0

Accession:KY074356.1

Alignment

Pseudomonas sp. strain BAV2562 16S ribosomal RNA gene, ..

Score:1568 Evalue:0

Accession:KY073974.1

Alignment

Pseudomonas graminis strain R5SpM3P2C1 16S ribosomal RN..

Score:1568 Evalue:0

Accession:KF147092.1

Alignment

Pseudomonas graminis strain R5SsM3P2C7 16S ribosomal RN..

Score:1568 Evalue:0

Accession:KF147091.1

Alignment

Pseudomonas graminis strain R1SpM3P3C4 16S ribosomal RN..

Score:1568 Evalue:0

Accession:KF147090.1

Alignment

Pseudomonas graminis strain R1SpM3P1C6 16S ribosomal RN..

Score:1568 Evalue:0

Accession:KF147089.1

Alignment

Pseudomonas sp. E9 partial 16S rRNA gene, isolate E9

Score:1568 Evalue:0

Accession:HE652089.1

Alignment

Pseudomonas graminis partial 16S rRNA gene, isolate Pcl..

Score:1563 Evalue:0

Accession:LN852371.1

Alignment

Pseudomonas sp. strain BAV2715 16S ribosomal RNA gene, ..

Score:1563 Evalue:0

Accession:KY073983.1

Alignment

Pseudomonas sp. strain BAV2549 16S ribosomal RNA gene, ..

Score:1563 Evalue:0

Accession:KY073955.1

Alignment

Pseudomonas graminis strain PDD-59b-25 16S ribosomal RN..

Score:1563 Evalue:0

Accession:KR922157.1

Alignment

Pseudomonas graminis strain EB25 16S ribosomal RNA gene..

Score:1563 Evalue:0

Accession:KP209394.1

Alignment

Pseudomonas graminis strain 8B2 16S ribosomal RNA gene,..

Score:1563 Evalue:0

Accession:KJ589448.1

Alignment

Pseudomonas graminis strain QWKB2 16S ribosomal RNA gen..

Score:1563 Evalue:0

Accession:KJ589434.1

Alignment

Pseudomonas graminis strain CKB2 16S ribosomal RNA gene..

Score:1563 Evalue:0

Accession:KJ589428.1

Alignment

Pseudomonas sp. PN829.3 16S ribosomal RNA gene, partial..

Score:1563 Evalue:0

Accession:KC822794.1

Alignment

Pseudomonas sp. R1SsM3P3C4 16S ribosomal RNA gene, part..

Score:1563 Evalue:0

Accession:KF147102.1

Alignment

Pseudomonas sp. R1SpM3P3C2 16S ribosomal RNA gene, part..

Score:1563 Evalue:0

Accession:KF147101.1

Alignment

Pseudomonas sp. R1SsM3P2C4 16S ribosomal RNA gene, part..

Score:1563 Evalue:0

Accession:KF147100.1

Alignment

Pseudomonas sp. R1SpM3P2C4 16S ribosomal RNA gene, part..

Score:1563 Evalue:0

Accession:KF147099.1

Alignment

Pseudomonas sp. R5SsM3P3C9 16S ribosomal RNA gene, part..

Score:1563 Evalue:0

Accession:KF147098.1

Alignment

Pseudomonas sp. R2SpM3P2C5 16S ribosomal RNA gene, part..

Score:1563 Evalue:0

Accession:KF147097.1

Alignment

Pseudomonas graminis strain R2SsM3P1C4 16S ribosomal RN..

Score:1563 Evalue:0

Accession:KF147094.1

Alignment

Pseudomonas graminis strain R2SsM3P1C3 16S ribosomal RN..

Score:1563 Evalue:0

Accession:KF147093.1

Alignment

Pseudomonas graminis strain R2SpM3P2C3 16S ribosomal RN..

Score:1563 Evalue:0

Accession:KF147086.1

Alignment

Pseudomonas sp. isolate PS3.1 16S ribosomal RNA gene, p..

Score:1563 Evalue:0

Accession:KF019109.1

Alignment

Pseudomonas sp. SAP829.3 16S ribosomal RNA gene, partia..

Score:1563 Evalue:0

Accession:JX067709.1

Alignment

Pseudomonas sp. SAP707.1 16S ribosomal RNA gene, partia..

Score:1563 Evalue:0

Accession:JX067671.1

Alignment

Bacterium OC34(2011) 16S ribosomal RNA gene, partial se..

Score:1563 Evalue:0

Accession:HQ179006.1

Alignment

Pseudomonas graminis strain RMPP6 16S ribosomal RNA gen..

Score:1563 Evalue:0

Accession:GU396281.1

Alignment

Uncultured bacterium partial 16S rRNA gene, isolate BF0..

Score:1563 Evalue:0

Accession:AM697261.1

Alignment

Pseudomonas graminis strain DSM 11363 16S ribosomal RNA..

Score:1563 Evalue:0

Accession:NR\_026395.1

Alignment

Pseudomonas sp. HP7D 16S ribosomal RNA gene, partial se..

Score:1559 Evalue:0

Accession:KM187516.1

Alignment

Uncultured bacterium clone HL201309-60 16S ribosomal RN..

Score:1557 Evalue:0

Accession:KU515434.1

Alignment

Uncultured bacterium gene for 16S rRNA, partial sequenc..

Score:1557 Evalue:0

Accession:AB990781.1

Alignment

Pseudomonas graminis strain PE24 16S ribosomal RNA gene..

Score:1557 Evalue:0

Accession:KJ127243.1

Alignment

Bacterium 3t 16S ribosomal RNA gene, partial sequence

Score:1557 Evalue:0

Accession:JX042421.1

Alignment

Pseudomonas graminis strain R2SsM3P1C6 16S ribosomal RN..

Score:1557 Evalue:0

Accession:KF147083.1

Alignment

Pseudomonas graminis strain R2SpM3P1C2 16S ribosomal RN..

Score:1557 Evalue:0

Accession:KF147081.1

Alignment

Pseudomonas graminis strain R3ScM3P1C4 16S ribosomal RN..

Score:1557 Evalue:0

Accession:KF147078.1

Alignment

Pseudomonas sp. isolate PS4.1 16S ribosomal RNA gene, p..

Score:1557 Evalue:0

Accession:KF019107.1

Alignment

Pseudomonas sp. Axa8 16S ribosomal RNA gene, partial se..

Score:1557 Evalue:0

Accession:JQ977394.1

Alignment

Pseudomonas sp. Na1 16S ribosomal RNA gene, partial seq..

Score:1557 Evalue:0

Accession:GU391488.1

Alignment

Pseudomonas lutea strain OK2 16S ribosomal RNA, partial..

Score:1557 Evalue:0

Accession:NR\_029103.1

Alignment

Pseudomonas sp. Enf30 16S ribosomal RNA gene, partial s..

Score:1557 Evalue:0

Accession:DQ339606.1

Alignment

Pseudomonas sp. strain BAV2862 16S ribosomal RNA gene, ..

Score:1555 Evalue:0

Accession:KY074040.1

Alignment

Pseudomonas sp. C-3-38 16S ribosomal RNA gene, partial ..

Score:1555 Evalue:0

Accession:KT583556.1

Alignment

Pseudomonas graminis strain R2SsM3P1C5 16S ribosomal RN..

Score:1555 Evalue:0

Accession:KF147079.1

Alignment

Pseudomonas graminis strain R1SpM3P3C3 16S ribosomal RN..

Score:1555 Evalue:0

Accession:KF147064.1

Alignment

Pseudomonas graminis isolate HhSaOsb 16S ribosomal RNA ..

Score:1555 Evalue:0

Accession:AF511514.1

Alignment

Pseudomonas graminis strain R2SsM3P2C4 16S ribosomal RN..

Score:1552 Evalue:0

Accession:KF147080.1

Alignment

Pseudomonas graminis strain R2SpM3P3C1 16S ribosomal RN..

Score:1552 Evalue:0

Accession:KF147077.1

Alignment

Pseudomonas sp. E06 16S ribosomal RNA gene, partial seq..

Score:1552 Evalue:0

Accession:JQ977048.1

Alignment

Pseudomonas graminis strain EQH15 16S ribosomal RNA gen..

Score:1552 Evalue:0

Accession:FJ999944.1

Alignment

## Descriptions

, Reading indexes 1-5, displaying indexes 1-5


Load next setPrevious Match

Sequences producing significant alignments:

Show all columns  of the table presenting sequences producing significant alignments 

Select:AllNone
Selected:0

Alignments
Download

FASTA (complete sequence)

FASTA (aligned sequences)

GenBank (complete sequence)

Hit Table (text)

Hit Table (CSV)

Text

XML

ASN.1

Continue
Cancel

GenBank 
Graphics
Distance tree of results
Multiple alignment
Show/hide columns of the table presenting sequences producing significant alignments 

Available columns

Description  
Max Score  
Total Score  
Coverage  
E-value  
IdentN  
Accession  
Restore Defaults
Ok
Cancel

Sequences producing significant alignments:

| Select for downloading or viewing reports | Description | Max score | Total score | Query cover | E value | Ident | Accession |
| --- | --- | --- | --- | --- | --- | --- | --- |
| 1Select seq MG860023.1 | Pseudomonas sp. strain CF24P20 16S ribosomal RNA gene, partial sequence | 1568 | 1568 | 99% | 0.0 | 99% | MG860023.1 |
| 2Select seq KY907017.1 | Pseudomonas sp. strain MSM-2-10-15 16S ribosomal RNA gene, partial sequence | 1568 | 1568 | 99% | 0.0 | 99% | KY907017.1 |
| 3Select seq KY074356.1 | Pseudomonas sp. strain BAV3487 16S ribosomal RNA gene, partial sequence | 1568 | 1568 | 99% | 0.0 | 99% | KY074356.1 |
| 4Select seq KY073974.1 | Pseudomonas sp. strain BAV2562 16S ribosomal RNA gene, partial sequence | 1568 | 1568 | 99% | 0.0 | 99% | KY073974.1 |
| 5Select seq KF147092.1 | Pseudomonas graminis strain R5SpM3P2C1 16S ribosomal RNA gene, partial sequence | 1568 | 1568 | 99% | 0.0 | 99% | KF147092.1 |
| 6Select seq KF147091.1 | Pseudomonas graminis strain R5SsM3P2C7 16S ribosomal RNA gene, partial sequence | 1568 | 1568 | 99% | 0.0 | 99% | KF147091.1 |
| 7Select seq KF147090.1 | Pseudomonas graminis strain R1SpM3P3C4 16S ribosomal RNA gene, partial sequence | 1568 | 1568 | 99% | 0.0 | 99% | KF147090.1 |
| 8Select seq KF147089.1 | Pseudomonas graminis strain R1SpM3P1C6 16S ribosomal RNA gene, partial sequence | 1568 | 1568 | 99% | 0.0 | 99% | KF147089.1 |
| 9Select seq HE652089.1 | Pseudomonas sp. E9 partial 16S rRNA gene, isolate E9 | 1568 | 1568 | 99% | 0.0 | 99% | HE652089.1 |
| 10Select seq LN852371.1 | Pseudomonas graminis partial 16S rRNA gene, isolate PclHEPES2 | 1563 | 1563 | 98% | 0.0 | 99% | LN852371.1 |
| 11Select seq KY073983.1 | Pseudomonas sp. strain BAV2715 16S ribosomal RNA gene, partial sequence | 1563 | 1563 | 99% | 0.0 | 99% | KY073983.1 |
| 12Select seq KY073955.1 | Pseudomonas sp. strain BAV2549 16S ribosomal RNA gene, partial sequence | 1563 | 1563 | 99% | 0.0 | 99% | KY073955.1 |
| 13Select seq KR922157.1 | Pseudomonas graminis strain PDD-59b-25 16S ribosomal RNA gene, complete sequence | 1563 | 1563 | 98% | 0.0 | 99% | KR922157.1 |
| 14Select seq KP209394.1 | Pseudomonas graminis strain EB25 16S ribosomal RNA gene, partial sequence | 1563 | 1563 | 99% | 0.0 | 99% | KP209394.1 |
| 15Select seq KJ589448.1 | Pseudomonas graminis strain 8B2 16S ribosomal RNA gene, partial sequence | 1563 | 1563 | 99% | 0.0 | 99% | KJ589448.1 |
| 16Select seq KJ589434.1 | Pseudomonas graminis strain QWKB2 16S ribosomal RNA gene, partial sequence | 1563 | 1563 | 99% | 0.0 | 99% | KJ589434.1 |
| 17Select seq KJ589428.1 | Pseudomonas graminis strain CKB2 16S ribosomal RNA gene, partial sequence | 1563 | 1563 | 99% | 0.0 | 99% | KJ589428.1 |
| 18Select seq KC822794.1 | Pseudomonas sp. PN829.3 16S ribosomal RNA gene, partial sequence | 1563 | 1563 | 99% | 0.0 | 99% | KC822794.1 |
| 19Select seq KF147102.1 | Pseudomonas sp. R1SsM3P3C4 16S ribosomal RNA gene, partial sequence | 1563 | 1563 | 99% | 0.0 | 99% | KF147102.1 |
| 20Select seq KF147101.1 | Pseudomonas sp. R1SpM3P3C2 16S ribosomal RNA gene, partial sequence | 1563 | 1563 | 99% | 0.0 | 99% | KF147101.1 |
| 21Select seq KF147100.1 | Pseudomonas sp. R1SsM3P2C4 16S ribosomal RNA gene, partial sequence | 1563 | 1563 | 99% | 0.0 | 99% | KF147100.1 |
| 22Select seq KF147099.1 | Pseudomonas sp. R1SpM3P2C4 16S ribosomal RNA gene, partial sequence | 1563 | 1563 | 99% | 0.0 | 99% | KF147099.1 |
| 23Select seq KF147098.1 | Pseudomonas sp. R5SsM3P3C9 16S ribosomal RNA gene, partial sequence | 1563 | 1563 | 99% | 0.0 | 99% | KF147098.1 |
| 24Select seq KF147097.1 | Pseudomonas sp. R2SpM3P2C5 16S ribosomal RNA gene, partial sequence | 1563 | 1563 | 99% | 0.0 | 99% | KF147097.1 |
| 25Select seq KF147094.1 | Pseudomonas graminis strain R2SsM3P1C4 16S ribosomal RNA gene, partial sequence | 1563 | 1563 | 99% | 0.0 | 99% | KF147094.1 |
| 26Select seq KF147093.1 | Pseudomonas graminis strain R2SsM3P1C3 16S ribosomal RNA gene, partial sequence | 1563 | 1563 | 99% | 0.0 | 99% | KF147093.1 |
| 27Select seq KF147086.1 | Pseudomonas graminis strain R2SpM3P2C3 16S ribosomal RNA gene, partial sequence | 1563 | 1563 | 99% | 0.0 | 99% | KF147086.1 |
| 28Select seq KF019109.1 | Pseudomonas sp. isolate PS3.1 16S ribosomal RNA gene, partial sequence | 1563 | 1563 | 98% | 0.0 | 99% | KF019109.1 |
| 29Select seq JX067709.1 | Pseudomonas sp. SAP829.3 16S ribosomal RNA gene, partial sequence | 1563 | 1563 | 99% | 0.0 | 99% | JX067709.1 |
| 30Select seq JX067671.1 | Pseudomonas sp. SAP707.1 16S ribosomal RNA gene, partial sequence | 1563 | 1563 | 99% | 0.0 | 99% | JX067671.1 |
| 31Select seq HQ179006.1 | Bacterium OC34(2011) 16S ribosomal RNA gene, partial sequence | 1563 | 1563 | 99% | 0.0 | 99% | HQ179006.1 |
| 32Select seq GU396281.1 | Pseudomonas graminis strain RMPP6 16S ribosomal RNA gene, partial sequence | 1563 | 1563 | 99% | 0.0 | 99% | GU396281.1 |
| 33Select seq AM697261.1 | Uncultured bacterium partial 16S rRNA gene, isolate BF0001C102 | 1563 | 1563 | 99% | 0.0 | 99% | AM697261.1 |
| 34Select seq NR\_026395.1 | Pseudomonas graminis strain DSM 11363 16S ribosomal RNA, partial sequence | 1563 | 1563 | 99% | 0.0 | 99% | NR\_026395.1 |
| 35Select seq KM187516.1 | Pseudomonas sp. HP7D 16S ribosomal RNA gene, partial sequence | 1559 | 1559 | 98% | 0.0 | 99% | KM187516.1 |
| 36Select seq KU515434.1 | Uncultured bacterium clone HL201309-60 16S ribosomal RNA gene, partial sequence | 1557 | 1557 | 99% | 0.0 | 99% | KU515434.1 |
| 37Select seq AB990781.1 | Uncultured bacterium gene for 16S rRNA, partial sequence, clone: Ms-55-St2mBy-2-065 | 1557 | 1557 | 99% | 0.0 | 99% | AB990781.1 |
| 38Select seq KJ127243.1 | Pseudomonas graminis strain PE24 16S ribosomal RNA gene, partial sequence | 1557 | 1557 | 99% | 0.0 | 99% | KJ127243.1 |
| 39Select seq JX042421.1 | Bacterium 3t 16S ribosomal RNA gene, partial sequence | 1557 | 1557 | 98% | 0.0 | 99% | JX042421.1 |
| 40Select seq KF147083.1 | Pseudomonas graminis strain R2SsM3P1C6 16S ribosomal RNA gene, partial sequence | 1557 | 1557 | 99% | 0.0 | 99% | KF147083.1 |
| 41Select seq KF147081.1 | Pseudomonas graminis strain R2SpM3P1C2 16S ribosomal RNA gene, partial sequence | 1557 | 1557 | 99% | 0.0 | 99% | KF147081.1 |
| 42Select seq KF147078.1 | Pseudomonas graminis strain R3ScM3P1C4 16S ribosomal RNA gene, partial sequence | 1557 | 1557 | 99% | 0.0 | 99% | KF147078.1 |
| 43Select seq KF019107.1 | Pseudomonas sp. isolate PS4.1 16S ribosomal RNA gene, partial sequence | 1557 | 1557 | 98% | 0.0 | 99% | KF019107.1 |
| 44Select seq JQ977394.1 | Pseudomonas sp. Axa8 16S ribosomal RNA gene, partial sequence | 1557 | 1557 | 98% | 0.0 | 99% | JQ977394.1 |
| 45Select seq GU391488.1 | Pseudomonas sp. Na1 16S ribosomal RNA gene, partial sequence | 1557 | 1557 | 99% | 0.0 | 99% | GU391488.1 |
| 46Select seq NR\_029103.1 | Pseudomonas lutea strain OK2 16S ribosomal RNA, partial sequence | 1557 | 1557 | 99% | 0.0 | 99% | NR\_029103.1 |
| 47Select seq DQ339606.1 | Pseudomonas sp. Enf30 16S ribosomal RNA gene, partial sequence | 1557 | 1557 | 99% | 0.0 | 99% | DQ339606.1 |
| 48Select seq KY074040.1 | Pseudomonas sp. strain BAV2862 16S ribosomal RNA gene, partial sequence | 1555 | 1555 | 99% | 0.0 | 99% | KY074040.1 |
| 49Select seq KT583556.1 | Pseudomonas sp. C-3-38 16S ribosomal RNA gene, partial sequence | 1555 | 1555 | 98% | 0.0 | 99% | KT583556.1 |
| 50Select seq KF147079.1 | Pseudomonas graminis strain R2SsM3P1C5 16S ribosomal RNA gene, partial sequence | 1555 | 1555 | 98% | 0.0 | 99% | KF147079.1 |
| 51Select seq KF147064.1 | Pseudomonas graminis strain R1SpM3P3C3 16S ribosomal RNA gene, partial sequence | 1555 | 1555 | 98% | 0.0 | 99% | KF147064.1 |
| 52Select seq AF511514.1 | Pseudomonas graminis isolate HhSaOsb 16S ribosomal RNA gene, partial sequence | 1555 | 1555 | 99% | 0.0 | 99% | AF511514.1 |
| 53Select seq KF147080.1 | Pseudomonas graminis strain R2SsM3P2C4 16S ribosomal RNA gene, partial sequence | 1552 | 1552 | 98% | 0.0 | 99% | KF147080.1 |
| 54Select seq KF147077.1 | Pseudomonas graminis strain R2SpM3P3C1 16S ribosomal RNA gene, partial sequence | 1552 | 1552 | 98% | 0.0 | 99% | KF147077.1 |
| 55Select seq JQ977048.1 | Pseudomonas sp. E06 16S ribosomal RNA gene, partial sequence | 1552 | 1552 | 98% | 0.0 | 99% | JQ977048.1 |
| 56Select seq FJ999944.1 | Pseudomonas graminis strain EQH15 16S ribosomal RNA gene, partial sequence | 1552 | 1552 | 99% | 0.0 | 99% | FJ999944.1 |
| 57Select seq DQ339614.1 | Pseudomonas sp. Enf53 16S ribosomal RNA gene, partial sequence | 1552 | 1552 | 99% | 0.0 | 99% | DQ339614.1 |
| 58Select seq DQ339611.1 | Pseudomonas sp. Enf26 16S ribosomal RNA gene, partial sequence | 1552 | 1552 | 99% | 0.0 | 99% | DQ339611.1 |
| 59Select seq DQ339600.1 | Pseudomonas sp. Enf34 16S ribosomal RNA gene, partial sequence | 1552 | 1552 | 99% | 0.0 | 99% | DQ339600.1 |
| 60Select seq DQ339586.1 | Pseudomonas sp. Enf5 16S ribosomal RNA gene, partial sequence | 1552 | 1552 | 99% | 0.0 | 99% | DQ339586.1 |
| 61Select seq KY074010.1 | Pseudomonas graminis strain BAV2708 16S ribosomal RNA gene, partial sequence | 1550 | 1550 | 98% | 0.0 | 99% | KY074010.1 |
| 62Select seq AB991630.1 | Pseudomonas sp. MsC-05-4CB2-02 gene for 16S rRNA, partial sequence | 1550 | 1550 | 99% | 0.0 | 99% | AB991630.1 |
| 63Select seq EU118771.1 | Pseudomonas lutea strain AGL 2 16S ribosomal RNA gene, partial sequence | 1550 | 1550 | 99% | 0.0 | 99% | EU118771.1 |
| 64Select seq LT601010.1 | Pseudomonas sp. CCUG 63225 partial 16S rRNA gene, strain CCUG 63225 | 1548 | 1548 | 97% | 0.0 | 99% | LT601010.1 |
| 65Select seq KM187506.1 | Pseudomonas sp. HP6G 16S ribosomal RNA gene, partial sequence | 1548 | 1548 | 98% | 0.0 | 99% | KM187506.1 |
| 66Select seq KM187192.1 | Pseudomonas sp. CC15J 16S ribosomal RNA gene, partial sequence | 1548 | 1548 | 98% | 0.0 | 99% | KM187192.1 |
| 67Select seq JQ977250.1 | Pseudomonas sp. Bza15 16S ribosomal RNA gene, partial sequence | 1548 | 1548 | 99% | 0.0 | 99% | JQ977250.1 |
| 68Select seq HE814629.1 | Pseudomonas sp. M1U22 partial 16S rRNA gene, isolate M1U22 | 1548 | 1548 | 98% | 0.0 | 99% | HE814629.1 |
| 69Select seq KU921557.1 | Pseudomonas graminis strain IHBB 9249 16S ribosomal RNA gene, partial sequence | 1546 | 1546 | 99% | 0.0 | 99% | KU921557.1 |
| 70Select seq KX507934.1 | Uncultured bacterium clone BJ201306-41 16S ribosomal RNA gene, partial sequence | 1546 | 1546 | 99% | 0.0 | 99% | KX507934.1 |
| 71Select seq KR922175.1 | Pseudomonas sp. PDD-59b-53 16S ribosomal RNA gene, complete sequence | 1546 | 1546 | 98% | 0.0 | 99% | KR922175.1 |
| 72Select seq KM891559.1 | Pseudomonas graminis strain AP10-7B 16S ribosomal RNA gene, partial sequence | 1546 | 1546 | 98% | 0.0 | 99% | KM891559.1 |
| 73Select seq KY075949.1 | Pseudomonas graminis strain CMN4 16S ribosomal RNA gene, partial sequence | 1544 | 1544 | 97% | 0.0 | 99% | KY075949.1 |
| 74Select seq KU587965.1 | Pseudomonas graminis strain A3 16S ribosomal RNA gene, partial sequence | 1544 | 1544 | 97% | 0.0 | 99% | KU587965.1 |
| 75Select seq KM187449.1 | Pseudomonas sp. HP3I 16S ribosomal RNA gene, partial sequence | 1544 | 1544 | 97% | 0.0 | 99% | KM187449.1 |
| 76Select seq MG576177.1 | Pseudomonas lutea strain KBL24 16S ribosomal RNA gene, partial sequence | 1543 | 1543 | 99% | 0.0 | 99% | MG576177.1 |
| 77Select seq KR922154.1 | Pseudomonas sp. PDD-59b-17 16S ribosomal RNA gene, complete sequence | 1543 | 1543 | 99% | 0.0 | 99% | KR922154.1 |
| 78Select seq KR922133.1 | Pseudomonas graminis strain PDD-58b-20 16S ribosomal RNA gene, complete sequence | 1543 | 1543 | 99% | 0.0 | 99% | KR922133.1 |
| 79Select seq KF147082.1 | Pseudomonas graminis strain R5SsM3P3C7 16S ribosomal RNA gene, partial sequence | 1543 | 1543 | 99% | 0.0 | 99% | KF147082.1 |
| 80Select seq KF147066.1 | Pseudomonas graminis strain R1SpM3P1C5 16S ribosomal RNA gene, partial sequence | 1543 | 1543 | 98% | 0.0 | 99% | KF147066.1 |
| 81Select seq FN421687.1 | Uncultured bacterium partial 16S rRNA gene, clone 3\_E03 | 1543 | 1543 | 99% | 0.0 | 99% | FN421687.1 |
| 82Select seq MF111452.1 | Pseudomonas putida strain R5-7 16S ribosomal RNA gene, partial sequence | 1541 | 1541 | 99% | 0.0 | 99% | MF111452.1 |
| 83Select seq KX507990.1 | Uncultured bacterium clone BJ201306-97 16S ribosomal RNA gene, partial sequence | 1541 | 1541 | 99% | 0.0 | 99% | KX507990.1 |
| 84Select seq KX507942.1 | Uncultured bacterium clone BJ201306-49 16S ribosomal RNA gene, partial sequence | 1541 | 1541 | 99% | 0.0 | 99% | KX507942.1 |
| 85Select seq KX507917.1 | Uncultured bacterium clone BJ201306-23 16S ribosomal RNA gene, partial sequence | 1541 | 1541 | 99% | 0.0 | 99% | KX507917.1 |
| 86Select seq KR922063.1 | Pseudomonas sp. PDD-48b-10 16S ribosomal RNA gene, complete sequence | 1541 | 1541 | 98% | 0.0 | 99% | KR922063.1 |
| 87Select seq AP014637.1 | Pseudomonas sp. StFLB209 DNA, complete genome | 1541 | 9247 | 99% | 0.0 | 99% | AP014637.1 |
| 88Select seq KF147075.1 | Pseudomonas graminis strain R5SpM3P1C3 16S ribosomal RNA gene, partial sequence | 1541 | 1541 | 98% | 0.0 | 99% | KF147075.1 |
| 89Select seq KF147074.1 | Pseudomonas graminis strain R5SsM3P3C8 16S ribosomal RNA gene, partial sequence | 1541 | 1541 | 98% | 0.0 | 99% | KF147074.1 |
| 90Select seq KF387661.1 | Pseudomonas graminis strain BDb2 16S ribosomal RNA gene, partial sequence | 1541 | 1541 | 97% | 0.0 | 99% | KF387661.1 |
| 91Select seq AB681704.1 | Pseudomonas putida gene for 16S rRNA, partial sequence, strain: NBRC 102093 | 1541 | 1541 | 99% | 0.0 | 99% | AB681704.1 |
| 92Select seq GU201572.1 | Uncultured Pseudomonas sp. clone EfT307\_C07 16S ribosomal RNA gene, partial sequence | 1541 | 1541 | 99% | 0.0 | 99% | GU201572.1 |
| 93Select seq GU272231.1 | Uncultured bacterium clone TF32 16S ribosomal RNA gene, partial sequence | 1541 | 1541 | 99% | 0.0 | 99% | GU272231.1 |
| 94Select seq AB109886.1 | Pseudomonas graminis gene for 16S rRNA, partial sequence, strain: KF701 | 1541 | 1541 | 99% | 0.0 | 99% | AB109886.1 |
| 95Select seq KM187190.1 | Pseudomonas sp. CC15H2 16S ribosomal RNA gene, partial sequence | 1539 | 1539 | 98% | 0.0 | 99% | KM187190.1 |
| 96Select seq KJ997739.1 | Pseudomonas graminis strain BG6 16S ribosomal RNA gene, partial sequence | 1539 | 1539 | 99% | 0.0 | 99% | KJ997739.1 |
| 97Select seq KF147071.1 | Pseudomonas graminis strain R5SpM3P1C2 16S ribosomal RNA gene, partial sequence | 1539 | 1539 | 98% | 0.0 | 99% | KF147071.1 |
| 98Select seq KX186935.1 | Pseudomonas abietaniphila culture DSM:6506 16S ribosomal RNA gene, partial sequence | 1535 | 1535 | 98% | 0.0 | 99% | KX186935.1 |
| 99Select seq KU587964.1 | Pseudomonas graminis strain A2 16S ribosomal RNA gene, partial sequence | 1535 | 1535 | 96% | 0.0 | 99% | KU587964.1 |
| 100Select seq KT984787.1 | Pseudomonas sp. SB15-Ch27-6 16S ribosomal RNA gene, partial sequence | 1535 | 1535 | 98% | 0.0 | 99% | KT984787.1 |


## Alignments

Loading alignment... for sequences gi|1336609592,gi|1174580246,gi|1115571102,gi|1115570720,gi|530252209 Reading indexes 1-5

Download

FASTA (complete sequence)

FASTA (aligned sequences)

GenBank (complete sequence)

Continue
Cancel

GenBankGraphics

Next
Previous
Descriptions

Pseudomonas sp. strain CF24P20 16S ribosomal RNA gene, partial sequence

Sequence ID: MG860023.1Length: 936Number of Matches: 1

Related Information

Range 1: 47 to 909GenBankGraphics

Next Match
Previous Match
First Match

Alignment statistics for match #1

| Score | Expect | Identities | Gaps | Strand | Frame |
| --- | --- | --- | --- | --- | --- |
| 1568 bits(849) | 0.0() | 856/863(99%) | 0/863(0%) | Plus/Minus |  |

Features:

```
Query  4    GTGNTANCCGTCCTCCCGAAGGTTAGACTAGCTACTTCTGGTNCAACCCACTCCCATGGT  63
            ||| || ||||||||||||||||||||||||||||||||||| |||||||||||||||||
Sbjct  909  GTGGTAACCGTCCTCCCGAAGGTTAGACTAGCTACTTCTGGTGCAACCCACTCCCATGGT  850

Query  64   GTGACGGGCGGTGTGTACAAGGCCCGGGAACGTATTCACCGCGACATTCTGATTCGCGAT  123
            ||||||||||||||||||||||||||||||||||||||||||||||||||||||||||||
Sbjct  849  GTGACGGGCGGTGTGTACAAGGCCCGGGAACGTATTCACCGCGACATTCTGATTCGCGAT  790

Query  124  TACTAGCGATTCCGACTTCACGCAGTCGAGTTGCAGACTGCGATCCGGACTACGATCGGT  183
            ||||||||||||||||||||||||||||||||||||||||||||||||||||||||||||
Sbjct  789  TACTAGCGATTCCGACTTCACGCAGTCGAGTTGCAGACTGCGATCCGGACTACGATCGGT  730

Query  184  TTTCTGGGATTAGCTCCACCTCGCGGCTTGGCAACCCTCTGTACCGACCATTGTAGCACG  243
            ||||||||||||||||||||||||||||||||||||||||||||||||||||||||||||
Sbjct  729  TTTCTGGGATTAGCTCCACCTCGCGGCTTGGCAACCCTCTGTACCGACCATTGTAGCACG  670

Query  244  TGTGTAGCCCAGGCCGTAAGGGCCATGATGACTTGACGTCATCCCCACCTTCCTCCGGTT  303
            ||||||||||||||||||||||||||||||||||||||||||||||||||||||||||||
Sbjct  669  TGTGTAGCCCAGGCCGTAAGGGCCATGATGACTTGACGTCATCCCCACCTTCCTCCGGTT  610

Query  304  TGTCACCGGCAGTCTCCTTAGAGTGCCCACCATAATGTGCTGGTAACTAAGGACAAGGGT  363
            ||||||||||||||||||||||||||||||||||||||||||||||||||||||||||||
Sbjct  609  TGTCACCGGCAGTCTCCTTAGAGTGCCCACCATAATGTGCTGGTAACTAAGGACAAGGGT  550

Query  364  TGCGCTCGTTACGGGACTTAACCCAACATCTCACGACACGAGCTGACGACAGCCATGCAG  423
            ||||||||||||||||||||||||||||||||||||||||||||||||||||||||||||
Sbjct  549  TGCGCTCGTTACGGGACTTAACCCAACATCTCACGACACGAGCTGACGACAGCCATGCAG  490

Query  424  CACCTGTCTCAATGTTCCCGAAGGCACCAATCCATCTCTGGAAAGTTCATTGGATGTCAA  483
            ||||||||||||||||||||||||||||||||||||||||||||||||||||||||||||
Sbjct  489  CACCTGTCTCAATGTTCCCGAAGGCACCAATCCATCTCTGGAAAGTTCATTGGATGTCAA  430

Query  484  GGCCTGGTAAGGTTCTTCGCGTTGCTTCGAATTAAACCACATGCTCCACCGCTTGTGCGG  543
            ||||||||||||||||||||||||||||||||||||||||||||||||||||||||||||
Sbjct  429  GGCCTGGTAAGGTTCTTCGCGTTGCTTCGAATTAAACCACATGCTCCACCGCTTGTGCGG  370

Query  544  GCCCCCGTCAATTCATTTGAGTTTTAACCTTGCGGCCGTACTCCCCAGGCGGTCAACTTA  603
            ||||||||||||||||||||||||||||||||||||||||||||||||||||||||||||
Sbjct  369  GCCCCCGTCAATTCATTTGAGTTTTAACCTTGCGGCCGTACTCCCCAGGCGGTCAACTTA  310

Query  604  ATGCGTTAGCTGCGCCACTAAAAGCTCAAGGCTTCCAACGGCTAGTTGACATCGTTTACG  663
            ||||||||||||||||||||||||||||||||||||||||||||||||||||||||||||
Sbjct  309  ATGCGTTAGCTGCGCCACTAAAAGCTCAAGGCTTCCAACGGCTAGTTGACATCGTTTACG  250

Query  664  GCGTGGACTACCAGGGTATCTAATCCTGTTTGCTCCCCACGCTTTCGCACCTCAGTGTCA  723
            ||||||||||||||||||||||||||||||||||||||||||||||||||||||||||||
Sbjct  249  GCGTGGACTACCAGGGTATCTAATCCTGTTTGCTCCCCACGCTTTCGCACCTCAGTGTCA  190

Query  724  GTATGAGCCCAGGTGGTCGCCTTCGCCACTGGTNTTCCTTCCTATATCTACGCATTTCAC  783
            ||||||||||||||||||||||||||||||||| ||||||||||||||||||||||||||
Sbjct  189  GTATGAGCCCAGGTGGTCGCCTTCGCCACTGGTGTTCCTTCCTATATCTACGCATTTCAC  130

Query  784  CGCTANNNAGGAAATTCCACCACCCTCTGCCCTACTCTAGCTTGCCAGTTTTGGATGCAG  843
            |||||   ||||||||||||||||||||||||||||||||||||||||||||||||||||
Sbjct  129  CGCTACACAGGAAATTCCACCACCCTCTGCCCTACTCTAGCTTGCCAGTTTTGGATGCAG  70

Query  844  TTCCCAGGTTGAGCCCGGGGATT  866
            |||||||||||||||||||||||
Sbjct  69   TTCCCAGGTTGAGCCCGGGGATT  47
```

Download

FASTA (complete sequence)

FASTA (aligned sequences)

GenBank (complete sequence)

Continue
Cancel

GenBankGraphics

Next
Previous
Descriptions

Pseudomonas sp. strain MSM-2-10-15 16S ribosomal RNA gene, partial sequence

Sequence ID: KY907017.1Length: 1473Number of Matches: 1

Related Information

Range 1: 583 to 1445GenBankGraphics

Next Match
Previous Match
First Match

Alignment statistics for match #1

| Score | Expect | Identities | Gaps | Strand | Frame |
| --- | --- | --- | --- | --- | --- |
| 1568 bits(849) | 0.0() | 856/863(99%) | 0/863(0%) | Plus/Minus |  |

Features:

```
Query  4     GTGNTANCCGTCCTCCCGAAGGTTAGACTAGCTACTTCTGGTNCAACCCACTCCCATGGT  63
             ||| || ||||||||||||||||||||||||||||||||||| |||||||||||||||||
Sbjct  1445  GTGGTAACCGTCCTCCCGAAGGTTAGACTAGCTACTTCTGGTGCAACCCACTCCCATGGT  1386

Query  64    GTGACGGGCGGTGTGTACAAGGCCCGGGAACGTATTCACCGCGACATTCTGATTCGCGAT  123
             ||||||||||||||||||||||||||||||||||||||||||||||||||||||||||||
Sbjct  1385  GTGACGGGCGGTGTGTACAAGGCCCGGGAACGTATTCACCGCGACATTCTGATTCGCGAT  1326

Query  124   TACTAGCGATTCCGACTTCACGCAGTCGAGTTGCAGACTGCGATCCGGACTACGATCGGT  183
             ||||||||||||||||||||||||||||||||||||||||||||||||||||||||||||
Sbjct  1325  TACTAGCGATTCCGACTTCACGCAGTCGAGTTGCAGACTGCGATCCGGACTACGATCGGT  1266

Query  184   TTTCTGGGATTAGCTCCACCTCGCGGCTTGGCAACCCTCTGTACCGACCATTGTAGCACG  243
             ||||||||||||||||||||||||||||||||||||||||||||||||||||||||||||
Sbjct  1265  TTTCTGGGATTAGCTCCACCTCGCGGCTTGGCAACCCTCTGTACCGACCATTGTAGCACG  1206

Query  244   TGTGTAGCCCAGGCCGTAAGGGCCATGATGACTTGACGTCATCCCCACCTTCCTCCGGTT  303
             ||||||||||||||||||||||||||||||||||||||||||||||||||||||||||||
Sbjct  1205  TGTGTAGCCCAGGCCGTAAGGGCCATGATGACTTGACGTCATCCCCACCTTCCTCCGGTT  1146

Query  304   TGTCACCGGCAGTCTCCTTAGAGTGCCCACCATAATGTGCTGGTAACTAAGGACAAGGGT  363
             ||||||||||||||||||||||||||||||||||||||||||||||||||||||||||||
Sbjct  1145  TGTCACCGGCAGTCTCCTTAGAGTGCCCACCATAATGTGCTGGTAACTAAGGACAAGGGT  1086

Query  364   TGCGCTCGTTACGGGACTTAACCCAACATCTCACGACACGAGCTGACGACAGCCATGCAG  423
             ||||||||||||||||||||||||||||||||||||||||||||||||||||||||||||
Sbjct  1085  TGCGCTCGTTACGGGACTTAACCCAACATCTCACGACACGAGCTGACGACAGCCATGCAG  1026

Query  424   CACCTGTCTCAATGTTCCCGAAGGCACCAATCCATCTCTGGAAAGTTCATTGGATGTCAA  483
             ||||||||||||||||||||||||||||||||||||||||||||||||||||||||||||
Sbjct  1025  CACCTGTCTCAATGTTCCCGAAGGCACCAATCCATCTCTGGAAAGTTCATTGGATGTCAA  966

Query  484   GGCCTGGTAAGGTTCTTCGCGTTGCTTCGAATTAAACCACATGCTCCACCGCTTGTGCGG  543
             ||||||||||||||||||||||||||||||||||||||||||||||||||||||||||||
Sbjct  965   GGCCTGGTAAGGTTCTTCGCGTTGCTTCGAATTAAACCACATGCTCCACCGCTTGTGCGG  906

Query  544   GCCCCCGTCAATTCATTTGAGTTTTAACCTTGCGGCCGTACTCCCCAGGCGGTCAACTTA  603
             ||||||||||||||||||||||||||||||||||||||||||||||||||||||||||||
Sbjct  905   GCCCCCGTCAATTCATTTGAGTTTTAACCTTGCGGCCGTACTCCCCAGGCGGTCAACTTA  846

Query  604   ATGCGTTAGCTGCGCCACTAAAAGCTCAAGGCTTCCAACGGCTAGTTGACATCGTTTACG  663
             ||||||||||||||||||||||||||||||||||||||||||||||||||||||||||||
Sbjct  845   ATGCGTTAGCTGCGCCACTAAAAGCTCAAGGCTTCCAACGGCTAGTTGACATCGTTTACG  786

Query  664   GCGTGGACTACCAGGGTATCTAATCCTGTTTGCTCCCCACGCTTTCGCACCTCAGTGTCA  723
             ||||||||||||||||||||||||||||||||||||||||||||||||||||||||||||
Sbjct  785   GCGTGGACTACCAGGGTATCTAATCCTGTTTGCTCCCCACGCTTTCGCACCTCAGTGTCA  726

Query  724   GTATGAGCCCAGGTGGTCGCCTTCGCCACTGGTNTTCCTTCCTATATCTACGCATTTCAC  783
             ||||||||||||||||||||||||||||||||| ||||||||||||||||||||||||||
Sbjct  725   GTATGAGCCCAGGTGGTCGCCTTCGCCACTGGTGTTCCTTCCTATATCTACGCATTTCAC  666

Query  784   CGCTANNNAGGAAATTCCACCACCCTCTGCCCTACTCTAGCTTGCCAGTTTTGGATGCAG  843
             |||||   ||||||||||||||||||||||||||||||||||||||||||||||||||||
Sbjct  665   CGCTACACAGGAAATTCCACCACCCTCTGCCCTACTCTAGCTTGCCAGTTTTGGATGCAG  606

Query  844   TTCCCAGGTTGAGCCCGGGGATT  866
             |||||||||||||||||||||||
Sbjct  605   TTCCCAGGTTGAGCCCGGGGATT  583
```

Download

FASTA (complete sequence)

FASTA (aligned sequences)

GenBank (complete sequence)

Continue
Cancel

GenBankGraphics

Next
Previous
Descriptions

Pseudomonas sp. strain BAV3487 16S ribosomal RNA gene, partial sequence

Sequence ID: KY074356.1Length: 927Number of Matches: 1

Related Information

Range 1: 34 to 896GenBankGraphics

Next Match
Previous Match
First Match

Alignment statistics for match #1

| Score | Expect | Identities | Gaps | Strand | Frame |
| --- | --- | --- | --- | --- | --- |
| 1568 bits(849) | 0.0() | 856/863(99%) | 0/863(0%) | Plus/Minus |  |

Features:

```
Query  4    GTGNTANCCGTCCTCCCGAAGGTTAGACTAGCTACTTCTGGTNCAACCCACTCCCATGGT  63
            ||| || ||||||||||||||||||||||||||||||||||| |||||||||||||||||
Sbjct  896  GTGGTAACCGTCCTCCCGAAGGTTAGACTAGCTACTTCTGGTGCAACCCACTCCCATGGT  837

Query  64   GTGACGGGCGGTGTGTACAAGGCCCGGGAACGTATTCACCGCGACATTCTGATTCGCGAT  123
            ||||||||||||||||||||||||||||||||||||||||||||||||||||||||||||
Sbjct  836  GTGACGGGCGGTGTGTACAAGGCCCGGGAACGTATTCACCGCGACATTCTGATTCGCGAT  777

Query  124  TACTAGCGATTCCGACTTCACGCAGTCGAGTTGCAGACTGCGATCCGGACTACGATCGGT  183
            ||||||||||||||||||||||||||||||||||||||||||||||||||||||||||||
Sbjct  776  TACTAGCGATTCCGACTTCACGCAGTCGAGTTGCAGACTGCGATCCGGACTACGATCGGT  717

Query  184  TTTCTGGGATTAGCTCCACCTCGCGGCTTGGCAACCCTCTGTACCGACCATTGTAGCACG  243
            ||||||||||||||||||||||||||||||||||||||||||||||||||||||||||||
Sbjct  716  TTTCTGGGATTAGCTCCACCTCGCGGCTTGGCAACCCTCTGTACCGACCATTGTAGCACG  657

Query  244  TGTGTAGCCCAGGCCGTAAGGGCCATGATGACTTGACGTCATCCCCACCTTCCTCCGGTT  303
            ||||||||||||||||||||||||||||||||||||||||||||||||||||||||||||
Sbjct  656  TGTGTAGCCCAGGCCGTAAGGGCCATGATGACTTGACGTCATCCCCACCTTCCTCCGGTT  597

Query  304  TGTCACCGGCAGTCTCCTTAGAGTGCCCACCATAATGTGCTGGTAACTAAGGACAAGGGT  363
            ||||||||||||||||||||||||||||||||||||||||||||||||||||||||||||
Sbjct  596  TGTCACCGGCAGTCTCCTTAGAGTGCCCACCATAATGTGCTGGTAACTAAGGACAAGGGT  537

Query  364  TGCGCTCGTTACGGGACTTAACCCAACATCTCACGACACGAGCTGACGACAGCCATGCAG  423
            ||||||||||||||||||||||||||||||||||||||||||||||||||||||||||||
Sbjct  536  TGCGCTCGTTACGGGACTTAACCCAACATCTCACGACACGAGCTGACGACAGCCATGCAG  477

Query  424  CACCTGTCTCAATGTTCCCGAAGGCACCAATCCATCTCTGGAAAGTTCATTGGATGTCAA  483
            ||||||||||||||||||||||||||||||||||||||||||||||||||||||||||||
Sbjct  476  CACCTGTCTCAATGTTCCCGAAGGCACCAATCCATCTCTGGAAAGTTCATTGGATGTCAA  417

Query  484  GGCCTGGTAAGGTTCTTCGCGTTGCTTCGAATTAAACCACATGCTCCACCGCTTGTGCGG  543
            ||||||||||||||||||||||||||||||||||||||||||||||||||||||||||||
Sbjct  416  GGCCTGGTAAGGTTCTTCGCGTTGCTTCGAATTAAACCACATGCTCCACCGCTTGTGCGG  357

Query  544  GCCCCCGTCAATTCATTTGAGTTTTAACCTTGCGGCCGTACTCCCCAGGCGGTCAACTTA  603
            ||||||||||||||||||||||||||||||||||||||||||||||||||||||||||||
Sbjct  356  GCCCCCGTCAATTCATTTGAGTTTTAACCTTGCGGCCGTACTCCCCAGGCGGTCAACTTA  297

Query  604  ATGCGTTAGCTGCGCCACTAAAAGCTCAAGGCTTCCAACGGCTAGTTGACATCGTTTACG  663
            ||||||||||||||||||||||||||||||||||||||||||||||||||||||||||||
Sbjct  296  ATGCGTTAGCTGCGCCACTAAAAGCTCAAGGCTTCCAACGGCTAGTTGACATCGTTTACG  237

Query  664  GCGTGGACTACCAGGGTATCTAATCCTGTTTGCTCCCCACGCTTTCGCACCTCAGTGTCA  723
            ||||||||||||||||||||||||||||||||||||||||||||||||||||||||||||
Sbjct  236  GCGTGGACTACCAGGGTATCTAATCCTGTTTGCTCCCCACGCTTTCGCACCTCAGTGTCA  177

Query  724  GTATGAGCCCAGGTGGTCGCCTTCGCCACTGGTNTTCCTTCCTATATCTACGCATTTCAC  783
            ||||||||||||||||||||||||||||||||| ||||||||||||||||||||||||||
Sbjct  176  GTATGAGCCCAGGTGGTCGCCTTCGCCACTGGTGTTCCTTCCTATATCTACGCATTTCAC  117

Query  784  CGCTANNNAGGAAATTCCACCACCCTCTGCCCTACTCTAGCTTGCCAGTTTTGGATGCAG  843
            |||||   ||||||||||||||||||||||||||||||||||||||||||||||||||||
Sbjct  116  CGCTACACAGGAAATTCCACCACCCTCTGCCCTACTCTAGCTTGCCAGTTTTGGATGCAG  57

Query  844  TTCCCAGGTTGAGCCCGGGGATT  866
            |||||||||||||||||||||||
Sbjct  56   TTCCCAGGTTGAGCCCGGGGATT  34
```

Download

FASTA (complete sequence)

FASTA (aligned sequences)

GenBank (complete sequence)

Continue
Cancel

GenBankGraphics

Next
Previous
Descriptions

Pseudomonas sp. strain BAV2562 16S ribosomal RNA gene, partial sequence

Sequence ID: KY073974.1Length: 926Number of Matches: 1

#### See 6 more title(s)

Pseudomonas sp. strain BAV2602 16S ribosomal RNA gene, partial sequence

Sequence ID: KY073996.1Length: 926Number of Matches:

Pseudomonas sp. strain BAV2867 16S ribosomal RNA gene, partial sequence

Sequence ID: KY074043.1Length: 926Number of Matches:

Pseudomonas sp. strain BAV3494 16S ribosomal RNA gene, partial sequence

Sequence ID: KY074361.1Length: 926Number of Matches:

Pseudomonas sp. strain BAV4069 16S ribosomal RNA gene, partial sequence

Sequence ID: KY074525.1Length: 926Number of Matches:

Pseudomonas sp. strain BAV4070 16S ribosomal RNA gene, partial sequence

Sequence ID: KY074526.1Length: 926Number of Matches:

Pseudomonas sp. strain BAV4092 16S ribosomal RNA gene, partial sequence

Sequence ID: KY074536.1Length: 926Number of Matches:

Related Information

Range 1: 34 to 896GenBankGraphics

Next Match
Previous Match
First Match

Alignment statistics for match #1

| Score | Expect | Identities | Gaps | Strand | Frame |
| --- | --- | --- | --- | --- | --- |
| 1568 bits(849) | 0.0() | 856/863(99%) | 0/863(0%) | Plus/Minus |  |

Features:

```
Query  4    GTGNTANCCGTCCTCCCGAAGGTTAGACTAGCTACTTCTGGTNCAACCCACTCCCATGGT  63
            ||| || ||||||||||||||||||||||||||||||||||| |||||||||||||||||
Sbjct  896  GTGGTAACCGTCCTCCCGAAGGTTAGACTAGCTACTTCTGGTGCAACCCACTCCCATGGT  837

Query  64   GTGACGGGCGGTGTGTACAAGGCCCGGGAACGTATTCACCGCGACATTCTGATTCGCGAT  123
            ||||||||||||||||||||||||||||||||||||||||||||||||||||||||||||
Sbjct  836  GTGACGGGCGGTGTGTACAAGGCCCGGGAACGTATTCACCGCGACATTCTGATTCGCGAT  777

Query  124  TACTAGCGATTCCGACTTCACGCAGTCGAGTTGCAGACTGCGATCCGGACTACGATCGGT  183
            ||||||||||||||||||||||||||||||||||||||||||||||||||||||||||||
Sbjct  776  TACTAGCGATTCCGACTTCACGCAGTCGAGTTGCAGACTGCGATCCGGACTACGATCGGT  717

Query  184  TTTCTGGGATTAGCTCCACCTCGCGGCTTGGCAACCCTCTGTACCGACCATTGTAGCACG  243
            ||||||||||||||||||||||||||||||||||||||||||||||||||||||||||||
Sbjct  716  TTTCTGGGATTAGCTCCACCTCGCGGCTTGGCAACCCTCTGTACCGACCATTGTAGCACG  657

Query  244  TGTGTAGCCCAGGCCGTAAGGGCCATGATGACTTGACGTCATCCCCACCTTCCTCCGGTT  303
            ||||||||||||||||||||||||||||||||||||||||||||||||||||||||||||
Sbjct  656  TGTGTAGCCCAGGCCGTAAGGGCCATGATGACTTGACGTCATCCCCACCTTCCTCCGGTT  597

Query  304  TGTCACCGGCAGTCTCCTTAGAGTGCCCACCATAATGTGCTGGTAACTAAGGACAAGGGT  363
            ||||||||||||||||||||||||||||||||||||||||||||||||||||||||||||
Sbjct  596  TGTCACCGGCAGTCTCCTTAGAGTGCCCACCATAATGTGCTGGTAACTAAGGACAAGGGT  537

Query  364  TGCGCTCGTTACGGGACTTAACCCAACATCTCACGACACGAGCTGACGACAGCCATGCAG  423
            ||||||||||||||||||||||||||||||||||||||||||||||||||||||||||||
Sbjct  536  TGCGCTCGTTACGGGACTTAACCCAACATCTCACGACACGAGCTGACGACAGCCATGCAG  477

Query  424  CACCTGTCTCAATGTTCCCGAAGGCACCAATCCATCTCTGGAAAGTTCATTGGATGTCAA  483
            ||||||||||||||||||||||||||||||||||||||||||||||||||||||||||||
Sbjct  476  CACCTGTCTCAATGTTCCCGAAGGCACCAATCCATCTCTGGAAAGTTCATTGGATGTCAA  417

Query  484  GGCCTGGTAAGGTTCTTCGCGTTGCTTCGAATTAAACCACATGCTCCACCGCTTGTGCGG  543
            ||||||||||||||||||||||||||||||||||||||||||||||||||||||||||||
Sbjct  416  GGCCTGGTAAGGTTCTTCGCGTTGCTTCGAATTAAACCACATGCTCCACCGCTTGTGCGG  357

Query  544  GCCCCCGTCAATTCATTTGAGTTTTAACCTTGCGGCCGTACTCCCCAGGCGGTCAACTTA  603
            ||||||||||||||||||||||||||||||||||||||||||||||||||||||||||||
Sbjct  356  GCCCCCGTCAATTCATTTGAGTTTTAACCTTGCGGCCGTACTCCCCAGGCGGTCAACTTA  297

Query  604  ATGCGTTAGCTGCGCCACTAAAAGCTCAAGGCTTCCAACGGCTAGTTGACATCGTTTACG  663
            ||||||||||||||||||||||||||||||||||||||||||||||||||||||||||||
Sbjct  296  ATGCGTTAGCTGCGCCACTAAAAGCTCAAGGCTTCCAACGGCTAGTTGACATCGTTTACG  237

Query  664  GCGTGGACTACCAGGGTATCTAATCCTGTTTGCTCCCCACGCTTTCGCACCTCAGTGTCA  723
            ||||||||||||||||||||||||||||||||||||||||||||||||||||||||||||
Sbjct  236  GCGTGGACTACCAGGGTATCTAATCCTGTTTGCTCCCCACGCTTTCGCACCTCAGTGTCA  177

Query  724  GTATGAGCCCAGGTGGTCGCCTTCGCCACTGGTNTTCCTTCCTATATCTACGCATTTCAC  783
            ||||||||||||||||||||||||||||||||| ||||||||||||||||||||||||||
Sbjct  176  GTATGAGCCCAGGTGGTCGCCTTCGCCACTGGTGTTCCTTCCTATATCTACGCATTTCAC  117

Query  784  CGCTANNNAGGAAATTCCACCACCCTCTGCCCTACTCTAGCTTGCCAGTTTTGGATGCAG  843
            |||||   ||||||||||||||||||||||||||||||||||||||||||||||||||||
Sbjct  116  CGCTACACAGGAAATTCCACCACCCTCTGCCCTACTCTAGCTTGCCAGTTTTGGATGCAG  57

Query  844  TTCCCAGGTTGAGCCCGGGGATT  866
            |||||||||||||||||||||||
Sbjct  56   TTCCCAGGTTGAGCCCGGGGATT  34
```

Download

FASTA (complete sequence)

FASTA (aligned sequences)

GenBank (complete sequence)

Continue
Cancel

GenBankGraphics

Next
Previous
Descriptions

Pseudomonas graminis strain R5SpM3P2C1 16S ribosomal RNA gene, partial sequence

Sequence ID: KF147092.1Length: 1436Number of Matches: 1

Related Information

Range 1: 551 to 1413GenBankGraphics

Next Match
Previous Match
First Match

Alignment statistics for match #1

| Score | Expect | Identities | Gaps | Strand | Frame |
| --- | --- | --- | --- | --- | --- |
| 1568 bits(849) | 0.0() | 856/863(99%) | 0/863(0%) | Plus/Minus |  |

Features:

```
Query  4     GTGNTANCCGTCCTCCCGAAGGTTAGACTAGCTACTTCTGGTNCAACCCACTCCCATGGT  63
             ||| || ||||||||||||||||||||||||||||||||||| |||||||||||||||||
Sbjct  1413  GTGGTAACCGTCCTCCCGAAGGTTAGACTAGCTACTTCTGGTGCAACCCACTCCCATGGT  1354

Query  64    GTGACGGGCGGTGTGTACAAGGCCCGGGAACGTATTCACCGCGACATTCTGATTCGCGAT  123
             ||||||||||||||||||||||||||||||||||||||||||||||||||||||||||||
Sbjct  1353  GTGACGGGCGGTGTGTACAAGGCCCGGGAACGTATTCACCGCGACATTCTGATTCGCGAT  1294

Query  124   TACTAGCGATTCCGACTTCACGCAGTCGAGTTGCAGACTGCGATCCGGACTACGATCGGT  183
             ||||||||||||||||||||||||||||||||||||||||||||||||||||||||||||
Sbjct  1293  TACTAGCGATTCCGACTTCACGCAGTCGAGTTGCAGACTGCGATCCGGACTACGATCGGT  1234

Query  184   TTTCTGGGATTAGCTCCACCTCGCGGCTTGGCAACCCTCTGTACCGACCATTGTAGCACG  243
             ||||||||||||||||||||||||||||||||||||||||||||||||||||||||||||
Sbjct  1233  TTTCTGGGATTAGCTCCACCTCGCGGCTTGGCAACCCTCTGTACCGACCATTGTAGCACG  1174

Query  244   TGTGTAGCCCAGGCCGTAAGGGCCATGATGACTTGACGTCATCCCCACCTTCCTCCGGTT  303
             ||||||||||||||||||||||||||||||||||||||||||||||||||||||||||||
Sbjct  1173  TGTGTAGCCCAGGCCGTAAGGGCCATGATGACTTGACGTCATCCCCACCTTCCTCCGGTT  1114

Query  304   TGTCACCGGCAGTCTCCTTAGAGTGCCCACCATAATGTGCTGGTAACTAAGGACAAGGGT  363
             ||||||||||||||||||||||||||||||||||||||||||||||||||||||||||||
Sbjct  1113  TGTCACCGGCAGTCTCCTTAGAGTGCCCACCATAATGTGCTGGTAACTAAGGACAAGGGT  1054

Query  364   TGCGCTCGTTACGGGACTTAACCCAACATCTCACGACACGAGCTGACGACAGCCATGCAG  423
             ||||||||||||||||||||||||||||||||||||||||||||||||||||||||||||
Sbjct  1053  TGCGCTCGTTACGGGACTTAACCCAACATCTCACGACACGAGCTGACGACAGCCATGCAG  994

Query  424   CACCTGTCTCAATGTTCCCGAAGGCACCAATCCATCTCTGGAAAGTTCATTGGATGTCAA  483
             ||||||||||||||||||||||||||||||||||||||||||||||||||||||||||||
Sbjct  993   CACCTGTCTCAATGTTCCCGAAGGCACCAATCCATCTCTGGAAAGTTCATTGGATGTCAA  934

Query  484   GGCCTGGTAAGGTTCTTCGCGTTGCTTCGAATTAAACCACATGCTCCACCGCTTGTGCGG  543
             ||||||||||||||||||||||||||||||||||||||||||||||||||||||||||||
Sbjct  933   GGCCTGGTAAGGTTCTTCGCGTTGCTTCGAATTAAACCACATGCTCCACCGCTTGTGCGG  874

Query  544   GCCCCCGTCAATTCATTTGAGTTTTAACCTTGCGGCCGTACTCCCCAGGCGGTCAACTTA  603
             ||||||||||||||||||||||||||||||||||||||||||||||||||||||||||||
Sbjct  873   GCCCCCGTCAATTCATTTGAGTTTTAACCTTGCGGCCGTACTCCCCAGGCGGTCAACTTA  814

Query  604   ATGCGTTAGCTGCGCCACTAAAAGCTCAAGGCTTCCAACGGCTAGTTGACATCGTTTACG  663
             ||||||||||||||||||||||||||||||||||||||||||||||||||||||||||||
Sbjct  813   ATGCGTTAGCTGCGCCACTAAAAGCTCAAGGCTTCCAACGGCTAGTTGACATCGTTTACG  754

Query  664   GCGTGGACTACCAGGGTATCTAATCCTGTTTGCTCCCCACGCTTTCGCACCTCAGTGTCA  723
             ||||||||||||||||||||||||||||||||||||||||||||||||||||||||||||
Sbjct  753   GCGTGGACTACCAGGGTATCTAATCCTGTTTGCTCCCCACGCTTTCGCACCTCAGTGTCA  694

Query  724   GTATGAGCCCAGGTGGTCGCCTTCGCCACTGGTNTTCCTTCCTATATCTACGCATTTCAC  783
             ||||||||||||||||||||||||||||||||| ||||||||||||||||||||||||||
Sbjct  693   GTATGAGCCCAGGTGGTCGCCTTCGCCACTGGTGTTCCTTCCTATATCTACGCATTTCAC  634

Query  784   CGCTANNNAGGAAATTCCACCACCCTCTGCCCTACTCTAGCTTGCCAGTTTTGGATGCAG  843
             |||||   ||||||||||||||||||||||||||||||||||||||||||||||||||||
Sbjct  633   CGCTACACAGGAAATTCCACCACCCTCTGCCCTACTCTAGCTTGCCAGTTTTGGATGCAG  574

Query  844   TTCCCAGGTTGAGCCCGGGGATT  866
             |||||||||||||||||||||||
Sbjct  573   TTCCCAGGTTGAGCCCGGGGATT  551
```


BLAST is a registered trademark of the National Library of Medicine

Support center
Mailing list


YouTube

- National Library Of Medicine
- National Institutes Of Health
- U.S. Department of Health & Human Services
- USA.gov

### NCBI


National Center for Biotechnology Information,
 U.S. National Library of Medicine

8600 Rockville Pike,
Bethesda
 MD,
20894
USA

Policies and Guidelines
|
Contact


PreferencesTurn off

External link. Please review our privacy policy.
