# Supplementary material for: Isothermal microcalorimetry for thermal viable count of microorganisms in pure cultures and stabilized formulations
Source: BMC Microbiol. 2019 Mar 21;19:65. doi: 10.1186/s12866-019-1432-8 (PMC6429831; doi:10.1186/s12866-019-1432-8)
Supplement: Supplementary file 3 — 16S rRNA gene sequences’ BLASTN hits in zipped HTML format. (ZIP 15810 kb) [file 12866_2019_1432_MOESM3_ESM.zip › Best blastn hits/NCBI Blast_48 white negative F -- 15..1003 of sequence.html]

NCBI Blast:48 white negative F -- 15..1003 of sequence


- NCBI Home
- Sign in to NCBI
- Skip to Main Content
- Skip to Navigation
- About NCBI Accesskeys

U.S. National Library of Medicine

NCBI
National Center for Biotechnology Information

- My NCBI
- Sign in to NCBI
- Register
- Sign Out

BLAST ^®^ » blastn suite » RID-A4W7BCKZ014


- Home
- Recent Results
- Saved Strategies
- Help

BLAST Results


Edit and Resubmit
Save Search Strategies
[Sign in above to save your search strategy]

Formatting options 


Download


How to read this page
Blast report description
Questions/comments


|  |  |
| --- | --- |
| Formatting options | |
| Show | Alignment as  HTML Plain text   Old View Reset form to defaults [?]  These options control formatting of alignments in results pages. The default is HTML, but other formats (including plain text) are available. PSSM and PssmWithParameters are representations of Position Specific Scoring Matrices and are only available for PSI-BLAST. The Advanced view option allows the database descriptions to be sorted by various indices in a table. |
| Alignment View | Pairwise Pairwise with dots for identities Query-anchored with dots for identities Query-anchored with letters for identities Flat query-anchored with dots for identities Flat query-anchored with letters for identities [?]  Choose how to view alignments. The default "pairwise" view shows how each subject sequence aligns individually to the query sequence. The "query-anchored" view shows how all subject sequences align to the query sequence. For each view type, you can choose to show "identities" (matching residues) as letters or dots. more... |
| Display | Graphical Overview   Linkout   Sequence Retrieval  NCBI-gi   CDS feature [?]  - Graphical Overview: Graphical Overview: Show graph of similar sequence regions aligned to query.   more... - NCBI-gi: Show NCBI gi identifiers. - CDS feature: Show annotated coding region and translation.   more... |
| Masking | Character:   X for protein, n for nucleotide Lower Case  Color:  Black Grey Red [?]  - Masking Character: Display masked (filtered) sequence regions as lower-case or as specific letters (N for nucleotide, P for protein). - Masking Color: Display masked sequence regions in the given color. |
| Limit results | Descriptions:  10 50 100 Graphical overview:  0 10 50 100  Alignments:  0 10 50 100 Line length:  60 90 120 150 [?]  - Descriptions: Show short descriptions for up to the given number of sequences. - Alignments: Show alignments for up to the given number of sequences, in order of statistical significance. - Line lenghth: Number of letters to show on one line in an alignment. |
|  | Organism Type common name, binomial, taxid, or group name. Only 20 top taxa will be shown.     Exclude   [?]  Show only sequences from the given organism. |
|  | Entrez query:  [?]  Show only those sequences that match the given Entrez query. more... |
|  | Expect Min:  Expect Max:  [?]  Show only sequences with expect values in the given range. more... |
|  | Percent Identity Min:  Percent Identity Max:  [?]  Show only sequences with percent identity values in the given range. |
| Format for | PSI-BLAST with inclusion threshold:  [?]  - Format for PSI-BLAST: The Position-Specific Iterated BLAST (PSI-BLAST) program performs iterative searches with a protein query,   in which sequences found in one round of search are used to build a custom score model for the next round.   more... - Inclusion Threshold: This sets the statistical significance threshold for including a sequence in the model used   by PSI-BLAST to create the PSSM on the next iteration. |

|  |  |  |  |  |  |
| --- | --- | --- | --- | --- | --- |
| Download | | | | | |
| Alignment  Text XML ASN.1 JSON Seq-align Hit Table(text) Hit Table(csv) Multiple-file XML2 Single-file XML2 Multiple-file JSON Single-file JSON SAM | Search Strategies  ASN.1 | PSSM to restart search  PSSM | [?] |  |  |

The Download link provides BLAST output that may be used as input to another program.
This includes parseable formats such as the tabular report or XML as well as the Search Strategy files read by the BLAST+ applications.
More details on the parseable (XML, tabular, and ASN.1) reports can be found at
https://www.ncbi.nlm.nih.gov/books/NBK153387/  
  

The following formats are offered under the Alignment section:  
1). "Text". Non-HTML standard BLAST report.  
2). "XML". XML report based upon the DTD at https://www.ncbi.nlm.nih.gov/data\_specs/dtd/NCBI\_BlastOutput.dtd  
3). "ASN.1". Alignment written out in Abstract Syntax Notation 1.  
4). "JSON Seq-align". Alignment written out in JSON.  
4). "Hit Table(text)". The tabular report as text.  
5). "Hit Table(csv)". The tabular report ready for import into spread-sheet programs like Excel.  
6). "XML2". New XML format described at ftp://ftp.ncbi.nlm.nih.gov/blast/documents/NEWXML/xml2.pdf.  
7). "JSON". New JSON format described at ftp://ftp.ncbi.nlm.nih.gov/blast/documents/NEWXML/xml2.pdf.  
8). "SAM". Sequence Alignment Map format.

XML2 and JSON can be downloaded either as one file per query (multiple-file) or one file for all queries (single-file). These formats are listed as Multiple-file XML2 (and JSON) or Single-file XML (and JSON).

The following report is offered under the Search Strategy section:  
1). "ASN.1" Search Strategy. A record of the parameters, query, and database used in the search. This file can be used to start a stand-alone BLAST search, see
https://www.ncbi.nlm.nih.gov/books/NBK1763/#CmdLineAppsManual.I455\_BLAST\_search\_stra


# Job title: 48 white negative F -- 15..1003 of sequence

Results for:

lcl|Query\_187559 48 white negative F -- 15..1003 of sequence(989bp)
[?]

Your BLAST job specified more than one input sequence.
This box lets you choose which input sequence to show BLAST results for.

RID
:   A4W7BCKZ014 (Expires on 03-10 18:32 pm)

Query ID
:   lcl|Query\_187559
:   lcl|Query\_187559

Description
:   48 white negative F -- 15..1003 of sequence

Molecule type
:   nucleic acid

Query Length
:   989

Database Name
:   nr

Description
:   Nucleotide collection (nt) See details

Program
:   BLASTN 2.8.0+ Citation

  

Reference 

Zheng Zhang, Scott Schwartz, Lukas Wagner, and Webb Miller (2000), "A greedy algorithm for aligning DNA sequences", J Comput Biol 2000; 7(1-2):203-14.

Reference - database indexing

Aleksandr Morgulis, George Coulouris, Yan Raytselis, Thomas L. Madden, Richa Agarwala, Alejandro A. Schäffer (2008), "Database Indexing for Production MegaBLAST Searches", Bioinformatics 24:1757-1764.

Other reports:
Search Summary

[Taxonomy reports]
[Distance tree of results]
[MSA viewer]

Search Parameters

| Search parameter name | Search parameter value |
| --- | --- |
| Program | blastn |
| Word size | 28 |
| Expect value | 10 |
| Hitlist size | 100 |
| Match/Mismatch scores | 1,-2 |
| Gapcosts | 0,2.5 |
| Low Complexity Filter | Yes |
| Filter string | L;m; |
| Genetic Code | 1 |

Database

| Database parameter name | Database parameter value |
| --- | --- |
| Posted date | Mar 7, 2018 1:58 PM |
| Number of letters | 174,044,644,244 |
| Number of sequences | 46,882,714 |
| Entrez query | Includes:  Excludes:  None |

Karlin-Altschul statistics

| Params | Ungapped | Gapped |
| --- | --- | --- |
| Lambda | 1.33271 | 1.28 |
| K | 0.620991 | 0.46 |
| H | 1.12409 | 0.85 |

Results Statistics

| Results Statistics parameter name | Results Statistics parameter value |
| --- | --- |
| Length adjustment | 35 |
| Effective length of query | 954 |
| Effective length of database | 172403749254 |
| Effective search space | 164473176788316 |
| Effective search space used | 164473176788316 |


## Graphic Summary

### Distribution of the top 121 Blast Hits on 100 subject sequences [?]

The graphic is an overview of the database sequences aligned to the query sequence. These are represented horizontal bars colored coded by score and showing the extent
of the alignment on the query sequence. Separate aligned regions on the same database sequence are connected by a thin grey line.
Mousing over an alignment shows the database sequence title. Clicking an alignment displays a box with more details about the alignment and
link to the sequence alignment itself in the Alignments section of the report.

Mouse over to see the title, click to show alignments

Color key for alignment scores

<40

40-50

50-80

80-200

>=200

Query

1

150

300

450

600

750

900

Pseudomonas trivialis strain BS3111 genome assembly, ch..

Score:1799 Evalue:0

Accession:LT629760.1

Alignment

Pseudomonas poae strain BS2776 genome assembly, chromos..

Score:1799 Evalue:0

Accession:LT629706.1

Alignment

Pseudomonas poae RE\*1-1-14, complete genome

Score:1799 Evalue:0

Accession:CP004045.1

Alignment

uncultured bacterium partial 16S rRNA gene, clone K15.8..

Score:1799 Evalue:0

Accession:HE576021.1

Alignment

Pseudomonas sp. PX7\_S1 16S ribosomal RNA gene, partial ..

Score:1799 Evalue:0

Accession:JF274937.1

Alignment

Uncultured bacterium clone ncd564g06c1 16S ribosomal RN..

Score:1799 Evalue:0

Accession:HM274624.1

Alignment

Pseudomonas trivialis strain P 513/19 16S ribosomal RNA..

Score:1799 Evalue:0

Accession:NR\_028987.1

Alignment

Pseudomonas poae strain P 527/13 16S ribosomal RNA gene..

Score:1799 Evalue:0

Accession:NR\_028986.1

Alignment

Pseudomonas poae strain T23 16S ribosomal RNA gene, com..

Score:1799 Evalue:0

Accession:EU111704.2

Alignment

Pseudomonas sp. LBSA1 16S ribosomal RNA gene, partial s..

Score:1799 Evalue:0

Accession:AY756059.1

Alignment

Pseudomonas trivialis partial 16S rRNA gene, type strai..

Score:1799 Evalue:0

Accession:AJ492831.1

Alignment

Pseudomonas poae partial 16S rRNA gene, type strain DSM..

Score:1799 Evalue:0

Accession:AJ492829.1

Alignment

Pseudomonas sp. BBI9 16S ribosomal RNA gene, partial se..

Score:1796 Evalue:0

Accession:GQ868355.1

Alignment

Pseudomonas sp. BR3-39 16S ribosomal RNA gene, partial ..

Score:1796 Evalue:0

Accession:EU853186.1

Alignment

Pseudomonas sp. BR2-22 16S ribosomal RNA gene, partial ..

Score:1796 Evalue:0

Accession:EU853183.1

Alignment

Pseudomonas sp. BR2-02 16S ribosomal RNA gene, partial ..

Score:1796 Evalue:0

Accession:EU853182.1

Alignment

Pseudomonas sp. S09G 359 chromosome

Score:1794 Evalue:0

Accession:CP025263.1

Alignment

Pseudomonas trivialis strain LMTK28 16S ribosomal RNA g..

Score:1794 Evalue:0

Accession:KY614180.1

Alignment

Pseudomonas poae strain ICE349 16S ribosomal RNA gene, ..

Score:1794 Evalue:0

Accession:KX588598.1

Alignment

Pseudomonas sp. 8K8 16S ribosomal RNA gene, partial seq..

Score:1794 Evalue:0

Accession:KT825845.1

Alignment

Pseudomonas sp. 23K33a 16S ribosomal RNA gene, partial ..

Score:1794 Evalue:0

Accession:KT825765.1

Alignment

Pseudomonas sp. 17K8 16S ribosomal RNA gene, partial se..

Score:1794 Evalue:0

Accession:KT825736.1

Alignment

Pseudomonas sp. LB8 16S ribosomal RNA gene, partial seq..

Score:1794 Evalue:0

Accession:KT424973.1

Alignment

Pseudomonas poae strain B42 16S ribosomal RNA gene, par..

Score:1794 Evalue:0

Accession:KT767705.1

Alignment

Uncultured Pseudomonas sp. partial 16S rRNA gene, clone..

Score:1794 Evalue:0

Accession:LN875096.1

Alignment

Pseudomonas poae strain IHBB 11151 16S ribosomal RNA ge..

Score:1794 Evalue:0

Accession:KR085862.1

Alignment

Pseudomonas poae strain IHBB 9211 16S ribosomal RNA gen..

Score:1794 Evalue:0

Accession:KR085822.1

Alignment

Uncultured bacterium clone OTU51 16S ribosomal RNA gene..

Score:1794 Evalue:0

Accession:KP975307.1

Alignment

Uncultured bacterium clone 19-16 16S ribosomal RNA gene..

Score:1794 Evalue:0

Accession:KT029380.1

Alignment

Uncultured bacterium clone 19-14 16S ribosomal RNA gene..

Score:1794 Evalue:0

Accession:KT029378.1

Alignment

Pseudomonas trivialis strain IHBB745, complete genome

Score:1794 Evalue:0

Accession:CP011507.1

Alignment

Bacterium MA\_26 16S ribosomal RNA gene, partial sequenc..

Score:1794 Evalue:0

Accession:KP204130.1

Alignment

Pseudomonas poae strain MA\_25 16S ribosomal RNA gene, p..

Score:1794 Evalue:0

Accession:KP204129.1

Alignment

Pseudomonas sp. Bma14 16S ribosomal RNA gene, partial s..

Score:1794 Evalue:0

Accession:JQ977483.1

Alignment

Pseudomonas sp. Ms16 16S ribosomal RNA gene, partial se..

Score:1794 Evalue:0

Accession:JQ977324.1

Alignment

Pseudomonas sp. Ms14 16S ribosomal RNA gene, partial se..

Score:1794 Evalue:0

Accession:JQ977322.1

Alignment

Pseudomonas salomonii strain CCNWQLS112 16S ribosomal R..

Score:1794 Evalue:0

Accession:JX840381.1

Alignment

Pseudomonas poae strain EB20 16S ribosomal RNA gene, pa..

Score:1794 Evalue:0

Accession:JX515573.1

Alignment

Pseudomonas sp. SAP770.2 16S ribosomal RNA gene, partia..

Score:1794 Evalue:0

Accession:JX067683.1

Alignment

Pseudomonas poae strain zol-15 16S ribosomal RNA gene, ..

Score:1794 Evalue:0

Accession:JQ782898.1

Alignment

Pseudomonas sp. ZBC1 16S ribosomal RNA gene, partial se..

Score:1794 Evalue:0

Accession:JQ638271.1

Alignment

Pseudomonas poae strain MML1950 16S ribosomal RNA gene,..

Score:1794 Evalue:0

Accession:JQ336974.1

Alignment

Pseudomonas sp. 5(2011) 16S ribosomal RNA gene, partial..

Score:1794 Evalue:0

Accession:JF808098.1

Alignment

Uncultured bacterium clone 3-42017 16S ribosomal RNA ge..

Score:1794 Evalue:0

Accession:JN033052.1

Alignment

Pseudomonas poae strain P6 16S ribosomal RNA gene, part..

Score:1794 Evalue:0

Accession:HQ647258.1

Alignment

Pseudomonas poae strain FBRh6 16S ribosomal RNA gene, p..

Score:1794 Evalue:0

Accession:HQ443230.1

Alignment

Uncultured bacterium clone EDW07B001\_111 16S ribosomal ..

Score:1794 Evalue:0

Accession:HM066261.1

Alignment

Pseudomonas trivialis isolate PDD-32b-53 16S ribosomal ..

Score:1794 Evalue:0

Accession:HQ256851.1

Alignment

Uncultured bacterium clone ncd2648e11c1 16S ribosomal R..

Score:1794 Evalue:0

Accession:JF230879.1

Alignment

Uncultured bacterium clone ncd2619g02c1 16S ribosomal R..

Score:1794 Evalue:0

Accession:JF228956.1

Alignment

Uncultured bacterium clone ncd2408f08c1 16S ribosomal R..

Score:1794 Evalue:0

Accession:JF209455.1

Alignment

Uncultured bacterium clone ncd2405h01c1 16S ribosomal R..

Score:1794 Evalue:0

Accession:JF209277.1

Alignment

Uncultured bacterium clone ncd2396f05c1 16S ribosomal R..

Score:1794 Evalue:0

Accession:JF208644.1

Alignment

Uncultured bacterium clone ncd2408f01c1 16S ribosomal R..

Score:1794 Evalue:0

Accession:JF200837.1

Alignment

Marine bacterium B23 gene for 16S ribosomal RNA, partia..

Score:1794 Evalue:0

Accession:AB607148.1

Alignment

Pseudomonas poae strain BCHCNZ253 16S ribosomal RNA gen..

Score:1794 Evalue:0

Accession:GU188947.1

Alignment

## Descriptions

, Reading indexes 1-5, displaying indexes 1-5


Load next setPrevious Match

Sequences producing significant alignments:

Show all columns  of the table presenting sequences producing significant alignments 

Select:AllNone
Selected:0

Alignments
Download

FASTA (complete sequence)

FASTA (aligned sequences)

GenBank (complete sequence)

Hit Table (text)

Hit Table (CSV)

Text

XML

ASN.1

Continue
Cancel

GenBank 
Graphics
Distance tree of results
Multiple alignment
Show/hide columns of the table presenting sequences producing significant alignments 

Available columns

Description  
Max Score  
Total Score  
Coverage  
E-value  
IdentN  
Accession  
Restore Defaults
Ok
Cancel

Sequences producing significant alignments:

| Select for downloading or viewing reports | Description | Max score | Total score | Query cover | E value | Ident | Accession |
| --- | --- | --- | --- | --- | --- | --- | --- |
| 1Select seq LT629760.1 | Pseudomonas trivialis strain BS3111 genome assembly, chromosome: I | 1799 | 8993 | 99% | 0.0 | 99% | LT629760.1 |
| 2Select seq LT629706.1 | Pseudomonas poae strain BS2776 genome assembly, chromosome: I | 1799 | 8998 | 99% | 0.0 | 99% | LT629706.1 |
| 3Select seq CP004045.1 | Pseudomonas poae RE\*1-1-14, complete genome | 1799 | 8993 | 99% | 0.0 | 99% | CP004045.1 |
| 4Select seq HE576021.1 | uncultured bacterium partial 16S rRNA gene, clone K15.87 KSS | 1799 | 1799 | 99% | 0.0 | 99% | HE576021.1 |
| 5Select seq JF274937.1 | Pseudomonas sp. PX7\_S1 16S ribosomal RNA gene, partial sequence | 1799 | 1799 | 99% | 0.0 | 99% | JF274937.1 |
| 6Select seq HM274624.1 | Uncultured bacterium clone ncd564g06c1 16S ribosomal RNA gene, partial sequence | 1799 | 1799 | 99% | 0.0 | 99% | HM274624.1 |
| 7Select seq NR\_028987.1 | Pseudomonas trivialis strain P 513/19 16S ribosomal RNA gene, partial sequence | 1799 | 1799 | 99% | 0.0 | 99% | NR\_028987.1 |
| 8Select seq NR\_028986.1 | Pseudomonas poae strain P 527/13 16S ribosomal RNA gene, partial sequence | 1799 | 1799 | 99% | 0.0 | 99% | NR\_028986.1 |
| 9Select seq EU111704.2 | Pseudomonas poae strain T23 16S ribosomal RNA gene, complete sequence | 1799 | 1799 | 99% | 0.0 | 99% | EU111704.2 |
| 10Select seq AY756059.1 | Pseudomonas sp. LBSA1 16S ribosomal RNA gene, partial sequence; internal transcribed spacer 1, complete sequence; and 23S ribosomal RNA gene, partial sequence | 1799 | 1799 | 99% | 0.0 | 99% | AY756059.1 |
| 11Select seq AJ492831.1 | Pseudomonas trivialis partial 16S rRNA gene, type strain DSM 14937T | 1799 | 1799 | 99% | 0.0 | 99% | AJ492831.1 |
| 12Select seq AJ492829.1 | Pseudomonas poae partial 16S rRNA gene, type strain DSM 14936T | 1799 | 1799 | 99% | 0.0 | 99% | AJ492829.1 |
| 13Select seq GQ868355.1 | Pseudomonas sp. BBI9 16S ribosomal RNA gene, partial sequence | 1796 | 1796 | 99% | 0.0 | 99% | GQ868355.1 |
| 14Select seq EU853186.1 | Pseudomonas sp. BR3-39 16S ribosomal RNA gene, partial sequence | 1796 | 1796 | 99% | 0.0 | 99% | EU853186.1 |
| 15Select seq EU853183.1 | Pseudomonas sp. BR2-22 16S ribosomal RNA gene, partial sequence | 1796 | 1796 | 99% | 0.0 | 99% | EU853183.1 |
| 16Select seq EU853182.1 | Pseudomonas sp. BR2-02 16S ribosomal RNA gene, partial sequence | 1796 | 1796 | 99% | 0.0 | 99% | EU853182.1 |
| 17Select seq CP025263.1 | Pseudomonas sp. S09G 359 chromosome | 1794 | 10759 | 99% | 0.0 | 99% | CP025263.1 |
| 18Select seq KY614180.1 | Pseudomonas trivialis strain LMTK28 16S ribosomal RNA gene, partial sequence | 1794 | 1794 | 99% | 0.0 | 99% | KY614180.1 |
| 19Select seq KX588598.1 | Pseudomonas poae strain ICE349 16S ribosomal RNA gene, partial sequence | 1794 | 1794 | 99% | 0.0 | 99% | KX588598.1 |
| 20Select seq KT825845.1 | Pseudomonas sp. 8K8 16S ribosomal RNA gene, partial sequence | 1794 | 1794 | 99% | 0.0 | 99% | KT825845.1 |
| 21Select seq KT825765.1 | Pseudomonas sp. 23K33a 16S ribosomal RNA gene, partial sequence | 1794 | 1794 | 99% | 0.0 | 99% | KT825765.1 |
| 22Select seq KT825736.1 | Pseudomonas sp. 17K8 16S ribosomal RNA gene, partial sequence | 1794 | 1794 | 99% | 0.0 | 99% | KT825736.1 |
| 23Select seq KT424973.1 | Pseudomonas sp. LB8 16S ribosomal RNA gene, partial sequence | 1794 | 1794 | 99% | 0.0 | 99% | KT424973.1 |
| 24Select seq KT767705.1 | Pseudomonas poae strain B42 16S ribosomal RNA gene, partial sequence | 1794 | 1794 | 99% | 0.0 | 99% | KT767705.1 |
| 25Select seq LN875096.1 | Uncultured Pseudomonas sp. partial 16S rRNA gene, clone 133X76C\_34 | 1794 | 1794 | 99% | 0.0 | 99% | LN875096.1 |
| 26Select seq KR085862.1 | Pseudomonas poae strain IHBB 11151 16S ribosomal RNA gene, partial sequence | 1794 | 1794 | 99% | 0.0 | 99% | KR085862.1 |
| 27Select seq KR085822.1 | Pseudomonas poae strain IHBB 9211 16S ribosomal RNA gene, partial sequence | 1794 | 1794 | 99% | 0.0 | 99% | KR085822.1 |
| 28Select seq KP975307.1 | Uncultured bacterium clone OTU51 16S ribosomal RNA gene, partial sequence | 1794 | 1794 | 99% | 0.0 | 99% | KP975307.1 |
| 29Select seq KT029380.1 | Uncultured bacterium clone 19-16 16S ribosomal RNA gene, partial sequence | 1794 | 1794 | 99% | 0.0 | 99% | KT029380.1 |
| 30Select seq KT029378.1 | Uncultured bacterium clone 19-14 16S ribosomal RNA gene, partial sequence | 1794 | 1794 | 99% | 0.0 | 99% | KT029378.1 |
| 31Select seq CP011507.1 | Pseudomonas trivialis strain IHBB745, complete genome | 1794 | 8965 | 99% | 0.0 | 99% | CP011507.1 |
| 32Select seq KP204130.1 | Bacterium MA\_26 16S ribosomal RNA gene, partial sequence | 1794 | 1794 | 99% | 0.0 | 99% | KP204130.1 |
| 33Select seq KP204129.1 | Pseudomonas poae strain MA\_25 16S ribosomal RNA gene, partial sequence | 1794 | 1794 | 99% | 0.0 | 99% | KP204129.1 |
| 34Select seq JQ977483.1 | Pseudomonas sp. Bma14 16S ribosomal RNA gene, partial sequence | 1794 | 1794 | 99% | 0.0 | 99% | JQ977483.1 |
| 35Select seq JQ977324.1 | Pseudomonas sp. Ms16 16S ribosomal RNA gene, partial sequence | 1794 | 1794 | 99% | 0.0 | 99% | JQ977324.1 |
| 36Select seq JQ977322.1 | Pseudomonas sp. Ms14 16S ribosomal RNA gene, partial sequence | 1794 | 1794 | 99% | 0.0 | 99% | JQ977322.1 |
| 37Select seq JX840381.1 | Pseudomonas salomonii strain CCNWQLS112 16S ribosomal RNA gene, partial sequence | 1794 | 1794 | 99% | 0.0 | 99% | JX840381.1 |
| 38Select seq JX515573.1 | Pseudomonas poae strain EB20 16S ribosomal RNA gene, partial sequence | 1794 | 1794 | 99% | 0.0 | 99% | JX515573.1 |
| 39Select seq JX067683.1 | Pseudomonas sp. SAP770.2 16S ribosomal RNA gene, partial sequence | 1794 | 1794 | 99% | 0.0 | 99% | JX067683.1 |
| 40Select seq JQ782898.1 | Pseudomonas poae strain zol-15 16S ribosomal RNA gene, partial sequence | 1794 | 1794 | 99% | 0.0 | 99% | JQ782898.1 |
| 41Select seq JQ638271.1 | Pseudomonas sp. ZBC1 16S ribosomal RNA gene, partial sequence | 1794 | 1794 | 99% | 0.0 | 99% | JQ638271.1 |
| 42Select seq JQ336974.1 | Pseudomonas poae strain MML1950 16S ribosomal RNA gene, partial sequence | 1794 | 1794 | 99% | 0.0 | 99% | JQ336974.1 |
| 43Select seq JF808098.1 | Pseudomonas sp. 5(2011) 16S ribosomal RNA gene, partial sequence | 1794 | 1794 | 99% | 0.0 | 99% | JF808098.1 |
| 44Select seq JN033052.1 | Uncultured bacterium clone 3-42017 16S ribosomal RNA gene, partial sequence | 1794 | 1794 | 99% | 0.0 | 99% | JN033052.1 |
| 45Select seq HQ647258.1 | Pseudomonas poae strain P6 16S ribosomal RNA gene, partial sequence | 1794 | 1794 | 99% | 0.0 | 99% | HQ647258.1 |
| 46Select seq HQ443230.1 | Pseudomonas poae strain FBRh6 16S ribosomal RNA gene, partial sequence | 1794 | 1794 | 99% | 0.0 | 99% | HQ443230.1 |
| 47Select seq HM066261.1 | Uncultured bacterium clone EDW07B001\_111 16S ribosomal RNA gene, partial sequence | 1794 | 1794 | 99% | 0.0 | 99% | HM066261.1 |
| 48Select seq HQ256851.1 | Pseudomonas trivialis isolate PDD-32b-53 16S ribosomal RNA gene, partial sequence | 1794 | 1794 | 99% | 0.0 | 99% | HQ256851.1 |
| 49Select seq JF230879.1 | Uncultured bacterium clone ncd2648e11c1 16S ribosomal RNA gene, partial sequence | 1794 | 1794 | 99% | 0.0 | 99% | JF230879.1 |
| 50Select seq JF228956.1 | Uncultured bacterium clone ncd2619g02c1 16S ribosomal RNA gene, partial sequence | 1794 | 1794 | 99% | 0.0 | 99% | JF228956.1 |
| 51Select seq JF209455.1 | Uncultured bacterium clone ncd2408f08c1 16S ribosomal RNA gene, partial sequence | 1794 | 1794 | 99% | 0.0 | 99% | JF209455.1 |
| 52Select seq JF209277.1 | Uncultured bacterium clone ncd2405h01c1 16S ribosomal RNA gene, partial sequence | 1794 | 1794 | 99% | 0.0 | 99% | JF209277.1 |
| 53Select seq JF208644.1 | Uncultured bacterium clone ncd2396f05c1 16S ribosomal RNA gene, partial sequence | 1794 | 1794 | 99% | 0.0 | 99% | JF208644.1 |
| 54Select seq JF200837.1 | Uncultured bacterium clone ncd2408f01c1 16S ribosomal RNA gene, partial sequence | 1794 | 1794 | 99% | 0.0 | 99% | JF200837.1 |
| 55Select seq AB607148.1 | Marine bacterium B23 gene for 16S ribosomal RNA, partial sequence | 1794 | 1794 | 99% | 0.0 | 99% | AB607148.1 |
| 56Select seq GU188947.1 | Pseudomonas poae strain BCHCNZ253 16S ribosomal RNA gene, partial sequence | 1794 | 1794 | 99% | 0.0 | 99% | GU188947.1 |
| 57Select seq FJ937929.1 | Pseudomonas sp. LS227 16S ribosomal RNA gene, partial sequence | 1794 | 1794 | 99% | 0.0 | 99% | FJ937929.1 |
| 58Select seq FJ937922.1 | Pseudomonas poae strain LS172 16S ribosomal RNA gene, partial sequence | 1794 | 1794 | 99% | 0.0 | 99% | FJ937922.1 |
| 59Select seq FJ179366.1 | Pseudomonas trivialis strain BIHB 750 16S ribosomal RNA gene, partial sequence | 1794 | 1794 | 99% | 0.0 | 99% | FJ179366.1 |
| 60Select seq EU540146.1 | Uncultured bacterium clone nbt246b03 16S ribosomal RNA gene, partial sequence | 1794 | 1794 | 99% | 0.0 | 99% | EU540146.1 |
| 61Select seq EU360313.1 | Pseudomonas fluorescens strain 2312 16S ribosomal RNA gene and 16S-23S ribosomal RNA intergenic spacer, complete sequence; and 23S ribosomal RNA gene, partial sequence | 1794 | 1794 | 99% | 0.0 | 99% | EU360313.1 |
| 62Select seq EF028122.1 | Pseudomonas sp. Dui-7 16S ribosomal RNA gene, partial sequence | 1794 | 1794 | 99% | 0.0 | 99% | EF028122.1 |
| 63Select seq DQ885949.1 | Pseudomonas trivialis strain BIHB 749 16S ribosomal RNA gene, partial sequence | 1794 | 1794 | 99% | 0.0 | 99% | DQ885949.1 |
| 64Select seq DQ536516.1 | Pseudomonas trivialis strain BIHB 745 16S ribosomal RNA gene, partial sequence | 1794 | 1794 | 99% | 0.0 | 99% | DQ536516.1 |
| 65Select seq AY263477.1 | Pseudomonas sp. SE22#1a 16S ribosomal RNA gene, partial sequence | 1794 | 1794 | 99% | 0.0 | 99% | AY263477.1 |
| 66Select seq AF320987.1 | Pseudomonas reactans 16S ribosomal RNA gene, partial sequence | 1794 | 1794 | 99% | 0.0 | 99% | AF320987.1 |
| 67Select seq AY959122.1 | Uncultured bacterium clone rRNA349 16S ribosomal RNA gene, partial sequence | 1794 | 1794 | 99% | 0.0 | 99% | AY959122.1 |
| 68Select seq AY599721.1 | Pseudomonas sp. TB3-6-I 16S ribosomal RNA gene, partial sequence | 1794 | 1794 | 99% | 0.0 | 99% | AY599721.1 |
| 69Select seq AJ556801.1 | Pseudomonas sp. MWH1 partial 16S rRNA gene | 1794 | 1794 | 99% | 0.0 | 99% | AJ556801.1 |
| 70Select seq KR922148.1 | Pseudomonas sp. PDD-59b-10 16S ribosomal RNA gene, complete sequence | 1792 | 1792 | 99% | 0.0 | 99% | KR922148.1 |
| 71Select seq MG576155.1 | Pseudomonas sp. strain KBL2 16S ribosomal RNA gene, partial sequence | 1790 | 1790 | 99% | 0.0 | 99% | MG576155.1 |
| 72Select seq KY940313.1 | Pseudomonas poae strain A2B5 16S ribosomal RNA gene, partial sequence | 1790 | 1790 | 99% | 0.0 | 99% | KY940313.1 |
| 73Select seq KY810658.1 | Pseudomonas poae strain AAS-144 16S ribosomal RNA gene, partial sequence | 1790 | 1790 | 98% | 0.0 | 99% | KY810658.1 |
| 74Select seq KX588597.1 | Pseudomonas poae strain ICE259 16S ribosomal RNA gene, partial sequence | 1790 | 1790 | 99% | 0.0 | 99% | KX588597.1 |
| 75Select seq KX378900.1 | Pseudomonas sp. strain 1-29 16S ribosomal RNA gene, partial sequence | 1790 | 1790 | 98% | 0.0 | 99% | KX378900.1 |
| 76Select seq KT710812.1 | Pseudomonas sp. RZ110 16S ribosomal RNA gene, partial sequence | 1790 | 1790 | 99% | 0.0 | 99% | KT710812.1 |
| 77Select seq JQ691542.1 | Pseudomonas sp. FI 1007 16S ribosomal RNA gene, partial sequence | 1790 | 1790 | 99% | 0.0 | 99% | JQ691542.1 |
| 78Select seq HQ224624.1 | Pseudomonas sp. SGb136 16S ribosomal RNA gene, partial sequence | 1790 | 1790 | 98% | 0.0 | 99% | HQ224624.1 |
| 79Select seq JN377670.1 | Pseudomonas trivialis strain B24 16S ribosomal RNA gene, partial sequence | 1790 | 1790 | 99% | 0.0 | 99% | JN377670.1 |
| 80Select seq JF820107.1 | Pseudomonas sp. PG-3-10 16S ribosomal RNA gene, partial sequence | 1790 | 1790 | 99% | 0.0 | 99% | JF820107.1 |
| 81Select seq FJ662886.1 | Pseudomonas sp. VS05\_25 16S ribosomal RNA gene, partial sequence | 1790 | 1790 | 99% | 0.0 | 99% | FJ662886.1 |
| 82Select seq KX378948.1 | Pseudomonas sp. strain 3-10 16S ribosomal RNA gene, partial sequence | 1788 | 1788 | 98% | 0.0 | 99% | KX378948.1 |
| 83Select seq KX509247.1 | Uncultured bacterium clone MTWL201306-19 16S ribosomal RNA gene, partial sequence | 1788 | 1788 | 99% | 0.0 | 99% | KX509247.1 |
| 84Select seq KX079774.1 | Pseudomonas sp. MYb117 16S ribosomal RNA gene, partial sequence | 1788 | 1788 | 99% | 0.0 | 99% | KX079774.1 |
| 85Select seq KX079768.1 | Pseudomonas sp. MYb114 16S ribosomal RNA gene, partial sequence | 1788 | 1788 | 99% | 0.0 | 99% | KX079768.1 |
| 86Select seq LC140795.1 | Uncultured Pseudomonas sp. gene for 16S ribosomal RNA, partial sequence, clone: 1d18 | 1788 | 1788 | 99% | 0.0 | 99% | LC140795.1 |
| 87Select seq KT825760.1 | Pseudomonas sp. 23K17 16S ribosomal RNA gene, partial sequence | 1788 | 1788 | 99% | 0.0 | 99% | KT825760.1 |
| 88Select seq HG805797.1 | Pseudomonas sp. FBF128 partial 16S rRNA gene, isolate FBF128 | 1788 | 1788 | 99% | 0.0 | 99% | HG805797.1 |
| 89Select seq KT890311.1 | Pseudomonas sp. S35 16S ribosomal RNA gene, partial sequence | 1788 | 1788 | 99% | 0.0 | 99% | KT890311.1 |
| 90Select seq KT767829.1 | Pseudomonas fluorescens strain C40 16S ribosomal RNA gene, partial sequence | 1788 | 1788 | 99% | 0.0 | 99% | KT767829.1 |
| 91Select seq KT029445.1 | Uncultured bacterium clone 21-10 16S ribosomal RNA gene, partial sequence | 1788 | 1788 | 99% | 0.0 | 99% | KT029445.1 |
| 92Select seq KT029387.1 | Uncultured bacterium clone 19-23 16S ribosomal RNA gene, partial sequence | 1788 | 1788 | 99% | 0.0 | 99% | KT029387.1 |
| 93Select seq KT029382.1 | Uncultured bacterium clone 19-18 16S ribosomal RNA gene, partial sequence | 1788 | 1788 | 99% | 0.0 | 99% | KT029382.1 |
| 94Select seq KP204146.1 | Bacterium NSA\_23 16S ribosomal RNA gene, partial sequence | 1788 | 1788 | 99% | 0.0 | 99% | KP204146.1 |
| 95Select seq KP204140.1 | Pseudomonas poae strain NA\_1 16S ribosomal RNA gene, partial sequence | 1788 | 1788 | 99% | 0.0 | 99% | KP204140.1 |
| 96Select seq JQ727461.1 | Bacterium enrichment culture clone SaD1 16S ribosomal RNA gene, partial sequence | 1788 | 1788 | 98% | 0.0 | 99% | JQ727461.1 |
| 97Select seq JQ236630.1 | Pseudomonas poae strain ORC5 16S ribosomal RNA gene, partial sequence | 1788 | 1788 | 98% | 0.0 | 99% | JQ236630.1 |
| 98Select seq GQ868350.1 | Pseudomonas sp. BBI2 16S ribosomal RNA gene, partial sequence | 1788 | 1788 | 98% | 0.0 | 99% | GQ868350.1 |
| 99Select seq KY742759.1 | Pseudomonas sp. strain 8.12.1 16S ribosomal RNA gene, partial sequence | 1786 | 1786 | 99% | 0.0 | 99% | KY742759.1 |
| 100Select seq JQ727438.1 | Bacterium enrichment culture clone SaA1 16S ribosomal RNA gene, partial sequence | 1786 | 1786 | 98% | 0.0 | 99% | JQ727438.1 |


## Alignments

Loading alignment... for sequences gi|1085875934,gi|1085717528,gi|445198867,gi|377550157,gi|332000891 Reading indexes 1-5

Download

FASTA (complete sequence)

FASTA (aligned sequences)

GenBank (complete sequence)

Continue
Cancel

GenBankGraphics

Sort by:

E value
 Score
Percent identity
Query start position
Subject start position

Next
Previous
Descriptions

Pseudomonas trivialis strain BS3111 genome assembly, chromosome: I

Sequence ID: LT629760.1Length: 5634667Number of Matches: 5

Related Information

Range 1: 453454 to 454433GenBankGraphics

Next Match
Previous Match
First Match

Alignment statistics for match #1

| Score | Expect | Identities | Gaps | Strand | Frame |
| --- | --- | --- | --- | --- | --- |
| 1799 bits(974) | 0.0() | 977/980(99%) | 0/980(0%) | Plus/Minus |  |

Features:

rRNA-16S ribosomal RNA . Bacterial SSU

```
Query  6       TGCNAGTCGAGCGGTAGAGAGAAGCTTGCTTCTCTTGAGAGCGGCGGACGGGTGAGTAAT  65
               ||| ||||||||||||||||||||||||||||||||||||||||||||||||||||||||
Sbjct  454433  TGCAAGTCGAGCGGTAGAGAGAAGCTTGCTTCTCTTGAGAGCGGCGGACGGGTGAGTAAT  454374

Query  66      GCCTAGGAATCTGCCTGGTAGTGGGGGATAACGTTCGGAAACGGACGCTAATACCGCATA  125
               ||||||||||||||||||||||||||||||||||||||||||||||||||||||||||||
Sbjct  454373  GCCTAGGAATCTGCCTGGTAGTGGGGGATAACGTTCGGAAACGGACGCTAATACCGCATA  454314

Query  126     CGTCCTACGGGAGAAAGCAGGGGACCTTCGGGCCTTGCGCTATCAGATGAGCCTAGGTCG  185
               ||||||||||||||||||||||||||||||||||||||||||||||||||||||||||||
Sbjct  454313  CGTCCTACGGGAGAAAGCAGGGGACCTTCGGGCCTTGCGCTATCAGATGAGCCTAGGTCG  454254

Query  186     GATTAGCTAGTTGGTGGGGTAATGGCTCACCAAGGCGACGATCCGTAACTGGTCTGAGAG  245
               ||||||||||||||||||||||||||||||||||||||||||||||||||||||||||||
Sbjct  454253  GATTAGCTAGTTGGTGGGGTAATGGCTCACCAAGGCGACGATCCGTAACTGGTCTGAGAG  454194

Query  246     GATGATCAGTCACACTGGAACTGAGACACGGTCCAGACTCCTACGGGAGGCAGCAGTGGG  305
               ||||||||||||||||||||||||||||||||||||||||||||||||||||||||||||
Sbjct  454193  GATGATCAGTCACACTGGAACTGAGACACGGTCCAGACTCCTACGGGAGGCAGCAGTGGG  454134

Query  306     GAATATTGGACAATGGGCGAAAGCCTGATCCAGCCATGCCGCGTGTGTGAAGAAGGTCTT  365
               ||||||||||||||||||||||||||||||||||||||||||||||||||||||||||||
Sbjct  454133  GAATATTGGACAATGGGCGAAAGCCTGATCCAGCCATGCCGCGTGTGTGAAGAAGGTCTT  454074

Query  366     CGGATTGTAAAGCACTTTAAGTTGGGAGGAAGGGCAGTTACCTAATACGTGATTGTTTTG  425
               ||||||||||||||||||||||||||||||||||||||||||||||||||||||||||||
Sbjct  454073  CGGATTGTAAAGCACTTTAAGTTGGGAGGAAGGGCAGTTACCTAATACGTGATTGTTTTG  454014

Query  426     ACGTTACCGACAGAATAAGCACCGGCTAACTCTGTGCCAGCAGCCGCGGTAATACAGAGG  485
               ||||||||||||||||||||||||||||||||||||||||||||||||||||||||||||
Sbjct  454013  ACGTTACCGACAGAATAAGCACCGGCTAACTCTGTGCCAGCAGCCGCGGTAATACAGAGG  453954

Query  486     GTGCAAGCGTTAATCGGAATTACTGGGCGTAAAGCGCGCGTAGGTGGTTTGTTAAGTTGG  545
               ||||||||||||||||||||||||||||||||||||||||||||||||||||||||||||
Sbjct  453953  GTGCAAGCGTTAATCGGAATTACTGGGCGTAAAGCGCGCGTAGGTGGTTTGTTAAGTTGG  453894

Query  546     ATGTGAAATCCCCGGGCTCAACCTGGGAACTGCATTCAAAACTGACTGACTAGAGTATGG  605
               ||||||||||||||||||||||||||||||||||||||||||||||||||||||||||||
Sbjct  453893  ATGTGAAATCCCCGGGCTCAACCTGGGAACTGCATTCAAAACTGACTGACTAGAGTATGG  453834

Query  606     TAGAGGGTGGTGGAATTTCCTGTGTAGCGGTGAAATGCGTAGATATAGGAAGGAACACCA  665
               ||||||||||||||||||||||||||||||||||||||||||||||||||||||||||||
Sbjct  453833  TAGAGGGTGGTGGAATTTCCTGTGTAGCGGTGAAATGCGTAGATATAGGAAGGAACACCA  453774

Query  666     GTGGCGAAGGCGACCACCTGGACTAATACTGACACTGAGGTGCGAAAGCGTGGGGAGCAA  725
               ||||||||||||||||||||||||||||||||||||||||||||||||||||||||||||
Sbjct  453773  GTGGCGAAGGCGACCACCTGGACTAATACTGACACTGAGGTGCGAAAGCGTGGGGAGCAA  453714

Query  726     ACAGGATTAGATACCCTGGTAGTCCACGCCGTAAACGATGTCAACTAGCCGTTGGAAGCC  785
               ||||||||||||||||||||||||||||||||||||||||||||||||||||||||||||
Sbjct  453713  ACAGGATTAGATACCCTGGTAGTCCACGCCGTAAACGATGTCAACTAGCCGTTGGAAGCC  453654

Query  786     TTGAGCTTTTAGTGGCGCAGCTAACGCATTAAGTTGACCGCCTGGGGAGTACGGCCGCAA  845
               ||||||||||||||||||||||||||||||||||||||||||||||||||||||||||||
Sbjct  453653  TTGAGCTTTTAGTGGCGCAGCTAACGCATTAAGTTGACCGCCTGGGGAGTACGGCCGCAA  453594

Query  846     GGTTAAAACTCAAATGAATTGACGGGGGCCCGCACAAGCGNTGGNGCATGTGGTTTAATT  905
               |||||||||||||||||||||||||||||||||||||||| ||| |||||||||||||||
Sbjct  453593  GGTTAAAACTCAAATGAATTGACGGGGGCCCGCACAAGCGGTGGAGCATGTGGTTTAATT  453534

Query  906     CGAAGCAACGCGAAGAACCTTACCAGGCCTTGACATCCAATGAACTTTCTAGAGATAGAT  965
               ||||||||||||||||||||||||||||||||||||||||||||||||||||||||||||
Sbjct  453533  CGAAGCAACGCGAAGAACCTTACCAGGCCTTGACATCCAATGAACTTTCTAGAGATAGAT  453474

Query  966     TGGTGCCTTCGGGAACATTG  985
               ||||||||||||||||||||
Sbjct  453473  TGGTGCCTTCGGGAACATTG  453454
```

Range 2: 812105 to 813084GenBankGraphics

Next Match
Previous Match
First Match

Alignment statistics for match #2

| Score | Expect | Identities | Gaps | Strand | Frame |
| --- | --- | --- | --- | --- | --- |
| 1799 bits(974) | 0.0() | 977/980(99%) | 0/980(0%) | Plus/Minus |  |

Features:

rRNA-16S ribosomal RNA . Bacterial SSU

```
Query  6       TGCNAGTCGAGCGGTAGAGAGAAGCTTGCTTCTCTTGAGAGCGGCGGACGGGTGAGTAAT  65
               ||| ||||||||||||||||||||||||||||||||||||||||||||||||||||||||
Sbjct  813084  TGCAAGTCGAGCGGTAGAGAGAAGCTTGCTTCTCTTGAGAGCGGCGGACGGGTGAGTAAT  813025

Query  66      GCCTAGGAATCTGCCTGGTAGTGGGGGATAACGTTCGGAAACGGACGCTAATACCGCATA  125
               ||||||||||||||||||||||||||||||||||||||||||||||||||||||||||||
Sbjct  813024  GCCTAGGAATCTGCCTGGTAGTGGGGGATAACGTTCGGAAACGGACGCTAATACCGCATA  812965

Query  126     CGTCCTACGGGAGAAAGCAGGGGACCTTCGGGCCTTGCGCTATCAGATGAGCCTAGGTCG  185
               ||||||||||||||||||||||||||||||||||||||||||||||||||||||||||||
Sbjct  812964  CGTCCTACGGGAGAAAGCAGGGGACCTTCGGGCCTTGCGCTATCAGATGAGCCTAGGTCG  812905

Query  186     GATTAGCTAGTTGGTGGGGTAATGGCTCACCAAGGCGACGATCCGTAACTGGTCTGAGAG  245
               ||||||||||||||||||||||||||||||||||||||||||||||||||||||||||||
Sbjct  812904  GATTAGCTAGTTGGTGGGGTAATGGCTCACCAAGGCGACGATCCGTAACTGGTCTGAGAG  812845

Query  246     GATGATCAGTCACACTGGAACTGAGACACGGTCCAGACTCCTACGGGAGGCAGCAGTGGG  305
               ||||||||||||||||||||||||||||||||||||||||||||||||||||||||||||
Sbjct  812844  GATGATCAGTCACACTGGAACTGAGACACGGTCCAGACTCCTACGGGAGGCAGCAGTGGG  812785

Query  306     GAATATTGGACAATGGGCGAAAGCCTGATCCAGCCATGCCGCGTGTGTGAAGAAGGTCTT  365
               ||||||||||||||||||||||||||||||||||||||||||||||||||||||||||||
Sbjct  812784  GAATATTGGACAATGGGCGAAAGCCTGATCCAGCCATGCCGCGTGTGTGAAGAAGGTCTT  812725

Query  366     CGGATTGTAAAGCACTTTAAGTTGGGAGGAAGGGCAGTTACCTAATACGTGATTGTTTTG  425
               ||||||||||||||||||||||||||||||||||||||||||||||||||||||||||||
Sbjct  812724  CGGATTGTAAAGCACTTTAAGTTGGGAGGAAGGGCAGTTACCTAATACGTGATTGTTTTG  812665

Query  426     ACGTTACCGACAGAATAAGCACCGGCTAACTCTGTGCCAGCAGCCGCGGTAATACAGAGG  485
               ||||||||||||||||||||||||||||||||||||||||||||||||||||||||||||
Sbjct  812664  ACGTTACCGACAGAATAAGCACCGGCTAACTCTGTGCCAGCAGCCGCGGTAATACAGAGG  812605

Query  486     GTGCAAGCGTTAATCGGAATTACTGGGCGTAAAGCGCGCGTAGGTGGTTTGTTAAGTTGG  545
               ||||||||||||||||||||||||||||||||||||||||||||||||||||||||||||
Sbjct  812604  GTGCAAGCGTTAATCGGAATTACTGGGCGTAAAGCGCGCGTAGGTGGTTTGTTAAGTTGG  812545

Query  546     ATGTGAAATCCCCGGGCTCAACCTGGGAACTGCATTCAAAACTGACTGACTAGAGTATGG  605
               ||||||||||||||||||||||||||||||||||||||||||||||||||||||||||||
Sbjct  812544  ATGTGAAATCCCCGGGCTCAACCTGGGAACTGCATTCAAAACTGACTGACTAGAGTATGG  812485

Query  606     TAGAGGGTGGTGGAATTTCCTGTGTAGCGGTGAAATGCGTAGATATAGGAAGGAACACCA  665
               ||||||||||||||||||||||||||||||||||||||||||||||||||||||||||||
Sbjct  812484  TAGAGGGTGGTGGAATTTCCTGTGTAGCGGTGAAATGCGTAGATATAGGAAGGAACACCA  812425

Query  666     GTGGCGAAGGCGACCACCTGGACTAATACTGACACTGAGGTGCGAAAGCGTGGGGAGCAA  725
               ||||||||||||||||||||||||||||||||||||||||||||||||||||||||||||
Sbjct  812424  GTGGCGAAGGCGACCACCTGGACTAATACTGACACTGAGGTGCGAAAGCGTGGGGAGCAA  812365

Query  726     ACAGGATTAGATACCCTGGTAGTCCACGCCGTAAACGATGTCAACTAGCCGTTGGAAGCC  785
               ||||||||||||||||||||||||||||||||||||||||||||||||||||||||||||
Sbjct  812364  ACAGGATTAGATACCCTGGTAGTCCACGCCGTAAACGATGTCAACTAGCCGTTGGAAGCC  812305

Query  786     TTGAGCTTTTAGTGGCGCAGCTAACGCATTAAGTTGACCGCCTGGGGAGTACGGCCGCAA  845
               ||||||||||||||||||||||||||||||||||||||||||||||||||||||||||||
Sbjct  812304  TTGAGCTTTTAGTGGCGCAGCTAACGCATTAAGTTGACCGCCTGGGGAGTACGGCCGCAA  812245

Query  846     GGTTAAAACTCAAATGAATTGACGGGGGCCCGCACAAGCGNTGGNGCATGTGGTTTAATT  905
               |||||||||||||||||||||||||||||||||||||||| ||| |||||||||||||||
Sbjct  812244  GGTTAAAACTCAAATGAATTGACGGGGGCCCGCACAAGCGGTGGAGCATGTGGTTTAATT  812185

Query  906     CGAAGCAACGCGAAGAACCTTACCAGGCCTTGACATCCAATGAACTTTCTAGAGATAGAT  965
               ||||||||||||||||||||||||||||||||||||||||||||||||||||||||||||
Sbjct  812184  CGAAGCAACGCGAAGAACCTTACCAGGCCTTGACATCCAATGAACTTTCTAGAGATAGAT  812125

Query  966     TGGTGCCTTCGGGAACATTG  985
               ||||||||||||||||||||
Sbjct  812124  TGGTGCCTTCGGGAACATTG  812105
```

Range 3: 1552447 to 1553426GenBankGraphics

Next Match
Previous Match
First Match

Alignment statistics for match #3

| Score | Expect | Identities | Gaps | Strand | Frame |
| --- | --- | --- | --- | --- | --- |
| 1799 bits(974) | 0.0() | 977/980(99%) | 0/980(0%) | Plus/Plus |  |

Features:

rRNA-16S ribosomal RNA . Bacterial SSU

```
Query  6        TGCNAGTCGAGCGGTAGAGAGAAGCTTGCTTCTCTTGAGAGCGGCGGACGGGTGAGTAAT  65
                ||| ||||||||||||||||||||||||||||||||||||||||||||||||||||||||
Sbjct  1552447  TGCAAGTCGAGCGGTAGAGAGAAGCTTGCTTCTCTTGAGAGCGGCGGACGGGTGAGTAAT  1552506

Query  66       GCCTAGGAATCTGCCTGGTAGTGGGGGATAACGTTCGGAAACGGACGCTAATACCGCATA  125
                ||||||||||||||||||||||||||||||||||||||||||||||||||||||||||||
Sbjct  1552507  GCCTAGGAATCTGCCTGGTAGTGGGGGATAACGTTCGGAAACGGACGCTAATACCGCATA  1552566

Query  126      CGTCCTACGGGAGAAAGCAGGGGACCTTCGGGCCTTGCGCTATCAGATGAGCCTAGGTCG  185
                ||||||||||||||||||||||||||||||||||||||||||||||||||||||||||||
Sbjct  1552567  CGTCCTACGGGAGAAAGCAGGGGACCTTCGGGCCTTGCGCTATCAGATGAGCCTAGGTCG  1552626

Query  186      GATTAGCTAGTTGGTGGGGTAATGGCTCACCAAGGCGACGATCCGTAACTGGTCTGAGAG  245
                ||||||||||||||||||||||||||||||||||||||||||||||||||||||||||||
Sbjct  1552627  GATTAGCTAGTTGGTGGGGTAATGGCTCACCAAGGCGACGATCCGTAACTGGTCTGAGAG  1552686

Query  246      GATGATCAGTCACACTGGAACTGAGACACGGTCCAGACTCCTACGGGAGGCAGCAGTGGG  305
                ||||||||||||||||||||||||||||||||||||||||||||||||||||||||||||
Sbjct  1552687  GATGATCAGTCACACTGGAACTGAGACACGGTCCAGACTCCTACGGGAGGCAGCAGTGGG  1552746

Query  306      GAATATTGGACAATGGGCGAAAGCCTGATCCAGCCATGCCGCGTGTGTGAAGAAGGTCTT  365
                ||||||||||||||||||||||||||||||||||||||||||||||||||||||||||||
Sbjct  1552747  GAATATTGGACAATGGGCGAAAGCCTGATCCAGCCATGCCGCGTGTGTGAAGAAGGTCTT  1552806

Query  366      CGGATTGTAAAGCACTTTAAGTTGGGAGGAAGGGCAGTTACCTAATACGTGATTGTTTTG  425
                ||||||||||||||||||||||||||||||||||||||||||||||||||||||||||||
Sbjct  1552807  CGGATTGTAAAGCACTTTAAGTTGGGAGGAAGGGCAGTTACCTAATACGTGATTGTTTTG  1552866

Query  426      ACGTTACCGACAGAATAAGCACCGGCTAACTCTGTGCCAGCAGCCGCGGTAATACAGAGG  485
                ||||||||||||||||||||||||||||||||||||||||||||||||||||||||||||
Sbjct  1552867  ACGTTACCGACAGAATAAGCACCGGCTAACTCTGTGCCAGCAGCCGCGGTAATACAGAGG  1552926

Query  486      GTGCAAGCGTTAATCGGAATTACTGGGCGTAAAGCGCGCGTAGGTGGTTTGTTAAGTTGG  545
                ||||||||||||||||||||||||||||||||||||||||||||||||||||||||||||
Sbjct  1552927  GTGCAAGCGTTAATCGGAATTACTGGGCGTAAAGCGCGCGTAGGTGGTTTGTTAAGTTGG  1552986

Query  546      ATGTGAAATCCCCGGGCTCAACCTGGGAACTGCATTCAAAACTGACTGACTAGAGTATGG  605
                ||||||||||||||||||||||||||||||||||||||||||||||||||||||||||||
Sbjct  1552987  ATGTGAAATCCCCGGGCTCAACCTGGGAACTGCATTCAAAACTGACTGACTAGAGTATGG  1553046

Query  606      TAGAGGGTGGTGGAATTTCCTGTGTAGCGGTGAAATGCGTAGATATAGGAAGGAACACCA  665
                ||||||||||||||||||||||||||||||||||||||||||||||||||||||||||||
Sbjct  1553047  TAGAGGGTGGTGGAATTTCCTGTGTAGCGGTGAAATGCGTAGATATAGGAAGGAACACCA  1553106

Query  666      GTGGCGAAGGCGACCACCTGGACTAATACTGACACTGAGGTGCGAAAGCGTGGGGAGCAA  725
                ||||||||||||||||||||||||||||||||||||||||||||||||||||||||||||
Sbjct  1553107  GTGGCGAAGGCGACCACCTGGACTAATACTGACACTGAGGTGCGAAAGCGTGGGGAGCAA  1553166

Query  726      ACAGGATTAGATACCCTGGTAGTCCACGCCGTAAACGATGTCAACTAGCCGTTGGAAGCC  785
                ||||||||||||||||||||||||||||||||||||||||||||||||||||||||||||
Sbjct  1553167  ACAGGATTAGATACCCTGGTAGTCCACGCCGTAAACGATGTCAACTAGCCGTTGGAAGCC  1553226

Query  786      TTGAGCTTTTAGTGGCGCAGCTAACGCATTAAGTTGACCGCCTGGGGAGTACGGCCGCAA  845
                ||||||||||||||||||||||||||||||||||||||||||||||||||||||||||||
Sbjct  1553227  TTGAGCTTTTAGTGGCGCAGCTAACGCATTAAGTTGACCGCCTGGGGAGTACGGCCGCAA  1553286

Query  846      GGTTAAAACTCAAATGAATTGACGGGGGCCCGCACAAGCGNTGGNGCATGTGGTTTAATT  905
                |||||||||||||||||||||||||||||||||||||||| ||| |||||||||||||||
Sbjct  1553287  GGTTAAAACTCAAATGAATTGACGGGGGCCCGCACAAGCGGTGGAGCATGTGGTTTAATT  1553346

Query  906      CGAAGCAACGCGAAGAACCTTACCAGGCCTTGACATCCAATGAACTTTCTAGAGATAGAT  965
                ||||||||||||||||||||||||||||||||||||||||||||||||||||||||||||
Sbjct  1553347  CGAAGCAACGCGAAGAACCTTACCAGGCCTTGACATCCAATGAACTTTCTAGAGATAGAT  1553406

Query  966      TGGTGCCTTCGGGAACATTG  985
                ||||||||||||||||||||
Sbjct  1553407  TGGTGCCTTCGGGAACATTG  1553426
```

Range 4: 3431042 to 3432021GenBankGraphics

Next Match
Previous Match
First Match

Alignment statistics for match #4

| Score | Expect | Identities | Gaps | Strand | Frame |
| --- | --- | --- | --- | --- | --- |
| 1799 bits(974) | 0.0() | 977/980(99%) | 0/980(0%) | Plus/Plus |  |

Features:

rRNA-16S ribosomal RNA . Bacterial SSU

```
Query  6        TGCNAGTCGAGCGGTAGAGAGAAGCTTGCTTCTCTTGAGAGCGGCGGACGGGTGAGTAAT  65
                ||| ||||||||||||||||||||||||||||||||||||||||||||||||||||||||
Sbjct  3431042  TGCAAGTCGAGCGGTAGAGAGAAGCTTGCTTCTCTTGAGAGCGGCGGACGGGTGAGTAAT  3431101

Query  66       GCCTAGGAATCTGCCTGGTAGTGGGGGATAACGTTCGGAAACGGACGCTAATACCGCATA  125
                ||||||||||||||||||||||||||||||||||||||||||||||||||||||||||||
Sbjct  3431102  GCCTAGGAATCTGCCTGGTAGTGGGGGATAACGTTCGGAAACGGACGCTAATACCGCATA  3431161

Query  126      CGTCCTACGGGAGAAAGCAGGGGACCTTCGGGCCTTGCGCTATCAGATGAGCCTAGGTCG  185
                ||||||||||||||||||||||||||||||||||||||||||||||||||||||||||||
Sbjct  3431162  CGTCCTACGGGAGAAAGCAGGGGACCTTCGGGCCTTGCGCTATCAGATGAGCCTAGGTCG  3431221

Query  186      GATTAGCTAGTTGGTGGGGTAATGGCTCACCAAGGCGACGATCCGTAACTGGTCTGAGAG  245
                ||||||||||||||||||||||||||||||||||||||||||||||||||||||||||||
Sbjct  3431222  GATTAGCTAGTTGGTGGGGTAATGGCTCACCAAGGCGACGATCCGTAACTGGTCTGAGAG  3431281

Query  246      GATGATCAGTCACACTGGAACTGAGACACGGTCCAGACTCCTACGGGAGGCAGCAGTGGG  305
                ||||||||||||||||||||||||||||||||||||||||||||||||||||||||||||
Sbjct  3431282  GATGATCAGTCACACTGGAACTGAGACACGGTCCAGACTCCTACGGGAGGCAGCAGTGGG  3431341

Query  306      GAATATTGGACAATGGGCGAAAGCCTGATCCAGCCATGCCGCGTGTGTGAAGAAGGTCTT  365
                ||||||||||||||||||||||||||||||||||||||||||||||||||||||||||||
Sbjct  3431342  GAATATTGGACAATGGGCGAAAGCCTGATCCAGCCATGCCGCGTGTGTGAAGAAGGTCTT  3431401

Query  366      CGGATTGTAAAGCACTTTAAGTTGGGAGGAAGGGCAGTTACCTAATACGTGATTGTTTTG  425
                ||||||||||||||||||||||||||||||||||||||||||||||||||||||||||||
Sbjct  3431402  CGGATTGTAAAGCACTTTAAGTTGGGAGGAAGGGCAGTTACCTAATACGTGATTGTTTTG  3431461

Query  426      ACGTTACCGACAGAATAAGCACCGGCTAACTCTGTGCCAGCAGCCGCGGTAATACAGAGG  485
                ||||||||||||||||||||||||||||||||||||||||||||||||||||||||||||
Sbjct  3431462  ACGTTACCGACAGAATAAGCACCGGCTAACTCTGTGCCAGCAGCCGCGGTAATACAGAGG  3431521

Query  486      GTGCAAGCGTTAATCGGAATTACTGGGCGTAAAGCGCGCGTAGGTGGTTTGTTAAGTTGG  545
                ||||||||||||||||||||||||||||||||||||||||||||||||||||||||||||
Sbjct  3431522  GTGCAAGCGTTAATCGGAATTACTGGGCGTAAAGCGCGCGTAGGTGGTTTGTTAAGTTGG  3431581

Query  546      ATGTGAAATCCCCGGGCTCAACCTGGGAACTGCATTCAAAACTGACTGACTAGAGTATGG  605
                ||||||||||||||||||||||||||||||||||||||||||||||||||||||||||||
Sbjct  3431582  ATGTGAAATCCCCGGGCTCAACCTGGGAACTGCATTCAAAACTGACTGACTAGAGTATGG  3431641

Query  606      TAGAGGGTGGTGGAATTTCCTGTGTAGCGGTGAAATGCGTAGATATAGGAAGGAACACCA  665
                ||||||||||||||||||||||||||||||||||||||||||||||||||||||||||||
Sbjct  3431642  TAGAGGGTGGTGGAATTTCCTGTGTAGCGGTGAAATGCGTAGATATAGGAAGGAACACCA  3431701

Query  666      GTGGCGAAGGCGACCACCTGGACTAATACTGACACTGAGGTGCGAAAGCGTGGGGAGCAA  725
                ||||||||||||||||||||||||||||||||||||||||||||||||||||||||||||
Sbjct  3431702  GTGGCGAAGGCGACCACCTGGACTAATACTGACACTGAGGTGCGAAAGCGTGGGGAGCAA  3431761

Query  726      ACAGGATTAGATACCCTGGTAGTCCACGCCGTAAACGATGTCAACTAGCCGTTGGAAGCC  785
                ||||||||||||||||||||||||||||||||||||||||||||||||||||||||||||
Sbjct  3431762  ACAGGATTAGATACCCTGGTAGTCCACGCCGTAAACGATGTCAACTAGCCGTTGGAAGCC  3431821

Query  786      TTGAGCTTTTAGTGGCGCAGCTAACGCATTAAGTTGACCGCCTGGGGAGTACGGCCGCAA  845
                ||||||||||||||||||||||||||||||||||||||||||||||||||||||||||||
Sbjct  3431822  TTGAGCTTTTAGTGGCGCAGCTAACGCATTAAGTTGACCGCCTGGGGAGTACGGCCGCAA  3431881

Query  846      GGTTAAAACTCAAATGAATTGACGGGGGCCCGCACAAGCGNTGGNGCATGTGGTTTAATT  905
                |||||||||||||||||||||||||||||||||||||||| ||| |||||||||||||||
Sbjct  3431882  GGTTAAAACTCAAATGAATTGACGGGGGCCCGCACAAGCGGTGGAGCATGTGGTTTAATT  3431941

Query  906      CGAAGCAACGCGAAGAACCTTACCAGGCCTTGACATCCAATGAACTTTCTAGAGATAGAT  965
                ||||||||||||||||||||||||||||||||||||||||||||||||||||||||||||
Sbjct  3431942  CGAAGCAACGCGAAGAACCTTACCAGGCCTTGACATCCAATGAACTTTCTAGAGATAGAT  3432001

Query  966      TGGTGCCTTCGGGAACATTG  985
                ||||||||||||||||||||
Sbjct  3432002  TGGTGCCTTCGGGAACATTG  3432021
```

Range 5: 2158681 to 2159660GenBankGraphics

Next Match
Previous Match
First Match

Alignment statistics for match #5

| Score | Expect | Identities | Gaps | Strand | Frame |
| --- | --- | --- | --- | --- | --- |
| 1794 bits(971) | 0.0() | 976/980(99%) | 0/980(0%) | Plus/Plus |  |

Features:

rRNA-16S ribosomal RNA . Bacterial SSU

```
Query  6        TGCNAGTCGAGCGGTAGAGAGAAGCTTGCTTCTCTTGAGAGCGGCGGACGGGTGAGTAAT  65
                ||| ||||||||||||||||||||||||||||||||||||||||||||||||||||||||
Sbjct  2158681  TGCAAGTCGAGCGGTAGAGAGAAGCTTGCTTCTCTTGAGAGCGGCGGACGGGTGAGTAAT  2158740

Query  66       GCCTAGGAATCTGCCTGGTAGTGGGGGATAACGTTCGGAAACGGACGCTAATACCGCATA  125
                ||||||||||||||||||||||||||||||||||||||||||||||||||||||||||||
Sbjct  2158741  GCCTAGGAATCTGCCTGGTAGTGGGGGATAACGTTCGGAAACGGACGCTAATACCGCATA  2158800

Query  126      CGTCCTACGGGAGAAAGCAGGGGACCTTCGGGCCTTGCGCTATCAGATGAGCCTAGGTCG  185
                ||||||||||||||||||||||||||||||||||||||||||||||||||||||||||||
Sbjct  2158801  CGTCCTACGGGAGAAAGCAGGGGACCTTCGGGCCTTGCGCTATCAGATGAGCCTAGGTCG  2158860

Query  186      GATTAGCTAGTTGGTGGGGTAATGGCTCACCAAGGCGACGATCCGTAACTGGTCTGAGAG  245
                ||||||||||||||||||||||||||||||||||||||||||||||||||||||||||||
Sbjct  2158861  GATTAGCTAGTTGGTGGGGTAATGGCTCACCAAGGCGACGATCCGTAACTGGTCTGAGAG  2158920

Query  246      GATGATCAGTCACACTGGAACTGAGACACGGTCCAGACTCCTACGGGAGGCAGCAGTGGG  305
                ||||||||||||||||||||||||||||||||||||||||||||||||||||||||||||
Sbjct  2158921  GATGATCAGTCACACTGGAACTGAGACACGGTCCAGACTCCTACGGGAGGCAGCAGTGGG  2158980

Query  306      GAATATTGGACAATGGGCGAAAGCCTGATCCAGCCATGCCGCGTGTGTGAAGAAGGTCTT  365
                ||||||||||||||||||||||||||||||||||||||||||||||||||||||||||||
Sbjct  2158981  GAATATTGGACAATGGGCGAAAGCCTGATCCAGCCATGCCGCGTGTGTGAAGAAGGTCTT  2159040

Query  366      CGGATTGTAAAGCACTTTAAGTTGGGAGGAAGGGCAGTTACCTAATACGTGATTGTTTTG  425
                ||||||||||||||||||||||||||||||||||||||||| ||||||||||||||||||
Sbjct  2159041  CGGATTGTAAAGCACTTTAAGTTGGGAGGAAGGGCAGTTACTTAATACGTGATTGTTTTG  2159100

Query  426      ACGTTACCGACAGAATAAGCACCGGCTAACTCTGTGCCAGCAGCCGCGGTAATACAGAGG  485
                ||||||||||||||||||||||||||||||||||||||||||||||||||||||||||||
Sbjct  2159101  ACGTTACCGACAGAATAAGCACCGGCTAACTCTGTGCCAGCAGCCGCGGTAATACAGAGG  2159160

Query  486      GTGCAAGCGTTAATCGGAATTACTGGGCGTAAAGCGCGCGTAGGTGGTTTGTTAAGTTGG  545
                ||||||||||||||||||||||||||||||||||||||||||||||||||||||||||||
Sbjct  2159161  GTGCAAGCGTTAATCGGAATTACTGGGCGTAAAGCGCGCGTAGGTGGTTTGTTAAGTTGG  2159220

Query  546      ATGTGAAATCCCCGGGCTCAACCTGGGAACTGCATTCAAAACTGACTGACTAGAGTATGG  605
                ||||||||||||||||||||||||||||||||||||||||||||||||||||||||||||
Sbjct  2159221  ATGTGAAATCCCCGGGCTCAACCTGGGAACTGCATTCAAAACTGACTGACTAGAGTATGG  2159280

Query  606      TAGAGGGTGGTGGAATTTCCTGTGTAGCGGTGAAATGCGTAGATATAGGAAGGAACACCA  665
                ||||||||||||||||||||||||||||||||||||||||||||||||||||||||||||
Sbjct  2159281  TAGAGGGTGGTGGAATTTCCTGTGTAGCGGTGAAATGCGTAGATATAGGAAGGAACACCA  2159340

Query  666      GTGGCGAAGGCGACCACCTGGACTAATACTGACACTGAGGTGCGAAAGCGTGGGGAGCAA  725
                ||||||||||||||||||||||||||||||||||||||||||||||||||||||||||||
Sbjct  2159341  GTGGCGAAGGCGACCACCTGGACTAATACTGACACTGAGGTGCGAAAGCGTGGGGAGCAA  2159400

Query  726      ACAGGATTAGATACCCTGGTAGTCCACGCCGTAAACGATGTCAACTAGCCGTTGGAAGCC  785
                ||||||||||||||||||||||||||||||||||||||||||||||||||||||||||||
Sbjct  2159401  ACAGGATTAGATACCCTGGTAGTCCACGCCGTAAACGATGTCAACTAGCCGTTGGAAGCC  2159460

Query  786      TTGAGCTTTTAGTGGCGCAGCTAACGCATTAAGTTGACCGCCTGGGGAGTACGGCCGCAA  845
                ||||||||||||||||||||||||||||||||||||||||||||||||||||||||||||
Sbjct  2159461  TTGAGCTTTTAGTGGCGCAGCTAACGCATTAAGTTGACCGCCTGGGGAGTACGGCCGCAA  2159520

Query  846      GGTTAAAACTCAAATGAATTGACGGGGGCCCGCACAAGCGNTGGNGCATGTGGTTTAATT  905
                |||||||||||||||||||||||||||||||||||||||| ||| |||||||||||||||
Sbjct  2159521  GGTTAAAACTCAAATGAATTGACGGGGGCCCGCACAAGCGGTGGAGCATGTGGTTTAATT  2159580

Query  906      CGAAGCAACGCGAAGAACCTTACCAGGCCTTGACATCCAATGAACTTTCTAGAGATAGAT  965
                ||||||||||||||||||||||||||||||||||||||||||||||||||||||||||||
Sbjct  2159581  CGAAGCAACGCGAAGAACCTTACCAGGCCTTGACATCCAATGAACTTTCTAGAGATAGAT  2159640

Query  966      TGGTGCCTTCGGGAACATTG  985
                ||||||||||||||||||||
Sbjct  2159641  TGGTGCCTTCGGGAACATTG  2159660
```

Download

FASTA (complete sequence)

FASTA (aligned sequences)

GenBank (complete sequence)

Continue
Cancel

GenBankGraphics

Sort by:

E value
 Score
Percent identity
Query start position
Subject start position

Next
Previous
Descriptions

Pseudomonas poae strain BS2776 genome assembly, chromosome: I

Sequence ID: LT629706.1Length: 5950238Number of Matches: 5

Related Information

Range 1: 1521603 to 1522582GenBankGraphics

Next Match
Previous Match
First Match

Alignment statistics for match #1

| Score | Expect | Identities | Gaps | Strand | Frame |
| --- | --- | --- | --- | --- | --- |
| 1799 bits(974) | 0.0() | 977/980(99%) | 0/980(0%) | Plus/Minus |  |

Features:

rRNA-16S ribosomal RNA . Bacterial SSU

```
Query  6        TGCNAGTCGAGCGGTAGAGAGAAGCTTGCTTCTCTTGAGAGCGGCGGACGGGTGAGTAAT  65
                ||| ||||||||||||||||||||||||||||||||||||||||||||||||||||||||
Sbjct  1522582  TGCAAGTCGAGCGGTAGAGAGAAGCTTGCTTCTCTTGAGAGCGGCGGACGGGTGAGTAAT  1522523

Query  66       GCCTAGGAATCTGCCTGGTAGTGGGGGATAACGTTCGGAAACGGACGCTAATACCGCATA  125
                ||||||||||||||||||||||||||||||||||||||||||||||||||||||||||||
Sbjct  1522522  GCCTAGGAATCTGCCTGGTAGTGGGGGATAACGTTCGGAAACGGACGCTAATACCGCATA  1522463

Query  126      CGTCCTACGGGAGAAAGCAGGGGACCTTCGGGCCTTGCGCTATCAGATGAGCCTAGGTCG  185
                ||||||||||||||||||||||||||||||||||||||||||||||||||||||||||||
Sbjct  1522462  CGTCCTACGGGAGAAAGCAGGGGACCTTCGGGCCTTGCGCTATCAGATGAGCCTAGGTCG  1522403

Query  186      GATTAGCTAGTTGGTGGGGTAATGGCTCACCAAGGCGACGATCCGTAACTGGTCTGAGAG  245
                ||||||||||||||||||||||||||||||||||||||||||||||||||||||||||||
Sbjct  1522402  GATTAGCTAGTTGGTGGGGTAATGGCTCACCAAGGCGACGATCCGTAACTGGTCTGAGAG  1522343

Query  246      GATGATCAGTCACACTGGAACTGAGACACGGTCCAGACTCCTACGGGAGGCAGCAGTGGG  305
                ||||||||||||||||||||||||||||||||||||||||||||||||||||||||||||
Sbjct  1522342  GATGATCAGTCACACTGGAACTGAGACACGGTCCAGACTCCTACGGGAGGCAGCAGTGGG  1522283

Query  306      GAATATTGGACAATGGGCGAAAGCCTGATCCAGCCATGCCGCGTGTGTGAAGAAGGTCTT  365
                ||||||||||||||||||||||||||||||||||||||||||||||||||||||||||||
Sbjct  1522282  GAATATTGGACAATGGGCGAAAGCCTGATCCAGCCATGCCGCGTGTGTGAAGAAGGTCTT  1522223

Query  366      CGGATTGTAAAGCACTTTAAGTTGGGAGGAAGGGCAGTTACCTAATACGTGATTGTTTTG  425
                ||||||||||||||||||||||||||||||||||||||||||||||||||||||||||||
Sbjct  1522222  CGGATTGTAAAGCACTTTAAGTTGGGAGGAAGGGCAGTTACCTAATACGTGATTGTTTTG  1522163

Query  426      ACGTTACCGACAGAATAAGCACCGGCTAACTCTGTGCCAGCAGCCGCGGTAATACAGAGG  485
                ||||||||||||||||||||||||||||||||||||||||||||||||||||||||||||
Sbjct  1522162  ACGTTACCGACAGAATAAGCACCGGCTAACTCTGTGCCAGCAGCCGCGGTAATACAGAGG  1522103

Query  486      GTGCAAGCGTTAATCGGAATTACTGGGCGTAAAGCGCGCGTAGGTGGTTTGTTAAGTTGG  545
                ||||||||||||||||||||||||||||||||||||||||||||||||||||||||||||
Sbjct  1522102  GTGCAAGCGTTAATCGGAATTACTGGGCGTAAAGCGCGCGTAGGTGGTTTGTTAAGTTGG  1522043

Query  546      ATGTGAAATCCCCGGGCTCAACCTGGGAACTGCATTCAAAACTGACTGACTAGAGTATGG  605
                ||||||||||||||||||||||||||||||||||||||||||||||||||||||||||||
Sbjct  1522042  ATGTGAAATCCCCGGGCTCAACCTGGGAACTGCATTCAAAACTGACTGACTAGAGTATGG  1521983

Query  606      TAGAGGGTGGTGGAATTTCCTGTGTAGCGGTGAAATGCGTAGATATAGGAAGGAACACCA  665
                ||||||||||||||||||||||||||||||||||||||||||||||||||||||||||||
Sbjct  1521982  TAGAGGGTGGTGGAATTTCCTGTGTAGCGGTGAAATGCGTAGATATAGGAAGGAACACCA  1521923

Query  666      GTGGCGAAGGCGACCACCTGGACTAATACTGACACTGAGGTGCGAAAGCGTGGGGAGCAA  725
                ||||||||||||||||||||||||||||||||||||||||||||||||||||||||||||
Sbjct  1521922  GTGGCGAAGGCGACCACCTGGACTAATACTGACACTGAGGTGCGAAAGCGTGGGGAGCAA  1521863

Query  726      ACAGGATTAGATACCCTGGTAGTCCACGCCGTAAACGATGTCAACTAGCCGTTGGAAGCC  785
                ||||||||||||||||||||||||||||||||||||||||||||||||||||||||||||
Sbjct  1521862  ACAGGATTAGATACCCTGGTAGTCCACGCCGTAAACGATGTCAACTAGCCGTTGGAAGCC  1521803

Query  786      TTGAGCTTTTAGTGGCGCAGCTAACGCATTAAGTTGACCGCCTGGGGAGTACGGCCGCAA  845
                ||||||||||||||||||||||||||||||||||||||||||||||||||||||||||||
Sbjct  1521802  TTGAGCTTTTAGTGGCGCAGCTAACGCATTAAGTTGACCGCCTGGGGAGTACGGCCGCAA  1521743

Query  846      GGTTAAAACTCAAATGAATTGACGGGGGCCCGCACAAGCGNTGGNGCATGTGGTTTAATT  905
                |||||||||||||||||||||||||||||||||||||||| ||| |||||||||||||||
Sbjct  1521742  GGTTAAAACTCAAATGAATTGACGGGGGCCCGCACAAGCGGTGGAGCATGTGGTTTAATT  1521683

Query  906      CGAAGCAACGCGAAGAACCTTACCAGGCCTTGACATCCAATGAACTTTCTAGAGATAGAT  965
                ||||||||||||||||||||||||||||||||||||||||||||||||||||||||||||
Sbjct  1521682  CGAAGCAACGCGAAGAACCTTACCAGGCCTTGACATCCAATGAACTTTCTAGAGATAGAT  1521623

Query  966      TGGTGCCTTCGGGAACATTG  985
                ||||||||||||||||||||
Sbjct  1521622  TGGTGCCTTCGGGAACATTG  1521603
```

Range 2: 2816487 to 2817466GenBankGraphics

Next Match
Previous Match
First Match

Alignment statistics for match #2

| Score | Expect | Identities | Gaps | Strand | Frame |
| --- | --- | --- | --- | --- | --- |
| 1799 bits(974) | 0.0() | 977/980(99%) | 0/980(0%) | Plus/Minus |  |

Features:

rRNA-16S ribosomal RNA . Bacterial SSU

```
Query  6        TGCNAGTCGAGCGGTAGAGAGAAGCTTGCTTCTCTTGAGAGCGGCGGACGGGTGAGTAAT  65
                ||| ||||||||||||||||||||||||||||||||||||||||||||||||||||||||
Sbjct  2817466  TGCAAGTCGAGCGGTAGAGAGAAGCTTGCTTCTCTTGAGAGCGGCGGACGGGTGAGTAAT  2817407

Query  66       GCCTAGGAATCTGCCTGGTAGTGGGGGATAACGTTCGGAAACGGACGCTAATACCGCATA  125
                ||||||||||||||||||||||||||||||||||||||||||||||||||||||||||||
Sbjct  2817406  GCCTAGGAATCTGCCTGGTAGTGGGGGATAACGTTCGGAAACGGACGCTAATACCGCATA  2817347

Query  126      CGTCCTACGGGAGAAAGCAGGGGACCTTCGGGCCTTGCGCTATCAGATGAGCCTAGGTCG  185
                ||||||||||||||||||||||||||||||||||||||||||||||||||||||||||||
Sbjct  2817346  CGTCCTACGGGAGAAAGCAGGGGACCTTCGGGCCTTGCGCTATCAGATGAGCCTAGGTCG  2817287

Query  186      GATTAGCTAGTTGGTGGGGTAATGGCTCACCAAGGCGACGATCCGTAACTGGTCTGAGAG  245
                ||||||||||||||||||||||||||||||||||||||||||||||||||||||||||||
Sbjct  2817286  GATTAGCTAGTTGGTGGGGTAATGGCTCACCAAGGCGACGATCCGTAACTGGTCTGAGAG  2817227

Query  246      GATGATCAGTCACACTGGAACTGAGACACGGTCCAGACTCCTACGGGAGGCAGCAGTGGG  305
                ||||||||||||||||||||||||||||||||||||||||||||||||||||||||||||
Sbjct  2817226  GATGATCAGTCACACTGGAACTGAGACACGGTCCAGACTCCTACGGGAGGCAGCAGTGGG  2817167

Query  306      GAATATTGGACAATGGGCGAAAGCCTGATCCAGCCATGCCGCGTGTGTGAAGAAGGTCTT  365
                ||||||||||||||||||||||||||||||||||||||||||||||||||||||||||||
Sbjct  2817166  GAATATTGGACAATGGGCGAAAGCCTGATCCAGCCATGCCGCGTGTGTGAAGAAGGTCTT  2817107

Query  366      CGGATTGTAAAGCACTTTAAGTTGGGAGGAAGGGCAGTTACCTAATACGTGATTGTTTTG  425
                ||||||||||||||||||||||||||||||||||||||||||||||||||||||||||||
Sbjct  2817106  CGGATTGTAAAGCACTTTAAGTTGGGAGGAAGGGCAGTTACCTAATACGTGATTGTTTTG  2817047

Query  426      ACGTTACCGACAGAATAAGCACCGGCTAACTCTGTGCCAGCAGCCGCGGTAATACAGAGG  485
                ||||||||||||||||||||||||||||||||||||||||||||||||||||||||||||
Sbjct  2817046  ACGTTACCGACAGAATAAGCACCGGCTAACTCTGTGCCAGCAGCCGCGGTAATACAGAGG  2816987

Query  486      GTGCAAGCGTTAATCGGAATTACTGGGCGTAAAGCGCGCGTAGGTGGTTTGTTAAGTTGG  545
                ||||||||||||||||||||||||||||||||||||||||||||||||||||||||||||
Sbjct  2816986  GTGCAAGCGTTAATCGGAATTACTGGGCGTAAAGCGCGCGTAGGTGGTTTGTTAAGTTGG  2816927

Query  546      ATGTGAAATCCCCGGGCTCAACCTGGGAACTGCATTCAAAACTGACTGACTAGAGTATGG  605
                ||||||||||||||||||||||||||||||||||||||||||||||||||||||||||||
Sbjct  2816926  ATGTGAAATCCCCGGGCTCAACCTGGGAACTGCATTCAAAACTGACTGACTAGAGTATGG  2816867

Query  606      TAGAGGGTGGTGGAATTTCCTGTGTAGCGGTGAAATGCGTAGATATAGGAAGGAACACCA  665
                ||||||||||||||||||||||||||||||||||||||||||||||||||||||||||||
Sbjct  2816866  TAGAGGGTGGTGGAATTTCCTGTGTAGCGGTGAAATGCGTAGATATAGGAAGGAACACCA  2816807

Query  666      GTGGCGAAGGCGACCACCTGGACTAATACTGACACTGAGGTGCGAAAGCGTGGGGAGCAA  725
                ||||||||||||||||||||||||||||||||||||||||||||||||||||||||||||
Sbjct  2816806  GTGGCGAAGGCGACCACCTGGACTAATACTGACACTGAGGTGCGAAAGCGTGGGGAGCAA  2816747

Query  726      ACAGGATTAGATACCCTGGTAGTCCACGCCGTAAACGATGTCAACTAGCCGTTGGAAGCC  785
                ||||||||||||||||||||||||||||||||||||||||||||||||||||||||||||
Sbjct  2816746  ACAGGATTAGATACCCTGGTAGTCCACGCCGTAAACGATGTCAACTAGCCGTTGGAAGCC  2816687

Query  786      TTGAGCTTTTAGTGGCGCAGCTAACGCATTAAGTTGACCGCCTGGGGAGTACGGCCGCAA  845
                ||||||||||||||||||||||||||||||||||||||||||||||||||||||||||||
Sbjct  2816686  TTGAGCTTTTAGTGGCGCAGCTAACGCATTAAGTTGACCGCCTGGGGAGTACGGCCGCAA  2816627

Query  846      GGTTAAAACTCAAATGAATTGACGGGGGCCCGCACAAGCGNTGGNGCATGTGGTTTAATT  905
                |||||||||||||||||||||||||||||||||||||||| ||| |||||||||||||||
Sbjct  2816626  GGTTAAAACTCAAATGAATTGACGGGGGCCCGCACAAGCGGTGGAGCATGTGGTTTAATT  2816567

Query  906      CGAAGCAACGCGAAGAACCTTACCAGGCCTTGACATCCAATGAACTTTCTAGAGATAGAT  965
                ||||||||||||||||||||||||||||||||||||||||||||||||||||||||||||
Sbjct  2816566  CGAAGCAACGCGAAGAACCTTACCAGGCCTTGACATCCAATGAACTTTCTAGAGATAGAT  2816507

Query  966      TGGTGCCTTCGGGAACATTG  985
                ||||||||||||||||||||
Sbjct  2816506  TGGTGCCTTCGGGAACATTG  2816487
```

Range 3: 3457563 to 3458542GenBankGraphics

Next Match
Previous Match
First Match

Alignment statistics for match #3

| Score | Expect | Identities | Gaps | Strand | Frame |
| --- | --- | --- | --- | --- | --- |
| 1799 bits(974) | 0.0() | 977/980(99%) | 0/980(0%) | Plus/Minus |  |

Features:

rRNA-16S ribosomal RNA . Bacterial SSU

```
Query  6        TGCNAGTCGAGCGGTAGAGAGAAGCTTGCTTCTCTTGAGAGCGGCGGACGGGTGAGTAAT  65
                ||| ||||||||||||||||||||||||||||||||||||||||||||||||||||||||
Sbjct  3458542  TGCAAGTCGAGCGGTAGAGAGAAGCTTGCTTCTCTTGAGAGCGGCGGACGGGTGAGTAAT  3458483

Query  66       GCCTAGGAATCTGCCTGGTAGTGGGGGATAACGTTCGGAAACGGACGCTAATACCGCATA  125
                ||||||||||||||||||||||||||||||||||||||||||||||||||||||||||||
Sbjct  3458482  GCCTAGGAATCTGCCTGGTAGTGGGGGATAACGTTCGGAAACGGACGCTAATACCGCATA  3458423

Query  126      CGTCCTACGGGAGAAAGCAGGGGACCTTCGGGCCTTGCGCTATCAGATGAGCCTAGGTCG  185
                ||||||||||||||||||||||||||||||||||||||||||||||||||||||||||||
Sbjct  3458422  CGTCCTACGGGAGAAAGCAGGGGACCTTCGGGCCTTGCGCTATCAGATGAGCCTAGGTCG  3458363

Query  186      GATTAGCTAGTTGGTGGGGTAATGGCTCACCAAGGCGACGATCCGTAACTGGTCTGAGAG  245
                ||||||||||||||||||||||||||||||||||||||||||||||||||||||||||||
Sbjct  3458362  GATTAGCTAGTTGGTGGGGTAATGGCTCACCAAGGCGACGATCCGTAACTGGTCTGAGAG  3458303

Query  246      GATGATCAGTCACACTGGAACTGAGACACGGTCCAGACTCCTACGGGAGGCAGCAGTGGG  305
                ||||||||||||||||||||||||||||||||||||||||||||||||||||||||||||
Sbjct  3458302  GATGATCAGTCACACTGGAACTGAGACACGGTCCAGACTCCTACGGGAGGCAGCAGTGGG  3458243

Query  306      GAATATTGGACAATGGGCGAAAGCCTGATCCAGCCATGCCGCGTGTGTGAAGAAGGTCTT  365
                ||||||||||||||||||||||||||||||||||||||||||||||||||||||||||||
Sbjct  3458242  GAATATTGGACAATGGGCGAAAGCCTGATCCAGCCATGCCGCGTGTGTGAAGAAGGTCTT  3458183

Query  366      CGGATTGTAAAGCACTTTAAGTTGGGAGGAAGGGCAGTTACCTAATACGTGATTGTTTTG  425
                ||||||||||||||||||||||||||||||||||||||||||||||||||||||||||||
Sbjct  3458182  CGGATTGTAAAGCACTTTAAGTTGGGAGGAAGGGCAGTTACCTAATACGTGATTGTTTTG  3458123

Query  426      ACGTTACCGACAGAATAAGCACCGGCTAACTCTGTGCCAGCAGCCGCGGTAATACAGAGG  485
                ||||||||||||||||||||||||||||||||||||||||||||||||||||||||||||
Sbjct  3458122  ACGTTACCGACAGAATAAGCACCGGCTAACTCTGTGCCAGCAGCCGCGGTAATACAGAGG  3458063

Query  486      GTGCAAGCGTTAATCGGAATTACTGGGCGTAAAGCGCGCGTAGGTGGTTTGTTAAGTTGG  545
                ||||||||||||||||||||||||||||||||||||||||||||||||||||||||||||
Sbjct  3458062  GTGCAAGCGTTAATCGGAATTACTGGGCGTAAAGCGCGCGTAGGTGGTTTGTTAAGTTGG  3458003

Query  546      ATGTGAAATCCCCGGGCTCAACCTGGGAACTGCATTCAAAACTGACTGACTAGAGTATGG  605
                ||||||||||||||||||||||||||||||||||||||||||||||||||||||||||||
Sbjct  3458002  ATGTGAAATCCCCGGGCTCAACCTGGGAACTGCATTCAAAACTGACTGACTAGAGTATGG  3457943

Query  606      TAGAGGGTGGTGGAATTTCCTGTGTAGCGGTGAAATGCGTAGATATAGGAAGGAACACCA  665
                ||||||||||||||||||||||||||||||||||||||||||||||||||||||||||||
Sbjct  3457942  TAGAGGGTGGTGGAATTTCCTGTGTAGCGGTGAAATGCGTAGATATAGGAAGGAACACCA  3457883

Query  666      GTGGCGAAGGCGACCACCTGGACTAATACTGACACTGAGGTGCGAAAGCGTGGGGAGCAA  725
                ||||||||||||||||||||||||||||||||||||||||||||||||||||||||||||
Sbjct  3457882  GTGGCGAAGGCGACCACCTGGACTAATACTGACACTGAGGTGCGAAAGCGTGGGGAGCAA  3457823

Query  726      ACAGGATTAGATACCCTGGTAGTCCACGCCGTAAACGATGTCAACTAGCCGTTGGAAGCC  785
                ||||||||||||||||||||||||||||||||||||||||||||||||||||||||||||
Sbjct  3457822  ACAGGATTAGATACCCTGGTAGTCCACGCCGTAAACGATGTCAACTAGCCGTTGGAAGCC  3457763

Query  786      TTGAGCTTTTAGTGGCGCAGCTAACGCATTAAGTTGACCGCCTGGGGAGTACGGCCGCAA  845
                ||||||||||||||||||||||||||||||||||||||||||||||||||||||||||||
Sbjct  3457762  TTGAGCTTTTAGTGGCGCAGCTAACGCATTAAGTTGACCGCCTGGGGAGTACGGCCGCAA  3457703

Query  846      GGTTAAAACTCAAATGAATTGACGGGGGCCCGCACAAGCGNTGGNGCATGTGGTTTAATT  905
                |||||||||||||||||||||||||||||||||||||||| ||| |||||||||||||||
Sbjct  3457702  GGTTAAAACTCAAATGAATTGACGGGGGCCCGCACAAGCGGTGGAGCATGTGGTTTAATT  3457643

Query  906      CGAAGCAACGCGAAGAACCTTACCAGGCCTTGACATCCAATGAACTTTCTAGAGATAGAT  965
                ||||||||||||||||||||||||||||||||||||||||||||||||||||||||||||
Sbjct  3457642  CGAAGCAACGCGAAGAACCTTACCAGGCCTTGACATCCAATGAACTTTCTAGAGATAGAT  3457583

Query  966      TGGTGCCTTCGGGAACATTG  985
                ||||||||||||||||||||
Sbjct  3457582  TGGTGCCTTCGGGAACATTG  3457563
```

Range 4: 4255112 to 4256091GenBankGraphics

Next Match
Previous Match
First Match

Alignment statistics for match #4

| Score | Expect | Identities | Gaps | Strand | Frame |
| --- | --- | --- | --- | --- | --- |
| 1799 bits(974) | 0.0() | 977/980(99%) | 0/980(0%) | Plus/Plus |  |

Features:

rRNA-16S ribosomal RNA . Bacterial SSU

```
Query  6        TGCNAGTCGAGCGGTAGAGAGAAGCTTGCTTCTCTTGAGAGCGGCGGACGGGTGAGTAAT  65
                ||| ||||||||||||||||||||||||||||||||||||||||||||||||||||||||
Sbjct  4255112  TGCAAGTCGAGCGGTAGAGAGAAGCTTGCTTCTCTTGAGAGCGGCGGACGGGTGAGTAAT  4255171

Query  66       GCCTAGGAATCTGCCTGGTAGTGGGGGATAACGTTCGGAAACGGACGCTAATACCGCATA  125
                ||||||||||||||||||||||||||||||||||||||||||||||||||||||||||||
Sbjct  4255172  GCCTAGGAATCTGCCTGGTAGTGGGGGATAACGTTCGGAAACGGACGCTAATACCGCATA  4255231

Query  126      CGTCCTACGGGAGAAAGCAGGGGACCTTCGGGCCTTGCGCTATCAGATGAGCCTAGGTCG  185
                ||||||||||||||||||||||||||||||||||||||||||||||||||||||||||||
Sbjct  4255232  CGTCCTACGGGAGAAAGCAGGGGACCTTCGGGCCTTGCGCTATCAGATGAGCCTAGGTCG  4255291

Query  186      GATTAGCTAGTTGGTGGGGTAATGGCTCACCAAGGCGACGATCCGTAACTGGTCTGAGAG  245
                ||||||||||||||||||||||||||||||||||||||||||||||||||||||||||||
Sbjct  4255292  GATTAGCTAGTTGGTGGGGTAATGGCTCACCAAGGCGACGATCCGTAACTGGTCTGAGAG  4255351

Query  246      GATGATCAGTCACACTGGAACTGAGACACGGTCCAGACTCCTACGGGAGGCAGCAGTGGG  305
                ||||||||||||||||||||||||||||||||||||||||||||||||||||||||||||
Sbjct  4255352  GATGATCAGTCACACTGGAACTGAGACACGGTCCAGACTCCTACGGGAGGCAGCAGTGGG  4255411

Query  306      GAATATTGGACAATGGGCGAAAGCCTGATCCAGCCATGCCGCGTGTGTGAAGAAGGTCTT  365
                ||||||||||||||||||||||||||||||||||||||||||||||||||||||||||||
Sbjct  4255412  GAATATTGGACAATGGGCGAAAGCCTGATCCAGCCATGCCGCGTGTGTGAAGAAGGTCTT  4255471

Query  366      CGGATTGTAAAGCACTTTAAGTTGGGAGGAAGGGCAGTTACCTAATACGTGATTGTTTTG  425
                ||||||||||||||||||||||||||||||||||||||||||||||||||||||||||||
Sbjct  4255472  CGGATTGTAAAGCACTTTAAGTTGGGAGGAAGGGCAGTTACCTAATACGTGATTGTTTTG  4255531

Query  426      ACGTTACCGACAGAATAAGCACCGGCTAACTCTGTGCCAGCAGCCGCGGTAATACAGAGG  485
                ||||||||||||||||||||||||||||||||||||||||||||||||||||||||||||
Sbjct  4255532  ACGTTACCGACAGAATAAGCACCGGCTAACTCTGTGCCAGCAGCCGCGGTAATACAGAGG  4255591

Query  486      GTGCAAGCGTTAATCGGAATTACTGGGCGTAAAGCGCGCGTAGGTGGTTTGTTAAGTTGG  545
                ||||||||||||||||||||||||||||||||||||||||||||||||||||||||||||
Sbjct  4255592  GTGCAAGCGTTAATCGGAATTACTGGGCGTAAAGCGCGCGTAGGTGGTTTGTTAAGTTGG  4255651

Query  546      ATGTGAAATCCCCGGGCTCAACCTGGGAACTGCATTCAAAACTGACTGACTAGAGTATGG  605
                ||||||||||||||||||||||||||||||||||||||||||||||||||||||||||||
Sbjct  4255652  ATGTGAAATCCCCGGGCTCAACCTGGGAACTGCATTCAAAACTGACTGACTAGAGTATGG  4255711

Query  606      TAGAGGGTGGTGGAATTTCCTGTGTAGCGGTGAAATGCGTAGATATAGGAAGGAACACCA  665
                ||||||||||||||||||||||||||||||||||||||||||||||||||||||||||||
Sbjct  4255712  TAGAGGGTGGTGGAATTTCCTGTGTAGCGGTGAAATGCGTAGATATAGGAAGGAACACCA  4255771

Query  666      GTGGCGAAGGCGACCACCTGGACTAATACTGACACTGAGGTGCGAAAGCGTGGGGAGCAA  725
                ||||||||||||||||||||||||||||||||||||||||||||||||||||||||||||
Sbjct  4255772  GTGGCGAAGGCGACCACCTGGACTAATACTGACACTGAGGTGCGAAAGCGTGGGGAGCAA  4255831

Query  726      ACAGGATTAGATACCCTGGTAGTCCACGCCGTAAACGATGTCAACTAGCCGTTGGAAGCC  785
                ||||||||||||||||||||||||||||||||||||||||||||||||||||||||||||
Sbjct  4255832  ACAGGATTAGATACCCTGGTAGTCCACGCCGTAAACGATGTCAACTAGCCGTTGGAAGCC  4255891

Query  786      TTGAGCTTTTAGTGGCGCAGCTAACGCATTAAGTTGACCGCCTGGGGAGTACGGCCGCAA  845
                ||||||||||||||||||||||||||||||||||||||||||||||||||||||||||||
Sbjct  4255892  TTGAGCTTTTAGTGGCGCAGCTAACGCATTAAGTTGACCGCCTGGGGAGTACGGCCGCAA  4255951

Query  846      GGTTAAAACTCAAATGAATTGACGGGGGCCCGCACAAGCGNTGGNGCATGTGGTTTAATT  905
                |||||||||||||||||||||||||||||||||||||||| ||| |||||||||||||||
Sbjct  4255952  GGTTAAAACTCAAATGAATTGACGGGGGCCCGCACAAGCGGTGGAGCATGTGGTTTAATT  4256011

Query  906      CGAAGCAACGCGAAGAACCTTACCAGGCCTTGACATCCAATGAACTTTCTAGAGATAGAT  965
                ||||||||||||||||||||||||||||||||||||||||||||||||||||||||||||
Sbjct  4256012  CGAAGCAACGCGAAGAACCTTACCAGGCCTTGACATCCAATGAACTTTCTAGAGATAGAT  4256071

Query  966      TGGTGCCTTCGGGAACATTG  985
                ||||||||||||||||||||
Sbjct  4256072  TGGTGCCTTCGGGAACATTG  4256091
```

Range 5: 4574161 to 4575140GenBankGraphics

Next Match
Previous Match
First Match

Alignment statistics for match #5

| Score | Expect | Identities | Gaps | Strand | Frame |
| --- | --- | --- | --- | --- | --- |
| 1799 bits(974) | 0.0() | 977/980(99%) | 0/980(0%) | Plus/Plus |  |

Features:

rRNA-16S ribosomal RNA . Bacterial SSU

```
Query  6        TGCNAGTCGAGCGGTAGAGAGAAGCTTGCTTCTCTTGAGAGCGGCGGACGGGTGAGTAAT  65
                ||| ||||||||||||||||||||||||||||||||||||||||||||||||||||||||
Sbjct  4574161  TGCAAGTCGAGCGGTAGAGAGAAGCTTGCTTCTCTTGAGAGCGGCGGACGGGTGAGTAAT  4574220

Query  66       GCCTAGGAATCTGCCTGGTAGTGGGGGATAACGTTCGGAAACGGACGCTAATACCGCATA  125
                ||||||||||||||||||||||||||||||||||||||||||||||||||||||||||||
Sbjct  4574221  GCCTAGGAATCTGCCTGGTAGTGGGGGATAACGTTCGGAAACGGACGCTAATACCGCATA  4574280

Query  126      CGTCCTACGGGAGAAAGCAGGGGACCTTCGGGCCTTGCGCTATCAGATGAGCCTAGGTCG  185
                ||||||||||||||||||||||||||||||||||||||||||||||||||||||||||||
Sbjct  4574281  CGTCCTACGGGAGAAAGCAGGGGACCTTCGGGCCTTGCGCTATCAGATGAGCCTAGGTCG  4574340

Query  186      GATTAGCTAGTTGGTGGGGTAATGGCTCACCAAGGCGACGATCCGTAACTGGTCTGAGAG  245
                ||||||||||||||||||||||||||||||||||||||||||||||||||||||||||||
Sbjct  4574341  GATTAGCTAGTTGGTGGGGTAATGGCTCACCAAGGCGACGATCCGTAACTGGTCTGAGAG  4574400

Query  246      GATGATCAGTCACACTGGAACTGAGACACGGTCCAGACTCCTACGGGAGGCAGCAGTGGG  305
                ||||||||||||||||||||||||||||||||||||||||||||||||||||||||||||
Sbjct  4574401  GATGATCAGTCACACTGGAACTGAGACACGGTCCAGACTCCTACGGGAGGCAGCAGTGGG  4574460

Query  306      GAATATTGGACAATGGGCGAAAGCCTGATCCAGCCATGCCGCGTGTGTGAAGAAGGTCTT  365
                ||||||||||||||||||||||||||||||||||||||||||||||||||||||||||||
Sbjct  4574461  GAATATTGGACAATGGGCGAAAGCCTGATCCAGCCATGCCGCGTGTGTGAAGAAGGTCTT  4574520

Query  366      CGGATTGTAAAGCACTTTAAGTTGGGAGGAAGGGCAGTTACCTAATACGTGATTGTTTTG  425
                ||||||||||||||||||||||||||||||||||||||||||||||||||||||||||||
Sbjct  4574521  CGGATTGTAAAGCACTTTAAGTTGGGAGGAAGGGCAGTTACCTAATACGTGATTGTTTTG  4574580

Query  426      ACGTTACCGACAGAATAAGCACCGGCTAACTCTGTGCCAGCAGCCGCGGTAATACAGAGG  485
                ||||||||||||||||||||||||||||||||||||||||||||||||||||||||||||
Sbjct  4574581  ACGTTACCGACAGAATAAGCACCGGCTAACTCTGTGCCAGCAGCCGCGGTAATACAGAGG  4574640

Query  486      GTGCAAGCGTTAATCGGAATTACTGGGCGTAAAGCGCGCGTAGGTGGTTTGTTAAGTTGG  545
                ||||||||||||||||||||||||||||||||||||||||||||||||||||||||||||
Sbjct  4574641  GTGCAAGCGTTAATCGGAATTACTGGGCGTAAAGCGCGCGTAGGTGGTTTGTTAAGTTGG  4574700

Query  546      ATGTGAAATCCCCGGGCTCAACCTGGGAACTGCATTCAAAACTGACTGACTAGAGTATGG  605
                ||||||||||||||||||||||||||||||||||||||||||||||||||||||||||||
Sbjct  4574701  ATGTGAAATCCCCGGGCTCAACCTGGGAACTGCATTCAAAACTGACTGACTAGAGTATGG  4574760

Query  606      TAGAGGGTGGTGGAATTTCCTGTGTAGCGGTGAAATGCGTAGATATAGGAAGGAACACCA  665
                ||||||||||||||||||||||||||||||||||||||||||||||||||||||||||||
Sbjct  4574761  TAGAGGGTGGTGGAATTTCCTGTGTAGCGGTGAAATGCGTAGATATAGGAAGGAACACCA  4574820

Query  666      GTGGCGAAGGCGACCACCTGGACTAATACTGACACTGAGGTGCGAAAGCGTGGGGAGCAA  725
                ||||||||||||||||||||||||||||||||||||||||||||||||||||||||||||
Sbjct  4574821  GTGGCGAAGGCGACCACCTGGACTAATACTGACACTGAGGTGCGAAAGCGTGGGGAGCAA  4574880

Query  726      ACAGGATTAGATACCCTGGTAGTCCACGCCGTAAACGATGTCAACTAGCCGTTGGAAGCC  785
                ||||||||||||||||||||||||||||||||||||||||||||||||||||||||||||
Sbjct  4574881  ACAGGATTAGATACCCTGGTAGTCCACGCCGTAAACGATGTCAACTAGCCGTTGGAAGCC  4574940

Query  786      TTGAGCTTTTAGTGGCGCAGCTAACGCATTAAGTTGACCGCCTGGGGAGTACGGCCGCAA  845
                ||||||||||||||||||||||||||||||||||||||||||||||||||||||||||||
Sbjct  4574941  TTGAGCTTTTAGTGGCGCAGCTAACGCATTAAGTTGACCGCCTGGGGAGTACGGCCGCAA  4575000

Query  846      GGTTAAAACTCAAATGAATTGACGGGGGCCCGCACAAGCGNTGGNGCATGTGGTTTAATT  905
                |||||||||||||||||||||||||||||||||||||||| ||| |||||||||||||||
Sbjct  4575001  GGTTAAAACTCAAATGAATTGACGGGGGCCCGCACAAGCGGTGGAGCATGTGGTTTAATT  4575060

Query  906      CGAAGCAACGCGAAGAACCTTACCAGGCCTTGACATCCAATGAACTTTCTAGAGATAGAT  965
                ||||||||||||||||||||||||||||||||||||||||||||||||||||||||||||
Sbjct  4575061  CGAAGCAACGCGAAGAACCTTACCAGGCCTTGACATCCAATGAACTTTCTAGAGATAGAT  4575120

Query  966      TGGTGCCTTCGGGAACATTG  985
                ||||||||||||||||||||
Sbjct  4575121  TGGTGCCTTCGGGAACATTG  4575140
```

Download

FASTA (complete sequence)

FASTA (aligned sequences)

GenBank (complete sequence)

Continue
Cancel

GenBankGraphics

Sort by:

E value
 Score
Percent identity
Query start position
Subject start position

Next
Previous
Descriptions

Pseudomonas poae RE\*1-1-14, complete genome

Sequence ID: CP004045.1Length: 5512241Number of Matches: 5

Related Information

Range 1: 1279340 to 1280319GenBankGraphics

Next Match
Previous Match
First Match

Alignment statistics for match #1

| Score | Expect | Identities | Gaps | Strand | Frame |
| --- | --- | --- | --- | --- | --- |
| 1799 bits(974) | 0.0() | 977/980(99%) | 0/980(0%) | Plus/Plus |  |

Features:

```
Query  6        TGCNAGTCGAGCGGTAGAGAGAAGCTTGCTTCTCTTGAGAGCGGCGGACGGGTGAGTAAT  65
                ||| ||||||||||||||||||||||||||||||||||||||||||||||||||||||||
Sbjct  1279340  TGCAAGTCGAGCGGTAGAGAGAAGCTTGCTTCTCTTGAGAGCGGCGGACGGGTGAGTAAT  1279399

Query  66       GCCTAGGAATCTGCCTGGTAGTGGGGGATAACGTTCGGAAACGGACGCTAATACCGCATA  125
                ||||||||||||||||||||||||||||||||||||||||||||||||||||||||||||
Sbjct  1279400  GCCTAGGAATCTGCCTGGTAGTGGGGGATAACGTTCGGAAACGGACGCTAATACCGCATA  1279459

Query  126      CGTCCTACGGGAGAAAGCAGGGGACCTTCGGGCCTTGCGCTATCAGATGAGCCTAGGTCG  185
                ||||||||||||||||||||||||||||||||||||||||||||||||||||||||||||
Sbjct  1279460  CGTCCTACGGGAGAAAGCAGGGGACCTTCGGGCCTTGCGCTATCAGATGAGCCTAGGTCG  1279519

Query  186      GATTAGCTAGTTGGTGGGGTAATGGCTCACCAAGGCGACGATCCGTAACTGGTCTGAGAG  245
                ||||||||||||||||||||||||||||||||||||||||||||||||||||||||||||
Sbjct  1279520  GATTAGCTAGTTGGTGGGGTAATGGCTCACCAAGGCGACGATCCGTAACTGGTCTGAGAG  1279579

Query  246      GATGATCAGTCACACTGGAACTGAGACACGGTCCAGACTCCTACGGGAGGCAGCAGTGGG  305
                ||||||||||||||||||||||||||||||||||||||||||||||||||||||||||||
Sbjct  1279580  GATGATCAGTCACACTGGAACTGAGACACGGTCCAGACTCCTACGGGAGGCAGCAGTGGG  1279639

Query  306      GAATATTGGACAATGGGCGAAAGCCTGATCCAGCCATGCCGCGTGTGTGAAGAAGGTCTT  365
                ||||||||||||||||||||||||||||||||||||||||||||||||||||||||||||
Sbjct  1279640  GAATATTGGACAATGGGCGAAAGCCTGATCCAGCCATGCCGCGTGTGTGAAGAAGGTCTT  1279699

Query  366      CGGATTGTAAAGCACTTTAAGTTGGGAGGAAGGGCAGTTACCTAATACGTGATTGTTTTG  425
                ||||||||||||||||||||||||||||||||||||||||||||||||||||||||||||
Sbjct  1279700  CGGATTGTAAAGCACTTTAAGTTGGGAGGAAGGGCAGTTACCTAATACGTGATTGTTTTG  1279759

Query  426      ACGTTACCGACAGAATAAGCACCGGCTAACTCTGTGCCAGCAGCCGCGGTAATACAGAGG  485
                ||||||||||||||||||||||||||||||||||||||||||||||||||||||||||||
Sbjct  1279760  ACGTTACCGACAGAATAAGCACCGGCTAACTCTGTGCCAGCAGCCGCGGTAATACAGAGG  1279819

Query  486      GTGCAAGCGTTAATCGGAATTACTGGGCGTAAAGCGCGCGTAGGTGGTTTGTTAAGTTGG  545
                ||||||||||||||||||||||||||||||||||||||||||||||||||||||||||||
Sbjct  1279820  GTGCAAGCGTTAATCGGAATTACTGGGCGTAAAGCGCGCGTAGGTGGTTTGTTAAGTTGG  1279879

Query  546      ATGTGAAATCCCCGGGCTCAACCTGGGAACTGCATTCAAAACTGACTGACTAGAGTATGG  605
                ||||||||||||||||||||||||||||||||||||||||||||||||||||||||||||
Sbjct  1279880  ATGTGAAATCCCCGGGCTCAACCTGGGAACTGCATTCAAAACTGACTGACTAGAGTATGG  1279939

Query  606      TAGAGGGTGGTGGAATTTCCTGTGTAGCGGTGAAATGCGTAGATATAGGAAGGAACACCA  665
                ||||||||||||||||||||||||||||||||||||||||||||||||||||||||||||
Sbjct  1279940  TAGAGGGTGGTGGAATTTCCTGTGTAGCGGTGAAATGCGTAGATATAGGAAGGAACACCA  1279999

Query  666      GTGGCGAAGGCGACCACCTGGACTAATACTGACACTGAGGTGCGAAAGCGTGGGGAGCAA  725
                ||||||||||||||||||||||||||||||||||||||||||||||||||||||||||||
Sbjct  1280000  GTGGCGAAGGCGACCACCTGGACTAATACTGACACTGAGGTGCGAAAGCGTGGGGAGCAA  1280059

Query  726      ACAGGATTAGATACCCTGGTAGTCCACGCCGTAAACGATGTCAACTAGCCGTTGGAAGCC  785
                ||||||||||||||||||||||||||||||||||||||||||||||||||||||||||||
Sbjct  1280060  ACAGGATTAGATACCCTGGTAGTCCACGCCGTAAACGATGTCAACTAGCCGTTGGAAGCC  1280119

Query  786      TTGAGCTTTTAGTGGCGCAGCTAACGCATTAAGTTGACCGCCTGGGGAGTACGGCCGCAA  845
                ||||||||||||||||||||||||||||||||||||||||||||||||||||||||||||
Sbjct  1280120  TTGAGCTTTTAGTGGCGCAGCTAACGCATTAAGTTGACCGCCTGGGGAGTACGGCCGCAA  1280179

Query  846      GGTTAAAACTCAAATGAATTGACGGGGGCCCGCACAAGCGNTGGNGCATGTGGTTTAATT  905
                |||||||||||||||||||||||||||||||||||||||| ||| |||||||||||||||
Sbjct  1280180  GGTTAAAACTCAAATGAATTGACGGGGGCCCGCACAAGCGGTGGAGCATGTGGTTTAATT  1280239

Query  906      CGAAGCAACGCGAAGAACCTTACCAGGCCTTGACATCCAATGAACTTTCTAGAGATAGAT  965
                ||||||||||||||||||||||||||||||||||||||||||||||||||||||||||||
Sbjct  1280240  CGAAGCAACGCGAAGAACCTTACCAGGCCTTGACATCCAATGAACTTTCTAGAGATAGAT  1280299

Query  966      TGGTGCCTTCGGGAACATTG  985
                ||||||||||||||||||||
Sbjct  1280300  TGGTGCCTTCGGGAACATTG  1280319
```

Range 2: 3808808 to 3809787GenBankGraphics

Next Match
Previous Match
First Match

Alignment statistics for match #2

| Score | Expect | Identities | Gaps | Strand | Frame |
| --- | --- | --- | --- | --- | --- |
| 1799 bits(974) | 0.0() | 977/980(99%) | 0/980(0%) | Plus/Minus |  |

Features:

```
Query  6        TGCNAGTCGAGCGGTAGAGAGAAGCTTGCTTCTCTTGAGAGCGGCGGACGGGTGAGTAAT  65
                ||| ||||||||||||||||||||||||||||||||||||||||||||||||||||||||
Sbjct  3809787  TGCAAGTCGAGCGGTAGAGAGAAGCTTGCTTCTCTTGAGAGCGGCGGACGGGTGAGTAAT  3809728

Query  66       GCCTAGGAATCTGCCTGGTAGTGGGGGATAACGTTCGGAAACGGACGCTAATACCGCATA  125
                ||||||||||||||||||||||||||||||||||||||||||||||||||||||||||||
Sbjct  3809727  GCCTAGGAATCTGCCTGGTAGTGGGGGATAACGTTCGGAAACGGACGCTAATACCGCATA  3809668

Query  126      CGTCCTACGGGAGAAAGCAGGGGACCTTCGGGCCTTGCGCTATCAGATGAGCCTAGGTCG  185
                ||||||||||||||||||||||||||||||||||||||||||||||||||||||||||||
Sbjct  3809667  CGTCCTACGGGAGAAAGCAGGGGACCTTCGGGCCTTGCGCTATCAGATGAGCCTAGGTCG  3809608

Query  186      GATTAGCTAGTTGGTGGGGTAATGGCTCACCAAGGCGACGATCCGTAACTGGTCTGAGAG  245
                ||||||||||||||||||||||||||||||||||||||||||||||||||||||||||||
Sbjct  3809607  GATTAGCTAGTTGGTGGGGTAATGGCTCACCAAGGCGACGATCCGTAACTGGTCTGAGAG  3809548

Query  246      GATGATCAGTCACACTGGAACTGAGACACGGTCCAGACTCCTACGGGAGGCAGCAGTGGG  305
                ||||||||||||||||||||||||||||||||||||||||||||||||||||||||||||
Sbjct  3809547  GATGATCAGTCACACTGGAACTGAGACACGGTCCAGACTCCTACGGGAGGCAGCAGTGGG  3809488

Query  306      GAATATTGGACAATGGGCGAAAGCCTGATCCAGCCATGCCGCGTGTGTGAAGAAGGTCTT  365
                ||||||||||||||||||||||||||||||||||||||||||||||||||||||||||||
Sbjct  3809487  GAATATTGGACAATGGGCGAAAGCCTGATCCAGCCATGCCGCGTGTGTGAAGAAGGTCTT  3809428

Query  366      CGGATTGTAAAGCACTTTAAGTTGGGAGGAAGGGCAGTTACCTAATACGTGATTGTTTTG  425
                ||||||||||||||||||||||||||||||||||||||||||||||||||||||||||||
Sbjct  3809427  CGGATTGTAAAGCACTTTAAGTTGGGAGGAAGGGCAGTTACCTAATACGTGATTGTTTTG  3809368

Query  426      ACGTTACCGACAGAATAAGCACCGGCTAACTCTGTGCCAGCAGCCGCGGTAATACAGAGG  485
                ||||||||||||||||||||||||||||||||||||||||||||||||||||||||||||
Sbjct  3809367  ACGTTACCGACAGAATAAGCACCGGCTAACTCTGTGCCAGCAGCCGCGGTAATACAGAGG  3809308

Query  486      GTGCAAGCGTTAATCGGAATTACTGGGCGTAAAGCGCGCGTAGGTGGTTTGTTAAGTTGG  545
                ||||||||||||||||||||||||||||||||||||||||||||||||||||||||||||
Sbjct  3809307  GTGCAAGCGTTAATCGGAATTACTGGGCGTAAAGCGCGCGTAGGTGGTTTGTTAAGTTGG  3809248

Query  546      ATGTGAAATCCCCGGGCTCAACCTGGGAACTGCATTCAAAACTGACTGACTAGAGTATGG  605
                ||||||||||||||||||||||||||||||||||||||||||||||||||||||||||||
Sbjct  3809247  ATGTGAAATCCCCGGGCTCAACCTGGGAACTGCATTCAAAACTGACTGACTAGAGTATGG  3809188

Query  606      TAGAGGGTGGTGGAATTTCCTGTGTAGCGGTGAAATGCGTAGATATAGGAAGGAACACCA  665
                ||||||||||||||||||||||||||||||||||||||||||||||||||||||||||||
Sbjct  3809187  TAGAGGGTGGTGGAATTTCCTGTGTAGCGGTGAAATGCGTAGATATAGGAAGGAACACCA  3809128

Query  666      GTGGCGAAGGCGACCACCTGGACTAATACTGACACTGAGGTGCGAAAGCGTGGGGAGCAA  725
                ||||||||||||||||||||||||||||||||||||||||||||||||||||||||||||
Sbjct  3809127  GTGGCGAAGGCGACCACCTGGACTAATACTGACACTGAGGTGCGAAAGCGTGGGGAGCAA  3809068

Query  726      ACAGGATTAGATACCCTGGTAGTCCACGCCGTAAACGATGTCAACTAGCCGTTGGAAGCC  785
                ||||||||||||||||||||||||||||||||||||||||||||||||||||||||||||
Sbjct  3809067  ACAGGATTAGATACCCTGGTAGTCCACGCCGTAAACGATGTCAACTAGCCGTTGGAAGCC  3809008

Query  786      TTGAGCTTTTAGTGGCGCAGCTAACGCATTAAGTTGACCGCCTGGGGAGTACGGCCGCAA  845
                ||||||||||||||||||||||||||||||||||||||||||||||||||||||||||||
Sbjct  3809007  TTGAGCTTTTAGTGGCGCAGCTAACGCATTAAGTTGACCGCCTGGGGAGTACGGCCGCAA  3808948

Query  846      GGTTAAAACTCAAATGAATTGACGGGGGCCCGCACAAGCGNTGGNGCATGTGGTTTAATT  905
                |||||||||||||||||||||||||||||||||||||||| ||| |||||||||||||||
Sbjct  3808947  GGTTAAAACTCAAATGAATTGACGGGGGCCCGCACAAGCGGTGGAGCATGTGGTTTAATT  3808888

Query  906      CGAAGCAACGCGAAGAACCTTACCAGGCCTTGACATCCAATGAACTTTCTAGAGATAGAT  965
                ||||||||||||||||||||||||||||||||||||||||||||||||||||||||||||
Sbjct  3808887  CGAAGCAACGCGAAGAACCTTACCAGGCCTTGACATCCAATGAACTTTCTAGAGATAGAT  3808828

Query  966      TGGTGCCTTCGGGAACATTG  985
                ||||||||||||||||||||
Sbjct  3808827  TGGTGCCTTCGGGAACATTG  3808808
```

Range 3: 4137216 to 4138195GenBankGraphics

Next Match
Previous Match
First Match

Alignment statistics for match #3

| Score | Expect | Identities | Gaps | Strand | Frame |
| --- | --- | --- | --- | --- | --- |
| 1799 bits(974) | 0.0() | 977/980(99%) | 0/980(0%) | Plus/Minus |  |

Features:

```
Query  6        TGCNAGTCGAGCGGTAGAGAGAAGCTTGCTTCTCTTGAGAGCGGCGGACGGGTGAGTAAT  65
                ||| ||||||||||||||||||||||||||||||||||||||||||||||||||||||||
Sbjct  4138195  TGCAAGTCGAGCGGTAGAGAGAAGCTTGCTTCTCTTGAGAGCGGCGGACGGGTGAGTAAT  4138136

Query  66       GCCTAGGAATCTGCCTGGTAGTGGGGGATAACGTTCGGAAACGGACGCTAATACCGCATA  125
                ||||||||||||||||||||||||||||||||||||||||||||||||||||||||||||
Sbjct  4138135  GCCTAGGAATCTGCCTGGTAGTGGGGGATAACGTTCGGAAACGGACGCTAATACCGCATA  4138076

Query  126      CGTCCTACGGGAGAAAGCAGGGGACCTTCGGGCCTTGCGCTATCAGATGAGCCTAGGTCG  185
                ||||||||||||||||||||||||||||||||||||||||||||||||||||||||||||
Sbjct  4138075  CGTCCTACGGGAGAAAGCAGGGGACCTTCGGGCCTTGCGCTATCAGATGAGCCTAGGTCG  4138016

Query  186      GATTAGCTAGTTGGTGGGGTAATGGCTCACCAAGGCGACGATCCGTAACTGGTCTGAGAG  245
                ||||||||||||||||||||||||||||||||||||||||||||||||||||||||||||
Sbjct  4138015  GATTAGCTAGTTGGTGGGGTAATGGCTCACCAAGGCGACGATCCGTAACTGGTCTGAGAG  4137956

Query  246      GATGATCAGTCACACTGGAACTGAGACACGGTCCAGACTCCTACGGGAGGCAGCAGTGGG  305
                ||||||||||||||||||||||||||||||||||||||||||||||||||||||||||||
Sbjct  4137955  GATGATCAGTCACACTGGAACTGAGACACGGTCCAGACTCCTACGGGAGGCAGCAGTGGG  4137896

Query  306      GAATATTGGACAATGGGCGAAAGCCTGATCCAGCCATGCCGCGTGTGTGAAGAAGGTCTT  365
                ||||||||||||||||||||||||||||||||||||||||||||||||||||||||||||
Sbjct  4137895  GAATATTGGACAATGGGCGAAAGCCTGATCCAGCCATGCCGCGTGTGTGAAGAAGGTCTT  4137836

Query  366      CGGATTGTAAAGCACTTTAAGTTGGGAGGAAGGGCAGTTACCTAATACGTGATTGTTTTG  425
                ||||||||||||||||||||||||||||||||||||||||||||||||||||||||||||
Sbjct  4137835  CGGATTGTAAAGCACTTTAAGTTGGGAGGAAGGGCAGTTACCTAATACGTGATTGTTTTG  4137776

Query  426      ACGTTACCGACAGAATAAGCACCGGCTAACTCTGTGCCAGCAGCCGCGGTAATACAGAGG  485
                ||||||||||||||||||||||||||||||||||||||||||||||||||||||||||||
Sbjct  4137775  ACGTTACCGACAGAATAAGCACCGGCTAACTCTGTGCCAGCAGCCGCGGTAATACAGAGG  4137716

Query  486      GTGCAAGCGTTAATCGGAATTACTGGGCGTAAAGCGCGCGTAGGTGGTTTGTTAAGTTGG  545
                ||||||||||||||||||||||||||||||||||||||||||||||||||||||||||||
Sbjct  4137715  GTGCAAGCGTTAATCGGAATTACTGGGCGTAAAGCGCGCGTAGGTGGTTTGTTAAGTTGG  4137656

Query  546      ATGTGAAATCCCCGGGCTCAACCTGGGAACTGCATTCAAAACTGACTGACTAGAGTATGG  605
                ||||||||||||||||||||||||||||||||||||||||||||||||||||||||||||
Sbjct  4137655  ATGTGAAATCCCCGGGCTCAACCTGGGAACTGCATTCAAAACTGACTGACTAGAGTATGG  4137596

Query  606      TAGAGGGTGGTGGAATTTCCTGTGTAGCGGTGAAATGCGTAGATATAGGAAGGAACACCA  665
                ||||||||||||||||||||||||||||||||||||||||||||||||||||||||||||
Sbjct  4137595  TAGAGGGTGGTGGAATTTCCTGTGTAGCGGTGAAATGCGTAGATATAGGAAGGAACACCA  4137536

Query  666      GTGGCGAAGGCGACCACCTGGACTAATACTGACACTGAGGTGCGAAAGCGTGGGGAGCAA  725
                ||||||||||||||||||||||||||||||||||||||||||||||||||||||||||||
Sbjct  4137535  GTGGCGAAGGCGACCACCTGGACTAATACTGACACTGAGGTGCGAAAGCGTGGGGAGCAA  4137476

Query  726      ACAGGATTAGATACCCTGGTAGTCCACGCCGTAAACGATGTCAACTAGCCGTTGGAAGCC  785
                ||||||||||||||||||||||||||||||||||||||||||||||||||||||||||||
Sbjct  4137475  ACAGGATTAGATACCCTGGTAGTCCACGCCGTAAACGATGTCAACTAGCCGTTGGAAGCC  4137416

Query  786      TTGAGCTTTTAGTGGCGCAGCTAACGCATTAAGTTGACCGCCTGGGGAGTACGGCCGCAA  845
                ||||||||||||||||||||||||||||||||||||||||||||||||||||||||||||
Sbjct  4137415  TTGAGCTTTTAGTGGCGCAGCTAACGCATTAAGTTGACCGCCTGGGGAGTACGGCCGCAA  4137356

Query  846      GGTTAAAACTCAAATGAATTGACGGGGGCCCGCACAAGCGNTGGNGCATGTGGTTTAATT  905
                |||||||||||||||||||||||||||||||||||||||| ||| |||||||||||||||
Sbjct  4137355  GGTTAAAACTCAAATGAATTGACGGGGGCCCGCACAAGCGGTGGAGCATGTGGTTTAATT  4137296

Query  906      CGAAGCAACGCGAAGAACCTTACCAGGCCTTGACATCCAATGAACTTTCTAGAGATAGAT  965
                ||||||||||||||||||||||||||||||||||||||||||||||||||||||||||||
Sbjct  4137295  CGAAGCAACGCGAAGAACCTTACCAGGCCTTGACATCCAATGAACTTTCTAGAGATAGAT  4137236

Query  966      TGGTGCCTTCGGGAACATTG  985
                ||||||||||||||||||||
Sbjct  4137235  TGGTGCCTTCGGGAACATTG  4137216
```

Range 4: 5506943 to 5507922GenBankGraphics

Next Match
Previous Match
First Match

Alignment statistics for match #4

| Score | Expect | Identities | Gaps | Strand | Frame |
| --- | --- | --- | --- | --- | --- |
| 1799 bits(974) | 0.0() | 977/980(99%) | 0/980(0%) | Plus/Plus |  |

Features:

```
Query  6        TGCNAGTCGAGCGGTAGAGAGAAGCTTGCTTCTCTTGAGAGCGGCGGACGGGTGAGTAAT  65
                ||| ||||||||||||||||||||||||||||||||||||||||||||||||||||||||
Sbjct  5506943  TGCAAGTCGAGCGGTAGAGAGAAGCTTGCTTCTCTTGAGAGCGGCGGACGGGTGAGTAAT  5507002

Query  66       GCCTAGGAATCTGCCTGGTAGTGGGGGATAACGTTCGGAAACGGACGCTAATACCGCATA  125
                ||||||||||||||||||||||||||||||||||||||||||||||||||||||||||||
Sbjct  5507003  GCCTAGGAATCTGCCTGGTAGTGGGGGATAACGTTCGGAAACGGACGCTAATACCGCATA  5507062

Query  126      CGTCCTACGGGAGAAAGCAGGGGACCTTCGGGCCTTGCGCTATCAGATGAGCCTAGGTCG  185
                ||||||||||||||||||||||||||||||||||||||||||||||||||||||||||||
Sbjct  5507063  CGTCCTACGGGAGAAAGCAGGGGACCTTCGGGCCTTGCGCTATCAGATGAGCCTAGGTCG  5507122

Query  186      GATTAGCTAGTTGGTGGGGTAATGGCTCACCAAGGCGACGATCCGTAACTGGTCTGAGAG  245
                ||||||||||||||||||||||||||||||||||||||||||||||||||||||||||||
Sbjct  5507123  GATTAGCTAGTTGGTGGGGTAATGGCTCACCAAGGCGACGATCCGTAACTGGTCTGAGAG  5507182

Query  246      GATGATCAGTCACACTGGAACTGAGACACGGTCCAGACTCCTACGGGAGGCAGCAGTGGG  305
                ||||||||||||||||||||||||||||||||||||||||||||||||||||||||||||
Sbjct  5507183  GATGATCAGTCACACTGGAACTGAGACACGGTCCAGACTCCTACGGGAGGCAGCAGTGGG  5507242

Query  306      GAATATTGGACAATGGGCGAAAGCCTGATCCAGCCATGCCGCGTGTGTGAAGAAGGTCTT  365
                ||||||||||||||||||||||||||||||||||||||||||||||||||||||||||||
Sbjct  5507243  GAATATTGGACAATGGGCGAAAGCCTGATCCAGCCATGCCGCGTGTGTGAAGAAGGTCTT  5507302

Query  366      CGGATTGTAAAGCACTTTAAGTTGGGAGGAAGGGCAGTTACCTAATACGTGATTGTTTTG  425
                ||||||||||||||||||||||||||||||||||||||||||||||||||||||||||||
Sbjct  5507303  CGGATTGTAAAGCACTTTAAGTTGGGAGGAAGGGCAGTTACCTAATACGTGATTGTTTTG  5507362

Query  426      ACGTTACCGACAGAATAAGCACCGGCTAACTCTGTGCCAGCAGCCGCGGTAATACAGAGG  485
                ||||||||||||||||||||||||||||||||||||||||||||||||||||||||||||
Sbjct  5507363  ACGTTACCGACAGAATAAGCACCGGCTAACTCTGTGCCAGCAGCCGCGGTAATACAGAGG  5507422

Query  486      GTGCAAGCGTTAATCGGAATTACTGGGCGTAAAGCGCGCGTAGGTGGTTTGTTAAGTTGG  545
                ||||||||||||||||||||||||||||||||||||||||||||||||||||||||||||
Sbjct  5507423  GTGCAAGCGTTAATCGGAATTACTGGGCGTAAAGCGCGCGTAGGTGGTTTGTTAAGTTGG  5507482

Query  546      ATGTGAAATCCCCGGGCTCAACCTGGGAACTGCATTCAAAACTGACTGACTAGAGTATGG  605
                ||||||||||||||||||||||||||||||||||||||||||||||||||||||||||||
Sbjct  5507483  ATGTGAAATCCCCGGGCTCAACCTGGGAACTGCATTCAAAACTGACTGACTAGAGTATGG  5507542

Query  606      TAGAGGGTGGTGGAATTTCCTGTGTAGCGGTGAAATGCGTAGATATAGGAAGGAACACCA  665
                ||||||||||||||||||||||||||||||||||||||||||||||||||||||||||||
Sbjct  5507543  TAGAGGGTGGTGGAATTTCCTGTGTAGCGGTGAAATGCGTAGATATAGGAAGGAACACCA  5507602

Query  666      GTGGCGAAGGCGACCACCTGGACTAATACTGACACTGAGGTGCGAAAGCGTGGGGAGCAA  725
                ||||||||||||||||||||||||||||||||||||||||||||||||||||||||||||
Sbjct  5507603  GTGGCGAAGGCGACCACCTGGACTAATACTGACACTGAGGTGCGAAAGCGTGGGGAGCAA  5507662

Query  726      ACAGGATTAGATACCCTGGTAGTCCACGCCGTAAACGATGTCAACTAGCCGTTGGAAGCC  785
                ||||||||||||||||||||||||||||||||||||||||||||||||||||||||||||
Sbjct  5507663  ACAGGATTAGATACCCTGGTAGTCCACGCCGTAAACGATGTCAACTAGCCGTTGGAAGCC  5507722

Query  786      TTGAGCTTTTAGTGGCGCAGCTAACGCATTAAGTTGACCGCCTGGGGAGTACGGCCGCAA  845
                ||||||||||||||||||||||||||||||||||||||||||||||||||||||||||||
Sbjct  5507723  TTGAGCTTTTAGTGGCGCAGCTAACGCATTAAGTTGACCGCCTGGGGAGTACGGCCGCAA  5507782

Query  846      GGTTAAAACTCAAATGAATTGACGGGGGCCCGCACAAGCGNTGGNGCATGTGGTTTAATT  905
                |||||||||||||||||||||||||||||||||||||||| ||| |||||||||||||||
Sbjct  5507783  GGTTAAAACTCAAATGAATTGACGGGGGCCCGCACAAGCGGTGGAGCATGTGGTTTAATT  5507842

Query  906      CGAAGCAACGCGAAGAACCTTACCAGGCCTTGACATCCAATGAACTTTCTAGAGATAGAT  965
                ||||||||||||||||||||||||||||||||||||||||||||||||||||||||||||
Sbjct  5507843  CGAAGCAACGCGAAGAACCTTACCAGGCCTTGACATCCAATGAACTTTCTAGAGATAGAT  5507902

Query  966      TGGTGCCTTCGGGAACATTG  985
                ||||||||||||||||||||
Sbjct  5507903  TGGTGCCTTCGGGAACATTG  5507922
```

Range 5: 4900345 to 4901324GenBankGraphics

Next Match
Previous Match
First Match

Alignment statistics for match #5

| Score | Expect | Identities | Gaps | Strand | Frame |
| --- | --- | --- | --- | --- | --- |
| 1794 bits(971) | 0.0() | 976/980(99%) | 0/980(0%) | Plus/Plus |  |

Features:

```
Query  6        TGCNAGTCGAGCGGTAGAGAGAAGCTTGCTTCTCTTGAGAGCGGCGGACGGGTGAGTAAT  65
                ||| |||||||||||||||||||||| |||||||||||||||||||||||||||||||||
Sbjct  4900345  TGCAAGTCGAGCGGTAGAGAGAAGCTCGCTTCTCTTGAGAGCGGCGGACGGGTGAGTAAT  4900404

Query  66       GCCTAGGAATCTGCCTGGTAGTGGGGGATAACGTTCGGAAACGGACGCTAATACCGCATA  125
                ||||||||||||||||||||||||||||||||||||||||||||||||||||||||||||
Sbjct  4900405  GCCTAGGAATCTGCCTGGTAGTGGGGGATAACGTTCGGAAACGGACGCTAATACCGCATA  4900464

Query  126      CGTCCTACGGGAGAAAGCAGGGGACCTTCGGGCCTTGCGCTATCAGATGAGCCTAGGTCG  185
                ||||||||||||||||||||||||||||||||||||||||||||||||||||||||||||
Sbjct  4900465  CGTCCTACGGGAGAAAGCAGGGGACCTTCGGGCCTTGCGCTATCAGATGAGCCTAGGTCG  4900524

Query  186      GATTAGCTAGTTGGTGGGGTAATGGCTCACCAAGGCGACGATCCGTAACTGGTCTGAGAG  245
                ||||||||||||||||||||||||||||||||||||||||||||||||||||||||||||
Sbjct  4900525  GATTAGCTAGTTGGTGGGGTAATGGCTCACCAAGGCGACGATCCGTAACTGGTCTGAGAG  4900584

Query  246      GATGATCAGTCACACTGGAACTGAGACACGGTCCAGACTCCTACGGGAGGCAGCAGTGGG  305
                ||||||||||||||||||||||||||||||||||||||||||||||||||||||||||||
Sbjct  4900585  GATGATCAGTCACACTGGAACTGAGACACGGTCCAGACTCCTACGGGAGGCAGCAGTGGG  4900644

Query  306      GAATATTGGACAATGGGCGAAAGCCTGATCCAGCCATGCCGCGTGTGTGAAGAAGGTCTT  365
                ||||||||||||||||||||||||||||||||||||||||||||||||||||||||||||
Sbjct  4900645  GAATATTGGACAATGGGCGAAAGCCTGATCCAGCCATGCCGCGTGTGTGAAGAAGGTCTT  4900704

Query  366      CGGATTGTAAAGCACTTTAAGTTGGGAGGAAGGGCAGTTACCTAATACGTGATTGTTTTG  425
                ||||||||||||||||||||||||||||||||||||||||||||||||||||||||||||
Sbjct  4900705  CGGATTGTAAAGCACTTTAAGTTGGGAGGAAGGGCAGTTACCTAATACGTGATTGTTTTG  4900764

Query  426      ACGTTACCGACAGAATAAGCACCGGCTAACTCTGTGCCAGCAGCCGCGGTAATACAGAGG  485
                ||||||||||||||||||||||||||||||||||||||||||||||||||||||||||||
Sbjct  4900765  ACGTTACCGACAGAATAAGCACCGGCTAACTCTGTGCCAGCAGCCGCGGTAATACAGAGG  4900824

Query  486      GTGCAAGCGTTAATCGGAATTACTGGGCGTAAAGCGCGCGTAGGTGGTTTGTTAAGTTGG  545
                ||||||||||||||||||||||||||||||||||||||||||||||||||||||||||||
Sbjct  4900825  GTGCAAGCGTTAATCGGAATTACTGGGCGTAAAGCGCGCGTAGGTGGTTTGTTAAGTTGG  4900884

Query  546      ATGTGAAATCCCCGGGCTCAACCTGGGAACTGCATTCAAAACTGACTGACTAGAGTATGG  605
                ||||||||||||||||||||||||||||||||||||||||||||||||||||||||||||
Sbjct  4900885  ATGTGAAATCCCCGGGCTCAACCTGGGAACTGCATTCAAAACTGACTGACTAGAGTATGG  4900944

Query  606      TAGAGGGTGGTGGAATTTCCTGTGTAGCGGTGAAATGCGTAGATATAGGAAGGAACACCA  665
                ||||||||||||||||||||||||||||||||||||||||||||||||||||||||||||
Sbjct  4900945  TAGAGGGTGGTGGAATTTCCTGTGTAGCGGTGAAATGCGTAGATATAGGAAGGAACACCA  4901004

Query  666      GTGGCGAAGGCGACCACCTGGACTAATACTGACACTGAGGTGCGAAAGCGTGGGGAGCAA  725
                ||||||||||||||||||||||||||||||||||||||||||||||||||||||||||||
Sbjct  4901005  GTGGCGAAGGCGACCACCTGGACTAATACTGACACTGAGGTGCGAAAGCGTGGGGAGCAA  4901064

Query  726      ACAGGATTAGATACCCTGGTAGTCCACGCCGTAAACGATGTCAACTAGCCGTTGGAAGCC  785
                ||||||||||||||||||||||||||||||||||||||||||||||||||||||||||||
Sbjct  4901065  ACAGGATTAGATACCCTGGTAGTCCACGCCGTAAACGATGTCAACTAGCCGTTGGAAGCC  4901124

Query  786      TTGAGCTTTTAGTGGCGCAGCTAACGCATTAAGTTGACCGCCTGGGGAGTACGGCCGCAA  845
                ||||||||||||||||||||||||||||||||||||||||||||||||||||||||||||
Sbjct  4901125  TTGAGCTTTTAGTGGCGCAGCTAACGCATTAAGTTGACCGCCTGGGGAGTACGGCCGCAA  4901184

Query  846      GGTTAAAACTCAAATGAATTGACGGGGGCCCGCACAAGCGNTGGNGCATGTGGTTTAATT  905
                |||||||||||||||||||||||||||||||||||||||| ||| |||||||||||||||
Sbjct  4901185  GGTTAAAACTCAAATGAATTGACGGGGGCCCGCACAAGCGGTGGAGCATGTGGTTTAATT  4901244

Query  906      CGAAGCAACGCGAAGAACCTTACCAGGCCTTGACATCCAATGAACTTTCTAGAGATAGAT  965
                ||||||||||||||||||||||||||||||||||||||||||||||||||||||||||||
Sbjct  4901245  CGAAGCAACGCGAAGAACCTTACCAGGCCTTGACATCCAATGAACTTTCTAGAGATAGAT  4901304

Query  966      TGGTGCCTTCGGGAACATTG  985
                ||||||||||||||||||||
Sbjct  4901305  TGGTGCCTTCGGGAACATTG  4901324
```

Download

FASTA (complete sequence)

FASTA (aligned sequences)

GenBank (complete sequence)

Continue
Cancel

GenBankGraphics

Next
Previous
Descriptions

uncultured bacterium partial 16S rRNA gene, clone K15.87 KSS

Sequence ID: HE576021.1Length: 1459Number of Matches: 1

Related Information

Range 1: 29 to 1008GenBankGraphics

Next Match
Previous Match
First Match

Alignment statistics for match #1

| Score | Expect | Identities | Gaps | Strand | Frame |
| --- | --- | --- | --- | --- | --- |
| 1799 bits(974) | 0.0() | 977/980(99%) | 0/980(0%) | Plus/Plus |  |

Features:

```
Query  6     TGCNAGTCGAGCGGTAGAGAGAAGCTTGCTTCTCTTGAGAGCGGCGGACGGGTGAGTAAT  65
             ||| ||||||||||||||||||||||||||||||||||||||||||||||||||||||||
Sbjct  29    TGCAAGTCGAGCGGTAGAGAGAAGCTTGCTTCTCTTGAGAGCGGCGGACGGGTGAGTAAT  88

Query  66    GCCTAGGAATCTGCCTGGTAGTGGGGGATAACGTTCGGAAACGGACGCTAATACCGCATA  125
             ||||||||||||||||||||||||||||||||||||||||||||||||||||||||||||
Sbjct  89    GCCTAGGAATCTGCCTGGTAGTGGGGGATAACGTTCGGAAACGGACGCTAATACCGCATA  148

Query  126   CGTCCTACGGGAGAAAGCAGGGGACCTTCGGGCCTTGCGCTATCAGATGAGCCTAGGTCG  185
             ||||||||||||||||||||||||||||||||||||||||||||||||||||||||||||
Sbjct  149   CGTCCTACGGGAGAAAGCAGGGGACCTTCGGGCCTTGCGCTATCAGATGAGCCTAGGTCG  208

Query  186   GATTAGCTAGTTGGTGGGGTAATGGCTCACCAAGGCGACGATCCGTAACTGGTCTGAGAG  245
             ||||||||||||||||||||||||||||||||||||||||||||||||||||||||||||
Sbjct  209   GATTAGCTAGTTGGTGGGGTAATGGCTCACCAAGGCGACGATCCGTAACTGGTCTGAGAG  268

Query  246   GATGATCAGTCACACTGGAACTGAGACACGGTCCAGACTCCTACGGGAGGCAGCAGTGGG  305
             ||||||||||||||||||||||||||||||||||||||||||||||||||||||||||||
Sbjct  269   GATGATCAGTCACACTGGAACTGAGACACGGTCCAGACTCCTACGGGAGGCAGCAGTGGG  328

Query  306   GAATATTGGACAATGGGCGAAAGCCTGATCCAGCCATGCCGCGTGTGTGAAGAAGGTCTT  365
             ||||||||||||||||||||||||||||||||||||||||||||||||||||||||||||
Sbjct  329   GAATATTGGACAATGGGCGAAAGCCTGATCCAGCCATGCCGCGTGTGTGAAGAAGGTCTT  388

Query  366   CGGATTGTAAAGCACTTTAAGTTGGGAGGAAGGGCAGTTACCTAATACGTGATTGTTTTG  425
             ||||||||||||||||||||||||||||||||||||||||||||||||||||||||||||
Sbjct  389   CGGATTGTAAAGCACTTTAAGTTGGGAGGAAGGGCAGTTACCTAATACGTGATTGTTTTG  448

Query  426   ACGTTACCGACAGAATAAGCACCGGCTAACTCTGTGCCAGCAGCCGCGGTAATACAGAGG  485
             ||||||||||||||||||||||||||||||||||||||||||||||||||||||||||||
Sbjct  449   ACGTTACCGACAGAATAAGCACCGGCTAACTCTGTGCCAGCAGCCGCGGTAATACAGAGG  508

Query  486   GTGCAAGCGTTAATCGGAATTACTGGGCGTAAAGCGCGCGTAGGTGGTTTGTTAAGTTGG  545
             ||||||||||||||||||||||||||||||||||||||||||||||||||||||||||||
Sbjct  509   GTGCAAGCGTTAATCGGAATTACTGGGCGTAAAGCGCGCGTAGGTGGTTTGTTAAGTTGG  568

Query  546   ATGTGAAATCCCCGGGCTCAACCTGGGAACTGCATTCAAAACTGACTGACTAGAGTATGG  605
             ||||||||||||||||||||||||||||||||||||||||||||||||||||||||||||
Sbjct  569   ATGTGAAATCCCCGGGCTCAACCTGGGAACTGCATTCAAAACTGACTGACTAGAGTATGG  628

Query  606   TAGAGGGTGGTGGAATTTCCTGTGTAGCGGTGAAATGCGTAGATATAGGAAGGAACACCA  665
             ||||||||||||||||||||||||||||||||||||||||||||||||||||||||||||
Sbjct  629   TAGAGGGTGGTGGAATTTCCTGTGTAGCGGTGAAATGCGTAGATATAGGAAGGAACACCA  688

Query  666   GTGGCGAAGGCGACCACCTGGACTAATACTGACACTGAGGTGCGAAAGCGTGGGGAGCAA  725
             ||||||||||||||||||||||||||||||||||||||||||||||||||||||||||||
Sbjct  689   GTGGCGAAGGCGACCACCTGGACTAATACTGACACTGAGGTGCGAAAGCGTGGGGAGCAA  748

Query  726   ACAGGATTAGATACCCTGGTAGTCCACGCCGTAAACGATGTCAACTAGCCGTTGGAAGCC  785
             ||||||||||||||||||||||||||||||||||||||||||||||||||||||||||||
Sbjct  749   ACAGGATTAGATACCCTGGTAGTCCACGCCGTAAACGATGTCAACTAGCCGTTGGAAGCC  808

Query  786   TTGAGCTTTTAGTGGCGCAGCTAACGCATTAAGTTGACCGCCTGGGGAGTACGGCCGCAA  845
             ||||||||||||||||||||||||||||||||||||||||||||||||||||||||||||
Sbjct  809   TTGAGCTTTTAGTGGCGCAGCTAACGCATTAAGTTGACCGCCTGGGGAGTACGGCCGCAA  868

Query  846   GGTTAAAACTCAAATGAATTGACGGGGGCCCGCACAAGCGNTGGNGCATGTGGTTTAATT  905
             |||||||||||||||||||||||||||||||||||||||| ||| |||||||||||||||
Sbjct  869   GGTTAAAACTCAAATGAATTGACGGGGGCCCGCACAAGCGGTGGAGCATGTGGTTTAATT  928

Query  906   CGAAGCAACGCGAAGAACCTTACCAGGCCTTGACATCCAATGAACTTTCTAGAGATAGAT  965
             ||||||||||||||||||||||||||||||||||||||||||||||||||||||||||||
Sbjct  929   CGAAGCAACGCGAAGAACCTTACCAGGCCTTGACATCCAATGAACTTTCTAGAGATAGAT  988

Query  966   TGGTGCCTTCGGGAACATTG  985
             ||||||||||||||||||||
Sbjct  989   TGGTGCCTTCGGGAACATTG  1008
```

Download

FASTA (complete sequence)

FASTA (aligned sequences)

GenBank (complete sequence)

Continue
Cancel

GenBankGraphics

Next
Previous
Descriptions

Pseudomonas sp. PX7\_S1 16S ribosomal RNA gene, partial sequence

Sequence ID: JF274937.1Length: 1516Number of Matches: 1

Related Information

Range 1: 50 to 1029GenBankGraphics

Next Match
Previous Match
First Match

Alignment statistics for match #1

| Score | Expect | Identities | Gaps | Strand | Frame |
| --- | --- | --- | --- | --- | --- |
| 1799 bits(974) | 0.0() | 977/980(99%) | 0/980(0%) | Plus/Plus |  |

Features:

```
Query  6     TGCNAGTCGAGCGGTAGAGAGAAGCTTGCTTCTCTTGAGAGCGGCGGACGGGTGAGTAAT  65
             ||| ||||||||||||||||||||||||||||||||||||||||||||||||||||||||
Sbjct  50    TGCAAGTCGAGCGGTAGAGAGAAGCTTGCTTCTCTTGAGAGCGGCGGACGGGTGAGTAAT  109

Query  66    GCCTAGGAATCTGCCTGGTAGTGGGGGATAACGTTCGGAAACGGACGCTAATACCGCATA  125
             ||||||||||||||||||||||||||||||||||||||||||||||||||||||||||||
Sbjct  110   GCCTAGGAATCTGCCTGGTAGTGGGGGATAACGTTCGGAAACGGACGCTAATACCGCATA  169

Query  126   CGTCCTACGGGAGAAAGCAGGGGACCTTCGGGCCTTGCGCTATCAGATGAGCCTAGGTCG  185
             ||||||||||||||||||||||||||||||||||||||||||||||||||||||||||||
Sbjct  170   CGTCCTACGGGAGAAAGCAGGGGACCTTCGGGCCTTGCGCTATCAGATGAGCCTAGGTCG  229

Query  186   GATTAGCTAGTTGGTGGGGTAATGGCTCACCAAGGCGACGATCCGTAACTGGTCTGAGAG  245
             ||||||||||||||||||||||||||||||||||||||||||||||||||||||||||||
Sbjct  230   GATTAGCTAGTTGGTGGGGTAATGGCTCACCAAGGCGACGATCCGTAACTGGTCTGAGAG  289

Query  246   GATGATCAGTCACACTGGAACTGAGACACGGTCCAGACTCCTACGGGAGGCAGCAGTGGG  305
             ||||||||||||||||||||||||||||||||||||||||||||||||||||||||||||
Sbjct  290   GATGATCAGTCACACTGGAACTGAGACACGGTCCAGACTCCTACGGGAGGCAGCAGTGGG  349

Query  306   GAATATTGGACAATGGGCGAAAGCCTGATCCAGCCATGCCGCGTGTGTGAAGAAGGTCTT  365
             ||||||||||||||||||||||||||||||||||||||||||||||||||||||||||||
Sbjct  350   GAATATTGGACAATGGGCGAAAGCCTGATCCAGCCATGCCGCGTGTGTGAAGAAGGTCTT  409

Query  366   CGGATTGTAAAGCACTTTAAGTTGGGAGGAAGGGCAGTTACCTAATACGTGATTGTTTTG  425
             ||||||||||||||||||||||||||||||||||||||||||||||||||||||||||||
Sbjct  410   CGGATTGTAAAGCACTTTAAGTTGGGAGGAAGGGCAGTTACCTAATACGTGATTGTTTTG  469

Query  426   ACGTTACCGACAGAATAAGCACCGGCTAACTCTGTGCCAGCAGCCGCGGTAATACAGAGG  485
             ||||||||||||||||||||||||||||||||||||||||||||||||||||||||||||
Sbjct  470   ACGTTACCGACAGAATAAGCACCGGCTAACTCTGTGCCAGCAGCCGCGGTAATACAGAGG  529

Query  486   GTGCAAGCGTTAATCGGAATTACTGGGCGTAAAGCGCGCGTAGGTGGTTTGTTAAGTTGG  545
             ||||||||||||||||||||||||||||||||||||||||||||||||||||||||||||
Sbjct  530   GTGCAAGCGTTAATCGGAATTACTGGGCGTAAAGCGCGCGTAGGTGGTTTGTTAAGTTGG  589

Query  546   ATGTGAAATCCCCGGGCTCAACCTGGGAACTGCATTCAAAACTGACTGACTAGAGTATGG  605
             ||||||||||||||||||||||||||||||||||||||||||||||||||||||||||||
Sbjct  590   ATGTGAAATCCCCGGGCTCAACCTGGGAACTGCATTCAAAACTGACTGACTAGAGTATGG  649

Query  606   TAGAGGGTGGTGGAATTTCCTGTGTAGCGGTGAAATGCGTAGATATAGGAAGGAACACCA  665
             ||||||||||||||||||||||||||||||||||||||||||||||||||||||||||||
Sbjct  650   TAGAGGGTGGTGGAATTTCCTGTGTAGCGGTGAAATGCGTAGATATAGGAAGGAACACCA  709

Query  666   GTGGCGAAGGCGACCACCTGGACTAATACTGACACTGAGGTGCGAAAGCGTGGGGAGCAA  725
             ||||||||||||||||||||||||||||||||||||||||||||||||||||||||||||
Sbjct  710   GTGGCGAAGGCGACCACCTGGACTAATACTGACACTGAGGTGCGAAAGCGTGGGGAGCAA  769

Query  726   ACAGGATTAGATACCCTGGTAGTCCACGCCGTAAACGATGTCAACTAGCCGTTGGAAGCC  785
             ||||||||||||||||||||||||||||||||||||||||||||||||||||||||||||
Sbjct  770   ACAGGATTAGATACCCTGGTAGTCCACGCCGTAAACGATGTCAACTAGCCGTTGGAAGCC  829

Query  786   TTGAGCTTTTAGTGGCGCAGCTAACGCATTAAGTTGACCGCCTGGGGAGTACGGCCGCAA  845
             ||||||||||||||||||||||||||||||||||||||||||||||||||||||||||||
Sbjct  830   TTGAGCTTTTAGTGGCGCAGCTAACGCATTAAGTTGACCGCCTGGGGAGTACGGCCGCAA  889

Query  846   GGTTAAAACTCAAATGAATTGACGGGGGCCCGCACAAGCGNTGGNGCATGTGGTTTAATT  905
             |||||||||||||||||||||||||||||||||||||||| ||| |||||||||||||||
Sbjct  890   GGTTAAAACTCAAATGAATTGACGGGGGCCCGCACAAGCGGTGGAGCATGTGGTTTAATT  949

Query  906   CGAAGCAACGCGAAGAACCTTACCAGGCCTTGACATCCAATGAACTTTCTAGAGATAGAT  965
             ||||||||||||||||||||||||||||||||||||||||||||||||||||||||||||
Sbjct  950   CGAAGCAACGCGAAGAACCTTACCAGGCCTTGACATCCAATGAACTTTCTAGAGATAGAT  1009

Query  966   TGGTGCCTTCGGGAACATTG  985
             ||||||||||||||||||||
Sbjct  1010  TGGTGCCTTCGGGAACATTG  1029
```


BLAST is a registered trademark of the National Library of Medicine

Support center
Mailing list


YouTube

- National Library Of Medicine
- National Institutes Of Health
- U.S. Department of Health & Human Services
- USA.gov

### NCBI


National Center for Biotechnology Information,
 U.S. National Library of Medicine

8600 Rockville Pike,
Bethesda
 MD,
20894
USA

Policies and Guidelines
|
Contact


PreferencesTurn off

External link. Please review our privacy policy.
