# Supplementary material for: Isothermal microcalorimetry for thermal viable count of microorganisms in pure cultures and stabilized formulations
Source: BMC Microbiol. 2019 Mar 21;19:65. doi: 10.1186/s12866-019-1432-8 (PMC6429831; doi:10.1186/s12866-019-1432-8)
Supplement: Supplementary file 3 — 16S rRNA gene sequences’ BLASTN hits in zipped HTML format. (ZIP 15810 kb) [file 12866_2019_1432_MOESM3_ESM.zip › Best blastn hits/NCBI Blast_59 pinkish positive F -- 16..1001 of sequence.html]

NCBI Blast:59 pinkish positive F -- 16..1001 of sequence


- NCBI Home
- Sign in to NCBI
- Skip to Main Content
- Skip to Navigation
- About NCBI Accesskeys

U.S. National Library of Medicine

NCBI
National Center for Biotechnology Information

- My NCBI
- Sign in to NCBI
- Register
- Sign Out

BLAST ^®^ » blastn suite » RID-A4W873J1015


- Home
- Recent Results
- Saved Strategies
- Help

BLAST Results


Edit and Resubmit
Save Search Strategies
[Sign in above to save your search strategy]

Formatting options 


Download


How to read this page
Blast report description
Questions/comments


|  |  |
| --- | --- |
| Formatting options | |
| Show | Alignment as  HTML Plain text   Old View Reset form to defaults [?]  These options control formatting of alignments in results pages. The default is HTML, but other formats (including plain text) are available. PSSM and PssmWithParameters are representations of Position Specific Scoring Matrices and are only available for PSI-BLAST. The Advanced view option allows the database descriptions to be sorted by various indices in a table. |
| Alignment View | Pairwise Pairwise with dots for identities Query-anchored with dots for identities Query-anchored with letters for identities Flat query-anchored with dots for identities Flat query-anchored with letters for identities [?]  Choose how to view alignments. The default "pairwise" view shows how each subject sequence aligns individually to the query sequence. The "query-anchored" view shows how all subject sequences align to the query sequence. For each view type, you can choose to show "identities" (matching residues) as letters or dots. more... |
| Display | Graphical Overview   Linkout   Sequence Retrieval  NCBI-gi   CDS feature [?]  - Graphical Overview: Graphical Overview: Show graph of similar sequence regions aligned to query.   more... - NCBI-gi: Show NCBI gi identifiers. - CDS feature: Show annotated coding region and translation.   more... |
| Masking | Character:   X for protein, n for nucleotide Lower Case  Color:  Black Grey Red [?]  - Masking Character: Display masked (filtered) sequence regions as lower-case or as specific letters (N for nucleotide, P for protein). - Masking Color: Display masked sequence regions in the given color. |
| Limit results | Descriptions:  10 50 100 Graphical overview:  0 10 50 100  Alignments:  0 10 50 100 Line length:  60 90 120 150 [?]  - Descriptions: Show short descriptions for up to the given number of sequences. - Alignments: Show alignments for up to the given number of sequences, in order of statistical significance. - Line lenghth: Number of letters to show on one line in an alignment. |
|  | Organism Type common name, binomial, taxid, or group name. Only 20 top taxa will be shown.     Exclude   [?]  Show only sequences from the given organism. |
|  | Entrez query:  [?]  Show only those sequences that match the given Entrez query. more... |
|  | Expect Min:  Expect Max:  [?]  Show only sequences with expect values in the given range. more... |
|  | Percent Identity Min:  Percent Identity Max:  [?]  Show only sequences with percent identity values in the given range. |
| Format for | PSI-BLAST with inclusion threshold:  [?]  - Format for PSI-BLAST: The Position-Specific Iterated BLAST (PSI-BLAST) program performs iterative searches with a protein query,   in which sequences found in one round of search are used to build a custom score model for the next round.   more... - Inclusion Threshold: This sets the statistical significance threshold for including a sequence in the model used   by PSI-BLAST to create the PSSM on the next iteration. |

|  |  |  |  |  |  |
| --- | --- | --- | --- | --- | --- |
| Download | | | | | |
| Alignment  Text XML ASN.1 JSON Seq-align Hit Table(text) Hit Table(csv) Multiple-file XML2 Single-file XML2 Multiple-file JSON Single-file JSON SAM | Search Strategies  ASN.1 | PSSM to restart search  PSSM | [?] |  |  |

The Download link provides BLAST output that may be used as input to another program.
This includes parseable formats such as the tabular report or XML as well as the Search Strategy files read by the BLAST+ applications.
More details on the parseable (XML, tabular, and ASN.1) reports can be found at
https://www.ncbi.nlm.nih.gov/books/NBK153387/  
  

The following formats are offered under the Alignment section:  
1). "Text". Non-HTML standard BLAST report.  
2). "XML". XML report based upon the DTD at https://www.ncbi.nlm.nih.gov/data\_specs/dtd/NCBI\_BlastOutput.dtd  
3). "ASN.1". Alignment written out in Abstract Syntax Notation 1.  
4). "JSON Seq-align". Alignment written out in JSON.  
4). "Hit Table(text)". The tabular report as text.  
5). "Hit Table(csv)". The tabular report ready for import into spread-sheet programs like Excel.  
6). "XML2". New XML format described at ftp://ftp.ncbi.nlm.nih.gov/blast/documents/NEWXML/xml2.pdf.  
7). "JSON". New JSON format described at ftp://ftp.ncbi.nlm.nih.gov/blast/documents/NEWXML/xml2.pdf.  
8). "SAM". Sequence Alignment Map format.

XML2 and JSON can be downloaded either as one file per query (multiple-file) or one file for all queries (single-file). These formats are listed as Multiple-file XML2 (and JSON) or Single-file XML (and JSON).

The following report is offered under the Search Strategy section:  
1). "ASN.1" Search Strategy. A record of the parameters, query, and database used in the search. This file can be used to start a stand-alone BLAST search, see
https://www.ncbi.nlm.nih.gov/books/NBK1763/#CmdLineAppsManual.I455\_BLAST\_search\_stra


# Job title: 59 pinkish positive F -- 16..1001 of sequence

Results for:

lcl|Query\_199379 59 pinkish positive F -- 16..1001 of sequence(986bp)
[?]

Your BLAST job specified more than one input sequence.
This box lets you choose which input sequence to show BLAST results for.

RID
:   A4W873J1015 (Expires on 03-10 18:33 pm)

Query ID
:   lcl|Query\_199379
:   lcl|Query\_199379

Description
:   59 pinkish positive F -- 16..1001 of sequence

Molecule type
:   nucleic acid

Query Length
:   986

Database Name
:   nr

Description
:   Nucleotide collection (nt) See details

Program
:   BLASTN 2.8.0+ Citation

  

Reference 

Zheng Zhang, Scott Schwartz, Lukas Wagner, and Webb Miller (2000), "A greedy algorithm for aligning DNA sequences", J Comput Biol 2000; 7(1-2):203-14.

Reference - database indexing

Aleksandr Morgulis, George Coulouris, Yan Raytselis, Thomas L. Madden, Richa Agarwala, Alejandro A. Schäffer (2008), "Database Indexing for Production MegaBLAST Searches", Bioinformatics 24:1757-1764.

Other reports:
Search Summary

[Taxonomy reports]
[Distance tree of results]
[MSA viewer]

Search Parameters

| Search parameter name | Search parameter value |
| --- | --- |
| Program | blastn |
| Word size | 28 |
| Expect value | 10 |
| Hitlist size | 100 |
| Match/Mismatch scores | 1,-2 |
| Gapcosts | 0,2.5 |
| Low Complexity Filter | Yes |
| Filter string | L;m; |
| Genetic Code | 1 |

Database

| Database parameter name | Database parameter value |
| --- | --- |
| Posted date | Mar 7, 2018 1:58 PM |
| Number of letters | 174,044,644,244 |
| Number of sequences | 46,882,714 |
| Entrez query | Includes:  Excludes:  None |

Karlin-Altschul statistics

| Params | Ungapped | Gapped |
| --- | --- | --- |
| Lambda | 1.33271 | 1.28 |
| K | 0.620991 | 0.46 |
| H | 1.12409 | 0.85 |

Results Statistics

| Results Statistics parameter name | Results Statistics parameter value |
| --- | --- |
| Length adjustment | 35 |
| Effective length of query | 951 |
| Effective length of database | 172403749254 |
| Effective search space | 163955965540554 |
| Effective search space used | 163955965540554 |


## Graphic Summary

### Distribution of the top 128 Blast Hits on 100 subject sequences [?]

The graphic is an overview of the database sequences aligned to the query sequence. These are represented horizontal bars colored coded by score and showing the extent
of the alignment on the query sequence. Separate aligned regions on the same database sequence are connected by a thin grey line.
Mousing over an alignment shows the database sequence title. Clicking an alignment displays a box with more details about the alignment and
link to the sequence alignment itself in the Alignments section of the report.

Mouse over to see the title, click to show alignments

Color key for alignment scores

<40

40-50

50-80

80-200

>=200

Query

1

150

300

450

600

750

900

Pseudomonas sp. strain 7.3 16S ribosomal RNA gene, part..

Score:1796 Evalue:0

Accession:KY542120.1

Alignment

Pseudomonas sp. MAR9909 16S ribosomal RNA gene, partial..

Score:1796 Evalue:0

Accession:KU882744.1

Alignment

Pseudomonas sp. cpRA293 16S ribosomal RNA gene, partial..

Score:1796 Evalue:0

Accession:KJ510220.1

Alignment

Pseudomonas thivervalensis strain PE32 16S ribosomal RN..

Score:1796 Evalue:0

Accession:KJ420530.1

Alignment

Uncultured Pseudomonas sp. clone QL0ABY37ZD02 16S ribos..

Score:1796 Evalue:0

Accession:JQ712549.1

Alignment

Pseudomonas thivervalensis 16S ribosomal RNA gene, part..

Score:1796 Evalue:0

Accession:JN628032.1

Alignment

Pseudomonas brassicacearum strain Zy-2-1 16S ribosomal ..

Score:1796 Evalue:0

Accession:GU201849.1

Alignment

Pseudomonas brassicacearum isolate MA250 16S ribosomal ..

Score:1796 Evalue:0

Accession:DQ886486.1

Alignment

Pseudomonas sp. strain C50T3 16S ribosomal RNA gene, pa..

Score:1790 Evalue:0

Accession:KX953866.1

Alignment

Pseudomonas brassicacearum strain LBUM300 16S ribosomal..

Score:1790 Evalue:0

Accession:MG461459.1

Alignment

Pseudomonas sp. strain A18 16S ribosomal RNA gene, part..

Score:1790 Evalue:0

Accession:KX859159.1

Alignment

Pseudomonas sp. strain A13 16S ribosomal RNA gene, part..

Score:1790 Evalue:0

Accession:KX859154.1

Alignment

Pseudomonas sp. strain A12 16S ribosomal RNA gene, part..

Score:1790 Evalue:0

Accession:KX859153.1

Alignment

Pseudomonas sp. strain A10 16S ribosomal RNA gene, part..

Score:1790 Evalue:0

Accession:KX859151.1

Alignment

Pseudomonas sp. strain A9 16S ribosomal RNA gene, parti..

Score:1790 Evalue:0

Accession:KX859150.1

Alignment

Pseudomonas sp. strain A8 16S ribosomal RNA gene, parti..

Score:1790 Evalue:0

Accession:KX859149.1

Alignment

Pseudomonas sp. strain A4 16S ribosomal RNA gene, parti..

Score:1790 Evalue:0

Accession:KX859145.1

Alignment

Pseudomonas sp. HA-09 partial 16S rRNA gene, isolate HA..

Score:1790 Evalue:0

Accession:LT844660.1

Alignment

Uncultured Pseudomonas sp. partial 16S rRNA gene, isola..

Score:1790 Evalue:0

Accession:LT718479.1

Alignment

Pseudomonas brassicacearum strain BS3663 genome assembl..

Score:1790 Evalue:0

Accession:LT629713.1

Alignment

Pseudomonas brassicacearum strain Delaware 16S ribosoma..

Score:1790 Evalue:0

Accession:KT695846.1

Alignment

Pseudomonas brassicacearum strain Wood1 16S ribosomal R..

Score:1790 Evalue:0

Accession:KT695843.1

Alignment

Pseudomonas brassicacearum strain 93F8 16S ribosomal RN..

Score:1790 Evalue:0

Accession:KT695841.1

Alignment

Pseudomonas brassicacearum strain L13-6-12, complete ge..

Score:1790 Evalue:0

Accession:CP014693.1

Alignment

Pseudomonas brassicacearum subsp. neoaurantiaca partial..

Score:1790 Evalue:0

Accession:LT547829.1

Alignment

Pseudomonas brassicacearum strain PG17 16S ribosomal RN..

Score:1790 Evalue:0

Accession:KU350605.1

Alignment

Pseudomonas brassicacearum strain PG16 16S ribosomal RN..

Score:1790 Evalue:0

Accession:KU350593.1

Alignment

Pseudomonas brassicacearum strain LBUM300, complete gen..

Score:1790 Evalue:0

Accession:CP012680.1

Alignment

Pseudomonas fluorescens strain FW300-N2C3, complete gen..

Score:1790 Evalue:0

Accession:CP012831.1

Alignment

Pseudomonas brassicacearum strain KAR22 16S ribosomal R..

Score:1790 Evalue:0

Accession:KR054984.1

Alignment

Pseudomonas brassicacearum strain IHB B 13650 16S ribos..

Score:1790 Evalue:0

Accession:KP762561.1

Alignment

Pseudomonas brassicacearum subsp. neoaurantiaca strain ..

Score:1790 Evalue:0

Accession:KP762555.1

Alignment

Pseudomonas fluorescens strain WCS365 16S ribosomal RNA..

Score:1790 Evalue:0

Accession:KP253039.1

Alignment

Pseudomonas brassicacearum subsp. brassicacearum gene f..

Score:1790 Evalue:0

Accession:LC015569.1

Alignment

Pseudomonas sp. DT 5-12 16S ribosomal RNA gene, partial..

Score:1790 Evalue:0

Accession:KM253057.1

Alignment

Pseudomonas sp. DR 2-03 16S ribosomal RNA gene, partial..

Score:1790 Evalue:0

Accession:KM253000.1

Alignment

Pseudomonas fluorescens strain JK15 16S ribosomal RNA g..

Score:1790 Evalue:0

Accession:KF148637.1

Alignment

Pseudomonas brassicacearum partial 16S rRNA gene, strai..

Score:1790 Evalue:0

Accession:HF952549.1

Alignment

Pseudomonas brassicacearum partial 16S rRNA gene, strai..

Score:1790 Evalue:0

Accession:HF952537.1

Alignment

Pseudomonas fluorescens strain IBFC2012-45 16S ribosoma..

Score:1790 Evalue:0

Accession:KC246049.1

Alignment

Pseudomonas sp. EA3(2012) 16S ribosomal RNA gene, parti..

Score:1790 Evalue:0

Accession:JX912367.1

Alignment

Pseudomonas brassicacearum strain J12 16S ribosomal RNA..

Score:1790 Evalue:0

Accession:JN605747.1

Alignment

Pseudomonas sp. LB184 partial 16S rRNA gene, isolate LB..

Score:1790 Evalue:0

Accession:FR675976.1

Alignment

Pseudomonas sp. LB183 partial 16S rRNA gene, isolate LB..

Score:1790 Evalue:0

Accession:FR675975.1

Alignment

Pseudomonas sp. SHB3 16S ribosomal RNA gene, partial se..

Score:1790 Evalue:0

Accession:HQ848635.1

Alignment

Uncultured Pseudomonas sp. clone 101 16S ribosomal RNA ..

Score:1790 Evalue:0

Accession:JF500978.1

Alignment

Uncultured Pseudomonas sp. clone 52 16S ribosomal RNA g..

Score:1790 Evalue:0

Accession:JF500932.1

Alignment

Pseudomonas brassicacearum subsp. brassicacearum NFM421..

Score:1790 Evalue:0

Accession:CP002585.1

Alignment

Pseudomonas sp. G68 partial 16S rRNA gene, strain G68

Score:1790 Evalue:0

Accession:FN547411.1

Alignment

Pseudomonas sp. P96.25 16S ribosomal RNA gene, partial ..

Score:1790 Evalue:0

Accession:DQ453838.1

Alignment

Pseudomonas sp. K93.3 16S ribosomal RNA gene, partial s..

Score:1790 Evalue:0

Accession:DQ453837.1

Alignment

Pseudomonas sp. C\*1A1 16S ribosomal RNA gene, partial s..

Score:1790 Evalue:0

Accession:DQ453836.1

Alignment

Pseudomonas sp. TM1A3 16S ribosomal RNA gene, partial s..

Score:1790 Evalue:0

Accession:DQ453835.1

Alignment

Pseudomonas sp. K94.37 16S ribosomal RNA gene, complete..

Score:1790 Evalue:0

Accession:DQ453820.1

Alignment

Pseudomonas sp. K93.2 16S ribosomal RNA gene, complete ..

Score:1790 Evalue:0

Accession:DQ453818.1

Alignment

Pseudomonas aurantiaca VKM B-1524 16S ribosomal RNA gen..

Score:1790 Evalue:0

Accession:AY271792.1

Alignment

## Descriptions

, Reading indexes 1-5, displaying indexes 1-5


Load next setPrevious Match

Sequences producing significant alignments:

Show all columns  of the table presenting sequences producing significant alignments 

Select:AllNone
Selected:0

Alignments
Download

FASTA (complete sequence)

FASTA (aligned sequences)

GenBank (complete sequence)

Hit Table (text)

Hit Table (CSV)

Text

XML

ASN.1

Continue
Cancel

GenBank 
Graphics
Distance tree of results
Multiple alignment
Show/hide columns of the table presenting sequences producing significant alignments 

Available columns

Description  
Max Score  
Total Score  
Coverage  
E-value  
IdentN  
Accession  
Restore Defaults
Ok
Cancel

Sequences producing significant alignments:

| Select for downloading or viewing reports | Description | Max score | Total score | Query cover | E value | Ident | Accession |
| --- | --- | --- | --- | --- | --- | --- | --- |
| 1Select seq KY542120.1 | Pseudomonas sp. strain 7.3 16S ribosomal RNA gene, partial sequence | 1796 | 1796 | 99% | 0.0 | 99% | KY542120.1 |
| 2Select seq KU882744.1 | Pseudomonas sp. MAR9909 16S ribosomal RNA gene, partial sequence | 1796 | 1796 | 99% | 0.0 | 99% | KU882744.1 |
| 3Select seq KJ510220.1 | Pseudomonas sp. cpRA293 16S ribosomal RNA gene, partial sequence | 1796 | 1796 | 99% | 0.0 | 99% | KJ510220.1 |
| 4Select seq KJ420530.1 | Pseudomonas thivervalensis strain PE32 16S ribosomal RNA gene, partial sequence | 1796 | 1796 | 99% | 0.0 | 99% | KJ420530.1 |
| 5Select seq JQ712549.1 | Uncultured Pseudomonas sp. clone QL0ABY37ZD02 16S ribosomal RNA gene, partial sequence | 1796 | 1796 | 99% | 0.0 | 99% | JQ712549.1 |
| 6Select seq JN628032.1 | Pseudomonas thivervalensis 16S ribosomal RNA gene, partial sequence | 1796 | 1796 | 99% | 0.0 | 99% | JN628032.1 |
| 7Select seq GU201849.1 | Pseudomonas brassicacearum strain Zy-2-1 16S ribosomal RNA gene, partial sequence | 1796 | 1796 | 99% | 0.0 | 99% | GU201849.1 |
| 8Select seq DQ886486.1 | Pseudomonas brassicacearum isolate MA250 16S ribosomal RNA gene, partial sequence | 1796 | 1796 | 99% | 0.0 | 99% | DQ886486.1 |
| 9Select seq KX953866.1 | Pseudomonas sp. strain C50T3 16S ribosomal RNA gene, partial sequence | 1790 | 1790 | 99% | 0.0 | 99% | KX953866.1 |
| 10Select seq MG461459.1 | Pseudomonas brassicacearum strain LBUM300 16S ribosomal RNA gene, partial sequence | 1790 | 1790 | 99% | 0.0 | 99% | MG461459.1 |
| 11Select seq KX859159.1 | Pseudomonas sp. strain A18 16S ribosomal RNA gene, partial sequence | 1790 | 1790 | 99% | 0.0 | 99% | KX859159.1 |
| 12Select seq KX859154.1 | Pseudomonas sp. strain A13 16S ribosomal RNA gene, partial sequence | 1790 | 1790 | 99% | 0.0 | 99% | KX859154.1 |
| 13Select seq KX859153.1 | Pseudomonas sp. strain A12 16S ribosomal RNA gene, partial sequence | 1790 | 1790 | 99% | 0.0 | 99% | KX859153.1 |
| 14Select seq KX859151.1 | Pseudomonas sp. strain A10 16S ribosomal RNA gene, partial sequence | 1790 | 1790 | 99% | 0.0 | 99% | KX859151.1 |
| 15Select seq KX859150.1 | Pseudomonas sp. strain A9 16S ribosomal RNA gene, partial sequence | 1790 | 1790 | 99% | 0.0 | 99% | KX859150.1 |
| 16Select seq KX859149.1 | Pseudomonas sp. strain A8 16S ribosomal RNA gene, partial sequence | 1790 | 1790 | 99% | 0.0 | 99% | KX859149.1 |
| 17Select seq KX859145.1 | Pseudomonas sp. strain A4 16S ribosomal RNA gene, partial sequence | 1790 | 1790 | 99% | 0.0 | 99% | KX859145.1 |
| 18Select seq LT844660.1 | Pseudomonas sp. HA-09 partial 16S rRNA gene, isolate HA-09 | 1790 | 1790 | 99% | 0.0 | 99% | LT844660.1 |
| 19Select seq LT718479.1 | Uncultured Pseudomonas sp. partial 16S rRNA gene, isolate VF5 | 1790 | 1790 | 99% | 0.0 | 99% | LT718479.1 |
| 20Select seq LT629713.1 | Pseudomonas brassicacearum strain BS3663 genome assembly, chromosome: I | 1790 | 8947 | 99% | 0.0 | 99% | LT629713.1 |
| 21Select seq KT695846.1 | Pseudomonas brassicacearum strain Delaware 16S ribosomal RNA gene, partial sequence | 1790 | 1790 | 99% | 0.0 | 99% | KT695846.1 |
| 22Select seq KT695843.1 | Pseudomonas brassicacearum strain Wood1 16S ribosomal RNA gene, partial sequence | 1790 | 1790 | 99% | 0.0 | 99% | KT695843.1 |
| 23Select seq KT695841.1 | Pseudomonas brassicacearum strain 93F8 16S ribosomal RNA gene, partial sequence | 1790 | 1790 | 99% | 0.0 | 99% | KT695841.1 |
| 24Select seq CP014693.1 | Pseudomonas brassicacearum strain L13-6-12, complete genome | 1790 | 8952 | 99% | 0.0 | 99% | CP014693.1 |
| 25Select seq LT547829.1 | Pseudomonas brassicacearum subsp. neoaurantiaca partial 16S rRNA gene, isolate 3\_C | 1790 | 1790 | 99% | 0.0 | 99% | LT547829.1 |
| 26Select seq KU350605.1 | Pseudomonas brassicacearum strain PG17 16S ribosomal RNA gene, partial sequence | 1790 | 1790 | 99% | 0.0 | 99% | KU350605.1 |
| 27Select seq KU350593.1 | Pseudomonas brassicacearum strain PG16 16S ribosomal RNA gene, partial sequence | 1790 | 1790 | 99% | 0.0 | 99% | KU350593.1 |
| 28Select seq CP012680.1 | Pseudomonas brassicacearum strain LBUM300, complete genome | 1790 | 8947 | 99% | 0.0 | 99% | CP012680.1 |
| 29Select seq CP012831.1 | Pseudomonas fluorescens strain FW300-N2C3, complete genome | 1790 | 8952 | 99% | 0.0 | 99% | CP012831.1 |
| 30Select seq KR054984.1 | Pseudomonas brassicacearum strain KAR22 16S ribosomal RNA gene, complete sequence | 1790 | 1790 | 99% | 0.0 | 99% | KR054984.1 |
| 31Select seq KP762561.1 | Pseudomonas brassicacearum strain IHB B 13650 16S ribosomal RNA gene, partial sequence | 1790 | 1790 | 99% | 0.0 | 99% | KP762561.1 |
| 32Select seq KP762555.1 | Pseudomonas brassicacearum subsp. neoaurantiaca strain IHB B 13645 16S ribosomal RNA gene, partial sequence | 1790 | 1790 | 99% | 0.0 | 99% | KP762555.1 |
| 33Select seq KP253039.1 | Pseudomonas fluorescens strain WCS365 16S ribosomal RNA gene, partial sequence | 1790 | 1790 | 99% | 0.0 | 99% | KP253039.1 |
| 34Select seq LC015569.1 | Pseudomonas brassicacearum subsp. brassicacearum gene for 16S ribosomal RNA, partial sequence, strain: AF5 | 1790 | 1790 | 99% | 0.0 | 99% | LC015569.1 |
| 35Select seq KM253057.1 | Pseudomonas sp. DT 5-12 16S ribosomal RNA gene, partial sequence | 1790 | 1790 | 99% | 0.0 | 99% | KM253057.1 |
| 36Select seq KM253000.1 | Pseudomonas sp. DR 2-03 16S ribosomal RNA gene, partial sequence | 1790 | 1790 | 99% | 0.0 | 99% | KM253000.1 |
| 37Select seq KF148637.1 | Pseudomonas fluorescens strain JK15 16S ribosomal RNA gene, partial sequence | 1790 | 1790 | 99% | 0.0 | 99% | KF148637.1 |
| 38Select seq HF952549.1 | Pseudomonas brassicacearum partial 16S rRNA gene, strain HMGU196 | 1790 | 1790 | 99% | 0.0 | 99% | HF952549.1 |
| 39Select seq HF952537.1 | Pseudomonas brassicacearum partial 16S rRNA gene, strain HMGU70 | 1790 | 1790 | 99% | 0.0 | 99% | HF952537.1 |
| 40Select seq KC246049.1 | Pseudomonas fluorescens strain IBFC2012-45 16S ribosomal RNA gene, partial sequence | 1790 | 1790 | 99% | 0.0 | 99% | KC246049.1 |
| 41Select seq JX912367.1 | Pseudomonas sp. EA3(2012) 16S ribosomal RNA gene, partial sequence | 1790 | 1790 | 99% | 0.0 | 99% | JX912367.1 |
| 42Select seq JN605747.1 | Pseudomonas brassicacearum strain J12 16S ribosomal RNA gene, partial sequence | 1790 | 1790 | 99% | 0.0 | 99% | JN605747.1 |
| 43Select seq FR675976.1 | Pseudomonas sp. LB184 partial 16S rRNA gene, isolate LB184 | 1790 | 1790 | 99% | 0.0 | 99% | FR675976.1 |
| 44Select seq FR675975.1 | Pseudomonas sp. LB183 partial 16S rRNA gene, isolate LB183 | 1790 | 1790 | 99% | 0.0 | 99% | FR675975.1 |
| 45Select seq HQ848635.1 | Pseudomonas sp. SHB3 16S ribosomal RNA gene, partial sequence | 1790 | 1790 | 99% | 0.0 | 99% | HQ848635.1 |
| 46Select seq JF500978.1 | Uncultured Pseudomonas sp. clone 101 16S ribosomal RNA gene, partial sequence | 1790 | 1790 | 99% | 0.0 | 99% | JF500978.1 |
| 47Select seq JF500932.1 | Uncultured Pseudomonas sp. clone 52 16S ribosomal RNA gene, partial sequence | 1790 | 1790 | 99% | 0.0 | 99% | JF500932.1 |
| 48Select seq CP002585.1 | Pseudomonas brassicacearum subsp. brassicacearum NFM421, complete genome | 1790 | 8947 | 99% | 0.0 | 99% | CP002585.1 |
| 49Select seq FN547411.1 | Pseudomonas sp. G68 partial 16S rRNA gene, strain G68 | 1790 | 1790 | 99% | 0.0 | 99% | FN547411.1 |
| 50Select seq DQ453838.1 | Pseudomonas sp. P96.25 16S ribosomal RNA gene, partial sequence | 1790 | 1790 | 99% | 0.0 | 99% | DQ453838.1 |
| 51Select seq DQ453837.1 | Pseudomonas sp. K93.3 16S ribosomal RNA gene, partial sequence | 1790 | 1790 | 99% | 0.0 | 99% | DQ453837.1 |
| 52Select seq DQ453836.1 | Pseudomonas sp. C\*1A1 16S ribosomal RNA gene, partial sequence | 1790 | 1790 | 99% | 0.0 | 99% | DQ453836.1 |
| 53Select seq DQ453835.1 | Pseudomonas sp. TM1A3 16S ribosomal RNA gene, partial sequence | 1790 | 1790 | 99% | 0.0 | 99% | DQ453835.1 |
| 54Select seq DQ453820.1 | Pseudomonas sp. K94.37 16S ribosomal RNA gene, complete sequence | 1790 | 1790 | 99% | 0.0 | 99% | DQ453820.1 |
| 55Select seq DQ453818.1 | Pseudomonas sp. K93.2 16S ribosomal RNA gene, complete sequence | 1790 | 1790 | 99% | 0.0 | 99% | DQ453818.1 |
| 56Select seq AY271792.1 | Pseudomonas aurantiaca VKM B-1524 16S ribosomal RNA gene, partial sequence | 1790 | 1790 | 99% | 0.0 | 99% | AY271792.1 |
| 57Select seq AY512624.1 | Pseudomonas sp. A1Y13 16S ribosomal RNA gene, partial sequence | 1790 | 1790 | 99% | 0.0 | 99% | AY512624.1 |
| 58Select seq AJ417074.1 | Pseudomonas sp. Q65c-80 16S rRNA gene, strain Q65c-80 | 1790 | 1790 | 99% | 0.0 | 99% | AJ417074.1 |
| 59Select seq AJ417068.1 | Pseudomonas sp. CM1'A2 16S rRNA gene, strain CM1'A2 | 1790 | 1790 | 99% | 0.0 | 99% | AJ417068.1 |
| 60Select seq AJ292381.1 | Pseudomonas brassicacearum 16S rRNA gene, strain 520-1 | 1790 | 1790 | 99% | 0.0 | 99% | AJ292381.1 |
| 61Select seq KU882739.1 | Pseudomonas sp. MAR9910 16S ribosomal RNA gene, partial sequence | 1788 | 1788 | 99% | 0.0 | 99% | KU882739.1 |
| 62Select seq KT997466.1 | Pseudomonas brassicacearum subsp. neoaurantiaca strain MLS-8-1 16S ribosomal RNA gene, partial sequence | 1788 | 1788 | 98% | 0.0 | 99% | KT997466.1 |
| 63Select seq KT997442.1 | Pseudomonas brassicacearum subsp. neoaurantiaca strain MLS-2-8 16S ribosomal RNA gene, partial sequence | 1788 | 1788 | 98% | 0.0 | 99% | KT997442.1 |
| 64Select seq KP730603.1 | Pseudomonas brassicacearum subsp. brassicacearum strain BW0808 16S ribosomal RNA gene, partial sequence | 1788 | 1788 | 99% | 0.0 | 99% | KP730603.1 |
| 65Select seq KM269192.1 | Pseudomonas sp. SAM1 16S ribosomal RNA gene, partial sequence | 1788 | 1788 | 99% | 0.0 | 99% | KM269192.1 |
| 66Select seq KF460526.1 | Pseudomonas fluorescens strain ALEB 7B 16S ribosomal RNA gene, partial sequence | 1788 | 1788 | 99% | 0.0 | 99% | KF460526.1 |
| 67Select seq KC139440.1 | Pseudomonas thivervalensis strain h-23 16S ribosomal RNA gene, partial sequence | 1788 | 1788 | 99% | 0.0 | 99% | KC139440.1 |
| 68Select seq CP025542.1 | Pseudomonas fluorescens strain 2P24 chromosome, complete genome | 1784 | 8913 | 99% | 0.0 | 99% | CP025542.1 |
| 69Select seq KX859163.1 | Pseudomonas sp. strain A22 16S ribosomal RNA gene, partial sequence | 1784 | 1784 | 99% | 0.0 | 99% | KX859163.1 |
| 70Select seq KX859162.1 | Pseudomonas sp. strain A21 16S ribosomal RNA gene, partial sequence | 1784 | 1784 | 99% | 0.0 | 99% | KX859162.1 |
| 71Select seq KY111476.1 | Pseudomonas sp. strain B10 16S ribosomal RNA gene, partial sequence | 1784 | 1784 | 99% | 0.0 | 99% | KY111476.1 |
| 72Select seq LT629691.1 | Pseudomonas thivervalensis strain BS3779 genome assembly, chromosome: I | 1784 | 8902 | 99% | 0.0 | 99% | LT629691.1 |
| 73Select seq KR855698.1 | Pseudomonas brassicacearum strain SM27 16S ribosomal RNA gene, partial sequence | 1784 | 1784 | 99% | 0.0 | 99% | KR855698.1 |
| 74Select seq LN880129.1 | Uncultured Pseudomonas sp. partial 16S rRNA gene, clone W3S26 | 1784 | 1784 | 99% | 0.0 | 99% | LN880129.1 |
| 75Select seq KT580640.1 | Pseudomonas fluorescens strain CanR-1 16S ribosomal RNA gene, partial sequence | 1784 | 1784 | 99% | 0.0 | 99% | KT580640.1 |
| 76Select seq LC015568.1 | Pseudomonas brassicacearum subsp. brassicacearum gene for 16S ribosomal RNA, partial sequence, strain: AF78 | 1784 | 1784 | 99% | 0.0 | 99% | LC015568.1 |
| 77Select seq KJ642348.1 | Pseudomonas sp. EA\_S\_74 16S ribosomal RNA gene, partial sequence | 1784 | 1784 | 99% | 0.0 | 99% | KJ642348.1 |
| 78Select seq KJ642346.1 | Pseudomonas sp. EA\_S\_77 16S ribosomal RNA gene, partial sequence | 1784 | 1784 | 99% | 0.0 | 99% | KJ642346.1 |
| 79Select seq KJ642307.1 | Pseudomonas sp. EA\_S\_71 16S ribosomal RNA gene, partial sequence | 1784 | 1784 | 99% | 0.0 | 99% | KJ642307.1 |
| 80Select seq KJ642306.1 | Pseudomonas sp. EA\_S\_7 16S ribosomal RNA gene, partial sequence | 1784 | 1784 | 99% | 0.0 | 99% | KJ642306.1 |
| 81Select seq KJ642305.1 | Pseudomonas sp. EA\_S\_69 16S ribosomal RNA gene, partial sequence | 1784 | 1784 | 99% | 0.0 | 99% | KJ642305.1 |
| 82Select seq KJ642304.1 | Pseudomonas sp. EA\_S\_66 16S ribosomal RNA gene, partial sequence | 1784 | 1784 | 99% | 0.0 | 99% | KJ642304.1 |
| 83Select seq KJ642301.1 | Pseudomonas sp. EA\_S\_62 16S ribosomal RNA gene, partial sequence | 1784 | 1784 | 99% | 0.0 | 99% | KJ642301.1 |
| 84Select seq KJ642267.1 | Pseudomonas sp. EA\_R\_17 16S ribosomal RNA gene, partial sequence | 1784 | 1784 | 99% | 0.0 | 99% | KJ642267.1 |
| 85Select seq KJ642263.1 | Pseudomonas sp. EA\_R\_9 16S ribosomal RNA gene, partial sequence | 1784 | 1784 | 99% | 0.0 | 99% | KJ642263.1 |
| 86Select seq KJ642252.1 | Pseudomonas sp. EA\_R\_69 16S ribosomal RNA gene, partial sequence | 1784 | 1784 | 99% | 0.0 | 99% | KJ642252.1 |
| 87Select seq KF835389.1 | Pseudomonas sp. SDW-16 16S ribosomal RNA gene, partial sequence | 1784 | 1784 | 99% | 0.0 | 99% | KF835389.1 |
| 88Select seq KF312468.1 | Pseudomonas sp. SJ7b 16S ribosomal RNA gene, partial sequence | 1784 | 1784 | 99% | 0.0 | 99% | KF312468.1 |
| 89Select seq JQ977216.1 | Pseudomonas sp. Bcb3 16S ribosomal RNA gene, partial sequence | 1784 | 1784 | 99% | 0.0 | 99% | JQ977216.1 |
| 90Select seq KC428747.1 | Pseudomonas sp. X123 16S ribosomal RNA gene, partial sequence | 1784 | 1784 | 99% | 0.0 | 99% | KC428747.1 |
| 91Select seq ANOR01000024.1 | Pseudomonas fluorescens Pf29Arp contig024, whole genome shotgun sequence | 1784 | 1784 | 99% | 0.0 | 99% | ANOR01000024.1 |
| 92Select seq JX912423.1 | Pseudomonas sp. ED5(2012) 16S ribosomal RNA gene, partial sequence | 1784 | 1784 | 99% | 0.0 | 99% | JX912423.1 |
| 93Select seq AB621592.1 | Pseudomonas fluorescens gene for 16S rRNA, partial sequence, strain: MPF25 | 1784 | 1784 | 99% | 0.0 | 99% | AB621592.1 |
| 94Select seq AB621591.1 | Pseudomonas fluorescens gene for 16S rRNA, partial sequence, strain: MPF18 | 1784 | 1784 | 99% | 0.0 | 99% | AB621591.1 |
| 95Select seq AB602399.1 | Pseudomonas fluorescens gene for 16S ribosomal RNA, partial sequence, strain: MPF3 | 1784 | 1784 | 99% | 0.0 | 99% | AB602399.1 |
| 96Select seq JF500893.1 | Uncultured Pseudomonas sp. clone 7 16S ribosomal RNA gene, partial sequence | 1784 | 1784 | 99% | 0.0 | 99% | JF500893.1 |
| 97Select seq JF500889.1 | Uncultured Pseudomonas sp. clone 3 16S ribosomal RNA gene, partial sequence | 1784 | 1784 | 99% | 0.0 | 99% | JF500889.1 |
| 98Select seq AB621593.1 | Pseudomonas fluorescens gene for 16S rRNA, partial sequence, strain: MPF29 | 1781 | 1781 | 99% | 0.0 | 99% | AB621593.1 |
| 99Select seq AB665552.1 | Pseudomonas fluorescens gene for 16S rRNA, partial sequence, strain: PRS3 | 1781 | 1781 | 99% | 0.0 | 99% | AB665552.1 |
| 100Select seq KX953859.1 | Pseudomonas sp. strain 25C5 16S ribosomal RNA gene, partial sequence | 1779 | 1779 | 99% | 0.0 | 99% | KX953859.1 |


## Alignments

Loading alignment... for sequences gi|1137473154,gi|1008987913,gi|673537586,gi|625293645,gi|385766699 Reading indexes 1-5

Download

FASTA (complete sequence)

FASTA (aligned sequences)

GenBank (complete sequence)

Continue
Cancel

GenBankGraphics

Next
Previous
Descriptions

Pseudomonas sp. strain 7.3 16S ribosomal RNA gene, partial sequence

Sequence ID: KY542120.1Length: 1546Number of Matches: 1

Related Information

Range 1: 56 to 1035GenBankGraphics

Next Match
Previous Match
First Match

Alignment statistics for match #1

| Score | Expect | Identities | Gaps | Strand | Frame |
| --- | --- | --- | --- | --- | --- |
| 1796 bits(972) | 0.0() | 976/980(99%) | 0/980(0%) | Plus/Plus |  |

Features:

```
Query  5     TGCAAGTCGAGCGGTAGAGAGGTGCTTGCACCTCTTGAGAGCGGCGGACGGGTGAGTAAT  64
             ||||||||||||||||||||||||||||||||||||||||||||||||||||||||||||
Sbjct  56    TGCAAGTCGAGCGGTAGAGAGGTGCTTGCACCTCTTGAGAGCGGCGGACGGGTGAGTAAT  115

Query  65    GCCTAGGAATCTGCCTGGTAGTGGGGGATAACGCTCGGAAACGGACGCTAATACCGCATA  124
             ||||||||||||||||||||||||||||||||||||||||||||||||||||||||||||
Sbjct  116   GCCTAGGAATCTGCCTGGTAGTGGGGGATAACGCTCGGAAACGGACGCTAATACCGCATA  175

Query  125   CGTCCTACGGGAGAAAGCAGGGGACCTTCGGGCCTTGCGCTATCAGATGAGCCTAGGTCG  184
             ||||||||||||||||||||||||||||||||||||||||||||||||||||||||||||
Sbjct  176   CGTCCTACGGGAGAAAGCAGGGGACCTTCGGGCCTTGCGCTATCAGATGAGCCTAGGTCG  235

Query  185   GATTAGCTAGTTGGTGGGGTAATGGCTCACCAAGGCGACGATCCGTAACTGGTCTGAGAG  244
             ||||||||||||||||||||||||||||||||||||||||||||||||||||||||||||
Sbjct  236   GATTAGCTAGTTGGTGGGGTAATGGCTCACCAAGGCGACGATCCGTAACTGGTCTGAGAG  295

Query  245   GATGATCAGTCACACTGGAACTGAGACACGGTCCAGACTCCTACGGGAGGCAGCAGTGGG  304
             ||||||||||||||||||||||||||||||||||||||||||||||||||||||||||||
Sbjct  296   GATGATCAGTCACACTGGAACTGAGACACGGTCCAGACTCCTACGGGAGGCAGCAGTGGG  355

Query  305   GAATATTGGACAATGGGCGAAAGCCTGATCCAGCCATGCCGCGTGTGTGAAGAAGGTCTT  364
             ||||||||||||||||||||||||||||||||||||||||||||||||||||||||||||
Sbjct  356   GAATATTGGACAATGGGCGAAAGCCTGATCCAGCCATGCCGCGTGTGTGAAGAAGGTCTT  415

Query  365   CGGATTGTAAAGCACTTTAAGTTGGGAGGAAGGGCATTAACCTAATACGTTAGTGTTTTG  424
             ||||||||||||||||||||||||||||||||||||||||||||||||||||||||||||
Sbjct  416   CGGATTGTAAAGCACTTTAAGTTGGGAGGAAGGGCATTAACCTAATACGTTAGTGTTTTG  475

Query  425   ACGTTACCGACAGAATAAGCACCGGCTAACTCTGTGCCAGCAGCCGCGGTAATACAGAGG  484
             ||||||||||||||||||||||||||||||||||||||||||||||||||||||||||||
Sbjct  476   ACGTTACCGACAGAATAAGCACCGGCTAACTCTGTGCCAGCAGCCGCGGTAATACAGAGG  535

Query  485   GTGCAAGCGTTAATCGGAATTACTGGGCGTAAAGCGCGCGTAGGTGGTTCGTTAAGTTGG  544
             ||||||||||||||||||||||||||||||||||||||||||||||||||||||||||||
Sbjct  536   GTGCAAGCGTTAATCGGAATTACTGGGCGTAAAGCGCGCGTAGGTGGTTCGTTAAGTTGG  595

Query  545   ATGTGAAAGCCCCGGGCTCAACCTGGGAACTGCATTCAAAACTGTCGAGCTAGAGTATGG  604
             ||||||||||||||||||||||||||||||||||||||||||||||||||||||||||||
Sbjct  596   ATGTGAAAGCCCCGGGCTCAACCTGGGAACTGCATTCAAAACTGTCGAGCTAGAGTATGG  655

Query  605   TAGAGGGTGGTGGAATTTCCTGTGTAGCGGTGAAATGCGTAGATATAGGAAGGAACACCA  664
             ||||||||||||||||||||||||||||||||||||||||||||||||||||||||||||
Sbjct  656   TAGAGGGTGGTGGAATTTCCTGTGTAGCGGTGAAATGCGTAGATATAGGAAGGAACACCA  715

Query  665   GTGGCGAAGGCGACCACCTGGACTGATACTGACACTGAGGTGCGAAAGCGTGGGGAGCAA  724
             ||||||||||||||||||||||||||||||||||||||||||||||||||||||||||||
Sbjct  716   GTGGCGAAGGCGACCACCTGGACTGATACTGACACTGAGGTGCGAAAGCGTGGGGAGCAA  775

Query  725   ACAGGATTAGATACCCTGGTAGTCCACGCCGTAAACGATGTCAACTAGCCGTTGGGAGCC  784
             ||||||||||||||||||||||||||||||||||||||||||||||||||||||||||||
Sbjct  776   ACAGGATTAGATACCCTGGTAGTCCACGCCGTAAACGATGTCAACTAGCCGTTGGGAGCC  835

Query  785   TTGAGCTCTTAGTGGCGCAGCTAACGCATTAAGTTGACCGCCTGGGGAGTACGGCCGCAA  844
             ||||||||||||||||||||||||||||||||||||||||||||||||||||||||||||
Sbjct  836   TTGAGCTCTTAGTGGCGCAGCTAACGCATTAAGTTGACCGCCTGGGGAGTACGGCCGCAA  895

Query  845   GGTTAAAACTCAAATGAATTGACGGGGGCCCGCACAAGCGGTGGNNNATGTGGTTTAATT  904
             ||||||||||||||||||||||||||||||||||||||||||||   |||||||||||||
Sbjct  896   GGTTAAAACTCAAATGAATTGACGGGGGCCCGCACAAGCGGTGGAGCATGTGGTTTAATT  955

Query  905   CGAAGCAACGCGAAGAACCTTACCAGGCCTTGACATCCAATGAACTTTCCAGANATGGAT  964
             ||||||||||||||||||||||||||||||||||||||||||||||||||||| ||||||
Sbjct  956   CGAAGCAACGCGAAGAACCTTACCAGGCCTTGACATCCAATGAACTTTCCAGAGATGGAT  1015

Query  965   TGGTGCCTTCGGGAACATTG  984
             ||||||||||||||||||||
Sbjct  1016  TGGTGCCTTCGGGAACATTG  1035
```

Download

FASTA (complete sequence)

FASTA (aligned sequences)

GenBank (complete sequence)

Continue
Cancel

GenBankGraphics

Next
Previous
Descriptions

Pseudomonas sp. MAR9909 16S ribosomal RNA gene, partial sequence

Sequence ID: KU882744.1Length: 1435Number of Matches: 1

Related Information

Range 1: 1 to 980GenBankGraphics

Next Match
Previous Match
First Match

Alignment statistics for match #1

| Score | Expect | Identities | Gaps | Strand | Frame |
| --- | --- | --- | --- | --- | --- |
| 1796 bits(972) | 0.0() | 976/980(99%) | 0/980(0%) | Plus/Plus |  |

Features:

```
Query  5    TGCAAGTCGAGCGGTAGAGAGGTGCTTGCACCTCTTGAGAGCGGCGGACGGGTGAGTAAT  64
            ||||||||||||||||||||||||||||||||||||||||||||||||||||||||||||
Sbjct  1    TGCAAGTCGAGCGGTAGAGAGGTGCTTGCACCTCTTGAGAGCGGCGGACGGGTGAGTAAT  60

Query  65   GCCTAGGAATCTGCCTGGTAGTGGGGGATAACGCTCGGAAACGGACGCTAATACCGCATA  124
            ||||||||||||||||||||||||||||||||||||||||||||||||||||||||||||
Sbjct  61   GCCTAGGAATCTGCCTGGTAGTGGGGGATAACGCTCGGAAACGGACGCTAATACCGCATA  120

Query  125  CGTCCTACGGGAGAAAGCAGGGGACCTTCGGGCCTTGCGCTATCAGATGAGCCTAGGTCG  184
            ||||||||||||||||||||||||||||||||||||||||||||||||||||||||||||
Sbjct  121  CGTCCTACGGGAGAAAGCAGGGGACCTTCGGGCCTTGCGCTATCAGATGAGCCTAGGTCG  180

Query  185  GATTAGCTAGTTGGTGGGGTAATGGCTCACCAAGGCGACGATCCGTAACTGGTCTGAGAG  244
            ||||||||||||||||||||||||||||||||||||||||||||||||||||||||||||
Sbjct  181  GATTAGCTAGTTGGTGGGGTAATGGCTCACCAAGGCGACGATCCGTAACTGGTCTGAGAG  240

Query  245  GATGATCAGTCACACTGGAACTGAGACACGGTCCAGACTCCTACGGGAGGCAGCAGTGGG  304
            ||||||||||||||||||||||||||||||||||||||||||||||||||||||||||||
Sbjct  241  GATGATCAGTCACACTGGAACTGAGACACGGTCCAGACTCCTACGGGAGGCAGCAGTGGG  300

Query  305  GAATATTGGACAATGGGCGAAAGCCTGATCCAGCCATGCCGCGTGTGTGAAGAAGGTCTT  364
            ||||||||||||||||||||||||||||||||||||||||||||||||||||||||||||
Sbjct  301  GAATATTGGACAATGGGCGAAAGCCTGATCCAGCCATGCCGCGTGTGTGAAGAAGGTCTT  360

Query  365  CGGATTGTAAAGCACTTTAAGTTGGGAGGAAGGGCATTAACCTAATACGTTAGTGTTTTG  424
            ||||||||||||||||||||||||||||||||||||||||||||||||||||||||||||
Sbjct  361  CGGATTGTAAAGCACTTTAAGTTGGGAGGAAGGGCATTAACCTAATACGTTAGTGTTTTG  420

Query  425  ACGTTACCGACAGAATAAGCACCGGCTAACTCTGTGCCAGCAGCCGCGGTAATACAGAGG  484
            ||||||||||||||||||||||||||||||||||||||||||||||||||||||||||||
Sbjct  421  ACGTTACCGACAGAATAAGCACCGGCTAACTCTGTGCCAGCAGCCGCGGTAATACAGAGG  480

Query  485  GTGCAAGCGTTAATCGGAATTACTGGGCGTAAAGCGCGCGTAGGTGGTTCGTTAAGTTGG  544
            ||||||||||||||||||||||||||||||||||||||||||||||||||||||||||||
Sbjct  481  GTGCAAGCGTTAATCGGAATTACTGGGCGTAAAGCGCGCGTAGGTGGTTCGTTAAGTTGG  540

Query  545  ATGTGAAAGCCCCGGGCTCAACCTGGGAACTGCATTCAAAACTGTCGAGCTAGAGTATGG  604
            ||||||||||||||||||||||||||||||||||||||||||||||||||||||||||||
Sbjct  541  ATGTGAAAGCCCCGGGCTCAACCTGGGAACTGCATTCAAAACTGTCGAGCTAGAGTATGG  600

Query  605  TAGAGGGTGGTGGAATTTCCTGTGTAGCGGTGAAATGCGTAGATATAGGAAGGAACACCA  664
            ||||||||||||||||||||||||||||||||||||||||||||||||||||||||||||
Sbjct  601  TAGAGGGTGGTGGAATTTCCTGTGTAGCGGTGAAATGCGTAGATATAGGAAGGAACACCA  660

Query  665  GTGGCGAAGGCGACCACCTGGACTGATACTGACACTGAGGTGCGAAAGCGTGGGGAGCAA  724
            ||||||||||||||||||||||||||||||||||||||||||||||||||||||||||||
Sbjct  661  GTGGCGAAGGCGACCACCTGGACTGATACTGACACTGAGGTGCGAAAGCGTGGGGAGCAA  720

Query  725  ACAGGATTAGATACCCTGGTAGTCCACGCCGTAAACGATGTCAACTAGCCGTTGGGAGCC  784
            ||||||||||||||||||||||||||||||||||||||||||||||||||||||||||||
Sbjct  721  ACAGGATTAGATACCCTGGTAGTCCACGCCGTAAACGATGTCAACTAGCCGTTGGGAGCC  780

Query  785  TTGAGCTCTTAGTGGCGCAGCTAACGCATTAAGTTGACCGCCTGGGGAGTACGGCCGCAA  844
            ||||||||||||||||||||||||||||||||||||||||||||||||||||||||||||
Sbjct  781  TTGAGCTCTTAGTGGCGCAGCTAACGCATTAAGTTGACCGCCTGGGGAGTACGGCCGCAA  840

Query  845  GGTTAAAACTCAAATGAATTGACGGGGGCCCGCACAAGCGGTGGNNNATGTGGTTTAATT  904
            ||||||||||||||||||||||||||||||||||||||||||||   |||||||||||||
Sbjct  841  GGTTAAAACTCAAATGAATTGACGGGGGCCCGCACAAGCGGTGGAGCATGTGGTTTAATT  900

Query  905  CGAAGCAACGCGAAGAACCTTACCAGGCCTTGACATCCAATGAACTTTCCAGANATGGAT  964
            ||||||||||||||||||||||||||||||||||||||||||||||||||||| ||||||
Sbjct  901  CGAAGCAACGCGAAGAACCTTACCAGGCCTTGACATCCAATGAACTTTCCAGAGATGGAT  960

Query  965  TGGTGCCTTCGGGAACATTG  984
            ||||||||||||||||||||
Sbjct  961  TGGTGCCTTCGGGAACATTG  980
```

Download

FASTA (complete sequence)

FASTA (aligned sequences)

GenBank (complete sequence)

Continue
Cancel

GenBankGraphics

Next
Previous
Descriptions

Pseudomonas sp. cpRA293 16S ribosomal RNA gene, partial sequence

Sequence ID: KJ510220.1Length: 1420Number of Matches: 1

Related Information

Range 1: 15 to 994GenBankGraphics

Next Match
Previous Match
First Match

Alignment statistics for match #1

| Score | Expect | Identities | Gaps | Strand | Frame |
| --- | --- | --- | --- | --- | --- |
| 1796 bits(972) | 0.0() | 976/980(99%) | 0/980(0%) | Plus/Plus |  |

Features:

```
Query  5    TGCAAGTCGAGCGGTAGAGAGGTGCTTGCACCTCTTGAGAGCGGCGGACGGGTGAGTAAT  64
            ||||||||||||||||||||||||||||||||||||||||||||||||||||||||||||
Sbjct  15   TGCAAGTCGAGCGGTAGAGAGGTGCTTGCACCTCTTGAGAGCGGCGGACGGGTGAGTAAT  74

Query  65   GCCTAGGAATCTGCCTGGTAGTGGGGGATAACGCTCGGAAACGGACGCTAATACCGCATA  124
            ||||||||||||||||||||||||||||||||||||||||||||||||||||||||||||
Sbjct  75   GCCTAGGAATCTGCCTGGTAGTGGGGGATAACGCTCGGAAACGGACGCTAATACCGCATA  134

Query  125  CGTCCTACGGGAGAAAGCAGGGGACCTTCGGGCCTTGCGCTATCAGATGAGCCTAGGTCG  184
            ||||||||||||||||||||||||||||||||||||||||||||||||||||||||||||
Sbjct  135  CGTCCTACGGGAGAAAGCAGGGGACCTTCGGGCCTTGCGCTATCAGATGAGCCTAGGTCG  194

Query  185  GATTAGCTAGTTGGTGGGGTAATGGCTCACCAAGGCGACGATCCGTAACTGGTCTGAGAG  244
            ||||||||||||||||||||||||||||||||||||||||||||||||||||||||||||
Sbjct  195  GATTAGCTAGTTGGTGGGGTAATGGCTCACCAAGGCGACGATCCGTAACTGGTCTGAGAG  254

Query  245  GATGATCAGTCACACTGGAACTGAGACACGGTCCAGACTCCTACGGGAGGCAGCAGTGGG  304
            ||||||||||||||||||||||||||||||||||||||||||||||||||||||||||||
Sbjct  255  GATGATCAGTCACACTGGAACTGAGACACGGTCCAGACTCCTACGGGAGGCAGCAGTGGG  314

Query  305  GAATATTGGACAATGGGCGAAAGCCTGATCCAGCCATGCCGCGTGTGTGAAGAAGGTCTT  364
            ||||||||||||||||||||||||||||||||||||||||||||||||||||||||||||
Sbjct  315  GAATATTGGACAATGGGCGAAAGCCTGATCCAGCCATGCCGCGTGTGTGAAGAAGGTCTT  374

Query  365  CGGATTGTAAAGCACTTTAAGTTGGGAGGAAGGGCATTAACCTAATACGTTAGTGTTTTG  424
            ||||||||||||||||||||||||||||||||||||||||||||||||||||||||||||
Sbjct  375  CGGATTGTAAAGCACTTTAAGTTGGGAGGAAGGGCATTAACCTAATACGTTAGTGTTTTG  434

Query  425  ACGTTACCGACAGAATAAGCACCGGCTAACTCTGTGCCAGCAGCCGCGGTAATACAGAGG  484
            ||||||||||||||||||||||||||||||||||||||||||||||||||||||||||||
Sbjct  435  ACGTTACCGACAGAATAAGCACCGGCTAACTCTGTGCCAGCAGCCGCGGTAATACAGAGG  494

Query  485  GTGCAAGCGTTAATCGGAATTACTGGGCGTAAAGCGCGCGTAGGTGGTTCGTTAAGTTGG  544
            ||||||||||||||||||||||||||||||||||||||||||||||||||||||||||||
Sbjct  495  GTGCAAGCGTTAATCGGAATTACTGGGCGTAAAGCGCGCGTAGGTGGTTCGTTAAGTTGG  554

Query  545  ATGTGAAAGCCCCGGGCTCAACCTGGGAACTGCATTCAAAACTGTCGAGCTAGAGTATGG  604
            ||||||||||||||||||||||||||||||||||||||||||||||||||||||||||||
Sbjct  555  ATGTGAAAGCCCCGGGCTCAACCTGGGAACTGCATTCAAAACTGTCGAGCTAGAGTATGG  614

Query  605  TAGAGGGTGGTGGAATTTCCTGTGTAGCGGTGAAATGCGTAGATATAGGAAGGAACACCA  664
            ||||||||||||||||||||||||||||||||||||||||||||||||||||||||||||
Sbjct  615  TAGAGGGTGGTGGAATTTCCTGTGTAGCGGTGAAATGCGTAGATATAGGAAGGAACACCA  674

Query  665  GTGGCGAAGGCGACCACCTGGACTGATACTGACACTGAGGTGCGAAAGCGTGGGGAGCAA  724
            ||||||||||||||||||||||||||||||||||||||||||||||||||||||||||||
Sbjct  675  GTGGCGAAGGCGACCACCTGGACTGATACTGACACTGAGGTGCGAAAGCGTGGGGAGCAA  734

Query  725  ACAGGATTAGATACCCTGGTAGTCCACGCCGTAAACGATGTCAACTAGCCGTTGGGAGCC  784
            ||||||||||||||||||||||||||||||||||||||||||||||||||||||||||||
Sbjct  735  ACAGGATTAGATACCCTGGTAGTCCACGCCGTAAACGATGTCAACTAGCCGTTGGGAGCC  794

Query  785  TTGAGCTCTTAGTGGCGCAGCTAACGCATTAAGTTGACCGCCTGGGGAGTACGGCCGCAA  844
            ||||||||||||||||||||||||||||||||||||||||||||||||||||||||||||
Sbjct  795  TTGAGCTCTTAGTGGCGCAGCTAACGCATTAAGTTGACCGCCTGGGGAGTACGGCCGCAA  854

Query  845  GGTTAAAACTCAAATGAATTGACGGGGGCCCGCACAAGCGGTGGNNNATGTGGTTTAATT  904
            ||||||||||||||||||||||||||||||||||||||||||||   |||||||||||||
Sbjct  855  GGTTAAAACTCAAATGAATTGACGGGGGCCCGCACAAGCGGTGGAGCATGTGGTTTAATT  914

Query  905  CGAAGCAACGCGAAGAACCTTACCAGGCCTTGACATCCAATGAACTTTCCAGANATGGAT  964
            ||||||||||||||||||||||||||||||||||||||||||||||||||||| ||||||
Sbjct  915  CGAAGCAACGCGAAGAACCTTACCAGGCCTTGACATCCAATGAACTTTCCAGAGATGGAT  974

Query  965  TGGTGCCTTCGGGAACATTG  984
            ||||||||||||||||||||
Sbjct  975  TGGTGCCTTCGGGAACATTG  994
```

Download

FASTA (complete sequence)

FASTA (aligned sequences)

GenBank (complete sequence)

Continue
Cancel

GenBankGraphics

Next
Previous
Descriptions

Pseudomonas thivervalensis strain PE32 16S ribosomal RNA gene, partial sequence

Sequence ID: KJ420530.1Length: 1426Number of Matches: 1

Related Information

Range 1: 7 to 986GenBankGraphics

Next Match
Previous Match
First Match

Alignment statistics for match #1

| Score | Expect | Identities | Gaps | Strand | Frame |
| --- | --- | --- | --- | --- | --- |
| 1796 bits(972) | 0.0() | 976/980(99%) | 0/980(0%) | Plus/Plus |  |

Features:

```
Query  5    TGCAAGTCGAGCGGTAGAGAGGTGCTTGCACCTCTTGAGAGCGGCGGACGGGTGAGTAAT  64
            ||||||||||||||||||||||||||||||||||||||||||||||||||||||||||||
Sbjct  7    TGCAAGTCGAGCGGTAGAGAGGTGCTTGCACCTCTTGAGAGCGGCGGACGGGTGAGTAAT  66

Query  65   GCCTAGGAATCTGCCTGGTAGTGGGGGATAACGCTCGGAAACGGACGCTAATACCGCATA  124
            ||||||||||||||||||||||||||||||||||||||||||||||||||||||||||||
Sbjct  67   GCCTAGGAATCTGCCTGGTAGTGGGGGATAACGCTCGGAAACGGACGCTAATACCGCATA  126

Query  125  CGTCCTACGGGAGAAAGCAGGGGACCTTCGGGCCTTGCGCTATCAGATGAGCCTAGGTCG  184
            ||||||||||||||||||||||||||||||||||||||||||||||||||||||||||||
Sbjct  127  CGTCCTACGGGAGAAAGCAGGGGACCTTCGGGCCTTGCGCTATCAGATGAGCCTAGGTCG  186

Query  185  GATTAGCTAGTTGGTGGGGTAATGGCTCACCAAGGCGACGATCCGTAACTGGTCTGAGAG  244
            ||||||||||||||||||||||||||||||||||||||||||||||||||||||||||||
Sbjct  187  GATTAGCTAGTTGGTGGGGTAATGGCTCACCAAGGCGACGATCCGTAACTGGTCTGAGAG  246

Query  245  GATGATCAGTCACACTGGAACTGAGACACGGTCCAGACTCCTACGGGAGGCAGCAGTGGG  304
            ||||||||||||||||||||||||||||||||||||||||||||||||||||||||||||
Sbjct  247  GATGATCAGTCACACTGGAACTGAGACACGGTCCAGACTCCTACGGGAGGCAGCAGTGGG  306

Query  305  GAATATTGGACAATGGGCGAAAGCCTGATCCAGCCATGCCGCGTGTGTGAAGAAGGTCTT  364
            ||||||||||||||||||||||||||||||||||||||||||||||||||||||||||||
Sbjct  307  GAATATTGGACAATGGGCGAAAGCCTGATCCAGCCATGCCGCGTGTGTGAAGAAGGTCTT  366

Query  365  CGGATTGTAAAGCACTTTAAGTTGGGAGGAAGGGCATTAACCTAATACGTTAGTGTTTTG  424
            ||||||||||||||||||||||||||||||||||||||||||||||||||||||||||||
Sbjct  367  CGGATTGTAAAGCACTTTAAGTTGGGAGGAAGGGCATTAACCTAATACGTTAGTGTTTTG  426

Query  425  ACGTTACCGACAGAATAAGCACCGGCTAACTCTGTGCCAGCAGCCGCGGTAATACAGAGG  484
            ||||||||||||||||||||||||||||||||||||||||||||||||||||||||||||
Sbjct  427  ACGTTACCGACAGAATAAGCACCGGCTAACTCTGTGCCAGCAGCCGCGGTAATACAGAGG  486

Query  485  GTGCAAGCGTTAATCGGAATTACTGGGCGTAAAGCGCGCGTAGGTGGTTCGTTAAGTTGG  544
            ||||||||||||||||||||||||||||||||||||||||||||||||||||||||||||
Sbjct  487  GTGCAAGCGTTAATCGGAATTACTGGGCGTAAAGCGCGCGTAGGTGGTTCGTTAAGTTGG  546

Query  545  ATGTGAAAGCCCCGGGCTCAACCTGGGAACTGCATTCAAAACTGTCGAGCTAGAGTATGG  604
            ||||||||||||||||||||||||||||||||||||||||||||||||||||||||||||
Sbjct  547  ATGTGAAAGCCCCGGGCTCAACCTGGGAACTGCATTCAAAACTGTCGAGCTAGAGTATGG  606

Query  605  TAGAGGGTGGTGGAATTTCCTGTGTAGCGGTGAAATGCGTAGATATAGGAAGGAACACCA  664
            ||||||||||||||||||||||||||||||||||||||||||||||||||||||||||||
Sbjct  607  TAGAGGGTGGTGGAATTTCCTGTGTAGCGGTGAAATGCGTAGATATAGGAAGGAACACCA  666

Query  665  GTGGCGAAGGCGACCACCTGGACTGATACTGACACTGAGGTGCGAAAGCGTGGGGAGCAA  724
            ||||||||||||||||||||||||||||||||||||||||||||||||||||||||||||
Sbjct  667  GTGGCGAAGGCGACCACCTGGACTGATACTGACACTGAGGTGCGAAAGCGTGGGGAGCAA  726

Query  725  ACAGGATTAGATACCCTGGTAGTCCACGCCGTAAACGATGTCAACTAGCCGTTGGGAGCC  784
            ||||||||||||||||||||||||||||||||||||||||||||||||||||||||||||
Sbjct  727  ACAGGATTAGATACCCTGGTAGTCCACGCCGTAAACGATGTCAACTAGCCGTTGGGAGCC  786

Query  785  TTGAGCTCTTAGTGGCGCAGCTAACGCATTAAGTTGACCGCCTGGGGAGTACGGCCGCAA  844
            ||||||||||||||||||||||||||||||||||||||||||||||||||||||||||||
Sbjct  787  TTGAGCTCTTAGTGGCGCAGCTAACGCATTAAGTTGACCGCCTGGGGAGTACGGCCGCAA  846

Query  845  GGTTAAAACTCAAATGAATTGACGGGGGCCCGCACAAGCGGTGGNNNATGTGGTTTAATT  904
            ||||||||||||||||||||||||||||||||||||||||||||   |||||||||||||
Sbjct  847  GGTTAAAACTCAAATGAATTGACGGGGGCCCGCACAAGCGGTGGAGCATGTGGTTTAATT  906

Query  905  CGAAGCAACGCGAAGAACCTTACCAGGCCTTGACATCCAATGAACTTTCCAGANATGGAT  964
            ||||||||||||||||||||||||||||||||||||||||||||||||||||| ||||||
Sbjct  907  CGAAGCAACGCGAAGAACCTTACCAGGCCTTGACATCCAATGAACTTTCCAGAGATGGAT  966

Query  965  TGGTGCCTTCGGGAACATTG  984
            ||||||||||||||||||||
Sbjct  967  TGGTGCCTTCGGGAACATTG  986
```

Download

FASTA (complete sequence)

FASTA (aligned sequences)

GenBank (complete sequence)

Continue
Cancel

GenBankGraphics

Next
Previous
Descriptions

Uncultured Pseudomonas sp. clone QL0ABY37ZD02 16S ribosomal RNA gene, partial sequence

Sequence ID: JQ712549.1Length: 1327Number of Matches: 1

Related Information

Range 1: 23 to 1002GenBankGraphics

Next Match
Previous Match
First Match

Alignment statistics for match #1

| Score | Expect | Identities | Gaps | Strand | Frame |
| --- | --- | --- | --- | --- | --- |
| 1796 bits(972) | 0.0() | 976/980(99%) | 0/980(0%) | Plus/Plus |  |

Features:

```
Query  5     TGCAAGTCGAGCGGTAGAGAGGTGCTTGCACCTCTTGAGAGCGGCGGACGGGTGAGTAAT  64
             ||||||||||||||||||||||||||||||||||||||||||||||||||||||||||||
Sbjct  23    TGCAAGTCGAGCGGTAGAGAGGTGCTTGCACCTCTTGAGAGCGGCGGACGGGTGAGTAAT  82

Query  65    GCCTAGGAATCTGCCTGGTAGTGGGGGATAACGCTCGGAAACGGACGCTAATACCGCATA  124
             ||||||||||||||||||||||||||||||||||||||||||||||||||||||||||||
Sbjct  83    GCCTAGGAATCTGCCTGGTAGTGGGGGATAACGCTCGGAAACGGACGCTAATACCGCATA  142

Query  125   CGTCCTACGGGAGAAAGCAGGGGACCTTCGGGCCTTGCGCTATCAGATGAGCCTAGGTCG  184
             ||||||||||||||||||||||||||||||||||||||||||||||||||||||||||||
Sbjct  143   CGTCCTACGGGAGAAAGCAGGGGACCTTCGGGCCTTGCGCTATCAGATGAGCCTAGGTCG  202

Query  185   GATTAGCTAGTTGGTGGGGTAATGGCTCACCAAGGCGACGATCCGTAACTGGTCTGAGAG  244
             ||||||||||||||||||||||||||||||||||||||||||||||||||||||||||||
Sbjct  203   GATTAGCTAGTTGGTGGGGTAATGGCTCACCAAGGCGACGATCCGTAACTGGTCTGAGAG  262

Query  245   GATGATCAGTCACACTGGAACTGAGACACGGTCCAGACTCCTACGGGAGGCAGCAGTGGG  304
             ||||||||||||||||||||||||||||||||||||||||||||||||||||||||||||
Sbjct  263   GATGATCAGTCACACTGGAACTGAGACACGGTCCAGACTCCTACGGGAGGCAGCAGTGGG  322

Query  305   GAATATTGGACAATGGGCGAAAGCCTGATCCAGCCATGCCGCGTGTGTGAAGAAGGTCTT  364
             ||||||||||||||||||||||||||||||||||||||||||||||||||||||||||||
Sbjct  323   GAATATTGGACAATGGGCGAAAGCCTGATCCAGCCATGCCGCGTGTGTGAAGAAGGTCTT  382

Query  365   CGGATTGTAAAGCACTTTAAGTTGGGAGGAAGGGCATTAACCTAATACGTTAGTGTTTTG  424
             ||||||||||||||||||||||||||||||||||||||||||||||||||||||||||||
Sbjct  383   CGGATTGTAAAGCACTTTAAGTTGGGAGGAAGGGCATTAACCTAATACGTTAGTGTTTTG  442

Query  425   ACGTTACCGACAGAATAAGCACCGGCTAACTCTGTGCCAGCAGCCGCGGTAATACAGAGG  484
             ||||||||||||||||||||||||||||||||||||||||||||||||||||||||||||
Sbjct  443   ACGTTACCGACAGAATAAGCACCGGCTAACTCTGTGCCAGCAGCCGCGGTAATACAGAGG  502

Query  485   GTGCAAGCGTTAATCGGAATTACTGGGCGTAAAGCGCGCGTAGGTGGTTCGTTAAGTTGG  544
             ||||||||||||||||||||||||||||||||||||||||||||||||||||||||||||
Sbjct  503   GTGCAAGCGTTAATCGGAATTACTGGGCGTAAAGCGCGCGTAGGTGGTTCGTTAAGTTGG  562

Query  545   ATGTGAAAGCCCCGGGCTCAACCTGGGAACTGCATTCAAAACTGTCGAGCTAGAGTATGG  604
             ||||||||||||||||||||||||||||||||||||||||||||||||||||||||||||
Sbjct  563   ATGTGAAAGCCCCGGGCTCAACCTGGGAACTGCATTCAAAACTGTCGAGCTAGAGTATGG  622

Query  605   TAGAGGGTGGTGGAATTTCCTGTGTAGCGGTGAAATGCGTAGATATAGGAAGGAACACCA  664
             ||||||||||||||||||||||||||||||||||||||||||||||||||||||||||||
Sbjct  623   TAGAGGGTGGTGGAATTTCCTGTGTAGCGGTGAAATGCGTAGATATAGGAAGGAACACCA  682

Query  665   GTGGCGAAGGCGACCACCTGGACTGATACTGACACTGAGGTGCGAAAGCGTGGGGAGCAA  724
             ||||||||||||||||||||||||||||||||||||||||||||||||||||||||||||
Sbjct  683   GTGGCGAAGGCGACCACCTGGACTGATACTGACACTGAGGTGCGAAAGCGTGGGGAGCAA  742

Query  725   ACAGGATTAGATACCCTGGTAGTCCACGCCGTAAACGATGTCAACTAGCCGTTGGGAGCC  784
             ||||||||||||||||||||||||||||||||||||||||||||||||||||||||||||
Sbjct  743   ACAGGATTAGATACCCTGGTAGTCCACGCCGTAAACGATGTCAACTAGCCGTTGGGAGCC  802

Query  785   TTGAGCTCTTAGTGGCGCAGCTAACGCATTAAGTTGACCGCCTGGGGAGTACGGCCGCAA  844
             ||||||||||||||||||||||||||||||||||||||||||||||||||||||||||||
Sbjct  803   TTGAGCTCTTAGTGGCGCAGCTAACGCATTAAGTTGACCGCCTGGGGAGTACGGCCGCAA  862

Query  845   GGTTAAAACTCAAATGAATTGACGGGGGCCCGCACAAGCGGTGGNNNATGTGGTTTAATT  904
             ||||||||||||||||||||||||||||||||||||||||||||   |||||||||||||
Sbjct  863   GGTTAAAACTCAAATGAATTGACGGGGGCCCGCACAAGCGGTGGAGCATGTGGTTTAATT  922

Query  905   CGAAGCAACGCGAAGAACCTTACCAGGCCTTGACATCCAATGAACTTTCCAGANATGGAT  964
             ||||||||||||||||||||||||||||||||||||||||||||||||||||| ||||||
Sbjct  923   CGAAGCAACGCGAAGAACCTTACCAGGCCTTGACATCCAATGAACTTTCCAGAGATGGAT  982

Query  965   TGGTGCCTTCGGGAACATTG  984
             ||||||||||||||||||||
Sbjct  983   TGGTGCCTTCGGGAACATTG  1002
```


BLAST is a registered trademark of the National Library of Medicine

Support center
Mailing list


YouTube

- National Library Of Medicine
- National Institutes Of Health
- U.S. Department of Health & Human Services
- USA.gov

### NCBI


National Center for Biotechnology Information,
 U.S. National Library of Medicine

8600 Rockville Pike,
Bethesda
 MD,
20894
USA

Policies and Guidelines
|
Contact


PreferencesTurn off

External link. Please review our privacy policy.
