# Supplementary material for: Isothermal microcalorimetry for thermal viable count of microorganisms in pure cultures and stabilized formulations
Source: BMC Microbiol. 2019 Mar 21;19:65. doi: 10.1186/s12866-019-1432-8 (PMC6429831; doi:10.1186/s12866-019-1432-8)
Supplement: Supplementary file 3 — 16S rRNA gene sequences’ BLASTN hits in zipped HTML format. (ZIP 15810 kb) [file 12866_2019_1432_MOESM3_ESM.zip › Best blastn hits/NCBI Blast_60 yellow negative R -- 13..1016 of sequence.html]

NCBI Blast:60 yellow negative R -- 13..1016 of sequence


- NCBI Home
- Sign in to NCBI
- Skip to Main Content
- Skip to Navigation
- About NCBI Accesskeys

U.S. National Library of Medicine

NCBI
National Center for Biotechnology Information

- My NCBI
- Sign in to NCBI
- Register
- Sign Out

BLAST ^®^ » blastn suite » RID-A4X0CX6N015


- Home
- Recent Results
- Saved Strategies
- Help

BLAST Results


Edit and Resubmit
Save Search Strategies
[Sign in above to save your search strategy]

Formatting options 


Download


How to read this page
Blast report description
Questions/comments


|  |  |
| --- | --- |
| Formatting options | |
| Show | Alignment as  HTML Plain text   Old View Reset form to defaults [?]  These options control formatting of alignments in results pages. The default is HTML, but other formats (including plain text) are available. PSSM and PssmWithParameters are representations of Position Specific Scoring Matrices and are only available for PSI-BLAST. The Advanced view option allows the database descriptions to be sorted by various indices in a table. |
| Alignment View | Pairwise Pairwise with dots for identities Query-anchored with dots for identities Query-anchored with letters for identities Flat query-anchored with dots for identities Flat query-anchored with letters for identities [?]  Choose how to view alignments. The default "pairwise" view shows how each subject sequence aligns individually to the query sequence. The "query-anchored" view shows how all subject sequences align to the query sequence. For each view type, you can choose to show "identities" (matching residues) as letters or dots. more... |
| Display | Graphical Overview   Linkout   Sequence Retrieval  NCBI-gi   CDS feature [?]  - Graphical Overview: Graphical Overview: Show graph of similar sequence regions aligned to query.   more... - NCBI-gi: Show NCBI gi identifiers. - CDS feature: Show annotated coding region and translation.   more... |
| Masking | Character:   X for protein, n for nucleotide Lower Case  Color:  Black Grey Red [?]  - Masking Character: Display masked (filtered) sequence regions as lower-case or as specific letters (N for nucleotide, P for protein). - Masking Color: Display masked sequence regions in the given color. |
| Limit results | Descriptions:  10 50 100 Graphical overview:  0 10 50 100  Alignments:  0 10 50 100 Line length:  60 90 120 150 [?]  - Descriptions: Show short descriptions for up to the given number of sequences. - Alignments: Show alignments for up to the given number of sequences, in order of statistical significance. - Line lenghth: Number of letters to show on one line in an alignment. |
|  | Organism Type common name, binomial, taxid, or group name. Only 20 top taxa will be shown.     Exclude   [?]  Show only sequences from the given organism. |
|  | Entrez query:  [?]  Show only those sequences that match the given Entrez query. more... |
|  | Expect Min:  Expect Max:  [?]  Show only sequences with expect values in the given range. more... |
|  | Percent Identity Min:  Percent Identity Max:  [?]  Show only sequences with percent identity values in the given range. |
| Format for | PSI-BLAST with inclusion threshold:  [?]  - Format for PSI-BLAST: The Position-Specific Iterated BLAST (PSI-BLAST) program performs iterative searches with a protein query,   in which sequences found in one round of search are used to build a custom score model for the next round.   more... - Inclusion Threshold: This sets the statistical significance threshold for including a sequence in the model used   by PSI-BLAST to create the PSSM on the next iteration. |

|  |  |  |  |  |  |
| --- | --- | --- | --- | --- | --- |
| Download | | | | | |
| Alignment  Text XML ASN.1 JSON Seq-align Hit Table(text) Hit Table(csv) Multiple-file XML2 Single-file XML2 Multiple-file JSON Single-file JSON SAM | Search Strategies  ASN.1 | PSSM to restart search  PSSM | [?] |  |  |

The Download link provides BLAST output that may be used as input to another program.
This includes parseable formats such as the tabular report or XML as well as the Search Strategy files read by the BLAST+ applications.
More details on the parseable (XML, tabular, and ASN.1) reports can be found at
https://www.ncbi.nlm.nih.gov/books/NBK153387/  
  

The following formats are offered under the Alignment section:  
1). "Text". Non-HTML standard BLAST report.  
2). "XML". XML report based upon the DTD at https://www.ncbi.nlm.nih.gov/data\_specs/dtd/NCBI\_BlastOutput.dtd  
3). "ASN.1". Alignment written out in Abstract Syntax Notation 1.  
4). "JSON Seq-align". Alignment written out in JSON.  
4). "Hit Table(text)". The tabular report as text.  
5). "Hit Table(csv)". The tabular report ready for import into spread-sheet programs like Excel.  
6). "XML2". New XML format described at ftp://ftp.ncbi.nlm.nih.gov/blast/documents/NEWXML/xml2.pdf.  
7). "JSON". New JSON format described at ftp://ftp.ncbi.nlm.nih.gov/blast/documents/NEWXML/xml2.pdf.  
8). "SAM". Sequence Alignment Map format.

XML2 and JSON can be downloaded either as one file per query (multiple-file) or one file for all queries (single-file). These formats are listed as Multiple-file XML2 (and JSON) or Single-file XML (and JSON).

The following report is offered under the Search Strategy section:  
1). "ASN.1" Search Strategy. A record of the parameters, query, and database used in the search. This file can be used to start a stand-alone BLAST search, see
https://www.ncbi.nlm.nih.gov/books/NBK1763/#CmdLineAppsManual.I455\_BLAST\_search\_stra


# Job title: 60 yellow negative R -- 13..1016 of sequence

Results for:

lcl|Query\_205835 60 yellow negative R -- 13..1016 of sequence(1004bp)
[?]

Your BLAST job specified more than one input sequence.
This box lets you choose which input sequence to show BLAST results for.

RID
:   A4X0CX6N015 (Expires on 03-10 18:46 pm)

Query ID
:   lcl|Query\_205835
:   lcl|Query\_205835

Description
:   60 yellow negative R -- 13..1016 of sequence

Molecule type
:   nucleic acid

Query Length
:   1004

Database Name
:   nr

Description
:   Nucleotide collection (nt) See details

Program
:   BLASTN 2.8.0+ Citation

  

Reference 

Zheng Zhang, Scott Schwartz, Lukas Wagner, and Webb Miller (2000), "A greedy algorithm for aligning DNA sequences", J Comput Biol 2000; 7(1-2):203-14.

Reference - database indexing

Aleksandr Morgulis, George Coulouris, Yan Raytselis, Thomas L. Madden, Richa Agarwala, Alejandro A. Schäffer (2008), "Database Indexing for Production MegaBLAST Searches", Bioinformatics 24:1757-1764.

Other reports:
Search Summary

[Taxonomy reports]
[Distance tree of results]
[MSA viewer]

Search Parameters

| Search parameter name | Search parameter value |
| --- | --- |
| Program | blastn |
| Word size | 28 |
| Expect value | 10 |
| Hitlist size | 100 |
| Match/Mismatch scores | 1,-2 |
| Gapcosts | 0,2.5 |
| Low Complexity Filter | Yes |
| Filter string | L;m; |
| Genetic Code | 1 |

Database

| Database parameter name | Database parameter value |
| --- | --- |
| Posted date | Mar 7, 2018 1:58 PM |
| Number of letters | 174,044,644,244 |
| Number of sequences | 46,882,714 |
| Entrez query | Includes:  Excludes:  None |

Karlin-Altschul statistics

| Params | Ungapped | Gapped |
| --- | --- | --- |
| Lambda | 1.33271 | 1.28 |
| K | 0.620991 | 0.46 |
| H | 1.12409 | 0.85 |

Results Statistics

| Results Statistics parameter name | Results Statistics parameter value |
| --- | --- |
| Length adjustment | 35 |
| Effective length of query | 969 |
| Effective length of database | 172403749254 |
| Effective search space | 167059233027126 |
| Effective search space used | 167059233027126 |


## Graphic Summary

### Distribution of the top 118 Blast Hits on 100 subject sequences [?]

The graphic is an overview of the database sequences aligned to the query sequence. These are represented horizontal bars colored coded by score and showing the extent
of the alignment on the query sequence. Separate aligned regions on the same database sequence are connected by a thin grey line.
Mousing over an alignment shows the database sequence title. Clicking an alignment displays a box with more details about the alignment and
link to the sequence alignment itself in the Alignments section of the report.

Mouse over to see the title, click to show alignments

Color key for alignment scores

<40

40-50

50-80

80-200

>=200

Query

1

200

400

600

800

1000

Pantoea vagans strain FDAARGOS\_160 chromosome, complete..

Score:1788 Evalue:0

Accession:CP014129.2

Alignment

Pantoea agglomerans strain AL14 16S ribosomal RNA gene,..

Score:1788 Evalue:0

Accession:MG819218.1

Alignment

Pantoea agglomerans strain Rk92 16S ribosomal RNA gene,..

Score:1788 Evalue:0

Accession:MG547769.1

Alignment

Pantoea agglomerans strain P3FNa\_1 16S ribosomal RNA ge..

Score:1788 Evalue:0

Accession:KX891531.1

Alignment

Pantoea agglomerans strain P2FNb\_1 16S ribosomal RNA ge..

Score:1788 Evalue:0

Accession:KX891517.1

Alignment

Pantoea agglomerans strain P2FNa\_2 16S ribosomal RNA ge..

Score:1788 Evalue:0

Accession:KX891516.1

Alignment

Pantoea agglomerans strain P2FNa\_1 16S ribosomal RNA ge..

Score:1788 Evalue:0

Accession:KX891515.1

Alignment

Pantoea agglomerans strain P1FPa\_2 16S ribosomal RNA ge..

Score:1788 Evalue:0

Accession:KX891502.1

Alignment

Pantoea agglomerans strain P1FNd\_1 16S ribosomal RNA ge..

Score:1788 Evalue:0

Accession:KX891501.1

Alignment

Pantoea agglomerans strain P1FNc\_2 16S ribosomal RNA ge..

Score:1788 Evalue:0

Accession:KX891500.1

Alignment

Pantoea agglomerans strain P1FNc\_1 16S ribosomal RNA ge..

Score:1788 Evalue:0

Accession:KX891499.1

Alignment

Pantoea agglomerans strain P1FNb\_1 16S ribosomal RNA ge..

Score:1788 Evalue:0

Accession:KX891498.1

Alignment

Pantoea agglomerans strain P1FNa\_1 16S ribosomal RNA ge..

Score:1788 Evalue:0

Accession:KX891497.1

Alignment

Pantoea agglomerans strain 62 16S ribosomal RNA gene, p..

Score:1788 Evalue:0

Accession:MF767519.1

Alignment

Pantoea agglomerans strain 22MB 16S ribosomal RNA gene,..

Score:1788 Evalue:0

Accession:MF372619.1

Alignment

Uncultured bacterium clone EHB-P0445 #05 16S ribosomal ..

Score:1788 Evalue:0

Accession:KU978278.1

Alignment

Pantoea agglomerans strain C410P1, complete genome

Score:1788 Evalue:0

Accession:CP016889.1

Alignment

Uncultured Pantoea sp. clone GJ\_(2)\_73 16S ribosomal RN..

Score:1788 Evalue:0

Accession:KX603481.1

Alignment

Uncultured Pantoea sp. clone GJ\_(2)\_7 16S ribosomal RNA..

Score:1788 Evalue:0

Accession:KX603479.1

Alignment

Pantoea sp. CanR-85 16S ribosomal RNA gene, partial seq..

Score:1788 Evalue:0

Accession:KT580675.1

Alignment

Pantoea agglomerans strain SSE 16S ribosomal RNA gene, ..

Score:1788 Evalue:0

Accession:KT075193.1

Alignment

Pantoea agglomerans strain NSF 16S ribosomal RNA gene, ..

Score:1788 Evalue:0

Accession:KT075171.1

Alignment

Pantoea agglomerans strain NSD 16S ribosomal RNA gene, ..

Score:1788 Evalue:0

Accession:KT075169.1

Alignment

Pantoea agglomerans strain GSC 16S ribosomal RNA gene, ..

Score:1788 Evalue:0

Accession:KT075163.1

Alignment

Pantoea agglomerans strain GSB 16S ribosomal RNA gene, ..

Score:1788 Evalue:0

Accession:KT075162.1

Alignment

Pantoea agglomerans strain B81 16S ribosomal RNA gene, ..

Score:1788 Evalue:0

Accession:KJ781904.1

Alignment

Curtobacterium plantarum strain S4 16S ribosomal RNA ge..

Score:1788 Evalue:0

Accession:KP099434.1

Alignment

Curtobacterium plantarum strain S2 16S ribosomal RNA ge..

Score:1788 Evalue:0

Accession:KP099433.1

Alignment

Enterobacteriaceae bacterium X1/SB37 gene for 16S ribos..

Score:1788 Evalue:0

Accession:LC007922.1

Alignment

Enterobacteriaceae bacterium X1/SB86 gene for 16S ribos..

Score:1788 Evalue:0

Accession:LC007911.1

Alignment

Enterobacteriaceae bacterium X1/SB52 gene for 16S ribos..

Score:1788 Evalue:0

Accession:LC007878.1

Alignment

Enterobacteriaceae bacterium X1/SB44 gene for 16S ribos..

Score:1788 Evalue:0

Accession:LC007874.1

Alignment

Enterobacteriaceae bacterium X1/SB18 gene for 16S ribos..

Score:1788 Evalue:0

Accession:LC007863.1

Alignment

Pantoea agglomerans strain ex2 culture-collection CGMCC..

Score:1788 Evalue:0

Accession:KF317872.1

Alignment

Pantoea agglomerans strain ABAC22 16S ribosomal RNA gen..

Score:1788 Evalue:0

Accession:KF003386.1

Alignment

Enterobacteriaceae bacterium SAP952.2 16S ribosomal RNA..

Score:1788 Evalue:0

Accession:JX067721.1

Alignment

Enterobacteriaceae bacterium SAP770.3 16S ribosomal RNA..

Score:1788 Evalue:0

Accession:JX067684.1

Alignment

Enterobacteriaceae bacterium SAP108.1 16S ribosomal RNA..

Score:1788 Evalue:0

Accession:JX067654.1

Alignment

Pantoea agglomerans strain Z01 16S ribosomal RNA gene, ..

Score:1788 Evalue:0

Accession:JX257179.1

Alignment

Uncultured Pantoea sp. clone WRFC18 16S ribosomal RNA g..

Score:1788 Evalue:0

Accession:JX279936.1

Alignment

Pantoea agglomerans strain -Y115 16S ribosomal RNA gene..

Score:1788 Evalue:0

Accession:JX134624.1

Alignment

Pantoea agglomerans strain Y34 16S ribosomal RNA gene, ..

Score:1788 Evalue:0

Accession:JX113242.1

Alignment

Pantoea agglomerans strain KB64 16S ribosomal RNA gene,..

Score:1788 Evalue:0

Accession:JF327484.1

Alignment

Pantoea agglomerans strain KB38 16S ribosomal RNA gene,..

Score:1788 Evalue:0

Accession:JF327464.1

Alignment

Pantoea agglomerans strain CJ-G-R2A5 16S ribosomal RNA ..

Score:1788 Evalue:0

Accession:HM584289.1

Alignment

[Curtobacterium] plantarum strain CL63 16S ribosomal RN..

Score:1788 Evalue:0

Accession:NR\_104943.1

Alignment

Pantoea agglomerans isolate PSB27 16S ribosomal RNA gen..

Score:1788 Evalue:0

Accession:HQ242740.1

Alignment

Pantoea agglomerans isolate PSB26 16S ribosomal RNA gen..

Score:1788 Evalue:0

Accession:HQ242739.1

Alignment

Pantoea vagans C9-1, complete genome

Score:1788 Evalue:0

Accession:CP002206.1

Alignment

Pantoea agglomerans strain 1.2244 16S ribosomal RNA gen..

Score:1788 Evalue:0

Accession:HM130689.1

Alignment

Pantoea agglomerans strain SR-1 16S ribosomal RNA gene,..

Score:1788 Evalue:0

Accession:FJ593000.1

Alignment

Pantoea agglomerans strain SN-2 16S ribosomal RNA gene,..

Score:1788 Evalue:0

Accession:FJ592996.1

Alignment

Pantoea agglomerans strain NZ 16S ribosomal RNA gene, p..

Score:1788 Evalue:0

Accession:FJ756348.1

Alignment

Pantoea agglomerans strain VA21971 16S ribosomal RNA ge..

Score:1788 Evalue:0

Accession:FJ611833.1

Alignment

Pantoea agglomerans strain P5WAM 16S ribosomal RNA gene..

Score:1788 Evalue:0

Accession:FJ611822.1

Alignment

Pantoea agglomerans pv. gypsophilae strain ATCC 43348 1..

Score:1788 Evalue:0

Accession:FJ611821.1

Alignment

## Descriptions

, Reading indexes 1-5, displaying indexes 1-5


Load next setPrevious Match

Sequences producing significant alignments:

Show all columns  of the table presenting sequences producing significant alignments 

Select:AllNone
Selected:0

Alignments
Download

FASTA (complete sequence)

FASTA (aligned sequences)

GenBank (complete sequence)

Hit Table (text)

Hit Table (CSV)

Text

XML

ASN.1

Continue
Cancel

GenBank 
Graphics
Distance tree of results
Multiple alignment
Show/hide columns of the table presenting sequences producing significant alignments 

Available columns

Description  
Max Score  
Total Score  
Coverage  
E-value  
IdentN  
Accession  
Restore Defaults
Ok
Cancel

Sequences producing significant alignments:

| Select for downloading or viewing reports | Description | Max score | Total score | Query cover | E value | Ident | Accession |
| --- | --- | --- | --- | --- | --- | --- | --- |
| 1Select seq CP014129.2 | Pantoea vagans strain FDAARGOS\_160 chromosome, complete genome | 1788 | 12493 | 98% | 0.0 | 99% | CP014129.2 |
| 2Select seq MG819218.1 | Pantoea agglomerans strain AL14 16S ribosomal RNA gene, partial sequence | 1788 | 1788 | 98% | 0.0 | 99% | MG819218.1 |
| 3Select seq MG547769.1 | Pantoea agglomerans strain Rk92 16S ribosomal RNA gene, partial sequence | 1788 | 1788 | 98% | 0.0 | 99% | MG547769.1 |
| 4Select seq KX891531.1 | Pantoea agglomerans strain P3FNa\_1 16S ribosomal RNA gene, partial sequence | 1788 | 1788 | 98% | 0.0 | 99% | KX891531.1 |
| 5Select seq KX891517.1 | Pantoea agglomerans strain P2FNb\_1 16S ribosomal RNA gene, partial sequence | 1788 | 1788 | 98% | 0.0 | 99% | KX891517.1 |
| 6Select seq KX891516.1 | Pantoea agglomerans strain P2FNa\_2 16S ribosomal RNA gene, partial sequence | 1788 | 1788 | 98% | 0.0 | 99% | KX891516.1 |
| 7Select seq KX891515.1 | Pantoea agglomerans strain P2FNa\_1 16S ribosomal RNA gene, partial sequence | 1788 | 1788 | 98% | 0.0 | 99% | KX891515.1 |
| 8Select seq KX891502.1 | Pantoea agglomerans strain P1FPa\_2 16S ribosomal RNA gene, partial sequence | 1788 | 1788 | 98% | 0.0 | 99% | KX891502.1 |
| 9Select seq KX891501.1 | Pantoea agglomerans strain P1FNd\_1 16S ribosomal RNA gene, partial sequence | 1788 | 1788 | 98% | 0.0 | 99% | KX891501.1 |
| 10Select seq KX891500.1 | Pantoea agglomerans strain P1FNc\_2 16S ribosomal RNA gene, partial sequence | 1788 | 1788 | 98% | 0.0 | 99% | KX891500.1 |
| 11Select seq KX891499.1 | Pantoea agglomerans strain P1FNc\_1 16S ribosomal RNA gene, partial sequence | 1788 | 1788 | 98% | 0.0 | 99% | KX891499.1 |
| 12Select seq KX891498.1 | Pantoea agglomerans strain P1FNb\_1 16S ribosomal RNA gene, partial sequence | 1788 | 1788 | 98% | 0.0 | 99% | KX891498.1 |
| 13Select seq KX891497.1 | Pantoea agglomerans strain P1FNa\_1 16S ribosomal RNA gene, partial sequence | 1788 | 1788 | 98% | 0.0 | 99% | KX891497.1 |
| 14Select seq MF767519.1 | Pantoea agglomerans strain 62 16S ribosomal RNA gene, partial sequence | 1788 | 1788 | 98% | 0.0 | 99% | MF767519.1 |
| 15Select seq MF372619.1 | Pantoea agglomerans strain 22MB 16S ribosomal RNA gene, partial sequence | 1788 | 1788 | 98% | 0.0 | 99% | MF372619.1 |
| 16Select seq KU978278.1 | Uncultured bacterium clone EHB-P0445 #05 16S ribosomal RNA gene, partial sequence | 1788 | 1788 | 98% | 0.0 | 99% | KU978278.1 |
| 17Select seq CP016889.1 | Pantoea agglomerans strain C410P1, complete genome | 1788 | 12409 | 98% | 0.0 | 99% | CP016889.1 |
| 18Select seq KX603481.1 | Uncultured Pantoea sp. clone GJ\_(2)\_73 16S ribosomal RNA gene, partial sequence | 1788 | 1788 | 98% | 0.0 | 99% | KX603481.1 |
| 19Select seq KX603479.1 | Uncultured Pantoea sp. clone GJ\_(2)\_7 16S ribosomal RNA gene, partial sequence | 1788 | 1788 | 98% | 0.0 | 99% | KX603479.1 |
| 20Select seq KT580675.1 | Pantoea sp. CanR-85 16S ribosomal RNA gene, partial sequence | 1788 | 1788 | 98% | 0.0 | 99% | KT580675.1 |
| 21Select seq KT075193.1 | Pantoea agglomerans strain SSE 16S ribosomal RNA gene, partial sequence | 1788 | 1788 | 98% | 0.0 | 99% | KT075193.1 |
| 22Select seq KT075171.1 | Pantoea agglomerans strain NSF 16S ribosomal RNA gene, partial sequence | 1788 | 1788 | 98% | 0.0 | 99% | KT075171.1 |
| 23Select seq KT075169.1 | Pantoea agglomerans strain NSD 16S ribosomal RNA gene, partial sequence | 1788 | 1788 | 98% | 0.0 | 99% | KT075169.1 |
| 24Select seq KT075163.1 | Pantoea agglomerans strain GSC 16S ribosomal RNA gene, partial sequence | 1788 | 1788 | 98% | 0.0 | 99% | KT075163.1 |
| 25Select seq KT075162.1 | Pantoea agglomerans strain GSB 16S ribosomal RNA gene, partial sequence | 1788 | 1788 | 98% | 0.0 | 99% | KT075162.1 |
| 26Select seq KJ781904.1 | Pantoea agglomerans strain B81 16S ribosomal RNA gene, partial sequence | 1788 | 1788 | 98% | 0.0 | 99% | KJ781904.1 |
| 27Select seq KP099434.1 | Curtobacterium plantarum strain S4 16S ribosomal RNA gene, partial sequence | 1788 | 1788 | 98% | 0.0 | 99% | KP099434.1 |
| 28Select seq KP099433.1 | Curtobacterium plantarum strain S2 16S ribosomal RNA gene, partial sequence | 1788 | 1788 | 98% | 0.0 | 99% | KP099433.1 |
| 29Select seq LC007922.1 | Enterobacteriaceae bacterium X1/SB37 gene for 16S ribosomal RNA, partial sequence, strain: X1/SB37 | 1788 | 1788 | 98% | 0.0 | 99% | LC007922.1 |
| 30Select seq LC007911.1 | Enterobacteriaceae bacterium X1/SB86 gene for 16S ribosomal RNA, partial sequence, strain: X1/SB86 | 1788 | 1788 | 98% | 0.0 | 99% | LC007911.1 |
| 31Select seq LC007878.1 | Enterobacteriaceae bacterium X1/SB52 gene for 16S ribosomal RNA, partial sequence, strain: X1/SB52 | 1788 | 1788 | 98% | 0.0 | 99% | LC007878.1 |
| 32Select seq LC007874.1 | Enterobacteriaceae bacterium X1/SB44 gene for 16S ribosomal RNA, partial sequence, strain: X1/SB44 | 1788 | 1788 | 98% | 0.0 | 99% | LC007874.1 |
| 33Select seq LC007863.1 | Enterobacteriaceae bacterium X1/SB18 gene for 16S ribosomal RNA, partial sequence, strain: X1/SB18 | 1788 | 1788 | 98% | 0.0 | 99% | LC007863.1 |
| 34Select seq KF317872.1 | Pantoea agglomerans strain ex2 culture-collection CGMCC:7.103 16S ribosomal RNA gene, partial sequence | 1788 | 1788 | 98% | 0.0 | 99% | KF317872.1 |
| 35Select seq KF003386.1 | Pantoea agglomerans strain ABAC22 16S ribosomal RNA gene, partial sequence | 1788 | 1788 | 98% | 0.0 | 99% | KF003386.1 |
| 36Select seq JX067721.1 | Enterobacteriaceae bacterium SAP952.2 16S ribosomal RNA gene, partial sequence | 1788 | 1788 | 98% | 0.0 | 99% | JX067721.1 |
| 37Select seq JX067684.1 | Enterobacteriaceae bacterium SAP770.3 16S ribosomal RNA gene, partial sequence | 1788 | 1788 | 98% | 0.0 | 99% | JX067684.1 |
| 38Select seq JX067654.1 | Enterobacteriaceae bacterium SAP108.1 16S ribosomal RNA gene, partial sequence | 1788 | 1788 | 98% | 0.0 | 99% | JX067654.1 |
| 39Select seq JX257179.1 | Pantoea agglomerans strain Z01 16S ribosomal RNA gene, partial sequence | 1788 | 1788 | 98% | 0.0 | 99% | JX257179.1 |
| 40Select seq JX279936.1 | Uncultured Pantoea sp. clone WRFC18 16S ribosomal RNA gene, partial sequence | 1788 | 1788 | 98% | 0.0 | 99% | JX279936.1 |
| 41Select seq JX134624.1 | Pantoea agglomerans strain -Y115 16S ribosomal RNA gene, partial sequence | 1788 | 1788 | 98% | 0.0 | 99% | JX134624.1 |
| 42Select seq JX113242.1 | Pantoea agglomerans strain Y34 16S ribosomal RNA gene, partial sequence | 1788 | 1788 | 98% | 0.0 | 99% | JX113242.1 |
| 43Select seq JF327484.1 | Pantoea agglomerans strain KB64 16S ribosomal RNA gene, partial sequence | 1788 | 1788 | 98% | 0.0 | 99% | JF327484.1 |
| 44Select seq JF327464.1 | Pantoea agglomerans strain KB38 16S ribosomal RNA gene, partial sequence | 1788 | 1788 | 98% | 0.0 | 99% | JF327464.1 |
| 45Select seq HM584289.1 | Pantoea agglomerans strain CJ-G-R2A5 16S ribosomal RNA gene, partial sequence | 1788 | 1788 | 98% | 0.0 | 99% | HM584289.1 |
| 46Select seq NR\_104943.1 | [Curtobacterium] plantarum strain CL63 16S ribosomal RNA, partial sequence | 1788 | 1788 | 98% | 0.0 | 99% | NR\_104943.1 |
| 47Select seq HQ242740.1 | Pantoea agglomerans isolate PSB27 16S ribosomal RNA gene, partial sequence | 1788 | 1788 | 98% | 0.0 | 99% | HQ242740.1 |
| 48Select seq HQ242739.1 | Pantoea agglomerans isolate PSB26 16S ribosomal RNA gene, partial sequence | 1788 | 1788 | 98% | 0.0 | 99% | HQ242739.1 |
| 49Select seq CP002206.1 | Pantoea vagans C9-1, complete genome | 1788 | 12354 | 98% | 0.0 | 99% | CP002206.1 |
| 50Select seq HM130689.1 | Pantoea agglomerans strain 1.2244 16S ribosomal RNA gene, partial sequence | 1788 | 1788 | 98% | 0.0 | 99% | HM130689.1 |
| 51Select seq FJ593000.1 | Pantoea agglomerans strain SR-1 16S ribosomal RNA gene, partial sequence | 1788 | 1788 | 98% | 0.0 | 99% | FJ593000.1 |
| 52Select seq FJ592996.1 | Pantoea agglomerans strain SN-2 16S ribosomal RNA gene, partial sequence | 1788 | 1788 | 98% | 0.0 | 99% | FJ592996.1 |
| 53Select seq FJ756348.1 | Pantoea agglomerans strain NZ 16S ribosomal RNA gene, partial sequence | 1788 | 1788 | 98% | 0.0 | 99% | FJ756348.1 |
| 54Select seq FJ611833.1 | Pantoea agglomerans strain VA21971 16S ribosomal RNA gene, partial sequence | 1788 | 1788 | 98% | 0.0 | 99% | FJ611833.1 |
| 55Select seq FJ611822.1 | Pantoea agglomerans strain P5WAM 16S ribosomal RNA gene, partial sequence | 1788 | 1788 | 98% | 0.0 | 99% | FJ611822.1 |
| 56Select seq FJ611821.1 | Pantoea agglomerans pv. gypsophilae strain ATCC 43348 16S ribosomal RNA gene, partial sequence | 1788 | 1788 | 98% | 0.0 | 99% | FJ611821.1 |
| 57Select seq EU409856.1 | Uncultured gamma proteobacterium clone 16S5 16S ribosomal RNA gene, partial sequence | 1788 | 1788 | 98% | 0.0 | 99% | EU409856.1 |
| 58Select seq FJ357809.1 | Pantoea agglomerans strain BBPE0103202 16S ribosomal RNA gene, partial sequence | 1788 | 1788 | 98% | 0.0 | 99% | FJ357809.1 |
| 59Select seq EU828365.1 | Pantoea sp. Wd5760 16S ribosomal RNA gene, partial sequence | 1788 | 1788 | 98% | 0.0 | 99% | EU828365.1 |
| 60Select seq AF130927.2 | Enterobacter agglomerans strain A57 16S ribosomal RNA, partial sequence | 1788 | 1788 | 98% | 0.0 | 99% | AF130927.2 |
| 61Select seq EU753130.1 | Marine bacterium MSC106 16S ribosomal RNA gene, partial sequence | 1788 | 1788 | 98% | 0.0 | 99% | EU753130.1 |
| 62Select seq EU598802.1 | Pantoea agglomerans 16S ribosomal RNA gene, partial sequence | 1788 | 1788 | 98% | 0.0 | 99% | EU598802.1 |
| 63Select seq EU047555.1 | Pantoea agglomerans strain TMPSB-P1 16S ribosomal RNA gene, partial sequence | 1788 | 1788 | 98% | 0.0 | 99% | EU047555.1 |
| 64Select seq DQ307453.1 | Pantoea agglomerans strain MM2 16S ribosomal RNA gene, partial sequence | 1788 | 1788 | 98% | 0.0 | 99% | DQ307453.1 |
| 65Select seq DQ307452.1 | Pantoea agglomerans strain FL1 16S ribosomal RNA gene, partial sequence | 1788 | 1788 | 98% | 0.0 | 99% | DQ307452.1 |
| 66Select seq DQ531643.1 | Pantoea sp. 2-2-SS-001 16S ribosomal RNA gene, partial sequence | 1788 | 1788 | 98% | 0.0 | 99% | DQ531643.1 |
| 67Select seq AB004757.2 | Pantoea agglomerans gene for 16S ribosomal RNA, partial sequence | 1788 | 1788 | 98% | 0.0 | 99% | AB004757.2 |
| 68Select seq DQ311006.1 | Pantoea sp. FL9 16S ribosomal RNA gene, partial sequence | 1788 | 1788 | 98% | 0.0 | 99% | DQ311006.1 |
| 69Select seq AJ583011.1 | Pantoea agglomerans partial 16S rRNA gene, strain EPS125 | 1788 | 1788 | 98% | 0.0 | 99% | AJ583011.1 |
| 70Select seq NR\_041978.1 | Pantoea agglomerans strain DSM 3493 16S ribosomal RNA gene, partial sequence | 1788 | 1788 | 98% | 0.0 | 99% | NR\_041978.1 |
| 71Select seq KU258277.1 | Pantoea agglomerans strain BL26 16S ribosomal RNA gene, partial sequence | 1786 | 1786 | 98% | 0.0 | 99% | KU258277.1 |
| 72Select seq KT075184.1 | Pantoea agglomerans strain QSC 16S ribosomal RNA gene, partial sequence | 1786 | 1786 | 98% | 0.0 | 99% | KT075184.1 |
| 73Select seq KF153221.1 | Pantoea sp. E100 16S ribosomal RNA gene, partial sequence | 1786 | 1786 | 98% | 0.0 | 99% | KF153221.1 |
| 74Select seq FJ267564.1 | Pantoea sp. II\_Gauze\_W\_10\_17 16S ribosomal RNA gene, partial sequence | 1786 | 1786 | 98% | 0.0 | 99% | FJ267564.1 |
| 75Select seq AJ583835.1 | Pantoea agglomerans partial 16S rRNA gene, strain Pa850T | 1786 | 1786 | 98% | 0.0 | 99% | AJ583835.1 |
| 76Select seq MG819352.1 | Curtobacterium sp. strain AL228 16S ribosomal RNA gene, partial sequence | 1784 | 1784 | 98% | 0.0 | 99% | MG819352.1 |
| 77Select seq KY451836.1 | Pantoea deleyi strain 10DM4-1 16S ribosomal RNA gene, partial sequence | 1784 | 1784 | 98% | 0.0 | 99% | KY451836.1 |
| 78Select seq MG388298.1 | Pantoea agglomerans strain MT4 16S ribosomal RNA gene, partial sequence | 1784 | 1784 | 98% | 0.0 | 99% | MG388298.1 |
| 79Select seq KY907002.1 | Curtobacterium sp. strain D-130 16S ribosomal RNA gene, partial sequence | 1784 | 1784 | 98% | 0.0 | 99% | KY907002.1 |
| 80Select seq KT766084.1 | Pantoea agglomerans strain TGJ2 16S ribosomal RNA gene, partial sequence | 1784 | 1784 | 98% | 0.0 | 99% | KT766084.1 |
| 81Select seq KT766082.1 | Pantoea agglomerans strain TPJ1 16S ribosomal RNA gene, partial sequence | 1784 | 1784 | 98% | 0.0 | 99% | KT766082.1 |
| 82Select seq KT075200.1 | Pantoea agglomerans strain SXD 16S ribosomal RNA gene, partial sequence | 1784 | 1784 | 98% | 0.0 | 99% | KT075200.1 |
| 83Select seq KT075181.1 | Pantoea agglomerans strain NXG 16S ribosomal RNA gene, partial sequence | 1784 | 1784 | 98% | 0.0 | 99% | KT075181.1 |
| 84Select seq KT075179.1 | Pantoea agglomerans strain NXE 16S ribosomal RNA gene, partial sequence | 1784 | 1784 | 98% | 0.0 | 99% | KT075179.1 |
| 85Select seq KT075177.1 | Pantoea agglomerans strain NXC 16S ribosomal RNA gene, partial sequence | 1784 | 1784 | 98% | 0.0 | 99% | KT075177.1 |
| 86Select seq KT075170.1 | Pantoea agglomerans strain NSE 16S ribosomal RNA gene, partial sequence | 1784 | 1784 | 98% | 0.0 | 99% | KT075170.1 |
| 87Select seq KT075168.1 | Pantoea agglomerans strain NSC 16S ribosomal RNA gene, partial sequence | 1784 | 1784 | 98% | 0.0 | 99% | KT075168.1 |
| 88Select seq LC007926.1 | Enterobacteriaceae bacterium X1/SB91 gene for 16S ribosomal RNA, partial sequence, strain: X1/SB91 | 1784 | 1784 | 98% | 0.0 | 99% | LC007926.1 |
| 89Select seq LC007903.1 | Enterobacteriaceae bacterium X1/SB55 gene for 16S ribosomal RNA, partial sequence, strain: X1/SB55 | 1784 | 1784 | 98% | 0.0 | 99% | LC007903.1 |
| 90Select seq JF825482.1 | Uncultured gamma proteobacterium clone ASC108 16S ribosomal RNA gene, partial sequence | 1784 | 1784 | 98% | 0.0 | 99% | JF825482.1 |
| 91Select seq AB552921.1 | Pantoea sp. NCCP-140 gene for 16S rRNA, partial sequence | 1784 | 1784 | 98% | 0.0 | 99% | AB552921.1 |
| 92Select seq GQ494018.1 | Pantoea agglomerans strain 14 16S ribosomal RNA gene, partial sequence | 1784 | 1784 | 98% | 0.0 | 99% | GQ494018.1 |
| 93Select seq FJ357814.1 | Pantoea agglomerans strain CUETM8553 16S ribosomal RNA gene, partial sequence | 1784 | 1784 | 98% | 0.0 | 99% | FJ357814.1 |
| 94Select seq FJ006863.1 | Pantoea sp. WPCB006 16S ribosomal RNA gene, partial sequence | 1784 | 1784 | 98% | 0.0 | 99% | FJ006863.1 |
| 95Select seq KX891508.1 | Pantoea agglomerans strain P1MNa\_1 16S ribosomal RNA gene, partial sequence | 1783 | 1783 | 98% | 0.0 | 99% | KX891508.1 |
| 96Select seq KU978258.1 | Uncultured bacterium clone EHB-P0362 #05 16S ribosomal RNA gene, partial sequence | 1783 | 1783 | 98% | 0.0 | 99% | KU978258.1 |
| 97Select seq KU978201.1 | Uncultured bacterium clone EHB-P0048 #15 16S ribosomal RNA gene, partial sequence | 1783 | 1783 | 98% | 0.0 | 99% | KU978201.1 |
| 98Select seq KY357288.1 | Pantoea agglomerans strain ACBP2 16S ribosomal RNA gene, partial sequence | 1783 | 1783 | 98% | 0.0 | 99% | KY357288.1 |
| 99Select seq KY357286.1 | Pantoea agglomerans strain ACBC2 16S ribosomal RNA gene, partial sequence | 1783 | 1783 | 98% | 0.0 | 99% | KY357286.1 |
| 100Select seq KT779903.1 | Pantoea agglomerans strain CCRI01 16S ribosomal RNA gene, partial sequence | 1783 | 1783 | 98% | 0.0 | 99% | KT779903.1 |


## Alignments

Loading alignment... for sequences gi|1344462721,gi|1332982380,gi|1278992521,gi|1070257896,gi|1070257882 Reading indexes 1-5

Download

FASTA (complete sequence)

FASTA (aligned sequences)

GenBank (complete sequence)

Continue
Cancel

GenBankGraphics

Sort by:

E value
 Score
Percent identity
Query start position
Subject start position

Next
Previous
Descriptions

Pantoea vagans strain FDAARGOS\_160 chromosome, complete genome

Sequence ID: CP014129.2Length: 4050121Number of Matches: 7

Related Information

Range 1: 49768 to 50755GenBankGraphics

Next Match
Previous Match
First Match

Alignment statistics for match #1

| Score | Expect | Identities | Gaps | Strand | Frame |
| --- | --- | --- | --- | --- | --- |
| 1788 bits(968) | 0.0() | 978/988(99%) | 0/988(0%) | Plus/Plus |  |

Features:

```
Query  9      CGCCCTCCCGAAGGNTNNGCTACCTACTTCTTTTNNANCCCACTCCCATGGTGTGACGGG  68
              |||||||||||||| |  ||||||||||||||||  | ||||||||||||||||||||||
Sbjct  49768  CGCCCTCCCGAAGGTTAAGCTACCTACTTCTTTTGCAACCCACTCCCATGGTGTGACGGG  49827

Query  69     CGGTGTGTACAAGGCCCGGGAACGTATTCACCGTGGCATTCTGATCCACGATTACTAGCG  128
              ||||||||||||||||||||||||||||||||||||||||||||||||||||||||||||
Sbjct  49828  CGGTGTGTACAAGGCCCGGGAACGTATTCACCGTGGCATTCTGATCCACGATTACTAGCG  49887

Query  129    ATTCCGACTTCACGGAGTCGAGTTGCAGACTCCGATCCGGACTACGACGCACTTTGTGAG  188
              ||||||||||||||||||||||||||||||||||||||||||||||||||||||||||||
Sbjct  49888  ATTCCGACTTCACGGAGTCGAGTTGCAGACTCCGATCCGGACTACGACGCACTTTGTGAG  49947

Query  189    GTCCGCTTGCTCTCGCGAGGTCGCTTCTCTTTGTATGCGCCATTGTAGCACGTGTGTAGC  248
              ||||||||||||||||||||||||||||||||||||||||||||||||||||||||||||
Sbjct  49948  GTCCGCTTGCTCTCGCGAGGTCGCTTCTCTTTGTATGCGCCATTGTAGCACGTGTGTAGC  50007

Query  249    CCTACTCGTAAGGGCCATGATGACTTGACGTCATCCCCACCTTCCTCCGGTTTATCACCG  308
              ||||||||||||||||||||||||||||||||||||||||||||||||||||||||||||
Sbjct  50008  CCTACTCGTAAGGGCCATGATGACTTGACGTCATCCCCACCTTCCTCCGGTTTATCACCG  50067

Query  309    GCAGTCTCCTTTGAGTTCCCGACCGAATCGCTGGCAACAAAGGATAAGGGTTGCGCTCGT  368
              ||||||||||||||||||||||||||||||||||||||||||||||||||||||||||||
Sbjct  50068  GCAGTCTCCTTTGAGTTCCCGACCGAATCGCTGGCAACAAAGGATAAGGGTTGCGCTCGT  50127

Query  369    TGCGGGACTTAACCCAACATTTCACAACACGAGCTGACGACAGCCATGCAGCACCTGTCT  428
              ||||||||||||||||||||||||||||||||||||||||||||||||||||||||||||
Sbjct  50128  TGCGGGACTTAACCCAACATTTCACAACACGAGCTGACGACAGCCATGCAGCACCTGTCT  50187

Query  429    CACGGTTCCCGAAGGCACTAAGGCATCTCTGNNNAATTCCGTGGATGTCAAGAGTAGGTA  488
              |||||||||||||||||||||||||||||||   ||||||||||||||||||||||||||
Sbjct  50188  CACGGTTCCCGAAGGCACTAAGGCATCTCTGCCAAATTCCGTGGATGTCAAGAGTAGGTA  50247

Query  489    AGGTTCTTCGCGTTGCATCGAATTAAACCACATGCTCCACCGCTTGTGCGGGCCCCCGTC  548
              ||||||||||||||||||||||||||||||||||||||||||||||||||||||||||||
Sbjct  50248  AGGTTCTTCGCGTTGCATCGAATTAAACCACATGCTCCACCGCTTGTGCGGGCCCCCGTC  50307

Query  549    AATTCATTTGAGTTTTAACCTTGCGGCCGTACTCCCCAGGCGGTCGACTTAACGCGTTAG  608
              ||||||||||||||||||||||||||||||||||||||||||||||||||||||||||||
Sbjct  50308  AATTCATTTGAGTTTTAACCTTGCGGCCGTACTCCCCAGGCGGTCGACTTAACGCGTTAG  50367

Query  609    CTCCGGAAGCCACTCCTCAAGGGAACAACCTCCAAGTCGACATCGTTTACGGCGTGGACT  668
              ||||||||||||||||||||||||||||||||||||||||||||||||||||||||||||
Sbjct  50368  CTCCGGAAGCCACTCCTCAAGGGAACAACCTCCAAGTCGACATCGTTTACGGCGTGGACT  50427

Query  669    ACCAGGGTATCTAATCCTGTTTGCTCCCCACGCTTTCGCACCTGAGCGTCAGTCTTTGTC  728
              ||||||||||||||||||||||||||||||||||||||||||||||||||||||||||||
Sbjct  50428  ACCAGGGTATCTAATCCTGTTTGCTCCCCACGCTTTCGCACCTGAGCGTCAGTCTTTGTC  50487

Query  729    CAGGGGGCCGCCTTCGCCACCGGTATTCCTCCAGATCTCTACGCATTTCACCGCTACACC  788
              ||||||||||||||||||||||||||||||||||||||||||||||||||||||||||||
Sbjct  50488  CAGGGGGCCGCCTTCGCCACCGGTATTCCTCCAGATCTCTACGCATTTCACCGCTACACC  50547

Query  789    TGGAATTCTACCCCCCTCTACAAGACTCAAGCCTGCCAGTTTCAAATGCAGTTCCCAGGT  848
              ||||||||||||||||||||||||||||||||||||||||||||||||||||||||||||
Sbjct  50548  TGGAATTCTACCCCCCTCTACAAGACTCAAGCCTGCCAGTTTCAAATGCAGTTCCCAGGT  50607

Query  849    TAAGCCCGGGGATTTCACATCTGACTTAACAGACCGCCTGCGTGCGCTTTACGCCCAGTA  908
              ||||||||||||||||||||||||||||||||||||||||||||||||||||||||||||
Sbjct  50608  TAAGCCCGGGGATTTCACATCTGACTTAACAGACCGCCTGCGTGCGCTTTACGCCCAGTA  50667

Query  909    ATTCCGATTAACGCTTGCACCCTCCGTATTACCGCGGCTGCTGGCACGGNGTTAGCCGGT  968
              ||||||||||||||||||||||||||||||||||||||||||||||||| ||||||||||
Sbjct  50668  ATTCCGATTAACGCTTGCACCCTCCGTATTACCGCGGCTGCTGGCACGGAGTTAGCCGGT  50727

Query  969    GCTTCTTCTGCGGGTAACGTCAATCGAC  996
              ||||||||||||||||||||||||||||
Sbjct  50728  GCTTCTTCTGCGGGTAACGTCAATCGAC  50755
```

Range 2: 171036 to 172021GenBankGraphics

Next Match
Previous Match
First Match

Alignment statistics for match #2

| Score | Expect | Identities | Gaps | Strand | Frame |
| --- | --- | --- | --- | --- | --- |
| 1784 bits(966) | 0.0() | 976/986(99%) | 0/986(0%) | Plus/Plus |  |

Features:

```
Query  9       CGCCCTCCCGAAGGNTNNGCTACCTACTTCTTTTNNANCCCACTCCCATGGTGTGACGGG  68
               |||||||||||||| |  ||||||||||||||||  | ||||||||||||||||||||||
Sbjct  171036  CGCCCTCCCGAAGGTTAAGCTACCTACTTCTTTTGCAACCCACTCCCATGGTGTGACGGG  171095

Query  69      CGGTGTGTACAAGGCCCGGGAACGTATTCACCGTGGCATTCTGATCCACGATTACTAGCG  128
               ||||||||||||||||||||||||||||||||||||||||||||||||||||||||||||
Sbjct  171096  CGGTGTGTACAAGGCCCGGGAACGTATTCACCGTGGCATTCTGATCCACGATTACTAGCG  171155

Query  129     ATTCCGACTTCACGGAGTCGAGTTGCAGACTCCGATCCGGACTACGACGCACTTTGTGAG  188
               ||||||||||||||||||||||||||||||||||||||||||||||||||||||||||||
Sbjct  171156  ATTCCGACTTCACGGAGTCGAGTTGCAGACTCCGATCCGGACTACGACGCACTTTGTGAG  171215

Query  189     GTCCGCTTGCTCTCGCGAGGTCGCTTCTCTTTGTATGCGCCATTGTAGCACGTGTGTAGC  248
               ||||||||||||||||||||||||||||||||||||||||||||||||||||||||||||
Sbjct  171216  GTCCGCTTGCTCTCGCGAGGTCGCTTCTCTTTGTATGCGCCATTGTAGCACGTGTGTAGC  171275

Query  249     CCTACTCGTAAGGGCCATGATGACTTGACGTCATCCCCACCTTCCTCCGGTTTATCACCG  308
               ||||||||||||||||||||||||||||||||||||||||||||||||||||||||||||
Sbjct  171276  CCTACTCGTAAGGGCCATGATGACTTGACGTCATCCCCACCTTCCTCCGGTTTATCACCG  171335

Query  309     GCAGTCTCCTTTGAGTTCCCGACCGAATCGCTGGCAACAAAGGATAAGGGTTGCGCTCGT  368
               ||||||||||||||||||||||||||||||||||||||||||||||||||||||||||||
Sbjct  171336  GCAGTCTCCTTTGAGTTCCCGACCGAATCGCTGGCAACAAAGGATAAGGGTTGCGCTCGT  171395

Query  369     TGCGGGACTTAACCCAACATTTCACAACACGAGCTGACGACAGCCATGCAGCACCTGTCT  428
               ||||||||||||||||||||||||||||||||||||||||||||||||||||||||||||
Sbjct  171396  TGCGGGACTTAACCCAACATTTCACAACACGAGCTGACGACAGCCATGCAGCACCTGTCT  171455

Query  429     CACGGTTCCCGAAGGCACTAAGGCATCTCTGNNNAATTCCGTGGATGTCAAGAGTAGGTA  488
               |||||||||||||||||||||||||||||||   ||||||||||||||||||||||||||
Sbjct  171456  CACGGTTCCCGAAGGCACTAAGGCATCTCTGCCAAATTCCGTGGATGTCAAGAGTAGGTA  171515

Query  489     AGGTTCTTCGCGTTGCATCGAATTAAACCACATGCTCCACCGCTTGTGCGGGCCCCCGTC  548
               ||||||||||||||||||||||||||||||||||||||||||||||||||||||||||||
Sbjct  171516  AGGTTCTTCGCGTTGCATCGAATTAAACCACATGCTCCACCGCTTGTGCGGGCCCCCGTC  171575

Query  549     AATTCATTTGAGTTTTAACCTTGCGGCCGTACTCCCCAGGCGGTCGACTTAACGCGTTAG  608
               ||||||||||||||||||||||||||||||||||||||||||||||||||||||||||||
Sbjct  171576  AATTCATTTGAGTTTTAACCTTGCGGCCGTACTCCCCAGGCGGTCGACTTAACGCGTTAG  171635

Query  609     CTCCGGAAGCCACTCCTCAAGGGAACAACCTCCAAGTCGACATCGTTTACGGCGTGGACT  668
               ||||||||||||||||||||||||||||||||||||||||||||||||||||||||||||
Sbjct  171636  CTCCGGAAGCCACTCCTCAAGGGAACAACCTCCAAGTCGACATCGTTTACGGCGTGGACT  171695

Query  669     ACCAGGGTATCTAATCCTGTTTGCTCCCCACGCTTTCGCACCTGAGCGTCAGTCTTTGTC  728
               ||||||||||||||||||||||||||||||||||||||||||||||||||||||||||||
Sbjct  171696  ACCAGGGTATCTAATCCTGTTTGCTCCCCACGCTTTCGCACCTGAGCGTCAGTCTTTGTC  171755

Query  729     CAGGGGGCCGCCTTCGCCACCGGTATTCCTCCAGATCTCTACGCATTTCACCGCTACACC  788
               ||||||||||||||||||||||||||||||||||||||||||||||||||||||||||||
Sbjct  171756  CAGGGGGCCGCCTTCGCCACCGGTATTCCTCCAGATCTCTACGCATTTCACCGCTACACC  171815

Query  789     TGGAATTCTACCCCCCTCTACAAGACTCAAGCCTGCCAGTTTCAAATGCAGTTCCCAGGT  848
               ||||||||||||||||||||||||||||||||||||||||||||||||||||||||||||
Sbjct  171816  TGGAATTCTACCCCCCTCTACAAGACTCAAGCCTGCCAGTTTCAAATGCAGTTCCCAGGT  171875

Query  849     TAAGCCCGGGGATTTCACATCTGACTTAACAGACCGCCTGCGTGCGCTTTACGCCCAGTA  908
               ||||||||||||||||||||||||||||||||||||||||||||||||||||||||||||
Sbjct  171876  TAAGCCCGGGGATTTCACATCTGACTTAACAGACCGCCTGCGTGCGCTTTACGCCCAGTA  171935

Query  909     ATTCCGATTAACGCTTGCACCCTCCGTATTACCGCGGCTGCTGGCACGGNGTTAGCCGGT  968
               ||||||||||||||||||||||||||||||||||||||||||||||||| ||||||||||
Sbjct  171936  ATTCCGATTAACGCTTGCACCCTCCGTATTACCGCGGCTGCTGGCACGGAGTTAGCCGGT  171995

Query  969     GCTTCTTCTGCGGGTAACGTCAATCG  994
               ||||||||||||||||||||||||||
Sbjct  171996  GCTTCTTCTGCGGGTAACGTCAATCG  172021
```

Range 3: 447691 to 448676GenBankGraphics

Next Match
Previous Match
First Match

Alignment statistics for match #3

| Score | Expect | Identities | Gaps | Strand | Frame |
| --- | --- | --- | --- | --- | --- |
| 1784 bits(966) | 0.0() | 976/986(99%) | 0/986(0%) | Plus/Minus |  |

Features:

```
Query  9       CGCCCTCCCGAAGGNTNNGCTACCTACTTCTTTTNNANCCCACTCCCATGGTGTGACGGG  68
               |||||||||||||| |  ||||||||||||||||  | ||||||||||||||||||||||
Sbjct  448676  CGCCCTCCCGAAGGTTAAGCTACCTACTTCTTTTGCAACCCACTCCCATGGTGTGACGGG  448617

Query  69      CGGTGTGTACAAGGCCCGGGAACGTATTCACCGTGGCATTCTGATCCACGATTACTAGCG  128
               ||||||||||||||||||||||||||||||||||||||||||||||||||||||||||||
Sbjct  448616  CGGTGTGTACAAGGCCCGGGAACGTATTCACCGTGGCATTCTGATCCACGATTACTAGCG  448557

Query  129     ATTCCGACTTCACGGAGTCGAGTTGCAGACTCCGATCCGGACTACGACGCACTTTGTGAG  188
               ||||||||||||||||||||||||||||||||||||||||||||||||||||||||||||
Sbjct  448556  ATTCCGACTTCACGGAGTCGAGTTGCAGACTCCGATCCGGACTACGACGCACTTTGTGAG  448497

Query  189     GTCCGCTTGCTCTCGCGAGGTCGCTTCTCTTTGTATGCGCCATTGTAGCACGTGTGTAGC  248
               ||||||||||||||||||||||||||||||||||||||||||||||||||||||||||||
Sbjct  448496  GTCCGCTTGCTCTCGCGAGGTCGCTTCTCTTTGTATGCGCCATTGTAGCACGTGTGTAGC  448437

Query  249     CCTACTCGTAAGGGCCATGATGACTTGACGTCATCCCCACCTTCCTCCGGTTTATCACCG  308
               ||||||||||||||||||||||||||||||||||||||||||||||||||||||||||||
Sbjct  448436  CCTACTCGTAAGGGCCATGATGACTTGACGTCATCCCCACCTTCCTCCGGTTTATCACCG  448377

Query  309     GCAGTCTCCTTTGAGTTCCCGACCGAATCGCTGGCAACAAAGGATAAGGGTTGCGCTCGT  368
               ||||||||||||||||||||||||||||||||||||||||||||||||||||||||||||
Sbjct  448376  GCAGTCTCCTTTGAGTTCCCGACCGAATCGCTGGCAACAAAGGATAAGGGTTGCGCTCGT  448317

Query  369     TGCGGGACTTAACCCAACATTTCACAACACGAGCTGACGACAGCCATGCAGCACCTGTCT  428
               ||||||||||||||||||||||||||||||||||||||||||||||||||||||||||||
Sbjct  448316  TGCGGGACTTAACCCAACATTTCACAACACGAGCTGACGACAGCCATGCAGCACCTGTCT  448257

Query  429     CACGGTTCCCGAAGGCACTAAGGCATCTCTGNNNAATTCCGTGGATGTCAAGAGTAGGTA  488
               |||||||||||||||||||||||||||||||   ||||||||||||||||||||||||||
Sbjct  448256  CACGGTTCCCGAAGGCACTAAGGCATCTCTGCCAAATTCCGTGGATGTCAAGAGTAGGTA  448197

Query  489     AGGTTCTTCGCGTTGCATCGAATTAAACCACATGCTCCACCGCTTGTGCGGGCCCCCGTC  548
               ||||||||||||||||||||||||||||||||||||||||||||||||||||||||||||
Sbjct  448196  AGGTTCTTCGCGTTGCATCGAATTAAACCACATGCTCCACCGCTTGTGCGGGCCCCCGTC  448137

Query  549     AATTCATTTGAGTTTTAACCTTGCGGCCGTACTCCCCAGGCGGTCGACTTAACGCGTTAG  608
               ||||||||||||||||||||||||||||||||||||||||||||||||||||||||||||
Sbjct  448136  AATTCATTTGAGTTTTAACCTTGCGGCCGTACTCCCCAGGCGGTCGACTTAACGCGTTAG  448077

Query  609     CTCCGGAAGCCACTCCTCAAGGGAACAACCTCCAAGTCGACATCGTTTACGGCGTGGACT  668
               ||||||||||||||||||||||||||||||||||||||||||||||||||||||||||||
Sbjct  448076  CTCCGGAAGCCACTCCTCAAGGGAACAACCTCCAAGTCGACATCGTTTACGGCGTGGACT  448017

Query  669     ACCAGGGTATCTAATCCTGTTTGCTCCCCACGCTTTCGCACCTGAGCGTCAGTCTTTGTC  728
               ||||||||||||||||||||||||||||||||||||||||||||||||||||||||||||
Sbjct  448016  ACCAGGGTATCTAATCCTGTTTGCTCCCCACGCTTTCGCACCTGAGCGTCAGTCTTTGTC  447957

Query  729     CAGGGGGCCGCCTTCGCCACCGGTATTCCTCCAGATCTCTACGCATTTCACCGCTACACC  788
               ||||||||||||||||||||||||||||||||||||||||||||||||||||||||||||
Sbjct  447956  CAGGGGGCCGCCTTCGCCACCGGTATTCCTCCAGATCTCTACGCATTTCACCGCTACACC  447897

Query  789     TGGAATTCTACCCCCCTCTACAAGACTCAAGCCTGCCAGTTTCAAATGCAGTTCCCAGGT  848
               ||||||||||||||||||||||||||||||||||||||||||||||||||||||||||||
Sbjct  447896  TGGAATTCTACCCCCCTCTACAAGACTCAAGCCTGCCAGTTTCAAATGCAGTTCCCAGGT  447837

Query  849     TAAGCCCGGGGATTTCACATCTGACTTAACAGACCGCCTGCGTGCGCTTTACGCCCAGTA  908
               ||||||||||||||||||||||||||||||||||||||||||||||||||||||||||||
Sbjct  447836  TAAGCCCGGGGATTTCACATCTGACTTAACAGACCGCCTGCGTGCGCTTTACGCCCAGTA  447777

Query  909     ATTCCGATTAACGCTTGCACCCTCCGTATTACCGCGGCTGCTGGCACGGNGTTAGCCGGT  968
               ||||||||||||||||||||||||||||||||||||||||||||||||| ||||||||||
Sbjct  447776  ATTCCGATTAACGCTTGCACCCTCCGTATTACCGCGGCTGCTGGCACGGAGTTAGCCGGT  447717

Query  969     GCTTCTTCTGCGGGTAACGTCAATCG  994
               ||||||||||||||||||||||||||
Sbjct  447716  GCTTCTTCTGCGGGTAACGTCAATCG  447691
```

Range 4: 1038308 to 1039293GenBankGraphics

Next Match
Previous Match
First Match

Alignment statistics for match #4

| Score | Expect | Identities | Gaps | Strand | Frame |
| --- | --- | --- | --- | --- | --- |
| 1784 bits(966) | 0.0() | 976/986(99%) | 0/986(0%) | Plus/Minus |  |

Features:

```
Query  9        CGCCCTCCCGAAGGNTNNGCTACCTACTTCTTTTNNANCCCACTCCCATGGTGTGACGGG  68
                |||||||||||||| |  ||||||||||||||||  | ||||||||||||||||||||||
Sbjct  1039293  CGCCCTCCCGAAGGTTAAGCTACCTACTTCTTTTGCAACCCACTCCCATGGTGTGACGGG  1039234

Query  69       CGGTGTGTACAAGGCCCGGGAACGTATTCACCGTGGCATTCTGATCCACGATTACTAGCG  128
                ||||||||||||||||||||||||||||||||||||||||||||||||||||||||||||
Sbjct  1039233  CGGTGTGTACAAGGCCCGGGAACGTATTCACCGTGGCATTCTGATCCACGATTACTAGCG  1039174

Query  129      ATTCCGACTTCACGGAGTCGAGTTGCAGACTCCGATCCGGACTACGACGCACTTTGTGAG  188
                ||||||||||||||||||||||||||||||||||||||||||||||||||||||||||||
Sbjct  1039173  ATTCCGACTTCACGGAGTCGAGTTGCAGACTCCGATCCGGACTACGACGCACTTTGTGAG  1039114

Query  189      GTCCGCTTGCTCTCGCGAGGTCGCTTCTCTTTGTATGCGCCATTGTAGCACGTGTGTAGC  248
                ||||||||||||||||||||||||||||||||||||||||||||||||||||||||||||
Sbjct  1039113  GTCCGCTTGCTCTCGCGAGGTCGCTTCTCTTTGTATGCGCCATTGTAGCACGTGTGTAGC  1039054

Query  249      CCTACTCGTAAGGGCCATGATGACTTGACGTCATCCCCACCTTCCTCCGGTTTATCACCG  308
                ||||||||||||||||||||||||||||||||||||||||||||||||||||||||||||
Sbjct  1039053  CCTACTCGTAAGGGCCATGATGACTTGACGTCATCCCCACCTTCCTCCGGTTTATCACCG  1038994

Query  309      GCAGTCTCCTTTGAGTTCCCGACCGAATCGCTGGCAACAAAGGATAAGGGTTGCGCTCGT  368
                ||||||||||||||||||||||||||||||||||||||||||||||||||||||||||||
Sbjct  1038993  GCAGTCTCCTTTGAGTTCCCGACCGAATCGCTGGCAACAAAGGATAAGGGTTGCGCTCGT  1038934

Query  369      TGCGGGACTTAACCCAACATTTCACAACACGAGCTGACGACAGCCATGCAGCACCTGTCT  428
                ||||||||||||||||||||||||||||||||||||||||||||||||||||||||||||
Sbjct  1038933  TGCGGGACTTAACCCAACATTTCACAACACGAGCTGACGACAGCCATGCAGCACCTGTCT  1038874

Query  429      CACGGTTCCCGAAGGCACTAAGGCATCTCTGNNNAATTCCGTGGATGTCAAGAGTAGGTA  488
                |||||||||||||||||||||||||||||||   ||||||||||||||||||||||||||
Sbjct  1038873  CACGGTTCCCGAAGGCACTAAGGCATCTCTGCCAAATTCCGTGGATGTCAAGAGTAGGTA  1038814

Query  489      AGGTTCTTCGCGTTGCATCGAATTAAACCACATGCTCCACCGCTTGTGCGGGCCCCCGTC  548
                ||||||||||||||||||||||||||||||||||||||||||||||||||||||||||||
Sbjct  1038813  AGGTTCTTCGCGTTGCATCGAATTAAACCACATGCTCCACCGCTTGTGCGGGCCCCCGTC  1038754

Query  549      AATTCATTTGAGTTTTAACCTTGCGGCCGTACTCCCCAGGCGGTCGACTTAACGCGTTAG  608
                ||||||||||||||||||||||||||||||||||||||||||||||||||||||||||||
Sbjct  1038753  AATTCATTTGAGTTTTAACCTTGCGGCCGTACTCCCCAGGCGGTCGACTTAACGCGTTAG  1038694

Query  609      CTCCGGAAGCCACTCCTCAAGGGAACAACCTCCAAGTCGACATCGTTTACGGCGTGGACT  668
                ||||||||||||||||||||||||||||||||||||||||||||||||||||||||||||
Sbjct  1038693  CTCCGGAAGCCACTCCTCAAGGGAACAACCTCCAAGTCGACATCGTTTACGGCGTGGACT  1038634

Query  669      ACCAGGGTATCTAATCCTGTTTGCTCCCCACGCTTTCGCACCTGAGCGTCAGTCTTTGTC  728
                ||||||||||||||||||||||||||||||||||||||||||||||||||||||||||||
Sbjct  1038633  ACCAGGGTATCTAATCCTGTTTGCTCCCCACGCTTTCGCACCTGAGCGTCAGTCTTTGTC  1038574

Query  729      CAGGGGGCCGCCTTCGCCACCGGTATTCCTCCAGATCTCTACGCATTTCACCGCTACACC  788
                ||||||||||||||||||||||||||||||||||||||||||||||||||||||||||||
Sbjct  1038573  CAGGGGGCCGCCTTCGCCACCGGTATTCCTCCAGATCTCTACGCATTTCACCGCTACACC  1038514

Query  789      TGGAATTCTACCCCCCTCTACAAGACTCAAGCCTGCCAGTTTCAAATGCAGTTCCCAGGT  848
                ||||||||||||||||||||||||||||||||||||||||||||||||||||||||||||
Sbjct  1038513  TGGAATTCTACCCCCCTCTACAAGACTCAAGCCTGCCAGTTTCAAATGCAGTTCCCAGGT  1038454

Query  849      TAAGCCCGGGGATTTCACATCTGACTTAACAGACCGCCTGCGTGCGCTTTACGCCCAGTA  908
                ||||||||||||||||||||||||||||||||||||||||||||||||||||||||||||
Sbjct  1038453  TAAGCCCGGGGATTTCACATCTGACTTAACAGACCGCCTGCGTGCGCTTTACGCCCAGTA  1038394

Query  909      ATTCCGATTAACGCTTGCACCCTCCGTATTACCGCGGCTGCTGGCACGGNGTTAGCCGGT  968
                ||||||||||||||||||||||||||||||||||||||||||||||||| ||||||||||
Sbjct  1038393  ATTCCGATTAACGCTTGCACCCTCCGTATTACCGCGGCTGCTGGCACGGAGTTAGCCGGT  1038334

Query  969      GCTTCTTCTGCGGGTAACGTCAATCG  994
                ||||||||||||||||||||||||||
Sbjct  1038333  GCTTCTTCTGCGGGTAACGTCAATCG  1038308
```

Range 5: 3350388 to 3351373GenBankGraphics

Next Match
Previous Match
First Match

Alignment statistics for match #5

| Score | Expect | Identities | Gaps | Strand | Frame |
| --- | --- | --- | --- | --- | --- |
| 1784 bits(966) | 0.0() | 976/986(99%) | 0/986(0%) | Plus/Plus |  |

Features:

```
Query  9        CGCCCTCCCGAAGGNTNNGCTACCTACTTCTTTTNNANCCCACTCCCATGGTGTGACGGG  68
                |||||||||||||| |  ||||||||||||||||  | ||||||||||||||||||||||
Sbjct  3350388  CGCCCTCCCGAAGGTTAAGCTACCTACTTCTTTTGCAACCCACTCCCATGGTGTGACGGG  3350447

Query  69       CGGTGTGTACAAGGCCCGGGAACGTATTCACCGTGGCATTCTGATCCACGATTACTAGCG  128
                ||||||||||||||||||||||||||||||||||||||||||||||||||||||||||||
Sbjct  3350448  CGGTGTGTACAAGGCCCGGGAACGTATTCACCGTGGCATTCTGATCCACGATTACTAGCG  3350507

Query  129      ATTCCGACTTCACGGAGTCGAGTTGCAGACTCCGATCCGGACTACGACGCACTTTGTGAG  188
                ||||||||||||||||||||||||||||||||||||||||||||||||||||||||||||
Sbjct  3350508  ATTCCGACTTCACGGAGTCGAGTTGCAGACTCCGATCCGGACTACGACGCACTTTGTGAG  3350567

Query  189      GTCCGCTTGCTCTCGCGAGGTCGCTTCTCTTTGTATGCGCCATTGTAGCACGTGTGTAGC  248
                ||||||||||||||||||||||||||||||||||||||||||||||||||||||||||||
Sbjct  3350568  GTCCGCTTGCTCTCGCGAGGTCGCTTCTCTTTGTATGCGCCATTGTAGCACGTGTGTAGC  3350627

Query  249      CCTACTCGTAAGGGCCATGATGACTTGACGTCATCCCCACCTTCCTCCGGTTTATCACCG  308
                ||||||||||||||||||||||||||||||||||||||||||||||||||||||||||||
Sbjct  3350628  CCTACTCGTAAGGGCCATGATGACTTGACGTCATCCCCACCTTCCTCCGGTTTATCACCG  3350687

Query  309      GCAGTCTCCTTTGAGTTCCCGACCGAATCGCTGGCAACAAAGGATAAGGGTTGCGCTCGT  368
                ||||||||||||||||||||||||||||||||||||||||||||||||||||||||||||
Sbjct  3350688  GCAGTCTCCTTTGAGTTCCCGACCGAATCGCTGGCAACAAAGGATAAGGGTTGCGCTCGT  3350747

Query  369      TGCGGGACTTAACCCAACATTTCACAACACGAGCTGACGACAGCCATGCAGCACCTGTCT  428
                ||||||||||||||||||||||||||||||||||||||||||||||||||||||||||||
Sbjct  3350748  TGCGGGACTTAACCCAACATTTCACAACACGAGCTGACGACAGCCATGCAGCACCTGTCT  3350807

Query  429      CACGGTTCCCGAAGGCACTAAGGCATCTCTGNNNAATTCCGTGGATGTCAAGAGTAGGTA  488
                |||||||||||||||||||||||||||||||   ||||||||||||||||||||||||||
Sbjct  3350808  CACGGTTCCCGAAGGCACTAAGGCATCTCTGCCGAATTCCGTGGATGTCAAGAGTAGGTA  3350867

Query  489      AGGTTCTTCGCGTTGCATCGAATTAAACCACATGCTCCACCGCTTGTGCGGGCCCCCGTC  548
                ||||||||||||||||||||||||||||||||||||||||||||||||||||||||||||
Sbjct  3350868  AGGTTCTTCGCGTTGCATCGAATTAAACCACATGCTCCACCGCTTGTGCGGGCCCCCGTC  3350927

Query  549      AATTCATTTGAGTTTTAACCTTGCGGCCGTACTCCCCAGGCGGTCGACTTAACGCGTTAG  608
                ||||||||||||||||||||||||||||||||||||||||||||||||||||||||||||
Sbjct  3350928  AATTCATTTGAGTTTTAACCTTGCGGCCGTACTCCCCAGGCGGTCGACTTAACGCGTTAG  3350987

Query  609      CTCCGGAAGCCACTCCTCAAGGGAACAACCTCCAAGTCGACATCGTTTACGGCGTGGACT  668
                ||||||||||||||||||||||||||||||||||||||||||||||||||||||||||||
Sbjct  3350988  CTCCGGAAGCCACTCCTCAAGGGAACAACCTCCAAGTCGACATCGTTTACGGCGTGGACT  3351047

Query  669      ACCAGGGTATCTAATCCTGTTTGCTCCCCACGCTTTCGCACCTGAGCGTCAGTCTTTGTC  728
                ||||||||||||||||||||||||||||||||||||||||||||||||||||||||||||
Sbjct  3351048  ACCAGGGTATCTAATCCTGTTTGCTCCCCACGCTTTCGCACCTGAGCGTCAGTCTTTGTC  3351107

Query  729      CAGGGGGCCGCCTTCGCCACCGGTATTCCTCCAGATCTCTACGCATTTCACCGCTACACC  788
                ||||||||||||||||||||||||||||||||||||||||||||||||||||||||||||
Sbjct  3351108  CAGGGGGCCGCCTTCGCCACCGGTATTCCTCCAGATCTCTACGCATTTCACCGCTACACC  3351167

Query  789      TGGAATTCTACCCCCCTCTACAAGACTCAAGCCTGCCAGTTTCAAATGCAGTTCCCAGGT  848
                ||||||||||||||||||||||||||||||||||||||||||||||||||||||||||||
Sbjct  3351168  TGGAATTCTACCCCCCTCTACAAGACTCAAGCCTGCCAGTTTCAAATGCAGTTCCCAGGT  3351227

Query  849      TAAGCCCGGGGATTTCACATCTGACTTAACAGACCGCCTGCGTGCGCTTTACGCCCAGTA  908
                ||||||||||||||||||||||||||||||||||||||||||||||||||||||||||||
Sbjct  3351228  TAAGCCCGGGGATTTCACATCTGACTTAACAGACCGCCTGCGTGCGCTTTACGCCCAGTA  3351287

Query  909      ATTCCGATTAACGCTTGCACCCTCCGTATTACCGCGGCTGCTGGCACGGNGTTAGCCGGT  968
                ||||||||||||||||||||||||||||||||||||||||||||||||| ||||||||||
Sbjct  3351288  ATTCCGATTAACGCTTGCACCCTCCGTATTACCGCGGCTGCTGGCACGGAGTTAGCCGGT  3351347

Query  969      GCTTCTTCTGCGGGTAACGTCAATCG  994
                ||||||||||||||||||||||||||
Sbjct  3351348  GCTTCTTCTGCGGGTAACGTCAATCG  3351373
```

Range 6: 3833145 to 3834130GenBankGraphics

Next Match
Previous Match
First Match

Alignment statistics for match #6

| Score | Expect | Identities | Gaps | Strand | Frame |
| --- | --- | --- | --- | --- | --- |
| 1784 bits(966) | 0.0() | 976/986(99%) | 0/986(0%) | Plus/Plus |  |

Features:

```
Query  9        CGCCCTCCCGAAGGNTNNGCTACCTACTTCTTTTNNANCCCACTCCCATGGTGTGACGGG  68
                |||||||||||||| |  ||||||||||||||||  | ||||||||||||||||||||||
Sbjct  3833145  CGCCCTCCCGAAGGTTAAGCTACCTACTTCTTTTGCAACCCACTCCCATGGTGTGACGGG  3833204

Query  69       CGGTGTGTACAAGGCCCGGGAACGTATTCACCGTGGCATTCTGATCCACGATTACTAGCG  128
                ||||||||||||||||||||||||||||||||||||||||||||||||||||||||||||
Sbjct  3833205  CGGTGTGTACAAGGCCCGGGAACGTATTCACCGTGGCATTCTGATCCACGATTACTAGCG  3833264

Query  129      ATTCCGACTTCACGGAGTCGAGTTGCAGACTCCGATCCGGACTACGACGCACTTTGTGAG  188
                ||||||||||||||||||||||||||||||||||||||||||||||||||||||||||||
Sbjct  3833265  ATTCCGACTTCACGGAGTCGAGTTGCAGACTCCGATCCGGACTACGACGCACTTTGTGAG  3833324

Query  189      GTCCGCTTGCTCTCGCGAGGTCGCTTCTCTTTGTATGCGCCATTGTAGCACGTGTGTAGC  248
                ||||||||||||||||||||||||||||||||||||||||||||||||||||||||||||
Sbjct  3833325  GTCCGCTTGCTCTCGCGAGGTCGCTTCTCTTTGTATGCGCCATTGTAGCACGTGTGTAGC  3833384

Query  249      CCTACTCGTAAGGGCCATGATGACTTGACGTCATCCCCACCTTCCTCCGGTTTATCACCG  308
                ||||||||||||||||||||||||||||||||||||||||||||||||||||||||||||
Sbjct  3833385  CCTACTCGTAAGGGCCATGATGACTTGACGTCATCCCCACCTTCCTCCGGTTTATCACCG  3833444

Query  309      GCAGTCTCCTTTGAGTTCCCGACCGAATCGCTGGCAACAAAGGATAAGGGTTGCGCTCGT  368
                ||||||||||||||||||||||||||||||||||||||||||||||||||||||||||||
Sbjct  3833445  GCAGTCTCCTTTGAGTTCCCGACCGAATCGCTGGCAACAAAGGATAAGGGTTGCGCTCGT  3833504

Query  369      TGCGGGACTTAACCCAACATTTCACAACACGAGCTGACGACAGCCATGCAGCACCTGTCT  428
                ||||||||||||||||||||||||||||||||||||||||||||||||||||||||||||
Sbjct  3833505  TGCGGGACTTAACCCAACATTTCACAACACGAGCTGACGACAGCCATGCAGCACCTGTCT  3833564

Query  429      CACGGTTCCCGAAGGCACTAAGGCATCTCTGNNNAATTCCGTGGATGTCAAGAGTAGGTA  488
                |||||||||||||||||||||||||||||||   ||||||||||||||||||||||||||
Sbjct  3833565  CACGGTTCCCGAAGGCACTAAGGCATCTCTGCCGAATTCCGTGGATGTCAAGAGTAGGTA  3833624

Query  489      AGGTTCTTCGCGTTGCATCGAATTAAACCACATGCTCCACCGCTTGTGCGGGCCCCCGTC  548
                ||||||||||||||||||||||||||||||||||||||||||||||||||||||||||||
Sbjct  3833625  AGGTTCTTCGCGTTGCATCGAATTAAACCACATGCTCCACCGCTTGTGCGGGCCCCCGTC  3833684

Query  549      AATTCATTTGAGTTTTAACCTTGCGGCCGTACTCCCCAGGCGGTCGACTTAACGCGTTAG  608
                ||||||||||||||||||||||||||||||||||||||||||||||||||||||||||||
Sbjct  3833685  AATTCATTTGAGTTTTAACCTTGCGGCCGTACTCCCCAGGCGGTCGACTTAACGCGTTAG  3833744

Query  609      CTCCGGAAGCCACTCCTCAAGGGAACAACCTCCAAGTCGACATCGTTTACGGCGTGGACT  668
                ||||||||||||||||||||||||||||||||||||||||||||||||||||||||||||
Sbjct  3833745  CTCCGGAAGCCACTCCTCAAGGGAACAACCTCCAAGTCGACATCGTTTACGGCGTGGACT  3833804

Query  669      ACCAGGGTATCTAATCCTGTTTGCTCCCCACGCTTTCGCACCTGAGCGTCAGTCTTTGTC  728
                ||||||||||||||||||||||||||||||||||||||||||||||||||||||||||||
Sbjct  3833805  ACCAGGGTATCTAATCCTGTTTGCTCCCCACGCTTTCGCACCTGAGCGTCAGTCTTTGTC  3833864

Query  729      CAGGGGGCCGCCTTCGCCACCGGTATTCCTCCAGATCTCTACGCATTTCACCGCTACACC  788
                ||||||||||||||||||||||||||||||||||||||||||||||||||||||||||||
Sbjct  3833865  CAGGGGGCCGCCTTCGCCACCGGTATTCCTCCAGATCTCTACGCATTTCACCGCTACACC  3833924

Query  789      TGGAATTCTACCCCCCTCTACAAGACTCAAGCCTGCCAGTTTCAAATGCAGTTCCCAGGT  848
                ||||||||||||||||||||||||||||||||||||||||||||||||||||||||||||
Sbjct  3833925  TGGAATTCTACCCCCCTCTACAAGACTCAAGCCTGCCAGTTTCAAATGCAGTTCCCAGGT  3833984

Query  849      TAAGCCCGGGGATTTCACATCTGACTTAACAGACCGCCTGCGTGCGCTTTACGCCCAGTA  908
                ||||||||||||||||||||||||||||||||||||||||||||||||||||||||||||
Sbjct  3833985  TAAGCCCGGGGATTTCACATCTGACTTAACAGACCGCCTGCGTGCGCTTTACGCCCAGTA  3834044

Query  909      ATTCCGATTAACGCTTGCACCCTCCGTATTACCGCGGCTGCTGGCACGGNGTTAGCCGGT  968
                ||||||||||||||||||||||||||||||||||||||||||||||||| ||||||||||
Sbjct  3834045  ATTCCGATTAACGCTTGCACCCTCCGTATTACCGCGGCTGCTGGCACGGAGTTAGCCGGT  3834104

Query  969      GCTTCTTCTGCGGGTAACGTCAATCG  994
                ||||||||||||||||||||||||||
Sbjct  3834105  GCTTCTTCTGCGGGTAACGTCAATCG  3834130
```

Range 7: 410982 to 411967GenBankGraphics

Next Match
Previous Match
First Match

Alignment statistics for match #7

| Score | Expect | Identities | Gaps | Strand | Frame |
| --- | --- | --- | --- | --- | --- |
| 1779 bits(963) | 0.0() | 975/986(99%) | 0/986(0%) | Plus/Minus |  |

Features:

```
Query  9       CGCCCTCCCGAAGGNTNNGCTACCTACTTCTTTTNNANCCCACTCCCATGGTGTGACGGG  68
               |||||||||||||| |  ||||||||||||||||  | ||||||||||||||||||||||
Sbjct  411967  CGCCCTCCCGAAGGTTAAGCTACCTACTTCTTTTGCAACCCACTCCCATGGTGTGACGGG  411908

Query  69      CGGTGTGTACAAGGCCCGGGAACGTATTCACCGTGGCATTCTGATCCACGATTACTAGCG  128
               ||||||||||||||||||||||||||||||||||||||||||||||||||||||||||||
Sbjct  411907  CGGTGTGTACAAGGCCCGGGAACGTATTCACCGTGGCATTCTGATCCACGATTACTAGCG  411848

Query  129     ATTCCGACTTCACGGAGTCGAGTTGCAGACTCCGATCCGGACTACGACGCACTTTGTGAG  188
               ||||||||||||||||||||||||||||||||||||||||||||||| ||||||||||||
Sbjct  411847  ATTCCGACTTCACGGAGTCGAGTTGCAGACTCCGATCCGGACTACGATGCACTTTGTGAG  411788

Query  189     GTCCGCTTGCTCTCGCGAGGTCGCTTCTCTTTGTATGCGCCATTGTAGCACGTGTGTAGC  248
               ||||||||||||||||||||||||||||||||||||||||||||||||||||||||||||
Sbjct  411787  GTCCGCTTGCTCTCGCGAGGTCGCTTCTCTTTGTATGCGCCATTGTAGCACGTGTGTAGC  411728

Query  249     CCTACTCGTAAGGGCCATGATGACTTGACGTCATCCCCACCTTCCTCCGGTTTATCACCG  308
               ||||||||||||||||||||||||||||||||||||||||||||||||||||||||||||
Sbjct  411727  CCTACTCGTAAGGGCCATGATGACTTGACGTCATCCCCACCTTCCTCCGGTTTATCACCG  411668

Query  309     GCAGTCTCCTTTGAGTTCCCGACCGAATCGCTGGCAACAAAGGATAAGGGTTGCGCTCGT  368
               ||||||||||||||||||||||||||||||||||||||||||||||||||||||||||||
Sbjct  411667  GCAGTCTCCTTTGAGTTCCCGACCGAATCGCTGGCAACAAAGGATAAGGGTTGCGCTCGT  411608

Query  369     TGCGGGACTTAACCCAACATTTCACAACACGAGCTGACGACAGCCATGCAGCACCTGTCT  428
               ||||||||||||||||||||||||||||||||||||||||||||||||||||||||||||
Sbjct  411607  TGCGGGACTTAACCCAACATTTCACAACACGAGCTGACGACAGCCATGCAGCACCTGTCT  411548

Query  429     CACGGTTCCCGAAGGCACTAAGGCATCTCTGNNNAATTCCGTGGATGTCAAGAGTAGGTA  488
               |||||||||||||||||||||||||||||||   ||||||||||||||||||||||||||
Sbjct  411547  CACGGTTCCCGAAGGCACTAAGGCATCTCTGCCAAATTCCGTGGATGTCAAGAGTAGGTA  411488

Query  489     AGGTTCTTCGCGTTGCATCGAATTAAACCACATGCTCCACCGCTTGTGCGGGCCCCCGTC  548
               ||||||||||||||||||||||||||||||||||||||||||||||||||||||||||||
Sbjct  411487  AGGTTCTTCGCGTTGCATCGAATTAAACCACATGCTCCACCGCTTGTGCGGGCCCCCGTC  411428

Query  549     AATTCATTTGAGTTTTAACCTTGCGGCCGTACTCCCCAGGCGGTCGACTTAACGCGTTAG  608
               ||||||||||||||||||||||||||||||||||||||||||||||||||||||||||||
Sbjct  411427  AATTCATTTGAGTTTTAACCTTGCGGCCGTACTCCCCAGGCGGTCGACTTAACGCGTTAG  411368

Query  609     CTCCGGAAGCCACTCCTCAAGGGAACAACCTCCAAGTCGACATCGTTTACGGCGTGGACT  668
               ||||||||||||||||||||||||||||||||||||||||||||||||||||||||||||
Sbjct  411367  CTCCGGAAGCCACTCCTCAAGGGAACAACCTCCAAGTCGACATCGTTTACGGCGTGGACT  411308

Query  669     ACCAGGGTATCTAATCCTGTTTGCTCCCCACGCTTTCGCACCTGAGCGTCAGTCTTTGTC  728
               ||||||||||||||||||||||||||||||||||||||||||||||||||||||||||||
Sbjct  411307  ACCAGGGTATCTAATCCTGTTTGCTCCCCACGCTTTCGCACCTGAGCGTCAGTCTTTGTC  411248

Query  729     CAGGGGGCCGCCTTCGCCACCGGTATTCCTCCAGATCTCTACGCATTTCACCGCTACACC  788
               ||||||||||||||||||||||||||||||||||||||||||||||||||||||||||||
Sbjct  411247  CAGGGGGCCGCCTTCGCCACCGGTATTCCTCCAGATCTCTACGCATTTCACCGCTACACC  411188

Query  789     TGGAATTCTACCCCCCTCTACAAGACTCAAGCCTGCCAGTTTCAAATGCAGTTCCCAGGT  848
               ||||||||||||||||||||||||||||||||||||||||||||||||||||||||||||
Sbjct  411187  TGGAATTCTACCCCCCTCTACAAGACTCAAGCCTGCCAGTTTCAAATGCAGTTCCCAGGT  411128

Query  849     TAAGCCCGGGGATTTCACATCTGACTTAACAGACCGCCTGCGTGCGCTTTACGCCCAGTA  908
               ||||||||||||||||||||||||||||||||||||||||||||||||||||||||||||
Sbjct  411127  TAAGCCCGGGGATTTCACATCTGACTTAACAGACCGCCTGCGTGCGCTTTACGCCCAGTA  411068

Query  909     ATTCCGATTAACGCTTGCACCCTCCGTATTACCGCGGCTGCTGGCACGGNGTTAGCCGGT  968
               ||||||||||||||||||||||||||||||||||||||||||||||||| ||||||||||
Sbjct  411067  ATTCCGATTAACGCTTGCACCCTCCGTATTACCGCGGCTGCTGGCACGGAGTTAGCCGGT  411008

Query  969     GCTTCTTCTGCGGGTAACGTCAATCG  994
               ||||||||||||||||||||||||||
Sbjct  411007  GCTTCTTCTGCGGGTAACGTCAATCG  410982
```

Download

FASTA (complete sequence)

FASTA (aligned sequences)

GenBank (complete sequence)

Continue
Cancel

GenBankGraphics

Next
Previous
Descriptions

Pantoea agglomerans strain AL14 16S ribosomal RNA gene, partial sequence

Sequence ID: MG819218.1Length: 1416Number of Matches: 1

Related Information

Range 1: 385 to 1372GenBankGraphics

Next Match
Previous Match
First Match

Alignment statistics for match #1

| Score | Expect | Identities | Gaps | Strand | Frame |
| --- | --- | --- | --- | --- | --- |
| 1788 bits(968) | 0.0() | 978/988(99%) | 0/988(0%) | Plus/Minus |  |

Features:

```
Query  9     CGCCCTCCCGAAGGNTNNGCTACCTACTTCTTTTNNANCCCACTCCCATGGTGTGACGGG  68
             |||||||||||||| |  ||||||||||||||||  | ||||||||||||||||||||||
Sbjct  1372  CGCCCTCCCGAAGGTTAAGCTACCTACTTCTTTTGCAACCCACTCCCATGGTGTGACGGG  1313

Query  69    CGGTGTGTACAAGGCCCGGGAACGTATTCACCGTGGCATTCTGATCCACGATTACTAGCG  128
             ||||||||||||||||||||||||||||||||||||||||||||||||||||||||||||
Sbjct  1312  CGGTGTGTACAAGGCCCGGGAACGTATTCACCGTGGCATTCTGATCCACGATTACTAGCG  1253

Query  129   ATTCCGACTTCACGGAGTCGAGTTGCAGACTCCGATCCGGACTACGACGCACTTTGTGAG  188
             ||||||||||||||||||||||||||||||||||||||||||||||||||||||||||||
Sbjct  1252  ATTCCGACTTCACGGAGTCGAGTTGCAGACTCCGATCCGGACTACGACGCACTTTGTGAG  1193

Query  189   GTCCGCTTGCTCTCGCGAGGTCGCTTCTCTTTGTATGCGCCATTGTAGCACGTGTGTAGC  248
             ||||||||||||||||||||||||||||||||||||||||||||||||||||||||||||
Sbjct  1192  GTCCGCTTGCTCTCGCGAGGTCGCTTCTCTTTGTATGCGCCATTGTAGCACGTGTGTAGC  1133

Query  249   CCTACTCGTAAGGGCCATGATGACTTGACGTCATCCCCACCTTCCTCCGGTTTATCACCG  308
             ||||||||||||||||||||||||||||||||||||||||||||||||||||||||||||
Sbjct  1132  CCTACTCGTAAGGGCCATGATGACTTGACGTCATCCCCACCTTCCTCCGGTTTATCACCG  1073

Query  309   GCAGTCTCCTTTGAGTTCCCGACCGAATCGCTGGCAACAAAGGATAAGGGTTGCGCTCGT  368
             ||||||||||||||||||||||||||||||||||||||||||||||||||||||||||||
Sbjct  1072  GCAGTCTCCTTTGAGTTCCCGACCGAATCGCTGGCAACAAAGGATAAGGGTTGCGCTCGT  1013

Query  369   TGCGGGACTTAACCCAACATTTCACAACACGAGCTGACGACAGCCATGCAGCACCTGTCT  428
             ||||||||||||||||||||||||||||||||||||||||||||||||||||||||||||
Sbjct  1012  TGCGGGACTTAACCCAACATTTCACAACACGAGCTGACGACAGCCATGCAGCACCTGTCT  953

Query  429   CACGGTTCCCGAAGGCACTAAGGCATCTCTGNNNAATTCCGTGGATGTCAAGAGTAGGTA  488
             |||||||||||||||||||||||||||||||   ||||||||||||||||||||||||||
Sbjct  952   CACGGTTCCCGAAGGCACTAAGGCATCTCTGCCAAATTCCGTGGATGTCAAGAGTAGGTA  893

Query  489   AGGTTCTTCGCGTTGCATCGAATTAAACCACATGCTCCACCGCTTGTGCGGGCCCCCGTC  548
             ||||||||||||||||||||||||||||||||||||||||||||||||||||||||||||
Sbjct  892   AGGTTCTTCGCGTTGCATCGAATTAAACCACATGCTCCACCGCTTGTGCGGGCCCCCGTC  833

Query  549   AATTCATTTGAGTTTTAACCTTGCGGCCGTACTCCCCAGGCGGTCGACTTAACGCGTTAG  608
             ||||||||||||||||||||||||||||||||||||||||||||||||||||||||||||
Sbjct  832   AATTCATTTGAGTTTTAACCTTGCGGCCGTACTCCCCAGGCGGTCGACTTAACGCGTTAG  773

Query  609   CTCCGGAAGCCACTCCTCAAGGGAACAACCTCCAAGTCGACATCGTTTACGGCGTGGACT  668
             ||||||||||||||||||||||||||||||||||||||||||||||||||||||||||||
Sbjct  772   CTCCGGAAGCCACTCCTCAAGGGAACAACCTCCAAGTCGACATCGTTTACGGCGTGGACT  713

Query  669   ACCAGGGTATCTAATCCTGTTTGCTCCCCACGCTTTCGCACCTGAGCGTCAGTCTTTGTC  728
             ||||||||||||||||||||||||||||||||||||||||||||||||||||||||||||
Sbjct  712   ACCAGGGTATCTAATCCTGTTTGCTCCCCACGCTTTCGCACCTGAGCGTCAGTCTTTGTC  653

Query  729   CAGGGGGCCGCCTTCGCCACCGGTATTCCTCCAGATCTCTACGCATTTCACCGCTACACC  788
             ||||||||||||||||||||||||||||||||||||||||||||||||||||||||||||
Sbjct  652   CAGGGGGCCGCCTTCGCCACCGGTATTCCTCCAGATCTCTACGCATTTCACCGCTACACC  593

Query  789   TGGAATTCTACCCCCCTCTACAAGACTCAAGCCTGCCAGTTTCAAATGCAGTTCCCAGGT  848
             ||||||||||||||||||||||||||||||||||||||||||||||||||||||||||||
Sbjct  592   TGGAATTCTACCCCCCTCTACAAGACTCAAGCCTGCCAGTTTCAAATGCAGTTCCCAGGT  533

Query  849   TAAGCCCGGGGATTTCACATCTGACTTAACAGACCGCCTGCGTGCGCTTTACGCCCAGTA  908
             ||||||||||||||||||||||||||||||||||||||||||||||||||||||||||||
Sbjct  532   TAAGCCCGGGGATTTCACATCTGACTTAACAGACCGCCTGCGTGCGCTTTACGCCCAGTA  473

Query  909   ATTCCGATTAACGCTTGCACCCTCCGTATTACCGCGGCTGCTGGCACGGNGTTAGCCGGT  968
             ||||||||||||||||||||||||||||||||||||||||||||||||| ||||||||||
Sbjct  472   ATTCCGATTAACGCTTGCACCCTCCGTATTACCGCGGCTGCTGGCACGGAGTTAGCCGGT  413

Query  969   GCTTCTTCTGCGGGTAACGTCAATCGAC  996
             ||||||||||||||||||||||||||||
Sbjct  412   GCTTCTTCTGCGGGTAACGTCAATCGAC  385
```

Download

FASTA (complete sequence)

FASTA (aligned sequences)

GenBank (complete sequence)

Continue
Cancel

GenBankGraphics

Next
Previous
Descriptions

Pantoea agglomerans strain Rk92 16S ribosomal RNA gene, partial sequence

Sequence ID: MG547769.1Length: 1395Number of Matches: 1

Related Information

Range 1: 360 to 1347GenBankGraphics

Next Match
Previous Match
First Match

Alignment statistics for match #1

| Score | Expect | Identities | Gaps | Strand | Frame |
| --- | --- | --- | --- | --- | --- |
| 1788 bits(968) | 0.0() | 978/988(99%) | 0/988(0%) | Plus/Minus |  |

Features:

```
Query  9     CGCCCTCCCGAAGGNTNNGCTACCTACTTCTTTTNNANCCCACTCCCATGGTGTGACGGG  68
             |||||||||||||| |  ||||||||||||||||  | ||||||||||||||||||||||
Sbjct  1347  CGCCCTCCCGAAGGTTAAGCTACCTACTTCTTTTGCAACCCACTCCCATGGTGTGACGGG  1288

Query  69    CGGTGTGTACAAGGCCCGGGAACGTATTCACCGTGGCATTCTGATCCACGATTACTAGCG  128
             ||||||||||||||||||||||||||||||||||||||||||||||||||||||||||||
Sbjct  1287  CGGTGTGTACAAGGCCCGGGAACGTATTCACCGTGGCATTCTGATCCACGATTACTAGCG  1228

Query  129   ATTCCGACTTCACGGAGTCGAGTTGCAGACTCCGATCCGGACTACGACGCACTTTGTGAG  188
             ||||||||||||||||||||||||||||||||||||||||||||||||||||||||||||
Sbjct  1227  ATTCCGACTTCACGGAGTCGAGTTGCAGACTCCGATCCGGACTACGACGCACTTTGTGAG  1168

Query  189   GTCCGCTTGCTCTCGCGAGGTCGCTTCTCTTTGTATGCGCCATTGTAGCACGTGTGTAGC  248
             ||||||||||||||||||||||||||||||||||||||||||||||||||||||||||||
Sbjct  1167  GTCCGCTTGCTCTCGCGAGGTCGCTTCTCTTTGTATGCGCCATTGTAGCACGTGTGTAGC  1108

Query  249   CCTACTCGTAAGGGCCATGATGACTTGACGTCATCCCCACCTTCCTCCGGTTTATCACCG  308
             ||||||||||||||||||||||||||||||||||||||||||||||||||||||||||||
Sbjct  1107  CCTACTCGTAAGGGCCATGATGACTTGACGTCATCCCCACCTTCCTCCGGTTTATCACCG  1048

Query  309   GCAGTCTCCTTTGAGTTCCCGACCGAATCGCTGGCAACAAAGGATAAGGGTTGCGCTCGT  368
             ||||||||||||||||||||||||||||||||||||||||||||||||||||||||||||
Sbjct  1047  GCAGTCTCCTTTGAGTTCCCGACCGAATCGCTGGCAACAAAGGATAAGGGTTGCGCTCGT  988

Query  369   TGCGGGACTTAACCCAACATTTCACAACACGAGCTGACGACAGCCATGCAGCACCTGTCT  428
             ||||||||||||||||||||||||||||||||||||||||||||||||||||||||||||
Sbjct  987   TGCGGGACTTAACCCAACATTTCACAACACGAGCTGACGACAGCCATGCAGCACCTGTCT  928

Query  429   CACGGTTCCCGAAGGCACTAAGGCATCTCTGNNNAATTCCGTGGATGTCAAGAGTAGGTA  488
             |||||||||||||||||||||||||||||||   ||||||||||||||||||||||||||
Sbjct  927   CACGGTTCCCGAAGGCACTAAGGCATCTCTGCCAAATTCCGTGGATGTCAAGAGTAGGTA  868

Query  489   AGGTTCTTCGCGTTGCATCGAATTAAACCACATGCTCCACCGCTTGTGCGGGCCCCCGTC  548
             ||||||||||||||||||||||||||||||||||||||||||||||||||||||||||||
Sbjct  867   AGGTTCTTCGCGTTGCATCGAATTAAACCACATGCTCCACCGCTTGTGCGGGCCCCCGTC  808

Query  549   AATTCATTTGAGTTTTAACCTTGCGGCCGTACTCCCCAGGCGGTCGACTTAACGCGTTAG  608
             ||||||||||||||||||||||||||||||||||||||||||||||||||||||||||||
Sbjct  807   AATTCATTTGAGTTTTAACCTTGCGGCCGTACTCCCCAGGCGGTCGACTTAACGCGTTAG  748

Query  609   CTCCGGAAGCCACTCCTCAAGGGAACAACCTCCAAGTCGACATCGTTTACGGCGTGGACT  668
             ||||||||||||||||||||||||||||||||||||||||||||||||||||||||||||
Sbjct  747   CTCCGGAAGCCACTCCTCAAGGGAACAACCTCCAAGTCGACATCGTTTACGGCGTGGACT  688

Query  669   ACCAGGGTATCTAATCCTGTTTGCTCCCCACGCTTTCGCACCTGAGCGTCAGTCTTTGTC  728
             ||||||||||||||||||||||||||||||||||||||||||||||||||||||||||||
Sbjct  687   ACCAGGGTATCTAATCCTGTTTGCTCCCCACGCTTTCGCACCTGAGCGTCAGTCTTTGTC  628

Query  729   CAGGGGGCCGCCTTCGCCACCGGTATTCCTCCAGATCTCTACGCATTTCACCGCTACACC  788
             ||||||||||||||||||||||||||||||||||||||||||||||||||||||||||||
Sbjct  627   CAGGGGGCCGCCTTCGCCACCGGTATTCCTCCAGATCTCTACGCATTTCACCGCTACACC  568

Query  789   TGGAATTCTACCCCCCTCTACAAGACTCAAGCCTGCCAGTTTCAAATGCAGTTCCCAGGT  848
             ||||||||||||||||||||||||||||||||||||||||||||||||||||||||||||
Sbjct  567   TGGAATTCTACCCCCCTCTACAAGACTCAAGCCTGCCAGTTTCAAATGCAGTTCCCAGGT  508

Query  849   TAAGCCCGGGGATTTCACATCTGACTTAACAGACCGCCTGCGTGCGCTTTACGCCCAGTA  908
             ||||||||||||||||||||||||||||||||||||||||||||||||||||||||||||
Sbjct  507   TAAGCCCGGGGATTTCACATCTGACTTAACAGACCGCCTGCGTGCGCTTTACGCCCAGTA  448

Query  909   ATTCCGATTAACGCTTGCACCCTCCGTATTACCGCGGCTGCTGGCACGGNGTTAGCCGGT  968
             ||||||||||||||||||||||||||||||||||||||||||||||||| ||||||||||
Sbjct  447   ATTCCGATTAACGCTTGCACCCTCCGTATTACCGCGGCTGCTGGCACGGAGTTAGCCGGT  388

Query  969   GCTTCTTCTGCGGGTAACGTCAATCGAC  996
             ||||||||||||||||||||||||||||
Sbjct  387   GCTTCTTCTGCGGGTAACGTCAATCGAC  360
```

Download

FASTA (complete sequence)

FASTA (aligned sequences)

GenBank (complete sequence)

Continue
Cancel

GenBankGraphics

Next
Previous
Descriptions

Pantoea agglomerans strain P3FNa\_1 16S ribosomal RNA gene, partial sequence

Sequence ID: KX891531.1Length: 1113Number of Matches: 1

Related Information

Range 1: 106 to 1093GenBankGraphics

Next Match
Previous Match
First Match

Alignment statistics for match #1

| Score | Expect | Identities | Gaps | Strand | Frame |
| --- | --- | --- | --- | --- | --- |
| 1788 bits(968) | 0.0() | 978/988(99%) | 0/988(0%) | Plus/Minus |  |

Features:

```
Query  9     CGCCCTCCCGAAGGNTNNGCTACCTACTTCTTTTNNANCCCACTCCCATGGTGTGACGGG  68
             |||||||||||||| |  ||||||||||||||||  | ||||||||||||||||||||||
Sbjct  1093  CGCCCTCCCGAAGGTTAAGCTACCTACTTCTTTTGCAACCCACTCCCATGGTGTGACGGG  1034

Query  69    CGGTGTGTACAAGGCCCGGGAACGTATTCACCGTGGCATTCTGATCCACGATTACTAGCG  128
             ||||||||||||||||||||||||||||||||||||||||||||||||||||||||||||
Sbjct  1033  CGGTGTGTACAAGGCCCGGGAACGTATTCACCGTGGCATTCTGATCCACGATTACTAGCG  974

Query  129   ATTCCGACTTCACGGAGTCGAGTTGCAGACTCCGATCCGGACTACGACGCACTTTGTGAG  188
             ||||||||||||||||||||||||||||||||||||||||||||||||||||||||||||
Sbjct  973   ATTCCGACTTCACGGAGTCGAGTTGCAGACTCCGATCCGGACTACGACGCACTTTGTGAG  914

Query  189   GTCCGCTTGCTCTCGCGAGGTCGCTTCTCTTTGTATGCGCCATTGTAGCACGTGTGTAGC  248
             ||||||||||||||||||||||||||||||||||||||||||||||||||||||||||||
Sbjct  913   GTCCGCTTGCTCTCGCGAGGTCGCTTCTCTTTGTATGCGCCATTGTAGCACGTGTGTAGC  854

Query  249   CCTACTCGTAAGGGCCATGATGACTTGACGTCATCCCCACCTTCCTCCGGTTTATCACCG  308
             ||||||||||||||||||||||||||||||||||||||||||||||||||||||||||||
Sbjct  853   CCTACTCGTAAGGGCCATGATGACTTGACGTCATCCCCACCTTCCTCCGGTTTATCACCG  794

Query  309   GCAGTCTCCTTTGAGTTCCCGACCGAATCGCTGGCAACAAAGGATAAGGGTTGCGCTCGT  368
             ||||||||||||||||||||||||||||||||||||||||||||||||||||||||||||
Sbjct  793   GCAGTCTCCTTTGAGTTCCCGACCGAATCGCTGGCAACAAAGGATAAGGGTTGCGCTCGT  734

Query  369   TGCGGGACTTAACCCAACATTTCACAACACGAGCTGACGACAGCCATGCAGCACCTGTCT  428
             ||||||||||||||||||||||||||||||||||||||||||||||||||||||||||||
Sbjct  733   TGCGGGACTTAACCCAACATTTCACAACACGAGCTGACGACAGCCATGCAGCACCTGTCT  674

Query  429   CACGGTTCCCGAAGGCACTAAGGCATCTCTGNNNAATTCCGTGGATGTCAAGAGTAGGTA  488
             |||||||||||||||||||||||||||||||   ||||||||||||||||||||||||||
Sbjct  673   CACGGTTCCCGAAGGCACTAAGGCATCTCTGCCAAATTCCGTGGATGTCAAGAGTAGGTA  614

Query  489   AGGTTCTTCGCGTTGCATCGAATTAAACCACATGCTCCACCGCTTGTGCGGGCCCCCGTC  548
             ||||||||||||||||||||||||||||||||||||||||||||||||||||||||||||
Sbjct  613   AGGTTCTTCGCGTTGCATCGAATTAAACCACATGCTCCACCGCTTGTGCGGGCCCCCGTC  554

Query  549   AATTCATTTGAGTTTTAACCTTGCGGCCGTACTCCCCAGGCGGTCGACTTAACGCGTTAG  608
             ||||||||||||||||||||||||||||||||||||||||||||||||||||||||||||
Sbjct  553   AATTCATTTGAGTTTTAACCTTGCGGCCGTACTCCCCAGGCGGTCGACTTAACGCGTTAG  494

Query  609   CTCCGGAAGCCACTCCTCAAGGGAACAACCTCCAAGTCGACATCGTTTACGGCGTGGACT  668
             ||||||||||||||||||||||||||||||||||||||||||||||||||||||||||||
Sbjct  493   CTCCGGAAGCCACTCCTCAAGGGAACAACCTCCAAGTCGACATCGTTTACGGCGTGGACT  434

Query  669   ACCAGGGTATCTAATCCTGTTTGCTCCCCACGCTTTCGCACCTGAGCGTCAGTCTTTGTC  728
             ||||||||||||||||||||||||||||||||||||||||||||||||||||||||||||
Sbjct  433   ACCAGGGTATCTAATCCTGTTTGCTCCCCACGCTTTCGCACCTGAGCGTCAGTCTTTGTC  374

Query  729   CAGGGGGCCGCCTTCGCCACCGGTATTCCTCCAGATCTCTACGCATTTCACCGCTACACC  788
             ||||||||||||||||||||||||||||||||||||||||||||||||||||||||||||
Sbjct  373   CAGGGGGCCGCCTTCGCCACCGGTATTCCTCCAGATCTCTACGCATTTCACCGCTACACC  314

Query  789   TGGAATTCTACCCCCCTCTACAAGACTCAAGCCTGCCAGTTTCAAATGCAGTTCCCAGGT  848
             ||||||||||||||||||||||||||||||||||||||||||||||||||||||||||||
Sbjct  313   TGGAATTCTACCCCCCTCTACAAGACTCAAGCCTGCCAGTTTCAAATGCAGTTCCCAGGT  254

Query  849   TAAGCCCGGGGATTTCACATCTGACTTAACAGACCGCCTGCGTGCGCTTTACGCCCAGTA  908
             ||||||||||||||||||||||||||||||||||||||||||||||||||||||||||||
Sbjct  253   TAAGCCCGGGGATTTCACATCTGACTTAACAGACCGCCTGCGTGCGCTTTACGCCCAGTA  194

Query  909   ATTCCGATTAACGCTTGCACCCTCCGTATTACCGCGGCTGCTGGCACGGNGTTAGCCGGT  968
             ||||||||||||||||||||||||||||||||||||||||||||||||| ||||||||||
Sbjct  193   ATTCCGATTAACGCTTGCACCCTCCGTATTACCGCGGCTGCTGGCACGGAGTTAGCCGGT  134

Query  969   GCTTCTTCTGCGGGTAACGTCAATCGAC  996
             ||||||||||||||||||||||||||||
Sbjct  133   GCTTCTTCTGCGGGTAACGTCAATCGAC  106
```

Download

FASTA (complete sequence)

FASTA (aligned sequences)

GenBank (complete sequence)

Continue
Cancel

GenBankGraphics

Next
Previous
Descriptions

Pantoea agglomerans strain P2FNb\_1 16S ribosomal RNA gene, partial sequence

Sequence ID: KX891517.1Length: 1028Number of Matches: 1

Related Information

Range 1: 18 to 1005GenBankGraphics

Next Match
Previous Match
First Match

Alignment statistics for match #1

| Score | Expect | Identities | Gaps | Strand | Frame |
| --- | --- | --- | --- | --- | --- |
| 1788 bits(968) | 0.0() | 978/988(99%) | 0/988(0%) | Plus/Minus |  |

Features:

```
Query  9     CGCCCTCCCGAAGGNTNNGCTACCTACTTCTTTTNNANCCCACTCCCATGGTGTGACGGG  68
             |||||||||||||| |  ||||||||||||||||  | ||||||||||||||||||||||
Sbjct  1005  CGCCCTCCCGAAGGTTAAGCTACCTACTTCTTTTGCAACCCACTCCCATGGTGTGACGGG  946

Query  69    CGGTGTGTACAAGGCCCGGGAACGTATTCACCGTGGCATTCTGATCCACGATTACTAGCG  128
             ||||||||||||||||||||||||||||||||||||||||||||||||||||||||||||
Sbjct  945   CGGTGTGTACAAGGCCCGGGAACGTATTCACCGTGGCATTCTGATCCACGATTACTAGCG  886

Query  129   ATTCCGACTTCACGGAGTCGAGTTGCAGACTCCGATCCGGACTACGACGCACTTTGTGAG  188
             ||||||||||||||||||||||||||||||||||||||||||||||||||||||||||||
Sbjct  885   ATTCCGACTTCACGGAGTCGAGTTGCAGACTCCGATCCGGACTACGACGCACTTTGTGAG  826

Query  189   GTCCGCTTGCTCTCGCGAGGTCGCTTCTCTTTGTATGCGCCATTGTAGCACGTGTGTAGC  248
             ||||||||||||||||||||||||||||||||||||||||||||||||||||||||||||
Sbjct  825   GTCCGCTTGCTCTCGCGAGGTCGCTTCTCTTTGTATGCGCCATTGTAGCACGTGTGTAGC  766

Query  249   CCTACTCGTAAGGGCCATGATGACTTGACGTCATCCCCACCTTCCTCCGGTTTATCACCG  308
             ||||||||||||||||||||||||||||||||||||||||||||||||||||||||||||
Sbjct  765   CCTACTCGTAAGGGCCATGATGACTTGACGTCATCCCCACCTTCCTCCGGTTTATCACCG  706

Query  309   GCAGTCTCCTTTGAGTTCCCGACCGAATCGCTGGCAACAAAGGATAAGGGTTGCGCTCGT  368
             ||||||||||||||||||||||||||||||||||||||||||||||||||||||||||||
Sbjct  705   GCAGTCTCCTTTGAGTTCCCGACCGAATCGCTGGCAACAAAGGATAAGGGTTGCGCTCGT  646

Query  369   TGCGGGACTTAACCCAACATTTCACAACACGAGCTGACGACAGCCATGCAGCACCTGTCT  428
             ||||||||||||||||||||||||||||||||||||||||||||||||||||||||||||
Sbjct  645   TGCGGGACTTAACCCAACATTTCACAACACGAGCTGACGACAGCCATGCAGCACCTGTCT  586

Query  429   CACGGTTCCCGAAGGCACTAAGGCATCTCTGNNNAATTCCGTGGATGTCAAGAGTAGGTA  488
             |||||||||||||||||||||||||||||||   ||||||||||||||||||||||||||
Sbjct  585   CACGGTTCCCGAAGGCACTAAGGCATCTCTGCCAAATTCCGTGGATGTCAAGAGTAGGTA  526

Query  489   AGGTTCTTCGCGTTGCATCGAATTAAACCACATGCTCCACCGCTTGTGCGGGCCCCCGTC  548
             ||||||||||||||||||||||||||||||||||||||||||||||||||||||||||||
Sbjct  525   AGGTTCTTCGCGTTGCATCGAATTAAACCACATGCTCCACCGCTTGTGCGGGCCCCCGTC  466

Query  549   AATTCATTTGAGTTTTAACCTTGCGGCCGTACTCCCCAGGCGGTCGACTTAACGCGTTAG  608
             ||||||||||||||||||||||||||||||||||||||||||||||||||||||||||||
Sbjct  465   AATTCATTTGAGTTTTAACCTTGCGGCCGTACTCCCCAGGCGGTCGACTTAACGCGTTAG  406

Query  609   CTCCGGAAGCCACTCCTCAAGGGAACAACCTCCAAGTCGACATCGTTTACGGCGTGGACT  668
             ||||||||||||||||||||||||||||||||||||||||||||||||||||||||||||
Sbjct  405   CTCCGGAAGCCACTCCTCAAGGGAACAACCTCCAAGTCGACATCGTTTACGGCGTGGACT  346

Query  669   ACCAGGGTATCTAATCCTGTTTGCTCCCCACGCTTTCGCACCTGAGCGTCAGTCTTTGTC  728
             ||||||||||||||||||||||||||||||||||||||||||||||||||||||||||||
Sbjct  345   ACCAGGGTATCTAATCCTGTTTGCTCCCCACGCTTTCGCACCTGAGCGTCAGTCTTTGTC  286

Query  729   CAGGGGGCCGCCTTCGCCACCGGTATTCCTCCAGATCTCTACGCATTTCACCGCTACACC  788
             ||||||||||||||||||||||||||||||||||||||||||||||||||||||||||||
Sbjct  285   CAGGGGGCCGCCTTCGCCACCGGTATTCCTCCAGATCTCTACGCATTTCACCGCTACACC  226

Query  789   TGGAATTCTACCCCCCTCTACAAGACTCAAGCCTGCCAGTTTCAAATGCAGTTCCCAGGT  848
             ||||||||||||||||||||||||||||||||||||||||||||||||||||||||||||
Sbjct  225   TGGAATTCTACCCCCCTCTACAAGACTCAAGCCTGCCAGTTTCAAATGCAGTTCCCAGGT  166

Query  849   TAAGCCCGGGGATTTCACATCTGACTTAACAGACCGCCTGCGTGCGCTTTACGCCCAGTA  908
             ||||||||||||||||||||||||||||||||||||||||||||||||||||||||||||
Sbjct  165   TAAGCCCGGGGATTTCACATCTGACTTAACAGACCGCCTGCGTGCGCTTTACGCCCAGTA  106

Query  909   ATTCCGATTAACGCTTGCACCCTCCGTATTACCGCGGCTGCTGGCACGGNGTTAGCCGGT  968
             ||||||||||||||||||||||||||||||||||||||||||||||||| ||||||||||
Sbjct  105   ATTCCGATTAACGCTTGCACCCTCCGTATTACCGCGGCTGCTGGCACGGAGTTAGCCGGT  46

Query  969   GCTTCTTCTGCGGGTAACGTCAATCGAC  996
             ||||||||||||||||||||||||||||
Sbjct  45    GCTTCTTCTGCGGGTAACGTCAATCGAC  18
```


BLAST is a registered trademark of the National Library of Medicine

Support center
Mailing list


YouTube

- National Library Of Medicine
- National Institutes Of Health
- U.S. Department of Health & Human Services
- USA.gov

### NCBI


National Center for Biotechnology Information,
 U.S. National Library of Medicine

8600 Rockville Pike,
Bethesda
 MD,
20894
USA

Policies and Guidelines
|
Contact


PreferencesTurn off

External link. Please review our privacy policy.
