# Supplementary material for: Isothermal microcalorimetry for thermal viable count of microorganisms in pure cultures and stabilized formulations
Source: BMC Microbiol. 2019 Mar 21;19:65. doi: 10.1186/s12866-019-1432-8 (PMC6429831; doi:10.1186/s12866-019-1432-8)
Supplement: Supplementary file 3 — 16S rRNA gene sequences’ BLASTN hits in zipped HTML format. (ZIP 15810 kb) [file 12866_2019_1432_MOESM3_ESM.zip › Best blastn hits/NCBI Blast_79 yellowish negative R -- 17..1016 of.html]

NCBI Blast:79 yellowish negative R -- 17..1016 of


- NCBI Home
- Sign in to NCBI
- Skip to Main Content
- Skip to Navigation
- About NCBI Accesskeys

U.S. National Library of Medicine

NCBI
National Center for Biotechnology Information

- My NCBI
- Sign in to NCBI
- Register
- Sign Out

BLAST ^®^ » blastn suite » RID-A4WASJER015


- Home
- Recent Results
- Saved Strategies
- Help

BLAST Results


Edit and Resubmit
Save Search Strategies
[Sign in above to save your search strategy]

Formatting options 


Download


How to read this page
Blast report description
Questions/comments


|  |  |
| --- | --- |
| Formatting options | |
| Show | Alignment as  HTML Plain text   Old View Reset form to defaults [?]  These options control formatting of alignments in results pages. The default is HTML, but other formats (including plain text) are available. PSSM and PssmWithParameters are representations of Position Specific Scoring Matrices and are only available for PSI-BLAST. The Advanced view option allows the database descriptions to be sorted by various indices in a table. |
| Alignment View | Pairwise Pairwise with dots for identities Query-anchored with dots for identities Query-anchored with letters for identities Flat query-anchored with dots for identities Flat query-anchored with letters for identities [?]  Choose how to view alignments. The default "pairwise" view shows how each subject sequence aligns individually to the query sequence. The "query-anchored" view shows how all subject sequences align to the query sequence. For each view type, you can choose to show "identities" (matching residues) as letters or dots. more... |
| Display | Graphical Overview   Linkout   Sequence Retrieval  NCBI-gi   CDS feature [?]  - Graphical Overview: Graphical Overview: Show graph of similar sequence regions aligned to query.   more... - NCBI-gi: Show NCBI gi identifiers. - CDS feature: Show annotated coding region and translation.   more... |
| Masking | Character:   X for protein, n for nucleotide Lower Case  Color:  Black Grey Red [?]  - Masking Character: Display masked (filtered) sequence regions as lower-case or as specific letters (N for nucleotide, P for protein). - Masking Color: Display masked sequence regions in the given color. |
| Limit results | Descriptions:  10 50 100 Graphical overview:  0 10 50 100  Alignments:  0 10 50 100 Line length:  60 90 120 150 [?]  - Descriptions: Show short descriptions for up to the given number of sequences. - Alignments: Show alignments for up to the given number of sequences, in order of statistical significance. - Line lenghth: Number of letters to show on one line in an alignment. |
|  | Organism Type common name, binomial, taxid, or group name. Only 20 top taxa will be shown.     Exclude   [?]  Show only sequences from the given organism. |
|  | Entrez query:  [?]  Show only those sequences that match the given Entrez query. more... |
|  | Expect Min:  Expect Max:  [?]  Show only sequences with expect values in the given range. more... |
|  | Percent Identity Min:  Percent Identity Max:  [?]  Show only sequences with percent identity values in the given range. |
| Format for | PSI-BLAST with inclusion threshold:  [?]  - Format for PSI-BLAST: The Position-Specific Iterated BLAST (PSI-BLAST) program performs iterative searches with a protein query,   in which sequences found in one round of search are used to build a custom score model for the next round.   more... - Inclusion Threshold: This sets the statistical significance threshold for including a sequence in the model used   by PSI-BLAST to create the PSSM on the next iteration. |

|  |  |  |  |  |  |
| --- | --- | --- | --- | --- | --- |
| Download | | | | | |
| Alignment  Text XML ASN.1 JSON Seq-align Hit Table(text) Hit Table(csv) Multiple-file XML2 Single-file XML2 Multiple-file JSON Single-file JSON SAM | Search Strategies  ASN.1 | PSSM to restart search  PSSM | [?] |  |  |

The Download link provides BLAST output that may be used as input to another program.
This includes parseable formats such as the tabular report or XML as well as the Search Strategy files read by the BLAST+ applications.
More details on the parseable (XML, tabular, and ASN.1) reports can be found at
https://www.ncbi.nlm.nih.gov/books/NBK153387/  
  

The following formats are offered under the Alignment section:  
1). "Text". Non-HTML standard BLAST report.  
2). "XML". XML report based upon the DTD at https://www.ncbi.nlm.nih.gov/data\_specs/dtd/NCBI\_BlastOutput.dtd  
3). "ASN.1". Alignment written out in Abstract Syntax Notation 1.  
4). "JSON Seq-align". Alignment written out in JSON.  
4). "Hit Table(text)". The tabular report as text.  
5). "Hit Table(csv)". The tabular report ready for import into spread-sheet programs like Excel.  
6). "XML2". New XML format described at ftp://ftp.ncbi.nlm.nih.gov/blast/documents/NEWXML/xml2.pdf.  
7). "JSON". New JSON format described at ftp://ftp.ncbi.nlm.nih.gov/blast/documents/NEWXML/xml2.pdf.  
8). "SAM". Sequence Alignment Map format.

XML2 and JSON can be downloaded either as one file per query (multiple-file) or one file for all queries (single-file). These formats are listed as Multiple-file XML2 (and JSON) or Single-file XML (and JSON).

The following report is offered under the Search Strategy section:  
1). "ASN.1" Search Strategy. A record of the parameters, query, and database used in the search. This file can be used to start a stand-alone BLAST search, see
https://www.ncbi.nlm.nih.gov/books/NBK1763/#CmdLineAppsManual.I455\_BLAST\_search\_stra


# Job title: 79 yellowish negative R -- 17..1016 of

Results for:

lcl|Query\_228735 79 yellowish negative R -- 17..1016 of sequence(1000bp)
[?]

Your BLAST job specified more than one input sequence.
This box lets you choose which input sequence to show BLAST results for.

RID
:   A4WASJER015 (Expires on 03-10 18:34 pm)

Query ID
:   lcl|Query\_228735
:   lcl|Query\_228735

Description
:   79 yellowish negative R -- 17..1016 of sequence

Molecule type
:   nucleic acid

Query Length
:   1000

Database Name
:   nr

Description
:   Nucleotide collection (nt) See details

Program
:   BLASTN 2.8.0+ Citation

  

Reference 

Zheng Zhang, Scott Schwartz, Lukas Wagner, and Webb Miller (2000), "A greedy algorithm for aligning DNA sequences", J Comput Biol 2000; 7(1-2):203-14.

Reference - database indexing

Aleksandr Morgulis, George Coulouris, Yan Raytselis, Thomas L. Madden, Richa Agarwala, Alejandro A. Schäffer (2008), "Database Indexing for Production MegaBLAST Searches", Bioinformatics 24:1757-1764.

Other reports:
Search Summary

[Taxonomy reports]
[Distance tree of results]
[MSA viewer]

Search Parameters

| Search parameter name | Search parameter value |
| --- | --- |
| Program | blastn |
| Word size | 28 |
| Expect value | 10 |
| Hitlist size | 100 |
| Match/Mismatch scores | 1,-2 |
| Gapcosts | 0,2.5 |
| Low Complexity Filter | Yes |
| Filter string | L;m; |
| Genetic Code | 1 |

Database

| Database parameter name | Database parameter value |
| --- | --- |
| Posted date | Mar 7, 2018 1:58 PM |
| Number of letters | 174,044,644,244 |
| Number of sequences | 46,882,714 |
| Entrez query | Includes:  Excludes:  None |

Karlin-Altschul statistics

| Params | Ungapped | Gapped |
| --- | --- | --- |
| Lambda | 1.33271 | 1.28 |
| K | 0.620991 | 0.46 |
| H | 1.12409 | 0.85 |

Results Statistics

| Results Statistics parameter name | Results Statistics parameter value |
| --- | --- |
| Length adjustment | 35 |
| Effective length of query | 965 |
| Effective length of database | 172403749254 |
| Effective search space | 166369618030110 |
| Effective search space used | 166369618030110 |


## Graphic Summary

### Distribution of the top 112 Blast Hits on 100 subject sequences [?]

The graphic is an overview of the database sequences aligned to the query sequence. These are represented horizontal bars colored coded by score and showing the extent
of the alignment on the query sequence. Separate aligned regions on the same database sequence are connected by a thin grey line.
Mousing over an alignment shows the database sequence title. Clicking an alignment displays a box with more details about the alignment and
link to the sequence alignment itself in the Alignments section of the report.

Mouse over to see the title, click to show alignments

Color key for alignment scores

<40

40-50

50-80

80-200

>=200

Query

1

200

400

600

800

1000

Pantoea vagans strain FDAARGOS\_160 chromosome, complete..

Score:1784 Evalue:0

Accession:CP014129.2

Alignment

Pantoea agglomerans strain AL14 16S ribosomal RNA gene,..

Score:1784 Evalue:0

Accession:MG819218.1

Alignment

Pantoea agglomerans strain Rk92 16S ribosomal RNA gene,..

Score:1784 Evalue:0

Accession:MG547769.1

Alignment

Pantoea agglomerans strain P3FNa\_1 16S ribosomal RNA ge..

Score:1784 Evalue:0

Accession:KX891531.1

Alignment

Pantoea agglomerans strain P2FNb\_1 16S ribosomal RNA ge..

Score:1784 Evalue:0

Accession:KX891517.1

Alignment

Pantoea agglomerans strain P2FNa\_2 16S ribosomal RNA ge..

Score:1784 Evalue:0

Accession:KX891516.1

Alignment

Pantoea agglomerans strain P2FNa\_1 16S ribosomal RNA ge..

Score:1784 Evalue:0

Accession:KX891515.1

Alignment

Pantoea agglomerans strain P1FPa\_2 16S ribosomal RNA ge..

Score:1784 Evalue:0

Accession:KX891502.1

Alignment

Pantoea agglomerans strain P1FNd\_1 16S ribosomal RNA ge..

Score:1784 Evalue:0

Accession:KX891501.1

Alignment

Pantoea agglomerans strain P1FNc\_2 16S ribosomal RNA ge..

Score:1784 Evalue:0

Accession:KX891500.1

Alignment

Pantoea agglomerans strain P1FNc\_1 16S ribosomal RNA ge..

Score:1784 Evalue:0

Accession:KX891499.1

Alignment

Pantoea agglomerans strain P1FNb\_1 16S ribosomal RNA ge..

Score:1784 Evalue:0

Accession:KX891498.1

Alignment

Pantoea agglomerans strain P1FNa\_1 16S ribosomal RNA ge..

Score:1784 Evalue:0

Accession:KX891497.1

Alignment

Pantoea agglomerans strain 62 16S ribosomal RNA gene, p..

Score:1784 Evalue:0

Accession:MF767519.1

Alignment

Pantoea agglomerans strain 22MB 16S ribosomal RNA gene,..

Score:1784 Evalue:0

Accession:MF372619.1

Alignment

Pantoea agglomerans strain C410P1, complete genome

Score:1784 Evalue:0

Accession:CP016889.1

Alignment

Uncultured Pantoea sp. clone GJ\_(2)\_73 16S ribosomal RN..

Score:1784 Evalue:0

Accession:KX603481.1

Alignment

Uncultured Pantoea sp. clone GJ\_(2)\_7 16S ribosomal RNA..

Score:1784 Evalue:0

Accession:KX603479.1

Alignment

Pantoea agglomerans strain BL26 16S ribosomal RNA gene,..

Score:1784 Evalue:0

Accession:KU258277.1

Alignment

Pantoea sp. CanR-85 16S ribosomal RNA gene, partial seq..

Score:1784 Evalue:0

Accession:KT580675.1

Alignment

Pantoea agglomerans strain SSE 16S ribosomal RNA gene, ..

Score:1784 Evalue:0

Accession:KT075193.1

Alignment

Pantoea agglomerans strain QSC 16S ribosomal RNA gene, ..

Score:1784 Evalue:0

Accession:KT075184.1

Alignment

Pantoea agglomerans strain NSF 16S ribosomal RNA gene, ..

Score:1784 Evalue:0

Accession:KT075171.1

Alignment

Pantoea agglomerans strain NSD 16S ribosomal RNA gene, ..

Score:1784 Evalue:0

Accession:KT075169.1

Alignment

Pantoea agglomerans strain GSC 16S ribosomal RNA gene, ..

Score:1784 Evalue:0

Accession:KT075163.1

Alignment

Pantoea agglomerans strain GSB 16S ribosomal RNA gene, ..

Score:1784 Evalue:0

Accession:KT075162.1

Alignment

Pantoea agglomerans strain B81 16S ribosomal RNA gene, ..

Score:1784 Evalue:0

Accession:KJ781904.1

Alignment

Curtobacterium plantarum strain S4 16S ribosomal RNA ge..

Score:1784 Evalue:0

Accession:KP099434.1

Alignment

Curtobacterium plantarum strain S2 16S ribosomal RNA ge..

Score:1784 Evalue:0

Accession:KP099433.1

Alignment

Enterobacteriaceae bacterium X1/SB37 gene for 16S ribos..

Score:1784 Evalue:0

Accession:LC007922.1

Alignment

Enterobacteriaceae bacterium X1/SB52 gene for 16S ribos..

Score:1784 Evalue:0

Accession:LC007878.1

Alignment

Enterobacteriaceae bacterium X1/SB44 gene for 16S ribos..

Score:1784 Evalue:0

Accession:LC007874.1

Alignment

Pantoea agglomerans strain ex2 culture-collection CGMCC..

Score:1784 Evalue:0

Accession:KF317872.1

Alignment

Pantoea sp. E100 16S ribosomal RNA gene, partial sequen..

Score:1784 Evalue:0

Accession:KF153221.1

Alignment

Pantoea agglomerans strain ABAC22 16S ribosomal RNA gen..

Score:1784 Evalue:0

Accession:KF003386.1

Alignment

Enterobacteriaceae bacterium SAP952.2 16S ribosomal RNA..

Score:1784 Evalue:0

Accession:JX067721.1

Alignment

Enterobacteriaceae bacterium SAP770.3 16S ribosomal RNA..

Score:1784 Evalue:0

Accession:JX067684.1

Alignment

Enterobacteriaceae bacterium SAP108.1 16S ribosomal RNA..

Score:1784 Evalue:0

Accession:JX067654.1

Alignment

Pantoea agglomerans strain Z01 16S ribosomal RNA gene, ..

Score:1784 Evalue:0

Accession:JX257179.1

Alignment

Uncultured Pantoea sp. clone WRFC18 16S ribosomal RNA g..

Score:1784 Evalue:0

Accession:JX279936.1

Alignment

Pantoea agglomerans strain KB64 16S ribosomal RNA gene,..

Score:1784 Evalue:0

Accession:JF327484.1

Alignment

Pantoea agglomerans strain KB38 16S ribosomal RNA gene,..

Score:1784 Evalue:0

Accession:JF327464.1

Alignment

Pantoea agglomerans strain CJ-G-R2A5 16S ribosomal RNA ..

Score:1784 Evalue:0

Accession:HM584289.1

Alignment

[Curtobacterium] plantarum strain CL63 16S ribosomal RN..

Score:1784 Evalue:0

Accession:NR\_104943.1

Alignment

Pantoea agglomerans strain 1.2244 16S ribosomal RNA gen..

Score:1784 Evalue:0

Accession:HM130689.1

Alignment

Pantoea agglomerans strain SR-1 16S ribosomal RNA gene,..

Score:1784 Evalue:0

Accession:FJ593000.1

Alignment

Pantoea agglomerans strain SN-2 16S ribosomal RNA gene,..

Score:1784 Evalue:0

Accession:FJ592996.1

Alignment

Pantoea agglomerans strain NZ 16S ribosomal RNA gene, p..

Score:1784 Evalue:0

Accession:FJ756348.1

Alignment

Pantoea agglomerans strain VA21971 16S ribosomal RNA ge..

Score:1784 Evalue:0

Accession:FJ611833.1

Alignment

Pantoea agglomerans strain P5WAM 16S ribosomal RNA gene..

Score:1784 Evalue:0

Accession:FJ611822.1

Alignment

Pantoea agglomerans pv. gypsophilae strain ATCC 43348 1..

Score:1784 Evalue:0

Accession:FJ611821.1

Alignment

Pantoea agglomerans strain BBPE0103202 16S ribosomal RN..

Score:1784 Evalue:0

Accession:FJ357809.1

Alignment

Pantoea sp. II\_Gauze\_W\_10\_17 16S ribosomal RNA gene, pa..

Score:1784 Evalue:0

Accession:FJ267564.1

Alignment

Pantoea sp. Wd5760 16S ribosomal RNA gene, partial sequ..

Score:1784 Evalue:0

Accession:EU828365.1

Alignment

Enterobacter agglomerans strain A57 16S ribosomal RNA, ..

Score:1784 Evalue:0

Accession:AF130927.2

Alignment

Marine bacterium MSC106 16S ribosomal RNA gene, partial..

Score:1784 Evalue:0

Accession:EU753130.1

Alignment

## Descriptions

, Reading indexes 1-5, displaying indexes 1-5


Load next setPrevious Match

Sequences producing significant alignments:

Show all columns  of the table presenting sequences producing significant alignments 

Select:AllNone
Selected:0

Alignments
Download

FASTA (complete sequence)

FASTA (aligned sequences)

GenBank (complete sequence)

Hit Table (text)

Hit Table (CSV)

Text

XML

ASN.1

Continue
Cancel

GenBank 
Graphics
Distance tree of results
Multiple alignment
Show/hide columns of the table presenting sequences producing significant alignments 

Available columns

Description  
Max Score  
Total Score  
Coverage  
E-value  
IdentN  
Accession  
Restore Defaults
Ok
Cancel

Sequences producing significant alignments:

| Select for downloading or viewing reports | Description | Max score | Total score | Query cover | E value | Ident | Accession |
| --- | --- | --- | --- | --- | --- | --- | --- |
| 1Select seq CP014129.2 | Pantoea vagans strain FDAARGOS\_160 chromosome, complete genome | 1784 | 12467 | 98% | 0.0 | 99% | CP014129.2 |
| 2Select seq MG819218.1 | Pantoea agglomerans strain AL14 16S ribosomal RNA gene, partial sequence | 1784 | 1784 | 98% | 0.0 | 99% | MG819218.1 |
| 3Select seq MG547769.1 | Pantoea agglomerans strain Rk92 16S ribosomal RNA gene, partial sequence | 1784 | 1784 | 98% | 0.0 | 99% | MG547769.1 |
| 4Select seq KX891531.1 | Pantoea agglomerans strain P3FNa\_1 16S ribosomal RNA gene, partial sequence | 1784 | 1784 | 98% | 0.0 | 99% | KX891531.1 |
| 5Select seq KX891517.1 | Pantoea agglomerans strain P2FNb\_1 16S ribosomal RNA gene, partial sequence | 1784 | 1784 | 98% | 0.0 | 99% | KX891517.1 |
| 6Select seq KX891516.1 | Pantoea agglomerans strain P2FNa\_2 16S ribosomal RNA gene, partial sequence | 1784 | 1784 | 98% | 0.0 | 99% | KX891516.1 |
| 7Select seq KX891515.1 | Pantoea agglomerans strain P2FNa\_1 16S ribosomal RNA gene, partial sequence | 1784 | 1784 | 98% | 0.0 | 99% | KX891515.1 |
| 8Select seq KX891502.1 | Pantoea agglomerans strain P1FPa\_2 16S ribosomal RNA gene, partial sequence | 1784 | 1784 | 98% | 0.0 | 99% | KX891502.1 |
| 9Select seq KX891501.1 | Pantoea agglomerans strain P1FNd\_1 16S ribosomal RNA gene, partial sequence | 1784 | 1784 | 98% | 0.0 | 99% | KX891501.1 |
| 10Select seq KX891500.1 | Pantoea agglomerans strain P1FNc\_2 16S ribosomal RNA gene, partial sequence | 1784 | 1784 | 98% | 0.0 | 99% | KX891500.1 |
| 11Select seq KX891499.1 | Pantoea agglomerans strain P1FNc\_1 16S ribosomal RNA gene, partial sequence | 1784 | 1784 | 98% | 0.0 | 99% | KX891499.1 |
| 12Select seq KX891498.1 | Pantoea agglomerans strain P1FNb\_1 16S ribosomal RNA gene, partial sequence | 1784 | 1784 | 98% | 0.0 | 99% | KX891498.1 |
| 13Select seq KX891497.1 | Pantoea agglomerans strain P1FNa\_1 16S ribosomal RNA gene, partial sequence | 1784 | 1784 | 98% | 0.0 | 99% | KX891497.1 |
| 14Select seq MF767519.1 | Pantoea agglomerans strain 62 16S ribosomal RNA gene, partial sequence | 1784 | 1784 | 98% | 0.0 | 99% | MF767519.1 |
| 15Select seq MF372619.1 | Pantoea agglomerans strain 22MB 16S ribosomal RNA gene, partial sequence | 1784 | 1784 | 98% | 0.0 | 99% | MF372619.1 |
| 16Select seq CP016889.1 | Pantoea agglomerans strain C410P1, complete genome | 1784 | 12411 | 98% | 0.0 | 99% | CP016889.1 |
| 17Select seq KX603481.1 | Uncultured Pantoea sp. clone GJ\_(2)\_73 16S ribosomal RNA gene, partial sequence | 1784 | 1784 | 98% | 0.0 | 99% | KX603481.1 |
| 18Select seq KX603479.1 | Uncultured Pantoea sp. clone GJ\_(2)\_7 16S ribosomal RNA gene, partial sequence | 1784 | 1784 | 98% | 0.0 | 99% | KX603479.1 |
| 19Select seq KU258277.1 | Pantoea agglomerans strain BL26 16S ribosomal RNA gene, partial sequence | 1784 | 1784 | 98% | 0.0 | 99% | KU258277.1 |
| 20Select seq KT580675.1 | Pantoea sp. CanR-85 16S ribosomal RNA gene, partial sequence | 1784 | 1784 | 98% | 0.0 | 99% | KT580675.1 |
| 21Select seq KT075193.1 | Pantoea agglomerans strain SSE 16S ribosomal RNA gene, partial sequence | 1784 | 1784 | 98% | 0.0 | 99% | KT075193.1 |
| 22Select seq KT075184.1 | Pantoea agglomerans strain QSC 16S ribosomal RNA gene, partial sequence | 1784 | 1784 | 98% | 0.0 | 99% | KT075184.1 |
| 23Select seq KT075171.1 | Pantoea agglomerans strain NSF 16S ribosomal RNA gene, partial sequence | 1784 | 1784 | 98% | 0.0 | 99% | KT075171.1 |
| 24Select seq KT075169.1 | Pantoea agglomerans strain NSD 16S ribosomal RNA gene, partial sequence | 1784 | 1784 | 98% | 0.0 | 99% | KT075169.1 |
| 25Select seq KT075163.1 | Pantoea agglomerans strain GSC 16S ribosomal RNA gene, partial sequence | 1784 | 1784 | 98% | 0.0 | 99% | KT075163.1 |
| 26Select seq KT075162.1 | Pantoea agglomerans strain GSB 16S ribosomal RNA gene, partial sequence | 1784 | 1784 | 98% | 0.0 | 99% | KT075162.1 |
| 27Select seq KJ781904.1 | Pantoea agglomerans strain B81 16S ribosomal RNA gene, partial sequence | 1784 | 1784 | 98% | 0.0 | 99% | KJ781904.1 |
| 28Select seq KP099434.1 | Curtobacterium plantarum strain S4 16S ribosomal RNA gene, partial sequence | 1784 | 1784 | 98% | 0.0 | 99% | KP099434.1 |
| 29Select seq KP099433.1 | Curtobacterium plantarum strain S2 16S ribosomal RNA gene, partial sequence | 1784 | 1784 | 98% | 0.0 | 99% | KP099433.1 |
| 30Select seq LC007922.1 | Enterobacteriaceae bacterium X1/SB37 gene for 16S ribosomal RNA, partial sequence, strain: X1/SB37 | 1784 | 1784 | 98% | 0.0 | 99% | LC007922.1 |
| 31Select seq LC007878.1 | Enterobacteriaceae bacterium X1/SB52 gene for 16S ribosomal RNA, partial sequence, strain: X1/SB52 | 1784 | 1784 | 98% | 0.0 | 99% | LC007878.1 |
| 32Select seq LC007874.1 | Enterobacteriaceae bacterium X1/SB44 gene for 16S ribosomal RNA, partial sequence, strain: X1/SB44 | 1784 | 1784 | 98% | 0.0 | 99% | LC007874.1 |
| 33Select seq KF317872.1 | Pantoea agglomerans strain ex2 culture-collection CGMCC:7.103 16S ribosomal RNA gene, partial sequence | 1784 | 1784 | 98% | 0.0 | 99% | KF317872.1 |
| 34Select seq KF153221.1 | Pantoea sp. E100 16S ribosomal RNA gene, partial sequence | 1784 | 1784 | 98% | 0.0 | 99% | KF153221.1 |
| 35Select seq KF003386.1 | Pantoea agglomerans strain ABAC22 16S ribosomal RNA gene, partial sequence | 1784 | 1784 | 98% | 0.0 | 99% | KF003386.1 |
| 36Select seq JX067721.1 | Enterobacteriaceae bacterium SAP952.2 16S ribosomal RNA gene, partial sequence | 1784 | 1784 | 98% | 0.0 | 99% | JX067721.1 |
| 37Select seq JX067684.1 | Enterobacteriaceae bacterium SAP770.3 16S ribosomal RNA gene, partial sequence | 1784 | 1784 | 98% | 0.0 | 99% | JX067684.1 |
| 38Select seq JX067654.1 | Enterobacteriaceae bacterium SAP108.1 16S ribosomal RNA gene, partial sequence | 1784 | 1784 | 98% | 0.0 | 99% | JX067654.1 |
| 39Select seq JX257179.1 | Pantoea agglomerans strain Z01 16S ribosomal RNA gene, partial sequence | 1784 | 1784 | 98% | 0.0 | 99% | JX257179.1 |
| 40Select seq JX279936.1 | Uncultured Pantoea sp. clone WRFC18 16S ribosomal RNA gene, partial sequence | 1784 | 1784 | 98% | 0.0 | 99% | JX279936.1 |
| 41Select seq JF327484.1 | Pantoea agglomerans strain KB64 16S ribosomal RNA gene, partial sequence | 1784 | 1784 | 98% | 0.0 | 99% | JF327484.1 |
| 42Select seq JF327464.1 | Pantoea agglomerans strain KB38 16S ribosomal RNA gene, partial sequence | 1784 | 1784 | 98% | 0.0 | 99% | JF327464.1 |
| 43Select seq HM584289.1 | Pantoea agglomerans strain CJ-G-R2A5 16S ribosomal RNA gene, partial sequence | 1784 | 1784 | 98% | 0.0 | 99% | HM584289.1 |
| 44Select seq NR\_104943.1 | [Curtobacterium] plantarum strain CL63 16S ribosomal RNA, partial sequence | 1784 | 1784 | 98% | 0.0 | 99% | NR\_104943.1 |
| 45Select seq HM130689.1 | Pantoea agglomerans strain 1.2244 16S ribosomal RNA gene, partial sequence | 1784 | 1784 | 98% | 0.0 | 99% | HM130689.1 |
| 46Select seq FJ593000.1 | Pantoea agglomerans strain SR-1 16S ribosomal RNA gene, partial sequence | 1784 | 1784 | 98% | 0.0 | 99% | FJ593000.1 |
| 47Select seq FJ592996.1 | Pantoea agglomerans strain SN-2 16S ribosomal RNA gene, partial sequence | 1784 | 1784 | 98% | 0.0 | 99% | FJ592996.1 |
| 48Select seq FJ756348.1 | Pantoea agglomerans strain NZ 16S ribosomal RNA gene, partial sequence | 1784 | 1784 | 98% | 0.0 | 99% | FJ756348.1 |
| 49Select seq FJ611833.1 | Pantoea agglomerans strain VA21971 16S ribosomal RNA gene, partial sequence | 1784 | 1784 | 98% | 0.0 | 99% | FJ611833.1 |
| 50Select seq FJ611822.1 | Pantoea agglomerans strain P5WAM 16S ribosomal RNA gene, partial sequence | 1784 | 1784 | 98% | 0.0 | 99% | FJ611822.1 |
| 51Select seq FJ611821.1 | Pantoea agglomerans pv. gypsophilae strain ATCC 43348 16S ribosomal RNA gene, partial sequence | 1784 | 1784 | 98% | 0.0 | 99% | FJ611821.1 |
| 52Select seq FJ357809.1 | Pantoea agglomerans strain BBPE0103202 16S ribosomal RNA gene, partial sequence | 1784 | 1784 | 98% | 0.0 | 99% | FJ357809.1 |
| 53Select seq FJ267564.1 | Pantoea sp. II\_Gauze\_W\_10\_17 16S ribosomal RNA gene, partial sequence | 1784 | 1784 | 98% | 0.0 | 99% | FJ267564.1 |
| 54Select seq EU828365.1 | Pantoea sp. Wd5760 16S ribosomal RNA gene, partial sequence | 1784 | 1784 | 98% | 0.0 | 99% | EU828365.1 |
| 55Select seq AF130927.2 | Enterobacter agglomerans strain A57 16S ribosomal RNA, partial sequence | 1784 | 1784 | 98% | 0.0 | 99% | AF130927.2 |
| 56Select seq EU753130.1 | Marine bacterium MSC106 16S ribosomal RNA gene, partial sequence | 1784 | 1784 | 98% | 0.0 | 99% | EU753130.1 |
| 57Select seq EU598802.1 | Pantoea agglomerans 16S ribosomal RNA gene, partial sequence | 1784 | 1784 | 98% | 0.0 | 99% | EU598802.1 |
| 58Select seq AB244440.1 | Pantoea sp. An4-1 gene for 16S rRNA, partial sequence, strain: An4-1 | 1784 | 1784 | 98% | 0.0 | 99% | AB244440.1 |
| 59Select seq EU047555.1 | Pantoea agglomerans strain TMPSB-P1 16S ribosomal RNA gene, partial sequence | 1784 | 1784 | 98% | 0.0 | 99% | EU047555.1 |
| 60Select seq DQ307453.1 | Pantoea agglomerans strain MM2 16S ribosomal RNA gene, partial sequence | 1784 | 1784 | 98% | 0.0 | 99% | DQ307453.1 |
| 61Select seq DQ307452.1 | Pantoea agglomerans strain FL1 16S ribosomal RNA gene, partial sequence | 1784 | 1784 | 98% | 0.0 | 99% | DQ307452.1 |
| 62Select seq DQ531643.1 | Pantoea sp. 2-2-SS-001 16S ribosomal RNA gene, partial sequence | 1784 | 1784 | 98% | 0.0 | 99% | DQ531643.1 |
| 63Select seq AB004757.2 | Pantoea agglomerans gene for 16S ribosomal RNA, partial sequence | 1784 | 1784 | 98% | 0.0 | 99% | AB004757.2 |
| 64Select seq DQ311006.1 | Pantoea sp. FL9 16S ribosomal RNA gene, partial sequence | 1784 | 1784 | 98% | 0.0 | 99% | DQ311006.1 |
| 65Select seq AJ583835.1 | Pantoea agglomerans partial 16S rRNA gene, strain Pa850T | 1784 | 1784 | 98% | 0.0 | 99% | AJ583835.1 |
| 66Select seq AJ583011.1 | Pantoea agglomerans partial 16S rRNA gene, strain EPS125 | 1784 | 1784 | 98% | 0.0 | 99% | AJ583011.1 |
| 67Select seq NR\_041978.1 | Pantoea agglomerans strain DSM 3493 16S ribosomal RNA gene, partial sequence | 1784 | 1784 | 98% | 0.0 | 99% | NR\_041978.1 |
| 68Select seq MF962590.1 | Pantoea sp. strain DIB76BC6 16S ribosomal RNA gene, partial sequence | 1781 | 1781 | 98% | 0.0 | 99% | MF962590.1 |
| 69Select seq KY357288.1 | Pantoea agglomerans strain ACBP2 16S ribosomal RNA gene, partial sequence | 1781 | 1781 | 98% | 0.0 | 99% | KY357288.1 |
| 70Select seq KY357286.1 | Pantoea agglomerans strain ACBC2 16S ribosomal RNA gene, partial sequence | 1781 | 1781 | 98% | 0.0 | 99% | KY357286.1 |
| 71Select seq KT779903.1 | Pantoea agglomerans strain CCRI01 16S ribosomal RNA gene, partial sequence | 1781 | 1781 | 98% | 0.0 | 99% | KT779903.1 |
| 72Select seq KM985370.1 | Pantoea agglomerans strain gs1 16S ribosomal RNA gene, partial sequence | 1781 | 1781 | 98% | 0.0 | 99% | KM985370.1 |
| 73Select seq KF465938.1 | Pantoea sp. G2Ec2 16S ribosomal RNA gene, partial sequence | 1781 | 1781 | 98% | 0.0 | 99% | KF465938.1 |
| 74Select seq KF003418.1 | Pantoea agglomerans strain ABAC77 16S ribosomal RNA gene, partial sequence | 1781 | 1781 | 98% | 0.0 | 99% | KF003418.1 |
| 75Select seq FJ357814.1 | Pantoea agglomerans strain CUETM8553 16S ribosomal RNA gene, partial sequence | 1781 | 1781 | 98% | 0.0 | 99% | FJ357814.1 |
| 76Select seq AF130895.1 | Pantoea agglomerans strain new\*16 16S ribosomal RNA gene, partial sequence | 1781 | 1781 | 98% | 0.0 | 99% | AF130895.1 |
| 77Select seq KX891508.1 | Pantoea agglomerans strain P1MNa\_1 16S ribosomal RNA gene, partial sequence | 1779 | 1779 | 98% | 0.0 | 99% | KX891508.1 |
| 78Select seq KU978278.1 | Uncultured bacterium clone EHB-P0445 #05 16S ribosomal RNA gene, partial sequence | 1779 | 1779 | 98% | 0.0 | 99% | KU978278.1 |
| 79Select seq KU978258.1 | Uncultured bacterium clone EHB-P0362 #05 16S ribosomal RNA gene, partial sequence | 1779 | 1779 | 98% | 0.0 | 99% | KU978258.1 |
| 80Select seq KR361753.1 | Pantoea agglomerans strain E60Y 16S ribosomal RNA gene, partial sequence | 1779 | 1779 | 98% | 0.0 | 99% | KR361753.1 |
| 81Select seq KT075208.1 | Pantoea agglomerans strain WSA 16S ribosomal RNA gene, partial sequence | 1779 | 1779 | 98% | 0.0 | 99% | KT075208.1 |
| 82Select seq KT075191.1 | Pantoea agglomerans strain SSC 16S ribosomal RNA gene, partial sequence | 1779 | 1779 | 98% | 0.0 | 99% | KT075191.1 |
| 83Select seq KT075185.1 | Pantoea agglomerans strain QSD 16S ribosomal RNA gene, partial sequence | 1779 | 1779 | 98% | 0.0 | 99% | KT075185.1 |
| 84Select seq KP279978.1 | Curtobacterium plantarum strain NS1 16S ribosomal RNA gene, partial sequence | 1779 | 1779 | 98% | 0.0 | 99% | KP279978.1 |
| 85Select seq KP279964.1 | Curtobacterium plantarum strain KS1 16S ribosomal RNA gene, partial sequence | 1779 | 1779 | 98% | 0.0 | 99% | KP279964.1 |
| 86Select seq LC007911.1 | Enterobacteriaceae bacterium X1/SB86 gene for 16S ribosomal RNA, partial sequence, strain: X1/SB86 | 1779 | 1779 | 98% | 0.0 | 99% | LC007911.1 |
| 87Select seq LC007863.1 | Enterobacteriaceae bacterium X1/SB18 gene for 16S ribosomal RNA, partial sequence, strain: X1/SB18 | 1779 | 1779 | 98% | 0.0 | 99% | LC007863.1 |
| 88Select seq JX458430.1 | Pantoea sp. W1.09-234-1 16S ribosomal RNA gene, complete sequence | 1779 | 1779 | 98% | 0.0 | 99% | JX458430.1 |
| 89Select seq KF465834.1 | Pantoea agglomerans strain P19 16S ribosomal RNA gene, partial sequence | 1779 | 1779 | 98% | 0.0 | 99% | KF465834.1 |
| 90Select seq KC178591.1 | Pantoea agglomerans strain Cl-17 16S ribosomal RNA gene, partial sequence | 1779 | 1779 | 98% | 0.0 | 99% | KC178591.1 |
| 91Select seq JX852648.1 | Uncultured Pantoea sp. clone WCFC7 16S ribosomal RNA gene, partial sequence | 1779 | 1779 | 98% | 0.0 | 99% | JX852648.1 |
| 92Select seq JX294750.1 | Uncultured Pantoea sp. clone WRFC196 16S ribosomal RNA gene, partial sequence | 1779 | 1779 | 98% | 0.0 | 99% | JX294750.1 |
| 93Select seq JX134624.1 | Pantoea agglomerans strain -Y115 16S ribosomal RNA gene, partial sequence | 1779 | 1779 | 98% | 0.0 | 99% | JX134624.1 |
| 94Select seq JX113242.1 | Pantoea agglomerans strain Y34 16S ribosomal RNA gene, partial sequence | 1779 | 1779 | 98% | 0.0 | 99% | JX113242.1 |
| 95Select seq JQ412064.1 | Pantoea sp. PR 16S ribosomal RNA gene, partial sequence | 1779 | 1779 | 98% | 0.0 | 99% | JQ412064.1 |
| 96Select seq FR873495.1 | Pantoea agglomerans partial 16S rRNA gene, isolate D3-B-P8ASC2009 | 1779 | 1779 | 98% | 0.0 | 99% | FR873495.1 |
| 97Select seq AB907779.1 | Pantoea agglomerans gene for 16S ribosomal RNA, partial sequence, strain: JCM 1236 | 1777 | 1777 | 98% | 0.0 | 99% | AB907779.1 |
| 98Select seq KC283045.1 | Uncultured Pantoea sp. clone WCFC2 16S ribosomal RNA gene, partial sequence | 1775 | 1775 | 98% | 0.0 | 99% | KC283045.1 |
| 99Select seq JX067704.1 | Enterobacteriaceae bacterium SAP822.1 16S ribosomal RNA gene, partial sequence | 1770 | 1770 | 98% | 0.0 | 99% | JX067704.1 |
| 100Select seq KC311262.1 | Pantoea sp. SBPci 16S ribosomal RNA gene, partial sequence | 1766 | 1766 | 98% | 0.0 | 99% | KC311262.1 |


## Alignments

Loading alignment... for sequences gi|1344462721,gi|1332982380,gi|1278992521,gi|1070257896,gi|1070257882 Reading indexes 1-5

Download

FASTA (complete sequence)

FASTA (aligned sequences)

GenBank (complete sequence)

Continue
Cancel

GenBankGraphics

Sort by:

E value
 Score
Percent identity
Query start position
Subject start position

Next
Previous
Descriptions

Pantoea vagans strain FDAARGOS\_160 chromosome, complete genome

Sequence ID: CP014129.2Length: 4050121Number of Matches: 7

Related Information

Range 1: 49772 to 50754GenBankGraphics

Next Match
Previous Match
First Match

Alignment statistics for match #1

| Score | Expect | Identities | Gaps | Strand | Frame |
| --- | --- | --- | --- | --- | --- |
| 1784 bits(966) | 0.0() | 976/984(99%) | 1/984(0%) | Plus/Plus |  |

Features:

```
Query  10     CTCCCGAAGGTTANGCTACCTACTTCTTTTGCANCCCACTCCCATGGTGTGACGGGCGGT  69
              ||||||||||||| ||||||||||||||||||| ||||||||||||||||||||||||||
Sbjct  49772  CTCCCGAAGGTTAAGCTACCTACTTCTTTTGCAACCCACTCCCATGGTGTGACGGGCGGT  49831

Query  70     GTGTACAAGGCCCGGGAACGTATTCACCGTGGCATTCTGATCCACGATTACTAGCGATTC  129
              ||||||||||||||||||||||||||||||||||||||||||||||||||||||||||||
Sbjct  49832  GTGTACAAGGCCCGGGAACGTATTCACCGTGGCATTCTGATCCACGATTACTAGCGATTC  49891

Query  130    CGACTTCACGGAGTCGAGTTGCAGACTCCGATCCGGACTACGACGCACTTTGTGAGGTCC  189
              ||||||||||||||||||||||||||||||||||||||||||||||||||||||||||||
Sbjct  49892  CGACTTCACGGAGTCGAGTTGCAGACTCCGATCCGGACTACGACGCACTTTGTGAGGTCC  49951

Query  190    GCTTGCTCTCGCGAGGTCGCTTCTCTTTGTATGCGCCATTGTAGCACGTGTGTAGCCCTA  249
              ||||||||||||||||||||||||||||||||||||||||||||||||||||||||||||
Sbjct  49952  GCTTGCTCTCGCGAGGTCGCTTCTCTTTGTATGCGCCATTGTAGCACGTGTGTAGCCCTA  50011

Query  250    CTCGTAAGGGCCATGATGACTTGACGTCATCCCCACCTTCCTCCGGTTTATCACCGGCAG  309
              ||||||||||||||||||||||||||||||||||||||||||||||||||||||||||||
Sbjct  50012  CTCGTAAGGGCCATGATGACTTGACGTCATCCCCACCTTCCTCCGGTTTATCACCGGCAG  50071

Query  310    TCTCCTTTGAGTTCCCGACCGAATCGCTGGCAACAAAGGATAAGGGTTGCGCTCGTTGCG  369
              ||||||||||||||||||||||||||||||||||||||||||||||||||||||||||||
Sbjct  50072  TCTCCTTTGAGTTCCCGACCGAATCGCTGGCAACAAAGGATAAGGGTTGCGCTCGTTGCG  50131

Query  370    GGACTTAACCCAACATTTCACAACACGAGCTGACGACAGCCATGCAGCACCTGTCTCANN  429
              ||||||||||||||||||||||||||||||||||||||||||||||||||||||||||  
Sbjct  50132  GGACTTAACCCAACATTTCACAACACGAGCTGACGACAGCCATGCAGCACCTGTCTCACG  50191

Query  430    GTTCCCGAAGGCACTAAGGCATCTCTGNNAAATTCCGTGGATGTCAAGAGTAGGTAAGGT  489
              |||||||||||||||||||||||||||  |||||||||||||||||||||||||||||||
Sbjct  50192  GTTCCCGAAGGCACTAAGGCATCTCTGCCAAATTCCGTGGATGTCAAGAGTAGGTAAGGT  50251

Query  490    TCTTCGCGTTGCATCGAATTAAACCACATGCTCCACCGCTTGTGCGGGCCCCCGTCAATT  549
              ||||||||||||||||||||||||||||||||||||||||||||||||||||||||||||
Sbjct  50252  TCTTCGCGTTGCATCGAATTAAACCACATGCTCCACCGCTTGTGCGGGCCCCCGTCAATT  50311

Query  550    CATTTGAGTTTTAACCTTGCGGCCGTACTCCCCAGGCGGTCGACTTAACGCGTTAGCTCC  609
              ||||||||||||||||||||||||||||||||||||||||||||||||||||||||||||
Sbjct  50312  CATTTGAGTTTTAACCTTGCGGCCGTACTCCCCAGGCGGTCGACTTAACGCGTTAGCTCC  50371

Query  610    GGAAGCCACTCCTCAAGGGAACAACCTCCAAGTCGACATCGTTTACGGCGTGGACTACCA  669
              ||||||||||||||||||||||||||||||||||||||||||||||||||||||||||||
Sbjct  50372  GGAAGCCACTCCTCAAGGGAACAACCTCCAAGTCGACATCGTTTACGGCGTGGACTACCA  50431

Query  670    GGGTATCTAATCCTGTTTGCTCCCCACGCTTTCGCACCTGAGCGTCAGTCTTTGTCCAGG  729
              ||||||||||||||||||||||||||||||||||||||||||||||||||||||||||||
Sbjct  50432  GGGTATCTAATCCTGTTTGCTCCCCACGCTTTCGCACCTGAGCGTCAGTCTTTGTCCAGG  50491

Query  730    GGGCCGCCTTCGCCACCGGTATTCCTCCAGATCTCTACGCATTTCACCGCTACACCTGGA  789
              ||||||||||||||||||||||||||||||||||||||||||||||||||||||||||||
Sbjct  50492  GGGCCGCCTTCGCCACCGGTATTCCTCCAGATCTCTACGCATTTCACCGCTACACCTGGA  50551

Query  790    ATTCTACCCCCCTCTACAAGACTCAAGCCTGCCAGTTTCAAATGCAGTTCCCAGGTTAAG  849
              ||||||||||||||||||||||||||||||||||||||||||||||||||||||||||||
Sbjct  50552  ATTCTACCCCCCTCTACAAGACTCAAGCCTGCCAGTTTCAAATGCAGTTCCCAGGTTAAG  50611

Query  850    CCCGGGGGATTTCACATCTGACTTAACAGACCGCCTNCGTGCGCTTTACGCCCAGTAATT  909
              ||| |||||||||||||||||||||||||||||||| |||||||||||||||||||||||
Sbjct  50612  CCC-GGGGATTTCACATCTGACTTAACAGACCGCCTGCGTGCGCTTTACGCCCAGTAATT  50670

Query  910    CCGATTAACGCTTGCACCCTCCGTATTACCGCGGCTGCTGGCACGGAGTTAGCCGGTGCT  969
              ||||||||||||||||||||||||||||||||||||||||||||||||||||||||||||
Sbjct  50671  CCGATTAACGCTTGCACCCTCCGTATTACCGCGGCTGCTGGCACGGAGTTAGCCGGTGCT  50730

Query  970    TCTTCTGCGGGTAACGTCAATCGA  993
              ||||||||||||||||||||||||
Sbjct  50731  TCTTCTGCGGGTAACGTCAATCGA  50754
```

Range 2: 171040 to 172021GenBankGraphics

Next Match
Previous Match
First Match

Alignment statistics for match #2

| Score | Expect | Identities | Gaps | Strand | Frame |
| --- | --- | --- | --- | --- | --- |
| 1783 bits(965) | 0.0() | 975/983(99%) | 1/983(0%) | Plus/Plus |  |

Features:

```
Query  10      CTCCCGAAGGTTANGCTACCTACTTCTTTTGCANCCCACTCCCATGGTGTGACGGGCGGT  69
               ||||||||||||| ||||||||||||||||||| ||||||||||||||||||||||||||
Sbjct  171040  CTCCCGAAGGTTAAGCTACCTACTTCTTTTGCAACCCACTCCCATGGTGTGACGGGCGGT  171099

Query  70      GTGTACAAGGCCCGGGAACGTATTCACCGTGGCATTCTGATCCACGATTACTAGCGATTC  129
               ||||||||||||||||||||||||||||||||||||||||||||||||||||||||||||
Sbjct  171100  GTGTACAAGGCCCGGGAACGTATTCACCGTGGCATTCTGATCCACGATTACTAGCGATTC  171159

Query  130     CGACTTCACGGAGTCGAGTTGCAGACTCCGATCCGGACTACGACGCACTTTGTGAGGTCC  189
               ||||||||||||||||||||||||||||||||||||||||||||||||||||||||||||
Sbjct  171160  CGACTTCACGGAGTCGAGTTGCAGACTCCGATCCGGACTACGACGCACTTTGTGAGGTCC  171219

Query  190     GCTTGCTCTCGCGAGGTCGCTTCTCTTTGTATGCGCCATTGTAGCACGTGTGTAGCCCTA  249
               ||||||||||||||||||||||||||||||||||||||||||||||||||||||||||||
Sbjct  171220  GCTTGCTCTCGCGAGGTCGCTTCTCTTTGTATGCGCCATTGTAGCACGTGTGTAGCCCTA  171279

Query  250     CTCGTAAGGGCCATGATGACTTGACGTCATCCCCACCTTCCTCCGGTTTATCACCGGCAG  309
               ||||||||||||||||||||||||||||||||||||||||||||||||||||||||||||
Sbjct  171280  CTCGTAAGGGCCATGATGACTTGACGTCATCCCCACCTTCCTCCGGTTTATCACCGGCAG  171339

Query  310     TCTCCTTTGAGTTCCCGACCGAATCGCTGGCAACAAAGGATAAGGGTTGCGCTCGTTGCG  369
               ||||||||||||||||||||||||||||||||||||||||||||||||||||||||||||
Sbjct  171340  TCTCCTTTGAGTTCCCGACCGAATCGCTGGCAACAAAGGATAAGGGTTGCGCTCGTTGCG  171399

Query  370     GGACTTAACCCAACATTTCACAACACGAGCTGACGACAGCCATGCAGCACCTGTCTCANN  429
               ||||||||||||||||||||||||||||||||||||||||||||||||||||||||||  
Sbjct  171400  GGACTTAACCCAACATTTCACAACACGAGCTGACGACAGCCATGCAGCACCTGTCTCACG  171459

Query  430     GTTCCCGAAGGCACTAAGGCATCTCTGNNAAATTCCGTGGATGTCAAGAGTAGGTAAGGT  489
               |||||||||||||||||||||||||||  |||||||||||||||||||||||||||||||
Sbjct  171460  GTTCCCGAAGGCACTAAGGCATCTCTGCCAAATTCCGTGGATGTCAAGAGTAGGTAAGGT  171519

Query  490     TCTTCGCGTTGCATCGAATTAAACCACATGCTCCACCGCTTGTGCGGGCCCCCGTCAATT  549
               ||||||||||||||||||||||||||||||||||||||||||||||||||||||||||||
Sbjct  171520  TCTTCGCGTTGCATCGAATTAAACCACATGCTCCACCGCTTGTGCGGGCCCCCGTCAATT  171579

Query  550     CATTTGAGTTTTAACCTTGCGGCCGTACTCCCCAGGCGGTCGACTTAACGCGTTAGCTCC  609
               ||||||||||||||||||||||||||||||||||||||||||||||||||||||||||||
Sbjct  171580  CATTTGAGTTTTAACCTTGCGGCCGTACTCCCCAGGCGGTCGACTTAACGCGTTAGCTCC  171639

Query  610     GGAAGCCACTCCTCAAGGGAACAACCTCCAAGTCGACATCGTTTACGGCGTGGACTACCA  669
               ||||||||||||||||||||||||||||||||||||||||||||||||||||||||||||
Sbjct  171640  GGAAGCCACTCCTCAAGGGAACAACCTCCAAGTCGACATCGTTTACGGCGTGGACTACCA  171699

Query  670     GGGTATCTAATCCTGTTTGCTCCCCACGCTTTCGCACCTGAGCGTCAGTCTTTGTCCAGG  729
               ||||||||||||||||||||||||||||||||||||||||||||||||||||||||||||
Sbjct  171700  GGGTATCTAATCCTGTTTGCTCCCCACGCTTTCGCACCTGAGCGTCAGTCTTTGTCCAGG  171759

Query  730     GGGCCGCCTTCGCCACCGGTATTCCTCCAGATCTCTACGCATTTCACCGCTACACCTGGA  789
               ||||||||||||||||||||||||||||||||||||||||||||||||||||||||||||
Sbjct  171760  GGGCCGCCTTCGCCACCGGTATTCCTCCAGATCTCTACGCATTTCACCGCTACACCTGGA  171819

Query  790     ATTCTACCCCCCTCTACAAGACTCAAGCCTGCCAGTTTCAAATGCAGTTCCCAGGTTAAG  849
               ||||||||||||||||||||||||||||||||||||||||||||||||||||||||||||
Sbjct  171820  ATTCTACCCCCCTCTACAAGACTCAAGCCTGCCAGTTTCAAATGCAGTTCCCAGGTTAAG  171879

Query  850     CCCGGGGGATTTCACATCTGACTTAACAGACCGCCTNCGTGCGCTTTACGCCCAGTAATT  909
               ||| |||||||||||||||||||||||||||||||| |||||||||||||||||||||||
Sbjct  171880  CCC-GGGGATTTCACATCTGACTTAACAGACCGCCTGCGTGCGCTTTACGCCCAGTAATT  171938

Query  910     CCGATTAACGCTTGCACCCTCCGTATTACCGCGGCTGCTGGCACGGAGTTAGCCGGTGCT  969
               ||||||||||||||||||||||||||||||||||||||||||||||||||||||||||||
Sbjct  171939  CCGATTAACGCTTGCACCCTCCGTATTACCGCGGCTGCTGGCACGGAGTTAGCCGGTGCT  171998

Query  970     TCTTCTGCGGGTAACGTCAATCG  992
               |||||||||||||||||||||||
Sbjct  171999  TCTTCTGCGGGTAACGTCAATCG  172021
```

Range 3: 447691 to 448672GenBankGraphics

Next Match
Previous Match
First Match

Alignment statistics for match #3

| Score | Expect | Identities | Gaps | Strand | Frame |
| --- | --- | --- | --- | --- | --- |
| 1783 bits(965) | 0.0() | 975/983(99%) | 1/983(0%) | Plus/Minus |  |

Features:

```
Query  10      CTCCCGAAGGTTANGCTACCTACTTCTTTTGCANCCCACTCCCATGGTGTGACGGGCGGT  69
               ||||||||||||| ||||||||||||||||||| ||||||||||||||||||||||||||
Sbjct  448672  CTCCCGAAGGTTAAGCTACCTACTTCTTTTGCAACCCACTCCCATGGTGTGACGGGCGGT  448613

Query  70      GTGTACAAGGCCCGGGAACGTATTCACCGTGGCATTCTGATCCACGATTACTAGCGATTC  129
               ||||||||||||||||||||||||||||||||||||||||||||||||||||||||||||
Sbjct  448612  GTGTACAAGGCCCGGGAACGTATTCACCGTGGCATTCTGATCCACGATTACTAGCGATTC  448553

Query  130     CGACTTCACGGAGTCGAGTTGCAGACTCCGATCCGGACTACGACGCACTTTGTGAGGTCC  189
               ||||||||||||||||||||||||||||||||||||||||||||||||||||||||||||
Sbjct  448552  CGACTTCACGGAGTCGAGTTGCAGACTCCGATCCGGACTACGACGCACTTTGTGAGGTCC  448493

Query  190     GCTTGCTCTCGCGAGGTCGCTTCTCTTTGTATGCGCCATTGTAGCACGTGTGTAGCCCTA  249
               ||||||||||||||||||||||||||||||||||||||||||||||||||||||||||||
Sbjct  448492  GCTTGCTCTCGCGAGGTCGCTTCTCTTTGTATGCGCCATTGTAGCACGTGTGTAGCCCTA  448433

Query  250     CTCGTAAGGGCCATGATGACTTGACGTCATCCCCACCTTCCTCCGGTTTATCACCGGCAG  309
               ||||||||||||||||||||||||||||||||||||||||||||||||||||||||||||
Sbjct  448432  CTCGTAAGGGCCATGATGACTTGACGTCATCCCCACCTTCCTCCGGTTTATCACCGGCAG  448373

Query  310     TCTCCTTTGAGTTCCCGACCGAATCGCTGGCAACAAAGGATAAGGGTTGCGCTCGTTGCG  369
               ||||||||||||||||||||||||||||||||||||||||||||||||||||||||||||
Sbjct  448372  TCTCCTTTGAGTTCCCGACCGAATCGCTGGCAACAAAGGATAAGGGTTGCGCTCGTTGCG  448313

Query  370     GGACTTAACCCAACATTTCACAACACGAGCTGACGACAGCCATGCAGCACCTGTCTCANN  429
               ||||||||||||||||||||||||||||||||||||||||||||||||||||||||||  
Sbjct  448312  GGACTTAACCCAACATTTCACAACACGAGCTGACGACAGCCATGCAGCACCTGTCTCACG  448253

Query  430     GTTCCCGAAGGCACTAAGGCATCTCTGNNAAATTCCGTGGATGTCAAGAGTAGGTAAGGT  489
               |||||||||||||||||||||||||||  |||||||||||||||||||||||||||||||
Sbjct  448252  GTTCCCGAAGGCACTAAGGCATCTCTGCCAAATTCCGTGGATGTCAAGAGTAGGTAAGGT  448193

Query  490     TCTTCGCGTTGCATCGAATTAAACCACATGCTCCACCGCTTGTGCGGGCCCCCGTCAATT  549
               ||||||||||||||||||||||||||||||||||||||||||||||||||||||||||||
Sbjct  448192  TCTTCGCGTTGCATCGAATTAAACCACATGCTCCACCGCTTGTGCGGGCCCCCGTCAATT  448133

Query  550     CATTTGAGTTTTAACCTTGCGGCCGTACTCCCCAGGCGGTCGACTTAACGCGTTAGCTCC  609
               ||||||||||||||||||||||||||||||||||||||||||||||||||||||||||||
Sbjct  448132  CATTTGAGTTTTAACCTTGCGGCCGTACTCCCCAGGCGGTCGACTTAACGCGTTAGCTCC  448073

Query  610     GGAAGCCACTCCTCAAGGGAACAACCTCCAAGTCGACATCGTTTACGGCGTGGACTACCA  669
               ||||||||||||||||||||||||||||||||||||||||||||||||||||||||||||
Sbjct  448072  GGAAGCCACTCCTCAAGGGAACAACCTCCAAGTCGACATCGTTTACGGCGTGGACTACCA  448013

Query  670     GGGTATCTAATCCTGTTTGCTCCCCACGCTTTCGCACCTGAGCGTCAGTCTTTGTCCAGG  729
               ||||||||||||||||||||||||||||||||||||||||||||||||||||||||||||
Sbjct  448012  GGGTATCTAATCCTGTTTGCTCCCCACGCTTTCGCACCTGAGCGTCAGTCTTTGTCCAGG  447953

Query  730     GGGCCGCCTTCGCCACCGGTATTCCTCCAGATCTCTACGCATTTCACCGCTACACCTGGA  789
               ||||||||||||||||||||||||||||||||||||||||||||||||||||||||||||
Sbjct  447952  GGGCCGCCTTCGCCACCGGTATTCCTCCAGATCTCTACGCATTTCACCGCTACACCTGGA  447893

Query  790     ATTCTACCCCCCTCTACAAGACTCAAGCCTGCCAGTTTCAAATGCAGTTCCCAGGTTAAG  849
               ||||||||||||||||||||||||||||||||||||||||||||||||||||||||||||
Sbjct  447892  ATTCTACCCCCCTCTACAAGACTCAAGCCTGCCAGTTTCAAATGCAGTTCCCAGGTTAAG  447833

Query  850     CCCGGGGGATTTCACATCTGACTTAACAGACCGCCTNCGTGCGCTTTACGCCCAGTAATT  909
               ||||||| |||||||||||||||||||||||||||| |||||||||||||||||||||||
Sbjct  447832  CCCGGGG-ATTTCACATCTGACTTAACAGACCGCCTGCGTGCGCTTTACGCCCAGTAATT  447774

Query  910     CCGATTAACGCTTGCACCCTCCGTATTACCGCGGCTGCTGGCACGGAGTTAGCCGGTGCT  969
               ||||||||||||||||||||||||||||||||||||||||||||||||||||||||||||
Sbjct  447773  CCGATTAACGCTTGCACCCTCCGTATTACCGCGGCTGCTGGCACGGAGTTAGCCGGTGCT  447714

Query  970     TCTTCTGCGGGTAACGTCAATCG  992
               |||||||||||||||||||||||
Sbjct  447713  TCTTCTGCGGGTAACGTCAATCG  447691
```

Range 4: 1038308 to 1039289GenBankGraphics

Next Match
Previous Match
First Match

Alignment statistics for match #4

| Score | Expect | Identities | Gaps | Strand | Frame |
| --- | --- | --- | --- | --- | --- |
| 1783 bits(965) | 0.0() | 975/983(99%) | 1/983(0%) | Plus/Minus |  |

Features:

```
Query  10       CTCCCGAAGGTTANGCTACCTACTTCTTTTGCANCCCACTCCCATGGTGTGACGGGCGGT  69
                ||||||||||||| ||||||||||||||||||| ||||||||||||||||||||||||||
Sbjct  1039289  CTCCCGAAGGTTAAGCTACCTACTTCTTTTGCAACCCACTCCCATGGTGTGACGGGCGGT  1039230

Query  70       GTGTACAAGGCCCGGGAACGTATTCACCGTGGCATTCTGATCCACGATTACTAGCGATTC  129
                ||||||||||||||||||||||||||||||||||||||||||||||||||||||||||||
Sbjct  1039229  GTGTACAAGGCCCGGGAACGTATTCACCGTGGCATTCTGATCCACGATTACTAGCGATTC  1039170

Query  130      CGACTTCACGGAGTCGAGTTGCAGACTCCGATCCGGACTACGACGCACTTTGTGAGGTCC  189
                ||||||||||||||||||||||||||||||||||||||||||||||||||||||||||||
Sbjct  1039169  CGACTTCACGGAGTCGAGTTGCAGACTCCGATCCGGACTACGACGCACTTTGTGAGGTCC  1039110

Query  190      GCTTGCTCTCGCGAGGTCGCTTCTCTTTGTATGCGCCATTGTAGCACGTGTGTAGCCCTA  249
                ||||||||||||||||||||||||||||||||||||||||||||||||||||||||||||
Sbjct  1039109  GCTTGCTCTCGCGAGGTCGCTTCTCTTTGTATGCGCCATTGTAGCACGTGTGTAGCCCTA  1039050

Query  250      CTCGTAAGGGCCATGATGACTTGACGTCATCCCCACCTTCCTCCGGTTTATCACCGGCAG  309
                ||||||||||||||||||||||||||||||||||||||||||||||||||||||||||||
Sbjct  1039049  CTCGTAAGGGCCATGATGACTTGACGTCATCCCCACCTTCCTCCGGTTTATCACCGGCAG  1038990

Query  310      TCTCCTTTGAGTTCCCGACCGAATCGCTGGCAACAAAGGATAAGGGTTGCGCTCGTTGCG  369
                ||||||||||||||||||||||||||||||||||||||||||||||||||||||||||||
Sbjct  1038989  TCTCCTTTGAGTTCCCGACCGAATCGCTGGCAACAAAGGATAAGGGTTGCGCTCGTTGCG  1038930

Query  370      GGACTTAACCCAACATTTCACAACACGAGCTGACGACAGCCATGCAGCACCTGTCTCANN  429
                ||||||||||||||||||||||||||||||||||||||||||||||||||||||||||  
Sbjct  1038929  GGACTTAACCCAACATTTCACAACACGAGCTGACGACAGCCATGCAGCACCTGTCTCACG  1038870

Query  430      GTTCCCGAAGGCACTAAGGCATCTCTGNNAAATTCCGTGGATGTCAAGAGTAGGTAAGGT  489
                |||||||||||||||||||||||||||  |||||||||||||||||||||||||||||||
Sbjct  1038869  GTTCCCGAAGGCACTAAGGCATCTCTGCCAAATTCCGTGGATGTCAAGAGTAGGTAAGGT  1038810

Query  490      TCTTCGCGTTGCATCGAATTAAACCACATGCTCCACCGCTTGTGCGGGCCCCCGTCAATT  549
                ||||||||||||||||||||||||||||||||||||||||||||||||||||||||||||
Sbjct  1038809  TCTTCGCGTTGCATCGAATTAAACCACATGCTCCACCGCTTGTGCGGGCCCCCGTCAATT  1038750

Query  550      CATTTGAGTTTTAACCTTGCGGCCGTACTCCCCAGGCGGTCGACTTAACGCGTTAGCTCC  609
                ||||||||||||||||||||||||||||||||||||||||||||||||||||||||||||
Sbjct  1038749  CATTTGAGTTTTAACCTTGCGGCCGTACTCCCCAGGCGGTCGACTTAACGCGTTAGCTCC  1038690

Query  610      GGAAGCCACTCCTCAAGGGAACAACCTCCAAGTCGACATCGTTTACGGCGTGGACTACCA  669
                ||||||||||||||||||||||||||||||||||||||||||||||||||||||||||||
Sbjct  1038689  GGAAGCCACTCCTCAAGGGAACAACCTCCAAGTCGACATCGTTTACGGCGTGGACTACCA  1038630

Query  670      GGGTATCTAATCCTGTTTGCTCCCCACGCTTTCGCACCTGAGCGTCAGTCTTTGTCCAGG  729
                ||||||||||||||||||||||||||||||||||||||||||||||||||||||||||||
Sbjct  1038629  GGGTATCTAATCCTGTTTGCTCCCCACGCTTTCGCACCTGAGCGTCAGTCTTTGTCCAGG  1038570

Query  730      GGGCCGCCTTCGCCACCGGTATTCCTCCAGATCTCTACGCATTTCACCGCTACACCTGGA  789
                ||||||||||||||||||||||||||||||||||||||||||||||||||||||||||||
Sbjct  1038569  GGGCCGCCTTCGCCACCGGTATTCCTCCAGATCTCTACGCATTTCACCGCTACACCTGGA  1038510

Query  790      ATTCTACCCCCCTCTACAAGACTCAAGCCTGCCAGTTTCAAATGCAGTTCCCAGGTTAAG  849
                ||||||||||||||||||||||||||||||||||||||||||||||||||||||||||||
Sbjct  1038509  ATTCTACCCCCCTCTACAAGACTCAAGCCTGCCAGTTTCAAATGCAGTTCCCAGGTTAAG  1038450

Query  850      CCCGGGGGATTTCACATCTGACTTAACAGACCGCCTNCGTGCGCTTTACGCCCAGTAATT  909
                ||||||| |||||||||||||||||||||||||||| |||||||||||||||||||||||
Sbjct  1038449  CCCGGGG-ATTTCACATCTGACTTAACAGACCGCCTGCGTGCGCTTTACGCCCAGTAATT  1038391

Query  910      CCGATTAACGCTTGCACCCTCCGTATTACCGCGGCTGCTGGCACGGAGTTAGCCGGTGCT  969
                ||||||||||||||||||||||||||||||||||||||||||||||||||||||||||||
Sbjct  1038390  CCGATTAACGCTTGCACCCTCCGTATTACCGCGGCTGCTGGCACGGAGTTAGCCGGTGCT  1038331

Query  970      TCTTCTGCGGGTAACGTCAATCG  992
                |||||||||||||||||||||||
Sbjct  1038330  TCTTCTGCGGGTAACGTCAATCG  1038308
```

Range 5: 410982 to 411963GenBankGraphics

Next Match
Previous Match
First Match

Alignment statistics for match #5

| Score | Expect | Identities | Gaps | Strand | Frame |
| --- | --- | --- | --- | --- | --- |
| 1777 bits(962) | 0.0() | 974/983(99%) | 1/983(0%) | Plus/Minus |  |

Features:

```
Query  10      CTCCCGAAGGTTANGCTACCTACTTCTTTTGCANCCCACTCCCATGGTGTGACGGGCGGT  69
               ||||||||||||| ||||||||||||||||||| ||||||||||||||||||||||||||
Sbjct  411963  CTCCCGAAGGTTAAGCTACCTACTTCTTTTGCAACCCACTCCCATGGTGTGACGGGCGGT  411904

Query  70      GTGTACAAGGCCCGGGAACGTATTCACCGTGGCATTCTGATCCACGATTACTAGCGATTC  129
               ||||||||||||||||||||||||||||||||||||||||||||||||||||||||||||
Sbjct  411903  GTGTACAAGGCCCGGGAACGTATTCACCGTGGCATTCTGATCCACGATTACTAGCGATTC  411844

Query  130     CGACTTCACGGAGTCGAGTTGCAGACTCCGATCCGGACTACGACGCACTTTGTGAGGTCC  189
               ||||||||||||||||||||||||||||||||||||||||||| ||||||||||||||||
Sbjct  411843  CGACTTCACGGAGTCGAGTTGCAGACTCCGATCCGGACTACGATGCACTTTGTGAGGTCC  411784

Query  190     GCTTGCTCTCGCGAGGTCGCTTCTCTTTGTATGCGCCATTGTAGCACGTGTGTAGCCCTA  249
               ||||||||||||||||||||||||||||||||||||||||||||||||||||||||||||
Sbjct  411783  GCTTGCTCTCGCGAGGTCGCTTCTCTTTGTATGCGCCATTGTAGCACGTGTGTAGCCCTA  411724

Query  250     CTCGTAAGGGCCATGATGACTTGACGTCATCCCCACCTTCCTCCGGTTTATCACCGGCAG  309
               ||||||||||||||||||||||||||||||||||||||||||||||||||||||||||||
Sbjct  411723  CTCGTAAGGGCCATGATGACTTGACGTCATCCCCACCTTCCTCCGGTTTATCACCGGCAG  411664

Query  310     TCTCCTTTGAGTTCCCGACCGAATCGCTGGCAACAAAGGATAAGGGTTGCGCTCGTTGCG  369
               ||||||||||||||||||||||||||||||||||||||||||||||||||||||||||||
Sbjct  411663  TCTCCTTTGAGTTCCCGACCGAATCGCTGGCAACAAAGGATAAGGGTTGCGCTCGTTGCG  411604

Query  370     GGACTTAACCCAACATTTCACAACACGAGCTGACGACAGCCATGCAGCACCTGTCTCANN  429
               ||||||||||||||||||||||||||||||||||||||||||||||||||||||||||  
Sbjct  411603  GGACTTAACCCAACATTTCACAACACGAGCTGACGACAGCCATGCAGCACCTGTCTCACG  411544

Query  430     GTTCCCGAAGGCACTAAGGCATCTCTGNNAAATTCCGTGGATGTCAAGAGTAGGTAAGGT  489
               |||||||||||||||||||||||||||  |||||||||||||||||||||||||||||||
Sbjct  411543  GTTCCCGAAGGCACTAAGGCATCTCTGCCAAATTCCGTGGATGTCAAGAGTAGGTAAGGT  411484

Query  490     TCTTCGCGTTGCATCGAATTAAACCACATGCTCCACCGCTTGTGCGGGCCCCCGTCAATT  549
               ||||||||||||||||||||||||||||||||||||||||||||||||||||||||||||
Sbjct  411483  TCTTCGCGTTGCATCGAATTAAACCACATGCTCCACCGCTTGTGCGGGCCCCCGTCAATT  411424

Query  550     CATTTGAGTTTTAACCTTGCGGCCGTACTCCCCAGGCGGTCGACTTAACGCGTTAGCTCC  609
               ||||||||||||||||||||||||||||||||||||||||||||||||||||||||||||
Sbjct  411423  CATTTGAGTTTTAACCTTGCGGCCGTACTCCCCAGGCGGTCGACTTAACGCGTTAGCTCC  411364

Query  610     GGAAGCCACTCCTCAAGGGAACAACCTCCAAGTCGACATCGTTTACGGCGTGGACTACCA  669
               ||||||||||||||||||||||||||||||||||||||||||||||||||||||||||||
Sbjct  411363  GGAAGCCACTCCTCAAGGGAACAACCTCCAAGTCGACATCGTTTACGGCGTGGACTACCA  411304

Query  670     GGGTATCTAATCCTGTTTGCTCCCCACGCTTTCGCACCTGAGCGTCAGTCTTTGTCCAGG  729
               ||||||||||||||||||||||||||||||||||||||||||||||||||||||||||||
Sbjct  411303  GGGTATCTAATCCTGTTTGCTCCCCACGCTTTCGCACCTGAGCGTCAGTCTTTGTCCAGG  411244

Query  730     GGGCCGCCTTCGCCACCGGTATTCCTCCAGATCTCTACGCATTTCACCGCTACACCTGGA  789
               ||||||||||||||||||||||||||||||||||||||||||||||||||||||||||||
Sbjct  411243  GGGCCGCCTTCGCCACCGGTATTCCTCCAGATCTCTACGCATTTCACCGCTACACCTGGA  411184

Query  790     ATTCTACCCCCCTCTACAAGACTCAAGCCTGCCAGTTTCAAATGCAGTTCCCAGGTTAAG  849
               ||||||||||||||||||||||||||||||||||||||||||||||||||||||||||||
Sbjct  411183  ATTCTACCCCCCTCTACAAGACTCAAGCCTGCCAGTTTCAAATGCAGTTCCCAGGTTAAG  411124

Query  850     CCCGGGGGATTTCACATCTGACTTAACAGACCGCCTNCGTGCGCTTTACGCCCAGTAATT  909
               ||||||| |||||||||||||||||||||||||||| |||||||||||||||||||||||
Sbjct  411123  CCCGGGG-ATTTCACATCTGACTTAACAGACCGCCTGCGTGCGCTTTACGCCCAGTAATT  411065

Query  910     CCGATTAACGCTTGCACCCTCCGTATTACCGCGGCTGCTGGCACGGAGTTAGCCGGTGCT  969
               ||||||||||||||||||||||||||||||||||||||||||||||||||||||||||||
Sbjct  411064  CCGATTAACGCTTGCACCCTCCGTATTACCGCGGCTGCTGGCACGGAGTTAGCCGGTGCT  411005

Query  970     TCTTCTGCGGGTAACGTCAATCG  992
               |||||||||||||||||||||||
Sbjct  411004  TCTTCTGCGGGTAACGTCAATCG  410982
```

Range 6: 3350392 to 3351373GenBankGraphics

Next Match
Previous Match
First Match

Alignment statistics for match #6

| Score | Expect | Identities | Gaps | Strand | Frame |
| --- | --- | --- | --- | --- | --- |
| 1777 bits(962) | 0.0() | 974/983(99%) | 1/983(0%) | Plus/Plus |  |

Features:

```
Query  10       CTCCCGAAGGTTANGCTACCTACTTCTTTTGCANCCCACTCCCATGGTGTGACGGGCGGT  69
                ||||||||||||| ||||||||||||||||||| ||||||||||||||||||||||||||
Sbjct  3350392  CTCCCGAAGGTTAAGCTACCTACTTCTTTTGCAACCCACTCCCATGGTGTGACGGGCGGT  3350451

Query  70       GTGTACAAGGCCCGGGAACGTATTCACCGTGGCATTCTGATCCACGATTACTAGCGATTC  129
                ||||||||||||||||||||||||||||||||||||||||||||||||||||||||||||
Sbjct  3350452  GTGTACAAGGCCCGGGAACGTATTCACCGTGGCATTCTGATCCACGATTACTAGCGATTC  3350511

Query  130      CGACTTCACGGAGTCGAGTTGCAGACTCCGATCCGGACTACGACGCACTTTGTGAGGTCC  189
                ||||||||||||||||||||||||||||||||||||||||||||||||||||||||||||
Sbjct  3350512  CGACTTCACGGAGTCGAGTTGCAGACTCCGATCCGGACTACGACGCACTTTGTGAGGTCC  3350571

Query  190      GCTTGCTCTCGCGAGGTCGCTTCTCTTTGTATGCGCCATTGTAGCACGTGTGTAGCCCTA  249
                ||||||||||||||||||||||||||||||||||||||||||||||||||||||||||||
Sbjct  3350572  GCTTGCTCTCGCGAGGTCGCTTCTCTTTGTATGCGCCATTGTAGCACGTGTGTAGCCCTA  3350631

Query  250      CTCGTAAGGGCCATGATGACTTGACGTCATCCCCACCTTCCTCCGGTTTATCACCGGCAG  309
                ||||||||||||||||||||||||||||||||||||||||||||||||||||||||||||
Sbjct  3350632  CTCGTAAGGGCCATGATGACTTGACGTCATCCCCACCTTCCTCCGGTTTATCACCGGCAG  3350691

Query  310      TCTCCTTTGAGTTCCCGACCGAATCGCTGGCAACAAAGGATAAGGGTTGCGCTCGTTGCG  369
                ||||||||||||||||||||||||||||||||||||||||||||||||||||||||||||
Sbjct  3350692  TCTCCTTTGAGTTCCCGACCGAATCGCTGGCAACAAAGGATAAGGGTTGCGCTCGTTGCG  3350751

Query  370      GGACTTAACCCAACATTTCACAACACGAGCTGACGACAGCCATGCAGCACCTGTCTCANN  429
                ||||||||||||||||||||||||||||||||||||||||||||||||||||||||||  
Sbjct  3350752  GGACTTAACCCAACATTTCACAACACGAGCTGACGACAGCCATGCAGCACCTGTCTCACG  3350811

Query  430      GTTCCCGAAGGCACTAAGGCATCTCTGNNAAATTCCGTGGATGTCAAGAGTAGGTAAGGT  489
                |||||||||||||||||||||||||||   ||||||||||||||||||||||||||||||
Sbjct  3350812  GTTCCCGAAGGCACTAAGGCATCTCTGCCGAATTCCGTGGATGTCAAGAGTAGGTAAGGT  3350871

Query  490      TCTTCGCGTTGCATCGAATTAAACCACATGCTCCACCGCTTGTGCGGGCCCCCGTCAATT  549
                ||||||||||||||||||||||||||||||||||||||||||||||||||||||||||||
Sbjct  3350872  TCTTCGCGTTGCATCGAATTAAACCACATGCTCCACCGCTTGTGCGGGCCCCCGTCAATT  3350931

Query  550      CATTTGAGTTTTAACCTTGCGGCCGTACTCCCCAGGCGGTCGACTTAACGCGTTAGCTCC  609
                ||||||||||||||||||||||||||||||||||||||||||||||||||||||||||||
Sbjct  3350932  CATTTGAGTTTTAACCTTGCGGCCGTACTCCCCAGGCGGTCGACTTAACGCGTTAGCTCC  3350991

Query  610      GGAAGCCACTCCTCAAGGGAACAACCTCCAAGTCGACATCGTTTACGGCGTGGACTACCA  669
                ||||||||||||||||||||||||||||||||||||||||||||||||||||||||||||
Sbjct  3350992  GGAAGCCACTCCTCAAGGGAACAACCTCCAAGTCGACATCGTTTACGGCGTGGACTACCA  3351051

Query  670      GGGTATCTAATCCTGTTTGCTCCCCACGCTTTCGCACCTGAGCGTCAGTCTTTGTCCAGG  729
                ||||||||||||||||||||||||||||||||||||||||||||||||||||||||||||
Sbjct  3351052  GGGTATCTAATCCTGTTTGCTCCCCACGCTTTCGCACCTGAGCGTCAGTCTTTGTCCAGG  3351111

Query  730      GGGCCGCCTTCGCCACCGGTATTCCTCCAGATCTCTACGCATTTCACCGCTACACCTGGA  789
                ||||||||||||||||||||||||||||||||||||||||||||||||||||||||||||
Sbjct  3351112  GGGCCGCCTTCGCCACCGGTATTCCTCCAGATCTCTACGCATTTCACCGCTACACCTGGA  3351171

Query  790      ATTCTACCCCCCTCTACAAGACTCAAGCCTGCCAGTTTCAAATGCAGTTCCCAGGTTAAG  849
                ||||||||||||||||||||||||||||||||||||||||||||||||||||||||||||
Sbjct  3351172  ATTCTACCCCCCTCTACAAGACTCAAGCCTGCCAGTTTCAAATGCAGTTCCCAGGTTAAG  3351231

Query  850      CCCGGGGGATTTCACATCTGACTTAACAGACCGCCTNCGTGCGCTTTACGCCCAGTAATT  909
                ||| |||||||||||||||||||||||||||||||| |||||||||||||||||||||||
Sbjct  3351232  CCC-GGGGATTTCACATCTGACTTAACAGACCGCCTGCGTGCGCTTTACGCCCAGTAATT  3351290

Query  910      CCGATTAACGCTTGCACCCTCCGTATTACCGCGGCTGCTGGCACGGAGTTAGCCGGTGCT  969
                ||||||||||||||||||||||||||||||||||||||||||||||||||||||||||||
Sbjct  3351291  CCGATTAACGCTTGCACCCTCCGTATTACCGCGGCTGCTGGCACGGAGTTAGCCGGTGCT  3351350

Query  970      TCTTCTGCGGGTAACGTCAATCG  992
                |||||||||||||||||||||||
Sbjct  3351351  TCTTCTGCGGGTAACGTCAATCG  3351373
```

Range 7: 3833149 to 3834130GenBankGraphics

Next Match
Previous Match
First Match

Alignment statistics for match #7

| Score | Expect | Identities | Gaps | Strand | Frame |
| --- | --- | --- | --- | --- | --- |
| 1777 bits(962) | 0.0() | 974/983(99%) | 1/983(0%) | Plus/Plus |  |

Features:

```
Query  10       CTCCCGAAGGTTANGCTACCTACTTCTTTTGCANCCCACTCCCATGGTGTGACGGGCGGT  69
                ||||||||||||| ||||||||||||||||||| ||||||||||||||||||||||||||
Sbjct  3833149  CTCCCGAAGGTTAAGCTACCTACTTCTTTTGCAACCCACTCCCATGGTGTGACGGGCGGT  3833208

Query  70       GTGTACAAGGCCCGGGAACGTATTCACCGTGGCATTCTGATCCACGATTACTAGCGATTC  129
                ||||||||||||||||||||||||||||||||||||||||||||||||||||||||||||
Sbjct  3833209  GTGTACAAGGCCCGGGAACGTATTCACCGTGGCATTCTGATCCACGATTACTAGCGATTC  3833268

Query  130      CGACTTCACGGAGTCGAGTTGCAGACTCCGATCCGGACTACGACGCACTTTGTGAGGTCC  189
                ||||||||||||||||||||||||||||||||||||||||||||||||||||||||||||
Sbjct  3833269  CGACTTCACGGAGTCGAGTTGCAGACTCCGATCCGGACTACGACGCACTTTGTGAGGTCC  3833328

Query  190      GCTTGCTCTCGCGAGGTCGCTTCTCTTTGTATGCGCCATTGTAGCACGTGTGTAGCCCTA  249
                ||||||||||||||||||||||||||||||||||||||||||||||||||||||||||||
Sbjct  3833329  GCTTGCTCTCGCGAGGTCGCTTCTCTTTGTATGCGCCATTGTAGCACGTGTGTAGCCCTA  3833388

Query  250      CTCGTAAGGGCCATGATGACTTGACGTCATCCCCACCTTCCTCCGGTTTATCACCGGCAG  309
                ||||||||||||||||||||||||||||||||||||||||||||||||||||||||||||
Sbjct  3833389  CTCGTAAGGGCCATGATGACTTGACGTCATCCCCACCTTCCTCCGGTTTATCACCGGCAG  3833448

Query  310      TCTCCTTTGAGTTCCCGACCGAATCGCTGGCAACAAAGGATAAGGGTTGCGCTCGTTGCG  369
                ||||||||||||||||||||||||||||||||||||||||||||||||||||||||||||
Sbjct  3833449  TCTCCTTTGAGTTCCCGACCGAATCGCTGGCAACAAAGGATAAGGGTTGCGCTCGTTGCG  3833508

Query  370      GGACTTAACCCAACATTTCACAACACGAGCTGACGACAGCCATGCAGCACCTGTCTCANN  429
                ||||||||||||||||||||||||||||||||||||||||||||||||||||||||||  
Sbjct  3833509  GGACTTAACCCAACATTTCACAACACGAGCTGACGACAGCCATGCAGCACCTGTCTCACG  3833568

Query  430      GTTCCCGAAGGCACTAAGGCATCTCTGNNAAATTCCGTGGATGTCAAGAGTAGGTAAGGT  489
                |||||||||||||||||||||||||||   ||||||||||||||||||||||||||||||
Sbjct  3833569  GTTCCCGAAGGCACTAAGGCATCTCTGCCGAATTCCGTGGATGTCAAGAGTAGGTAAGGT  3833628

Query  490      TCTTCGCGTTGCATCGAATTAAACCACATGCTCCACCGCTTGTGCGGGCCCCCGTCAATT  549
                ||||||||||||||||||||||||||||||||||||||||||||||||||||||||||||
Sbjct  3833629  TCTTCGCGTTGCATCGAATTAAACCACATGCTCCACCGCTTGTGCGGGCCCCCGTCAATT  3833688

Query  550      CATTTGAGTTTTAACCTTGCGGCCGTACTCCCCAGGCGGTCGACTTAACGCGTTAGCTCC  609
                ||||||||||||||||||||||||||||||||||||||||||||||||||||||||||||
Sbjct  3833689  CATTTGAGTTTTAACCTTGCGGCCGTACTCCCCAGGCGGTCGACTTAACGCGTTAGCTCC  3833748

Query  610      GGAAGCCACTCCTCAAGGGAACAACCTCCAAGTCGACATCGTTTACGGCGTGGACTACCA  669
                ||||||||||||||||||||||||||||||||||||||||||||||||||||||||||||
Sbjct  3833749  GGAAGCCACTCCTCAAGGGAACAACCTCCAAGTCGACATCGTTTACGGCGTGGACTACCA  3833808

Query  670      GGGTATCTAATCCTGTTTGCTCCCCACGCTTTCGCACCTGAGCGTCAGTCTTTGTCCAGG  729
                ||||||||||||||||||||||||||||||||||||||||||||||||||||||||||||
Sbjct  3833809  GGGTATCTAATCCTGTTTGCTCCCCACGCTTTCGCACCTGAGCGTCAGTCTTTGTCCAGG  3833868

Query  730      GGGCCGCCTTCGCCACCGGTATTCCTCCAGATCTCTACGCATTTCACCGCTACACCTGGA  789
                ||||||||||||||||||||||||||||||||||||||||||||||||||||||||||||
Sbjct  3833869  GGGCCGCCTTCGCCACCGGTATTCCTCCAGATCTCTACGCATTTCACCGCTACACCTGGA  3833928

Query  790      ATTCTACCCCCCTCTACAAGACTCAAGCCTGCCAGTTTCAAATGCAGTTCCCAGGTTAAG  849
                ||||||||||||||||||||||||||||||||||||||||||||||||||||||||||||
Sbjct  3833929  ATTCTACCCCCCTCTACAAGACTCAAGCCTGCCAGTTTCAAATGCAGTTCCCAGGTTAAG  3833988

Query  850      CCCGGGGGATTTCACATCTGACTTAACAGACCGCCTNCGTGCGCTTTACGCCCAGTAATT  909
                ||| |||||||||||||||||||||||||||||||| |||||||||||||||||||||||
Sbjct  3833989  CCC-GGGGATTTCACATCTGACTTAACAGACCGCCTGCGTGCGCTTTACGCCCAGTAATT  3834047

Query  910      CCGATTAACGCTTGCACCCTCCGTATTACCGCGGCTGCTGGCACGGAGTTAGCCGGTGCT  969
                ||||||||||||||||||||||||||||||||||||||||||||||||||||||||||||
Sbjct  3834048  CCGATTAACGCTTGCACCCTCCGTATTACCGCGGCTGCTGGCACGGAGTTAGCCGGTGCT  3834107

Query  970      TCTTCTGCGGGTAACGTCAATCG  992
                |||||||||||||||||||||||
Sbjct  3834108  TCTTCTGCGGGTAACGTCAATCG  3834130
```

Download

FASTA (complete sequence)

FASTA (aligned sequences)

GenBank (complete sequence)

Continue
Cancel

GenBankGraphics

Next
Previous
Descriptions

Pantoea agglomerans strain AL14 16S ribosomal RNA gene, partial sequence

Sequence ID: MG819218.1Length: 1416Number of Matches: 1

Related Information

Range 1: 386 to 1368GenBankGraphics

Next Match
Previous Match
First Match

Alignment statistics for match #1

| Score | Expect | Identities | Gaps | Strand | Frame |
| --- | --- | --- | --- | --- | --- |
| 1784 bits(966) | 0.0() | 976/984(99%) | 1/984(0%) | Plus/Minus |  |

Features:

```
Query  10    CTCCCGAAGGTTANGCTACCTACTTCTTTTGCANCCCACTCCCATGGTGTGACGGGCGGT  69
             ||||||||||||| ||||||||||||||||||| ||||||||||||||||||||||||||
Sbjct  1368  CTCCCGAAGGTTAAGCTACCTACTTCTTTTGCAACCCACTCCCATGGTGTGACGGGCGGT  1309

Query  70    GTGTACAAGGCCCGGGAACGTATTCACCGTGGCATTCTGATCCACGATTACTAGCGATTC  129
             ||||||||||||||||||||||||||||||||||||||||||||||||||||||||||||
Sbjct  1308  GTGTACAAGGCCCGGGAACGTATTCACCGTGGCATTCTGATCCACGATTACTAGCGATTC  1249

Query  130   CGACTTCACGGAGTCGAGTTGCAGACTCCGATCCGGACTACGACGCACTTTGTGAGGTCC  189
             ||||||||||||||||||||||||||||||||||||||||||||||||||||||||||||
Sbjct  1248  CGACTTCACGGAGTCGAGTTGCAGACTCCGATCCGGACTACGACGCACTTTGTGAGGTCC  1189

Query  190   GCTTGCTCTCGCGAGGTCGCTTCTCTTTGTATGCGCCATTGTAGCACGTGTGTAGCCCTA  249
             ||||||||||||||||||||||||||||||||||||||||||||||||||||||||||||
Sbjct  1188  GCTTGCTCTCGCGAGGTCGCTTCTCTTTGTATGCGCCATTGTAGCACGTGTGTAGCCCTA  1129

Query  250   CTCGTAAGGGCCATGATGACTTGACGTCATCCCCACCTTCCTCCGGTTTATCACCGGCAG  309
             ||||||||||||||||||||||||||||||||||||||||||||||||||||||||||||
Sbjct  1128  CTCGTAAGGGCCATGATGACTTGACGTCATCCCCACCTTCCTCCGGTTTATCACCGGCAG  1069

Query  310   TCTCCTTTGAGTTCCCGACCGAATCGCTGGCAACAAAGGATAAGGGTTGCGCTCGTTGCG  369
             ||||||||||||||||||||||||||||||||||||||||||||||||||||||||||||
Sbjct  1068  TCTCCTTTGAGTTCCCGACCGAATCGCTGGCAACAAAGGATAAGGGTTGCGCTCGTTGCG  1009

Query  370   GGACTTAACCCAACATTTCACAACACGAGCTGACGACAGCCATGCAGCACCTGTCTCANN  429
             ||||||||||||||||||||||||||||||||||||||||||||||||||||||||||  
Sbjct  1008  GGACTTAACCCAACATTTCACAACACGAGCTGACGACAGCCATGCAGCACCTGTCTCACG  949

Query  430   GTTCCCGAAGGCACTAAGGCATCTCTGNNAAATTCCGTGGATGTCAAGAGTAGGTAAGGT  489
             |||||||||||||||||||||||||||  |||||||||||||||||||||||||||||||
Sbjct  948   GTTCCCGAAGGCACTAAGGCATCTCTGCCAAATTCCGTGGATGTCAAGAGTAGGTAAGGT  889

Query  490   TCTTCGCGTTGCATCGAATTAAACCACATGCTCCACCGCTTGTGCGGGCCCCCGTCAATT  549
             ||||||||||||||||||||||||||||||||||||||||||||||||||||||||||||
Sbjct  888   TCTTCGCGTTGCATCGAATTAAACCACATGCTCCACCGCTTGTGCGGGCCCCCGTCAATT  829

Query  550   CATTTGAGTTTTAACCTTGCGGCCGTACTCCCCAGGCGGTCGACTTAACGCGTTAGCTCC  609
             ||||||||||||||||||||||||||||||||||||||||||||||||||||||||||||
Sbjct  828   CATTTGAGTTTTAACCTTGCGGCCGTACTCCCCAGGCGGTCGACTTAACGCGTTAGCTCC  769

Query  610   GGAAGCCACTCCTCAAGGGAACAACCTCCAAGTCGACATCGTTTACGGCGTGGACTACCA  669
             ||||||||||||||||||||||||||||||||||||||||||||||||||||||||||||
Sbjct  768   GGAAGCCACTCCTCAAGGGAACAACCTCCAAGTCGACATCGTTTACGGCGTGGACTACCA  709

Query  670   GGGTATCTAATCCTGTTTGCTCCCCACGCTTTCGCACCTGAGCGTCAGTCTTTGTCCAGG  729
             ||||||||||||||||||||||||||||||||||||||||||||||||||||||||||||
Sbjct  708   GGGTATCTAATCCTGTTTGCTCCCCACGCTTTCGCACCTGAGCGTCAGTCTTTGTCCAGG  649

Query  730   GGGCCGCCTTCGCCACCGGTATTCCTCCAGATCTCTACGCATTTCACCGCTACACCTGGA  789
             ||||||||||||||||||||||||||||||||||||||||||||||||||||||||||||
Sbjct  648   GGGCCGCCTTCGCCACCGGTATTCCTCCAGATCTCTACGCATTTCACCGCTACACCTGGA  589

Query  790   ATTCTACCCCCCTCTACAAGACTCAAGCCTGCCAGTTTCAAATGCAGTTCCCAGGTTAAG  849
             ||||||||||||||||||||||||||||||||||||||||||||||||||||||||||||
Sbjct  588   ATTCTACCCCCCTCTACAAGACTCAAGCCTGCCAGTTTCAAATGCAGTTCCCAGGTTAAG  529

Query  850   CCCGGGGGATTTCACATCTGACTTAACAGACCGCCTNCGTGCGCTTTACGCCCAGTAATT  909
             ||||||| |||||||||||||||||||||||||||| |||||||||||||||||||||||
Sbjct  528   CCCGGGG-ATTTCACATCTGACTTAACAGACCGCCTGCGTGCGCTTTACGCCCAGTAATT  470

Query  910   CCGATTAACGCTTGCACCCTCCGTATTACCGCGGCTGCTGGCACGGAGTTAGCCGGTGCT  969
             ||||||||||||||||||||||||||||||||||||||||||||||||||||||||||||
Sbjct  469   CCGATTAACGCTTGCACCCTCCGTATTACCGCGGCTGCTGGCACGGAGTTAGCCGGTGCT  410

Query  970   TCTTCTGCGGGTAACGTCAATCGA  993
             ||||||||||||||||||||||||
Sbjct  409   TCTTCTGCGGGTAACGTCAATCGA  386
```

Download

FASTA (complete sequence)

FASTA (aligned sequences)

GenBank (complete sequence)

Continue
Cancel

GenBankGraphics

Next
Previous
Descriptions

Pantoea agglomerans strain Rk92 16S ribosomal RNA gene, partial sequence

Sequence ID: MG547769.1Length: 1395Number of Matches: 1

Related Information

Range 1: 361 to 1343GenBankGraphics

Next Match
Previous Match
First Match

Alignment statistics for match #1

| Score | Expect | Identities | Gaps | Strand | Frame |
| --- | --- | --- | --- | --- | --- |
| 1784 bits(966) | 0.0() | 976/984(99%) | 1/984(0%) | Plus/Minus |  |

Features:

```
Query  10    CTCCCGAAGGTTANGCTACCTACTTCTTTTGCANCCCACTCCCATGGTGTGACGGGCGGT  69
             ||||||||||||| ||||||||||||||||||| ||||||||||||||||||||||||||
Sbjct  1343  CTCCCGAAGGTTAAGCTACCTACTTCTTTTGCAACCCACTCCCATGGTGTGACGGGCGGT  1284

Query  70    GTGTACAAGGCCCGGGAACGTATTCACCGTGGCATTCTGATCCACGATTACTAGCGATTC  129
             ||||||||||||||||||||||||||||||||||||||||||||||||||||||||||||
Sbjct  1283  GTGTACAAGGCCCGGGAACGTATTCACCGTGGCATTCTGATCCACGATTACTAGCGATTC  1224

Query  130   CGACTTCACGGAGTCGAGTTGCAGACTCCGATCCGGACTACGACGCACTTTGTGAGGTCC  189
             ||||||||||||||||||||||||||||||||||||||||||||||||||||||||||||
Sbjct  1223  CGACTTCACGGAGTCGAGTTGCAGACTCCGATCCGGACTACGACGCACTTTGTGAGGTCC  1164

Query  190   GCTTGCTCTCGCGAGGTCGCTTCTCTTTGTATGCGCCATTGTAGCACGTGTGTAGCCCTA  249
             ||||||||||||||||||||||||||||||||||||||||||||||||||||||||||||
Sbjct  1163  GCTTGCTCTCGCGAGGTCGCTTCTCTTTGTATGCGCCATTGTAGCACGTGTGTAGCCCTA  1104

Query  250   CTCGTAAGGGCCATGATGACTTGACGTCATCCCCACCTTCCTCCGGTTTATCACCGGCAG  309
             ||||||||||||||||||||||||||||||||||||||||||||||||||||||||||||
Sbjct  1103  CTCGTAAGGGCCATGATGACTTGACGTCATCCCCACCTTCCTCCGGTTTATCACCGGCAG  1044

Query  310   TCTCCTTTGAGTTCCCGACCGAATCGCTGGCAACAAAGGATAAGGGTTGCGCTCGTTGCG  369
             ||||||||||||||||||||||||||||||||||||||||||||||||||||||||||||
Sbjct  1043  TCTCCTTTGAGTTCCCGACCGAATCGCTGGCAACAAAGGATAAGGGTTGCGCTCGTTGCG  984

Query  370   GGACTTAACCCAACATTTCACAACACGAGCTGACGACAGCCATGCAGCACCTGTCTCANN  429
             ||||||||||||||||||||||||||||||||||||||||||||||||||||||||||  
Sbjct  983   GGACTTAACCCAACATTTCACAACACGAGCTGACGACAGCCATGCAGCACCTGTCTCACG  924

Query  430   GTTCCCGAAGGCACTAAGGCATCTCTGNNAAATTCCGTGGATGTCAAGAGTAGGTAAGGT  489
             |||||||||||||||||||||||||||  |||||||||||||||||||||||||||||||
Sbjct  923   GTTCCCGAAGGCACTAAGGCATCTCTGCCAAATTCCGTGGATGTCAAGAGTAGGTAAGGT  864

Query  490   TCTTCGCGTTGCATCGAATTAAACCACATGCTCCACCGCTTGTGCGGGCCCCCGTCAATT  549
             ||||||||||||||||||||||||||||||||||||||||||||||||||||||||||||
Sbjct  863   TCTTCGCGTTGCATCGAATTAAACCACATGCTCCACCGCTTGTGCGGGCCCCCGTCAATT  804

Query  550   CATTTGAGTTTTAACCTTGCGGCCGTACTCCCCAGGCGGTCGACTTAACGCGTTAGCTCC  609
             ||||||||||||||||||||||||||||||||||||||||||||||||||||||||||||
Sbjct  803   CATTTGAGTTTTAACCTTGCGGCCGTACTCCCCAGGCGGTCGACTTAACGCGTTAGCTCC  744

Query  610   GGAAGCCACTCCTCAAGGGAACAACCTCCAAGTCGACATCGTTTACGGCGTGGACTACCA  669
             ||||||||||||||||||||||||||||||||||||||||||||||||||||||||||||
Sbjct  743   GGAAGCCACTCCTCAAGGGAACAACCTCCAAGTCGACATCGTTTACGGCGTGGACTACCA  684

Query  670   GGGTATCTAATCCTGTTTGCTCCCCACGCTTTCGCACCTGAGCGTCAGTCTTTGTCCAGG  729
             ||||||||||||||||||||||||||||||||||||||||||||||||||||||||||||
Sbjct  683   GGGTATCTAATCCTGTTTGCTCCCCACGCTTTCGCACCTGAGCGTCAGTCTTTGTCCAGG  624

Query  730   GGGCCGCCTTCGCCACCGGTATTCCTCCAGATCTCTACGCATTTCACCGCTACACCTGGA  789
             ||||||||||||||||||||||||||||||||||||||||||||||||||||||||||||
Sbjct  623   GGGCCGCCTTCGCCACCGGTATTCCTCCAGATCTCTACGCATTTCACCGCTACACCTGGA  564

Query  790   ATTCTACCCCCCTCTACAAGACTCAAGCCTGCCAGTTTCAAATGCAGTTCCCAGGTTAAG  849
             ||||||||||||||||||||||||||||||||||||||||||||||||||||||||||||
Sbjct  563   ATTCTACCCCCCTCTACAAGACTCAAGCCTGCCAGTTTCAAATGCAGTTCCCAGGTTAAG  504

Query  850   CCCGGGGGATTTCACATCTGACTTAACAGACCGCCTNCGTGCGCTTTACGCCCAGTAATT  909
             ||||||| |||||||||||||||||||||||||||| |||||||||||||||||||||||
Sbjct  503   CCCGGGG-ATTTCACATCTGACTTAACAGACCGCCTGCGTGCGCTTTACGCCCAGTAATT  445

Query  910   CCGATTAACGCTTGCACCCTCCGTATTACCGCGGCTGCTGGCACGGAGTTAGCCGGTGCT  969
             ||||||||||||||||||||||||||||||||||||||||||||||||||||||||||||
Sbjct  444   CCGATTAACGCTTGCACCCTCCGTATTACCGCGGCTGCTGGCACGGAGTTAGCCGGTGCT  385

Query  970   TCTTCTGCGGGTAACGTCAATCGA  993
             ||||||||||||||||||||||||
Sbjct  384   TCTTCTGCGGGTAACGTCAATCGA  361
```

Download

FASTA (complete sequence)

FASTA (aligned sequences)

GenBank (complete sequence)

Continue
Cancel

GenBankGraphics

Next
Previous
Descriptions

Pantoea agglomerans strain P3FNa\_1 16S ribosomal RNA gene, partial sequence

Sequence ID: KX891531.1Length: 1113Number of Matches: 1

Related Information

Range 1: 107 to 1089GenBankGraphics

Next Match
Previous Match
First Match

Alignment statistics for match #1

| Score | Expect | Identities | Gaps | Strand | Frame |
| --- | --- | --- | --- | --- | --- |
| 1784 bits(966) | 0.0() | 976/984(99%) | 1/984(0%) | Plus/Minus |  |

Features:

```
Query  10    CTCCCGAAGGTTANGCTACCTACTTCTTTTGCANCCCACTCCCATGGTGTGACGGGCGGT  69
             ||||||||||||| ||||||||||||||||||| ||||||||||||||||||||||||||
Sbjct  1089  CTCCCGAAGGTTAAGCTACCTACTTCTTTTGCAACCCACTCCCATGGTGTGACGGGCGGT  1030

Query  70    GTGTACAAGGCCCGGGAACGTATTCACCGTGGCATTCTGATCCACGATTACTAGCGATTC  129
             ||||||||||||||||||||||||||||||||||||||||||||||||||||||||||||
Sbjct  1029  GTGTACAAGGCCCGGGAACGTATTCACCGTGGCATTCTGATCCACGATTACTAGCGATTC  970

Query  130   CGACTTCACGGAGTCGAGTTGCAGACTCCGATCCGGACTACGACGCACTTTGTGAGGTCC  189
             ||||||||||||||||||||||||||||||||||||||||||||||||||||||||||||
Sbjct  969   CGACTTCACGGAGTCGAGTTGCAGACTCCGATCCGGACTACGACGCACTTTGTGAGGTCC  910

Query  190   GCTTGCTCTCGCGAGGTCGCTTCTCTTTGTATGCGCCATTGTAGCACGTGTGTAGCCCTA  249
             ||||||||||||||||||||||||||||||||||||||||||||||||||||||||||||
Sbjct  909   GCTTGCTCTCGCGAGGTCGCTTCTCTTTGTATGCGCCATTGTAGCACGTGTGTAGCCCTA  850

Query  250   CTCGTAAGGGCCATGATGACTTGACGTCATCCCCACCTTCCTCCGGTTTATCACCGGCAG  309
             ||||||||||||||||||||||||||||||||||||||||||||||||||||||||||||
Sbjct  849   CTCGTAAGGGCCATGATGACTTGACGTCATCCCCACCTTCCTCCGGTTTATCACCGGCAG  790

Query  310   TCTCCTTTGAGTTCCCGACCGAATCGCTGGCAACAAAGGATAAGGGTTGCGCTCGTTGCG  369
             ||||||||||||||||||||||||||||||||||||||||||||||||||||||||||||
Sbjct  789   TCTCCTTTGAGTTCCCGACCGAATCGCTGGCAACAAAGGATAAGGGTTGCGCTCGTTGCG  730

Query  370   GGACTTAACCCAACATTTCACAACACGAGCTGACGACAGCCATGCAGCACCTGTCTCANN  429
             ||||||||||||||||||||||||||||||||||||||||||||||||||||||||||  
Sbjct  729   GGACTTAACCCAACATTTCACAACACGAGCTGACGACAGCCATGCAGCACCTGTCTCACG  670

Query  430   GTTCCCGAAGGCACTAAGGCATCTCTGNNAAATTCCGTGGATGTCAAGAGTAGGTAAGGT  489
             |||||||||||||||||||||||||||  |||||||||||||||||||||||||||||||
Sbjct  669   GTTCCCGAAGGCACTAAGGCATCTCTGCCAAATTCCGTGGATGTCAAGAGTAGGTAAGGT  610

Query  490   TCTTCGCGTTGCATCGAATTAAACCACATGCTCCACCGCTTGTGCGGGCCCCCGTCAATT  549
             ||||||||||||||||||||||||||||||||||||||||||||||||||||||||||||
Sbjct  609   TCTTCGCGTTGCATCGAATTAAACCACATGCTCCACCGCTTGTGCGGGCCCCCGTCAATT  550

Query  550   CATTTGAGTTTTAACCTTGCGGCCGTACTCCCCAGGCGGTCGACTTAACGCGTTAGCTCC  609
             ||||||||||||||||||||||||||||||||||||||||||||||||||||||||||||
Sbjct  549   CATTTGAGTTTTAACCTTGCGGCCGTACTCCCCAGGCGGTCGACTTAACGCGTTAGCTCC  490

Query  610   GGAAGCCACTCCTCAAGGGAACAACCTCCAAGTCGACATCGTTTACGGCGTGGACTACCA  669
             ||||||||||||||||||||||||||||||||||||||||||||||||||||||||||||
Sbjct  489   GGAAGCCACTCCTCAAGGGAACAACCTCCAAGTCGACATCGTTTACGGCGTGGACTACCA  430

Query  670   GGGTATCTAATCCTGTTTGCTCCCCACGCTTTCGCACCTGAGCGTCAGTCTTTGTCCAGG  729
             ||||||||||||||||||||||||||||||||||||||||||||||||||||||||||||
Sbjct  429   GGGTATCTAATCCTGTTTGCTCCCCACGCTTTCGCACCTGAGCGTCAGTCTTTGTCCAGG  370

Query  730   GGGCCGCCTTCGCCACCGGTATTCCTCCAGATCTCTACGCATTTCACCGCTACACCTGGA  789
             ||||||||||||||||||||||||||||||||||||||||||||||||||||||||||||
Sbjct  369   GGGCCGCCTTCGCCACCGGTATTCCTCCAGATCTCTACGCATTTCACCGCTACACCTGGA  310

Query  790   ATTCTACCCCCCTCTACAAGACTCAAGCCTGCCAGTTTCAAATGCAGTTCCCAGGTTAAG  849
             ||||||||||||||||||||||||||||||||||||||||||||||||||||||||||||
Sbjct  309   ATTCTACCCCCCTCTACAAGACTCAAGCCTGCCAGTTTCAAATGCAGTTCCCAGGTTAAG  250

Query  850   CCCGGGGGATTTCACATCTGACTTAACAGACCGCCTNCGTGCGCTTTACGCCCAGTAATT  909
             ||||||| |||||||||||||||||||||||||||| |||||||||||||||||||||||
Sbjct  249   CCCGGGG-ATTTCACATCTGACTTAACAGACCGCCTGCGTGCGCTTTACGCCCAGTAATT  191

Query  910   CCGATTAACGCTTGCACCCTCCGTATTACCGCGGCTGCTGGCACGGAGTTAGCCGGTGCT  969
             ||||||||||||||||||||||||||||||||||||||||||||||||||||||||||||
Sbjct  190   CCGATTAACGCTTGCACCCTCCGTATTACCGCGGCTGCTGGCACGGAGTTAGCCGGTGCT  131

Query  970   TCTTCTGCGGGTAACGTCAATCGA  993
             ||||||||||||||||||||||||
Sbjct  130   TCTTCTGCGGGTAACGTCAATCGA  107
```

Download

FASTA (complete sequence)

FASTA (aligned sequences)

GenBank (complete sequence)

Continue
Cancel

GenBankGraphics

Next
Previous
Descriptions

Pantoea agglomerans strain P2FNb\_1 16S ribosomal RNA gene, partial sequence

Sequence ID: KX891517.1Length: 1028Number of Matches: 1

Related Information

Range 1: 19 to 1001GenBankGraphics

Next Match
Previous Match
First Match

Alignment statistics for match #1

| Score | Expect | Identities | Gaps | Strand | Frame |
| --- | --- | --- | --- | --- | --- |
| 1784 bits(966) | 0.0() | 976/984(99%) | 1/984(0%) | Plus/Minus |  |

Features:

```
Query  10    CTCCCGAAGGTTANGCTACCTACTTCTTTTGCANCCCACTCCCATGGTGTGACGGGCGGT  69
             ||||||||||||| ||||||||||||||||||| ||||||||||||||||||||||||||
Sbjct  1001  CTCCCGAAGGTTAAGCTACCTACTTCTTTTGCAACCCACTCCCATGGTGTGACGGGCGGT  942

Query  70    GTGTACAAGGCCCGGGAACGTATTCACCGTGGCATTCTGATCCACGATTACTAGCGATTC  129
             ||||||||||||||||||||||||||||||||||||||||||||||||||||||||||||
Sbjct  941   GTGTACAAGGCCCGGGAACGTATTCACCGTGGCATTCTGATCCACGATTACTAGCGATTC  882

Query  130   CGACTTCACGGAGTCGAGTTGCAGACTCCGATCCGGACTACGACGCACTTTGTGAGGTCC  189
             ||||||||||||||||||||||||||||||||||||||||||||||||||||||||||||
Sbjct  881   CGACTTCACGGAGTCGAGTTGCAGACTCCGATCCGGACTACGACGCACTTTGTGAGGTCC  822

Query  190   GCTTGCTCTCGCGAGGTCGCTTCTCTTTGTATGCGCCATTGTAGCACGTGTGTAGCCCTA  249
             ||||||||||||||||||||||||||||||||||||||||||||||||||||||||||||
Sbjct  821   GCTTGCTCTCGCGAGGTCGCTTCTCTTTGTATGCGCCATTGTAGCACGTGTGTAGCCCTA  762

Query  250   CTCGTAAGGGCCATGATGACTTGACGTCATCCCCACCTTCCTCCGGTTTATCACCGGCAG  309
             ||||||||||||||||||||||||||||||||||||||||||||||||||||||||||||
Sbjct  761   CTCGTAAGGGCCATGATGACTTGACGTCATCCCCACCTTCCTCCGGTTTATCACCGGCAG  702

Query  310   TCTCCTTTGAGTTCCCGACCGAATCGCTGGCAACAAAGGATAAGGGTTGCGCTCGTTGCG  369
             ||||||||||||||||||||||||||||||||||||||||||||||||||||||||||||
Sbjct  701   TCTCCTTTGAGTTCCCGACCGAATCGCTGGCAACAAAGGATAAGGGTTGCGCTCGTTGCG  642

Query  370   GGACTTAACCCAACATTTCACAACACGAGCTGACGACAGCCATGCAGCACCTGTCTCANN  429
             ||||||||||||||||||||||||||||||||||||||||||||||||||||||||||  
Sbjct  641   GGACTTAACCCAACATTTCACAACACGAGCTGACGACAGCCATGCAGCACCTGTCTCACG  582

Query  430   GTTCCCGAAGGCACTAAGGCATCTCTGNNAAATTCCGTGGATGTCAAGAGTAGGTAAGGT  489
             |||||||||||||||||||||||||||  |||||||||||||||||||||||||||||||
Sbjct  581   GTTCCCGAAGGCACTAAGGCATCTCTGCCAAATTCCGTGGATGTCAAGAGTAGGTAAGGT  522

Query  490   TCTTCGCGTTGCATCGAATTAAACCACATGCTCCACCGCTTGTGCGGGCCCCCGTCAATT  549
             ||||||||||||||||||||||||||||||||||||||||||||||||||||||||||||
Sbjct  521   TCTTCGCGTTGCATCGAATTAAACCACATGCTCCACCGCTTGTGCGGGCCCCCGTCAATT  462

Query  550   CATTTGAGTTTTAACCTTGCGGCCGTACTCCCCAGGCGGTCGACTTAACGCGTTAGCTCC  609
             ||||||||||||||||||||||||||||||||||||||||||||||||||||||||||||
Sbjct  461   CATTTGAGTTTTAACCTTGCGGCCGTACTCCCCAGGCGGTCGACTTAACGCGTTAGCTCC  402

Query  610   GGAAGCCACTCCTCAAGGGAACAACCTCCAAGTCGACATCGTTTACGGCGTGGACTACCA  669
             ||||||||||||||||||||||||||||||||||||||||||||||||||||||||||||
Sbjct  401   GGAAGCCACTCCTCAAGGGAACAACCTCCAAGTCGACATCGTTTACGGCGTGGACTACCA  342

Query  670   GGGTATCTAATCCTGTTTGCTCCCCACGCTTTCGCACCTGAGCGTCAGTCTTTGTCCAGG  729
             ||||||||||||||||||||||||||||||||||||||||||||||||||||||||||||
Sbjct  341   GGGTATCTAATCCTGTTTGCTCCCCACGCTTTCGCACCTGAGCGTCAGTCTTTGTCCAGG  282

Query  730   GGGCCGCCTTCGCCACCGGTATTCCTCCAGATCTCTACGCATTTCACCGCTACACCTGGA  789
             ||||||||||||||||||||||||||||||||||||||||||||||||||||||||||||
Sbjct  281   GGGCCGCCTTCGCCACCGGTATTCCTCCAGATCTCTACGCATTTCACCGCTACACCTGGA  222

Query  790   ATTCTACCCCCCTCTACAAGACTCAAGCCTGCCAGTTTCAAATGCAGTTCCCAGGTTAAG  849
             ||||||||||||||||||||||||||||||||||||||||||||||||||||||||||||
Sbjct  221   ATTCTACCCCCCTCTACAAGACTCAAGCCTGCCAGTTTCAAATGCAGTTCCCAGGTTAAG  162

Query  850   CCCGGGGGATTTCACATCTGACTTAACAGACCGCCTNCGTGCGCTTTACGCCCAGTAATT  909
             ||||||| |||||||||||||||||||||||||||| |||||||||||||||||||||||
Sbjct  161   CCCGGGG-ATTTCACATCTGACTTAACAGACCGCCTGCGTGCGCTTTACGCCCAGTAATT  103

Query  910   CCGATTAACGCTTGCACCCTCCGTATTACCGCGGCTGCTGGCACGGAGTTAGCCGGTGCT  969
             ||||||||||||||||||||||||||||||||||||||||||||||||||||||||||||
Sbjct  102   CCGATTAACGCTTGCACCCTCCGTATTACCGCGGCTGCTGGCACGGAGTTAGCCGGTGCT  43

Query  970   TCTTCTGCGGGTAACGTCAATCGA  993
             ||||||||||||||||||||||||
Sbjct  42    TCTTCTGCGGGTAACGTCAATCGA  19
```


BLAST is a registered trademark of the National Library of Medicine

Support center
Mailing list


YouTube

- National Library Of Medicine
- National Institutes Of Health
- U.S. Department of Health & Human Services
- USA.gov

### NCBI


National Center for Biotechnology Information,
 U.S. National Library of Medicine

8600 Rockville Pike,
Bethesda
 MD,
20894
USA

Policies and Guidelines
|
Contact


PreferencesTurn off

External link. Please review our privacy policy.
