# Supplementary material for: Isothermal microcalorimetry for thermal viable count of microorganisms in pure cultures and stabilized formulations
Source: BMC Microbiol. 2019 Mar 21;19:65. doi: 10.1186/s12866-019-1432-8 (PMC6429831; doi:10.1186/s12866-019-1432-8)
Supplement: Supplementary file 3 — 16S rRNA gene sequences’ BLASTN hits in zipped HTML format. (ZIP 15810 kb) [file 12866_2019_1432_MOESM3_ESM.zip › Best blastn hits/NCBI Blast_8 pale upper band R -- 8..1068 of sequence.html]

NCBI Blast:8 pale upper band R -- 8..1068 of sequence


- NCBI Home
- Sign in to NCBI
- Skip to Main Content
- Skip to Navigation
- About NCBI Accesskeys

U.S. National Library of Medicine

NCBI
National Center for Biotechnology Information

- My NCBI
- Sign in to NCBI
- Register
- Sign Out

BLAST ^®^ » blastn suite » RID-A4V05YV8015


- Home
- Recent Results
- Saved Strategies
- Help

BLAST Results


Edit and Resubmit
Save Search Strategies
[Sign in above to save your search strategy]

Formatting options 


Download


How to read this page
Blast report description
Questions/comments


|  |  |
| --- | --- |
| Formatting options | |
| Show | Alignment as  HTML Plain text   Old View Reset form to defaults [?]  These options control formatting of alignments in results pages. The default is HTML, but other formats (including plain text) are available. PSSM and PssmWithParameters are representations of Position Specific Scoring Matrices and are only available for PSI-BLAST. The Advanced view option allows the database descriptions to be sorted by various indices in a table. |
| Alignment View | Pairwise Pairwise with dots for identities Query-anchored with dots for identities Query-anchored with letters for identities Flat query-anchored with dots for identities Flat query-anchored with letters for identities [?]  Choose how to view alignments. The default "pairwise" view shows how each subject sequence aligns individually to the query sequence. The "query-anchored" view shows how all subject sequences align to the query sequence. For each view type, you can choose to show "identities" (matching residues) as letters or dots. more... |
| Display | Graphical Overview   Linkout   Sequence Retrieval  NCBI-gi   CDS feature [?]  - Graphical Overview: Graphical Overview: Show graph of similar sequence regions aligned to query.   more... - NCBI-gi: Show NCBI gi identifiers. - CDS feature: Show annotated coding region and translation.   more... |
| Masking | Character:   X for protein, n for nucleotide Lower Case  Color:  Black Grey Red [?]  - Masking Character: Display masked (filtered) sequence regions as lower-case or as specific letters (N for nucleotide, P for protein). - Masking Color: Display masked sequence regions in the given color. |
| Limit results | Descriptions:  10 50 100 Graphical overview:  0 10 50 100  Alignments:  0 10 50 100 Line length:  60 90 120 150 [?]  - Descriptions: Show short descriptions for up to the given number of sequences. - Alignments: Show alignments for up to the given number of sequences, in order of statistical significance. - Line lenghth: Number of letters to show on one line in an alignment. |
|  | Organism Type common name, binomial, taxid, or group name. Only 20 top taxa will be shown.     Exclude   [?]  Show only sequences from the given organism. |
|  | Entrez query:  [?]  Show only those sequences that match the given Entrez query. more... |
|  | Expect Min:  Expect Max:  [?]  Show only sequences with expect values in the given range. more... |
|  | Percent Identity Min:  Percent Identity Max:  [?]  Show only sequences with percent identity values in the given range. |
| Format for | PSI-BLAST with inclusion threshold:  [?]  - Format for PSI-BLAST: The Position-Specific Iterated BLAST (PSI-BLAST) program performs iterative searches with a protein query,   in which sequences found in one round of search are used to build a custom score model for the next round.   more... - Inclusion Threshold: This sets the statistical significance threshold for including a sequence in the model used   by PSI-BLAST to create the PSSM on the next iteration. |

|  |  |  |  |  |  |
| --- | --- | --- | --- | --- | --- |
| Download | | | | | |
| Alignment  Text XML ASN.1 JSON Seq-align Hit Table(text) Hit Table(csv) Multiple-file XML2 Single-file XML2 Multiple-file JSON Single-file JSON SAM | Search Strategies  ASN.1 | PSSM to restart search  PSSM | [?] |  |  |

The Download link provides BLAST output that may be used as input to another program.
This includes parseable formats such as the tabular report or XML as well as the Search Strategy files read by the BLAST+ applications.
More details on the parseable (XML, tabular, and ASN.1) reports can be found at
https://www.ncbi.nlm.nih.gov/books/NBK153387/  
  

The following formats are offered under the Alignment section:  
1). "Text". Non-HTML standard BLAST report.  
2). "XML". XML report based upon the DTD at https://www.ncbi.nlm.nih.gov/data\_specs/dtd/NCBI\_BlastOutput.dtd  
3). "ASN.1". Alignment written out in Abstract Syntax Notation 1.  
4). "JSON Seq-align". Alignment written out in JSON.  
4). "Hit Table(text)". The tabular report as text.  
5). "Hit Table(csv)". The tabular report ready for import into spread-sheet programs like Excel.  
6). "XML2". New XML format described at ftp://ftp.ncbi.nlm.nih.gov/blast/documents/NEWXML/xml2.pdf.  
7). "JSON". New JSON format described at ftp://ftp.ncbi.nlm.nih.gov/blast/documents/NEWXML/xml2.pdf.  
8). "SAM". Sequence Alignment Map format.

XML2 and JSON can be downloaded either as one file per query (multiple-file) or one file for all queries (single-file). These formats are listed as Multiple-file XML2 (and JSON) or Single-file XML (and JSON).

The following report is offered under the Search Strategy section:  
1). "ASN.1" Search Strategy. A record of the parameters, query, and database used in the search. This file can be used to start a stand-alone BLAST search, see
https://www.ncbi.nlm.nih.gov/books/NBK1763/#CmdLineAppsManual.I455\_BLAST\_search\_stra


# Job title: 8 pale upper band R -- 8..1068 of sequence

Results for:

lcl|Query\_196045 8 pale upper band R -- 8..1068 of sequence(1061bp)
[?]

Your BLAST job specified more than one input sequence.
This box lets you choose which input sequence to show BLAST results for.

RID
:   A4V05YV8015 (Expires on 03-10 18:12 pm)

Query ID
:   lcl|Query\_196045
:   lcl|Query\_196045

Description
:   8 pale upper band R -- 8..1068 of sequence

Molecule type
:   nucleic acid

Query Length
:   1061

Database Name
:   nr

Description
:   Nucleotide collection (nt) See details

Program
:   BLASTN 2.8.0+ Citation

  

Reference 

Zheng Zhang, Scott Schwartz, Lukas Wagner, and Webb Miller (2000), "A greedy algorithm for aligning DNA sequences", J Comput Biol 2000; 7(1-2):203-14.

Reference - database indexing

Aleksandr Morgulis, George Coulouris, Yan Raytselis, Thomas L. Madden, Richa Agarwala, Alejandro A. Schäffer (2008), "Database Indexing for Production MegaBLAST Searches", Bioinformatics 24:1757-1764.

Other reports:
Search Summary

[Taxonomy reports]
[Distance tree of results]
[MSA viewer]

Search Parameters

| Search parameter name | Search parameter value |
| --- | --- |
| Program | blastn |
| Word size | 28 |
| Expect value | 10 |
| Hitlist size | 100 |
| Match/Mismatch scores | 1,-2 |
| Gapcosts | 0,2.5 |
| Low Complexity Filter | Yes |
| Filter string | L;m; |
| Genetic Code | 1 |

Database

| Database parameter name | Database parameter value |
| --- | --- |
| Posted date | Mar 7, 2018 1:58 PM |
| Number of letters | 174,044,644,244 |
| Number of sequences | 46,882,714 |
| Entrez query | Includes:  Excludes:  None |

Karlin-Altschul statistics

| Params | Ungapped | Gapped |
| --- | --- | --- |
| Lambda | 1.33271 | 1.28 |
| K | 0.620991 | 0.46 |
| H | 1.12409 | 0.85 |

Results Statistics

| Results Statistics parameter name | Results Statistics parameter value |
| --- | --- |
| Length adjustment | 35 |
| Effective length of query | 1026 |
| Effective length of database | 172403749254 |
| Effective search space | 176886246734604 |
| Effective search space used | 176886246734604 |


## Graphic Summary

### Distribution of the top 134 Blast Hits on 100 subject sequences [?]

The graphic is an overview of the database sequences aligned to the query sequence. These are represented horizontal bars colored coded by score and showing the extent
of the alignment on the query sequence. Separate aligned regions on the same database sequence are connected by a thin grey line.
Mousing over an alignment shows the database sequence title. Clicking an alignment displays a box with more details about the alignment and
link to the sequence alignment itself in the Alignments section of the report.

Mouse over to see the title, click to show alignments

Color key for alignment scores

<40

40-50

50-80

80-200

>=200

Query

1

200

400

600

800

1000

Pseudomonas putida partial 16S rRNA gene, strain CFBP 4..

Score:1914 Evalue:0

Accession:HF545841.1

Alignment

Pseudomonas sp. S-3 16S ribosomal RNA gene, partial seq..

Score:1914 Evalue:0

Accession:KC207086.1

Alignment

Pseudomonas brassicacearum isolate MA250 16S ribosomal ..

Score:1914 Evalue:0

Accession:DQ886486.1

Alignment

Pseudomonas brassicacearum strain YC-1 16S ribosomal RN..

Score:1908 Evalue:0

Accession:KY753310.1

Alignment

Pseudomonas sp. strain 7.3 16S ribosomal RNA gene, part..

Score:1908 Evalue:0

Accession:KY542120.1

Alignment

Pseudomonas fluorescens strain SBR10 16S ribosomal RNA ..

Score:1908 Evalue:0

Accession:KX018310.1

Alignment

Pseudomonas brassicacearum strain Kr21 16S ribosomal RN..

Score:1908 Evalue:0

Accession:KT215482.1

Alignment

Pseudomonas brassicacearum strain IHB B 13650 16S ribos..

Score:1908 Evalue:0

Accession:KP762561.1

Alignment

Pseudomonas brassicacearum subsp. neoaurantiaca strain ..

Score:1908 Evalue:0

Accession:KP762555.1

Alignment

Pseudomonas brassicacearum strain 13B-23 16S ribosomal ..

Score:1908 Evalue:0

Accession:KR083019.1

Alignment

Pseudomonas brassicacearum strain 3B1 16S ribosomal RNA..

Score:1908 Evalue:0

Accession:KR611047.1

Alignment

Pseudomonas sp. 41(2015) 16S ribosomal RNA gene, partia..

Score:1908 Evalue:0

Accession:KR080568.1

Alignment

Pseudomonas sp. SAM1 16S ribosomal RNA gene, partial se..

Score:1908 Evalue:0

Accession:KM269192.1

Alignment

Uncultured Pseudomonas sp. clone CGMCG 6071 16S ribosom..

Score:1908 Evalue:0

Accession:JX077087.1

Alignment

Pseudomonas fluorescens gene for 16S rRNA, partial sequ..

Score:1908 Evalue:0

Accession:AB621593.1

Alignment

Pseudomonas thivervalensis 16S ribosomal RNA gene, part..

Score:1908 Evalue:0

Accession:JN628032.1

Alignment

Pseudomonas fluorescens 16S rRNA gene, strain C7R12

Score:1908 Evalue:0

Accession:AM229082.1

Alignment

Pseudomonas brassicacearum strain FC-7 16S ribosomal RN..

Score:1906 Evalue:0

Accession:KY649379.1

Alignment

Pseudomonas sp. cpRA293 16S ribosomal RNA gene, partial..

Score:1906 Evalue:0

Accession:KJ510220.1

Alignment

Pseudomonas brassicacearum strain WK-444s 16S ribosomal..

Score:1906 Evalue:0

Accession:KF580861.1

Alignment

Uncultured bacterium clone Untreatedsoil-0day-94 16S ri..

Score:1903 Evalue:0

Accession:MF314815.2

Alignment

Uncultured bacterium clone Untreatedsoil-0day-1 16S rib..

Score:1903 Evalue:0

Accession:MF314725.2

Alignment

Pseudomonas fluorescens strain 2P24 chromosome, complet..

Score:1903 Evalue:0

Accession:CP025542.1

Alignment

Pseudomonas brassicacearum strain LBUM300 16S ribosomal..

Score:1903 Evalue:0

Accession:MG461459.1

Alignment

Pseudomonas sp. strain KF-8 16S ribosomal RNA gene, par..

Score:1903 Evalue:0

Accession:MG266327.1

Alignment

Uncultured bacterium clone SPN400-90day-85 16S ribosoma..

Score:1903 Evalue:0

Accession:MF314628.1

Alignment

Uncultured bacterium clone SPN400-90day-76 16S ribosoma..

Score:1903 Evalue:0

Accession:MF314621.1

Alignment

Uncultured bacterium clone SPN400-90day-50 16S ribosoma..

Score:1903 Evalue:0

Accession:MF314595.1

Alignment

Uncultured bacterium clone SPN400-90day-46 16S ribosoma..

Score:1903 Evalue:0

Accession:MF314592.1

Alignment

Uncultured bacterium clone SPN400-90day-43 16S ribosoma..

Score:1903 Evalue:0

Accession:MF314589.1

Alignment

Uncultured bacterium clone SPN400-90day-20 16S ribosoma..

Score:1903 Evalue:0

Accession:MF314569.1

Alignment

Uncultured bacterium clone SPN400-90day-6 16S ribosomal..

Score:1903 Evalue:0

Accession:MF314558.1

Alignment

Pseudomonas brassicacearum strain Pv10 16S ribosomal RN..

Score:1903 Evalue:0

Accession:MF624719.1

Alignment

Pseudomonas jessenii strain AP3\_16 16S ribosomal RNA ge..

Score:1903 Evalue:0

Accession:MF498772.1

Alignment

Pseudomonas sp. strain A9 16S ribosomal RNA gene, parti..

Score:1903 Evalue:0

Accession:KX859150.1

Alignment

Pseudomonas fluorescens strain PFR1 16S ribosomal RNA g..

Score:1903 Evalue:0

Accession:MF000304.1

Alignment

Pseudomonas brassicacearum strain SKUAST-K21 16S riboso..

Score:1903 Evalue:0

Accession:KY612271.1

Alignment

Pseudomonas syringae strain yangyueP4 16S ribosomal RNA..

Score:1903 Evalue:0

Accession:KU977139.1

Alignment

Pseudomonas brassicacearum strain B04 16S ribosomal RNA..

Score:1903 Evalue:0

Accession:KU878092.1

Alignment

Pseudomonas synxantha strain LMG 2190 genome assembly, ..

Score:1903 Evalue:0

Accession:LT629786.1

Alignment

Pseudomonas brassicacearum strain BS3663 genome assembl..

Score:1903 Evalue:0

Accession:LT629713.1

Alignment

Pseudomonas brassicacearum strain TSDS1 16S ribosomal R..

Score:1903 Evalue:0

Accession:KX984045.1

Alignment

Pseudomonas brassicacearum strain Delaware 16S ribosoma..

Score:1903 Evalue:0

Accession:KT695846.1

Alignment

Pseudomonas brassicacearum strain Wood1 16S ribosomal R..

Score:1903 Evalue:0

Accession:KT695843.1

Alignment

Pseudomonas brassicacearum strain 93F8 16S ribosomal RN..

Score:1903 Evalue:0

Accession:KT695841.1

Alignment

Pseudomonas brassicacearum strain L13-6-12, complete ge..

Score:1903 Evalue:0

Accession:CP014693.1

Alignment

Pseudomonas brassicacearum strain KK 5 16S ribosomal RN..

Score:1903 Evalue:0

Accession:KP858915.1

Alignment

Pseudomonas fluorescens strain FW300-N2E2 genome

Score:1903 Evalue:0

Accession:CP015225.1

Alignment

Pseudomonas jessenii partial 16S rRNA gene, strain R-52..

Score:1903 Evalue:0

Accession:LN995697.1

Alignment

Pseudomonas brassicacearum strain LBUM300, complete gen..

Score:1903 Evalue:0

Accession:CP012680.1

Alignment

Pseudomonas corrugata strain G1 16S ribosomal RNA gene,..

Score:1903 Evalue:0

Accession:KT957303.1

Alignment

Pseudomonas fluorescens strain FW300-N2C3, complete gen..

Score:1903 Evalue:0

Accession:CP012831.1

Alignment

Pseudomonas migulae strain DD290 16S ribosomal RNA gene..

Score:1903 Evalue:0

Accession:KR822273.1

Alignment

Pseudomonas thivervalensis strain MAH1 16S ribosomal RN..

Score:1903 Evalue:0

Accession:KP742980.1

Alignment

Pseudomonas brassicacearum strain 21B8 16S ribosomal RN..

Score:1903 Evalue:0

Accession:KR611050.1

Alignment

Pseudomonas sp. 15(2015) 16S ribosomal RNA gene, partia..

Score:1903 Evalue:0

Accession:KR080563.1

Alignment

## Descriptions

, Reading indexes 1-5, displaying indexes 1-5


Load next setPrevious Match

Sequences producing significant alignments:

Show all columns  of the table presenting sequences producing significant alignments 

Select:AllNone
Selected:0

Alignments
Download

FASTA (complete sequence)

FASTA (aligned sequences)

GenBank (complete sequence)

Hit Table (text)

Hit Table (CSV)

Text

XML

ASN.1

Continue
Cancel

GenBank 
Graphics
Distance tree of results
Multiple alignment
Show/hide columns of the table presenting sequences producing significant alignments 

Available columns

Description  
Max Score  
Total Score  
Coverage  
E-value  
IdentN  
Accession  
Restore Defaults
Ok
Cancel

Sequences producing significant alignments:

| Select for downloading or viewing reports | Description | Max score | Total score | Query cover | E value | Ident | Accession |
| --- | --- | --- | --- | --- | --- | --- | --- |
| 1Select seq HF545841.1 | Pseudomonas putida partial 16S rRNA gene, strain CFBP 4629 | 1914 | 1914 | 99% | 0.0 | 99% | HF545841.1 |
| 2Select seq KC207086.1 | Pseudomonas sp. S-3 16S ribosomal RNA gene, partial sequence | 1914 | 1914 | 99% | 0.0 | 99% | KC207086.1 |
| 3Select seq DQ886486.1 | Pseudomonas brassicacearum isolate MA250 16S ribosomal RNA gene, partial sequence | 1914 | 1914 | 99% | 0.0 | 99% | DQ886486.1 |
| 4Select seq KY753310.1 | Pseudomonas brassicacearum strain YC-1 16S ribosomal RNA gene, partial sequence | 1908 | 1908 | 99% | 0.0 | 99% | KY753310.1 |
| 5Select seq KY542120.1 | Pseudomonas sp. strain 7.3 16S ribosomal RNA gene, partial sequence | 1908 | 1908 | 99% | 0.0 | 99% | KY542120.1 |
| 6Select seq KX018310.1 | Pseudomonas fluorescens strain SBR10 16S ribosomal RNA gene, partial sequence | 1908 | 1908 | 99% | 0.0 | 99% | KX018310.1 |
| 7Select seq KT215482.1 | Pseudomonas brassicacearum strain Kr21 16S ribosomal RNA gene, partial sequence | 1908 | 1908 | 99% | 0.0 | 99% | KT215482.1 |
| 8Select seq KP762561.1 | Pseudomonas brassicacearum strain IHB B 13650 16S ribosomal RNA gene, partial sequence | 1908 | 1908 | 99% | 0.0 | 99% | KP762561.1 |
| 9Select seq KP762555.1 | Pseudomonas brassicacearum subsp. neoaurantiaca strain IHB B 13645 16S ribosomal RNA gene, partial sequence | 1908 | 1908 | 99% | 0.0 | 99% | KP762555.1 |
| 10Select seq KR083019.1 | Pseudomonas brassicacearum strain 13B-23 16S ribosomal RNA gene, partial sequence | 1908 | 1908 | 99% | 0.0 | 99% | KR083019.1 |
| 11Select seq KR611047.1 | Pseudomonas brassicacearum strain 3B1 16S ribosomal RNA gene, partial sequence | 1908 | 1908 | 99% | 0.0 | 99% | KR611047.1 |
| 12Select seq KR080568.1 | Pseudomonas sp. 41(2015) 16S ribosomal RNA gene, partial sequence | 1908 | 1908 | 99% | 0.0 | 99% | KR080568.1 |
| 13Select seq KM269192.1 | Pseudomonas sp. SAM1 16S ribosomal RNA gene, partial sequence | 1908 | 1908 | 99% | 0.0 | 99% | KM269192.1 |
| 14Select seq JX077087.1 | Uncultured Pseudomonas sp. clone CGMCG 6071 16S ribosomal RNA gene, partial sequence | 1908 | 1908 | 99% | 0.0 | 99% | JX077087.1 |
| 15Select seq AB621593.1 | Pseudomonas fluorescens gene for 16S rRNA, partial sequence, strain: MPF29 | 1908 | 1908 | 99% | 0.0 | 99% | AB621593.1 |
| 16Select seq JN628032.1 | Pseudomonas thivervalensis 16S ribosomal RNA gene, partial sequence | 1908 | 1908 | 99% | 0.0 | 99% | JN628032.1 |
| 17Select seq AM229082.1 | Pseudomonas fluorescens 16S rRNA gene, strain C7R12 | 1908 | 1908 | 99% | 0.0 | 99% | AM229082.1 |
| 18Select seq KY649379.1 | Pseudomonas brassicacearum strain FC-7 16S ribosomal RNA gene, partial sequence | 1906 | 1906 | 99% | 0.0 | 99% | KY649379.1 |
| 19Select seq KJ510220.1 | Pseudomonas sp. cpRA293 16S ribosomal RNA gene, partial sequence | 1906 | 1906 | 99% | 0.0 | 99% | KJ510220.1 |
| 20Select seq KF580861.1 | Pseudomonas brassicacearum strain WK-444s 16S ribosomal RNA gene, partial sequence | 1906 | 1906 | 99% | 0.0 | 99% | KF580861.1 |
| 21Select seq MF314815.2 | Uncultured bacterium clone Untreatedsoil-0day-94 16S ribosomal RNA gene, partial sequence | 1903 | 1903 | 99% | 0.0 | 99% | MF314815.2 |
| 22Select seq MF314725.2 | Uncultured bacterium clone Untreatedsoil-0day-1 16S ribosomal RNA gene, partial sequence | 1903 | 1903 | 99% | 0.0 | 99% | MF314725.2 |
| 23Select seq CP025542.1 | Pseudomonas fluorescens strain 2P24 chromosome, complete genome | 1903 | 9504 | 99% | 0.0 | 99% | CP025542.1 |
| 24Select seq MG461459.1 | Pseudomonas brassicacearum strain LBUM300 16S ribosomal RNA gene, partial sequence | 1903 | 1903 | 99% | 0.0 | 99% | MG461459.1 |
| 25Select seq MG266327.1 | Pseudomonas sp. strain KF-8 16S ribosomal RNA gene, partial sequence | 1903 | 1903 | 99% | 0.0 | 99% | MG266327.1 |
| 26Select seq MF314628.1 | Uncultured bacterium clone SPN400-90day-85 16S ribosomal RNA gene, partial sequence | 1903 | 1903 | 99% | 0.0 | 99% | MF314628.1 |
| 27Select seq MF314621.1 | Uncultured bacterium clone SPN400-90day-76 16S ribosomal RNA gene, partial sequence | 1903 | 1903 | 99% | 0.0 | 99% | MF314621.1 |
| 28Select seq MF314595.1 | Uncultured bacterium clone SPN400-90day-50 16S ribosomal RNA gene, partial sequence | 1903 | 1903 | 99% | 0.0 | 99% | MF314595.1 |
| 29Select seq MF314592.1 | Uncultured bacterium clone SPN400-90day-46 16S ribosomal RNA gene, partial sequence | 1903 | 1903 | 99% | 0.0 | 99% | MF314592.1 |
| 30Select seq MF314589.1 | Uncultured bacterium clone SPN400-90day-43 16S ribosomal RNA gene, partial sequence | 1903 | 1903 | 99% | 0.0 | 99% | MF314589.1 |
| 31Select seq MF314569.1 | Uncultured bacterium clone SPN400-90day-20 16S ribosomal RNA gene, partial sequence | 1903 | 1903 | 99% | 0.0 | 99% | MF314569.1 |
| 32Select seq MF314558.1 | Uncultured bacterium clone SPN400-90day-6 16S ribosomal RNA gene, partial sequence | 1903 | 1903 | 99% | 0.0 | 99% | MF314558.1 |
| 33Select seq MF624719.1 | Pseudomonas brassicacearum strain Pv10 16S ribosomal RNA gene, partial sequence | 1903 | 1903 | 99% | 0.0 | 99% | MF624719.1 |
| 34Select seq MF498772.1 | Pseudomonas jessenii strain AP3\_16 16S ribosomal RNA gene, partial sequence | 1903 | 1903 | 99% | 0.0 | 99% | MF498772.1 |
| 35Select seq KX859150.1 | Pseudomonas sp. strain A9 16S ribosomal RNA gene, partial sequence | 1903 | 1903 | 99% | 0.0 | 99% | KX859150.1 |
| 36Select seq MF000304.1 | Pseudomonas fluorescens strain PFR1 16S ribosomal RNA gene, partial sequence | 1903 | 1903 | 99% | 0.0 | 99% | MF000304.1 |
| 37Select seq KY612271.1 | Pseudomonas brassicacearum strain SKUAST-K21 16S ribosomal RNA gene, partial sequence | 1903 | 1903 | 99% | 0.0 | 99% | KY612271.1 |
| 38Select seq KU977139.1 | Pseudomonas syringae strain yangyueP4 16S ribosomal RNA gene, partial sequence | 1903 | 1903 | 99% | 0.0 | 99% | KU977139.1 |
| 39Select seq KU878092.1 | Pseudomonas brassicacearum strain B04 16S ribosomal RNA gene, partial sequence | 1903 | 1903 | 99% | 0.0 | 99% | KU878092.1 |
| 40Select seq LT629786.1 | Pseudomonas synxantha strain LMG 2190 genome assembly, chromosome: I | 1903 | 12668 | 99% | 0.0 | 99% | LT629786.1 |
| 41Select seq LT629713.1 | Pseudomonas brassicacearum strain BS3663 genome assembly, chromosome: I | 1903 | 9515 | 99% | 0.0 | 99% | LT629713.1 |
| 42Select seq KX984045.1 | Pseudomonas brassicacearum strain TSDS1 16S ribosomal RNA gene, partial sequence | 1903 | 1903 | 99% | 0.0 | 99% | KX984045.1 |
| 43Select seq KT695846.1 | Pseudomonas brassicacearum strain Delaware 16S ribosomal RNA gene, partial sequence | 1903 | 1903 | 99% | 0.0 | 99% | KT695846.1 |
| 44Select seq KT695843.1 | Pseudomonas brassicacearum strain Wood1 16S ribosomal RNA gene, partial sequence | 1903 | 1903 | 99% | 0.0 | 99% | KT695843.1 |
| 45Select seq KT695841.1 | Pseudomonas brassicacearum strain 93F8 16S ribosomal RNA gene, partial sequence | 1903 | 1903 | 99% | 0.0 | 99% | KT695841.1 |
| 46Select seq CP014693.1 | Pseudomonas brassicacearum strain L13-6-12, complete genome | 1903 | 9515 | 99% | 0.0 | 99% | CP014693.1 |
| 47Select seq KP858915.1 | Pseudomonas brassicacearum strain KK 5 16S ribosomal RNA gene, partial sequence | 1903 | 1903 | 99% | 0.0 | 99% | KP858915.1 |
| 48Select seq CP015225.1 | Pseudomonas fluorescens strain FW300-N2E2 genome | 1903 | 9510 | 99% | 0.0 | 99% | CP015225.1 |
| 49Select seq LN995697.1 | Pseudomonas jessenii partial 16S rRNA gene, strain R-52636 | 1903 | 1903 | 99% | 0.0 | 99% | LN995697.1 |
| 50Select seq CP012680.1 | Pseudomonas brassicacearum strain LBUM300, complete genome | 1903 | 9515 | 99% | 0.0 | 99% | CP012680.1 |
| 51Select seq KT957303.1 | Pseudomonas corrugata strain G1 16S ribosomal RNA gene, partial sequence | 1903 | 1903 | 99% | 0.0 | 99% | KT957303.1 |
| 52Select seq CP012831.1 | Pseudomonas fluorescens strain FW300-N2C3, complete genome | 1903 | 9515 | 99% | 0.0 | 99% | CP012831.1 |
| 53Select seq KR822273.1 | Pseudomonas migulae strain DD290 16S ribosomal RNA gene, partial sequence | 1903 | 1903 | 99% | 0.0 | 99% | KR822273.1 |
| 54Select seq KP742980.1 | Pseudomonas thivervalensis strain MAH1 16S ribosomal RNA gene, partial sequence | 1903 | 1903 | 99% | 0.0 | 99% | KP742980.1 |
| 55Select seq KR611050.1 | Pseudomonas brassicacearum strain 21B8 16S ribosomal RNA gene, partial sequence | 1903 | 1903 | 99% | 0.0 | 99% | KR611050.1 |
| 56Select seq KR080563.1 | Pseudomonas sp. 15(2015) 16S ribosomal RNA gene, partial sequence | 1903 | 1903 | 99% | 0.0 | 99% | KR080563.1 |
| 57Select seq KR080562.1 | Pseudomonas sp. 12(2015) 16S ribosomal RNA gene, partial sequence | 1903 | 1903 | 99% | 0.0 | 99% | KR080562.1 |
| 58Select seq LC015570.1 | Pseudomonas brassicacearum subsp. brassicacearum gene for 16S ribosomal RNA, partial sequence, strain: AF129 | 1903 | 1903 | 99% | 0.0 | 99% | LC015570.1 |
| 59Select seq LC015569.1 | Pseudomonas brassicacearum subsp. brassicacearum gene for 16S ribosomal RNA, partial sequence, strain: AF5 | 1903 | 1903 | 99% | 0.0 | 99% | LC015569.1 |
| 60Select seq LC015567.1 | Pseudomonas brassicacearum subsp. neoaurantiaca gene for 16S ribosomal RNA, partial sequence, strain: AF82 | 1903 | 1903 | 99% | 0.0 | 99% | LC015567.1 |
| 61Select seq KM030057.1 | Pseudomonas sp. TY1210 16S ribosomal RNA gene, partial sequence | 1903 | 1903 | 99% | 0.0 | 99% | KM030057.1 |
| 62Select seq KJ420530.1 | Pseudomonas thivervalensis strain PE32 16S ribosomal RNA gene, partial sequence | 1903 | 1903 | 99% | 0.0 | 99% | KJ420530.1 |
| 63Select seq KF148637.1 | Pseudomonas fluorescens strain JK15 16S ribosomal RNA gene, partial sequence | 1903 | 1903 | 99% | 0.0 | 99% | KF148637.1 |
| 64Select seq KJ027533.1 | Pseudomonas fluorescens strain dqe01 16S ribosomal RNA gene, partial sequence | 1903 | 1903 | 99% | 0.0 | 99% | KJ027533.1 |
| 65Select seq KF876865.1 | Pseudomonas thivervalensis strain YJ-RT11 16S ribosomal RNA gene, partial sequence | 1903 | 1903 | 99% | 0.0 | 99% | KF876865.1 |
| 66Select seq KF840730.1 | Pseudomonas kilonensis strain JX22 16S ribosomal RNA gene, partial sequence | 1903 | 1903 | 99% | 0.0 | 99% | KF840730.1 |
| 67Select seq KF835389.1 | Pseudomonas sp. SDW-16 16S ribosomal RNA gene, partial sequence | 1903 | 1903 | 99% | 0.0 | 99% | KF835389.1 |
| 68Select seq KF444431.1 | Pseudomonas fluorescens strain PB4 16S ribosomal RNA gene, partial sequence | 1903 | 1903 | 99% | 0.0 | 99% | KF444431.1 |
| 69Select seq KF475874.1 | Pseudomonas brassicacearum strain IHB B 821 16S ribosomal RNA gene, partial sequence | 1903 | 1903 | 99% | 0.0 | 99% | KF475874.1 |
| 70Select seq KF360067.1 | Pseudomonas sp. BSP5 16S ribosomal RNA gene, partial sequence | 1903 | 1903 | 99% | 0.0 | 99% | KF360067.1 |
| 71Select seq KF318402.1 | Pseudomonas brassicacearum strain R-15 16S ribosomal RNA gene, partial sequence | 1903 | 1903 | 99% | 0.0 | 99% | KF318402.1 |
| 72Select seq KF312468.1 | Pseudomonas sp. SJ7b 16S ribosomal RNA gene, partial sequence | 1903 | 1903 | 99% | 0.0 | 99% | KF312468.1 |
| 73Select seq KC246049.1 | Pseudomonas fluorescens strain IBFC2012-45 16S ribosomal RNA gene, partial sequence | 1903 | 1903 | 99% | 0.0 | 99% | KC246049.1 |
| 74Select seq HQ888871.1 | Pseudomonas fluorescens strain P-72-10 16S ribosomal RNA gene, partial sequence | 1903 | 1903 | 99% | 0.0 | 99% | HQ888871.1 |
| 75Select seq JN216880.1 | Pseudomonas sp. N-128 16S ribosomal RNA gene, partial sequence | 1903 | 1903 | 99% | 0.0 | 99% | JN216880.1 |
| 76Select seq JN399995.1 | Pseudomonas fluorescens strain J2 16S ribosomal RNA gene, partial sequence | 1903 | 1903 | 99% | 0.0 | 99% | JN399995.1 |
| 77Select seq JN605747.1 | Pseudomonas brassicacearum strain J12 16S ribosomal RNA gene, partial sequence | 1903 | 1903 | 99% | 0.0 | 99% | JN605747.1 |
| 78Select seq JQ779065.1 | Bacterium NTL501 16S ribosomal RNA gene, partial sequence | 1903 | 1903 | 99% | 0.0 | 99% | JQ779065.1 |
| 79Select seq JQ779056.1 | Bacterium NTL264 16S ribosomal RNA gene, partial sequence | 1903 | 1903 | 99% | 0.0 | 99% | JQ779056.1 |
| 80Select seq JQ779041.1 | Bacterium NTL206 16S ribosomal RNA gene, partial sequence | 1903 | 1903 | 99% | 0.0 | 99% | JQ779041.1 |
| 81Select seq JN628030.1 | Pseudomonas brassicacearum subsp. neoaurantiaca 16S ribosomal RNA gene, partial sequence | 1903 | 1903 | 99% | 0.0 | 99% | JN628030.1 |
| 82Select seq JN033549.1 | Pseudomonas brassicacearum strain ME BHU2 16S ribosomal RNA gene, partial sequence | 1903 | 1903 | 99% | 0.0 | 99% | JN033549.1 |
| 83Select seq FR675976.1 | Pseudomonas sp. LB184 partial 16S rRNA gene, isolate LB184 | 1903 | 1903 | 99% | 0.0 | 99% | FR675976.1 |
| 84Select seq JF500978.1 | Uncultured Pseudomonas sp. clone 101 16S ribosomal RNA gene, partial sequence | 1903 | 1903 | 99% | 0.0 | 99% | JF500978.1 |
| 85Select seq JF500937.1 | Uncultured Pseudomonas sp. clone 57 16S ribosomal RNA gene, partial sequence | 1903 | 1903 | 99% | 0.0 | 99% | JF500937.1 |
| 86Select seq JF500932.1 | Uncultured Pseudomonas sp. clone 52 16S ribosomal RNA gene, partial sequence | 1903 | 1903 | 99% | 0.0 | 99% | JF500932.1 |
| 87Select seq JF500908.1 | Uncultured Pseudomonas sp. clone 24 16S ribosomal RNA gene, partial sequence | 1903 | 1903 | 99% | 0.0 | 99% | JF500908.1 |
| 88Select seq CP002585.1 | Pseudomonas brassicacearum subsp. brassicacearum NFM421, complete genome | 1903 | 9510 | 99% | 0.0 | 99% | CP002585.1 |
| 89Select seq HM854217.1 | Pseudomonas fluorescens strain STAD384 16S ribosomal RNA gene, partial sequence | 1903 | 1903 | 99% | 0.0 | 99% | HM854217.1 |
| 90Select seq HM057101.1 | Pseudomonas sp. 3-10 16S ribosomal RNA gene, partial sequence | 1903 | 1903 | 99% | 0.0 | 99% | HM057101.1 |
| 91Select seq GU201849.1 | Pseudomonas brassicacearum strain Zy-2-1 16S ribosomal RNA gene, partial sequence | 1903 | 1903 | 99% | 0.0 | 99% | GU201849.1 |
| 92Select seq MF062638.1 | Pseudomonas putida strain 42R-P6 16S ribosomal RNA gene, partial sequence | 1901 | 1901 | 99% | 0.0 | 99% | MF062638.1 |
| 93Select seq LT844660.1 | Pseudomonas sp. HA-09 partial 16S rRNA gene, isolate HA-09 | 1901 | 1901 | 98% | 0.0 | 99% | LT844660.1 |
| 94Select seq KU882739.1 | Pseudomonas sp. MAR9910 16S ribosomal RNA gene, partial sequence | 1901 | 1901 | 99% | 0.0 | 99% | KU882739.1 |
| 95Select seq KR855698.1 | Pseudomonas brassicacearum strain SM27 16S ribosomal RNA gene, partial sequence | 1901 | 1901 | 99% | 0.0 | 99% | KR855698.1 |
| 96Select seq KR611048.1 | Pseudomonas brassicacearum strain 11B23 16S ribosomal RNA gene, partial sequence | 1901 | 1901 | 99% | 0.0 | 99% | KR611048.1 |
| 97Select seq KP730603.1 | Pseudomonas brassicacearum subsp. brassicacearum strain BW0808 16S ribosomal RNA gene, partial sequence | 1901 | 1901 | 98% | 0.0 | 99% | KP730603.1 |
| 98Select seq KC428747.1 | Pseudomonas sp. X123 16S ribosomal RNA gene, partial sequence | 1901 | 1901 | 99% | 0.0 | 99% | KC428747.1 |
| 99Select seq JF901350.1 | Endophytic bacterium 41P-2 16S ribosomal RNA gene, partial sequence | 1901 | 1901 | 99% | 0.0 | 99% | JF901350.1 |
| 100Select seq HM579795.1 | Pseudomonas sp. HY4(2010) 16S ribosomal RNA gene, partial sequence | 1901 | 1901 | 99% | 0.0 | 99% | HM579795.1 |


## Alignments

Loading alignment... for sequences gi|515781775,gi|442571814,gi|115315703,gi|1206483241,gi|1137473154 Reading indexes 1-5

Download

FASTA (complete sequence)

FASTA (aligned sequences)

GenBank (complete sequence)

Continue
Cancel

GenBankGraphics

Next
Previous
Descriptions

Pseudomonas putida partial 16S rRNA gene, strain CFBP 4629

Sequence ID: HF545841.1Length: 1418Number of Matches: 1

Related Information

Range 1: 360 to 1416GenBankGraphics

Next Match
Previous Match
First Match

Alignment statistics for match #1

| Score | Expect | Identities | Gaps | Strand | Frame |
| --- | --- | --- | --- | --- | --- |
| 1914 bits(1036) | 0.0() | 1047/1057(99%) | 0/1057(0%) | Plus/Minus |  |

Features:

```
Query  5     CGTGGNACCGTCCTNNCGAAGGTTAGACTAGCTACTTCTGGTNCAACCCACTCCCATGGT  64
             ||||| ||||||||  |||||||||||||||||||||||||| |||||||||||||||||
Sbjct  1416  CGTGGTACCGTCCTCCCGAAGGTTAGACTAGCTACTTCTGGTGCAACCCACTCCCATGGT  1357

Query  65    GTGACGGGCGGTGTGTACAAGGCCCGGGAACGTATTCACCGCGACATTCTGATTCGCGAT  124
             ||||||||||||||||||||||||||||||||||||||||||||||||||||||||||||
Sbjct  1356  GTGACGGGCGGTGTGTACAAGGCCCGGGAACGTATTCACCGCGACATTCTGATTCGCGAT  1297

Query  125   TACTAGCGATTCCGACTTCACGCAGTCGAGTTGCAGACTGCGATCCGGACTACGATCGGT  184
             ||||||||||||||||||||||||||||||||||||||||||||||||||||||||||||
Sbjct  1296  TACTAGCGATTCCGACTTCACGCAGTCGAGTTGCAGACTGCGATCCGGACTACGATCGGT  1237

Query  185   TTTGTGGGATTAGCTCCACCTCGCGGCTTGGCAACCCTCTGTACCGACCATTGTAGCACG  244
             ||||||||||||||||||||||||||||||||||||||||||||||||||||||||||||
Sbjct  1236  TTTGTGGGATTAGCTCCACCTCGCGGCTTGGCAACCCTCTGTACCGACCATTGTAGCACG  1177

Query  245   TGTGTAGCCCAGGCCGTAAGGGCCATGATGACTTGACGTCATCCCCACCTTCCTCCGGTT  304
             ||||||||||||||||||||||||||||||||||||||||||||||||||||||||||||
Sbjct  1176  TGTGTAGCCCAGGCCGTAAGGGCCATGATGACTTGACGTCATCCCCACCTTCCTCCGGTT  1117

Query  305   TGTCACCGGCAGTCTCCTTAGAGTGCCCACCATGACGTGCTGGTAACTAAGGACAAGGGT  364
             ||||||||||||||||||||||||||||||||| ||||||||||||||||||||||||||
Sbjct  1116  TGTCACCGGCAGTCTCCTTAGAGTGCCCACCATAACGTGCTGGTAACTAAGGACAAGGGT  1057

Query  365   TGCGCTCGTTACGGGACTTAACCCAACATCTCACGACACGAGCTGACGACAGCCATGCAG  424
             ||||||||||||||||||||||||||||||||||||||||||||||||||||||||||||
Sbjct  1056  TGCGCTCGTTACGGGACTTAACCCAACATCTCACGACACGAGCTGACGACAGCCATGCAG  997

Query  425   CACCTGTCTCAATNNTCCCGAAGGCACCAATCCATCTCTGGAAAGTTCATTGGATGTCAA  484
             |||||||||||||  |||||||||||||||||||||||||||||||||||||||||||||
Sbjct  996   CACCTGTCTCAATGTTCCCGAAGGCACCAATCCATCTCTGGAAAGTTCATTGGATGTCAA  937

Query  485   GGCCTGGTAAGGTTCTTCGCGTTGCTTCGAATTAAACCACATGCTCCACCGCTTGTGCGG  544
             ||||||||||||||||||||||||||||||||||||||||||||||||||||||||||||
Sbjct  936   GGCCTGGTAAGGTTCTTCGCGTTGCTTCGAATTAAACCACATGCTCCACCGCTTGTGCGG  877

Query  545   GCCCCCGTCAATTCATTTGAGTTTTAACCTTGCGGCCGTACTCCCCAGGCGGTCAACTTA  604
             ||||||||||||||||||||||||||||||||||||||||||||||||||||||||||||
Sbjct  876   GCCCCCGTCAATTCATTTGAGTTTTAACCTTGCGGCCGTACTCCCCAGGCGGTCAACTTA  817

Query  605   ATGCGTTAGCTGCGCCACTAAGAGCTCAAGGCTCCCAACGGCTAGTTGACATCGTTTACG  664
             ||||||||||||||||||||||||||||||||||||||||||||||||||||||||||||
Sbjct  816   ATGCGTTAGCTGCGCCACTAAGAGCTCAAGGCTCCCAACGGCTAGTTGACATCGTTTACG  757

Query  665   GCGTGGACTACCAGGGTATCTAATCCTGTTTGCTCCCCACGCTTTCGCACCTCAGTGTCA  724
             ||||||||||||||||||||||||||||||||||||||||||||||||||||||||||||
Sbjct  756   GCGTGGACTACCAGGGTATCTAATCCTGTTTGCTCCCCACGCTTTCGCACCTCAGTGTCA  697

Query  725   GTATCAGTCCAGGTGGTCGCCTTCGCCACTGGTGTTCCTTCCTATATCTACGCATTTCAC  784
             ||||||||||||||||||||||||||||||||||||||||||||||||||||||||||||
Sbjct  696   GTATCAGTCCAGGTGGTCGCCTTCGCCACTGGTGTTCCTTCCTATATCTACGCATTTCAC  637

Query  785   CGCTACACAGGAAATTCCACCACCCTCTACCATACTCTAGCTCGACAGTTTTGAATGCAG  844
             ||||||||||||||||||||||||||||||||||||||||||||||||||||||||||||
Sbjct  636   CGCTACACAGGAAATTCCACCACCCTCTACCATACTCTAGCTCGACAGTTTTGAATGCAG  577

Query  845   TTCCCAGGTTGAGCCCGGGGCTTTCACATCCAACTTAACGAACCACCTACGCGCGCTTTA  904
             ||||||||||||||||||||||||||||||||||||||||||||||||||||||||||||
Sbjct  576   TTCCCAGGTTGAGCCCGGGGCTTTCACATCCAACTTAACGAACCACCTACGCGCGCTTTA  517

Query  905   CGCCCAGTAATTCCGATTAACGCTTGCACCCTCTGTATTACCGCGGCTGCTGGCACNNAG  964
             ||||||||||||||||||||||||||||||||||||||||||||||||||||||||  ||
Sbjct  516   CGCCCAGTAATTCCGATTAACGCTTGCACCCTCTGTATTACCGCGGCTGCTGGCACAGAG  457

Query  965   TTAGCCGGTGCTTATTCTGTCGGTAACGTCAAAACACTAACGTATTAGGTTAATGCCCTT  1024
             ||||||||||||||||||||||||||||||||||||||||||||||||||||||||||||
Sbjct  456   TTAGCCGGTGCTTATTCTGTCGGTAACGTCAAAACACTAACGTATTAGGTTAATGCCCTT  397

Query  1025  CCTCCCAACTTAAAGTGCTTTACAATCCGAAGNCCTT  1061
             |||||||||||||||||||||||||||||||| ||||
Sbjct  396   CCTCCCAACTTAAAGTGCTTTACAATCCGAAGACCTT  360
```

Download

FASTA (complete sequence)

FASTA (aligned sequences)

GenBank (complete sequence)

Continue
Cancel

GenBankGraphics

Next
Previous
Descriptions

Pseudomonas sp. S-3 16S ribosomal RNA gene, partial sequence

Sequence ID: KC207086.1Length: 1444Number of Matches: 1

Related Information

Range 1: 376 to 1432GenBankGraphics

Next Match
Previous Match
First Match

Alignment statistics for match #1

| Score | Expect | Identities | Gaps | Strand | Frame |
| --- | --- | --- | --- | --- | --- |
| 1914 bits(1036) | 0.0() | 1047/1057(99%) | 0/1057(0%) | Plus/Minus |  |

Features:

```
Query  5     CGTGGNACCGTCCTNNCGAAGGTTAGACTAGCTACTTCTGGTNCAACCCACTCCCATGGT  64
             ||||| |||||||   |||||||||||||||||||||||||| |||||||||||||||||
Sbjct  1432  CGTGGTACCGTCCCCCCGAAGGTTAGACTAGCTACTTCTGGTGCAACCCACTCCCATGGT  1373

Query  65    GTGACGGGCGGTGTGTACAAGGCCCGGGAACGTATTCACCGCGACATTCTGATTCGCGAT  124
             ||||||||||||||||||||||||||||||||||||||||||||||||||||||||||||
Sbjct  1372  GTGACGGGCGGTGTGTACAAGGCCCGGGAACGTATTCACCGCGACATTCTGATTCGCGAT  1313

Query  125   TACTAGCGATTCCGACTTCACGCAGTCGAGTTGCAGACTGCGATCCGGACTACGATCGGT  184
             ||||||||||||||||||||||||||||||||||||||||||||||||||||||||||||
Sbjct  1312  TACTAGCGATTCCGACTTCACGCAGTCGAGTTGCAGACTGCGATCCGGACTACGATCGGT  1253

Query  185   TTTGTGGGATTAGCTCCACCTCGCGGCTTGGCAACCCTCTGTACCGACCATTGTAGCACG  244
             ||||||||||||||||||||||||||||||||||||||||||||||||||||||||||||
Sbjct  1252  TTTGTGGGATTAGCTCCACCTCGCGGCTTGGCAACCCTCTGTACCGACCATTGTAGCACG  1193

Query  245   TGTGTAGCCCAGGCCGTAAGGGCCATGATGACTTGACGTCATCCCCACCTTCCTCCGGTT  304
             ||||||||||||||||||||||||||||||||||||||||||||||||||||||||||||
Sbjct  1192  TGTGTAGCCCAGGCCGTAAGGGCCATGATGACTTGACGTCATCCCCACCTTCCTCCGGTT  1133

Query  305   TGTCACCGGCAGTCTCCTTAGAGTGCCCACCATGACGTGCTGGTAACTAAGGACAAGGGT  364
             ||||||||||||||||||||||||||||||||||||||||||||||||||||||||||||
Sbjct  1132  TGTCACCGGCAGTCTCCTTAGAGTGCCCACCATGACGTGCTGGTAACTAAGGACAAGGGT  1073

Query  365   TGCGCTCGTTACGGGACTTAACCCAACATCTCACGACACGAGCTGACGACAGCCATGCAG  424
             ||||||||||||||||||||||||||||||||||||||||||||||||||||||||||||
Sbjct  1072  TGCGCTCGTTACGGGACTTAACCCAACATCTCACGACACGAGCTGACGACAGCCATGCAG  1013

Query  425   CACCTGTCTCAATNNTCCCGAAGGCACCAATCCATCTCTGGAAAGTTCATTGGATGTCAA  484
             |||||||||||||  |||||||||||||||||||||||||||||||||||||||||||||
Sbjct  1012  CACCTGTCTCAATGCTCCCGAAGGCACCAATCCATCTCTGGAAAGTTCATTGGATGTCAA  953

Query  485   GGCCTGGTAAGGTTCTTCGCGTTGCTTCGAATTAAACCACATGCTCCACCGCTTGTGCGG  544
             ||||||||||||||||||||||||||||||||||||||||||||||||||||||||||||
Sbjct  952   GGCCTGGTAAGGTTCTTCGCGTTGCTTCGAATTAAACCACATGCTCCACCGCTTGTGCGG  893

Query  545   GCCCCCGTCAATTCATTTGAGTTTTAACCTTGCGGCCGTACTCCCCAGGCGGTCAACTTA  604
             ||||||||||||||||||||||||||||||||||||||||||||||||||||||||||||
Sbjct  892   GCCCCCGTCAATTCATTTGAGTTTTAACCTTGCGGCCGTACTCCCCAGGCGGTCAACTTA  833

Query  605   ATGCGTTAGCTGCGCCACTAAGAGCTCAAGGCTCCCAACGGCTAGTTGACATCGTTTACG  664
             ||||||||||||||||||||||||||||||||||||||||||||||||||||||||||||
Sbjct  832   ATGCGTTAGCTGCGCCACTAAGAGCTCAAGGCTCCCAACGGCTAGTTGACATCGTTTACG  773

Query  665   GCGTGGACTACCAGGGTATCTAATCCTGTTTGCTCCCCACGCTTTCGCACCTCAGTGTCA  724
             ||||||||||||||||||||||||||||||||||||||||||||||||||||||||||||
Sbjct  772   GCGTGGACTACCAGGGTATCTAATCCTGTTTGCTCCCCACGCTTTCGCACCTCAGTGTCA  713

Query  725   GTATCAGTCCAGGTGGTCGCCTTCGCCACTGGTGTTCCTTCCTATATCTACGCATTTCAC  784
             ||||||||||||||||||||||||||||||||||||||||||||||||||||||||||||
Sbjct  712   GTATCAGTCCAGGTGGTCGCCTTCGCCACTGGTGTTCCTTCCTATATCTACGCATTTCAC  653

Query  785   CGCTACACAGGAAATTCCACCACCCTCTACCATACTCTAGCTCGACAGTTTTGAATGCAG  844
             ||||||||||||||||||||||||||||||||||||||||||||||||||||||||||||
Sbjct  652   CGCTACACAGGAAATTCCACCACCCTCTACCATACTCTAGCTCGACAGTTTTGAATGCAG  593

Query  845   TTCCCAGGTTGAGCCCGGGGCTTTCACATCCAACTTAACGAACCACCTACGCGCGCTTTA  904
             ||||||||||||||||||||||||||||||||||||||||||||||||||||||||||||
Sbjct  592   TTCCCAGGTTGAGCCCGGGGCTTTCACATCCAACTTAACGAACCACCTACGCGCGCTTTA  533

Query  905   CGCCCAGTAATTCCGATTAACGCTTGCACCCTCTGTATTACCGCGGCTGCTGGCACNNAG  964
             ||||||||||||||||||||||||||||||||||||||||||||||||||||||||  ||
Sbjct  532   CGCCCAGTAATTCCGATTAACGCTTGCACCCTCTGTATTACCGCGGCTGCTGGCACAGAG  473

Query  965   TTAGCCGGTGCTTATTCTGTCGGTAACGTCAAAACACTAACGTATTAGGTTAATGCCCTT  1024
             ||||||||||||||||||||||||||||||||||||||||||||||||||||||||||||
Sbjct  472   TTAGCCGGTGCTTATTCTGTCGGTAACGTCAAAACACTAACGTATTAGGTTAATGCCCTT  413

Query  1025  CCTCCCAACTTAAAGTGCTTTACAATCCGAAGNCCTT  1061
             |||||||||||||||||||||||||||||||| ||||
Sbjct  412   CCTCCCAACTTAAAGTGCTTTACAATCCGAAGACCTT  376
```

Download

FASTA (complete sequence)

FASTA (aligned sequences)

GenBank (complete sequence)

Continue
Cancel

GenBankGraphics

Next
Previous
Descriptions

Pseudomonas brassicacearum isolate MA250 16S ribosomal RNA gene, partial sequence

Sequence ID: DQ886486.1Length: 1515Number of Matches: 1

Related Information

Range 1: 395 to 1452GenBankGraphics

Next Match
Previous Match
First Match

Alignment statistics for match #1

| Score | Expect | Identities | Gaps | Strand | Frame |
| --- | --- | --- | --- | --- | --- |
| 1914 bits(1036) | 0.0() | 1048/1058(99%) | 1/1058(0%) | Plus/Minus |  |

Features:

```
Query  5     CGTGG-NACCGTCCTNNCGAAGGTTAGACTAGCTACTTCTGGTNCAACCCACTCCCATGG  63
             |||||  ||||||||  |||||||||||||||||||||||||| ||||||||||||||||
Sbjct  1452  CGTGGTAACCGTCCTCCCGAAGGTTAGACTAGCTACTTCTGGTGCAACCCACTCCCATGG  1393

Query  64    TGTGACGGGCGGTGTGTACAAGGCCCGGGAACGTATTCACCGCGACATTCTGATTCGCGA  123
             ||||||||||||||||||||||||||||||||||||||||||||||||||||||||||||
Sbjct  1392  TGTGACGGGCGGTGTGTACAAGGCCCGGGAACGTATTCACCGCGACATTCTGATTCGCGA  1333

Query  124   TTACTAGCGATTCCGACTTCACGCAGTCGAGTTGCAGACTGCGATCCGGACTACGATCGG  183
             ||||||||||||||||||||||||||||||||||||||||||||||||||||||||||||
Sbjct  1332  TTACTAGCGATTCCGACTTCACGCAGTCGAGTTGCAGACTGCGATCCGGACTACGATCGG  1273

Query  184   TTTTGTGGGATTAGCTCCACCTCGCGGCTTGGCAACCCTCTGTACCGACCATTGTAGCAC  243
             ||||||||||||||||||||||||||||||||||||||||||||||||||||||||||||
Sbjct  1272  TTTTGTGGGATTAGCTCCACCTCGCGGCTTGGCAACCCTCTGTACCGACCATTGTAGCAC  1213

Query  244   GTGTGTAGCCCAGGCCGTAAGGGCCATGATGACTTGACGTCATCCCCACCTTCCTCCGGT  303
             ||||||||||||||||||||||||||||||||||||||||||||||||||||||||||||
Sbjct  1212  GTGTGTAGCCCAGGCCGTAAGGGCCATGATGACTTGACGTCATCCCCACCTTCCTCCGGT  1153

Query  304   TTGTCACCGGCAGTCTCCTTAGAGTGCCCACCATGACGTGCTGGTAACTAAGGACAAGGG  363
             ||||||||||||||||||||||||||||||||||||||||||||||||||||||||||||
Sbjct  1152  TTGTCACCGGCAGTCTCCTTAGAGTGCCCACCATGACGTGCTGGTAACTAAGGACAAGGG  1093

Query  364   TTGCGCTCGTTACGGGACTTAACCCAACATCTCACGACACGAGCTGACGACAGCCATGCA  423
             ||||||||||||||||||||||||||||||||||||||||||||||||||||||||||||
Sbjct  1092  TTGCGCTCGTTACGGGACTTAACCCAACATCTCACGACACGAGCTGACGACAGCCATGCA  1033

Query  424   GCACCTGTCTCAATNNTCCCGAAGGCACCAATCCATCTCTGGAAAGTTCATTGGATGTCA  483
             ||||||||||||||  ||||||||||||||||||||||||||||||||||||||||||||
Sbjct  1032  GCACCTGTCTCAATGTTCCCGAAGGCACCAATCCATCTCTGGAAAGTTCATTGGATGTCA  973

Query  484   AGGCCTGGTAAGGTTCTTCGCGTTGCTTCGAATTAAACCACATGCTCCACCGCTTGTGCG  543
             ||||||||||||||||||||||||||||||||||||||||||||||||||||||||||||
Sbjct  972   AGGCCTGGTAAGGTTCTTCGCGTTGCTTCGAATTAAACCACATGCTCCACCGCTTGTGCG  913

Query  544   GGCCCCCGTCAATTCATTTGAGTTTTAACCTTGCGGCCGTACTCCCCAGGCGGTCAACTT  603
             ||||||||||||||||||||||||||||||||||||||||||||||||||||||||||||
Sbjct  912   GGCCCCCGTCAATTCATTTGAGTTTTAACCTTGCGGCCGTACTCCCCAGGCGGTCAACTT  853

Query  604   AATGCGTTAGCTGCGCCACTAAGAGCTCAAGGCTCCCAACGGCTAGTTGACATCGTTTAC  663
             ||||||||||||||||||||||||||||||||||||||||||||||||||||||||||||
Sbjct  852   AATGCGTTAGCTGCGCCACTAAGAGCTCAAGGCTCCCAACGGCTAGTTGACATCGTTTAC  793

Query  664   GGCGTGGACTACCAGGGTATCTAATCCTGTTTGCTCCCCACGCTTTCGCACCTCAGTGTC  723
             ||||||||||||||||||||||||||||||||||||||||||||||||||||||||||||
Sbjct  792   GGCGTGGACTACCAGGGTATCTAATCCTGTTTGCTCCCCACGCTTTCGCACCTCAGTGTC  733

Query  724   AGTATCAGTCCAGGTGGTCGCCTTCGCCACTGGTGTTCCTTCCTATATCTACGCATTTCA  783
             ||||||||||||||||||||||||||||||||||||||||||||||||||||||||||||
Sbjct  732   AGTATCAGTCCAGGTGGTCGCCTTCGCCACTGGTGTTCCTTCCTATATCTACGCATTTCA  673

Query  784   CCGCTACACAGGAAATTCCACCACCCTCTACCATACTCTAGCTCGACAGTTTTGAATGCA  843
             ||||||||||||||||||||||||||||||||||||||||||||||||||||||||||||
Sbjct  672   CCGCTACACAGGAAATTCCACCACCCTCTACCATACTCTAGCTCGACAGTTTTGAATGCA  613

Query  844   GTTCCCAGGTTGAGCCCGGGGCTTTCACATCCAACTTAACGAACCACCTACGCGCGCTTT  903
             ||||||||||||||||||||||||||||||||||||||||||||||||||||||||||||
Sbjct  612   GTTCCCAGGTTGAGCCCGGGGCTTTCACATCCAACTTAACGAACCACCTACGCGCGCTTT  553

Query  904   ACGCCCAGTAATTCCGATTAACGCTTGCACCCTCTGTATTACCGCGGCTGCTGGCACNNA  963
             |||||||||||||||||||||||||||||||||||||||||||||||||||||||||  |
Sbjct  552   ACGCCCAGTAATTCCGATTAACGCTTGCACCCTCTGTATTACCGCGGCTGCTGGCACAGA  493

Query  964   GTTAGCCGGTGCTTATTCTGTCGGTAACGTCAAAACACTAACGTATTAGGTTAATGCCCT  1023
             ||||||||||||||||||||||||||||||||||||||||||||||||||||||||||||
Sbjct  492   GTTAGCCGGTGCTTATTCTGTCGGTAACGTCAAAACACTAACGTATTAGGTTAATGCCCT  433

Query  1024  TCCTCCCAACTTAAAGTGCTTTACAATCCGAAGNCCTT  1061
             ||||||||||||||||||||||||||||||||| ||||
Sbjct  432   TCCTCCCAACTTAAAGTGCTTTACAATCCGAAGACCTT  395
```

Download

FASTA (complete sequence)

FASTA (aligned sequences)

GenBank (complete sequence)

Continue
Cancel

GenBankGraphics

Next
Previous
Descriptions

Pseudomonas brassicacearum strain YC-1 16S ribosomal RNA gene, partial sequence

Sequence ID: KY753310.1Length: 1430Number of Matches: 1

Related Information

Range 1: 362 to 1418GenBankGraphics

Next Match
Previous Match
First Match

Alignment statistics for match #1

| Score | Expect | Identities | Gaps | Strand | Frame |
| --- | --- | --- | --- | --- | --- |
| 1908 bits(1033) | 0.0() | 1046/1057(99%) | 0/1057(0%) | Plus/Minus |  |

Features:

```
Query  5     CGTGGNACCGTCCTNNCGAAGGTTAGACTAGCTACTTCTGGTNCAACCCACTCCCATGGT  64
             ||||| |||||||   |||||||||||||||||||||||||| |||||||||||||||||
Sbjct  1418  CGTGGTACCGTCCCCCCGAAGGTTAGACTAGCTACTTCTGGTGCAACCCACTCCCATGGT  1359

Query  65    GTGACGGGCGGTGTGTACAAGGCCCGGGAACGTATTCACCGCGACATTCTGATTCGCGAT  124
             ||||||||||||||||||||||||||||||||||||||||||||||||||||||||||||
Sbjct  1358  GTGACGGGCGGTGTGTACAAGGCCCGGGAACGTATTCACCGCGACATTCTGATTCGCGAT  1299

Query  125   TACTAGCGATTCCGACTTCACGCAGTCGAGTTGCAGACTGCGATCCGGACTACGATCGGT  184
             ||||||||||||||||||||||||||||||||||||||||||||||||||||||||||||
Sbjct  1298  TACTAGCGATTCCGACTTCACGCAGTCGAGTTGCAGACTGCGATCCGGACTACGATCGGT  1239

Query  185   TTTGTGGGATTAGCTCCACCTCGCGGCTTGGCAACCCTCTGTACCGACCATTGTAGCACG  244
             ||||||||||||||||||||||||||||||||||||||||||||||||||||||||||||
Sbjct  1238  TTTGTGGGATTAGCTCCACCTCGCGGCTTGGCAACCCTCTGTACCGACCATTGTAGCACG  1179

Query  245   TGTGTAGCCCAGGCCGTAAGGGCCATGATGACTTGACGTCATCCCCACCTTCCTCCGGTT  304
             ||||||||||||||||||||||||||||||||||||||||||||||||||||||||||||
Sbjct  1178  TGTGTAGCCCAGGCCGTAAGGGCCATGATGACTTGACGTCATCCCCACCTTCCTCCGGTT  1119

Query  305   TGTCACCGGCAGTCTCCTTAGAGTGCCCACCATGACGTGCTGGTAACTAAGGACAAGGGT  364
             ||||||||||||||||||||||||||||||||| ||||||||||||||||||||||||||
Sbjct  1118  TGTCACCGGCAGTCTCCTTAGAGTGCCCACCATCACGTGCTGGTAACTAAGGACAAGGGT  1059

Query  365   TGCGCTCGTTACGGGACTTAACCCAACATCTCACGACACGAGCTGACGACAGCCATGCAG  424
             ||||||||||||||||||||||||||||||||||||||||||||||||||||||||||||
Sbjct  1058  TGCGCTCGTTACGGGACTTAACCCAACATCTCACGACACGAGCTGACGACAGCCATGCAG  999

Query  425   CACCTGTCTCAATNNTCCCGAAGGCACCAATCCATCTCTGGAAAGTTCATTGGATGTCAA  484
             |||||||||||||  |||||||||||||||||||||||||||||||||||||||||||||
Sbjct  998   CACCTGTCTCAATGTTCCCGAAGGCACCAATCCATCTCTGGAAAGTTCATTGGATGTCAA  939

Query  485   GGCCTGGTAAGGTTCTTCGCGTTGCTTCGAATTAAACCACATGCTCCACCGCTTGTGCGG  544
             ||||||||||||||||||||||||||||||||||||||||||||||||||||||||||||
Sbjct  938   GGCCTGGTAAGGTTCTTCGCGTTGCTTCGAATTAAACCACATGCTCCACCGCTTGTGCGG  879

Query  545   GCCCCCGTCAATTCATTTGAGTTTTAACCTTGCGGCCGTACTCCCCAGGCGGTCAACTTA  604
             ||||||||||||||||||||||||||||||||||||||||||||||||||||||||||||
Sbjct  878   GCCCCCGTCAATTCATTTGAGTTTTAACCTTGCGGCCGTACTCCCCAGGCGGTCAACTTA  819

Query  605   ATGCGTTAGCTGCGCCACTAAGAGCTCAAGGCTCCCAACGGCTAGTTGACATCGTTTACG  664
             ||||||||||||||||||||||||||||||||||||||||||||||||||||||||||||
Sbjct  818   ATGCGTTAGCTGCGCCACTAAGAGCTCAAGGCTCCCAACGGCTAGTTGACATCGTTTACG  759

Query  665   GCGTGGACTACCAGGGTATCTAATCCTGTTTGCTCCCCACGCTTTCGCACCTCAGTGTCA  724
             ||||||||||||||||||||||||||||||||||||||||||||||||||||||||||||
Sbjct  758   GCGTGGACTACCAGGGTATCTAATCCTGTTTGCTCCCCACGCTTTCGCACCTCAGTGTCA  699

Query  725   GTATCAGTCCAGGTGGTCGCCTTCGCCACTGGTGTTCCTTCCTATATCTACGCATTTCAC  784
             ||||||||||||||||||||||||||||||||||||||||||||||||||||||||||||
Sbjct  698   GTATCAGTCCAGGTGGTCGCCTTCGCCACTGGTGTTCCTTCCTATATCTACGCATTTCAC  639

Query  785   CGCTACACAGGAAATTCCACCACCCTCTACCATACTCTAGCTCGACAGTTTTGAATGCAG  844
             ||||||||||||||||||||||||||||||||||||||||||||||||||||||||||||
Sbjct  638   CGCTACACAGGAAATTCCACCACCCTCTACCATACTCTAGCTCGACAGTTTTGAATGCAG  579

Query  845   TTCCCAGGTTGAGCCCGGGGCTTTCACATCCAACTTAACGAACCACCTACGCGCGCTTTA  904
             ||||||||||||||||||||||||||||||||||||||||||||||||||||||||||||
Sbjct  578   TTCCCAGGTTGAGCCCGGGGCTTTCACATCCAACTTAACGAACCACCTACGCGCGCTTTA  519

Query  905   CGCCCAGTAATTCCGATTAACGCTTGCACCCTCTGTATTACCGCGGCTGCTGGCACNNAG  964
             ||||||||||||||||||||||||||||||||||||||||||||||||||||||||  ||
Sbjct  518   CGCCCAGTAATTCCGATTAACGCTTGCACCCTCTGTATTACCGCGGCTGCTGGCACAGAG  459

Query  965   TTAGCCGGTGCTTATTCTGTCGGTAACGTCAAAACACTAACGTATTAGGTTAATGCCCTT  1024
             ||||||||||||||||||||||||||||||||||||||||||||||||||||||||||||
Sbjct  458   TTAGCCGGTGCTTATTCTGTCGGTAACGTCAAAACACTAACGTATTAGGTTAATGCCCTT  399

Query  1025  CCTCCCAACTTAAAGTGCTTTACAATCCGAAGNCCTT  1061
             |||||||||||||||||||||||||||||||| ||||
Sbjct  398   CCTCCCAACTTAAAGTGCTTTACAATCCGAAGACCTT  362
```

Download

FASTA (complete sequence)

FASTA (aligned sequences)

GenBank (complete sequence)

Continue
Cancel

GenBankGraphics

Next
Previous
Descriptions

Pseudomonas sp. strain 7.3 16S ribosomal RNA gene, partial sequence

Sequence ID: KY542120.1Length: 1546Number of Matches: 1

Related Information

Range 1: 408 to 1465GenBankGraphics

Next Match
Previous Match
First Match

Alignment statistics for match #1

| Score | Expect | Identities | Gaps | Strand | Frame |
| --- | --- | --- | --- | --- | --- |
| 1908 bits(1033) | 0.0() | 1047/1058(99%) | 1/1058(0%) | Plus/Minus |  |

Features:

```
Query  5     CGTGG-NACCGTCCTNNCGAAGGTTAGACTAGCTACTTCTGGTNCAACCCACTCCCATGG  63
             |||||  ||||||||  |||||||||||||||||||||||||| ||||||||||||||||
Sbjct  1465  CGTGGTAACCGTCCTCCCGAAGGTTAGACTAGCTACTTCTGGTGCAACCCACTCCCATGG  1406

Query  64    TGTGACGGGCGGTGTGTACAAGGCCCGGGAACGTATTCACCGCGACATTCTGATTCGCGA  123
             ||||||||||||||||||||||||||||||||||||||||||||||||||||||||||||
Sbjct  1405  TGTGACGGGCGGTGTGTACAAGGCCCGGGAACGTATTCACCGCGACATTCTGATTCGCGA  1346

Query  124   TTACTAGCGATTCCGACTTCACGCAGTCGAGTTGCAGACTGCGATCCGGACTACGATCGG  183
             ||||||||||||||||||||||||||||||||||||||||||||||||||||||||||||
Sbjct  1345  TTACTAGCGATTCCGACTTCACGCAGTCGAGTTGCAGACTGCGATCCGGACTACGATCGG  1286

Query  184   TTTTGTGGGATTAGCTCCACCTCGCGGCTTGGCAACCCTCTGTACCGACCATTGTAGCAC  243
             ||||||||||||||||||||||||||||||||||||||||||||||||||||||||||||
Sbjct  1285  TTTTGTGGGATTAGCTCCACCTCGCGGCTTGGCAACCCTCTGTACCGACCATTGTAGCAC  1226

Query  244   GTGTGTAGCCCAGGCCGTAAGGGCCATGATGACTTGACGTCATCCCCACCTTCCTCCGGT  303
             ||||||||||||||||||||||||||||||||||||||||||||||||||||||||||||
Sbjct  1225  GTGTGTAGCCCAGGCCGTAAGGGCCATGATGACTTGACGTCATCCCCACCTTCCTCCGGT  1166

Query  304   TTGTCACCGGCAGTCTCCTTAGAGTGCCCACCATGACGTGCTGGTAACTAAGGACAAGGG  363
             |||||||||||||||||||||||||||||||||| |||||||||||||||||||||||||
Sbjct  1165  TTGTCACCGGCAGTCTCCTTAGAGTGCCCACCATAACGTGCTGGTAACTAAGGACAAGGG  1106

Query  364   TTGCGCTCGTTACGGGACTTAACCCAACATCTCACGACACGAGCTGACGACAGCCATGCA  423
             ||||||||||||||||||||||||||||||||||||||||||||||||||||||||||||
Sbjct  1105  TTGCGCTCGTTACGGGACTTAACCCAACATCTCACGACACGAGCTGACGACAGCCATGCA  1046

Query  424   GCACCTGTCTCAATNNTCCCGAAGGCACCAATCCATCTCTGGAAAGTTCATTGGATGTCA  483
             ||||||||||||||  ||||||||||||||||||||||||||||||||||||||||||||
Sbjct  1045  GCACCTGTCTCAATGTTCCCGAAGGCACCAATCCATCTCTGGAAAGTTCATTGGATGTCA  986

Query  484   AGGCCTGGTAAGGTTCTTCGCGTTGCTTCGAATTAAACCACATGCTCCACCGCTTGTGCG  543
             ||||||||||||||||||||||||||||||||||||||||||||||||||||||||||||
Sbjct  985   AGGCCTGGTAAGGTTCTTCGCGTTGCTTCGAATTAAACCACATGCTCCACCGCTTGTGCG  926

Query  544   GGCCCCCGTCAATTCATTTGAGTTTTAACCTTGCGGCCGTACTCCCCAGGCGGTCAACTT  603
             ||||||||||||||||||||||||||||||||||||||||||||||||||||||||||||
Sbjct  925   GGCCCCCGTCAATTCATTTGAGTTTTAACCTTGCGGCCGTACTCCCCAGGCGGTCAACTT  866

Query  604   AATGCGTTAGCTGCGCCACTAAGAGCTCAAGGCTCCCAACGGCTAGTTGACATCGTTTAC  663
             ||||||||||||||||||||||||||||||||||||||||||||||||||||||||||||
Sbjct  865   AATGCGTTAGCTGCGCCACTAAGAGCTCAAGGCTCCCAACGGCTAGTTGACATCGTTTAC  806

Query  664   GGCGTGGACTACCAGGGTATCTAATCCTGTTTGCTCCCCACGCTTTCGCACCTCAGTGTC  723
             ||||||||||||||||||||||||||||||||||||||||||||||||||||||||||||
Sbjct  805   GGCGTGGACTACCAGGGTATCTAATCCTGTTTGCTCCCCACGCTTTCGCACCTCAGTGTC  746

Query  724   AGTATCAGTCCAGGTGGTCGCCTTCGCCACTGGTGTTCCTTCCTATATCTACGCATTTCA  783
             ||||||||||||||||||||||||||||||||||||||||||||||||||||||||||||
Sbjct  745   AGTATCAGTCCAGGTGGTCGCCTTCGCCACTGGTGTTCCTTCCTATATCTACGCATTTCA  686

Query  784   CCGCTACACAGGAAATTCCACCACCCTCTACCATACTCTAGCTCGACAGTTTTGAATGCA  843
             ||||||||||||||||||||||||||||||||||||||||||||||||||||||||||||
Sbjct  685   CCGCTACACAGGAAATTCCACCACCCTCTACCATACTCTAGCTCGACAGTTTTGAATGCA  626

Query  844   GTTCCCAGGTTGAGCCCGGGGCTTTCACATCCAACTTAACGAACCACCTACGCGCGCTTT  903
             ||||||||||||||||||||||||||||||||||||||||||||||||||||||||||||
Sbjct  625   GTTCCCAGGTTGAGCCCGGGGCTTTCACATCCAACTTAACGAACCACCTACGCGCGCTTT  566

Query  904   ACGCCCAGTAATTCCGATTAACGCTTGCACCCTCTGTATTACCGCGGCTGCTGGCACNNA  963
             |||||||||||||||||||||||||||||||||||||||||||||||||||||||||  |
Sbjct  565   ACGCCCAGTAATTCCGATTAACGCTTGCACCCTCTGTATTACCGCGGCTGCTGGCACAGA  506

Query  964   GTTAGCCGGTGCTTATTCTGTCGGTAACGTCAAAACACTAACGTATTAGGTTAATGCCCT  1023
             ||||||||||||||||||||||||||||||||||||||||||||||||||||||||||||
Sbjct  505   GTTAGCCGGTGCTTATTCTGTCGGTAACGTCAAAACACTAACGTATTAGGTTAATGCCCT  446

Query  1024  TCCTCCCAACTTAAAGTGCTTTACAATCCGAAGNCCTT  1061
             ||||||||||||||||||||||||||||||||| ||||
Sbjct  445   TCCTCCCAACTTAAAGTGCTTTACAATCCGAAGACCTT  408
```


BLAST is a registered trademark of the National Library of Medicine

Support center
Mailing list


YouTube

- National Library Of Medicine
- National Institutes Of Health
- U.S. Department of Health & Human Services
- USA.gov

### NCBI


National Center for Biotechnology Information,
 U.S. National Library of Medicine

8600 Rockville Pike,
Bethesda
 MD,
20894
USA

Policies and Guidelines
|
Contact


PreferencesTurn off

External link. Please review our privacy policy.
