# Supplementary material for: Isothermal microcalorimetry for thermal viable count of microorganisms in pure cultures and stabilized formulations
Source: BMC Microbiol. 2019 Mar 21;19:65. doi: 10.1186/s12866-019-1432-8 (PMC6429831; doi:10.1186/s12866-019-1432-8)
Supplement: Supplementary file 3 — 16S rRNA gene sequences’ BLASTN hits in zipped HTML format. (ZIP 15810 kb) [file 12866_2019_1432_MOESM3_ESM.zip › Best blastn hits/NCBI Blast_98 white faint positive R -- 10..1070 of.html]

NCBI Blast:98 white faint positive R -- 10..1070 of


- NCBI Home
- Sign in to NCBI
- Skip to Main Content
- Skip to Navigation
- About NCBI Accesskeys

U.S. National Library of Medicine

NCBI
National Center for Biotechnology Information

- My NCBI
- Sign in to NCBI
- Register
- Sign Out

BLAST ^®^ » blastn suite » RID-A4X3N5U5014


- Home
- Recent Results
- Saved Strategies
- Help

BLAST Results


Edit and Resubmit
Save Search Strategies
[Sign in above to save your search strategy]

Formatting options 


Download


How to read this page
Blast report description
Questions/comments


|  |  |
| --- | --- |
| Formatting options | |
| Show | Alignment as  HTML Plain text   Old View Reset form to defaults [?]  These options control formatting of alignments in results pages. The default is HTML, but other formats (including plain text) are available. PSSM and PssmWithParameters are representations of Position Specific Scoring Matrices and are only available for PSI-BLAST. The Advanced view option allows the database descriptions to be sorted by various indices in a table. |
| Alignment View | Pairwise Pairwise with dots for identities Query-anchored with dots for identities Query-anchored with letters for identities Flat query-anchored with dots for identities Flat query-anchored with letters for identities [?]  Choose how to view alignments. The default "pairwise" view shows how each subject sequence aligns individually to the query sequence. The "query-anchored" view shows how all subject sequences align to the query sequence. For each view type, you can choose to show "identities" (matching residues) as letters or dots. more... |
| Display | Graphical Overview   Linkout   Sequence Retrieval  NCBI-gi   CDS feature [?]  - Graphical Overview: Graphical Overview: Show graph of similar sequence regions aligned to query.   more... - NCBI-gi: Show NCBI gi identifiers. - CDS feature: Show annotated coding region and translation.   more... |
| Masking | Character:   X for protein, n for nucleotide Lower Case  Color:  Black Grey Red [?]  - Masking Character: Display masked (filtered) sequence regions as lower-case or as specific letters (N for nucleotide, P for protein). - Masking Color: Display masked sequence regions in the given color. |
| Limit results | Descriptions:  10 50 100 Graphical overview:  0 10 50 100  Alignments:  0 10 50 100 Line length:  60 90 120 150 [?]  - Descriptions: Show short descriptions for up to the given number of sequences. - Alignments: Show alignments for up to the given number of sequences, in order of statistical significance. - Line lenghth: Number of letters to show on one line in an alignment. |
|  | Organism Type common name, binomial, taxid, or group name. Only 20 top taxa will be shown.     Exclude   [?]  Show only sequences from the given organism. |
|  | Entrez query:  [?]  Show only those sequences that match the given Entrez query. more... |
|  | Expect Min:  Expect Max:  [?]  Show only sequences with expect values in the given range. more... |
|  | Percent Identity Min:  Percent Identity Max:  [?]  Show only sequences with percent identity values in the given range. |
| Format for | PSI-BLAST with inclusion threshold:  [?]  - Format for PSI-BLAST: The Position-Specific Iterated BLAST (PSI-BLAST) program performs iterative searches with a protein query,   in which sequences found in one round of search are used to build a custom score model for the next round.   more... - Inclusion Threshold: This sets the statistical significance threshold for including a sequence in the model used   by PSI-BLAST to create the PSSM on the next iteration. |

|  |  |  |  |  |  |
| --- | --- | --- | --- | --- | --- |
| Download | | | | | |
| Alignment  Text XML ASN.1 JSON Seq-align Hit Table(text) Hit Table(csv) Multiple-file XML2 Single-file XML2 Multiple-file JSON Single-file JSON SAM | Search Strategies  ASN.1 | PSSM to restart search  PSSM | [?] |  |  |

The Download link provides BLAST output that may be used as input to another program.
This includes parseable formats such as the tabular report or XML as well as the Search Strategy files read by the BLAST+ applications.
More details on the parseable (XML, tabular, and ASN.1) reports can be found at
https://www.ncbi.nlm.nih.gov/books/NBK153387/  
  

The following formats are offered under the Alignment section:  
1). "Text". Non-HTML standard BLAST report.  
2). "XML". XML report based upon the DTD at https://www.ncbi.nlm.nih.gov/data\_specs/dtd/NCBI\_BlastOutput.dtd  
3). "ASN.1". Alignment written out in Abstract Syntax Notation 1.  
4). "JSON Seq-align". Alignment written out in JSON.  
4). "Hit Table(text)". The tabular report as text.  
5). "Hit Table(csv)". The tabular report ready for import into spread-sheet programs like Excel.  
6). "XML2". New XML format described at ftp://ftp.ncbi.nlm.nih.gov/blast/documents/NEWXML/xml2.pdf.  
7). "JSON". New JSON format described at ftp://ftp.ncbi.nlm.nih.gov/blast/documents/NEWXML/xml2.pdf.  
8). "SAM". Sequence Alignment Map format.

XML2 and JSON can be downloaded either as one file per query (multiple-file) or one file for all queries (single-file). These formats are listed as Multiple-file XML2 (and JSON) or Single-file XML (and JSON).

The following report is offered under the Search Strategy section:  
1). "ASN.1" Search Strategy. A record of the parameters, query, and database used in the search. This file can be used to start a stand-alone BLAST search, see
https://www.ncbi.nlm.nih.gov/books/NBK1763/#CmdLineAppsManual.I455\_BLAST\_search\_stra


# Job title: 98 white faint positive R -- 10..1070 of

Results for:

lcl|Query\_237287 98 white faint positive R -- 10..1070 of sequence(1061bp)
[?]

Your BLAST job specified more than one input sequence.
This box lets you choose which input sequence to show BLAST results for.

RID
:   A4X3N5U5014 (Expires on 03-10 18:48 pm)

Query ID
:   lcl|Query\_237287
:   lcl|Query\_237287

Description
:   98 white faint positive R -- 10..1070 of sequence

Molecule type
:   nucleic acid

Query Length
:   1061

Database Name
:   nr

Description
:   Nucleotide collection (nt) See details

Program
:   BLASTN 2.8.0+ Citation

  

Reference 

Zheng Zhang, Scott Schwartz, Lukas Wagner, and Webb Miller (2000), "A greedy algorithm for aligning DNA sequences", J Comput Biol 2000; 7(1-2):203-14.

Reference - database indexing

Aleksandr Morgulis, George Coulouris, Yan Raytselis, Thomas L. Madden, Richa Agarwala, Alejandro A. Schäffer (2008), "Database Indexing for Production MegaBLAST Searches", Bioinformatics 24:1757-1764.

Other reports:
Search Summary

[Taxonomy reports]
[Distance tree of results]
[MSA viewer]

Search Parameters

| Search parameter name | Search parameter value |
| --- | --- |
| Program | blastn |
| Word size | 28 |
| Expect value | 10 |
| Hitlist size | 100 |
| Match/Mismatch scores | 1,-2 |
| Gapcosts | 0,2.5 |
| Low Complexity Filter | Yes |
| Filter string | L;m; |
| Genetic Code | 1 |

Database

| Database parameter name | Database parameter value |
| --- | --- |
| Posted date | Mar 7, 2018 1:58 PM |
| Number of letters | 174,044,644,244 |
| Number of sequences | 46,882,714 |
| Entrez query | Includes:  Excludes:  None |

Karlin-Altschul statistics

| Params | Ungapped | Gapped |
| --- | --- | --- |
| Lambda | 1.33271 | 1.28 |
| K | 0.620991 | 0.46 |
| H | 1.12409 | 0.85 |

Results Statistics

| Results Statistics parameter name | Results Statistics parameter value |
| --- | --- |
| Length adjustment | 35 |
| Effective length of query | 1026 |
| Effective length of database | 172403749254 |
| Effective search space | 176886246734604 |
| Effective search space used | 176886246734604 |


## Graphic Summary

### Distribution of the top 132 Blast Hits on 100 subject sequences [?]

The graphic is an overview of the database sequences aligned to the query sequence. These are represented horizontal bars colored coded by score and showing the extent
of the alignment on the query sequence. Separate aligned regions on the same database sequence are connected by a thin grey line.
Mousing over an alignment shows the database sequence title. Clicking an alignment displays a box with more details about the alignment and
link to the sequence alignment itself in the Alignments section of the report.

Mouse over to see the title, click to show alignments

Color key for alignment scores

<40

40-50

50-80

80-200

>=200

Query

1

200

400

600

800

1000

Pseudomonas sp. 231 16S ribosomal RNA gene, partial seq..

Score:1941 Evalue:0

Accession:GU213409.1

Alignment

Pseudomonas sp. 2-36(2010) 16S ribosomal RNA gene, part..

Score:1938 Evalue:0

Accession:HQ403088.2

Alignment

Pseudomonas putida partial 16S rRNA gene, strain CFBP 5..

Score:1938 Evalue:0

Accession:HF545843.1

Alignment

Pseudomonas sp. ABAC21 16S ribosomal RNA gene, partial ..

Score:1938 Evalue:0

Accession:KF003385.1

Alignment

Pseudomonas poae RE\*1-1-14, complete genome

Score:1938 Evalue:0

Accession:CP004045.1

Alignment

Pseudomonas sp. SAP770.2 16S ribosomal RNA gene, partia..

Score:1938 Evalue:0

Accession:JX067683.1

Alignment

Pseudomonas sp. PX7\_S1 16S ribosomal RNA gene, partial ..

Score:1938 Evalue:0

Accession:JF274937.1

Alignment

Pseudomonas poae strain T23 16S ribosomal RNA gene, com..

Score:1938 Evalue:0

Accession:EU111704.2

Alignment

Pseudomonas sp. WH1 16S ribosomal RNA gene, partial seq..

Score:1938 Evalue:0

Accession:FJ262366.1

Alignment

Pseudomonas trivialis strain MAF3 16S ribosomal RNA gen..

Score:1936 Evalue:0

Accession:MF590113.1

Alignment

Pseudomonas sp. BR2-02 16S ribosomal RNA gene, partial ..

Score:1936 Evalue:0

Accession:EU853182.1

Alignment

Pseudomonas sp. HP10C 16S ribosomal RNA gene, partial s..

Score:1934 Evalue:0

Accession:KM187375.1

Alignment

Pseudomonas fluorescens strain SWFU22 16S ribosomal RNA..

Score:1934 Evalue:0

Accession:KJ756337.1

Alignment

Pseudomonas fluorescens strain SWFU21 16S ribosomal RNA..

Score:1934 Evalue:0

Accession:KJ756336.1

Alignment

Pseudomonas sp. YXE2-f 16S ribosomal RNA gene, partial ..

Score:1934 Evalue:0

Accession:JF701975.1

Alignment

Pseudomonas sp. BR2-22 16S ribosomal RNA gene, partial ..

Score:1934 Evalue:0

Accession:EU853183.1

Alignment

Pseudomonas sp. CBCEN8 16S ribosomal RNA gene, partial ..

Score:1934 Evalue:0

Accession:EF427849.1

Alignment

Pseudomonas sp. CBMB25 16S ribosomal RNA gene, partial ..

Score:1934 Evalue:0

Accession:EF126755.1

Alignment

Pseudomonas sp. CBMB18 16S ribosomal RNA gene, partial ..

Score:1934 Evalue:0

Accession:EF126753.1

Alignment

Pseudomonas sp. S09G 359 chromosome

Score:1932 Evalue:0

Accession:CP025263.1

Alignment

Pseudomonas sp. strain KBL2 16S ribosomal RNA gene, par..

Score:1932 Evalue:0

Accession:MG576155.1

Alignment

Pseudomonas poae strain AAS-144 16S ribosomal RNA gene,..

Score:1932 Evalue:0

Accession:KY810658.1

Alignment

Pseudomonas poae strain ICE349 16S ribosomal RNA gene, ..

Score:1932 Evalue:0

Accession:KX588598.1

Alignment

Pseudomonas poae strain ICE259 16S ribosomal RNA gene, ..

Score:1932 Evalue:0

Accession:KX588597.1

Alignment

Pseudomonas poae strain BS2776 genome assembly, chromos..

Score:1932 Evalue:0

Accession:LT629706.1

Alignment

Uncultured bacterium partial 16S rRNA gene, clone clone..

Score:1932 Evalue:0

Accession:LT595870.1

Alignment

Uncultured bacterium clone MTWL201306-19 16S ribosomal ..

Score:1932 Evalue:0

Accession:KX509247.1

Alignment

Uncultured Pseudomonas sp. gene for 16S ribosomal RNA, ..

Score:1932 Evalue:0

Accession:LC140869.1

Alignment

Pseudomonas sp. 23K33a 16S ribosomal RNA gene, partial ..

Score:1932 Evalue:0

Accession:KT825765.1

Alignment

Pseudomonas sp. 17K8 16S ribosomal RNA gene, partial se..

Score:1932 Evalue:0

Accession:KT825736.1

Alignment

Pseudomonas poae strain B42 16S ribosomal RNA gene, par..

Score:1932 Evalue:0

Accession:KT767705.1

Alignment

Pseudomonas poae strain IHBB 11151 16S ribosomal RNA ge..

Score:1932 Evalue:0

Accession:KR085862.1

Alignment

Uncultured bacterium clone OTU51 16S ribosomal RNA gene..

Score:1932 Evalue:0

Accession:KP975307.1

Alignment

Pseudomonas fluorescens PICF7, complete genome

Score:1932 Evalue:0

Accession:CP005975.1

Alignment

Pseudomonas fluorescens strain PCL1751, complete genome

Score:1932 Evalue:0

Accession:CP010896.1

Alignment

Pseudomonas simiae strain WCS417 genome

Score:1932 Evalue:0

Accession:CP007637.1

Alignment

Pseudomonas fluorescens strain PE8 16S ribosomal RNA ge..

Score:1932 Evalue:0

Accession:KJ420524.1

Alignment

Pseudomonas extremorientalis strain CNU082017 16S ribos..

Score:1932 Evalue:0

Accession:KF979139.1

Alignment

Pseudomonas mandelii 16S ribosomal RNA gene, partial se..

Score:1932 Evalue:0

Accession:KC618443.1

Alignment

Pseudomonas sp. isolate PS2.1 16S ribosomal RNA gene, p..

Score:1932 Evalue:0

Accession:KF019106.1

Alignment

Pseudomonas fluorescens strain 20130311XA1 16S ribosoma..

Score:1932 Evalue:0

Accession:KC773764.1

Alignment

Pseudomonas poae strain EB20 16S ribosomal RNA gene, pa..

Score:1932 Evalue:0

Accession:JX515573.1

Alignment

Pseudomonas poae strain zol-15 16S ribosomal RNA gene, ..

Score:1932 Evalue:0

Accession:JQ782898.1

Alignment

Pseudomonas sp. ZBC1 16S ribosomal RNA gene, partial se..

Score:1932 Evalue:0

Accession:JQ638271.1

Alignment

Pseudomonas sp. TK-3 gene for 16S rRNA, partial sequenc..

Score:1932 Evalue:0

Accession:AB705621.1

Alignment

uncultured bacterium partial 16S rRNA gene, clone K15.8..

Score:1932 Evalue:0

Accession:HE576021.1

Alignment

Pseudomonas poae strain ORC5 16S ribosomal RNA gene, pa..

Score:1932 Evalue:0

Accession:JQ236630.1

Alignment

Pseudomonas sp. SGb192 16S ribosomal RNA gene, partial ..

Score:1932 Evalue:0

Accession:HQ224626.1

Alignment

Pseudomonas sp. SGb136 16S ribosomal RNA gene, partial ..

Score:1932 Evalue:0

Accession:HQ224624.1

Alignment

Pseudomonas sp. 5(2011) 16S ribosomal RNA gene, partial..

Score:1932 Evalue:0

Accession:JF808098.1

Alignment

Uncultured Pseudomonas sp. clone EK\_An818 16S ribosomal..

Score:1932 Evalue:0

Accession:JN038283.1

Alignment

Uncultured bacterium clone 3-42017 16S ribosomal RNA ge..

Score:1932 Evalue:0

Accession:JN033052.1

Alignment

Pseudomonas sp. PG-3-10 16S ribosomal RNA gene, partial..

Score:1932 Evalue:0

Accession:JF820107.1

Alignment

Pseudomonas poae strain P6 16S ribosomal RNA gene, part..

Score:1932 Evalue:0

Accession:HQ647258.1

Alignment

Uncultured bacterium clone EDW07B003\_41 16S ribosomal R..

Score:1932 Evalue:0

Accession:HM066455.1

Alignment

Uncultured bacterium clone EDW07B001\_111 16S ribosomal ..

Score:1932 Evalue:0

Accession:HM066261.1

Alignment

## Descriptions

, Reading indexes 1-5, displaying indexes 1-5


Load next setPrevious Match

Sequences producing significant alignments:

Show all columns  of the table presenting sequences producing significant alignments 

Select:AllNone
Selected:0

Alignments
Download

FASTA (complete sequence)

FASTA (aligned sequences)

GenBank (complete sequence)

Hit Table (text)

Hit Table (CSV)

Text

XML

ASN.1

Continue
Cancel

GenBank 
Graphics
Distance tree of results
Multiple alignment
Show/hide columns of the table presenting sequences producing significant alignments 

Available columns

Description  
Max Score  
Total Score  
Coverage  
E-value  
IdentN  
Accession  
Restore Defaults
Ok
Cancel

Sequences producing significant alignments:

| Select for downloading or viewing reports | Description | Max score | Total score | Query cover | E value | Ident | Accession |
| --- | --- | --- | --- | --- | --- | --- | --- |
| 1Select seq GU213409.1 | Pseudomonas sp. 231 16S ribosomal RNA gene, partial sequence | 1941 | 1941 | 99% | 0.0 | 99% | GU213409.1 |
| 2Select seq HQ403088.2 | Pseudomonas sp. 2-36(2010) 16S ribosomal RNA gene, partial sequence | 1938 | 1938 | 99% | 0.0 | 99% | HQ403088.2 |
| 3Select seq HF545843.1 | Pseudomonas putida partial 16S rRNA gene, strain CFBP 5898 | 1938 | 1938 | 99% | 0.0 | 99% | HF545843.1 |
| 4Select seq KF003385.1 | Pseudomonas sp. ABAC21 16S ribosomal RNA gene, partial sequence | 1938 | 1938 | 99% | 0.0 | 99% | KF003385.1 |
| 5Select seq CP004045.1 | Pseudomonas poae RE\*1-1-14, complete genome | 1938 | 9691 | 99% | 0.0 | 99% | CP004045.1 |
| 6Select seq JX067683.1 | Pseudomonas sp. SAP770.2 16S ribosomal RNA gene, partial sequence | 1938 | 1938 | 99% | 0.0 | 99% | JX067683.1 |
| 7Select seq JF274937.1 | Pseudomonas sp. PX7\_S1 16S ribosomal RNA gene, partial sequence | 1938 | 1938 | 99% | 0.0 | 99% | JF274937.1 |
| 8Select seq EU111704.2 | Pseudomonas poae strain T23 16S ribosomal RNA gene, complete sequence | 1938 | 1938 | 99% | 0.0 | 99% | EU111704.2 |
| 9Select seq FJ262366.1 | Pseudomonas sp. WH1 16S ribosomal RNA gene, partial sequence | 1938 | 1938 | 99% | 0.0 | 99% | FJ262366.1 |
| 10Select seq MF590113.1 | Pseudomonas trivialis strain MAF3 16S ribosomal RNA gene, partial sequence | 1936 | 1936 | 99% | 0.0 | 99% | MF590113.1 |
| 11Select seq EU853182.1 | Pseudomonas sp. BR2-02 16S ribosomal RNA gene, partial sequence | 1936 | 1936 | 99% | 0.0 | 99% | EU853182.1 |
| 12Select seq KM187375.1 | Pseudomonas sp. HP10C 16S ribosomal RNA gene, partial sequence | 1934 | 1934 | 99% | 0.0 | 99% | KM187375.1 |
| 13Select seq KJ756337.1 | Pseudomonas fluorescens strain SWFU22 16S ribosomal RNA gene, partial sequence | 1934 | 1934 | 99% | 0.0 | 99% | KJ756337.1 |
| 14Select seq KJ756336.1 | Pseudomonas fluorescens strain SWFU21 16S ribosomal RNA gene, partial sequence | 1934 | 1934 | 99% | 0.0 | 99% | KJ756336.1 |
| 15Select seq JF701975.1 | Pseudomonas sp. YXE2-f 16S ribosomal RNA gene, partial sequence | 1934 | 1934 | 99% | 0.0 | 99% | JF701975.1 |
| 16Select seq EU853183.1 | Pseudomonas sp. BR2-22 16S ribosomal RNA gene, partial sequence | 1934 | 1934 | 99% | 0.0 | 99% | EU853183.1 |
| 17Select seq EF427849.1 | Pseudomonas sp. CBCEN8 16S ribosomal RNA gene, partial sequence | 1934 | 1934 | 99% | 0.0 | 99% | EF427849.1 |
| 18Select seq EF126755.1 | Pseudomonas sp. CBMB25 16S ribosomal RNA gene, partial sequence | 1934 | 1934 | 99% | 0.0 | 99% | EF126755.1 |
| 19Select seq EF126753.1 | Pseudomonas sp. CBMB18 16S ribosomal RNA gene, partial sequence | 1934 | 1934 | 99% | 0.0 | 99% | EF126753.1 |
| 20Select seq CP025263.1 | Pseudomonas sp. S09G 359 chromosome | 1932 | 11574 | 99% | 0.0 | 99% | CP025263.1 |
| 21Select seq MG576155.1 | Pseudomonas sp. strain KBL2 16S ribosomal RNA gene, partial sequence | 1932 | 1932 | 99% | 0.0 | 99% | MG576155.1 |
| 22Select seq KY810658.1 | Pseudomonas poae strain AAS-144 16S ribosomal RNA gene, partial sequence | 1932 | 1932 | 99% | 0.0 | 99% | KY810658.1 |
| 23Select seq KX588598.1 | Pseudomonas poae strain ICE349 16S ribosomal RNA gene, partial sequence | 1932 | 1932 | 99% | 0.0 | 99% | KX588598.1 |
| 24Select seq KX588597.1 | Pseudomonas poae strain ICE259 16S ribosomal RNA gene, partial sequence | 1932 | 1932 | 99% | 0.0 | 99% | KX588597.1 |
| 25Select seq LT629706.1 | Pseudomonas poae strain BS2776 genome assembly, chromosome: I | 1932 | 9663 | 99% | 0.0 | 99% | LT629706.1 |
| 26Select seq LT595870.1 | Uncultured bacterium partial 16S rRNA gene, clone clone AF-36051 | 1932 | 1932 | 99% | 0.0 | 99% | LT595870.1 |
| 27Select seq KX509247.1 | Uncultured bacterium clone MTWL201306-19 16S ribosomal RNA gene, partial sequence | 1932 | 1932 | 99% | 0.0 | 99% | KX509247.1 |
| 28Select seq LC140869.1 | Uncultured Pseudomonas sp. gene for 16S ribosomal RNA, partial sequence, clone: 89d159 | 1932 | 1932 | 99% | 0.0 | 99% | LC140869.1 |
| 29Select seq KT825765.1 | Pseudomonas sp. 23K33a 16S ribosomal RNA gene, partial sequence | 1932 | 1932 | 99% | 0.0 | 99% | KT825765.1 |
| 30Select seq KT825736.1 | Pseudomonas sp. 17K8 16S ribosomal RNA gene, partial sequence | 1932 | 1932 | 99% | 0.0 | 99% | KT825736.1 |
| 31Select seq KT767705.1 | Pseudomonas poae strain B42 16S ribosomal RNA gene, partial sequence | 1932 | 1932 | 99% | 0.0 | 99% | KT767705.1 |
| 32Select seq KR085862.1 | Pseudomonas poae strain IHBB 11151 16S ribosomal RNA gene, partial sequence | 1932 | 1932 | 99% | 0.0 | 99% | KR085862.1 |
| 33Select seq KP975307.1 | Uncultured bacterium clone OTU51 16S ribosomal RNA gene, partial sequence | 1932 | 1932 | 99% | 0.0 | 99% | KP975307.1 |
| 34Select seq CP005975.1 | Pseudomonas fluorescens PICF7, complete genome | 1932 | 11596 | 99% | 0.0 | 99% | CP005975.1 |
| 35Select seq CP010896.1 | Pseudomonas fluorescens strain PCL1751, complete genome | 1932 | 11579 | 99% | 0.0 | 99% | CP010896.1 |
| 36Select seq CP007637.1 | Pseudomonas simiae strain WCS417 genome | 1932 | 3865 | 99% | 0.0 | 99% | CP007637.1 |
| 37Select seq KJ420524.1 | Pseudomonas fluorescens strain PE8 16S ribosomal RNA gene, partial sequence | 1932 | 1932 | 99% | 0.0 | 99% | KJ420524.1 |
| 38Select seq KF979139.1 | Pseudomonas extremorientalis strain CNU082017 16S ribosomal RNA gene, partial sequence | 1932 | 1932 | 99% | 0.0 | 99% | KF979139.1 |
| 39Select seq KC618443.1 | Pseudomonas mandelii 16S ribosomal RNA gene, partial sequence | 1932 | 1932 | 99% | 0.0 | 99% | KC618443.1 |
| 40Select seq KF019106.1 | Pseudomonas sp. isolate PS2.1 16S ribosomal RNA gene, partial sequence | 1932 | 1932 | 99% | 0.0 | 99% | KF019106.1 |
| 41Select seq KC773764.1 | Pseudomonas fluorescens strain 20130311XA1 16S ribosomal RNA gene, partial sequence | 1932 | 1932 | 99% | 0.0 | 99% | KC773764.1 |
| 42Select seq JX515573.1 | Pseudomonas poae strain EB20 16S ribosomal RNA gene, partial sequence | 1932 | 1932 | 99% | 0.0 | 99% | JX515573.1 |
| 43Select seq JQ782898.1 | Pseudomonas poae strain zol-15 16S ribosomal RNA gene, partial sequence | 1932 | 1932 | 99% | 0.0 | 99% | JQ782898.1 |
| 44Select seq JQ638271.1 | Pseudomonas sp. ZBC1 16S ribosomal RNA gene, partial sequence | 1932 | 1932 | 99% | 0.0 | 99% | JQ638271.1 |
| 45Select seq AB705621.1 | Pseudomonas sp. TK-3 gene for 16S rRNA, partial sequence | 1932 | 1932 | 99% | 0.0 | 99% | AB705621.1 |
| 46Select seq HE576021.1 | uncultured bacterium partial 16S rRNA gene, clone K15.87 KSS | 1932 | 1932 | 99% | 0.0 | 99% | HE576021.1 |
| 47Select seq JQ236630.1 | Pseudomonas poae strain ORC5 16S ribosomal RNA gene, partial sequence | 1932 | 1932 | 99% | 0.0 | 99% | JQ236630.1 |
| 48Select seq HQ224626.1 | Pseudomonas sp. SGb192 16S ribosomal RNA gene, partial sequence | 1932 | 1932 | 99% | 0.0 | 99% | HQ224626.1 |
| 49Select seq HQ224624.1 | Pseudomonas sp. SGb136 16S ribosomal RNA gene, partial sequence | 1932 | 1932 | 99% | 0.0 | 99% | HQ224624.1 |
| 50Select seq JF808098.1 | Pseudomonas sp. 5(2011) 16S ribosomal RNA gene, partial sequence | 1932 | 1932 | 99% | 0.0 | 99% | JF808098.1 |
| 51Select seq JN038283.1 | Uncultured Pseudomonas sp. clone EK\_An818 16S ribosomal RNA gene, partial sequence | 1932 | 1932 | 99% | 0.0 | 99% | JN038283.1 |
| 52Select seq JN033052.1 | Uncultured bacterium clone 3-42017 16S ribosomal RNA gene, partial sequence | 1932 | 1932 | 99% | 0.0 | 99% | JN033052.1 |
| 53Select seq JF820107.1 | Pseudomonas sp. PG-3-10 16S ribosomal RNA gene, partial sequence | 1932 | 1932 | 99% | 0.0 | 99% | JF820107.1 |
| 54Select seq HQ647258.1 | Pseudomonas poae strain P6 16S ribosomal RNA gene, partial sequence | 1932 | 1932 | 99% | 0.0 | 99% | HQ647258.1 |
| 55Select seq HM066455.1 | Uncultured bacterium clone EDW07B003\_41 16S ribosomal RNA gene, partial sequence | 1932 | 1932 | 99% | 0.0 | 99% | HM066455.1 |
| 56Select seq HM066261.1 | Uncultured bacterium clone EDW07B001\_111 16S ribosomal RNA gene, partial sequence | 1932 | 1932 | 99% | 0.0 | 99% | HM066261.1 |
| 57Select seq HQ406827.1 | Pseudomonas poae strain Z6 16S ribosomal RNA gene, partial sequence | 1932 | 1932 | 99% | 0.0 | 99% | HQ406827.1 |
| 58Select seq GU563752.1 | Uncultured Pseudomonas sp. clone CHINA4 16S ribosomal RNA gene, partial sequence | 1932 | 1932 | 99% | 0.0 | 99% | GU563752.1 |
| 59Select seq NR\_028986.1 | Pseudomonas poae strain P 527/13 16S ribosomal RNA gene, partial sequence | 1932 | 1932 | 99% | 0.0 | 99% | NR\_028986.1 |
| 60Select seq EU853186.1 | Pseudomonas sp. BR3-39 16S ribosomal RNA gene, partial sequence | 1932 | 1932 | 98% | 0.0 | 99% | EU853186.1 |
| 61Select seq FJ937922.1 | Pseudomonas poae strain LS172 16S ribosomal RNA gene, partial sequence | 1932 | 1932 | 99% | 0.0 | 99% | FJ937922.1 |
| 62Select seq FJ179369.1 | Pseudomonas poae strain BIHB 808 16S ribosomal RNA gene, partial sequence | 1932 | 1932 | 99% | 0.0 | 99% | FJ179369.1 |
| 63Select seq EU360313.1 | Pseudomonas fluorescens strain 2312 16S ribosomal RNA gene and 16S-23S ribosomal RNA intergenic spacer, complete sequence; and 23S ribosomal RNA gene, partial sequence | 1932 | 1932 | 99% | 0.0 | 99% | EU360313.1 |
| 64Select seq EU364532.1 | Pseudomonas poae strain CN017 16S ribosomal RNA gene, partial sequence | 1932 | 1932 | 99% | 0.0 | 99% | EU364532.1 |
| 65Select seq EU169159.1 | Pseudomonas mandelii strain B61 16S ribosomal RNA gene, partial sequence | 1932 | 1932 | 99% | 0.0 | 99% | EU169159.1 |
| 66Select seq EF126756.1 | Pseudomonas sp. CBMB39 16S ribosomal RNA gene, partial sequence | 1932 | 1932 | 99% | 0.0 | 99% | EF126756.1 |
| 67Select seq DQ536513.1 | Pseudomonas poae strain BIHB 730 16S ribosomal RNA gene, partial sequence | 1932 | 1932 | 99% | 0.0 | 99% | DQ536513.1 |
| 68Select seq AJ492829.1 | Pseudomonas poae partial 16S rRNA gene, type strain DSM 14936T | 1932 | 1932 | 99% | 0.0 | 99% | AJ492829.1 |
| 69Select seq HG805798.1 | Pseudomonas sp. FBF130 partial 16S rRNA gene, isolate FBF130 | 1930 | 1930 | 99% | 0.0 | 99% | HG805798.1 |
| 70Select seq HG805797.1 | Pseudomonas sp. FBF128 partial 16S rRNA gene, isolate FBF128 | 1930 | 1930 | 99% | 0.0 | 99% | HG805797.1 |
| 71Select seq AY756059.1 | Pseudomonas sp. LBSA1 16S ribosomal RNA gene, partial sequence; internal transcribed spacer 1, complete sequence; and 23S ribosomal RNA gene, partial sequence | 1930 | 1930 | 99% | 0.0 | 99% | AY756059.1 |
| 72Select seq KX378900.1 | Pseudomonas sp. strain 1-29 16S ribosomal RNA gene, partial sequence | 1929 | 1929 | 99% | 0.0 | 99% | KX378900.1 |
| 73Select seq EU680981.1 | Pseudomonas sp. W15Feb3 16S ribosomal RNA gene, partial sequence | 1929 | 1929 | 99% | 0.0 | 99% | EU680981.1 |
| 74Select seq MG269664.1 | Pseudomonas extremorientalis strain 35D7 16S ribosomal RNA gene, partial sequence | 1927 | 1927 | 99% | 0.0 | 99% | MG269664.1 |
| 75Select seq MG269661.1 | Pseudomonas extremorientalis strain 35D2 16S ribosomal RNA gene, partial sequence | 1927 | 1927 | 99% | 0.0 | 99% | MG269661.1 |
| 76Select seq KY940313.1 | Pseudomonas poae strain A2B5 16S ribosomal RNA gene, partial sequence | 1927 | 1927 | 99% | 0.0 | 99% | KY940313.1 |
| 77Select seq KY228955.1 | Pseudomonas sp. strain 2 16S ribosomal RNA gene, partial sequence | 1927 | 1927 | 99% | 0.0 | 99% | KY228955.1 |
| 78Select seq LT629760.1 | Pseudomonas trivialis strain BS3111 genome assembly, chromosome: I | 1927 | 9630 | 99% | 0.0 | 99% | LT629760.1 |
| 79Select seq KT710812.1 | Pseudomonas sp. RZ110 16S ribosomal RNA gene, partial sequence | 1927 | 1927 | 99% | 0.0 | 99% | KT710812.1 |
| 80Select seq KT825845.1 | Pseudomonas sp. 8K8 16S ribosomal RNA gene, partial sequence | 1927 | 1927 | 99% | 0.0 | 99% | KT825845.1 |
| 81Select seq KT825760.1 | Pseudomonas sp. 23K17 16S ribosomal RNA gene, partial sequence | 1927 | 1927 | 99% | 0.0 | 99% | KT825760.1 |
| 82Select seq KT424973.1 | Pseudomonas sp. LB8 16S ribosomal RNA gene, partial sequence | 1927 | 1927 | 99% | 0.0 | 99% | KT424973.1 |
| 83Select seq HG805781.1 | Pseudomonas sp. FBF110 partial 16S rRNA gene, isolate FBF110 | 1927 | 1927 | 98% | 0.0 | 99% | HG805781.1 |
| 84Select seq LN875096.1 | Uncultured Pseudomonas sp. partial 16S rRNA gene, clone 133X76C\_34 | 1927 | 1927 | 99% | 0.0 | 99% | LN875096.1 |
| 85Select seq KR085822.1 | Pseudomonas poae strain IHBB 9211 16S ribosomal RNA gene, partial sequence | 1927 | 1927 | 99% | 0.0 | 99% | KR085822.1 |
| 86Select seq KT029390.1 | Uncultured bacterium clone 19-26 16S ribosomal RNA gene, partial sequence | 1927 | 1927 | 99% | 0.0 | 99% | KT029390.1 |
| 87Select seq KT029380.1 | Uncultured bacterium clone 19-16 16S ribosomal RNA gene, partial sequence | 1927 | 1927 | 99% | 0.0 | 99% | KT029380.1 |
| 88Select seq KT029378.1 | Uncultured bacterium clone 19-14 16S ribosomal RNA gene, partial sequence | 1927 | 1927 | 99% | 0.0 | 99% | KT029378.1 |
| 89Select seq CP011507.1 | Pseudomonas trivialis strain IHBB745, complete genome | 1927 | 9635 | 99% | 0.0 | 99% | CP011507.1 |
| 90Select seq KP204140.1 | Pseudomonas poae strain NA\_1 16S ribosomal RNA gene, partial sequence | 1927 | 1927 | 99% | 0.0 | 99% | KP204140.1 |
| 91Select seq KM589026.1 | Pseudomonas poae strain Pf9 16S ribosomal RNA gene, partial sequence | 1927 | 1927 | 99% | 0.0 | 99% | KM589026.1 |
| 92Select seq KM461107.1 | Pseudomonas sp. WHAS15 16S ribosomal RNA gene, partial sequence | 1927 | 1927 | 99% | 0.0 | 99% | KM461107.1 |
| 93Select seq KJ848593.1 | Pseudomonas poae strain BT 89 16S ribosomal RNA gene, partial sequence | 1927 | 1927 | 99% | 0.0 | 99% | KJ848593.1 |
| 94Select seq KJ420526.1 | Pseudomonas fluorescens strain PE16 16S ribosomal RNA gene, partial sequence | 1927 | 1927 | 99% | 0.0 | 99% | KJ420526.1 |
| 95Select seq KJ127239.1 | Pseudomonas fluorescens strain PE3 16S ribosomal RNA gene, partial sequence | 1927 | 1927 | 99% | 0.0 | 99% | KJ127239.1 |
| 96Select seq JQ977483.1 | Pseudomonas sp. Bma14 16S ribosomal RNA gene, partial sequence | 1927 | 1927 | 99% | 0.0 | 99% | JQ977483.1 |
| 97Select seq KC329818.1 | Pseudomonas extremorientalis strain 9F 16S ribosomal RNA gene, partial sequence | 1927 | 1927 | 99% | 0.0 | 99% | KC329818.1 |
| 98Select seq JX840381.1 | Pseudomonas salomonii strain CCNWQLS112 16S ribosomal RNA gene, partial sequence | 1927 | 1927 | 99% | 0.0 | 99% | JX840381.1 |
| 99Select seq JX949554.1 | Pseudomonas sp. MDT1-21 16S ribosomal RNA gene, partial sequence | 1927 | 1927 | 98% | 0.0 | 99% | JX949554.1 |
| 100Select seq JX994132.1 | Pseudomonas poae strain A2-R10 16S ribosomal RNA gene, partial sequence | 1927 | 1927 | 99% | 0.0 | 99% | JX994132.1 |


## Alignments

Loading alignment... for sequences gi|270282547,gi|752303983,gi|515782672,gi|514228662,gi|445198867 Reading indexes 1-5

Download

FASTA (complete sequence)

FASTA (aligned sequences)

GenBank (complete sequence)

Continue
Cancel

GenBankGraphics

Next
Previous
Descriptions

Pseudomonas sp. 231 16S ribosomal RNA gene, partial sequence

Sequence ID: GU213409.1Length: 1351Number of Matches: 1

Related Information

Range 1: 295 to 1351GenBankGraphics

Next Match
Previous Match
First Match

Alignment statistics for match #1

| Score | Expect | Identities | Gaps | Strand | Frame |
| --- | --- | --- | --- | --- | --- |
| 1941 bits(1051) | 0.0() | 1054/1057(99%) | 0/1057(0%) | Plus/Minus |  |

Features:

```
Query  5     TGGNACCGTCCTCCCGAAGGTTAGACTAGCTACTTCTGGTGCAACCCACTCCCATGGTGT  64
             ||| ||||||||||||||||||||||||||||||||||||||||||||||||||||||||
Sbjct  1351  TGGTACCGTCCTCCCGAAGGTTAGACTAGCTACTTCTGGTGCAACCCACTCCCATGGTGT  1292

Query  65    GACGGGCGGTGTGTACAAGGCCCGGGAACGTATTCACCGCGACATTCTGATTCGCGATTA  124
             ||||||||||||||||||||||||||||||||||||||||||||||||||||||||||||
Sbjct  1291  GACGGGCGGTGTGTACAAGGCCCGGGAACGTATTCACCGCGACATTCTGATTCGCGATTA  1232

Query  125   CTAGCGATTCCGACTTCACGCAGTCGAGTTGCAGACTGCGATCCGGACTACGATCGGTTT  184
             ||||||||||||||||||||||||||||||||||||||||||||||||||||||||||||
Sbjct  1231  CTAGCGATTCCGACTTCACGCAGTCGAGTTGCAGACTGCGATCCGGACTACGATCGGTTT  1172

Query  185   TATGGGATTAGCTCCACCTCGCGGCTTGGCAACCCTCTGTACCGACCATTGTAGCACGTG  244
             ||||||||||||||||||||||||||||||||||||||||||||||||||||||||||||
Sbjct  1171  TATGGGATTAGCTCCACCTCGCGGCTTGGCAACCCTCTGTACCGACCATTGTAGCACGTG  1112

Query  245   TGTAGCCCAGGCCGTAAGGGCCATGATGACTTGACGTCATCCCCACCTTCCTCCGGTTTG  304
             ||||||||||||||||||||||||||||||||||||||||||||||||||||||||||||
Sbjct  1111  TGTAGCCCAGGCCGTAAGGGCCATGATGACTTGACGTCATCCCCACCTTCCTCCGGTTTG  1052

Query  305   TCACCGGCAGTCTCCTTAGAGTGCCCACCATGACGTGCTGGTAACTAAGGACAAGGGTTG  364
             ||||||||||||||||||||||||||||||||||||||||||||||||||||||||||||
Sbjct  1051  TCACCGGCAGTCTCCTTAGAGTGCCCACCATGACGTGCTGGTAACTAAGGACAAGGGTTG  992

Query  365   CGCTCGTTACGGGACTTAACCCAACATCTCACGACACGAGCTGACGACAGCCATGCAGCA  424
             ||||||||||||||||||||||||||||||||||||||||||||||||||||||||||||
Sbjct  991   CGCTCGTTACGGGACTTAACCCAACATCTCACGACACGAGCTGACGACAGCCATGCAGCA  932

Query  425   CCTGTCTCAATGTTCCCGAAGGCACCAATCTATCTCTAGAAAGTTCATTGGATGTCAAGG  484
             ||||||||||||||||||||||||||||||||||||||||||||||||||||||||||||
Sbjct  931   CCTGTCTCAATGTTCCCGAAGGCACCAATCTATCTCTAGAAAGTTCATTGGATGTCAAGG  872

Query  485   CCTGGTAAGGTTCTTCGCGTTGCTTCGAATTAAACCACATGCTCCACCGCTTGTGCGGGC  544
             ||||||||||||||||||||||||||||||||||||||||||||||||||||||||||||
Sbjct  871   CCTGGTAAGGTTCTTCGCGTTGCTTCGAATTAAACCACATGCTCCACCGCTTGTGCGGGC  812

Query  545   CCCCGTCAATTCATTTGAGTTTTAACCTTGCGGCCGTACTCCCCAGGCGGTCAACTTAAT  604
             ||||||||||||||||||||||||||||||||||||||||||||||||||||||||||||
Sbjct  811   CCCCGTCAATTCATTTGAGTTTTAACCTTGCGGCCGTACTCCCCAGGCGGTCAACTTAAT  752

Query  605   GCGTTAGCTGCGCCACTAAAAGCTCAAGGCTTCCAACGGCTAGTTGACATCGTTTACGGC  664
             ||||||||||||||||||||||||||||||||||||||||||||||||||||||||||||
Sbjct  751   GCGTTAGCTGCGCCACTAAAAGCTCAAGGCTTCCAACGGCTAGTTGACATCGTTTACGGC  692

Query  665   GTGGACTACCAGGGTATCTAATCCTGTTTGCTCCCCACGCTTTCGCACCTCAGTGTCAGT  724
             ||||||||||||||||||||||||||||||||||||||||||||||||||||||||||||
Sbjct  691   GTGGACTACCAGGGTATCTAATCCTGTTTGCTCCCCACGCTTTCGCACCTCAGTGTCAGT  632

Query  725   ATTAGTCCAGGTGGTCGCCTTCGCCACTGGTGTTCCTTCCTATATCTACGCATTTCACCG  784
             ||||||||||||||||||||||||||||||||||||||||||||||||||||||||||||
Sbjct  631   ATTAGTCCAGGTGGTCGCCTTCGCCACTGGTGTTCCTTCCTATATCTACGCATTTCACCG  572

Query  785   CTACACAGGAAATTCCACCACCCTCTACCATACTCTAGTCAGTCAGTTTTGAATGCAGTT  844
             ||||||||||||||||||||||||||||||||||||||||||||||||||||||||||||
Sbjct  571   CTACACAGGAAATTCCACCACCCTCTACCATACTCTAGTCAGTCAGTTTTGAATGCAGTT  512

Query  845   CCCAGGTTGAGCCCGGGGATTTCACATCCAACTTAACAAACCACCTACGCGCGCTTTACG  904
             ||||||||||||||||||||||||||||||||||||||||||||||||||||||||||||
Sbjct  511   CCCAGGTTGAGCCCGGGGATTTCACATCCAACTTAACAAACCACCTACGCGCGCTTTACG  452

Query  905   CCCAGTAATTCCGATTAACGCTTGCACCCTCTGTATTACCGCGGCTGCTGGCACAGAGTT  964
             ||||||||||||||||||||||||||||||||||||||||||||||||||||||||||||
Sbjct  451   CCCAGTAATTCCGATTAACGCTTGCACCCTCTGTATTACCGCGGCTGCTGGCACAGAGTT  392

Query  965   AGCCGGTGCTTATTCTGTCGGTAACGTCAAAACAATCACGTATTAGGTAACTGCCCTTCC  1024
             ||||||||||||||||||||||||||||||||||||||||||||||||||||||||||||
Sbjct  391   AGCCGGTGCTTATTCTGTCGGTAACGTCAAAACAATCACGTATTAGGTAACTGCCCTTCC  332

Query  1025  TCCCAACTTAAAGTGCTTTACAATCCGAANNCCTTCT  1061
             |||||||||||||||||||||||||||||  ||||||
Sbjct  331   TCCCAACTTAAAGTGCTTTACAATCCGAAGACCTTCT  295
```

Download

FASTA (complete sequence)

FASTA (aligned sequences)

GenBank (complete sequence)

Continue
Cancel

GenBankGraphics

Next
Previous
Descriptions

Pseudomonas sp. 2-36(2010) 16S ribosomal RNA gene, partial sequence

Sequence ID: HQ403088.2Length: 1410Number of Matches: 1

Related Information

Range 1: 350 to 1407GenBankGraphics

Next Match
Previous Match
First Match

Alignment statistics for match #1

| Score | Expect | Identities | Gaps | Strand | Frame |
| --- | --- | --- | --- | --- | --- |
| 1938 bits(1049) | 0.0() | 1054/1058(99%) | 0/1058(0%) | Plus/Minus |  |

Features:

```
Query  4     GTGGNACCGTCCTCCCGAAGGTTAGACTAGCTACTTCTGGTGCAACCCACTCCCATGGTG  63
             |||| |||||||||||||||||||||||||||||||||||||||||||||||||||||||
Sbjct  1407  GTGGTACCGTCCTCCCGAAGGTTAGACTAGCTACTTCTGGTGCAACCCACTCCCATGGTG  1348

Query  64    TGACGGGCGGTGTGTACAAGGCCCGGGAACGTATTCACCGCGACATTCTGATTCGCGATT  123
             ||||||||||||||||||||||||||||||||||||||||||||||||||||||||||||
Sbjct  1347  TGACGGGCGGTGTGTACAAGGCCCGGGAACGTATTCACCGCGACATTCTGATTCGCGATT  1288

Query  124   ACTAGCGATTCCGACTTCACGCAGTCGAGTTGCAGACTGCGATCCGGACTACGATCGGTT  183
             ||||||||||||||||||||||||||||||||||||||||||||||||||||||||||||
Sbjct  1287  ACTAGCGATTCCGACTTCACGCAGTCGAGTTGCAGACTGCGATCCGGACTACGATCGGTT  1228

Query  184   TTATGGGATTAGCTCCACCTCGCGGCTTGGCAACCCTCTGTACCGACCATTGTAGCACGT  243
             ||||||||||||||||||||||||||||||||||||||||||||||||||||||||||||
Sbjct  1227  TTATGGGATTAGCTCCACCTCGCGGCTTGGCAACCCTCTGTACCGACCATTGTAGCACGT  1168

Query  244   GTGTAGCCCAGGCCGTAAGGGCCATGATGACTTGACGTCATCCCCACCTTCCTCCGGTTT  303
             ||||||||||||||||||||||||||||||||||||||||||||||||||||||||||||
Sbjct  1167  GTGTAGCCCAGGCCGTAAGGGCCATGATGACTTGACGTCATCCCCACCTTCCTCCGGTTT  1108

Query  304   GTCACCGGCAGTCTCCTTAGAGTGCCCACCATGACGTGCTGGTAACTAAGGACAAGGGTT  363
             |||||||||||||||||||||||||||||||| |||||||||||||||||||||||||||
Sbjct  1107  GTCACCGGCAGTCTCCTTAGAGTGCCCACCATTACGTGCTGGTAACTAAGGACAAGGGTT  1048

Query  364   GCGCTCGTTACGGGACTTAACCCAACATCTCACGACACGAGCTGACGACAGCCATGCAGC  423
             ||||||||||||||||||||||||||||||||||||||||||||||||||||||||||||
Sbjct  1047  GCGCTCGTTACGGGACTTAACCCAACATCTCACGACACGAGCTGACGACAGCCATGCAGC  988

Query  424   ACCTGTCTCAATGTTCCCGAAGGCACCAATCTATCTCTAGAAAGTTCATTGGATGTCAAG  483
             ||||||||||||||||||||||||||||||||||||||||||||||||||||||||||||
Sbjct  987   ACCTGTCTCAATGTTCCCGAAGGCACCAATCTATCTCTAGAAAGTTCATTGGATGTCAAG  928

Query  484   GCCTGGTAAGGTTCTTCGCGTTGCTTCGAATTAAACCACATGCTCCACCGCTTGTGCGGG  543
             ||||||||||||||||||||||||||||||||||||||||||||||||||||||||||||
Sbjct  927   GCCTGGTAAGGTTCTTCGCGTTGCTTCGAATTAAACCACATGCTCCACCGCTTGTGCGGG  868

Query  544   CCCCCGTCAATTCATTTGAGTTTTAACCTTGCGGCCGTACTCCCCAGGCGGTCAACTTAA  603
             ||||||||||||||||||||||||||||||||||||||||||||||||||||||||||||
Sbjct  867   CCCCCGTCAATTCATTTGAGTTTTAACCTTGCGGCCGTACTCCCCAGGCGGTCAACTTAA  808

Query  604   TGCGTTAGCTGCGCCACTAAAAGCTCAAGGCTTCCAACGGCTAGTTGACATCGTTTACGG  663
             ||||||||||||||||||||||||||||||||||||||||||||||||||||||||||||
Sbjct  807   TGCGTTAGCTGCGCCACTAAAAGCTCAAGGCTTCCAACGGCTAGTTGACATCGTTTACGG  748

Query  664   CGTGGACTACCAGGGTATCTAATCCTGTTTGCTCCCCACGCTTTCGCACCTCAGTGTCAG  723
             ||||||||||||||||||||||||||||||||||||||||||||||||||||||||||||
Sbjct  747   CGTGGACTACCAGGGTATCTAATCCTGTTTGCTCCCCACGCTTTCGCACCTCAGTGTCAG  688

Query  724   TATTAGTCCAGGTGGTCGCCTTCGCCACTGGTGTTCCTTCCTATATCTACGCATTTCACC  783
             ||||||||||||||||||||||||||||||||||||||||||||||||||||||||||||
Sbjct  687   TATTAGTCCAGGTGGTCGCCTTCGCCACTGGTGTTCCTTCCTATATCTACGCATTTCACC  628

Query  784   GCTACACAGGAAATTCCACCACCCTCTACCATACTCTAGTCAGTCAGTTTTGAATGCAGT  843
             ||||||||||||||||||||||||||||||||||||||||||||||||||||||||||||
Sbjct  627   GCTACACAGGAAATTCCACCACCCTCTACCATACTCTAGTCAGTCAGTTTTGAATGCAGT  568

Query  844   TCCCAGGTTGAGCCCGGGGATTTCACATCCAACTTAACAAACCACCTACGCGCGCTTTAC  903
             ||||||||||||||||||||||||||||||||||||||||||||||||||||||||||||
Sbjct  567   TCCCAGGTTGAGCCCGGGGATTTCACATCCAACTTAACAAACCACCTACGCGCGCTTTAC  508

Query  904   GCCCAGTAATTCCGATTAACGCTTGCACCCTCTGTATTACCGCGGCTGCTGGCACAGAGT  963
             ||||||||||||||||||||||||||||||||||||||||||||||||||||||||||||
Sbjct  507   GCCCAGTAATTCCGATTAACGCTTGCACCCTCTGTATTACCGCGGCTGCTGGCACAGAGT  448

Query  964   TAGCCGGTGCTTATTCTGTCGGTAACGTCAAAACAATCACGTATTAGGTAACTGCCCTTC  1023
             ||||||||||||||||||||||||||||||||||||||||||||||||||||||||||||
Sbjct  447   TAGCCGGTGCTTATTCTGTCGGTAACGTCAAAACAATCACGTATTAGGTAACTGCCCTTC  388

Query  1024  CTCCCAACTTAAAGTGCTTTACAATCCGAANNCCTTCT  1061
             ||||||||||||||||||||||||||||||  ||||||
Sbjct  387   CTCCCAACTTAAAGTGCTTTACAATCCGAAGACCTTCT  350
```

Download

FASTA (complete sequence)

FASTA (aligned sequences)

GenBank (complete sequence)

Continue
Cancel

GenBankGraphics

Next
Previous
Descriptions

Pseudomonas putida partial 16S rRNA gene, strain CFBP 5898

Sequence ID: HF545843.1Length: 1418Number of Matches: 1

Related Information

Range 1: 358 to 1415GenBankGraphics

Next Match
Previous Match
First Match

Alignment statistics for match #1

| Score | Expect | Identities | Gaps | Strand | Frame |
| --- | --- | --- | --- | --- | --- |
| 1938 bits(1049) | 0.0() | 1054/1058(99%) | 0/1058(0%) | Plus/Minus |  |

Features:

```
Query  4     GTGGNACCGTCCTCCCGAAGGTTAGACTAGCTACTTCTGGTGCAACCCACTCCCATGGTG  63
             |||| |||||||||||||||||||||||||||||||||||||||||||||||||||||||
Sbjct  1415  GTGGTACCGTCCTCCCGAAGGTTAGACTAGCTACTTCTGGTGCAACCCACTCCCATGGTG  1356

Query  64    TGACGGGCGGTGTGTACAAGGCCCGGGAACGTATTCACCGCGACATTCTGATTCGCGATT  123
             ||||||||||||||||||||||||||||||||||||||||||||||||||||||||||||
Sbjct  1355  TGACGGGCGGTGTGTACAAGGCCCGGGAACGTATTCACCGCGACATTCTGATTCGCGATT  1296

Query  124   ACTAGCGATTCCGACTTCACGCAGTCGAGTTGCAGACTGCGATCCGGACTACGATCGGTT  183
             ||||||||||||||||||||||||||||||||||||||||||||||||||||||||||||
Sbjct  1295  ACTAGCGATTCCGACTTCACGCAGTCGAGTTGCAGACTGCGATCCGGACTACGATCGGTT  1236

Query  184   TTATGGGATTAGCTCCACCTCGCGGCTTGGCAACCCTCTGTACCGACCATTGTAGCACGT  243
             ||||||||||||||||||||||||||||||||||||||||||||||||||||||||||||
Sbjct  1235  TTATGGGATTAGCTCCACCTCGCGGCTTGGCAACCCTCTGTACCGACCATTGTAGCACGT  1176

Query  244   GTGTAGCCCAGGCCGTAAGGGCCATGATGACTTGACGTCATCCCCACCTTCCTCCGGTTT  303
             ||||||||||||||||||||||||||||||||||||||||||||||||||||||||||||
Sbjct  1175  GTGTAGCCCAGGCCGTAAGGGCCATGATGACTTGACGTCATCCCCACCTTCCTCCGGTTT  1116

Query  304   GTCACCGGCAGTCTCCTTAGAGTGCCCACCATGACGTGCTGGTAACTAAGGACAAGGGTT  363
             |||||||||||||||||||||||||||||||| |||||||||||||||||||||||||||
Sbjct  1115  GTCACCGGCAGTCTCCTTAGAGTGCCCACCATTACGTGCTGGTAACTAAGGACAAGGGTT  1056

Query  364   GCGCTCGTTACGGGACTTAACCCAACATCTCACGACACGAGCTGACGACAGCCATGCAGC  423
             ||||||||||||||||||||||||||||||||||||||||||||||||||||||||||||
Sbjct  1055  GCGCTCGTTACGGGACTTAACCCAACATCTCACGACACGAGCTGACGACAGCCATGCAGC  996

Query  424   ACCTGTCTCAATGTTCCCGAAGGCACCAATCTATCTCTAGAAAGTTCATTGGATGTCAAG  483
             ||||||||||||||||||||||||||||||||||||||||||||||||||||||||||||
Sbjct  995   ACCTGTCTCAATGTTCCCGAAGGCACCAATCTATCTCTAGAAAGTTCATTGGATGTCAAG  936

Query  484   GCCTGGTAAGGTTCTTCGCGTTGCTTCGAATTAAACCACATGCTCCACCGCTTGTGCGGG  543
             ||||||||||||||||||||||||||||||||||||||||||||||||||||||||||||
Sbjct  935   GCCTGGTAAGGTTCTTCGCGTTGCTTCGAATTAAACCACATGCTCCACCGCTTGTGCGGG  876

Query  544   CCCCCGTCAATTCATTTGAGTTTTAACCTTGCGGCCGTACTCCCCAGGCGGTCAACTTAA  603
             ||||||||||||||||||||||||||||||||||||||||||||||||||||||||||||
Sbjct  875   CCCCCGTCAATTCATTTGAGTTTTAACCTTGCGGCCGTACTCCCCAGGCGGTCAACTTAA  816

Query  604   TGCGTTAGCTGCGCCACTAAAAGCTCAAGGCTTCCAACGGCTAGTTGACATCGTTTACGG  663
             ||||||||||||||||||||||||||||||||||||||||||||||||||||||||||||
Sbjct  815   TGCGTTAGCTGCGCCACTAAAAGCTCAAGGCTTCCAACGGCTAGTTGACATCGTTTACGG  756

Query  664   CGTGGACTACCAGGGTATCTAATCCTGTTTGCTCCCCACGCTTTCGCACCTCAGTGTCAG  723
             ||||||||||||||||||||||||||||||||||||||||||||||||||||||||||||
Sbjct  755   CGTGGACTACCAGGGTATCTAATCCTGTTTGCTCCCCACGCTTTCGCACCTCAGTGTCAG  696

Query  724   TATTAGTCCAGGTGGTCGCCTTCGCCACTGGTGTTCCTTCCTATATCTACGCATTTCACC  783
             ||||||||||||||||||||||||||||||||||||||||||||||||||||||||||||
Sbjct  695   TATTAGTCCAGGTGGTCGCCTTCGCCACTGGTGTTCCTTCCTATATCTACGCATTTCACC  636

Query  784   GCTACACAGGAAATTCCACCACCCTCTACCATACTCTAGTCAGTCAGTTTTGAATGCAGT  843
             ||||||||||||||||||||||||||||||||||||||||||||||||||||||||||||
Sbjct  635   GCTACACAGGAAATTCCACCACCCTCTACCATACTCTAGTCAGTCAGTTTTGAATGCAGT  576

Query  844   TCCCAGGTTGAGCCCGGGGATTTCACATCCAACTTAACAAACCACCTACGCGCGCTTTAC  903
             ||||||||||||||||||||||||||||||||||||||||||||||||||||||||||||
Sbjct  575   TCCCAGGTTGAGCCCGGGGATTTCACATCCAACTTAACAAACCACCTACGCGCGCTTTAC  516

Query  904   GCCCAGTAATTCCGATTAACGCTTGCACCCTCTGTATTACCGCGGCTGCTGGCACAGAGT  963
             ||||||||||||||||||||||||||||||||||||||||||||||||||||||||||||
Sbjct  515   GCCCAGTAATTCCGATTAACGCTTGCACCCTCTGTATTACCGCGGCTGCTGGCACAGAGT  456

Query  964   TAGCCGGTGCTTATTCTGTCGGTAACGTCAAAACAATCACGTATTAGGTAACTGCCCTTC  1023
             ||||||||||||||||||||||||||||||||||||||||||||||||||||||||||||
Sbjct  455   TAGCCGGTGCTTATTCTGTCGGTAACGTCAAAACAATCACGTATTAGGTAACTGCCCTTC  396

Query  1024  CTCCCAACTTAAAGTGCTTTACAATCCGAANNCCTTCT  1061
             ||||||||||||||||||||||||||||||  ||||||
Sbjct  395   CTCCCAACTTAAAGTGCTTTACAATCCGAAGACCTTCT  358
```

Download

FASTA (complete sequence)

FASTA (aligned sequences)

GenBank (complete sequence)

Continue
Cancel

GenBankGraphics

Next
Previous
Descriptions

Pseudomonas sp. ABAC21 16S ribosomal RNA gene, partial sequence

Sequence ID: KF003385.1Length: 1343Number of Matches: 1

Related Information

Range 1: 290 to 1342GenBankGraphics

Next Match
Previous Match
First Match

Alignment statistics for match #1

| Score | Expect | Identities | Gaps | Strand | Frame |
| --- | --- | --- | --- | --- | --- |
| 1938 bits(1049) | 0.0() | 1051/1053(99%) | 0/1053(0%) | Plus/Minus |  |

Features:

```
Query  9     ACCGTCCTCCCGAAGGTTAGACTAGCTACTTCTGGTGCAACCCACTCCCATGGTGTGACG  68
             ||||||||||||||||||||||||||||||||||||||||||||||||||||||||||||
Sbjct  1342  ACCGTCCTCCCGAAGGTTAGACTAGCTACTTCTGGTGCAACCCACTCCCATGGTGTGACG  1283

Query  69    GGCGGTGTGTACAAGGCCCGGGAACGTATTCACCGCGACATTCTGATTCGCGATTACTAG  128
             ||||||||||||||||||||||||||||||||||||||||||||||||||||||||||||
Sbjct  1282  GGCGGTGTGTACAAGGCCCGGGAACGTATTCACCGCGACATTCTGATTCGCGATTACTAG  1223

Query  129   CGATTCCGACTTCACGCAGTCGAGTTGCAGACTGCGATCCGGACTACGATCGGTTTTATG  188
             ||||||||||||||||||||||||||||||||||||||||||||||||||||||||||||
Sbjct  1222  CGATTCCGACTTCACGCAGTCGAGTTGCAGACTGCGATCCGGACTACGATCGGTTTTATG  1163

Query  189   GGATTAGCTCCACCTCGCGGCTTGGCAACCCTCTGTACCGACCATTGTAGCACGTGTGTA  248
             ||||||||||||||||||||||||||||||||||||||||||||||||||||||||||||
Sbjct  1162  GGATTAGCTCCACCTCGCGGCTTGGCAACCCTCTGTACCGACCATTGTAGCACGTGTGTA  1103

Query  249   GCCCAGGCCGTAAGGGCCATGATGACTTGACGTCATCCCCACCTTCCTCCGGTTTGTCAC  308
             ||||||||||||||||||||||||||||||||||||||||||||||||||||||||||||
Sbjct  1102  GCCCAGGCCGTAAGGGCCATGATGACTTGACGTCATCCCCACCTTCCTCCGGTTTGTCAC  1043

Query  309   CGGCAGTCTCCTTAGAGTGCCCACCATGACGTGCTGGTAACTAAGGACAAGGGTTGCGCT  368
             ||||||||||||||||||||||||||||||||||||||||||||||||||||||||||||
Sbjct  1042  CGGCAGTCTCCTTAGAGTGCCCACCATGACGTGCTGGTAACTAAGGACAAGGGTTGCGCT  983

Query  369   CGTTACGGGACTTAACCCAACATCTCACGACACGAGCTGACGACAGCCATGCAGCACCTG  428
             ||||||||||||||||||||||||||||||||||||||||||||||||||||||||||||
Sbjct  982   CGTTACGGGACTTAACCCAACATCTCACGACACGAGCTGACGACAGCCATGCAGCACCTG  923

Query  429   TCTCAATGTTCCCGAAGGCACCAATCTATCTCTAGAAAGTTCATTGGATGTCAAGGCCTG  488
             ||||||||||||||||||||||||||||||||||||||||||||||||||||||||||||
Sbjct  922   TCTCAATGTTCCCGAAGGCACCAATCTATCTCTAGAAAGTTCATTGGATGTCAAGGCCTG  863

Query  489   GTAAGGTTCTTCGCGTTGCTTCGAATTAAACCACATGCTCCACCGCTTGTGCGGGCCCCC  548
             ||||||||||||||||||||||||||||||||||||||||||||||||||||||||||||
Sbjct  862   GTAAGGTTCTTCGCGTTGCTTCGAATTAAACCACATGCTCCACCGCTTGTGCGGGCCCCC  803

Query  549   GTCAATTCATTTGAGTTTTAACCTTGCGGCCGTACTCCCCAGGCGGTCAACTTAATGCGT  608
             ||||||||||||||||||||||||||||||||||||||||||||||||||||||||||||
Sbjct  802   GTCAATTCATTTGAGTTTTAACCTTGCGGCCGTACTCCCCAGGCGGTCAACTTAATGCGT  743

Query  609   TAGCTGCGCCACTAAAAGCTCAAGGCTTCCAACGGCTAGTTGACATCGTTTACGGCGTGG  668
             ||||||||||||||||||||||||||||||||||||||||||||||||||||||||||||
Sbjct  742   TAGCTGCGCCACTAAAAGCTCAAGGCTTCCAACGGCTAGTTGACATCGTTTACGGCGTGG  683

Query  669   ACTACCAGGGTATCTAATCCTGTTTGCTCCCCACGCTTTCGCACCTCAGTGTCAGTATTA  728
             ||||||||||||||||||||||||||||||||||||||||||||||||||||||||||||
Sbjct  682   ACTACCAGGGTATCTAATCCTGTTTGCTCCCCACGCTTTCGCACCTCAGTGTCAGTATTA  623

Query  729   GTCCAGGTGGTCGCCTTCGCCACTGGTGTTCCTTCCTATATCTACGCATTTCACCGCTAC  788
             ||||||||||||||||||||||||||||||||||||||||||||||||||||||||||||
Sbjct  622   GTCCAGGTGGTCGCCTTCGCCACTGGTGTTCCTTCCTATATCTACGCATTTCACCGCTAC  563

Query  789   ACAGGAAATTCCACCACCCTCTACCATACTCTAGTCAGTCAGTTTTGAATGCAGTTCCCA  848
             ||||||||||||||||||||||||||||||||||||||||||||||||||||||||||||
Sbjct  562   ACAGGAAATTCCACCACCCTCTACCATACTCTAGTCAGTCAGTTTTGAATGCAGTTCCCA  503

Query  849   GGTTGAGCCCGGGGATTTCACATCCAACTTAACAAACCACCTACGCGCGCTTTACGCCCA  908
             ||||||||||||||||||||||||||||||||||||||||||||||||||||||||||||
Sbjct  502   GGTTGAGCCCGGGGATTTCACATCCAACTTAACAAACCACCTACGCGCGCTTTACGCCCA  443

Query  909   GTAATTCCGATTAACGCTTGCACCCTCTGTATTACCGCGGCTGCTGGCACAGAGTTAGCC  968
             ||||||||||||||||||||||||||||||||||||||||||||||||||||||||||||
Sbjct  442   GTAATTCCGATTAACGCTTGCACCCTCTGTATTACCGCGGCTGCTGGCACAGAGTTAGCC  383

Query  969   GGTGCTTATTCTGTCGGTAACGTCAAAACAATCACGTATTAGGTAACTGCCCTTCCTCCC  1028
             ||||||||||||||||||||||||||||||||||||||||||||||||||||||||||||
Sbjct  382   GGTGCTTATTCTGTCGGTAACGTCAAAACAATCACGTATTAGGTAACTGCCCTTCCTCCC  323

Query  1029  AACTTAAAGTGCTTTACAATCCGAANNCCTTCT  1061
             |||||||||||||||||||||||||  ||||||
Sbjct  322   AACTTAAAGTGCTTTACAATCCGAAGACCTTCT  290
```

Download

FASTA (complete sequence)

FASTA (aligned sequences)

GenBank (complete sequence)

Continue
Cancel

GenBankGraphics

Sort by:

E value
 Score
Percent identity
Query start position
Subject start position

Next
Previous
Descriptions

Pseudomonas poae RE\*1-1-14, complete genome

Sequence ID: CP004045.1Length: 5512241Number of Matches: 5

Related Information

Range 1: 1279690 to 1280742GenBankGraphics

Next Match
Previous Match
First Match

Alignment statistics for match #1

| Score | Expect | Identities | Gaps | Strand | Frame |
| --- | --- | --- | --- | --- | --- |
| 1938 bits(1049) | 0.0() | 1051/1053(99%) | 0/1053(0%) | Plus/Minus |  |

Features:

```
Query  9        ACCGTCCTCCCGAAGGTTAGACTAGCTACTTCTGGTGCAACCCACTCCCATGGTGTGACG  68
                ||||||||||||||||||||||||||||||||||||||||||||||||||||||||||||
Sbjct  1280742  ACCGTCCTCCCGAAGGTTAGACTAGCTACTTCTGGTGCAACCCACTCCCATGGTGTGACG  1280683

Query  69       GGCGGTGTGTACAAGGCCCGGGAACGTATTCACCGCGACATTCTGATTCGCGATTACTAG  128
                ||||||||||||||||||||||||||||||||||||||||||||||||||||||||||||
Sbjct  1280682  GGCGGTGTGTACAAGGCCCGGGAACGTATTCACCGCGACATTCTGATTCGCGATTACTAG  1280623

Query  129      CGATTCCGACTTCACGCAGTCGAGTTGCAGACTGCGATCCGGACTACGATCGGTTTTATG  188
                ||||||||||||||||||||||||||||||||||||||||||||||||||||||||||||
Sbjct  1280622  CGATTCCGACTTCACGCAGTCGAGTTGCAGACTGCGATCCGGACTACGATCGGTTTTATG  1280563

Query  189      GGATTAGCTCCACCTCGCGGCTTGGCAACCCTCTGTACCGACCATTGTAGCACGTGTGTA  248
                ||||||||||||||||||||||||||||||||||||||||||||||||||||||||||||
Sbjct  1280562  GGATTAGCTCCACCTCGCGGCTTGGCAACCCTCTGTACCGACCATTGTAGCACGTGTGTA  1280503

Query  249      GCCCAGGCCGTAAGGGCCATGATGACTTGACGTCATCCCCACCTTCCTCCGGTTTGTCAC  308
                ||||||||||||||||||||||||||||||||||||||||||||||||||||||||||||
Sbjct  1280502  GCCCAGGCCGTAAGGGCCATGATGACTTGACGTCATCCCCACCTTCCTCCGGTTTGTCAC  1280443

Query  309      CGGCAGTCTCCTTAGAGTGCCCACCATGACGTGCTGGTAACTAAGGACAAGGGTTGCGCT  368
                ||||||||||||||||||||||||||||||||||||||||||||||||||||||||||||
Sbjct  1280442  CGGCAGTCTCCTTAGAGTGCCCACCATGACGTGCTGGTAACTAAGGACAAGGGTTGCGCT  1280383

Query  369      CGTTACGGGACTTAACCCAACATCTCACGACACGAGCTGACGACAGCCATGCAGCACCTG  428
                ||||||||||||||||||||||||||||||||||||||||||||||||||||||||||||
Sbjct  1280382  CGTTACGGGACTTAACCCAACATCTCACGACACGAGCTGACGACAGCCATGCAGCACCTG  1280323

Query  429      TCTCAATGTTCCCGAAGGCACCAATCTATCTCTAGAAAGTTCATTGGATGTCAAGGCCTG  488
                ||||||||||||||||||||||||||||||||||||||||||||||||||||||||||||
Sbjct  1280322  TCTCAATGTTCCCGAAGGCACCAATCTATCTCTAGAAAGTTCATTGGATGTCAAGGCCTG  1280263

Query  489      GTAAGGTTCTTCGCGTTGCTTCGAATTAAACCACATGCTCCACCGCTTGTGCGGGCCCCC  548
                ||||||||||||||||||||||||||||||||||||||||||||||||||||||||||||
Sbjct  1280262  GTAAGGTTCTTCGCGTTGCTTCGAATTAAACCACATGCTCCACCGCTTGTGCGGGCCCCC  1280203

Query  549      GTCAATTCATTTGAGTTTTAACCTTGCGGCCGTACTCCCCAGGCGGTCAACTTAATGCGT  608
                ||||||||||||||||||||||||||||||||||||||||||||||||||||||||||||
Sbjct  1280202  GTCAATTCATTTGAGTTTTAACCTTGCGGCCGTACTCCCCAGGCGGTCAACTTAATGCGT  1280143

Query  609      TAGCTGCGCCACTAAAAGCTCAAGGCTTCCAACGGCTAGTTGACATCGTTTACGGCGTGG  668
                ||||||||||||||||||||||||||||||||||||||||||||||||||||||||||||
Sbjct  1280142  TAGCTGCGCCACTAAAAGCTCAAGGCTTCCAACGGCTAGTTGACATCGTTTACGGCGTGG  1280083

Query  669      ACTACCAGGGTATCTAATCCTGTTTGCTCCCCACGCTTTCGCACCTCAGTGTCAGTATTA  728
                ||||||||||||||||||||||||||||||||||||||||||||||||||||||||||||
Sbjct  1280082  ACTACCAGGGTATCTAATCCTGTTTGCTCCCCACGCTTTCGCACCTCAGTGTCAGTATTA  1280023

Query  729      GTCCAGGTGGTCGCCTTCGCCACTGGTGTTCCTTCCTATATCTACGCATTTCACCGCTAC  788
                ||||||||||||||||||||||||||||||||||||||||||||||||||||||||||||
Sbjct  1280022  GTCCAGGTGGTCGCCTTCGCCACTGGTGTTCCTTCCTATATCTACGCATTTCACCGCTAC  1279963

Query  789      ACAGGAAATTCCACCACCCTCTACCATACTCTAGTCAGTCAGTTTTGAATGCAGTTCCCA  848
                ||||||||||||||||||||||||||||||||||||||||||||||||||||||||||||
Sbjct  1279962  ACAGGAAATTCCACCACCCTCTACCATACTCTAGTCAGTCAGTTTTGAATGCAGTTCCCA  1279903

Query  849      GGTTGAGCCCGGGGATTTCACATCCAACTTAACAAACCACCTACGCGCGCTTTACGCCCA  908
                ||||||||||||||||||||||||||||||||||||||||||||||||||||||||||||
Sbjct  1279902  GGTTGAGCCCGGGGATTTCACATCCAACTTAACAAACCACCTACGCGCGCTTTACGCCCA  1279843

Query  909      GTAATTCCGATTAACGCTTGCACCCTCTGTATTACCGCGGCTGCTGGCACAGAGTTAGCC  968
                ||||||||||||||||||||||||||||||||||||||||||||||||||||||||||||
Sbjct  1279842  GTAATTCCGATTAACGCTTGCACCCTCTGTATTACCGCGGCTGCTGGCACAGAGTTAGCC  1279783

Query  969      GGTGCTTATTCTGTCGGTAACGTCAAAACAATCACGTATTAGGTAACTGCCCTTCCTCCC  1028
                ||||||||||||||||||||||||||||||||||||||||||||||||||||||||||||
Sbjct  1279782  GGTGCTTATTCTGTCGGTAACGTCAAAACAATCACGTATTAGGTAACTGCCCTTCCTCCC  1279723

Query  1029     AACTTAAAGTGCTTTACAATCCGAANNCCTTCT  1061
                |||||||||||||||||||||||||  ||||||
Sbjct  1279722  AACTTAAAGTGCTTTACAATCCGAAGACCTTCT  1279690
```

Range 2: 3808385 to 3809437GenBankGraphics

Next Match
Previous Match
First Match

Alignment statistics for match #2

| Score | Expect | Identities | Gaps | Strand | Frame |
| --- | --- | --- | --- | --- | --- |
| 1938 bits(1049) | 0.0() | 1051/1053(99%) | 0/1053(0%) | Plus/Plus |  |

Features:

```
Query  9        ACCGTCCTCCCGAAGGTTAGACTAGCTACTTCTGGTGCAACCCACTCCCATGGTGTGACG  68
                ||||||||||||||||||||||||||||||||||||||||||||||||||||||||||||
Sbjct  3808385  ACCGTCCTCCCGAAGGTTAGACTAGCTACTTCTGGTGCAACCCACTCCCATGGTGTGACG  3808444

Query  69       GGCGGTGTGTACAAGGCCCGGGAACGTATTCACCGCGACATTCTGATTCGCGATTACTAG  128
                ||||||||||||||||||||||||||||||||||||||||||||||||||||||||||||
Sbjct  3808445  GGCGGTGTGTACAAGGCCCGGGAACGTATTCACCGCGACATTCTGATTCGCGATTACTAG  3808504

Query  129      CGATTCCGACTTCACGCAGTCGAGTTGCAGACTGCGATCCGGACTACGATCGGTTTTATG  188
                ||||||||||||||||||||||||||||||||||||||||||||||||||||||||||||
Sbjct  3808505  CGATTCCGACTTCACGCAGTCGAGTTGCAGACTGCGATCCGGACTACGATCGGTTTTATG  3808564

Query  189      GGATTAGCTCCACCTCGCGGCTTGGCAACCCTCTGTACCGACCATTGTAGCACGTGTGTA  248
                ||||||||||||||||||||||||||||||||||||||||||||||||||||||||||||
Sbjct  3808565  GGATTAGCTCCACCTCGCGGCTTGGCAACCCTCTGTACCGACCATTGTAGCACGTGTGTA  3808624

Query  249      GCCCAGGCCGTAAGGGCCATGATGACTTGACGTCATCCCCACCTTCCTCCGGTTTGTCAC  308
                ||||||||||||||||||||||||||||||||||||||||||||||||||||||||||||
Sbjct  3808625  GCCCAGGCCGTAAGGGCCATGATGACTTGACGTCATCCCCACCTTCCTCCGGTTTGTCAC  3808684

Query  309      CGGCAGTCTCCTTAGAGTGCCCACCATGACGTGCTGGTAACTAAGGACAAGGGTTGCGCT  368
                ||||||||||||||||||||||||||||||||||||||||||||||||||||||||||||
Sbjct  3808685  CGGCAGTCTCCTTAGAGTGCCCACCATGACGTGCTGGTAACTAAGGACAAGGGTTGCGCT  3808744

Query  369      CGTTACGGGACTTAACCCAACATCTCACGACACGAGCTGACGACAGCCATGCAGCACCTG  428
                ||||||||||||||||||||||||||||||||||||||||||||||||||||||||||||
Sbjct  3808745  CGTTACGGGACTTAACCCAACATCTCACGACACGAGCTGACGACAGCCATGCAGCACCTG  3808804

Query  429      TCTCAATGTTCCCGAAGGCACCAATCTATCTCTAGAAAGTTCATTGGATGTCAAGGCCTG  488
                ||||||||||||||||||||||||||||||||||||||||||||||||||||||||||||
Sbjct  3808805  TCTCAATGTTCCCGAAGGCACCAATCTATCTCTAGAAAGTTCATTGGATGTCAAGGCCTG  3808864

Query  489      GTAAGGTTCTTCGCGTTGCTTCGAATTAAACCACATGCTCCACCGCTTGTGCGGGCCCCC  548
                ||||||||||||||||||||||||||||||||||||||||||||||||||||||||||||
Sbjct  3808865  GTAAGGTTCTTCGCGTTGCTTCGAATTAAACCACATGCTCCACCGCTTGTGCGGGCCCCC  3808924

Query  549      GTCAATTCATTTGAGTTTTAACCTTGCGGCCGTACTCCCCAGGCGGTCAACTTAATGCGT  608
                ||||||||||||||||||||||||||||||||||||||||||||||||||||||||||||
Sbjct  3808925  GTCAATTCATTTGAGTTTTAACCTTGCGGCCGTACTCCCCAGGCGGTCAACTTAATGCGT  3808984

Query  609      TAGCTGCGCCACTAAAAGCTCAAGGCTTCCAACGGCTAGTTGACATCGTTTACGGCGTGG  668
                ||||||||||||||||||||||||||||||||||||||||||||||||||||||||||||
Sbjct  3808985  TAGCTGCGCCACTAAAAGCTCAAGGCTTCCAACGGCTAGTTGACATCGTTTACGGCGTGG  3809044

Query  669      ACTACCAGGGTATCTAATCCTGTTTGCTCCCCACGCTTTCGCACCTCAGTGTCAGTATTA  728
                ||||||||||||||||||||||||||||||||||||||||||||||||||||||||||||
Sbjct  3809045  ACTACCAGGGTATCTAATCCTGTTTGCTCCCCACGCTTTCGCACCTCAGTGTCAGTATTA  3809104

Query  729      GTCCAGGTGGTCGCCTTCGCCACTGGTGTTCCTTCCTATATCTACGCATTTCACCGCTAC  788
                ||||||||||||||||||||||||||||||||||||||||||||||||||||||||||||
Sbjct  3809105  GTCCAGGTGGTCGCCTTCGCCACTGGTGTTCCTTCCTATATCTACGCATTTCACCGCTAC  3809164

Query  789      ACAGGAAATTCCACCACCCTCTACCATACTCTAGTCAGTCAGTTTTGAATGCAGTTCCCA  848
                ||||||||||||||||||||||||||||||||||||||||||||||||||||||||||||
Sbjct  3809165  ACAGGAAATTCCACCACCCTCTACCATACTCTAGTCAGTCAGTTTTGAATGCAGTTCCCA  3809224

Query  849      GGTTGAGCCCGGGGATTTCACATCCAACTTAACAAACCACCTACGCGCGCTTTACGCCCA  908
                ||||||||||||||||||||||||||||||||||||||||||||||||||||||||||||
Sbjct  3809225  GGTTGAGCCCGGGGATTTCACATCCAACTTAACAAACCACCTACGCGCGCTTTACGCCCA  3809284

Query  909      GTAATTCCGATTAACGCTTGCACCCTCTGTATTACCGCGGCTGCTGGCACAGAGTTAGCC  968
                ||||||||||||||||||||||||||||||||||||||||||||||||||||||||||||
Sbjct  3809285  GTAATTCCGATTAACGCTTGCACCCTCTGTATTACCGCGGCTGCTGGCACAGAGTTAGCC  3809344

Query  969      GGTGCTTATTCTGTCGGTAACGTCAAAACAATCACGTATTAGGTAACTGCCCTTCCTCCC  1028
                ||||||||||||||||||||||||||||||||||||||||||||||||||||||||||||
Sbjct  3809345  GGTGCTTATTCTGTCGGTAACGTCAAAACAATCACGTATTAGGTAACTGCCCTTCCTCCC  3809404

Query  1029     AACTTAAAGTGCTTTACAATCCGAANNCCTTCT  1061
                |||||||||||||||||||||||||  ||||||
Sbjct  3809405  AACTTAAAGTGCTTTACAATCCGAAGACCTTCT  3809437
```

Range 3: 4136793 to 4137845GenBankGraphics

Next Match
Previous Match
First Match

Alignment statistics for match #3

| Score | Expect | Identities | Gaps | Strand | Frame |
| --- | --- | --- | --- | --- | --- |
| 1938 bits(1049) | 0.0() | 1051/1053(99%) | 0/1053(0%) | Plus/Plus |  |

Features:

```
Query  9        ACCGTCCTCCCGAAGGTTAGACTAGCTACTTCTGGTGCAACCCACTCCCATGGTGTGACG  68
                ||||||||||||||||||||||||||||||||||||||||||||||||||||||||||||
Sbjct  4136793  ACCGTCCTCCCGAAGGTTAGACTAGCTACTTCTGGTGCAACCCACTCCCATGGTGTGACG  4136852

Query  69       GGCGGTGTGTACAAGGCCCGGGAACGTATTCACCGCGACATTCTGATTCGCGATTACTAG  128
                ||||||||||||||||||||||||||||||||||||||||||||||||||||||||||||
Sbjct  4136853  GGCGGTGTGTACAAGGCCCGGGAACGTATTCACCGCGACATTCTGATTCGCGATTACTAG  4136912

Query  129      CGATTCCGACTTCACGCAGTCGAGTTGCAGACTGCGATCCGGACTACGATCGGTTTTATG  188
                ||||||||||||||||||||||||||||||||||||||||||||||||||||||||||||
Sbjct  4136913  CGATTCCGACTTCACGCAGTCGAGTTGCAGACTGCGATCCGGACTACGATCGGTTTTATG  4136972

Query  189      GGATTAGCTCCACCTCGCGGCTTGGCAACCCTCTGTACCGACCATTGTAGCACGTGTGTA  248
                ||||||||||||||||||||||||||||||||||||||||||||||||||||||||||||
Sbjct  4136973  GGATTAGCTCCACCTCGCGGCTTGGCAACCCTCTGTACCGACCATTGTAGCACGTGTGTA  4137032

Query  249      GCCCAGGCCGTAAGGGCCATGATGACTTGACGTCATCCCCACCTTCCTCCGGTTTGTCAC  308
                ||||||||||||||||||||||||||||||||||||||||||||||||||||||||||||
Sbjct  4137033  GCCCAGGCCGTAAGGGCCATGATGACTTGACGTCATCCCCACCTTCCTCCGGTTTGTCAC  4137092

Query  309      CGGCAGTCTCCTTAGAGTGCCCACCATGACGTGCTGGTAACTAAGGACAAGGGTTGCGCT  368
                ||||||||||||||||||||||||||||||||||||||||||||||||||||||||||||
Sbjct  4137093  CGGCAGTCTCCTTAGAGTGCCCACCATGACGTGCTGGTAACTAAGGACAAGGGTTGCGCT  4137152

Query  369      CGTTACGGGACTTAACCCAACATCTCACGACACGAGCTGACGACAGCCATGCAGCACCTG  428
                ||||||||||||||||||||||||||||||||||||||||||||||||||||||||||||
Sbjct  4137153  CGTTACGGGACTTAACCCAACATCTCACGACACGAGCTGACGACAGCCATGCAGCACCTG  4137212

Query  429      TCTCAATGTTCCCGAAGGCACCAATCTATCTCTAGAAAGTTCATTGGATGTCAAGGCCTG  488
                ||||||||||||||||||||||||||||||||||||||||||||||||||||||||||||
Sbjct  4137213  TCTCAATGTTCCCGAAGGCACCAATCTATCTCTAGAAAGTTCATTGGATGTCAAGGCCTG  4137272

Query  489      GTAAGGTTCTTCGCGTTGCTTCGAATTAAACCACATGCTCCACCGCTTGTGCGGGCCCCC  548
                ||||||||||||||||||||||||||||||||||||||||||||||||||||||||||||
Sbjct  4137273  GTAAGGTTCTTCGCGTTGCTTCGAATTAAACCACATGCTCCACCGCTTGTGCGGGCCCCC  4137332

Query  549      GTCAATTCATTTGAGTTTTAACCTTGCGGCCGTACTCCCCAGGCGGTCAACTTAATGCGT  608
                ||||||||||||||||||||||||||||||||||||||||||||||||||||||||||||
Sbjct  4137333  GTCAATTCATTTGAGTTTTAACCTTGCGGCCGTACTCCCCAGGCGGTCAACTTAATGCGT  4137392

Query  609      TAGCTGCGCCACTAAAAGCTCAAGGCTTCCAACGGCTAGTTGACATCGTTTACGGCGTGG  668
                ||||||||||||||||||||||||||||||||||||||||||||||||||||||||||||
Sbjct  4137393  TAGCTGCGCCACTAAAAGCTCAAGGCTTCCAACGGCTAGTTGACATCGTTTACGGCGTGG  4137452

Query  669      ACTACCAGGGTATCTAATCCTGTTTGCTCCCCACGCTTTCGCACCTCAGTGTCAGTATTA  728
                ||||||||||||||||||||||||||||||||||||||||||||||||||||||||||||
Sbjct  4137453  ACTACCAGGGTATCTAATCCTGTTTGCTCCCCACGCTTTCGCACCTCAGTGTCAGTATTA  4137512

Query  729      GTCCAGGTGGTCGCCTTCGCCACTGGTGTTCCTTCCTATATCTACGCATTTCACCGCTAC  788
                ||||||||||||||||||||||||||||||||||||||||||||||||||||||||||||
Sbjct  4137513  GTCCAGGTGGTCGCCTTCGCCACTGGTGTTCCTTCCTATATCTACGCATTTCACCGCTAC  4137572

Query  789      ACAGGAAATTCCACCACCCTCTACCATACTCTAGTCAGTCAGTTTTGAATGCAGTTCCCA  848
                ||||||||||||||||||||||||||||||||||||||||||||||||||||||||||||
Sbjct  4137573  ACAGGAAATTCCACCACCCTCTACCATACTCTAGTCAGTCAGTTTTGAATGCAGTTCCCA  4137632

Query  849      GGTTGAGCCCGGGGATTTCACATCCAACTTAACAAACCACCTACGCGCGCTTTACGCCCA  908
                ||||||||||||||||||||||||||||||||||||||||||||||||||||||||||||
Sbjct  4137633  GGTTGAGCCCGGGGATTTCACATCCAACTTAACAAACCACCTACGCGCGCTTTACGCCCA  4137692

Query  909      GTAATTCCGATTAACGCTTGCACCCTCTGTATTACCGCGGCTGCTGGCACAGAGTTAGCC  968
                ||||||||||||||||||||||||||||||||||||||||||||||||||||||||||||
Sbjct  4137693  GTAATTCCGATTAACGCTTGCACCCTCTGTATTACCGCGGCTGCTGGCACAGAGTTAGCC  4137752

Query  969      GGTGCTTATTCTGTCGGTAACGTCAAAACAATCACGTATTAGGTAACTGCCCTTCCTCCC  1028
                ||||||||||||||||||||||||||||||||||||||||||||||||||||||||||||
Sbjct  4137753  GGTGCTTATTCTGTCGGTAACGTCAAAACAATCACGTATTAGGTAACTGCCCTTCCTCCC  4137812

Query  1029     AACTTAAAGTGCTTTACAATCCGAANNCCTTCT  1061
                |||||||||||||||||||||||||  ||||||
Sbjct  4137813  AACTTAAAGTGCTTTACAATCCGAAGACCTTCT  4137845
```

Range 4: 4900695 to 4901747GenBankGraphics

Next Match
Previous Match
First Match

Alignment statistics for match #4

| Score | Expect | Identities | Gaps | Strand | Frame |
| --- | --- | --- | --- | --- | --- |
| 1938 bits(1049) | 0.0() | 1051/1053(99%) | 0/1053(0%) | Plus/Minus |  |

Features:

```
Query  9        ACCGTCCTCCCGAAGGTTAGACTAGCTACTTCTGGTGCAACCCACTCCCATGGTGTGACG  68
                ||||||||||||||||||||||||||||||||||||||||||||||||||||||||||||
Sbjct  4901747  ACCGTCCTCCCGAAGGTTAGACTAGCTACTTCTGGTGCAACCCACTCCCATGGTGTGACG  4901688

Query  69       GGCGGTGTGTACAAGGCCCGGGAACGTATTCACCGCGACATTCTGATTCGCGATTACTAG  128
                ||||||||||||||||||||||||||||||||||||||||||||||||||||||||||||
Sbjct  4901687  GGCGGTGTGTACAAGGCCCGGGAACGTATTCACCGCGACATTCTGATTCGCGATTACTAG  4901628

Query  129      CGATTCCGACTTCACGCAGTCGAGTTGCAGACTGCGATCCGGACTACGATCGGTTTTATG  188
                ||||||||||||||||||||||||||||||||||||||||||||||||||||||||||||
Sbjct  4901627  CGATTCCGACTTCACGCAGTCGAGTTGCAGACTGCGATCCGGACTACGATCGGTTTTATG  4901568

Query  189      GGATTAGCTCCACCTCGCGGCTTGGCAACCCTCTGTACCGACCATTGTAGCACGTGTGTA  248
                ||||||||||||||||||||||||||||||||||||||||||||||||||||||||||||
Sbjct  4901567  GGATTAGCTCCACCTCGCGGCTTGGCAACCCTCTGTACCGACCATTGTAGCACGTGTGTA  4901508

Query  249      GCCCAGGCCGTAAGGGCCATGATGACTTGACGTCATCCCCACCTTCCTCCGGTTTGTCAC  308
                ||||||||||||||||||||||||||||||||||||||||||||||||||||||||||||
Sbjct  4901507  GCCCAGGCCGTAAGGGCCATGATGACTTGACGTCATCCCCACCTTCCTCCGGTTTGTCAC  4901448

Query  309      CGGCAGTCTCCTTAGAGTGCCCACCATGACGTGCTGGTAACTAAGGACAAGGGTTGCGCT  368
                ||||||||||||||||||||||||||||||||||||||||||||||||||||||||||||
Sbjct  4901447  CGGCAGTCTCCTTAGAGTGCCCACCATGACGTGCTGGTAACTAAGGACAAGGGTTGCGCT  4901388

Query  369      CGTTACGGGACTTAACCCAACATCTCACGACACGAGCTGACGACAGCCATGCAGCACCTG  428
                ||||||||||||||||||||||||||||||||||||||||||||||||||||||||||||
Sbjct  4901387  CGTTACGGGACTTAACCCAACATCTCACGACACGAGCTGACGACAGCCATGCAGCACCTG  4901328

Query  429      TCTCAATGTTCCCGAAGGCACCAATCTATCTCTAGAAAGTTCATTGGATGTCAAGGCCTG  488
                ||||||||||||||||||||||||||||||||||||||||||||||||||||||||||||
Sbjct  4901327  TCTCAATGTTCCCGAAGGCACCAATCTATCTCTAGAAAGTTCATTGGATGTCAAGGCCTG  4901268

Query  489      GTAAGGTTCTTCGCGTTGCTTCGAATTAAACCACATGCTCCACCGCTTGTGCGGGCCCCC  548
                ||||||||||||||||||||||||||||||||||||||||||||||||||||||||||||
Sbjct  4901267  GTAAGGTTCTTCGCGTTGCTTCGAATTAAACCACATGCTCCACCGCTTGTGCGGGCCCCC  4901208

Query  549      GTCAATTCATTTGAGTTTTAACCTTGCGGCCGTACTCCCCAGGCGGTCAACTTAATGCGT  608
                ||||||||||||||||||||||||||||||||||||||||||||||||||||||||||||
Sbjct  4901207  GTCAATTCATTTGAGTTTTAACCTTGCGGCCGTACTCCCCAGGCGGTCAACTTAATGCGT  4901148

Query  609      TAGCTGCGCCACTAAAAGCTCAAGGCTTCCAACGGCTAGTTGACATCGTTTACGGCGTGG  668
                ||||||||||||||||||||||||||||||||||||||||||||||||||||||||||||
Sbjct  4901147  TAGCTGCGCCACTAAAAGCTCAAGGCTTCCAACGGCTAGTTGACATCGTTTACGGCGTGG  4901088

Query  669      ACTACCAGGGTATCTAATCCTGTTTGCTCCCCACGCTTTCGCACCTCAGTGTCAGTATTA  728
                ||||||||||||||||||||||||||||||||||||||||||||||||||||||||||||
Sbjct  4901087  ACTACCAGGGTATCTAATCCTGTTTGCTCCCCACGCTTTCGCACCTCAGTGTCAGTATTA  4901028

Query  729      GTCCAGGTGGTCGCCTTCGCCACTGGTGTTCCTTCCTATATCTACGCATTTCACCGCTAC  788
                ||||||||||||||||||||||||||||||||||||||||||||||||||||||||||||
Sbjct  4901027  GTCCAGGTGGTCGCCTTCGCCACTGGTGTTCCTTCCTATATCTACGCATTTCACCGCTAC  4900968

Query  789      ACAGGAAATTCCACCACCCTCTACCATACTCTAGTCAGTCAGTTTTGAATGCAGTTCCCA  848
                ||||||||||||||||||||||||||||||||||||||||||||||||||||||||||||
Sbjct  4900967  ACAGGAAATTCCACCACCCTCTACCATACTCTAGTCAGTCAGTTTTGAATGCAGTTCCCA  4900908

Query  849      GGTTGAGCCCGGGGATTTCACATCCAACTTAACAAACCACCTACGCGCGCTTTACGCCCA  908
                ||||||||||||||||||||||||||||||||||||||||||||||||||||||||||||
Sbjct  4900907  GGTTGAGCCCGGGGATTTCACATCCAACTTAACAAACCACCTACGCGCGCTTTACGCCCA  4900848

Query  909      GTAATTCCGATTAACGCTTGCACCCTCTGTATTACCGCGGCTGCTGGCACAGAGTTAGCC  968
                ||||||||||||||||||||||||||||||||||||||||||||||||||||||||||||
Sbjct  4900847  GTAATTCCGATTAACGCTTGCACCCTCTGTATTACCGCGGCTGCTGGCACAGAGTTAGCC  4900788

Query  969      GGTGCTTATTCTGTCGGTAACGTCAAAACAATCACGTATTAGGTAACTGCCCTTCCTCCC  1028
                ||||||||||||||||||||||||||||||||||||||||||||||||||||||||||||
Sbjct  4900787  GGTGCTTATTCTGTCGGTAACGTCAAAACAATCACGTATTAGGTAACTGCCCTTCCTCCC  4900728

Query  1029     AACTTAAAGTGCTTTACAATCCGAANNCCTTCT  1061
                |||||||||||||||||||||||||  ||||||
Sbjct  4900727  AACTTAAAGTGCTTTACAATCCGAAGACCTTCT  4900695
```

Range 5: 5507293 to 5508345GenBankGraphics

Next Match
Previous Match
First Match

Alignment statistics for match #5

| Score | Expect | Identities | Gaps | Strand | Frame |
| --- | --- | --- | --- | --- | --- |
| 1938 bits(1049) | 0.0() | 1051/1053(99%) | 0/1053(0%) | Plus/Minus |  |

Features:

```
Query  9        ACCGTCCTCCCGAAGGTTAGACTAGCTACTTCTGGTGCAACCCACTCCCATGGTGTGACG  68
                ||||||||||||||||||||||||||||||||||||||||||||||||||||||||||||
Sbjct  5508345  ACCGTCCTCCCGAAGGTTAGACTAGCTACTTCTGGTGCAACCCACTCCCATGGTGTGACG  5508286

Query  69       GGCGGTGTGTACAAGGCCCGGGAACGTATTCACCGCGACATTCTGATTCGCGATTACTAG  128
                ||||||||||||||||||||||||||||||||||||||||||||||||||||||||||||
Sbjct  5508285  GGCGGTGTGTACAAGGCCCGGGAACGTATTCACCGCGACATTCTGATTCGCGATTACTAG  5508226

Query  129      CGATTCCGACTTCACGCAGTCGAGTTGCAGACTGCGATCCGGACTACGATCGGTTTTATG  188
                ||||||||||||||||||||||||||||||||||||||||||||||||||||||||||||
Sbjct  5508225  CGATTCCGACTTCACGCAGTCGAGTTGCAGACTGCGATCCGGACTACGATCGGTTTTATG  5508166

Query  189      GGATTAGCTCCACCTCGCGGCTTGGCAACCCTCTGTACCGACCATTGTAGCACGTGTGTA  248
                ||||||||||||||||||||||||||||||||||||||||||||||||||||||||||||
Sbjct  5508165  GGATTAGCTCCACCTCGCGGCTTGGCAACCCTCTGTACCGACCATTGTAGCACGTGTGTA  5508106

Query  249      GCCCAGGCCGTAAGGGCCATGATGACTTGACGTCATCCCCACCTTCCTCCGGTTTGTCAC  308
                ||||||||||||||||||||||||||||||||||||||||||||||||||||||||||||
Sbjct  5508105  GCCCAGGCCGTAAGGGCCATGATGACTTGACGTCATCCCCACCTTCCTCCGGTTTGTCAC  5508046

Query  309      CGGCAGTCTCCTTAGAGTGCCCACCATGACGTGCTGGTAACTAAGGACAAGGGTTGCGCT  368
                ||||||||||||||||||||||||||||||||||||||||||||||||||||||||||||
Sbjct  5508045  CGGCAGTCTCCTTAGAGTGCCCACCATGACGTGCTGGTAACTAAGGACAAGGGTTGCGCT  5507986

Query  369      CGTTACGGGACTTAACCCAACATCTCACGACACGAGCTGACGACAGCCATGCAGCACCTG  428
                ||||||||||||||||||||||||||||||||||||||||||||||||||||||||||||
Sbjct  5507985  CGTTACGGGACTTAACCCAACATCTCACGACACGAGCTGACGACAGCCATGCAGCACCTG  5507926

Query  429      TCTCAATGTTCCCGAAGGCACCAATCTATCTCTAGAAAGTTCATTGGATGTCAAGGCCTG  488
                ||||||||||||||||||||||||||||||||||||||||||||||||||||||||||||
Sbjct  5507925  TCTCAATGTTCCCGAAGGCACCAATCTATCTCTAGAAAGTTCATTGGATGTCAAGGCCTG  5507866

Query  489      GTAAGGTTCTTCGCGTTGCTTCGAATTAAACCACATGCTCCACCGCTTGTGCGGGCCCCC  548
                ||||||||||||||||||||||||||||||||||||||||||||||||||||||||||||
Sbjct  5507865  GTAAGGTTCTTCGCGTTGCTTCGAATTAAACCACATGCTCCACCGCTTGTGCGGGCCCCC  5507806

Query  549      GTCAATTCATTTGAGTTTTAACCTTGCGGCCGTACTCCCCAGGCGGTCAACTTAATGCGT  608
                ||||||||||||||||||||||||||||||||||||||||||||||||||||||||||||
Sbjct  5507805  GTCAATTCATTTGAGTTTTAACCTTGCGGCCGTACTCCCCAGGCGGTCAACTTAATGCGT  5507746

Query  609      TAGCTGCGCCACTAAAAGCTCAAGGCTTCCAACGGCTAGTTGACATCGTTTACGGCGTGG  668
                ||||||||||||||||||||||||||||||||||||||||||||||||||||||||||||
Sbjct  5507745  TAGCTGCGCCACTAAAAGCTCAAGGCTTCCAACGGCTAGTTGACATCGTTTACGGCGTGG  5507686

Query  669      ACTACCAGGGTATCTAATCCTGTTTGCTCCCCACGCTTTCGCACCTCAGTGTCAGTATTA  728
                ||||||||||||||||||||||||||||||||||||||||||||||||||||||||||||
Sbjct  5507685  ACTACCAGGGTATCTAATCCTGTTTGCTCCCCACGCTTTCGCACCTCAGTGTCAGTATTA  5507626

Query  729      GTCCAGGTGGTCGCCTTCGCCACTGGTGTTCCTTCCTATATCTACGCATTTCACCGCTAC  788
                ||||||||||||||||||||||||||||||||||||||||||||||||||||||||||||
Sbjct  5507625  GTCCAGGTGGTCGCCTTCGCCACTGGTGTTCCTTCCTATATCTACGCATTTCACCGCTAC  5507566

Query  789      ACAGGAAATTCCACCACCCTCTACCATACTCTAGTCAGTCAGTTTTGAATGCAGTTCCCA  848
                ||||||||||||||||||||||||||||||||||||||||||||||||||||||||||||
Sbjct  5507565  ACAGGAAATTCCACCACCCTCTACCATACTCTAGTCAGTCAGTTTTGAATGCAGTTCCCA  5507506

Query  849      GGTTGAGCCCGGGGATTTCACATCCAACTTAACAAACCACCTACGCGCGCTTTACGCCCA  908
                ||||||||||||||||||||||||||||||||||||||||||||||||||||||||||||
Sbjct  5507505  GGTTGAGCCCGGGGATTTCACATCCAACTTAACAAACCACCTACGCGCGCTTTACGCCCA  5507446

Query  909      GTAATTCCGATTAACGCTTGCACCCTCTGTATTACCGCGGCTGCTGGCACAGAGTTAGCC  968
                ||||||||||||||||||||||||||||||||||||||||||||||||||||||||||||
Sbjct  5507445  GTAATTCCGATTAACGCTTGCACCCTCTGTATTACCGCGGCTGCTGGCACAGAGTTAGCC  5507386

Query  969      GGTGCTTATTCTGTCGGTAACGTCAAAACAATCACGTATTAGGTAACTGCCCTTCCTCCC  1028
                ||||||||||||||||||||||||||||||||||||||||||||||||||||||||||||
Sbjct  5507385  GGTGCTTATTCTGTCGGTAACGTCAAAACAATCACGTATTAGGTAACTGCCCTTCCTCCC  5507326

Query  1029     AACTTAAAGTGCTTTACAATCCGAANNCCTTCT  1061
                |||||||||||||||||||||||||  ||||||
Sbjct  5507325  AACTTAAAGTGCTTTACAATCCGAAGACCTTCT  5507293
```


BLAST is a registered trademark of the National Library of Medicine

Support center
Mailing list


YouTube

- National Library Of Medicine
- National Institutes Of Health
- U.S. Department of Health & Human Services
- USA.gov

### NCBI


National Center for Biotechnology Information,
 U.S. National Library of Medicine

8600 Rockville Pike,
Bethesda
 MD,
20894
USA

Policies and Guidelines
|
Contact


PreferencesTurn off

External link. Please review our privacy policy.
