# Supplementary material for: Isothermal microcalorimetry for thermal viable count of microorganisms in pure cultures and stabilized formulations
Source: BMC Microbiol. 2019 Mar 21;19:65. doi: 10.1186/s12866-019-1432-8 (PMC6429831; doi:10.1186/s12866-019-1432-8)
Supplement: Supplementary file 3 — 16S rRNA gene sequences’ BLASTN hits in zipped HTML format. (ZIP 15810 kb) [file 12866_2019_1432_MOESM3_ESM.zip › Best blastn hits/NCBI Blast_B pale R -- 20..1069 of sequence (1050.html]

NCBI Blast:B pale R -- 20..1069 of sequence (1050


- NCBI Home
- Sign in to NCBI
- Skip to Main Content
- Skip to Navigation
- About NCBI Accesskeys

U.S. National Library of Medicine

NCBI
National Center for Biotechnology Information

- My NCBI
- Sign in to NCBI
- Register
- Sign Out

BLAST ^®^ » blastn suite » RID-A4XGZT1M014


- Home
- Recent Results
- Saved Strategies
- Help

BLAST Results


Edit and Resubmit
Save Search Strategies
[Sign in above to save your search strategy]

Formatting options 


Download


How to read this page
Blast report description
Questions/comments


|  |  |
| --- | --- |
| Formatting options | |
| Show | Alignment as  HTML Plain text   Old View Reset form to defaults [?]  These options control formatting of alignments in results pages. The default is HTML, but other formats (including plain text) are available. PSSM and PssmWithParameters are representations of Position Specific Scoring Matrices and are only available for PSI-BLAST. The Advanced view option allows the database descriptions to be sorted by various indices in a table. |
| Alignment View | Pairwise Pairwise with dots for identities Query-anchored with dots for identities Query-anchored with letters for identities Flat query-anchored with dots for identities Flat query-anchored with letters for identities [?]  Choose how to view alignments. The default "pairwise" view shows how each subject sequence aligns individually to the query sequence. The "query-anchored" view shows how all subject sequences align to the query sequence. For each view type, you can choose to show "identities" (matching residues) as letters or dots. more... |
| Display | Graphical Overview   Linkout   Sequence Retrieval  NCBI-gi   CDS feature [?]  - Graphical Overview: Graphical Overview: Show graph of similar sequence regions aligned to query.   more... - NCBI-gi: Show NCBI gi identifiers. - CDS feature: Show annotated coding region and translation.   more... |
| Masking | Character:   X for protein, n for nucleotide Lower Case  Color:  Black Grey Red [?]  - Masking Character: Display masked (filtered) sequence regions as lower-case or as specific letters (N for nucleotide, P for protein). - Masking Color: Display masked sequence regions in the given color. |
| Limit results | Descriptions:  10 50 100 Graphical overview:  0 10 50 100  Alignments:  0 10 50 100 Line length:  60 90 120 150 [?]  - Descriptions: Show short descriptions for up to the given number of sequences. - Alignments: Show alignments for up to the given number of sequences, in order of statistical significance. - Line lenghth: Number of letters to show on one line in an alignment. |
|  | Organism Type common name, binomial, taxid, or group name. Only 20 top taxa will be shown.     Exclude   [?]  Show only sequences from the given organism. |
|  | Entrez query:  [?]  Show only those sequences that match the given Entrez query. more... |
|  | Expect Min:  Expect Max:  [?]  Show only sequences with expect values in the given range. more... |
|  | Percent Identity Min:  Percent Identity Max:  [?]  Show only sequences with percent identity values in the given range. |
| Format for | PSI-BLAST with inclusion threshold:  [?]  - Format for PSI-BLAST: The Position-Specific Iterated BLAST (PSI-BLAST) program performs iterative searches with a protein query,   in which sequences found in one round of search are used to build a custom score model for the next round.   more... - Inclusion Threshold: This sets the statistical significance threshold for including a sequence in the model used   by PSI-BLAST to create the PSSM on the next iteration. |

|  |  |  |  |  |  |
| --- | --- | --- | --- | --- | --- |
| Download | | | | | |
| Alignment  Text XML ASN.1 JSON Seq-align Hit Table(text) Hit Table(csv) Multiple-file XML2 Single-file XML2 Multiple-file JSON Single-file JSON SAM | Search Strategies  ASN.1 | PSSM to restart search  PSSM | [?] |  |  |

The Download link provides BLAST output that may be used as input to another program.
This includes parseable formats such as the tabular report or XML as well as the Search Strategy files read by the BLAST+ applications.
More details on the parseable (XML, tabular, and ASN.1) reports can be found at
https://www.ncbi.nlm.nih.gov/books/NBK153387/  
  

The following formats are offered under the Alignment section:  
1). "Text". Non-HTML standard BLAST report.  
2). "XML". XML report based upon the DTD at https://www.ncbi.nlm.nih.gov/data\_specs/dtd/NCBI\_BlastOutput.dtd  
3). "ASN.1". Alignment written out in Abstract Syntax Notation 1.  
4). "JSON Seq-align". Alignment written out in JSON.  
4). "Hit Table(text)". The tabular report as text.  
5). "Hit Table(csv)". The tabular report ready for import into spread-sheet programs like Excel.  
6). "XML2". New XML format described at ftp://ftp.ncbi.nlm.nih.gov/blast/documents/NEWXML/xml2.pdf.  
7). "JSON". New JSON format described at ftp://ftp.ncbi.nlm.nih.gov/blast/documents/NEWXML/xml2.pdf.  
8). "SAM". Sequence Alignment Map format.

XML2 and JSON can be downloaded either as one file per query (multiple-file) or one file for all queries (single-file). These formats are listed as Multiple-file XML2 (and JSON) or Single-file XML (and JSON).

The following report is offered under the Search Strategy section:  
1). "ASN.1" Search Strategy. A record of the parameters, query, and database used in the search. This file can be used to start a stand-alone BLAST search, see
https://www.ncbi.nlm.nih.gov/books/NBK1763/#CmdLineAppsManual.I455\_BLAST\_search\_stra


# Job title: B pale R -- 20..1069 of sequence (1050

Results for:

lcl|Query\_104847 B pale R -- 20..1069 of sequence(1050bp)
[?]

Your BLAST job specified more than one input sequence.
This box lets you choose which input sequence to show BLAST results for.

RID
:   A4XGZT1M014 (Expires on 03-10 18:55 pm)

Query ID
:   lcl|Query\_104847
:   lcl|Query\_104847

Description
:   B pale R -- 20..1069 of sequence

Molecule type
:   nucleic acid

Query Length
:   1050

Database Name
:   nr

Description
:   Nucleotide collection (nt) See details

Program
:   BLASTN 2.8.0+ Citation

  

Reference 

Zheng Zhang, Scott Schwartz, Lukas Wagner, and Webb Miller (2000), "A greedy algorithm for aligning DNA sequences", J Comput Biol 2000; 7(1-2):203-14.

Reference - database indexing

Aleksandr Morgulis, George Coulouris, Yan Raytselis, Thomas L. Madden, Richa Agarwala, Alejandro A. Schäffer (2008), "Database Indexing for Production MegaBLAST Searches", Bioinformatics 24:1757-1764.

Other reports:
Search Summary

[Taxonomy reports]
[Distance tree of results]
[MSA viewer]

Search Parameters

| Search parameter name | Search parameter value |
| --- | --- |
| Program | blastn |
| Word size | 28 |
| Expect value | 10 |
| Hitlist size | 100 |
| Match/Mismatch scores | 1,-2 |
| Gapcosts | 0,2.5 |
| Low Complexity Filter | Yes |
| Filter string | L;m; |
| Genetic Code | 1 |

Database

| Database parameter name | Database parameter value |
| --- | --- |
| Posted date | Mar 7, 2018 1:58 PM |
| Number of letters | 174,044,644,244 |
| Number of sequences | 46,882,714 |
| Entrez query | Includes:  Excludes:  None |

Karlin-Altschul statistics

| Params | Ungapped | Gapped |
| --- | --- | --- |
| Lambda | 1.33271 | 1.28 |
| K | 0.620991 | 0.46 |
| H | 1.12409 | 0.85 |

Results Statistics

| Results Statistics parameter name | Results Statistics parameter value |
| --- | --- |
| Length adjustment | 35 |
| Effective length of query | 1015 |
| Effective length of database | 172403749254 |
| Effective search space | 174989805492810 |
| Effective search space used | 174989805492810 |


## Graphic Summary

### Distribution of the top 116 Blast Hits on 100 subject sequences [?]

The graphic is an overview of the database sequences aligned to the query sequence. These are represented horizontal bars colored coded by score and showing the extent
of the alignment on the query sequence. Separate aligned regions on the same database sequence are connected by a thin grey line.
Mousing over an alignment shows the database sequence title. Clicking an alignment displays a box with more details about the alignment and
link to the sequence alignment itself in the Alignments section of the report.

Mouse over to see the title, click to show alignments

Color key for alignment scores

<40

40-50

50-80

80-200

>=200

Query

1

200

400

600

800

1000

Pseudomonas sp. S-3 16S ribosomal RNA gene, partial seq..

Score:1906 Evalue:0

Accession:KC207086.1

Alignment

Pseudomonas brassicacearum isolate MA250 16S ribosomal ..

Score:1906 Evalue:0

Accession:DQ886486.1

Alignment

Uncultured bacterium clone Untreatedsoil-0day-94 16S ri..

Score:1901 Evalue:0

Accession:MF314815.2

Alignment

Uncultured bacterium clone Untreatedsoil-0day-1 16S rib..

Score:1901 Evalue:0

Accession:MF314725.2

Alignment

Pseudomonas fluorescens strain 2P24 chromosome, complet..

Score:1901 Evalue:0

Accession:CP025542.1

Alignment

Uncultured bacterium clone SPN400-90day-85 16S ribosoma..

Score:1901 Evalue:0

Accession:MF314628.1

Alignment

Uncultured bacterium clone SPN400-90day-76 16S ribosoma..

Score:1901 Evalue:0

Accession:MF314621.1

Alignment

Uncultured bacterium clone SPN400-90day-50 16S ribosoma..

Score:1901 Evalue:0

Accession:MF314595.1

Alignment

Uncultured bacterium clone SPN400-90day-46 16S ribosoma..

Score:1901 Evalue:0

Accession:MF314592.1

Alignment

Uncultured bacterium clone SPN400-90day-43 16S ribosoma..

Score:1901 Evalue:0

Accession:MF314589.1

Alignment

Uncultured bacterium clone SPN400-90day-20 16S ribosoma..

Score:1901 Evalue:0

Accession:MF314569.1

Alignment

Uncultured bacterium clone SPN400-90day-6 16S ribosomal..

Score:1901 Evalue:0

Accession:MF314558.1

Alignment

Pseudomonas sp. HA-09 partial 16S rRNA gene, isolate HA..

Score:1901 Evalue:0

Accession:LT844660.1

Alignment

Pseudomonas brassicacearum strain YC-1 16S ribosomal RN..

Score:1901 Evalue:0

Accession:KY753310.1

Alignment

Pseudomonas brassicacearum strain FC-7 16S ribosomal RN..

Score:1901 Evalue:0

Accession:KY649379.1

Alignment

Pseudomonas fluorescens strain PFR1 16S ribosomal RNA g..

Score:1901 Evalue:0

Accession:MF000304.1

Alignment

Pseudomonas sp. strain 7.3 16S ribosomal RNA gene, part..

Score:1901 Evalue:0

Accession:KY542120.1

Alignment

Pseudomonas syringae strain yangyueP4 16S ribosomal RNA..

Score:1901 Evalue:0

Accession:KU977139.1

Alignment

Pseudomonas fluorescens strain FW300-N2E2 genome

Score:1901 Evalue:0

Accession:CP015225.1

Alignment

Pseudomonas fluorescens strain SBR10 16S ribosomal RNA ..

Score:1901 Evalue:0

Accession:KX018310.1

Alignment

Pseudomonas sp. B055-40 16S ribosomal RNA gene, partial..

Score:1901 Evalue:0

Accession:KJ191010.1

Alignment

Pseudomonas sp. cpRA293 16S ribosomal RNA gene, partial..

Score:1901 Evalue:0

Accession:KJ510220.1

Alignment

Pseudomonas corrugata strain G1 16S ribosomal RNA gene,..

Score:1901 Evalue:0

Accession:KT957303.1

Alignment

Pseudomonas fluorescens strain FW300-N2C3, complete gen..

Score:1901 Evalue:0

Accession:CP012831.1

Alignment

Pseudomonas migulae strain DD290 16S ribosomal RNA gene..

Score:1901 Evalue:0

Accession:KR822273.1

Alignment

Pseudomonas fluorescens 16S ribosomal RNA gene, partial..

Score:1901 Evalue:0

Accession:KT321681.1

Alignment

Pseudomonas brassicacearum strain Kr21 16S ribosomal RN..

Score:1901 Evalue:0

Accession:KT215482.1

Alignment

Pseudomonas brassicacearum strain IHB B 13650 16S ribos..

Score:1901 Evalue:0

Accession:KP762561.1

Alignment

Pseudomonas brassicacearum subsp. neoaurantiaca strain ..

Score:1901 Evalue:0

Accession:KP762555.1

Alignment

Pseudomonas brassicacearum strain 13B-23 16S ribosomal ..

Score:1901 Evalue:0

Accession:KR083019.1

Alignment

Pseudomonas brassicacearum strain 10C-30 16S ribosomal ..

Score:1901 Evalue:0

Accession:KR061416.1

Alignment

Pseudomonas thivervalensis strain MAH1 16S ribosomal RN..

Score:1901 Evalue:0

Accession:KP742980.1

Alignment

Pseudomonas brassicacearum strain 11B23 16S ribosomal R..

Score:1901 Evalue:0

Accession:KR611048.1

Alignment

Pseudomonas brassicacearum strain 3B1 16S ribosomal RNA..

Score:1901 Evalue:0

Accession:KR611047.1

Alignment

Pseudomonas sp. 41(2015) 16S ribosomal RNA gene, partia..

Score:1901 Evalue:0

Accession:KR080568.1

Alignment

Pseudomonas sp. 15(2015) 16S ribosomal RNA gene, partia..

Score:1901 Evalue:0

Accession:KR080563.1

Alignment

Pseudomonas brassicacearum subsp. brassicacearum strain..

Score:1901 Evalue:0

Accession:KP730603.1

Alignment

Pseudomonas sp. SAM1 16S ribosomal RNA gene, partial se..

Score:1901 Evalue:0

Accession:KM269192.1

Alignment

Pseudomonas sp. TY1210 16S ribosomal RNA gene, partial ..

Score:1901 Evalue:0

Accession:KM030057.1

Alignment

Pseudomonas thivervalensis strain PE32 16S ribosomal RN..

Score:1901 Evalue:0

Accession:KJ420530.1

Alignment

Pseudomonas fluorescens strain JK15 16S ribosomal RNA g..

Score:1901 Evalue:0

Accession:KF148637.1

Alignment

Pseudomonas kilonensis strain JX22 16S ribosomal RNA ge..

Score:1901 Evalue:0

Accession:KF840730.1

Alignment

Pseudomonas sp. SDW-16 16S ribosomal RNA gene, partial ..

Score:1901 Evalue:0

Accession:KF835389.1

Alignment

Pseudomonas brassicacearum strain WK-444s 16S ribosomal..

Score:1901 Evalue:0

Accession:KF580861.1

Alignment

Pseudomonas brassicacearum strain IHB B 821 16S ribosom..

Score:1901 Evalue:0

Accession:KF475874.1

Alignment

Pseudomonas sp. SJ7b 16S ribosomal RNA gene, partial se..

Score:1901 Evalue:0

Accession:KF312468.1

Alignment

Pseudomonas putida partial 16S rRNA gene, strain CFBP 4..

Score:1901 Evalue:0

Accession:HF545841.1

Alignment

Pseudomonas sp. X123 16S ribosomal RNA gene, partial se..

Score:1901 Evalue:0

Accession:KC428747.1

Alignment

Pseudomonas fluorescens strain IBFC2012-45 16S ribosoma..

Score:1901 Evalue:0

Accession:KC246049.1

Alignment

Pseudomonas fluorescens strain P-72-10 16S ribosomal RN..

Score:1901 Evalue:0

Accession:HQ888871.1

Alignment

Pseudomonas sp. N-128 16S ribosomal RNA gene, partial s..

Score:1901 Evalue:0

Accession:JN216880.1

Alignment

Uncultured Pseudomonas sp. clone CGMCG 6071 16S ribosom..

Score:1901 Evalue:0

Accession:JX077087.1

Alignment

Pseudomonas fluorescens strain J2 16S ribosomal RNA gen..

Score:1901 Evalue:0

Accession:JN399995.1

Alignment

Pseudomonas sp. R25(2012) 16S ribosomal RNA gene, parti..

Score:1901 Evalue:0

Accession:JX082293.1

Alignment

Endophytic bacterium 41P-2 16S ribosomal RNA gene, part..

Score:1901 Evalue:0

Accession:JF901350.1

Alignment

Bacterium NTL501 16S ribosomal RNA gene, partial sequen..

Score:1901 Evalue:0

Accession:JQ779065.1

Alignment

## Descriptions

, Reading indexes 1-5, displaying indexes 1-5


Load next setPrevious Match

Sequences producing significant alignments:

Show all columns  of the table presenting sequences producing significant alignments 

Select:AllNone
Selected:0

Alignments
Download

FASTA (complete sequence)

FASTA (aligned sequences)

GenBank (complete sequence)

Hit Table (text)

Hit Table (CSV)

Text

XML

ASN.1

Continue
Cancel

GenBank 
Graphics
Distance tree of results
Multiple alignment
Show/hide columns of the table presenting sequences producing significant alignments 

Available columns

Description  
Max Score  
Total Score  
Coverage  
E-value  
IdentN  
Accession  
Restore Defaults
Ok
Cancel

Sequences producing significant alignments:

| Select for downloading or viewing reports | Description | Max score | Total score | Query cover | E value | Ident | Accession |
| --- | --- | --- | --- | --- | --- | --- | --- |
| 1Select seq KC207086.1 | Pseudomonas sp. S-3 16S ribosomal RNA gene, partial sequence | 1906 | 1906 | 98% | 0.0 | 99% | KC207086.1 |
| 2Select seq DQ886486.1 | Pseudomonas brassicacearum isolate MA250 16S ribosomal RNA gene, partial sequence | 1906 | 1906 | 98% | 0.0 | 99% | DQ886486.1 |
| 3Select seq MF314815.2 | Uncultured bacterium clone Untreatedsoil-0day-94 16S ribosomal RNA gene, partial sequence | 1901 | 1901 | 98% | 0.0 | 99% | MF314815.2 |
| 4Select seq MF314725.2 | Uncultured bacterium clone Untreatedsoil-0day-1 16S ribosomal RNA gene, partial sequence | 1901 | 1901 | 98% | 0.0 | 99% | MF314725.2 |
| 5Select seq CP025542.1 | Pseudomonas fluorescens strain 2P24 chromosome, complete genome | 1901 | 9495 | 98% | 0.0 | 99% | CP025542.1 |
| 6Select seq MF314628.1 | Uncultured bacterium clone SPN400-90day-85 16S ribosomal RNA gene, partial sequence | 1901 | 1901 | 98% | 0.0 | 99% | MF314628.1 |
| 7Select seq MF314621.1 | Uncultured bacterium clone SPN400-90day-76 16S ribosomal RNA gene, partial sequence | 1901 | 1901 | 98% | 0.0 | 99% | MF314621.1 |
| 8Select seq MF314595.1 | Uncultured bacterium clone SPN400-90day-50 16S ribosomal RNA gene, partial sequence | 1901 | 1901 | 98% | 0.0 | 99% | MF314595.1 |
| 9Select seq MF314592.1 | Uncultured bacterium clone SPN400-90day-46 16S ribosomal RNA gene, partial sequence | 1901 | 1901 | 98% | 0.0 | 99% | MF314592.1 |
| 10Select seq MF314589.1 | Uncultured bacterium clone SPN400-90day-43 16S ribosomal RNA gene, partial sequence | 1901 | 1901 | 98% | 0.0 | 99% | MF314589.1 |
| 11Select seq MF314569.1 | Uncultured bacterium clone SPN400-90day-20 16S ribosomal RNA gene, partial sequence | 1901 | 1901 | 98% | 0.0 | 99% | MF314569.1 |
| 12Select seq MF314558.1 | Uncultured bacterium clone SPN400-90day-6 16S ribosomal RNA gene, partial sequence | 1901 | 1901 | 98% | 0.0 | 99% | MF314558.1 |
| 13Select seq LT844660.1 | Pseudomonas sp. HA-09 partial 16S rRNA gene, isolate HA-09 | 1901 | 1901 | 98% | 0.0 | 99% | LT844660.1 |
| 14Select seq KY753310.1 | Pseudomonas brassicacearum strain YC-1 16S ribosomal RNA gene, partial sequence | 1901 | 1901 | 98% | 0.0 | 99% | KY753310.1 |
| 15Select seq KY649379.1 | Pseudomonas brassicacearum strain FC-7 16S ribosomal RNA gene, partial sequence | 1901 | 1901 | 98% | 0.0 | 99% | KY649379.1 |
| 16Select seq MF000304.1 | Pseudomonas fluorescens strain PFR1 16S ribosomal RNA gene, partial sequence | 1901 | 1901 | 98% | 0.0 | 99% | MF000304.1 |
| 17Select seq KY542120.1 | Pseudomonas sp. strain 7.3 16S ribosomal RNA gene, partial sequence | 1901 | 1901 | 98% | 0.0 | 99% | KY542120.1 |
| 18Select seq KU977139.1 | Pseudomonas syringae strain yangyueP4 16S ribosomal RNA gene, partial sequence | 1901 | 1901 | 98% | 0.0 | 99% | KU977139.1 |
| 19Select seq CP015225.1 | Pseudomonas fluorescens strain FW300-N2E2 genome | 1901 | 9499 | 98% | 0.0 | 99% | CP015225.1 |
| 20Select seq KX018310.1 | Pseudomonas fluorescens strain SBR10 16S ribosomal RNA gene, partial sequence | 1901 | 1901 | 98% | 0.0 | 99% | KX018310.1 |
| 21Select seq KJ191010.1 | Pseudomonas sp. B055-40 16S ribosomal RNA gene, partial sequence | 1901 | 1901 | 98% | 0.0 | 99% | KJ191010.1 |
| 22Select seq KJ510220.1 | Pseudomonas sp. cpRA293 16S ribosomal RNA gene, partial sequence | 1901 | 1901 | 98% | 0.0 | 99% | KJ510220.1 |
| 23Select seq KT957303.1 | Pseudomonas corrugata strain G1 16S ribosomal RNA gene, partial sequence | 1901 | 1901 | 98% | 0.0 | 99% | KT957303.1 |
| 24Select seq CP012831.1 | Pseudomonas fluorescens strain FW300-N2C3, complete genome | 1901 | 9506 | 98% | 0.0 | 99% | CP012831.1 |
| 25Select seq KR822273.1 | Pseudomonas migulae strain DD290 16S ribosomal RNA gene, partial sequence | 1901 | 1901 | 98% | 0.0 | 99% | KR822273.1 |
| 26Select seq KT321681.1 | Pseudomonas fluorescens 16S ribosomal RNA gene, partial sequence | 1901 | 1901 | 98% | 0.0 | 99% | KT321681.1 |
| 27Select seq KT215482.1 | Pseudomonas brassicacearum strain Kr21 16S ribosomal RNA gene, partial sequence | 1901 | 1901 | 98% | 0.0 | 99% | KT215482.1 |
| 28Select seq KP762561.1 | Pseudomonas brassicacearum strain IHB B 13650 16S ribosomal RNA gene, partial sequence | 1901 | 1901 | 98% | 0.0 | 99% | KP762561.1 |
| 29Select seq KP762555.1 | Pseudomonas brassicacearum subsp. neoaurantiaca strain IHB B 13645 16S ribosomal RNA gene, partial sequence | 1901 | 1901 | 98% | 0.0 | 99% | KP762555.1 |
| 30Select seq KR083019.1 | Pseudomonas brassicacearum strain 13B-23 16S ribosomal RNA gene, partial sequence | 1901 | 1901 | 98% | 0.0 | 99% | KR083019.1 |
| 31Select seq KR061416.1 | Pseudomonas brassicacearum strain 10C-30 16S ribosomal RNA gene, partial sequence | 1901 | 1901 | 98% | 0.0 | 99% | KR061416.1 |
| 32Select seq KP742980.1 | Pseudomonas thivervalensis strain MAH1 16S ribosomal RNA gene, partial sequence | 1901 | 1901 | 98% | 0.0 | 99% | KP742980.1 |
| 33Select seq KR611048.1 | Pseudomonas brassicacearum strain 11B23 16S ribosomal RNA gene, partial sequence | 1901 | 1901 | 98% | 0.0 | 99% | KR611048.1 |
| 34Select seq KR611047.1 | Pseudomonas brassicacearum strain 3B1 16S ribosomal RNA gene, partial sequence | 1901 | 1901 | 98% | 0.0 | 99% | KR611047.1 |
| 35Select seq KR080568.1 | Pseudomonas sp. 41(2015) 16S ribosomal RNA gene, partial sequence | 1901 | 1901 | 98% | 0.0 | 99% | KR080568.1 |
| 36Select seq KR080563.1 | Pseudomonas sp. 15(2015) 16S ribosomal RNA gene, partial sequence | 1901 | 1901 | 98% | 0.0 | 99% | KR080563.1 |
| 37Select seq KP730603.1 | Pseudomonas brassicacearum subsp. brassicacearum strain BW0808 16S ribosomal RNA gene, partial sequence | 1901 | 1901 | 98% | 0.0 | 99% | KP730603.1 |
| 38Select seq KM269192.1 | Pseudomonas sp. SAM1 16S ribosomal RNA gene, partial sequence | 1901 | 1901 | 98% | 0.0 | 99% | KM269192.1 |
| 39Select seq KM030057.1 | Pseudomonas sp. TY1210 16S ribosomal RNA gene, partial sequence | 1901 | 1901 | 98% | 0.0 | 99% | KM030057.1 |
| 40Select seq KJ420530.1 | Pseudomonas thivervalensis strain PE32 16S ribosomal RNA gene, partial sequence | 1901 | 1901 | 98% | 0.0 | 99% | KJ420530.1 |
| 41Select seq KF148637.1 | Pseudomonas fluorescens strain JK15 16S ribosomal RNA gene, partial sequence | 1901 | 1901 | 98% | 0.0 | 99% | KF148637.1 |
| 42Select seq KF840730.1 | Pseudomonas kilonensis strain JX22 16S ribosomal RNA gene, partial sequence | 1901 | 1901 | 98% | 0.0 | 99% | KF840730.1 |
| 43Select seq KF835389.1 | Pseudomonas sp. SDW-16 16S ribosomal RNA gene, partial sequence | 1901 | 1901 | 98% | 0.0 | 99% | KF835389.1 |
| 44Select seq KF580861.1 | Pseudomonas brassicacearum strain WK-444s 16S ribosomal RNA gene, partial sequence | 1901 | 1901 | 98% | 0.0 | 99% | KF580861.1 |
| 45Select seq KF475874.1 | Pseudomonas brassicacearum strain IHB B 821 16S ribosomal RNA gene, partial sequence | 1901 | 1901 | 98% | 0.0 | 99% | KF475874.1 |
| 46Select seq KF312468.1 | Pseudomonas sp. SJ7b 16S ribosomal RNA gene, partial sequence | 1901 | 1901 | 98% | 0.0 | 99% | KF312468.1 |
| 47Select seq HF545841.1 | Pseudomonas putida partial 16S rRNA gene, strain CFBP 4629 | 1901 | 1901 | 98% | 0.0 | 99% | HF545841.1 |
| 48Select seq KC428747.1 | Pseudomonas sp. X123 16S ribosomal RNA gene, partial sequence | 1901 | 1901 | 98% | 0.0 | 99% | KC428747.1 |
| 49Select seq KC246049.1 | Pseudomonas fluorescens strain IBFC2012-45 16S ribosomal RNA gene, partial sequence | 1901 | 1901 | 98% | 0.0 | 99% | KC246049.1 |
| 50Select seq HQ888871.1 | Pseudomonas fluorescens strain P-72-10 16S ribosomal RNA gene, partial sequence | 1901 | 1901 | 98% | 0.0 | 99% | HQ888871.1 |
| 51Select seq JN216880.1 | Pseudomonas sp. N-128 16S ribosomal RNA gene, partial sequence | 1901 | 1901 | 98% | 0.0 | 99% | JN216880.1 |
| 52Select seq JX077087.1 | Uncultured Pseudomonas sp. clone CGMCG 6071 16S ribosomal RNA gene, partial sequence | 1901 | 1901 | 98% | 0.0 | 99% | JX077087.1 |
| 53Select seq JN399995.1 | Pseudomonas fluorescens strain J2 16S ribosomal RNA gene, partial sequence | 1901 | 1901 | 98% | 0.0 | 99% | JN399995.1 |
| 54Select seq JX082293.1 | Pseudomonas sp. R25(2012) 16S ribosomal RNA gene, partial sequence | 1901 | 1901 | 98% | 0.0 | 99% | JX082293.1 |
| 55Select seq JF901350.1 | Endophytic bacterium 41P-2 16S ribosomal RNA gene, partial sequence | 1901 | 1901 | 98% | 0.0 | 99% | JF901350.1 |
| 56Select seq JQ779065.1 | Bacterium NTL501 16S ribosomal RNA gene, partial sequence | 1901 | 1901 | 98% | 0.0 | 99% | JQ779065.1 |
| 57Select seq JQ779056.1 | Bacterium NTL264 16S ribosomal RNA gene, partial sequence | 1901 | 1901 | 98% | 0.0 | 99% | JQ779056.1 |
| 58Select seq JQ779041.1 | Bacterium NTL206 16S ribosomal RNA gene, partial sequence | 1901 | 1901 | 98% | 0.0 | 99% | JQ779041.1 |
| 59Select seq AB621593.1 | Pseudomonas fluorescens gene for 16S rRNA, partial sequence, strain: MPF29 | 1901 | 1901 | 98% | 0.0 | 99% | AB621593.1 |
| 60Select seq JQ237663.1 | Pseudomonas sp. hswx161 16S ribosomal RNA gene, partial sequence | 1901 | 1901 | 98% | 0.0 | 99% | JQ237663.1 |
| 61Select seq JN628032.1 | Pseudomonas thivervalensis 16S ribosomal RNA gene, partial sequence | 1901 | 1901 | 98% | 0.0 | 99% | JN628032.1 |
| 62Select seq JN628030.1 | Pseudomonas brassicacearum subsp. neoaurantiaca 16S ribosomal RNA gene, partial sequence | 1901 | 1901 | 98% | 0.0 | 99% | JN628030.1 |
| 63Select seq JN033549.1 | Pseudomonas brassicacearum strain ME BHU2 16S ribosomal RNA gene, partial sequence | 1901 | 1901 | 98% | 0.0 | 99% | JN033549.1 |
| 64Select seq HQ143608.1 | Pseudomonas sp. strain MTQ15 16S ribosomal RNA gene, partial sequence | 1901 | 1901 | 98% | 0.0 | 99% | HQ143608.1 |
| 65Select seq HM854217.1 | Pseudomonas fluorescens strain STAD384 16S ribosomal RNA gene, partial sequence | 1901 | 1901 | 98% | 0.0 | 99% | HM854217.1 |
| 66Select seq HM579795.1 | Pseudomonas sp. HY4(2010) 16S ribosomal RNA gene, partial sequence | 1901 | 1901 | 98% | 0.0 | 99% | HM579795.1 |
| 67Select seq GU201849.1 | Pseudomonas brassicacearum strain Zy-2-1 16S ribosomal RNA gene, partial sequence | 1901 | 1901 | 98% | 0.0 | 99% | GU201849.1 |
| 68Select seq EF540490.1 | Pseudomonas sp. 4\_C7/16\_5 16S ribosomal RNA gene, partial sequence | 1901 | 1901 | 98% | 0.0 | 99% | EF540490.1 |
| 69Select seq DQ453833.1 | Pseudomonas sp. P97.26 16S ribosomal RNA gene, partial sequence | 1901 | 1901 | 98% | 0.0 | 99% | DQ453833.1 |
| 70Select seq DQ431467.1 | Pseudomonas fluorescens strain XG32 16S ribosomal RNA gene, partial sequence | 1901 | 1901 | 98% | 0.0 | 99% | DQ431467.1 |
| 71Select seq AY447045.1 | Pseudomonas fluorescens strain 2P24 16S ribosomal RNA gene, partial sequence | 1901 | 1901 | 98% | 0.0 | 99% | AY447045.1 |
| 72Select seq AB204716.1 | Pseudomonas fluorescens gene for 16S rRNA, partial sequence, strain:LRB3W1 | 1901 | 1901 | 98% | 0.0 | 99% | AB204716.1 |
| 73Select seq AM229082.1 | Pseudomonas fluorescens 16S rRNA gene, strain C7R12 | 1901 | 1901 | 98% | 0.0 | 99% | AM229082.1 |
| 74Select seq KT318824.1 | Pseudomonas sp. RhizorgN 16S ribosomal RNA gene, partial sequence | 1899 | 1899 | 98% | 0.0 | 99% | KT318824.1 |
| 75Select seq KT318813.1 | Pseudomonas sp. RhizorgB 16S ribosomal RNA gene, partial sequence | 1899 | 1899 | 98% | 0.0 | 99% | KT318813.1 |
| 76Select seq LT629931.1 | Pseudomonas sp. 1A partial 16S rRNA gene, strain 1A | 1897 | 1897 | 98% | 0.0 | 99% | LT629931.1 |
| 77Select seq KR061398.1 | Pseudomonas brassicacearum strain 1Cg-22 16S ribosomal RNA gene, partial sequence | 1897 | 1897 | 98% | 0.0 | 99% | KR061398.1 |
| 78Select seq MF314711.2 | Uncultured bacterium clone SPN2000-90day-84 16S ribosomal RNA gene, partial sequence | 1895 | 1895 | 98% | 0.0 | 99% | MF314711.2 |
| 79Select seq MG461480.1 | Pseudomonas fluorescens strain LBUM770 16S ribosomal RNA gene, partial sequence | 1895 | 1895 | 98% | 0.0 | 99% | MG461480.1 |
| 80Select seq MG461459.1 | Pseudomonas brassicacearum strain LBUM300 16S ribosomal RNA gene, partial sequence | 1895 | 1895 | 98% | 0.0 | 99% | MG461459.1 |
| 81Select seq MG266327.1 | Pseudomonas sp. strain KF-8 16S ribosomal RNA gene, partial sequence | 1895 | 1895 | 98% | 0.0 | 99% | MG266327.1 |
| 82Select seq MF062638.1 | Pseudomonas putida strain 42R-P6 16S ribosomal RNA gene, partial sequence | 1895 | 1895 | 98% | 0.0 | 99% | MF062638.1 |
| 83Select seq MF314613.1 | Uncultured bacterium clone SPN400-90day-68 16S ribosomal RNA gene, partial sequence | 1895 | 1895 | 98% | 0.0 | 99% | MF314613.1 |
| 84Select seq MF314601.1 | Uncultured bacterium clone SPN400-90day-56 16S ribosomal RNA gene, partial sequence | 1895 | 1895 | 98% | 0.0 | 99% | MF314601.1 |
| 85Select seq MF314557.1 | Uncultured bacterium clone SPN400-90day-5 16S ribosomal RNA gene, partial sequence | 1895 | 1895 | 98% | 0.0 | 99% | MF314557.1 |
| 86Select seq MF314553.1 | Uncultured bacterium clone SPN400-90day-1 16S ribosomal RNA gene, partial sequence | 1895 | 1895 | 98% | 0.0 | 99% | MF314553.1 |
| 87Select seq MF314478.1 | Uncultured bacterium clone SPN0-90day-18 16S ribosomal RNA gene, partial sequence | 1895 | 1895 | 98% | 0.0 | 99% | MF314478.1 |
| 88Select seq MF314475.1 | Uncultured bacterium clone SPN0-90day-15 16S ribosomal RNA gene, partial sequence | 1895 | 1895 | 98% | 0.0 | 99% | MF314475.1 |
| 89Select seq MF314468.1 | Uncultured bacterium clone SPN0-90day-7 16S ribosomal RNA gene, partial sequence | 1895 | 1895 | 98% | 0.0 | 99% | MF314468.1 |
| 90Select seq MF624719.1 | Pseudomonas brassicacearum strain Pv10 16S ribosomal RNA gene, partial sequence | 1895 | 1895 | 98% | 0.0 | 99% | MF624719.1 |
| 91Select seq MF498772.1 | Pseudomonas jessenii strain AP3\_16 16S ribosomal RNA gene, partial sequence | 1895 | 1895 | 98% | 0.0 | 99% | MF498772.1 |
| 92Select seq KX859150.1 | Pseudomonas sp. strain A9 16S ribosomal RNA gene, partial sequence | 1895 | 1895 | 98% | 0.0 | 99% | KX859150.1 |
| 93Select seq KY347907.1 | Pseudomonas sp. strain YP2 16S ribosomal RNA gene, partial sequence | 1895 | 1895 | 98% | 0.0 | 99% | KY347907.1 |
| 94Select seq MF445218.1 | Pseudomonas sp. strain nenu\_DS-R11 16S ribosomal RNA gene, partial sequence | 1895 | 1895 | 98% | 0.0 | 99% | MF445218.1 |
| 95Select seq MF445217.1 | Pseudomonas sp. strain nenu\_DS-R08 16S ribosomal RNA gene, partial sequence | 1895 | 1895 | 98% | 0.0 | 99% | MF445217.1 |
| 96Select seq KY122024.1 | Pseudomonas sp. strain SX2 16S ribosomal RNA gene, partial sequence | 1895 | 1895 | 98% | 0.0 | 99% | KY122024.1 |
| 97Select seq KY962733.1 | Pseudomonas sp. strain Mong-1 16S ribosomal RNA gene, partial sequence | 1895 | 1895 | 98% | 0.0 | 99% | KY962733.1 |
| 98Select seq KY907019.1 | Pseudomonas sp. strain MSM-10-1 16S ribosomal RNA gene, partial sequence | 1895 | 1895 | 98% | 0.0 | 99% | KY907019.1 |
| 99Select seq KY612271.1 | Pseudomonas brassicacearum strain SKUAST-K21 16S ribosomal RNA gene, partial sequence | 1895 | 1895 | 98% | 0.0 | 99% | KY612271.1 |
| 100Select seq LT707062.1 | Pseudomonas sp. A214 genome assembly, chromosome: I | 1895 | 9462 | 98% | 0.0 | 99% | LT707062.1 |


## Alignments

Loading alignment... for sequences gi|442571814,gi|115315703,gi|1321449613,gi|1321449529,gi|1321363606 Reading indexes 1-5

Download

FASTA (complete sequence)

FASTA (aligned sequences)

GenBank (complete sequence)

Continue
Cancel

GenBankGraphics

Next
Previous
Descriptions

Pseudomonas sp. S-3 16S ribosomal RNA gene, partial sequence

Sequence ID: KC207086.1Length: 1444Number of Matches: 1

Related Information

Range 1: 383 to 1418GenBankGraphics

Next Match
Previous Match
First Match

Alignment statistics for match #1

| Score | Expect | Identities | Gaps | Strand | Frame |
| --- | --- | --- | --- | --- | --- |
| 1906 bits(1032) | 0.0() | 1034/1036(99%) | 0/1036(0%) | Plus/Minus |  |

Features:

```
Query  8     CCCGAAGGTTAGACTAGCTACTTCTGGTGCAACCCACTCCCATGGTGTGACGGGCGGTGT  67
             ||||||||||||||||||||||||||||||||||||||||||||||||||||||||||||
Sbjct  1418  CCCGAAGGTTAGACTAGCTACTTCTGGTGCAACCCACTCCCATGGTGTGACGGGCGGTGT  1359

Query  68    GTACAAGGCCCGGGAACGTATTCACCGCGACATTCTGATTCGCGATTACTAGCGATTCCG  127
             ||||||||||||||||||||||||||||||||||||||||||||||||||||||||||||
Sbjct  1358  GTACAAGGCCCGGGAACGTATTCACCGCGACATTCTGATTCGCGATTACTAGCGATTCCG  1299

Query  128   ACTTCACGCAGTCGAGTTGCAGACTGCGATCCGGACTACGATCGGTTTTGTGGGATTAGC  187
             ||||||||||||||||||||||||||||||||||||||||||||||||||||||||||||
Sbjct  1298  ACTTCACGCAGTCGAGTTGCAGACTGCGATCCGGACTACGATCGGTTTTGTGGGATTAGC  1239

Query  188   TCCACCTCGCGGCTTGGCAACCCTCTGTACCGACCATTGTAGCACGTGTGTAGCCCAGGC  247
             ||||||||||||||||||||||||||||||||||||||||||||||||||||||||||||
Sbjct  1238  TCCACCTCGCGGCTTGGCAACCCTCTGTACCGACCATTGTAGCACGTGTGTAGCCCAGGC  1179

Query  248   CGTAAGGGCCATGATGACTTGACGTCATCCCCACCTTCCTCCGGTTTGTCACCGGCAGTC  307
             ||||||||||||||||||||||||||||||||||||||||||||||||||||||||||||
Sbjct  1178  CGTAAGGGCCATGATGACTTGACGTCATCCCCACCTTCCTCCGGTTTGTCACCGGCAGTC  1119

Query  308   TCCTTAGAGTGCCCACCATGACGTGCTGGTAACTAAGGACAAGGGTTGCGCTCGTTACGG  367
             ||||||||||||||||||||||||||||||||||||||||||||||||||||||||||||
Sbjct  1118  TCCTTAGAGTGCCCACCATGACGTGCTGGTAACTAAGGACAAGGGTTGCGCTCGTTACGG  1059

Query  368   GACTTAACCCAACATCTCACGACACGAGCTGACGACAGCCATGCAGCACCTGTCTCAATG  427
             ||||||||||||||||||||||||||||||||||||||||||||||||||||||||||||
Sbjct  1058  GACTTAACCCAACATCTCACGACACGAGCTGACGACAGCCATGCAGCACCTGTCTCAATG  999

Query  428   NNCCCGAAGGCACCAATCCATCTCTGGAAAGTTCATTGGATGTCAAGGCCTGGTAAGGTT  487
               ||||||||||||||||||||||||||||||||||||||||||||||||||||||||||
Sbjct  998   CTCCCGAAGGCACCAATCCATCTCTGGAAAGTTCATTGGATGTCAAGGCCTGGTAAGGTT  939

Query  488   CTTCGCGTTGCTTCGAATTAAACCACATGCTCCACCGCTTGTGCGGGCCCCCGTCAATTC  547
             ||||||||||||||||||||||||||||||||||||||||||||||||||||||||||||
Sbjct  938   CTTCGCGTTGCTTCGAATTAAACCACATGCTCCACCGCTTGTGCGGGCCCCCGTCAATTC  879

Query  548   ATTTGAGTTTTAACCTTGCGGCCGTACTCCCCAGGCGGTCAACTTAATGCGTTAGCTGCG  607
             ||||||||||||||||||||||||||||||||||||||||||||||||||||||||||||
Sbjct  878   ATTTGAGTTTTAACCTTGCGGCCGTACTCCCCAGGCGGTCAACTTAATGCGTTAGCTGCG  819

Query  608   CCACTAAGAGCTCAAGGCTCCCAACGGCTAGTTGACATCGTTTACGGCGTGGACTACCAG  667
             ||||||||||||||||||||||||||||||||||||||||||||||||||||||||||||
Sbjct  818   CCACTAAGAGCTCAAGGCTCCCAACGGCTAGTTGACATCGTTTACGGCGTGGACTACCAG  759

Query  668   GGTATCTAATCCTGTTTGCTCCCCACGCTTTCGCACCTCAGTGTCAGTATCAGTCCAGGT  727
             ||||||||||||||||||||||||||||||||||||||||||||||||||||||||||||
Sbjct  758   GGTATCTAATCCTGTTTGCTCCCCACGCTTTCGCACCTCAGTGTCAGTATCAGTCCAGGT  699

Query  728   GGTCGCCTTCGCCACTGGTGTTCCTTCCTATATCTACGCATTTCACCGCTACACAGGAAA  787
             ||||||||||||||||||||||||||||||||||||||||||||||||||||||||||||
Sbjct  698   GGTCGCCTTCGCCACTGGTGTTCCTTCCTATATCTACGCATTTCACCGCTACACAGGAAA  639

Query  788   TTCCACCACCCTCTACCATACTCTAGCTCGACAGTTTTGAATGCAGTTCCCAGGTTGAGC  847
             ||||||||||||||||||||||||||||||||||||||||||||||||||||||||||||
Sbjct  638   TTCCACCACCCTCTACCATACTCTAGCTCGACAGTTTTGAATGCAGTTCCCAGGTTGAGC  579

Query  848   CCGGGGCTTTCACATCCAACTTAACGAACCACCTACGCGCGCTTTACGCCCAGTAATTCC  907
             ||||||||||||||||||||||||||||||||||||||||||||||||||||||||||||
Sbjct  578   CCGGGGCTTTCACATCCAACTTAACGAACCACCTACGCGCGCTTTACGCCCAGTAATTCC  519

Query  908   GATTAACGCTTGCACCCTCTGTATTACCGCGGCTGCTGGCACAGAGTTAGCCGGTGCTTA  967
             ||||||||||||||||||||||||||||||||||||||||||||||||||||||||||||
Sbjct  518   GATTAACGCTTGCACCCTCTGTATTACCGCGGCTGCTGGCACAGAGTTAGCCGGTGCTTA  459

Query  968   TTCTGTCGGTAACGTCAAAACACTAACGTATTAGGTTAATGCCCTTCCTCCCAACTTAAA  1027
             ||||||||||||||||||||||||||||||||||||||||||||||||||||||||||||
Sbjct  458   TTCTGTCGGTAACGTCAAAACACTAACGTATTAGGTTAATGCCCTTCCTCCCAACTTAAA  399

Query  1028  GTGCTTTACAATCCGA  1043
             ||||||||||||||||
Sbjct  398   GTGCTTTACAATCCGA  383
```

Download

FASTA (complete sequence)

FASTA (aligned sequences)

GenBank (complete sequence)

Continue
Cancel

GenBankGraphics

Next
Previous
Descriptions

Pseudomonas brassicacearum isolate MA250 16S ribosomal RNA gene, partial sequence

Sequence ID: DQ886486.1Length: 1515Number of Matches: 1

Related Information

Range 1: 402 to 1437GenBankGraphics

Next Match
Previous Match
First Match

Alignment statistics for match #1

| Score | Expect | Identities | Gaps | Strand | Frame |
| --- | --- | --- | --- | --- | --- |
| 1906 bits(1032) | 0.0() | 1034/1036(99%) | 0/1036(0%) | Plus/Minus |  |

Features:

```
Query  8     CCCGAAGGTTAGACTAGCTACTTCTGGTGCAACCCACTCCCATGGTGTGACGGGCGGTGT  67
             ||||||||||||||||||||||||||||||||||||||||||||||||||||||||||||
Sbjct  1437  CCCGAAGGTTAGACTAGCTACTTCTGGTGCAACCCACTCCCATGGTGTGACGGGCGGTGT  1378

Query  68    GTACAAGGCCCGGGAACGTATTCACCGCGACATTCTGATTCGCGATTACTAGCGATTCCG  127
             ||||||||||||||||||||||||||||||||||||||||||||||||||||||||||||
Sbjct  1377  GTACAAGGCCCGGGAACGTATTCACCGCGACATTCTGATTCGCGATTACTAGCGATTCCG  1318

Query  128   ACTTCACGCAGTCGAGTTGCAGACTGCGATCCGGACTACGATCGGTTTTGTGGGATTAGC  187
             ||||||||||||||||||||||||||||||||||||||||||||||||||||||||||||
Sbjct  1317  ACTTCACGCAGTCGAGTTGCAGACTGCGATCCGGACTACGATCGGTTTTGTGGGATTAGC  1258

Query  188   TCCACCTCGCGGCTTGGCAACCCTCTGTACCGACCATTGTAGCACGTGTGTAGCCCAGGC  247
             ||||||||||||||||||||||||||||||||||||||||||||||||||||||||||||
Sbjct  1257  TCCACCTCGCGGCTTGGCAACCCTCTGTACCGACCATTGTAGCACGTGTGTAGCCCAGGC  1198

Query  248   CGTAAGGGCCATGATGACTTGACGTCATCCCCACCTTCCTCCGGTTTGTCACCGGCAGTC  307
             ||||||||||||||||||||||||||||||||||||||||||||||||||||||||||||
Sbjct  1197  CGTAAGGGCCATGATGACTTGACGTCATCCCCACCTTCCTCCGGTTTGTCACCGGCAGTC  1138

Query  308   TCCTTAGAGTGCCCACCATGACGTGCTGGTAACTAAGGACAAGGGTTGCGCTCGTTACGG  367
             ||||||||||||||||||||||||||||||||||||||||||||||||||||||||||||
Sbjct  1137  TCCTTAGAGTGCCCACCATGACGTGCTGGTAACTAAGGACAAGGGTTGCGCTCGTTACGG  1078

Query  368   GACTTAACCCAACATCTCACGACACGAGCTGACGACAGCCATGCAGCACCTGTCTCAATG  427
             ||||||||||||||||||||||||||||||||||||||||||||||||||||||||||||
Sbjct  1077  GACTTAACCCAACATCTCACGACACGAGCTGACGACAGCCATGCAGCACCTGTCTCAATG  1018

Query  428   NNCCCGAAGGCACCAATCCATCTCTGGAAAGTTCATTGGATGTCAAGGCCTGGTAAGGTT  487
               ||||||||||||||||||||||||||||||||||||||||||||||||||||||||||
Sbjct  1017  TTCCCGAAGGCACCAATCCATCTCTGGAAAGTTCATTGGATGTCAAGGCCTGGTAAGGTT  958

Query  488   CTTCGCGTTGCTTCGAATTAAACCACATGCTCCACCGCTTGTGCGGGCCCCCGTCAATTC  547
             ||||||||||||||||||||||||||||||||||||||||||||||||||||||||||||
Sbjct  957   CTTCGCGTTGCTTCGAATTAAACCACATGCTCCACCGCTTGTGCGGGCCCCCGTCAATTC  898

Query  548   ATTTGAGTTTTAACCTTGCGGCCGTACTCCCCAGGCGGTCAACTTAATGCGTTAGCTGCG  607
             ||||||||||||||||||||||||||||||||||||||||||||||||||||||||||||
Sbjct  897   ATTTGAGTTTTAACCTTGCGGCCGTACTCCCCAGGCGGTCAACTTAATGCGTTAGCTGCG  838

Query  608   CCACTAAGAGCTCAAGGCTCCCAACGGCTAGTTGACATCGTTTACGGCGTGGACTACCAG  667
             ||||||||||||||||||||||||||||||||||||||||||||||||||||||||||||
Sbjct  837   CCACTAAGAGCTCAAGGCTCCCAACGGCTAGTTGACATCGTTTACGGCGTGGACTACCAG  778

Query  668   GGTATCTAATCCTGTTTGCTCCCCACGCTTTCGCACCTCAGTGTCAGTATCAGTCCAGGT  727
             ||||||||||||||||||||||||||||||||||||||||||||||||||||||||||||
Sbjct  777   GGTATCTAATCCTGTTTGCTCCCCACGCTTTCGCACCTCAGTGTCAGTATCAGTCCAGGT  718

Query  728   GGTCGCCTTCGCCACTGGTGTTCCTTCCTATATCTACGCATTTCACCGCTACACAGGAAA  787
             ||||||||||||||||||||||||||||||||||||||||||||||||||||||||||||
Sbjct  717   GGTCGCCTTCGCCACTGGTGTTCCTTCCTATATCTACGCATTTCACCGCTACACAGGAAA  658

Query  788   TTCCACCACCCTCTACCATACTCTAGCTCGACAGTTTTGAATGCAGTTCCCAGGTTGAGC  847
             ||||||||||||||||||||||||||||||||||||||||||||||||||||||||||||
Sbjct  657   TTCCACCACCCTCTACCATACTCTAGCTCGACAGTTTTGAATGCAGTTCCCAGGTTGAGC  598

Query  848   CCGGGGCTTTCACATCCAACTTAACGAACCACCTACGCGCGCTTTACGCCCAGTAATTCC  907
             ||||||||||||||||||||||||||||||||||||||||||||||||||||||||||||
Sbjct  597   CCGGGGCTTTCACATCCAACTTAACGAACCACCTACGCGCGCTTTACGCCCAGTAATTCC  538

Query  908   GATTAACGCTTGCACCCTCTGTATTACCGCGGCTGCTGGCACAGAGTTAGCCGGTGCTTA  967
             ||||||||||||||||||||||||||||||||||||||||||||||||||||||||||||
Sbjct  537   GATTAACGCTTGCACCCTCTGTATTACCGCGGCTGCTGGCACAGAGTTAGCCGGTGCTTA  478

Query  968   TTCTGTCGGTAACGTCAAAACACTAACGTATTAGGTTAATGCCCTTCCTCCCAACTTAAA  1027
             ||||||||||||||||||||||||||||||||||||||||||||||||||||||||||||
Sbjct  477   TTCTGTCGGTAACGTCAAAACACTAACGTATTAGGTTAATGCCCTTCCTCCCAACTTAAA  418

Query  1028  GTGCTTTACAATCCGA  1043
             ||||||||||||||||
Sbjct  417   GTGCTTTACAATCCGA  402
```

Download

FASTA (complete sequence)

FASTA (aligned sequences)

GenBank (complete sequence)

Continue
Cancel

GenBankGraphics

Next
Previous
Descriptions

Uncultured bacterium clone Untreatedsoil-0day-94 16S ribosomal RNA gene, partial sequence

Sequence ID: MF314815.2Length: 1515Number of Matches: 1

Related Information

Range 1: 409 to 1444GenBankGraphics

Next Match
Previous Match
First Match

Alignment statistics for match #1

| Score | Expect | Identities | Gaps | Strand | Frame |
| --- | --- | --- | --- | --- | --- |
| 1901 bits(1029) | 0.0() | 1033/1036(99%) | 0/1036(0%) | Plus/Minus |  |

Features:

```
Query  8     CCCGAAGGTTAGACTAGCTACTTCTGGTGCAACCCACTCCCATGGTGTGACGGGCGGTGT  67
             ||||||||||||||||||||||||||||||||||||||||||||||||||||||||||||
Sbjct  1444  CCCGAAGGTTAGACTAGCTACTTCTGGTGCAACCCACTCCCATGGTGTGACGGGCGGTGT  1385

Query  68    GTACAAGGCCCGGGAACGTATTCACCGCGACATTCTGATTCGCGATTACTAGCGATTCCG  127
             ||||||||||||||||||||||||||||||||||||||||||||||||||||||||||||
Sbjct  1384  GTACAAGGCCCGGGAACGTATTCACCGCGACATTCTGATTCGCGATTACTAGCGATTCCG  1325

Query  128   ACTTCACGCAGTCGAGTTGCAGACTGCGATCCGGACTACGATCGGTTTTGTGGGATTAGC  187
             ||||||||||||||||||||||||||||||||||||||||||||||||||||||||||||
Sbjct  1324  ACTTCACGCAGTCGAGTTGCAGACTGCGATCCGGACTACGATCGGTTTTGTGGGATTAGC  1265

Query  188   TCCACCTCGCGGCTTGGCAACCCTCTGTACCGACCATTGTAGCACGTGTGTAGCCCAGGC  247
             ||||||||||||||||||||||||||||||||||||||||||||||||||||||||||||
Sbjct  1264  TCCACCTCGCGGCTTGGCAACCCTCTGTACCGACCATTGTAGCACGTGTGTAGCCCAGGC  1205

Query  248   CGTAAGGGCCATGATGACTTGACGTCATCCCCACCTTCCTCCGGTTTGTCACCGGCAGTC  307
             ||||||||||||||||||||||||||||||||||||||||||||||||||||||||||||
Sbjct  1204  CGTAAGGGCCATGATGACTTGACGTCATCCCCACCTTCCTCCGGTTTGTCACCGGCAGTC  1145

Query  308   TCCTTAGAGTGCCCACCATGACGTGCTGGTAACTAAGGACAAGGGTTGCGCTCGTTACGG  367
             ||||||||||||||||||||||||||||||||||||||||||||||||||||||||||||
Sbjct  1144  TCCTTAGAGTGCCCACCATGACGTGCTGGTAACTAAGGACAAGGGTTGCGCTCGTTACGG  1085

Query  368   GACTTAACCCAACATCTCACGACACGAGCTGACGACAGCCATGCAGCACCTGTCTCAATG  427
             ||||||||||||||||||||||||||||||||||||||||||||||||||||||||||||
Sbjct  1084  GACTTAACCCAACATCTCACGACACGAGCTGACGACAGCCATGCAGCACCTGTCTCAATG  1025

Query  428   NNCCCGAAGGCACCAATCCATCTCTGGAAAGTTCATTGGATGTCAAGGCCTGGTAAGGTT  487
               ||||||||||||||||||||||||||||||||||||||||||||||||||||||||||
Sbjct  1024  TTCCCGAAGGCACCAATCCATCTCTGGAAAGTTCATTGGATGTCAAGGCCTGGTAAGGTT  965

Query  488   CTTCGCGTTGCTTCGAATTAAACCACATGCTCCACCGCTTGTGCGGGCCCCCGTCAATTC  547
             ||||||||||||||||||||||||||||||||||||||||||||||||||||||||||||
Sbjct  964   CTTCGCGTTGCTTCGAATTAAACCACATGCTCCACCGCTTGTGCGGGCCCCCGTCAATTC  905

Query  548   ATTTGAGTTTTAACCTTGCGGCCGTACTCCCCAGGCGGTCAACTTAATGCGTTAGCTGCG  607
             ||||||||||||||||||||||||||||||||||||||||||||||||||||||||||||
Sbjct  904   ATTTGAGTTTTAACCTTGCGGCCGTACTCCCCAGGCGGTCAACTTAATGCGTTAGCTGCG  845

Query  608   CCACTAAGAGCTCAAGGCTCCCAACGGCTAGTTGACATCGTTTACGGCGTGGACTACCAG  667
             ||||||||||||||||||||||||||||||||||||||||||||||||||||||||||||
Sbjct  844   CCACTAAGAGCTCAAGGCTCCCAACGGCTAGTTGACATCGTTTACGGCGTGGACTACCAG  785

Query  668   GGTATCTAATCCTGTTTGCTCCCCACGCTTTCGCACCTCAGTGTCAGTATCAGTCCAGGT  727
             ||||||||||||||||||||||||||||||||||||||||||||||||||||||||||||
Sbjct  784   GGTATCTAATCCTGTTTGCTCCCCACGCTTTCGCACCTCAGTGTCAGTATCAGTCCAGGT  725

Query  728   GGTCGCCTTCGCCACTGGTGTTCCTTCCTATATCTACGCATTTCACCGCTACACAGGAAA  787
             ||||||||||||||||||||||||||||||||||||||||||||||||||||||||||||
Sbjct  724   GGTCGCCTTCGCCACTGGTGTTCCTTCCTATATCTACGCATTTCACCGCTACACAGGAAA  665

Query  788   TTCCACCACCCTCTACCATACTCTAGCTCGACAGTTTTGAATGCAGTTCCCAGGTTGAGC  847
             ||||||||||||||||||||||||||||||||||||||||||||||||||||||||||||
Sbjct  664   TTCCACCACCCTCTACCATACTCTAGCTCGACAGTTTTGAATGCAGTTCCCAGGTTGAGC  605

Query  848   CCGGGGCTTTCACATCCAACTTAACGAACCACCTACGCGCGCTTTACGCCCAGTAATTCC  907
             |||||| |||||||||||||||||||||||||||||||||||||||||||||||||||||
Sbjct  604   CCGGGGATTTCACATCCAACTTAACGAACCACCTACGCGCGCTTTACGCCCAGTAATTCC  545

Query  908   GATTAACGCTTGCACCCTCTGTATTACCGCGGCTGCTGGCACAGAGTTAGCCGGTGCTTA  967
             ||||||||||||||||||||||||||||||||||||||||||||||||||||||||||||
Sbjct  544   GATTAACGCTTGCACCCTCTGTATTACCGCGGCTGCTGGCACAGAGTTAGCCGGTGCTTA  485

Query  968   TTCTGTCGGTAACGTCAAAACACTAACGTATTAGGTTAATGCCCTTCCTCCCAACTTAAA  1027
             ||||||||||||||||||||||||||||||||||||||||||||||||||||||||||||
Sbjct  484   TTCTGTCGGTAACGTCAAAACACTAACGTATTAGGTTAATGCCCTTCCTCCCAACTTAAA  425

Query  1028  GTGCTTTACAATCCGA  1043
             ||||||||||||||||
Sbjct  424   GTGCTTTACAATCCGA  409
```

Download

FASTA (complete sequence)

FASTA (aligned sequences)

GenBank (complete sequence)

Continue
Cancel

GenBankGraphics

Next
Previous
Descriptions

Uncultured bacterium clone Untreatedsoil-0day-1 16S ribosomal RNA gene, partial sequence

Sequence ID: MF314725.2Length: 1502Number of Matches: 1

Related Information

Range 1: 409 to 1444GenBankGraphics

Next Match
Previous Match
First Match

Alignment statistics for match #1

| Score | Expect | Identities | Gaps | Strand | Frame |
| --- | --- | --- | --- | --- | --- |
| 1901 bits(1029) | 0.0() | 1033/1036(99%) | 0/1036(0%) | Plus/Minus |  |

Features:

```
Query  8     CCCGAAGGTTAGACTAGCTACTTCTGGTGCAACCCACTCCCATGGTGTGACGGGCGGTGT  67
             ||||||||||||||||||||||||||||||||||||||||||||||||||||||||||||
Sbjct  1444  CCCGAAGGTTAGACTAGCTACTTCTGGTGCAACCCACTCCCATGGTGTGACGGGCGGTGT  1385

Query  68    GTACAAGGCCCGGGAACGTATTCACCGCGACATTCTGATTCGCGATTACTAGCGATTCCG  127
             ||||||||||||||||||||||||||||||||||||||||||||||||||||||||||||
Sbjct  1384  GTACAAGGCCCGGGAACGTATTCACCGCGACATTCTGATTCGCGATTACTAGCGATTCCG  1325

Query  128   ACTTCACGCAGTCGAGTTGCAGACTGCGATCCGGACTACGATCGGTTTTGTGGGATTAGC  187
             ||||||||||||||||||||||||||||||||||||||||||||||||||||||||||||
Sbjct  1324  ACTTCACGCAGTCGAGTTGCAGACTGCGATCCGGACTACGATCGGTTTTGTGGGATTAGC  1265

Query  188   TCCACCTCGCGGCTTGGCAACCCTCTGTACCGACCATTGTAGCACGTGTGTAGCCCAGGC  247
             ||||||||||||||||||||||||||||||||||||||||||||||||||||||||||||
Sbjct  1264  TCCACCTCGCGGCTTGGCAACCCTCTGTACCGACCATTGTAGCACGTGTGTAGCCCAGGC  1205

Query  248   CGTAAGGGCCATGATGACTTGACGTCATCCCCACCTTCCTCCGGTTTGTCACCGGCAGTC  307
             ||||||||||||||||||||||||||||||||||||||||||||||||||||||||||||
Sbjct  1204  CGTAAGGGCCATGATGACTTGACGTCATCCCCACCTTCCTCCGGTTTGTCACCGGCAGTC  1145

Query  308   TCCTTAGAGTGCCCACCATGACGTGCTGGTAACTAAGGACAAGGGTTGCGCTCGTTACGG  367
             ||||||||||||||||||||||||||||||||||||||||||||||||||||||||||||
Sbjct  1144  TCCTTAGAGTGCCCACCATGACGTGCTGGTAACTAAGGACAAGGGTTGCGCTCGTTACGG  1085

Query  368   GACTTAACCCAACATCTCACGACACGAGCTGACGACAGCCATGCAGCACCTGTCTCAATG  427
             ||||||||||||||||||||||||||||||||||||||||||||||||||||||||||||
Sbjct  1084  GACTTAACCCAACATCTCACGACACGAGCTGACGACAGCCATGCAGCACCTGTCTCAATG  1025

Query  428   NNCCCGAAGGCACCAATCCATCTCTGGAAAGTTCATTGGATGTCAAGGCCTGGTAAGGTT  487
               ||||||||||||||||||||||||||||||||||||||||||||||||||||||||||
Sbjct  1024  TTCCCGAAGGCACCAATCCATCTCTGGAAAGTTCATTGGATGTCAAGGCCTGGTAAGGTT  965

Query  488   CTTCGCGTTGCTTCGAATTAAACCACATGCTCCACCGCTTGTGCGGGCCCCCGTCAATTC  547
             ||||||||||||||||||||||||||||||||||||||||||||||||||||||||||||
Sbjct  964   CTTCGCGTTGCTTCGAATTAAACCACATGCTCCACCGCTTGTGCGGGCCCCCGTCAATTC  905

Query  548   ATTTGAGTTTTAACCTTGCGGCCGTACTCCCCAGGCGGTCAACTTAATGCGTTAGCTGCG  607
             ||||||||||||||||||||||||||||||||||||||||||||||||||||||||||||
Sbjct  904   ATTTGAGTTTTAACCTTGCGGCCGTACTCCCCAGGCGGTCAACTTAATGCGTTAGCTGCG  845

Query  608   CCACTAAGAGCTCAAGGCTCCCAACGGCTAGTTGACATCGTTTACGGCGTGGACTACCAG  667
             ||||||||||||||||||||||||||||||||||||||||||||||||||||||||||||
Sbjct  844   CCACTAAGAGCTCAAGGCTCCCAACGGCTAGTTGACATCGTTTACGGCGTGGACTACCAG  785

Query  668   GGTATCTAATCCTGTTTGCTCCCCACGCTTTCGCACCTCAGTGTCAGTATCAGTCCAGGT  727
             ||||||||||||||||||||||||||||||||||||||||||||||||||||||||||||
Sbjct  784   GGTATCTAATCCTGTTTGCTCCCCACGCTTTCGCACCTCAGTGTCAGTATCAGTCCAGGT  725

Query  728   GGTCGCCTTCGCCACTGGTGTTCCTTCCTATATCTACGCATTTCACCGCTACACAGGAAA  787
             ||||||||||||||||||||||||||||||||||||||||||||||||||||||||||||
Sbjct  724   GGTCGCCTTCGCCACTGGTGTTCCTTCCTATATCTACGCATTTCACCGCTACACAGGAAA  665

Query  788   TTCCACCACCCTCTACCATACTCTAGCTCGACAGTTTTGAATGCAGTTCCCAGGTTGAGC  847
             ||||||||||||||||||||||||||||||||||||||||||||||||||||||||||||
Sbjct  664   TTCCACCACCCTCTACCATACTCTAGCTCGACAGTTTTGAATGCAGTTCCCAGGTTGAGC  605

Query  848   CCGGGGCTTTCACATCCAACTTAACGAACCACCTACGCGCGCTTTACGCCCAGTAATTCC  907
             |||||| |||||||||||||||||||||||||||||||||||||||||||||||||||||
Sbjct  604   CCGGGGATTTCACATCCAACTTAACGAACCACCTACGCGCGCTTTACGCCCAGTAATTCC  545

Query  908   GATTAACGCTTGCACCCTCTGTATTACCGCGGCTGCTGGCACAGAGTTAGCCGGTGCTTA  967
             ||||||||||||||||||||||||||||||||||||||||||||||||||||||||||||
Sbjct  544   GATTAACGCTTGCACCCTCTGTATTACCGCGGCTGCTGGCACAGAGTTAGCCGGTGCTTA  485

Query  968   TTCTGTCGGTAACGTCAAAACACTAACGTATTAGGTTAATGCCCTTCCTCCCAACTTAAA  1027
             ||||||||||||||||||||||||||||||||||||||||||||||||||||||||||||
Sbjct  484   TTCTGTCGGTAACGTCAAAACACTAACGTATTAGGTTAATGCCCTTCCTCCCAACTTAAA  425

Query  1028  GTGCTTTACAATCCGA  1043
             ||||||||||||||||
Sbjct  424   GTGCTTTACAATCCGA  409
```

Download

FASTA (complete sequence)

FASTA (aligned sequences)

GenBank (complete sequence)

Continue
Cancel

GenBankGraphics

Sort by:

E value
 Score
Percent identity
Query start position
Subject start position

Next
Previous
Descriptions

Pseudomonas fluorescens strain 2P24 chromosome, complete genome

Sequence ID: CP025542.1Length: 6610571Number of Matches: 5

Related Information

Range 1: 891436 to 892471GenBankGraphics

Next Match
Previous Match
First Match

Alignment statistics for match #1

| Score | Expect | Identities | Gaps | Strand | Frame |
| --- | --- | --- | --- | --- | --- |
| 1901 bits(1029) | 0.0() | 1033/1036(99%) | 0/1036(0%) | Plus/Minus |  |

Features:

rRNA-16S ribosomal RNA

```
Query  8       CCCGAAGGTTAGACTAGCTACTTCTGGTGCAACCCACTCCCATGGTGTGACGGGCGGTGT  67
               ||||||||||||||||||||||||||||||||||||||||||||||||||||||||||||
Sbjct  892471  CCCGAAGGTTAGACTAGCTACTTCTGGTGCAACCCACTCCCATGGTGTGACGGGCGGTGT  892412

Query  68      GTACAAGGCCCGGGAACGTATTCACCGCGACATTCTGATTCGCGATTACTAGCGATTCCG  127
               ||||||||||||||||||||||||||||||||||||||||||||||||||||||||||||
Sbjct  892411  GTACAAGGCCCGGGAACGTATTCACCGCGACATTCTGATTCGCGATTACTAGCGATTCCG  892352

Query  128     ACTTCACGCAGTCGAGTTGCAGACTGCGATCCGGACTACGATCGGTTTTGTGGGATTAGC  187
               ||||||||||||||||||||||||||||||||||||||||||||||||||||||||||||
Sbjct  892351  ACTTCACGCAGTCGAGTTGCAGACTGCGATCCGGACTACGATCGGTTTTGTGGGATTAGC  892292

Query  188     TCCACCTCGCGGCTTGGCAACCCTCTGTACCGACCATTGTAGCACGTGTGTAGCCCAGGC  247
               ||||||||||||||||||||||||||||||||||||||||||||||||||||||||||||
Sbjct  892291  TCCACCTCGCGGCTTGGCAACCCTCTGTACCGACCATTGTAGCACGTGTGTAGCCCAGGC  892232

Query  248     CGTAAGGGCCATGATGACTTGACGTCATCCCCACCTTCCTCCGGTTTGTCACCGGCAGTC  307
               ||||||||||||||||||||||||||||||||||||||||||||||||||||||||||||
Sbjct  892231  CGTAAGGGCCATGATGACTTGACGTCATCCCCACCTTCCTCCGGTTTGTCACCGGCAGTC  892172

Query  308     TCCTTAGAGTGCCCACCATGACGTGCTGGTAACTAAGGACAAGGGTTGCGCTCGTTACGG  367
               ||||||||||||||||||| ||||||||||||||||||||||||||||||||||||||||
Sbjct  892171  TCCTTAGAGTGCCCACCATAACGTGCTGGTAACTAAGGACAAGGGTTGCGCTCGTTACGG  892112

Query  368     GACTTAACCCAACATCTCACGACACGAGCTGACGACAGCCATGCAGCACCTGTCTCAATG  427
               ||||||||||||||||||||||||||||||||||||||||||||||||||||||||||||
Sbjct  892111  GACTTAACCCAACATCTCACGACACGAGCTGACGACAGCCATGCAGCACCTGTCTCAATG  892052

Query  428     NNCCCGAAGGCACCAATCCATCTCTGGAAAGTTCATTGGATGTCAAGGCCTGGTAAGGTT  487
                 ||||||||||||||||||||||||||||||||||||||||||||||||||||||||||
Sbjct  892051  CTCCCGAAGGCACCAATCCATCTCTGGAAAGTTCATTGGATGTCAAGGCCTGGTAAGGTT  891992

Query  488     CTTCGCGTTGCTTCGAATTAAACCACATGCTCCACCGCTTGTGCGGGCCCCCGTCAATTC  547
               ||||||||||||||||||||||||||||||||||||||||||||||||||||||||||||
Sbjct  891991  CTTCGCGTTGCTTCGAATTAAACCACATGCTCCACCGCTTGTGCGGGCCCCCGTCAATTC  891932

Query  548     ATTTGAGTTTTAACCTTGCGGCCGTACTCCCCAGGCGGTCAACTTAATGCGTTAGCTGCG  607
               ||||||||||||||||||||||||||||||||||||||||||||||||||||||||||||
Sbjct  891931  ATTTGAGTTTTAACCTTGCGGCCGTACTCCCCAGGCGGTCAACTTAATGCGTTAGCTGCG  891872

Query  608     CCACTAAGAGCTCAAGGCTCCCAACGGCTAGTTGACATCGTTTACGGCGTGGACTACCAG  667
               ||||||||||||||||||||||||||||||||||||||||||||||||||||||||||||
Sbjct  891871  CCACTAAGAGCTCAAGGCTCCCAACGGCTAGTTGACATCGTTTACGGCGTGGACTACCAG  891812

Query  668     GGTATCTAATCCTGTTTGCTCCCCACGCTTTCGCACCTCAGTGTCAGTATCAGTCCAGGT  727
               ||||||||||||||||||||||||||||||||||||||||||||||||||||||||||||
Sbjct  891811  GGTATCTAATCCTGTTTGCTCCCCACGCTTTCGCACCTCAGTGTCAGTATCAGTCCAGGT  891752

Query  728     GGTCGCCTTCGCCACTGGTGTTCCTTCCTATATCTACGCATTTCACCGCTACACAGGAAA  787
               ||||||||||||||||||||||||||||||||||||||||||||||||||||||||||||
Sbjct  891751  GGTCGCCTTCGCCACTGGTGTTCCTTCCTATATCTACGCATTTCACCGCTACACAGGAAA  891692

Query  788     TTCCACCACCCTCTACCATACTCTAGCTCGACAGTTTTGAATGCAGTTCCCAGGTTGAGC  847
               ||||||||||||||||||||||||||||||||||||||||||||||||||||||||||||
Sbjct  891691  TTCCACCACCCTCTACCATACTCTAGCTCGACAGTTTTGAATGCAGTTCCCAGGTTGAGC  891632

Query  848     CCGGGGCTTTCACATCCAACTTAACGAACCACCTACGCGCGCTTTACGCCCAGTAATTCC  907
               ||||||||||||||||||||||||||||||||||||||||||||||||||||||||||||
Sbjct  891631  CCGGGGCTTTCACATCCAACTTAACGAACCACCTACGCGCGCTTTACGCCCAGTAATTCC  891572

Query  908     GATTAACGCTTGCACCCTCTGTATTACCGCGGCTGCTGGCACAGAGTTAGCCGGTGCTTA  967
               ||||||||||||||||||||||||||||||||||||||||||||||||||||||||||||
Sbjct  891571  GATTAACGCTTGCACCCTCTGTATTACCGCGGCTGCTGGCACAGAGTTAGCCGGTGCTTA  891512

Query  968     TTCTGTCGGTAACGTCAAAACACTAACGTATTAGGTTAATGCCCTTCCTCCCAACTTAAA  1027
               ||||||||||||||||||||||||||||||||||||||||||||||||||||||||||||
Sbjct  891511  TTCTGTCGGTAACGTCAAAACACTAACGTATTAGGTTAATGCCCTTCCTCCCAACTTAAA  891452

Query  1028    GTGCTTTACAATCCGA  1043
               ||||||||||||||||
Sbjct  891451  GTGCTTTACAATCCGA  891436
```

Range 2: 4564277 to 4565312GenBankGraphics

Next Match
Previous Match
First Match

Alignment statistics for match #2

| Score | Expect | Identities | Gaps | Strand | Frame |
| --- | --- | --- | --- | --- | --- |
| 1901 bits(1029) | 0.0() | 1033/1036(99%) | 0/1036(0%) | Plus/Plus |  |

Features:

rRNA-16S ribosomal RNA

```
Query  8        CCCGAAGGTTAGACTAGCTACTTCTGGTGCAACCCACTCCCATGGTGTGACGGGCGGTGT  67
                ||||||||||||||||||||||||||||||||||||||||||||||||||||||||||||
Sbjct  4564277  CCCGAAGGTTAGACTAGCTACTTCTGGTGCAACCCACTCCCATGGTGTGACGGGCGGTGT  4564336

Query  68       GTACAAGGCCCGGGAACGTATTCACCGCGACATTCTGATTCGCGATTACTAGCGATTCCG  127
                ||||||||||||||||||||||||||||||||||||||||||||||||||||||||||||
Sbjct  4564337  GTACAAGGCCCGGGAACGTATTCACCGCGACATTCTGATTCGCGATTACTAGCGATTCCG  4564396

Query  128      ACTTCACGCAGTCGAGTTGCAGACTGCGATCCGGACTACGATCGGTTTTGTGGGATTAGC  187
                ||||||||||||||||||||||||||||||||||||||||||||||||||||||||||||
Sbjct  4564397  ACTTCACGCAGTCGAGTTGCAGACTGCGATCCGGACTACGATCGGTTTTGTGGGATTAGC  4564456

Query  188      TCCACCTCGCGGCTTGGCAACCCTCTGTACCGACCATTGTAGCACGTGTGTAGCCCAGGC  247
                ||||||||||||||||||||||||||||||||||||||||||||||||||||||||||||
Sbjct  4564457  TCCACCTCGCGGCTTGGCAACCCTCTGTACCGACCATTGTAGCACGTGTGTAGCCCAGGC  4564516

Query  248      CGTAAGGGCCATGATGACTTGACGTCATCCCCACCTTCCTCCGGTTTGTCACCGGCAGTC  307
                ||||||||||||||||||||||||||||||||||||||||||||||||||||||||||||
Sbjct  4564517  CGTAAGGGCCATGATGACTTGACGTCATCCCCACCTTCCTCCGGTTTGTCACCGGCAGTC  4564576

Query  308      TCCTTAGAGTGCCCACCATGACGTGCTGGTAACTAAGGACAAGGGTTGCGCTCGTTACGG  367
                ||||||||||||||||||| ||||||||||||||||||||||||||||||||||||||||
Sbjct  4564577  TCCTTAGAGTGCCCACCATAACGTGCTGGTAACTAAGGACAAGGGTTGCGCTCGTTACGG  4564636

Query  368      GACTTAACCCAACATCTCACGACACGAGCTGACGACAGCCATGCAGCACCTGTCTCAATG  427
                ||||||||||||||||||||||||||||||||||||||||||||||||||||||||||||
Sbjct  4564637  GACTTAACCCAACATCTCACGACACGAGCTGACGACAGCCATGCAGCACCTGTCTCAATG  4564696

Query  428      NNCCCGAAGGCACCAATCCATCTCTGGAAAGTTCATTGGATGTCAAGGCCTGGTAAGGTT  487
                  ||||||||||||||||||||||||||||||||||||||||||||||||||||||||||
Sbjct  4564697  CTCCCGAAGGCACCAATCCATCTCTGGAAAGTTCATTGGATGTCAAGGCCTGGTAAGGTT  4564756

Query  488      CTTCGCGTTGCTTCGAATTAAACCACATGCTCCACCGCTTGTGCGGGCCCCCGTCAATTC  547
                ||||||||||||||||||||||||||||||||||||||||||||||||||||||||||||
Sbjct  4564757  CTTCGCGTTGCTTCGAATTAAACCACATGCTCCACCGCTTGTGCGGGCCCCCGTCAATTC  4564816

Query  548      ATTTGAGTTTTAACCTTGCGGCCGTACTCCCCAGGCGGTCAACTTAATGCGTTAGCTGCG  607
                ||||||||||||||||||||||||||||||||||||||||||||||||||||||||||||
Sbjct  4564817  ATTTGAGTTTTAACCTTGCGGCCGTACTCCCCAGGCGGTCAACTTAATGCGTTAGCTGCG  4564876

Query  608      CCACTAAGAGCTCAAGGCTCCCAACGGCTAGTTGACATCGTTTACGGCGTGGACTACCAG  667
                ||||||||||||||||||||||||||||||||||||||||||||||||||||||||||||
Sbjct  4564877  CCACTAAGAGCTCAAGGCTCCCAACGGCTAGTTGACATCGTTTACGGCGTGGACTACCAG  4564936

Query  668      GGTATCTAATCCTGTTTGCTCCCCACGCTTTCGCACCTCAGTGTCAGTATCAGTCCAGGT  727
                ||||||||||||||||||||||||||||||||||||||||||||||||||||||||||||
Sbjct  4564937  GGTATCTAATCCTGTTTGCTCCCCACGCTTTCGCACCTCAGTGTCAGTATCAGTCCAGGT  4564996

Query  728      GGTCGCCTTCGCCACTGGTGTTCCTTCCTATATCTACGCATTTCACCGCTACACAGGAAA  787
                ||||||||||||||||||||||||||||||||||||||||||||||||||||||||||||
Sbjct  4564997  GGTCGCCTTCGCCACTGGTGTTCCTTCCTATATCTACGCATTTCACCGCTACACAGGAAA  4565056

Query  788      TTCCACCACCCTCTACCATACTCTAGCTCGACAGTTTTGAATGCAGTTCCCAGGTTGAGC  847
                ||||||||||||||||||||||||||||||||||||||||||||||||||||||||||||
Sbjct  4565057  TTCCACCACCCTCTACCATACTCTAGCTCGACAGTTTTGAATGCAGTTCCCAGGTTGAGC  4565116

Query  848      CCGGGGCTTTCACATCCAACTTAACGAACCACCTACGCGCGCTTTACGCCCAGTAATTCC  907
                ||||||||||||||||||||||||||||||||||||||||||||||||||||||||||||
Sbjct  4565117  CCGGGGCTTTCACATCCAACTTAACGAACCACCTACGCGCGCTTTACGCCCAGTAATTCC  4565176

Query  908      GATTAACGCTTGCACCCTCTGTATTACCGCGGCTGCTGGCACAGAGTTAGCCGGTGCTTA  967
                ||||||||||||||||||||||||||||||||||||||||||||||||||||||||||||
Sbjct  4565177  GATTAACGCTTGCACCCTCTGTATTACCGCGGCTGCTGGCACAGAGTTAGCCGGTGCTTA  4565236

Query  968      TTCTGTCGGTAACGTCAAAACACTAACGTATTAGGTTAATGCCCTTCCTCCCAACTTAAA  1027
                ||||||||||||||||||||||||||||||||||||||||||||||||||||||||||||
Sbjct  4565237  TTCTGTCGGTAACGTCAAAACACTAACGTATTAGGTTAATGCCCTTCCTCCCAACTTAAA  4565296

Query  1028     GTGCTTTACAATCCGA  1043
                ||||||||||||||||
Sbjct  4565297  GTGCTTTACAATCCGA  4565312
```

Range 3: 5981149 to 5982184GenBankGraphics

Next Match
Previous Match
First Match

Alignment statistics for match #3

| Score | Expect | Identities | Gaps | Strand | Frame |
| --- | --- | --- | --- | --- | --- |
| 1901 bits(1029) | 0.0() | 1033/1036(99%) | 0/1036(0%) | Plus/Plus |  |

Features:

rRNA-16S ribosomal RNA

```
Query  8        CCCGAAGGTTAGACTAGCTACTTCTGGTGCAACCCACTCCCATGGTGTGACGGGCGGTGT  67
                ||||||||||||||||||||||||||||||||||||||||||||||||||||||||||||
Sbjct  5981149  CCCGAAGGTTAGACTAGCTACTTCTGGTGCAACCCACTCCCATGGTGTGACGGGCGGTGT  5981208

Query  68       GTACAAGGCCCGGGAACGTATTCACCGCGACATTCTGATTCGCGATTACTAGCGATTCCG  127
                ||||||||||||||||||||||||||||||||||||||||||||||||||||||||||||
Sbjct  5981209  GTACAAGGCCCGGGAACGTATTCACCGCGACATTCTGATTCGCGATTACTAGCGATTCCG  5981268

Query  128      ACTTCACGCAGTCGAGTTGCAGACTGCGATCCGGACTACGATCGGTTTTGTGGGATTAGC  187
                ||||||||||||||||||||||||||||||||||||||||||||||||||||||||||||
Sbjct  5981269  ACTTCACGCAGTCGAGTTGCAGACTGCGATCCGGACTACGATCGGTTTTGTGGGATTAGC  5981328

Query  188      TCCACCTCGCGGCTTGGCAACCCTCTGTACCGACCATTGTAGCACGTGTGTAGCCCAGGC  247
                ||||||||||||||||||||||||||||||||||||||||||||||||||||||||||||
Sbjct  5981329  TCCACCTCGCGGCTTGGCAACCCTCTGTACCGACCATTGTAGCACGTGTGTAGCCCAGGC  5981388

Query  248      CGTAAGGGCCATGATGACTTGACGTCATCCCCACCTTCCTCCGGTTTGTCACCGGCAGTC  307
                ||||||||||||||||||||||||||||||||||||||||||||||||||||||||||||
Sbjct  5981389  CGTAAGGGCCATGATGACTTGACGTCATCCCCACCTTCCTCCGGTTTGTCACCGGCAGTC  5981448

Query  308      TCCTTAGAGTGCCCACCATGACGTGCTGGTAACTAAGGACAAGGGTTGCGCTCGTTACGG  367
                ||||||||||||||||||| ||||||||||||||||||||||||||||||||||||||||
Sbjct  5981449  TCCTTAGAGTGCCCACCATAACGTGCTGGTAACTAAGGACAAGGGTTGCGCTCGTTACGG  5981508

Query  368      GACTTAACCCAACATCTCACGACACGAGCTGACGACAGCCATGCAGCACCTGTCTCAATG  427
                ||||||||||||||||||||||||||||||||||||||||||||||||||||||||||||
Sbjct  5981509  GACTTAACCCAACATCTCACGACACGAGCTGACGACAGCCATGCAGCACCTGTCTCAATG  5981568

Query  428      NNCCCGAAGGCACCAATCCATCTCTGGAAAGTTCATTGGATGTCAAGGCCTGGTAAGGTT  487
                  ||||||||||||||||||||||||||||||||||||||||||||||||||||||||||
Sbjct  5981569  CTCCCGAAGGCACCAATCCATCTCTGGAAAGTTCATTGGATGTCAAGGCCTGGTAAGGTT  5981628

Query  488      CTTCGCGTTGCTTCGAATTAAACCACATGCTCCACCGCTTGTGCGGGCCCCCGTCAATTC  547
                ||||||||||||||||||||||||||||||||||||||||||||||||||||||||||||
Sbjct  5981629  CTTCGCGTTGCTTCGAATTAAACCACATGCTCCACCGCTTGTGCGGGCCCCCGTCAATTC  5981688

Query  548      ATTTGAGTTTTAACCTTGCGGCCGTACTCCCCAGGCGGTCAACTTAATGCGTTAGCTGCG  607
                ||||||||||||||||||||||||||||||||||||||||||||||||||||||||||||
Sbjct  5981689  ATTTGAGTTTTAACCTTGCGGCCGTACTCCCCAGGCGGTCAACTTAATGCGTTAGCTGCG  5981748

Query  608      CCACTAAGAGCTCAAGGCTCCCAACGGCTAGTTGACATCGTTTACGGCGTGGACTACCAG  667
                ||||||||||||||||||||||||||||||||||||||||||||||||||||||||||||
Sbjct  5981749  CCACTAAGAGCTCAAGGCTCCCAACGGCTAGTTGACATCGTTTACGGCGTGGACTACCAG  5981808

Query  668      GGTATCTAATCCTGTTTGCTCCCCACGCTTTCGCACCTCAGTGTCAGTATCAGTCCAGGT  727
                ||||||||||||||||||||||||||||||||||||||||||||||||||||||||||||
Sbjct  5981809  GGTATCTAATCCTGTTTGCTCCCCACGCTTTCGCACCTCAGTGTCAGTATCAGTCCAGGT  5981868

Query  728      GGTCGCCTTCGCCACTGGTGTTCCTTCCTATATCTACGCATTTCACCGCTACACAGGAAA  787
                ||||||||||||||||||||||||||||||||||||||||||||||||||||||||||||
Sbjct  5981869  GGTCGCCTTCGCCACTGGTGTTCCTTCCTATATCTACGCATTTCACCGCTACACAGGAAA  5981928

Query  788      TTCCACCACCCTCTACCATACTCTAGCTCGACAGTTTTGAATGCAGTTCCCAGGTTGAGC  847
                ||||||||||||||||||||||||||||||||||||||||||||||||||||||||||||
Sbjct  5981929  TTCCACCACCCTCTACCATACTCTAGCTCGACAGTTTTGAATGCAGTTCCCAGGTTGAGC  5981988

Query  848      CCGGGGCTTTCACATCCAACTTAACGAACCACCTACGCGCGCTTTACGCCCAGTAATTCC  907
                ||||||||||||||||||||||||||||||||||||||||||||||||||||||||||||
Sbjct  5981989  CCGGGGCTTTCACATCCAACTTAACGAACCACCTACGCGCGCTTTACGCCCAGTAATTCC  5982048

Query  908      GATTAACGCTTGCACCCTCTGTATTACCGCGGCTGCTGGCACAGAGTTAGCCGGTGCTTA  967
                ||||||||||||||||||||||||||||||||||||||||||||||||||||||||||||
Sbjct  5982049  GATTAACGCTTGCACCCTCTGTATTACCGCGGCTGCTGGCACAGAGTTAGCCGGTGCTTA  5982108

Query  968      TTCTGTCGGTAACGTCAAAACACTAACGTATTAGGTTAATGCCCTTCCTCCCAACTTAAA  1027
                ||||||||||||||||||||||||||||||||||||||||||||||||||||||||||||
Sbjct  5982109  TTCTGTCGGTAACGTCAAAACACTAACGTATTAGGTTAATGCCCTTCCTCCCAACTTAAA  5982168

Query  1028     GTGCTTTACAATCCGA  1043
                ||||||||||||||||
Sbjct  5982169  GTGCTTTACAATCCGA  5982184
```

Range 4: 224739 to 225774GenBankGraphics

Next Match
Previous Match
First Match

Alignment statistics for match #4

| Score | Expect | Identities | Gaps | Strand | Frame |
| --- | --- | --- | --- | --- | --- |
| 1895 bits(1026) | 0.0() | 1032/1036(99%) | 0/1036(0%) | Plus/Minus |  |

Features:

rRNA-16S ribosomal RNA

```
Query  8       CCCGAAGGTTAGACTAGCTACTTCTGGTGCAACCCACTCCCATGGTGTGACGGGCGGTGT  67
               ||||||||||||||||||||||||||||||||||||||||||||||||||||||||||||
Sbjct  225774  CCCGAAGGTTAGACTAGCTACTTCTGGTGCAACCCACTCCCATGGTGTGACGGGCGGTGT  225715

Query  68      GTACAAGGCCCGGGAACGTATTCACCGCGACATTCTGATTCGCGATTACTAGCGATTCCG  127
               ||||||||||||||||||||||||||||||||||||||||||||||||||||||||||||
Sbjct  225714  GTACAAGGCCCGGGAACGTATTCACCGCGACATTCTGATTCGCGATTACTAGCGATTCCG  225655

Query  128     ACTTCACGCAGTCGAGTTGCAGACTGCGATCCGGACTACGATCGGTTTTGTGGGATTAGC  187
               ||||||||||||||||||||||||||||||||||||||||||||||||||||||||||||
Sbjct  225654  ACTTCACGCAGTCGAGTTGCAGACTGCGATCCGGACTACGATCGGTTTTGTGGGATTAGC  225595

Query  188     TCCACCTCGCGGCTTGGCAACCCTCTGTACCGACCATTGTAGCACGTGTGTAGCCCAGGC  247
               ||||||||||||||||||||||||||||||||||||||||||||||||||||||||||||
Sbjct  225594  TCCACCTCGCGGCTTGGCAACCCTCTGTACCGACCATTGTAGCACGTGTGTAGCCCAGGC  225535

Query  248     CGTAAGGGCCATGATGACTTGACGTCATCCCCACCTTCCTCCGGTTTGTCACCGGCAGTC  307
               ||||||||||||||||||||||||||||||||||||||||||||||||||||||||||||
Sbjct  225534  CGTAAGGGCCATGATGACTTGACGTCATCCCCACCTTCCTCCGGTTTGTCACCGGCAGTC  225475

Query  308     TCCTTAGAGTGCCCACCATGACGTGCTGGTAACTAAGGACAAGGGTTGCGCTCGTTACGG  367
               ||||||||||||||||||| ||||||||||||||||||||||||||||||||||||||||
Sbjct  225474  TCCTTAGAGTGCCCACCATCACGTGCTGGTAACTAAGGACAAGGGTTGCGCTCGTTACGG  225415

Query  368     GACTTAACCCAACATCTCACGACACGAGCTGACGACAGCCATGCAGCACCTGTCTCAATG  427
               ||||||||||||||||||||||||||||||||||||||||||||||||||||||||||||
Sbjct  225414  GACTTAACCCAACATCTCACGACACGAGCTGACGACAGCCATGCAGCACCTGTCTCAATG  225355

Query  428     NNCCCGAAGGCACCAATCCATCTCTGGAAAGTTCATTGGATGTCAAGGCCTGGTAAGGTT  487
                 ||||||||||||||||||||||||||||||||||||||||||||||||||||||||||
Sbjct  225354  CTCCCGAAGGCACCAATCCATCTCTGGAAAGTTCATTGGATGTCAAGGCCTGGTAAGGTT  225295

Query  488     CTTCGCGTTGCTTCGAATTAAACCACATGCTCCACCGCTTGTGCGGGCCCCCGTCAATTC  547
               ||||||||||||||||||||||||||||||||||||||||||||||||||||||||||||
Sbjct  225294  CTTCGCGTTGCTTCGAATTAAACCACATGCTCCACCGCTTGTGCGGGCCCCCGTCAATTC  225235

Query  548     ATTTGAGTTTTAACCTTGCGGCCGTACTCCCCAGGCGGTCAACTTAATGCGTTAGCTGCG  607
               ||||||||||||||||||||||||||||||||||||||||||||||||||||||||||||
Sbjct  225234  ATTTGAGTTTTAACCTTGCGGCCGTACTCCCCAGGCGGTCAACTTAATGCGTTAGCTGCG  225175

Query  608     CCACTAAGAGCTCAAGGCTCCCAACGGCTAGTTGACATCGTTTACGGCGTGGACTACCAG  667
               ||||||||||||||||||||||||||||||||||||||||||||||||||||||||||||
Sbjct  225174  CCACTAAGAGCTCAAGGCTCCCAACGGCTAGTTGACATCGTTTACGGCGTGGACTACCAG  225115

Query  668     GGTATCTAATCCTGTTTGCTCCCCACGCTTTCGCACCTCAGTGTCAGTATCAGTCCAGGT  727
               ||||||||||||||||||||||||||||||||||||||||||||||||||||||||||||
Sbjct  225114  GGTATCTAATCCTGTTTGCTCCCCACGCTTTCGCACCTCAGTGTCAGTATCAGTCCAGGT  225055

Query  728     GGTCGCCTTCGCCACTGGTGTTCCTTCCTATATCTACGCATTTCACCGCTACACAGGAAA  787
               ||||||||||||||||||||||||||||||||||||||||||||||||||||||||||||
Sbjct  225054  GGTCGCCTTCGCCACTGGTGTTCCTTCCTATATCTACGCATTTCACCGCTACACAGGAAA  224995

Query  788     TTCCACCACCCTCTACCATACTCTAGCTCGACAGTTTTGAATGCAGTTCCCAGGTTGAGC  847
               ||||||||||||||||||||||||||||||||||||||||||||||||||||||||||||
Sbjct  224994  TTCCACCACCCTCTACCATACTCTAGCTCGACAGTTTTGAATGCAGTTCCCAGGTTGAGC  224935

Query  848     CCGGGGCTTTCACATCCAACTTAACGAACCACCTACGCGCGCTTTACGCCCAGTAATTCC  907
               ||||||||||||||||||||||||||||||||||||||||||||||||||||||||||||
Sbjct  224934  CCGGGGCTTTCACATCCAACTTAACGAACCACCTACGCGCGCTTTACGCCCAGTAATTCC  224875

Query  908     GATTAACGCTTGCACCCTCTGTATTACCGCGGCTGCTGGCACAGAGTTAGCCGGTGCTTA  967
               ||||||||||||||||||||||||||||||||||||||||||||||||||||||||||||
Sbjct  224874  GATTAACGCTTGCACCCTCTGTATTACCGCGGCTGCTGGCACAGAGTTAGCCGGTGCTTA  224815

Query  968     TTCTGTCGGTAACGTCAAAACACTAACGTATTAGGTTAATGCCCTTCCTCCCAACTTAAA  1027
               |||||||||||||||||||||||||| |||||||||||||||||||||||||||||||||
Sbjct  224814  TTCTGTCGGTAACGTCAAAACACTAATGTATTAGGTTAATGCCCTTCCTCCCAACTTAAA  224755

Query  1028    GTGCTTTACAATCCGA  1043
               ||||||||||||||||
Sbjct  224754  GTGCTTTACAATCCGA  224739
```

Range 5: 5595054 to 5596089GenBankGraphics

Next Match
Previous Match
First Match

Alignment statistics for match #5

| Score | Expect | Identities | Gaps | Strand | Frame |
| --- | --- | --- | --- | --- | --- |
| 1895 bits(1026) | 0.0() | 1032/1036(99%) | 0/1036(0%) | Plus/Plus |  |

Features:

rRNA-16S ribosomal RNA

```
Query  8        CCCGAAGGTTAGACTAGCTACTTCTGGTGCAACCCACTCCCATGGTGTGACGGGCGGTGT  67
                ||||||||||||||||||||||||||||||||||||||||||||||||||||||||||||
Sbjct  5595054  CCCGAAGGTTAGACTAGCTACTTCTGGTGCAACCCACTCCCATGGTGTGACGGGCGGTGT  5595113

Query  68       GTACAAGGCCCGGGAACGTATTCACCGCGACATTCTGATTCGCGATTACTAGCGATTCCG  127
                ||||||||||||||||||||||||||||||||||||||||||||||||||||||||||||
Sbjct  5595114  GTACAAGGCCCGGGAACGTATTCACCGCGACATTCTGATTCGCGATTACTAGCGATTCCG  5595173

Query  128      ACTTCACGCAGTCGAGTTGCAGACTGCGATCCGGACTACGATCGGTTTTGTGGGATTAGC  187
                ||||||||||||||||||||||||||||||||||||||||||||||||||||||||||||
Sbjct  5595174  ACTTCACGCAGTCGAGTTGCAGACTGCGATCCGGACTACGATCGGTTTTGTGGGATTAGC  5595233

Query  188      TCCACCTCGCGGCTTGGCAACCCTCTGTACCGACCATTGTAGCACGTGTGTAGCCCAGGC  247
                ||||||||||||||||||||||||||||||||||||||||||||||||||||||||||||
Sbjct  5595234  TCCACCTCGCGGCTTGGCAACCCTCTGTACCGACCATTGTAGCACGTGTGTAGCCCAGGC  5595293

Query  248      CGTAAGGGCCATGATGACTTGACGTCATCCCCACCTTCCTCCGGTTTGTCACCGGCAGTC  307
                ||||||||||||||||||||||||||||||||||||||||||||||||||||||||||||
Sbjct  5595294  CGTAAGGGCCATGATGACTTGACGTCATCCCCACCTTCCTCCGGTTTGTCACCGGCAGTC  5595353

Query  308      TCCTTAGAGTGCCCACCATGACGTGCTGGTAACTAAGGACAAGGGTTGCGCTCGTTACGG  367
                ||||||||||||||||||| |||||||||||| |||||||||||||||||||||||||||
Sbjct  5595354  TCCTTAGAGTGCCCACCATAACGTGCTGGTAAGTAAGGACAAGGGTTGCGCTCGTTACGG  5595413

Query  368      GACTTAACCCAACATCTCACGACACGAGCTGACGACAGCCATGCAGCACCTGTCTCAATG  427
                ||||||||||||||||||||||||||||||||||||||||||||||||||||||||||||
Sbjct  5595414  GACTTAACCCAACATCTCACGACACGAGCTGACGACAGCCATGCAGCACCTGTCTCAATG  5595473

Query  428      NNCCCGAAGGCACCAATCCATCTCTGGAAAGTTCATTGGATGTCAAGGCCTGGTAAGGTT  487
                  ||||||||||||||||||||||||||||||||||||||||||||||||||||||||||
Sbjct  5595474  CTCCCGAAGGCACCAATCCATCTCTGGAAAGTTCATTGGATGTCAAGGCCTGGTAAGGTT  5595533

Query  488      CTTCGCGTTGCTTCGAATTAAACCACATGCTCCACCGCTTGTGCGGGCCCCCGTCAATTC  547
                ||||||||||||||||||||||||||||||||||||||||||||||||||||||||||||
Sbjct  5595534  CTTCGCGTTGCTTCGAATTAAACCACATGCTCCACCGCTTGTGCGGGCCCCCGTCAATTC  5595593

Query  548      ATTTGAGTTTTAACCTTGCGGCCGTACTCCCCAGGCGGTCAACTTAATGCGTTAGCTGCG  607
                ||||||||||||||||||||||||||||||||||||||||||||||||||||||||||||
Sbjct  5595594  ATTTGAGTTTTAACCTTGCGGCCGTACTCCCCAGGCGGTCAACTTAATGCGTTAGCTGCG  5595653

Query  608      CCACTAAGAGCTCAAGGCTCCCAACGGCTAGTTGACATCGTTTACGGCGTGGACTACCAG  667
                ||||||||||||||||||||||||||||||||||||||||||||||||||||||||||||
Sbjct  5595654  CCACTAAGAGCTCAAGGCTCCCAACGGCTAGTTGACATCGTTTACGGCGTGGACTACCAG  5595713

Query  668      GGTATCTAATCCTGTTTGCTCCCCACGCTTTCGCACCTCAGTGTCAGTATCAGTCCAGGT  727
                ||||||||||||||||||||||||||||||||||||||||||||||||||||||||||||
Sbjct  5595714  GGTATCTAATCCTGTTTGCTCCCCACGCTTTCGCACCTCAGTGTCAGTATCAGTCCAGGT  5595773

Query  728      GGTCGCCTTCGCCACTGGTGTTCCTTCCTATATCTACGCATTTCACCGCTACACAGGAAA  787
                ||||||||||||||||||||||||||||||||||||||||||||||||||||||||||||
Sbjct  5595774  GGTCGCCTTCGCCACTGGTGTTCCTTCCTATATCTACGCATTTCACCGCTACACAGGAAA  5595833

Query  788      TTCCACCACCCTCTACCATACTCTAGCTCGACAGTTTTGAATGCAGTTCCCAGGTTGAGC  847
                ||||||||||||||||||||||||||||||||||||||||||||||||||||||||||||
Sbjct  5595834  TTCCACCACCCTCTACCATACTCTAGCTCGACAGTTTTGAATGCAGTTCCCAGGTTGAGC  5595893

Query  848      CCGGGGCTTTCACATCCAACTTAACGAACCACCTACGCGCGCTTTACGCCCAGTAATTCC  907
                ||||||||||||||||||||||||||||||||||||||||||||||||||||||||||||
Sbjct  5595894  CCGGGGCTTTCACATCCAACTTAACGAACCACCTACGCGCGCTTTACGCCCAGTAATTCC  5595953

Query  908      GATTAACGCTTGCACCCTCTGTATTACCGCGGCTGCTGGCACAGAGTTAGCCGGTGCTTA  967
                ||||||||||||||||||||||||||||||||||||||||||||||||||||||||||||
Sbjct  5595954  GATTAACGCTTGCACCCTCTGTATTACCGCGGCTGCTGGCACAGAGTTAGCCGGTGCTTA  5596013

Query  968      TTCTGTCGGTAACGTCAAAACACTAACGTATTAGGTTAATGCCCTTCCTCCCAACTTAAA  1027
                ||||||||||||||||||||||||||||||||||||||||||||||||||||||||||||
Sbjct  5596014  TTCTGTCGGTAACGTCAAAACACTAACGTATTAGGTTAATGCCCTTCCTCCCAACTTAAA  5596073

Query  1028     GTGCTTTACAATCCGA  1043
                ||||||||||||||||
Sbjct  5596074  GTGCTTTACAATCCGA  5596089
```


BLAST is a registered trademark of the National Library of Medicine

Support center
Mailing list


YouTube

- National Library Of Medicine
- National Institutes Of Health
- U.S. Department of Health & Human Services
- USA.gov

### NCBI


National Center for Biotechnology Information,
 U.S. National Library of Medicine

8600 Rockville Pike,
Bethesda
 MD,
20894
USA

Policies and Guidelines
|
Contact


PreferencesTurn off

External link. Please review our privacy policy.
