# Supplementary material for: Isothermal microcalorimetry for thermal viable count of microorganisms in pure cultures and stabilized formulations
Source: BMC Microbiol. 2019 Mar 21;19:65. doi: 10.1186/s12866-019-1432-8 (PMC6429831; doi:10.1186/s12866-019-1432-8)
Supplement: Supplementary file 3 — 16S rRNA gene sequences’ BLASTN hits in zipped HTML format. (ZIP 15810 kb) [file 12866_2019_1432_MOESM3_ESM.zip › Best blastn hits/NCBI Blast_C yellow R -- 14..1074 of sequence (1061.html]

NCBI Blast:C yellow R -- 14..1074 of sequence (1061


- NCBI Home
- Sign in to NCBI
- Skip to Main Content
- Skip to Navigation
- About NCBI Accesskeys

U.S. National Library of Medicine

NCBI
National Center for Biotechnology Information

- My NCBI
- Sign in to NCBI
- Register
- Sign Out

BLAST ^®^ » blastn suite » RID-A4XJJMWX014


- Home
- Recent Results
- Saved Strategies
- Help

BLAST Results


Edit and Resubmit
Save Search Strategies
[Sign in above to save your search strategy]

Formatting options 


Download


How to read this page
Blast report description
Questions/comments


|  |  |
| --- | --- |
| Formatting options | |
| Show | Alignment as  HTML Plain text   Old View Reset form to defaults [?]  These options control formatting of alignments in results pages. The default is HTML, but other formats (including plain text) are available. PSSM and PssmWithParameters are representations of Position Specific Scoring Matrices and are only available for PSI-BLAST. The Advanced view option allows the database descriptions to be sorted by various indices in a table. |
| Alignment View | Pairwise Pairwise with dots for identities Query-anchored with dots for identities Query-anchored with letters for identities Flat query-anchored with dots for identities Flat query-anchored with letters for identities [?]  Choose how to view alignments. The default "pairwise" view shows how each subject sequence aligns individually to the query sequence. The "query-anchored" view shows how all subject sequences align to the query sequence. For each view type, you can choose to show "identities" (matching residues) as letters or dots. more... |
| Display | Graphical Overview   Linkout   Sequence Retrieval  NCBI-gi   CDS feature [?]  - Graphical Overview: Graphical Overview: Show graph of similar sequence regions aligned to query.   more... - NCBI-gi: Show NCBI gi identifiers. - CDS feature: Show annotated coding region and translation.   more... |
| Masking | Character:   X for protein, n for nucleotide Lower Case  Color:  Black Grey Red [?]  - Masking Character: Display masked (filtered) sequence regions as lower-case or as specific letters (N for nucleotide, P for protein). - Masking Color: Display masked sequence regions in the given color. |
| Limit results | Descriptions:  10 50 100 Graphical overview:  0 10 50 100  Alignments:  0 10 50 100 Line length:  60 90 120 150 [?]  - Descriptions: Show short descriptions for up to the given number of sequences. - Alignments: Show alignments for up to the given number of sequences, in order of statistical significance. - Line lenghth: Number of letters to show on one line in an alignment. |
|  | Organism Type common name, binomial, taxid, or group name. Only 20 top taxa will be shown.     Exclude   [?]  Show only sequences from the given organism. |
|  | Entrez query:  [?]  Show only those sequences that match the given Entrez query. more... |
|  | Expect Min:  Expect Max:  [?]  Show only sequences with expect values in the given range. more... |
|  | Percent Identity Min:  Percent Identity Max:  [?]  Show only sequences with percent identity values in the given range. |
| Format for | PSI-BLAST with inclusion threshold:  [?]  - Format for PSI-BLAST: The Position-Specific Iterated BLAST (PSI-BLAST) program performs iterative searches with a protein query,   in which sequences found in one round of search are used to build a custom score model for the next round.   more... - Inclusion Threshold: This sets the statistical significance threshold for including a sequence in the model used   by PSI-BLAST to create the PSSM on the next iteration. |

|  |  |  |  |  |  |
| --- | --- | --- | --- | --- | --- |
| Download | | | | | |
| Alignment  Text XML ASN.1 JSON Seq-align Hit Table(text) Hit Table(csv) Multiple-file XML2 Single-file XML2 Multiple-file JSON Single-file JSON SAM | Search Strategies  ASN.1 | PSSM to restart search  PSSM | [?] |  |  |

The Download link provides BLAST output that may be used as input to another program.
This includes parseable formats such as the tabular report or XML as well as the Search Strategy files read by the BLAST+ applications.
More details on the parseable (XML, tabular, and ASN.1) reports can be found at
https://www.ncbi.nlm.nih.gov/books/NBK153387/  
  

The following formats are offered under the Alignment section:  
1). "Text". Non-HTML standard BLAST report.  
2). "XML". XML report based upon the DTD at https://www.ncbi.nlm.nih.gov/data\_specs/dtd/NCBI\_BlastOutput.dtd  
3). "ASN.1". Alignment written out in Abstract Syntax Notation 1.  
4). "JSON Seq-align". Alignment written out in JSON.  
4). "Hit Table(text)". The tabular report as text.  
5). "Hit Table(csv)". The tabular report ready for import into spread-sheet programs like Excel.  
6). "XML2". New XML format described at ftp://ftp.ncbi.nlm.nih.gov/blast/documents/NEWXML/xml2.pdf.  
7). "JSON". New JSON format described at ftp://ftp.ncbi.nlm.nih.gov/blast/documents/NEWXML/xml2.pdf.  
8). "SAM". Sequence Alignment Map format.

XML2 and JSON can be downloaded either as one file per query (multiple-file) or one file for all queries (single-file). These formats are listed as Multiple-file XML2 (and JSON) or Single-file XML (and JSON).

The following report is offered under the Search Strategy section:  
1). "ASN.1" Search Strategy. A record of the parameters, query, and database used in the search. This file can be used to start a stand-alone BLAST search, see
https://www.ncbi.nlm.nih.gov/books/NBK1763/#CmdLineAppsManual.I455\_BLAST\_search\_stra


# Job title: C yellow R -- 14..1074 of sequence (1061

Results for:

lcl|Query\_125223 C yellow R -- 14..1074 of sequence(1061bp)
[?]

Your BLAST job specified more than one input sequence.
This box lets you choose which input sequence to show BLAST results for.

RID
:   A4XJJMWX014 (Expires on 03-10 18:56 pm)

Query ID
:   lcl|Query\_125223
:   lcl|Query\_125223

Description
:   C yellow R -- 14..1074 of sequence

Molecule type
:   nucleic acid

Query Length
:   1061

Database Name
:   nr

Description
:   Nucleotide collection (nt) See details

Program
:   BLASTN 2.8.0+ Citation

  

Reference 

Zheng Zhang, Scott Schwartz, Lukas Wagner, and Webb Miller (2000), "A greedy algorithm for aligning DNA sequences", J Comput Biol 2000; 7(1-2):203-14.

Reference - database indexing

Aleksandr Morgulis, George Coulouris, Yan Raytselis, Thomas L. Madden, Richa Agarwala, Alejandro A. Schäffer (2008), "Database Indexing for Production MegaBLAST Searches", Bioinformatics 24:1757-1764.

Other reports:
Search Summary

[Taxonomy reports]
[Distance tree of results]
[MSA viewer]

Search Parameters

| Search parameter name | Search parameter value |
| --- | --- |
| Program | blastn |
| Word size | 28 |
| Expect value | 10 |
| Hitlist size | 100 |
| Match/Mismatch scores | 1,-2 |
| Gapcosts | 0,2.5 |
| Low Complexity Filter | Yes |
| Filter string | L;m; |
| Genetic Code | 1 |

Database

| Database parameter name | Database parameter value |
| --- | --- |
| Posted date | Mar 3, 2018 9:32 PM |
| Number of letters | 171,598,911,005 |
| Number of sequences | 46,870,701 |
| Entrez query | Includes:  Excludes:  None |

Karlin-Altschul statistics

| Params | Ungapped | Gapped |
| --- | --- | --- |
| Lambda | 1.33271 | 1.28 |
| K | 0.620991 | 0.46 |
| H | 1.12409 | 0.85 |

Results Statistics

| Results Statistics parameter name | Results Statistics parameter value |
| --- | --- |
| Length adjustment | 35 |
| Effective length of query | 1026 |
| Effective length of database | 169958436470 |
| Effective search space | 174377355818220 |
| Effective search space used | 174377355818220 |


## Graphic Summary

### Distribution of the top 112 Blast Hits on 100 subject sequences [?]

The graphic is an overview of the database sequences aligned to the query sequence. These are represented horizontal bars colored coded by score and showing the extent
of the alignment on the query sequence. Separate aligned regions on the same database sequence are connected by a thin grey line.
Mousing over an alignment shows the database sequence title. Clicking an alignment displays a box with more details about the alignment and
link to the sequence alignment itself in the Alignments section of the report.

Mouse over to see the title, click to show alignments

Color key for alignment scores

<40

40-50

50-80

80-200

>=200

Query

1

200

400

600

800

1000

Pantoea agglomerans strain AL14 16S ribosomal RNA gene,..

Score:1923 Evalue:0

Accession:MG819218.1

Alignment

Pantoea agglomerans strain Rk92 16S ribosomal RNA gene,..

Score:1923 Evalue:0

Accession:MG547769.1

Alignment

Pantoea agglomerans strain C410P1, complete genome

Score:1923 Evalue:0

Accession:CP016889.1

Alignment

Uncultured Pantoea sp. clone GJ\_(2)\_7 16S ribosomal RNA..

Score:1923 Evalue:0

Accession:KX603479.1

Alignment

Pantoea agglomerans strain SSE 16S ribosomal RNA gene, ..

Score:1923 Evalue:0

Accession:KT075193.1

Alignment

Pantoea agglomerans strain NSF 16S ribosomal RNA gene, ..

Score:1923 Evalue:0

Accession:KT075171.1

Alignment

Pantoea agglomerans strain GSB 16S ribosomal RNA gene, ..

Score:1923 Evalue:0

Accession:KT075162.1

Alignment

Pantoea agglomerans strain B81 16S ribosomal RNA gene, ..

Score:1923 Evalue:0

Accession:KJ781904.1

Alignment

Curtobacterium plantarum strain S4 16S ribosomal RNA ge..

Score:1923 Evalue:0

Accession:KP099434.1

Alignment

Curtobacterium plantarum strain S2 16S ribosomal RNA ge..

Score:1923 Evalue:0

Accession:KP099433.1

Alignment

Pantoea agglomerans strain ex2 culture-collection CGMCC..

Score:1923 Evalue:0

Accession:KF317872.1

Alignment

Pantoea agglomerans strain ABAC22 16S ribosomal RNA gen..

Score:1923 Evalue:0

Accession:KF003386.1

Alignment

Enterobacteriaceae bacterium SAP770.3 16S ribosomal RNA..

Score:1923 Evalue:0

Accession:JX067684.1

Alignment

Pantoea agglomerans strain Z01 16S ribosomal RNA gene, ..

Score:1923 Evalue:0

Accession:JX257179.1

Alignment

Uncultured Pantoea sp. clone WRFC18 16S ribosomal RNA g..

Score:1923 Evalue:0

Accession:JX279936.1

Alignment

Pantoea agglomerans strain SR-1 16S ribosomal RNA gene,..

Score:1923 Evalue:0

Accession:FJ593000.1

Alignment

Pantoea agglomerans strain SN-2 16S ribosomal RNA gene,..

Score:1923 Evalue:0

Accession:FJ592996.1

Alignment

Pantoea agglomerans strain BBPE0103202 16S ribosomal RN..

Score:1923 Evalue:0

Accession:FJ357809.1

Alignment

Marine bacterium MSC106 16S ribosomal RNA gene, partial..

Score:1923 Evalue:0

Accession:EU753130.1

Alignment

Pantoea agglomerans strain TMPSB-P1 16S ribosomal RNA g..

Score:1923 Evalue:0

Accession:EU047555.1

Alignment

Pantoea agglomerans strain FL1 16S ribosomal RNA gene, ..

Score:1923 Evalue:0

Accession:DQ307452.1

Alignment

Enterobacteriaceae bacterium SAP108.1 16S ribosomal RNA..

Score:1919 Evalue:0

Accession:JX067654.1

Alignment

Pantoea agglomerans strain CCRI01 16S ribosomal RNA gen..

Score:1917 Evalue:0

Accession:KT779903.1

Alignment

Pantoea sp. CanR-85 16S ribosomal RNA gene, partial seq..

Score:1917 Evalue:0

Accession:KT580675.1

Alignment

Enterobacteriaceae bacterium X1/SB37 gene for 16S ribos..

Score:1917 Evalue:0

Accession:LC007922.1

Alignment

Pantoea agglomerans strain P19 16S ribosomal RNA gene, ..

Score:1917 Evalue:0

Accession:KF465834.1

Alignment

Pantoea agglomerans strain Cl-17 16S ribosomal RNA gene..

Score:1917 Evalue:0

Accession:KC178591.1

Alignment

Enterobacteriaceae bacterium SAP952.2 16S ribosomal RNA..

Score:1917 Evalue:0

Accession:JX067721.1

Alignment

Uncultured Pantoea sp. clone WRFC196 16S ribosomal RNA ..

Score:1917 Evalue:0

Accession:JX294750.1

Alignment

Pantoea sp. PR 16S ribosomal RNA gene, partial sequence

Score:1917 Evalue:0

Accession:JQ412064.1

Alignment

Pantoea agglomerans strain KB64 16S ribosomal RNA gene,..

Score:1917 Evalue:0

Accession:JF327484.1

Alignment

Pantoea agglomerans strain KB38 16S ribosomal RNA gene,..

Score:1917 Evalue:0

Accession:JF327464.1

Alignment

[Curtobacterium] plantarum strain CL63 16S ribosomal RN..

Score:1917 Evalue:0

Accession:NR\_104943.1

Alignment

Uncultured Enterobacter sp. gene for 16S rRNA, partial ..

Score:1917 Evalue:0

Accession:AB476621.1

Alignment

Pantoea sp. II\_Gauze\_W\_10\_17 16S ribosomal RNA gene, pa..

Score:1917 Evalue:0

Accession:FJ267564.1

Alignment

Pantoea sp. Wd5760 16S ribosomal RNA gene, partial sequ..

Score:1917 Evalue:0

Accession:EU828365.1

Alignment

Enterobacter agglomerans strain A57 16S ribosomal RNA, ..

Score:1917 Evalue:0

Accession:AF130927.2

Alignment

Pantoea agglomerans 16S ribosomal RNA gene, partial seq..

Score:1917 Evalue:0

Accession:EU598802.1

Alignment

Pantoea agglomerans partial 16S rRNA gene, strain EPS12..

Score:1917 Evalue:0

Accession:AJ583011.1

Alignment

Pantoea agglomerans strain new\*16 16S ribosomal RNA gen..

Score:1917 Evalue:0

Accession:AF130895.1

Alignment

Pantoea agglomerans partial 16S rRNA gene, isolate D3-B..

Score:1916 Evalue:0

Accession:FR873495.1

Alignment

Enterobacter agglomerans strain A65 16S ribosomal RNA, ..

Score:1916 Evalue:0

Accession:AF130934.2

Alignment

Pantoea sp. strain DIB76BC6 16S ribosomal RNA gene, par..

Score:1914 Evalue:0

Accession:MF962590.1

Alignment

Pantoea agglomerans strain CPA-2 16S ribosomal RNA gene..

Score:1914 Evalue:0

Accession:FJ611834.1

Alignment

Pantoea agglomerans strain P5WAM 16S ribosomal RNA gene..

Score:1914 Evalue:0

Accession:FJ611822.1

Alignment

Pantoea agglomerans pv. gypsophilae strain ATCC 43348 1..

Score:1914 Evalue:0

Accession:FJ611821.1

Alignment

Pantoea agglomerans strain MK26 16S ribosomal RNA gene,..

Score:1914 Evalue:0

Accession:EU272867.1

Alignment

Pantoea agglomerans strain P2FNa\_1 16S ribosomal RNA ge..

Score:1912 Evalue:0

Accession:KX891515.1

Alignment

Pantoea agglomerans strain CJ-G-R2A5 16S ribosomal RNA ..

Score:1912 Evalue:0

Accession:HM584289.1

Alignment

Pantoea agglomerans strain VA21971 16S ribosomal RNA ge..

Score:1912 Evalue:0

Accession:FJ611833.1

Alignment

Pantoea agglomerans strain GSPB 450 16S ribosomal RNA g..

Score:1912 Evalue:0

Accession:AF373197.1

Alignment

Pantoea agglomerans strain LMG 2565 16S ribosomal RNA g..

Score:1912 Evalue:0

Accession:AF373196.1

Alignment

Enterobacter agglomerans strain A79 16S ribosomal RNA, ..

Score:1912 Evalue:0

Accession:AF130944.1

Alignment

Pantoea agglomerans strain DSM 3493 16S ribosomal RNA g..

Score:1912 Evalue:0

Accession:NR\_041978.1

Alignment

Pantoea agglomerans LMG 2565 16S ribosomal RNA

Score:1912 Evalue:0

Accession:Z96082.1

Alignment

Pantoea agglomerans strain LMG 2595 16S ribosomal RNA g..

Score:1910 Evalue:0

Accession:FJ611835.1

Alignment

## Descriptions

, Reading indexes 1-5, displaying indexes 1-5


Load next setPrevious Match

Sequences producing significant alignments:

Show all columns  of the table presenting sequences producing significant alignments 

Select:AllNone
Selected:0

Alignments
Download

FASTA (complete sequence)

FASTA (aligned sequences)

GenBank (complete sequence)

Hit Table (text)

Hit Table (CSV)

Text

XML

ASN.1

Continue
Cancel

GenBank 
Graphics
Distance tree of results
Multiple alignment
Show/hide columns of the table presenting sequences producing significant alignments 

Available columns

Description  
Max Score  
Total Score  
Coverage  
E-value  
IdentN  
Accession  
Restore Defaults
Ok
Cancel

Sequences producing significant alignments:

| Select for downloading or viewing reports | Description | Max score | Total score | Query cover | E value | Ident | Accession |
| --- | --- | --- | --- | --- | --- | --- | --- |
| 1Select seq MG819218.1 | Pantoea agglomerans strain AL14 16S ribosomal RNA gene, partial sequence | 1923 | 1923 | 99% | 0.0 | 99% | MG819218.1 |
| 2Select seq MG547769.1 | Pantoea agglomerans strain Rk92 16S ribosomal RNA gene, partial sequence | 1923 | 1923 | 99% | 0.0 | 99% | MG547769.1 |
| 3Select seq CP016889.1 | Pantoea agglomerans strain C410P1, complete genome | 1923 | 13320 | 99% | 0.0 | 99% | CP016889.1 |
| 4Select seq KX603479.1 | Uncultured Pantoea sp. clone GJ\_(2)\_7 16S ribosomal RNA gene, partial sequence | 1923 | 1923 | 99% | 0.0 | 99% | KX603479.1 |
| 5Select seq KT075193.1 | Pantoea agglomerans strain SSE 16S ribosomal RNA gene, partial sequence | 1923 | 1923 | 99% | 0.0 | 99% | KT075193.1 |
| 6Select seq KT075171.1 | Pantoea agglomerans strain NSF 16S ribosomal RNA gene, partial sequence | 1923 | 1923 | 99% | 0.0 | 99% | KT075171.1 |
| 7Select seq KT075162.1 | Pantoea agglomerans strain GSB 16S ribosomal RNA gene, partial sequence | 1923 | 1923 | 99% | 0.0 | 99% | KT075162.1 |
| 8Select seq KJ781904.1 | Pantoea agglomerans strain B81 16S ribosomal RNA gene, partial sequence | 1923 | 1923 | 99% | 0.0 | 99% | KJ781904.1 |
| 9Select seq KP099434.1 | Curtobacterium plantarum strain S4 16S ribosomal RNA gene, partial sequence | 1923 | 1923 | 99% | 0.0 | 99% | KP099434.1 |
| 10Select seq KP099433.1 | Curtobacterium plantarum strain S2 16S ribosomal RNA gene, partial sequence | 1923 | 1923 | 99% | 0.0 | 99% | KP099433.1 |
| 11Select seq KF317872.1 | Pantoea agglomerans strain ex2 culture-collection CGMCC:7.103 16S ribosomal RNA gene, partial sequence | 1923 | 1923 | 99% | 0.0 | 99% | KF317872.1 |
| 12Select seq KF003386.1 | Pantoea agglomerans strain ABAC22 16S ribosomal RNA gene, partial sequence | 1923 | 1923 | 99% | 0.0 | 99% | KF003386.1 |
| 13Select seq JX067684.1 | Enterobacteriaceae bacterium SAP770.3 16S ribosomal RNA gene, partial sequence | 1923 | 1923 | 99% | 0.0 | 99% | JX067684.1 |
| 14Select seq JX257179.1 | Pantoea agglomerans strain Z01 16S ribosomal RNA gene, partial sequence | 1923 | 1923 | 99% | 0.0 | 99% | JX257179.1 |
| 15Select seq JX279936.1 | Uncultured Pantoea sp. clone WRFC18 16S ribosomal RNA gene, partial sequence | 1923 | 1923 | 99% | 0.0 | 99% | JX279936.1 |
| 16Select seq FJ593000.1 | Pantoea agglomerans strain SR-1 16S ribosomal RNA gene, partial sequence | 1923 | 1923 | 99% | 0.0 | 99% | FJ593000.1 |
| 17Select seq FJ592996.1 | Pantoea agglomerans strain SN-2 16S ribosomal RNA gene, partial sequence | 1923 | 1923 | 99% | 0.0 | 99% | FJ592996.1 |
| 18Select seq FJ357809.1 | Pantoea agglomerans strain BBPE0103202 16S ribosomal RNA gene, partial sequence | 1923 | 1923 | 99% | 0.0 | 99% | FJ357809.1 |
| 19Select seq EU753130.1 | Marine bacterium MSC106 16S ribosomal RNA gene, partial sequence | 1923 | 1923 | 99% | 0.0 | 99% | EU753130.1 |
| 20Select seq EU047555.1 | Pantoea agglomerans strain TMPSB-P1 16S ribosomal RNA gene, partial sequence | 1923 | 1923 | 99% | 0.0 | 99% | EU047555.1 |
| 21Select seq DQ307452.1 | Pantoea agglomerans strain FL1 16S ribosomal RNA gene, partial sequence | 1923 | 1923 | 99% | 0.0 | 99% | DQ307452.1 |
| 22Select seq JX067654.1 | Enterobacteriaceae bacterium SAP108.1 16S ribosomal RNA gene, partial sequence | 1919 | 1919 | 99% | 0.0 | 99% | JX067654.1 |
| 23Select seq KT779903.1 | Pantoea agglomerans strain CCRI01 16S ribosomal RNA gene, partial sequence | 1917 | 1917 | 99% | 0.0 | 99% | KT779903.1 |
| 24Select seq KT580675.1 | Pantoea sp. CanR-85 16S ribosomal RNA gene, partial sequence | 1917 | 1917 | 99% | 0.0 | 99% | KT580675.1 |
| 25Select seq LC007922.1 | Enterobacteriaceae bacterium X1/SB37 gene for 16S ribosomal RNA, partial sequence, strain: X1/SB37 | 1917 | 1917 | 99% | 0.0 | 99% | LC007922.1 |
| 26Select seq KF465834.1 | Pantoea agglomerans strain P19 16S ribosomal RNA gene, partial sequence | 1917 | 1917 | 99% | 0.0 | 99% | KF465834.1 |
| 27Select seq KC178591.1 | Pantoea agglomerans strain Cl-17 16S ribosomal RNA gene, partial sequence | 1917 | 1917 | 99% | 0.0 | 99% | KC178591.1 |
| 28Select seq JX067721.1 | Enterobacteriaceae bacterium SAP952.2 16S ribosomal RNA gene, partial sequence | 1917 | 1917 | 99% | 0.0 | 99% | JX067721.1 |
| 29Select seq JX294750.1 | Uncultured Pantoea sp. clone WRFC196 16S ribosomal RNA gene, partial sequence | 1917 | 1917 | 99% | 0.0 | 99% | JX294750.1 |
| 30Select seq JQ412064.1 | Pantoea sp. PR 16S ribosomal RNA gene, partial sequence | 1917 | 1917 | 99% | 0.0 | 99% | JQ412064.1 |
| 31Select seq JF327484.1 | Pantoea agglomerans strain KB64 16S ribosomal RNA gene, partial sequence | 1917 | 1917 | 99% | 0.0 | 99% | JF327484.1 |
| 32Select seq JF327464.1 | Pantoea agglomerans strain KB38 16S ribosomal RNA gene, partial sequence | 1917 | 1917 | 99% | 0.0 | 99% | JF327464.1 |
| 33Select seq NR\_104943.1 | [Curtobacterium] plantarum strain CL63 16S ribosomal RNA, partial sequence | 1917 | 1917 | 99% | 0.0 | 99% | NR\_104943.1 |
| 34Select seq AB476621.1 | Uncultured Enterobacter sp. gene for 16S rRNA, partial sequence, clone: B16\_RoGB | 1917 | 1917 | 99% | 0.0 | 99% | AB476621.1 |
| 35Select seq FJ267564.1 | Pantoea sp. II\_Gauze\_W\_10\_17 16S ribosomal RNA gene, partial sequence | 1917 | 1917 | 99% | 0.0 | 99% | FJ267564.1 |
| 36Select seq EU828365.1 | Pantoea sp. Wd5760 16S ribosomal RNA gene, partial sequence | 1917 | 1917 | 99% | 0.0 | 99% | EU828365.1 |
| 37Select seq AF130927.2 | Enterobacter agglomerans strain A57 16S ribosomal RNA, partial sequence | 1917 | 1917 | 99% | 0.0 | 99% | AF130927.2 |
| 38Select seq EU598802.1 | Pantoea agglomerans 16S ribosomal RNA gene, partial sequence | 1917 | 1917 | 99% | 0.0 | 99% | EU598802.1 |
| 39Select seq AJ583011.1 | Pantoea agglomerans partial 16S rRNA gene, strain EPS125 | 1917 | 1917 | 99% | 0.0 | 99% | AJ583011.1 |
| 40Select seq AF130895.1 | Pantoea agglomerans strain new\*16 16S ribosomal RNA gene, partial sequence | 1917 | 1917 | 99% | 0.0 | 99% | AF130895.1 |
| 41Select seq FR873495.1 | Pantoea agglomerans partial 16S rRNA gene, isolate D3-B-P8ASC2009 | 1916 | 1916 | 99% | 0.0 | 99% | FR873495.1 |
| 42Select seq AF130934.2 | Enterobacter agglomerans strain A65 16S ribosomal RNA, partial sequence | 1916 | 1916 | 99% | 0.0 | 99% | AF130934.2 |
| 43Select seq MF962590.1 | Pantoea sp. strain DIB76BC6 16S ribosomal RNA gene, partial sequence | 1914 | 1914 | 98% | 0.0 | 99% | MF962590.1 |
| 44Select seq FJ611834.1 | Pantoea agglomerans strain CPA-2 16S ribosomal RNA gene, partial sequence | 1914 | 1914 | 99% | 0.0 | 99% | FJ611834.1 |
| 45Select seq FJ611822.1 | Pantoea agglomerans strain P5WAM 16S ribosomal RNA gene, partial sequence | 1914 | 1914 | 99% | 0.0 | 99% | FJ611822.1 |
| 46Select seq FJ611821.1 | Pantoea agglomerans pv. gypsophilae strain ATCC 43348 16S ribosomal RNA gene, partial sequence | 1914 | 1914 | 99% | 0.0 | 99% | FJ611821.1 |
| 47Select seq EU272867.1 | Pantoea agglomerans strain MK26 16S ribosomal RNA gene, partial sequence | 1914 | 1914 | 99% | 0.0 | 99% | EU272867.1 |
| 48Select seq KX891515.1 | Pantoea agglomerans strain P2FNa\_1 16S ribosomal RNA gene, partial sequence | 1912 | 1912 | 99% | 0.0 | 99% | KX891515.1 |
| 49Select seq HM584289.1 | Pantoea agglomerans strain CJ-G-R2A5 16S ribosomal RNA gene, partial sequence | 1912 | 1912 | 99% | 0.0 | 99% | HM584289.1 |
| 50Select seq FJ611833.1 | Pantoea agglomerans strain VA21971 16S ribosomal RNA gene, partial sequence | 1912 | 1912 | 99% | 0.0 | 99% | FJ611833.1 |
| 51Select seq AF373197.1 | Pantoea agglomerans strain GSPB 450 16S ribosomal RNA gene, partial sequence | 1912 | 1912 | 99% | 0.0 | 99% | AF373197.1 |
| 52Select seq AF373196.1 | Pantoea agglomerans strain LMG 2565 16S ribosomal RNA gene, partial sequence | 1912 | 1912 | 99% | 0.0 | 99% | AF373196.1 |
| 53Select seq AF130944.1 | Enterobacter agglomerans strain A79 16S ribosomal RNA, partial sequence | 1912 | 1912 | 99% | 0.0 | 99% | AF130944.1 |
| 54Select seq NR\_041978.1 | Pantoea agglomerans strain DSM 3493 16S ribosomal RNA gene, partial sequence | 1912 | 1912 | 99% | 0.0 | 99% | NR\_041978.1 |
| 55Select seq Z96082.1 | Pantoea agglomerans LMG 2565 16S ribosomal RNA | 1912 | 1912 | 99% | 0.0 | 99% | Z96082.1 |
| 56Select seq FJ611835.1 | Pantoea agglomerans strain LMG 2595 16S ribosomal RNA gene, partial sequence | 1910 | 1910 | 99% | 0.0 | 99% | FJ611835.1 |
| 57Select seq DQ311006.1 | Pantoea sp. FL9 16S ribosomal RNA gene, partial sequence | 1908 | 1908 | 99% | 0.0 | 99% | DQ311006.1 |
| 58Select seq AJ583835.1 | Pantoea agglomerans partial 16S rRNA gene, strain Pa850T | 1908 | 1908 | 99% | 0.0 | 99% | AJ583835.1 |
| 59Select seq KY357288.1 | Pantoea agglomerans strain ACBP2 16S ribosomal RNA gene, partial sequence | 1906 | 1906 | 99% | 0.0 | 99% | KY357288.1 |
| 60Select seq KY357286.1 | Pantoea agglomerans strain ACBC2 16S ribosomal RNA gene, partial sequence | 1906 | 1906 | 99% | 0.0 | 99% | KY357286.1 |
| 61Select seq KU258277.1 | Pantoea agglomerans strain BL26 16S ribosomal RNA gene, partial sequence | 1906 | 1906 | 99% | 0.0 | 99% | KU258277.1 |
| 62Select seq KT361120.1 | Curtobacterium sp. F118 16S ribosomal RNA gene, partial sequence | 1906 | 1906 | 98% | 0.0 | 99% | KT361120.1 |
| 63Select seq KT276390.1 | Pantoea sp. sw0106-22 16S ribosomal RNA gene, partial sequence | 1906 | 1906 | 99% | 0.0 | 99% | KT276390.1 |
| 64Select seq KT075184.1 | Pantoea agglomerans strain QSC 16S ribosomal RNA gene, partial sequence | 1906 | 1906 | 99% | 0.0 | 99% | KT075184.1 |
| 65Select seq KT075163.1 | Pantoea agglomerans strain GSC 16S ribosomal RNA gene, partial sequence | 1906 | 1906 | 99% | 0.0 | 99% | KT075163.1 |
| 66Select seq KT029485.1 | Uncultured bacterium clone 22-24 16S ribosomal RNA gene, partial sequence | 1906 | 1906 | 99% | 0.0 | 99% | KT029485.1 |
| 67Select seq KP279978.1 | Curtobacterium plantarum strain NS1 16S ribosomal RNA gene, partial sequence | 1906 | 1906 | 99% | 0.0 | 99% | KP279978.1 |
| 68Select seq KP279964.1 | Curtobacterium plantarum strain KS1 16S ribosomal RNA gene, partial sequence | 1906 | 1906 | 99% | 0.0 | 99% | KP279964.1 |
| 69Select seq KF580857.1 | Pantoea agglomerans strain WK-442s 16S ribosomal RNA gene, partial sequence | 1906 | 1906 | 99% | 0.0 | 99% | KF580857.1 |
| 70Select seq KF153221.1 | Pantoea sp. E100 16S ribosomal RNA gene, partial sequence | 1906 | 1906 | 99% | 0.0 | 99% | KF153221.1 |
| 71Select seq KF003418.1 | Pantoea agglomerans strain ABAC77 16S ribosomal RNA gene, partial sequence | 1906 | 1906 | 99% | 0.0 | 99% | KF003418.1 |
| 72Select seq DQ307454.1 | Pantoea agglomerans strain SC1 16S ribosomal RNA gene, partial sequence | 1906 | 1906 | 99% | 0.0 | 99% | DQ307454.1 |
| 73Select seq DQ531643.1 | Pantoea sp. 2-2-SS-001 16S ribosomal RNA gene, partial sequence | 1906 | 1906 | 99% | 0.0 | 99% | DQ531643.1 |
| 74Select seq AB004757.2 | Pantoea agglomerans gene for 16S ribosomal RNA, partial sequence | 1906 | 1906 | 99% | 0.0 | 99% | AB004757.2 |
| 75Select seq JX279938.1 | Uncultured Pantoea sp. clone WRFC33 16S ribosomal RNA gene, partial sequence | 1905 | 1905 | 99% | 0.0 | 99% | JX279938.1 |
| 76Select seq AB523724.1 | Pantoea sp. NES2 gene for 16S rRNA, partial sequence | 1905 | 1905 | 99% | 0.0 | 99% | AB523724.1 |
| 77Select seq AF130952.2 | Enterobacter agglomerans strain A92 16S ribosomal RNA, partial sequence | 1905 | 1905 | 99% | 0.0 | 99% | AF130952.2 |
| 78Select seq NR\_114735.1 | Pantoea agglomerans strain NCTC9381 16S ribosomal RNA gene, partial sequence | 1905 | 1905 | 99% | 0.0 | 99% | NR\_114735.1 |
| 79Select seq AF130924.1 | Enterobacter agglomerans strain A54 16S ribosomal RNA, partial sequence | 1905 | 1905 | 99% | 0.0 | 99% | AF130924.1 |
| 80Select seq U80183.1 | Erwinia milletiae 16S ribosomal RNA gene, partial sequence | 1905 | 1905 | 99% | 0.0 | 99% | U80183.1 |
| 81Select seq LT161877.1 | Pantoea sp. Y-38 partial 16S rRNA gene, strain YK1, isolate Y-38 | 1903 | 1903 | 99% | 0.0 | 99% | LT161877.1 |
| 82Select seq FJ357814.1 | Pantoea agglomerans strain CUETM8553 16S ribosomal RNA gene, partial sequence | 1903 | 1903 | 99% | 0.0 | 99% | FJ357814.1 |
| 83Select seq AF130930.1 | Enterobacter agglomerans strain A60 16S ribosomal RNA, partial sequence | 1903 | 1903 | 99% | 0.0 | 99% | AF130930.1 |
| 84Select seq CP014129.2 | Pantoea vagans strain FDAARGOS\_160 chromosome, complete genome | 1901 | 13226 | 99% | 0.0 | 99% | CP014129.2 |
| 85Select seq MG517434.1 | Pantoea agglomerans strain PA3432CH 16S ribosomal RNA gene, partial sequence | 1901 | 1901 | 99% | 0.0 | 99% | MG517434.1 |
| 86Select seq KX891501.1 | Pantoea agglomerans strain P1FNd\_1 16S ribosomal RNA gene, partial sequence | 1901 | 1901 | 99% | 0.0 | 99% | KX891501.1 |
| 87Select seq MF767519.1 | Pantoea agglomerans strain 62 16S ribosomal RNA gene, partial sequence | 1901 | 1901 | 99% | 0.0 | 99% | MF767519.1 |
| 88Select seq KY423868.1 | Pantoea sp. strain PYR16 16S ribosomal RNA gene, partial sequence | 1901 | 1901 | 99% | 0.0 | 99% | KY423868.1 |
| 89Select seq KX603481.1 | Uncultured Pantoea sp. clone GJ\_(2)\_73 16S ribosomal RNA gene, partial sequence | 1901 | 1901 | 99% | 0.0 | 99% | KX603481.1 |
| 90Select seq KT075169.1 | Pantoea agglomerans strain NSD 16S ribosomal RNA gene, partial sequence | 1901 | 1901 | 99% | 0.0 | 99% | KT075169.1 |
| 91Select seq KM586992.1 | Pantoea sp. AnA5.1 16S ribosomal RNA gene, partial sequence | 1901 | 1901 | 97% | 0.0 | 99% | KM586992.1 |
| 92Select seq LC007878.1 | Enterobacteriaceae bacterium X1/SB52 gene for 16S ribosomal RNA, partial sequence, strain: X1/SB52 | 1901 | 1901 | 99% | 0.0 | 99% | LC007878.1 |
| 93Select seq LC007874.1 | Enterobacteriaceae bacterium X1/SB44 gene for 16S ribosomal RNA, partial sequence, strain: X1/SB44 | 1901 | 1901 | 99% | 0.0 | 99% | LC007874.1 |
| 94Select seq AB934979.1 | Pantoea agglomerans gene for 16S ribosomal RNA, partial sequence | 1901 | 1901 | 97% | 0.0 | 99% | AB934979.1 |
| 95Select seq JX067704.1 | Enterobacteriaceae bacterium SAP822.1 16S ribosomal RNA gene, partial sequence | 1901 | 1901 | 99% | 0.0 | 99% | JX067704.1 |
| 96Select seq DQ365572.1 | Pantoea agglomerans strain JA07 16S ribosomal RNA gene, partial sequence | 1901 | 1901 | 99% | 0.0 | 99% | DQ365572.1 |
| 97Select seq FR821191.1 | Pantoea sp. AB365d partial 16S rRNA gene, isolate AB365d | 1899 | 1899 | 99% | 0.0 | 99% | FR821191.1 |
| 98Select seq KC311262.1 | Pantoea sp. SBPci 16S ribosomal RNA gene, partial sequence | 1893 | 1893 | 99% | 0.0 | 99% | KC311262.1 |
| 99Select seq AF130922.1 | Enterobacter agglomerans strain A51 16S ribosomal RNA, partial sequence | 1890 | 1890 | 99% | 0.0 | 99% | AF130922.1 |
| 100Select seq Z96083.1 | Pantoea agglomerans LMG 2660 16S ribosomal RNA | 1879 | 1879 | 99% | 0.0 | 98% | Z96083.1 |


## Alignments

Loading alignment... for sequences gi|1332982380,gi|1278992521,gi|1057481511,gi|1046779577,gi|902659505 Reading indexes 1-5

Download

FASTA (complete sequence)

FASTA (aligned sequences)

GenBank (complete sequence)

Continue
Cancel

GenBankGraphics

Next
Previous
Descriptions

Pantoea agglomerans strain AL14 16S ribosomal RNA gene, partial sequence

Sequence ID: MG819218.1Length: 1416Number of Matches: 1

Related Information

Range 1: 321 to 1371GenBankGraphics

Next Match
Previous Match
First Match

Alignment statistics for match #1

| Score | Expect | Identities | Gaps | Strand | Frame |
| --- | --- | --- | --- | --- | --- |
| 1923 bits(1041) | 0.0() | 1046/1051(99%) | 0/1051(0%) | Plus/Minus |  |

Features:

```
Query  9     GCCCTCCCGAAGGTTANGCTACCTACTTCTTTTNNANCCCACTCCCATGGTGTGACGGGC  68
             |||||||||||||||| ||||||||||||||||  | |||||||||||||||||||||||
Sbjct  1371  GCCCTCCCGAAGGTTAAGCTACCTACTTCTTTTGCAACCCACTCCCATGGTGTGACGGGC  1312

Query  69    GGTGTGTACAAGGCCCGGGAACGTATTCACCGTGGCATTCTGATCCACGATTACTAGCGA  128
             ||||||||||||||||||||||||||||||||||||||||||||||||||||||||||||
Sbjct  1311  GGTGTGTACAAGGCCCGGGAACGTATTCACCGTGGCATTCTGATCCACGATTACTAGCGA  1252

Query  129   TTCCGACTTCACGGAGTCGAGTTGCAGACTCCGATCCGGACTACGACGCACTTTGTGAGG  188
             ||||||||||||||||||||||||||||||||||||||||||||||||||||||||||||
Sbjct  1251  TTCCGACTTCACGGAGTCGAGTTGCAGACTCCGATCCGGACTACGACGCACTTTGTGAGG  1192

Query  189   TCCGCTTGCTCTCGCGAGGTCGCTTCTCTTTGTATGCGCCATTGTAGCACGTGTGTAGCC  248
             ||||||||||||||||||||||||||||||||||||||||||||||||||||||||||||
Sbjct  1191  TCCGCTTGCTCTCGCGAGGTCGCTTCTCTTTGTATGCGCCATTGTAGCACGTGTGTAGCC  1132

Query  249   CTACTCGTAAGGGCCATGATGACTTGACGTCATCCCCACCTTCCTCCGGTTTATCACCGG  308
             ||||||||||||||||||||||||||||||||||||||||||||||||||||||||||||
Sbjct  1131  CTACTCGTAAGGGCCATGATGACTTGACGTCATCCCCACCTTCCTCCGGTTTATCACCGG  1072

Query  309   CAGTCTCCTTTGAGTTCCCGACCGAATCGCTGGCAACAAAGGATAAGGGTTGCGCTCGTT  368
             ||||||||||||||||||||||||||||||||||||||||||||||||||||||||||||
Sbjct  1071  CAGTCTCCTTTGAGTTCCCGACCGAATCGCTGGCAACAAAGGATAAGGGTTGCGCTCGTT  1012

Query  369   GCGGGACTTAACCCAACATTTCACAACACGAGCTGACGACAGCCATGCAGCACCTGTCTC  428
             ||||||||||||||||||||||||||||||||||||||||||||||||||||||||||||
Sbjct  1011  GCGGGACTTAACCCAACATTTCACAACACGAGCTGACGACAGCCATGCAGCACCTGTCTC  952

Query  429   ACGGTTCCCGAAGGCACTAAGGCATCTCTGCCAAATTCCGTGGATGTCAAGAGTAGGTAA  488
             ||||||||||||||||||||||||||||||||||||||||||||||||||||||||||||
Sbjct  951   ACGGTTCCCGAAGGCACTAAGGCATCTCTGCCAAATTCCGTGGATGTCAAGAGTAGGTAA  892

Query  489   GGTTCTTCGCGTTGCATCGAATTAAACCACATGCTCCACCGCTTGTGCGGGCCCCCGTCA  548
             ||||||||||||||||||||||||||||||||||||||||||||||||||||||||||||
Sbjct  891   GGTTCTTCGCGTTGCATCGAATTAAACCACATGCTCCACCGCTTGTGCGGGCCCCCGTCA  832

Query  549   ATTCATTTGAGTTTTAACCTTGCGGCCGTACTCCCCAGGCGGTCGACTTAACGCGTTAGC  608
             ||||||||||||||||||||||||||||||||||||||||||||||||||||||||||||
Sbjct  831   ATTCATTTGAGTTTTAACCTTGCGGCCGTACTCCCCAGGCGGTCGACTTAACGCGTTAGC  772

Query  609   TCCGGAAGCCACTCCTCAAGGGAACAACCTCCAAGTCGACATCGTTTACGGCGTGGACTA  668
             ||||||||||||||||||||||||||||||||||||||||||||||||||||||||||||
Sbjct  771   TCCGGAAGCCACTCCTCAAGGGAACAACCTCCAAGTCGACATCGTTTACGGCGTGGACTA  712

Query  669   CCAGGGTATCTAATCCTGTTTGCTCCCCACGCTTTCGCACCTGAGCGTCAGTCTTTGTCC  728
             ||||||||||||||||||||||||||||||||||||||||||||||||||||||||||||
Sbjct  711   CCAGGGTATCTAATCCTGTTTGCTCCCCACGCTTTCGCACCTGAGCGTCAGTCTTTGTCC  652

Query  729   AGGGGGCCGCCTTCGCCACCGGTATTCCTCCAGATCTCTACGCATTTCACCGCTACACCT  788
             ||||||||||||||||||||||||||||||||||||||||||||||||||||||||||||
Sbjct  651   AGGGGGCCGCCTTCGCCACCGGTATTCCTCCAGATCTCTACGCATTTCACCGCTACACCT  592

Query  789   GGAATTCTACCCCCCTCTACAAGACTCAAGCCTGCCAGTTTCAAATGCAGTTCCCAGGTT  848
             ||||||||||||||||||||||||||||||||||||||||||||||||||||||||||||
Sbjct  591   GGAATTCTACCCCCCTCTACAAGACTCAAGCCTGCCAGTTTCAAATGCAGTTCCCAGGTT  532

Query  849   AAGCCCGGGGATTTCACATCTGACTTAACAGACCGCCTGCGTGCGCTTTACGCCCAGTAA  908
             ||||||||||||||||||||||||||||||||||||||||||||||||||||||||||||
Sbjct  531   AAGCCCGGGGATTTCACATCTGACTTAACAGACCGCCTGCGTGCGCTTTACGCCCAGTAA  472

Query  909   TTCCGATTAACGCTTGCACCCTCCGTATTACCGCGGCTGCTGGCACGGAGTTAGCCGGTG  968
             ||||||||||||||||||||||||||||||||||||||||||||||||||||||||||||
Sbjct  471   TTCCGATTAACGCTTGCACCCTCCGTATTACCGCGGCTGCTGGCACGGAGTTAGCCGGTG  412

Query  969   CTTCTTCTGCGGGTAACGTCAATCGACAGGGTTATTAACCCCGTCGCCTTCCTCCCCGCT  1028
             ||||||||||||||||||||||||||||||||||||||||||||||||||||||||||||
Sbjct  411   CTTCTTCTGCGGGTAACGTCAATCGACAGGGTTATTAACCCCGTCGCCTTCCTCCCCGCT  352

Query  1029  GAAAGTACTTTACNACCCGAAGGCCTTCTTC  1059
             ||||||||||||| |||||||||||||||||
Sbjct  351   GAAAGTACTTTACAACCCGAAGGCCTTCTTC  321
```

Download

FASTA (complete sequence)

FASTA (aligned sequences)

GenBank (complete sequence)

Continue
Cancel

GenBankGraphics

Next
Previous
Descriptions

Pantoea agglomerans strain Rk92 16S ribosomal RNA gene, partial sequence

Sequence ID: MG547769.1Length: 1395Number of Matches: 1

Related Information

Range 1: 296 to 1346GenBankGraphics

Next Match
Previous Match
First Match

Alignment statistics for match #1

| Score | Expect | Identities | Gaps | Strand | Frame |
| --- | --- | --- | --- | --- | --- |
| 1923 bits(1041) | 0.0() | 1046/1051(99%) | 0/1051(0%) | Plus/Minus |  |

Features:

```
Query  9     GCCCTCCCGAAGGTTANGCTACCTACTTCTTTTNNANCCCACTCCCATGGTGTGACGGGC  68
             |||||||||||||||| ||||||||||||||||  | |||||||||||||||||||||||
Sbjct  1346  GCCCTCCCGAAGGTTAAGCTACCTACTTCTTTTGCAACCCACTCCCATGGTGTGACGGGC  1287

Query  69    GGTGTGTACAAGGCCCGGGAACGTATTCACCGTGGCATTCTGATCCACGATTACTAGCGA  128
             ||||||||||||||||||||||||||||||||||||||||||||||||||||||||||||
Sbjct  1286  GGTGTGTACAAGGCCCGGGAACGTATTCACCGTGGCATTCTGATCCACGATTACTAGCGA  1227

Query  129   TTCCGACTTCACGGAGTCGAGTTGCAGACTCCGATCCGGACTACGACGCACTTTGTGAGG  188
             ||||||||||||||||||||||||||||||||||||||||||||||||||||||||||||
Sbjct  1226  TTCCGACTTCACGGAGTCGAGTTGCAGACTCCGATCCGGACTACGACGCACTTTGTGAGG  1167

Query  189   TCCGCTTGCTCTCGCGAGGTCGCTTCTCTTTGTATGCGCCATTGTAGCACGTGTGTAGCC  248
             ||||||||||||||||||||||||||||||||||||||||||||||||||||||||||||
Sbjct  1166  TCCGCTTGCTCTCGCGAGGTCGCTTCTCTTTGTATGCGCCATTGTAGCACGTGTGTAGCC  1107

Query  249   CTACTCGTAAGGGCCATGATGACTTGACGTCATCCCCACCTTCCTCCGGTTTATCACCGG  308
             ||||||||||||||||||||||||||||||||||||||||||||||||||||||||||||
Sbjct  1106  CTACTCGTAAGGGCCATGATGACTTGACGTCATCCCCACCTTCCTCCGGTTTATCACCGG  1047

Query  309   CAGTCTCCTTTGAGTTCCCGACCGAATCGCTGGCAACAAAGGATAAGGGTTGCGCTCGTT  368
             ||||||||||||||||||||||||||||||||||||||||||||||||||||||||||||
Sbjct  1046  CAGTCTCCTTTGAGTTCCCGACCGAATCGCTGGCAACAAAGGATAAGGGTTGCGCTCGTT  987

Query  369   GCGGGACTTAACCCAACATTTCACAACACGAGCTGACGACAGCCATGCAGCACCTGTCTC  428
             ||||||||||||||||||||||||||||||||||||||||||||||||||||||||||||
Sbjct  986   GCGGGACTTAACCCAACATTTCACAACACGAGCTGACGACAGCCATGCAGCACCTGTCTC  927

Query  429   ACGGTTCCCGAAGGCACTAAGGCATCTCTGCCAAATTCCGTGGATGTCAAGAGTAGGTAA  488
             ||||||||||||||||||||||||||||||||||||||||||||||||||||||||||||
Sbjct  926   ACGGTTCCCGAAGGCACTAAGGCATCTCTGCCAAATTCCGTGGATGTCAAGAGTAGGTAA  867

Query  489   GGTTCTTCGCGTTGCATCGAATTAAACCACATGCTCCACCGCTTGTGCGGGCCCCCGTCA  548
             ||||||||||||||||||||||||||||||||||||||||||||||||||||||||||||
Sbjct  866   GGTTCTTCGCGTTGCATCGAATTAAACCACATGCTCCACCGCTTGTGCGGGCCCCCGTCA  807

Query  549   ATTCATTTGAGTTTTAACCTTGCGGCCGTACTCCCCAGGCGGTCGACTTAACGCGTTAGC  608
             ||||||||||||||||||||||||||||||||||||||||||||||||||||||||||||
Sbjct  806   ATTCATTTGAGTTTTAACCTTGCGGCCGTACTCCCCAGGCGGTCGACTTAACGCGTTAGC  747

Query  609   TCCGGAAGCCACTCCTCAAGGGAACAACCTCCAAGTCGACATCGTTTACGGCGTGGACTA  668
             ||||||||||||||||||||||||||||||||||||||||||||||||||||||||||||
Sbjct  746   TCCGGAAGCCACTCCTCAAGGGAACAACCTCCAAGTCGACATCGTTTACGGCGTGGACTA  687

Query  669   CCAGGGTATCTAATCCTGTTTGCTCCCCACGCTTTCGCACCTGAGCGTCAGTCTTTGTCC  728
             ||||||||||||||||||||||||||||||||||||||||||||||||||||||||||||
Sbjct  686   CCAGGGTATCTAATCCTGTTTGCTCCCCACGCTTTCGCACCTGAGCGTCAGTCTTTGTCC  627

Query  729   AGGGGGCCGCCTTCGCCACCGGTATTCCTCCAGATCTCTACGCATTTCACCGCTACACCT  788
             ||||||||||||||||||||||||||||||||||||||||||||||||||||||||||||
Sbjct  626   AGGGGGCCGCCTTCGCCACCGGTATTCCTCCAGATCTCTACGCATTTCACCGCTACACCT  567

Query  789   GGAATTCTACCCCCCTCTACAAGACTCAAGCCTGCCAGTTTCAAATGCAGTTCCCAGGTT  848
             ||||||||||||||||||||||||||||||||||||||||||||||||||||||||||||
Sbjct  566   GGAATTCTACCCCCCTCTACAAGACTCAAGCCTGCCAGTTTCAAATGCAGTTCCCAGGTT  507

Query  849   AAGCCCGGGGATTTCACATCTGACTTAACAGACCGCCTGCGTGCGCTTTACGCCCAGTAA  908
             ||||||||||||||||||||||||||||||||||||||||||||||||||||||||||||
Sbjct  506   AAGCCCGGGGATTTCACATCTGACTTAACAGACCGCCTGCGTGCGCTTTACGCCCAGTAA  447

Query  909   TTCCGATTAACGCTTGCACCCTCCGTATTACCGCGGCTGCTGGCACGGAGTTAGCCGGTG  968
             ||||||||||||||||||||||||||||||||||||||||||||||||||||||||||||
Sbjct  446   TTCCGATTAACGCTTGCACCCTCCGTATTACCGCGGCTGCTGGCACGGAGTTAGCCGGTG  387

Query  969   CTTCTTCTGCGGGTAACGTCAATCGACAGGGTTATTAACCCCGTCGCCTTCCTCCCCGCT  1028
             ||||||||||||||||||||||||||||||||||||||||||||||||||||||||||||
Sbjct  386   CTTCTTCTGCGGGTAACGTCAATCGACAGGGTTATTAACCCCGTCGCCTTCCTCCCCGCT  327

Query  1029  GAAAGTACTTTACNACCCGAAGGCCTTCTTC  1059
             ||||||||||||| |||||||||||||||||
Sbjct  326   GAAAGTACTTTACAACCCGAAGGCCTTCTTC  296
```

Download

FASTA (complete sequence)

FASTA (aligned sequences)

GenBank (complete sequence)

Continue
Cancel

GenBankGraphics

Sort by:

E value
 Score
Percent identity
Query start position
Subject start position

Next
Previous
Descriptions

Pantoea agglomerans strain C410P1, complete genome

Sequence ID: CP016889.1Length: 4182028Number of Matches: 7

Related Information

Range 1: 1470753 to 1471803GenBankGraphics

Next Match
Previous Match
First Match

Alignment statistics for match #1

| Score | Expect | Identities | Gaps | Strand | Frame |
| --- | --- | --- | --- | --- | --- |
| 1923 bits(1041) | 0.0() | 1046/1051(99%) | 0/1051(0%) | Plus/Plus |  |

Features:

```
Query  9        GCCCTCCCGAAGGTTANGCTACCTACTTCTTTTNNANCCCACTCCCATGGTGTGACGGGC  68
                |||||||||||||||| ||||||||||||||||  | |||||||||||||||||||||||
Sbjct  1470753  GCCCTCCCGAAGGTTAAGCTACCTACTTCTTTTGCAACCCACTCCCATGGTGTGACGGGC  1470812

Query  69       GGTGTGTACAAGGCCCGGGAACGTATTCACCGTGGCATTCTGATCCACGATTACTAGCGA  128
                ||||||||||||||||||||||||||||||||||||||||||||||||||||||||||||
Sbjct  1470813  GGTGTGTACAAGGCCCGGGAACGTATTCACCGTGGCATTCTGATCCACGATTACTAGCGA  1470872

Query  129      TTCCGACTTCACGGAGTCGAGTTGCAGACTCCGATCCGGACTACGACGCACTTTGTGAGG  188
                ||||||||||||||||||||||||||||||||||||||||||||||||||||||||||||
Sbjct  1470873  TTCCGACTTCACGGAGTCGAGTTGCAGACTCCGATCCGGACTACGACGCACTTTGTGAGG  1470932

Query  189      TCCGCTTGCTCTCGCGAGGTCGCTTCTCTTTGTATGCGCCATTGTAGCACGTGTGTAGCC  248
                ||||||||||||||||||||||||||||||||||||||||||||||||||||||||||||
Sbjct  1470933  TCCGCTTGCTCTCGCGAGGTCGCTTCTCTTTGTATGCGCCATTGTAGCACGTGTGTAGCC  1470992

Query  249      CTACTCGTAAGGGCCATGATGACTTGACGTCATCCCCACCTTCCTCCGGTTTATCACCGG  308
                ||||||||||||||||||||||||||||||||||||||||||||||||||||||||||||
Sbjct  1470993  CTACTCGTAAGGGCCATGATGACTTGACGTCATCCCCACCTTCCTCCGGTTTATCACCGG  1471052

Query  309      CAGTCTCCTTTGAGTTCCCGACCGAATCGCTGGCAACAAAGGATAAGGGTTGCGCTCGTT  368
                ||||||||||||||||||||||||||||||||||||||||||||||||||||||||||||
Sbjct  1471053  CAGTCTCCTTTGAGTTCCCGACCGAATCGCTGGCAACAAAGGATAAGGGTTGCGCTCGTT  1471112

Query  369      GCGGGACTTAACCCAACATTTCACAACACGAGCTGACGACAGCCATGCAGCACCTGTCTC  428
                ||||||||||||||||||||||||||||||||||||||||||||||||||||||||||||
Sbjct  1471113  GCGGGACTTAACCCAACATTTCACAACACGAGCTGACGACAGCCATGCAGCACCTGTCTC  1471172

Query  429      ACGGTTCCCGAAGGCACTAAGGCATCTCTGCCAAATTCCGTGGATGTCAAGAGTAGGTAA  488
                ||||||||||||||||||||||||||||||||||||||||||||||||||||||||||||
Sbjct  1471173  ACGGTTCCCGAAGGCACTAAGGCATCTCTGCCAAATTCCGTGGATGTCAAGAGTAGGTAA  1471232

Query  489      GGTTCTTCGCGTTGCATCGAATTAAACCACATGCTCCACCGCTTGTGCGGGCCCCCGTCA  548
                ||||||||||||||||||||||||||||||||||||||||||||||||||||||||||||
Sbjct  1471233  GGTTCTTCGCGTTGCATCGAATTAAACCACATGCTCCACCGCTTGTGCGGGCCCCCGTCA  1471292

Query  549      ATTCATTTGAGTTTTAACCTTGCGGCCGTACTCCCCAGGCGGTCGACTTAACGCGTTAGC  608
                ||||||||||||||||||||||||||||||||||||||||||||||||||||||||||||
Sbjct  1471293  ATTCATTTGAGTTTTAACCTTGCGGCCGTACTCCCCAGGCGGTCGACTTAACGCGTTAGC  1471352

Query  609      TCCGGAAGCCACTCCTCAAGGGAACAACCTCCAAGTCGACATCGTTTACGGCGTGGACTA  668
                ||||||||||||||||||||||||||||||||||||||||||||||||||||||||||||
Sbjct  1471353  TCCGGAAGCCACTCCTCAAGGGAACAACCTCCAAGTCGACATCGTTTACGGCGTGGACTA  1471412

Query  669      CCAGGGTATCTAATCCTGTTTGCTCCCCACGCTTTCGCACCTGAGCGTCAGTCTTTGTCC  728
                ||||||||||||||||||||||||||||||||||||||||||||||||||||||||||||
Sbjct  1471413  CCAGGGTATCTAATCCTGTTTGCTCCCCACGCTTTCGCACCTGAGCGTCAGTCTTTGTCC  1471472

Query  729      AGGGGGCCGCCTTCGCCACCGGTATTCCTCCAGATCTCTACGCATTTCACCGCTACACCT  788
                ||||||||||||||||||||||||||||||||||||||||||||||||||||||||||||
Sbjct  1471473  AGGGGGCCGCCTTCGCCACCGGTATTCCTCCAGATCTCTACGCATTTCACCGCTACACCT  1471532

Query  789      GGAATTCTACCCCCCTCTACAAGACTCAAGCCTGCCAGTTTCAAATGCAGTTCCCAGGTT  848
                ||||||||||||||||||||||||||||||||||||||||||||||||||||||||||||
Sbjct  1471533  GGAATTCTACCCCCCTCTACAAGACTCAAGCCTGCCAGTTTCAAATGCAGTTCCCAGGTT  1471592

Query  849      AAGCCCGGGGATTTCACATCTGACTTAACAGACCGCCTGCGTGCGCTTTACGCCCAGTAA  908
                ||||||||||||||||||||||||||||||||||||||||||||||||||||||||||||
Sbjct  1471593  AAGCCCGGGGATTTCACATCTGACTTAACAGACCGCCTGCGTGCGCTTTACGCCCAGTAA  1471652

Query  909      TTCCGATTAACGCTTGCACCCTCCGTATTACCGCGGCTGCTGGCACGGAGTTAGCCGGTG  968
                ||||||||||||||||||||||||||||||||||||||||||||||||||||||||||||
Sbjct  1471653  TTCCGATTAACGCTTGCACCCTCCGTATTACCGCGGCTGCTGGCACGGAGTTAGCCGGTG  1471712

Query  969      CTTCTTCTGCGGGTAACGTCAATCGACAGGGTTATTAACCCCGTCGCCTTCCTCCCCGCT  1028
                ||||||||||||||||||||||||||||||||||||||||||||||||||||||||||||
Sbjct  1471713  CTTCTTCTGCGGGTAACGTCAATCGACAGGGTTATTAACCCCGTCGCCTTCCTCCCCGCT  1471772

Query  1029     GAAAGTACTTTACNACCCGAAGGCCTTCTTC  1059
                ||||||||||||| |||||||||||||||||
Sbjct  1471773  GAAAGTACTTTACAACCCGAAGGCCTTCTTC  1471803
```

Range 2: 1718519 to 1719569GenBankGraphics

Next Match
Previous Match
First Match

Alignment statistics for match #2

| Score | Expect | Identities | Gaps | Strand | Frame |
| --- | --- | --- | --- | --- | --- |
| 1923 bits(1041) | 0.0() | 1046/1051(99%) | 0/1051(0%) | Plus/Minus |  |

Features:

```
Query  9        GCCCTCCCGAAGGTTANGCTACCTACTTCTTTTNNANCCCACTCCCATGGTGTGACGGGC  68
                |||||||||||||||| ||||||||||||||||  | |||||||||||||||||||||||
Sbjct  1719569  GCCCTCCCGAAGGTTAAGCTACCTACTTCTTTTGCAACCCACTCCCATGGTGTGACGGGC  1719510

Query  69       GGTGTGTACAAGGCCCGGGAACGTATTCACCGTGGCATTCTGATCCACGATTACTAGCGA  128
                ||||||||||||||||||||||||||||||||||||||||||||||||||||||||||||
Sbjct  1719509  GGTGTGTACAAGGCCCGGGAACGTATTCACCGTGGCATTCTGATCCACGATTACTAGCGA  1719450

Query  129      TTCCGACTTCACGGAGTCGAGTTGCAGACTCCGATCCGGACTACGACGCACTTTGTGAGG  188
                ||||||||||||||||||||||||||||||||||||||||||||||||||||||||||||
Sbjct  1719449  TTCCGACTTCACGGAGTCGAGTTGCAGACTCCGATCCGGACTACGACGCACTTTGTGAGG  1719390

Query  189      TCCGCTTGCTCTCGCGAGGTCGCTTCTCTTTGTATGCGCCATTGTAGCACGTGTGTAGCC  248
                ||||||||||||||||||||||||||||||||||||||||||||||||||||||||||||
Sbjct  1719389  TCCGCTTGCTCTCGCGAGGTCGCTTCTCTTTGTATGCGCCATTGTAGCACGTGTGTAGCC  1719330

Query  249      CTACTCGTAAGGGCCATGATGACTTGACGTCATCCCCACCTTCCTCCGGTTTATCACCGG  308
                ||||||||||||||||||||||||||||||||||||||||||||||||||||||||||||
Sbjct  1719329  CTACTCGTAAGGGCCATGATGACTTGACGTCATCCCCACCTTCCTCCGGTTTATCACCGG  1719270

Query  309      CAGTCTCCTTTGAGTTCCCGACCGAATCGCTGGCAACAAAGGATAAGGGTTGCGCTCGTT  368
                ||||||||||||||||||||||||||||||||||||||||||||||||||||||||||||
Sbjct  1719269  CAGTCTCCTTTGAGTTCCCGACCGAATCGCTGGCAACAAAGGATAAGGGTTGCGCTCGTT  1719210

Query  369      GCGGGACTTAACCCAACATTTCACAACACGAGCTGACGACAGCCATGCAGCACCTGTCTC  428
                ||||||||||||||||||||||||||||||||||||||||||||||||||||||||||||
Sbjct  1719209  GCGGGACTTAACCCAACATTTCACAACACGAGCTGACGACAGCCATGCAGCACCTGTCTC  1719150

Query  429      ACGGTTCCCGAAGGCACTAAGGCATCTCTGCCAAATTCCGTGGATGTCAAGAGTAGGTAA  488
                ||||||||||||||||||||||||||||||||||||||||||||||||||||||||||||
Sbjct  1719149  ACGGTTCCCGAAGGCACTAAGGCATCTCTGCCAAATTCCGTGGATGTCAAGAGTAGGTAA  1719090

Query  489      GGTTCTTCGCGTTGCATCGAATTAAACCACATGCTCCACCGCTTGTGCGGGCCCCCGTCA  548
                ||||||||||||||||||||||||||||||||||||||||||||||||||||||||||||
Sbjct  1719089  GGTTCTTCGCGTTGCATCGAATTAAACCACATGCTCCACCGCTTGTGCGGGCCCCCGTCA  1719030

Query  549      ATTCATTTGAGTTTTAACCTTGCGGCCGTACTCCCCAGGCGGTCGACTTAACGCGTTAGC  608
                ||||||||||||||||||||||||||||||||||||||||||||||||||||||||||||
Sbjct  1719029  ATTCATTTGAGTTTTAACCTTGCGGCCGTACTCCCCAGGCGGTCGACTTAACGCGTTAGC  1718970

Query  609      TCCGGAAGCCACTCCTCAAGGGAACAACCTCCAAGTCGACATCGTTTACGGCGTGGACTA  668
                ||||||||||||||||||||||||||||||||||||||||||||||||||||||||||||
Sbjct  1718969  TCCGGAAGCCACTCCTCAAGGGAACAACCTCCAAGTCGACATCGTTTACGGCGTGGACTA  1718910

Query  669      CCAGGGTATCTAATCCTGTTTGCTCCCCACGCTTTCGCACCTGAGCGTCAGTCTTTGTCC  728
                ||||||||||||||||||||||||||||||||||||||||||||||||||||||||||||
Sbjct  1718909  CCAGGGTATCTAATCCTGTTTGCTCCCCACGCTTTCGCACCTGAGCGTCAGTCTTTGTCC  1718850

Query  729      AGGGGGCCGCCTTCGCCACCGGTATTCCTCCAGATCTCTACGCATTTCACCGCTACACCT  788
                ||||||||||||||||||||||||||||||||||||||||||||||||||||||||||||
Sbjct  1718849  AGGGGGCCGCCTTCGCCACCGGTATTCCTCCAGATCTCTACGCATTTCACCGCTACACCT  1718790

Query  789      GGAATTCTACCCCCCTCTACAAGACTCAAGCCTGCCAGTTTCAAATGCAGTTCCCAGGTT  848
                ||||||||||||||||||||||||||||||||||||||||||||||||||||||||||||
Sbjct  1718789  GGAATTCTACCCCCCTCTACAAGACTCAAGCCTGCCAGTTTCAAATGCAGTTCCCAGGTT  1718730

Query  849      AAGCCCGGGGATTTCACATCTGACTTAACAGACCGCCTGCGTGCGCTTTACGCCCAGTAA  908
                ||||||||||||||||||||||||||||||||||||||||||||||||||||||||||||
Sbjct  1718729  AAGCCCGGGGATTTCACATCTGACTTAACAGACCGCCTGCGTGCGCTTTACGCCCAGTAA  1718670

Query  909      TTCCGATTAACGCTTGCACCCTCCGTATTACCGCGGCTGCTGGCACGGAGTTAGCCGGTG  968
                ||||||||||||||||||||||||||||||||||||||||||||||||||||||||||||
Sbjct  1718669  TTCCGATTAACGCTTGCACCCTCCGTATTACCGCGGCTGCTGGCACGGAGTTAGCCGGTG  1718610

Query  969      CTTCTTCTGCGGGTAACGTCAATCGACAGGGTTATTAACCCCGTCGCCTTCCTCCCCGCT  1028
                ||||||||||||||||||||||||||||||||||||||||||||||||||||||||||||
Sbjct  1718609  CTTCTTCTGCGGGTAACGTCAATCGACAGGGTTATTAACCCCGTCGCCTTCCTCCCCGCT  1718550

Query  1029     GAAAGTACTTTACNACCCGAAGGCCTTCTTC  1059
                ||||||||||||| |||||||||||||||||
Sbjct  1718549  GAAAGTACTTTACAACCCGAAGGCCTTCTTC  1718519
```

Range 3: 1755425 to 1756475GenBankGraphics

Next Match
Previous Match
First Match

Alignment statistics for match #3

| Score | Expect | Identities | Gaps | Strand | Frame |
| --- | --- | --- | --- | --- | --- |
| 1923 bits(1041) | 0.0() | 1046/1051(99%) | 0/1051(0%) | Plus/Minus |  |

Features:

```
Query  9        GCCCTCCCGAAGGTTANGCTACCTACTTCTTTTNNANCCCACTCCCATGGTGTGACGGGC  68
                |||||||||||||||| ||||||||||||||||  | |||||||||||||||||||||||
Sbjct  1756475  GCCCTCCCGAAGGTTAAGCTACCTACTTCTTTTGCAACCCACTCCCATGGTGTGACGGGC  1756416

Query  69       GGTGTGTACAAGGCCCGGGAACGTATTCACCGTGGCATTCTGATCCACGATTACTAGCGA  128
                ||||||||||||||||||||||||||||||||||||||||||||||||||||||||||||
Sbjct  1756415  GGTGTGTACAAGGCCCGGGAACGTATTCACCGTGGCATTCTGATCCACGATTACTAGCGA  1756356

Query  129      TTCCGACTTCACGGAGTCGAGTTGCAGACTCCGATCCGGACTACGACGCACTTTGTGAGG  188
                ||||||||||||||||||||||||||||||||||||||||||||||||||||||||||||
Sbjct  1756355  TTCCGACTTCACGGAGTCGAGTTGCAGACTCCGATCCGGACTACGACGCACTTTGTGAGG  1756296

Query  189      TCCGCTTGCTCTCGCGAGGTCGCTTCTCTTTGTATGCGCCATTGTAGCACGTGTGTAGCC  248
                ||||||||||||||||||||||||||||||||||||||||||||||||||||||||||||
Sbjct  1756295  TCCGCTTGCTCTCGCGAGGTCGCTTCTCTTTGTATGCGCCATTGTAGCACGTGTGTAGCC  1756236

Query  249      CTACTCGTAAGGGCCATGATGACTTGACGTCATCCCCACCTTCCTCCGGTTTATCACCGG  308
                ||||||||||||||||||||||||||||||||||||||||||||||||||||||||||||
Sbjct  1756235  CTACTCGTAAGGGCCATGATGACTTGACGTCATCCCCACCTTCCTCCGGTTTATCACCGG  1756176

Query  309      CAGTCTCCTTTGAGTTCCCGACCGAATCGCTGGCAACAAAGGATAAGGGTTGCGCTCGTT  368
                ||||||||||||||||||||||||||||||||||||||||||||||||||||||||||||
Sbjct  1756175  CAGTCTCCTTTGAGTTCCCGACCGAATCGCTGGCAACAAAGGATAAGGGTTGCGCTCGTT  1756116

Query  369      GCGGGACTTAACCCAACATTTCACAACACGAGCTGACGACAGCCATGCAGCACCTGTCTC  428
                ||||||||||||||||||||||||||||||||||||||||||||||||||||||||||||
Sbjct  1756115  GCGGGACTTAACCCAACATTTCACAACACGAGCTGACGACAGCCATGCAGCACCTGTCTC  1756056

Query  429      ACGGTTCCCGAAGGCACTAAGGCATCTCTGCCAAATTCCGTGGATGTCAAGAGTAGGTAA  488
                ||||||||||||||||||||||||||||||||||||||||||||||||||||||||||||
Sbjct  1756055  ACGGTTCCCGAAGGCACTAAGGCATCTCTGCCAAATTCCGTGGATGTCAAGAGTAGGTAA  1755996

Query  489      GGTTCTTCGCGTTGCATCGAATTAAACCACATGCTCCACCGCTTGTGCGGGCCCCCGTCA  548
                ||||||||||||||||||||||||||||||||||||||||||||||||||||||||||||
Sbjct  1755995  GGTTCTTCGCGTTGCATCGAATTAAACCACATGCTCCACCGCTTGTGCGGGCCCCCGTCA  1755936

Query  549      ATTCATTTGAGTTTTAACCTTGCGGCCGTACTCCCCAGGCGGTCGACTTAACGCGTTAGC  608
                ||||||||||||||||||||||||||||||||||||||||||||||||||||||||||||
Sbjct  1755935  ATTCATTTGAGTTTTAACCTTGCGGCCGTACTCCCCAGGCGGTCGACTTAACGCGTTAGC  1755876

Query  609      TCCGGAAGCCACTCCTCAAGGGAACAACCTCCAAGTCGACATCGTTTACGGCGTGGACTA  668
                ||||||||||||||||||||||||||||||||||||||||||||||||||||||||||||
Sbjct  1755875  TCCGGAAGCCACTCCTCAAGGGAACAACCTCCAAGTCGACATCGTTTACGGCGTGGACTA  1755816

Query  669      CCAGGGTATCTAATCCTGTTTGCTCCCCACGCTTTCGCACCTGAGCGTCAGTCTTTGTCC  728
                ||||||||||||||||||||||||||||||||||||||||||||||||||||||||||||
Sbjct  1755815  CCAGGGTATCTAATCCTGTTTGCTCCCCACGCTTTCGCACCTGAGCGTCAGTCTTTGTCC  1755756

Query  729      AGGGGGCCGCCTTCGCCACCGGTATTCCTCCAGATCTCTACGCATTTCACCGCTACACCT  788
                ||||||||||||||||||||||||||||||||||||||||||||||||||||||||||||
Sbjct  1755755  AGGGGGCCGCCTTCGCCACCGGTATTCCTCCAGATCTCTACGCATTTCACCGCTACACCT  1755696

Query  789      GGAATTCTACCCCCCTCTACAAGACTCAAGCCTGCCAGTTTCAAATGCAGTTCCCAGGTT  848
                ||||||||||||||||||||||||||||||||||||||||||||||||||||||||||||
Sbjct  1755695  GGAATTCTACCCCCCTCTACAAGACTCAAGCCTGCCAGTTTCAAATGCAGTTCCCAGGTT  1755636

Query  849      AAGCCCGGGGATTTCACATCTGACTTAACAGACCGCCTGCGTGCGCTTTACGCCCAGTAA  908
                ||||||||||||||||||||||||||||||||||||||||||||||||||||||||||||
Sbjct  1755635  AAGCCCGGGGATTTCACATCTGACTTAACAGACCGCCTGCGTGCGCTTTACGCCCAGTAA  1755576

Query  909      TTCCGATTAACGCTTGCACCCTCCGTATTACCGCGGCTGCTGGCACGGAGTTAGCCGGTG  968
                ||||||||||||||||||||||||||||||||||||||||||||||||||||||||||||
Sbjct  1755575  TTCCGATTAACGCTTGCACCCTCCGTATTACCGCGGCTGCTGGCACGGAGTTAGCCGGTG  1755516

Query  969      CTTCTTCTGCGGGTAACGTCAATCGACAGGGTTATTAACCCCGTCGCCTTCCTCCCCGCT  1028
                ||||||||||||||||||||||||||||||||||||||||||||||||||||||||||||
Sbjct  1755515  CTTCTTCTGCGGGTAACGTCAATCGACAGGGTTATTAACCCCGTCGCCTTCCTCCCCGCT  1755456

Query  1029     GAAAGTACTTTACNACCCGAAGGCCTTCTTC  1059
                ||||||||||||| |||||||||||||||||
Sbjct  1755455  GAAAGTACTTTACAACCCGAAGGCCTTCTTC  1755425
```

Range 4: 597173 to 598223GenBankGraphics

Next Match
Previous Match
First Match

Alignment statistics for match #4

| Score | Expect | Identities | Gaps | Strand | Frame |
| --- | --- | --- | --- | --- | --- |
| 1917 bits(1038) | 0.0() | 1045/1051(99%) | 0/1051(0%) | Plus/Plus |  |

Features:

```
Query  9       GCCCTCCCGAAGGTTANGCTACCTACTTCTTTTNNANCCCACTCCCATGGTGTGACGGGC  68
               |||||||||||||||| ||||||||||||||||  | |||||||||||||||||||||||
Sbjct  597173  GCCCTCCCGAAGGTTAAGCTACCTACTTCTTTTGCAACCCACTCCCATGGTGTGACGGGC  597232

Query  69      GGTGTGTACAAGGCCCGGGAACGTATTCACCGTGGCATTCTGATCCACGATTACTAGCGA  128
               ||||||||||||||||||||||||||||||||||||||||||||||||||||||||||||
Sbjct  597233  GGTGTGTACAAGGCCCGGGAACGTATTCACCGTGGCATTCTGATCCACGATTACTAGCGA  597292

Query  129     TTCCGACTTCACGGAGTCGAGTTGCAGACTCCGATCCGGACTACGACGCACTTTGTGAGG  188
               ||||||||||||||||||||||||||||||||||||||||||||||||||||||||||||
Sbjct  597293  TTCCGACTTCACGGAGTCGAGTTGCAGACTCCGATCCGGACTACGACGCACTTTGTGAGG  597352

Query  189     TCCGCTTGCTCTCGCGAGGTCGCTTCTCTTTGTATGCGCCATTGTAGCACGTGTGTAGCC  248
               ||||||||||||||||||||||||||||||||||||||||||||||||||||||||||||
Sbjct  597353  TCCGCTTGCTCTCGCGAGGTCGCTTCTCTTTGTATGCGCCATTGTAGCACGTGTGTAGCC  597412

Query  249     CTACTCGTAAGGGCCATGATGACTTGACGTCATCCCCACCTTCCTCCGGTTTATCACCGG  308
               ||||||||||||||||||||||||||||||||||||||||||||||||||||||||||||
Sbjct  597413  CTACTCGTAAGGGCCATGATGACTTGACGTCATCCCCACCTTCCTCCGGTTTATCACCGG  597472

Query  309     CAGTCTCCTTTGAGTTCCCGACCGAATCGCTGGCAACAAAGGATAAGGGTTGCGCTCGTT  368
               ||||||||||||||||||||||||||||||||||||||||||||||||||||||||||||
Sbjct  597473  CAGTCTCCTTTGAGTTCCCGACCGAATCGCTGGCAACAAAGGATAAGGGTTGCGCTCGTT  597532

Query  369     GCGGGACTTAACCCAACATTTCACAACACGAGCTGACGACAGCCATGCAGCACCTGTCTC  428
               ||||||||||||||||||||||||||||||||||||||||||||||||||||||||||||
Sbjct  597533  GCGGGACTTAACCCAACATTTCACAACACGAGCTGACGACAGCCATGCAGCACCTGTCTC  597592

Query  429     ACGGTTCCCGAAGGCACTAAGGCATCTCTGCCAAATTCCGTGGATGTCAAGAGTAGGTAA  488
               ||||||||||||||||||||||||||||||||||||||||||||||||||||||||||||
Sbjct  597593  ACGGTTCCCGAAGGCACTAAGGCATCTCTGCCAAATTCCGTGGATGTCAAGAGTAGGTAA  597652

Query  489     GGTTCTTCGCGTTGCATCGAATTAAACCACATGCTCCACCGCTTGTGCGGGCCCCCGTCA  548
               ||||||||||||||||||||||||||||||||||||||||||||||||||||||||||||
Sbjct  597653  GGTTCTTCGCGTTGCATCGAATTAAACCACATGCTCCACCGCTTGTGCGGGCCCCCGTCA  597712

Query  549     ATTCATTTGAGTTTTAACCTTGCGGCCGTACTCCCCAGGCGGTCGACTTAACGCGTTAGC  608
               ||||||||||||||||||||||||||||||||||||||||||||||||||||||||||||
Sbjct  597713  ATTCATTTGAGTTTTAACCTTGCGGCCGTACTCCCCAGGCGGTCGACTTAACGCGTTAGC  597772

Query  609     TCCGGAAGCCACTCCTCAAGGGAACAACCTCCAAGTCGACATCGTTTACGGCGTGGACTA  668
               ||||||||||||||||||||||||||||||||||||||||||||||||||||||||||||
Sbjct  597773  TCCGGAAGCCACTCCTCAAGGGAACAACCTCCAAGTCGACATCGTTTACGGCGTGGACTA  597832

Query  669     CCAGGGTATCTAATCCTGTTTGCTCCCCACGCTTTCGCACCTGAGCGTCAGTCTTTGTCC  728
               ||||||||||||||||||||||||||||||||||||||||||||||||||||||||||||
Sbjct  597833  CCAGGGTATCTAATCCTGTTTGCTCCCCACGCTTTCGCACCTGAGCGTCAGTCTTTGTCC  597892

Query  729     AGGGGGCCGCCTTCGCCACCGGTATTCCTCCAGATCTCTACGCATTTCACCGCTACACCT  788
               ||||||||||||||||||||||||||||||||||||||||||||||||||||||||||||
Sbjct  597893  AGGGGGCCGCCTTCGCCACCGGTATTCCTCCAGATCTCTACGCATTTCACCGCTACACCT  597952

Query  789     GGAATTCTACCCCCCTCTACAAGACTCAAGCCTGCCAGTTTCAAATGCAGTTCCCAGGTT  848
               ||||||||||||||||||||||||||||||||||||||||||||||||||||||||||||
Sbjct  597953  GGAATTCTACCCCCCTCTACAAGACTCAAGCCTGCCAGTTTCAAATGCAGTTCCCAGGTT  598012

Query  849     AAGCCCGGGGATTTCACATCTGACTTAACAGACCGCCTGCGTGCGCTTTACGCCCAGTAA  908
               ||||||||||||||||||||||||||||||||||||||||||||||||||||||||||||
Sbjct  598013  AAGCCCGGGGATTTCACATCTGACTTAACAGACCGCCTGCGTGCGCTTTACGCCCAGTAA  598072

Query  909     TTCCGATTAACGCTTGCACCCTCCGTATTACCGCGGCTGCTGGCACGGAGTTAGCCGGTG  968
               ||||||||||||||||||||||||||||||||||||||||||||||||||||||||||||
Sbjct  598073  TTCCGATTAACGCTTGCACCCTCCGTATTACCGCGGCTGCTGGCACGGAGTTAGCCGGTG  598132

Query  969     CTTCTTCTGCGGGTAACGTCAATCGACAGGGTTATTAACCCCGTCGCCTTCCTCCCCGCT  1028
               |||||||||||||||||||||||||||||||||||||||||| |||||||||||||||||
Sbjct  598133  CTTCTTCTGCGGGTAACGTCAATCGACAGGGTTATTAACCCCATCGCCTTCCTCCCCGCT  598192

Query  1029    GAAAGTACTTTACNACCCGAAGGCCTTCTTC  1059
               ||||||||||||| |||||||||||||||||
Sbjct  598193  GAAAGTACTTTACAACCCGAAGGCCTTCTTC  598223
```

Range 5: 1343498 to 1344547GenBankGraphics

Next Match
Previous Match
First Match

Alignment statistics for match #5

| Score | Expect | Identities | Gaps | Strand | Frame |
| --- | --- | --- | --- | --- | --- |
| 1910 bits(1034) | 0.0() | 1044/1051(99%) | 1/1051(0%) | Plus/Plus |  |

Features:

```
Query  9        GCCCTCCCGAAGGTTANGCTACCTACTTCTTTTNNANCCCACTCCCATGGTGTGACGGGC  68
                |||||||||||||||| ||||||||||||||||  | |||||||||||||||||||||||
Sbjct  1343498  GCCCTCCCGAAGGTTAAGCTACCTACTTCTTTTGCAACCCACTCCCATGGTGTGACGGGC  1343557

Query  69       GGTGTGTACAAGGCCCGGGAACGTATTCACCGTGGCATTCTGATCCACGATTACTAGCGA  128
                ||||||||||||||||||||||||||||||||||||||||||||||||||||||||||||
Sbjct  1343558  GGTGTGTACAAGGCCCGGGAACGTATTCACCGTGGCATTCTGATCCACGATTACTAGCGA  1343617

Query  129      TTCCGACTTCACGGAGTCGAGTTGCAGACTCCGATCCGGACTACGACGCACTTTGTGAGG  188
                ||||||||||||||||||||||||||||||||||||||||||||||||||||||||||||
Sbjct  1343618  TTCCGACTTCACGGAGTCGAGTTGCAGACTCCGATCCGGACTACGACGCACTTTGTGAGG  1343677

Query  189      TCCGCTTGCTCTCGCGAGGTCGCTTCTCTTTGTATGCGCCATTGTAGCACGTGTGTAGCC  248
                ||||||||||||||||||||||||||||||||||||||||||||||||||||||||||||
Sbjct  1343678  TCCGCTTGCTCTCGCGAGGTCGCTTCTCTTTGTATGCGCCATTGTAGCACGTGTGTAGCC  1343737

Query  249      CTACTCGTAAGGGCCATGATGACTTGACGTCATCCCCACCTTCCTCCGGTTTATCACCGG  308
                ||||||||||||||||||||||||||||||||||||||||||||||||||||||||||||
Sbjct  1343738  CTACTCGTAAGGGCCATGATGACTTGACGTCATCCCCACCTTCCTCCGGTTTATCACCGG  1343797

Query  309      CAGTCTCCTTTGAGTTCCCGACCGAATCGCTGGCAACAAAGGATAAGGGTTGCGCTCGTT  368
                ||||||||||||||||||||||||||||||||||||||||||||||||||||||||||||
Sbjct  1343798  CAGTCTCCTTTGAGTTCCCGACCGAATCGCTGGCAACAAAGGATAAGGGTTGCGCTCGTT  1343857

Query  369      GCGGGACTTAACCCAACATTTCACAACACGAGCTGACGACAGCCATGCAGCACCTGTCTC  428
                ||||||||||||||||||||||||||||||||||||||||||||||||||||||||||||
Sbjct  1343858  GCGGGACTTAACCCAACATTTCACAACACGAGCTGACGACAGCCATGCAGCACCTGTCTC  1343917

Query  429      ACGGTTCCCGAAGGCACTAAGGCATCTCTGCCAAATTCCGTGGATGTCAAGAGTAGGTAA  488
                ||||||||||||||||||||||||||||||||||||||||||||||||||||||||||||
Sbjct  1343918  ACGGTTCCCGAAGGCACTAAGGCATCTCTGCCAAATTCCGTGGATGTCAAGAGTAGGTAA  1343977

Query  489      GGTTCTTCGCGTTGCATCGAATTAAACCACATGCTCCACCGCTTGTGCGGGCCCCCGTCA  548
                ||||||||||||||||||||||||||||||||||||||||||||||||||||||||||||
Sbjct  1343978  GGTTCTTCGCGTTGCATCGAATTAAACCACATGCTCCACCGCTTGTGCGGGCCCCCGTCA  1344037

Query  549      ATTCATTTGAGTTTTAACCTTGCGGCCGTACTCCCCAGGCGGTCGACTTAACGCGTTAGC  608
                ||||||||||||||||||||||||||||||||||||||||||||||||||||||||||||
Sbjct  1344038  ATTCATTTGAGTTTTAACCTTGCGGCCGTACTCCCCAGGCGGTCGACTTAACGCGTTAGC  1344097

Query  609      TCCGGAAGCCACTCCTCAAGGGAACAACCTCCAAGTCGACATCGTTTACGGCGTGGACTA  668
                ||||||||||||||||||||||||||||||||||||||||||||||||||||||||||||
Sbjct  1344098  TCCGGAAGCCACTCCTCAAGGGAACAACCTCCAAGTCGACATCGTTTACGGCGTGGACTA  1344157

Query  669      CCAGGGTATCTAATCCTGTTTGCTCCCCACGCTTTCGCACCTGAGCGTCAGTCTTTGTCC  728
                ||||||||||||||||||||||||||||||||||||||||||||||||||||||||||||
Sbjct  1344158  CCAGGGTATCTAATCCTGTTTGCTCCCCACGCTTTCGCACCTGAGCGTCAGTCTTTGTCC  1344217

Query  729      AGGGGGCCGCCTTCGCCACCGGTATTCCTCCAGATCTCTACGCATTTCACCGCTACACCT  788
                ||||||||||||||||||||||||||||||||||||||||||||||||||||||||||||
Sbjct  1344218  AGGGGGCCGCCTTCGCCACCGGTATTCCTCCAGATCTCTACGCATTTCACCGCTACACCT  1344277

Query  789      GGAATTCTACCCCCCTCTACAAGACTCAAGCCTGCCAGTTTCAAATGCAGTTCCCAGGTT  848
                ||||||||| ||||||||||||||||||||||||||||||||||||||||||||||||||
Sbjct  1344278  GGAATTCTA-CCCCCTCTACAAGACTCAAGCCTGCCAGTTTCAAATGCAGTTCCCAGGTT  1344336

Query  849      AAGCCCGGGGATTTCACATCTGACTTAACAGACCGCCTGCGTGCGCTTTACGCCCAGTAA  908
                ||||||||||||||||||||||||||||||||||||||||||||||||||||||||||||
Sbjct  1344337  AAGCCCGGGGATTTCACATCTGACTTAACAGACCGCCTGCGTGCGCTTTACGCCCAGTAA  1344396

Query  909      TTCCGATTAACGCTTGCACCCTCCGTATTACCGCGGCTGCTGGCACGGAGTTAGCCGGTG  968
                ||||||||||||||||||||||||||||||||||||||||||||||||||||||||||||
Sbjct  1344397  TTCCGATTAACGCTTGCACCCTCCGTATTACCGCGGCTGCTGGCACGGAGTTAGCCGGTG  1344456

Query  969      CTTCTTCTGCGGGTAACGTCAATCGACAGGGTTATTAACCCCGTCGCCTTCCTCCCCGCT  1028
                |||||||||||||||||||||||||||||||||||||||||| |||||||||||||||||
Sbjct  1344457  CTTCTTCTGCGGGTAACGTCAATCGACAGGGTTATTAACCCCATCGCCTTCCTCCCCGCT  1344516

Query  1029     GAAAGTACTTTACNACCCGAAGGCCTTCTTC  1059
                ||||||||||||| |||||||||||||||||
Sbjct  1344517  GAAAGTACTTTACAACCCGAAGGCCTTCTTC  1344547
```

Range 6: 1071585 to 1072634GenBankGraphics

Next Match
Previous Match
First Match

Alignment statistics for match #6

| Score | Expect | Identities | Gaps | Strand | Frame |
| --- | --- | --- | --- | --- | --- |
| 1860 bits(1007) | 0.0() | 1035/1051(98%) | 1/1051(0%) | Plus/Plus |  |

Features:

```
Query  9        GCCCTCCCGAAGGTTANGCTACCTACTTCTTTTNNANCCCACTCCCATGGTGTGACGGGC  68
                |||||||||||||||| ||||||||||||||||  | |||||||||||||||||||||||
Sbjct  1071585  GCCCTCCCGAAGGTTAAGCTACCTACTTCTTTTGCAACCCACTCCCATGGTGTGACGGGC  1071644

Query  69       GGTGTGTACAAGGCCCGGGAACGTATTCACCGTGGCATTCTGATCCACGATTACTAGCGA  128
                ||||||||||||||||||||||||||||||||||||||||||||||||||||||||||||
Sbjct  1071645  GGTGTGTACAAGGCCCGGGAACGTATTCACCGTGGCATTCTGATCCACGATTACTAGCGA  1071704

Query  129      TTCCGACTTCACGGAGTCGAGTTGCAGACTCCGATCCGGACTACGACGCACTTTGTGAGG  188
                ||||||||||||||||||||||||||||||||||||||||||||||||||||||||||||
Sbjct  1071705  TTCCGACTTCACGGAGTCGAGTTGCAGACTCCGATCCGGACTACGACGCACTTTGTGAGG  1071764

Query  189      TCCGCTTGCTCTCGCGAGGTCGCTTCTCTTTGTATGCGCCATTGTAGCACGTGTGTAGCC  248
                ||||||||||||||||||||||||||||||||||||||||||||||||||||||||||||
Sbjct  1071765  TCCGCTTGCTCTCGCGAGGTCGCTTCTCTTTGTATGCGCCATTGTAGCACGTGTGTAGCC  1071824

Query  249      CTACTCGTAAGGGCCATGATGACTTGACGTCATCCCCACCTTCCTCCGGTTTATCACCGG  308
                ||||||||||||||||||||||||||||||||||||||||||||||||||||||||||||
Sbjct  1071825  CTACTCGTAAGGGCCATGATGACTTGACGTCATCCCCACCTTCCTCCGGTTTATCACCGG  1071884

Query  309      CAGTCTCCTTTGAGTTCCCGACCGAATCGCTGGCAACAAAGGATAAGGGTTGCGCTCGTT  368
                ||||||||||||||||||||||||||||||||||||||||||||||||||||||||||||
Sbjct  1071885  CAGTCTCCTTTGAGTTCCCGACCGAATCGCTGGCAACAAAGGATAAGGGTTGCGCTCGTT  1071944

Query  369      GCGGGACTTAACCCAACATTTCACAACACGAGCTGACGACAGCCATGCAGCACCTGTCTC  428
                ||||||||||||||||||||||||||||||||||||||||||||||||||||||||||||
Sbjct  1071945  GCGGGACTTAACCCAACATTTCACAACACGAGCTGACGACAGCCATGCAGCACCTGTCTC  1072004

Query  429      ACGGTTCCCGAAGGCACTAAGGCATCTCTGCCAAATTCCGTGGATGTCAAGAGTAGGTAA  488
                |  |||||||||||||| ||  ||||||||  || |||  ||||||||||||||||||||
Sbjct  1072005  AGCGTTCCCGAAGGCACCAATCCATCTCTGGAAAGTTCGCTGGATGTCAAGAGTAGGTAA  1072064

Query  489      GGTTCTTCGCGTTGCATCGAATTAAACCACATGCTCCACCGCTTGTGCGGGCCCCCGTCA  548
                ||||||||||||||||||||||||||||||||||||||||||||||||||||||||||||
Sbjct  1072065  GGTTCTTCGCGTTGCATCGAATTAAACCACATGCTCCACCGCTTGTGCGGGCCCCCGTCA  1072124

Query  549      ATTCATTTGAGTTTTAACCTTGCGGCCGTACTCCCCAGGCGGTCGACTTAACGCGTTAGC  608
                ||||||||||||||||||||||||||||||||||||||||||||||||||||||||||||
Sbjct  1072125  ATTCATTTGAGTTTTAACCTTGCGGCCGTACTCCCCAGGCGGTCGACTTAACGCGTTAGC  1072184

Query  609      TCCGGAAGCCACTCCTCAAGGGAACAACCTCCAAGTCGACATCGTTTACGGCGTGGACTA  668
                ||||||||||||||||||||||||||||||||||||||||||||||||||||||||||||
Sbjct  1072185  TCCGGAAGCCACTCCTCAAGGGAACAACCTCCAAGTCGACATCGTTTACGGCGTGGACTA  1072244

Query  669      CCAGGGTATCTAATCCTGTTTGCTCCCCACGCTTTCGCACCTGAGCGTCAGTCTTTGTCC  728
                ||||||||||||||||||||||||||||||||||||||||||||||||||||||||||||
Sbjct  1072245  CCAGGGTATCTAATCCTGTTTGCTCCCCACGCTTTCGCACCTGAGCGTCAGTCTTTGTCC  1072304

Query  729      AGGGGGCCGCCTTCGCCACCGGTATTCCTCCAGATCTCTACGCATTTCACCGCTACACCT  788
                ||||||||||||||||||||||||||||||||||||||||||||||||||||||||||||
Sbjct  1072305  AGGGGGCCGCCTTCGCCACCGGTATTCCTCCAGATCTCTACGCATTTCACCGCTACACCT  1072364

Query  789      GGAATTCTACCCCCCTCTACAAGACTCAAGCCTGCCAGTTTCAAATGCAGTTCCCAGGTT  848
                ||||||||| ||||||||||||||||||||||||||||||||||||||||||||||||||
Sbjct  1072365  GGAATTCTA-CCCCCTCTACAAGACTCAAGCCTGCCAGTTTCAAATGCAGTTCCCAGGTT  1072423

Query  849      AAGCCCGGGGATTTCACATCTGACTTAACAGACCGCCTGCGTGCGCTTTACGCCCAGTAA  908
                ||||||||||||||||||||||||||||||||||||||||||||||||||||||||||||
Sbjct  1072424  AAGCCCGGGGATTTCACATCTGACTTAACAGACCGCCTGCGTGCGCTTTACGCCCAGTAA  1072483

Query  909      TTCCGATTAACGCTTGCACCCTCCGTATTACCGCGGCTGCTGGCACGGAGTTAGCCGGTG  968
                ||||||||||||||||||||||||||||||||||||||||||||||||||||||||||||
Sbjct  1072484  TTCCGATTAACGCTTGCACCCTCCGTATTACCGCGGCTGCTGGCACGGAGTTAGCCGGTG  1072543

Query  969      CTTCTTCTGCGGGTAACGTCAATCGACAGGGTTATTAACCCCGTCGCCTTCCTCCCCGCT  1028
                ||||||||||||||||||||||||||||||||||||||||||||||||||||||||||||
Sbjct  1072544  CTTCTTCTGCGGGTAACGTCAATCGACAGGGTTATTAACCCCGTCGCCTTCCTCCCCGCT  1072603

Query  1029     GAAAGTACTTTACNACCCGAAGGCCTTCTTC  1059
                ||||||||||||| |||||||||||||||||
Sbjct  1072604  GAAAGTACTTTACAACCCGAAGGCCTTCTTC  1072634
```

Range 7: 2415830 to 2416879GenBankGraphics

Next Match
Previous Match
First Match

Alignment statistics for match #7

| Score | Expect | Identities | Gaps | Strand | Frame |
| --- | --- | --- | --- | --- | --- |
| 1860 bits(1007) | 0.0() | 1035/1051(98%) | 1/1051(0%) | Plus/Minus |  |

Features:

```
Query  9        GCCCTCCCGAAGGTTANGCTACCTACTTCTTTTNNANCCCACTCCCATGGTGTGACGGGC  68
                |||||||||||||||| ||||||||||||||||  | |||||||||||||||||||||||
Sbjct  2416879  GCCCTCCCGAAGGTTAAGCTACCTACTTCTTTTGCAACCCACTCCCATGGTGTGACGGGC  2416820

Query  69       GGTGTGTACAAGGCCCGGGAACGTATTCACCGTGGCATTCTGATCCACGATTACTAGCGA  128
                ||||||||||||||||||||||||||||||||||||||||||||||||||||||||||||
Sbjct  2416819  GGTGTGTACAAGGCCCGGGAACGTATTCACCGTGGCATTCTGATCCACGATTACTAGCGA  2416760

Query  129      TTCCGACTTCACGGAGTCGAGTTGCAGACTCCGATCCGGACTACGACGCACTTTGTGAGG  188
                ||||||||||||||||||||||||||||||||||||||||||||||||||||||||||||
Sbjct  2416759  TTCCGACTTCACGGAGTCGAGTTGCAGACTCCGATCCGGACTACGACGCACTTTGTGAGG  2416700

Query  189      TCCGCTTGCTCTCGCGAGGTCGCTTCTCTTTGTATGCGCCATTGTAGCACGTGTGTAGCC  248
                ||||||||||||||||||||||||||||||||||||||||||||||||||||||||||||
Sbjct  2416699  TCCGCTTGCTCTCGCGAGGTCGCTTCTCTTTGTATGCGCCATTGTAGCACGTGTGTAGCC  2416640

Query  249      CTACTCGTAAGGGCCATGATGACTTGACGTCATCCCCACCTTCCTCCGGTTTATCACCGG  308
                ||||||||||||||||||||||||||||||||||||||||||||||||||||||||||||
Sbjct  2416639  CTACTCGTAAGGGCCATGATGACTTGACGTCATCCCCACCTTCCTCCGGTTTATCACCGG  2416580

Query  309      CAGTCTCCTTTGAGTTCCCGACCGAATCGCTGGCAACAAAGGATAAGGGTTGCGCTCGTT  368
                ||||||||||||||||||||||||||||||||||||||||||||||||||||||||||||
Sbjct  2416579  CAGTCTCCTTTGAGTTCCCGACCGAATCGCTGGCAACAAAGGATAAGGGTTGCGCTCGTT  2416520

Query  369      GCGGGACTTAACCCAACATTTCACAACACGAGCTGACGACAGCCATGCAGCACCTGTCTC  428
                ||||||||||||||||||||||||||||||||||||||||||||||||||||||||||||
Sbjct  2416519  GCGGGACTTAACCCAACATTTCACAACACGAGCTGACGACAGCCATGCAGCACCTGTCTC  2416460

Query  429      ACGGTTCCCGAAGGCACTAAGGCATCTCTGCCAAATTCCGTGGATGTCAAGAGTAGGTAA  488
                |  |||||||||||||| ||  ||||||||  || |||  ||||||||||||||||||||
Sbjct  2416459  AGCGTTCCCGAAGGCACCAATCCATCTCTGGAAAGTTCGCTGGATGTCAAGAGTAGGTAA  2416400

Query  489      GGTTCTTCGCGTTGCATCGAATTAAACCACATGCTCCACCGCTTGTGCGGGCCCCCGTCA  548
                ||||||||||||||||||||||||||||||||||||||||||||||||||||||||||||
Sbjct  2416399  GGTTCTTCGCGTTGCATCGAATTAAACCACATGCTCCACCGCTTGTGCGGGCCCCCGTCA  2416340

Query  549      ATTCATTTGAGTTTTAACCTTGCGGCCGTACTCCCCAGGCGGTCGACTTAACGCGTTAGC  608
                ||||||||||||||||||||||||||||||||||||||||||||||||||||||||||||
Sbjct  2416339  ATTCATTTGAGTTTTAACCTTGCGGCCGTACTCCCCAGGCGGTCGACTTAACGCGTTAGC  2416280

Query  609      TCCGGAAGCCACTCCTCAAGGGAACAACCTCCAAGTCGACATCGTTTACGGCGTGGACTA  668
                ||||||||||||||||||||||||||||||||||||||||||||||||||||||||||||
Sbjct  2416279  TCCGGAAGCCACTCCTCAAGGGAACAACCTCCAAGTCGACATCGTTTACGGCGTGGACTA  2416220

Query  669      CCAGGGTATCTAATCCTGTTTGCTCCCCACGCTTTCGCACCTGAGCGTCAGTCTTTGTCC  728
                ||||||||||||||||||||||||||||||||||||||||||||||||||||||||||||
Sbjct  2416219  CCAGGGTATCTAATCCTGTTTGCTCCCCACGCTTTCGCACCTGAGCGTCAGTCTTTGTCC  2416160

Query  729      AGGGGGCCGCCTTCGCCACCGGTATTCCTCCAGATCTCTACGCATTTCACCGCTACACCT  788
                ||||||||||||||||||||||||||||||||||||||||||||||||||||||||||||
Sbjct  2416159  AGGGGGCCGCCTTCGCCACCGGTATTCCTCCAGATCTCTACGCATTTCACCGCTACACCT  2416100

Query  789      GGAATTCTACCCCCCTCTACAAGACTCAAGCCTGCCAGTTTCAAATGCAGTTCCCAGGTT  848
                |||||||||||||| |||||||||||||||||||||||||||||||||||||||||||||
Sbjct  2416099  GGAATTCTACCCCC-TCTACAAGACTCAAGCCTGCCAGTTTCAAATGCAGTTCCCAGGTT  2416041

Query  849      AAGCCCGGGGATTTCACATCTGACTTAACAGACCGCCTGCGTGCGCTTTACGCCCAGTAA  908
                ||||||||||||||||||||||||||||||||||||||||||||||||||||||||||||
Sbjct  2416040  AAGCCCGGGGATTTCACATCTGACTTAACAGACCGCCTGCGTGCGCTTTACGCCCAGTAA  2415981

Query  909      TTCCGATTAACGCTTGCACCCTCCGTATTACCGCGGCTGCTGGCACGGAGTTAGCCGGTG  968
                ||||||||||||||||||||||||||||||||||||||||||||||||||||||||||||
Sbjct  2415980  TTCCGATTAACGCTTGCACCCTCCGTATTACCGCGGCTGCTGGCACGGAGTTAGCCGGTG  2415921

Query  969      CTTCTTCTGCGGGTAACGTCAATCGACAGGGTTATTAACCCCGTCGCCTTCCTCCCCGCT  1028
                ||||||||||||||||||||||||||||||||||||||||||||||||||||||||||||
Sbjct  2415920  CTTCTTCTGCGGGTAACGTCAATCGACAGGGTTATTAACCCCGTCGCCTTCCTCCCCGCT  2415861

Query  1029     GAAAGTACTTTACNACCCGAAGGCCTTCTTC  1059
                ||||||||||||| |||||||||||||||||
Sbjct  2415860  GAAAGTACTTTACAACCCGAAGGCCTTCTTC  2415830
```

Download

FASTA (complete sequence)

FASTA (aligned sequences)

GenBank (complete sequence)

Continue
Cancel

GenBankGraphics

Next
Previous
Descriptions

Uncultured Pantoea sp. clone GJ\_(2)\_7 16S ribosomal RNA gene, partial sequence

Sequence ID: KX603479.1Length: 1394Number of Matches: 1

Related Information

Range 1: 298 to 1348GenBankGraphics

Next Match
Previous Match
First Match

Alignment statistics for match #1

| Score | Expect | Identities | Gaps | Strand | Frame |
| --- | --- | --- | --- | --- | --- |
| 1923 bits(1041) | 0.0() | 1046/1051(99%) | 0/1051(0%) | Plus/Minus |  |

Features:

```
Query  9     GCCCTCCCGAAGGTTANGCTACCTACTTCTTTTNNANCCCACTCCCATGGTGTGACGGGC  68
             |||||||||||||||| ||||||||||||||||  | |||||||||||||||||||||||
Sbjct  1348  GCCCTCCCGAAGGTTAAGCTACCTACTTCTTTTGCAACCCACTCCCATGGTGTGACGGGC  1289

Query  69    GGTGTGTACAAGGCCCGGGAACGTATTCACCGTGGCATTCTGATCCACGATTACTAGCGA  128
             ||||||||||||||||||||||||||||||||||||||||||||||||||||||||||||
Sbjct  1288  GGTGTGTACAAGGCCCGGGAACGTATTCACCGTGGCATTCTGATCCACGATTACTAGCGA  1229

Query  129   TTCCGACTTCACGGAGTCGAGTTGCAGACTCCGATCCGGACTACGACGCACTTTGTGAGG  188
             ||||||||||||||||||||||||||||||||||||||||||||||||||||||||||||
Sbjct  1228  TTCCGACTTCACGGAGTCGAGTTGCAGACTCCGATCCGGACTACGACGCACTTTGTGAGG  1169

Query  189   TCCGCTTGCTCTCGCGAGGTCGCTTCTCTTTGTATGCGCCATTGTAGCACGTGTGTAGCC  248
             ||||||||||||||||||||||||||||||||||||||||||||||||||||||||||||
Sbjct  1168  TCCGCTTGCTCTCGCGAGGTCGCTTCTCTTTGTATGCGCCATTGTAGCACGTGTGTAGCC  1109

Query  249   CTACTCGTAAGGGCCATGATGACTTGACGTCATCCCCACCTTCCTCCGGTTTATCACCGG  308
             ||||||||||||||||||||||||||||||||||||||||||||||||||||||||||||
Sbjct  1108  CTACTCGTAAGGGCCATGATGACTTGACGTCATCCCCACCTTCCTCCGGTTTATCACCGG  1049

Query  309   CAGTCTCCTTTGAGTTCCCGACCGAATCGCTGGCAACAAAGGATAAGGGTTGCGCTCGTT  368
             ||||||||||||||||||||||||||||||||||||||||||||||||||||||||||||
Sbjct  1048  CAGTCTCCTTTGAGTTCCCGACCGAATCGCTGGCAACAAAGGATAAGGGTTGCGCTCGTT  989

Query  369   GCGGGACTTAACCCAACATTTCACAACACGAGCTGACGACAGCCATGCAGCACCTGTCTC  428
             ||||||||||||||||||||||||||||||||||||||||||||||||||||||||||||
Sbjct  988   GCGGGACTTAACCCAACATTTCACAACACGAGCTGACGACAGCCATGCAGCACCTGTCTC  929

Query  429   ACGGTTCCCGAAGGCACTAAGGCATCTCTGCCAAATTCCGTGGATGTCAAGAGTAGGTAA  488
             ||||||||||||||||||||||||||||||||||||||||||||||||||||||||||||
Sbjct  928   ACGGTTCCCGAAGGCACTAAGGCATCTCTGCCAAATTCCGTGGATGTCAAGAGTAGGTAA  869

Query  489   GGTTCTTCGCGTTGCATCGAATTAAACCACATGCTCCACCGCTTGTGCGGGCCCCCGTCA  548
             ||||||||||||||||||||||||||||||||||||||||||||||||||||||||||||
Sbjct  868   GGTTCTTCGCGTTGCATCGAATTAAACCACATGCTCCACCGCTTGTGCGGGCCCCCGTCA  809

Query  549   ATTCATTTGAGTTTTAACCTTGCGGCCGTACTCCCCAGGCGGTCGACTTAACGCGTTAGC  608
             ||||||||||||||||||||||||||||||||||||||||||||||||||||||||||||
Sbjct  808   ATTCATTTGAGTTTTAACCTTGCGGCCGTACTCCCCAGGCGGTCGACTTAACGCGTTAGC  749

Query  609   TCCGGAAGCCACTCCTCAAGGGAACAACCTCCAAGTCGACATCGTTTACGGCGTGGACTA  668
             ||||||||||||||||||||||||||||||||||||||||||||||||||||||||||||
Sbjct  748   TCCGGAAGCCACTCCTCAAGGGAACAACCTCCAAGTCGACATCGTTTACGGCGTGGACTA  689

Query  669   CCAGGGTATCTAATCCTGTTTGCTCCCCACGCTTTCGCACCTGAGCGTCAGTCTTTGTCC  728
             ||||||||||||||||||||||||||||||||||||||||||||||||||||||||||||
Sbjct  688   CCAGGGTATCTAATCCTGTTTGCTCCCCACGCTTTCGCACCTGAGCGTCAGTCTTTGTCC  629

Query  729   AGGGGGCCGCCTTCGCCACCGGTATTCCTCCAGATCTCTACGCATTTCACCGCTACACCT  788
             ||||||||||||||||||||||||||||||||||||||||||||||||||||||||||||
Sbjct  628   AGGGGGCCGCCTTCGCCACCGGTATTCCTCCAGATCTCTACGCATTTCACCGCTACACCT  569

Query  789   GGAATTCTACCCCCCTCTACAAGACTCAAGCCTGCCAGTTTCAAATGCAGTTCCCAGGTT  848
             ||||||||||||||||||||||||||||||||||||||||||||||||||||||||||||
Sbjct  568   GGAATTCTACCCCCCTCTACAAGACTCAAGCCTGCCAGTTTCAAATGCAGTTCCCAGGTT  509

Query  849   AAGCCCGGGGATTTCACATCTGACTTAACAGACCGCCTGCGTGCGCTTTACGCCCAGTAA  908
             ||||||||||||||||||||||||||||||||||||||||||||||||||||||||||||
Sbjct  508   AAGCCCGGGGATTTCACATCTGACTTAACAGACCGCCTGCGTGCGCTTTACGCCCAGTAA  449

Query  909   TTCCGATTAACGCTTGCACCCTCCGTATTACCGCGGCTGCTGGCACGGAGTTAGCCGGTG  968
             ||||||||||||||||||||||||||||||||||||||||||||||||||||||||||||
Sbjct  448   TTCCGATTAACGCTTGCACCCTCCGTATTACCGCGGCTGCTGGCACGGAGTTAGCCGGTG  389

Query  969   CTTCTTCTGCGGGTAACGTCAATCGACAGGGTTATTAACCCCGTCGCCTTCCTCCCCGCT  1028
             ||||||||||||||||||||||||||||||||||||||||||||||||||||||||||||
Sbjct  388   CTTCTTCTGCGGGTAACGTCAATCGACAGGGTTATTAACCCCGTCGCCTTCCTCCCCGCT  329

Query  1029  GAAAGTACTTTACNACCCGAAGGCCTTCTTC  1059
             ||||||||||||| |||||||||||||||||
Sbjct  328   GAAAGTACTTTACAACCCGAAGGCCTTCTTC  298
```

Download

FASTA (complete sequence)

FASTA (aligned sequences)

GenBank (complete sequence)

Continue
Cancel

GenBankGraphics

Next
Previous
Descriptions

Pantoea agglomerans strain SSE 16S ribosomal RNA gene, partial sequence

Sequence ID: KT075193.1Length: 1500Number of Matches: 1

Related Information

Range 1: 400 to 1450GenBankGraphics

Next Match
Previous Match
First Match

Alignment statistics for match #1

| Score | Expect | Identities | Gaps | Strand | Frame |
| --- | --- | --- | --- | --- | --- |
| 1923 bits(1041) | 0.0() | 1046/1051(99%) | 0/1051(0%) | Plus/Minus |  |

Features:

```
Query  9     GCCCTCCCGAAGGTTANGCTACCTACTTCTTTTNNANCCCACTCCCATGGTGTGACGGGC  68
             |||||||||||||||| ||||||||||||||||  | |||||||||||||||||||||||
Sbjct  1450  GCCCTCCCGAAGGTTAAGCTACCTACTTCTTTTGCAACCCACTCCCATGGTGTGACGGGC  1391

Query  69    GGTGTGTACAAGGCCCGGGAACGTATTCACCGTGGCATTCTGATCCACGATTACTAGCGA  128
             ||||||||||||||||||||||||||||||||||||||||||||||||||||||||||||
Sbjct  1390  GGTGTGTACAAGGCCCGGGAACGTATTCACCGTGGCATTCTGATCCACGATTACTAGCGA  1331

Query  129   TTCCGACTTCACGGAGTCGAGTTGCAGACTCCGATCCGGACTACGACGCACTTTGTGAGG  188
             ||||||||||||||||||||||||||||||||||||||||||||||||||||||||||||
Sbjct  1330  TTCCGACTTCACGGAGTCGAGTTGCAGACTCCGATCCGGACTACGACGCACTTTGTGAGG  1271

Query  189   TCCGCTTGCTCTCGCGAGGTCGCTTCTCTTTGTATGCGCCATTGTAGCACGTGTGTAGCC  248
             ||||||||||||||||||||||||||||||||||||||||||||||||||||||||||||
Sbjct  1270  TCCGCTTGCTCTCGCGAGGTCGCTTCTCTTTGTATGCGCCATTGTAGCACGTGTGTAGCC  1211

Query  249   CTACTCGTAAGGGCCATGATGACTTGACGTCATCCCCACCTTCCTCCGGTTTATCACCGG  308
             ||||||||||||||||||||||||||||||||||||||||||||||||||||||||||||
Sbjct  1210  CTACTCGTAAGGGCCATGATGACTTGACGTCATCCCCACCTTCCTCCGGTTTATCACCGG  1151

Query  309   CAGTCTCCTTTGAGTTCCCGACCGAATCGCTGGCAACAAAGGATAAGGGTTGCGCTCGTT  368
             ||||||||||||||||||||||||||||||||||||||||||||||||||||||||||||
Sbjct  1150  CAGTCTCCTTTGAGTTCCCGACCGAATCGCTGGCAACAAAGGATAAGGGTTGCGCTCGTT  1091

Query  369   GCGGGACTTAACCCAACATTTCACAACACGAGCTGACGACAGCCATGCAGCACCTGTCTC  428
             ||||||||||||||||||||||||||||||||||||||||||||||||||||||||||||
Sbjct  1090  GCGGGACTTAACCCAACATTTCACAACACGAGCTGACGACAGCCATGCAGCACCTGTCTC  1031

Query  429   ACGGTTCCCGAAGGCACTAAGGCATCTCTGCCAAATTCCGTGGATGTCAAGAGTAGGTAA  488
             ||||||||||||||||||||||||||||||||||||||||||||||||||||||||||||
Sbjct  1030  ACGGTTCCCGAAGGCACTAAGGCATCTCTGCCAAATTCCGTGGATGTCAAGAGTAGGTAA  971

Query  489   GGTTCTTCGCGTTGCATCGAATTAAACCACATGCTCCACCGCTTGTGCGGGCCCCCGTCA  548
             ||||||||||||||||||||||||||||||||||||||||||||||||||||||||||||
Sbjct  970   GGTTCTTCGCGTTGCATCGAATTAAACCACATGCTCCACCGCTTGTGCGGGCCCCCGTCA  911

Query  549   ATTCATTTGAGTTTTAACCTTGCGGCCGTACTCCCCAGGCGGTCGACTTAACGCGTTAGC  608
             ||||||||||||||||||||||||||||||||||||||||||||||||||||||||||||
Sbjct  910   ATTCATTTGAGTTTTAACCTTGCGGCCGTACTCCCCAGGCGGTCGACTTAACGCGTTAGC  851

Query  609   TCCGGAAGCCACTCCTCAAGGGAACAACCTCCAAGTCGACATCGTTTACGGCGTGGACTA  668
             ||||||||||||||||||||||||||||||||||||||||||||||||||||||||||||
Sbjct  850   TCCGGAAGCCACTCCTCAAGGGAACAACCTCCAAGTCGACATCGTTTACGGCGTGGACTA  791

Query  669   CCAGGGTATCTAATCCTGTTTGCTCCCCACGCTTTCGCACCTGAGCGTCAGTCTTTGTCC  728
             ||||||||||||||||||||||||||||||||||||||||||||||||||||||||||||
Sbjct  790   CCAGGGTATCTAATCCTGTTTGCTCCCCACGCTTTCGCACCTGAGCGTCAGTCTTTGTCC  731

Query  729   AGGGGGCCGCCTTCGCCACCGGTATTCCTCCAGATCTCTACGCATTTCACCGCTACACCT  788
             ||||||||||||||||||||||||||||||||||||||||||||||||||||||||||||
Sbjct  730   AGGGGGCCGCCTTCGCCACCGGTATTCCTCCAGATCTCTACGCATTTCACCGCTACACCT  671

Query  789   GGAATTCTACCCCCCTCTACAAGACTCAAGCCTGCCAGTTTCAAATGCAGTTCCCAGGTT  848
             ||||||||||||||||||||||||||||||||||||||||||||||||||||||||||||
Sbjct  670   GGAATTCTACCCCCCTCTACAAGACTCAAGCCTGCCAGTTTCAAATGCAGTTCCCAGGTT  611

Query  849   AAGCCCGGGGATTTCACATCTGACTTAACAGACCGCCTGCGTGCGCTTTACGCCCAGTAA  908
             ||||||||||||||||||||||||||||||||||||||||||||||||||||||||||||
Sbjct  610   AAGCCCGGGGATTTCACATCTGACTTAACAGACCGCCTGCGTGCGCTTTACGCCCAGTAA  551

Query  909   TTCCGATTAACGCTTGCACCCTCCGTATTACCGCGGCTGCTGGCACGGAGTTAGCCGGTG  968
             ||||||||||||||||||||||||||||||||||||||||||||||||||||||||||||
Sbjct  550   TTCCGATTAACGCTTGCACCCTCCGTATTACCGCGGCTGCTGGCACGGAGTTAGCCGGTG  491

Query  969   CTTCTTCTGCGGGTAACGTCAATCGACAGGGTTATTAACCCCGTCGCCTTCCTCCCCGCT  1028
             ||||||||||||||||||||||||||||||||||||||||||||||||||||||||||||
Sbjct  490   CTTCTTCTGCGGGTAACGTCAATCGACAGGGTTATTAACCCCGTCGCCTTCCTCCCCGCT  431

Query  1029  GAAAGTACTTTACNACCCGAAGGCCTTCTTC  1059
             ||||||||||||| |||||||||||||||||
Sbjct  430   GAAAGTACTTTACAACCCGAAGGCCTTCTTC  400
```


BLAST is a registered trademark of the National Library of Medicine

Support center
Mailing list


YouTube

- National Library Of Medicine
- National Institutes Of Health
- U.S. Department of Health & Human Services
- USA.gov

### NCBI


National Center for Biotechnology Information,
 U.S. National Library of Medicine

8600 Rockville Pike,
Bethesda
 MD,
20894
USA

Policies and Guidelines
|
Contact


PreferencesTurn off

External link. Please review our privacy policy.
