# Supplementary material for: Isothermal microcalorimetry for thermal viable count of microorganisms in pure cultures and stabilized formulations
Source: BMC Microbiol. 2019 Mar 21;19:65. doi: 10.1186/s12866-019-1432-8 (PMC6429831; doi:10.1186/s12866-019-1432-8)
Supplement: Supplementary file 3 — 16S rRNA gene sequences’ BLASTN hits in zipped HTML format. (ZIP 15810 kb) [file 12866_2019_1432_MOESM3_ESM.zip › Best blastn hits/NCBI Blast_D pink F -- 15..1018 of sequence (1004.html]

NCBI Blast:D pink F -- 15..1018 of sequence (1004


- NCBI Home
- Sign in to NCBI
- Skip to Main Content
- Skip to Navigation
- About NCBI Accesskeys

U.S. National Library of Medicine

NCBI
National Center for Biotechnology Information

- My NCBI
- Sign in to NCBI
- Register
- Sign Out

BLAST ^®^ » blastn suite » RID-A4XJU6FF014


- Home
- Recent Results
- Saved Strategies
- Help

BLAST Results


Edit and Resubmit
Save Search Strategies
[Sign in above to save your search strategy]

Formatting options 


Download


How to read this page
Blast report description
Questions/comments


|  |  |
| --- | --- |
| Formatting options | |
| Show | Alignment as  HTML Plain text   Old View Reset form to defaults [?]  These options control formatting of alignments in results pages. The default is HTML, but other formats (including plain text) are available. PSSM and PssmWithParameters are representations of Position Specific Scoring Matrices and are only available for PSI-BLAST. The Advanced view option allows the database descriptions to be sorted by various indices in a table. |
| Alignment View | Pairwise Pairwise with dots for identities Query-anchored with dots for identities Query-anchored with letters for identities Flat query-anchored with dots for identities Flat query-anchored with letters for identities [?]  Choose how to view alignments. The default "pairwise" view shows how each subject sequence aligns individually to the query sequence. The "query-anchored" view shows how all subject sequences align to the query sequence. For each view type, you can choose to show "identities" (matching residues) as letters or dots. more... |
| Display | Graphical Overview   Linkout   Sequence Retrieval  NCBI-gi   CDS feature [?]  - Graphical Overview: Graphical Overview: Show graph of similar sequence regions aligned to query.   more... - NCBI-gi: Show NCBI gi identifiers. - CDS feature: Show annotated coding region and translation.   more... |
| Masking | Character:   X for protein, n for nucleotide Lower Case  Color:  Black Grey Red [?]  - Masking Character: Display masked (filtered) sequence regions as lower-case or as specific letters (N for nucleotide, P for protein). - Masking Color: Display masked sequence regions in the given color. |
| Limit results | Descriptions:  10 50 100 Graphical overview:  0 10 50 100  Alignments:  0 10 50 100 Line length:  60 90 120 150 [?]  - Descriptions: Show short descriptions for up to the given number of sequences. - Alignments: Show alignments for up to the given number of sequences, in order of statistical significance. - Line lenghth: Number of letters to show on one line in an alignment. |
|  | Organism Type common name, binomial, taxid, or group name. Only 20 top taxa will be shown.     Exclude   [?]  Show only sequences from the given organism. |
|  | Entrez query:  [?]  Show only those sequences that match the given Entrez query. more... |
|  | Expect Min:  Expect Max:  [?]  Show only sequences with expect values in the given range. more... |
|  | Percent Identity Min:  Percent Identity Max:  [?]  Show only sequences with percent identity values in the given range. |
| Format for | PSI-BLAST with inclusion threshold:  [?]  - Format for PSI-BLAST: The Position-Specific Iterated BLAST (PSI-BLAST) program performs iterative searches with a protein query,   in which sequences found in one round of search are used to build a custom score model for the next round.   more... - Inclusion Threshold: This sets the statistical significance threshold for including a sequence in the model used   by PSI-BLAST to create the PSSM on the next iteration. |

|  |  |  |  |  |  |
| --- | --- | --- | --- | --- | --- |
| Download | | | | | |
| Alignment  Text XML ASN.1 JSON Seq-align Hit Table(text) Hit Table(csv) Multiple-file XML2 Single-file XML2 Multiple-file JSON Single-file JSON SAM | Search Strategies  ASN.1 | PSSM to restart search  PSSM | [?] |  |  |

The Download link provides BLAST output that may be used as input to another program.
This includes parseable formats such as the tabular report or XML as well as the Search Strategy files read by the BLAST+ applications.
More details on the parseable (XML, tabular, and ASN.1) reports can be found at
https://www.ncbi.nlm.nih.gov/books/NBK153387/  
  

The following formats are offered under the Alignment section:  
1). "Text". Non-HTML standard BLAST report.  
2). "XML". XML report based upon the DTD at https://www.ncbi.nlm.nih.gov/data\_specs/dtd/NCBI\_BlastOutput.dtd  
3). "ASN.1". Alignment written out in Abstract Syntax Notation 1.  
4). "JSON Seq-align". Alignment written out in JSON.  
4). "Hit Table(text)". The tabular report as text.  
5). "Hit Table(csv)". The tabular report ready for import into spread-sheet programs like Excel.  
6). "XML2". New XML format described at ftp://ftp.ncbi.nlm.nih.gov/blast/documents/NEWXML/xml2.pdf.  
7). "JSON". New JSON format described at ftp://ftp.ncbi.nlm.nih.gov/blast/documents/NEWXML/xml2.pdf.  
8). "SAM". Sequence Alignment Map format.

XML2 and JSON can be downloaded either as one file per query (multiple-file) or one file for all queries (single-file). These formats are listed as Multiple-file XML2 (and JSON) or Single-file XML (and JSON).

The following report is offered under the Search Strategy section:  
1). "ASN.1" Search Strategy. A record of the parameters, query, and database used in the search. This file can be used to start a stand-alone BLAST search, see
https://www.ncbi.nlm.nih.gov/books/NBK1763/#CmdLineAppsManual.I455\_BLAST\_search\_stra


# Job title: D pink F -- 15..1018 of sequence (1004

Results for:

lcl|Query\_128653 D pink F -- 15..1018 of sequence(1004bp)
[?]

Your BLAST job specified more than one input sequence.
This box lets you choose which input sequence to show BLAST results for.

RID
:   A4XJU6FF014 (Expires on 03-10 18:56 pm)

Query ID
:   lcl|Query\_128653
:   lcl|Query\_128653

Description
:   D pink F -- 15..1018 of sequence

Molecule type
:   nucleic acid

Query Length
:   1004

Database Name
:   nr

Description
:   Nucleotide collection (nt) See details

Program
:   BLASTN 2.8.0+ Citation

  

Reference 

Zheng Zhang, Scott Schwartz, Lukas Wagner, and Webb Miller (2000), "A greedy algorithm for aligning DNA sequences", J Comput Biol 2000; 7(1-2):203-14.

Reference - database indexing

Aleksandr Morgulis, George Coulouris, Yan Raytselis, Thomas L. Madden, Richa Agarwala, Alejandro A. Schäffer (2008), "Database Indexing for Production MegaBLAST Searches", Bioinformatics 24:1757-1764.

Other reports:
Search Summary

[Taxonomy reports]
[Distance tree of results]
[MSA viewer]

Search Parameters

| Search parameter name | Search parameter value |
| --- | --- |
| Program | blastn |
| Word size | 28 |
| Expect value | 10 |
| Hitlist size | 100 |
| Match/Mismatch scores | 1,-2 |
| Gapcosts | 0,2.5 |
| Low Complexity Filter | Yes |
| Filter string | L;m; |
| Genetic Code | 1 |

Database

| Database parameter name | Database parameter value |
| --- | --- |
| Posted date | Mar 3, 2018 9:32 PM |
| Number of letters | 171,598,911,005 |
| Number of sequences | 46,870,701 |
| Entrez query | Includes:  Excludes:  None |

Karlin-Altschul statistics

| Params | Ungapped | Gapped |
| --- | --- | --- |
| Lambda | 1.33271 | 1.28 |
| K | 0.620991 | 0.46 |
| H | 1.12409 | 0.85 |

Results Statistics

| Results Statistics parameter name | Results Statistics parameter value |
| --- | --- |
| Length adjustment | 35 |
| Effective length of query | 969 |
| Effective length of database | 169958436470 |
| Effective search space | 164689724939430 |
| Effective search space used | 164689724939430 |


## Graphic Summary

### Distribution of the top 100 Blast Hits on 100 subject sequences [?]

The graphic is an overview of the database sequences aligned to the query sequence. These are represented horizontal bars colored coded by score and showing the extent
of the alignment on the query sequence. Separate aligned regions on the same database sequence are connected by a thin grey line.
Mousing over an alignment shows the database sequence title. Clicking an alignment displays a box with more details about the alignment and
link to the sequence alignment itself in the Alignments section of the report.

Mouse over to see the title, click to show alignments

Color key for alignment scores

<40

40-50

50-80

80-200

>=200

Query

1

200

400

600

800

1000

Paenibacillus sp. 3Cp1 16S ribosomal RNA gene, partial ..

Score:1831 Evalue:0

Accession:KP852522.1

Alignment

Uncultured bacterium clone 4L13 16S ribosomal RNA gene,..

Score:1831 Evalue:0

Accession:KP843054.1

Alignment

Paenibacillus sp. TA\_EU partial 16S rRNA gene, strain T..

Score:1831 Evalue:0

Accession:HG942127.1

Alignment

Bacterium 4R9 16S ribosomal RNA gene, partial sequence

Score:1827 Evalue:0

Accession:KP965575.1

Alignment

Paenibacillus sp. strain PS-K-17 16S ribosomal RNA gene..

Score:1821 Evalue:0

Accession:MF443394.1

Alignment

Paenibacillus sp. TI45-13ar 16S ribosomal RNA gene, par..

Score:1812 Evalue:0

Accession:KY419705.1

Alignment

Paenibacillus sp. Rs5 16S ribosomal RNA gene, partial s..

Score:1810 Evalue:0

Accession:JQ977193.1

Alignment

Paenibacillus sp. strain BSW35 16S ribosomal RNA gene, ..

Score:1794 Evalue:0

Accession:KY446062.1

Alignment

Paenibacillus sp. 9N4 16S ribosomal RNA gene, partial s..

Score:1794 Evalue:0

Accession:HQ284889.1

Alignment

Bacterium 4L18 16S ribosomal RNA gene, partial sequence

Score:1779 Evalue:0

Accession:KP965574.1

Alignment

Bacterium 4L13 16S ribosomal RNA gene, partial sequence

Score:1773 Evalue:0

Accession:KP965573.1

Alignment

Uncultured bacterium clone nbw208h09c1 16S ribosomal RN..

Score:1772 Evalue:0

Accession:GQ073426.1

Alignment

Paenibacillus sp. Q1-R10 16S ribosomal RNA gene, partia..

Score:1768 Evalue:0

Accession:JX994123.1

Alignment

Paenibacillus sp. HDDMM03 16S ribosomal RNA gene, parti..

Score:1760 Evalue:0

Accession:EU723825.1

Alignment

Paenibacillus sp. NCCB 100172 16S ribosomal RNA gene, p..

Score:1760 Evalue:0

Accession:EU003588.1

Alignment

Bacterium 4R1 16S ribosomal RNA gene, partial sequence

Score:1755 Evalue:0

Accession:KP965572.1

Alignment

Uncultured bacterium clone 10-9BM 16S ribosomal RNA gen..

Score:1753 Evalue:0

Accession:HQ143281.1

Alignment

Uncultured bacterium clone ncd2164d05c1 16S ribosomal R..

Score:1749 Evalue:0

Accession:JF185937.1

Alignment

Paenibacillus illinoisensis strain WR\_17 16S ribosomal ..

Score:1746 Evalue:0

Accession:HQ661869.1

Alignment

Uncultured bacterium clone ncd2162h02c1 16S ribosomal R..

Score:1744 Evalue:0

Accession:JF185833.1

Alignment

Uncultured bacterium clone ncd2163a03c1 16S ribosomal R..

Score:1738 Evalue:0

Accession:JF185844.1

Alignment

Paenibacillus nicotianae strain YIM h-19 16S ribosomal ..

Score:1733 Evalue:0

Accession:NR\_134783.2

Alignment

Paenibacillus nicotianae strain YIM h-19 16S ribosomal ..

Score:1733 Evalue:0

Accession:KJ792471.1

Alignment

Paenibacillus kyungheensis strain DCY88 16S ribosomal R..

Score:1724 Evalue:0

Accession:NR\_145628.1

Alignment

Paenibacillus kyungheensis strain DCY88 16S ribosomal R..

Score:1724 Evalue:0

Accession:KF793934.1

Alignment

Paenibacillus sp. R1 16S ribosomal RNA gene, partial se..

Score:1722 Evalue:0

Accession:KR263165.1

Alignment

Paenibacillus hordei strain RH-N24 16S ribosomal RNA ge..

Score:1720 Evalue:0

Accession:NR\_109318.1

Alignment

Paenibacillus illinoisensis genomic DNA containing 16S-..

Score:1690 Evalue:0

Accession:LM994777.1

Alignment

Paenibacillus illinoisensis partial 16S rRNA gene, isol..

Score:1677 Evalue:0

Accession:HE587985.1

Alignment

Paenibacillus sp. SC22 partial 16S rRNA gene, isolate S..

Score:1650 Evalue:0

Accession:HF566260.1

Alignment

Paenibacillus sp. UPUM12 16S ribosomal RNA gene, partia..

Score:1640 Evalue:0

Accession:KP889056.1

Alignment

Paenibacillus illinoisensis partial 16S rRNA gene, isol..

Score:1639 Evalue:0

Accession:HE587984.1

Alignment

Paenibacillus hordei strain poapink1 16S ribosomal RNA ..

Score:1635 Evalue:0

Accession:KJ528493.1

Alignment

Uncultured bacterium clone 56-9BM 16S ribosomal RNA gen..

Score:1613 Evalue:0

Accession:HQ143315.1

Alignment

Paenibacillus sp. WS26 16S ribosomal RNA gene, partial ..

Score:1587 Evalue:0

Accession:JN899576.1

Alignment

Paenibacillus sp. H316 16S ribosomal RNA gene, partial ..

Score:1583 Evalue:0

Accession:KT205397.1

Alignment

Paenibacillus sp. WS28 16S ribosomal RNA gene, partial ..

Score:1583 Evalue:0

Accession:JN899577.1

Alignment

Paenibacillus sp. WS16 16S ribosomal RNA gene, partial ..

Score:1583 Evalue:0

Accession:JN899571.1

Alignment

Paenibacillus durus strain hswX78 16S ribosomal RNA gen..

Score:1583 Evalue:0

Accession:JQ236815.1

Alignment

Paenibacillus sp. strain B1 16S ribosomal RNA gene, par..

Score:1581 Evalue:0

Accession:KY111475.1

Alignment

Paenibacillus sp. BL13 16S ribosomal RNA gene, partial ..

Score:1581 Evalue:0

Accession:EU912463.1

Alignment

Paenibacillus illinoisensis strain Z002 16S ribosomal R..

Score:1580 Evalue:0

Accession:MG266466.1

Alignment

Paenibacillus sp. EN-26 16S ribosomal RNA gene, partial..

Score:1580 Evalue:0

Accession:KP702927.1

Alignment

Paenibacillus sp. A10 16S ribosomal RNA gene, partial s..

Score:1578 Evalue:0

Accession:KP126817.1

Alignment

Paenibacillus illinoisensis strain SBANHCa16 16S riboso..

Score:1578 Evalue:0

Accession:KR152299.1

Alignment

Bacterium enrichment culture clone JYJ-11 16S ribosomal..

Score:1578 Evalue:0

Accession:KR996781.1

Alignment

Paenibacillus illinoisensis strain P9S 16S ribosomal RN..

Score:1578 Evalue:0

Accession:KF010797.1

Alignment

Paenibacillus sp. MBEU37 gene for 16S rRNA, partial seq..

Score:1578 Evalue:0

Accession:AB733580.1

Alignment

Paenibacillus illinoisensis strain F24 16S ribosomal RN..

Score:1578 Evalue:0

Accession:JQ579623.1

Alignment

Paenibacillus sp. BL17-2 16S ribosomal RNA gene, partia..

Score:1578 Evalue:0

Accession:EU912464.1

Alignment

Paenibacillus durus strain YUPP-3 16S ribosomal RNA gen..

Score:1578 Evalue:0

Accession:FJ696708.1

Alignment

Paenibacillus illinoisensis partial 16S rRNA gene, stra..

Score:1578 Evalue:0

Accession:FN422001.1

Alignment

Paenibacillus sp. CRRI-82\_WR13B 16S ribosomal RNA gene,..

Score:1576 Evalue:0

Accession:KF840379.1

Alignment

Paenibacillus sp. WS30 16S ribosomal RNA gene, partial ..

Score:1576 Evalue:0

Accession:JN899578.1

Alignment

Paenibacillus illinoisensis strain 3728 16S ribosomal R..

Score:1576 Evalue:0

Accession:JN867752.1

Alignment

Paenibacillus illinoisensis strain JCM 9907 16S ribosom..

Score:1576 Evalue:0

Accession:NR\_040884.1

Alignment

## Descriptions

, Reading indexes 1-5, displaying indexes 1-5


Load next setPrevious Match

Sequences producing significant alignments:

Show all columns  of the table presenting sequences producing significant alignments 

Select:AllNone
Selected:0

Alignments
Download

FASTA (complete sequence)

FASTA (aligned sequences)

GenBank (complete sequence)

Hit Table (text)

Hit Table (CSV)

Text

XML

ASN.1

Continue
Cancel

GenBank 
Graphics
Distance tree of results
Multiple alignment
Show/hide columns of the table presenting sequences producing significant alignments 

Available columns

Description  
Max Score  
Total Score  
Coverage  
E-value  
IdentN  
Accession  
Restore Defaults
Ok
Cancel

Sequences producing significant alignments:

| Select for downloading or viewing reports | Description | Max score | Total score | Query cover | E value | Ident | Accession |
| --- | --- | --- | --- | --- | --- | --- | --- |
| 1Select seq KP852522.1 | Paenibacillus sp. 3Cp1 16S ribosomal RNA gene, partial sequence | 1831 | 1831 | 98% | 0.0 | 99% | KP852522.1 |
| 2Select seq KP843054.1 | Uncultured bacterium clone 4L13 16S ribosomal RNA gene, partial sequence | 1831 | 1831 | 98% | 0.0 | 99% | KP843054.1 |
| 3Select seq HG942127.1 | Paenibacillus sp. TA\_EU partial 16S rRNA gene, strain TA\_EU | 1831 | 1831 | 98% | 0.0 | 99% | HG942127.1 |
| 4Select seq KP965575.1 | Bacterium 4R9 16S ribosomal RNA gene, partial sequence | 1827 | 1827 | 99% | 0.0 | 99% | KP965575.1 |
| 5Select seq MF443394.1 | Paenibacillus sp. strain PS-K-17 16S ribosomal RNA gene, partial sequence | 1821 | 1821 | 99% | 0.0 | 99% | MF443394.1 |
| 6Select seq KY419705.1 | Paenibacillus sp. TI45-13ar 16S ribosomal RNA gene, partial sequence | 1812 | 1812 | 99% | 0.0 | 99% | KY419705.1 |
| 7Select seq JQ977193.1 | Paenibacillus sp. Rs5 16S ribosomal RNA gene, partial sequence | 1810 | 1810 | 99% | 0.0 | 99% | JQ977193.1 |
| 8Select seq KY446062.1 | Paenibacillus sp. strain BSW35 16S ribosomal RNA gene, partial sequence | 1794 | 1794 | 98% | 0.0 | 99% | KY446062.1 |
| 9Select seq HQ284889.1 | Paenibacillus sp. 9N4 16S ribosomal RNA gene, partial sequence | 1794 | 1794 | 98% | 0.0 | 99% | HQ284889.1 |
| 10Select seq KP965574.1 | Bacterium 4L18 16S ribosomal RNA gene, partial sequence | 1779 | 1779 | 99% | 0.0 | 99% | KP965574.1 |
| 11Select seq KP965573.1 | Bacterium 4L13 16S ribosomal RNA gene, partial sequence | 1773 | 1773 | 99% | 0.0 | 99% | KP965573.1 |
| 12Select seq GQ073426.1 | Uncultured bacterium clone nbw208h09c1 16S ribosomal RNA gene, partial sequence | 1772 | 1772 | 99% | 0.0 | 99% | GQ073426.1 |
| 13Select seq JX994123.1 | Paenibacillus sp. Q1-R10 16S ribosomal RNA gene, partial sequence | 1768 | 1768 | 98% | 0.0 | 99% | JX994123.1 |
| 14Select seq EU723825.1 | Paenibacillus sp. HDDMM03 16S ribosomal RNA gene, partial sequence | 1760 | 1760 | 99% | 0.0 | 99% | EU723825.1 |
| 15Select seq EU003588.1 | Paenibacillus sp. NCCB 100172 16S ribosomal RNA gene, partial sequence | 1760 | 1760 | 99% | 0.0 | 99% | EU003588.1 |
| 16Select seq KP965572.1 | Bacterium 4R1 16S ribosomal RNA gene, partial sequence | 1755 | 1755 | 99% | 0.0 | 98% | KP965572.1 |
| 17Select seq HQ143281.1 | Uncultured bacterium clone 10-9BM 16S ribosomal RNA gene, partial sequence | 1753 | 1753 | 99% | 0.0 | 98% | HQ143281.1 |
| 18Select seq JF185937.1 | Uncultured bacterium clone ncd2164d05c1 16S ribosomal RNA gene, partial sequence | 1749 | 1749 | 99% | 0.0 | 98% | JF185937.1 |
| 19Select seq HQ661869.1 | Paenibacillus illinoisensis strain WR\_17 16S ribosomal RNA gene, partial sequence | 1746 | 1746 | 95% | 0.0 | 99% | HQ661869.1 |
| 20Select seq JF185833.1 | Uncultured bacterium clone ncd2162h02c1 16S ribosomal RNA gene, partial sequence | 1744 | 1744 | 99% | 0.0 | 98% | JF185833.1 |
| 21Select seq JF185844.1 | Uncultured bacterium clone ncd2163a03c1 16S ribosomal RNA gene, partial sequence | 1738 | 1738 | 99% | 0.0 | 98% | JF185844.1 |
| 22Select seq NR\_134783.2 | Paenibacillus nicotianae strain YIM h-19 16S ribosomal RNA, complete sequence | 1733 | 1733 | 99% | 0.0 | 98% | NR\_134783.2 |
| 23Select seq KJ792471.1 | Paenibacillus nicotianae strain YIM h-19 16S ribosomal RNA gene, partial sequence | 1733 | 1733 | 99% | 0.0 | 98% | KJ792471.1 |
| 24Select seq NR\_145628.1 | Paenibacillus kyungheensis strain DCY88 16S ribosomal RNA, partial sequence | 1724 | 1724 | 98% | 0.0 | 98% | NR\_145628.1 |
| 25Select seq KF793934.1 | Paenibacillus kyungheensis strain DCY88 16S ribosomal RNA gene, partial sequence | 1724 | 1724 | 98% | 0.0 | 98% | KF793934.1 |
| 26Select seq KR263165.1 | Paenibacillus sp. R1 16S ribosomal RNA gene, partial sequence | 1722 | 1722 | 99% | 0.0 | 98% | KR263165.1 |
| 27Select seq NR\_109318.1 | Paenibacillus hordei strain RH-N24 16S ribosomal RNA gene, partial sequence | 1720 | 1720 | 99% | 0.0 | 98% | NR\_109318.1 |
| 28Select seq LM994777.1 | Paenibacillus illinoisensis genomic DNA containing 16S-23S intergenic spacer region, strain PuiB5.2 | 1690 | 1690 | 93% | 0.0 | 99% | LM994777.1 |
| 29Select seq HE587985.1 | Paenibacillus illinoisensis partial 16S rRNA gene, isolate C5 1197 | 1677 | 1677 | 93% | 0.0 | 99% | HE587985.1 |
| 30Select seq HF566260.1 | Paenibacillus sp. SC22 partial 16S rRNA gene, isolate SC22 | 1650 | 1650 | 94% | 0.0 | 98% | HF566260.1 |
| 31Select seq KP889056.1 | Paenibacillus sp. UPUM12 16S ribosomal RNA gene, partial sequence | 1640 | 1640 | 94% | 0.0 | 98% | KP889056.1 |
| 32Select seq HE587984.1 | Paenibacillus illinoisensis partial 16S rRNA gene, isolate C5 1196 | 1639 | 1639 | 95% | 0.0 | 98% | HE587984.1 |
| 33Select seq KJ528493.1 | Paenibacillus hordei strain poapink1 16S ribosomal RNA gene, partial sequence | 1635 | 1635 | 89% | 0.0 | 99% | KJ528493.1 |
| 34Select seq HQ143315.1 | Uncultured bacterium clone 56-9BM 16S ribosomal RNA gene, partial sequence | 1613 | 1613 | 90% | 0.0 | 99% | HQ143315.1 |
| 35Select seq JN899576.1 | Paenibacillus sp. WS26 16S ribosomal RNA gene, partial sequence | 1587 | 1587 | 98% | 0.0 | 95% | JN899576.1 |
| 36Select seq KT205397.1 | Paenibacillus sp. H316 16S ribosomal RNA gene, partial sequence | 1583 | 1583 | 98% | 0.0 | 96% | KT205397.1 |
| 37Select seq JN899577.1 | Paenibacillus sp. WS28 16S ribosomal RNA gene, partial sequence | 1583 | 1583 | 99% | 0.0 | 95% | JN899577.1 |
| 38Select seq JN899571.1 | Paenibacillus sp. WS16 16S ribosomal RNA gene, partial sequence | 1583 | 1583 | 99% | 0.0 | 95% | JN899571.1 |
| 39Select seq JQ236815.1 | Paenibacillus durus strain hswX78 16S ribosomal RNA gene, partial sequence | 1583 | 1583 | 99% | 0.0 | 95% | JQ236815.1 |
| 40Select seq KY111475.1 | Paenibacillus sp. strain B1 16S ribosomal RNA gene, partial sequence | 1581 | 1581 | 98% | 0.0 | 95% | KY111475.1 |
| 41Select seq EU912463.1 | Paenibacillus sp. BL13 16S ribosomal RNA gene, partial sequence | 1581 | 1581 | 98% | 0.0 | 95% | EU912463.1 |
| 42Select seq MG266466.1 | Paenibacillus illinoisensis strain Z002 16S ribosomal RNA gene, partial sequence | 1580 | 1580 | 98% | 0.0 | 95% | MG266466.1 |
| 43Select seq KP702927.1 | Paenibacillus sp. EN-26 16S ribosomal RNA gene, partial sequence | 1580 | 1580 | 98% | 0.0 | 95% | KP702927.1 |
| 44Select seq KP126817.1 | Paenibacillus sp. A10 16S ribosomal RNA gene, partial sequence | 1578 | 1578 | 99% | 0.0 | 95% | KP126817.1 |
| 45Select seq KR152299.1 | Paenibacillus illinoisensis strain SBANHCa16 16S ribosomal RNA gene, partial sequence | 1578 | 1578 | 99% | 0.0 | 95% | KR152299.1 |
| 46Select seq KR996781.1 | Bacterium enrichment culture clone JYJ-11 16S ribosomal RNA gene, partial sequence | 1578 | 1578 | 99% | 0.0 | 95% | KR996781.1 |
| 47Select seq KF010797.1 | Paenibacillus illinoisensis strain P9S 16S ribosomal RNA gene, partial sequence | 1578 | 1578 | 99% | 0.0 | 95% | KF010797.1 |
| 48Select seq AB733580.1 | Paenibacillus sp. MBEU37 gene for 16S rRNA, partial sequence | 1578 | 1578 | 99% | 0.0 | 95% | AB733580.1 |
| 49Select seq JQ579623.1 | Paenibacillus illinoisensis strain F24 16S ribosomal RNA gene, partial sequence | 1578 | 1578 | 99% | 0.0 | 95% | JQ579623.1 |
| 50Select seq EU912464.1 | Paenibacillus sp. BL17-2 16S ribosomal RNA gene, partial sequence | 1578 | 1578 | 99% | 0.0 | 95% | EU912464.1 |
| 51Select seq FJ696708.1 | Paenibacillus durus strain YUPP-3 16S ribosomal RNA gene, partial sequence | 1578 | 1578 | 99% | 0.0 | 95% | FJ696708.1 |
| 52Select seq FN422001.1 | Paenibacillus illinoisensis partial 16S rRNA gene, strain IB-1087 | 1578 | 1578 | 99% | 0.0 | 95% | FN422001.1 |
| 53Select seq KF840379.1 | Paenibacillus sp. CRRI-82\_WR13B 16S ribosomal RNA gene, partial sequence | 1576 | 1576 | 99% | 0.0 | 95% | KF840379.1 |
| 54Select seq JN899578.1 | Paenibacillus sp. WS30 16S ribosomal RNA gene, partial sequence | 1576 | 1576 | 98% | 0.0 | 95% | JN899578.1 |
| 55Select seq JN867752.1 | Paenibacillus illinoisensis strain 3728 16S ribosomal RNA gene, partial sequence | 1576 | 1576 | 98% | 0.0 | 95% | JN867752.1 |
| 56Select seq NR\_040884.1 | Paenibacillus illinoisensis strain JCM 9907 16S ribosomal RNA gene, partial sequence | 1576 | 1576 | 99% | 0.0 | 95% | NR\_040884.1 |
| 57Select seq KX350030.1 | Paenibacillus xylanilyticus strain F2-2-22 16S ribosomal RNA gene, partial sequence | 1574 | 1574 | 98% | 0.0 | 95% | KX350030.1 |
| 58Select seq NR\_113828.1 | Paenibacillus illinoisensis strain NBRC 15959 16S ribosomal RNA gene, partial sequence | 1574 | 1574 | 99% | 0.0 | 95% | NR\_113828.1 |
| 59Select seq KM817264.1 | Paenibacillus illinoisensis strain IHB B 15700 16S ribosomal RNA gene, partial sequence | 1572 | 1572 | 99% | 0.0 | 95% | KM817264.1 |
| 60Select seq KF054892.1 | Paenibacillus durus strain IARI-IIWP-40 16S ribosomal RNA gene, partial sequence | 1572 | 1572 | 99% | 0.0 | 95% | KF054892.1 |
| 61Select seq JQ063001.1 | Paenibacillus sp. XGL212 16S ribosomal RNA gene, partial sequence | 1572 | 1572 | 99% | 0.0 | 95% | JQ063001.1 |
| 62Select seq JF892727.1 | Paenibacillus sp. F37 16S ribosomal RNA gene, partial sequence | 1572 | 1572 | 99% | 0.0 | 95% | JF892727.1 |
| 63Select seq KJ769171.1 | Paenibacillus sp. THK-CF25 16S ribosomal RNA gene, partial sequence | 1570 | 1570 | 98% | 0.0 | 95% | KJ769171.1 |
| 64Select seq KJ577635.1 | Paenibacillus illinoisensis strain AAU M 17 16S ribosomal RNA gene, partial sequence | 1570 | 1570 | 98% | 0.0 | 95% | KJ577635.1 |
| 65Select seq AB680852.1 | Paenibacillus illinoisensis gene for 16S rRNA, partial sequence, strain: NBRC 15379 | 1570 | 1570 | 99% | 0.0 | 95% | AB680852.1 |
| 66Select seq GU080334.1 | Paenibacillus sp. XP13 16S ribosomal RNA gene, partial sequence | 1570 | 1570 | 98% | 0.0 | 95% | GU080334.1 |
| 67Select seq KF535141.1 | Paenibacillus xylanilyticus strain BAB-1610 16S ribosomal RNA gene, partial sequence | 1568 | 1568 | 98% | 0.0 | 95% | KF535141.1 |
| 68Select seq KR856190.1 | Paenibacillus illinoisensis strain C1 16S ribosomal RNA gene, partial sequence | 1567 | 1567 | 98% | 0.0 | 95% | KR856190.1 |
| 69Select seq KR856189.1 | Paenibacillus illinoisensis strain D4 16S ribosomal RNA gene, partial sequence | 1567 | 1567 | 98% | 0.0 | 95% | KR856189.1 |
| 70Select seq KP670215.1 | Paenibacillus sp. DB14410 16S ribosomal RNA gene, partial sequence | 1567 | 1567 | 98% | 0.0 | 95% | KP670215.1 |
| 71Select seq LC011515.1 | Paenibacillus sp. MTB37-3 gene for 16S ribosomal RNA, partial sequence | 1567 | 1567 | 99% | 0.0 | 95% | LC011515.1 |
| 72Select seq KJ951054.1 | Paenibacillus illinoisensis strain YZ29 16S ribosomal RNA gene, partial sequence | 1567 | 1567 | 99% | 0.0 | 95% | KJ951054.1 |
| 73Select seq KJ600908.1 | Uncultured bacterium clone 18B 16S ribosomal RNA gene, partial sequence | 1567 | 1567 | 99% | 0.0 | 95% | KJ600908.1 |
| 74Select seq FJ232954.1 | Paenibacillus sp. ZY-8 16S ribosomal RNA gene, partial sequence | 1567 | 1567 | 99% | 0.0 | 95% | FJ232954.1 |
| 75Select seq DQ105965.1 | P-decomposing bacterium 1 16S ribosomal RNA gene, partial sequence | 1567 | 1567 | 99% | 0.0 | 95% | DQ105965.1 |
| 76Select seq KF475708.1 | Bacterium R-22 16S ribosomal RNA gene, partial sequence | 1565 | 1565 | 98% | 0.0 | 95% | KF475708.1 |
| 77Select seq GQ202680.1 | Paenibacillus sp. U5 STR 16S ribosomal RNA gene, partial sequence | 1565 | 1565 | 98% | 0.0 | 95% | GQ202680.1 |
| 78Select seq KC000067.1 | Uncultured bacterium clone Water1 16S ribosomal RNA gene, partial sequence | 1563 | 1563 | 99% | 0.0 | 95% | KC000067.1 |
| 79Select seq KP126829.1 | Paenibacillus sp. CF7 16S ribosomal RNA gene, partial sequence | 1561 | 1561 | 99% | 0.0 | 95% | KP126829.1 |
| 80Select seq KX825907.1 | Paenibacillus sp. strain I2746 16S ribosomal RNA gene, partial sequence | 1561 | 1561 | 98% | 0.0 | 95% | KX825907.1 |
| 81Select seq LC057692.1 | Paenibacillus sp. TBTK040501B gene for 16S ribosomal RNA, partial sequence | 1561 | 1561 | 99% | 0.0 | 95% | LC057692.1 |
| 82Select seq KP172212.1 | Paenibacillus sp. EgN2201 16S ribosomal RNA gene, partial sequence | 1561 | 1561 | 99% | 0.0 | 95% | KP172212.1 |
| 83Select seq AB748941.1 | Paenibacillus xylanilyticus gene for 16S ribosomal RNA, partial sequence, strain: AECSP02 | 1561 | 1561 | 98% | 0.0 | 95% | AB748941.1 |
| 84Select seq AB366401.1 | Paenibacillus pabuli gene for 16S ribosomal RNA, partial sequence, strain: SW12 | 1561 | 1561 | 99% | 0.0 | 95% | AB366401.1 |
| 85Select seq AB366400.1 | Paenibacillus pabuli gene for 16S ribosomal RNA, partial sequence, strain: SW10 | 1561 | 1561 | 99% | 0.0 | 95% | AB366400.1 |
| 86Select seq EU218535.1 | Paenibacillus sp. K1 16S ribosomal RNA gene, complete sequence | 1561 | 1561 | 99% | 0.0 | 95% | EU218535.1 |
| 87Select seq DQ288948.1 | Paenibacillus pabuli isolate TG12 16S ribosomal RNA gene, partial sequence | 1561 | 1561 | 99% | 0.0 | 95% | DQ288948.1 |
| 88Select seq MF948342.1 | Bacillus sp. strain FJAT-29882 16S ribosomal RNA gene, partial sequence | 1559 | 1559 | 98% | 0.0 | 95% | MF948342.1 |
| 89Select seq KX212148.1 | Uncultured bacterium clone HT3 16S ribosomal RNA gene, partial sequence | 1559 | 1559 | 98% | 0.0 | 95% | KX212148.1 |
| 90Select seq KM019819.1 | Paenibacillus taichungensis strain F9 16S ribosomal RNA gene, partial sequence | 1559 | 1559 | 98% | 0.0 | 95% | KM019819.1 |
| 91Select seq KF848956.1 | Paenibacillus sp. FZ104 16S ribosomal RNA gene, partial sequence | 1559 | 1559 | 99% | 0.0 | 95% | KF848956.1 |
| 92Select seq NR\_115624.1 | Paenibacillus illinoisensis strain NRRL NRS-1356 16S ribosomal RNA, partial sequence | 1559 | 1559 | 99% | 0.0 | 95% | NR\_115624.1 |
| 93Select seq AB110989.1 | Paenibacillus sp. W-61 gene for 16S rRNA, partial sequence | 1559 | 1559 | 98% | 0.0 | 95% | AB110989.1 |
| 94Select seq KY622502.1 | Paenibacillus sp. strain LB033 16S ribosomal RNA gene, partial sequence | 1555 | 1555 | 98% | 0.0 | 95% | KY622502.1 |
| 95Select seq MG266469.1 | Paenibacillus xylanilyticus strain Z007 16S ribosomal RNA gene, partial sequence | 1555 | 1555 | 99% | 0.0 | 95% | MG266469.1 |
| 96Select seq MG266463.1 | Paenibacillus xylanilyticus strain M033 16S ribosomal RNA gene, partial sequence | 1555 | 1555 | 99% | 0.0 | 95% | MG266463.1 |
| 97Select seq MG266462.1 | Paenibacillus xylanilyticus strain M032 16S ribosomal RNA gene, partial sequence | 1555 | 1555 | 99% | 0.0 | 95% | MG266462.1 |
| 98Select seq MG266460.1 | Paenibacillus xylanilyticus strain M030 16S ribosomal RNA gene, partial sequence | 1555 | 1555 | 99% | 0.0 | 95% | MG266460.1 |
| 99Select seq MG027626.1 | Paenibacillus illinoisensis strain VBE24 16S ribosomal RNA gene, partial sequence | 1555 | 1555 | 98% | 0.0 | 95% | MG027626.1 |
| 100Select seq FJ006870.1 | Paenibacillus sp. WPCB023 16S ribosomal RNA gene, partial sequence | 1554 | 1554 | 99% | 0.0 | 95% | FJ006870.1 |


## Alignments

Loading alignment... for sequences gi|915515201,gi|915512955,gi|631782762,gi|846924131,gi|1214741488 Reading indexes 1-5

Download

FASTA (complete sequence)

FASTA (aligned sequences)

GenBank (complete sequence)

Continue
Cancel

GenBankGraphics

Next
Previous
Descriptions

Paenibacillus sp. 3Cp1 16S ribosomal RNA gene, partial sequence

Sequence ID: KP852522.1Length: 1434Number of Matches: 1

Related Information

Range 1: 12 to 1004GenBankGraphics

Next Match
Previous Match
First Match

Alignment statistics for match #1

| Score | Expect | Identities | Gaps | Strand | Frame |
| --- | --- | --- | --- | --- | --- |
| 1831 bits(991) | 0.0() | 992/993(99%) | 0/993(0%) | Plus/Plus |  |

Features:

```
Query  9     AGTCGAGCGGAGTTGANGAGAAGCTTGCTTCTTTGATGCTTAGCGGCGGACGGGTGAGTA  68
             |||||||||||||||| |||||||||||||||||||||||||||||||||||||||||||
Sbjct  12    AGTCGAGCGGAGTTGACGAGAAGCTTGCTTCTTTGATGCTTAGCGGCGGACGGGTGAGTA  71

Query  69    ACACGTAGGCAACCTGCCCTCAAGCTTGGGACAACTACCGGAAACGGTAGCTAATACCGA  128
             ||||||||||||||||||||||||||||||||||||||||||||||||||||||||||||
Sbjct  72    ACACGTAGGCAACCTGCCCTCAAGCTTGGGACAACTACCGGAAACGGTAGCTAATACCGA  131

Query  129   ATACATGATTTGTTCGCCTGAACGAATTTGGAAAGACGGAGCAATCTGTCACTTGAGGAT  188
             ||||||||||||||||||||||||||||||||||||||||||||||||||||||||||||
Sbjct  132   ATACATGATTTGTTCGCCTGAACGAATTTGGAAAGACGGAGCAATCTGTCACTTGAGGAT  191

Query  189   GGGCCTGCGGCGCATTAGCTAGTTGGTGAGGTAACGGCTCACCAAGGCGACGATGCGTAG  248
             ||||||||||||||||||||||||||||||||||||||||||||||||||||||||||||
Sbjct  192   GGGCCTGCGGCGCATTAGCTAGTTGGTGAGGTAACGGCTCACCAAGGCGACGATGCGTAG  251

Query  249   CCGACCTGAGAGGGTGATCGGCCACACTGGGACTGAGACACGGCCCAGACTCCTACGGGA  308
             ||||||||||||||||||||||||||||||||||||||||||||||||||||||||||||
Sbjct  252   CCGACCTGAGAGGGTGATCGGCCACACTGGGACTGAGACACGGCCCAGACTCCTACGGGA  311

Query  309   GGCAGCAGTAGGGAATCTTCCGCAATGGACGCAAGTCTGACGGAGCAACGCCGCGTGAGT  368
             ||||||||||||||||||||||||||||||||||||||||||||||||||||||||||||
Sbjct  312   GGCAGCAGTAGGGAATCTTCCGCAATGGACGCAAGTCTGACGGAGCAACGCCGCGTGAGT  371

Query  369   GATGAAGGTTTTCGGATCGTAAAGCTCTGTTGCCAGGGAAGAACGTCCGATAGAGTAACT  428
             ||||||||||||||||||||||||||||||||||||||||||||||||||||||||||||
Sbjct  372   GATGAAGGTTTTCGGATCGTAAAGCTCTGTTGCCAGGGAAGAACGTCCGATAGAGTAACT  431

Query  429   GCTATCGGAGTGACGGTACCTGAGAAGAAAGCCCCGGCTAACTACGTGCCAGCAGCCGCG  488
             ||||||||||||||||||||||||||||||||||||||||||||||||||||||||||||
Sbjct  432   GCTATCGGAGTGACGGTACCTGAGAAGAAAGCCCCGGCTAACTACGTGCCAGCAGCCGCG  491

Query  489   GTAATACGTAGGGGGCAAGCGTTGTCCGGAATTATTGGGCGTAAAGCGCGCGCAGGCGGC  548
             ||||||||||||||||||||||||||||||||||||||||||||||||||||||||||||
Sbjct  492   GTAATACGTAGGGGGCAAGCGTTGTCCGGAATTATTGGGCGTAAAGCGCGCGCAGGCGGC  551

Query  549   TTTTTAAGTCCGGTGTCACAGCCCAAGGCTCAACCTTGGGTCGCACTGGAAACTGGAGAG  608
             ||||||||||||||||||||||||||||||||||||||||||||||||||||||||||||
Sbjct  552   TTTTTAAGTCCGGTGTCACAGCCCAAGGCTCAACCTTGGGTCGCACTGGAAACTGGAGAG  611

Query  609   CTTGAGTACAGAAGAGGAAAGTGGAATTCCACGTGTAGCGGTGAAATGCGTAGAGATGTG  668
             ||||||||||||||||||||||||||||||||||||||||||||||||||||||||||||
Sbjct  612   CTTGAGTACAGAAGAGGAAAGTGGAATTCCACGTGTAGCGGTGAAATGCGTAGAGATGTG  671

Query  669   GAGGAACACCAGTGGCGAAGGCGACTTTCTGGGCTGTAACTGACGCTGAGGCGCGAAAGC  728
             ||||||||||||||||||||||||||||||||||||||||||||||||||||||||||||
Sbjct  672   GAGGAACACCAGTGGCGAAGGCGACTTTCTGGGCTGTAACTGACGCTGAGGCGCGAAAGC  731

Query  729   GTGGGGAGCAAACAGGATTAGATACCCTGGTAGTCCACGCCGTAAACGATGAATGCTAGG  788
             ||||||||||||||||||||||||||||||||||||||||||||||||||||||||||||
Sbjct  732   GTGGGGAGCAAACAGGATTAGATACCCTGGTAGTCCACGCCGTAAACGATGAATGCTAGG  791

Query  789   TGTTAGGGGTTTCGATACCCTTGGTGCCGAAGTTAACACATTAAGCATTCCGCCTGGGGA  848
             ||||||||||||||||||||||||||||||||||||||||||||||||||||||||||||
Sbjct  792   TGTTAGGGGTTTCGATACCCTTGGTGCCGAAGTTAACACATTAAGCATTCCGCCTGGGGA  851

Query  849   GTACGGTCGCAAGACTGAAACTCAAAGGAATTGACGGGGACCCGCACAAGCAGTGGAGTA  908
             ||||||||||||||||||||||||||||||||||||||||||||||||||||||||||||
Sbjct  852   GTACGGTCGCAAGACTGAAACTCAAAGGAATTGACGGGGACCCGCACAAGCAGTGGAGTA  911

Query  909   TGTGGTTTAATTCGAAGCAACGCGAAGAACCTTACCAAGTCTTGACATCCCTTTGACCGG  968
             ||||||||||||||||||||||||||||||||||||||||||||||||||||||||||||
Sbjct  912   TGTGGTTTAATTCGAAGCAACGCGAAGAACCTTACCAAGTCTTGACATCCCTTTGACCGG  971

Query  969   ACTAGAGATAGTCTTTTCCTTCGGGACAAAGGA  1001
             |||||||||||||||||||||||||||||||||
Sbjct  972   ACTAGAGATAGTCTTTTCCTTCGGGACAAAGGA  1004
```

Download

FASTA (complete sequence)

FASTA (aligned sequences)

GenBank (complete sequence)

Continue
Cancel

GenBankGraphics

Next
Previous
Descriptions

Uncultured bacterium clone 4L13 16S ribosomal RNA gene, partial sequence

Sequence ID: KP843054.1Length: 1460Number of Matches: 1

Related Information

Range 1: 24 to 1016GenBankGraphics

Next Match
Previous Match
First Match

Alignment statistics for match #1

| Score | Expect | Identities | Gaps | Strand | Frame |
| --- | --- | --- | --- | --- | --- |
| 1831 bits(991) | 0.0() | 992/993(99%) | 0/993(0%) | Plus/Plus |  |

Features:

```
Query  9     AGTCGAGCGGAGTTGANGAGAAGCTTGCTTCTTTGATGCTTAGCGGCGGACGGGTGAGTA  68
             |||||||||||||||| |||||||||||||||||||||||||||||||||||||||||||
Sbjct  24    AGTCGAGCGGAGTTGACGAGAAGCTTGCTTCTTTGATGCTTAGCGGCGGACGGGTGAGTA  83

Query  69    ACACGTAGGCAACCTGCCCTCAAGCTTGGGACAACTACCGGAAACGGTAGCTAATACCGA  128
             ||||||||||||||||||||||||||||||||||||||||||||||||||||||||||||
Sbjct  84    ACACGTAGGCAACCTGCCCTCAAGCTTGGGACAACTACCGGAAACGGTAGCTAATACCGA  143

Query  129   ATACATGATTTGTTCGCCTGAACGAATTTGGAAAGACGGAGCAATCTGTCACTTGAGGAT  188
             ||||||||||||||||||||||||||||||||||||||||||||||||||||||||||||
Sbjct  144   ATACATGATTTGTTCGCCTGAACGAATTTGGAAAGACGGAGCAATCTGTCACTTGAGGAT  203

Query  189   GGGCCTGCGGCGCATTAGCTAGTTGGTGAGGTAACGGCTCACCAAGGCGACGATGCGTAG  248
             ||||||||||||||||||||||||||||||||||||||||||||||||||||||||||||
Sbjct  204   GGGCCTGCGGCGCATTAGCTAGTTGGTGAGGTAACGGCTCACCAAGGCGACGATGCGTAG  263

Query  249   CCGACCTGAGAGGGTGATCGGCCACACTGGGACTGAGACACGGCCCAGACTCCTACGGGA  308
             ||||||||||||||||||||||||||||||||||||||||||||||||||||||||||||
Sbjct  264   CCGACCTGAGAGGGTGATCGGCCACACTGGGACTGAGACACGGCCCAGACTCCTACGGGA  323

Query  309   GGCAGCAGTAGGGAATCTTCCGCAATGGACGCAAGTCTGACGGAGCAACGCCGCGTGAGT  368
             ||||||||||||||||||||||||||||||||||||||||||||||||||||||||||||
Sbjct  324   GGCAGCAGTAGGGAATCTTCCGCAATGGACGCAAGTCTGACGGAGCAACGCCGCGTGAGT  383

Query  369   GATGAAGGTTTTCGGATCGTAAAGCTCTGTTGCCAGGGAAGAACGTCCGATAGAGTAACT  428
             ||||||||||||||||||||||||||||||||||||||||||||||||||||||||||||
Sbjct  384   GATGAAGGTTTTCGGATCGTAAAGCTCTGTTGCCAGGGAAGAACGTCCGATAGAGTAACT  443

Query  429   GCTATCGGAGTGACGGTACCTGAGAAGAAAGCCCCGGCTAACTACGTGCCAGCAGCCGCG  488
             ||||||||||||||||||||||||||||||||||||||||||||||||||||||||||||
Sbjct  444   GCTATCGGAGTGACGGTACCTGAGAAGAAAGCCCCGGCTAACTACGTGCCAGCAGCCGCG  503

Query  489   GTAATACGTAGGGGGCAAGCGTTGTCCGGAATTATTGGGCGTAAAGCGCGCGCAGGCGGC  548
             ||||||||||||||||||||||||||||||||||||||||||||||||||||||||||||
Sbjct  504   GTAATACGTAGGGGGCAAGCGTTGTCCGGAATTATTGGGCGTAAAGCGCGCGCAGGCGGC  563

Query  549   TTTTTAAGTCCGGTGTCACAGCCCAAGGCTCAACCTTGGGTCGCACTGGAAACTGGAGAG  608
             ||||||||||||||||||||||||||||||||||||||||||||||||||||||||||||
Sbjct  564   TTTTTAAGTCCGGTGTCACAGCCCAAGGCTCAACCTTGGGTCGCACTGGAAACTGGAGAG  623

Query  609   CTTGAGTACAGAAGAGGAAAGTGGAATTCCACGTGTAGCGGTGAAATGCGTAGAGATGTG  668
             ||||||||||||||||||||||||||||||||||||||||||||||||||||||||||||
Sbjct  624   CTTGAGTACAGAAGAGGAAAGTGGAATTCCACGTGTAGCGGTGAAATGCGTAGAGATGTG  683

Query  669   GAGGAACACCAGTGGCGAAGGCGACTTTCTGGGCTGTAACTGACGCTGAGGCGCGAAAGC  728
             ||||||||||||||||||||||||||||||||||||||||||||||||||||||||||||
Sbjct  684   GAGGAACACCAGTGGCGAAGGCGACTTTCTGGGCTGTAACTGACGCTGAGGCGCGAAAGC  743

Query  729   GTGGGGAGCAAACAGGATTAGATACCCTGGTAGTCCACGCCGTAAACGATGAATGCTAGG  788
             ||||||||||||||||||||||||||||||||||||||||||||||||||||||||||||
Sbjct  744   GTGGGGAGCAAACAGGATTAGATACCCTGGTAGTCCACGCCGTAAACGATGAATGCTAGG  803

Query  789   TGTTAGGGGTTTCGATACCCTTGGTGCCGAAGTTAACACATTAAGCATTCCGCCTGGGGA  848
             ||||||||||||||||||||||||||||||||||||||||||||||||||||||||||||
Sbjct  804   TGTTAGGGGTTTCGATACCCTTGGTGCCGAAGTTAACACATTAAGCATTCCGCCTGGGGA  863

Query  849   GTACGGTCGCAAGACTGAAACTCAAAGGAATTGACGGGGACCCGCACAAGCAGTGGAGTA  908
             ||||||||||||||||||||||||||||||||||||||||||||||||||||||||||||
Sbjct  864   GTACGGTCGCAAGACTGAAACTCAAAGGAATTGACGGGGACCCGCACAAGCAGTGGAGTA  923

Query  909   TGTGGTTTAATTCGAAGCAACGCGAAGAACCTTACCAAGTCTTGACATCCCTTTGACCGG  968
             ||||||||||||||||||||||||||||||||||||||||||||||||||||||||||||
Sbjct  924   TGTGGTTTAATTCGAAGCAACGCGAAGAACCTTACCAAGTCTTGACATCCCTTTGACCGG  983

Query  969   ACTAGAGATAGTCTTTTCCTTCGGGACAAAGGA  1001
             |||||||||||||||||||||||||||||||||
Sbjct  984   ACTAGAGATAGTCTTTTCCTTCGGGACAAAGGA  1016
```

Download

FASTA (complete sequence)

FASTA (aligned sequences)

GenBank (complete sequence)

Continue
Cancel

GenBankGraphics

Next
Previous
Descriptions

Paenibacillus sp. TA\_EU partial 16S rRNA gene, strain TA\_EU

Sequence ID: HG942127.1Length: 1310Number of Matches: 1

Related Information

Range 1: 3 to 995GenBankGraphics

Next Match
Previous Match
First Match

Alignment statistics for match #1

| Score | Expect | Identities | Gaps | Strand | Frame |
| --- | --- | --- | --- | --- | --- |
| 1831 bits(991) | 0.0() | 992/993(99%) | 0/993(0%) | Plus/Plus |  |

Features:

```
Query  9     AGTCGAGCGGAGTTGANGAGAAGCTTGCTTCTTTGATGCTTAGCGGCGGACGGGTGAGTA  68
             |||||||||||||||| |||||||||||||||||||||||||||||||||||||||||||
Sbjct  3     AGTCGAGCGGAGTTGACGAGAAGCTTGCTTCTTTGATGCTTAGCGGCGGACGGGTGAGTA  62

Query  69    ACACGTAGGCAACCTGCCCTCAAGCTTGGGACAACTACCGGAAACGGTAGCTAATACCGA  128
             ||||||||||||||||||||||||||||||||||||||||||||||||||||||||||||
Sbjct  63    ACACGTAGGCAACCTGCCCTCAAGCTTGGGACAACTACCGGAAACGGTAGCTAATACCGA  122

Query  129   ATACATGATTTGTTCGCCTGAACGAATTTGGAAAGACGGAGCAATCTGTCACTTGAGGAT  188
             ||||||||||||||||||||||||||||||||||||||||||||||||||||||||||||
Sbjct  123   ATACATGATTTGTTCGCCTGAACGAATTTGGAAAGACGGAGCAATCTGTCACTTGAGGAT  182

Query  189   GGGCCTGCGGCGCATTAGCTAGTTGGTGAGGTAACGGCTCACCAAGGCGACGATGCGTAG  248
             ||||||||||||||||||||||||||||||||||||||||||||||||||||||||||||
Sbjct  183   GGGCCTGCGGCGCATTAGCTAGTTGGTGAGGTAACGGCTCACCAAGGCGACGATGCGTAG  242

Query  249   CCGACCTGAGAGGGTGATCGGCCACACTGGGACTGAGACACGGCCCAGACTCCTACGGGA  308
             ||||||||||||||||||||||||||||||||||||||||||||||||||||||||||||
Sbjct  243   CCGACCTGAGAGGGTGATCGGCCACACTGGGACTGAGACACGGCCCAGACTCCTACGGGA  302

Query  309   GGCAGCAGTAGGGAATCTTCCGCAATGGACGCAAGTCTGACGGAGCAACGCCGCGTGAGT  368
             ||||||||||||||||||||||||||||||||||||||||||||||||||||||||||||
Sbjct  303   GGCAGCAGTAGGGAATCTTCCGCAATGGACGCAAGTCTGACGGAGCAACGCCGCGTGAGT  362

Query  369   GATGAAGGTTTTCGGATCGTAAAGCTCTGTTGCCAGGGAAGAACGTCCGATAGAGTAACT  428
             ||||||||||||||||||||||||||||||||||||||||||||||||||||||||||||
Sbjct  363   GATGAAGGTTTTCGGATCGTAAAGCTCTGTTGCCAGGGAAGAACGTCCGATAGAGTAACT  422

Query  429   GCTATCGGAGTGACGGTACCTGAGAAGAAAGCCCCGGCTAACTACGTGCCAGCAGCCGCG  488
             ||||||||||||||||||||||||||||||||||||||||||||||||||||||||||||
Sbjct  423   GCTATCGGAGTGACGGTACCTGAGAAGAAAGCCCCGGCTAACTACGTGCCAGCAGCCGCG  482

Query  489   GTAATACGTAGGGGGCAAGCGTTGTCCGGAATTATTGGGCGTAAAGCGCGCGCAGGCGGC  548
             ||||||||||||||||||||||||||||||||||||||||||||||||||||||||||||
Sbjct  483   GTAATACGTAGGGGGCAAGCGTTGTCCGGAATTATTGGGCGTAAAGCGCGCGCAGGCGGC  542

Query  549   TTTTTAAGTCCGGTGTCACAGCCCAAGGCTCAACCTTGGGTCGCACTGGAAACTGGAGAG  608
             ||||||||||||||||||||||||||||||||||||||||||||||||||||||||||||
Sbjct  543   TTTTTAAGTCCGGTGTCACAGCCCAAGGCTCAACCTTGGGTCGCACTGGAAACTGGAGAG  602

Query  609   CTTGAGTACAGAAGAGGAAAGTGGAATTCCACGTGTAGCGGTGAAATGCGTAGAGATGTG  668
             ||||||||||||||||||||||||||||||||||||||||||||||||||||||||||||
Sbjct  603   CTTGAGTACAGAAGAGGAAAGTGGAATTCCACGTGTAGCGGTGAAATGCGTAGAGATGTG  662

Query  669   GAGGAACACCAGTGGCGAAGGCGACTTTCTGGGCTGTAACTGACGCTGAGGCGCGAAAGC  728
             ||||||||||||||||||||||||||||||||||||||||||||||||||||||||||||
Sbjct  663   GAGGAACACCAGTGGCGAAGGCGACTTTCTGGGCTGTAACTGACGCTGAGGCGCGAAAGC  722

Query  729   GTGGGGAGCAAACAGGATTAGATACCCTGGTAGTCCACGCCGTAAACGATGAATGCTAGG  788
             ||||||||||||||||||||||||||||||||||||||||||||||||||||||||||||
Sbjct  723   GTGGGGAGCAAACAGGATTAGATACCCTGGTAGTCCACGCCGTAAACGATGAATGCTAGG  782

Query  789   TGTTAGGGGTTTCGATACCCTTGGTGCCGAAGTTAACACATTAAGCATTCCGCCTGGGGA  848
             ||||||||||||||||||||||||||||||||||||||||||||||||||||||||||||
Sbjct  783   TGTTAGGGGTTTCGATACCCTTGGTGCCGAAGTTAACACATTAAGCATTCCGCCTGGGGA  842

Query  849   GTACGGTCGCAAGACTGAAACTCAAAGGAATTGACGGGGACCCGCACAAGCAGTGGAGTA  908
             ||||||||||||||||||||||||||||||||||||||||||||||||||||||||||||
Sbjct  843   GTACGGTCGCAAGACTGAAACTCAAAGGAATTGACGGGGACCCGCACAAGCAGTGGAGTA  902

Query  909   TGTGGTTTAATTCGAAGCAACGCGAAGAACCTTACCAAGTCTTGACATCCCTTTGACCGG  968
             ||||||||||||||||||||||||||||||||||||||||||||||||||||||||||||
Sbjct  903   TGTGGTTTAATTCGAAGCAACGCGAAGAACCTTACCAAGTCTTGACATCCCTTTGACCGG  962

Query  969   ACTAGAGATAGTCTTTTCCTTCGGGACAAAGGA  1001
             |||||||||||||||||||||||||||||||||
Sbjct  963   ACTAGAGATAGTCTTTTCCTTCGGGACAAAGGA  995
```

Download

FASTA (complete sequence)

FASTA (aligned sequences)

GenBank (complete sequence)

Continue
Cancel

GenBankGraphics

Next
Previous
Descriptions

Bacterium 4R9 16S ribosomal RNA gene, partial sequence

Sequence ID: KP965575.1Length: 1517Number of Matches: 1

Related Information

Range 1: 52 to 1046GenBankGraphics

Next Match
Previous Match
First Match

Alignment statistics for match #1

| Score | Expect | Identities | Gaps | Strand | Frame |
| --- | --- | --- | --- | --- | --- |
| 1827 bits(989) | 0.0() | 993/995(99%) | 1/995(0%) | Plus/Plus |  |

Features:

```
Query  8     AAGTCGAGCGGAGTTGANGAGAAGCTTGCTTCTTTGATGCTTAGCGGCGGACGGGTGAGT  67
             ||||||||||||||||| ||||||||||||||||||||||||||||||||||||||||||
Sbjct  52    AAGTCGAGCGGAGTTGACGAGAAGCTTGCTTCTTTGATGCTTAGCGGCGGACGGGTGAGT  111

Query  68    AACACGTAGGCAACCTGCCCTCAAGCTTGGGACAACTACCGGAAACGGTAGCTAATACCG  127
             ||||||||||||||||||||||||||||||||||||||||||||||||||||||||||||
Sbjct  112   AACACGTAGGCAACCTGCCCTCAAGCTTGGGACAACTACCGGAAACGGTAGCTAATACCG  171

Query  128   AATACATGATTTGTTCGCCTGAACGAATTTGGAAAGACGGAGCAATCTGTCACTTGAGGA  187
             ||||||||||||||||||||||||||||||||||||||||||||||||||||||||||||
Sbjct  172   AATACATGATTTGTTCGCCTGAACGAATTTGGAAAGACGGAGCAATCTGTCACTTGAGGA  231

Query  188   TGGGCCTGCGGCGCATTAGCTAGTTGGTGAGGTAACGGCTCACCAAGGCGACGATGCGTA  247
             ||||||||||||||||||||||||||||||||||||||||||||||||||||||||||||
Sbjct  232   TGGGCCTGCGGCGCATTAGCTAGTTGGTGAGGTAACGGCTCACCAAGGCGACGATGCGTA  291

Query  248   GCCGACCTGAGAGGGTGATCGGCCACACTGGGACTGAGACACGGCCCAGACTCCTACGGG  307
             ||||||||||||||||||||||||||||||||||||||||||||||||||||||||||||
Sbjct  292   GCCGACCTGAGAGGGTGATCGGCCACACTGGGACTGAGACACGGCCCAGACTCCTACGGG  351

Query  308   AGGCAGCAGTAGGGAATCTTCCGCAATGGACGCAAGTCTGACGGAGCAACGCCGCGTGAG  367
             ||||||||||||||||||||||||||||||||||||||||||||||||||||||||||||
Sbjct  352   AGGCAGCAGTAGGGAATCTTCCGCAATGGACGCAAGTCTGACGGAGCAACGCCGCGTGAG  411

Query  368   TGATGAAGGTTTTCGGATCGTAAAGCTCTGTTGCCAGGGAAGAACGTCCGATAGAGTAAC  427
             ||||||||||||||||||||||||||||||||||||||||||||||||||||||||||||
Sbjct  412   TGATGAAGGTTTTCGGATCGTAAAGCTCTGTTGCCAGGGAAGAACGTCCGATAGAGTAAC  471

Query  428   TGCTATCGGAGTGACGGTACCTGAGAAGAAAGCCCCGGCTAACTACGTGCCAGCAGCCGC  487
             ||||||||||||||||||||||||||||||||||||||||||||||||||||||||||||
Sbjct  472   TGCTATCGGAGTGACGGTACCTGAGAAGAAAGCCCCGGCTAACTACGTGCCAGCAGCCGC  531

Query  488   GGTAATACGTAGGGGGCAAGCGTTGTCCGGAATTATTGGGCGTAAAGCGCGCGCAGGCGG  547
             ||||||||||||||||||||||||||||||||||||||||||||||||||||||||||||
Sbjct  532   GGTAATACGTAGGGGGCAAGCGTTGTCCGGAATTATTGGGCGTAAAGCGCGCGCAGGCGG  591

Query  548   CTTTTTAAGTCCGGTGTCACAGCCCAAGGCTCAA-CCTTGGGTCGCACTGGAAACTGGAG  606
             |||||||||||||||||||||||||||||||||| |||||||||||||||||||||||||
Sbjct  592   CTTTTTAAGTCCGGTGTCACAGCCCAAGGCTCAACCCTTGGGTCGCACTGGAAACTGGAG  651

Query  607   AGCTTGAGTACAGAAGAGGAAAGTGGAATTCCACGTGTAGCGGTGAAATGCGTAGAGATG  666
             ||||||||||||||||||||||||||||||||||||||||||||||||||||||||||||
Sbjct  652   AGCTTGAGTACAGAAGAGGAAAGTGGAATTCCACGTGTAGCGGTGAAATGCGTAGAGATG  711

Query  667   TGGAGGAACACCAGTGGCGAAGGCGACTTTCTGGGCTGTAACTGACGCTGAGGCGCGAAA  726
             ||||||||||||||||||||||||||||||||||||||||||||||||||||||||||||
Sbjct  712   TGGAGGAACACCAGTGGCGAAGGCGACTTTCTGGGCTGTAACTGACGCTGAGGCGCGAAA  771

Query  727   GCGTGGGGAGCAAACAGGATTAGATACCCTGGTAGTCCACGCCGTAAACGATGAATGCTA  786
             ||||||||||||||||||||||||||||||||||||||||||||||||||||||||||||
Sbjct  772   GCGTGGGGAGCAAACAGGATTAGATACCCTGGTAGTCCACGCCGTAAACGATGAATGCTA  831

Query  787   GGTGTTAGGGGTTTCGATACCCTTGGTGCCGAAGTTAACACATTAAGCATTCCGCCTGGG  846
             ||||||||||||||||||||||||||||||||||||||||||||||||||||||||||||
Sbjct  832   GGTGTTAGGGGTTTCGATACCCTTGGTGCCGAAGTTAACACATTAAGCATTCCGCCTGGG  891

Query  847   GAGTACGGTCGCAAGACTGAAACTCAAAGGAATTGACGGGGACCCGCACAAGCAGTGGAG  906
             ||||||||||||||||||||||||||||||||||||||||||||||||||||||||||||
Sbjct  892   GAGTACGGTCGCAAGACTGAAACTCAAAGGAATTGACGGGGACCCGCACAAGCAGTGGAG  951

Query  907   TATGTGGTTTAATTCGAAGCAACGCGAAGAACCTTACCAAGTCTTGACATCCCTTTGACC  966
             ||||||||||||||||||||||||||||||||||||||||||||||||||||||||||||
Sbjct  952   TATGTGGTTTAATTCGAAGCAACGCGAAGAACCTTACCAAGTCTTGACATCCCTTTGACC  1011

Query  967   GGACTAGAGATAGTCTTTTCCTTCGGGACAAAGGA  1001
             |||||||||||||||||||||||||||||||||||
Sbjct  1012  GGACTAGAGATAGTCTTTTCCTTCGGGACAAAGGA  1046
```

Download

FASTA (complete sequence)

FASTA (aligned sequences)

GenBank (complete sequence)

Continue
Cancel

GenBankGraphics

Next
Previous
Descriptions

Paenibacillus sp. strain PS-K-17 16S ribosomal RNA gene, partial sequence

Sequence ID: MF443394.1Length: 1462Number of Matches: 1

Related Information

Range 1: 4 to 997GenBankGraphics

Next Match
Previous Match
First Match

Alignment statistics for match #1

| Score | Expect | Identities | Gaps | Strand | Frame |
| --- | --- | --- | --- | --- | --- |
| 1821 bits(986) | 0.0() | 991/994(99%) | 0/994(0%) | Plus/Plus |  |

Features:

```
Query  8     AAGTCGAGCGGAGTTGANGAGAAGCTTGCTTCTTTGATGCTTAGCGGCGGACGGGTGAGT  67
             ||||||||||||||||| |||||||||||||||  |||||||||||||||||||||||||
Sbjct  4     AAGTCGAGCGGAGTTGAYGAGAAGCTTGCTTCTCGGATGCTTAGCGGCGGACGGGTGAGT  63

Query  68    AACACGTAGGCAACCTGCCCTCAAGCTTGGGACAACTACCGGAAACGGTAGCTAATACCG  127
             ||||||||||||||||||||||||||||||||||||||||||||||||||||||||||||
Sbjct  64    AACACGTAGGCAACCTGCCCTCAAGCTTGGGACAACTACCGGAAACGGTAGCTAATACCG  123

Query  128   AATACATGATTTGTTCGCCTGAACGAATTTGGAAAGACGGAGCAATCTGTCACTTGAGGA  187
             ||||||||||||||||||||||||||||||||||||||||||||||||||||||||||||
Sbjct  124   AATACATGATTTGTTCGCCTGAACGAATTTGGAAAGACGGAGCAATCTGTCACTTGAGGA  183

Query  188   TGGGCCTGCGGCGCATTAGCTAGTTGGTGAGGTAACGGCTCACCAAGGCGACGATGCGTA  247
             ||||||||||||||||||||||||||||||||||||||||||||||||||||||||||||
Sbjct  184   TGGGCCTGCGGCGCATTAGCTAGTTGGTGAGGTAACGGCTCACCAAGGCGACGATGCGTA  243

Query  248   GCCGACCTGAGAGGGTGATCGGCCACACTGGGACTGAGACACGGCCCAGACTCCTACGGG  307
             ||||||||||||||||||||||||||||||||||||||||||||||||||||||||||||
Sbjct  244   GCCGACCTGAGAGGGTGATCGGCCACACTGGGACTGAGACACGGCCCAGACTCCTACGGG  303

Query  308   AGGCAGCAGTAGGGAATCTTCCGCAATGGACGCAAGTCTGACGGAGCAACGCCGCGTGAG  367
             ||||||||||||||||||||||||||||||||||||||||||||||||||||||||||||
Sbjct  304   AGGCAGCAGTAGGGAATCTTCCGCAATGGACGCAAGTCTGACGGAGCAACGCCGCGTGAG  363

Query  368   TGATGAAGGTTTTCGGATCGTAAAGCTCTGTTGCCAGGGAAGAACGTCCGATAGAGTAAC  427
             ||||||||||||||||||||||||||||||||||||||||||||||||||||||||||||
Sbjct  364   TGATGAAGGTTTTCGGATCGTAAAGCTCTGTTGCCAGGGAAGAACGTCCGATAGAGTAAC  423

Query  428   TGCTATCGGAGTGACGGTACCTGAGAAGAAAGCCCCGGCTAACTACGTGCCAGCAGCCGC  487
             ||||||||||||||||||||||||||||||||||||||||||||||||||||||||||||
Sbjct  424   TGCTATCGGAGTGACGGTACCTGAGAAGAAAGCCCCGGCTAACTACGTGCCAGCAGCCGC  483

Query  488   GGTAATACGTAGGGGGCAAGCGTTGTCCGGAATTATTGGGCGTAAAGCGCGCGCAGGCGG  547
             ||||||||||||||||||||||||||||||||||||||||||||||||||||||||||||
Sbjct  484   GGTAATACGTAGGGGGCAAGCGTTGTCCGGAATTATTGGGCGTAAAGCGCGCGCAGGCGG  543

Query  548   CTTTTTAAGTCCGGTGTCACAGCCCAAGGCTCAACCTTGGGTCGCACTGGAAACTGGAGA  607
             ||||||||||||||||||||||||||||||||||||||||||||||||||||||||||||
Sbjct  544   CTTTTTAAGTCCGGTGTCACAGCCCAAGGCTCAACCTTGGGTCGCACTGGAAACTGGAGA  603

Query  608   GCTTGAGTACAGAAGAGGAAAGTGGAATTCCACGTGTAGCGGTGAAATGCGTAGAGATGT  667
             ||||||||||||||||||||||||||||||||||||||||||||||||||||||||||||
Sbjct  604   GCTTGAGTACAGAAGAGGAAAGTGGAATTCCACGTGTAGCGGTGAAATGCGTAGAGATGT  663

Query  668   GGAGGAACACCAGTGGCGAAGGCGACTTTCTGGGCTGTAACTGACGCTGAGGCGCGAAAG  727
             ||||||||||||||||||||||||||||||||||||||||||||||||||||||||||||
Sbjct  664   GGAGGAACACCAGTGGCGAAGGCGACTTTCTGGGCTGTAACTGACGCTGAGGCGCGAAAG  723

Query  728   CGTGGGGAGCAAACAGGATTAGATACCCTGGTAGTCCACGCCGTAAACGATGAATGCTAG  787
             ||||||||||||||||||||||||||||||||||||||||||||||||||||||||||||
Sbjct  724   CGTGGGGAGCAAACAGGATTAGATACCCTGGTAGTCCACGCCGTAAACGATGAATGCTAG  783

Query  788   GTGTTAGGGGTTTCGATACCCTTGGTGCCGAAGTTAACACATTAAGCATTCCGCCTGGGG  847
             ||||||||||||||||||||||||||||||||||||||||||||||||||||||||||||
Sbjct  784   GTGTTAGGGGTTTCGATACCCTTGGTGCCGAAGTTAACACATTAAGCATTCCGCCTGGGG  843

Query  848   AGTACGGTCGCAAGACTGAAACTCAAAGGAATTGACGGGGACCCGCACAAGCAGTGGAGT  907
             ||||||||||||||||||||||||||||||||||||||||||||||||||||||||||||
Sbjct  844   AGTACGGTCGCAAGACTGAAACTCAAAGGAATTGACGGGGACCCGCACAAGCAGTGGAGT  903

Query  908   ATGTGGTTTAATTCGAAGCAACGCGAAGAACCTTACCAAGTCTTGACATCCCTTTGACCG  967
             ||||||||||||||||||||||||||||||||||||||||||||||||||||||||||||
Sbjct  904   ATGTGGTTTAATTCGAAGCAACGCGAAGAACCTTACCAAGTCTTGACATCCCTTTGACCG  963

Query  968   GACTAGAGATAGTCTTTTCCTTCGGGACAAAGGA  1001
             ||||||||||||||||||||||||||||||||||
Sbjct  964   GACTAGAGATAGTCTTTTCCTTCGGGACAAAGGA  997
```


BLAST is a registered trademark of the National Library of Medicine

Support center
Mailing list


YouTube

- National Library Of Medicine
- National Institutes Of Health
- U.S. Department of Health & Human Services
- USA.gov

### NCBI


National Center for Biotechnology Information,
 U.S. National Library of Medicine

8600 Rockville Pike,
Bethesda
 MD,
20894
USA

Policies and Guidelines
|
Contact


PreferencesTurn off

External link. Please review our privacy policy.
