# Supplementary material for: Isothermal microcalorimetry for thermal viable count of microorganisms in pure cultures and stabilized formulations
Source: BMC Microbiol. 2019 Mar 21;19:65. doi: 10.1186/s12866-019-1432-8 (PMC6429831; doi:10.1186/s12866-019-1432-8)
Supplement: Supplementary file 3 — 16S rRNA gene sequences’ BLASTN hits in zipped HTML format. (ZIP 15810 kb) [file 12866_2019_1432_MOESM3_ESM.zip › Best blastn hits/NCBI Blast_D pink R -- 31..1030 of sequence (1000.html]

NCBI Blast:D pink R -- 31..1030 of sequence (1000


- NCBI Home
- Sign in to NCBI
- Skip to Main Content
- Skip to Navigation
- About NCBI Accesskeys

U.S. National Library of Medicine

NCBI
National Center for Biotechnology Information

- My NCBI
- Sign in to NCBI
- Register
- Sign Out

BLAST ^®^ » blastn suite » RID-A4XK8G3R015


- Home
- Recent Results
- Saved Strategies
- Help

BLAST Results


Edit and Resubmit
Save Search Strategies
[Sign in above to save your search strategy]

Formatting options 


Download


How to read this page
Blast report description
Questions/comments


|  |  |
| --- | --- |
| Formatting options | |
| Show | Alignment as  HTML Plain text   Old View Reset form to defaults [?]  These options control formatting of alignments in results pages. The default is HTML, but other formats (including plain text) are available. PSSM and PssmWithParameters are representations of Position Specific Scoring Matrices and are only available for PSI-BLAST. The Advanced view option allows the database descriptions to be sorted by various indices in a table. |
| Alignment View | Pairwise Pairwise with dots for identities Query-anchored with dots for identities Query-anchored with letters for identities Flat query-anchored with dots for identities Flat query-anchored with letters for identities [?]  Choose how to view alignments. The default "pairwise" view shows how each subject sequence aligns individually to the query sequence. The "query-anchored" view shows how all subject sequences align to the query sequence. For each view type, you can choose to show "identities" (matching residues) as letters or dots. more... |
| Display | Graphical Overview   Linkout   Sequence Retrieval  NCBI-gi   CDS feature [?]  - Graphical Overview: Graphical Overview: Show graph of similar sequence regions aligned to query.   more... - NCBI-gi: Show NCBI gi identifiers. - CDS feature: Show annotated coding region and translation.   more... |
| Masking | Character:   X for protein, n for nucleotide Lower Case  Color:  Black Grey Red [?]  - Masking Character: Display masked (filtered) sequence regions as lower-case or as specific letters (N for nucleotide, P for protein). - Masking Color: Display masked sequence regions in the given color. |
| Limit results | Descriptions:  10 50 100 Graphical overview:  0 10 50 100  Alignments:  0 10 50 100 Line length:  60 90 120 150 [?]  - Descriptions: Show short descriptions for up to the given number of sequences. - Alignments: Show alignments for up to the given number of sequences, in order of statistical significance. - Line lenghth: Number of letters to show on one line in an alignment. |
|  | Organism Type common name, binomial, taxid, or group name. Only 20 top taxa will be shown.     Exclude   [?]  Show only sequences from the given organism. |
|  | Entrez query:  [?]  Show only those sequences that match the given Entrez query. more... |
|  | Expect Min:  Expect Max:  [?]  Show only sequences with expect values in the given range. more... |
|  | Percent Identity Min:  Percent Identity Max:  [?]  Show only sequences with percent identity values in the given range. |
| Format for | PSI-BLAST with inclusion threshold:  [?]  - Format for PSI-BLAST: The Position-Specific Iterated BLAST (PSI-BLAST) program performs iterative searches with a protein query,   in which sequences found in one round of search are used to build a custom score model for the next round.   more... - Inclusion Threshold: This sets the statistical significance threshold for including a sequence in the model used   by PSI-BLAST to create the PSSM on the next iteration. |

|  |  |  |  |  |  |
| --- | --- | --- | --- | --- | --- |
| Download | | | | | |
| Alignment  Text XML ASN.1 JSON Seq-align Hit Table(text) Hit Table(csv) Multiple-file XML2 Single-file XML2 Multiple-file JSON Single-file JSON SAM | Search Strategies  ASN.1 | PSSM to restart search  PSSM | [?] |  |  |

The Download link provides BLAST output that may be used as input to another program.
This includes parseable formats such as the tabular report or XML as well as the Search Strategy files read by the BLAST+ applications.
More details on the parseable (XML, tabular, and ASN.1) reports can be found at
https://www.ncbi.nlm.nih.gov/books/NBK153387/  
  

The following formats are offered under the Alignment section:  
1). "Text". Non-HTML standard BLAST report.  
2). "XML". XML report based upon the DTD at https://www.ncbi.nlm.nih.gov/data\_specs/dtd/NCBI\_BlastOutput.dtd  
3). "ASN.1". Alignment written out in Abstract Syntax Notation 1.  
4). "JSON Seq-align". Alignment written out in JSON.  
4). "Hit Table(text)". The tabular report as text.  
5). "Hit Table(csv)". The tabular report ready for import into spread-sheet programs like Excel.  
6). "XML2". New XML format described at ftp://ftp.ncbi.nlm.nih.gov/blast/documents/NEWXML/xml2.pdf.  
7). "JSON". New JSON format described at ftp://ftp.ncbi.nlm.nih.gov/blast/documents/NEWXML/xml2.pdf.  
8). "SAM". Sequence Alignment Map format.

XML2 and JSON can be downloaded either as one file per query (multiple-file) or one file for all queries (single-file). These formats are listed as Multiple-file XML2 (and JSON) or Single-file XML (and JSON).

The following report is offered under the Search Strategy section:  
1). "ASN.1" Search Strategy. A record of the parameters, query, and database used in the search. This file can be used to start a stand-alone BLAST search, see
https://www.ncbi.nlm.nih.gov/books/NBK1763/#CmdLineAppsManual.I455\_BLAST\_search\_stra


# Job title: D pink R -- 31..1030 of sequence (1000

Results for:

lcl|Query\_134981 D pink R -- 31..1030 of sequence(1000bp)
[?]

Your BLAST job specified more than one input sequence.
This box lets you choose which input sequence to show BLAST results for.

RID
:   A4XK8G3R015 (Expires on 03-10 18:56 pm)

Query ID
:   lcl|Query\_134981
:   lcl|Query\_134981

Description
:   D pink R -- 31..1030 of sequence

Molecule type
:   nucleic acid

Query Length
:   1000

Database Name
:   nr

Description
:   Nucleotide collection (nt) See details

Program
:   BLASTN 2.8.0+ Citation

  

Reference 

Zheng Zhang, Scott Schwartz, Lukas Wagner, and Webb Miller (2000), "A greedy algorithm for aligning DNA sequences", J Comput Biol 2000; 7(1-2):203-14.

Reference - database indexing

Aleksandr Morgulis, George Coulouris, Yan Raytselis, Thomas L. Madden, Richa Agarwala, Alejandro A. Schäffer (2008), "Database Indexing for Production MegaBLAST Searches", Bioinformatics 24:1757-1764.

Other reports:
Search Summary

[Taxonomy reports]
[Distance tree of results]
[MSA viewer]

Search Parameters

| Search parameter name | Search parameter value |
| --- | --- |
| Program | blastn |
| Word size | 28 |
| Expect value | 10 |
| Hitlist size | 100 |
| Match/Mismatch scores | 1,-2 |
| Gapcosts | 0,2.5 |
| Low Complexity Filter | Yes |
| Filter string | L;m; |
| Genetic Code | 1 |

Database

| Database parameter name | Database parameter value |
| --- | --- |
| Posted date | Mar 7, 2018 1:58 PM |
| Number of letters | 174,044,644,244 |
| Number of sequences | 46,882,714 |
| Entrez query | Includes:  Excludes:  None |

Karlin-Altschul statistics

| Params | Ungapped | Gapped |
| --- | --- | --- |
| Lambda | 1.33271 | 1.28 |
| K | 0.620991 | 0.46 |
| H | 1.12409 | 0.85 |

Results Statistics

| Results Statistics parameter name | Results Statistics parameter value |
| --- | --- |
| Length adjustment | 35 |
| Effective length of query | 965 |
| Effective length of database | 172403749254 |
| Effective search space | 166369618030110 |
| Effective search space used | 166369618030110 |


## Graphic Summary

### Distribution of the top 199 Blast Hits on 99 subject sequences [?]

The graphic is an overview of the database sequences aligned to the query sequence. These are represented horizontal bars colored coded by score and showing the extent
of the alignment on the query sequence. Separate aligned regions on the same database sequence are connected by a thin grey line.
Mousing over an alignment shows the database sequence title. Clicking an alignment displays a box with more details about the alignment and
link to the sequence alignment itself in the Alignments section of the report.

Mouse over to see the title, click to show alignments

Color key for alignment scores

<40

40-50

50-80

80-200

>=200

Query

1

200

400

600

800

1000

Paenibacillus sp. strain PS-K-17 16S ribosomal RNA gene..

Score:1832 Evalue:0

Accession:MF443394.1

Alignment

Paenibacillus sp. 3Cp1 16S ribosomal RNA gene, partial ..

Score:1832 Evalue:0

Accession:KP852522.1

Alignment

Uncultured bacterium clone 4L13 16S ribosomal RNA gene,..

Score:1832 Evalue:0

Accession:KP843054.1

Alignment

Paenibacillus sp. TI45-13ar 16S ribosomal RNA gene, par..

Score:1827 Evalue:0

Accession:KY419705.1

Alignment

Bacterium 4R9 16S ribosomal RNA gene, partial sequence

Score:1821 Evalue:0

Accession:KP965575.1

Alignment

Paenibacillus sp. strain BSW35 16S ribosomal RNA gene, ..

Score:1810 Evalue:0

Accession:KY446062.1

Alignment

Paenibacillus sp. 9N4 16S ribosomal RNA gene, partial s..

Score:1808 Evalue:0

Accession:HQ284889.1

Alignment

Paenibacillus illinoisensis strain WR\_17 16S ribosomal ..

Score:1808 Evalue:0

Accession:HQ661869.1

Alignment

Paenibacillus sp. Rs5 16S ribosomal RNA gene, partial s..

Score:1805 Evalue:0

Accession:JQ977193.1

Alignment

Bacterium 4R1 16S ribosomal RNA gene, partial sequence

Score:1792 Evalue:0

Accession:KP965572.1

Alignment

Paenibacillus sp. Q1-R10 16S ribosomal RNA gene, partia..

Score:1786 Evalue:0

Accession:JX994123.1

Alignment

Bacterium 4L18 16S ribosomal RNA gene, partial sequence

Score:1783 Evalue:0

Accession:KP965574.1

Alignment

Bacterium 4L13 16S ribosomal RNA gene, partial sequence

Score:1783 Evalue:0

Accession:KP965573.1

Alignment

Paenibacillus sp. HDDMM03 16S ribosomal RNA gene, parti..

Score:1781 Evalue:0

Accession:EU723825.1

Alignment

Uncultured bacterium clone 10-9BM 16S ribosomal RNA gen..

Score:1779 Evalue:0

Accession:HQ143281.1

Alignment

Paenibacillus sp. NCCB 100172 16S ribosomal RNA gene, p..

Score:1770 Evalue:0

Accession:EU003588.1

Alignment

Paenibacillus nicotianae strain YIM h-19 16S ribosomal ..

Score:1751 Evalue:0

Accession:NR\_134783.2

Alignment

Paenibacillus sp. R1 16S ribosomal RNA gene, partial se..

Score:1751 Evalue:0

Accession:KR263165.1

Alignment

Paenibacillus nicotianae strain YIM h-19 16S ribosomal ..

Score:1751 Evalue:0

Accession:KJ792471.1

Alignment

Paenibacillus kyungheensis strain DCY88 16S ribosomal R..

Score:1744 Evalue:0

Accession:NR\_145628.1

Alignment

Paenibacillus kyungheensis strain DCY88 16S ribosomal R..

Score:1744 Evalue:0

Accession:KF793934.1

Alignment

Paenibacillus hordei strain RH-N24 16S ribosomal RNA ge..

Score:1727 Evalue:0

Accession:NR\_109318.1

Alignment

Uncultured bacterium clone ncd2164d05c1 16S ribosomal R..

Score:1694 Evalue:0

Accession:JF185937.1

Alignment

Uncultured bacterium clone ncd2162h02c1 16S ribosomal R..

Score:1694 Evalue:0

Accession:JF185833.1

Alignment

Uncultured bacterium clone ncd2163a03c1 16S ribosomal R..

Score:1688 Evalue:0

Accession:JF185844.1

Alignment

Uncultured bacterium clone nbw208h09c1 16S ribosomal RN..

Score:1677 Evalue:0

Accession:GQ073426.1

Alignment

Uncultured bacterium clone 56-9BM 16S ribosomal RNA gen..

Score:1670 Evalue:0

Accession:HQ143315.1

Alignment

Paenibacillus kyungheensis strain R2abR 16S ribosomal R..

Score:1666 Evalue:0

Accession:MG607391.1

Alignment

Paenibacillus sp. TA\_EU partial 16S rRNA gene, strain T..

Score:1666 Evalue:0

Accession:HG942127.1

Alignment

Uncultured bacterium clone HR-R2W6B52 16S ribosomal RNA..

Score:1642 Evalue:0

Accession:KM507259.1

Alignment

Paenibacillus peoriae strain DSM 8320 16S ribosomal RNA..

Score:1633 Evalue:0

Accession:NR\_117740.1

Alignment

Paenibacillus polymyxa partial 16S rRNA gene, strain KC..

Score:1626 Evalue:0

Accession:HE981768.1

Alignment

Paenibacillus sp. strain Avl-2 16S ribosomal RNA gene, ..

Score:1622 Evalue:0

Accession:KX907738.1

Alignment

Paenibacillus polymyxa strain J, complete genome

Score:1622 Evalue:0

Accession:CP015423.1

Alignment

Paenibacillus peoriae strain DSM 8320 16S ribosomal RNA..

Score:1622 Evalue:0

Accession:NR\_117739.1

Alignment

Paenibacillus polymyxa partial 16S rRNA gene, strain KC..

Score:1622 Evalue:0

Accession:HE981792.1

Alignment

Paenibacillus sp. GA5 16S ribosomal RNA gene, partial s..

Score:1622 Evalue:0

Accession:DQ358734.1

Alignment

Paenibacillus polymyxa strain DSM 36 16S ribosomal RNA,..

Score:1620 Evalue:0

Accession:NR\_117727.2

Alignment

Paenibacillus polymyxa strain DSM 36 16S ribosomal RNA,..

Score:1620 Evalue:0

Accession:NR\_117726.2

Alignment

Paenibacillus polymyxa partial 16S rRNA gene, type stra..

Score:1620 Evalue:0

Accession:HG324071.1

Alignment

Paenibacillus polymyxa partial 16S rRNA gene, type stra..

Score:1620 Evalue:0

Accession:HG324070.1

Alignment

Paenibacillus jamilae partial 16S rRNA gene, strain KCT..

Score:1620 Evalue:0

Accession:HE981796.1

Alignment

Paenibacillus polymyxa partial 16S rRNA gene, strain KC..

Score:1620 Evalue:0

Accession:HE981788.1

Alignment

Paenibacillus polymyxa strain HX-140 16S ribosomal RNA ..

Score:1616 Evalue:0

Accession:MF136611.1

Alignment

Paenibacillus sp. strain YN-3 16S ribosomal RNA gene, p..

Score:1616 Evalue:0

Accession:MF967298.1

Alignment

Paenibacillus sp. strain BJ-7 16S ribosomal RNA gene, p..

Score:1616 Evalue:0

Accession:MF967288.1

Alignment

Paenibacillus peoriae strain GQJK57 16S ribosomal RNA g..

Score:1616 Evalue:0

Accession:KY952711.1

Alignment

Paenibacillus polymyxa strain TS683 16S ribosomal RNA g..

Score:1616 Evalue:0

Accession:KU860093.1

Alignment

Paenibacillus polymyxa strain TS682 16S ribosomal RNA g..

Score:1616 Evalue:0

Accession:KU860092.1

Alignment

Paenibacillus polymyxa strain TS681 16S ribosomal RNA g..

Score:1616 Evalue:0

Accession:KU860091.1

Alignment

Paenibacillus polymyxa strain ATCC 15970, complete geno..

Score:1616 Evalue:0

Accession:CP011420.1

Alignment

Paenibacillus polymyxa strain JK1 16S ribosomal RNA gen..

Score:1616 Evalue:0

Accession:KU563771.1

Alignment

Paenibacillus polymyxa partial 16S rRNA gene, strain Ma..

Score:1616 Evalue:0

Accession:LT223607.1

Alignment

Paenibacillus polymyxa strain J 16S ribosomal RNA gene,..

Score:1616 Evalue:0

Accession:KT783525.1

Alignment

Paenibacillus polymyxa strain LY214 16S ribosomal RNA g..

Score:1616 Evalue:0

Accession:KR058350.1

Alignment

Paenibacillus peoriae strain IHB B 12520 16S ribosomal ..

Score:1616 Evalue:0

Accession:KJ767388.1

Alignment

## Descriptions

, AlignDB=on Reading indexes 1-5, displaying indexes 1-5


Load next setPrevious Match

Sequences producing significant alignments:

Show all columns  of the table presenting sequences producing significant alignments 

Select:AllNone
Selected:0

Alignments
Download

FASTA (complete sequence)

FASTA (aligned sequences)

GenBank (complete sequence)

Hit Table (text)

Hit Table (CSV)

Text

XML

ASN.1

Continue
Cancel

GenBank 
Graphics
Distance tree of results
Multiple alignment
Show/hide columns of the table presenting sequences producing significant alignments 

Available columns

Description  
Max Score  
Total Score  
Coverage  
E-value  
IdentN  
Accession  
Restore Defaults
Ok
Cancel

Sequences producing significant alignments:

| Select for downloading or viewing reports | Description | Max score | Total score | Query cover | E value | Ident | Accession |
| --- | --- | --- | --- | --- | --- | --- | --- |
| 1Select seq MF443394.1 | Paenibacillus sp. strain PS-K-17 16S ribosomal RNA gene, partial sequence | 1832 | 1832 | 100% | 0.0 | 99% | MF443394.1 |
| 2Select seq KP852522.1 | Paenibacillus sp. 3Cp1 16S ribosomal RNA gene, partial sequence | 1832 | 1832 | 100% | 0.0 | 99% | KP852522.1 |
| 3Select seq KP843054.1 | Uncultured bacterium clone 4L13 16S ribosomal RNA gene, partial sequence | 1832 | 1832 | 100% | 0.0 | 99% | KP843054.1 |
| 4Select seq KY419705.1 | Paenibacillus sp. TI45-13ar 16S ribosomal RNA gene, partial sequence | 1827 | 1827 | 100% | 0.0 | 99% | KY419705.1 |
| 5Select seq KP965575.1 | Bacterium 4R9 16S ribosomal RNA gene, partial sequence | 1821 | 1821 | 100% | 0.0 | 99% | KP965575.1 |
| 6Select seq KY446062.1 | Paenibacillus sp. strain BSW35 16S ribosomal RNA gene, partial sequence | 1810 | 1810 | 100% | 0.0 | 99% | KY446062.1 |
| 7Select seq HQ284889.1 | Paenibacillus sp. 9N4 16S ribosomal RNA gene, partial sequence | 1808 | 1808 | 100% | 0.0 | 99% | HQ284889.1 |
| 8Select seq HQ661869.1 | Paenibacillus illinoisensis strain WR\_17 16S ribosomal RNA gene, partial sequence | 1808 | 1808 | 99% | 0.0 | 99% | HQ661869.1 |
| 9Select seq JQ977193.1 | Paenibacillus sp. Rs5 16S ribosomal RNA gene, partial sequence | 1805 | 1805 | 100% | 0.0 | 99% | JQ977193.1 |
| 10Select seq KP965572.1 | Bacterium 4R1 16S ribosomal RNA gene, partial sequence | 1792 | 1792 | 99% | 0.0 | 99% | KP965572.1 |
| 11Select seq JX994123.1 | Paenibacillus sp. Q1-R10 16S ribosomal RNA gene, partial sequence | 1786 | 1786 | 99% | 0.0 | 99% | JX994123.1 |
| 12Select seq KP965574.1 | Bacterium 4L18 16S ribosomal RNA gene, partial sequence | 1783 | 1783 | 99% | 0.0 | 99% | KP965574.1 |
| 13Select seq KP965573.1 | Bacterium 4L13 16S ribosomal RNA gene, partial sequence | 1783 | 1783 | 99% | 0.0 | 99% | KP965573.1 |
| 14Select seq EU723825.1 | Paenibacillus sp. HDDMM03 16S ribosomal RNA gene, partial sequence | 1781 | 1781 | 98% | 0.0 | 99% | EU723825.1 |
| 15Select seq HQ143281.1 | Uncultured bacterium clone 10-9BM 16S ribosomal RNA gene, partial sequence | 1779 | 1779 | 98% | 0.0 | 99% | HQ143281.1 |
| 16Select seq EU003588.1 | Paenibacillus sp. NCCB 100172 16S ribosomal RNA gene, partial sequence | 1770 | 1770 | 98% | 0.0 | 99% | EU003588.1 |
| 17Select seq NR\_134783.2 | Paenibacillus nicotianae strain YIM h-19 16S ribosomal RNA, complete sequence | 1751 | 1751 | 98% | 0.0 | 99% | NR\_134783.2 |
| 18Select seq KR263165.1 | Paenibacillus sp. R1 16S ribosomal RNA gene, partial sequence | 1751 | 1751 | 98% | 0.0 | 99% | KR263165.1 |
| 19Select seq KJ792471.1 | Paenibacillus nicotianae strain YIM h-19 16S ribosomal RNA gene, partial sequence | 1751 | 1751 | 98% | 0.0 | 99% | KJ792471.1 |
| 20Select seq NR\_145628.1 | Paenibacillus kyungheensis strain DCY88 16S ribosomal RNA, partial sequence | 1744 | 1744 | 98% | 0.0 | 99% | NR\_145628.1 |
| 21Select seq KF793934.1 | Paenibacillus kyungheensis strain DCY88 16S ribosomal RNA gene, partial sequence | 1744 | 1744 | 98% | 0.0 | 99% | KF793934.1 |
| 22Select seq NR\_109318.1 | Paenibacillus hordei strain RH-N24 16S ribosomal RNA gene, partial sequence | 1727 | 1727 | 98% | 0.0 | 98% | NR\_109318.1 |
| 23Select seq JF185937.1 | Uncultured bacterium clone ncd2164d05c1 16S ribosomal RNA gene, partial sequence | 1694 | 1694 | 92% | 0.0 | 99% | JF185937.1 |
| 24Select seq JF185833.1 | Uncultured bacterium clone ncd2162h02c1 16S ribosomal RNA gene, partial sequence | 1694 | 1694 | 92% | 0.0 | 99% | JF185833.1 |
| 25Select seq JF185844.1 | Uncultured bacterium clone ncd2163a03c1 16S ribosomal RNA gene, partial sequence | 1688 | 1688 | 92% | 0.0 | 99% | JF185844.1 |
| 26Select seq GQ073426.1 | Uncultured bacterium clone nbw208h09c1 16S ribosomal RNA gene, partial sequence | 1677 | 1677 | 92% | 0.0 | 99% | GQ073426.1 |
| 27Select seq HQ143315.1 | Uncultured bacterium clone 56-9BM 16S ribosomal RNA gene, partial sequence | 1670 | 1670 | 93% | 0.0 | 99% | HQ143315.1 |
| 28Select seq MG607391.1 | Paenibacillus kyungheensis strain R2abR 16S ribosomal RNA gene, partial sequence | 1666 | 1666 | 90% | 0.0 | 99% | MG607391.1 |
| 29Select seq HG942127.1 | Paenibacillus sp. TA\_EU partial 16S rRNA gene, strain TA\_EU | 1666 | 1666 | 90% | 0.0 | 99% | HG942127.1 |
| 30Select seq KM507259.1 | Uncultured bacterium clone HR-R2W6B52 16S ribosomal RNA gene, partial sequence | 1642 | 1642 | 89% | 0.0 | 99% | KM507259.1 |
| 31Select seq NR\_117740.1 | Paenibacillus peoriae strain DSM 8320 16S ribosomal RNA gene, partial sequence | 1633 | 1633 | 98% | 0.0 | 97% | NR\_117740.1 |
| 32Select seq HE981768.1 | Paenibacillus polymyxa partial 16S rRNA gene, strain KCTC 3627, clone 3 | 1626 | 1626 | 98% | 0.0 | 97% | HE981768.1 |
| 33Select seq KX907738.1 | Paenibacillus sp. strain Avl-2 16S ribosomal RNA gene, partial sequence | 1622 | 1622 | 98% | 0.0 | 96% | KX907738.1 |
| 34Select seq CP015423.1 | Paenibacillus polymyxa strain J, complete genome | 1622 | 22626 | 98% | 0.0 | 96% | CP015423.1 |
| 35Select seq NR\_117739.1 | Paenibacillus peoriae strain DSM 8320 16S ribosomal RNA gene, partial sequence | 1622 | 1622 | 98% | 0.0 | 96% | NR\_117739.1 |
| 36Select seq HE981792.1 | Paenibacillus polymyxa partial 16S rRNA gene, strain KCTC 3627, clone 27 | 1622 | 1622 | 98% | 0.0 | 96% | HE981792.1 |
| 37Select seq DQ358734.1 | Paenibacillus sp. GA5 16S ribosomal RNA gene, partial sequence | 1622 | 1622 | 98% | 0.0 | 96% | DQ358734.1 |
| 38Select seq NR\_117727.2 | Paenibacillus polymyxa strain DSM 36 16S ribosomal RNA, partial sequence | 1620 | 1620 | 98% | 0.0 | 96% | NR\_117727.2 |
| 39Select seq NR\_117726.2 | Paenibacillus polymyxa strain DSM 36 16S ribosomal RNA, partial sequence | 1620 | 1620 | 98% | 0.0 | 96% | NR\_117726.2 |
| 40Select seq HG324071.1 | Paenibacillus polymyxa partial 16S rRNA gene, type strain DSM 36T, clone 8 | 1620 | 1620 | 98% | 0.0 | 96% | HG324071.1 |
| 41Select seq HG324070.1 | Paenibacillus polymyxa partial 16S rRNA gene, type strain DSM 36T, clone 7 | 1620 | 1620 | 98% | 0.0 | 96% | HG324070.1 |
| 42Select seq HE981796.1 | Paenibacillus jamilae partial 16S rRNA gene, strain KCTC 13919, clone 4 | 1620 | 1620 | 98% | 0.0 | 96% | HE981796.1 |
| 43Select seq HE981788.1 | Paenibacillus polymyxa partial 16S rRNA gene, strain KCTC 3627, clone 23 | 1620 | 1620 | 98% | 0.0 | 96% | HE981788.1 |
| 44Select seq MF136611.1 | Paenibacillus polymyxa strain HX-140 16S ribosomal RNA gene, partial sequence | 1616 | 1616 | 98% | 0.0 | 96% | MF136611.1 |
| 45Select seq MF967298.1 | Paenibacillus sp. strain YN-3 16S ribosomal RNA gene, partial sequence | 1616 | 1616 | 98% | 0.0 | 96% | MF967298.1 |
| 46Select seq MF967288.1 | Paenibacillus sp. strain BJ-7 16S ribosomal RNA gene, partial sequence | 1616 | 1616 | 98% | 0.0 | 96% | MF967288.1 |
| 47Select seq KY952711.1 | Paenibacillus peoriae strain GQJK57 16S ribosomal RNA gene, partial sequence | 1616 | 1616 | 98% | 0.0 | 96% | KY952711.1 |
| 48Select seq KU860093.1 | Paenibacillus polymyxa strain TS683 16S ribosomal RNA gene, partial sequence | 1616 | 1616 | 98% | 0.0 | 96% | KU860093.1 |
| 49Select seq KU860092.1 | Paenibacillus polymyxa strain TS682 16S ribosomal RNA gene, partial sequence | 1616 | 1616 | 98% | 0.0 | 96% | KU860092.1 |
| 50Select seq KU860091.1 | Paenibacillus polymyxa strain TS681 16S ribosomal RNA gene, partial sequence | 1616 | 1616 | 98% | 0.0 | 96% | KU860091.1 |
| 51Select seq CP011420.1 | Paenibacillus polymyxa strain ATCC 15970, complete genome | 1616 | 20937 | 98% | 0.0 | 96% | CP011420.1 |
| 52Select seq KU563771.1 | Paenibacillus polymyxa strain JK1 16S ribosomal RNA gene, partial sequence | 1616 | 1616 | 98% | 0.0 | 96% | KU563771.1 |
| 53Select seq LT223607.1 | Paenibacillus polymyxa partial 16S rRNA gene, strain Marseille-AA00092 | 1616 | 1616 | 98% | 0.0 | 96% | LT223607.1 |
| 54Select seq KT783525.1 | Paenibacillus polymyxa strain J 16S ribosomal RNA gene, partial sequence | 1616 | 1616 | 98% | 0.0 | 96% | KT783525.1 |
| 55Select seq KR058350.1 | Paenibacillus polymyxa strain LY214 16S ribosomal RNA gene, partial sequence | 1616 | 1616 | 98% | 0.0 | 96% | KR058350.1 |
| 56Select seq KJ767388.1 | Paenibacillus peoriae strain IHB B 12520 16S ribosomal RNA gene, partial sequence | 1616 | 1616 | 98% | 0.0 | 96% | KJ767388.1 |
| 57Select seq KF441710.1 | Paenibacillus sp. 7B-896 16S ribosomal RNA gene, partial sequence | 1616 | 1616 | 98% | 0.0 | 96% | KF441710.1 |
| 58Select seq KC581713.1 | Paenibacillus sp. LXfos16 16S ribosomal RNA gene, partial sequence | 1616 | 1616 | 98% | 0.0 | 96% | KC581713.1 |
| 59Select seq KC236521.1 | Paenibacillus sp. 2083 16S ribosomal RNA gene, partial sequence | 1616 | 1616 | 98% | 0.0 | 96% | KC236521.1 |
| 60Select seq JX517259.1 | Paenibacillus polymyxa strain A1-25c-13 16S ribosomal RNA gene, partial sequence | 1616 | 1616 | 98% | 0.0 | 96% | JX517259.1 |
| 61Select seq JQ860094.1 | Paenibacillus brasilensis strain 136 16S ribosomal RNA gene, partial sequence | 1616 | 1616 | 98% | 0.0 | 96% | JQ860094.1 |
| 62Select seq JN409465.1 | Paenibacillus polymyxa strain SP16 16S ribosomal RNA gene, partial sequence | 1616 | 1616 | 98% | 0.0 | 96% | JN409465.1 |
| 63Select seq HQ844466.1 | Paenibacillus peoriae strain HLJFQ25 16S ribosomal RNA gene, partial sequence | 1616 | 1616 | 98% | 0.0 | 96% | HQ844466.1 |
| 64Select seq JF970587.1 | Paenibacillus peoriae 16S ribosomal RNA gene, partial sequence | 1616 | 1616 | 98% | 0.0 | 96% | JF970587.1 |
| 65Select seq JF747221.1 | Paenibacillus polymyxa strain S 16S ribosomal RNA gene, partial sequence | 1616 | 1616 | 98% | 0.0 | 96% | JF747221.1 |
| 66Select seq JF683620.1 | Paenibacillus polymyxa strain RS-10 16S ribosomal RNA gene, partial sequence | 1616 | 1616 | 98% | 0.0 | 96% | JF683620.1 |
| 67Select seq GU328691.1 | Paenibacillus sp. Sx52 16S ribosomal RNA gene, partial sequence | 1616 | 1616 | 98% | 0.0 | 96% | GU328691.1 |
| 68Select seq EU362611.1 | Paenibacillus polymyxa isolate TN99 16S ribosomal RNA gene, partial sequence | 1616 | 1616 | 98% | 0.0 | 96% | EU362611.1 |
| 69Select seq EU362610.1 | Paenibacillus polymyxa isolate TN98 16S ribosomal RNA gene, partial sequence | 1616 | 1616 | 98% | 0.0 | 96% | EU362610.1 |
| 70Select seq AM900509.1 | Paenibacillus sp. 14A-7 partial 16S rRNA gene, strain 14A-7 | 1616 | 1616 | 98% | 0.0 | 96% | AM900509.1 |
| 71Select seq EF634024.1 | Paenibacillus polymyxa strain ISSDS-851 16S ribosomal RNA gene, partial sequence | 1616 | 1616 | 98% | 0.0 | 96% | EF634024.1 |
| 72Select seq EF620468.1 | Paenibacillus polymyxa strain ISSDS-792 16S ribosomal RNA gene, partial sequence | 1616 | 1616 | 98% | 0.0 | 96% | EF620468.1 |
| 73Select seq KP126652.1 | Paenibacillus sp. SP47B 16S ribosomal RNA gene, partial sequence | 1615 | 1615 | 98% | 0.0 | 96% | KP126652.1 |
| 74Select seq NR\_117732.2 | Paenibacillus polymyxa strain DSM 36 16S ribosomal RNA, partial sequence | 1615 | 1615 | 98% | 0.0 | 96% | NR\_117732.2 |
| 75Select seq KR095606.1 | Uncultured Paenibacillus sp. clone DDGJ02 16S ribosomal RNA gene, partial sequence | 1615 | 1615 | 98% | 0.0 | 96% | KR095606.1 |
| 76Select seq KR095605.1 | Uncultured Paenibacillus sp. clone DDGJ01 16S ribosomal RNA gene, partial sequence | 1615 | 1615 | 98% | 0.0 | 96% | KR095605.1 |
| 77Select seq CP002213.2 | Paenibacillus polymyxa SC2, complete genome | 1615 | 20852 | 98% | 0.0 | 96% | CP002213.2 |
| 78Select seq CP010268.1 | Paenibacillus polymyxa strain Sb3-1, complete genome | 1615 | 22467 | 98% | 0.0 | 96% | CP010268.1 |
| 79Select seq CP009909.1 | Paenibacillus polymyxa strain CF05 genome | 1615 | 22491 | 98% | 0.0 | 96% | CP009909.1 |
| 80Select seq CP006872.1 | Paenibacillus polymyxa SQR-21, complete genome | 1615 | 20676 | 98% | 0.0 | 96% | CP006872.1 |
| 81Select seq HG324076.1 | Paenibacillus polymyxa partial 16S rRNA gene, type strain DSM 36T, clone 13 | 1615 | 1615 | 98% | 0.0 | 96% | HG324076.1 |
| 82Select seq JX498909.1 | Paenibacillus jamilae strain NBI 16S ribosomal RNA gene, partial sequence | 1615 | 1615 | 98% | 0.0 | 96% | JX498909.1 |
| 83Select seq HE981782.1 | Paenibacillus polymyxa partial 16S rRNA gene, strain KCTC 3627, clone 17 | 1615 | 1615 | 98% | 0.0 | 96% | HE981782.1 |
| 84Select seq HE981780.1 | Paenibacillus polymyxa partial 16S rRNA gene, strain KCTC 3627, clone 15 | 1615 | 1615 | 98% | 0.0 | 96% | HE981780.1 |
| 85Select seq HE981777.1 | Paenibacillus polymyxa partial 16S rRNA gene, strain KCTC 3627, clone 12 | 1615 | 1615 | 98% | 0.0 | 96% | HE981777.1 |
| 86Select seq HE981776.1 | Paenibacillus polymyxa partial 16S rRNA gene, strain KCTC 3627, clone 11 | 1615 | 1615 | 98% | 0.0 | 96% | HE981776.1 |
| 87Select seq AB689757.1 | Paenibacillus polymyxa gene for 16S ribosomal RNA, partial sequence | 1615 | 1615 | 98% | 0.0 | 96% | AB689757.1 |
| 88Select seq HE577054.1 | Paenibacillus polymyxa M1 main chromosome, complete genome | 1615 | 22483 | 98% | 0.0 | 96% | HE577054.1 |
| 89Select seq FR727737.1 | Paenibacillus polymyxa partial 16S rRNA gene, strain M1 | 1615 | 1615 | 98% | 0.0 | 96% | FR727737.1 |
| 90Select seq KU312055.1 | Bacillus sp. JZ212 16S ribosomal RNA gene, partial sequence | 1613 | 1613 | 98% | 0.0 | 96% | KU312055.1 |
| 91Select seq HQ844465.1 | Paenibacillus brasilensis strain HLJFQ23 16S ribosomal RNA gene, partial sequence | 1613 | 1613 | 98% | 0.0 | 96% | HQ844465.1 |
| 92Select seq EF620470.1 | Paenibacillus polymyxa strain ISSDS-796 16S ribosomal RNA gene, partial sequence | 1613 | 1613 | 98% | 0.0 | 96% | EF620470.1 |
| 93Select seq EF108320.1 | Paenibacillus polymyxa strain DY1 16S ribosomal RNA gene, partial sequence | 1613 | 1613 | 98% | 0.0 | 96% | EF108320.1 |
| 94Select seq CP017968.3 | Paenibacillus polymyxa strain YC0573 chromosome, complete genome | 1611 | 19303 | 98% | 0.0 | 96% | CP017968.3 |
| 95Select seq CP017967.3 | Paenibacillus polymyxa strain YC0136 chromosome, complete genome | 1611 | 17658 | 98% | 0.0 | 96% | CP017967.3 |
| 96Select seq MF967296.1 | Paenibacillus sp. strain AH-1 16S ribosomal RNA gene, partial sequence | 1611 | 1611 | 98% | 0.0 | 96% | MF967296.1 |
| 97Select seq MF967286.1 | Paenibacillus sp. strain BJ-5 16S ribosomal RNA gene, partial sequence | 1611 | 1611 | 98% | 0.0 | 96% | MF967286.1 |
| 98Select seq JQ279510.1 | Paenibacillus polymyxa strain AB15 16S ribosomal RNA gene, partial sequence | 1609 | 1609 | 98% | 0.0 | 96% | JQ279510.1 |
| 99Select seq KX929058.1 | Paenibacillus polymyxa strain QH-G 16S ribosomal RNA gene, partial sequence | 1607 | 1607 | 98% | 0.0 | 96% | KX929058.1 |


## Alignments

Loading alignment... for sequences gi|1214741488,gi|915515201,gi|915512955,gi|1127353198,gi|846924131 AlignDB=on Reading indexes 1-5

Download

FASTA (complete sequence)

FASTA (aligned sequences)

GenBank (complete sequence)

Continue
Cancel

GenBankGraphics

Next
Previous
Descriptions

Paenibacillus sp. strain PS-K-17 16S ribosomal RNA gene, partial sequence

Sequence ID: MF443394.1Length: 1462Number of Matches: 1

Related Information

Range 1: 409 to 1408GenBankGraphics

Next Match
Previous Match
First Match

Alignment statistics for match #1

| Score | Expect | Identities | Gaps | Strand | Frame |
| --- | --- | --- | --- | --- | --- |
| 1832 bits(992) | 0.0() | 996/1000(99%) | 0/1000(0%) | Plus/Minus |  |

Features:

```
Query  1     GCAGGTNANNCCACCGACTTCGGGTGTTGCAAACTCTCGTGGTGTGACGGGCGGTGTGTA  60
             |||||| |  ||||||||||||||||||||||||||||||||||||||||||||||||||
Sbjct  1408  GCAGGTTACCCCACCGACTTCGGGTGTTGCAAACTCTCGTGGTGTGACGGGCGGTGTGTA  1349

Query  61    CAAGACCCGGGAACGTATTCACCGCGGCATGCTGATCCGCGATTACTAGCAATTCCGACT  120
             ||||||||||||||||||||||||||||||||||||||||||||||||||||||||||||
Sbjct  1348  CAAGACCCGGGAACGTATTCACCGCGGCATGCTGATCCGCGATTACTAGCAATTCCGACT  1289

Query  121   TCATGCAGGCGAGTTGCAGCCTGCAATCCGAACTGAGACCGGCTTTTAAGGATTGGCTCC  180
             ||||||||||||||||||||||||||||||||||||||||||||||||||||||||||||
Sbjct  1288  TCATGCAGGCGAGTTGCAGCCTGCAATCCGAACTGAGACCGGCTTTTAAGGATTGGCTCC  1229

Query  181   ATCTTGCGATATTGCTTCCCGTTGTACCGGCCATTGTAGTACGTGTGTAGCCCAAGTCAT  240
             ||||||||||||||||||||||||||||||||||||||||||||||||||||||||||||
Sbjct  1228  ATCTTGCGATATTGCTTCCCGTTGTACCGGCCATTGTAGTACGTGTGTAGCCCAAGTCAT  1169

Query  241   AAGGGGCATGATGATTTGACGTCATCCCCACCTTCCTCCGGTTTGTCACCGGCAGTCTGC  300
             ||||||||||||||||||||||||||||||||||||||||||||||||||||||||||||
Sbjct  1168  AAGGGGCATGATGATTTGACGTCATCCCCACCTTCCTCCGGTTTGTCACCGGCAGTCTGC  1109

Query  301   TTAGAGTGCCCACCATGATGTGCTGGCAACTAAGCATAAGGGTTGCGCTCGTTGCGGGAC  360
             ||||||||||||||||||||||||||||||||||||||||||||||||||||||||||||
Sbjct  1108  TTAGAGTGCCCACCATGATGTGCTGGCAACTAAGCATAAGGGTTGCGCTCGTTGCGGGAC  1049

Query  361   TTAACCCAACATCTCACGACACGAGCTGACGACAACCATGCACCACCTGTCTCCTTTGTC  420
             ||||||||||||||||||||||||||||||||||||||||||||||||||||||||||||
Sbjct  1048  TTAACCCAACATCTCACGACACGAGCTGACGACAACCATGCACCACCTGTCTCCTTTGTC  989

Query  421   CCGAAGGAAAAGACTATCTCTAGTCCGGTCAAAGGGATGTCAAGACTTGGTAAGGTTCTT  480
             ||||||||||||||||||||||||||||||||||||||||||||||||||||||||||||
Sbjct  988   CCGAAGGAAAAGACTATCTCTAGTCCGGTCAAAGGGATGTCAAGACTTGGTAAGGTTCTT  929

Query  481   CGCGTTGCTTCGAATTAAACCACATACTCCACTGCTTGTGCGGGTCCCCGTCAATTCCTT  540
             ||||||||||||||||||||||||||||||||||||||||||||||||||||||||||||
Sbjct  928   CGCGTTGCTTCGAATTAAACCACATACTCCACTGCTTGTGCGGGTCCCCGTCAATTCCTT  869

Query  541   TGAGTTTCAGTCTTGCGACCGTACTCCCCAGGCGGAATGCTTAATGTGTTAACTTCGGCA  600
             ||||||||||||||||||||||||||||||||||||||||||||||||||||||||||||
Sbjct  868   TGAGTTTCAGTCTTGCGACCGTACTCCCCAGGCGGAATGCTTAATGTGTTAACTTCGGCA  809

Query  601   CCAAGGGTATCGAAACCCCTAACACCTAGCATTCATCGTTTACGGCGTGGACTACCAGGG  660
             ||||||||||||||||||||||||||||||||||||||||||||||||||||||||||||
Sbjct  808   CCAAGGGTATCGAAACCCCTAACACCTAGCATTCATCGTTTACGGCGTGGACTACCAGGG  749

Query  661   TATCTAATCCTGTTTGCTCCCCACGCTTTCGCGCCTCAGCGTCAGTTACAGCCCAGAAAG  720
             ||||||||||||||||||||||||||||||||||||||||||||||||||||||||||||
Sbjct  748   TATCTAATCCTGTTTGCTCCCCACGCTTTCGCGCCTCAGCGTCAGTTACAGCCCAGAAAG  689

Query  721   TCGCCTTCGCCACTGGTGTTCCTCCACATCTCTACGCATTTCACCGCTACACGTGGAATT  780
             ||||||||||||||||||||||||||||||||||||||||||||||||||||||||||||
Sbjct  688   TCGCCTTCGCCACTGGTGTTCCTCCACATCTCTACGCATTTCACCGCTACACGTGGAATT  629

Query  781   CCACTTTCCTCTTCTGTACTCAAGCTCTCCAGTTTCCAGTGCGACCCAAGGTTGAGCCTT  840
             ||||||||||||||||||||||||||||||||||||||||||||||||||||||||||||
Sbjct  628   CCACTTTCCTCTTCTGTACTCAAGCTCTCCAGTTTCCAGTGCGACCCAAGGTTGAGCCTT  569

Query  841   GGGCTGTGACACCGGACTTAAAAAGCCGCCTGCGCGCGCTTTACGCCCAATAATTCCGGA  900
             ||||||||||||||||||||||||||||||||||||||||||||||||||||||||||||
Sbjct  568   GGGCTGTGACACCGGACTTAAAAAGCCGCCTGCGCGCGCTTTACGCCCAATAATTCCGGA  509

Query  901   CAACGCTTGCCCCCTACGTATTACCGCGGCTGCTGGCACGTAGTTAGCCGGGGCTTTCTT  960
             ||||||||||||||||||||||||||||||||||||||||||||||||||||||||||||
Sbjct  508   CAACGCTTGCCCCCTACGTATTACCGCGGCTGCTGGCACGTAGTTAGCCGGGGCTTTCTT  449

Query  961   CTCAGGTACCGTCACTCCGATAGCAGTTACTCTNTCGGAC  1000
             ||||||||||||||||||||||||||||||||| ||||||
Sbjct  448   CTCAGGTACCGTCACTCCGATAGCAGTTACTCTATCGGAC  409
```

Download

FASTA (complete sequence)

FASTA (aligned sequences)

GenBank (complete sequence)

Continue
Cancel

GenBankGraphics

Next
Previous
Descriptions

Paenibacillus sp. 3Cp1 16S ribosomal RNA gene, partial sequence

Sequence ID: KP852522.1Length: 1434Number of Matches: 1

Related Information

Range 1: 416 to 1415GenBankGraphics

Next Match
Previous Match
First Match

Alignment statistics for match #1

| Score | Expect | Identities | Gaps | Strand | Frame |
| --- | --- | --- | --- | --- | --- |
| 1832 bits(992) | 0.0() | 996/1000(99%) | 0/1000(0%) | Plus/Minus |  |

Features:

```
Query  1     GCAGGTNANNCCACCGACTTCGGGTGTTGCAAACTCTCGTGGTGTGACGGGCGGTGTGTA  60
             |||||| |  ||||||||||||||||||||||||||||||||||||||||||||||||||
Sbjct  1415  GCAGGTTACCCCACCGACTTCGGGTGTTGCAAACTCTCGTGGTGTGACGGGCGGTGTGTA  1356

Query  61    CAAGACCCGGGAACGTATTCACCGCGGCATGCTGATCCGCGATTACTAGCAATTCCGACT  120
             ||||||||||||||||||||||||||||||||||||||||||||||||||||||||||||
Sbjct  1355  CAAGACCCGGGAACGTATTCACCGCGGCATGCTGATCCGCGATTACTAGCAATTCCGACT  1296

Query  121   TCATGCAGGCGAGTTGCAGCCTGCAATCCGAACTGAGACCGGCTTTTAAGGATTGGCTCC  180
             ||||||||||||||||||||||||||||||||||||||||||||||||||||||||||||
Sbjct  1295  TCATGCAGGCGAGTTGCAGCCTGCAATCCGAACTGAGACCGGCTTTTAAGGATTGGCTCC  1236

Query  181   ATCTTGCGATATTGCTTCCCGTTGTACCGGCCATTGTAGTACGTGTGTAGCCCAAGTCAT  240
             ||||||||||||||||||||||||||||||||||||||||||||||||||||||||||||
Sbjct  1235  ATCTTGCGATATTGCTTCCCGTTGTACCGGCCATTGTAGTACGTGTGTAGCCCAAGTCAT  1176

Query  241   AAGGGGCATGATGATTTGACGTCATCCCCACCTTCCTCCGGTTTGTCACCGGCAGTCTGC  300
             ||||||||||||||||||||||||||||||||||||||||||||||||||||||||||||
Sbjct  1175  AAGGGGCATGATGATTTGACGTCATCCCCACCTTCCTCCGGTTTGTCACCGGCAGTCTGC  1116

Query  301   TTAGAGTGCCCACCATGATGTGCTGGCAACTAAGCATAAGGGTTGCGCTCGTTGCGGGAC  360
             ||||||||||||||||||||||||||||||||||||||||||||||||||||||||||||
Sbjct  1115  TTAGAGTGCCCACCATGATGTGCTGGCAACTAAGCATAAGGGTTGCGCTCGTTGCGGGAC  1056

Query  361   TTAACCCAACATCTCACGACACGAGCTGACGACAACCATGCACCACCTGTCTCCTTTGTC  420
             ||||||||||||||||||||||||||||||||||||||||||||||||||||||||||||
Sbjct  1055  TTAACCCAACATCTCACGACACGAGCTGACGACAACCATGCACCACCTGTCTCCTTTGTC  996

Query  421   CCGAAGGAAAAGACTATCTCTAGTCCGGTCAAAGGGATGTCAAGACTTGGTAAGGTTCTT  480
             ||||||||||||||||||||||||||||||||||||||||||||||||||||||||||||
Sbjct  995   CCGAAGGAAAAGACTATCTCTAGTCCGGTCAAAGGGATGTCAAGACTTGGTAAGGTTCTT  936

Query  481   CGCGTTGCTTCGAATTAAACCACATACTCCACTGCTTGTGCGGGTCCCCGTCAATTCCTT  540
             ||||||||||||||||||||||||||||||||||||||||||||||||||||||||||||
Sbjct  935   CGCGTTGCTTCGAATTAAACCACATACTCCACTGCTTGTGCGGGTCCCCGTCAATTCCTT  876

Query  541   TGAGTTTCAGTCTTGCGACCGTACTCCCCAGGCGGAATGCTTAATGTGTTAACTTCGGCA  600
             ||||||||||||||||||||||||||||||||||||||||||||||||||||||||||||
Sbjct  875   TGAGTTTCAGTCTTGCGACCGTACTCCCCAGGCGGAATGCTTAATGTGTTAACTTCGGCA  816

Query  601   CCAAGGGTATCGAAACCCCTAACACCTAGCATTCATCGTTTACGGCGTGGACTACCAGGG  660
             ||||||||||||||||||||||||||||||||||||||||||||||||||||||||||||
Sbjct  815   CCAAGGGTATCGAAACCCCTAACACCTAGCATTCATCGTTTACGGCGTGGACTACCAGGG  756

Query  661   TATCTAATCCTGTTTGCTCCCCACGCTTTCGCGCCTCAGCGTCAGTTACAGCCCAGAAAG  720
             ||||||||||||||||||||||||||||||||||||||||||||||||||||||||||||
Sbjct  755   TATCTAATCCTGTTTGCTCCCCACGCTTTCGCGCCTCAGCGTCAGTTACAGCCCAGAAAG  696

Query  721   TCGCCTTCGCCACTGGTGTTCCTCCACATCTCTACGCATTTCACCGCTACACGTGGAATT  780
             ||||||||||||||||||||||||||||||||||||||||||||||||||||||||||||
Sbjct  695   TCGCCTTCGCCACTGGTGTTCCTCCACATCTCTACGCATTTCACCGCTACACGTGGAATT  636

Query  781   CCACTTTCCTCTTCTGTACTCAAGCTCTCCAGTTTCCAGTGCGACCCAAGGTTGAGCCTT  840
             ||||||||||||||||||||||||||||||||||||||||||||||||||||||||||||
Sbjct  635   CCACTTTCCTCTTCTGTACTCAAGCTCTCCAGTTTCCAGTGCGACCCAAGGTTGAGCCTT  576

Query  841   GGGCTGTGACACCGGACTTAAAAAGCCGCCTGCGCGCGCTTTACGCCCAATAATTCCGGA  900
             ||||||||||||||||||||||||||||||||||||||||||||||||||||||||||||
Sbjct  575   GGGCTGTGACACCGGACTTAAAAAGCCGCCTGCGCGCGCTTTACGCCCAATAATTCCGGA  516

Query  901   CAACGCTTGCCCCCTACGTATTACCGCGGCTGCTGGCACGTAGTTAGCCGGGGCTTTCTT  960
             ||||||||||||||||||||||||||||||||||||||||||||||||||||||||||||
Sbjct  515   CAACGCTTGCCCCCTACGTATTACCGCGGCTGCTGGCACGTAGTTAGCCGGGGCTTTCTT  456

Query  961   CTCAGGTACCGTCACTCCGATAGCAGTTACTCTNTCGGAC  1000
             ||||||||||||||||||||||||||||||||| ||||||
Sbjct  455   CTCAGGTACCGTCACTCCGATAGCAGTTACTCTATCGGAC  416
```

Download

FASTA (complete sequence)

FASTA (aligned sequences)

GenBank (complete sequence)

Continue
Cancel

GenBankGraphics

Next
Previous
Descriptions

Uncultured bacterium clone 4L13 16S ribosomal RNA gene, partial sequence

Sequence ID: KP843054.1Length: 1460Number of Matches: 1

Related Information

Range 1: 428 to 1427GenBankGraphics

Next Match
Previous Match
First Match

Alignment statistics for match #1

| Score | Expect | Identities | Gaps | Strand | Frame |
| --- | --- | --- | --- | --- | --- |
| 1832 bits(992) | 0.0() | 996/1000(99%) | 0/1000(0%) | Plus/Minus |  |

Features:

```
Query  1     GCAGGTNANNCCACCGACTTCGGGTGTTGCAAACTCTCGTGGTGTGACGGGCGGTGTGTA  60
             |||||| |  ||||||||||||||||||||||||||||||||||||||||||||||||||
Sbjct  1427  GCAGGTTACCCCACCGACTTCGGGTGTTGCAAACTCTCGTGGTGTGACGGGCGGTGTGTA  1368

Query  61    CAAGACCCGGGAACGTATTCACCGCGGCATGCTGATCCGCGATTACTAGCAATTCCGACT  120
             ||||||||||||||||||||||||||||||||||||||||||||||||||||||||||||
Sbjct  1367  CAAGACCCGGGAACGTATTCACCGCGGCATGCTGATCCGCGATTACTAGCAATTCCGACT  1308

Query  121   TCATGCAGGCGAGTTGCAGCCTGCAATCCGAACTGAGACCGGCTTTTAAGGATTGGCTCC  180
             ||||||||||||||||||||||||||||||||||||||||||||||||||||||||||||
Sbjct  1307  TCATGCAGGCGAGTTGCAGCCTGCAATCCGAACTGAGACCGGCTTTTAAGGATTGGCTCC  1248

Query  181   ATCTTGCGATATTGCTTCCCGTTGTACCGGCCATTGTAGTACGTGTGTAGCCCAAGTCAT  240
             ||||||||||||||||||||||||||||||||||||||||||||||||||||||||||||
Sbjct  1247  ATCTTGCGATATTGCTTCCCGTTGTACCGGCCATTGTAGTACGTGTGTAGCCCAAGTCAT  1188

Query  241   AAGGGGCATGATGATTTGACGTCATCCCCACCTTCCTCCGGTTTGTCACCGGCAGTCTGC  300
             ||||||||||||||||||||||||||||||||||||||||||||||||||||||||||||
Sbjct  1187  AAGGGGCATGATGATTTGACGTCATCCCCACCTTCCTCCGGTTTGTCACCGGCAGTCTGC  1128

Query  301   TTAGAGTGCCCACCATGATGTGCTGGCAACTAAGCATAAGGGTTGCGCTCGTTGCGGGAC  360
             ||||||||||||||||||||||||||||||||||||||||||||||||||||||||||||
Sbjct  1127  TTAGAGTGCCCACCATGATGTGCTGGCAACTAAGCATAAGGGTTGCGCTCGTTGCGGGAC  1068

Query  361   TTAACCCAACATCTCACGACACGAGCTGACGACAACCATGCACCACCTGTCTCCTTTGTC  420
             ||||||||||||||||||||||||||||||||||||||||||||||||||||||||||||
Sbjct  1067  TTAACCCAACATCTCACGACACGAGCTGACGACAACCATGCACCACCTGTCTCCTTTGTC  1008

Query  421   CCGAAGGAAAAGACTATCTCTAGTCCGGTCAAAGGGATGTCAAGACTTGGTAAGGTTCTT  480
             ||||||||||||||||||||||||||||||||||||||||||||||||||||||||||||
Sbjct  1007  CCGAAGGAAAAGACTATCTCTAGTCCGGTCAAAGGGATGTCAAGACTTGGTAAGGTTCTT  948

Query  481   CGCGTTGCTTCGAATTAAACCACATACTCCACTGCTTGTGCGGGTCCCCGTCAATTCCTT  540
             ||||||||||||||||||||||||||||||||||||||||||||||||||||||||||||
Sbjct  947   CGCGTTGCTTCGAATTAAACCACATACTCCACTGCTTGTGCGGGTCCCCGTCAATTCCTT  888

Query  541   TGAGTTTCAGTCTTGCGACCGTACTCCCCAGGCGGAATGCTTAATGTGTTAACTTCGGCA  600
             ||||||||||||||||||||||||||||||||||||||||||||||||||||||||||||
Sbjct  887   TGAGTTTCAGTCTTGCGACCGTACTCCCCAGGCGGAATGCTTAATGTGTTAACTTCGGCA  828

Query  601   CCAAGGGTATCGAAACCCCTAACACCTAGCATTCATCGTTTACGGCGTGGACTACCAGGG  660
             ||||||||||||||||||||||||||||||||||||||||||||||||||||||||||||
Sbjct  827   CCAAGGGTATCGAAACCCCTAACACCTAGCATTCATCGTTTACGGCGTGGACTACCAGGG  768

Query  661   TATCTAATCCTGTTTGCTCCCCACGCTTTCGCGCCTCAGCGTCAGTTACAGCCCAGAAAG  720
             ||||||||||||||||||||||||||||||||||||||||||||||||||||||||||||
Sbjct  767   TATCTAATCCTGTTTGCTCCCCACGCTTTCGCGCCTCAGCGTCAGTTACAGCCCAGAAAG  708

Query  721   TCGCCTTCGCCACTGGTGTTCCTCCACATCTCTACGCATTTCACCGCTACACGTGGAATT  780
             ||||||||||||||||||||||||||||||||||||||||||||||||||||||||||||
Sbjct  707   TCGCCTTCGCCACTGGTGTTCCTCCACATCTCTACGCATTTCACCGCTACACGTGGAATT  648

Query  781   CCACTTTCCTCTTCTGTACTCAAGCTCTCCAGTTTCCAGTGCGACCCAAGGTTGAGCCTT  840
             ||||||||||||||||||||||||||||||||||||||||||||||||||||||||||||
Sbjct  647   CCACTTTCCTCTTCTGTACTCAAGCTCTCCAGTTTCCAGTGCGACCCAAGGTTGAGCCTT  588

Query  841   GGGCTGTGACACCGGACTTAAAAAGCCGCCTGCGCGCGCTTTACGCCCAATAATTCCGGA  900
             ||||||||||||||||||||||||||||||||||||||||||||||||||||||||||||
Sbjct  587   GGGCTGTGACACCGGACTTAAAAAGCCGCCTGCGCGCGCTTTACGCCCAATAATTCCGGA  528

Query  901   CAACGCTTGCCCCCTACGTATTACCGCGGCTGCTGGCACGTAGTTAGCCGGGGCTTTCTT  960
             ||||||||||||||||||||||||||||||||||||||||||||||||||||||||||||
Sbjct  527   CAACGCTTGCCCCCTACGTATTACCGCGGCTGCTGGCACGTAGTTAGCCGGGGCTTTCTT  468

Query  961   CTCAGGTACCGTCACTCCGATAGCAGTTACTCTNTCGGAC  1000
             ||||||||||||||||||||||||||||||||| ||||||
Sbjct  467   CTCAGGTACCGTCACTCCGATAGCAGTTACTCTATCGGAC  428
```

Download

FASTA (complete sequence)

FASTA (aligned sequences)

GenBank (complete sequence)

Continue
Cancel

GenBankGraphics

Next
Previous
Descriptions

Paenibacillus sp. TI45-13ar 16S ribosomal RNA gene, partial sequence

Sequence ID: KY419705.1Length: 1472Number of Matches: 1

Related Information

Range 1: 445 to 1445GenBankGraphics

Next Match
Previous Match
First Match

Alignment statistics for match #1

| Score | Expect | Identities | Gaps | Strand | Frame |
| --- | --- | --- | --- | --- | --- |
| 1827 bits(989) | 0.0() | 996/1001(99%) | 1/1001(0%) | Plus/Minus |  |

Features:

```
Query  1     GCAGGTNANNCCACCGACTTCGGGTGTTGCAAACTCTCGTGGTGTGACGGGCGGTGTGTA  60
             |||||| |  ||||||||||||||||||||||||||||||||||||||||||||||||||
Sbjct  1445  GCAGGTTACCCCACCGACTTCGGGTGTTGCAAACTCTCGTGGTGTGACGGGCGGTGTGTA  1386

Query  61    CAAGACCCGGGAACGTATTCACCGCGGCATGCTGATCCGCGATTACTAGCAATTCCGACT  120
             ||||||||||||||||||||||||||||||||||||||||||||||||||||||||||||
Sbjct  1385  CAAGACCCGGGAACGTATTCACCGCGGCATGCTGATCCGCGATTACTAGCAATTCCGACT  1326

Query  121   TCATGCAGGCGAGTTGCAGCCTGCAATCCGAACTGAGACCGGCTTTTAAGGATTGGCTCC  180
             ||||||||||||||||||||||||||||||||||||||||||||||||||||||||||||
Sbjct  1325  TCATGCAGGCGAGTTGCAGCCTGCAATCCGAACTGAGACCGGCTTTTAAGGATTGGCTCC  1266

Query  181   ATCTTGCGATATTGCTTCCCGTTGTACCGGCCATTGTAGTACGTGTGTAGCCCAAGTCAT  240
             ||||||||||||||||||||||||||||||||||||||||||||||||||||||||||||
Sbjct  1265  ATCTTGCGATATTGCTTCCCGTTGTACCGGCCATTGTAGTACGTGTGTAGCCCAAGTCAT  1206

Query  241   AAGGGGCATGATGATTTGACGTCATCCCCACCTTCCTCCGGTTTGTCACCGGCAGTCTGC  300
             ||||||||||||||||||||||||||||||||||||||||||||||||||||||||||||
Sbjct  1205  AAGGGGCATGATGATTTGACGTCATCCCCACCTTCCTCCGGTTTGTCACCGGCAGTCTGC  1146

Query  301   TTAGAGTGCCCACCATGATGTGCTGGCAACTAAGCATAAGGGTTGCGCTCGTTGCGGGAC  360
             ||||||||||||||||||||||||||||||||||||||||||||||||||||||||||||
Sbjct  1145  TTAGAGTGCCCACCATGATGTGCTGGCAACTAAGCATAAGGGTTGCGCTCGTTGCGGGAC  1086

Query  361   TTAACCCAACATCTCACGACACGAGCTGACGACAACCATGCACCACCTGTCTCCTTTGTC  420
             ||||||||||||||||||||||||||||||||||||||||||||||||||||||||||||
Sbjct  1085  TTAACCCAACATCTCACGACACGAGCTGACGACAACCATGCACCACCTGTCTCCTTTGTC  1026

Query  421   CCGAAGGAAAAGACTATCTCTAGTCCGGTCAAAGGGATGTCAAGACTTGGTAAGGTTCTT  480
             ||||||||||||||||||||||||||||||||||||||||||||||||||||||||||||
Sbjct  1025  CCGAAGGAAAAGACTATCTCTAGTCCGGTCAAAGGGATGTCAAGACTTGGTAAGGTTCTT  966

Query  481   CGCGTTGCTTCGAATTAAACCACATACTCCACTGCTTGTGCGGGTCCCCGTCAATTCCTT  540
             ||||||||||||||||||||||||||||||||||||||||||||||||||||||||||||
Sbjct  965   CGCGTTGCTTCGAATTAAACCACATACTCCACTGCTTGTGCGGGTCCCCGTCAATTCCTT  906

Query  541   TGAGTTTCAGTCTTGCGACCGTACTCCCCAGGCGGAATGCTTAATGTGTTAACTTCGGCA  600
             ||||||||||||||||||||||||||||||||||||||||||||||||||||||||||||
Sbjct  905   TGAGTTTCAGTCTTGCGACCGTACTCCCCAGGCGGAATGCTTAATGTGTTAACTTCGGCA  846

Query  601   CCAAGGGTATCGAAACCCCTAACACCTAGCATTCATCGTTTACGGCGTGGACTACCAGGG  660
             ||||||||||||||||||||||||||||||||||||||||||||||||||||||||||||
Sbjct  845   CCAAGGGTATCGAAACCCCTAACACCTAGCATTCATCGTTTACGGCGTGGACTACCAGGG  786

Query  661   TATCTAATCCTGTTTGCTCCCCACGCTTTCGCGCCTCAGCGTCAGTTACAGCCCAGAAAG  720
             ||||||||||||||||||||||||||||||||||||||||||||||||||||||||||||
Sbjct  785   TATCTAATCCTGTTTGCTCCCCACGCTTTCGCGCCTCAGCGTCAGTTACAGCCCAGAAAG  726

Query  721   TCGCCTTCGCCACTGGTGTTCCTCCACATCTCTACGCATTTCACCGCTACACGTGGAATT  780
             ||||||||||||||||||||||||||||||||||||||||||||||||||||||||||||
Sbjct  725   TCGCCTTCGCCACTGGTGTTCCTCCACATCTCTACGCATTTCACCGCTACACGTGGAATT  666

Query  781   C-CACTTTCCTCTTCTGTACTCAAGCTCTCCAGTTTCCAGTGCGACCCAAGGTTGAGCCT  839
             | ||||||||||||||||||||||||||||||||||||||||||||||||||||||||||
Sbjct  665   CTCACTTTCCTCTTCTGTACTCAAGCTCTCCAGTTTCCAGTGCGACCCAAGGTTGAGCCT  606

Query  840   TGGGCTGTGACACCGGACTTAAAAAGCCGCCTGCGCGCGCTTTACGCCCAATAATTCCGG  899
             ||||||||||||||||||||||||||||||||||||||||||||||||||||||||||||
Sbjct  605   TGGGCTGTGACACCGGACTTAAAAAGCCGCCTGCGCGCGCTTTACGCCCAATAATTCCGG  546

Query  900   ACAACGCTTGCCCCCTACGTATTACCGCGGCTGCTGGCACGTAGTTAGCCGGGGCTTTCT  959
             ||||||||||||||||||||||||||||||||||||||||||||||||||||||||||||
Sbjct  545   ACAACGCTTGCCCCCTACGTATTACCGCGGCTGCTGGCACGTAGTTAGCCGGGGCTTTCT  486

Query  960   TCTCAGGTACCGTCACTCCGATAGCAGTTACTCTNTCGGAC  1000
             |||||||||||||||||||||||||||||||||| ||||||
Sbjct  485   TCTCAGGTACCGTCACTCCGATAGCAGTTACTCTATCGGAC  445
```

Download

FASTA (complete sequence)

FASTA (aligned sequences)

GenBank (complete sequence)

Continue
Cancel

GenBankGraphics

Next
Previous
Descriptions

Bacterium 4R9 16S ribosomal RNA gene, partial sequence

Sequence ID: KP965575.1Length: 1517Number of Matches: 1

Related Information

Range 1: 457 to 1457GenBankGraphics

Next Match
Previous Match
First Match

Alignment statistics for match #1

| Score | Expect | Identities | Gaps | Strand | Frame |
| --- | --- | --- | --- | --- | --- |
| 1821 bits(986) | 0.0() | 995/1001(99%) | 1/1001(0%) | Plus/Minus |  |

Features:

```
Query  1     GCAGGTNANNCCACCGACTTCGGGTGTTGCAAACTCTCGTGGTGTGACGGGCGGTGTGTA  60
             |||||| |  ||||||||||||||||||||||||||||||||||||||||||||||||||
Sbjct  1457  GCAGGTTACCCCACCGACTTCGGGTGTTGCAAACTCTCGTGGTGTGACGGGCGGTGTGTA  1398

Query  61    CAAGACCCGGGAACGTATTCACCGCGGCATGCTGATCCGCGATTACTAGCAATTCCGACT  120
             ||||||||||||||||||||||||||||||||||||||||||||||||||||||||||||
Sbjct  1397  CAAGACCCGGGAACGTATTCACCGCGGCATGCTGATCCGCGATTACTAGCAATTCCGACT  1338

Query  121   TCATGCAGGCGAGTTGCAGCCTGCAATCCGAACTGAGACCGGCTTTTAAGGATTGGCTCC  180
             ||||| ||||||||||||||||||||||||||||||||||||||||||||||||||||||
Sbjct  1337  TCATGTAGGCGAGTTGCAGCCTGCAATCCGAACTGAGACCGGCTTTTAAGGATTGGCTCC  1278

Query  181   ATCTTGCGATATTGCTTCCCGTTGTACCGGCCATTGTAGTACGTGTGTAGCCCAAGTCAT  240
             ||||||||||||||||||||||||||||||||||||||||||||||||||||||||||||
Sbjct  1277  ATCTTGCGATATTGCTTCCCGTTGTACCGGCCATTGTAGTACGTGTGTAGCCCAAGTCAT  1218

Query  241   AAGGGGCATGATGATTTGACGTCATCCCCACCTTCCTCCGGTTTGTCACCGGCAGTCTGC  300
             ||||||||||||||||||||||||||||||||||||||||||||||||||||||||||||
Sbjct  1217  AAGGGGCATGATGATTTGACGTCATCCCCACCTTCCTCCGGTTTGTCACCGGCAGTCTGC  1158

Query  301   TTAGAGTGCCCACCATGATGTGCTGGCAACTAAGCATAAGGGTTGCGCTCGTTGCGGGAC  360
             ||||||||||||||||||||||||||||||||||||||||||||||||||||||||||||
Sbjct  1157  TTAGAGTGCCCACCATGATGTGCTGGCAACTAAGCATAAGGGTTGCGCTCGTTGCGGGAC  1098

Query  361   TTAACCCAACATCTCACGACACGAGCTGACGACAACCATGCACCACCTGTCTCCTTTGTC  420
             ||||||||||||||||||||||||||||||||||||||||||||||||||||||||||||
Sbjct  1097  TTAACCCAACATCTCACGACACGAGCTGACGACAACCATGCACCACCTGTCTCCTTTGTC  1038

Query  421   CCGAAGGAAAAGACTATCTCTAGTCCGGTCAAAGGGATGTCAAGACTTGGTAAGGTTCTT  480
             ||||||||||||||||||||||||||||||||||||||||||||||||||||||||||||
Sbjct  1037  CCGAAGGAAAAGACTATCTCTAGTCCGGTCAAAGGGATGTCAAGACTTGGTAAGGTTCTT  978

Query  481   CGCGTTGCTTCGAATTAAACCACATACTCCACTGCTTGTGCGGGTCCCCGTCAATTCCTT  540
             ||||||||||||||||||||||||||||||||||||||||||||||||||||||||||||
Sbjct  977   CGCGTTGCTTCGAATTAAACCACATACTCCACTGCTTGTGCGGGTCCCCGTCAATTCCTT  918

Query  541   TGAGTTTCAGTCTTGCGACCGTACTCCCCAGGCGGAATGCTTAATGTGTTAACTTCGGCA  600
             ||||||||||||||||||||||||||||||||||||||||||||||||||||||||||||
Sbjct  917   TGAGTTTCAGTCTTGCGACCGTACTCCCCAGGCGGAATGCTTAATGTGTTAACTTCGGCA  858

Query  601   CCAAGGGTATCGAAACCCCTAACACCTAGCATTCATCGTTTACGGCGTGGACTACCAGGG  660
             ||||||||||||||||||||||||||||||||||||||||||||||||||||||||||||
Sbjct  857   CCAAGGGTATCGAAACCCCTAACACCTAGCATTCATCGTTTACGGCGTGGACTACCAGGG  798

Query  661   TATCTAATCCTGTTTGCTCCCCACGCTTTCGCGCCTCAGCGTCAGTTACAGCCCAGAAAG  720
             ||||||||||||||||||||||||||||||||||||||||||||||||||||||||||||
Sbjct  797   TATCTAATCCTGTTTGCTCCCCACGCTTTCGCGCCTCAGCGTCAGTTACAGCCCAGAAAG  738

Query  721   TCGCCTTCGCCACTGGTGTTCCTCCACATCTCTACGCATTTCACCGCTACACGTGGAATT  780
             ||||||||||||||||||||||||||||||||||||||||||||||||||||||||||||
Sbjct  737   TCGCCTTCGCCACTGGTGTTCCTCCACATCTCTACGCATTTCACCGCTACACGTGGAATT  678

Query  781   CCACTTTCCTCTTCTGTACTCAAGCTCTCCAGTTTCCAGTGCGACCCAAGG-TTGAGCCT  839
             ||||||||||||||||||||||||||||||||||||||||||||||||||| ||||||||
Sbjct  677   CCACTTTCCTCTTCTGTACTCAAGCTCTCCAGTTTCCAGTGCGACCCAAGGGTTGAGCCT  618

Query  840   TGGGCTGTGACACCGGACTTAAAAAGCCGCCTGCGCGCGCTTTACGCCCAATAATTCCGG  899
             ||||||||||||||||||||||||||||||||||||||||||||||||||||||||||||
Sbjct  617   TGGGCTGTGACACCGGACTTAAAAAGCCGCCTGCGCGCGCTTTACGCCCAATAATTCCGG  558

Query  900   ACAACGCTTGCCCCCTACGTATTACCGCGGCTGCTGGCACGTAGTTAGCCGGGGCTTTCT  959
             ||||||||||||||||||||||||||||||||||||||||||||||||||||||||||||
Sbjct  557   ACAACGCTTGCCCCCTACGTATTACCGCGGCTGCTGGCACGTAGTTAGCCGGGGCTTTCT  498

Query  960   TCTCAGGTACCGTCACTCCGATAGCAGTTACTCTNTCGGAC  1000
             |||||||||||||||||||||||||||||||||| ||||||
Sbjct  497   TCTCAGGTACCGTCACTCCGATAGCAGTTACTCTATCGGAC  457
```


BLAST is a registered trademark of the National Library of Medicine

Support center
Mailing list


YouTube

- National Library Of Medicine
- National Institutes Of Health
- U.S. Department of Health & Human Services
- USA.gov

### NCBI


National Center for Biotechnology Information,
 U.S. National Library of Medicine

8600 Rockville Pike,
Bethesda
 MD,
20894
USA

Policies and Guidelines
|
Contact


PreferencesTurn off

External link. Please review our privacy policy.
