# Supplementary material for: Isothermal microcalorimetry for thermal viable count of microorganisms in pure cultures and stabilized formulations
Source: BMC Microbiol. 2019 Mar 21;19:65. doi: 10.1186/s12866-019-1432-8 (PMC6429831; doi:10.1186/s12866-019-1432-8)
Supplement: Supplementary file 3 — 16S rRNA gene sequences’ BLASTN hits in zipped HTML format. (ZIP 15810 kb) [file 12866_2019_1432_MOESM3_ESM.zip › Best blastn hits/NCBI Blast_DH5a negative F -- 14..982 of sequence.html]

NCBI Blast:DH5a negative F -- 14..982 of sequence


- NCBI Home
- Sign in to NCBI
- Skip to Main Content
- Skip to Navigation
- About NCBI Accesskeys

U.S. National Library of Medicine

NCBI
National Center for Biotechnology Information

- My NCBI
- Sign in to NCBI
- Register
- Sign Out

BLAST ^®^ » blastn suite » RID-A4XU5EU7015


- Home
- Recent Results
- Saved Strategies
- Help

BLAST Results


Edit and Resubmit
Save Search Strategies
[Sign in above to save your search strategy]

Formatting options 


Download


How to read this page
Blast report description
Questions/comments


|  |  |
| --- | --- |
| Formatting options | |
| Show | Alignment as  HTML Plain text   Old View Reset form to defaults [?]  These options control formatting of alignments in results pages. The default is HTML, but other formats (including plain text) are available. PSSM and PssmWithParameters are representations of Position Specific Scoring Matrices and are only available for PSI-BLAST. The Advanced view option allows the database descriptions to be sorted by various indices in a table. |
| Alignment View | Pairwise Pairwise with dots for identities Query-anchored with dots for identities Query-anchored with letters for identities Flat query-anchored with dots for identities Flat query-anchored with letters for identities [?]  Choose how to view alignments. The default "pairwise" view shows how each subject sequence aligns individually to the query sequence. The "query-anchored" view shows how all subject sequences align to the query sequence. For each view type, you can choose to show "identities" (matching residues) as letters or dots. more... |
| Display | Graphical Overview   Linkout   Sequence Retrieval  NCBI-gi   CDS feature [?]  - Graphical Overview: Graphical Overview: Show graph of similar sequence regions aligned to query.   more... - NCBI-gi: Show NCBI gi identifiers. - CDS feature: Show annotated coding region and translation.   more... |
| Masking | Character:   X for protein, n for nucleotide Lower Case  Color:  Black Grey Red [?]  - Masking Character: Display masked (filtered) sequence regions as lower-case or as specific letters (N for nucleotide, P for protein). - Masking Color: Display masked sequence regions in the given color. |
| Limit results | Descriptions:  10 50 100 Graphical overview:  0 10 50 100  Alignments:  0 10 50 100 Line length:  60 90 120 150 [?]  - Descriptions: Show short descriptions for up to the given number of sequences. - Alignments: Show alignments for up to the given number of sequences, in order of statistical significance. - Line lenghth: Number of letters to show on one line in an alignment. |
|  | Organism Type common name, binomial, taxid, or group name. Only 20 top taxa will be shown.     Exclude   [?]  Show only sequences from the given organism. |
|  | Entrez query:  [?]  Show only those sequences that match the given Entrez query. more... |
|  | Expect Min:  Expect Max:  [?]  Show only sequences with expect values in the given range. more... |
|  | Percent Identity Min:  Percent Identity Max:  [?]  Show only sequences with percent identity values in the given range. |
| Format for | PSI-BLAST with inclusion threshold:  [?]  - Format for PSI-BLAST: The Position-Specific Iterated BLAST (PSI-BLAST) program performs iterative searches with a protein query,   in which sequences found in one round of search are used to build a custom score model for the next round.   more... - Inclusion Threshold: This sets the statistical significance threshold for including a sequence in the model used   by PSI-BLAST to create the PSSM on the next iteration. |

|  |  |  |  |  |  |
| --- | --- | --- | --- | --- | --- |
| Download | | | | | |
| Alignment  Text XML ASN.1 JSON Seq-align Hit Table(text) Hit Table(csv) Multiple-file XML2 Single-file XML2 Multiple-file JSON Single-file JSON SAM | Search Strategies  ASN.1 | PSSM to restart search  PSSM | [?] |  |  |

The Download link provides BLAST output that may be used as input to another program.
This includes parseable formats such as the tabular report or XML as well as the Search Strategy files read by the BLAST+ applications.
More details on the parseable (XML, tabular, and ASN.1) reports can be found at
https://www.ncbi.nlm.nih.gov/books/NBK153387/  
  

The following formats are offered under the Alignment section:  
1). "Text". Non-HTML standard BLAST report.  
2). "XML". XML report based upon the DTD at https://www.ncbi.nlm.nih.gov/data\_specs/dtd/NCBI\_BlastOutput.dtd  
3). "ASN.1". Alignment written out in Abstract Syntax Notation 1.  
4). "JSON Seq-align". Alignment written out in JSON.  
4). "Hit Table(text)". The tabular report as text.  
5). "Hit Table(csv)". The tabular report ready for import into spread-sheet programs like Excel.  
6). "XML2". New XML format described at ftp://ftp.ncbi.nlm.nih.gov/blast/documents/NEWXML/xml2.pdf.  
7). "JSON". New JSON format described at ftp://ftp.ncbi.nlm.nih.gov/blast/documents/NEWXML/xml2.pdf.  
8). "SAM". Sequence Alignment Map format.

XML2 and JSON can be downloaded either as one file per query (multiple-file) or one file for all queries (single-file). These formats are listed as Multiple-file XML2 (and JSON) or Single-file XML (and JSON).

The following report is offered under the Search Strategy section:  
1). "ASN.1" Search Strategy. A record of the parameters, query, and database used in the search. This file can be used to start a stand-alone BLAST search, see
https://www.ncbi.nlm.nih.gov/books/NBK1763/#CmdLineAppsManual.I455\_BLAST\_search\_stra


# Job title: DH5a negative F -- 14..982 of sequence

Results for:

lcl|Query\_216661 DH5a negative F -- 14..982 of sequence(969bp)
[?]

Your BLAST job specified more than one input sequence.
This box lets you choose which input sequence to show BLAST results for.

RID
:   A4XU5EU7015 (Expires on 03-10 19:00 pm)

Query ID
:   lcl|Query\_216661
:   lcl|Query\_216661

Description
:   DH5a negative F -- 14..982 of sequence

Molecule type
:   nucleic acid

Query Length
:   969

Database Name
:   nr

Description
:   Nucleotide collection (nt) See details

Program
:   BLASTN 2.8.0+ Citation

  

Reference 

Zheng Zhang, Scott Schwartz, Lukas Wagner, and Webb Miller (2000), "A greedy algorithm for aligning DNA sequences", J Comput Biol 2000; 7(1-2):203-14.

Reference - database indexing

Aleksandr Morgulis, George Coulouris, Yan Raytselis, Thomas L. Madden, Richa Agarwala, Alejandro A. Schäffer (2008), "Database Indexing for Production MegaBLAST Searches", Bioinformatics 24:1757-1764.

Other reports:
Search Summary

[Taxonomy reports]
[Distance tree of results]
[MSA viewer]

Search Parameters

| Search parameter name | Search parameter value |
| --- | --- |
| Program | blastn |
| Word size | 28 |
| Expect value | 10 |
| Hitlist size | 100 |
| Match/Mismatch scores | 1,-2 |
| Gapcosts | 0,2.5 |
| Low Complexity Filter | Yes |
| Filter string | L;m; |
| Genetic Code | 1 |

Database

| Database parameter name | Database parameter value |
| --- | --- |
| Posted date | Mar 7, 2018 1:58 PM |
| Number of letters | 174,044,644,244 |
| Number of sequences | 46,882,714 |
| Entrez query | Includes:  Excludes:  None |

Karlin-Altschul statistics

| Params | Ungapped | Gapped |
| --- | --- | --- |
| Lambda | 1.33271 | 1.28 |
| K | 0.620991 | 0.46 |
| H | 1.12409 | 0.85 |

Results Statistics

| Results Statistics parameter name | Results Statistics parameter value |
| --- | --- |
| Length adjustment | 35 |
| Effective length of query | 934 |
| Effective length of database | 172403749254 |
| Effective search space | 161025101803236 |
| Effective search space used | 161025101803236 |


## Graphic Summary

### Distribution of the top 200 Blast Hits on 100 subject sequences [?]

The graphic is an overview of the database sequences aligned to the query sequence. These are represented horizontal bars colored coded by score and showing the extent
of the alignment on the query sequence. Separate aligned regions on the same database sequence are connected by a thin grey line.
Mousing over an alignment shows the database sequence title. Clicking an alignment displays a box with more details about the alignment and
link to the sequence alignment itself in the Alignments section of the report.

Mouse over to see the title, click to show alignments

Color key for alignment scores

<40

40-50

50-80

80-200

>=200

Query

1

150

300

450

600

750

900

Uncultured bacterium clone 8T12 16S ribosomal RNA gene,..

Score:1744 Evalue:0

Accession:KX212173.1

Alignment

Bacterium EM-2014-197 genomic DNA containing 16S-23S in..

Score:1744 Evalue:0

Accession:HG977405.1

Alignment

Escherichia fergusonii strain G30 16S ribosomal RNA gen..

Score:1744 Evalue:0

Accession:HQ259962.1

Alignment

Bacterium EM-2014-187 genomic DNA containing 16S-23S in..

Score:1742 Evalue:0

Accession:HG977395.1

Alignment

Escherichia coli strain IRZHN10 16S ribosomal RNA gene,..

Score:1740 Evalue:0

Accession:MG554686.1

Alignment

Escherichia coli strain ISO3 16S ribosomal RNA gene, pa..

Score:1740 Evalue:0

Accession:KY971288.1

Alignment

Escherichia coli strain E\_coli\_F\_KN 16S ribosomal RNA g..

Score:1740 Evalue:0

Accession:KY367395.1

Alignment

Escherichia coli strain NBRC 102203-89 16S ribosomal RN..

Score:1740 Evalue:0

Accession:KY655122.1

Alignment

Shigella sp. CH-12 16S ribosomal RNA gene, partial sequ..

Score:1740 Evalue:0

Accession:KR148963.1

Alignment

Uncultured bacterium clone PB149 16S ribosomal RNA gene..

Score:1740 Evalue:0

Accession:JX013430.1

Alignment

Uncultured bacterium clone PB129 16S ribosomal RNA gene..

Score:1740 Evalue:0

Accession:JX013412.1

Alignment

Uncultured bacterium clone PB118 16S ribosomal RNA gene..

Score:1740 Evalue:0

Accession:JX013402.1

Alignment

Escherichia coli strain E\_coli\_C\_K3N 16S ribosomal RNA ..

Score:1738 Evalue:0

Accession:KY367393.1

Alignment

Escherichia coli strain SUS11EC 16S ribosomal RNA gene,..

Score:1738 Evalue:0

Accession:KF991484.1

Alignment

Bacterium endosymbiont of Onthophagus Taurus clone 2L2\_..

Score:1738 Evalue:0

Accession:KF193178.1

Alignment

Uncultured bacterium clone PB220 16S ribosomal RNA gene..

Score:1738 Evalue:0

Accession:JX013449.1

Alignment

Uncultured bacterium clone PB114 16S ribosomal RNA gene..

Score:1738 Evalue:0

Accession:JX013398.1

Alignment

Uncultured bacterium clone PA129 16S ribosomal RNA gene..

Score:1738 Evalue:0

Accession:JX013298.1

Alignment

Uncultured bacterium clone PA104 16S ribosomal RNA gene..

Score:1738 Evalue:0

Accession:JX013275.1

Alignment

Uncultured Escherichia sp. clone GCL80 16S ribosomal RN..

Score:1738 Evalue:0

Accession:JX416353.1

Alignment

Escherichia coli O157 strain FDAARGOS\_293 chromosome, c..

Score:1736 Evalue:0

Accession:CP022050.2

Alignment

Escherichia coli str. K-12 substr. MG1655 strain K-12 c..

Score:1736 Evalue:0

Accession:CP027060.1

Alignment

Shigella boydii strain ATCC 49812 chromosome, complete ..

Score:1736 Evalue:0

Accession:CP026836.1

Alignment

Shigella dysenteriae strain ATCC 12039 chromosome, comp..

Score:1736 Evalue:0

Accession:CP026831.1

Alignment

Escherichia sp. strain W13a 16S ribosomal RNA gene, par..

Score:1736 Evalue:0

Accession:MG214528.1

Alignment

Escherichia coli strain DTU-1 chromosome, complete geno..

Score:1736 Evalue:0

Accession:CP026612.1

Alignment

Escherichia coli strain WCHEC005237 chromosome, complet..

Score:1736 Evalue:0

Accession:CP026580.1

Alignment

Escherichia coli strain FORC\_042 chromosome, complete g..

Score:1736 Evalue:0

Accession:CP025318.1

Alignment

Escherichia coli strain 2A chromosome, complete genome

Score:1736 Evalue:0

Accession:CP026358.1

Alignment

Escherichia coli strain 1A chromosome, complete genome

Score:1736 Evalue:0

Accession:CP026361.1

Alignment

Escherichia coli strain 2F\_0 chromosome, complete genom..

Score:1736 Evalue:0

Accession:CP026357.1

Alignment

Escherichia coli strain 4FA chromosome, complete genome

Score:1736 Evalue:0

Accession:CP026352.1

Alignment

Escherichia coli strain 4A chromosome, complete genome

Score:1736 Evalue:0

Accession:CP026353.1

Alignment

Escherichia coli strain 5A chromosome, complete genome

Score:1736 Evalue:0

Accession:CP026351.1

Alignment

Escherichia coli strain 1FA chromosome, complete genome

Score:1736 Evalue:0

Accession:CP026360.1

Alignment

Escherichia coli strain 3FA chromosome, complete genome

Score:1736 Evalue:0

Accession:CP026354.1

Alignment

Escherichia coli strain 8A chromosome, complete genome

Score:1736 Evalue:0

Accession:CP026345.1

Alignment

Escherichia coli strain 5FA chromosome, complete genome

Score:1736 Evalue:0

Accession:CP026350.1

Alignment

Escherichia coli strain 7A chromosome, complete genome

Score:1736 Evalue:0

Accession:CP026347.1

Alignment

Escherichia coli strain 7FA chromosome, complete genome

Score:1736 Evalue:0

Accession:CP026346.1

Alignment

Escherichia coli strain 9FA chromosome, complete genome

Score:1736 Evalue:0

Accession:CP026342.1

Alignment

Escherichia coli strain 9A chromosome, complete genome

Score:1736 Evalue:0

Accession:CP026343.1

Alignment

Escherichia coli strain 6FA chromosome, complete genome

Score:1736 Evalue:0

Accession:CP026348.1

Alignment

Escherichia coli strain 6A chromosome, complete genome

Score:1736 Evalue:0

Accession:CP026349.1

Alignment

Escherichia coli strain 2\_0 chromosome, complete genome

Score:1736 Evalue:0

Accession:CP026359.1

Alignment

Escherichia coli strain 8FA chromosome, complete genome

Score:1736 Evalue:0

Accession:CP026344.1

Alignment

Escherichia coli strain 2FA chromosome, complete genome

Score:1736 Evalue:0

Accession:CP026356.1

Alignment

Escherichia coli strain 3A chromosome, complete genome

Score:1736 Evalue:0

Accession:CP026355.1

Alignment

Escherichia coli strain ECONIH6 chromosome, complete ge..

Score:1736 Evalue:0

Accession:CP026199.1

Alignment

Escherichia coli strain DH5alpha chromosome, complete g..

Score:1736 Evalue:0

Accession:CP026085.1

Alignment

Escherichia coli strain LIM chromosome, complete genome

Score:1736 Evalue:0

Accession:CP026027.1

Alignment

Escherichia coli strain CAR chromosome, complete genome

Score:1736 Evalue:0

Accession:CP026026.1

Alignment

Escherichia coli strain CIT chromosome, complete genome

Score:1736 Evalue:0

Accession:CP026028.1

Alignment

Escherichia coli strain SCEC020023 chromosome, complete..

Score:1736 Evalue:0

Accession:CP025950.1

Alignment

Escherichia coli strain CV839-06 chromosome, complete g..

Score:1736 Evalue:0

Accession:CP025753.1

Alignment

Escherichia coli strain ML35 chromosome, complete genom..

Score:1736 Evalue:0

Accession:CP025747.1

Alignment

## Descriptions

, AlignDB=on Reading indexes 1-5, displaying indexes 1-5


Load next setPrevious Match

Sequences producing significant alignments:

Show all columns  of the table presenting sequences producing significant alignments 

Select:AllNone
Selected:0

Alignments
Download

FASTA (complete sequence)

FASTA (aligned sequences)

GenBank (complete sequence)

Hit Table (text)

Hit Table (CSV)

Text

XML

ASN.1

Continue
Cancel

GenBank 
Graphics
Distance tree of results
Multiple alignment
Show/hide columns of the table presenting sequences producing significant alignments 

Available columns

Description  
Max Score  
Total Score  
Coverage  
E-value  
IdentN  
Accession  
Restore Defaults
Ok
Cancel

Sequences producing significant alignments:

| Select for downloading or viewing reports | Description | Max score | Total score | Query cover | E value | Ident | Accession |
| --- | --- | --- | --- | --- | --- | --- | --- |
| 1Select seq KX212173.1 | Uncultured bacterium clone 8T12 16S ribosomal RNA gene, partial sequence | 1744 | 1744 | 98% | 0.0 | 99% | KX212173.1 |
| 2Select seq HG977405.1 | Bacterium EM-2014-197 genomic DNA containing 16S-23S intergenic spacer region, isolate 197 | 1744 | 1744 | 98% | 0.0 | 99% | HG977405.1 |
| 3Select seq HQ259962.1 | Escherichia fergusonii strain G30 16S ribosomal RNA gene, partial sequence | 1744 | 1744 | 98% | 0.0 | 99% | HQ259962.1 |
| 4Select seq HG977395.1 | Bacterium EM-2014-187 genomic DNA containing 16S-23S intergenic spacer region, isolate 187 | 1742 | 1742 | 98% | 0.0 | 99% | HG977395.1 |
| 5Select seq MG554686.1 | Escherichia coli strain IRZHN10 16S ribosomal RNA gene, partial sequence | 1740 | 1740 | 98% | 0.0 | 99% | MG554686.1 |
| 6Select seq KY971288.1 | Escherichia coli strain ISO3 16S ribosomal RNA gene, partial sequence | 1740 | 1740 | 98% | 0.0 | 99% | KY971288.1 |
| 7Select seq KY367395.1 | Escherichia coli strain E\_coli\_F\_KN 16S ribosomal RNA gene, partial sequence | 1740 | 1740 | 98% | 0.0 | 99% | KY367395.1 |
| 8Select seq KY655122.1 | Escherichia coli strain NBRC 102203-89 16S ribosomal RNA gene, partial sequence | 1740 | 1740 | 98% | 0.0 | 99% | KY655122.1 |
| 9Select seq KR148963.1 | Shigella sp. CH-12 16S ribosomal RNA gene, partial sequence | 1740 | 1740 | 98% | 0.0 | 99% | KR148963.1 |
| 10Select seq JX013430.1 | Uncultured bacterium clone PB149 16S ribosomal RNA gene, partial sequence | 1740 | 1740 | 98% | 0.0 | 99% | JX013430.1 |
| 11Select seq JX013412.1 | Uncultured bacterium clone PB129 16S ribosomal RNA gene, partial sequence | 1740 | 1740 | 98% | 0.0 | 99% | JX013412.1 |
| 12Select seq JX013402.1 | Uncultured bacterium clone PB118 16S ribosomal RNA gene, partial sequence | 1740 | 1740 | 98% | 0.0 | 99% | JX013402.1 |
| 13Select seq KY367393.1 | Escherichia coli strain E\_coli\_C\_K3N 16S ribosomal RNA gene, partial sequence | 1738 | 1738 | 98% | 0.0 | 99% | KY367393.1 |
| 14Select seq KF991484.1 | Escherichia coli strain SUS11EC 16S ribosomal RNA gene, partial sequence | 1738 | 1738 | 98% | 0.0 | 99% | KF991484.1 |
| 15Select seq KF193178.1 | Bacterium endosymbiont of Onthophagus Taurus clone 2L2\_NC1\_003 16S ribosomal RNA gene, partial sequence | 1738 | 1738 | 98% | 0.0 | 99% | KF193178.1 |
| 16Select seq JX013449.1 | Uncultured bacterium clone PB220 16S ribosomal RNA gene, partial sequence | 1738 | 1738 | 98% | 0.0 | 99% | JX013449.1 |
| 17Select seq JX013398.1 | Uncultured bacterium clone PB114 16S ribosomal RNA gene, partial sequence | 1738 | 1738 | 98% | 0.0 | 99% | JX013398.1 |
| 18Select seq JX013298.1 | Uncultured bacterium clone PA129 16S ribosomal RNA gene, partial sequence | 1738 | 1738 | 98% | 0.0 | 99% | JX013298.1 |
| 19Select seq JX013275.1 | Uncultured bacterium clone PA104 16S ribosomal RNA gene, partial sequence | 1738 | 1738 | 98% | 0.0 | 99% | JX013275.1 |
| 20Select seq JX416353.1 | Uncultured Escherichia sp. clone GCL80 16S ribosomal RNA gene, partial sequence | 1738 | 1738 | 98% | 0.0 | 99% | JX416353.1 |
| 21Select seq CP022050.2 | Escherichia coli O157 strain FDAARGOS\_293 chromosome, complete genome | 1736 | 12053 | 98% | 0.0 | 99% | CP022050.2 |
| 22Select seq CP027060.1 | Escherichia coli str. K-12 substr. MG1655 strain K-12 chromosome | 1736 | 12066 | 98% | 0.0 | 99% | CP027060.1 |
| 23Select seq CP026836.1 | Shigella boydii strain ATCC 49812 chromosome, complete genome | 1736 | 12125 | 98% | 0.0 | 99% | CP026836.1 |
| 24Select seq CP026831.1 | Shigella dysenteriae strain ATCC 12039 chromosome, complete genome | 1736 | 12131 | 98% | 0.0 | 99% | CP026831.1 |
| 25Select seq MG214528.1 | Escherichia sp. strain W13a 16S ribosomal RNA gene, partial sequence | 1736 | 1736 | 98% | 0.0 | 99% | MG214528.1 |
| 26Select seq CP026612.1 | Escherichia coli strain DTU-1 chromosome, complete genome | 1736 | 12072 | 98% | 0.0 | 99% | CP026612.1 |
| 27Select seq CP026580.1 | Escherichia coli strain WCHEC005237 chromosome, complete genome | 1736 | 12097 | 98% | 0.0 | 99% | CP026580.1 |
| 28Select seq CP025318.1 | Escherichia coli strain FORC\_042 chromosome, complete genome | 1736 | 12094 | 98% | 0.0 | 99% | CP025318.1 |
| 29Select seq CP026358.1 | Escherichia coli strain 2A chromosome, complete genome | 1736 | 12066 | 98% | 0.0 | 99% | CP026358.1 |
| 30Select seq CP026361.1 | Escherichia coli strain 1A chromosome, complete genome | 1736 | 12066 | 98% | 0.0 | 99% | CP026361.1 |
| 31Select seq CP026357.1 | Escherichia coli strain 2F\_0 chromosome, complete genome | 1736 | 12066 | 98% | 0.0 | 99% | CP026357.1 |
| 32Select seq CP026352.1 | Escherichia coli strain 4FA chromosome, complete genome | 1736 | 12066 | 98% | 0.0 | 99% | CP026352.1 |
| 33Select seq CP026353.1 | Escherichia coli strain 4A chromosome, complete genome | 1736 | 12066 | 98% | 0.0 | 99% | CP026353.1 |
| 34Select seq CP026351.1 | Escherichia coli strain 5A chromosome, complete genome | 1736 | 12066 | 98% | 0.0 | 99% | CP026351.1 |
| 35Select seq CP026360.1 | Escherichia coli strain 1FA chromosome, complete genome | 1736 | 12066 | 98% | 0.0 | 99% | CP026360.1 |
| 36Select seq CP026354.1 | Escherichia coli strain 3FA chromosome, complete genome | 1736 | 12066 | 98% | 0.0 | 99% | CP026354.1 |
| 37Select seq CP026345.1 | Escherichia coli strain 8A chromosome, complete genome | 1736 | 12066 | 98% | 0.0 | 99% | CP026345.1 |
| 38Select seq CP026350.1 | Escherichia coli strain 5FA chromosome, complete genome | 1736 | 12066 | 98% | 0.0 | 99% | CP026350.1 |
| 39Select seq CP026347.1 | Escherichia coli strain 7A chromosome, complete genome | 1736 | 12066 | 98% | 0.0 | 99% | CP026347.1 |
| 40Select seq CP026346.1 | Escherichia coli strain 7FA chromosome, complete genome | 1736 | 12066 | 98% | 0.0 | 99% | CP026346.1 |
| 41Select seq CP026342.1 | Escherichia coli strain 9FA chromosome, complete genome | 1736 | 12066 | 98% | 0.0 | 99% | CP026342.1 |
| 42Select seq CP026343.1 | Escherichia coli strain 9A chromosome, complete genome | 1736 | 12066 | 98% | 0.0 | 99% | CP026343.1 |
| 43Select seq CP026348.1 | Escherichia coli strain 6FA chromosome, complete genome | 1736 | 12066 | 98% | 0.0 | 99% | CP026348.1 |
| 44Select seq CP026349.1 | Escherichia coli strain 6A chromosome, complete genome | 1736 | 12066 | 98% | 0.0 | 99% | CP026349.1 |
| 45Select seq CP026359.1 | Escherichia coli strain 2\_0 chromosome, complete genome | 1736 | 12066 | 98% | 0.0 | 99% | CP026359.1 |
| 46Select seq CP026344.1 | Escherichia coli strain 8FA chromosome, complete genome | 1736 | 12066 | 98% | 0.0 | 99% | CP026344.1 |
| 47Select seq CP026356.1 | Escherichia coli strain 2FA chromosome, complete genome | 1736 | 12066 | 98% | 0.0 | 99% | CP026356.1 |
| 48Select seq CP026355.1 | Escherichia coli strain 3A chromosome, complete genome | 1736 | 12066 | 98% | 0.0 | 99% | CP026355.1 |
| 49Select seq CP026199.1 | Escherichia coli strain ECONIH6 chromosome, complete genome | 1736 | 12081 | 98% | 0.0 | 99% | CP026199.1 |
| 50Select seq CP026085.1 | Escherichia coli strain DH5alpha chromosome, complete genome | 1736 | 12014 | 98% | 0.0 | 99% | CP026085.1 |
| 51Select seq CP026027.1 | Escherichia coli strain LIM chromosome, complete genome | 1736 | 12066 | 98% | 0.0 | 99% | CP026027.1 |
| 52Select seq CP026026.1 | Escherichia coli strain CAR chromosome, complete genome | 1736 | 12066 | 98% | 0.0 | 99% | CP026026.1 |
| 53Select seq CP026028.1 | Escherichia coli strain CIT chromosome, complete genome | 1736 | 12066 | 98% | 0.0 | 99% | CP026028.1 |
| 54Select seq CP025950.1 | Escherichia coli strain SCEC020023 chromosome, complete genome | 1736 | 12153 | 98% | 0.0 | 99% | CP025950.1 |
| 55Select seq CP025753.1 | Escherichia coli strain CV839-06 chromosome, complete genome | 1736 | 12066 | 98% | 0.0 | 99% | CP025753.1 |
| 56Select seq CP025747.1 | Escherichia coli strain ML35 chromosome, complete genome | 1736 | 12088 | 98% | 0.0 | 99% | CP025747.1 |
| 57Select seq MF620019.1 | Uncultured bacterium clone P28H1 genomic sequence | 1736 | 1736 | 98% | 0.0 | 99% | MF620019.1 |
| 58Select seq CP025739.1 | Escherichia coli strain Ec40 chromosome | 1736 | 12057 | 98% | 0.0 | 99% | CP025739.1 |
| 59Select seq CP025627.1 | Escherichia coli strain SCEC020007 chromosome, complete genome | 1736 | 12092 | 98% | 0.0 | 99% | CP025627.1 |
| 60Select seq CP019071.1 | Escherichia coli strain CRE1493 chromosome, complete genome | 1736 | 12072 | 98% | 0.0 | 99% | CP019071.1 |
| 61Select seq CP024127.1 | Escherichia coli strain 14EC001 chromosome, complete genome | 1736 | 11957 | 98% | 0.0 | 99% | CP024127.1 |
| 62Select seq CP024141.1 | Escherichia coli strain 14EC029 chromosome, complete genome | 1736 | 12020 | 98% | 0.0 | 99% | CP024141.1 |
| 63Select seq CP025520.1 | Escherichia coli strain DH5alpha chromosome, complete genome | 1736 | 12055 | 98% | 0.0 | 99% | CP025520.1 |
| 64Select seq CP025268.1 | Escherichia coli str. K-12 substr. MG1655 strain K-12 chromosome, complete genome | 1736 | 12066 | 98% | 0.0 | 99% | CP025268.1 |
| 65Select seq CP025036.1 | Escherichia coli strain S17-20 chromosome, complete genome | 1736 | 12005 | 98% | 0.0 | 99% | CP025036.1 |
| 66Select seq CP025048.1 | Escherichia coli strain SC516 chromosome, complete genome | 1736 | 12094 | 98% | 0.0 | 99% | CP025048.1 |
| 67Select seq LT883142.1 | Escherichia coli isolate 6666666.257727.embl genome assembly, chromosome: IV | 1736 | 12053 | 98% | 0.0 | 99% | LT883142.1 |
| 68Select seq CP024997.1 | Escherichia coli strain S17-13 chromosome, complete genome | 1736 | 12005 | 98% | 0.0 | 99% | CP024997.1 |
| 69Select seq CP024859.1 | Escherichia coli strain AR\_0014, complete genome | 1736 | 12072 | 98% | 0.0 | 99% | CP024859.1 |
| 70Select seq CP024855.1 | Escherichia coli strain AR\_0011, complete genome | 1736 | 12072 | 98% | 0.0 | 99% | CP024855.1 |
| 71Select seq CP024830.1 | Escherichia coli strain CREC-532 chromosome, complete genome | 1736 | 12064 | 98% | 0.0 | 99% | CP024830.1 |
| 72Select seq CP024815.1 | Escherichia coli strain CREC-629 chromosome, complete genome | 1736 | 12072 | 98% | 0.0 | 99% | CP024815.1 |
| 73Select seq CP024661.1 | Escherichia coli strain 90-9269 chromosome | 1736 | 12136 | 98% | 0.0 | 99% | CP024661.1 |
| 74Select seq CP024090.1 | Escherichia coli strain WG5 chromosome, complete genome | 1736 | 12103 | 98% | 0.0 | 99% | CP024090.1 |
| 75Select seq CP024275.1 | Escherichia coli O6:H16 strain M9682-C1 chromosome, complete genome | 1736 | 12047 | 98% | 0.0 | 99% | CP024275.1 |
| 76Select seq CP024245.1 | Escherichia coli O27:H7 strain B4103-1 chromosome, complete genome | 1736 | 12096 | 98% | 0.0 | 99% | CP024245.1 |
| 77Select seq CP024239.1 | Escherichia coli O15:H11 strain 90-9272 chromosome, complete genome | 1736 | 12046 | 98% | 0.0 | 99% | CP024239.1 |
| 78Select seq CP023870.1 | Escherichia coli strain FDAARGOS\_434 chromosome, complete genome | 1736 | 12064 | 98% | 0.0 | 99% | CP023870.1 |
| 79Select seq CP023383.1 | Escherichia coli strain 1223 chromosome, complete genome | 1736 | 12099 | 98% | 0.0 | 99% | CP023383.1 |
| 80Select seq CP023371.1 | Escherichia coli strain 1283 chromosome, complete genome | 1736 | 12077 | 98% | 0.0 | 99% | CP023371.1 |
| 81Select seq CP023359.1 | Escherichia coli strain 1943 chromosome, complete genome | 1736 | 12023 | 98% | 0.0 | 99% | CP023359.1 |
| 82Select seq CP023353.1 | Escherichia coli strain 746 chromosome, complete genome | 1736 | 12055 | 98% | 0.0 | 99% | CP023353.1 |
| 83Select seq MF919609.1 | Escherichia coli strain ETEC 16S ribosomal RNA gene, partial sequence | 1736 | 1736 | 98% | 0.0 | 99% | MF919609.1 |
| 84Select seq CP023142.1 | Escherichia coli strain CFSAN061770, complete genome | 1736 | 12096 | 98% | 0.0 | 99% | CP023142.1 |
| 85Select seq LT906474.1 | Escherichia coli strain NCTC122 genome assembly, chromosome: 1 | 1736 | 12081 | 98% | 0.0 | 99% | LT906474.1 |
| 86Select seq CP015843.2 | Escherichia coli O157:H7 strain FRIK2455, complete genome | 1736 | 12084 | 98% | 0.0 | 99% | CP015843.2 |
| 87Select seq 5O2R\_AA | Chain a, Cryo-EM structure of the proline-rich antimicrobial peptide Api137 bound to the terminating ribosome | 1736 | 1736 | 98% | 0.0 | 99% | 5O2R\_AA |
| 88Select seq CP022414.1 | Escherichia coli strain AT01 genome | 1736 | 12066 | 98% | 0.0 | 99% | CP022414.1 |
| 89Select seq MF372556.1 | Escherichia coli strain 13482 16S ribosomal RNA gene, partial sequence | 1736 | 1736 | 98% | 0.0 | 99% | MF372556.1 |
| 90Select seq MF372555.1 | Escherichia coli strain 13481 16S ribosomal RNA gene, partial sequence | 1736 | 1736 | 98% | 0.0 | 99% | MF372555.1 |
| 91Select seq CP021879.1 | Escherichia coli strain AR\_0137, complete genome | 1736 | 12084 | 98% | 0.0 | 99% | CP021879.1 |
| 92Select seq CP021736.1 | Escherichia coli strain AR\_0150, complete genome | 1736 | 12057 | 98% | 0.0 | 99% | CP021736.1 |
| 93Select seq CP021732.1 | Escherichia coli strain AR\_0114, complete genome | 1736 | 12079 | 98% | 0.0 | 99% | CP021732.1 |
| 94Select seq CP021691.1 | Escherichia coli strain AR\_0151, complete genome | 1736 | 12057 | 98% | 0.0 | 99% | CP021691.1 |
| 95Select seq CP021683.1 | Escherichia coli strain AR\_0162, complete genome | 1736 | 12072 | 98% | 0.0 | 99% | CP021683.1 |
| 96Select seq CP021532.1 | Escherichia coli strain AR\_0149, complete genome | 1736 | 12066 | 98% | 0.0 | 99% | CP021532.1 |
| 97Select seq CP019558.1 | Escherichia coli strain KSC207 chromosome, complete genome | 1736 | 11939 | 98% | 0.0 | 99% | CP019558.1 |
| 98Select seq CP018840.1 | Escherichia coli strain KSC64 chromosome, complete genome | 1736 | 12090 | 98% | 0.0 | 99% | CP018840.1 |
| 99Select seq CP018323.1 | Escherichia coli strain KSC9 chromosome, complete genome | 1736 | 12062 | 98% | 0.0 | 99% | CP018323.1 |
| 100Select seq KF938590.1 | Escherichia fergusonii strain Sw2 16S ribosomal RNA gene, partial sequence | 1735 | 1735 | 98% | 0.0 | 99% | KF938590.1 |


## Alignments

Loading alignment... for sequences gi|1133373608,gi|713633968,gi|322392910,gi|713633945,gi|1279489882 AlignDB=on Reading indexes 1-5

Download

FASTA (complete sequence)

FASTA (aligned sequences)

GenBank (complete sequence)

Continue
Cancel

GenBankGraphics

Next
Previous
Descriptions

Uncultured bacterium clone 8T12 16S ribosomal RNA gene, partial sequence

Sequence ID: KX212173.1Length: 1389Number of Matches: 1

Related Information

Range 1: 2 to 957GenBankGraphics

Next Match
Previous Match
First Match

Alignment statistics for match #1

| Score | Expect | Identities | Gaps | Strand | Frame |
| --- | --- | --- | --- | --- | --- |
| 1744 bits(944) | 0.0() | 950/956(99%) | 0/956(0%) | Plus/Plus |  |

Features:

```
Query  6    TGCAGTCGNACGGTAACAGGAANNAAGCTTGCTNNTTNGCTGACGAGTGGCGGACGGGTG  65
            |||||||| |||||||||||||  |||||||||  || ||||||||||||||||||||||
Sbjct  2    TGCAGTCGAACGGTAACAGGAAGCAAGCTTGCTTCTTCGCTGACGAGTGGCGGACGGGTG  61

Query  66   AGTAATGTCTGGGAAACTGCCTGATGGAGGGGGATAACTACTGGAAACGGTAGCTAATAC  125
            ||||||||||||||||||||||||||||||||||||||||||||||||||||||||||||
Sbjct  62   AGTAATGTCTGGGAAACTGCCTGATGGAGGGGGATAACTACTGGAAACGGTAGCTAATAC  121

Query  126  CGCATAACGTCGCAAGACCAAAGAGGGGGACCTTCGGGCCTCTTGCCATCGGATGTGCCC  185
            ||||||||||||||||||||||||||||||||||||||||||||||||||||||||||||
Sbjct  122  CGCATAACGTCGCAAGACCAAAGAGGGGGACCTTCGGGCCTCTTGCCATCGGATGTGCCC  181

Query  186  AGATGGGATTAGCTAGTAGGTGGGGTAACGGCTCACCTAGGCGACGATCCCTAGCTGGTC  245
            ||||||||||||||||||||||||||||||||||||||||||||||||||||||||||||
Sbjct  182  AGATGGGATTAGCTAGTAGGTGGGGTAACGGCTCACCTAGGCGACGATCCCTAGCTGGTC  241

Query  246  TGAGAGGATGACCAGCCACACTGGAACTGAGACACGGTCCAGACTCCTACGGGAGGCAGC  305
            ||||||||||||||||||||||||||||||||||||||||||||||||||||||||||||
Sbjct  242  TGAGAGGATGACCAGCCACACTGGAACTGAGACACGGTCCAGACTCCTACGGGAGGCAGC  301

Query  306  AGTGGGGAATATTGCACAATGGGCGCAAGCCTGATGCAGCCATGCCGCGTGTATGAAGAA  365
            ||||||||||||||||||||||||||||||||||||||||||||||||||||||||||||
Sbjct  302  AGTGGGGAATATTGCACAATGGGCGCAAGCCTGATGCAGCCATGCCGCGTGTATGAAGAA  361

Query  366  GGCCTTCGGGTTGTAAAGTACTTTCAGCGGGGAGGAAGGGAGTAAAGTTAATACCTTTGC  425
            ||||||||||||||||||||||||||||||||||||||||||||||||||||||||||||
Sbjct  362  GGCCTTCGGGTTGTAAAGTACTTTCAGCGGGGAGGAAGGGAGTAAAGTTAATACCTTTGC  421

Query  426  TCATTGACGTTACCCGCAGAAGAAGCACCGGCTAACTCCGTGCCAGCAGCCGCGGTAATA  485
            ||||||||||||||||||||||||||||||||||||||||||||||||||||||||||||
Sbjct  422  TCATTGACGTTACCCGCAGAAGAAGCACCGGCTAACTCCGTGCCAGCAGCCGCGGTAATA  481

Query  486  CGGAGGGTGCAAGCGTTAATCGGAATTACTGGGCGTAAAGCGCACGCAGGCGGTTTGTTA  545
            ||||||||||||||||||||||||||||||||||||||||||||||||||||||||||||
Sbjct  482  CGGAGGGTGCAAGCGTTAATCGGAATTACTGGGCGTAAAGCGCACGCAGGCGGTTTGTTA  541

Query  546  AGTCAGATGTGAAATCCCCGGGCTCAACCTGGGAACTGCATCTGATACTGGCAAGCTTGA  605
            ||||||||||||||||||||||||||||||||||||||||||||||||||||||||||||
Sbjct  542  AGTCAGATGTGAAATCCCCGGGCTCAACCTGGGAACTGCATCTGATACTGGCAAGCTTGA  601

Query  606  GTCTCGTAGAGGGGGGTAGAATTCCAGGTGTAGCGGTGAAATGCGTAGAGATCTGGAGGA  665
            ||||||||||||||||||||||||||||||||||||||||||||||||||||||||||||
Sbjct  602  GTCTCGTAGAGGGGGGTAGAATTCCAGGTGTAGCGGTGAAATGCGTAGAGATCTGGAGGA  661

Query  666  ATACCGGTGGCGAAGGCGGCCCCCTGGACGAAGACTGACGCTCAGGTGCGAAAGCGTGGG  725
            ||||||||||||||||||||||||||||||||||||||||||||||||||||||||||||
Sbjct  662  ATACCGGTGGCGAAGGCGGCCCCCTGGACGAAGACTGACGCTCAGGTGCGAAAGCGTGGG  721

Query  726  GAGCAAACAGGATTAGATACCCTGGTAGTCCACGCCGTAAACGATGTCGACTTGGAGGTT  785
            ||||||||||||||||||||||||||||||||||||||||||||||||||||||||||||
Sbjct  722  GAGCAAACAGGATTAGATACCCTGGTAGTCCACGCCGTAAACGATGTCGACTTGGAGGTT  781

Query  786  GTGCCCTTGAGGCGTGGCTTCCGGAGCTAACGCGTTAAGTCGACCGCCTGGGGAGTACGG  845
            ||||||||||||||||||||||||||||||||||||||||||||||||||||||||||||
Sbjct  782  GTGCCCTTGAGGCGTGGCTTCCGGAGCTAACGCGTTAAGTCGACCGCCTGGGGAGTACGG  841

Query  846  CCGCAAGGTTAAAACTCAAATGAATTGACGGGGGCCCGCACAAGCGGTGGAGCATGTGGT  905
            ||||||||||||||||||||||||||||||||||||||||||||||||||||||||||||
Sbjct  842  CCGCAAGGTTAAAACTCAAATGAATTGACGGGGGCCCGCACAAGCGGTGGAGCATGTGGT  901

Query  906  TTAATTCGATGCAACGCGAAGAACCTTACCTGGTCTTGACATCCACGGAAGTTTTC  961
            ||||||||||||||||||||||||||||||||||||||||||||||||||||||||
Sbjct  902  TTAATTCGATGCAACGCGAAGAACCTTACCTGGTCTTGACATCCACGGAAGTTTTC  957
```

Download

FASTA (complete sequence)

FASTA (aligned sequences)

GenBank (complete sequence)

Continue
Cancel

GenBankGraphics

Next
Previous
Descriptions

Bacterium EM-2014-197 genomic DNA containing 16S-23S intergenic spacer region, isolate 197

Sequence ID: HG977405.1Length: 1393Number of Matches: 1

Related Information

Range 1: 438 to 1393GenBankGraphics

Next Match
Previous Match
First Match

Alignment statistics for match #1

| Score | Expect | Identities | Gaps | Strand | Frame |
| --- | --- | --- | --- | --- | --- |
| 1744 bits(944) | 0.0() | 950/956(99%) | 0/956(0%) | Plus/Minus |  |

Features:

```
Query  6     TGCAGTCGNACGGTAACAGGAANNAAGCTTGCTNNTTNGCTGACGAGTGGCGGACGGGTG  65
             |||||||| |||||||||||||  |||||||||  || ||||||||||||||||||||||
Sbjct  1393  TGCAGTCGAACGGTAACAGGAAACAAGCTTGCTTCTTTGCTGACGAGTGGCGGACGGGTG  1334

Query  66    AGTAATGTCTGGGAAACTGCCTGATGGAGGGGGATAACTACTGGAAACGGTAGCTAATAC  125
             ||||||||||||||||||||||||||||||||||||||||||||||||||||||||||||
Sbjct  1333  AGTAATGTCTGGGAAACTGCCTGATGGAGGGGGATAACTACTGGAAACGGTAGCTAATAC  1274

Query  126   CGCATAACGTCGCAAGACCAAAGAGGGGGACCTTCGGGCCTCTTGCCATCGGATGTGCCC  185
             ||||||||||||||||||||||||||||||||||||||||||||||||||||||||||||
Sbjct  1273  CGCATAACGTCGCAAGACCAAAGAGGGGGACCTTCGGGCCTCTTGCCATCGGATGTGCCC  1214

Query  186   AGATGGGATTAGCTAGTAGGTGGGGTAACGGCTCACCTAGGCGACGATCCCTAGCTGGTC  245
             ||||||||||||||||||||||||||||||||||||||||||||||||||||||||||||
Sbjct  1213  AGATGGGATTAGCTAGTAGGTGGGGTAACGGCTCACCTAGGCGACGATCCCTAGCTGGTC  1154

Query  246   TGAGAGGATGACCAGCCACACTGGAACTGAGACACGGTCCAGACTCCTACGGGAGGCAGC  305
             ||||||||||||||||||||||||||||||||||||||||||||||||||||||||||||
Sbjct  1153  TGAGAGGATGACCAGCCACACTGGAACTGAGACACGGTCCAGACTCCTACGGGAGGCAGC  1094

Query  306   AGTGGGGAATATTGCACAATGGGCGCAAGCCTGATGCAGCCATGCCGCGTGTATGAAGAA  365
             ||||||||||||||||||||||||||||||||||||||||||||||||||||||||||||
Sbjct  1093  AGTGGGGAATATTGCACAATGGGCGCAAGCCTGATGCAGCCATGCCGCGTGTATGAAGAA  1034

Query  366   GGCCTTCGGGTTGTAAAGTACTTTCAGCGGGGAGGAAGGGAGTAAAGTTAATACCTTTGC  425
             ||||||||||||||||||||||||||||||||||||||||||||||||||||||||||||
Sbjct  1033  GGCCTTCGGGTTGTAAAGTACTTTCAGCGGGGAGGAAGGGAGTAAAGTTAATACCTTTGC  974

Query  426   TCATTGACGTTACCCGCAGAAGAAGCACCGGCTAACTCCGTGCCAGCAGCCGCGGTAATA  485
             ||||||||||||||||||||||||||||||||||||||||||||||||||||||||||||
Sbjct  973   TCATTGACGTTACCCGCAGAAGAAGCACCGGCTAACTCCGTGCCAGCAGCCGCGGTAATA  914

Query  486   CGGAGGGTGCAAGCGTTAATCGGAATTACTGGGCGTAAAGCGCACGCAGGCGGTTTGTTA  545
             ||||||||||||||||||||||||||||||||||||||||||||||||||||||||||||
Sbjct  913   CGGAGGGTGCAAGCGTTAATCGGAATTACTGGGCGTAAAGCGCACGCAGGCGGTTTGTTA  854

Query  546   AGTCAGATGTGAAATCCCCGGGCTCAACCTGGGAACTGCATCTGATACTGGCAAGCTTGA  605
             ||||||||||||||||||||||||||||||||||||||||||||||||||||||||||||
Sbjct  853   AGTCAGATGTGAAATCCCCGGGCTCAACCTGGGAACTGCATCTGATACTGGCAAGCTTGA  794

Query  606   GTCTCGTAGAGGGGGGTAGAATTCCAGGTGTAGCGGTGAAATGCGTAGAGATCTGGAGGA  665
             ||||||||||||||||||||||||||||||||||||||||||||||||||||||||||||
Sbjct  793   GTCTCGTAGAGGGGGGTAGAATTCCAGGTGTAGCGGTGAAATGCGTAGAGATCTGGAGGA  734

Query  666   ATACCGGTGGCGAAGGCGGCCCCCTGGACGAAGACTGACGCTCAGGTGCGAAAGCGTGGG  725
             ||||||||||||||||||||||||||||||||||||||||||||||||||||||||||||
Sbjct  733   ATACCGGTGGCGAAGGCGGCCCCCTGGACGAAGACTGACGCTCAGGTGCGAAAGCGTGGG  674

Query  726   GAGCAAACAGGATTAGATACCCTGGTAGTCCACGCCGTAAACGATGTCGACTTGGAGGTT  785
             ||||||||||||||||||||||||||||||||||||||||||||||||||||||||||||
Sbjct  673   GAGCAAACAGGATTAGATACCCTGGTAGTCCACGCCGTAAACGATGTCGACTTGGAGGTT  614

Query  786   GTGCCCTTGAGGCGTGGCTTCCGGAGCTAACGCGTTAAGTCGACCGCCTGGGGAGTACGG  845
             ||||||||||||||||||||||||||||||||||||||||||||||||||||||||||||
Sbjct  613   GTGCCCTTGAGGCGTGGCTTCCGGAGCTAACGCGTTAAGTCGACCGCCTGGGGAGTACGG  554

Query  846   CCGCAAGGTTAAAACTCAAATGAATTGACGGGGGCCCGCACAAGCGGTGGAGCATGTGGT  905
             ||||||||||||||||||||||||||||||||||||||||||||||||||||||||||||
Sbjct  553   CCGCAAGGTTAAAACTCAAATGAATTGACGGGGGCCCGCACAAGCGGTGGAGCATGTGGT  494

Query  906   TTAATTCGATGCAACGCGAAGAACCTTACCTGGTCTTGACATCCACGGAAGTTTTC  961
             ||||||||||||||||||||||||||||||||||||||||||||||||||||||||
Sbjct  493   TTAATTCGATGCAACGCGAAGAACCTTACCTGGTCTTGACATCCACGGAAGTTTTC  438
```

Download

FASTA (complete sequence)

FASTA (aligned sequences)

GenBank (complete sequence)

Continue
Cancel

GenBankGraphics

Next
Previous
Descriptions

Escherichia fergusonii strain G30 16S ribosomal RNA gene, partial sequence

Sequence ID: HQ259962.1Length: 1449Number of Matches: 1

Related Information

Range 1: 22 to 977GenBankGraphics

Next Match
Previous Match
First Match

Alignment statistics for match #1

| Score | Expect | Identities | Gaps | Strand | Frame |
| --- | --- | --- | --- | --- | --- |
| 1744 bits(944) | 0.0() | 950/956(99%) | 0/956(0%) | Plus/Plus |  |

Features:

```
Query  6    TGCAGTCGNACGGTAACAGGAANNAAGCTTGCTNNTTNGCTGACGAGTGGCGGACGGGTG  65
            |||||||| |||||||||||||  |||||||||  || ||||||||||||||||||||||
Sbjct  22   TGCAGTCGAACGGTAACAGGAAACAAGCTTGCTGCTTCGCTGACGAGTGGCGGACGGGTG  81

Query  66   AGTAATGTCTGGGAAACTGCCTGATGGAGGGGGATAACTACTGGAAACGGTAGCTAATAC  125
            ||||||||||||||||||||||||||||||||||||||||||||||||||||||||||||
Sbjct  82   AGTAATGTCTGGGAAACTGCCTGATGGAGGGGGATAACTACTGGAAACGGTAGCTAATAC  141

Query  126  CGCATAACGTCGCAAGACCAAAGAGGGGGACCTTCGGGCCTCTTGCCATCGGATGTGCCC  185
            ||||||||||||||||||||||||||||||||||||||||||||||||||||||||||||
Sbjct  142  CGCATAACGTCGCAAGACCAAAGAGGGGGACCTTCGGGCCTCTTGCCATCGGATGTGCCC  201

Query  186  AGATGGGATTAGCTAGTAGGTGGGGTAACGGCTCACCTAGGCGACGATCCCTAGCTGGTC  245
            ||||||||||||||||||||||||||||||||||||||||||||||||||||||||||||
Sbjct  202  AGATGGGATTAGCTAGTAGGTGGGGTAACGGCTCACCTAGGCGACGATCCCTAGCTGGTC  261

Query  246  TGAGAGGATGACCAGCCACACTGGAACTGAGACACGGTCCAGACTCCTACGGGAGGCAGC  305
            ||||||||||||||||||||||||||||||||||||||||||||||||||||||||||||
Sbjct  262  TGAGAGGATGACCAGCCACACTGGAACTGAGACACGGTCCAGACTCCTACGGGAGGCAGC  321

Query  306  AGTGGGGAATATTGCACAATGGGCGCAAGCCTGATGCAGCCATGCCGCGTGTATGAAGAA  365
            ||||||||||||||||||||||||||||||||||||||||||||||||||||||||||||
Sbjct  322  AGTGGGGAATATTGCACAATGGGCGCAAGCCTGATGCAGCCATGCCGCGTGTATGAAGAA  381

Query  366  GGCCTTCGGGTTGTAAAGTACTTTCAGCGGGGAGGAAGGGAGTAAAGTTAATACCTTTGC  425
            ||||||||||||||||||||||||||||||||||||||||||||||||||||||||||||
Sbjct  382  GGCCTTCGGGTTGTAAAGTACTTTCAGCGGGGAGGAAGGGAGTAAAGTTAATACCTTTGC  441

Query  426  TCATTGACGTTACCCGCAGAAGAAGCACCGGCTAACTCCGTGCCAGCAGCCGCGGTAATA  485
            ||||||||||||||||||||||||||||||||||||||||||||||||||||||||||||
Sbjct  442  TCATTGACGTTACCCGCAGAAGAAGCACCGGCTAACTCCGTGCCAGCAGCCGCGGTAATA  501

Query  486  CGGAGGGTGCAAGCGTTAATCGGAATTACTGGGCGTAAAGCGCACGCAGGCGGTTTGTTA  545
            ||||||||||||||||||||||||||||||||||||||||||||||||||||||||||||
Sbjct  502  CGGAGGGTGCAAGCGTTAATCGGAATTACTGGGCGTAAAGCGCACGCAGGCGGTTTGTTA  561

Query  546  AGTCAGATGTGAAATCCCCGGGCTCAACCTGGGAACTGCATCTGATACTGGCAAGCTTGA  605
            ||||||||||||||||||||||||||||||||||||||||||||||||||||||||||||
Sbjct  562  AGTCAGATGTGAAATCCCCGGGCTCAACCTGGGAACTGCATCTGATACTGGCAAGCTTGA  621

Query  606  GTCTCGTAGAGGGGGGTAGAATTCCAGGTGTAGCGGTGAAATGCGTAGAGATCTGGAGGA  665
            ||||||||||||||||||||||||||||||||||||||||||||||||||||||||||||
Sbjct  622  GTCTCGTAGAGGGGGGTAGAATTCCAGGTGTAGCGGTGAAATGCGTAGAGATCTGGAGGA  681

Query  666  ATACCGGTGGCGAAGGCGGCCCCCTGGACGAAGACTGACGCTCAGGTGCGAAAGCGTGGG  725
            ||||||||||||||||||||||||||||||||||||||||||||||||||||||||||||
Sbjct  682  ATACCGGTGGCGAAGGCGGCCCCCTGGACGAAGACTGACGCTCAGGTGCGAAAGCGTGGG  741

Query  726  GAGCAAACAGGATTAGATACCCTGGTAGTCCACGCCGTAAACGATGTCGACTTGGAGGTT  785
            ||||||||||||||||||||||||||||||||||||||||||||||||||||||||||||
Sbjct  742  GAGCAAACAGGATTAGATACCCTGGTAGTCCACGCCGTAAACGATGTCGACTTGGAGGTT  801

Query  786  GTGCCCTTGAGGCGTGGCTTCCGGAGCTAACGCGTTAAGTCGACCGCCTGGGGAGTACGG  845
            ||||||||||||||||||||||||||||||||||||||||||||||||||||||||||||
Sbjct  802  GTGCCCTTGAGGCGTGGCTTCCGGAGCTAACGCGTTAAGTCGACCGCCTGGGGAGTACGG  861

Query  846  CCGCAAGGTTAAAACTCAAATGAATTGACGGGGGCCCGCACAAGCGGTGGAGCATGTGGT  905
            ||||||||||||||||||||||||||||||||||||||||||||||||||||||||||||
Sbjct  862  CCGCAAGGTTAAAACTCAAATGAATTGACGGGGGCCCGCACAAGCGGTGGAGCATGTGGT  921

Query  906  TTAATTCGATGCAACGCGAAGAACCTTACCTGGTCTTGACATCCACGGAAGTTTTC  961
            ||||||||||||||||||||||||||||||||||||||||||||||||||||||||
Sbjct  922  TTAATTCGATGCAACGCGAAGAACCTTACCTGGTCTTGACATCCACGGAAGTTTTC  977
```

Download

FASTA (complete sequence)

FASTA (aligned sequences)

GenBank (complete sequence)

Continue
Cancel

GenBankGraphics

Next
Previous
Descriptions

Bacterium EM-2014-187 genomic DNA containing 16S-23S intergenic spacer region, isolate 187

Sequence ID: HG977395.1Length: 1403Number of Matches: 1

Related Information

Range 1: 449 to 1403GenBankGraphics

Next Match
Previous Match
First Match

Alignment statistics for match #1

| Score | Expect | Identities | Gaps | Strand | Frame |
| --- | --- | --- | --- | --- | --- |
| 1742 bits(943) | 0.0() | 949/955(99%) | 0/955(0%) | Plus/Minus |  |

Features:

```
Query  7     GCAGTCGNACGGTAACAGGAANNAAGCTTGCTNNTTNGCTGACGAGTGGCGGACGGGTGA  66
             ||||||| |||||||||||||  |||||||||  || |||||||||||||||||||||||
Sbjct  1403  GCAGTCGAACGGTAACAGGAAACAAGCTTGCTGCTTCGCTGACGAGTGGCGGACGGGTGA  1344

Query  67    GTAATGTCTGGGAAACTGCCTGATGGAGGGGGATAACTACTGGAAACGGTAGCTAATACC  126
             ||||||||||||||||||||||||||||||||||||||||||||||||||||||||||||
Sbjct  1343  GTAATGTCTGGGAAACTGCCTGATGGAGGGGGATAACTACTGGAAACGGTAGCTAATACC  1284

Query  127   GCATAACGTCGCAAGACCAAAGAGGGGGACCTTCGGGCCTCTTGCCATCGGATGTGCCCA  186
             ||||||||||||||||||||||||||||||||||||||||||||||||||||||||||||
Sbjct  1283  GCATAACGTCGCAAGACCAAAGAGGGGGACCTTCGGGCCTCTTGCCATCGGATGTGCCCA  1224

Query  187   GATGGGATTAGCTAGTAGGTGGGGTAACGGCTCACCTAGGCGACGATCCCTAGCTGGTCT  246
             ||||||||||||||||||||||||||||||||||||||||||||||||||||||||||||
Sbjct  1223  GATGGGATTAGCTAGTAGGTGGGGTAACGGCTCACCTAGGCGACGATCCCTAGCTGGTCT  1164

Query  247   GAGAGGATGACCAGCCACACTGGAACTGAGACACGGTCCAGACTCCTACGGGAGGCAGCA  306
             ||||||||||||||||||||||||||||||||||||||||||||||||||||||||||||
Sbjct  1163  GAGAGGATGACCAGCCACACTGGAACTGAGACACGGTCCAGACTCCTACGGGAGGCAGCA  1104

Query  307   GTGGGGAATATTGCACAATGGGCGCAAGCCTGATGCAGCCATGCCGCGTGTATGAAGAAG  366
             ||||||||||||||||||||||||||||||||||||||||||||||||||||||||||||
Sbjct  1103  GTGGGGAATATTGCACAATGGGCGCAAGCCTGATGCAGCCATGCCGCGTGTATGAAGAAG  1044

Query  367   GCCTTCGGGTTGTAAAGTACTTTCAGCGGGGAGGAAGGGAGTAAAGTTAATACCTTTGCT  426
             ||||||||||||||||||||||||||||||||||||||||||||||||||||||||||||
Sbjct  1043  GCCTTCGGGTTGTAAAGTACTTTCAGCGGGGAGGAAGGGAGTAAAGTTAATACCTTTGCT  984

Query  427   CATTGACGTTACCCGCAGAAGAAGCACCGGCTAACTCCGTGCCAGCAGCCGCGGTAATAC  486
             ||||||||||||||||||||||||||||||||||||||||||||||||||||||||||||
Sbjct  983   CATTGACGTTACCCGCAGAAGAAGCACCGGCTAACTCCGTGCCAGCAGCCGCGGTAATAC  924

Query  487   GGAGGGTGCAAGCGTTAATCGGAATTACTGGGCGTAAAGCGCACGCAGGCGGTTTGTTAA  546
             ||||||||||||||||||||||||||||||||||||||||||||||||||||||||||||
Sbjct  923   GGAGGGTGCAAGCGTTAATCGGAATTACTGGGCGTAAAGCGCACGCAGGCGGTTTGTTAA  864

Query  547   GTCAGATGTGAAATCCCCGGGCTCAACCTGGGAACTGCATCTGATACTGGCAAGCTTGAG  606
             ||||||||||||||||||||||||||||||||||||||||||||||||||||||||||||
Sbjct  863   GTCAGATGTGAAATCCCCGGGCTCAACCTGGGAACTGCATCTGATACTGGCAAGCTTGAG  804

Query  607   TCTCGTAGAGGGGGGTAGAATTCCAGGTGTAGCGGTGAAATGCGTAGAGATCTGGAGGAA  666
             ||||||||||||||||||||||||||||||||||||||||||||||||||||||||||||
Sbjct  803   TCTCGTAGAGGGGGGTAGAATTCCAGGTGTAGCGGTGAAATGCGTAGAGATCTGGAGGAA  744

Query  667   TACCGGTGGCGAAGGCGGCCCCCTGGACGAAGACTGACGCTCAGGTGCGAAAGCGTGGGG  726
             ||||||||||||||||||||||||||||||||||||||||||||||||||||||||||||
Sbjct  743   TACCGGTGGCGAAGGCGGCCCCCTGGACGAAGACTGACGCTCAGGTGCGAAAGCGTGGGG  684

Query  727   AGCAAACAGGATTAGATACCCTGGTAGTCCACGCCGTAAACGATGTCGACTTGGAGGTTG  786
             ||||||||||||||||||||||||||||||||||||||||||||||||||||||||||||
Sbjct  683   AGCAAACAGGATTAGATACCCTGGTAGTCCACGCCGTAAACGATGTCGACTTGGAGGTTG  624

Query  787   TGCCCTTGAGGCGTGGCTTCCGGAGCTAACGCGTTAAGTCGACCGCCTGGGGAGTACGGC  846
             ||||||||||||||||||||||||||||||||||||||||||||||||||||||||||||
Sbjct  623   TGCCCTTGAGGCGTGGCTTCCGGAGCTAACGCGTTAAGTCGACCGCCTGGGGAGTACGGC  564

Query  847   CGCAAGGTTAAAACTCAAATGAATTGACGGGGGCCCGCACAAGCGGTGGAGCATGTGGTT  906
             ||||||||||||||||||||||||||||||||||||||||||||||||||||||||||||
Sbjct  563   CGCAAGGTTAAAACTCAAATGAATTGACGGGGGCCCGCACAAGCGGTGGAGCATGTGGTT  504

Query  907   TAATTCGATGCAACGCGAAGAACCTTACCTGGTCTTGACATCCACGGAAGTTTTC  961
             |||||||||||||||||||||||||||||||||||||||||||||||||||||||
Sbjct  503   TAATTCGATGCAACGCGAAGAACCTTACCTGGTCTTGACATCCACGGAAGTTTTC  449
```

Download

FASTA (complete sequence)

FASTA (aligned sequences)

GenBank (complete sequence)

Continue
Cancel

GenBankGraphics

Next
Previous
Descriptions

Escherichia coli strain IRZHN10 16S ribosomal RNA gene, partial sequence

Sequence ID: MG554686.1Length: 1409Number of Matches: 1

Related Information

Range 1: 3 to 957GenBankGraphics

Next Match
Previous Match
First Match

Alignment statistics for match #1

| Score | Expect | Identities | Gaps | Strand | Frame |
| --- | --- | --- | --- | --- | --- |
| 1740 bits(942) | 0.0() | 950/956(99%) | 1/956(0%) | Plus/Plus |  |

Features:

```
Query  6    TGCAGTCGNACGGTAACAGGAANNAAGCTTGCTNNTTNGCTGACGAGTGGCGGACGGGTG  65
            |||||||| |||||||||||||  |||||||||  || ||||||||||||||||||||||
Sbjct  3    TGCAGTCGAACGGTAACAGGAA-GAAGCTTGCTTCTTTGCTGACGAGTGGCGGACGGGTG  61

Query  66   AGTAATGTCTGGGAAACTGCCTGATGGAGGGGGATAACTACTGGAAACGGTAGCTAATAC  125
            ||||||||||||||||||||||||||||||||||||||||||||||||||||||||||||
Sbjct  62   AGTAATGTCTGGGAAACTGCCTGATGGAGGGGGATAACTACTGGAAACGGTAGCTAATAC  121

Query  126  CGCATAACGTCGCAAGACCAAAGAGGGGGACCTTCGGGCCTCTTGCCATCGGATGTGCCC  185
            ||||||||||||||||||||||||||||||||||||||||||||||||||||||||||||
Sbjct  122  CGCATAACGTCGCAAGACCAAAGAGGGGGACCTTCGGGCCTCTTGCCATCGGATGTGCCC  181

Query  186  AGATGGGATTAGCTAGTAGGTGGGGTAACGGCTCACCTAGGCGACGATCCCTAGCTGGTC  245
            ||||||||||||||||||||||||||||||||||||||||||||||||||||||||||||
Sbjct  182  AGATGGGATTAGCTAGTAGGTGGGGTAACGGCTCACCTAGGCGACGATCCCTAGCTGGTC  241

Query  246  TGAGAGGATGACCAGCCACACTGGAACTGAGACACGGTCCAGACTCCTACGGGAGGCAGC  305
            ||||||||||||||||||||||||||||||||||||||||||||||||||||||||||||
Sbjct  242  TGAGAGGATGACCAGCCACACTGGAACTGAGACACGGTCCAGACTCCTACGGGAGGCAGC  301

Query  306  AGTGGGGAATATTGCACAATGGGCGCAAGCCTGATGCAGCCATGCCGCGTGTATGAAGAA  365
            ||||||||||||||||||||||||||||||||||||||||||||||||||||||||||||
Sbjct  302  AGTGGGGAATATTGCACAATGGGCGCAAGCCTGATGCAGCCATGCCGCGTGTATGAAGAA  361

Query  366  GGCCTTCGGGTTGTAAAGTACTTTCAGCGGGGAGGAAGGGAGTAAAGTTAATACCTTTGC  425
            ||||||||||||||||||||||||||||||||||||||||||||||||||||||||||||
Sbjct  362  GGCCTTCGGGTTGTAAAGTACTTTCAGCGGGGAGGAAGGGAGTAAAGTTAATACCTTTGC  421

Query  426  TCATTGACGTTACCCGCAGAAGAAGCACCGGCTAACTCCGTGCCAGCAGCCGCGGTAATA  485
            ||||||||||||||||||||||||||||||||||||||||||||||||||||||||||||
Sbjct  422  TCATTGACGTTACCCGCAGAAGAAGCACCGGCTAACTCCGTGCCAGCAGCCGCGGTAATA  481

Query  486  CGGAGGGTGCAAGCGTTAATCGGAATTACTGGGCGTAAAGCGCACGCAGGCGGTTTGTTA  545
            ||||||||||||||||||||||||||||||||||||||||||||||||||||||||||||
Sbjct  482  CGGAGGGTGCAAGCGTTAATCGGAATTACTGGGCGTAAAGCGCACGCAGGCGGTTTGTTA  541

Query  546  AGTCAGATGTGAAATCCCCGGGCTCAACCTGGGAACTGCATCTGATACTGGCAAGCTTGA  605
            ||||||||||||||||||||||||||||||||||||||||||||||||||||||||||||
Sbjct  542  AGTCAGATGTGAAATCCCCGGGCTCAACCTGGGAACTGCATCTGATACTGGCAAGCTTGA  601

Query  606  GTCTCGTAGAGGGGGGTAGAATTCCAGGTGTAGCGGTGAAATGCGTAGAGATCTGGAGGA  665
            ||||||||||||||||||||||||||||||||||||||||||||||||||||||||||||
Sbjct  602  GTCTCGTAGAGGGGGGTAGAATTCCAGGTGTAGCGGTGAAATGCGTAGAGATCTGGAGGA  661

Query  666  ATACCGGTGGCGAAGGCGGCCCCCTGGACGAAGACTGACGCTCAGGTGCGAAAGCGTGGG  725
            ||||||||||||||||||||||||||||||||||||||||||||||||||||||||||||
Sbjct  662  ATACCGGTGGCGAAGGCGGCCCCCTGGACGAAGACTGACGCTCAGGTGCGAAAGCGTGGG  721

Query  726  GAGCAAACAGGATTAGATACCCTGGTAGTCCACGCCGTAAACGATGTCGACTTGGAGGTT  785
            ||||||||||||||||||||||||||||||||||||||||||||||||||||||||||||
Sbjct  722  GAGCAAACAGGATTAGATACCCTGGTAGTCCACGCCGTAAACGATGTCGACTTGGAGGTT  781

Query  786  GTGCCCTTGAGGCGTGGCTTCCGGAGCTAACGCGTTAAGTCGACCGCCTGGGGAGTACGG  845
            ||||||||||||||||||||||||||||||||||||||||||||||||||||||||||||
Sbjct  782  GTGCCCTTGAGGCGTGGCTTCCGGAGCTAACGCGTTAAGTCGACCGCCTGGGGAGTACGG  841

Query  846  CCGCAAGGTTAAAACTCAAATGAATTGACGGGGGCCCGCACAAGCGGTGGAGCATGTGGT  905
            ||||||||||||||||||||||||||||||||||||||||||||||||||||||||||||
Sbjct  842  CCGCAAGGTTAAAACTCAAATGAATTGACGGGGGCCCGCACAAGCGGTGGAGCATGTGGT  901

Query  906  TTAATTCGATGCAACGCGAAGAACCTTACCTGGTCTTGACATCCACGGAAGTTTTC  961
            ||||||||||||||||||||||||||||||||||||||||||||||||||||||||
Sbjct  902  TTAATTCGATGCAACGCGAAGAACCTTACCTGGTCTTGACATCCACGGAAGTTTTC  957
```


BLAST is a registered trademark of the National Library of Medicine

Support center
Mailing list


YouTube

- National Library Of Medicine
- National Institutes Of Health
- U.S. Department of Health & Human Services
- USA.gov

### NCBI


National Center for Biotechnology Information,
 U.S. National Library of Medicine

8600 Rockville Pike,
Bethesda
 MD,
20894
USA

Policies and Guidelines
|
Contact


PreferencesTurn off

External link. Please review our privacy policy.
