# Supplementary material for: Isothermal microcalorimetry for thermal viable count of microorganisms in pure cultures and stabilized formulations
Source: BMC Microbiol. 2019 Mar 21;19:65. doi: 10.1186/s12866-019-1432-8 (PMC6429831; doi:10.1186/s12866-019-1432-8)
Supplement: Supplementary file 3 — 16S rRNA gene sequences’ BLASTN hits in zipped HTML format. (ZIP 15810 kb) [file 12866_2019_1432_MOESM3_ESM.zip › Best blastn hits/NCBI Blast_DH5a negative R -- 13..1076 of sequence.html]

NCBI Blast:DH5a negative R -- 13..1076 of sequence


- NCBI Home
- Sign in to NCBI
- Skip to Main Content
- Skip to Navigation
- About NCBI Accesskeys

U.S. National Library of Medicine

NCBI
National Center for Biotechnology Information

- My NCBI
- Sign in to NCBI
- Register
- Sign Out

BLAST ^®^ » blastn suite » RID-A4XUDGFP015


- Home
- Recent Results
- Saved Strategies
- Help

BLAST Results


Edit and Resubmit
Save Search Strategies
[Sign in above to save your search strategy]

Formatting options 


Download


How to read this page
Blast report description
Questions/comments


|  |  |
| --- | --- |
| Formatting options | |
| Show | Alignment as  HTML Plain text   Old View Reset form to defaults [?]  These options control formatting of alignments in results pages. The default is HTML, but other formats (including plain text) are available. PSSM and PssmWithParameters are representations of Position Specific Scoring Matrices and are only available for PSI-BLAST. The Advanced view option allows the database descriptions to be sorted by various indices in a table. |
| Alignment View | Pairwise Pairwise with dots for identities Query-anchored with dots for identities Query-anchored with letters for identities Flat query-anchored with dots for identities Flat query-anchored with letters for identities [?]  Choose how to view alignments. The default "pairwise" view shows how each subject sequence aligns individually to the query sequence. The "query-anchored" view shows how all subject sequences align to the query sequence. For each view type, you can choose to show "identities" (matching residues) as letters or dots. more... |
| Display | Graphical Overview   Linkout   Sequence Retrieval  NCBI-gi   CDS feature [?]  - Graphical Overview: Graphical Overview: Show graph of similar sequence regions aligned to query.   more... - NCBI-gi: Show NCBI gi identifiers. - CDS feature: Show annotated coding region and translation.   more... |
| Masking | Character:   X for protein, n for nucleotide Lower Case  Color:  Black Grey Red [?]  - Masking Character: Display masked (filtered) sequence regions as lower-case or as specific letters (N for nucleotide, P for protein). - Masking Color: Display masked sequence regions in the given color. |
| Limit results | Descriptions:  10 50 100 Graphical overview:  0 10 50 100  Alignments:  0 10 50 100 Line length:  60 90 120 150 [?]  - Descriptions: Show short descriptions for up to the given number of sequences. - Alignments: Show alignments for up to the given number of sequences, in order of statistical significance. - Line lenghth: Number of letters to show on one line in an alignment. |
|  | Organism Type common name, binomial, taxid, or group name. Only 20 top taxa will be shown.     Exclude   [?]  Show only sequences from the given organism. |
|  | Entrez query:  [?]  Show only those sequences that match the given Entrez query. more... |
|  | Expect Min:  Expect Max:  [?]  Show only sequences with expect values in the given range. more... |
|  | Percent Identity Min:  Percent Identity Max:  [?]  Show only sequences with percent identity values in the given range. |
| Format for | PSI-BLAST with inclusion threshold:  [?]  - Format for PSI-BLAST: The Position-Specific Iterated BLAST (PSI-BLAST) program performs iterative searches with a protein query,   in which sequences found in one round of search are used to build a custom score model for the next round.   more... - Inclusion Threshold: This sets the statistical significance threshold for including a sequence in the model used   by PSI-BLAST to create the PSSM on the next iteration. |

|  |  |  |  |  |  |
| --- | --- | --- | --- | --- | --- |
| Download | | | | | |
| Alignment  Text XML ASN.1 JSON Seq-align Hit Table(text) Hit Table(csv) Multiple-file XML2 Single-file XML2 Multiple-file JSON Single-file JSON SAM | Search Strategies  ASN.1 | PSSM to restart search  PSSM | [?] |  |  |

The Download link provides BLAST output that may be used as input to another program.
This includes parseable formats such as the tabular report or XML as well as the Search Strategy files read by the BLAST+ applications.
More details on the parseable (XML, tabular, and ASN.1) reports can be found at
https://www.ncbi.nlm.nih.gov/books/NBK153387/  
  

The following formats are offered under the Alignment section:  
1). "Text". Non-HTML standard BLAST report.  
2). "XML". XML report based upon the DTD at https://www.ncbi.nlm.nih.gov/data\_specs/dtd/NCBI\_BlastOutput.dtd  
3). "ASN.1". Alignment written out in Abstract Syntax Notation 1.  
4). "JSON Seq-align". Alignment written out in JSON.  
4). "Hit Table(text)". The tabular report as text.  
5). "Hit Table(csv)". The tabular report ready for import into spread-sheet programs like Excel.  
6). "XML2". New XML format described at ftp://ftp.ncbi.nlm.nih.gov/blast/documents/NEWXML/xml2.pdf.  
7). "JSON". New JSON format described at ftp://ftp.ncbi.nlm.nih.gov/blast/documents/NEWXML/xml2.pdf.  
8). "SAM". Sequence Alignment Map format.

XML2 and JSON can be downloaded either as one file per query (multiple-file) or one file for all queries (single-file). These formats are listed as Multiple-file XML2 (and JSON) or Single-file XML (and JSON).

The following report is offered under the Search Strategy section:  
1). "ASN.1" Search Strategy. A record of the parameters, query, and database used in the search. This file can be used to start a stand-alone BLAST search, see
https://www.ncbi.nlm.nih.gov/books/NBK1763/#CmdLineAppsManual.I455\_BLAST\_search\_stra


# Job title: DH5a negative R -- 13..1076 of sequence

Results for:

lcl|Query\_229421 DH5a negative R -- 13..1076 of sequence(1064bp)
[?]

Your BLAST job specified more than one input sequence.
This box lets you choose which input sequence to show BLAST results for.

RID
:   A4XUDGFP015 (Expires on 03-10 19:00 pm)

Query ID
:   lcl|Query\_229421
:   lcl|Query\_229421

Description
:   DH5a negative R -- 13..1076 of sequence

Molecule type
:   nucleic acid

Query Length
:   1064

Database Name
:   nr

Description
:   Nucleotide collection (nt) See details

Program
:   BLASTN 2.8.0+ Citation

  

Reference 

Zheng Zhang, Scott Schwartz, Lukas Wagner, and Webb Miller (2000), "A greedy algorithm for aligning DNA sequences", J Comput Biol 2000; 7(1-2):203-14.

Reference - database indexing

Aleksandr Morgulis, George Coulouris, Yan Raytselis, Thomas L. Madden, Richa Agarwala, Alejandro A. Schäffer (2008), "Database Indexing for Production MegaBLAST Searches", Bioinformatics 24:1757-1764.

Other reports:
Search Summary

[Taxonomy reports]
[Distance tree of results]
[MSA viewer]

Search Parameters

| Search parameter name | Search parameter value |
| --- | --- |
| Program | blastn |
| Word size | 28 |
| Expect value | 10 |
| Hitlist size | 100 |
| Match/Mismatch scores | 1,-2 |
| Gapcosts | 0,2.5 |
| Low Complexity Filter | Yes |
| Filter string | L;m; |
| Genetic Code | 1 |

Database

| Database parameter name | Database parameter value |
| --- | --- |
| Posted date | Mar 3, 2018 9:32 PM |
| Number of letters | 171,598,911,005 |
| Number of sequences | 46,870,701 |
| Entrez query | Includes:  Excludes:  None |

Karlin-Altschul statistics

| Params | Ungapped | Gapped |
| --- | --- | --- |
| Lambda | 1.33271 | 1.28 |
| K | 0.620991 | 0.46 |
| H | 1.12409 | 0.85 |

Results Statistics

| Results Statistics parameter name | Results Statistics parameter value |
| --- | --- |
| Length adjustment | 35 |
| Effective length of query | 1029 |
| Effective length of database | 169958436470 |
| Effective search space | 174887231127630 |
| Effective search space used | 174887231127630 |


## Graphic Summary

### Distribution of the top 200 Blast Hits on 100 subject sequences [?]

The graphic is an overview of the database sequences aligned to the query sequence. These are represented horizontal bars colored coded by score and showing the extent
of the alignment on the query sequence. Separate aligned regions on the same database sequence are connected by a thin grey line.
Mousing over an alignment shows the database sequence title. Clicking an alignment displays a box with more details about the alignment and
link to the sequence alignment itself in the Alignments section of the report.

Mouse over to see the title, click to show alignments

Color key for alignment scores

<40

40-50

50-80

80-200

>=200

Query

1

200

400

600

800

1000

Escherichia coli O157 strain FDAARGOS\_293 chromosome, c..

Score:1929 Evalue:0

Accession:CP022050.2

Alignment

Escherichia coli str. K-12 substr. MG1655 strain K-12 c..

Score:1929 Evalue:0

Accession:CP027060.1

Alignment

Shigella flexneri 2a strain ATCC 29903 chromosome, comp..

Score:1929 Evalue:0

Accession:CP026788.1

Alignment

Shigella boydii strain ATCC BAA-1247 chromosome, comple..

Score:1929 Evalue:0

Accession:CP026795.1

Alignment

Shigella dysenteriae strain CFSAN010956 chromosome, com..

Score:1929 Evalue:0

Accession:CP026827.1

Alignment

Shigella dysenteriae strain CFSAN010954 chromosome, com..

Score:1929 Evalue:0

Accession:CP026824.1

Alignment

Shigella dysenteriae strain E670/74 chromosome, complet..

Score:1929 Evalue:0

Accession:CP027027.1

Alignment

Shigella boydii strain NCTC 9734 chromosome

Score:1929 Evalue:0

Accession:CP026875.1

Alignment

Shigella boydii strain NCTC 9733 chromosome, complete g..

Score:1929 Evalue:0

Accession:CP026845.1

Alignment

Shigella flexneri strain NCTC 9728 chromosome, complete..

Score:1929 Evalue:0

Accession:CP026799.1

Alignment

Shigella boydii strain NCTC 9353 chromosome, complete g..

Score:1929 Evalue:0

Accession:CP026797.1

Alignment

Shigella flexneri strain 61-4982 chromosome, complete g..

Score:1929 Evalue:0

Accession:CP026792.1

Alignment

Shigella flexneri strain 73-5612 chromosome

Score:1929 Evalue:0

Accession:CP026871.1

Alignment

Shigella dysenteriae strain ATCC 9753 chromosome, compl..

Score:1929 Evalue:0

Accession:CP026840.1

Alignment

Shigella dysenteriae strain ATCC 9752 chromosome, compl..

Score:1929 Evalue:0

Accession:CP026839.1

Alignment

Shigella boydii strain ATCC 49812 chromosome, complete ..

Score:1929 Evalue:0

Accession:CP026836.1

Alignment

Shigella dysenteriae strain ATCC 49347 chromosome, comp..

Score:1929 Evalue:0

Accession:CP026834.1

Alignment

Shigella boydii strain ATCC 35964 chromosome, complete ..

Score:1929 Evalue:0

Accession:CP026877.1

Alignment

Shigella dysenteriae strain ATCC 12039 chromosome, comp..

Score:1929 Evalue:0

Accession:CP026831.1

Alignment

Shigella boydii strain ATCC 12027 chromosome

Score:1929 Evalue:0

Accession:CP026866.1

Alignment

Shigella flexneri strain 98-3193 chromosome, complete g..

Score:1929 Evalue:0

Accession:CP026776.1

Alignment

Shigella dysenteriae strain 96-3162 chromosome, complet..

Score:1929 Evalue:0

Accession:CP026823.1

Alignment

Shigella dysenteriae strain 96-265 chromosome, complete..

Score:1929 Evalue:0

Accession:CP026819.1

Alignment

Shigella flexneri strain 95-3008 chromosome

Score:1929 Evalue:0

Accession:CP026772.1

Alignment

Shigella flexneri Y strain 93-3063 chromosome, complete..

Score:1929 Evalue:0

Accession:CP026768.1

Alignment

Shigella boydii strain 83-578 chromosome, complete geno..

Score:1929 Evalue:0

Accession:CP026814.1

Alignment

Shigella dysenteriae strain 204/96 chromosome, complete..

Score:1929 Evalue:0

Accession:CP026807.1

Alignment

Shigella dysenteriae strain 2017C-4522 chromosome, comp..

Score:1929 Evalue:0

Accession:CP026805.1

Alignment

Shigella flexneri strain 04-3145 chromosome, complete g..

Score:1929 Evalue:0

Accession:CP026765.1

Alignment

Shigella boydii strain NCTC 9850 chromosome, complete g..

Score:1929 Evalue:0

Accession:CP026762.1

Alignment

Shigella flexneri strain 89-141 chromosome, complete ge..

Score:1929 Evalue:0

Accession:CP026803.1

Alignment

Escherichia coli strain FDAARGOS\_144 chromosome, comple..

Score:1929 Evalue:0

Accession:CP014111.1

Alignment

Escherichia coli strain DTU-1 chromosome, complete geno..

Score:1929 Evalue:0

Accession:CP026612.1

Alignment

Escherichia coli strain UTI05 16S ribosomal RNA gene, p..

Score:1929 Evalue:0

Accession:MG913260.1

Alignment

Escherichia coli strain WCHEC005237 chromosome, complet..

Score:1929 Evalue:0

Accession:CP026580.1

Alignment

Escherichia coli strain FHI\_NMBU\_03 chromosome, complet..

Score:1929 Evalue:0

Accession:CP019455.1

Alignment

Escherichia sp. strain CG1008 16S ribosomal RNA gene, p..

Score:1929 Evalue:0

Accession:MG871203.1

Alignment

Escherichia coli strain KBN10P04869 chromosome, complet..

Score:1929 Evalue:0

Accession:CP026473.1

Alignment

Escherichia coli strain HS13-1 chromosome, complete gen..

Score:1929 Evalue:0

Accession:CP026491.1

Alignment

Escherichia coli strain ECONIH4 chromosome, complete ge..

Score:1929 Evalue:0

Accession:CP026399.1

Alignment

Escherichia coli strain FORC\_042 chromosome, complete g..

Score:1929 Evalue:0

Accession:CP025318.1

Alignment

Escherichia coli strain 2A chromosome, complete genome

Score:1929 Evalue:0

Accession:CP026358.1

Alignment

Escherichia coli strain 1A chromosome, complete genome

Score:1929 Evalue:0

Accession:CP026361.1

Alignment

Escherichia coli strain 2F\_0 chromosome, complete genom..

Score:1929 Evalue:0

Accession:CP026357.1

Alignment

Escherichia coli strain 4FA chromosome, complete genome

Score:1929 Evalue:0

Accession:CP026352.1

Alignment

Escherichia coli strain 4A chromosome, complete genome

Score:1929 Evalue:0

Accession:CP026353.1

Alignment

Escherichia coli strain 5A chromosome, complete genome

Score:1929 Evalue:0

Accession:CP026351.1

Alignment

Escherichia coli strain 1FA chromosome, complete genome

Score:1929 Evalue:0

Accession:CP026360.1

Alignment

Escherichia coli strain 3FA chromosome, complete genome

Score:1929 Evalue:0

Accession:CP026354.1

Alignment

Escherichia coli strain 8A chromosome, complete genome

Score:1929 Evalue:0

Accession:CP026345.1

Alignment

Escherichia coli strain 5FA chromosome, complete genome

Score:1929 Evalue:0

Accession:CP026350.1

Alignment

Escherichia coli strain 7A chromosome, complete genome

Score:1929 Evalue:0

Accession:CP026347.1

Alignment

Escherichia coli strain 7FA chromosome, complete genome

Score:1929 Evalue:0

Accession:CP026346.1

Alignment

Escherichia coli strain 9FA chromosome, complete genome

Score:1929 Evalue:0

Accession:CP026342.1

Alignment

Escherichia coli strain 9A chromosome, complete genome

Score:1929 Evalue:0

Accession:CP026343.1

Alignment

Escherichia coli strain 6FA chromosome, complete genome

Score:1929 Evalue:0

Accession:CP026348.1

Alignment

## Descriptions

, AlignDB=on Reading indexes 1-2, displaying indexes 1-2


Load next setPrevious Match

Sequences producing significant alignments:

Show all columns  of the table presenting sequences producing significant alignments 

Select:AllNone
Selected:0

Alignments
Download

FASTA (complete sequence)

FASTA (aligned sequences)

GenBank (complete sequence)

Hit Table (text)

Hit Table (CSV)

Text

XML

ASN.1

Continue
Cancel

GenBank 
Graphics
Distance tree of results
Multiple alignment
Show/hide columns of the table presenting sequences producing significant alignments 

Available columns

Description  
Max Score  
Total Score  
Coverage  
E-value  
IdentN  
Accession  
Restore Defaults
Ok
Cancel

Sequences producing significant alignments:

| Select for downloading or viewing reports | Description | Max score | Total score | Query cover | E value | Ident | Accession |
| --- | --- | --- | --- | --- | --- | --- | --- |
| 1Select seq CP022050.2 | Escherichia coli O157 strain FDAARGOS\_293 chromosome, complete genome | 1929 | 13248 | 98% | 0.0 | 99% | CP022050.2 |
| 2Select seq CP027060.1 | Escherichia coli str. K-12 substr. MG1655 strain K-12 chromosome | 1929 | 13458 | 98% | 0.0 | 99% | CP027060.1 |
| 3Select seq CP026788.1 | Shigella flexneri 2a strain ATCC 29903 chromosome, complete genome | 1929 | 13425 | 98% | 0.0 | 99% | CP026788.1 |
| 4Select seq CP026795.1 | Shigella boydii strain ATCC BAA-1247 chromosome, complete genome | 1929 | 13456 | 98% | 0.0 | 99% | CP026795.1 |
| 5Select seq CP026827.1 | Shigella dysenteriae strain CFSAN010956 chromosome, complete genome | 1929 | 13475 | 98% | 0.0 | 99% | CP026827.1 |
| 6Select seq CP026824.1 | Shigella dysenteriae strain CFSAN010954 chromosome, complete genome | 1929 | 13421 | 98% | 0.0 | 99% | CP026824.1 |
| 7Select seq CP027027.1 | Shigella dysenteriae strain E670/74 chromosome, complete genome | 1929 | 13495 | 98% | 0.0 | 99% | CP027027.1 |
| 8Select seq CP026875.1 | Shigella boydii strain NCTC 9734 chromosome | 1929 | 13464 | 98% | 0.0 | 99% | CP026875.1 |
| 9Select seq CP026845.1 | Shigella boydii strain NCTC 9733 chromosome, complete genome | 1929 | 13444 | 98% | 0.0 | 99% | CP026845.1 |
| 10Select seq CP026799.1 | Shigella flexneri strain NCTC 9728 chromosome, complete genome | 1929 | 13431 | 98% | 0.0 | 99% | CP026799.1 |
| 11Select seq CP026797.1 | Shigella boydii strain NCTC 9353 chromosome, complete genome | 1929 | 13440 | 98% | 0.0 | 99% | CP026797.1 |
| 12Select seq CP026792.1 | Shigella flexneri strain 61-4982 chromosome, complete genome | 1929 | 13436 | 98% | 0.0 | 99% | CP026792.1 |
| 13Select seq CP026871.1 | Shigella flexneri strain 73-5612 chromosome | 1929 | 13420 | 98% | 0.0 | 99% | CP026871.1 |
| 14Select seq CP026840.1 | Shigella dysenteriae strain ATCC 9753 chromosome, complete genome | 1929 | 13475 | 98% | 0.0 | 99% | CP026840.1 |
| 15Select seq CP026839.1 | Shigella dysenteriae strain ATCC 9752 chromosome, complete genome | 1929 | 13432 | 98% | 0.0 | 99% | CP026839.1 |
| 16Select seq CP026836.1 | Shigella boydii strain ATCC 49812 chromosome, complete genome | 1929 | 13503 | 98% | 0.0 | 99% | CP026836.1 |
| 17Select seq CP026834.1 | Shigella dysenteriae strain ATCC 49347 chromosome, complete genome | 1929 | 13436 | 98% | 0.0 | 99% | CP026834.1 |
| 18Select seq CP026877.1 | Shigella boydii strain ATCC 35964 chromosome, complete genome | 1929 | 13464 | 98% | 0.0 | 99% | CP026877.1 |
| 19Select seq CP026831.1 | Shigella dysenteriae strain ATCC 12039 chromosome, complete genome | 1929 | 13503 | 98% | 0.0 | 99% | CP026831.1 |
| 20Select seq CP026866.1 | Shigella boydii strain ATCC 12027 chromosome | 1929 | 13436 | 98% | 0.0 | 99% | CP026866.1 |
| 21Select seq CP026776.1 | Shigella flexneri strain 98-3193 chromosome, complete genome | 1929 | 13412 | 98% | 0.0 | 99% | CP026776.1 |
| 22Select seq CP026823.1 | Shigella dysenteriae strain 96-3162 chromosome, complete genome | 1929 | 13383 | 98% | 0.0 | 99% | CP026823.1 |
| 23Select seq CP026819.1 | Shigella dysenteriae strain 96-265 chromosome, complete genome | 1929 | 13431 | 98% | 0.0 | 99% | CP026819.1 |
| 24Select seq CP026772.1 | Shigella flexneri strain 95-3008 chromosome | 1929 | 13431 | 98% | 0.0 | 99% | CP026772.1 |
| 25Select seq CP026768.1 | Shigella flexneri Y strain 93-3063 chromosome, complete genome | 1929 | 13431 | 98% | 0.0 | 99% | CP026768.1 |
| 26Select seq CP026814.1 | Shigella boydii strain 83-578 chromosome, complete genome | 1929 | 13442 | 98% | 0.0 | 99% | CP026814.1 |
| 27Select seq CP026807.1 | Shigella dysenteriae strain 204/96 chromosome, complete genome | 1929 | 13431 | 98% | 0.0 | 99% | CP026807.1 |
| 28Select seq CP026805.1 | Shigella dysenteriae strain 2017C-4522 chromosome, complete genome | 1929 | 13421 | 98% | 0.0 | 99% | CP026805.1 |
| 29Select seq CP026765.1 | Shigella flexneri strain 04-3145 chromosome, complete genome | 1929 | 13445 | 98% | 0.0 | 99% | CP026765.1 |
| 30Select seq CP026762.1 | Shigella boydii strain NCTC 9850 chromosome, complete genome | 1929 | 13464 | 98% | 0.0 | 99% | CP026762.1 |
| 31Select seq CP026803.1 | Shigella flexneri strain 89-141 chromosome, complete genome | 1929 | 13486 | 98% | 0.0 | 99% | CP026803.1 |
| 32Select seq CP014111.1 | Escherichia coli strain FDAARGOS\_144 chromosome, complete genome | 1929 | 13503 | 98% | 0.0 | 99% | CP014111.1 |
| 33Select seq CP026612.1 | Escherichia coli strain DTU-1 chromosome, complete genome | 1929 | 13458 | 98% | 0.0 | 99% | CP026612.1 |
| 34Select seq MG913260.1 | Escherichia coli strain UTI05 16S ribosomal RNA gene, partial sequence | 1929 | 1929 | 98% | 0.0 | 99% | MG913260.1 |
| 35Select seq CP026580.1 | Escherichia coli strain WCHEC005237 chromosome, complete genome | 1929 | 13503 | 98% | 0.0 | 99% | CP026580.1 |
| 36Select seq CP019455.1 | Escherichia coli strain FHI\_NMBU\_03 chromosome, complete genome | 1929 | 13503 | 98% | 0.0 | 99% | CP019455.1 |
| 37Select seq MG871203.1 | Escherichia sp. strain CG1008 16S ribosomal RNA gene, partial sequence | 1929 | 1929 | 98% | 0.0 | 99% | MG871203.1 |
| 38Select seq CP026473.1 | Escherichia coli strain KBN10P04869 chromosome, complete genome | 1929 | 13270 | 98% | 0.0 | 99% | CP026473.1 |
| 39Select seq CP026491.1 | Escherichia coli strain HS13-1 chromosome, complete genome | 1929 | 13503 | 98% | 0.0 | 99% | CP026491.1 |
| 40Select seq CP026399.1 | Escherichia coli strain ECONIH4 chromosome, complete genome | 1929 | 13475 | 98% | 0.0 | 99% | CP026399.1 |
| 41Select seq CP025318.1 | Escherichia coli strain FORC\_042 chromosome, complete genome | 1929 | 13464 | 98% | 0.0 | 99% | CP025318.1 |
| 42Select seq CP026358.1 | Escherichia coli strain 2A chromosome, complete genome | 1929 | 13458 | 98% | 0.0 | 99% | CP026358.1 |
| 43Select seq CP026361.1 | Escherichia coli strain 1A chromosome, complete genome | 1929 | 13458 | 98% | 0.0 | 99% | CP026361.1 |
| 44Select seq CP026357.1 | Escherichia coli strain 2F\_0 chromosome, complete genome | 1929 | 13458 | 98% | 0.0 | 99% | CP026357.1 |
| 45Select seq CP026352.1 | Escherichia coli strain 4FA chromosome, complete genome | 1929 | 13458 | 98% | 0.0 | 99% | CP026352.1 |
| 46Select seq CP026353.1 | Escherichia coli strain 4A chromosome, complete genome | 1929 | 13458 | 98% | 0.0 | 99% | CP026353.1 |
| 47Select seq CP026351.1 | Escherichia coli strain 5A chromosome, complete genome | 1929 | 13458 | 98% | 0.0 | 99% | CP026351.1 |
| 48Select seq CP026360.1 | Escherichia coli strain 1FA chromosome, complete genome | 1929 | 13458 | 98% | 0.0 | 99% | CP026360.1 |
| 49Select seq CP026354.1 | Escherichia coli strain 3FA chromosome, complete genome | 1929 | 13458 | 98% | 0.0 | 99% | CP026354.1 |
| 50Select seq CP026345.1 | Escherichia coli strain 8A chromosome, complete genome | 1929 | 13458 | 98% | 0.0 | 99% | CP026345.1 |
| 51Select seq CP026350.1 | Escherichia coli strain 5FA chromosome, complete genome | 1929 | 13458 | 98% | 0.0 | 99% | CP026350.1 |
| 52Select seq CP026347.1 | Escherichia coli strain 7A chromosome, complete genome | 1929 | 13458 | 98% | 0.0 | 99% | CP026347.1 |
| 53Select seq CP026346.1 | Escherichia coli strain 7FA chromosome, complete genome | 1929 | 13458 | 98% | 0.0 | 99% | CP026346.1 |
| 54Select seq CP026342.1 | Escherichia coli strain 9FA chromosome, complete genome | 1929 | 13458 | 98% | 0.0 | 99% | CP026342.1 |
| 55Select seq CP026343.1 | Escherichia coli strain 9A chromosome, complete genome | 1929 | 13458 | 98% | 0.0 | 99% | CP026343.1 |
| 56Select seq CP026348.1 | Escherichia coli strain 6FA chromosome, complete genome | 1929 | 13458 | 98% | 0.0 | 99% | CP026348.1 |
| 57Select seq CP026349.1 | Escherichia coli strain 6A chromosome, complete genome | 1929 | 13458 | 98% | 0.0 | 99% | CP026349.1 |
| 58Select seq CP026359.1 | Escherichia coli strain 2\_0 chromosome, complete genome | 1929 | 13458 | 98% | 0.0 | 99% | CP026359.1 |
| 59Select seq CP026344.1 | Escherichia coli strain 8FA chromosome, complete genome | 1929 | 13458 | 98% | 0.0 | 99% | CP026344.1 |
| 60Select seq CP026356.1 | Escherichia coli strain 2FA chromosome, complete genome | 1929 | 13458 | 98% | 0.0 | 99% | CP026356.1 |
| 61Select seq CP026355.1 | Escherichia coli strain 3A chromosome, complete genome | 1929 | 13458 | 98% | 0.0 | 99% | CP026355.1 |
| 62Select seq CP026098.1 | Shigella flexneri strain FDAARGOS\_74 chromosome, complete genome | 1929 | 13436 | 98% | 0.0 | 99% | CP026098.1 |
| 63Select seq CP026202.1 | Escherichia coli strain ECONIH5 chromosome, complete genome | 1929 | 13486 | 98% | 0.0 | 99% | CP026202.1 |
| 64Select seq CP026199.1 | Escherichia coli strain ECONIH6 chromosome, complete genome | 1929 | 13503 | 98% | 0.0 | 99% | CP026199.1 |
| 65Select seq MG818962.1 | Escherichia fergusonii strain FA-5 16S ribosomal RNA gene, partial sequence | 1929 | 1929 | 98% | 0.0 | 99% | MG818962.1 |
| 66Select seq CP025703.1 | Escherichia coli BH100N substr. MG2017, complete genome | 1929 | 13503 | 98% | 0.0 | 99% | CP025703.1 |
| 67Select seq CP026085.1 | Escherichia coli strain DH5alpha chromosome, complete genome | 1929 | 13348 | 98% | 0.0 | 99% | CP026085.1 |
| 68Select seq CP026027.1 | Escherichia coli strain LIM chromosome, complete genome | 1929 | 13458 | 98% | 0.0 | 99% | CP026027.1 |
| 69Select seq CP026026.1 | Escherichia coli strain CAR chromosome, complete genome | 1929 | 13458 | 98% | 0.0 | 99% | CP026026.1 |
| 70Select seq CP026028.1 | Escherichia coli strain CIT chromosome, complete genome | 1929 | 13458 | 98% | 0.0 | 99% | CP026028.1 |
| 71Select seq CP025950.1 | Escherichia coli strain SCEC020023 chromosome, complete genome | 1929 | 13503 | 98% | 0.0 | 99% | CP025950.1 |
| 72Select seq CP025753.1 | Escherichia coli strain CV839-06 chromosome, complete genome | 1929 | 13325 | 98% | 0.0 | 99% | CP025753.1 |
| 73Select seq CP025716.1 | Escherichia coli BH100L substr. MG2017, complete genome | 1929 | 13207 | 98% | 0.0 | 99% | CP025716.1 |
| 74Select seq CP025747.1 | Escherichia coli strain ML35 chromosome, complete genome | 1929 | 13436 | 98% | 0.0 | 99% | CP025747.1 |
| 75Select seq MF620019.1 | Uncultured bacterium clone P28H1 genomic sequence | 1929 | 1929 | 98% | 0.0 | 99% | MF620019.1 |
| 76Select seq CP025739.1 | Escherichia coli strain Ec40 chromosome | 1929 | 13303 | 98% | 0.0 | 99% | CP025739.1 |
| 77Select seq CP025707.1 | Escherichia coli strain YDC107 chromosome, complete genome | 1929 | 13348 | 98% | 0.0 | 99% | CP025707.1 |
| 78Select seq CP025627.1 | Escherichia coli strain SCEC020007 chromosome, complete genome | 1929 | 13503 | 98% | 0.0 | 99% | CP025627.1 |
| 79Select seq CP024650.2 | Escherichia coli strain BH100 chromosome, complete genome | 1929 | 13503 | 98% | 0.0 | 99% | CP024650.2 |
| 80Select seq CP023673.1 | Escherichia coli strain SMN013SH2 chromosome, complete genome | 1929 | 13503 | 98% | 0.0 | 99% | CP023673.1 |
| 81Select seq CP022279.1 | Escherichia coli strain STEC299 chromosome, complete genome | 1929 | 13503 | 98% | 0.0 | 99% | CP022279.1 |
| 82Select seq CP024618.1 | Escherichia coli strain SMN152SH1 chromosome, complete genome | 1929 | 13196 | 98% | 0.0 | 99% | CP024618.1 |
| 83Select seq CP024056.1 | Escherichia coli strain SMN197SH3 chromosome, complete genome | 1929 | 13503 | 98% | 0.0 | 99% | CP024056.1 |
| 84Select seq CP019051.1 | Escherichia coli strain CRE1540 chromosome, complete genome | 1929 | 13386 | 98% | 0.0 | 99% | CP019051.1 |
| 85Select seq CP019071.1 | Escherichia coli strain CRE1493 chromosome, complete genome | 1929 | 13464 | 98% | 0.0 | 99% | CP019071.1 |
| 86Select seq CP025251.1 | Escherichia coli strain BH100 chromosome, complete genome | 1929 | 13233 | 98% | 0.0 | 99% | CP025251.1 |
| 87Select seq CP024127.1 | Escherichia coli strain 14EC001 chromosome, complete genome | 1929 | 13303 | 98% | 0.0 | 99% | CP024127.1 |
| 88Select seq CP024131.1 | Escherichia coli strain 14EC007 chromosome, complete genome | 1929 | 13360 | 98% | 0.0 | 99% | CP024131.1 |
| 89Select seq CP024134.1 | Escherichia coli strain 14EC017 chromosome, complete genome | 1929 | 13288 | 98% | 0.0 | 99% | CP024134.1 |
| 90Select seq CP024138.1 | Escherichia coli strain 14EC020 chromosome, complete genome | 1929 | 13187 | 98% | 0.0 | 99% | CP024138.1 |
| 91Select seq CP024141.1 | Escherichia coli strain 14EC029 chromosome, complete genome | 1929 | 13224 | 98% | 0.0 | 99% | CP024141.1 |
| 92Select seq MG736060.1 | Escherichia coli strain DL1501 16S ribosomal RNA gene, partial sequence | 1929 | 1929 | 98% | 0.0 | 99% | MG736060.1 |
| 93Select seq CP025401.1 | Escherichia coli strain MS8345 chromosome, complete genome | 1929 | 13456 | 98% | 0.0 | 99% | CP025401.1 |
| 94Select seq KY524294.1 | Shigella sp. strain 7 16S ribosomal RNA gene, partial sequence | 1929 | 1929 | 98% | 0.0 | 99% | KY524294.1 |
| 95Select seq KY524291.1 | Cronobacter sakazakii strain 6 16S ribosomal RNA gene, partial sequence | 1929 | 1929 | 98% | 0.0 | 99% | KY524291.1 |
| 96Select seq KY524290.1 | Cronobacter sakazakii strain 1 16S ribosomal RNA gene, partial sequence | 1929 | 1929 | 98% | 0.0 | 99% | KY524290.1 |
| 97Select seq CP025328.1 | Escherichia coli strain ExPEC XM chromosome, complete genome | 1929 | 13497 | 98% | 0.0 | 99% | CP025328.1 |
| 98Select seq MG669346.1 | Uncultured Escherichia sp. clone SH13 16S ribosomal RNA gene, partial sequence | 1929 | 1929 | 98% | 0.0 | 99% | MG669346.1 |
| 99Select seq CP025268.1 | Escherichia coli str. K-12 substr. MG1655 strain K-12 chromosome, complete genome | 1929 | 13458 | 98% | 0.0 | 99% | CP025268.1 |
| 100Select seq CP022407.1 | Escherichia coli O121:H19 strain 16-9255 chromosome, complete genome | 1929 | 13503 | 98% | 0.0 | 99% | CP022407.1 |


## Alignments

Loading alignment... for sequences gi|1352880448,gi|1352181442 AlignDB=on Reading indexes 1-2

Download

FASTA (complete sequence)

FASTA (aligned sequences)

GenBank (complete sequence)

Continue
Cancel

GenBankGraphics

Sort by:

E value
 Score
Percent identity
Query start position
Subject start position

Next
Previous
Descriptions

Escherichia coli O157 strain FDAARGOS\_293 chromosome, complete genome

Sequence ID: CP022050.2Length: 5548751Number of Matches: 7

Related Information

Range 1: 3721951 to 3723002GenBankGraphics

Next Match
Previous Match
First Match

Alignment statistics for match #1

| Score | Expect | Identities | Gaps | Strand | Frame |
| --- | --- | --- | --- | --- | --- |
| 1929 bits(1044) | 0.0() | 1048/1052(99%) | 0/1052(0%) | Plus/Minus |  |

Features:

```
Query  10       CGCCCTCCCGAAGGTTANGCTACCTACTTCTTTTGCANCCCACTCCCATGGTGTGACGGG  69
                ||||||||||||||||| ||||||||||||||||||| ||||||||||||||||||||||
Sbjct  3723002  CGCCCTCCCGAAGGTTAAGCTACCTACTTCTTTTGCAACCCACTCCCATGGTGTGACGGG  3722943

Query  70       CGGTGTGTACAAGGCCCGGGAACGTATTCACCGTGGCATTCTGATCCACGATTACTAGCG  129
                ||||||||||||||||||||||||||||||||||||||||||||||||||||||||||||
Sbjct  3722942  CGGTGTGTACAAGGCCCGGGAACGTATTCACCGTGGCATTCTGATCCACGATTACTAGCG  3722883

Query  130      ATTCCGACTTCATGGAGTCGAGTTGCAGACTCCAATCCGGACTACGACGCACTTTATGAG  189
                ||||||||||||||||||||||||||||||||||||||||||||||||||||||||||||
Sbjct  3722882  ATTCCGACTTCATGGAGTCGAGTTGCAGACTCCAATCCGGACTACGACGCACTTTATGAG  3722823

Query  190      GTCCGCTTGCTCTCGCGAGGTCGCTTCTCTTTGTATGCGCCATTGTAGCACGTGTGTAGC  249
                ||||||||||||||||||||||||||||||||||||||||||||||||||||||||||||
Sbjct  3722822  GTCCGCTTGCTCTCGCGAGGTCGCTTCTCTTTGTATGCGCCATTGTAGCACGTGTGTAGC  3722763

Query  250      CCTGGTCGTAAGGGCCATGATGACTTGACGTCATCCCCACCTTCCTCCAGTTTATCACTG  309
                ||||||||||||||||||||||||||||||||||||||||||||||||||||||||||||
Sbjct  3722762  CCTGGTCGTAAGGGCCATGATGACTTGACGTCATCCCCACCTTCCTCCAGTTTATCACTG  3722703

Query  310      GCAGTCTCCTTTGAGTTCCCGGCCGGACCGCTGGCAACAAAGGATAAGGGTTGCGCTCGT  369
                ||||||||||||||||||||||||||||||||||||||||||||||||||||||||||||
Sbjct  3722702  GCAGTCTCCTTTGAGTTCCCGGCCGGACCGCTGGCAACAAAGGATAAGGGTTGCGCTCGT  3722643

Query  370      TGCGGGACTTAACCCAACATTTCACAACACGAGCTGACGACAGCCATGCAGCACCTGTCT  429
                ||||||||||||||||||||||||||||||||||||||||||||||||||||||||||||
Sbjct  3722642  TGCGGGACTTAACCCAACATTTCACAACACGAGCTGACGACAGCCATGCAGCACCTGTCT  3722583

Query  430      CACGGTTCCCGAAGGCACATTCTCATCTCTGAAAACTTCCGTGGATGTCAAGACCAGGTA  489
                ||||||||||||||||||||||||||||||||||||||||||||||||||||||||||||
Sbjct  3722582  CACGGTTCCCGAAGGCACATTCTCATCTCTGAAAACTTCCGTGGATGTCAAGACCAGGTA  3722523

Query  490      AGGTTCTTCGCGTTGCATCGAATTAAACCACATGCTCCACCGCTTGTGCGGGCCCCCGTC  549
                ||||||||||||||||||||||||||||||||||||||||||||||||||||||||||||
Sbjct  3722522  AGGTTCTTCGCGTTGCATCGAATTAAACCACATGCTCCACCGCTTGTGCGGGCCCCCGTC  3722463

Query  550      AATTCATTTGAGTTTTAACCTTGCGGCCGTACTCCCCAGGCGGTCGACTTAACGCGTTAG  609
                ||||||||||||||||||||||||||||||||||||||||||||||||||||||||||||
Sbjct  3722462  AATTCATTTGAGTTTTAACCTTGCGGCCGTACTCCCCAGGCGGTCGACTTAACGCGTTAG  3722403

Query  610      CTCCGGAAGCCACGCCTCAAGGGCACAACCTCCAAGTCGACATCGTTTACGGCGTGGACT  669
                ||||||||||||||||||||||||||||||||||||||||||||||||||||||||||||
Sbjct  3722402  CTCCGGAAGCCACGCCTCAAGGGCACAACCTCCAAGTCGACATCGTTTACGGCGTGGACT  3722343

Query  670      ACCAGGGTATCTAATCCTGTTTGCTCCCCACGCTTTCGCACCTGAGCGTCAGTCTTCGTC  729
                ||||||||||||||||||||||||||||||||||||||||||||||||||||||||||||
Sbjct  3722342  ACCAGGGTATCTAATCCTGTTTGCTCCCCACGCTTTCGCACCTGAGCGTCAGTCTTCGTC  3722283

Query  730      CAGGGGGCCGCCTTCGCCACCGGTATTCCTCCAGATCTCTACGCATTTCACCGCTACACC  789
                ||||||||||||||||||||||||||||||||||||||||||||||||||||||||||||
Sbjct  3722282  CAGGGGGCCGCCTTCGCCACCGGTATTCCTCCAGATCTCTACGCATTTCACCGCTACACC  3722223

Query  790      TGGAATTCTACCCCCCTCTACGAGACTCAAGCTTGCCAGTATCAGATGCAGTTCCCAGGT  849
                ||||||||||||||||||||||||||||||||||||||||||||||||||||||||||||
Sbjct  3722222  TGGAATTCTACCCCCCTCTACGAGACTCAAGCTTGCCAGTATCAGATGCAGTTCCCAGGT  3722163

Query  850      TGAGCCCGGGGATTTCACATCTGACTTAACAAACCGCCTGCGTGCGCTTTACGCCCAGTA  909
                ||||||||||||||||||||||||||||||||||||||||||||||||||||||||||||
Sbjct  3722162  TGAGCCCGGGGATTTCACATCTGACTTAACAAACCGCCTGCGTGCGCTTTACGCCCAGTA  3722103

Query  910      ATTCCGATTAACGCTTGCACCCTCCGTATTACCGCGGCTGCTGGCACGGAGTTAGCCGGT  969
                ||||||||||||||||||||||||||||||||||||||||||||||||||||||||||||
Sbjct  3722102  ATTCCGATTAACGCTTGCACCCTCCGTATTACCGCGGCTGCTGGCACGGAGTTAGCCGGT  3722043

Query  970      GCTTCTTCTGCGGGTAACGTCAATGAGCAAAGGNNTTAACTTTACTCCCTTCCTCCCCGC  1029
                |||||||||||||||||||||||||||||||||  |||||||||||||||||||||||||
Sbjct  3722042  GCTTCTTCTGCGGGTAACGTCAATGAGCAAAGGTATTAACTTTACTCCCTTCCTCCCCGC  3721983

Query  1030     TGAAAGTACTTTACAACCCGAAGGCCTTCTTC  1061
                ||||||||||||||||||||||||||||||||
Sbjct  3721982  TGAAAGTACTTTACAACCCGAAGGCCTTCTTC  3721951
```

Range 2: 2163735 to 2164786GenBankGraphics

Next Match
Previous Match
First Match

Alignment statistics for match #2

| Score | Expect | Identities | Gaps | Strand | Frame |
| --- | --- | --- | --- | --- | --- |
| 1890 bits(1023) | 0.0() | 1042/1053(99%) | 2/1053(0%) | Plus/Plus |  |

Features:

```
Query  10       CGCCCTCCCGAAGGTTANGCTACCTACTTCTTTTGCANCCCACTCCCATGGTGTGACGGG  69
                ||||||||||||||||| ||||||||||||||||||| ||||||||||||||||||||||
Sbjct  2163735  CGCCCTCCCGAAGGTTAAGCTACCTACTTCTTTTGCAACCCACTCCCATGGTGTGACGGG  2163794

Query  70       CGGTGTGTACAAGGCCCGGGAACGTATTCACCGTGGCATTCTGATCCACGATTACTAGCG  129
                ||||||||||||||||||||||||||||||||||||||||||||||||||||||||||||
Sbjct  2163795  CGGTGTGTACAAGGCCCGGGAACGTATTCACCGTGGCATTCTGATCCACGATTACTAGCG  2163854

Query  130      ATTCCGACTTCATGGAGTCGAGTTGCAGACTCCAATCCGGACTACGACGCACTTTATGAG  189
                ||||||||||||||||||||||||||||||||||||||||||||||||||||||||||||
Sbjct  2163855  ATTCCGACTTCATGGAGTCGAGTTGCAGACTCCAATCCGGACTACGACGCACTTTATGAG  2163914

Query  190      GTCCGCTTGCTCTCGCGAGGTCGCTTCTCTTTGTATGCGCCATTGTAGCACGTGTGTAGC  249
                ||||||||||||||||||||||||||||||||||||||||||||||||||||||||||||
Sbjct  2163915  GTCCGCTTGCTCTCGCGAGGTCGCTTCTCTTTGTATGCGCCATTGTAGCACGTGTGTAGC  2163974

Query  250      CCTGGTCGTAAGGGCCATGATGACTTGACGTCATCCCCACCTTCCTCCAGTTTATCACTG  309
                ||||||||||||||||||||||||||||||||||||||||||||||||||||||||||||
Sbjct  2163975  CCTGGTCGTAAGGGCCATGATGACTTGACGTCATCCCCACCTTCCTCCAGTTTATCACTG  2164034

Query  310      GCAGTCTCCTTTGAGTTCCCGGCCGGACCGCTGGCAACAAAGGATAAGGGTTGCGCTCGT  369
                ||||||||||||||||||||||||||||||||||||||||||||||||||||||||||||
Sbjct  2164035  GCAGTCTCCTTTGAGTTCCCGGCCGGACCGCTGGCAACAAAGGATAAGGGTTGCGCTCGT  2164094

Query  370      TGCGGGACTTAACCCAACATTTCACAACACGAGCTGACGACAGCCATGCAGCACCTGTCT  429
                ||||||||||||||||||||||||||||||||||||||||||||||||||||||||||||
Sbjct  2164095  TGCGGGACTTAACCCAACATTTCACAACACGAGCTGACGACAGCCATGCAGCACCTGTCT  2164154

Query  430      CACGGTTCCCGAAGGCAC-ATTCTCATCTCTGAAAACTTCCGTGGATGTCAAGACCAGGT  488
                ||| |||||||||||||| | || |||||||| ||| ||| |||||||||||||||||||
Sbjct  2164155  CACAGTTCCCGAAGGCACCAATC-CATCTCTGGAAAGTTCTGTGGATGTCAAGACCAGGT  2164213

Query  489      AAGGTTCTTCGCGTTGCATCGAATTAAACCACATGCTCCACCGCTTGTGCGGGCCCCCGT  548
                ||||||||||||||||||||||||||||||||||||||||||||||||||||||||||||
Sbjct  2164214  AAGGTTCTTCGCGTTGCATCGAATTAAACCACATGCTCCACCGCTTGTGCGGGCCCCCGT  2164273

Query  549      CAATTCATTTGAGTTTTAACCTTGCGGCCGTACTCCCCAGGCGGTCGACTTAACGCGTTA  608
                ||||||||||||||||||||||||||||||||||||||||||||||||||||||||||||
Sbjct  2164274  CAATTCATTTGAGTTTTAACCTTGCGGCCGTACTCCCCAGGCGGTCGACTTAACGCGTTA  2164333

Query  609      GCTCCGGAAGCCACGCCTCAAGGGCACAACCTCCAAGTCGACATCGTTTACGGCGTGGAC  668
                ||||||||||||||||||||||||||||||||||||||||||||||||||||||||||||
Sbjct  2164334  GCTCCGGAAGCCACGCCTCAAGGGCACAACCTCCAAGTCGACATCGTTTACGGCGTGGAC  2164393

Query  669      TACCAGGGTATCTAATCCTGTTTGCTCCCCACGCTTTCGCACCTGAGCGTCAGTCTTCGT  728
                ||||||||||||||||||||||||||||||||||||||||||||||||||||||||||||
Sbjct  2164394  TACCAGGGTATCTAATCCTGTTTGCTCCCCACGCTTTCGCACCTGAGCGTCAGTCTTCGT  2164453

Query  729      CCAGGGGGCCGCCTTCGCCACCGGTATTCCTCCAGATCTCTACGCATTTCACCGCTACAC  788
                ||||||||||||||||||||||||||||||||||||||||||||||||||||||||||||
Sbjct  2164454  CCAGGGGGCCGCCTTCGCCACCGGTATTCCTCCAGATCTCTACGCATTTCACCGCTACAC  2164513

Query  789      CTGGAATTCTACCCCCCTCTACGAGACTCAAGCTTGCCAGTATCAGATGCAGTTCCCAGG  848
                ||||||||||||||||||||||||||||||||||||||||||||||||||||||||||||
Sbjct  2164514  CTGGAATTCTACCCCCCTCTACGAGACTCAAGCTTGCCAGTATCAGATGCAGTTCCCAGG  2164573

Query  849      TTGAGCCCGGGGATTTCACATCTGACTTAACAAACCGCCTGCGTGCGCTTTACGCCCAGT  908
                ||||||||||||||||||||||||||||||||||||||||||||||||||||||||||||
Sbjct  2164574  TTGAGCCCGGGGATTTCACATCTGACTTAACAAACCGCCTGCGTGCGCTTTACGCCCAGT  2164633

Query  909      AATTCCGATTAACGCTTGCACCCTCCGTATTACCGCGGCTGCTGGCACGGAGTTAGCCGG  968
                ||||||||||||||||||||||||||||||||||||||||||||||||||||||||||||
Sbjct  2164634  AATTCCGATTAACGCTTGCACCCTCCGTATTACCGCGGCTGCTGGCACGGAGTTAGCCGG  2164693

Query  969      TGCTTCTTCTGCGGGTAACGTCAATGAGCAAAGGNNTTAACTTTACTCCCTTCCTCCCCG  1028
                ||||||||||||||||||||||||||||||||||  ||||||||||||||||||||||||
Sbjct  2164694  TGCTTCTTCTGCGGGTAACGTCAATGAGCAAAGGTATTAACTTTACTCCCTTCCTCCCCG  2164753

Query  1029     CTGAAAGTACTTTACAACCCGAAGGCCTTCTTC  1061
                |||||||||||||||||||||||||||||||||
Sbjct  2164754  CTGAAAGTACTTTACAACCCGAAGGCCTTCTTC  2164786
```

Range 3: 2443750 to 2444801GenBankGraphics

Next Match
Previous Match
First Match

Alignment statistics for match #3

| Score | Expect | Identities | Gaps | Strand | Frame |
| --- | --- | --- | --- | --- | --- |
| 1890 bits(1023) | 0.0() | 1042/1053(99%) | 2/1053(0%) | Plus/Plus |  |

Features:

```
Query  10       CGCCCTCCCGAAGGTTANGCTACCTACTTCTTTTGCANCCCACTCCCATGGTGTGACGGG  69
                ||||||||||||||||| ||||||||||||||||||| ||||||||||||||||||||||
Sbjct  2443750  CGCCCTCCCGAAGGTTAAGCTACCTACTTCTTTTGCAACCCACTCCCATGGTGTGACGGG  2443809

Query  70       CGGTGTGTACAAGGCCCGGGAACGTATTCACCGTGGCATTCTGATCCACGATTACTAGCG  129
                ||||||||||||||||||||||||||||||||||||||||||||||||||||||||||||
Sbjct  2443810  CGGTGTGTACAAGGCCCGGGAACGTATTCACCGTGGCATTCTGATCCACGATTACTAGCG  2443869

Query  130      ATTCCGACTTCATGGAGTCGAGTTGCAGACTCCAATCCGGACTACGACGCACTTTATGAG  189
                ||||||||||||||||||||||||||||||||||||||||||||||||||||||||||||
Sbjct  2443870  ATTCCGACTTCATGGAGTCGAGTTGCAGACTCCAATCCGGACTACGACGCACTTTATGAG  2443929

Query  190      GTCCGCTTGCTCTCGCGAGGTCGCTTCTCTTTGTATGCGCCATTGTAGCACGTGTGTAGC  249
                ||||||||||||||||||||||||||||||||||||||||||||||||||||||||||||
Sbjct  2443930  GTCCGCTTGCTCTCGCGAGGTCGCTTCTCTTTGTATGCGCCATTGTAGCACGTGTGTAGC  2443989

Query  250      CCTGGTCGTAAGGGCCATGATGACTTGACGTCATCCCCACCTTCCTCCAGTTTATCACTG  309
                ||||||||||||||||||||||||||||||||||||||||||||||||||||||||||||
Sbjct  2443990  CCTGGTCGTAAGGGCCATGATGACTTGACGTCATCCCCACCTTCCTCCAGTTTATCACTG  2444049

Query  310      GCAGTCTCCTTTGAGTTCCCGGCCGGACCGCTGGCAACAAAGGATAAGGGTTGCGCTCGT  369
                ||||||||||||||||||||||||||||||||||||||||||||||||||||||||||||
Sbjct  2444050  GCAGTCTCCTTTGAGTTCCCGGCCGGACCGCTGGCAACAAAGGATAAGGGTTGCGCTCGT  2444109

Query  370      TGCGGGACTTAACCCAACATTTCACAACACGAGCTGACGACAGCCATGCAGCACCTGTCT  429
                ||||||||||||||||||||||||||||||||||||||||||||||||||||||||||||
Sbjct  2444110  TGCGGGACTTAACCCAACATTTCACAACACGAGCTGACGACAGCCATGCAGCACCTGTCT  2444169

Query  430      CACGGTTCCCGAAGGCAC-ATTCTCATCTCTGAAAACTTCCGTGGATGTCAAGACCAGGT  488
                ||| |||||||||||||| | || |||||||| ||| ||| |||||||||||||||||||
Sbjct  2444170  CACAGTTCCCGAAGGCACCAATC-CATCTCTGGAAAGTTCTGTGGATGTCAAGACCAGGT  2444228

Query  489      AAGGTTCTTCGCGTTGCATCGAATTAAACCACATGCTCCACCGCTTGTGCGGGCCCCCGT  548
                ||||||||||||||||||||||||||||||||||||||||||||||||||||||||||||
Sbjct  2444229  AAGGTTCTTCGCGTTGCATCGAATTAAACCACATGCTCCACCGCTTGTGCGGGCCCCCGT  2444288

Query  549      CAATTCATTTGAGTTTTAACCTTGCGGCCGTACTCCCCAGGCGGTCGACTTAACGCGTTA  608
                ||||||||||||||||||||||||||||||||||||||||||||||||||||||||||||
Sbjct  2444289  CAATTCATTTGAGTTTTAACCTTGCGGCCGTACTCCCCAGGCGGTCGACTTAACGCGTTA  2444348

Query  609      GCTCCGGAAGCCACGCCTCAAGGGCACAACCTCCAAGTCGACATCGTTTACGGCGTGGAC  668
                ||||||||||||||||||||||||||||||||||||||||||||||||||||||||||||
Sbjct  2444349  GCTCCGGAAGCCACGCCTCAAGGGCACAACCTCCAAGTCGACATCGTTTACGGCGTGGAC  2444408

Query  669      TACCAGGGTATCTAATCCTGTTTGCTCCCCACGCTTTCGCACCTGAGCGTCAGTCTTCGT  728
                ||||||||||||||||||||||||||||||||||||||||||||||||||||||||||||
Sbjct  2444409  TACCAGGGTATCTAATCCTGTTTGCTCCCCACGCTTTCGCACCTGAGCGTCAGTCTTCGT  2444468

Query  729      CCAGGGGGCCGCCTTCGCCACCGGTATTCCTCCAGATCTCTACGCATTTCACCGCTACAC  788
                ||||||||||||||||||||||||||||||||||||||||||||||||||||||||||||
Sbjct  2444469  CCAGGGGGCCGCCTTCGCCACCGGTATTCCTCCAGATCTCTACGCATTTCACCGCTACAC  2444528

Query  789      CTGGAATTCTACCCCCCTCTACGAGACTCAAGCTTGCCAGTATCAGATGCAGTTCCCAGG  848
                ||||||||||||||||||||||||||||||||||||||||||||||||||||||||||||
Sbjct  2444529  CTGGAATTCTACCCCCCTCTACGAGACTCAAGCTTGCCAGTATCAGATGCAGTTCCCAGG  2444588

Query  849      TTGAGCCCGGGGATTTCACATCTGACTTAACAAACCGCCTGCGTGCGCTTTACGCCCAGT  908
                ||||||||||||||||||||||||||||||||||||||||||||||||||||||||||||
Sbjct  2444589  TTGAGCCCGGGGATTTCACATCTGACTTAACAAACCGCCTGCGTGCGCTTTACGCCCAGT  2444648

Query  909      AATTCCGATTAACGCTTGCACCCTCCGTATTACCGCGGCTGCTGGCACGGAGTTAGCCGG  968
                ||||||||||||||||||||||||||||||||||||||||||||||||||||||||||||
Sbjct  2444649  AATTCCGATTAACGCTTGCACCCTCCGTATTACCGCGGCTGCTGGCACGGAGTTAGCCGG  2444708

Query  969      TGCTTCTTCTGCGGGTAACGTCAATGAGCAAAGGNNTTAACTTTACTCCCTTCCTCCCCG  1028
                ||||||||||||||||||||||||||||||||||  ||||||||||||||||||||||||
Sbjct  2444709  TGCTTCTTCTGCGGGTAACGTCAATGAGCAAAGGTATTAACTTTACTCCCTTCCTCCCCG  2444768

Query  1029     CTGAAAGTACTTTACAACCCGAAGGCCTTCTTC  1061
                |||||||||||||||||||||||||||||||||
Sbjct  2444769  CTGAAAGTACTTTACAACCCGAAGGCCTTCTTC  2444801
```

Range 4: 3028376 to 3029427GenBankGraphics

Next Match
Previous Match
First Match

Alignment statistics for match #4

| Score | Expect | Identities | Gaps | Strand | Frame |
| --- | --- | --- | --- | --- | --- |
| 1890 bits(1023) | 0.0() | 1042/1053(99%) | 2/1053(0%) | Plus/Minus |  |

Features:

```
Query  10       CGCCCTCCCGAAGGTTANGCTACCTACTTCTTTTGCANCCCACTCCCATGGTGTGACGGG  69
                ||||||||||||||||| ||||||||||||||||||| ||||||||||||||||||||||
Sbjct  3029427  CGCCCTCCCGAAGGTTAAGCTACCTACTTCTTTTGCAACCCACTCCCATGGTGTGACGGG  3029368

Query  70       CGGTGTGTACAAGGCCCGGGAACGTATTCACCGTGGCATTCTGATCCACGATTACTAGCG  129
                ||||||||||||||||||||||||||||||||||||||||||||||||||||||||||||
Sbjct  3029367  CGGTGTGTACAAGGCCCGGGAACGTATTCACCGTGGCATTCTGATCCACGATTACTAGCG  3029308

Query  130      ATTCCGACTTCATGGAGTCGAGTTGCAGACTCCAATCCGGACTACGACGCACTTTATGAG  189
                ||||||||||||||||||||||||||||||||||||||||||||||||||||||||||||
Sbjct  3029307  ATTCCGACTTCATGGAGTCGAGTTGCAGACTCCAATCCGGACTACGACGCACTTTATGAG  3029248

Query  190      GTCCGCTTGCTCTCGCGAGGTCGCTTCTCTTTGTATGCGCCATTGTAGCACGTGTGTAGC  249
                ||||||||||||||||||||||||||||||||||||||||||||||||||||||||||||
Sbjct  3029247  GTCCGCTTGCTCTCGCGAGGTCGCTTCTCTTTGTATGCGCCATTGTAGCACGTGTGTAGC  3029188

Query  250      CCTGGTCGTAAGGGCCATGATGACTTGACGTCATCCCCACCTTCCTCCAGTTTATCACTG  309
                ||||||||||||||||||||||||||||||||||||||||||||||||||||||||||||
Sbjct  3029187  CCTGGTCGTAAGGGCCATGATGACTTGACGTCATCCCCACCTTCCTCCAGTTTATCACTG  3029128

Query  310      GCAGTCTCCTTTGAGTTCCCGGCCGGACCGCTGGCAACAAAGGATAAGGGTTGCGCTCGT  369
                ||||||||||||||||||||||||||||||||||||||||||||||||||||||||||||
Sbjct  3029127  GCAGTCTCCTTTGAGTTCCCGGCCGGACCGCTGGCAACAAAGGATAAGGGTTGCGCTCGT  3029068

Query  370      TGCGGGACTTAACCCAACATTTCACAACACGAGCTGACGACAGCCATGCAGCACCTGTCT  429
                ||||||||||||||||||||||||||||||||||||||||||||||||||||||||||||
Sbjct  3029067  TGCGGGACTTAACCCAACATTTCACAACACGAGCTGACGACAGCCATGCAGCACCTGTCT  3029008

Query  430      CACGGTTCCCGAAGGCAC-ATTCTCATCTCTGAAAACTTCCGTGGATGTCAAGACCAGGT  488
                ||| |||||||||||||| | || |||||||| ||| ||| |||||||||||||||||||
Sbjct  3029007  CACAGTTCCCGAAGGCACCAATC-CATCTCTGGAAAGTTCTGTGGATGTCAAGACCAGGT  3028949

Query  489      AAGGTTCTTCGCGTTGCATCGAATTAAACCACATGCTCCACCGCTTGTGCGGGCCCCCGT  548
                ||||||||||||||||||||||||||||||||||||||||||||||||||||||||||||
Sbjct  3028948  AAGGTTCTTCGCGTTGCATCGAATTAAACCACATGCTCCACCGCTTGTGCGGGCCCCCGT  3028889

Query  549      CAATTCATTTGAGTTTTAACCTTGCGGCCGTACTCCCCAGGCGGTCGACTTAACGCGTTA  608
                ||||||||||||||||||||||||||||||||||||||||||||||||||||||||||||
Sbjct  3028888  CAATTCATTTGAGTTTTAACCTTGCGGCCGTACTCCCCAGGCGGTCGACTTAACGCGTTA  3028829

Query  609      GCTCCGGAAGCCACGCCTCAAGGGCACAACCTCCAAGTCGACATCGTTTACGGCGTGGAC  668
                ||||||||||||||||||||||||||||||||||||||||||||||||||||||||||||
Sbjct  3028828  GCTCCGGAAGCCACGCCTCAAGGGCACAACCTCCAAGTCGACATCGTTTACGGCGTGGAC  3028769

Query  669      TACCAGGGTATCTAATCCTGTTTGCTCCCCACGCTTTCGCACCTGAGCGTCAGTCTTCGT  728
                ||||||||||||||||||||||||||||||||||||||||||||||||||||||||||||
Sbjct  3028768  TACCAGGGTATCTAATCCTGTTTGCTCCCCACGCTTTCGCACCTGAGCGTCAGTCTTCGT  3028709

Query  729      CCAGGGGGCCGCCTTCGCCACCGGTATTCCTCCAGATCTCTACGCATTTCACCGCTACAC  788
                ||||||||||||||||||||||||||||||||||||||||||||||||||||||||||||
Sbjct  3028708  CCAGGGGGCCGCCTTCGCCACCGGTATTCCTCCAGATCTCTACGCATTTCACCGCTACAC  3028649

Query  789      CTGGAATTCTACCCCCCTCTACGAGACTCAAGCTTGCCAGTATCAGATGCAGTTCCCAGG  848
                ||||||||||||||||||||||||||||||||||||||||||||||||||||||||||||
Sbjct  3028648  CTGGAATTCTACCCCCCTCTACGAGACTCAAGCTTGCCAGTATCAGATGCAGTTCCCAGG  3028589

Query  849      TTGAGCCCGGGGATTTCACATCTGACTTAACAAACCGCCTGCGTGCGCTTTACGCCCAGT  908
                ||||||||||||||||||||||||||||||||||||||||||||||||||||||||||||
Sbjct  3028588  TTGAGCCCGGGGATTTCACATCTGACTTAACAAACCGCCTGCGTGCGCTTTACGCCCAGT  3028529

Query  909      AATTCCGATTAACGCTTGCACCCTCCGTATTACCGCGGCTGCTGGCACGGAGTTAGCCGG  968
                ||||||||||||||||||||||||||||||||||||||||||||||||||||||||||||
Sbjct  3028528  AATTCCGATTAACGCTTGCACCCTCCGTATTACCGCGGCTGCTGGCACGGAGTTAGCCGG  3028469

Query  969      TGCTTCTTCTGCGGGTAACGTCAATGAGCAAAGGNNTTAACTTTACTCCCTTCCTCCCCG  1028
                ||||||||||||||||||||||||||||||||||  ||||||||||||||||||||||||
Sbjct  3028468  TGCTTCTTCTGCGGGTAACGTCAATGAGCAAAGGTATTAACTTTACTCCCTTCCTCCCCG  3028409

Query  1029     CTGAAAGTACTTTACAACCCGAAGGCCTTCTTC  1061
                |||||||||||||||||||||||||||||||||
Sbjct  3028408  CTGAAAGTACTTTACAACCCGAAGGCCTTCTTC  3028376
```

Range 5: 1442277 to 1443328GenBankGraphics

Next Match
Previous Match
First Match

Alignment statistics for match #5

| Score | Expect | Identities | Gaps | Strand | Frame |
| --- | --- | --- | --- | --- | --- |
| 1884 bits(1020) | 0.0() | 1041/1053(99%) | 2/1053(0%) | Plus/Plus |  |

Features:

```
Query  10       CGCCCTCCCGAAGGTTANGCTACCTACTTCTTTTGCANCCCACTCCCATGGTGTGACGGG  69
                ||||||||||||||||| ||||||||||||||||||| ||||||||||||||||||||||
Sbjct  1442277  CGCCCTCCCGAAGGTTAAGCTACCTACTTCTTTTGCAACCCACTCCCATGGTGTGACGGG  1442336

Query  70       CGGTGTGTACAAGGCCCGGGAACGTATTCACCGTGGCATTCTGATCCACGATTACTAGCG  129
                ||||||||||||||||||||||||||||||||||||||||||||||||||||||||||||
Sbjct  1442337  CGGTGTGTACAAGGCCCGGGAACGTATTCACCGTGGCATTCTGATCCACGATTACTAGCG  1442396

Query  130      ATTCCGACTTCATGGAGTCGAGTTGCAGACTCCAATCCGGACTACGACGCACTTTATGAG  189
                |||||||||||||||| |||||||||||||||||||||||||||||||||||||||||||
Sbjct  1442397  ATTCCGACTTCATGGAATCGAGTTGCAGACTCCAATCCGGACTACGACGCACTTTATGAG  1442456

Query  190      GTCCGCTTGCTCTCGCGAGGTCGCTTCTCTTTGTATGCGCCATTGTAGCACGTGTGTAGC  249
                ||||||||||||||||||||||||||||||||||||||||||||||||||||||||||||
Sbjct  1442457  GTCCGCTTGCTCTCGCGAGGTCGCTTCTCTTTGTATGCGCCATTGTAGCACGTGTGTAGC  1442516

Query  250      CCTGGTCGTAAGGGCCATGATGACTTGACGTCATCCCCACCTTCCTCCAGTTTATCACTG  309
                ||||||||||||||||||||||||||||||||||||||||||||||||||||||||||||
Sbjct  1442517  CCTGGTCGTAAGGGCCATGATGACTTGACGTCATCCCCACCTTCCTCCAGTTTATCACTG  1442576

Query  310      GCAGTCTCCTTTGAGTTCCCGGCCGGACCGCTGGCAACAAAGGATAAGGGTTGCGCTCGT  369
                ||||||||||||||||||||||||||||||||||||||||||||||||||||||||||||
Sbjct  1442577  GCAGTCTCCTTTGAGTTCCCGGCCGGACCGCTGGCAACAAAGGATAAGGGTTGCGCTCGT  1442636

Query  370      TGCGGGACTTAACCCAACATTTCACAACACGAGCTGACGACAGCCATGCAGCACCTGTCT  429
                ||||||||||||||||||||||||||||||||||||||||||||||||||||||||||||
Sbjct  1442637  TGCGGGACTTAACCCAACATTTCACAACACGAGCTGACGACAGCCATGCAGCACCTGTCT  1442696

Query  430      CACGGTTCCCGAAGGCAC-ATTCTCATCTCTGAAAACTTCCGTGGATGTCAAGACCAGGT  488
                ||| |||||||||||||| | || |||||||| ||| ||| |||||||||||||||||||
Sbjct  1442697  CACAGTTCCCGAAGGCACCAATC-CATCTCTGGAAAGTTCTGTGGATGTCAAGACCAGGT  1442755

Query  489      AAGGTTCTTCGCGTTGCATCGAATTAAACCACATGCTCCACCGCTTGTGCGGGCCCCCGT  548
                ||||||||||||||||||||||||||||||||||||||||||||||||||||||||||||
Sbjct  1442756  AAGGTTCTTCGCGTTGCATCGAATTAAACCACATGCTCCACCGCTTGTGCGGGCCCCCGT  1442815

Query  549      CAATTCATTTGAGTTTTAACCTTGCGGCCGTACTCCCCAGGCGGTCGACTTAACGCGTTA  608
                ||||||||||||||||||||||||||||||||||||||||||||||||||||||||||||
Sbjct  1442816  CAATTCATTTGAGTTTTAACCTTGCGGCCGTACTCCCCAGGCGGTCGACTTAACGCGTTA  1442875

Query  609      GCTCCGGAAGCCACGCCTCAAGGGCACAACCTCCAAGTCGACATCGTTTACGGCGTGGAC  668
                ||||||||||||||||||||||||||||||||||||||||||||||||||||||||||||
Sbjct  1442876  GCTCCGGAAGCCACGCCTCAAGGGCACAACCTCCAAGTCGACATCGTTTACGGCGTGGAC  1442935

Query  669      TACCAGGGTATCTAATCCTGTTTGCTCCCCACGCTTTCGCACCTGAGCGTCAGTCTTCGT  728
                ||||||||||||||||||||||||||||||||||||||||||||||||||||||||||||
Sbjct  1442936  TACCAGGGTATCTAATCCTGTTTGCTCCCCACGCTTTCGCACCTGAGCGTCAGTCTTCGT  1442995

Query  729      CCAGGGGGCCGCCTTCGCCACCGGTATTCCTCCAGATCTCTACGCATTTCACCGCTACAC  788
                ||||||||||||||||||||||||||||||||||||||||||||||||||||||||||||
Sbjct  1442996  CCAGGGGGCCGCCTTCGCCACCGGTATTCCTCCAGATCTCTACGCATTTCACCGCTACAC  1443055

Query  789      CTGGAATTCTACCCCCCTCTACGAGACTCAAGCTTGCCAGTATCAGATGCAGTTCCCAGG  848
                ||||||||||||||||||||||||||||||||||||||||||||||||||||||||||||
Sbjct  1443056  CTGGAATTCTACCCCCCTCTACGAGACTCAAGCTTGCCAGTATCAGATGCAGTTCCCAGG  1443115

Query  849      TTGAGCCCGGGGATTTCACATCTGACTTAACAAACCGCCTGCGTGCGCTTTACGCCCAGT  908
                ||||||||||||||||||||||||||||||||||||||||||||||||||||||||||||
Sbjct  1443116  TTGAGCCCGGGGATTTCACATCTGACTTAACAAACCGCCTGCGTGCGCTTTACGCCCAGT  1443175

Query  909      AATTCCGATTAACGCTTGCACCCTCCGTATTACCGCGGCTGCTGGCACGGAGTTAGCCGG  968
                ||||||||||||||||||||||||||||||||||||||||||||||||||||||||||||
Sbjct  1443176  AATTCCGATTAACGCTTGCACCCTCCGTATTACCGCGGCTGCTGGCACGGAGTTAGCCGG  1443235

Query  969      TGCTTCTTCTGCGGGTAACGTCAATGAGCAAAGGNNTTAACTTTACTCCCTTCCTCCCCG  1028
                ||||||||||||||||||||||||||||||||||  ||||||||||||||||||||||||
Sbjct  1443236  TGCTTCTTCTGCGGGTAACGTCAATGAGCAAAGGTATTAACTTTACTCCCTTCCTCCCCG  1443295

Query  1029     CTGAAAGTACTTTACAACCCGAAGGCCTTCTTC  1061
                |||||||||||||||||||||||||||||||||
Sbjct  1443296  CTGAAAGTACTTTACAACCCGAAGGCCTTCTTC  1443328
```

Range 6: 2122713 to 2123764GenBankGraphics

Next Match
Previous Match
First Match

Alignment statistics for match #6

| Score | Expect | Identities | Gaps | Strand | Frame |
| --- | --- | --- | --- | --- | --- |
| 1884 bits(1020) | 0.0() | 1041/1053(99%) | 2/1053(0%) | Plus/Plus |  |

Features:

```
Query  10       CGCCCTCCCGAAGGTTANGCTACCTACTTCTTTTGCANCCCACTCCCATGGTGTGACGGG  69
                ||||||||||||||||| ||||||||||||||||||| | ||||||||||||||||||||
Sbjct  2122713  CGCCCTCCCGAAGGTTAAGCTACCTACTTCTTTTGCAACTCACTCCCATGGTGTGACGGG  2122772

Query  70       CGGTGTGTACAAGGCCCGGGAACGTATTCACCGTGGCATTCTGATCCACGATTACTAGCG  129
                ||||||||||||||||||||||||||||||||||||||||||||||||||||||||||||
Sbjct  2122773  CGGTGTGTACAAGGCCCGGGAACGTATTCACCGTGGCATTCTGATCCACGATTACTAGCG  2122832

Query  130      ATTCCGACTTCATGGAGTCGAGTTGCAGACTCCAATCCGGACTACGACGCACTTTATGAG  189
                ||||||||||||||||||||||||||||||||||||||||||||||||||||||||||||
Sbjct  2122833  ATTCCGACTTCATGGAGTCGAGTTGCAGACTCCAATCCGGACTACGACGCACTTTATGAG  2122892

Query  190      GTCCGCTTGCTCTCGCGAGGTCGCTTCTCTTTGTATGCGCCATTGTAGCACGTGTGTAGC  249
                ||||||||||||||||||||||||||||||||||||||||||||||||||||||||||||
Sbjct  2122893  GTCCGCTTGCTCTCGCGAGGTCGCTTCTCTTTGTATGCGCCATTGTAGCACGTGTGTAGC  2122952

Query  250      CCTGGTCGTAAGGGCCATGATGACTTGACGTCATCCCCACCTTCCTCCAGTTTATCACTG  309
                ||||||||||||||||||||||||||||||||||||||||||||||||||||||||||||
Sbjct  2122953  CCTGGTCGTAAGGGCCATGATGACTTGACGTCATCCCCACCTTCCTCCAGTTTATCACTG  2123012

Query  310      GCAGTCTCCTTTGAGTTCCCGGCCGGACCGCTGGCAACAAAGGATAAGGGTTGCGCTCGT  369
                ||||||||||||||||||||||||||||||||||||||||||||||||||||||||||||
Sbjct  2123013  GCAGTCTCCTTTGAGTTCCCGGCCGGACCGCTGGCAACAAAGGATAAGGGTTGCGCTCGT  2123072

Query  370      TGCGGGACTTAACCCAACATTTCACAACACGAGCTGACGACAGCCATGCAGCACCTGTCT  429
                ||||||||||||||||||||||||||||||||||||||||||||||||||||||||||||
Sbjct  2123073  TGCGGGACTTAACCCAACATTTCACAACACGAGCTGACGACAGCCATGCAGCACCTGTCT  2123132

Query  430      CACGGTTCCCGAAGGCAC-ATTCTCATCTCTGAAAACTTCCGTGGATGTCAAGACCAGGT  488
                ||| |||||||||||||| | || |||||||| ||| ||| |||||||||||||||||||
Sbjct  2123133  CACAGTTCCCGAAGGCACCAATC-CATCTCTGGAAAGTTCTGTGGATGTCAAGACCAGGT  2123191

Query  489      AAGGTTCTTCGCGTTGCATCGAATTAAACCACATGCTCCACCGCTTGTGCGGGCCCCCGT  548
                ||||||||||||||||||||||||||||||||||||||||||||||||||||||||||||
Sbjct  2123192  AAGGTTCTTCGCGTTGCATCGAATTAAACCACATGCTCCACCGCTTGTGCGGGCCCCCGT  2123251

Query  549      CAATTCATTTGAGTTTTAACCTTGCGGCCGTACTCCCCAGGCGGTCGACTTAACGCGTTA  608
                ||||||||||||||||||||||||||||||||||||||||||||||||||||||||||||
Sbjct  2123252  CAATTCATTTGAGTTTTAACCTTGCGGCCGTACTCCCCAGGCGGTCGACTTAACGCGTTA  2123311

Query  609      GCTCCGGAAGCCACGCCTCAAGGGCACAACCTCCAAGTCGACATCGTTTACGGCGTGGAC  668
                ||||||||||||||||||||||||||||||||||||||||||||||||||||||||||||
Sbjct  2123312  GCTCCGGAAGCCACGCCTCAAGGGCACAACCTCCAAGTCGACATCGTTTACGGCGTGGAC  2123371

Query  669      TACCAGGGTATCTAATCCTGTTTGCTCCCCACGCTTTCGCACCTGAGCGTCAGTCTTCGT  728
                ||||||||||||||||||||||||||||||||||||||||||||||||||||||||||||
Sbjct  2123372  TACCAGGGTATCTAATCCTGTTTGCTCCCCACGCTTTCGCACCTGAGCGTCAGTCTTCGT  2123431

Query  729      CCAGGGGGCCGCCTTCGCCACCGGTATTCCTCCAGATCTCTACGCATTTCACCGCTACAC  788
                ||||||||||||||||||||||||||||||||||||||||||||||||||||||||||||
Sbjct  2123432  CCAGGGGGCCGCCTTCGCCACCGGTATTCCTCCAGATCTCTACGCATTTCACCGCTACAC  2123491

Query  789      CTGGAATTCTACCCCCCTCTACGAGACTCAAGCTTGCCAGTATCAGATGCAGTTCCCAGG  848
                ||||||||||||||||||||||||||||||||||||||||||||||||||||||||||||
Sbjct  2123492  CTGGAATTCTACCCCCCTCTACGAGACTCAAGCTTGCCAGTATCAGATGCAGTTCCCAGG  2123551

Query  849      TTGAGCCCGGGGATTTCACATCTGACTTAACAAACCGCCTGCGTGCGCTTTACGCCCAGT  908
                ||||||||||||||||||||||||||||||||||||||||||||||||||||||||||||
Sbjct  2123552  TTGAGCCCGGGGATTTCACATCTGACTTAACAAACCGCCTGCGTGCGCTTTACGCCCAGT  2123611

Query  909      AATTCCGATTAACGCTTGCACCCTCCGTATTACCGCGGCTGCTGGCACGGAGTTAGCCGG  968
                ||||||||||||||||||||||||||||||||||||||||||||||||||||||||||||
Sbjct  2123612  AATTCCGATTAACGCTTGCACCCTCCGTATTACCGCGGCTGCTGGCACGGAGTTAGCCGG  2123671

Query  969      TGCTTCTTCTGCGGGTAACGTCAATGAGCAAAGGNNTTAACTTTACTCCCTTCCTCCCCG  1028
                ||||||||||||||||||||||||||||||||||  ||||||||||||||||||||||||
Sbjct  2123672  TGCTTCTTCTGCGGGTAACGTCAATGAGCAAAGGTATTAACTTTACTCCCTTCCTCCCCG  2123731

Query  1029     CTGAAAGTACTTTACAACCCGAAGGCCTTCTTC  1061
                |||||||||||||||||||||||||||||||||
Sbjct  2123732  CTGAAAGTACTTTACAACCCGAAGGCCTTCTTC  2123764
```

Range 7: 2347328 to 2348379GenBankGraphics

Next Match
Previous Match
First Match

Alignment statistics for match #7

| Score | Expect | Identities | Gaps | Strand | Frame |
| --- | --- | --- | --- | --- | --- |
| 1879 bits(1017) | 0.0() | 1040/1053(99%) | 2/1053(0%) | Plus/Plus |  |

Features:

```
Query  10       CGCCCTCCCGAAGGTTANGCTACCTACTTCTTTTGCANCCCACTCCCATGGTGTGACGGG  69
                ||||||||||||||||| ||||||||||||||||||| | ||||||||||||||||||||
Sbjct  2347328  CGCCCTCCCGAAGGTTAAGCTACCTACTTCTTTTGCAACTCACTCCCATGGTGTGACGGG  2347387

Query  70       CGGTGTGTACAAGGCCCGGGAACGTATTCACCGTGGCATTCTGATCCACGATTACTAGCG  129
                ||||||||||||||||||||||||||||||||||||||||||||||||||||||||||||
Sbjct  2347388  CGGTGTGTACAAGGCCCGGGAACGTATTCACCGTGGCATTCTGATCCACGATTACTAGCG  2347447

Query  130      ATTCCGACTTCATGGAGTCGAGTTGCAGACTCCAATCCGGACTACGACGCACTTTATGAG  189
                ||||||||||||||||||||||||||||||||||||||||||||||||||||||||||||
Sbjct  2347448  ATTCCGACTTCATGGAGTCGAGTTGCAGACTCCAATCCGGACTACGACGCACTTTATGAG  2347507

Query  190      GTCCGCTTGCTCTCGCGAGGTCGCTTCTCTTTGTATGCGCCATTGTAGCACGTGTGTAGC  249
                ||||||||||||||||||||||||||||||||||||||||||||||||||||||||||||
Sbjct  2347508  GTCCGCTTGCTCTCGCGAGGTCGCTTCTCTTTGTATGCGCCATTGTAGCACGTGTGTAGC  2347567

Query  250      CCTGGTCGTAAGGGCCATGATGACTTGACGTCATCCCCACCTTCCTCCAGTTTATCACTG  309
                ||||||||||||||||||||||||||||||||||||||||||||||||||||||||||||
Sbjct  2347568  CCTGGTCGTAAGGGCCATGATGACTTGACGTCATCCCCACCTTCCTCCAGTTTATCACTG  2347627

Query  310      GCAGTCTCCTTTGAGTTCCCGGCCGGACCGCTGGCAACAAAGGATAAGGGTTGCGCTCGT  369
                ||||||||||||||||||||||||||||||||||||||||||||||||||||||||||||
Sbjct  2347628  GCAGTCTCCTTTGAGTTCCCGGCCGGACCGCTGGCAACAAAGGATAAGGGTTGCGCTCGT  2347687

Query  370      TGCGGGACTTAACCCAACATTTCACAACACGAGCTGACGACAGCCATGCAGCACCTGTCT  429
                ||||||||||||||||||||||||||||||||||||||||||||||||||||||||||||
Sbjct  2347688  TGCGGGACTTAACCCAACATTTCACAACACGAGCTGACGACAGCCATGCAGCACCTGTCT  2347747

Query  430      CACGGTTCCCGAAGGCAC-ATTCTCATCTCTGAAAACTTCCGTGGATGTCAAGACCAGGT  488
                ||| |||||||||||||| | || |||||||| ||| ||| |||||||||||||||||||
Sbjct  2347748  CACAGTTCCCGAAGGCACCAATC-CATCTCTGGAAAGTTCTGTGGATGTCAAGACCAGGT  2347806

Query  489      AAGGTTCTTCGCGTTGCATCGAATTAAACCACATGCTCCACCGCTTGTGCGGGCCCCCGT  548
                ||||||||||||||||||||||||||||||||||||||||||||||||||||||||||||
Sbjct  2347807  AAGGTTCTTCGCGTTGCATCGAATTAAACCACATGCTCCACCGCTTGTGCGGGCCCCCGT  2347866

Query  549      CAATTCATTTGAGTTTTAACCTTGCGGCCGTACTCCCCAGGCGGTCGACTTAACGCGTTA  608
                ||||||||||||||||||||||||||||||||||||||||||||||||||||||||||||
Sbjct  2347867  CAATTCATTTGAGTTTTAACCTTGCGGCCGTACTCCCCAGGCGGTCGACTTAACGCGTTA  2347926

Query  609      GCTCCGGAAGCCACGCCTCAAGGGCACAACCTCCAAGTCGACATCGTTTACGGCGTGGAC  668
                ||||||||||||||||||||||||||||||||||||||||||||||||||||||||||||
Sbjct  2347927  GCTCCGGAAGCCACGCCTCAAGGGCACAACCTCCAAGTCGACATCGTTTACGGCGTGGAC  2347986

Query  669      TACCAGGGTATCTAATCCTGTTTGCTCCCCACGCTTTCGCACCTGAGCGTCAGTCTTCGT  728
                ||||||||||||||||||||||||||||||||||||||||||||||||||||||||||||
Sbjct  2347987  TACCAGGGTATCTAATCCTGTTTGCTCCCCACGCTTTCGCACCTGAGCGTCAGTCTTCGT  2348046

Query  729      CCAGGGGGCCGCCTTCGCCACCGGTATTCCTCCAGATCTCTACGCATTTCACCGCTACAC  788
                ||||||||||||||||||||||||||||||||||||||||||||||||||||||||||||
Sbjct  2348047  CCAGGGGGCCGCCTTCGCCACCGGTATTCCTCCAGATCTCTACGCATTTCACCGCTACAC  2348106

Query  789      CTGGAATTCTACCCCCCTCTACGAGACTCAAGCTTGCCAGTATCAGATGCAGTTCCCAGG  848
                |||||||||||||||||||||||||||||||||||||||||||||||||||||||||| |
Sbjct  2348107  CTGGAATTCTACCCCCCTCTACGAGACTCAAGCTTGCCAGTATCAGATGCAGTTCCCAAG  2348166

Query  849      TTGAGCCCGGGGATTTCACATCTGACTTAACAAACCGCCTGCGTGCGCTTTACGCCCAGT  908
                ||||||||||||||||||||||||||||||||||||||||||||||||||||||||||||
Sbjct  2348167  TTGAGCCCGGGGATTTCACATCTGACTTAACAAACCGCCTGCGTGCGCTTTACGCCCAGT  2348226

Query  909      AATTCCGATTAACGCTTGCACCCTCCGTATTACCGCGGCTGCTGGCACGGAGTTAGCCGG  968
                ||||||||||||||||||||||||||||||||||||||||||||||||||||||||||||
Sbjct  2348227  AATTCCGATTAACGCTTGCACCCTCCGTATTACCGCGGCTGCTGGCACGGAGTTAGCCGG  2348286

Query  969      TGCTTCTTCTGCGGGTAACGTCAATGAGCAAAGGNNTTAACTTTACTCCCTTCCTCCCCG  1028
                ||||||||||||||||||||||||||||||||||  ||||||||||||||||||||||||
Sbjct  2348287  TGCTTCTTCTGCGGGTAACGTCAATGAGCAAAGGTATTAACTTTACTCCCTTCCTCCCCG  2348346

Query  1029     CTGAAAGTACTTTACAACCCGAAGGCCTTCTTC  1061
                |||||||||||||||||||||||||||||||||
Sbjct  2348347  CTGAAAGTACTTTACAACCCGAAGGCCTTCTTC  2348379
```

Download

FASTA (complete sequence)

FASTA (aligned sequences)

GenBank (complete sequence)

Continue
Cancel

GenBankGraphics

Sort by:

E value
 Score
Percent identity
Query start position
Subject start position

Next
Previous
Descriptions

Escherichia coli str. K-12 substr. MG1655 strain K-12 chromosome

Sequence ID: CP027060.1Length: 4653240Number of Matches: 7

Related Information

Range 1: 361594 to 362645GenBankGraphics

Next Match
Previous Match
First Match

Alignment statistics for match #1

| Score | Expect | Identities | Gaps | Strand | Frame |
| --- | --- | --- | --- | --- | --- |
| 1929 bits(1044) | 0.0() | 1048/1052(99%) | 0/1052(0%) | Plus/Plus |  |

Features:

```
Query  10      CGCCCTCCCGAAGGTTANGCTACCTACTTCTTTTGCANCCCACTCCCATGGTGTGACGGG  69
               ||||||||||||||||| ||||||||||||||||||| ||||||||||||||||||||||
Sbjct  361594  CGCCCTCCCGAAGGTTAAGCTACCTACTTCTTTTGCAACCCACTCCCATGGTGTGACGGG  361653

Query  70      CGGTGTGTACAAGGCCCGGGAACGTATTCACCGTGGCATTCTGATCCACGATTACTAGCG  129
               ||||||||||||||||||||||||||||||||||||||||||||||||||||||||||||
Sbjct  361654  CGGTGTGTACAAGGCCCGGGAACGTATTCACCGTGGCATTCTGATCCACGATTACTAGCG  361713

Query  130     ATTCCGACTTCATGGAGTCGAGTTGCAGACTCCAATCCGGACTACGACGCACTTTATGAG  189
               ||||||||||||||||||||||||||||||||||||||||||||||||||||||||||||
Sbjct  361714  ATTCCGACTTCATGGAGTCGAGTTGCAGACTCCAATCCGGACTACGACGCACTTTATGAG  361773

Query  190     GTCCGCTTGCTCTCGCGAGGTCGCTTCTCTTTGTATGCGCCATTGTAGCACGTGTGTAGC  249
               ||||||||||||||||||||||||||||||||||||||||||||||||||||||||||||
Sbjct  361774  GTCCGCTTGCTCTCGCGAGGTCGCTTCTCTTTGTATGCGCCATTGTAGCACGTGTGTAGC  361833

Query  250     CCTGGTCGTAAGGGCCATGATGACTTGACGTCATCCCCACCTTCCTCCAGTTTATCACTG  309
               ||||||||||||||||||||||||||||||||||||||||||||||||||||||||||||
Sbjct  361834  CCTGGTCGTAAGGGCCATGATGACTTGACGTCATCCCCACCTTCCTCCAGTTTATCACTG  361893

Query  310     GCAGTCTCCTTTGAGTTCCCGGCCGGACCGCTGGCAACAAAGGATAAGGGTTGCGCTCGT  369
               ||||||||||||||||||||||||||||||||||||||||||||||||||||||||||||
Sbjct  361894  GCAGTCTCCTTTGAGTTCCCGGCCGGACCGCTGGCAACAAAGGATAAGGGTTGCGCTCGT  361953

Query  370     TGCGGGACTTAACCCAACATTTCACAACACGAGCTGACGACAGCCATGCAGCACCTGTCT  429
               ||||||||||||||||||||||||||||||||||||||||||||||||||||||||||||
Sbjct  361954  TGCGGGACTTAACCCAACATTTCACAACACGAGCTGACGACAGCCATGCAGCACCTGTCT  362013

Query  430     CACGGTTCCCGAAGGCACATTCTCATCTCTGAAAACTTCCGTGGATGTCAAGACCAGGTA  489
               ||||||||||||||||||||||||||||||||||||||||||||||||||||||||||||
Sbjct  362014  CACGGTTCCCGAAGGCACATTCTCATCTCTGAAAACTTCCGTGGATGTCAAGACCAGGTA  362073

Query  490     AGGTTCTTCGCGTTGCATCGAATTAAACCACATGCTCCACCGCTTGTGCGGGCCCCCGTC  549
               ||||||||||||||||||||||||||||||||||||||||||||||||||||||||||||
Sbjct  362074  AGGTTCTTCGCGTTGCATCGAATTAAACCACATGCTCCACCGCTTGTGCGGGCCCCCGTC  362133

Query  550     AATTCATTTGAGTTTTAACCTTGCGGCCGTACTCCCCAGGCGGTCGACTTAACGCGTTAG  609
               ||||||||||||||||||||||||||||||||||||||||||||||||||||||||||||
Sbjct  362134  AATTCATTTGAGTTTTAACCTTGCGGCCGTACTCCCCAGGCGGTCGACTTAACGCGTTAG  362193

Query  610     CTCCGGAAGCCACGCCTCAAGGGCACAACCTCCAAGTCGACATCGTTTACGGCGTGGACT  669
               ||||||||||||||||||||||||||||||||||||||||||||||||||||||||||||
Sbjct  362194  CTCCGGAAGCCACGCCTCAAGGGCACAACCTCCAAGTCGACATCGTTTACGGCGTGGACT  362253

Query  670     ACCAGGGTATCTAATCCTGTTTGCTCCCCACGCTTTCGCACCTGAGCGTCAGTCTTCGTC  729
               ||||||||||||||||||||||||||||||||||||||||||||||||||||||||||||
Sbjct  362254  ACCAGGGTATCTAATCCTGTTTGCTCCCCACGCTTTCGCACCTGAGCGTCAGTCTTCGTC  362313

Query  730     CAGGGGGCCGCCTTCGCCACCGGTATTCCTCCAGATCTCTACGCATTTCACCGCTACACC  789
               ||||||||||||||||||||||||||||||||||||||||||||||||||||||||||||
Sbjct  362314  CAGGGGGCCGCCTTCGCCACCGGTATTCCTCCAGATCTCTACGCATTTCACCGCTACACC  362373

Query  790     TGGAATTCTACCCCCCTCTACGAGACTCAAGCTTGCCAGTATCAGATGCAGTTCCCAGGT  849
               ||||||||||||||||||||||||||||||||||||||||||||||||||||||||||||
Sbjct  362374  TGGAATTCTACCCCCCTCTACGAGACTCAAGCTTGCCAGTATCAGATGCAGTTCCCAGGT  362433

Query  850     TGAGCCCGGGGATTTCACATCTGACTTAACAAACCGCCTGCGTGCGCTTTACGCCCAGTA  909
               ||||||||||||||||||||||||||||||||||||||||||||||||||||||||||||
Sbjct  362434  TGAGCCCGGGGATTTCACATCTGACTTAACAAACCGCCTGCGTGCGCTTTACGCCCAGTA  362493

Query  910     ATTCCGATTAACGCTTGCACCCTCCGTATTACCGCGGCTGCTGGCACGGAGTTAGCCGGT  969
               ||||||||||||||||||||||||||||||||||||||||||||||||||||||||||||
Sbjct  362494  ATTCCGATTAACGCTTGCACCCTCCGTATTACCGCGGCTGCTGGCACGGAGTTAGCCGGT  362553

Query  970     GCTTCTTCTGCGGGTAACGTCAATGAGCAAAGGNNTTAACTTTACTCCCTTCCTCCCCGC  1029
               |||||||||||||||||||||||||||||||||  |||||||||||||||||||||||||
Sbjct  362554  GCTTCTTCTGCGGGTAACGTCAATGAGCAAAGGTATTAACTTTACTCCCTTCCTCCCCGC  362613

Query  1030    TGAAAGTACTTTACAACCCGAAGGCCTTCTTC  1061
               ||||||||||||||||||||||||||||||||
Sbjct  362614  TGAAAGTACTTTACAACCCGAAGGCCTTCTTC  362645
```

Range 2: 1059199 to 1060250GenBankGraphics

Next Match
Previous Match
First Match

Alignment statistics for match #2

| Score | Expect | Identities | Gaps | Strand | Frame |
| --- | --- | --- | --- | --- | --- |
| 1929 bits(1044) | 0.0() | 1048/1052(99%) | 0/1052(0%) | Plus/Plus |  |

Features:

```
Query  10       CGCCCTCCCGAAGGTTANGCTACCTACTTCTTTTGCANCCCACTCCCATGGTGTGACGGG  69
                ||||||||||||||||| ||||||||||||||||||| ||||||||||||||||||||||
Sbjct  1059199  CGCCCTCCCGAAGGTTAAGCTACCTACTTCTTTTGCAACCCACTCCCATGGTGTGACGGG  1059258

Query  70       CGGTGTGTACAAGGCCCGGGAACGTATTCACCGTGGCATTCTGATCCACGATTACTAGCG  129
                ||||||||||||||||||||||||||||||||||||||||||||||||||||||||||||
Sbjct  1059259  CGGTGTGTACAAGGCCCGGGAACGTATTCACCGTGGCATTCTGATCCACGATTACTAGCG  1059318

Query  130      ATTCCGACTTCATGGAGTCGAGTTGCAGACTCCAATCCGGACTACGACGCACTTTATGAG  189
                ||||||||||||||||||||||||||||||||||||||||||||||||||||||||||||
Sbjct  1059319  ATTCCGACTTCATGGAGTCGAGTTGCAGACTCCAATCCGGACTACGACGCACTTTATGAG  1059378

Query  190      GTCCGCTTGCTCTCGCGAGGTCGCTTCTCTTTGTATGCGCCATTGTAGCACGTGTGTAGC  249
                ||||||||||||||||||||||||||||||||||||||||||||||||||||||||||||
Sbjct  1059379  GTCCGCTTGCTCTCGCGAGGTCGCTTCTCTTTGTATGCGCCATTGTAGCACGTGTGTAGC  1059438

Query  250      CCTGGTCGTAAGGGCCATGATGACTTGACGTCATCCCCACCTTCCTCCAGTTTATCACTG  309
                ||||||||||||||||||||||||||||||||||||||||||||||||||||||||||||
Sbjct  1059439  CCTGGTCGTAAGGGCCATGATGACTTGACGTCATCCCCACCTTCCTCCAGTTTATCACTG  1059498

Query  310      GCAGTCTCCTTTGAGTTCCCGGCCGGACCGCTGGCAACAAAGGATAAGGGTTGCGCTCGT  369
                ||||||||||||||||||||||||||||||||||||||||||||||||||||||||||||
Sbjct  1059499  GCAGTCTCCTTTGAGTTCCCGGCCGGACCGCTGGCAACAAAGGATAAGGGTTGCGCTCGT  1059558

Query  370      TGCGGGACTTAACCCAACATTTCACAACACGAGCTGACGACAGCCATGCAGCACCTGTCT  429
                ||||||||||||||||||||||||||||||||||||||||||||||||||||||||||||
Sbjct  1059559  TGCGGGACTTAACCCAACATTTCACAACACGAGCTGACGACAGCCATGCAGCACCTGTCT  1059618

Query  430      CACGGTTCCCGAAGGCACATTCTCATCTCTGAAAACTTCCGTGGATGTCAAGACCAGGTA  489
                ||||||||||||||||||||||||||||||||||||||||||||||||||||||||||||
Sbjct  1059619  CACGGTTCCCGAAGGCACATTCTCATCTCTGAAAACTTCCGTGGATGTCAAGACCAGGTA  1059678

Query  490      AGGTTCTTCGCGTTGCATCGAATTAAACCACATGCTCCACCGCTTGTGCGGGCCCCCGTC  549
                ||||||||||||||||||||||||||||||||||||||||||||||||||||||||||||
Sbjct  1059679  AGGTTCTTCGCGTTGCATCGAATTAAACCACATGCTCCACCGCTTGTGCGGGCCCCCGTC  1059738

Query  550      AATTCATTTGAGTTTTAACCTTGCGGCCGTACTCCCCAGGCGGTCGACTTAACGCGTTAG  609
                ||||||||||||||||||||||||||||||||||||||||||||||||||||||||||||
Sbjct  1059739  AATTCATTTGAGTTTTAACCTTGCGGCCGTACTCCCCAGGCGGTCGACTTAACGCGTTAG  1059798

Query  610      CTCCGGAAGCCACGCCTCAAGGGCACAACCTCCAAGTCGACATCGTTTACGGCGTGGACT  669
                ||||||||||||||||||||||||||||||||||||||||||||||||||||||||||||
Sbjct  1059799  CTCCGGAAGCCACGCCTCAAGGGCACAACCTCCAAGTCGACATCGTTTACGGCGTGGACT  1059858

Query  670      ACCAGGGTATCTAATCCTGTTTGCTCCCCACGCTTTCGCACCTGAGCGTCAGTCTTCGTC  729
                ||||||||||||||||||||||||||||||||||||||||||||||||||||||||||||
Sbjct  1059859  ACCAGGGTATCTAATCCTGTTTGCTCCCCACGCTTTCGCACCTGAGCGTCAGTCTTCGTC  1059918

Query  730      CAGGGGGCCGCCTTCGCCACCGGTATTCCTCCAGATCTCTACGCATTTCACCGCTACACC  789
                ||||||||||||||||||||||||||||||||||||||||||||||||||||||||||||
Sbjct  1059919  CAGGGGGCCGCCTTCGCCACCGGTATTCCTCCAGATCTCTACGCATTTCACCGCTACACC  1059978

Query  790      TGGAATTCTACCCCCCTCTACGAGACTCAAGCTTGCCAGTATCAGATGCAGTTCCCAGGT  849
                ||||||||||||||||||||||||||||||||||||||||||||||||||||||||||||
Sbjct  1059979  TGGAATTCTACCCCCCTCTACGAGACTCAAGCTTGCCAGTATCAGATGCAGTTCCCAGGT  1060038

Query  850      TGAGCCCGGGGATTTCACATCTGACTTAACAAACCGCCTGCGTGCGCTTTACGCCCAGTA  909
                ||||||||||||||||||||||||||||||||||||||||||||||||||||||||||||
Sbjct  1060039  TGAGCCCGGGGATTTCACATCTGACTTAACAAACCGCCTGCGTGCGCTTTACGCCCAGTA  1060098

Query  910      ATTCCGATTAACGCTTGCACCCTCCGTATTACCGCGGCTGCTGGCACGGAGTTAGCCGGT  969
                ||||||||||||||||||||||||||||||||||||||||||||||||||||||||||||
Sbjct  1060099  ATTCCGATTAACGCTTGCACCCTCCGTATTACCGCGGCTGCTGGCACGGAGTTAGCCGGT  1060158

Query  970      GCTTCTTCTGCGGGTAACGTCAATGAGCAAAGGNNTTAACTTTACTCCCTTCCTCCCCGC  1029
                |||||||||||||||||||||||||||||||||  |||||||||||||||||||||||||
Sbjct  1060159  GCTTCTTCTGCGGGTAACGTCAATGAGCAAAGGTATTAACTTTACTCCCTTCCTCCCCGC  1060218

Query  1030     TGAAAGTACTTTACAACCCGAAGGCCTTCTTC  1061
                ||||||||||||||||||||||||||||||||
Sbjct  1060219  TGAAAGTACTTTACAACCCGAAGGCCTTCTTC  1060250
```

Range 3: 1574114 to 1575165GenBankGraphics

Next Match
Previous Match
First Match

Alignment statistics for match #3

| Score | Expect | Identities | Gaps | Strand | Frame |
| --- | --- | --- | --- | --- | --- |
| 1929 bits(1044) | 0.0() | 1048/1052(99%) | 0/1052(0%) | Plus/Minus |  |

Features:

```
Query  10       CGCCCTCCCGAAGGTTANGCTACCTACTTCTTTTGCANCCCACTCCCATGGTGTGACGGG  69
                ||||||||||||||||| ||||||||||||||||||| ||||||||||||||||||||||
Sbjct  1575165  CGCCCTCCCGAAGGTTAAGCTACCTACTTCTTTTGCAACCCACTCCCATGGTGTGACGGG  1575106

Query  70       CGGTGTGTACAAGGCCCGGGAACGTATTCACCGTGGCATTCTGATCCACGATTACTAGCG  129
                ||||||||||||||||||||||||||||||||||||||||||||||||||||||||||||
Sbjct  1575105  CGGTGTGTACAAGGCCCGGGAACGTATTCACCGTGGCATTCTGATCCACGATTACTAGCG  1575046

Query  130      ATTCCGACTTCATGGAGTCGAGTTGCAGACTCCAATCCGGACTACGACGCACTTTATGAG  189
                ||||||||||||||||||||||||||||||||||||||||||||||||||||||||||||
Sbjct  1575045  ATTCCGACTTCATGGAGTCGAGTTGCAGACTCCAATCCGGACTACGACGCACTTTATGAG  1574986

Query  190      GTCCGCTTGCTCTCGCGAGGTCGCTTCTCTTTGTATGCGCCATTGTAGCACGTGTGTAGC  249
                ||||||||||||||||||||||||||||||||||||||||||||||||||||||||||||
Sbjct  1574985  GTCCGCTTGCTCTCGCGAGGTCGCTTCTCTTTGTATGCGCCATTGTAGCACGTGTGTAGC  1574926

Query  250      CCTGGTCGTAAGGGCCATGATGACTTGACGTCATCCCCACCTTCCTCCAGTTTATCACTG  309
                ||||||||||||||||||||||||||||||||||||||||||||||||||||||||||||
Sbjct  1574925  CCTGGTCGTAAGGGCCATGATGACTTGACGTCATCCCCACCTTCCTCCAGTTTATCACTG  1574866

Query  310      GCAGTCTCCTTTGAGTTCCCGGCCGGACCGCTGGCAACAAAGGATAAGGGTTGCGCTCGT  369
                ||||||||||||||||||||||||||||||||||||||||||||||||||||||||||||
Sbjct  1574865  GCAGTCTCCTTTGAGTTCCCGGCCGGACCGCTGGCAACAAAGGATAAGGGTTGCGCTCGT  1574806

Query  370      TGCGGGACTTAACCCAACATTTCACAACACGAGCTGACGACAGCCATGCAGCACCTGTCT  429
                ||||||||||||||||||||||||||||||||||||||||||||||||||||||||||||
Sbjct  1574805  TGCGGGACTTAACCCAACATTTCACAACACGAGCTGACGACAGCCATGCAGCACCTGTCT  1574746

Query  430      CACGGTTCCCGAAGGCACATTCTCATCTCTGAAAACTTCCGTGGATGTCAAGACCAGGTA  489
                ||||||||||||||||||||||||||||||||||||||||||||||||||||||||||||
Sbjct  1574745  CACGGTTCCCGAAGGCACATTCTCATCTCTGAAAACTTCCGTGGATGTCAAGACCAGGTA  1574686

Query  490      AGGTTCTTCGCGTTGCATCGAATTAAACCACATGCTCCACCGCTTGTGCGGGCCCCCGTC  549
                ||||||||||||||||||||||||||||||||||||||||||||||||||||||||||||
Sbjct  1574685  AGGTTCTTCGCGTTGCATCGAATTAAACCACATGCTCCACCGCTTGTGCGGGCCCCCGTC  1574626

Query  550      AATTCATTTGAGTTTTAACCTTGCGGCCGTACTCCCCAGGCGGTCGACTTAACGCGTTAG  609
                ||||||||||||||||||||||||||||||||||||||||||||||||||||||||||||
Sbjct  1574625  AATTCATTTGAGTTTTAACCTTGCGGCCGTACTCCCCAGGCGGTCGACTTAACGCGTTAG  1574566

Query  610      CTCCGGAAGCCACGCCTCAAGGGCACAACCTCCAAGTCGACATCGTTTACGGCGTGGACT  669
                ||||||||||||||||||||||||||||||||||||||||||||||||||||||||||||
Sbjct  1574565  CTCCGGAAGCCACGCCTCAAGGGCACAACCTCCAAGTCGACATCGTTTACGGCGTGGACT  1574506

Query  670      ACCAGGGTATCTAATCCTGTTTGCTCCCCACGCTTTCGCACCTGAGCGTCAGTCTTCGTC  729
                ||||||||||||||||||||||||||||||||||||||||||||||||||||||||||||
Sbjct  1574505  ACCAGGGTATCTAATCCTGTTTGCTCCCCACGCTTTCGCACCTGAGCGTCAGTCTTCGTC  1574446

Query  730      CAGGGGGCCGCCTTCGCCACCGGTATTCCTCCAGATCTCTACGCATTTCACCGCTACACC  789
                ||||||||||||||||||||||||||||||||||||||||||||||||||||||||||||
Sbjct  1574445  CAGGGGGCCGCCTTCGCCACCGGTATTCCTCCAGATCTCTACGCATTTCACCGCTACACC  1574386

Query  790      TGGAATTCTACCCCCCTCTACGAGACTCAAGCTTGCCAGTATCAGATGCAGTTCCCAGGT  849
                ||||||||||||||||||||||||||||||||||||||||||||||||||||||||||||
Sbjct  1574385  TGGAATTCTACCCCCCTCTACGAGACTCAAGCTTGCCAGTATCAGATGCAGTTCCCAGGT  1574326

Query  850      TGAGCCCGGGGATTTCACATCTGACTTAACAAACCGCCTGCGTGCGCTTTACGCCCAGTA  909
                ||||||||||||||||||||||||||||||||||||||||||||||||||||||||||||
Sbjct  1574325  TGAGCCCGGGGATTTCACATCTGACTTAACAAACCGCCTGCGTGCGCTTTACGCCCAGTA  1574266

Query  910      ATTCCGATTAACGCTTGCACCCTCCGTATTACCGCGGCTGCTGGCACGGAGTTAGCCGGT  969
                ||||||||||||||||||||||||||||||||||||||||||||||||||||||||||||
Sbjct  1574265  ATTCCGATTAACGCTTGCACCCTCCGTATTACCGCGGCTGCTGGCACGGAGTTAGCCGGT  1574206

Query  970      GCTTCTTCTGCGGGTAACGTCAATGAGCAAAGGNNTTAACTTTACTCCCTTCCTCCCCGC  1029
                |||||||||||||||||||||||||||||||||  |||||||||||||||||||||||||
Sbjct  1574205  GCTTCTTCTGCGGGTAACGTCAATGAGCAAAGGTATTAACTTTACTCCCTTCCTCCCCGC  1574146

Query  1030     TGAAAGTACTTTACAACCCGAAGGCCTTCTTC  1061
                ||||||||||||||||||||||||||||||||
Sbjct  1574145  TGAAAGTACTTTACAACCCGAAGGCCTTCTTC  1574114
```

Range 4: 1667837 to 1668888GenBankGraphics

Next Match
Previous Match
First Match

Alignment statistics for match #4

| Score | Expect | Identities | Gaps | Strand | Frame |
| --- | --- | --- | --- | --- | --- |
| 1929 bits(1044) | 0.0() | 1048/1052(99%) | 0/1052(0%) | Plus/Minus |  |

Features:

```
Query  10       CGCCCTCCCGAAGGTTANGCTACCTACTTCTTTTGCANCCCACTCCCATGGTGTGACGGG  69
                ||||||||||||||||| ||||||||||||||||||| ||||||||||||||||||||||
Sbjct  1668888  CGCCCTCCCGAAGGTTAAGCTACCTACTTCTTTTGCAACCCACTCCCATGGTGTGACGGG  1668829

Query  70       CGGTGTGTACAAGGCCCGGGAACGTATTCACCGTGGCATTCTGATCCACGATTACTAGCG  129
                ||||||||||||||||||||||||||||||||||||||||||||||||||||||||||||
Sbjct  1668828  CGGTGTGTACAAGGCCCGGGAACGTATTCACCGTGGCATTCTGATCCACGATTACTAGCG  1668769

Query  130      ATTCCGACTTCATGGAGTCGAGTTGCAGACTCCAATCCGGACTACGACGCACTTTATGAG  189
                ||||||||||||||||||||||||||||||||||||||||||||||||||||||||||||
Sbjct  1668768  ATTCCGACTTCATGGAGTCGAGTTGCAGACTCCAATCCGGACTACGACGCACTTTATGAG  1668709

Query  190      GTCCGCTTGCTCTCGCGAGGTCGCTTCTCTTTGTATGCGCCATTGTAGCACGTGTGTAGC  249
                ||||||||||||||||||||||||||||||||||||||||||||||||||||||||||||
Sbjct  1668708  GTCCGCTTGCTCTCGCGAGGTCGCTTCTCTTTGTATGCGCCATTGTAGCACGTGTGTAGC  1668649

Query  250      CCTGGTCGTAAGGGCCATGATGACTTGACGTCATCCCCACCTTCCTCCAGTTTATCACTG  309
                ||||||||||||||||||||||||||||||||||||||||||||||||||||||||||||
Sbjct  1668648  CCTGGTCGTAAGGGCCATGATGACTTGACGTCATCCCCACCTTCCTCCAGTTTATCACTG  1668589

Query  310      GCAGTCTCCTTTGAGTTCCCGGCCGGACCGCTGGCAACAAAGGATAAGGGTTGCGCTCGT  369
                ||||||||||||||||||||||||||||||||||||||||||||||||||||||||||||
Sbjct  1668588  GCAGTCTCCTTTGAGTTCCCGGCCGGACCGCTGGCAACAAAGGATAAGGGTTGCGCTCGT  1668529

Query  370      TGCGGGACTTAACCCAACATTTCACAACACGAGCTGACGACAGCCATGCAGCACCTGTCT  429
                ||||||||||||||||||||||||||||||||||||||||||||||||||||||||||||
Sbjct  1668528  TGCGGGACTTAACCCAACATTTCACAACACGAGCTGACGACAGCCATGCAGCACCTGTCT  1668469

Query  430      CACGGTTCCCGAAGGCACATTCTCATCTCTGAAAACTTCCGTGGATGTCAAGACCAGGTA  489
                ||||||||||||||||||||||||||||||||||||||||||||||||||||||||||||
Sbjct  1668468  CACGGTTCCCGAAGGCACATTCTCATCTCTGAAAACTTCCGTGGATGTCAAGACCAGGTA  1668409

Query  490      AGGTTCTTCGCGTTGCATCGAATTAAACCACATGCTCCACCGCTTGTGCGGGCCCCCGTC  549
                ||||||||||||||||||||||||||||||||||||||||||||||||||||||||||||
Sbjct  1668408  AGGTTCTTCGCGTTGCATCGAATTAAACCACATGCTCCACCGCTTGTGCGGGCCCCCGTC  1668349

Query  550      AATTCATTTGAGTTTTAACCTTGCGGCCGTACTCCCCAGGCGGTCGACTTAACGCGTTAG  609
                ||||||||||||||||||||||||||||||||||||||||||||||||||||||||||||
Sbjct  1668348  AATTCATTTGAGTTTTAACCTTGCGGCCGTACTCCCCAGGCGGTCGACTTAACGCGTTAG  1668289

Query  610      CTCCGGAAGCCACGCCTCAAGGGCACAACCTCCAAGTCGACATCGTTTACGGCGTGGACT  669
                ||||||||||||||||||||||||||||||||||||||||||||||||||||||||||||
Sbjct  1668288  CTCCGGAAGCCACGCCTCAAGGGCACAACCTCCAAGTCGACATCGTTTACGGCGTGGACT  1668229

Query  670      ACCAGGGTATCTAATCCTGTTTGCTCCCCACGCTTTCGCACCTGAGCGTCAGTCTTCGTC  729
                ||||||||||||||||||||||||||||||||||||||||||||||||||||||||||||
Sbjct  1668228  ACCAGGGTATCTAATCCTGTTTGCTCCCCACGCTTTCGCACCTGAGCGTCAGTCTTCGTC  1668169

Query  730      CAGGGGGCCGCCTTCGCCACCGGTATTCCTCCAGATCTCTACGCATTTCACCGCTACACC  789
                ||||||||||||||||||||||||||||||||||||||||||||||||||||||||||||
Sbjct  1668168  CAGGGGGCCGCCTTCGCCACCGGTATTCCTCCAGATCTCTACGCATTTCACCGCTACACC  1668109

Query  790      TGGAATTCTACCCCCCTCTACGAGACTCAAGCTTGCCAGTATCAGATGCAGTTCCCAGGT  849
                ||||||||||||||||||||||||||||||||||||||||||||||||||||||||||||
Sbjct  1668108  TGGAATTCTACCCCCCTCTACGAGACTCAAGCTTGCCAGTATCAGATGCAGTTCCCAGGT  1668049

Query  850      TGAGCCCGGGGATTTCACATCTGACTTAACAAACCGCCTGCGTGCGCTTTACGCCCAGTA  909
                ||||||||||||||||||||||||||||||||||||||||||||||||||||||||||||
Sbjct  1668048  TGAGCCCGGGGATTTCACATCTGACTTAACAAACCGCCTGCGTGCGCTTTACGCCCAGTA  1667989

Query  910      ATTCCGATTAACGCTTGCACCCTCCGTATTACCGCGGCTGCTGGCACGGAGTTAGCCGGT  969
                ||||||||||||||||||||||||||||||||||||||||||||||||||||||||||||
Sbjct  1667988  ATTCCGATTAACGCTTGCACCCTCCGTATTACCGCGGCTGCTGGCACGGAGTTAGCCGGT  1667929

Query  970      GCTTCTTCTGCGGGTAACGTCAATGAGCAAAGGNNTTAACTTTACTCCCTTCCTCCCCGC  1029
                |||||||||||||||||||||||||||||||||  |||||||||||||||||||||||||
Sbjct  1667928  GCTTCTTCTGCGGGTAACGTCAATGAGCAAAGGTATTAACTTTACTCCCTTCCTCCCCGC  1667869

Query  1030     TGAAAGTACTTTACAACCCGAAGGCCTTCTTC  1061
                ||||||||||||||||||||||||||||||||
Sbjct  1667868  TGAAAGTACTTTACAACCCGAAGGCCTTCTTC  1667837
```

Range 5: 1798965 to 1800016GenBankGraphics

Next Match
Previous Match
First Match

Alignment statistics for match #5

| Score | Expect | Identities | Gaps | Strand | Frame |
| --- | --- | --- | --- | --- | --- |
| 1929 bits(1044) | 0.0() | 1048/1052(99%) | 0/1052(0%) | Plus/Minus |  |

Features:

```
Query  10       CGCCCTCCCGAAGGTTANGCTACCTACTTCTTTTGCANCCCACTCCCATGGTGTGACGGG  69
                ||||||||||||||||| ||||||||||||||||||| ||||||||||||||||||||||
Sbjct  1800016  CGCCCTCCCGAAGGTTAAGCTACCTACTTCTTTTGCAACCCACTCCCATGGTGTGACGGG  1799957

Query  70       CGGTGTGTACAAGGCCCGGGAACGTATTCACCGTGGCATTCTGATCCACGATTACTAGCG  129
                ||||||||||||||||||||||||||||||||||||||||||||||||||||||||||||
Sbjct  1799956  CGGTGTGTACAAGGCCCGGGAACGTATTCACCGTGGCATTCTGATCCACGATTACTAGCG  1799897

Query  130      ATTCCGACTTCATGGAGTCGAGTTGCAGACTCCAATCCGGACTACGACGCACTTTATGAG  189
                ||||||||||||||||||||||||||||||||||||||||||||||||||||||||||||
Sbjct  1799896  ATTCCGACTTCATGGAGTCGAGTTGCAGACTCCAATCCGGACTACGACGCACTTTATGAG  1799837

Query  190      GTCCGCTTGCTCTCGCGAGGTCGCTTCTCTTTGTATGCGCCATTGTAGCACGTGTGTAGC  249
                ||||||||||||||||||||||||||||||||||||||||||||||||||||||||||||
Sbjct  1799836  GTCCGCTTGCTCTCGCGAGGTCGCTTCTCTTTGTATGCGCCATTGTAGCACGTGTGTAGC  1799777

Query  250      CCTGGTCGTAAGGGCCATGATGACTTGACGTCATCCCCACCTTCCTCCAGTTTATCACTG  309
                ||||||||||||||||||||||||||||||||||||||||||||||||||||||||||||
Sbjct  1799776  CCTGGTCGTAAGGGCCATGATGACTTGACGTCATCCCCACCTTCCTCCAGTTTATCACTG  1799717

Query  310      GCAGTCTCCTTTGAGTTCCCGGCCGGACCGCTGGCAACAAAGGATAAGGGTTGCGCTCGT  369
                ||||||||||||||||||||||||||||||||||||||||||||||||||||||||||||
Sbjct  1799716  GCAGTCTCCTTTGAGTTCCCGGCCGGACCGCTGGCAACAAAGGATAAGGGTTGCGCTCGT  1799657

Query  370      TGCGGGACTTAACCCAACATTTCACAACACGAGCTGACGACAGCCATGCAGCACCTGTCT  429
                ||||||||||||||||||||||||||||||||||||||||||||||||||||||||||||
Sbjct  1799656  TGCGGGACTTAACCCAACATTTCACAACACGAGCTGACGACAGCCATGCAGCACCTGTCT  1799597

Query  430      CACGGTTCCCGAAGGCACATTCTCATCTCTGAAAACTTCCGTGGATGTCAAGACCAGGTA  489
                ||||||||||||||||||||||||||||||||||||||||||||||||||||||||||||
Sbjct  1799596  CACGGTTCCCGAAGGCACATTCTCATCTCTGAAAACTTCCGTGGATGTCAAGACCAGGTA  1799537

Query  490      AGGTTCTTCGCGTTGCATCGAATTAAACCACATGCTCCACCGCTTGTGCGGGCCCCCGTC  549
                ||||||||||||||||||||||||||||||||||||||||||||||||||||||||||||
Sbjct  1799536  AGGTTCTTCGCGTTGCATCGAATTAAACCACATGCTCCACCGCTTGTGCGGGCCCCCGTC  1799477

Query  550      AATTCATTTGAGTTTTAACCTTGCGGCCGTACTCCCCAGGCGGTCGACTTAACGCGTTAG  609
                ||||||||||||||||||||||||||||||||||||||||||||||||||||||||||||
Sbjct  1799476  AATTCATTTGAGTTTTAACCTTGCGGCCGTACTCCCCAGGCGGTCGACTTAACGCGTTAG  1799417

Query  610      CTCCGGAAGCCACGCCTCAAGGGCACAACCTCCAAGTCGACATCGTTTACGGCGTGGACT  669
                ||||||||||||||||||||||||||||||||||||||||||||||||||||||||||||
Sbjct  1799416  CTCCGGAAGCCACGCCTCAAGGGCACAACCTCCAAGTCGACATCGTTTACGGCGTGGACT  1799357

Query  670      ACCAGGGTATCTAATCCTGTTTGCTCCCCACGCTTTCGCACCTGAGCGTCAGTCTTCGTC  729
                ||||||||||||||||||||||||||||||||||||||||||||||||||||||||||||
Sbjct  1799356  ACCAGGGTATCTAATCCTGTTTGCTCCCCACGCTTTCGCACCTGAGCGTCAGTCTTCGTC  1799297

Query  730      CAGGGGGCCGCCTTCGCCACCGGTATTCCTCCAGATCTCTACGCATTTCACCGCTACACC  789
                ||||||||||||||||||||||||||||||||||||||||||||||||||||||||||||
Sbjct  1799296  CAGGGGGCCGCCTTCGCCACCGGTATTCCTCCAGATCTCTACGCATTTCACCGCTACACC  1799237

Query  790      TGGAATTCTACCCCCCTCTACGAGACTCAAGCTTGCCAGTATCAGATGCAGTTCCCAGGT  849
                ||||||||||||||||||||||||||||||||||||||||||||||||||||||||||||
Sbjct  1799236  TGGAATTCTACCCCCCTCTACGAGACTCAAGCTTGCCAGTATCAGATGCAGTTCCCAGGT  1799177

Query  850      TGAGCCCGGGGATTTCACATCTGACTTAACAAACCGCCTGCGTGCGCTTTACGCCCAGTA  909
                ||||||||||||||||||||||||||||||||||||||||||||||||||||||||||||
Sbjct  1799176  TGAGCCCGGGGATTTCACATCTGACTTAACAAACCGCCTGCGTGCGCTTTACGCCCAGTA  1799117

Query  910      ATTCCGATTAACGCTTGCACCCTCCGTATTACCGCGGCTGCTGGCACGGAGTTAGCCGGT  969
                ||||||||||||||||||||||||||||||||||||||||||||||||||||||||||||
Sbjct  1799116  ATTCCGATTAACGCTTGCACCCTCCGTATTACCGCGGCTGCTGGCACGGAGTTAGCCGGT  1799057

Query  970      GCTTCTTCTGCGGGTAACGTCAATGAGCAAAGGNNTTAACTTTACTCCCTTCCTCCCCGC  1029
                |||||||||||||||||||||||||||||||||  |||||||||||||||||||||||||
Sbjct  1799056  GCTTCTTCTGCGGGTAACGTCAATGAGCAAAGGTATTAACTTTACTCCCTTCCTCCCCGC  1798997

Query  1030     TGAAAGTACTTTACAACCCGAAGGCCTTCTTC  1061
                ||||||||||||||||||||||||||||||||
Sbjct  1798996  TGAAAGTACTTTACAACCCGAAGGCCTTCTTC  1798965
```

Range 6: 1840453 to 1841504GenBankGraphics

Next Match
Previous Match
First Match

Alignment statistics for match #6

| Score | Expect | Identities | Gaps | Strand | Frame |
| --- | --- | --- | --- | --- | --- |
| 1929 bits(1044) | 0.0() | 1048/1052(99%) | 0/1052(0%) | Plus/Minus |  |

Features:

```
Query  10       CGCCCTCCCGAAGGTTANGCTACCTACTTCTTTTGCANCCCACTCCCATGGTGTGACGGG  69
                ||||||||||||||||| ||||||||||||||||||| ||||||||||||||||||||||
Sbjct  1841504  CGCCCTCCCGAAGGTTAAGCTACCTACTTCTTTTGCAACCCACTCCCATGGTGTGACGGG  1841445

Query  70       CGGTGTGTACAAGGCCCGGGAACGTATTCACCGTGGCATTCTGATCCACGATTACTAGCG  129
                ||||||||||||||||||||||||||||||||||||||||||||||||||||||||||||
Sbjct  1841444  CGGTGTGTACAAGGCCCGGGAACGTATTCACCGTGGCATTCTGATCCACGATTACTAGCG  1841385

Query  130      ATTCCGACTTCATGGAGTCGAGTTGCAGACTCCAATCCGGACTACGACGCACTTTATGAG  189
                ||||||||||||||||||||||||||||||||||||||||||||||||||||||||||||
Sbjct  1841384  ATTCCGACTTCATGGAGTCGAGTTGCAGACTCCAATCCGGACTACGACGCACTTTATGAG  1841325

Query  190      GTCCGCTTGCTCTCGCGAGGTCGCTTCTCTTTGTATGCGCCATTGTAGCACGTGTGTAGC  249
                ||||||||||||||||||||||||||||||||||||||||||||||||||||||||||||
Sbjct  1841324  GTCCGCTTGCTCTCGCGAGGTCGCTTCTCTTTGTATGCGCCATTGTAGCACGTGTGTAGC  1841265

Query  250      CCTGGTCGTAAGGGCCATGATGACTTGACGTCATCCCCACCTTCCTCCAGTTTATCACTG  309
                ||||||||||||||||||||||||||||||||||||||||||||||||||||||||||||
Sbjct  1841264  CCTGGTCGTAAGGGCCATGATGACTTGACGTCATCCCCACCTTCCTCCAGTTTATCACTG  1841205

Query  310      GCAGTCTCCTTTGAGTTCCCGGCCGGACCGCTGGCAACAAAGGATAAGGGTTGCGCTCGT  369
                ||||||||||||||||||||||||||||||||||||||||||||||||||||||||||||
Sbjct  1841204  GCAGTCTCCTTTGAGTTCCCGGCCGGACCGCTGGCAACAAAGGATAAGGGTTGCGCTCGT  1841145

Query  370      TGCGGGACTTAACCCAACATTTCACAACACGAGCTGACGACAGCCATGCAGCACCTGTCT  429
                ||||||||||||||||||||||||||||||||||||||||||||||||||||||||||||
Sbjct  1841144  TGCGGGACTTAACCCAACATTTCACAACACGAGCTGACGACAGCCATGCAGCACCTGTCT  1841085

Query  430      CACGGTTCCCGAAGGCACATTCTCATCTCTGAAAACTTCCGTGGATGTCAAGACCAGGTA  489
                ||||||||||||||||||||||||||||||||||||||||||||||||||||||||||||
Sbjct  1841084  CACGGTTCCCGAAGGCACATTCTCATCTCTGAAAACTTCCGTGGATGTCAAGACCAGGTA  1841025

Query  490      AGGTTCTTCGCGTTGCATCGAATTAAACCACATGCTCCACCGCTTGTGCGGGCCCCCGTC  549
                ||||||||||||||||||||||||||||||||||||||||||||||||||||||||||||
Sbjct  1841024  AGGTTCTTCGCGTTGCATCGAATTAAACCACATGCTCCACCGCTTGTGCGGGCCCCCGTC  1840965

Query  550      AATTCATTTGAGTTTTAACCTTGCGGCCGTACTCCCCAGGCGGTCGACTTAACGCGTTAG  609
                ||||||||||||||||||||||||||||||||||||||||||||||||||||||||||||
Sbjct  1840964  AATTCATTTGAGTTTTAACCTTGCGGCCGTACTCCCCAGGCGGTCGACTTAACGCGTTAG  1840905

Query  610      CTCCGGAAGCCACGCCTCAAGGGCACAACCTCCAAGTCGACATCGTTTACGGCGTGGACT  669
                ||||||||||||||||||||||||||||||||||||||||||||||||||||||||||||
Sbjct  1840904  CTCCGGAAGCCACGCCTCAAGGGCACAACCTCCAAGTCGACATCGTTTACGGCGTGGACT  1840845

Query  670      ACCAGGGTATCTAATCCTGTTTGCTCCCCACGCTTTCGCACCTGAGCGTCAGTCTTCGTC  729
                ||||||||||||||||||||||||||||||||||||||||||||||||||||||||||||
Sbjct  1840844  ACCAGGGTATCTAATCCTGTTTGCTCCCCACGCTTTCGCACCTGAGCGTCAGTCTTCGTC  1840785

Query  730      CAGGGGGCCGCCTTCGCCACCGGTATTCCTCCAGATCTCTACGCATTTCACCGCTACACC  789
                ||||||||||||||||||||||||||||||||||||||||||||||||||||||||||||
Sbjct  1840784  CAGGGGGCCGCCTTCGCCACCGGTATTCCTCCAGATCTCTACGCATTTCACCGCTACACC  1840725

Query  790      TGGAATTCTACCCCCCTCTACGAGACTCAAGCTTGCCAGTATCAGATGCAGTTCCCAGGT  849
                ||||||||||||||||||||||||||||||||||||||||||||||||||||||||||||
Sbjct  1840724  TGGAATTCTACCCCCCTCTACGAGACTCAAGCTTGCCAGTATCAGATGCAGTTCCCAGGT  1840665

Query  850      TGAGCCCGGGGATTTCACATCTGACTTAACAAACCGCCTGCGTGCGCTTTACGCCCAGTA  909
                ||||||||||||||||||||||||||||||||||||||||||||||||||||||||||||
Sbjct  1840664  TGAGCCCGGGGATTTCACATCTGACTTAACAAACCGCCTGCGTGCGCTTTACGCCCAGTA  1840605

Query  910      ATTCCGATTAACGCTTGCACCCTCCGTATTACCGCGGCTGCTGGCACGGAGTTAGCCGGT  969
                ||||||||||||||||||||||||||||||||||||||||||||||||||||||||||||
Sbjct  1840604  ATTCCGATTAACGCTTGCACCCTCCGTATTACCGCGGCTGCTGGCACGGAGTTAGCCGGT  1840545

Query  970      GCTTCTTCTGCGGGTAACGTCAATGAGCAAAGGNNTTAACTTTACTCCCTTCCTCCCCGC  1029
                |||||||||||||||||||||||||||||||||  |||||||||||||||||||||||||
Sbjct  1840544  GCTTCTTCTGCGGGTAACGTCAATGAGCAAAGGTATTAACTTTACTCCCTTCCTCCCCGC  1840485

Query  1030     TGAAAGTACTTTACAACCCGAAGGCCTTCTTC  1061
                ||||||||||||||||||||||||||||||||
Sbjct  1840484  TGAAAGTACTTTACAACCCGAAGGCCTTCTTC  1840453
```

Range 7: 2497618 to 2498669GenBankGraphics

Next Match
Previous Match
First Match

Alignment statistics for match #7

| Score | Expect | Identities | Gaps | Strand | Frame |
| --- | --- | --- | --- | --- | --- |
| 1884 bits(1020) | 0.0() | 1041/1053(99%) | 2/1053(0%) | Plus/Minus |  |

Features:

```
Query  10       CGCCCTCCCGAAGGTTANGCTACCTACTTCTTTTGCANCCCACTCCCATGGTGTGACGGG  69
                ||||||||||||||||| ||||||||||||||||||| ||||||||||||||||||||||
Sbjct  2498669  CGCCCTCCCGAAGGTTAAGCTACCTACTTCTTTTGCAACCCACTCCCATGGTGTGACGGG  2498610

Query  70       CGGTGTGTACAAGGCCCGGGAACGTATTCACCGTGGCATTCTGATCCACGATTACTAGCG  129
                ||||||||||||||||||||||||||||||||||||||||||||||||||||||||||||
Sbjct  2498609  CGGTGTGTACAAGGCCCGGGAACGTATTCACCGTGGCATTCTGATCCACGATTACTAGCG  2498550

Query  130      ATTCCGACTTCATGGAGTCGAGTTGCAGACTCCAATCCGGACTACGACGCACTTTATGAG  189
                ||||||||||||||||||||||||||||||||||||||||||||||||||||||||||||
Sbjct  2498549  ATTCCGACTTCATGGAGTCGAGTTGCAGACTCCAATCCGGACTACGACGCACTTTATGAG  2498490

Query  190      GTCCGCTTGCTCTCGCGAGGTCGCTTCTCTTTGTATGCGCCATTGTAGCACGTGTGTAGC  249
                ||||||||||||||||||||||||||||||||||||||||||||||||||||||||||||
Sbjct  2498489  GTCCGCTTGCTCTCGCGAGGTCGCTTCTCTTTGTATGCGCCATTGTAGCACGTGTGTAGC  2498430

Query  250      CCTGGTCGTAAGGGCCATGATGACTTGACGTCATCCCCACCTTCCTCCAGTTTATCACTG  309
                ||||||||||||||||||||||||||||||||||||||||||||||||||||||||||||
Sbjct  2498429  CCTGGTCGTAAGGGCCATGATGACTTGACGTCATCCCCACCTTCCTCCAGTTTATCACTG  2498370

Query  310      GCAGTCTCCTTTGAGTTCCCGGCCGGACCGCTGGCAACAAAGGATAAGGGTTGCGCTCGT  369
                ||||||||||||||||||||||||||||||||||||||||| ||||||||||||||||||
Sbjct  2498369  GCAGTCTCCTTTGAGTTCCCGGCCGGACCGCTGGCAACAAAAGATAAGGGTTGCGCTCGT  2498310

Query  370      TGCGGGACTTAACCCAACATTTCACAACACGAGCTGACGACAGCCATGCAGCACCTGTCT  429
                ||||||||||||||||||||||||||||||||||||||||||||||||||||||||||||
Sbjct  2498309  TGCGGGACTTAACCCAACATTTCACAACACGAGCTGACGACAGCCATGCAGCACCTGTCT  2498250

Query  430      CACGGTTCCCGAAGGCAC-ATTCTCATCTCTGAAAACTTCCGTGGATGTCAAGACCAGGT  488
                ||| |||||||||||||| | || |||||||| ||| ||| |||||||||||||||||||
Sbjct  2498249  CACAGTTCCCGAAGGCACCAATC-CATCTCTGGAAAGTTCTGTGGATGTCAAGACCAGGT  2498191

Query  489      AAGGTTCTTCGCGTTGCATCGAATTAAACCACATGCTCCACCGCTTGTGCGGGCCCCCGT  548
                ||||||||||||||||||||||||||||||||||||||||||||||||||||||||||||
Sbjct  2498190  AAGGTTCTTCGCGTTGCATCGAATTAAACCACATGCTCCACCGCTTGTGCGGGCCCCCGT  2498131

Query  549      CAATTCATTTGAGTTTTAACCTTGCGGCCGTACTCCCCAGGCGGTCGACTTAACGCGTTA  608
                ||||||||||||||||||||||||||||||||||||||||||||||||||||||||||||
Sbjct  2498130  CAATTCATTTGAGTTTTAACCTTGCGGCCGTACTCCCCAGGCGGTCGACTTAACGCGTTA  2498071

Query  609      GCTCCGGAAGCCACGCCTCAAGGGCACAACCTCCAAGTCGACATCGTTTACGGCGTGGAC  668
                ||||||||||||||||||||||||||||||||||||||||||||||||||||||||||||
Sbjct  2498070  GCTCCGGAAGCCACGCCTCAAGGGCACAACCTCCAAGTCGACATCGTTTACGGCGTGGAC  2498011

Query  669      TACCAGGGTATCTAATCCTGTTTGCTCCCCACGCTTTCGCACCTGAGCGTCAGTCTTCGT  728
                ||||||||||||||||||||||||||||||||||||||||||||||||||||||||||||
Sbjct  2498010  TACCAGGGTATCTAATCCTGTTTGCTCCCCACGCTTTCGCACCTGAGCGTCAGTCTTCGT  2497951

Query  729      CCAGGGGGCCGCCTTCGCCACCGGTATTCCTCCAGATCTCTACGCATTTCACCGCTACAC  788
                ||||||||||||||||||||||||||||||||||||||||||||||||||||||||||||
Sbjct  2497950  CCAGGGGGCCGCCTTCGCCACCGGTATTCCTCCAGATCTCTACGCATTTCACCGCTACAC  2497891

Query  789      CTGGAATTCTACCCCCCTCTACGAGACTCAAGCTTGCCAGTATCAGATGCAGTTCCCAGG  848
                ||||||||||||||||||||||||||||||||||||||||||||||||||||||||||||
Sbjct  2497890  CTGGAATTCTACCCCCCTCTACGAGACTCAAGCTTGCCAGTATCAGATGCAGTTCCCAGG  2497831

Query  849      TTGAGCCCGGGGATTTCACATCTGACTTAACAAACCGCCTGCGTGCGCTTTACGCCCAGT  908
                ||||||||||||||||||||||||||||||||||||||||||||||||||||||||||||
Sbjct  2497830  TTGAGCCCGGGGATTTCACATCTGACTTAACAAACCGCCTGCGTGCGCTTTACGCCCAGT  2497771

Query  909      AATTCCGATTAACGCTTGCACCCTCCGTATTACCGCGGCTGCTGGCACGGAGTTAGCCGG  968
                ||||||||||||||||||||||||||||||||||||||||||||||||||||||||||||
Sbjct  2497770  AATTCCGATTAACGCTTGCACCCTCCGTATTACCGCGGCTGCTGGCACGGAGTTAGCCGG  2497711

Query  969      TGCTTCTTCTGCGGGTAACGTCAATGAGCAAAGGNNTTAACTTTACTCCCTTCCTCCCCG  1028
                ||||||||||||||||||||||||||||||||||  ||||||||||||||||||||||||
Sbjct  2497710  TGCTTCTTCTGCGGGTAACGTCAATGAGCAAAGGTATTAACTTTACTCCCTTCCTCCCCG  2497651

Query  1029     CTGAAAGTACTTTACAACCCGAAGGCCTTCTTC  1061
                |||||||||||||||||||||||||||||||||
Sbjct  2497650  CTGAAAGTACTTTACAACCCGAAGGCCTTCTTC  2497618
```


BLAST is a registered trademark of the National Library of Medicine

Support center
Mailing list


YouTube

- National Library Of Medicine
- National Institutes Of Health
- U.S. Department of Health & Human Services
- USA.gov

### NCBI


National Center for Biotechnology Information,
 U.S. National Library of Medicine

8600 Rockville Pike,
Bethesda
 MD,
20894
USA

Policies and Guidelines
|
Contact


PreferencesTurn off

External link. Please review our privacy policy.
