# Supplementary material for: Isothermal microcalorimetry for thermal viable count of microorganisms in pure cultures and stabilized formulations
Source: BMC Microbiol. 2019 Mar 21;19:65. doi: 10.1186/s12866-019-1432-8 (PMC6429831; doi:10.1186/s12866-019-1432-8)
Supplement: Supplementary file 3 — 16S rRNA gene sequences’ BLASTN hits in zipped HTML format. (ZIP 15810 kb) [file 12866_2019_1432_MOESM3_ESM.zip › Best blastn hits/NCBI Blast_F pink F -- 15..1016 of sequence (1002.html]

NCBI Blast:F pink F -- 15..1016 of sequence (1002


- NCBI Home
- Sign in to NCBI
- Skip to Main Content
- Skip to Navigation
- About NCBI Accesskeys

U.S. National Library of Medicine

NCBI
National Center for Biotechnology Information

- My NCBI
- Sign in to NCBI
- Register
- Sign Out

BLAST ^®^ » blastn suite » RID-A4XT5JYR014


- Home
- Recent Results
- Saved Strategies
- Help

BLAST Results


Edit and Resubmit
Save Search Strategies
[Sign in above to save your search strategy]

Formatting options 


Download


How to read this page
Blast report description
Questions/comments


|  |  |
| --- | --- |
| Formatting options | |
| Show | Alignment as  HTML Plain text   Old View Reset form to defaults [?]  These options control formatting of alignments in results pages. The default is HTML, but other formats (including plain text) are available. PSSM and PssmWithParameters are representations of Position Specific Scoring Matrices and are only available for PSI-BLAST. The Advanced view option allows the database descriptions to be sorted by various indices in a table. |
| Alignment View | Pairwise Pairwise with dots for identities Query-anchored with dots for identities Query-anchored with letters for identities Flat query-anchored with dots for identities Flat query-anchored with letters for identities [?]  Choose how to view alignments. The default "pairwise" view shows how each subject sequence aligns individually to the query sequence. The "query-anchored" view shows how all subject sequences align to the query sequence. For each view type, you can choose to show "identities" (matching residues) as letters or dots. more... |
| Display | Graphical Overview   Linkout   Sequence Retrieval  NCBI-gi   CDS feature [?]  - Graphical Overview: Graphical Overview: Show graph of similar sequence regions aligned to query.   more... - NCBI-gi: Show NCBI gi identifiers. - CDS feature: Show annotated coding region and translation.   more... |
| Masking | Character:   X for protein, n for nucleotide Lower Case  Color:  Black Grey Red [?]  - Masking Character: Display masked (filtered) sequence regions as lower-case or as specific letters (N for nucleotide, P for protein). - Masking Color: Display masked sequence regions in the given color. |
| Limit results | Descriptions:  10 50 100 Graphical overview:  0 10 50 100  Alignments:  0 10 50 100 Line length:  60 90 120 150 [?]  - Descriptions: Show short descriptions for up to the given number of sequences. - Alignments: Show alignments for up to the given number of sequences, in order of statistical significance. - Line lenghth: Number of letters to show on one line in an alignment. |
|  | Organism Type common name, binomial, taxid, or group name. Only 20 top taxa will be shown.     Exclude   [?]  Show only sequences from the given organism. |
|  | Entrez query:  [?]  Show only those sequences that match the given Entrez query. more... |
|  | Expect Min:  Expect Max:  [?]  Show only sequences with expect values in the given range. more... |
|  | Percent Identity Min:  Percent Identity Max:  [?]  Show only sequences with percent identity values in the given range. |
| Format for | PSI-BLAST with inclusion threshold:  [?]  - Format for PSI-BLAST: The Position-Specific Iterated BLAST (PSI-BLAST) program performs iterative searches with a protein query,   in which sequences found in one round of search are used to build a custom score model for the next round.   more... - Inclusion Threshold: This sets the statistical significance threshold for including a sequence in the model used   by PSI-BLAST to create the PSSM on the next iteration. |

|  |  |  |  |  |  |
| --- | --- | --- | --- | --- | --- |
| Download | | | | | |
| Alignment  Text XML ASN.1 JSON Seq-align Hit Table(text) Hit Table(csv) Multiple-file XML2 Single-file XML2 Multiple-file JSON Single-file JSON SAM | Search Strategies  ASN.1 | PSSM to restart search  PSSM | [?] |  |  |

The Download link provides BLAST output that may be used as input to another program.
This includes parseable formats such as the tabular report or XML as well as the Search Strategy files read by the BLAST+ applications.
More details on the parseable (XML, tabular, and ASN.1) reports can be found at
https://www.ncbi.nlm.nih.gov/books/NBK153387/  
  

The following formats are offered under the Alignment section:  
1). "Text". Non-HTML standard BLAST report.  
2). "XML". XML report based upon the DTD at https://www.ncbi.nlm.nih.gov/data\_specs/dtd/NCBI\_BlastOutput.dtd  
3). "ASN.1". Alignment written out in Abstract Syntax Notation 1.  
4). "JSON Seq-align". Alignment written out in JSON.  
4). "Hit Table(text)". The tabular report as text.  
5). "Hit Table(csv)". The tabular report ready for import into spread-sheet programs like Excel.  
6). "XML2". New XML format described at ftp://ftp.ncbi.nlm.nih.gov/blast/documents/NEWXML/xml2.pdf.  
7). "JSON". New JSON format described at ftp://ftp.ncbi.nlm.nih.gov/blast/documents/NEWXML/xml2.pdf.  
8). "SAM". Sequence Alignment Map format.

XML2 and JSON can be downloaded either as one file per query (multiple-file) or one file for all queries (single-file). These formats are listed as Multiple-file XML2 (and JSON) or Single-file XML (and JSON).

The following report is offered under the Search Strategy section:  
1). "ASN.1" Search Strategy. A record of the parameters, query, and database used in the search. This file can be used to start a stand-alone BLAST search, see
https://www.ncbi.nlm.nih.gov/books/NBK1763/#CmdLineAppsManual.I455\_BLAST\_search\_stra


# Job title: F pink F -- 15..1016 of sequence (1002

Results for:

lcl|Query\_206283 F pink F -- 15..1016 of sequence(1002bp)
[?]

Your BLAST job specified more than one input sequence.
This box lets you choose which input sequence to show BLAST results for.

RID
:   A4XT5JYR014 (Expires on 03-10 18:59 pm)

Query ID
:   lcl|Query\_206283
:   lcl|Query\_206283

Description
:   F pink F -- 15..1016 of sequence

Molecule type
:   nucleic acid

Query Length
:   1002

Database Name
:   nr

Description
:   Nucleotide collection (nt) See details

Program
:   BLASTN 2.8.0+ Citation

  

Reference 

Zheng Zhang, Scott Schwartz, Lukas Wagner, and Webb Miller (2000), "A greedy algorithm for aligning DNA sequences", J Comput Biol 2000; 7(1-2):203-14.

Reference - database indexing

Aleksandr Morgulis, George Coulouris, Yan Raytselis, Thomas L. Madden, Richa Agarwala, Alejandro A. Schäffer (2008), "Database Indexing for Production MegaBLAST Searches", Bioinformatics 24:1757-1764.

Other reports:
Search Summary

[Taxonomy reports]
[Distance tree of results]
[MSA viewer]

Search Parameters

| Search parameter name | Search parameter value |
| --- | --- |
| Program | blastn |
| Word size | 28 |
| Expect value | 10 |
| Hitlist size | 100 |
| Match/Mismatch scores | 1,-2 |
| Gapcosts | 0,2.5 |
| Low Complexity Filter | Yes |
| Filter string | L;m; |
| Genetic Code | 1 |

Database

| Database parameter name | Database parameter value |
| --- | --- |
| Posted date | Mar 7, 2018 1:58 PM |
| Number of letters | 174,044,644,244 |
| Number of sequences | 46,882,714 |
| Entrez query | Includes:  Excludes:  None |

Karlin-Altschul statistics

| Params | Ungapped | Gapped |
| --- | --- | --- |
| Lambda | 1.33271 | 1.28 |
| K | 0.620991 | 0.46 |
| H | 1.12409 | 0.85 |

Results Statistics

| Results Statistics parameter name | Results Statistics parameter value |
| --- | --- |
| Length adjustment | 35 |
| Effective length of query | 967 |
| Effective length of database | 172403749254 |
| Effective search space | 166714425528618 |
| Effective search space used | 166714425528618 |


## Graphic Summary

### Distribution of the top 100 Blast Hits on 100 subject sequences [?]

The graphic is an overview of the database sequences aligned to the query sequence. These are represented horizontal bars colored coded by score and showing the extent
of the alignment on the query sequence. Separate aligned regions on the same database sequence are connected by a thin grey line.
Mousing over an alignment shows the database sequence title. Clicking an alignment displays a box with more details about the alignment and
link to the sequence alignment itself in the Alignments section of the report.

Mouse over to see the title, click to show alignments

Color key for alignment scores

<40

40-50

50-80

80-200

>=200

Query

1

200

400

600

800

1000

Uncultured bacterium clone nbw208h09c1 16S ribosomal RN..

Score:1821 Evalue:0

Accession:GQ073426.1

Alignment

Paenibacillus sp. strain BSW35 16S ribosomal RNA gene, ..

Score:1801 Evalue:0

Accession:KY446062.1

Alignment

Bacterium 4R1 16S ribosomal RNA gene, partial sequence

Score:1799 Evalue:0

Accession:KP965572.1

Alignment

Paenibacillus sp. strain PS-K-17 16S ribosomal RNA gene..

Score:1790 Evalue:0

Accession:MF443394.1

Alignment

Paenibacillus sp. TI45-13ar 16S ribosomal RNA gene, par..

Score:1790 Evalue:0

Accession:KY419705.1

Alignment

Bacterium 4L18 16S ribosomal RNA gene, partial sequence

Score:1790 Evalue:0

Accession:KP965574.1

Alignment

Bacterium 4L13 16S ribosomal RNA gene, partial sequence

Score:1784 Evalue:0

Accession:KP965573.1

Alignment

Paenibacillus sp. Rs5 16S ribosomal RNA gene, partial s..

Score:1781 Evalue:0

Accession:JQ977193.1

Alignment

Paenibacillus sp. 3Cp1 16S ribosomal RNA gene, partial ..

Score:1773 Evalue:0

Accession:KP852522.1

Alignment

Uncultured bacterium clone 4L13 16S ribosomal RNA gene,..

Score:1773 Evalue:0

Accession:KP843054.1

Alignment

Paenibacillus sp. TA\_EU partial 16S rRNA gene, strain T..

Score:1773 Evalue:0

Accession:HG942127.1

Alignment

Bacterium 4R9 16S ribosomal RNA gene, partial sequence

Score:1772 Evalue:0

Accession:KP965575.1

Alignment

Uncultured bacterium clone 10-9BM 16S ribosomal RNA gen..

Score:1772 Evalue:0

Accession:HQ143281.1

Alignment

Uncultured bacterium clone ncd2164d05c1 16S ribosomal R..

Score:1766 Evalue:0

Accession:JF185937.1

Alignment

Paenibacillus sp. Q1-R10 16S ribosomal RNA gene, partia..

Score:1762 Evalue:0

Accession:JX994123.1

Alignment

Paenibacillus sp. 9N4 16S ribosomal RNA gene, partial s..

Score:1760 Evalue:0

Accession:HQ284889.1

Alignment

Uncultured bacterium clone ncd2162h02c1 16S ribosomal R..

Score:1760 Evalue:0

Accession:JF185833.1

Alignment

Uncultured bacterium clone ncd2163a03c1 16S ribosomal R..

Score:1755 Evalue:0

Accession:JF185844.1

Alignment

Paenibacillus sp. NCCB 100172 16S ribosomal RNA gene, p..

Score:1755 Evalue:0

Accession:EU003588.1

Alignment

Paenibacillus hordei strain RH-N24 16S ribosomal RNA ge..

Score:1753 Evalue:0

Accession:NR\_109318.1

Alignment

Paenibacillus sp. HDDMM03 16S ribosomal RNA gene, parti..

Score:1749 Evalue:0

Accession:EU723825.1

Alignment

Paenibacillus kyungheensis strain DCY88 16S ribosomal R..

Score:1738 Evalue:0

Accession:NR\_145628.1

Alignment

Paenibacillus kyungheensis strain DCY88 16S ribosomal R..

Score:1738 Evalue:0

Accession:KF793934.1

Alignment

Paenibacillus nicotianae strain YIM h-19 16S ribosomal ..

Score:1733 Evalue:0

Accession:NR\_134783.2

Alignment

Paenibacillus sp. R1 16S ribosomal RNA gene, partial se..

Score:1733 Evalue:0

Accession:KR263165.1

Alignment

Paenibacillus nicotianae strain YIM h-19 16S ribosomal ..

Score:1733 Evalue:0

Accession:KJ792471.1

Alignment

Paenibacillus illinoisensis strain WR\_17 16S ribosomal ..

Score:1709 Evalue:0

Accession:HQ661869.1

Alignment

Paenibacillus illinoisensis genomic DNA containing 16S-..

Score:1696 Evalue:0

Accession:LM994777.1

Alignment

Paenibacillus illinoisensis partial 16S rRNA gene, isol..

Score:1681 Evalue:0

Accession:HE587985.1

Alignment

Paenibacillus sp. UPUM12 16S ribosomal RNA gene, partia..

Score:1653 Evalue:0

Accession:KP889056.1

Alignment

Paenibacillus sp. SC22 partial 16S rRNA gene, isolate S..

Score:1652 Evalue:0

Accession:HF566260.1

Alignment

Paenibacillus illinoisensis partial 16S rRNA gene, isol..

Score:1642 Evalue:0

Accession:HE587984.1

Alignment

Uncultured bacterium clone 56-9BM 16S ribosomal RNA gen..

Score:1642 Evalue:0

Accession:HQ143315.1

Alignment

Paenibacillus hordei strain poapink1 16S ribosomal RNA ..

Score:1615 Evalue:0

Accession:KJ528493.1

Alignment

Paenibacillus sp. MBEU37 gene for 16S rRNA, partial seq..

Score:1567 Evalue:0

Accession:AB733580.1

Alignment

Paenibacillus sp. strain B1 16S ribosomal RNA gene, par..

Score:1563 Evalue:0

Accession:KY111475.1

Alignment

Paenibacillus sp. EN-26 16S ribosomal RNA gene, partial..

Score:1563 Evalue:0

Accession:KP702927.1

Alignment

Paenibacillus sp. BL13 16S ribosomal RNA gene, partial ..

Score:1563 Evalue:0

Accession:EU912463.1

Alignment

Paenibacillus sp. A10 16S ribosomal RNA gene, partial s..

Score:1561 Evalue:0

Accession:KP126817.1

Alignment

Paenibacillus illinoisensis strain SBANHCa16 16S riboso..

Score:1561 Evalue:0

Accession:KR152299.1

Alignment

Paenibacillus illinoisensis strain P9S 16S ribosomal RN..

Score:1561 Evalue:0

Accession:KF010797.1

Alignment

Paenibacillus sp. BL17-2 16S ribosomal RNA gene, partia..

Score:1561 Evalue:0

Accession:EU912464.1

Alignment

Paenibacillus durus strain YUPP-3 16S ribosomal RNA gen..

Score:1561 Evalue:0

Accession:FJ696708.1

Alignment

Paenibacillus sp. XP13 16S ribosomal RNA gene, partial ..

Score:1561 Evalue:0

Accession:GU080334.1

Alignment

Paenibacillus illinoisensis partial 16S rRNA gene, stra..

Score:1561 Evalue:0

Accession:FN422001.1

Alignment

Paenibacillus illinoisensis strain JCM 9907 16S ribosom..

Score:1559 Evalue:0

Accession:NR\_040884.1

Alignment

Paenibacillus sp. CRRI-82\_WR13B 16S ribosomal RNA gene,..

Score:1557 Evalue:0

Accession:KF840379.1

Alignment

Paenibacillus illinoisensis strain 3728 16S ribosomal R..

Score:1557 Evalue:0

Accession:JN867752.1

Alignment

Paenibacillus illinoisensis strain NBRC 15959 16S ribos..

Score:1557 Evalue:0

Accession:NR\_113828.1

Alignment

Bacterium enrichment culture clone JYJ-11 16S ribosomal..

Score:1555 Evalue:0

Accession:KR996781.1

Alignment

Paenibacillus sp. H316 16S ribosomal RNA gene, partial ..

Score:1555 Evalue:0

Accession:KT205397.1

Alignment

Paenibacillus sp. THK-CF25 16S ribosomal RNA gene, part..

Score:1555 Evalue:0

Accession:KJ769171.1

Alignment

Paenibacillus durus strain IARI-IIWP-40 16S ribosomal R..

Score:1555 Evalue:0

Accession:KF054892.1

Alignment

Paenibacillus illinoisensis strain F24 16S ribosomal RN..

Score:1555 Evalue:0

Accession:JQ579623.1

Alignment

Paenibacillus durus strain hswX78 16S ribosomal RNA gen..

Score:1555 Evalue:0

Accession:JQ236815.1

Alignment

Paenibacillus sp. XGL212 16S ribosomal RNA gene, partia..

Score:1555 Evalue:0

Accession:JQ063001.1

Alignment

## Descriptions

, Reading indexes 1-5, displaying indexes 1-5


Load next setPrevious Match

Sequences producing significant alignments:

Show all columns  of the table presenting sequences producing significant alignments 

Select:AllNone
Selected:0

Alignments
Download

FASTA (complete sequence)

FASTA (aligned sequences)

GenBank (complete sequence)

Hit Table (text)

Hit Table (CSV)

Text

XML

ASN.1

Continue
Cancel

GenBank 
Graphics
Distance tree of results
Multiple alignment
Show/hide columns of the table presenting sequences producing significant alignments 

Available columns

Description  
Max Score  
Total Score  
Coverage  
E-value  
IdentN  
Accession  
Restore Defaults
Ok
Cancel

Sequences producing significant alignments:

| Select for downloading or viewing reports | Description | Max score | Total score | Query cover | E value | Ident | Accession |
| --- | --- | --- | --- | --- | --- | --- | --- |
| 1Select seq GQ073426.1 | Uncultured bacterium clone nbw208h09c1 16S ribosomal RNA gene, partial sequence | 1821 | 1821 | 99% | 0.0 | 99% | GQ073426.1 |
| 2Select seq KY446062.1 | Paenibacillus sp. strain BSW35 16S ribosomal RNA gene, partial sequence | 1801 | 1801 | 99% | 0.0 | 99% | KY446062.1 |
| 3Select seq KP965572.1 | Bacterium 4R1 16S ribosomal RNA gene, partial sequence | 1799 | 1799 | 99% | 0.0 | 99% | KP965572.1 |
| 4Select seq MF443394.1 | Paenibacillus sp. strain PS-K-17 16S ribosomal RNA gene, partial sequence | 1790 | 1790 | 99% | 0.0 | 99% | MF443394.1 |
| 5Select seq KY419705.1 | Paenibacillus sp. TI45-13ar 16S ribosomal RNA gene, partial sequence | 1790 | 1790 | 99% | 0.0 | 99% | KY419705.1 |
| 6Select seq KP965574.1 | Bacterium 4L18 16S ribosomal RNA gene, partial sequence | 1790 | 1790 | 99% | 0.0 | 99% | KP965574.1 |
| 7Select seq KP965573.1 | Bacterium 4L13 16S ribosomal RNA gene, partial sequence | 1784 | 1784 | 99% | 0.0 | 99% | KP965573.1 |
| 8Select seq JQ977193.1 | Paenibacillus sp. Rs5 16S ribosomal RNA gene, partial sequence | 1781 | 1781 | 99% | 0.0 | 99% | JQ977193.1 |
| 9Select seq KP852522.1 | Paenibacillus sp. 3Cp1 16S ribosomal RNA gene, partial sequence | 1773 | 1773 | 99% | 0.0 | 99% | KP852522.1 |
| 10Select seq KP843054.1 | Uncultured bacterium clone 4L13 16S ribosomal RNA gene, partial sequence | 1773 | 1773 | 99% | 0.0 | 99% | KP843054.1 |
| 11Select seq HG942127.1 | Paenibacillus sp. TA\_EU partial 16S rRNA gene, strain TA\_EU | 1773 | 1773 | 99% | 0.0 | 99% | HG942127.1 |
| 12Select seq KP965575.1 | Bacterium 4R9 16S ribosomal RNA gene, partial sequence | 1772 | 1772 | 99% | 0.0 | 99% | KP965575.1 |
| 13Select seq HQ143281.1 | Uncultured bacterium clone 10-9BM 16S ribosomal RNA gene, partial sequence | 1772 | 1772 | 99% | 0.0 | 99% | HQ143281.1 |
| 14Select seq JF185937.1 | Uncultured bacterium clone ncd2164d05c1 16S ribosomal RNA gene, partial sequence | 1766 | 1766 | 99% | 0.0 | 99% | JF185937.1 |
| 15Select seq JX994123.1 | Paenibacillus sp. Q1-R10 16S ribosomal RNA gene, partial sequence | 1762 | 1762 | 99% | 0.0 | 99% | JX994123.1 |
| 16Select seq HQ284889.1 | Paenibacillus sp. 9N4 16S ribosomal RNA gene, partial sequence | 1760 | 1760 | 98% | 0.0 | 99% | HQ284889.1 |
| 17Select seq JF185833.1 | Uncultured bacterium clone ncd2162h02c1 16S ribosomal RNA gene, partial sequence | 1760 | 1760 | 99% | 0.0 | 98% | JF185833.1 |
| 18Select seq JF185844.1 | Uncultured bacterium clone ncd2163a03c1 16S ribosomal RNA gene, partial sequence | 1755 | 1755 | 99% | 0.0 | 98% | JF185844.1 |
| 19Select seq EU003588.1 | Paenibacillus sp. NCCB 100172 16S ribosomal RNA gene, partial sequence | 1755 | 1755 | 99% | 0.0 | 98% | EU003588.1 |
| 20Select seq NR\_109318.1 | Paenibacillus hordei strain RH-N24 16S ribosomal RNA gene, partial sequence | 1753 | 1753 | 99% | 0.0 | 98% | NR\_109318.1 |
| 21Select seq EU723825.1 | Paenibacillus sp. HDDMM03 16S ribosomal RNA gene, partial sequence | 1749 | 1749 | 99% | 0.0 | 98% | EU723825.1 |
| 22Select seq NR\_145628.1 | Paenibacillus kyungheensis strain DCY88 16S ribosomal RNA, partial sequence | 1738 | 1738 | 99% | 0.0 | 98% | NR\_145628.1 |
| 23Select seq KF793934.1 | Paenibacillus kyungheensis strain DCY88 16S ribosomal RNA gene, partial sequence | 1738 | 1738 | 99% | 0.0 | 98% | KF793934.1 |
| 24Select seq NR\_134783.2 | Paenibacillus nicotianae strain YIM h-19 16S ribosomal RNA, complete sequence | 1733 | 1733 | 99% | 0.0 | 98% | NR\_134783.2 |
| 25Select seq KR263165.1 | Paenibacillus sp. R1 16S ribosomal RNA gene, partial sequence | 1733 | 1733 | 99% | 0.0 | 98% | KR263165.1 |
| 26Select seq KJ792471.1 | Paenibacillus nicotianae strain YIM h-19 16S ribosomal RNA gene, partial sequence | 1733 | 1733 | 99% | 0.0 | 98% | KJ792471.1 |
| 27Select seq HQ661869.1 | Paenibacillus illinoisensis strain WR\_17 16S ribosomal RNA gene, partial sequence | 1709 | 1709 | 95% | 0.0 | 99% | HQ661869.1 |
| 28Select seq LM994777.1 | Paenibacillus illinoisensis genomic DNA containing 16S-23S intergenic spacer region, strain PuiB5.2 | 1696 | 1696 | 94% | 0.0 | 99% | LM994777.1 |
| 29Select seq HE587985.1 | Paenibacillus illinoisensis partial 16S rRNA gene, isolate C5 1197 | 1681 | 1681 | 93% | 0.0 | 99% | HE587985.1 |
| 30Select seq KP889056.1 | Paenibacillus sp. UPUM12 16S ribosomal RNA gene, partial sequence | 1653 | 1653 | 94% | 0.0 | 98% | KP889056.1 |
| 31Select seq HF566260.1 | Paenibacillus sp. SC22 partial 16S rRNA gene, isolate SC22 | 1652 | 1652 | 94% | 0.0 | 98% | HF566260.1 |
| 32Select seq HE587984.1 | Paenibacillus illinoisensis partial 16S rRNA gene, isolate C5 1196 | 1642 | 1642 | 95% | 0.0 | 97% | HE587984.1 |
| 33Select seq HQ143315.1 | Uncultured bacterium clone 56-9BM 16S ribosomal RNA gene, partial sequence | 1642 | 1642 | 90% | 0.0 | 99% | HQ143315.1 |
| 34Select seq KJ528493.1 | Paenibacillus hordei strain poapink1 16S ribosomal RNA gene, partial sequence | 1615 | 1615 | 89% | 0.0 | 99% | KJ528493.1 |
| 35Select seq AB733580.1 | Paenibacillus sp. MBEU37 gene for 16S rRNA, partial sequence | 1567 | 1567 | 99% | 0.0 | 95% | AB733580.1 |
| 36Select seq KY111475.1 | Paenibacillus sp. strain B1 16S ribosomal RNA gene, partial sequence | 1563 | 1563 | 99% | 0.0 | 95% | KY111475.1 |
| 37Select seq KP702927.1 | Paenibacillus sp. EN-26 16S ribosomal RNA gene, partial sequence | 1563 | 1563 | 98% | 0.0 | 95% | KP702927.1 |
| 38Select seq EU912463.1 | Paenibacillus sp. BL13 16S ribosomal RNA gene, partial sequence | 1563 | 1563 | 99% | 0.0 | 95% | EU912463.1 |
| 39Select seq KP126817.1 | Paenibacillus sp. A10 16S ribosomal RNA gene, partial sequence | 1561 | 1561 | 99% | 0.0 | 95% | KP126817.1 |
| 40Select seq KR152299.1 | Paenibacillus illinoisensis strain SBANHCa16 16S ribosomal RNA gene, partial sequence | 1561 | 1561 | 99% | 0.0 | 95% | KR152299.1 |
| 41Select seq KF010797.1 | Paenibacillus illinoisensis strain P9S 16S ribosomal RNA gene, partial sequence | 1561 | 1561 | 99% | 0.0 | 95% | KF010797.1 |
| 42Select seq EU912464.1 | Paenibacillus sp. BL17-2 16S ribosomal RNA gene, partial sequence | 1561 | 1561 | 99% | 0.0 | 95% | EU912464.1 |
| 43Select seq FJ696708.1 | Paenibacillus durus strain YUPP-3 16S ribosomal RNA gene, partial sequence | 1561 | 1561 | 99% | 0.0 | 95% | FJ696708.1 |
| 44Select seq GU080334.1 | Paenibacillus sp. XP13 16S ribosomal RNA gene, partial sequence | 1561 | 1561 | 99% | 0.0 | 95% | GU080334.1 |
| 45Select seq FN422001.1 | Paenibacillus illinoisensis partial 16S rRNA gene, strain IB-1087 | 1561 | 1561 | 99% | 0.0 | 95% | FN422001.1 |
| 46Select seq NR\_040884.1 | Paenibacillus illinoisensis strain JCM 9907 16S ribosomal RNA gene, partial sequence | 1559 | 1559 | 99% | 0.0 | 95% | NR\_040884.1 |
| 47Select seq KF840379.1 | Paenibacillus sp. CRRI-82\_WR13B 16S ribosomal RNA gene, partial sequence | 1557 | 1557 | 99% | 0.0 | 95% | KF840379.1 |
| 48Select seq JN867752.1 | Paenibacillus illinoisensis strain 3728 16S ribosomal RNA gene, partial sequence | 1557 | 1557 | 98% | 0.0 | 95% | JN867752.1 |
| 49Select seq NR\_113828.1 | Paenibacillus illinoisensis strain NBRC 15959 16S ribosomal RNA gene, partial sequence | 1557 | 1557 | 99% | 0.0 | 95% | NR\_113828.1 |
| 50Select seq KR996781.1 | Bacterium enrichment culture clone JYJ-11 16S ribosomal RNA gene, partial sequence | 1555 | 1555 | 99% | 0.0 | 95% | KR996781.1 |
| 51Select seq KT205397.1 | Paenibacillus sp. H316 16S ribosomal RNA gene, partial sequence | 1555 | 1555 | 98% | 0.0 | 95% | KT205397.1 |
| 52Select seq KJ769171.1 | Paenibacillus sp. THK-CF25 16S ribosomal RNA gene, partial sequence | 1555 | 1555 | 98% | 0.0 | 95% | KJ769171.1 |
| 53Select seq KF054892.1 | Paenibacillus durus strain IARI-IIWP-40 16S ribosomal RNA gene, partial sequence | 1555 | 1555 | 99% | 0.0 | 95% | KF054892.1 |
| 54Select seq JQ579623.1 | Paenibacillus illinoisensis strain F24 16S ribosomal RNA gene, partial sequence | 1555 | 1555 | 99% | 0.0 | 95% | JQ579623.1 |
| 55Select seq JQ236815.1 | Paenibacillus durus strain hswX78 16S ribosomal RNA gene, partial sequence | 1555 | 1555 | 99% | 0.0 | 95% | JQ236815.1 |
| 56Select seq JQ063001.1 | Paenibacillus sp. XGL212 16S ribosomal RNA gene, partial sequence | 1555 | 1555 | 99% | 0.0 | 95% | JQ063001.1 |
| 57Select seq AB680852.1 | Paenibacillus illinoisensis gene for 16S rRNA, partial sequence, strain: NBRC 15379 | 1555 | 1555 | 99% | 0.0 | 95% | AB680852.1 |
| 58Select seq MG266466.1 | Paenibacillus illinoisensis strain Z002 16S ribosomal RNA gene, partial sequence | 1554 | 1554 | 98% | 0.0 | 95% | MG266466.1 |
| 59Select seq JN899576.1 | Paenibacillus sp. WS26 16S ribosomal RNA gene, partial sequence | 1554 | 1554 | 99% | 0.0 | 95% | JN899576.1 |
| 60Select seq KR856190.1 | Paenibacillus illinoisensis strain C1 16S ribosomal RNA gene, partial sequence | 1550 | 1550 | 98% | 0.0 | 95% | KR856190.1 |
| 61Select seq KJ951054.1 | Paenibacillus illinoisensis strain YZ29 16S ribosomal RNA gene, partial sequence | 1550 | 1550 | 99% | 0.0 | 95% | KJ951054.1 |
| 62Select seq KF475708.1 | Bacterium R-22 16S ribosomal RNA gene, partial sequence | 1550 | 1550 | 98% | 0.0 | 95% | KF475708.1 |
| 63Select seq JN899577.1 | Paenibacillus sp. WS28 16S ribosomal RNA gene, partial sequence | 1550 | 1550 | 99% | 0.0 | 95% | JN899577.1 |
| 64Select seq JN899571.1 | Paenibacillus sp. WS16 16S ribosomal RNA gene, partial sequence | 1550 | 1550 | 99% | 0.0 | 95% | JN899571.1 |
| 65Select seq HQ764278.1 | Uncultured organism clone ELU0055-T366-S-NIPCRAMgANa\_000412 small subunit ribosomal RNA gene, partial sequence | 1550 | 1550 | 99% | 0.0 | 95% | HQ764278.1 |
| 66Select seq JF892727.1 | Paenibacillus sp. F37 16S ribosomal RNA gene, partial sequence | 1550 | 1550 | 99% | 0.0 | 95% | JF892727.1 |
| 67Select seq KX350030.1 | Paenibacillus xylanilyticus strain F2-2-22 16S ribosomal RNA gene, partial sequence | 1548 | 1548 | 98% | 0.0 | 95% | KX350030.1 |
| 68Select seq KR856189.1 | Paenibacillus illinoisensis strain D4 16S ribosomal RNA gene, partial sequence | 1548 | 1548 | 98% | 0.0 | 95% | KR856189.1 |
| 69Select seq DQ105965.1 | P-decomposing bacterium 1 16S ribosomal RNA gene, partial sequence | 1548 | 1548 | 99% | 0.0 | 95% | DQ105965.1 |
| 70Select seq AB748941.1 | Paenibacillus xylanilyticus gene for 16S ribosomal RNA, partial sequence, strain: AECSP02 | 1546 | 1546 | 99% | 0.0 | 95% | AB748941.1 |
| 71Select seq LC011515.1 | Paenibacillus sp. MTB37-3 gene for 16S ribosomal RNA, partial sequence | 1544 | 1544 | 99% | 0.0 | 95% | LC011515.1 |
| 72Select seq KM817264.1 | Paenibacillus illinoisensis strain IHB B 15700 16S ribosomal RNA gene, partial sequence | 1544 | 1544 | 99% | 0.0 | 95% | KM817264.1 |
| 73Select seq KJ577635.1 | Paenibacillus illinoisensis strain AAU M 17 16S ribosomal RNA gene, partial sequence | 1543 | 1543 | 98% | 0.0 | 95% | KJ577635.1 |
| 74Select seq KF848956.1 | Paenibacillus sp. FZ104 16S ribosomal RNA gene, partial sequence | 1543 | 1543 | 99% | 0.0 | 94% | KF848956.1 |
| 75Select seq GQ202680.1 | Paenibacillus sp. U5 STR 16S ribosomal RNA gene, partial sequence | 1543 | 1543 | 99% | 0.0 | 95% | GQ202680.1 |
| 76Select seq KP126829.1 | Paenibacillus sp. CF7 16S ribosomal RNA gene, partial sequence | 1541 | 1541 | 98% | 0.0 | 95% | KP126829.1 |
| 77Select seq KC000067.1 | Uncultured bacterium clone Water1 16S ribosomal RNA gene, partial sequence | 1541 | 1541 | 99% | 0.0 | 94% | KC000067.1 |
| 78Select seq JN899578.1 | Paenibacillus sp. WS30 16S ribosomal RNA gene, partial sequence | 1541 | 1541 | 99% | 0.0 | 95% | JN899578.1 |
| 79Select seq MG266469.1 | Paenibacillus xylanilyticus strain Z007 16S ribosomal RNA gene, partial sequence | 1539 | 1539 | 99% | 0.0 | 94% | MG266469.1 |
| 80Select seq MG266463.1 | Paenibacillus xylanilyticus strain M033 16S ribosomal RNA gene, partial sequence | 1539 | 1539 | 99% | 0.0 | 94% | MG266463.1 |
| 81Select seq MG266462.1 | Paenibacillus xylanilyticus strain M032 16S ribosomal RNA gene, partial sequence | 1539 | 1539 | 99% | 0.0 | 94% | MG266462.1 |
| 82Select seq MG266460.1 | Paenibacillus xylanilyticus strain M030 16S ribosomal RNA gene, partial sequence | 1539 | 1539 | 99% | 0.0 | 94% | MG266460.1 |
| 83Select seq MG027626.1 | Paenibacillus illinoisensis strain VBE24 16S ribosomal RNA gene, partial sequence | 1539 | 1539 | 98% | 0.0 | 95% | MG027626.1 |
| 84Select seq KX212148.1 | Uncultured bacterium clone HT3 16S ribosomal RNA gene, partial sequence | 1539 | 1539 | 99% | 0.0 | 95% | KX212148.1 |
| 85Select seq KX825907.1 | Paenibacillus sp. strain I2746 16S ribosomal RNA gene, partial sequence | 1539 | 1539 | 98% | 0.0 | 95% | KX825907.1 |
| 86Select seq KP670215.1 | Paenibacillus sp. DB14410 16S ribosomal RNA gene, partial sequence | 1539 | 1539 | 98% | 0.0 | 95% | KP670215.1 |
| 87Select seq KP172212.1 | Paenibacillus sp. EgN2201 16S ribosomal RNA gene, partial sequence | 1539 | 1539 | 99% | 0.0 | 94% | KP172212.1 |
| 88Select seq KM019819.1 | Paenibacillus taichungensis strain F9 16S ribosomal RNA gene, partial sequence | 1539 | 1539 | 99% | 0.0 | 95% | KM019819.1 |
| 89Select seq KF923402.1 | Paenibacillus xylanilyticus strain 2-6L3 16S ribosomal RNA gene, partial sequence | 1539 | 1539 | 96% | 0.0 | 95% | KF923402.1 |
| 90Select seq HQ256551.1 | Paenibacillus illinoisensis strain NBB11 16S ribosomal RNA gene, partial sequence | 1539 | 1539 | 99% | 0.0 | 94% | HQ256551.1 |
| 91Select seq FJ232954.1 | Paenibacillus sp. ZY-8 16S ribosomal RNA gene, partial sequence | 1539 | 1539 | 99% | 0.0 | 94% | FJ232954.1 |
| 92Select seq FJ006887.1 | Paenibacillus sp. WPCB083 16S ribosomal RNA gene, partial sequence | 1539 | 1539 | 99% | 0.0 | 95% | FJ006887.1 |
| 93Select seq AB366401.1 | Paenibacillus pabuli gene for 16S ribosomal RNA, partial sequence, strain: SW12 | 1539 | 1539 | 99% | 0.0 | 94% | AB366401.1 |
| 94Select seq AB366400.1 | Paenibacillus pabuli gene for 16S ribosomal RNA, partial sequence, strain: SW10 | 1539 | 1539 | 99% | 0.0 | 94% | AB366400.1 |
| 95Select seq EU218535.1 | Paenibacillus sp. K1 16S ribosomal RNA gene, complete sequence | 1539 | 1539 | 99% | 0.0 | 94% | EU218535.1 |
| 96Select seq AM162339.1 | Paenibacillus sp. J17-18 partial 16S rRNA gene | 1539 | 1539 | 99% | 0.0 | 95% | AM162339.1 |
| 97Select seq KP670221.1 | Paenibacillus sp. DB14405 16S ribosomal RNA gene, partial sequence | 1537 | 1537 | 98% | 0.0 | 95% | KP670221.1 |
| 98Select seq GQ284463.1 | Paenibacillus sp. TSWCW18 16S ribosomal RNA gene, partial sequence | 1537 | 1537 | 99% | 0.0 | 94% | GQ284463.1 |
| 99Select seq AB110989.1 | Paenibacillus sp. W-61 gene for 16S rRNA, partial sequence | 1537 | 1537 | 98% | 0.0 | 95% | AB110989.1 |
| 100Select seq FJ006870.1 | Paenibacillus sp. WPCB023 16S ribosomal RNA gene, partial sequence | 1530 | 1530 | 99% | 0.0 | 94% | FJ006870.1 |


## Alignments

Loading alignment... for sequences gi|238309603,gi|1196644317,gi|846924041,gi|1214741488,gi|1127353198 Reading indexes 1-5

Download

FASTA (complete sequence)

FASTA (aligned sequences)

GenBank (complete sequence)

Continue
Cancel

GenBankGraphics

Next
Previous
Descriptions

Uncultured bacterium clone nbw208h09c1 16S ribosomal RNA gene, partial sequence

Sequence ID: GQ073426.1Length: 1374Number of Matches: 1

Related Information

Range 1: 30 to 1024GenBankGraphics

Next Match
Previous Match
First Match

Alignment statistics for match #1

| Score | Expect | Identities | Gaps | Strand | Frame |
| --- | --- | --- | --- | --- | --- |
| 1821 bits(986) | 0.0() | 991/995(99%) | 0/995(0%) | Plus/Plus |  |

Features:

```
Query  6     GCANGTCGAGCGGAGTTGATGAGAAGCTTGCTTCTCGGATACTTAGCGGCGGACGGGTGA  65
             ||| ||||||||||||||||||||||||||||||||||||||||||||||||||||||||
Sbjct  30    GCAAGTCGAGCGGAGTTGATGAGAAGCTTGCTTCTCGGATACTTAGCGGCGGACGGGTGA  89

Query  66    GTAACACGTAGGCAACCTGCCCTCAAGCTTGGGACAACTACCGGAAACGGTAGCTAATAC  125
             ||||||||||||||||||||||||||||||||||||||||||||||||||||||||||||
Sbjct  90    GTAACACGTAGGCAACCTGCCCTCAAGCTTGGGACAACTACCGGAAACGGTAGCTAATAC  149

Query  126   CGAATACATGATTTGTTCGCCTGAACGAATTTGGAAAGACGGAGCAATCTGTCACTTGAG  185
             ||||||||||||||||||||||||||||||||||||||||||||||||||||||||||||
Sbjct  150   CGAATACATGATTTGTTCGCCTGAACGAATTTGGAAAGACGGAGCAATCTGTCACTTGAG  209

Query  186   GATGGGCCTGCGGCGCATTAGCTAGTTGGTGAGGTAACGGCTCACCAAGGCGACGATGCG  245
             ||||||||||||||||||||||||||||||||||||||||||||||||||||||||||||
Sbjct  210   GATGGGCCTGCGGCGCATTAGCTAGTTGGTGAGGTAACGGCTCACCAAGGCGACGATGCG  269

Query  246   TAGCCGACCTGAGAGGGTGATCGGCCACACTGGGACTGAGACACGGCCCAGACTCCTACG  305
             ||||||||||||||||||||||||||||||||||||||||||||||||||||||||||||
Sbjct  270   TAGCCGACCTGAGAGGGTGATCGGCCACACTGGGACTGAGACACGGCCCAGACTCCTACG  329

Query  306   GGAGGCAGCAGTAGGGAATCTTCCGCAATGGACGCAAGTCTGACGGAGCAACGCCGCGTG  365
             ||||||||||||||||||||||||||||||||||||||||||||||||||||||||||||
Sbjct  330   GGAGGCAGCAGTAGGGAATCTTCCGCAATGGACGCAAGTCTGACGGAGCAACGCCGCGTG  389

Query  366   AGTGATGAAGGTTTTCGGATCGTAAAGCTCTGTTGCCAGGGAAGAACGTCCGGGTTAGTA  425
             ||||||||||||||||||||||||||||||||||||||||||||||||||||||||||||
Sbjct  390   AGTGATGAAGGTTTTCGGATCGTAAAGCTCTGTTGCCAGGGAAGAACGTCCGGGTTAGTA  449

Query  426   ACTGNANTCGGAGTGACGGTACCTGAGAAGAAAGCCCCGGCTAACTACGTGCCAGCAGCC  485
             |||| |  ||||||||||||||||||||||||||||||||||||||||||||||||||||
Sbjct  450   ACTGAACCCGGAGTGACGGTACCTGAGAAGAAAGCCCCGGCTAACTACGTGCCAGCAGCC  509

Query  486   GCGGTAATACGTAGGGGGCAAGCGTTGTCCGGAATTATTGGGCGTAAAGCGCGCGCAGGC  545
             ||||||||||||||||||||||||||||||||||||||||||||||||||||||||||||
Sbjct  510   GCGGTAATACGTAGGGGGCAAGCGTTGTCCGGAATTATTGGGCGTAAAGCGCGCGCAGGC  569

Query  546   GGCTTTTTAAGTCCGGTGTCACAGCCCAAGGCTCAACCTTGGGTCGCACTGGAAACTGGA  605
             ||||||||||||||||||||||||||||||||||||||||||||||||||||||||||||
Sbjct  570   GGCTTTTTAAGTCCGGTGTCACAGCCCAAGGCTCAACCTTGGGTCGCACTGGAAACTGGA  629

Query  606   GAGCTTGAGTACAGAAGAGGAAAGTGGAATTCCACGTGTAGCGGTGAAATGCGTAGAGAT  665
             ||||||||||||||||||||||||||||||||||||||||||||||||||||||||||||
Sbjct  630   GAGCTTGAGTACAGAAGAGGAAAGTGGAATTCCACGTGTAGCGGTGAAATGCGTAGAGAT  689

Query  666   GTGGAGGAACACCAGTGGCGAAGGCGACTTTCTGGGCTGTAACTGACGCTGAGGCGCGAA  725
             ||||||||||||||||||||||||||||||||||||||||||||||||||||||||||||
Sbjct  690   GTGGAGGAACACCAGTGGCGAAGGCGACTTTCTGGGCTGTAACTGACGCTGAGGCGCGAA  749

Query  726   AGCGTGGGGAGCAAACAGGATTAGATACCCTGGTAGTCCACGCCGTAAACGATGAATGCT  785
             ||||||||||||||||||||||||||||||||||||||||||||||||||||||||||||
Sbjct  750   AGCGTGGGGAGCAAACAGGATTAGATACCCTGGTAGTCCACGCCGTAAACGATGAATGCT  809

Query  786   AGGTGTTAGGGGTTTCGATACCCTTGGTGCCGAAGTTAACACATTAAGCATTCCGCCTGG  845
             ||||||||||||||||||||||||||||||||||||||||||||||||||||||||||||
Sbjct  810   AGGTGTTAGGGGTTTCGATACCCTTGGTGCCGAAGTTAACACATTAAGCATTCCGCCTGG  869

Query  846   GGAGTACGGTCGCAAGACTGAAACTCAAAGGAATTGACGGGGACCCGCACAAGCAGTGGA  905
             ||||||||||||||||||||||||||||||||||||||||||||||||||||||||||||
Sbjct  870   GGAGTACGGTCGCAAGACTGAAACTCAAAGGAATTGACGGGGACCCGCACAAGCAGTGGA  929

Query  906   GTATGTGGTTTAATTCGAAGCAACGCGAAGAACCTTACCAAGTCTTGACATCCCTTTGAC  965
             ||||||||||||||||||||||||||||||||||||||||||||||||||||||||||||
Sbjct  930   GTATGTGGTTTAATTCGAAGCAACGCGAAGAACCTTACCAAGTCTTGACATCCCTTTGAC  989

Query  966   CGGACTAGAGATAGTCTTTTCCTTCGGGACAAAGG  1000
             |||||||||||||||||||||||||||||||||||
Sbjct  990   CGGACTAGAGATAGTCTTTTCCTTCGGGACAAAGG  1024
```

Download

FASTA (complete sequence)

FASTA (aligned sequences)

GenBank (complete sequence)

Continue
Cancel

GenBankGraphics

Next
Previous
Descriptions

Paenibacillus sp. strain BSW35 16S ribosomal RNA gene, partial sequence

Sequence ID: KY446062.1Length: 1433Number of Matches: 1

Related Information

Range 1: 9 to 1001GenBankGraphics

Next Match
Previous Match
First Match

Alignment statistics for match #1

| Score | Expect | Identities | Gaps | Strand | Frame |
| --- | --- | --- | --- | --- | --- |
| 1801 bits(975) | 0.0() | 988/995(99%) | 2/995(0%) | Plus/Plus |  |

Features:

```
Query  6     GCANGTCGAGCGGAGTTGATGAGAAGCTTGCTTCTCGGATACTTAGCGGCGGACGGGTGA  65
             ||| ||||||||||||||| |||||||||||||||| |||||||||||||||||||||||
Sbjct  9     GCA-GTCGAGCGGAGTTGA-GAGAAGCTTGCTTCTCTGATACTTAGCGGCGGACGGGTGA  66

Query  66    GTAACACGTAGGCAACCTGCCCTCAAGCTTGGGACAACTACCGGAAACGGTAGCTAATAC  125
             ||||||||||||||||||||||||||||||||||||||||||||||||||||||||||||
Sbjct  67    GTAACACGTAGGCAACCTGCCCTCAAGCTTGGGACAACTACCGGAAACGGTAGCTAATAC  126

Query  126   CGAATACATGATTTGTTCGCCTGAACGAATTTGGAAAGACGGAGCAATCTGTCACTTGAG  185
             ||||||||||||||||||||||||||||||||||||||||||||||||||||||||||||
Sbjct  127   CGAATACATGATTTGTTCGCCTGAACGAATTTGGAAAGACGGAGCAATCTGTCACTTGAG  186

Query  186   GATGGGCCTGCGGCGCATTAGCTAGTTGGTGAGGTAACGGCTCACCAAGGCGACGATGCG  245
             ||||||||||||||||||||||||||||||||||||||||||||||||||||||||||||
Sbjct  187   GATGGGCCTGCGGCGCATTAGCTAGTTGGTGAGGTAACGGCTCACCAAGGCGACGATGCG  246

Query  246   TAGCCGACCTGAGAGGGTGATCGGCCACACTGGGACTGAGACACGGCCCAGACTCCTACG  305
             ||||||||||||||||||||||||||||||||||||||||||||||||||||||||||||
Sbjct  247   TAGCCGACCTGAGAGGGTGATCGGCCACACTGGGACTGAGACACGGCCCAGACTCCTACG  306

Query  306   GGAGGCAGCAGTAGGGAATCTTCCGCAATGGACGCAAGTCTGACGGAGCAACGCCGCGTG  365
             ||||||||||||||||||||||||||||||||||||||||||||||||||||||||||||
Sbjct  307   GGAGGCAGCAGTAGGGAATCTTCCGCAATGGACGCAAGTCTGACGGAGCAACGCCGCGTG  366

Query  366   AGTGATGAAGGTTTTCGGATCGTAAAGCTCTGTTGCCAGGGAAGAACGTCCGGGTTAGTA  425
             |||||||||||||||||||||||||||||||||||||||||||||||||||| || ||||
Sbjct  367   AGTGATGAAGGTTTTCGGATCGTAAAGCTCTGTTGCCAGGGAAGAACGTCCGAGTGAGTA  426

Query  426   ACTGNANTCGGAGTGACGGTACCTGAGAAGAAAGCCCCGGCTAACTACGTGCCAGCAGCC  485
             |||| | |||||||||||||||||||||||||||||||||||||||||||||||||||||
Sbjct  427   ACTGCACTCGGAGTGACGGTACCTGAGAAGAAAGCCCCGGCTAACTACGTGCCAGCAGCC  486

Query  486   GCGGTAATACGTAGGGGGCAAGCGTTGTCCGGAATTATTGGGCGTAAAGCGCGCGCAGGC  545
             ||||||||||||||||||||||||||||||||||||||||||||||||||||||||||||
Sbjct  487   GCGGTAATACGTAGGGGGCAAGCGTTGTCCGGAATTATTGGGCGTAAAGCGCGCGCAGGC  546

Query  546   GGCTTTTTAAGTCCGGTGTCACAGCCCAAGGCTCAACCTTGGGTCGCACTGGAAACTGGA  605
             ||||||||||||||||||||||||||||||||||||||||||||||||||||||||||||
Sbjct  547   GGCTTTTTAAGTCCGGTGTCACAGCCCAAGGCTCAACCTTGGGTCGCACTGGAAACTGGA  606

Query  606   GAGCTTGAGTACAGAAGAGGAAAGTGGAATTCCACGTGTAGCGGTGAAATGCGTAGAGAT  665
             ||||||||||||||||||||||||||||||||||||||||||||||||||||||||||||
Sbjct  607   GAGCTTGAGTACAGAAGAGGAAAGTGGAATTCCACGTGTAGCGGTGAAATGCGTAGAGAT  666

Query  666   GTGGAGGAACACCAGTGGCGAAGGCGACTTTCTGGGCTGTAACTGACGCTGAGGCGCGAA  725
             ||||||||||||||||||||||||||||||||||||||||||||||||||||||||||||
Sbjct  667   GTGGAGGAACACCAGTGGCGAAGGCGACTTTCTGGGCTGTAACTGACGCTGAGGCGCGAA  726

Query  726   AGCGTGGGGAGCAAACAGGATTAGATACCCTGGTAGTCCACGCCGTAAACGATGAATGCT  785
             ||||||||||||||||||||||||||||||||||||||||||||||||||||||||||||
Sbjct  727   AGCGTGGGGAGCAAACAGGATTAGATACCCTGGTAGTCCACGCCGTAAACGATGAATGCT  786

Query  786   AGGTGTTAGGGGTTTCGATACCCTTGGTGCCGAAGTTAACACATTAAGCATTCCGCCTGG  845
             ||||||||||||||||||||||||||||||||||||||||||||||||||||||||||||
Sbjct  787   AGGTGTTAGGGGTTTCGATACCCTTGGTGCCGAAGTTAACACATTAAGCATTCCGCCTGG  846

Query  846   GGAGTACGGTCGCAAGACTGAAACTCAAAGGAATTGACGGGGACCCGCACAAGCAGTGGA  905
             ||||||||||||||||||||||||||||||||||||||||||||||||||||||||||||
Sbjct  847   GGAGTACGGTCGCAAGACTGAAACTCAAAGGAATTGACGGGGACCCGCACAAGCAGTGGA  906

Query  906   GTATGTGGTTTAATTCGAAGCAACGCGAAGAACCTTACCAAGTCTTGACATCCCTTTGAC  965
             ||||||||||||||||||||||||||||||||||||||||||||||||||||||||||||
Sbjct  907   GTATGTGGTTTAATTCGAAGCAACGCGAAGAACCTTACCAAGTCTTGACATCCCTTTGAC  966

Query  966   CGGACTAGAGATAGTCTTTTCCTTCGGGACAAAGG  1000
             |||||||||||||||||||||||||||||||||||
Sbjct  967   CGGACTAGAGATAGTCTTTTCCTTCGGGACAAAGG  1001
```

Download

FASTA (complete sequence)

FASTA (aligned sequences)

GenBank (complete sequence)

Continue
Cancel

GenBankGraphics

Next
Previous
Descriptions

Bacterium 4R1 16S ribosomal RNA gene, partial sequence

Sequence ID: KP965572.1Length: 1516Number of Matches: 1

Related Information

Range 1: 50 to 1044GenBankGraphics

Next Match
Previous Match
First Match

Alignment statistics for match #1

| Score | Expect | Identities | Gaps | Strand | Frame |
| --- | --- | --- | --- | --- | --- |
| 1799 bits(974) | 0.0() | 987/995(99%) | 0/995(0%) | Plus/Plus |  |

Features:

```
Query  6     GCANGTCGAGCGGAGTTGATGAGAAGCTTGCTTCTCGGATACTTAGCGGCGGACGGGTGA  65
             ||| ||||||||||||    ||||||||||||||||||||||||||||||||||||||||
Sbjct  50    GCAAGTCGAGCGGAGTATCCGAGAAGCTTGCTTCTCGGATACTTAGCGGCGGACGGGTGA  109

Query  66    GTAACACGTAGGCAACCTGCCCTCAAGCTTGGGACAACTACCGGAAACGGTAGCTAATAC  125
             ||||||||||||||||||||||||||||||||||||||||||||||||||||||||||||
Sbjct  110   GTAACACGTAGGCAACCTGCCCTCAAGCTTGGGACAACTACCGGAAACGGTAGCTAATAC  169

Query  126   CGAATACATGATTTGTTCGCCTGAACGAATTTGGAAAGACGGAGCAATCTGTCACTTGAG  185
             ||||||||||||||||||||||||||||||||||||||||||||||||||||||||||||
Sbjct  170   CGAATACATGATTTGTTCGCCTGAACGAATTTGGAAAGACGGAGCAATCTGTCACTTGAG  229

Query  186   GATGGGCCTGCGGCGCATTAGCTAGTTGGTGAGGTAACGGCTCACCAAGGCGACGATGCG  245
             ||||||||||||||||||||||||||||||| ||||||||||||||||||||||||||||
Sbjct  230   GATGGGCCTGCGGCGCATTAGCTAGTTGGTGGGGTAACGGCTCACCAAGGCGACGATGCG  289

Query  246   TAGCCGACCTGAGAGGGTGATCGGCCACACTGGGACTGAGACACGGCCCAGACTCCTACG  305
             ||||||||||||||||||||||||||||||||||||||||||||||||||||||||||||
Sbjct  290   TAGCCGACCTGAGAGGGTGATCGGCCACACTGGGACTGAGACACGGCCCAGACTCCTACG  349

Query  306   GGAGGCAGCAGTAGGGAATCTTCCGCAATGGACGCAAGTCTGACGGAGCAACGCCGCGTG  365
             ||||||||||||||||||||||||||||||||||||||||||||||||||||||||||||
Sbjct  350   GGAGGCAGCAGTAGGGAATCTTCCGCAATGGACGCAAGTCTGACGGAGCAACGCCGCGTG  409

Query  366   AGTGATGAAGGTTTTCGGATCGTAAAGCTCTGTTGCCAGGGAAGAACGTCCGGGTTAGTA  425
             ||||||||||||||||||||||||||||||||||||||||||||||||||||||||||||
Sbjct  410   AGTGATGAAGGTTTTCGGATCGTAAAGCTCTGTTGCCAGGGAAGAACGTCCGGGTTAGTA  469

Query  426   ACTGNANTCGGAGTGACGGTACCTGAGAAGAAAGCCCCGGCTAACTACGTGCCAGCAGCC  485
             |||| | |||||||||||||||||||||||||||||||||||||||||||||||||||||
Sbjct  470   ACTGAACTCGGAGTGACGGTACCTGAGAAGAAAGCCCCGGCTAACTACGTGCCAGCAGCC  529

Query  486   GCGGTAATACGTAGGGGGCAAGCGTTGTCCGGAATTATTGGGCGTAAAGCGCGCGCAGGC  545
             ||||||||||||||||||||||||||||||||||||||||||||||||||||||||||||
Sbjct  530   GCGGTAATACGTAGGGGGCAAGCGTTGTCCGGAATTATTGGGCGTAAAGCGCGCGCAGGC  589

Query  546   GGCTTTTTAAGTCCGGTGTCACAGCCCAAGGCTCAACCTTGGGTCGCACTGGAAACTGGA  605
             ||||||||||||||||||||||||||||||||||||||||||||||||||||||||||||
Sbjct  590   GGCTTTTTAAGTCCGGTGTCACAGCCCAAGGCTCAACCTTGGGTCGCACTGGAAACTGGA  649

Query  606   GAGCTTGAGTACAGAAGAGGAAAGTGGAATTCCACGTGTAGCGGTGAAATGCGTAGAGAT  665
             ||||||||||||||||||||||||||||||||||||||||||||||||||||||||||||
Sbjct  650   GAGCTTGAGTACAGAAGAGGAAAGTGGAATTCCACGTGTAGCGGTGAAATGCGTAGAGAT  709

Query  666   GTGGAGGAACACCAGTGGCGAAGGCGACTTTCTGGGCTGTAACTGACGCTGAGGCGCGAA  725
             ||||||||||||||||||||||||||||||||||||||||||||||||||||||||||||
Sbjct  710   GTGGAGGAACACCAGTGGCGAAGGCGACTTTCTGGGCTGTAACTGACGCTGAGGCGCGAA  769

Query  726   AGCGTGGGGAGCAAACAGGATTAGATACCCTGGTAGTCCACGCCGTAAACGATGAATGCT  785
             ||||||||||||||||||||||||||||||||||||||||||||||||||||||||||||
Sbjct  770   AGCGTGGGGAGCAAACAGGATTAGATACCCTGGTAGTCCACGCCGTAAACGATGAATGCT  829

Query  786   AGGTGTTAGGGGTTTCGATACCCTTGGTGCCGAAGTTAACACATTAAGCATTCCGCCTGG  845
             ||||||||||||||||||||||||||||||||||||||||||||||||||||||||||||
Sbjct  830   AGGTGTTAGGGGTTTCGATACCCTTGGTGCCGAAGTTAACACATTAAGCATTCCGCCTGG  889

Query  846   GGAGTACGGTCGCAAGACTGAAACTCAAAGGAATTGACGGGGACCCGCACAAGCAGTGGA  905
             ||||||||||||||||||||||||||||||||||||||||||||||||||||||||||||
Sbjct  890   GGAGTACGGTCGCAAGACTGAAACTCAAAGGAATTGACGGGGACCCGCACAAGCAGTGGA  949

Query  906   GTATGTGGTTTAATTCGAAGCAACGCGAAGAACCTTACCAAGTCTTGACATCCCTTTGAC  965
             ||||||||||||||||||||||||||||||||||||||||||||||||||||||||||||
Sbjct  950   GTATGTGGTTTAATTCGAAGCAACGCGAAGAACCTTACCAAGTCTTGACATCCCTTTGAC  1009

Query  966   CGGACTAGAGATAGTCTTTTCCTTCGGGACAAAGG  1000
             |||||||||||||||||||||||||||||||||||
Sbjct  1010  CGGACTAGAGATAGTCTTTTCCTTCGGGACAAAGG  1044
```

Download

FASTA (complete sequence)

FASTA (aligned sequences)

GenBank (complete sequence)

Continue
Cancel

GenBankGraphics

Next
Previous
Descriptions

Paenibacillus sp. strain PS-K-17 16S ribosomal RNA gene, partial sequence

Sequence ID: MF443394.1Length: 1462Number of Matches: 1

Related Information

Range 1: 2 to 996GenBankGraphics

Next Match
Previous Match
First Match

Alignment statistics for match #1

| Score | Expect | Identities | Gaps | Strand | Frame |
| --- | --- | --- | --- | --- | --- |
| 1790 bits(969) | 0.0() | 985/995(99%) | 0/995(0%) | Plus/Plus |  |

Features:

```
Query  6    GCANGTCGAGCGGAGTTGATGAGAAGCTTGCTTCTCGGATACTTAGCGGCGGACGGGTGA  65
            ||| ||||||||||||||| |||||||||||||||||||| |||||||||||||||||||
Sbjct  2    GCAAGTCGAGCGGAGTTGAYGAGAAGCTTGCTTCTCGGATGCTTAGCGGCGGACGGGTGA  61

Query  66   GTAACACGTAGGCAACCTGCCCTCAAGCTTGGGACAACTACCGGAAACGGTAGCTAATAC  125
            ||||||||||||||||||||||||||||||||||||||||||||||||||||||||||||
Sbjct  62   GTAACACGTAGGCAACCTGCCCTCAAGCTTGGGACAACTACCGGAAACGGTAGCTAATAC  121

Query  126  CGAATACATGATTTGTTCGCCTGAACGAATTTGGAAAGACGGAGCAATCTGTCACTTGAG  185
            ||||||||||||||||||||||||||||||||||||||||||||||||||||||||||||
Sbjct  122  CGAATACATGATTTGTTCGCCTGAACGAATTTGGAAAGACGGAGCAATCTGTCACTTGAG  181

Query  186  GATGGGCCTGCGGCGCATTAGCTAGTTGGTGAGGTAACGGCTCACCAAGGCGACGATGCG  245
            ||||||||||||||||||||||||||||||||||||||||||||||||||||||||||||
Sbjct  182  GATGGGCCTGCGGCGCATTAGCTAGTTGGTGAGGTAACGGCTCACCAAGGCGACGATGCG  241

Query  246  TAGCCGACCTGAGAGGGTGATCGGCCACACTGGGACTGAGACACGGCCCAGACTCCTACG  305
            ||||||||||||||||||||||||||||||||||||||||||||||||||||||||||||
Sbjct  242  TAGCCGACCTGAGAGGGTGATCGGCCACACTGGGACTGAGACACGGCCCAGACTCCTACG  301

Query  306  GGAGGCAGCAGTAGGGAATCTTCCGCAATGGACGCAAGTCTGACGGAGCAACGCCGCGTG  365
            ||||||||||||||||||||||||||||||||||||||||||||||||||||||||||||
Sbjct  302  GGAGGCAGCAGTAGGGAATCTTCCGCAATGGACGCAAGTCTGACGGAGCAACGCCGCGTG  361

Query  366  AGTGATGAAGGTTTTCGGATCGTAAAGCTCTGTTGCCAGGGAAGAACGTCCGGGTTAGTA  425
            ||||||||||||||||||||||||||||||||||||||||||||||||||||    ||||
Sbjct  362  AGTGATGAAGGTTTTCGGATCGTAAAGCTCTGTTGCCAGGGAAGAACGTCCGATAGAGTA  421

Query  426  ACTGNANTCGGAGTGACGGTACCTGAGAAGAAAGCCCCGGCTAACTACGTGCCAGCAGCC  485
            ||||   |||||||||||||||||||||||||||||||||||||||||||||||||||||
Sbjct  422  ACTGCTATCGGAGTGACGGTACCTGAGAAGAAAGCCCCGGCTAACTACGTGCCAGCAGCC  481

Query  486  GCGGTAATACGTAGGGGGCAAGCGTTGTCCGGAATTATTGGGCGTAAAGCGCGCGCAGGC  545
            ||||||||||||||||||||||||||||||||||||||||||||||||||||||||||||
Sbjct  482  GCGGTAATACGTAGGGGGCAAGCGTTGTCCGGAATTATTGGGCGTAAAGCGCGCGCAGGC  541

Query  546  GGCTTTTTAAGTCCGGTGTCACAGCCCAAGGCTCAACCTTGGGTCGCACTGGAAACTGGA  605
            ||||||||||||||||||||||||||||||||||||||||||||||||||||||||||||
Sbjct  542  GGCTTTTTAAGTCCGGTGTCACAGCCCAAGGCTCAACCTTGGGTCGCACTGGAAACTGGA  601

Query  606  GAGCTTGAGTACAGAAGAGGAAAGTGGAATTCCACGTGTAGCGGTGAAATGCGTAGAGAT  665
            ||||||||||||||||||||||||||||||||||||||||||||||||||||||||||||
Sbjct  602  GAGCTTGAGTACAGAAGAGGAAAGTGGAATTCCACGTGTAGCGGTGAAATGCGTAGAGAT  661

Query  666  GTGGAGGAACACCAGTGGCGAAGGCGACTTTCTGGGCTGTAACTGACGCTGAGGCGCGAA  725
            ||||||||||||||||||||||||||||||||||||||||||||||||||||||||||||
Sbjct  662  GTGGAGGAACACCAGTGGCGAAGGCGACTTTCTGGGCTGTAACTGACGCTGAGGCGCGAA  721

Query  726  AGCGTGGGGAGCAAACAGGATTAGATACCCTGGTAGTCCACGCCGTAAACGATGAATGCT  785
            ||||||||||||||||||||||||||||||||||||||||||||||||||||||||||||
Sbjct  722  AGCGTGGGGAGCAAACAGGATTAGATACCCTGGTAGTCCACGCCGTAAACGATGAATGCT  781

Query  786  AGGTGTTAGGGGTTTCGATACCCTTGGTGCCGAAGTTAACACATTAAGCATTCCGCCTGG  845
            ||||||||||||||||||||||||||||||||||||||||||||||||||||||||||||
Sbjct  782  AGGTGTTAGGGGTTTCGATACCCTTGGTGCCGAAGTTAACACATTAAGCATTCCGCCTGG  841

Query  846  GGAGTACGGTCGCAAGACTGAAACTCAAAGGAATTGACGGGGACCCGCACAAGCAGTGGA  905
            ||||||||||||||||||||||||||||||||||||||||||||||||||||||||||||
Sbjct  842  GGAGTACGGTCGCAAGACTGAAACTCAAAGGAATTGACGGGGACCCGCACAAGCAGTGGA  901

Query  906  GTATGTGGTTTAATTCGAAGCAACGCGAAGAACCTTACCAAGTCTTGACATCCCTTTGAC  965
            ||||||||||||||||||||||||||||||||||||||||||||||||||||||||||||
Sbjct  902  GTATGTGGTTTAATTCGAAGCAACGCGAAGAACCTTACCAAGTCTTGACATCCCTTTGAC  961

Query  966  CGGACTAGAGATAGTCTTTTCCTTCGGGACAAAGG  1000
            |||||||||||||||||||||||||||||||||||
Sbjct  962  CGGACTAGAGATAGTCTTTTCCTTCGGGACAAAGG  996
```

Download

FASTA (complete sequence)

FASTA (aligned sequences)

GenBank (complete sequence)

Continue
Cancel

GenBankGraphics

Next
Previous
Descriptions

Paenibacillus sp. TI45-13ar 16S ribosomal RNA gene, partial sequence

Sequence ID: KY419705.1Length: 1472Number of Matches: 1

Related Information

Range 1: 38 to 1033GenBankGraphics

Next Match
Previous Match
First Match

Alignment statistics for match #1

| Score | Expect | Identities | Gaps | Strand | Frame |
| --- | --- | --- | --- | --- | --- |
| 1790 bits(969) | 0.0() | 986/996(99%) | 1/996(0%) | Plus/Plus |  |

Features:

```
Query  6     GCANGTCGAGCGGAGTTGATGAGAAGCTTGCTTCTCGGATACTTAGCGGCGGACGGGTGA  65
             ||| |||||||||||||||||||||||||||||||||||| |||||||||||||||||||
Sbjct  38    GCAAGTCGAGCGGAGTTGATGAGAAGCTTGCTTCTCGGATRCTTAGCGGCGGACGGGTGA  97

Query  66    GTAACACGTAGGCAACCTGCCCTCAAGCTTGGGACAACTACCGGAAACGGTAGCTAATAC  125
             ||||||||||||||||||||||||||||||||||||||||||||||||||||||||||||
Sbjct  98    GTAACACGTAGGCAACCTGCCCTCAAGCTTGGGACAACTACCGGAAACGGTAGCTAATAC  157

Query  126   CGAATACATGATTTGTTCGCCTGAACGAATTTGGAAAGACGGAGCAATCTGTCACTTGAG  185
             ||||||||||||||||||||||||||||||||||||||||||||||||||||||||||||
Sbjct  158   CGAATACATGATTTGTTCGCCTGAACGAATTTGGAAAGACGGAGCAATCTGTCACTTGAG  217

Query  186   GATGGGCCTGCGGCGCATTAGCTAGTTGGTGAGGTAACGGCTCACCAAGGCGACGATGCG  245
             ||||||||||||||||||||||||||||||||||||||||||||||||||||||||||||
Sbjct  218   GATGGGCCTGCGGCGCATTAGCTAGTTGGTGAGGTAACGGCTCACCAAGGCGACGATGCG  277

Query  246   TAGCCGACCTGAGAGGGTGATCGGCCACACTGGGACTGAGACACGGCCCAGACTCCTACG  305
             ||||||||||||||||||||||||||||||||||||||||||||||||||||||||||||
Sbjct  278   TAGCCGACCTGAGAGGGTGATCGGCCACACTGGGACTGAGACACGGCCCAGACTCCTACG  337

Query  306   GGAGGCAGCAGTAGGGAATCTTCCGCAATGGACGCAAGTCTGACGGAGCAACGCCGCGTG  365
             ||||||||||||||||||||||||||||||||||||||||||||||||||||||||||||
Sbjct  338   GGAGGCAGCAGTAGGGAATCTTCCGCAATGGACGCAAGTCTGACGGAGCAACGCCGCGTG  397

Query  366   AGTGATGAAGGTTTTCGGATCGTAAAGCTCTGTTGCCAGGGAAGAACGTCCGGGTTAGTA  425
             ||||||||||||||||||||||||||||||||||||||||||||||||||||    ||||
Sbjct  398   AGTGATGAAGGTTTTCGGATCGTAAAGCTCTGTTGCCAGGGAAGAACGTCCGATAGAGTA  457

Query  426   ACTGNANTCGGAGTGACGGTACCTGAGAAGAAAGCCCCGGCTAACTACGTGCCAGCAGCC  485
             ||||   |||||||||||||||||||||||||||||||||||||||||||||||||||||
Sbjct  458   ACTGCTATCGGAGTGACGGTACCTGAGAAGAAAGCCCCGGCTAACTACGTGCCAGCAGCC  517

Query  486   GCGGTAATACGTAGGGGGCAAGCGTTGTCCGGAATTATTGGGCGTAAAGCGCGCGCAGGC  545
             ||||||||||||||||||||||||||||||||||||||||||||||||||||||||||||
Sbjct  518   GCGGTAATACGTAGGGGGCAAGCGTTGTCCGGAATTATTGGGCGTAAAGCGCGCGCAGGC  577

Query  546   GGCTTTTTAAGTCCGGTGTCACAGCCCAAGGCTCAACCTTGGGTCGCACTGGAAACTGGA  605
             ||||||||||||||||||||||||||||||||||||||||||||||||||||||||||||
Sbjct  578   GGCTTTTTAAGTCCGGTGTCACAGCCCAAGGCTCAACCTTGGGTCGCACTGGAAACTGGA  637

Query  606   GAGCTTGAGTACAGAAGAGGAAAGTG-GAATTCCACGTGTAGCGGTGAAATGCGTAGAGA  664
             |||||||||||||||||||||||||| |||||||||||||||||||||||||||||||||
Sbjct  638   GAGCTTGAGTACAGAAGAGGAAAGTGAGAATTCCACGTGTAGCGGTGAAATGCGTAGAGA  697

Query  665   TGTGGAGGAACACCAGTGGCGAAGGCGACTTTCTGGGCTGTAACTGACGCTGAGGCGCGA  724
             ||||||||||||||||||||||||||||||||||||||||||||||||||||||||||||
Sbjct  698   TGTGGAGGAACACCAGTGGCGAAGGCGACTTTCTGGGCTGTAACTGACGCTGAGGCGCGA  757

Query  725   AAGCGTGGGGAGCAAACAGGATTAGATACCCTGGTAGTCCACGCCGTAAACGATGAATGC  784
             ||||||||||||||||||||||||||||||||||||||||||||||||||||||||||||
Sbjct  758   AAGCGTGGGGAGCAAACAGGATTAGATACCCTGGTAGTCCACGCCGTAAACGATGAATGC  817

Query  785   TAGGTGTTAGGGGTTTCGATACCCTTGGTGCCGAAGTTAACACATTAAGCATTCCGCCTG  844
             ||||||||||||||||||||||||||||||||||||||||||||||||||||||||||||
Sbjct  818   TAGGTGTTAGGGGTTTCGATACCCTTGGTGCCGAAGTTAACACATTAAGCATTCCGCCTG  877

Query  845   GGGAGTACGGTCGCAAGACTGAAACTCAAAGGAATTGACGGGGACCCGCACAAGCAGTGG  904
             ||||||||||||||||||||||||||||||||||||||||||||||||||||||||||||
Sbjct  878   GGGAGTACGGTCGCAAGACTGAAACTCAAAGGAATTGACGGGGACCCGCACAAGCAGTGG  937

Query  905   AGTATGTGGTTTAATTCGAAGCAACGCGAAGAACCTTACCAAGTCTTGACATCCCTTTGA  964
             ||||||||||||||||||||||||||||||||||||||||||||||||||||||||||||
Sbjct  938   AGTATGTGGTTTAATTCGAAGCAACGCGAAGAACCTTACCAAGTCTTGACATCCCTTTGA  997

Query  965   CCGGACTAGAGATAGTCTTTTCCTTCGGGACAAAGG  1000
             ||||||||||||||||||||||||||||||||||||
Sbjct  998   CCGGACTAGAGATAGTCTTTTCCTTCGGGACAAAGG  1033
```


BLAST is a registered trademark of the National Library of Medicine

Support center
Mailing list


YouTube

- National Library Of Medicine
- National Institutes Of Health
- U.S. Department of Health & Human Services
- USA.gov

### NCBI


National Center for Biotechnology Information,
 U.S. National Library of Medicine

8600 Rockville Pike,
Bethesda
 MD,
20894
USA

Policies and Guidelines
|
Contact


PreferencesTurn off

External link. Please review our privacy policy.
