# Supplementary material for: Isothermal microcalorimetry for thermal viable count of microorganisms in pure cultures and stabilized formulations
Source: BMC Microbiol. 2019 Mar 21;19:65. doi: 10.1186/s12866-019-1432-8 (PMC6429831; doi:10.1186/s12866-019-1432-8)
Supplement: Supplementary file 3 — 16S rRNA gene sequences’ BLASTN hits in zipped HTML format. (ZIP 15810 kb) [file 12866_2019_1432_MOESM3_ESM.zip › Best blastn hits/NCBI Blast_F pink R -- 32..1029 of sequence (998 letters).html]

NCBI Blast:F pink R -- 32..1029 of sequence (998 letters)


- NCBI Home
- Sign in to NCBI
- Skip to Main Content
- Skip to Navigation
- About NCBI Accesskeys

U.S. National Library of Medicine

NCBI
National Center for Biotechnology Information

- My NCBI
- Sign in to NCBI
- Register
- Sign Out

BLAST ^®^ » blastn suite » RID-A4XTDMNG015


- Home
- Recent Results
- Saved Strategies
- Help

BLAST Results


Edit and Resubmit
Save Search Strategies
[Sign in above to save your search strategy]

Formatting options 


Download


How to read this page
Blast report description
Questions/comments


|  |  |
| --- | --- |
| Formatting options | |
| Show | Alignment as  HTML Plain text   Old View Reset form to defaults [?]  These options control formatting of alignments in results pages. The default is HTML, but other formats (including plain text) are available. PSSM and PssmWithParameters are representations of Position Specific Scoring Matrices and are only available for PSI-BLAST. The Advanced view option allows the database descriptions to be sorted by various indices in a table. |
| Alignment View | Pairwise Pairwise with dots for identities Query-anchored with dots for identities Query-anchored with letters for identities Flat query-anchored with dots for identities Flat query-anchored with letters for identities [?]  Choose how to view alignments. The default "pairwise" view shows how each subject sequence aligns individually to the query sequence. The "query-anchored" view shows how all subject sequences align to the query sequence. For each view type, you can choose to show "identities" (matching residues) as letters or dots. more... |
| Display | Graphical Overview   Linkout   Sequence Retrieval  NCBI-gi   CDS feature [?]  - Graphical Overview: Graphical Overview: Show graph of similar sequence regions aligned to query.   more... - NCBI-gi: Show NCBI gi identifiers. - CDS feature: Show annotated coding region and translation.   more... |
| Masking | Character:   X for protein, n for nucleotide Lower Case  Color:  Black Grey Red [?]  - Masking Character: Display masked (filtered) sequence regions as lower-case or as specific letters (N for nucleotide, P for protein). - Masking Color: Display masked sequence regions in the given color. |
| Limit results | Descriptions:  10 50 100 Graphical overview:  0 10 50 100  Alignments:  0 10 50 100 Line length:  60 90 120 150 [?]  - Descriptions: Show short descriptions for up to the given number of sequences. - Alignments: Show alignments for up to the given number of sequences, in order of statistical significance. - Line lenghth: Number of letters to show on one line in an alignment. |
|  | Organism Type common name, binomial, taxid, or group name. Only 20 top taxa will be shown.     Exclude   [?]  Show only sequences from the given organism. |
|  | Entrez query:  [?]  Show only those sequences that match the given Entrez query. more... |
|  | Expect Min:  Expect Max:  [?]  Show only sequences with expect values in the given range. more... |
|  | Percent Identity Min:  Percent Identity Max:  [?]  Show only sequences with percent identity values in the given range. |
| Format for | PSI-BLAST with inclusion threshold:  [?]  - Format for PSI-BLAST: The Position-Specific Iterated BLAST (PSI-BLAST) program performs iterative searches with a protein query,   in which sequences found in one round of search are used to build a custom score model for the next round.   more... - Inclusion Threshold: This sets the statistical significance threshold for including a sequence in the model used   by PSI-BLAST to create the PSSM on the next iteration. |

|  |  |  |  |  |  |
| --- | --- | --- | --- | --- | --- |
| Download | | | | | |
| Alignment  Text XML ASN.1 JSON Seq-align Hit Table(text) Hit Table(csv) Multiple-file XML2 Single-file XML2 Multiple-file JSON Single-file JSON SAM | Search Strategies  ASN.1 | PSSM to restart search  PSSM | [?] |  |  |

The Download link provides BLAST output that may be used as input to another program.
This includes parseable formats such as the tabular report or XML as well as the Search Strategy files read by the BLAST+ applications.
More details on the parseable (XML, tabular, and ASN.1) reports can be found at
https://www.ncbi.nlm.nih.gov/books/NBK153387/  
  

The following formats are offered under the Alignment section:  
1). "Text". Non-HTML standard BLAST report.  
2). "XML". XML report based upon the DTD at https://www.ncbi.nlm.nih.gov/data\_specs/dtd/NCBI\_BlastOutput.dtd  
3). "ASN.1". Alignment written out in Abstract Syntax Notation 1.  
4). "JSON Seq-align". Alignment written out in JSON.  
4). "Hit Table(text)". The tabular report as text.  
5). "Hit Table(csv)". The tabular report ready for import into spread-sheet programs like Excel.  
6). "XML2". New XML format described at ftp://ftp.ncbi.nlm.nih.gov/blast/documents/NEWXML/xml2.pdf.  
7). "JSON". New JSON format described at ftp://ftp.ncbi.nlm.nih.gov/blast/documents/NEWXML/xml2.pdf.  
8). "SAM". Sequence Alignment Map format.

XML2 and JSON can be downloaded either as one file per query (multiple-file) or one file for all queries (single-file). These formats are listed as Multiple-file XML2 (and JSON) or Single-file XML (and JSON).

The following report is offered under the Search Strategy section:  
1). "ASN.1" Search Strategy. A record of the parameters, query, and database used in the search. This file can be used to start a stand-alone BLAST search, see
https://www.ncbi.nlm.nih.gov/books/NBK1763/#CmdLineAppsManual.I455\_BLAST\_search\_stra


# Job title: F pink R -- 32..1029 of sequence (998 letters)

Results for:

lcl|Query\_215405 F pink R -- 32..1029 of sequence(998bp)
[?]

Your BLAST job specified more than one input sequence.
This box lets you choose which input sequence to show BLAST results for.

RID
:   A4XTDMNG015 (Expires on 03-10 18:59 pm)

Query ID
:   lcl|Query\_215405
:   lcl|Query\_215405

Description
:   F pink R -- 32..1029 of sequence

Molecule type
:   nucleic acid

Query Length
:   998

Database Name
:   nr

Description
:   Nucleotide collection (nt) See details

Program
:   BLASTN 2.8.0+ Citation

  

Reference 

Zheng Zhang, Scott Schwartz, Lukas Wagner, and Webb Miller (2000), "A greedy algorithm for aligning DNA sequences", J Comput Biol 2000; 7(1-2):203-14.

Reference - database indexing

Aleksandr Morgulis, George Coulouris, Yan Raytselis, Thomas L. Madden, Richa Agarwala, Alejandro A. Schäffer (2008), "Database Indexing for Production MegaBLAST Searches", Bioinformatics 24:1757-1764.

Other reports:
Search Summary

[Taxonomy reports]
[Distance tree of results]
[MSA viewer]

Search Parameters

| Search parameter name | Search parameter value |
| --- | --- |
| Program | blastn |
| Word size | 28 |
| Expect value | 10 |
| Hitlist size | 100 |
| Match/Mismatch scores | 1,-2 |
| Gapcosts | 0,2.5 |
| Low Complexity Filter | Yes |
| Filter string | L;m; |
| Genetic Code | 1 |

Database

| Database parameter name | Database parameter value |
| --- | --- |
| Posted date | Mar 7, 2018 1:58 PM |
| Number of letters | 174,044,644,244 |
| Number of sequences | 46,882,714 |
| Entrez query | Includes:  Excludes:  None |

Karlin-Altschul statistics

| Params | Ungapped | Gapped |
| --- | --- | --- |
| Lambda | 1.33271 | 1.28 |
| K | 0.620991 | 0.46 |
| H | 1.12409 | 0.85 |

Results Statistics

| Results Statistics parameter name | Results Statistics parameter value |
| --- | --- |
| Length adjustment | 35 |
| Effective length of query | 963 |
| Effective length of database | 172403749254 |
| Effective search space | 166024810531602 |
| Effective search space used | 166024810531602 |


## Graphic Summary

### Distribution of the top 173 Blast Hits on 100 subject sequences [?]

The graphic is an overview of the database sequences aligned to the query sequence. These are represented horizontal bars colored coded by score and showing the extent
of the alignment on the query sequence. Separate aligned regions on the same database sequence are connected by a thin grey line.
Mousing over an alignment shows the database sequence title. Clicking an alignment displays a box with more details about the alignment and
link to the sequence alignment itself in the Alignments section of the report.

Mouse over to see the title, click to show alignments

Color key for alignment scores

<40

40-50

50-80

80-200

>=200

Query

1

150

300

450

600

750

900

Bacterium 4R1 16S ribosomal RNA gene, partial sequence

Score:1810 Evalue:0

Accession:KP965572.1

Alignment

Uncultured bacterium clone 10-9BM 16S ribosomal RNA gen..

Score:1805 Evalue:0

Accession:HQ143281.1

Alignment

Bacterium 4L18 16S ribosomal RNA gene, partial sequence

Score:1801 Evalue:0

Accession:KP965574.1

Alignment

Bacterium 4L13 16S ribosomal RNA gene, partial sequence

Score:1801 Evalue:0

Accession:KP965573.1

Alignment

Paenibacillus sp. strain BSW35 16S ribosomal RNA gene, ..

Score:1799 Evalue:0

Accession:KY446062.1

Alignment

Paenibacillus sp. Rs5 16S ribosomal RNA gene, partial s..

Score:1796 Evalue:0

Accession:JQ977193.1

Alignment

Paenibacillus sp. Q1-R10 16S ribosomal RNA gene, partia..

Score:1794 Evalue:0

Accession:JX994123.1

Alignment

Paenibacillus sp. strain PS-K-17 16S ribosomal RNA gene..

Score:1792 Evalue:0

Accession:MF443394.1

Alignment

Paenibacillus sp. 3Cp1 16S ribosomal RNA gene, partial ..

Score:1792 Evalue:0

Accession:KP852522.1

Alignment

Uncultured bacterium clone 4L13 16S ribosomal RNA gene,..

Score:1792 Evalue:0

Accession:KP843054.1

Alignment

Paenibacillus sp. TI45-13ar 16S ribosomal RNA gene, par..

Score:1786 Evalue:0

Accession:KY419705.1

Alignment

Bacterium 4R9 16S ribosomal RNA gene, partial sequence

Score:1781 Evalue:0

Accession:KP965575.1

Alignment

Paenibacillus illinoisensis strain WR\_17 16S ribosomal ..

Score:1781 Evalue:0

Accession:HQ661869.1

Alignment

Paenibacillus sp. HDDMM03 16S ribosomal RNA gene, parti..

Score:1781 Evalue:0

Accession:EU723825.1

Alignment

Paenibacillus sp. NCCB 100172 16S ribosomal RNA gene, p..

Score:1777 Evalue:0

Accession:EU003588.1

Alignment

Paenibacillus sp. R1 16S ribosomal RNA gene, partial se..

Score:1773 Evalue:0

Accession:KR263165.1

Alignment

Paenibacillus kyungheensis strain DCY88 16S ribosomal R..

Score:1770 Evalue:0

Accession:NR\_145628.1

Alignment

Paenibacillus kyungheensis strain DCY88 16S ribosomal R..

Score:1770 Evalue:0

Accession:KF793934.1

Alignment

Paenibacillus sp. 9N4 16S ribosomal RNA gene, partial s..

Score:1768 Evalue:0

Accession:HQ284889.1

Alignment

Paenibacillus nicotianae strain YIM h-19 16S ribosomal ..

Score:1760 Evalue:0

Accession:NR\_134783.2

Alignment

Paenibacillus nicotianae strain YIM h-19 16S ribosomal ..

Score:1760 Evalue:0

Accession:KJ792471.1

Alignment

Paenibacillus hordei strain RH-N24 16S ribosomal RNA ge..

Score:1751 Evalue:0

Accession:NR\_109318.1

Alignment

Uncultured bacterium clone ncd2164d05c1 16S ribosomal R..

Score:1720 Evalue:0

Accession:JF185937.1

Alignment

Uncultured bacterium clone ncd2162h02c1 16S ribosomal R..

Score:1720 Evalue:0

Accession:JF185833.1

Alignment

Uncultured bacterium clone ncd2163a03c1 16S ribosomal R..

Score:1714 Evalue:0

Accession:JF185844.1

Alignment

Uncultured bacterium clone nbw208h09c1 16S ribosomal RN..

Score:1703 Evalue:0

Accession:GQ073426.1

Alignment

Uncultured bacterium clone 56-9BM 16S ribosomal RNA gen..

Score:1696 Evalue:0

Accession:HQ143315.1

Alignment

Paenibacillus kyungheensis strain R2abR 16S ribosomal R..

Score:1633 Evalue:0

Accession:MG607391.1

Alignment

Paenibacillus sp. TA\_EU partial 16S rRNA gene, strain T..

Score:1633 Evalue:0

Accession:HG942127.1

Alignment

Paenibacillus peoriae strain DSM 8320 16S ribosomal RNA..

Score:1615 Evalue:0

Accession:NR\_117740.1

Alignment

Paenibacillus polymyxa partial 16S rRNA gene, strain KC..

Score:1613 Evalue:0

Accession:HE981792.1

Alignment

Paenibacillus polymyxa partial 16S rRNA gene, strain KC..

Score:1613 Evalue:0

Accession:HE981768.1

Alignment

Paenibacillus polymyxa strain J, complete genome

Score:1609 Evalue:0

Accession:CP015423.1

Alignment

Uncultured bacterium clone HR-R2W6B52 16S ribosomal RNA..

Score:1609 Evalue:0

Accession:KM507259.1

Alignment

Paenibacillus sp. GA5 16S ribosomal RNA gene, partial s..

Score:1609 Evalue:0

Accession:DQ358734.1

Alignment

Paenibacillus sp. SP47B 16S ribosomal RNA gene, partial..

Score:1607 Evalue:0

Accession:KP126652.1

Alignment

Paenibacillus polymyxa strain DSM 36 16S ribosomal RNA,..

Score:1607 Evalue:0

Accession:NR\_117727.2

Alignment

Paenibacillus polymyxa strain DSM 36 16S ribosomal RNA,..

Score:1607 Evalue:0

Accession:NR\_117726.2

Alignment

Paenibacillus polymyxa partial 16S rRNA gene, type stra..

Score:1607 Evalue:0

Accession:HG324071.1

Alignment

Paenibacillus polymyxa partial 16S rRNA gene, type stra..

Score:1607 Evalue:0

Accession:HG324070.1

Alignment

Paenibacillus jamilae partial 16S rRNA gene, strain KCT..

Score:1607 Evalue:0

Accession:HE981796.1

Alignment

Paenibacillus polymyxa partial 16S rRNA gene, strain KC..

Score:1607 Evalue:0

Accession:HE981788.1

Alignment

Paenibacillus polymyxa strain HX-140 16S ribosomal RNA ..

Score:1604 Evalue:0

Accession:MF136611.1

Alignment

Paenibacillus sp. strain YN-3 16S ribosomal RNA gene, p..

Score:1604 Evalue:0

Accession:MF967298.1

Alignment

Paenibacillus sp. strain BJ-7 16S ribosomal RNA gene, p..

Score:1604 Evalue:0

Accession:MF967288.1

Alignment

Paenibacillus peoriae strain GQJK57 16S ribosomal RNA g..

Score:1604 Evalue:0

Accession:KY952711.1

Alignment

Paenibacillus polymyxa strain TS683 16S ribosomal RNA g..

Score:1604 Evalue:0

Accession:KU860093.1

Alignment

Paenibacillus polymyxa strain TS682 16S ribosomal RNA g..

Score:1604 Evalue:0

Accession:KU860092.1

Alignment

Paenibacillus polymyxa strain TS681 16S ribosomal RNA g..

Score:1604 Evalue:0

Accession:KU860091.1

Alignment

Paenibacillus polymyxa strain ATCC 15970, complete geno..

Score:1604 Evalue:0

Accession:CP011420.1

Alignment

Paenibacillus sp. strain Avl-2 16S ribosomal RNA gene, ..

Score:1604 Evalue:0

Accession:KX907738.1

Alignment

Paenibacillus polymyxa strain JK1 16S ribosomal RNA gen..

Score:1604 Evalue:0

Accession:KU563771.1

Alignment

Paenibacillus polymyxa partial 16S rRNA gene, strain Ma..

Score:1604 Evalue:0

Accession:LT223607.1

Alignment

Paenibacillus polymyxa strain J 16S ribosomal RNA gene,..

Score:1604 Evalue:0

Accession:KT783525.1

Alignment

Bacterium YTM092504 from China 16S ribosomal RNA gene, ..

Score:1604 Evalue:0

Accession:KR029816.1

Alignment

Paenibacillus polymyxa strain LY214 16S ribosomal RNA g..

Score:1604 Evalue:0

Accession:KR058350.1

Alignment

## Descriptions

, Reading indexes 1-5, displaying indexes 1-5


Load next setPrevious Match

Sequences producing significant alignments:

Show all columns  of the table presenting sequences producing significant alignments 

Select:AllNone
Selected:0

Alignments
Download

FASTA (complete sequence)

FASTA (aligned sequences)

GenBank (complete sequence)

Hit Table (text)

Hit Table (CSV)

Text

XML

ASN.1

Continue
Cancel

GenBank 
Graphics
Distance tree of results
Multiple alignment
Show/hide columns of the table presenting sequences producing significant alignments 

Available columns

Description  
Max Score  
Total Score  
Coverage  
E-value  
IdentN  
Accession  
Restore Defaults
Ok
Cancel

Sequences producing significant alignments:

| Select for downloading or viewing reports | Description | Max score | Total score | Query cover | E value | Ident | Accession |
| --- | --- | --- | --- | --- | --- | --- | --- |
| 1Select seq KP965572.1 | Bacterium 4R1 16S ribosomal RNA gene, partial sequence | 1810 | 1810 | 98% | 0.0 | 99% | KP965572.1 |
| 2Select seq HQ143281.1 | Uncultured bacterium clone 10-9BM 16S ribosomal RNA gene, partial sequence | 1805 | 1805 | 98% | 0.0 | 99% | HQ143281.1 |
| 3Select seq KP965574.1 | Bacterium 4L18 16S ribosomal RNA gene, partial sequence | 1801 | 1801 | 98% | 0.0 | 99% | KP965574.1 |
| 4Select seq KP965573.1 | Bacterium 4L13 16S ribosomal RNA gene, partial sequence | 1801 | 1801 | 98% | 0.0 | 99% | KP965573.1 |
| 5Select seq KY446062.1 | Paenibacillus sp. strain BSW35 16S ribosomal RNA gene, partial sequence | 1799 | 1799 | 98% | 0.0 | 99% | KY446062.1 |
| 6Select seq JQ977193.1 | Paenibacillus sp. Rs5 16S ribosomal RNA gene, partial sequence | 1796 | 1796 | 98% | 0.0 | 99% | JQ977193.1 |
| 7Select seq JX994123.1 | Paenibacillus sp. Q1-R10 16S ribosomal RNA gene, partial sequence | 1794 | 1794 | 98% | 0.0 | 99% | JX994123.1 |
| 8Select seq MF443394.1 | Paenibacillus sp. strain PS-K-17 16S ribosomal RNA gene, partial sequence | 1792 | 1792 | 98% | 0.0 | 99% | MF443394.1 |
| 9Select seq KP852522.1 | Paenibacillus sp. 3Cp1 16S ribosomal RNA gene, partial sequence | 1792 | 1792 | 98% | 0.0 | 99% | KP852522.1 |
| 10Select seq KP843054.1 | Uncultured bacterium clone 4L13 16S ribosomal RNA gene, partial sequence | 1792 | 1792 | 98% | 0.0 | 99% | KP843054.1 |
| 11Select seq KY419705.1 | Paenibacillus sp. TI45-13ar 16S ribosomal RNA gene, partial sequence | 1786 | 1786 | 98% | 0.0 | 99% | KY419705.1 |
| 12Select seq KP965575.1 | Bacterium 4R9 16S ribosomal RNA gene, partial sequence | 1781 | 1781 | 98% | 0.0 | 99% | KP965575.1 |
| 13Select seq HQ661869.1 | Paenibacillus illinoisensis strain WR\_17 16S ribosomal RNA gene, partial sequence | 1781 | 1781 | 96% | 0.0 | 99% | HQ661869.1 |
| 14Select seq EU723825.1 | Paenibacillus sp. HDDMM03 16S ribosomal RNA gene, partial sequence | 1781 | 1781 | 98% | 0.0 | 99% | EU723825.1 |
| 15Select seq EU003588.1 | Paenibacillus sp. NCCB 100172 16S ribosomal RNA gene, partial sequence | 1777 | 1777 | 98% | 0.0 | 99% | EU003588.1 |
| 16Select seq KR263165.1 | Paenibacillus sp. R1 16S ribosomal RNA gene, partial sequence | 1773 | 1773 | 98% | 0.0 | 99% | KR263165.1 |
| 17Select seq NR\_145628.1 | Paenibacillus kyungheensis strain DCY88 16S ribosomal RNA, partial sequence | 1770 | 1770 | 98% | 0.0 | 99% | NR\_145628.1 |
| 18Select seq KF793934.1 | Paenibacillus kyungheensis strain DCY88 16S ribosomal RNA gene, partial sequence | 1770 | 1770 | 98% | 0.0 | 99% | KF793934.1 |
| 19Select seq HQ284889.1 | Paenibacillus sp. 9N4 16S ribosomal RNA gene, partial sequence | 1768 | 1768 | 98% | 0.0 | 99% | HQ284889.1 |
| 20Select seq NR\_134783.2 | Paenibacillus nicotianae strain YIM h-19 16S ribosomal RNA, complete sequence | 1760 | 1760 | 98% | 0.0 | 99% | NR\_134783.2 |
| 21Select seq KJ792471.1 | Paenibacillus nicotianae strain YIM h-19 16S ribosomal RNA gene, partial sequence | 1760 | 1760 | 98% | 0.0 | 99% | KJ792471.1 |
| 22Select seq NR\_109318.1 | Paenibacillus hordei strain RH-N24 16S ribosomal RNA gene, partial sequence | 1751 | 1751 | 98% | 0.0 | 99% | NR\_109318.1 |
| 23Select seq JF185937.1 | Uncultured bacterium clone ncd2164d05c1 16S ribosomal RNA gene, partial sequence | 1720 | 1720 | 93% | 0.0 | 99% | JF185937.1 |
| 24Select seq JF185833.1 | Uncultured bacterium clone ncd2162h02c1 16S ribosomal RNA gene, partial sequence | 1720 | 1720 | 93% | 0.0 | 99% | JF185833.1 |
| 25Select seq JF185844.1 | Uncultured bacterium clone ncd2163a03c1 16S ribosomal RNA gene, partial sequence | 1714 | 1714 | 93% | 0.0 | 99% | JF185844.1 |
| 26Select seq GQ073426.1 | Uncultured bacterium clone nbw208h09c1 16S ribosomal RNA gene, partial sequence | 1703 | 1703 | 93% | 0.0 | 99% | GQ073426.1 |
| 27Select seq HQ143315.1 | Uncultured bacterium clone 56-9BM 16S ribosomal RNA gene, partial sequence | 1696 | 1696 | 94% | 0.0 | 99% | HQ143315.1 |
| 28Select seq MG607391.1 | Paenibacillus kyungheensis strain R2abR 16S ribosomal RNA gene, partial sequence | 1633 | 1633 | 89% | 0.0 | 99% | MG607391.1 |
| 29Select seq HG942127.1 | Paenibacillus sp. TA\_EU partial 16S rRNA gene, strain TA\_EU | 1633 | 1633 | 89% | 0.0 | 99% | HG942127.1 |
| 30Select seq NR\_117740.1 | Paenibacillus peoriae strain DSM 8320 16S ribosomal RNA gene, partial sequence | 1615 | 1615 | 96% | 0.0 | 97% | NR\_117740.1 |
| 31Select seq HE981792.1 | Paenibacillus polymyxa partial 16S rRNA gene, strain KCTC 3627, clone 27 | 1613 | 1613 | 98% | 0.0 | 96% | HE981792.1 |
| 32Select seq HE981768.1 | Paenibacillus polymyxa partial 16S rRNA gene, strain KCTC 3627, clone 3 | 1613 | 1613 | 96% | 0.0 | 97% | HE981768.1 |
| 33Select seq CP015423.1 | Paenibacillus polymyxa strain J, complete genome | 1609 | 22445 | 96% | 0.0 | 97% | CP015423.1 |
| 34Select seq KM507259.1 | Uncultured bacterium clone HR-R2W6B52 16S ribosomal RNA gene, partial sequence | 1609 | 1609 | 88% | 0.0 | 99% | KM507259.1 |
| 35Select seq DQ358734.1 | Paenibacillus sp. GA5 16S ribosomal RNA gene, partial sequence | 1609 | 1609 | 96% | 0.0 | 97% | DQ358734.1 |
| 36Select seq KP126652.1 | Paenibacillus sp. SP47B 16S ribosomal RNA gene, partial sequence | 1607 | 1607 | 98% | 0.0 | 96% | KP126652.1 |
| 37Select seq NR\_117727.2 | Paenibacillus polymyxa strain DSM 36 16S ribosomal RNA, partial sequence | 1607 | 1607 | 96% | 0.0 | 97% | NR\_117727.2 |
| 38Select seq NR\_117726.2 | Paenibacillus polymyxa strain DSM 36 16S ribosomal RNA, partial sequence | 1607 | 1607 | 96% | 0.0 | 97% | NR\_117726.2 |
| 39Select seq HG324071.1 | Paenibacillus polymyxa partial 16S rRNA gene, type strain DSM 36T, clone 8 | 1607 | 1607 | 96% | 0.0 | 97% | HG324071.1 |
| 40Select seq HG324070.1 | Paenibacillus polymyxa partial 16S rRNA gene, type strain DSM 36T, clone 7 | 1607 | 1607 | 96% | 0.0 | 97% | HG324070.1 |
| 41Select seq HE981796.1 | Paenibacillus jamilae partial 16S rRNA gene, strain KCTC 13919, clone 4 | 1607 | 1607 | 96% | 0.0 | 97% | HE981796.1 |
| 42Select seq HE981788.1 | Paenibacillus polymyxa partial 16S rRNA gene, strain KCTC 3627, clone 23 | 1607 | 1607 | 96% | 0.0 | 97% | HE981788.1 |
| 43Select seq MF136611.1 | Paenibacillus polymyxa strain HX-140 16S ribosomal RNA gene, partial sequence | 1604 | 1604 | 96% | 0.0 | 97% | MF136611.1 |
| 44Select seq MF967298.1 | Paenibacillus sp. strain YN-3 16S ribosomal RNA gene, partial sequence | 1604 | 1604 | 96% | 0.0 | 97% | MF967298.1 |
| 45Select seq MF967288.1 | Paenibacillus sp. strain BJ-7 16S ribosomal RNA gene, partial sequence | 1604 | 1604 | 96% | 0.0 | 97% | MF967288.1 |
| 46Select seq KY952711.1 | Paenibacillus peoriae strain GQJK57 16S ribosomal RNA gene, partial sequence | 1604 | 1604 | 96% | 0.0 | 97% | KY952711.1 |
| 47Select seq KU860093.1 | Paenibacillus polymyxa strain TS683 16S ribosomal RNA gene, partial sequence | 1604 | 1604 | 96% | 0.0 | 97% | KU860093.1 |
| 48Select seq KU860092.1 | Paenibacillus polymyxa strain TS682 16S ribosomal RNA gene, partial sequence | 1604 | 1604 | 96% | 0.0 | 97% | KU860092.1 |
| 49Select seq KU860091.1 | Paenibacillus polymyxa strain TS681 16S ribosomal RNA gene, partial sequence | 1604 | 1604 | 96% | 0.0 | 97% | KU860091.1 |
| 50Select seq CP011420.1 | Paenibacillus polymyxa strain ATCC 15970, complete genome | 1604 | 20769 | 96% | 0.0 | 97% | CP011420.1 |
| 51Select seq KX907738.1 | Paenibacillus sp. strain Avl-2 16S ribosomal RNA gene, partial sequence | 1604 | 1604 | 96% | 0.0 | 97% | KX907738.1 |
| 52Select seq KU563771.1 | Paenibacillus polymyxa strain JK1 16S ribosomal RNA gene, partial sequence | 1604 | 1604 | 96% | 0.0 | 97% | KU563771.1 |
| 53Select seq LT223607.1 | Paenibacillus polymyxa partial 16S rRNA gene, strain Marseille-AA00092 | 1604 | 1604 | 96% | 0.0 | 97% | LT223607.1 |
| 54Select seq KT783525.1 | Paenibacillus polymyxa strain J 16S ribosomal RNA gene, partial sequence | 1604 | 1604 | 96% | 0.0 | 97% | KT783525.1 |
| 55Select seq KR029816.1 | Bacterium YTM092504 from China 16S ribosomal RNA gene, partial sequence | 1604 | 1604 | 98% | 0.0 | 96% | KR029816.1 |
| 56Select seq KR058350.1 | Paenibacillus polymyxa strain LY214 16S ribosomal RNA gene, partial sequence | 1604 | 1604 | 96% | 0.0 | 97% | KR058350.1 |
| 57Select seq KJ767388.1 | Paenibacillus peoriae strain IHB B 12520 16S ribosomal RNA gene, partial sequence | 1604 | 1604 | 96% | 0.0 | 97% | KJ767388.1 |
| 58Select seq KF441710.1 | Paenibacillus sp. 7B-896 16S ribosomal RNA gene, partial sequence | 1604 | 1604 | 96% | 0.0 | 97% | KF441710.1 |
| 59Select seq NR\_117739.1 | Paenibacillus peoriae strain DSM 8320 16S ribosomal RNA gene, partial sequence | 1604 | 1604 | 96% | 0.0 | 97% | NR\_117739.1 |
| 60Select seq KF484688.1 | Paenibacillus kribbensis strain NF59-3 16S ribosomal RNA gene, partial sequence | 1604 | 1604 | 98% | 0.0 | 96% | KF484688.1 |
| 61Select seq KC581713.1 | Paenibacillus sp. LXfos16 16S ribosomal RNA gene, partial sequence | 1604 | 1604 | 96% | 0.0 | 97% | KC581713.1 |
| 62Select seq KC236521.1 | Paenibacillus sp. 2083 16S ribosomal RNA gene, partial sequence | 1604 | 1604 | 96% | 0.0 | 97% | KC236521.1 |
| 63Select seq JX517259.1 | Paenibacillus polymyxa strain A1-25c-13 16S ribosomal RNA gene, partial sequence | 1604 | 1604 | 96% | 0.0 | 97% | JX517259.1 |
| 64Select seq JN409465.1 | Paenibacillus polymyxa strain SP16 16S ribosomal RNA gene, partial sequence | 1604 | 1604 | 96% | 0.0 | 97% | JN409465.1 |
| 65Select seq HQ844466.1 | Paenibacillus peoriae strain HLJFQ25 16S ribosomal RNA gene, partial sequence | 1604 | 1604 | 96% | 0.0 | 97% | HQ844466.1 |
| 66Select seq JF970587.1 | Paenibacillus peoriae 16S ribosomal RNA gene, partial sequence | 1604 | 1604 | 96% | 0.0 | 97% | JF970587.1 |
| 67Select seq JF747221.1 | Paenibacillus polymyxa strain S 16S ribosomal RNA gene, partial sequence | 1604 | 1604 | 96% | 0.0 | 97% | JF747221.1 |
| 68Select seq JF683620.1 | Paenibacillus polymyxa strain RS-10 16S ribosomal RNA gene, partial sequence | 1604 | 1604 | 96% | 0.0 | 97% | JF683620.1 |
| 69Select seq GU328691.1 | Paenibacillus sp. Sx52 16S ribosomal RNA gene, partial sequence | 1604 | 1604 | 96% | 0.0 | 97% | GU328691.1 |
| 70Select seq EU362611.1 | Paenibacillus polymyxa isolate TN99 16S ribosomal RNA gene, partial sequence | 1604 | 1604 | 96% | 0.0 | 97% | EU362611.1 |
| 71Select seq EU362610.1 | Paenibacillus polymyxa isolate TN98 16S ribosomal RNA gene, partial sequence | 1604 | 1604 | 96% | 0.0 | 97% | EU362610.1 |
| 72Select seq AM900509.1 | Paenibacillus sp. 14A-7 partial 16S rRNA gene, strain 14A-7 | 1604 | 1604 | 96% | 0.0 | 97% | AM900509.1 |
| 73Select seq EF634024.1 | Paenibacillus polymyxa strain ISSDS-851 16S ribosomal RNA gene, partial sequence | 1604 | 1604 | 96% | 0.0 | 97% | EF634024.1 |
| 74Select seq EF620468.1 | Paenibacillus polymyxa strain ISSDS-792 16S ribosomal RNA gene, partial sequence | 1604 | 1604 | 96% | 0.0 | 97% | EF620468.1 |
| 75Select seq CP025696.1 | Paenibacillus sp. lzh-N1 chromosome, complete genome | 1602 | 23899 | 96% | 0.0 | 97% | CP025696.1 |
| 76Select seq KR708857.1 | Paenibacillus polymyxa strain HGPJ-5 16S ribosomal RNA gene, partial sequence | 1602 | 1602 | 98% | 0.0 | 96% | KR708857.1 |
| 77Select seq NR\_117732.2 | Paenibacillus polymyxa strain DSM 36 16S ribosomal RNA, partial sequence | 1602 | 1602 | 96% | 0.0 | 97% | NR\_117732.2 |
| 78Select seq NR\_117722.2 | Paenibacillus polymyxa strain DSM 36 16S ribosomal RNA, partial sequence | 1602 | 1602 | 98% | 0.0 | 96% | NR\_117722.2 |
| 79Select seq CP009909.1 | Paenibacillus polymyxa strain CF05 genome | 1602 | 22310 | 96% | 0.0 | 97% | CP009909.1 |
| 80Select seq CP006872.1 | Paenibacillus polymyxa SQR-21, complete genome | 1602 | 20530 | 98% | 0.0 | 97% | CP006872.1 |
| 81Select seq HG324076.1 | Paenibacillus polymyxa partial 16S rRNA gene, type strain DSM 36T, clone 13 | 1602 | 1602 | 96% | 0.0 | 97% | HG324076.1 |
| 82Select seq HG324066.1 | Paenibacillus polymyxa partial 16S rRNA gene, type strain DSM 36T, clone 3 | 1602 | 1602 | 98% | 0.0 | 96% | HG324066.1 |
| 83Select seq JX849658.1 | Paenibacillus polymyxa strain Jaas ed2 16S ribosomal RNA gene, partial sequence | 1602 | 1602 | 98% | 0.0 | 96% | JX849658.1 |
| 84Select seq JX498909.1 | Paenibacillus jamilae strain NBI 16S ribosomal RNA gene, partial sequence | 1602 | 1602 | 96% | 0.0 | 97% | JX498909.1 |
| 85Select seq HE981782.1 | Paenibacillus polymyxa partial 16S rRNA gene, strain KCTC 3627, clone 17 | 1602 | 1602 | 96% | 0.0 | 97% | HE981782.1 |
| 86Select seq HE981780.1 | Paenibacillus polymyxa partial 16S rRNA gene, strain KCTC 3627, clone 15 | 1602 | 1602 | 96% | 0.0 | 97% | HE981780.1 |
| 87Select seq HE981777.1 | Paenibacillus polymyxa partial 16S rRNA gene, strain KCTC 3627, clone 12 | 1602 | 1602 | 96% | 0.0 | 97% | HE981777.1 |
| 88Select seq HE981776.1 | Paenibacillus polymyxa partial 16S rRNA gene, strain KCTC 3627, clone 11 | 1602 | 1602 | 96% | 0.0 | 97% | HE981776.1 |
| 89Select seq HE981773.1 | Paenibacillus polymyxa partial 16S rRNA gene, strain KCTC 3627, clone 8 | 1602 | 1602 | 98% | 0.0 | 96% | HE981773.1 |
| 90Select seq HE981772.1 | Paenibacillus polymyxa partial 16S rRNA gene, strain KCTC 3627, clone 7 | 1602 | 1602 | 98% | 0.0 | 96% | HE981772.1 |
| 91Select seq CP020028.1 | Paenibacillus kribbensis strain AM49, complete genome | 1600 | 15914 | 96% | 0.0 | 97% | CP020028.1 |
| 92Select seq KX929058.1 | Paenibacillus polymyxa strain QH-G 16S ribosomal RNA gene, partial sequence | 1600 | 1600 | 98% | 0.0 | 96% | KX929058.1 |
| 93Select seq KU312055.1 | Bacillus sp. JZ212 16S ribosomal RNA gene, partial sequence | 1600 | 1600 | 96% | 0.0 | 96% | KU312055.1 |
| 94Select seq KJ756541.1 | Paenibacillus sp. EB-111 16S ribosomal RNA gene, partial sequence | 1600 | 1600 | 96% | 0.0 | 97% | KJ756541.1 |
| 95Select seq AB733541.1 | Paenibacillus sp. MBEB48 gene for 16S rRNA, partial sequence | 1600 | 1600 | 96% | 0.0 | 97% | AB733541.1 |
| 96Select seq HQ844465.1 | Paenibacillus brasilensis strain HLJFQ23 16S ribosomal RNA gene, partial sequence | 1600 | 1600 | 96% | 0.0 | 96% | HQ844465.1 |
| 97Select seq EF620470.1 | Paenibacillus polymyxa strain ISSDS-796 16S ribosomal RNA gene, partial sequence | 1600 | 1600 | 96% | 0.0 | 96% | EF620470.1 |
| 98Select seq EF108320.1 | Paenibacillus polymyxa strain DY1 16S ribosomal RNA gene, partial sequence | 1600 | 1600 | 96% | 0.0 | 96% | EF108320.1 |
| 99Select seq JQ279510.1 | Paenibacillus polymyxa strain AB15 16S ribosomal RNA gene, partial sequence | 1596 | 1596 | 96% | 0.0 | 96% | JQ279510.1 |
| 100Select seq AB689757.1 | Paenibacillus polymyxa gene for 16S ribosomal RNA, partial sequence | 1596 | 1596 | 96% | 0.0 | 96% | AB689757.1 |


## Alignments

Loading alignment... for sequences gi|846924041,gi|319655369,gi|846924088,gi|846924061,gi|1196644317 Reading indexes 1-5

Download

FASTA (complete sequence)

FASTA (aligned sequences)

GenBank (complete sequence)

Continue
Cancel

GenBankGraphics

Next
Previous
Descriptions

Bacterium 4R1 16S ribosomal RNA gene, partial sequence

Sequence ID: KP965572.1Length: 1516Number of Matches: 1

Related Information

Range 1: 459 to 1446GenBankGraphics

Next Match
Previous Match
First Match

Alignment statistics for match #1

| Score | Expect | Identities | Gaps | Strand | Frame |
| --- | --- | --- | --- | --- | --- |
| 1810 bits(980) | 0.0() | 985/988(99%) | 0/988(0%) | Plus/Minus |  |

Features:

```
Query  9     CCACCGACTTCGGGTGTTGCAAACTCTCGTGGTGTGACGGGCGGTGTGTACAAGACCCGG  68
             ||||||||||||||||||||||||||||||||||||||||||||||||||||||||||||
Sbjct  1446  CCACCGACTTCGGGTGTTGCAAACTCTCGTGGTGTGACGGGCGGTGTGTACAAGACCCGG  1387

Query  69    GAACGTATTCACCGCGGCATGCTGATCCGCGATTACTAGCAATTCCGACTTCATGCAGGC  128
             ||||||||||||||||||||||||||||||||||||||||||||||||||||||| ||||
Sbjct  1386  GAACGTATTCACCGCGGCATGCTGATCCGCGATTACTAGCAATTCCGACTTCATGTAGGC  1327

Query  129   GAGTTGCAGCCTGCAATCCGAACTGAGACCGGCTTTTAAGGATTGGCTCCATCTTGCGAT  188
             ||||||||||||||||||||||||||||||||||||||||||||||||||||||||||||
Sbjct  1326  GAGTTGCAGCCTGCAATCCGAACTGAGACCGGCTTTTAAGGATTGGCTCCATCTTGCGAT  1267

Query  189   ATTGCTTCCCGTTGTACCGGCCATTGTAGTACGTGTGTAGCCCAAGTCATAAGGGGCATG  248
             |||||||||||||||||||||||||||||||||||||||||||| |||||||||||||||
Sbjct  1266  ATTGCTTCCCGTTGTACCGGCCATTGTAGTACGTGTGTAGCCCAGGTCATAAGGGGCATG  1207

Query  249   ATGATTTGACGTCATCCCCACCTTCCTCCGGTTTGTCACCGGCAGTCTGCTTAGAGTGCC  308
             ||||||||||||||||||||||||||||||||||||||||||||||||||||||||||||
Sbjct  1206  ATGATTTGACGTCATCCCCACCTTCCTCCGGTTTGTCACCGGCAGTCTGCTTAGAGTGCC  1147

Query  309   CACCATGATGTGCTGGCAACTAAGCATAAGGGTTGCGCTCGTTGCGGGACTTAACCCAAC  368
             ||||||||||||||||||||||||||||||||||||||||||||||||||||||||||||
Sbjct  1146  CACCATGATGTGCTGGCAACTAAGCATAAGGGTTGCGCTCGTTGCGGGACTTAACCCAAC  1087

Query  369   ATCTCACGACACGAGCTGACGACAACCATGCACCACCTGTCTCCTTTGTCCCGAAGGAAA  428
             ||||||||||||||||||||||||||||||||||||||||||||||||||||||||||||
Sbjct  1086  ATCTCACGACACGAGCTGACGACAACCATGCACCACCTGTCTCCTTTGTCCCGAAGGAAA  1027

Query  429   AGACTATCTCTAGTCCGGTCAAAGGGATGTCAAGACTTGGTAAGGTTCTTCGCGTTGCTT  488
             ||||||||||||||||||||||||||||||||||||||||||||||||||||||||||||
Sbjct  1026  AGACTATCTCTAGTCCGGTCAAAGGGATGTCAAGACTTGGTAAGGTTCTTCGCGTTGCTT  967

Query  489   CGAATTAAACCACATACTCCACTGCTTGTGCGGGTCCCCGTCAATTCCTTTGAGTTTCAG  548
             ||||||||||||||||||||||||||||||||||||||||||||||||||||||||||||
Sbjct  966   CGAATTAAACCACATACTCCACTGCTTGTGCGGGTCCCCGTCAATTCCTTTGAGTTTCAG  907

Query  549   TCTTGCGACCGTACTCCCCAGGCGGAATGCTTAATGTGTTAACTTCGGCACCAAGGGTAT  608
             ||||||||||||||||||||||||||||||||||||||||||||||||||||||||||||
Sbjct  906   TCTTGCGACCGTACTCCCCAGGCGGAATGCTTAATGTGTTAACTTCGGCACCAAGGGTAT  847

Query  609   CGAAACCCCTAACACCTAGCATTCATCGTTTACGGCGTGGACTACCAGGGTATCTAATCC  668
             ||||||||||||||||||||||||||||||||||||||||||||||||||||||||||||
Sbjct  846   CGAAACCCCTAACACCTAGCATTCATCGTTTACGGCGTGGACTACCAGGGTATCTAATCC  787

Query  669   TGTTTGCTCCCCACGCTTTCGCGCCTCAGCGTCAGTTACAGCCCAGAAAGTCGCCTTCGC  728
             ||||||||||||||||||||||||||||||||||||||||||||||||||||||||||||
Sbjct  786   TGTTTGCTCCCCACGCTTTCGCGCCTCAGCGTCAGTTACAGCCCAGAAAGTCGCCTTCGC  727

Query  729   CACTGGTGTTCCTCCACATCTCTACGCATTTCACCGCTACACGTGGAATTCCACTTTCCT  788
             ||||||||||||||||||||||||||||||||||||||||||||||||||||||||||||
Sbjct  726   CACTGGTGTTCCTCCACATCTCTACGCATTTCACCGCTACACGTGGAATTCCACTTTCCT  667

Query  789   CTTCTGTACTCAAGCTCTCCAGTTTCCAGTGCGACCCAAGGTTGAGCCTTGGGCTGTGAC  848
             ||||||||||||||||||||||||||||||||||||||||||||||||||||||||||||
Sbjct  666   CTTCTGTACTCAAGCTCTCCAGTTTCCAGTGCGACCCAAGGTTGAGCCTTGGGCTGTGAC  607

Query  849   ACCGGACTTAAAAAGCCGCCTGCGCGCGCTTTACGCCCAATAATTCCGGACAACGCTTGC  908
             ||||||||||||||||||||||||||||||||||||||||||||||||||||||||||||
Sbjct  606   ACCGGACTTAAAAAGCCGCCTGCGCGCGCTTTACGCCCAATAATTCCGGACAACGCTTGC  547

Query  909   CCCCTACGTATTACCGCGGCTGCTGGCACGTAGTTAGCCGGGGCTTTCTTCTCAGGTACC  968
             ||||||||||||||||||||||||||||||||||||||||||||||||||||||||||||
Sbjct  546   CCCCTACGTATTACCGCGGCTGCTGGCACGTAGTTAGCCGGGGCTTTCTTCTCAGGTACC  487

Query  969   GTCACTCCNAGTTCAGTTACTAACCCGG  996
             |||||||| |||||||||||||||||||
Sbjct  486   GTCACTCCGAGTTCAGTTACTAACCCGG  459
```

Download

FASTA (complete sequence)

FASTA (aligned sequences)

GenBank (complete sequence)

Continue
Cancel

GenBankGraphics

Next
Previous
Descriptions

Uncultured bacterium clone 10-9BM 16S ribosomal RNA gene, partial sequence

Sequence ID: HQ143281.1Length: 1517Number of Matches: 1

Related Information

Range 1: 458 to 1446GenBankGraphics

Next Match
Previous Match
First Match

Alignment statistics for match #1

| Score | Expect | Identities | Gaps | Strand | Frame |
| --- | --- | --- | --- | --- | --- |
| 1805 bits(977) | 0.0() | 985/989(99%) | 1/989(0%) | Plus/Minus |  |

Features:

```
Query  9     CCACCGACTTCGGGTGTTGCAAACTCTCGTGGTGTGACGGGCGGTGTGTACAAGACCCGG  68
             ||||||||||||||||||||||||||||||||||||||||||||||||||||||||||||
Sbjct  1446  CCACCGACTTCGGGTGTTGCAAACTCTCGTGGTGTGACGGGCGGTGTGTACAAGACCCGG  1387

Query  69    GAACGTATTCACCGCGGCATGCTGATCCGCGATTACTAGCAATTCCGACTTCATGCAGGC  128
             ||||||||||||||||||||||||||||||||||||||||||||||||||||||||||||
Sbjct  1386  GAACGTATTCACCGCGGCATGCTGATCCGCGATTACTAGCAATTCCGACTTCATGCAGGC  1327

Query  129   GAGTTGCAGCCTGCAATCCGAACTGAGACCGGCTTTTAAGGATTGGCTCCATCTTGCGAT  188
             ||||||||||||||||||||||||||||||||||||||||||||||||||||||||||||
Sbjct  1326  GAGTTGCAGCCTGCAATCCGAACTGAGACCGGCTTTTAAGGATTGGCTCCATCTTGCGAT  1267

Query  189   ATTGCTTCCCGTTGTACCGGCCATTGTAGTACGTGTGTAGCCCAAGTCATAAGGGGCATG  248
             ||||||||||||||||||||||||||||||||||||||||||||||||||||||||||||
Sbjct  1266  ATTGCTTCCCGTTGTACCGGCCATTGTAGTACGTGTGTAGCCCAAGTCATAAGGGGCATG  1207

Query  249   ATGATTTGACGTCATCCCCACCTTCCTCCGGTTTGTCACCGGCAGTCTGCTTAGAGTGCC  308
             ||||||||||||||||||||||||||||||||| ||||||||||||||||||||||||||
Sbjct  1206  ATGATTTGACGTCATCCCCACCTTCCTCCGGTTCGTCACCGGCAGTCTGCTTAGAGTGCC  1147

Query  309   CACCATGATGTGCTGGCAACTAAGCATAAGGGTTGCGCTCGTTGCGGGACTTAACCCAAC  368
             ||||||||||||||||||||||||||||||||||||||||||||||||||||||||||||
Sbjct  1146  CACCATGATGTGCTGGCAACTAAGCATAAGGGTTGCGCTCGTTGCGGGACTTAACCCAAC  1087

Query  369   ATCTCACGACACGAGCTGACGACAACCATGCACCACCTGTCTCCTTTGTCCCGAAGGAAA  428
             | ||||||||||||||||||||||||||||||||||||||||||||||||||||||||||
Sbjct  1086  ACCTCACGACACGAGCTGACGACAACCATGCACCACCTGTCTCCTTTGTCCCGAAGGAAA  1027

Query  429   AGACTATCTCTAGTCCGGTCAAAGGGATGTCAAGACTTGGTAAGGTTCTTCGCGTTGCTT  488
             ||||||||||||||||||||||||||||||||||||||||||||||||||||||||||||
Sbjct  1026  AGACTATCTCTAGTCCGGTCAAAGGGATGTCAAGACTTGGTAAGGTTCTTCGCGTTGCTT  967

Query  489   CGAATTAAACCACATACTCCACTGCTTGTGCGGGTCCCCGTCAATTCCTTTGAGTTTCAG  548
             ||||||||||||||||||||||||||||||||||||||||||||||||||||||||||||
Sbjct  966   CGAATTAAACCACATACTCCACTGCTTGTGCGGGTCCCCGTCAATTCCTTTGAGTTTCAG  907

Query  549   TCTTGCGACCGTACTCCCCAGGCGGAATGCTTAATGTGTTAACTTCGGCACCAAGGGTAT  608
             ||||||||||||||||||||||||||||||||||||||||||||||||||||||||||||
Sbjct  906   TCTTGCGACCGTACTCCCCAGGCGGAATGCTTAATGTGTTAACTTCGGCACCAAGGGTAT  847

Query  609   CGAAACCCCTAACACCTAGCATTCATCGTTTACGGCG-TGGACTACCAGGGTATCTAATC  667
             ||||||||||||||||||||||||||||||||||||| ||||||||||||||||||||||
Sbjct  846   CGAAACCCCTAACACCTAGCATTCATCGTTTACGGCGGTGGACTACCAGGGTATCTAATC  787

Query  668   CTGTTTGCTCCCCACGCTTTCGCGCCTCAGCGTCAGTTACAGCCCAGAAAGTCGCCTTCG  727
             ||||||||||||||||||||||||||||||||||||||||||||||||||||||||||||
Sbjct  786   CTGTTTGCTCCCCACGCTTTCGCGCCTCAGCGTCAGTTACAGCCCAGAAAGTCGCCTTCG  727

Query  728   CCACTGGTGTTCCTCCACATCTCTACGCATTTCACCGCTACACGTGGAATTCCACTTTCC  787
             ||||||||||||||||||||||||||||||||||||||||||||||||||||||||||||
Sbjct  726   CCACTGGTGTTCCTCCACATCTCTACGCATTTCACCGCTACACGTGGAATTCCACTTTCC  667

Query  788   TCTTCTGTACTCAAGCTCTCCAGTTTCCAGTGCGACCCAAGGTTGAGCCTTGGGCTGTGA  847
             ||||||||||||||||||||||||||||||||||||||||||||||||||||||||||||
Sbjct  666   TCTTCTGTACTCAAGCTCTCCAGTTTCCAGTGCGACCCAAGGTTGAGCCTTGGGCTGTGA  607

Query  848   CACCGGACTTAAAAAGCCGCCTGCGCGCGCTTTACGCCCAATAATTCCGGACAACGCTTG  907
             ||||||||||||||||||||||||||||||||||||||||||||||||||||||||||||
Sbjct  606   CACCGGACTTAAAAAGCCGCCTGCGCGCGCTTTACGCCCAATAATTCCGGACAACGCTTG  547

Query  908   CCCCCTACGTATTACCGCGGCTGCTGGCACGTAGTTAGCCGGGGCTTTCTTCTCAGGTAC  967
             ||||||||||||||||||||||||||||||||||||||||||||||||||||||||||||
Sbjct  546   CCCCCTACGTATTACCGCGGCTGCTGGCACGTAGTTAGCCGGGGCTTTCTTCTCAGGTAC  487

Query  968   CGTCACTCCNAGTTCAGTTACTAACCCGG  996
             ||||||||| |||||||||||||||||||
Sbjct  486   CGTCACTCCGAGTTCAGTTACTAACCCGG  458
```

Download

FASTA (complete sequence)

FASTA (aligned sequences)

GenBank (complete sequence)

Continue
Cancel

GenBankGraphics

Next
Previous
Descriptions

Bacterium 4L18 16S ribosomal RNA gene, partial sequence

Sequence ID: KP965574.1Length: 1518Number of Matches: 1

Related Information

Range 1: 459 to 1448GenBankGraphics

Next Match
Previous Match
First Match

Alignment statistics for match #1

| Score | Expect | Identities | Gaps | Strand | Frame |
| --- | --- | --- | --- | --- | --- |
| 1801 bits(975) | 0.0() | 985/990(99%) | 2/990(0%) | Plus/Minus |  |

Features:

```
Query  9     CCACCGACTTCGGGTGTTGCAAACTCTCGTGGTGTGACGGGCGGTGTGTACAAGACCCGG  68
             ||||||||||||||||||||||||||||||||||||||||||||||||||||||||||||
Sbjct  1448  CCACCGACTTCGGGTGTTGCAAACTCTCGTGGTGTGACGGGCGGTGTGTACAAGACCCGG  1389

Query  69    GAACGTATTCACCGCGGCATGCTGATCCGCGATTACTAGCAATTCCGACTTCATGCAGGC  128
             ||||||||||||||||||||||||||||||||||||||||||||||||||||||||||||
Sbjct  1388  GAACGTATTCACCGCGGCATGCTGATCCGCGATTACTAGCAATTCCGACTTCATGCAGGC  1329

Query  129   GAGTTGCAGCCTGCAATCCGAACTGAGACCGGCTTTTAAGGATTGGCTCCATCTTGCGAT  188
             ||||||||||||||||||||||||||||||||||||||||||||||||||||||||||||
Sbjct  1328  GAGTTGCAGCCTGCAATCCGAACTGAGACCGGCTTTTAAGGATTGGCTCCATCTTGCGAT  1269

Query  189   ATTGCTTCCCGTTGTACCGGCCATTGTAGTACGTGTGTAGCCCAAGTCATAAGGGGCATG  248
             ||||||||||||||||||| ||||||||||||||||||||||||||||||||||||||||
Sbjct  1268  ATTGCTTCCCGTTGTACCGACCATTGTAGTACGTGTGTAGCCCAAGTCATAAGGGGCATG  1209

Query  249   ATGATTTGACGTCATCCCCACCTTCCTCCGGTTTGTCACCGGCAGTCTGCTTAGAGTGCC  308
             ||||||||||||||||||||||||||||||||||||||||||||||||||||||||||||
Sbjct  1208  ATGATTTGACGTCATCCCCACCTTCCTCCGGTTTGTCACCGGCAGTCTGCTTAGAGTGCC  1149

Query  309   CACCATGATGTGCTGGCAACTAAGCATAAGGGTTGCGCTCGTTGCGGGACTTAACCCAAC  368
             |||||| |||||||||||||||||||||||||||||||||||||||||||||||||||||
Sbjct  1148  CACCATAATGTGCTGGCAACTAAGCATAAGGGTTGCGCTCGTTGCGGGACTTAACCCAAC  1089

Query  369   ATCTCACGACACGAGCTGACGACAACCATGCACCACCTGTCTCCTTTGTCCCGAAGGAAA  428
             ||||||||||||||||||||||||||||||||||||||||||||||||||||||||||||
Sbjct  1088  ATCTCACGACACGAGCTGACGACAACCATGCACCACCTGTCTCCTTTGTCCCGAAGGAAA  1029

Query  429   AGACTATCTCTAGTCCGGTCAAAGGGATGTCAAGACTTGGTAAGGTTCTTCGCGTTGCTT  488
             ||||||||||||||||||||||||||||||||||||||||||||||||||||||||||||
Sbjct  1028  AGACTATCTCTAGTCCGGTCAAAGGGATGTCAAGACTTGGTAAGGTTCTTCGCGTTGCTT  969

Query  489   CGAATTAAACCACATACTCCACTGCTTGTGCGGGTCCCCGTCAATTCCTTTGAGTTTCAG  548
             ||||||||||||||||||||||||||||||||||||||||||||||||||||||||||||
Sbjct  968   CGAATTAAACCACATACTCCACTGCTTGTGCGGGTCCCCGTCAATTCCTTTGAGTTTCAG  909

Query  549   TCTTGCGACCGTACTCCCCAGGCGGAATGCTTAATGTGTTAACTTCGGCACC-AAGGGTA  607
             |||||||||||||||||||||||||||||||||||||||||||||||||||| |||||||
Sbjct  908   TCTTGCGACCGTACTCCCCAGGCGGAATGCTTAATGTGTTAACTTCGGCACCCAAGGGTA  849

Query  608   TCGAAACCCCTAACACCTAGCATTCATCGTTTACGGCGTGGACTACCAGGGTATCTAATC  667
             ||||||||||||||||||||||||||||||||||||||||||||||||||||||||||||
Sbjct  848   TCGAAACCCCTAACACCTAGCATTCATCGTTTACGGCGTGGACTACCAGGGTATCTAATC  789

Query  668   CTGTTTGCTCCCCACGCTTTCGCGCCTCAGCGTCAGTTACAGCCCAGAAAGTCGCCTTCG  727
             ||||||||||||||||||||||||||||||||||||||||||||||||||||||||||||
Sbjct  788   CTGTTTGCTCCCCACGCTTTCGCGCCTCAGCGTCAGTTACAGCCCAGAAAGTCGCCTTCG  729

Query  728   CCACTGGTGTTCCTCCACATCTCTACGCATTTCACCGCTACACGTGGAATTCCACTTTCC  787
             ||||||||||||||||||||||||||||||||||||||||||||||||||||||||||||
Sbjct  728   CCACTGGTGTTCCTCCACATCTCTACGCATTTCACCGCTACACGTGGAATTCCACTTTCC  669

Query  788   TCTTCTGTACTCAAGCTCTCCAGTTTCCAGTGCGACCCAAGGTTGAGCCTTGGGCTGTGA  847
             ||||||||||||||||||||||||||||||||||||||||||||||||||||||||||||
Sbjct  668   TCTTCTGTACTCAAGCTCTCCAGTTTCCAGTGCGACCCAAGGTTGAGCCTTGGGCTGTGA  609

Query  848   CACCGGACTTAAAAAGCCGCCTGCGCGCGCTTTACGCCCAATAATTCCGGACAACGCTTG  907
             ||||||||||||||||||||||||||||||||||||||||||||||||||||||||||||
Sbjct  608   CACCGGACTTAAAAAGCCGCCTGCGCGCGCTTTACGCCCAATAATTCCGGACAACGCTTG  549

Query  908   CCCCCTACGTATTACCGCGGCTGCTGGCACGTAGTTAGCCGGGG-CTTTCTTCTCAGGTA  966
             |||||||||||||||||||||||||||||||||||||||||||| |||||||||||||||
Sbjct  548   CCCCCTACGTATTACCGCGGCTGCTGGCACGTAGTTAGCCGGGGGCTTTCTTCTCAGGTA  489

Query  967   CCGTCACTCCNAGTTCAGTTACTAACCCGG  996
             |||||||||| |||||||||||||||||||
Sbjct  488   CCGTCACTCCGAGTTCAGTTACTAACCCGG  459
```

Download

FASTA (complete sequence)

FASTA (aligned sequences)

GenBank (complete sequence)

Continue
Cancel

GenBankGraphics

Next
Previous
Descriptions

Bacterium 4L13 16S ribosomal RNA gene, partial sequence

Sequence ID: KP965573.1Length: 1518Number of Matches: 1

Related Information

Range 1: 459 to 1448GenBankGraphics

Next Match
Previous Match
First Match

Alignment statistics for match #1

| Score | Expect | Identities | Gaps | Strand | Frame |
| --- | --- | --- | --- | --- | --- |
| 1801 bits(975) | 0.0() | 985/990(99%) | 2/990(0%) | Plus/Minus |  |

Features:

```
Query  9     CCACCGACTTCGGGTGTTGCAAACTCTCGTGGTGTGACGGGCGGTGTGTACAAGACCCGG  68
             ||||||||||||||||||||||||||||||||||||||||||||||||||||||||||||
Sbjct  1448  CCACCGACTTCGGGTGTTGCAAACTCTCGTGGTGTGACGGGCGGTGTGTACAAGACCCGG  1389

Query  69    GAACGTATTCACCGCGGCATGCTGATCCGCGATTACTAGCAATTCCGACTTCATGCAGGC  128
             ||||||||||||||||||||||||||||||||||||||||||||||||||||||| ||||
Sbjct  1388  GAACGTATTCACCGCGGCATGCTGATCCGCGATTACTAGCAATTCCGACTTCATGTAGGC  1329

Query  129   GAGTTGCAGCCTGCAATCCGAACTGAGACCGGCTTTTAAGGATTGGCTCCATCTTGCGAT  188
             ||||||||||||||||||||||||||||||||||||||||||||||||||||||||||||
Sbjct  1328  GAGTTGCAGCCTGCAATCCGAACTGAGACCGGCTTTTAAGGATTGGCTCCATCTTGCGAT  1269

Query  189   ATTGCTTCCCGTTGTACCGGCCATTGTAGTACGTGTGTAGCCCAAGTCATAAGGGGCATG  248
             ||||||||||||||||||||||||||||||||||||||||||||||||||||||||||||
Sbjct  1268  ATTGCTTCCCGTTGTACCGGCCATTGTAGTACGTGTGTAGCCCAAGTCATAAGGGGCATG  1209

Query  249   ATGATTTGACGTCATCCCCACCTTCCTCCGGTTTGTCACCGGCAGTCTGCTTAGAGTGCC  308
             ||||||||||||||||||||||||||||||||||||||||||||||||||||||||||||
Sbjct  1208  ATGATTTGACGTCATCCCCACCTTCCTCCGGTTTGTCACCGGCAGTCTGCTTAGAGTGCC  1149

Query  309   CACCATGATGTGCTGGCAACTAAGCATAAGGGTTGCGCTCGTTGCGGGACTTAACCCAAC  368
             ||||||||||||||||||||||||||||||||||||||||||||||||||||||||||||
Sbjct  1148  CACCATGATGTGCTGGCAACTAAGCATAAGGGTTGCGCTCGTTGCGGGACTTAACCCAAC  1089

Query  369   ATCTCACGACACGAGCTGACGACAACCATGCACCACCTGTCTCCTTTGTCCCGAAGGAAA  428
             |||||||||||||||||||||||||||||||||| |||||||||||||||||||||||||
Sbjct  1088  ATCTCACGACACGAGCTGACGACAACCATGCACCGCCTGTCTCCTTTGTCCCGAAGGAAA  1029

Query  429   AGACTATCTCTAGTCCGGTCAAAGGGATGTCAAGACTTGGTAAGGTTCTTCGCGTTGCTT  488
             ||||||||||||||||||||||||||||||||||||||||||||||||||||||||||||
Sbjct  1028  AGACTATCTCTAGTCCGGTCAAAGGGATGTCAAGACTTGGTAAGGTTCTTCGCGTTGCTT  969

Query  489   CGAATTAAACCACATACTCCACTGCTTGTGCGGGTCCCCGTCAATTCCTTTGAGTTTCAG  548
             ||||||||||||||||||||||||||||||||||||||||||||||||||||||||||||
Sbjct  968   CGAATTAAACCACATACTCCACTGCTTGTGCGGGTCCCCGTCAATTCCTTTGAGTTTCAG  909

Query  549   TCTTGCGACCGTACTCCCCAGGCGGAATGCTTAATGTGTTAACTTCGGCACCAAGGGTAT  608
             ||||||||||||||||||||||||||||||||||||||||||||||||||||||||||||
Sbjct  908   TCTTGCGACCGTACTCCCCAGGCGGAATGCTTAATGTGTTAACTTCGGCACCAAGGGTAT  849

Query  609   CGAAACCCCTAACACCTAGCATTCATCGTTTACGGCGTGGACTACCAGGGTATCTAATCC  668
             ||||||||||||||||||||||||||||||||||||||||||||||||||||||||||||
Sbjct  848   CGAAACCCCTAACACCTAGCATTCATCGTTTACGGCGTGGACTACCAGGGTATCTAATCC  789

Query  669   TGTTTGCTCCCCACGCTTTCGCGCCTCAGCGTCAGTTACAGCCCAGAAAGTCGCCTTCGC  728
             ||||||||||||||||||||||||||||||||||||||||||||||||||||||||||||
Sbjct  788   TGTTTGCTCCCCACGCTTTCGCGCCTCAGCGTCAGTTACAGCCCAGAAAGTCGCCTTCGC  729

Query  729   CACTGGTGTTCCTCCACATCTCTACGCATTTCACCGCTACACGTGGAATTCCACTTTCCT  788
             ||||||||||||||||||||||||||||||||||||||||||||||||||||||||||||
Sbjct  728   CACTGGTGTTCCTCCACATCTCTACGCATTTCACCGCTACACGTGGAATTCCACTTTCCT  669

Query  789   CTTCTGTACTCAAGCTCTCCAGTTTCCAGTGCGACCCAAGGTTGAGCCTTGGGCTGTGAC  848
             ||||||||||||||||||||||||||||||||||||||||||||||||||||||||||||
Sbjct  668   CTTCTGTACTCAAGCTCTCCAGTTTCCAGTGCGACCCAAGGTTGAGCCTTGGGCTGTGAC  609

Query  849   ACCGGACTTAAAAAGCCGCCTGCGCGCGCTTTACGCCCAATAATTCCGGACAACGCTTGC  908
             ||||||||||||||||||||||||||||||||||||||||||||||||||||||||||||
Sbjct  608   ACCGGACTTAAAAAGCCGCCTGCGCGCGCTTTACGCCCAATAATTCCGGACAACGCTTGC  549

Query  909   CCCCTACGTATTACCGCGG-CTGCT-GGCACGTAGTTAGCCGGGGCTTTCTTCTCAGGTA  966
             ||||||||||||||||||| ||||| ||||||||||||||||||||||||||||||||||
Sbjct  548   CCCCTACGTATTACCGCGGGCTGCTGGGCACGTAGTTAGCCGGGGCTTTCTTCTCAGGTA  489

Query  967   CCGTCACTCCNAGTTCAGTTACTAACCCGG  996
             |||||||||| |||||||||||||||||||
Sbjct  488   CCGTCACTCCGAGTTCAGTTACTAACCCGG  459
```

Download

FASTA (complete sequence)

FASTA (aligned sequences)

GenBank (complete sequence)

Continue
Cancel

GenBankGraphics

Next
Previous
Descriptions

Paenibacillus sp. strain BSW35 16S ribosomal RNA gene, partial sequence

Sequence ID: KY446062.1Length: 1433Number of Matches: 1

Related Information

Range 1: 416 to 1403GenBankGraphics

Next Match
Previous Match
First Match

Alignment statistics for match #1

| Score | Expect | Identities | Gaps | Strand | Frame |
| --- | --- | --- | --- | --- | --- |
| 1799 bits(974) | 0.0() | 983/988(99%) | 0/988(0%) | Plus/Minus |  |

Features:

```
Query  9     CCACCGACTTCGGGTGTTGCAAACTCTCGTGGTGTGACGGGCGGTGTGTACAAGACCCGG  68
             ||||||||||||||||||||||||||||||||||||||||||||||||||||||||||||
Sbjct  1403  CCACCGACTTCGGGTGTTGCAAACTCTCGTGGTGTGACGGGCGGTGTGTACAAGACCCGG  1344

Query  69    GAACGTATTCACCGCGGCATGCTGATCCGCGATTACTAGCAATTCCGACTTCATGCAGGC  128
             ||||||||||||||||||||||||||||||||||||||||||||||||||||||||||||
Sbjct  1343  GAACGTATTCACCGCGGCATGCTGATCCGCGATTACTAGCAATTCCGACTTCATGCAGGC  1284

Query  129   GAGTTGCAGCCTGCAATCCGAACTGAGACCGGCTTTTAAGGATTGGCTCCATCTTGCGAT  188
             ||||||||||||||||||||||||||||||||||||||||||||||||||||||||||||
Sbjct  1283  GAGTTGCAGCCTGCAATCCGAACTGAGACCGGCTTTTAAGGATTGGCTCCATCTTGCGAT  1224

Query  189   ATTGCTTCCCGTTGTACCGGCCATTGTAGTACGTGTGTAGCCCAAGTCATAAGGGGCATG  248
             ||||||||||||||||||||||||||||||||||||||||||||||||||||||||||||
Sbjct  1223  ATTGCTTCCCGTTGTACCGGCCATTGTAGTACGTGTGTAGCCCAAGTCATAAGGGGCATG  1164

Query  249   ATGATTTGACGTCATCCCCACCTTCCTCCGGTTTGTCACCGGCAGTCTGCTTAGAGTGCC  308
             ||||||||||||||||||||||||||||||||||||||||||||||||||||||||||||
Sbjct  1163  ATGATTTGACGTCATCCCCACCTTCCTCCGGTTTGTCACCGGCAGTCTGCTTAGAGTGCC  1104

Query  309   CACCATGATGTGCTGGCAACTAAGCATAAGGGTTGCGCTCGTTGCGGGACTTAACCCAAC  368
             ||||||||||||||||||||||| ||||||||||||||||||||||||||||||||||||
Sbjct  1103  CACCATGATGTGCTGGCAACTAAACATAAGGGTTGCGCTCGTTGCGGGACTTAACCCAAC  1044

Query  369   ATCTCACGACACGAGCTGACGACAACCATGCACCACCTGTCTCCTTTGTCCCGAAGGAAA  428
             ||||||||||||||||||||||||||||||||||||||||||||||||||||||||||||
Sbjct  1043  ATCTCACGACACGAGCTGACGACAACCATGCACCACCTGTCTCCTTTGTCCCGAAGGAAA  984

Query  429   AGACTATCTCTAGTCCGGTCAAAGGGATGTCAAGACTTGGTAAGGTTCTTCGCGTTGCTT  488
             ||||||||||||||||||||||||||||||||||||||||||||||||||||||||||||
Sbjct  983   AGACTATCTCTAGTCCGGTCAAAGGGATGTCAAGACTTGGTAAGGTTCTTCGCGTTGCTT  924

Query  489   CGAATTAAACCACATACTCCACTGCTTGTGCGGGTCCCCGTCAATTCCTTTGAGTTTCAG  548
             ||||||||||||||||||||||||||||||||||||||||||||||||||||||||||||
Sbjct  923   CGAATTAAACCACATACTCCACTGCTTGTGCGGGTCCCCGTCAATTCCTTTGAGTTTCAG  864

Query  549   TCTTGCGACCGTACTCCCCAGGCGGAATGCTTAATGTGTTAACTTCGGCACCAAGGGTAT  608
             ||||||||||||||||||||||||||||||||||||||||||||||||||||||||||||
Sbjct  863   TCTTGCGACCGTACTCCCCAGGCGGAATGCTTAATGTGTTAACTTCGGCACCAAGGGTAT  804

Query  609   CGAAACCCCTAACACCTAGCATTCATCGTTTACGGCGTGGACTACCAGGGTATCTAATCC  668
             ||||||||||||||||||||||||||||||||||||||||||||||||||||||||||||
Sbjct  803   CGAAACCCCTAACACCTAGCATTCATCGTTTACGGCGTGGACTACCAGGGTATCTAATCC  744

Query  669   TGTTTGCTCCCCACGCTTTCGCGCCTCAGCGTCAGTTACAGCCCAGAAAGTCGCCTTCGC  728
             ||||||||||||||||||||||||||||||||||||||||||||||||||||||||||||
Sbjct  743   TGTTTGCTCCCCACGCTTTCGCGCCTCAGCGTCAGTTACAGCCCAGAAAGTCGCCTTCGC  684

Query  729   CACTGGTGTTCCTCCACATCTCTACGCATTTCACCGCTACACGTGGAATTCCACTTTCCT  788
             ||||||||||||||||||||||||||||||||||||||||||||||||||||||||||||
Sbjct  683   CACTGGTGTTCCTCCACATCTCTACGCATTTCACCGCTACACGTGGAATTCCACTTTCCT  624

Query  789   CTTCTGTACTCAAGCTCTCCAGTTTCCAGTGCGACCCAAGGTTGAGCCTTGGGCTGTGAC  848
             ||||||||||||||||||||||||||||||||||||||||||||||||||||||||||||
Sbjct  623   CTTCTGTACTCAAGCTCTCCAGTTTCCAGTGCGACCCAAGGTTGAGCCTTGGGCTGTGAC  564

Query  849   ACCGGACTTAAAAAGCCGCCTGCGCGCGCTTTACGCCCAATAATTCCGGACAACGCTTGC  908
             ||||||||||||||||||||||||||||||||||||||||||||||||||||||||||||
Sbjct  563   ACCGGACTTAAAAAGCCGCCTGCGCGCGCTTTACGCCCAATAATTCCGGACAACGCTTGC  504

Query  909   CCCCTACGTATTACCGCGGCTGCTGGCACGTAGTTAGCCGGGGCTTTCTTCTCAGGTACC  968
             ||||||||||||||||||||||||||||||||||||||||||||||||||||||||||||
Sbjct  503   CCCCTACGTATTACCGCGGCTGCTGGCACGTAGTTAGCCGGGGCTTTCTTCTCAGGTACC  444

Query  969   GTCACTCCNAGTTCAGTTACTAACCCGG  996
             |||||||| ||| |||||||| || |||
Sbjct  443   GTCACTCCGAGTGCAGTTACTCACTCGG  416
```


BLAST is a registered trademark of the National Library of Medicine

Support center
Mailing list


YouTube

- National Library Of Medicine
- National Institutes Of Health
- U.S. Department of Health & Human Services
- USA.gov

### NCBI


National Center for Biotechnology Information,
 U.S. National Library of Medicine

8600 Rockville Pike,
Bethesda
 MD,
20894
USA

Policies and Guidelines
|
Contact


PreferencesTurn off

External link. Please review our privacy policy.
