# Supplementary material for: Isothermal microcalorimetry for thermal viable count of microorganisms in pure cultures and stabilized formulations
Source: BMC Microbiol. 2019 Mar 21;19:65. doi: 10.1186/s12866-019-1432-8 (PMC6429831; doi:10.1186/s12866-019-1432-8)
Supplement: Supplementary file 3 — 16S rRNA gene sequences’ BLASTN hits in zipped HTML format. (ZIP 15810 kb) [file 12866_2019_1432_MOESM3_ESM.zip › Best blastn hits/NCBI Blast_H yellow R -- 18..1017 of sequence (1000.html]

NCBI Blast:H yellow R -- 18..1017 of sequence (1000


- NCBI Home
- Sign in to NCBI
- Skip to Main Content
- Skip to Navigation
- About NCBI Accesskeys

U.S. National Library of Medicine

NCBI
National Center for Biotechnology Information

- My NCBI
- Sign in to NCBI
- Register
- Sign Out

BLAST ^®^ » blastn suite » RID-A4XUVYJV014


- Home
- Recent Results
- Saved Strategies
- Help

BLAST Results


Edit and Resubmit
Save Search Strategies
[Sign in above to save your search strategy]

Formatting options 


Download


How to read this page
Blast report description
Questions/comments


|  |  |
| --- | --- |
| Formatting options | |
| Show | Alignment as  HTML Plain text   Old View Reset form to defaults [?]  These options control formatting of alignments in results pages. The default is HTML, but other formats (including plain text) are available. PSSM and PssmWithParameters are representations of Position Specific Scoring Matrices and are only available for PSI-BLAST. The Advanced view option allows the database descriptions to be sorted by various indices in a table. |
| Alignment View | Pairwise Pairwise with dots for identities Query-anchored with dots for identities Query-anchored with letters for identities Flat query-anchored with dots for identities Flat query-anchored with letters for identities [?]  Choose how to view alignments. The default "pairwise" view shows how each subject sequence aligns individually to the query sequence. The "query-anchored" view shows how all subject sequences align to the query sequence. For each view type, you can choose to show "identities" (matching residues) as letters or dots. more... |
| Display | Graphical Overview   Linkout   Sequence Retrieval  NCBI-gi   CDS feature [?]  - Graphical Overview: Graphical Overview: Show graph of similar sequence regions aligned to query.   more... - NCBI-gi: Show NCBI gi identifiers. - CDS feature: Show annotated coding region and translation.   more... |
| Masking | Character:   X for protein, n for nucleotide Lower Case  Color:  Black Grey Red [?]  - Masking Character: Display masked (filtered) sequence regions as lower-case or as specific letters (N for nucleotide, P for protein). - Masking Color: Display masked sequence regions in the given color. |
| Limit results | Descriptions:  10 50 100 Graphical overview:  0 10 50 100  Alignments:  0 10 50 100 Line length:  60 90 120 150 [?]  - Descriptions: Show short descriptions for up to the given number of sequences. - Alignments: Show alignments for up to the given number of sequences, in order of statistical significance. - Line lenghth: Number of letters to show on one line in an alignment. |
|  | Organism Type common name, binomial, taxid, or group name. Only 20 top taxa will be shown.     Exclude   [?]  Show only sequences from the given organism. |
|  | Entrez query:  [?]  Show only those sequences that match the given Entrez query. more... |
|  | Expect Min:  Expect Max:  [?]  Show only sequences with expect values in the given range. more... |
|  | Percent Identity Min:  Percent Identity Max:  [?]  Show only sequences with percent identity values in the given range. |
| Format for | PSI-BLAST with inclusion threshold:  [?]  - Format for PSI-BLAST: The Position-Specific Iterated BLAST (PSI-BLAST) program performs iterative searches with a protein query,   in which sequences found in one round of search are used to build a custom score model for the next round.   more... - Inclusion Threshold: This sets the statistical significance threshold for including a sequence in the model used   by PSI-BLAST to create the PSSM on the next iteration. |

|  |  |  |  |  |  |
| --- | --- | --- | --- | --- | --- |
| Download | | | | | |
| Alignment  Text XML ASN.1 JSON Seq-align Hit Table(text) Hit Table(csv) Multiple-file XML2 Single-file XML2 Multiple-file JSON Single-file JSON SAM | Search Strategies  ASN.1 | PSSM to restart search  PSSM | [?] |  |  |

The Download link provides BLAST output that may be used as input to another program.
This includes parseable formats such as the tabular report or XML as well as the Search Strategy files read by the BLAST+ applications.
More details on the parseable (XML, tabular, and ASN.1) reports can be found at
https://www.ncbi.nlm.nih.gov/books/NBK153387/  
  

The following formats are offered under the Alignment section:  
1). "Text". Non-HTML standard BLAST report.  
2). "XML". XML report based upon the DTD at https://www.ncbi.nlm.nih.gov/data\_specs/dtd/NCBI\_BlastOutput.dtd  
3). "ASN.1". Alignment written out in Abstract Syntax Notation 1.  
4). "JSON Seq-align". Alignment written out in JSON.  
4). "Hit Table(text)". The tabular report as text.  
5). "Hit Table(csv)". The tabular report ready for import into spread-sheet programs like Excel.  
6). "XML2". New XML format described at ftp://ftp.ncbi.nlm.nih.gov/blast/documents/NEWXML/xml2.pdf.  
7). "JSON". New JSON format described at ftp://ftp.ncbi.nlm.nih.gov/blast/documents/NEWXML/xml2.pdf.  
8). "SAM". Sequence Alignment Map format.

XML2 and JSON can be downloaded either as one file per query (multiple-file) or one file for all queries (single-file). These formats are listed as Multiple-file XML2 (and JSON) or Single-file XML (and JSON).

The following report is offered under the Search Strategy section:  
1). "ASN.1" Search Strategy. A record of the parameters, query, and database used in the search. This file can be used to start a stand-alone BLAST search, see
https://www.ncbi.nlm.nih.gov/books/NBK1763/#CmdLineAppsManual.I455\_BLAST\_search\_stra


# Job title: H yellow R -- 18..1017 of sequence (1000

Results for:

lcl|Query\_210253 H yellow R -- 18..1017 of sequence(1000bp)
[?]

Your BLAST job specified more than one input sequence.
This box lets you choose which input sequence to show BLAST results for.

RID
:   A4XUVYJV014 (Expires on 03-10 19:00 pm)

Query ID
:   lcl|Query\_210253
:   lcl|Query\_210253

Description
:   H yellow R -- 18..1017 of sequence

Molecule type
:   nucleic acid

Query Length
:   1000

Database Name
:   nr

Description
:   Nucleotide collection (nt) See details

Program
:   BLASTN 2.8.0+ Citation

  

Reference 

Zheng Zhang, Scott Schwartz, Lukas Wagner, and Webb Miller (2000), "A greedy algorithm for aligning DNA sequences", J Comput Biol 2000; 7(1-2):203-14.

Reference - database indexing

Aleksandr Morgulis, George Coulouris, Yan Raytselis, Thomas L. Madden, Richa Agarwala, Alejandro A. Schäffer (2008), "Database Indexing for Production MegaBLAST Searches", Bioinformatics 24:1757-1764.

Other reports:
Search Summary

[Taxonomy reports]
[Distance tree of results]
[MSA viewer]

Search Parameters

| Search parameter name | Search parameter value |
| --- | --- |
| Program | blastn |
| Word size | 28 |
| Expect value | 10 |
| Hitlist size | 100 |
| Match/Mismatch scores | 1,-2 |
| Gapcosts | 0,2.5 |
| Low Complexity Filter | Yes |
| Filter string | L;m; |
| Genetic Code | 1 |

Database

| Database parameter name | Database parameter value |
| --- | --- |
| Posted date | Mar 7, 2018 1:58 PM |
| Number of letters | 174,044,644,244 |
| Number of sequences | 46,882,714 |
| Entrez query | Includes:  Excludes:  None |

Karlin-Altschul statistics

| Params | Ungapped | Gapped |
| --- | --- | --- |
| Lambda | 1.33271 | 1.28 |
| K | 0.620991 | 0.46 |
| H | 1.12409 | 0.85 |

Results Statistics

| Results Statistics parameter name | Results Statistics parameter value |
| --- | --- |
| Length adjustment | 35 |
| Effective length of query | 965 |
| Effective length of database | 172403749254 |
| Effective search space | 166369618030110 |
| Effective search space used | 166369618030110 |


## Graphic Summary

### Distribution of the top 118 Blast Hits on 100 subject sequences [?]

The graphic is an overview of the database sequences aligned to the query sequence. These are represented horizontal bars colored coded by score and showing the extent
of the alignment on the query sequence. Separate aligned regions on the same database sequence are connected by a thin grey line.
Mousing over an alignment shows the database sequence title. Clicking an alignment displays a box with more details about the alignment and
link to the sequence alignment itself in the Alignments section of the report.

Mouse over to see the title, click to show alignments

Color key for alignment scores

<40

40-50

50-80

80-200

>=200

Query

1

200

400

600

800

1000

Pantoea agglomerans strain BL26 16S ribosomal RNA gene,..

Score:1801 Evalue:0

Accession:KU258277.1

Alignment

Pantoea agglomerans strain QSC 16S ribosomal RNA gene, ..

Score:1801 Evalue:0

Accession:KT075184.1

Alignment

Pantoea sp. E100 16S ribosomal RNA gene, partial sequen..

Score:1801 Evalue:0

Accession:KF153221.1

Alignment

Pantoea vagans strain FDAARGOS\_160 chromosome, complete..

Score:1799 Evalue:0

Accession:CP014129.2

Alignment

Pantoea agglomerans strain AL14 16S ribosomal RNA gene,..

Score:1799 Evalue:0

Accession:MG819218.1

Alignment

Pantoea agglomerans strain Rk92 16S ribosomal RNA gene,..

Score:1799 Evalue:0

Accession:MG547769.1

Alignment

Pantoea agglomerans strain P3FNa\_1 16S ribosomal RNA ge..

Score:1799 Evalue:0

Accession:KX891531.1

Alignment

Pantoea agglomerans strain P2FNb\_1 16S ribosomal RNA ge..

Score:1799 Evalue:0

Accession:KX891517.1

Alignment

Pantoea agglomerans strain P2FNa\_2 16S ribosomal RNA ge..

Score:1799 Evalue:0

Accession:KX891516.1

Alignment

Pantoea agglomerans strain P2FNa\_1 16S ribosomal RNA ge..

Score:1799 Evalue:0

Accession:KX891515.1

Alignment

Pantoea agglomerans strain P1FPa\_2 16S ribosomal RNA ge..

Score:1799 Evalue:0

Accession:KX891502.1

Alignment

Pantoea agglomerans strain P1FNd\_1 16S ribosomal RNA ge..

Score:1799 Evalue:0

Accession:KX891501.1

Alignment

Pantoea agglomerans strain P1FNc\_2 16S ribosomal RNA ge..

Score:1799 Evalue:0

Accession:KX891500.1

Alignment

Pantoea agglomerans strain P1FNc\_1 16S ribosomal RNA ge..

Score:1799 Evalue:0

Accession:KX891499.1

Alignment

Pantoea agglomerans strain P1FNb\_1 16S ribosomal RNA ge..

Score:1799 Evalue:0

Accession:KX891498.1

Alignment

Pantoea agglomerans strain P1FNa\_1 16S ribosomal RNA ge..

Score:1799 Evalue:0

Accession:KX891497.1

Alignment

Pantoea agglomerans strain 62 16S ribosomal RNA gene, p..

Score:1799 Evalue:0

Accession:MF767519.1

Alignment

Pantoea agglomerans strain 22MB 16S ribosomal RNA gene,..

Score:1799 Evalue:0

Accession:MF372619.1

Alignment

Pantoea agglomerans strain C410P1, complete genome

Score:1799 Evalue:0

Accession:CP016889.1

Alignment

Uncultured Pantoea sp. clone GJ\_(2)\_73 16S ribosomal RN..

Score:1799 Evalue:0

Accession:KX603481.1

Alignment

Uncultured Pantoea sp. clone GJ\_(2)\_7 16S ribosomal RNA..

Score:1799 Evalue:0

Accession:KX603479.1

Alignment

Pantoea sp. CanR-85 16S ribosomal RNA gene, partial seq..

Score:1799 Evalue:0

Accession:KT580675.1

Alignment

Pantoea agglomerans strain SSE 16S ribosomal RNA gene, ..

Score:1799 Evalue:0

Accession:KT075193.1

Alignment

Pantoea agglomerans strain NSF 16S ribosomal RNA gene, ..

Score:1799 Evalue:0

Accession:KT075171.1

Alignment

Pantoea agglomerans strain NSD 16S ribosomal RNA gene, ..

Score:1799 Evalue:0

Accession:KT075169.1

Alignment

Pantoea agglomerans strain GSC 16S ribosomal RNA gene, ..

Score:1799 Evalue:0

Accession:KT075163.1

Alignment

Pantoea agglomerans strain GSB 16S ribosomal RNA gene, ..

Score:1799 Evalue:0

Accession:KT075162.1

Alignment

Pantoea agglomerans strain B81 16S ribosomal RNA gene, ..

Score:1799 Evalue:0

Accession:KJ781904.1

Alignment

Curtobacterium plantarum strain S4 16S ribosomal RNA ge..

Score:1799 Evalue:0

Accession:KP099434.1

Alignment

Curtobacterium plantarum strain S2 16S ribosomal RNA ge..

Score:1799 Evalue:0

Accession:KP099433.1

Alignment

Enterobacteriaceae bacterium X1/SB37 gene for 16S ribos..

Score:1799 Evalue:0

Accession:LC007922.1

Alignment

Enterobacteriaceae bacterium X1/SB52 gene for 16S ribos..

Score:1799 Evalue:0

Accession:LC007878.1

Alignment

Enterobacteriaceae bacterium X1/SB44 gene for 16S ribos..

Score:1799 Evalue:0

Accession:LC007874.1

Alignment

Pantoea agglomerans strain ex2 culture-collection CGMCC..

Score:1799 Evalue:0

Accession:KF317872.1

Alignment

Pantoea agglomerans strain ABAC22 16S ribosomal RNA gen..

Score:1799 Evalue:0

Accession:KF003386.1

Alignment

Enterobacteriaceae bacterium SAP952.2 16S ribosomal RNA..

Score:1799 Evalue:0

Accession:JX067721.1

Alignment

Enterobacteriaceae bacterium SAP770.3 16S ribosomal RNA..

Score:1799 Evalue:0

Accession:JX067684.1

Alignment

Enterobacteriaceae bacterium SAP108.1 16S ribosomal RNA..

Score:1799 Evalue:0

Accession:JX067654.1

Alignment

Pantoea agglomerans strain Z01 16S ribosomal RNA gene, ..

Score:1799 Evalue:0

Accession:JX257179.1

Alignment

Uncultured Pantoea sp. clone WRFC18 16S ribosomal RNA g..

Score:1799 Evalue:0

Accession:JX279936.1

Alignment

Pantoea agglomerans strain KB64 16S ribosomal RNA gene,..

Score:1799 Evalue:0

Accession:JF327484.1

Alignment

Pantoea agglomerans strain KB38 16S ribosomal RNA gene,..

Score:1799 Evalue:0

Accession:JF327464.1

Alignment

Pantoea agglomerans strain CJ-G-R2A5 16S ribosomal RNA ..

Score:1799 Evalue:0

Accession:HM584289.1

Alignment

[Curtobacterium] plantarum strain CL63 16S ribosomal RN..

Score:1799 Evalue:0

Accession:NR\_104943.1

Alignment

Pantoea agglomerans strain 1.2244 16S ribosomal RNA gen..

Score:1799 Evalue:0

Accession:HM130689.1

Alignment

Pantoea agglomerans strain SR-1 16S ribosomal RNA gene,..

Score:1799 Evalue:0

Accession:FJ593000.1

Alignment

Pantoea agglomerans strain SN-2 16S ribosomal RNA gene,..

Score:1799 Evalue:0

Accession:FJ592996.1

Alignment

Pantoea agglomerans strain NZ 16S ribosomal RNA gene, p..

Score:1799 Evalue:0

Accession:FJ756348.1

Alignment

Pantoea agglomerans strain VA21971 16S ribosomal RNA ge..

Score:1799 Evalue:0

Accession:FJ611833.1

Alignment

Pantoea agglomerans strain P5WAM 16S ribosomal RNA gene..

Score:1799 Evalue:0

Accession:FJ611822.1

Alignment

Pantoea agglomerans pv. gypsophilae strain ATCC 43348 1..

Score:1799 Evalue:0

Accession:FJ611821.1

Alignment

Pantoea agglomerans strain BBPE0103202 16S ribosomal RN..

Score:1799 Evalue:0

Accession:FJ357809.1

Alignment

Pantoea sp. II\_Gauze\_W\_10\_17 16S ribosomal RNA gene, pa..

Score:1799 Evalue:0

Accession:FJ267564.1

Alignment

Pantoea sp. Wd5760 16S ribosomal RNA gene, partial sequ..

Score:1799 Evalue:0

Accession:EU828365.1

Alignment

Enterobacter agglomerans strain A57 16S ribosomal RNA, ..

Score:1799 Evalue:0

Accession:AF130927.2

Alignment

Marine bacterium MSC106 16S ribosomal RNA gene, partial..

Score:1799 Evalue:0

Accession:EU753130.1

Alignment

## Descriptions

, Reading indexes 1-5, displaying indexes 1-5


Load next setPrevious Match

Sequences producing significant alignments:

Show all columns  of the table presenting sequences producing significant alignments 

Select:AllNone
Selected:0

Alignments
Download

FASTA (complete sequence)

FASTA (aligned sequences)

GenBank (complete sequence)

Hit Table (text)

Hit Table (CSV)

Text

XML

ASN.1

Continue
Cancel

GenBank 
Graphics
Distance tree of results
Multiple alignment
Show/hide columns of the table presenting sequences producing significant alignments 

Available columns

Description  
Max Score  
Total Score  
Coverage  
E-value  
IdentN  
Accession  
Restore Defaults
Ok
Cancel

Sequences producing significant alignments:

| Select for downloading or viewing reports | Description | Max score | Total score | Query cover | E value | Ident | Accession |
| --- | --- | --- | --- | --- | --- | --- | --- |
| 1Select seq KU258277.1 | Pantoea agglomerans strain BL26 16S ribosomal RNA gene, partial sequence | 1801 | 1801 | 98% | 0.0 | 99% | KU258277.1 |
| 2Select seq KT075184.1 | Pantoea agglomerans strain QSC 16S ribosomal RNA gene, partial sequence | 1801 | 1801 | 98% | 0.0 | 99% | KT075184.1 |
| 3Select seq KF153221.1 | Pantoea sp. E100 16S ribosomal RNA gene, partial sequence | 1801 | 1801 | 98% | 0.0 | 99% | KF153221.1 |
| 4Select seq CP014129.2 | Pantoea vagans strain FDAARGOS\_160 chromosome, complete genome | 1799 | 12570 | 98% | 0.0 | 99% | CP014129.2 |
| 5Select seq MG819218.1 | Pantoea agglomerans strain AL14 16S ribosomal RNA gene, partial sequence | 1799 | 1799 | 98% | 0.0 | 99% | MG819218.1 |
| 6Select seq MG547769.1 | Pantoea agglomerans strain Rk92 16S ribosomal RNA gene, partial sequence | 1799 | 1799 | 98% | 0.0 | 99% | MG547769.1 |
| 7Select seq KX891531.1 | Pantoea agglomerans strain P3FNa\_1 16S ribosomal RNA gene, partial sequence | 1799 | 1799 | 98% | 0.0 | 99% | KX891531.1 |
| 8Select seq KX891517.1 | Pantoea agglomerans strain P2FNb\_1 16S ribosomal RNA gene, partial sequence | 1799 | 1799 | 98% | 0.0 | 99% | KX891517.1 |
| 9Select seq KX891516.1 | Pantoea agglomerans strain P2FNa\_2 16S ribosomal RNA gene, partial sequence | 1799 | 1799 | 98% | 0.0 | 99% | KX891516.1 |
| 10Select seq KX891515.1 | Pantoea agglomerans strain P2FNa\_1 16S ribosomal RNA gene, partial sequence | 1799 | 1799 | 98% | 0.0 | 99% | KX891515.1 |
| 11Select seq KX891502.1 | Pantoea agglomerans strain P1FPa\_2 16S ribosomal RNA gene, partial sequence | 1799 | 1799 | 98% | 0.0 | 99% | KX891502.1 |
| 12Select seq KX891501.1 | Pantoea agglomerans strain P1FNd\_1 16S ribosomal RNA gene, partial sequence | 1799 | 1799 | 98% | 0.0 | 99% | KX891501.1 |
| 13Select seq KX891500.1 | Pantoea agglomerans strain P1FNc\_2 16S ribosomal RNA gene, partial sequence | 1799 | 1799 | 98% | 0.0 | 99% | KX891500.1 |
| 14Select seq KX891499.1 | Pantoea agglomerans strain P1FNc\_1 16S ribosomal RNA gene, partial sequence | 1799 | 1799 | 98% | 0.0 | 99% | KX891499.1 |
| 15Select seq KX891498.1 | Pantoea agglomerans strain P1FNb\_1 16S ribosomal RNA gene, partial sequence | 1799 | 1799 | 98% | 0.0 | 99% | KX891498.1 |
| 16Select seq KX891497.1 | Pantoea agglomerans strain P1FNa\_1 16S ribosomal RNA gene, partial sequence | 1799 | 1799 | 98% | 0.0 | 99% | KX891497.1 |
| 17Select seq MF767519.1 | Pantoea agglomerans strain 62 16S ribosomal RNA gene, partial sequence | 1799 | 1799 | 98% | 0.0 | 99% | MF767519.1 |
| 18Select seq MF372619.1 | Pantoea agglomerans strain 22MB 16S ribosomal RNA gene, partial sequence | 1799 | 1799 | 98% | 0.0 | 99% | MF372619.1 |
| 19Select seq CP016889.1 | Pantoea agglomerans strain C410P1, complete genome | 1799 | 12487 | 98% | 0.0 | 99% | CP016889.1 |
| 20Select seq KX603481.1 | Uncultured Pantoea sp. clone GJ\_(2)\_73 16S ribosomal RNA gene, partial sequence | 1799 | 1799 | 98% | 0.0 | 99% | KX603481.1 |
| 21Select seq KX603479.1 | Uncultured Pantoea sp. clone GJ\_(2)\_7 16S ribosomal RNA gene, partial sequence | 1799 | 1799 | 98% | 0.0 | 99% | KX603479.1 |
| 22Select seq KT580675.1 | Pantoea sp. CanR-85 16S ribosomal RNA gene, partial sequence | 1799 | 1799 | 98% | 0.0 | 99% | KT580675.1 |
| 23Select seq KT075193.1 | Pantoea agglomerans strain SSE 16S ribosomal RNA gene, partial sequence | 1799 | 1799 | 98% | 0.0 | 99% | KT075193.1 |
| 24Select seq KT075171.1 | Pantoea agglomerans strain NSF 16S ribosomal RNA gene, partial sequence | 1799 | 1799 | 98% | 0.0 | 99% | KT075171.1 |
| 25Select seq KT075169.1 | Pantoea agglomerans strain NSD 16S ribosomal RNA gene, partial sequence | 1799 | 1799 | 98% | 0.0 | 99% | KT075169.1 |
| 26Select seq KT075163.1 | Pantoea agglomerans strain GSC 16S ribosomal RNA gene, partial sequence | 1799 | 1799 | 98% | 0.0 | 99% | KT075163.1 |
| 27Select seq KT075162.1 | Pantoea agglomerans strain GSB 16S ribosomal RNA gene, partial sequence | 1799 | 1799 | 98% | 0.0 | 99% | KT075162.1 |
| 28Select seq KJ781904.1 | Pantoea agglomerans strain B81 16S ribosomal RNA gene, partial sequence | 1799 | 1799 | 98% | 0.0 | 99% | KJ781904.1 |
| 29Select seq KP099434.1 | Curtobacterium plantarum strain S4 16S ribosomal RNA gene, partial sequence | 1799 | 1799 | 98% | 0.0 | 99% | KP099434.1 |
| 30Select seq KP099433.1 | Curtobacterium plantarum strain S2 16S ribosomal RNA gene, partial sequence | 1799 | 1799 | 98% | 0.0 | 99% | KP099433.1 |
| 31Select seq LC007922.1 | Enterobacteriaceae bacterium X1/SB37 gene for 16S ribosomal RNA, partial sequence, strain: X1/SB37 | 1799 | 1799 | 98% | 0.0 | 99% | LC007922.1 |
| 32Select seq LC007878.1 | Enterobacteriaceae bacterium X1/SB52 gene for 16S ribosomal RNA, partial sequence, strain: X1/SB52 | 1799 | 1799 | 98% | 0.0 | 99% | LC007878.1 |
| 33Select seq LC007874.1 | Enterobacteriaceae bacterium X1/SB44 gene for 16S ribosomal RNA, partial sequence, strain: X1/SB44 | 1799 | 1799 | 98% | 0.0 | 99% | LC007874.1 |
| 34Select seq KF317872.1 | Pantoea agglomerans strain ex2 culture-collection CGMCC:7.103 16S ribosomal RNA gene, partial sequence | 1799 | 1799 | 98% | 0.0 | 99% | KF317872.1 |
| 35Select seq KF003386.1 | Pantoea agglomerans strain ABAC22 16S ribosomal RNA gene, partial sequence | 1799 | 1799 | 98% | 0.0 | 99% | KF003386.1 |
| 36Select seq JX067721.1 | Enterobacteriaceae bacterium SAP952.2 16S ribosomal RNA gene, partial sequence | 1799 | 1799 | 98% | 0.0 | 99% | JX067721.1 |
| 37Select seq JX067684.1 | Enterobacteriaceae bacterium SAP770.3 16S ribosomal RNA gene, partial sequence | 1799 | 1799 | 98% | 0.0 | 99% | JX067684.1 |
| 38Select seq JX067654.1 | Enterobacteriaceae bacterium SAP108.1 16S ribosomal RNA gene, partial sequence | 1799 | 1799 | 98% | 0.0 | 99% | JX067654.1 |
| 39Select seq JX257179.1 | Pantoea agglomerans strain Z01 16S ribosomal RNA gene, partial sequence | 1799 | 1799 | 98% | 0.0 | 99% | JX257179.1 |
| 40Select seq JX279936.1 | Uncultured Pantoea sp. clone WRFC18 16S ribosomal RNA gene, partial sequence | 1799 | 1799 | 98% | 0.0 | 99% | JX279936.1 |
| 41Select seq JF327484.1 | Pantoea agglomerans strain KB64 16S ribosomal RNA gene, partial sequence | 1799 | 1799 | 98% | 0.0 | 99% | JF327484.1 |
| 42Select seq JF327464.1 | Pantoea agglomerans strain KB38 16S ribosomal RNA gene, partial sequence | 1799 | 1799 | 98% | 0.0 | 99% | JF327464.1 |
| 43Select seq HM584289.1 | Pantoea agglomerans strain CJ-G-R2A5 16S ribosomal RNA gene, partial sequence | 1799 | 1799 | 98% | 0.0 | 99% | HM584289.1 |
| 44Select seq NR\_104943.1 | [Curtobacterium] plantarum strain CL63 16S ribosomal RNA, partial sequence | 1799 | 1799 | 98% | 0.0 | 99% | NR\_104943.1 |
| 45Select seq HM130689.1 | Pantoea agglomerans strain 1.2244 16S ribosomal RNA gene, partial sequence | 1799 | 1799 | 98% | 0.0 | 99% | HM130689.1 |
| 46Select seq FJ593000.1 | Pantoea agglomerans strain SR-1 16S ribosomal RNA gene, partial sequence | 1799 | 1799 | 98% | 0.0 | 99% | FJ593000.1 |
| 47Select seq FJ592996.1 | Pantoea agglomerans strain SN-2 16S ribosomal RNA gene, partial sequence | 1799 | 1799 | 98% | 0.0 | 99% | FJ592996.1 |
| 48Select seq FJ756348.1 | Pantoea agglomerans strain NZ 16S ribosomal RNA gene, partial sequence | 1799 | 1799 | 98% | 0.0 | 99% | FJ756348.1 |
| 49Select seq FJ611833.1 | Pantoea agglomerans strain VA21971 16S ribosomal RNA gene, partial sequence | 1799 | 1799 | 98% | 0.0 | 99% | FJ611833.1 |
| 50Select seq FJ611822.1 | Pantoea agglomerans strain P5WAM 16S ribosomal RNA gene, partial sequence | 1799 | 1799 | 98% | 0.0 | 99% | FJ611822.1 |
| 51Select seq FJ611821.1 | Pantoea agglomerans pv. gypsophilae strain ATCC 43348 16S ribosomal RNA gene, partial sequence | 1799 | 1799 | 98% | 0.0 | 99% | FJ611821.1 |
| 52Select seq FJ357809.1 | Pantoea agglomerans strain BBPE0103202 16S ribosomal RNA gene, partial sequence | 1799 | 1799 | 98% | 0.0 | 99% | FJ357809.1 |
| 53Select seq FJ267564.1 | Pantoea sp. II\_Gauze\_W\_10\_17 16S ribosomal RNA gene, partial sequence | 1799 | 1799 | 98% | 0.0 | 99% | FJ267564.1 |
| 54Select seq EU828365.1 | Pantoea sp. Wd5760 16S ribosomal RNA gene, partial sequence | 1799 | 1799 | 98% | 0.0 | 99% | EU828365.1 |
| 55Select seq AF130927.2 | Enterobacter agglomerans strain A57 16S ribosomal RNA, partial sequence | 1799 | 1799 | 98% | 0.0 | 99% | AF130927.2 |
| 56Select seq EU753130.1 | Marine bacterium MSC106 16S ribosomal RNA gene, partial sequence | 1799 | 1799 | 98% | 0.0 | 99% | EU753130.1 |
| 57Select seq EU598802.1 | Pantoea agglomerans 16S ribosomal RNA gene, partial sequence | 1799 | 1799 | 98% | 0.0 | 99% | EU598802.1 |
| 58Select seq EU047555.1 | Pantoea agglomerans strain TMPSB-P1 16S ribosomal RNA gene, partial sequence | 1799 | 1799 | 98% | 0.0 | 99% | EU047555.1 |
| 59Select seq DQ307453.1 | Pantoea agglomerans strain MM2 16S ribosomal RNA gene, partial sequence | 1799 | 1799 | 98% | 0.0 | 99% | DQ307453.1 |
| 60Select seq DQ307452.1 | Pantoea agglomerans strain FL1 16S ribosomal RNA gene, partial sequence | 1799 | 1799 | 98% | 0.0 | 99% | DQ307452.1 |
| 61Select seq DQ531643.1 | Pantoea sp. 2-2-SS-001 16S ribosomal RNA gene, partial sequence | 1799 | 1799 | 98% | 0.0 | 99% | DQ531643.1 |
| 62Select seq AB004757.2 | Pantoea agglomerans gene for 16S ribosomal RNA, partial sequence | 1799 | 1799 | 98% | 0.0 | 99% | AB004757.2 |
| 63Select seq DQ311006.1 | Pantoea sp. FL9 16S ribosomal RNA gene, partial sequence | 1799 | 1799 | 98% | 0.0 | 99% | DQ311006.1 |
| 64Select seq AJ583835.1 | Pantoea agglomerans partial 16S rRNA gene, strain Pa850T | 1799 | 1799 | 98% | 0.0 | 99% | AJ583835.1 |
| 65Select seq AJ583011.1 | Pantoea agglomerans partial 16S rRNA gene, strain EPS125 | 1799 | 1799 | 98% | 0.0 | 99% | AJ583011.1 |
| 66Select seq NR\_041978.1 | Pantoea agglomerans strain DSM 3493 16S ribosomal RNA gene, partial sequence | 1799 | 1799 | 98% | 0.0 | 99% | NR\_041978.1 |
| 67Select seq KR361753.1 | Pantoea agglomerans strain E60Y 16S ribosomal RNA gene, partial sequence | 1796 | 1796 | 98% | 0.0 | 99% | KR361753.1 |
| 68Select seq FJ357814.1 | Pantoea agglomerans strain CUETM8553 16S ribosomal RNA gene, partial sequence | 1796 | 1796 | 98% | 0.0 | 99% | FJ357814.1 |
| 69Select seq NR\_114735.1 | Pantoea agglomerans strain NCTC9381 16S ribosomal RNA gene, partial sequence | 1796 | 1796 | 98% | 0.0 | 99% | NR\_114735.1 |
| 70Select seq KX891508.1 | Pantoea agglomerans strain P1MNa\_1 16S ribosomal RNA gene, partial sequence | 1794 | 1794 | 98% | 0.0 | 99% | KX891508.1 |
| 71Select seq KU978278.1 | Uncultured bacterium clone EHB-P0445 #05 16S ribosomal RNA gene, partial sequence | 1794 | 1794 | 98% | 0.0 | 99% | KU978278.1 |
| 72Select seq KU978258.1 | Uncultured bacterium clone EHB-P0362 #05 16S ribosomal RNA gene, partial sequence | 1794 | 1794 | 98% | 0.0 | 99% | KU978258.1 |
| 73Select seq KY357288.1 | Pantoea agglomerans strain ACBP2 16S ribosomal RNA gene, partial sequence | 1794 | 1794 | 98% | 0.0 | 99% | KY357288.1 |
| 74Select seq KY357286.1 | Pantoea agglomerans strain ACBC2 16S ribosomal RNA gene, partial sequence | 1794 | 1794 | 98% | 0.0 | 99% | KY357286.1 |
| 75Select seq KT779903.1 | Pantoea agglomerans strain CCRI01 16S ribosomal RNA gene, partial sequence | 1794 | 1794 | 98% | 0.0 | 99% | KT779903.1 |
| 76Select seq KT075208.1 | Pantoea agglomerans strain WSA 16S ribosomal RNA gene, partial sequence | 1794 | 1794 | 98% | 0.0 | 99% | KT075208.1 |
| 77Select seq KT075191.1 | Pantoea agglomerans strain SSC 16S ribosomal RNA gene, partial sequence | 1794 | 1794 | 98% | 0.0 | 99% | KT075191.1 |
| 78Select seq KT075185.1 | Pantoea agglomerans strain QSD 16S ribosomal RNA gene, partial sequence | 1794 | 1794 | 98% | 0.0 | 99% | KT075185.1 |
| 79Select seq KP279978.1 | Curtobacterium plantarum strain NS1 16S ribosomal RNA gene, partial sequence | 1794 | 1794 | 98% | 0.0 | 99% | KP279978.1 |
| 80Select seq KP279964.1 | Curtobacterium plantarum strain KS1 16S ribosomal RNA gene, partial sequence | 1794 | 1794 | 98% | 0.0 | 99% | KP279964.1 |
| 81Select seq LC007911.1 | Enterobacteriaceae bacterium X1/SB86 gene for 16S ribosomal RNA, partial sequence, strain: X1/SB86 | 1794 | 1794 | 98% | 0.0 | 99% | LC007911.1 |
| 82Select seq LC007863.1 | Enterobacteriaceae bacterium X1/SB18 gene for 16S ribosomal RNA, partial sequence, strain: X1/SB18 | 1794 | 1794 | 98% | 0.0 | 99% | LC007863.1 |
| 83Select seq JX458430.1 | Pantoea sp. W1.09-234-1 16S ribosomal RNA gene, complete sequence | 1794 | 1794 | 98% | 0.0 | 99% | JX458430.1 |
| 84Select seq KF465938.1 | Pantoea sp. G2Ec2 16S ribosomal RNA gene, partial sequence | 1794 | 1794 | 98% | 0.0 | 99% | KF465938.1 |
| 85Select seq KF465834.1 | Pantoea agglomerans strain P19 16S ribosomal RNA gene, partial sequence | 1794 | 1794 | 98% | 0.0 | 99% | KF465834.1 |
| 86Select seq KF003418.1 | Pantoea agglomerans strain ABAC77 16S ribosomal RNA gene, partial sequence | 1794 | 1794 | 98% | 0.0 | 99% | KF003418.1 |
| 87Select seq KC178591.1 | Pantoea agglomerans strain Cl-17 16S ribosomal RNA gene, partial sequence | 1794 | 1794 | 98% | 0.0 | 99% | KC178591.1 |
| 88Select seq JX852648.1 | Uncultured Pantoea sp. clone WCFC7 16S ribosomal RNA gene, partial sequence | 1794 | 1794 | 98% | 0.0 | 99% | JX852648.1 |
| 89Select seq JX294750.1 | Uncultured Pantoea sp. clone WRFC196 16S ribosomal RNA gene, partial sequence | 1794 | 1794 | 98% | 0.0 | 99% | JX294750.1 |
| 90Select seq JX134624.1 | Pantoea agglomerans strain -Y115 16S ribosomal RNA gene, partial sequence | 1794 | 1794 | 98% | 0.0 | 99% | JX134624.1 |
| 91Select seq JX113242.1 | Pantoea agglomerans strain Y34 16S ribosomal RNA gene, partial sequence | 1794 | 1794 | 98% | 0.0 | 99% | JX113242.1 |
| 92Select seq JQ412064.1 | Pantoea sp. PR 16S ribosomal RNA gene, partial sequence | 1794 | 1794 | 98% | 0.0 | 99% | JQ412064.1 |
| 93Select seq HQ242740.1 | Pantoea agglomerans isolate PSB27 16S ribosomal RNA gene, partial sequence | 1794 | 1794 | 98% | 0.0 | 99% | HQ242740.1 |
| 94Select seq HQ242739.1 | Pantoea agglomerans isolate PSB26 16S ribosomal RNA gene, partial sequence | 1794 | 1794 | 98% | 0.0 | 99% | HQ242739.1 |
| 95Select seq CP002206.1 | Pantoea vagans C9-1, complete genome | 1794 | 12415 | 98% | 0.0 | 99% | CP002206.1 |
| 96Select seq FJ611843.1 | Pantoea agglomerans strain EM21cb 16S ribosomal RNA gene, partial sequence | 1792 | 1792 | 98% | 0.0 | 99% | FJ611843.1 |
| 97Select seq FJ611842.1 | Pantoea agglomerans strain Eh325 16S ribosomal RNA gene, partial sequence | 1792 | 1792 | 98% | 0.0 | 99% | FJ611842.1 |
| 98Select seq NR\_116751.1 | Pantoea agglomerans strain LMG 1286 16S ribosomal RNA gene, partial sequence | 1792 | 1792 | 98% | 0.0 | 99% | NR\_116751.1 |
| 99Select seq KC283045.1 | Uncultured Pantoea sp. clone WCFC2 16S ribosomal RNA gene, partial sequence | 1790 | 1790 | 98% | 0.0 | 99% | KC283045.1 |
| 100Select seq JX067704.1 | Enterobacteriaceae bacterium SAP822.1 16S ribosomal RNA gene, partial sequence | 1781 | 1781 | 98% | 0.0 | 99% | JX067704.1 |


## Alignments

Loading alignment... for sequences gi|1003098379,gi|902659494,gi|523584558,gi|1344462721,gi|1332982380 Reading indexes 1-5

Download

FASTA (complete sequence)

FASTA (aligned sequences)

GenBank (complete sequence)

Continue
Cancel

GenBankGraphics

Next
Previous
Descriptions

Pantoea agglomerans strain BL26 16S ribosomal RNA gene, partial sequence

Sequence ID: KU258277.1Length: 1386Number of Matches: 1

Related Information

Range 1: 385 to 1371GenBankGraphics

Next Match
Previous Match
First Match

Alignment statistics for match #1

| Score | Expect | Identities | Gaps | Strand | Frame |
| --- | --- | --- | --- | --- | --- |
| 1801 bits(975) | 0.0() | 981/987(99%) | 0/987(0%) | Plus/Minus |  |

Features:

```
Query  7     GCCCTCCCGAAGGTTANGCTACCTACTTCTTTTNNANCCCACTCCCATGGTGTGACGGGC  66
             |||||||||||||||| ||||||||||||||||  | |||||||||||||||||||||||
Sbjct  1371  GCCCTCCCGAAGGTTAAGCTACCTACTTCTTTTGCAACCCACTCCCATGGTGTGACGGGC  1312

Query  67    GGTGTGTACAAGGCCCGGGAACGTATTCACCGTGGCATTCTGATCCACGATTACTAGCGA  126
             ||||||||||||||||||||||||||||||||||||||||||||||||||||||||||||
Sbjct  1311  GGTGTGTACAAGGCCCGGGAACGTATTCACCGTGGCATTCTGATCCACGATTACTAGCGA  1252

Query  127   TTCCGACTTCACGGAGTCGAGTTGCAGACTCCGATCCGGACTACGACGCACTTTGTGAGG  186
             ||||||||||||||||||||||||||||||||||||||||||||||||||||||||||||
Sbjct  1251  TTCCGACTTCACGGAGTCGAGTTGCAGACTCCGATCCGGACTACGACGCACTTTGTGAGG  1192

Query  187   TCCGCTTGCTCTCGCGAGGTCGCTTCTCTTTGTATGCGCCATTGTAGCACGTGTGTAGCC  246
             ||||||||||||||||||||||||||||||||||||||||||||||||||||||||||||
Sbjct  1191  TCCGCTTGCTCTCGCGAGGTCGCTTCTCTTTGTATGCGCCATTGTAGCACGTGTGTAGCC  1132

Query  247   CTACTCGTAAGGGCCATGATGACTTGACGTCATCCCCACCTTCCTCCGGTTTATCACCGG  306
             ||||||||||||||||||||||||||||||||||||||||||||||||||||||||||||
Sbjct  1131  CTACTCGTAAGGGCCATGATGACTTGACGTCATCCCCACCTTCCTCCGGTTTATCACCGG  1072

Query  307   CAGTCTCCTTTGAGTTCCCGACCGAATCGCTGGCAACAAAGGATAAGGGTTGCGCTCGTT  366
             ||||||||||||||||||||||||||||||||||||||||||||||||||||||||||||
Sbjct  1071  CAGTCTCCTTTGAGTTCCCGACCGAATCGCTGGCAACAAAGGATAAGGGTTGCGCTCGTT  1012

Query  367   GCGGGACTTAACCCAACATTTCACAACACGAGCTGACGACAGCCATGCAGCACCTGTCTC  426
             ||||||||||||||||||||||||||||||||||||||||||||||||||||||||||||
Sbjct  1011  GCGGGACTTAACCCAACATTTCACAACACGAGCTGACGACAGCCATGCAGCACCTGTCTC  952

Query  427   ACGGTTCCCGAAGGCACTAAGGCATCTCTGNNAAATTCCGTGGATGTCAAGAGTAGGTAA  486
             ||||||||||||||||||||||||||||||  ||||||||||||||||||||||||||||
Sbjct  951   ACGGTTCCCGAAGGCACTAAGGCATCTCTGCCAAATTCCGTGGATGTCAAGAGTAGGTAA  892

Query  487   GGTTCTTCGCGTTGCATCGAATTAAACCACATGCTCCACCGCTTGTGCGGGCCCCCGTCA  546
             ||||||||||||||||||||||||||||||||||||||||||||||||||||||||||||
Sbjct  891   GGTTCTTCGCGTTGCATCGAATTAAACCACATGCTCCACCGCTTGTGCGGGCCCCCGTCA  832

Query  547   ATTCATTTGAGTTTTAACCTTGCGGCCGTACTCCCCAGGCGGTCGACTTAACGCGTTAGC  606
             ||||||||||||||||||||||||||||||||||||||||||||||||||||||||||||
Sbjct  831   ATTCATTTGAGTTTTAACCTTGCGGCCGTACTCCCCAGGCGGTCGACTTAACGCGTTAGC  772

Query  607   TCCGGAAGCCACTCCTCAAGGGAACAACCTCCAAGTCGACATCGTTTACGGCGTGGACTA  666
             ||||||||||||||||||||||||||||||||||||||||||||||||||||||||||||
Sbjct  771   TCCGGAAGCCACTCCTCAAGGGAACAACCTCCAAGTCGACATCGTTTACGGCGTGGACTA  712

Query  667   CCAGGGTATCTAATCCTGTTTGCTCCCCACGCTTTCGCACCTGAGCGTCAGTCTTTGTCC  726
             ||||||||||||||||||||||||||||||||||||||||||||||||||||||||||||
Sbjct  711   CCAGGGTATCTAATCCTGTTTGCTCCCCACGCTTTCGCACCTGAGCGTCAGTCTTTGTCC  652

Query  727   AGGGGGCCGCCTTCGCCACCGGTATTCCTCCAGATCTCTACGCATTTCACCGCTACACCT  786
             ||||||||||||||||||||||||||||||||||||||||||||||||||||||||||||
Sbjct  651   AGGGGGCCGCCTTCGCCACCGGTATTCCTCCAGATCTCTACGCATTTCACCGCTACACCT  592

Query  787   GGAATTCTACCCCCCTCTACAAGACTCAAGCCTGCCAGTTTCAAATGCAGTTCCCAGGTT  846
             ||||||||||||||||||||||||||||||||||||||||||||||||||||||||||||
Sbjct  591   GGAATTCTACCCCCCTCTACAAGACTCAAGCCTGCCAGTTTCAAATGCAGTTCCCAGGTT  532

Query  847   AAGCCCGGGGATTTCACATCTGACTTAACAGACCGCCTGCGTGCGCTTTACGCCCAGTAA  906
             ||||||||||||||||||||||||||||||||||||||||||||||||||||||||||||
Sbjct  531   AAGCCCGGGGATTTCACATCTGACTTAACAGACCGCCTGCGTGCGCTTTACGCCCAGTAA  472

Query  907   TTCCGATTAACGCTTGCACCCTCCGTATTACCGCGGCTGCTGGCACGGAGTTAGCCGGTG  966
             ||||||||||||||||||||||||||||||||||||||||||||||||||||||||||||
Sbjct  471   TTCCGATTAACGCTTGCACCCTCCGTATTACCGCGGCTGCTGGCACGGAGTTAGCCGGTG  412

Query  967   CTTCTTCTGCGGGTAACGTCAATCGAT  993
             |||||||||||||||||||||||||||
Sbjct  411   CTTCTTCTGCGGGTAACGTCAATCGAT  385
```

Download

FASTA (complete sequence)

FASTA (aligned sequences)

GenBank (complete sequence)

Continue
Cancel

GenBankGraphics

Next
Previous
Descriptions

Pantoea agglomerans strain QSC 16S ribosomal RNA gene, partial sequence

Sequence ID: KT075184.1Length: 1502Number of Matches: 1

Related Information

Range 1: 466 to 1452GenBankGraphics

Next Match
Previous Match
First Match

Alignment statistics for match #1

| Score | Expect | Identities | Gaps | Strand | Frame |
| --- | --- | --- | --- | --- | --- |
| 1801 bits(975) | 0.0() | 981/987(99%) | 0/987(0%) | Plus/Minus |  |

Features:

```
Query  7     GCCCTCCCGAAGGTTANGCTACCTACTTCTTTTNNANCCCACTCCCATGGTGTGACGGGC  66
             |||||||||||||||| ||||||||||||||||  | |||||||||||||||||||||||
Sbjct  1452  GCCCTCCCGAAGGTTAAGCTACCTACTTCTTTTGCAACCCACTCCCATGGTGTGACGGGC  1393

Query  67    GGTGTGTACAAGGCCCGGGAACGTATTCACCGTGGCATTCTGATCCACGATTACTAGCGA  126
             ||||||||||||||||||||||||||||||||||||||||||||||||||||||||||||
Sbjct  1392  GGTGTGTACAAGGCCCGGGAACGTATTCACCGTGGCATTCTGATCCACGATTACTAGCGA  1333

Query  127   TTCCGACTTCACGGAGTCGAGTTGCAGACTCCGATCCGGACTACGACGCACTTTGTGAGG  186
             ||||||||||||||||||||||||||||||||||||||||||||||||||||||||||||
Sbjct  1332  TTCCGACTTCACGGAGTCGAGTTGCAGACTCCGATCCGGACTACGACGCACTTTGTGAGG  1273

Query  187   TCCGCTTGCTCTCGCGAGGTCGCTTCTCTTTGTATGCGCCATTGTAGCACGTGTGTAGCC  246
             ||||||||||||||||||||||||||||||||||||||||||||||||||||||||||||
Sbjct  1272  TCCGCTTGCTCTCGCGAGGTCGCTTCTCTTTGTATGCGCCATTGTAGCACGTGTGTAGCC  1213

Query  247   CTACTCGTAAGGGCCATGATGACTTGACGTCATCCCCACCTTCCTCCGGTTTATCACCGG  306
             ||||||||||||||||||||||||||||||||||||||||||||||||||||||||||||
Sbjct  1212  CTACTCGTAAGGGCCATGATGACTTGACGTCATCCCCACCTTCCTCCGGTTTATCACCGG  1153

Query  307   CAGTCTCCTTTGAGTTCCCGACCGAATCGCTGGCAACAAAGGATAAGGGTTGCGCTCGTT  366
             ||||||||||||||||||||||||||||||||||||||||||||||||||||||||||||
Sbjct  1152  CAGTCTCCTTTGAGTTCCCGACCGAATCGCTGGCAACAAAGGATAAGGGTTGCGCTCGTT  1093

Query  367   GCGGGACTTAACCCAACATTTCACAACACGAGCTGACGACAGCCATGCAGCACCTGTCTC  426
             ||||||||||||||||||||||||||||||||||||||||||||||||||||||||||||
Sbjct  1092  GCGGGACTTAACCCAACATTTCACAACACGAGCTGACGACAGCCATGCAGCACCTGTCTC  1033

Query  427   ACGGTTCCCGAAGGCACTAAGGCATCTCTGNNAAATTCCGTGGATGTCAAGAGTAGGTAA  486
             ||||||||||||||||||||||||||||||  ||||||||||||||||||||||||||||
Sbjct  1032  ACGGTTCCCGAAGGCACTAAGGCATCTCTGCCAAATTCCGTGGATGTCAAGAGTAGGTAA  973

Query  487   GGTTCTTCGCGTTGCATCGAATTAAACCACATGCTCCACCGCTTGTGCGGGCCCCCGTCA  546
             ||||||||||||||||||||||||||||||||||||||||||||||||||||||||||||
Sbjct  972   GGTTCTTCGCGTTGCATCGAATTAAACCACATGCTCCACCGCTTGTGCGGGCCCCCGTCA  913

Query  547   ATTCATTTGAGTTTTAACCTTGCGGCCGTACTCCCCAGGCGGTCGACTTAACGCGTTAGC  606
             ||||||||||||||||||||||||||||||||||||||||||||||||||||||||||||
Sbjct  912   ATTCATTTGAGTTTTAACCTTGCGGCCGTACTCCCCAGGCGGTCGACTTAACGCGTTAGC  853

Query  607   TCCGGAAGCCACTCCTCAAGGGAACAACCTCCAAGTCGACATCGTTTACGGCGTGGACTA  666
             ||||||||||||||||||||||||||||||||||||||||||||||||||||||||||||
Sbjct  852   TCCGGAAGCCACTCCTCAAGGGAACAACCTCCAAGTCGACATCGTTTACGGCGTGGACTA  793

Query  667   CCAGGGTATCTAATCCTGTTTGCTCCCCACGCTTTCGCACCTGAGCGTCAGTCTTTGTCC  726
             ||||||||||||||||||||||||||||||||||||||||||||||||||||||||||||
Sbjct  792   CCAGGGTATCTAATCCTGTTTGCTCCCCACGCTTTCGCACCTGAGCGTCAGTCTTTGTCC  733

Query  727   AGGGGGCCGCCTTCGCCACCGGTATTCCTCCAGATCTCTACGCATTTCACCGCTACACCT  786
             ||||||||||||||||||||||||||||||||||||||||||||||||||||||||||||
Sbjct  732   AGGGGGCCGCCTTCGCCACCGGTATTCCTCCAGATCTCTACGCATTTCACCGCTACACCT  673

Query  787   GGAATTCTACCCCCCTCTACAAGACTCAAGCCTGCCAGTTTCAAATGCAGTTCCCAGGTT  846
             ||||||||||||||||||||||||||||||||||||||||||||||||||||||||||||
Sbjct  672   GGAATTCTACCCCCCTCTACAAGACTCAAGCCTGCCAGTTTCAAATGCAGTTCCCAGGTT  613

Query  847   AAGCCCGGGGATTTCACATCTGACTTAACAGACCGCCTGCGTGCGCTTTACGCCCAGTAA  906
             ||||||||||||||||||||||||||||||||||||||||||||||||||||||||||||
Sbjct  612   AAGCCCGGGGATTTCACATCTGACTTAACAGACCGCCTGCGTGCGCTTTACGCCCAGTAA  553

Query  907   TTCCGATTAACGCTTGCACCCTCCGTATTACCGCGGCTGCTGGCACGGAGTTAGCCGGTG  966
             ||||||||||||||||||||||||||||||||||||||||||||||||||||||||||||
Sbjct  552   TTCCGATTAACGCTTGCACCCTCCGTATTACCGCGGCTGCTGGCACGGAGTTAGCCGGTG  493

Query  967   CTTCTTCTGCGGGTAACGTCAATCGAT  993
             |||||||||||||||||||||||||||
Sbjct  492   CTTCTTCTGCGGGTAACGTCAATCGAT  466
```

Download

FASTA (complete sequence)

FASTA (aligned sequences)

GenBank (complete sequence)

Continue
Cancel

GenBankGraphics

Next
Previous
Descriptions

Pantoea sp. E100 16S ribosomal RNA gene, partial sequence

Sequence ID: KF153221.1Length: 1501Number of Matches: 1

Related Information

Range 1: 465 to 1451GenBankGraphics

Next Match
Previous Match
First Match

Alignment statistics for match #1

| Score | Expect | Identities | Gaps | Strand | Frame |
| --- | --- | --- | --- | --- | --- |
| 1801 bits(975) | 0.0() | 981/987(99%) | 0/987(0%) | Plus/Minus |  |

Features:

```
Query  7     GCCCTCCCGAAGGTTANGCTACCTACTTCTTTTNNANCCCACTCCCATGGTGTGACGGGC  66
             |||||||||||||||| ||||||||||||||||  | |||||||||||||||||||||||
Sbjct  1451  GCCCTCCCGAAGGTTAAGCTACCTACTTCTTTTGCAACCCACTCCCATGGTGTGACGGGC  1392

Query  67    GGTGTGTACAAGGCCCGGGAACGTATTCACCGTGGCATTCTGATCCACGATTACTAGCGA  126
             ||||||||||||||||||||||||||||||||||||||||||||||||||||||||||||
Sbjct  1391  GGTGTGTACAAGGCCCGGGAACGTATTCACCGTGGCATTCTGATCCACGATTACTAGCGA  1332

Query  127   TTCCGACTTCACGGAGTCGAGTTGCAGACTCCGATCCGGACTACGACGCACTTTGTGAGG  186
             ||||||||||||||||||||||||||||||||||||||||||||||||||||||||||||
Sbjct  1331  TTCCGACTTCACGGAGTCGAGTTGCAGACTCCGATCCGGACTACGACGCACTTTGTGAGG  1272

Query  187   TCCGCTTGCTCTCGCGAGGTCGCTTCTCTTTGTATGCGCCATTGTAGCACGTGTGTAGCC  246
             ||||||||||||||||||||||||||||||||||||||||||||||||||||||||||||
Sbjct  1271  TCCGCTTGCTCTCGCGAGGTCGCTTCTCTTTGTATGCGCCATTGTAGCACGTGTGTAGCC  1212

Query  247   CTACTCGTAAGGGCCATGATGACTTGACGTCATCCCCACCTTCCTCCGGTTTATCACCGG  306
             ||||||||||||||||||||||||||||||||||||||||||||||||||||||||||||
Sbjct  1211  CTACTCGTAAGGGCCATGATGACTTGACGTCATCCCCACCTTCCTCCGGTTTATCACCGG  1152

Query  307   CAGTCTCCTTTGAGTTCCCGACCGAATCGCTGGCAACAAAGGATAAGGGTTGCGCTCGTT  366
             ||||||||||||||||||||||||||||||||||||||||||||||||||||||||||||
Sbjct  1151  CAGTCTCCTTTGAGTTCCCGACCGAATCGCTGGCAACAAAGGATAAGGGTTGCGCTCGTT  1092

Query  367   GCGGGACTTAACCCAACATTTCACAACACGAGCTGACGACAGCCATGCAGCACCTGTCTC  426
             ||||||||||||||||||||||||||||||||||||||||||||||||||||||||||||
Sbjct  1091  GCGGGACTTAACCCAACATTTCACAACACGAGCTGACGACAGCCATGCAGCACCTGTCTC  1032

Query  427   ACGGTTCCCGAAGGCACTAAGGCATCTCTGNNAAATTCCGTGGATGTCAAGAGTAGGTAA  486
             ||||||||||||||||||||||||||||||  ||||||||||||||||||||||||||||
Sbjct  1031  ACGGTTCCCGAAGGCACTAAGGCATCTCTGCCAAATTCCGTGGATGTCAAGAGTAGGTAA  972

Query  487   GGTTCTTCGCGTTGCATCGAATTAAACCACATGCTCCACCGCTTGTGCGGGCCCCCGTCA  546
             ||||||||||||||||||||||||||||||||||||||||||||||||||||||||||||
Sbjct  971   GGTTCTTCGCGTTGCATCGAATTAAACCACATGCTCCACCGCTTGTGCGGGCCCCCGTCA  912

Query  547   ATTCATTTGAGTTTTAACCTTGCGGCCGTACTCCCCAGGCGGTCGACTTAACGCGTTAGC  606
             ||||||||||||||||||||||||||||||||||||||||||||||||||||||||||||
Sbjct  911   ATTCATTTGAGTTTTAACCTTGCGGCCGTACTCCCCAGGCGGTCGACTTAACGCGTTAGC  852

Query  607   TCCGGAAGCCACTCCTCAAGGGAACAACCTCCAAGTCGACATCGTTTACGGCGTGGACTA  666
             ||||||||||||||||||||||||||||||||||||||||||||||||||||||||||||
Sbjct  851   TCCGGAAGCCACTCCTCAAGGGAACAACCTCCAAGTCGACATCGTTTACGGCGTGGACTA  792

Query  667   CCAGGGTATCTAATCCTGTTTGCTCCCCACGCTTTCGCACCTGAGCGTCAGTCTTTGTCC  726
             ||||||||||||||||||||||||||||||||||||||||||||||||||||||||||||
Sbjct  791   CCAGGGTATCTAATCCTGTTTGCTCCCCACGCTTTCGCACCTGAGCGTCAGTCTTTGTCC  732

Query  727   AGGGGGCCGCCTTCGCCACCGGTATTCCTCCAGATCTCTACGCATTTCACCGCTACACCT  786
             ||||||||||||||||||||||||||||||||||||||||||||||||||||||||||||
Sbjct  731   AGGGGGCCGCCTTCGCCACCGGTATTCCTCCAGATCTCTACGCATTTCACCGCTACACCT  672

Query  787   GGAATTCTACCCCCCTCTACAAGACTCAAGCCTGCCAGTTTCAAATGCAGTTCCCAGGTT  846
             ||||||||||||||||||||||||||||||||||||||||||||||||||||||||||||
Sbjct  671   GGAATTCTACCCCCCTCTACAAGACTCAAGCCTGCCAGTTTCAAATGCAGTTCCCAGGTT  612

Query  847   AAGCCCGGGGATTTCACATCTGACTTAACAGACCGCCTGCGTGCGCTTTACGCCCAGTAA  906
             ||||||||||||||||||||||||||||||||||||||||||||||||||||||||||||
Sbjct  611   AAGCCCGGGGATTTCACATCTGACTTAACAGACCGCCTGCGTGCGCTTTACGCCCAGTAA  552

Query  907   TTCCGATTAACGCTTGCACCCTCCGTATTACCGCGGCTGCTGGCACGGAGTTAGCCGGTG  966
             ||||||||||||||||||||||||||||||||||||||||||||||||||||||||||||
Sbjct  551   TTCCGATTAACGCTTGCACCCTCCGTATTACCGCGGCTGCTGGCACGGAGTTAGCCGGTG  492

Query  967   CTTCTTCTGCGGGTAACGTCAATCGAT  993
             |||||||||||||||||||||||||||
Sbjct  491   CTTCTTCTGCGGGTAACGTCAATCGAT  465
```

Download

FASTA (complete sequence)

FASTA (aligned sequences)

GenBank (complete sequence)

Continue
Cancel

GenBankGraphics

Sort by:

E value
 Score
Percent identity
Query start position
Subject start position

Next
Previous
Descriptions

Pantoea vagans strain FDAARGOS\_160 chromosome, complete genome

Sequence ID: CP014129.2Length: 4050121Number of Matches: 7

Related Information

Range 1: 49769 to 50754GenBankGraphics

Next Match
Previous Match
First Match

Alignment statistics for match #1

| Score | Expect | Identities | Gaps | Strand | Frame |
| --- | --- | --- | --- | --- | --- |
| 1799 bits(974) | 0.0() | 980/986(99%) | 0/986(0%) | Plus/Plus |  |

Features:

```
Query  7      GCCCTCCCGAAGGTTANGCTACCTACTTCTTTTNNANCCCACTCCCATGGTGTGACGGGC  66
              |||||||||||||||| ||||||||||||||||  | |||||||||||||||||||||||
Sbjct  49769  GCCCTCCCGAAGGTTAAGCTACCTACTTCTTTTGCAACCCACTCCCATGGTGTGACGGGC  49828

Query  67     GGTGTGTACAAGGCCCGGGAACGTATTCACCGTGGCATTCTGATCCACGATTACTAGCGA  126
              ||||||||||||||||||||||||||||||||||||||||||||||||||||||||||||
Sbjct  49829  GGTGTGTACAAGGCCCGGGAACGTATTCACCGTGGCATTCTGATCCACGATTACTAGCGA  49888

Query  127    TTCCGACTTCACGGAGTCGAGTTGCAGACTCCGATCCGGACTACGACGCACTTTGTGAGG  186
              ||||||||||||||||||||||||||||||||||||||||||||||||||||||||||||
Sbjct  49889  TTCCGACTTCACGGAGTCGAGTTGCAGACTCCGATCCGGACTACGACGCACTTTGTGAGG  49948

Query  187    TCCGCTTGCTCTCGCGAGGTCGCTTCTCTTTGTATGCGCCATTGTAGCACGTGTGTAGCC  246
              ||||||||||||||||||||||||||||||||||||||||||||||||||||||||||||
Sbjct  49949  TCCGCTTGCTCTCGCGAGGTCGCTTCTCTTTGTATGCGCCATTGTAGCACGTGTGTAGCC  50008

Query  247    CTACTCGTAAGGGCCATGATGACTTGACGTCATCCCCACCTTCCTCCGGTTTATCACCGG  306
              ||||||||||||||||||||||||||||||||||||||||||||||||||||||||||||
Sbjct  50009  CTACTCGTAAGGGCCATGATGACTTGACGTCATCCCCACCTTCCTCCGGTTTATCACCGG  50068

Query  307    CAGTCTCCTTTGAGTTCCCGACCGAATCGCTGGCAACAAAGGATAAGGGTTGCGCTCGTT  366
              ||||||||||||||||||||||||||||||||||||||||||||||||||||||||||||
Sbjct  50069  CAGTCTCCTTTGAGTTCCCGACCGAATCGCTGGCAACAAAGGATAAGGGTTGCGCTCGTT  50128

Query  367    GCGGGACTTAACCCAACATTTCACAACACGAGCTGACGACAGCCATGCAGCACCTGTCTC  426
              ||||||||||||||||||||||||||||||||||||||||||||||||||||||||||||
Sbjct  50129  GCGGGACTTAACCCAACATTTCACAACACGAGCTGACGACAGCCATGCAGCACCTGTCTC  50188

Query  427    ACGGTTCCCGAAGGCACTAAGGCATCTCTGNNAAATTCCGTGGATGTCAAGAGTAGGTAA  486
              ||||||||||||||||||||||||||||||  ||||||||||||||||||||||||||||
Sbjct  50189  ACGGTTCCCGAAGGCACTAAGGCATCTCTGCCAAATTCCGTGGATGTCAAGAGTAGGTAA  50248

Query  487    GGTTCTTCGCGTTGCATCGAATTAAACCACATGCTCCACCGCTTGTGCGGGCCCCCGTCA  546
              ||||||||||||||||||||||||||||||||||||||||||||||||||||||||||||
Sbjct  50249  GGTTCTTCGCGTTGCATCGAATTAAACCACATGCTCCACCGCTTGTGCGGGCCCCCGTCA  50308

Query  547    ATTCATTTGAGTTTTAACCTTGCGGCCGTACTCCCCAGGCGGTCGACTTAACGCGTTAGC  606
              ||||||||||||||||||||||||||||||||||||||||||||||||||||||||||||
Sbjct  50309  ATTCATTTGAGTTTTAACCTTGCGGCCGTACTCCCCAGGCGGTCGACTTAACGCGTTAGC  50368

Query  607    TCCGGAAGCCACTCCTCAAGGGAACAACCTCCAAGTCGACATCGTTTACGGCGTGGACTA  666
              ||||||||||||||||||||||||||||||||||||||||||||||||||||||||||||
Sbjct  50369  TCCGGAAGCCACTCCTCAAGGGAACAACCTCCAAGTCGACATCGTTTACGGCGTGGACTA  50428

Query  667    CCAGGGTATCTAATCCTGTTTGCTCCCCACGCTTTCGCACCTGAGCGTCAGTCTTTGTCC  726
              ||||||||||||||||||||||||||||||||||||||||||||||||||||||||||||
Sbjct  50429  CCAGGGTATCTAATCCTGTTTGCTCCCCACGCTTTCGCACCTGAGCGTCAGTCTTTGTCC  50488

Query  727    AGGGGGCCGCCTTCGCCACCGGTATTCCTCCAGATCTCTACGCATTTCACCGCTACACCT  786
              ||||||||||||||||||||||||||||||||||||||||||||||||||||||||||||
Sbjct  50489  AGGGGGCCGCCTTCGCCACCGGTATTCCTCCAGATCTCTACGCATTTCACCGCTACACCT  50548

Query  787    GGAATTCTACCCCCCTCTACAAGACTCAAGCCTGCCAGTTTCAAATGCAGTTCCCAGGTT  846
              ||||||||||||||||||||||||||||||||||||||||||||||||||||||||||||
Sbjct  50549  GGAATTCTACCCCCCTCTACAAGACTCAAGCCTGCCAGTTTCAAATGCAGTTCCCAGGTT  50608

Query  847    AAGCCCGGGGATTTCACATCTGACTTAACAGACCGCCTGCGTGCGCTTTACGCCCAGTAA  906
              ||||||||||||||||||||||||||||||||||||||||||||||||||||||||||||
Sbjct  50609  AAGCCCGGGGATTTCACATCTGACTTAACAGACCGCCTGCGTGCGCTTTACGCCCAGTAA  50668

Query  907    TTCCGATTAACGCTTGCACCCTCCGTATTACCGCGGCTGCTGGCACGGAGTTAGCCGGTG  966
              ||||||||||||||||||||||||||||||||||||||||||||||||||||||||||||
Sbjct  50669  TTCCGATTAACGCTTGCACCCTCCGTATTACCGCGGCTGCTGGCACGGAGTTAGCCGGTG  50728

Query  967    CTTCTTCTGCGGGTAACGTCAATCGA  992
              ||||||||||||||||||||||||||
Sbjct  50729  CTTCTTCTGCGGGTAACGTCAATCGA  50754
```

Range 2: 171037 to 172021GenBankGraphics

Next Match
Previous Match
First Match

Alignment statistics for match #2

| Score | Expect | Identities | Gaps | Strand | Frame |
| --- | --- | --- | --- | --- | --- |
| 1797 bits(973) | 0.0() | 979/985(99%) | 0/985(0%) | Plus/Plus |  |

Features:

```
Query  7       GCCCTCCCGAAGGTTANGCTACCTACTTCTTTTNNANCCCACTCCCATGGTGTGACGGGC  66
               |||||||||||||||| ||||||||||||||||  | |||||||||||||||||||||||
Sbjct  171037  GCCCTCCCGAAGGTTAAGCTACCTACTTCTTTTGCAACCCACTCCCATGGTGTGACGGGC  171096

Query  67      GGTGTGTACAAGGCCCGGGAACGTATTCACCGTGGCATTCTGATCCACGATTACTAGCGA  126
               ||||||||||||||||||||||||||||||||||||||||||||||||||||||||||||
Sbjct  171097  GGTGTGTACAAGGCCCGGGAACGTATTCACCGTGGCATTCTGATCCACGATTACTAGCGA  171156

Query  127     TTCCGACTTCACGGAGTCGAGTTGCAGACTCCGATCCGGACTACGACGCACTTTGTGAGG  186
               ||||||||||||||||||||||||||||||||||||||||||||||||||||||||||||
Sbjct  171157  TTCCGACTTCACGGAGTCGAGTTGCAGACTCCGATCCGGACTACGACGCACTTTGTGAGG  171216

Query  187     TCCGCTTGCTCTCGCGAGGTCGCTTCTCTTTGTATGCGCCATTGTAGCACGTGTGTAGCC  246
               ||||||||||||||||||||||||||||||||||||||||||||||||||||||||||||
Sbjct  171217  TCCGCTTGCTCTCGCGAGGTCGCTTCTCTTTGTATGCGCCATTGTAGCACGTGTGTAGCC  171276

Query  247     CTACTCGTAAGGGCCATGATGACTTGACGTCATCCCCACCTTCCTCCGGTTTATCACCGG  306
               ||||||||||||||||||||||||||||||||||||||||||||||||||||||||||||
Sbjct  171277  CTACTCGTAAGGGCCATGATGACTTGACGTCATCCCCACCTTCCTCCGGTTTATCACCGG  171336

Query  307     CAGTCTCCTTTGAGTTCCCGACCGAATCGCTGGCAACAAAGGATAAGGGTTGCGCTCGTT  366
               ||||||||||||||||||||||||||||||||||||||||||||||||||||||||||||
Sbjct  171337  CAGTCTCCTTTGAGTTCCCGACCGAATCGCTGGCAACAAAGGATAAGGGTTGCGCTCGTT  171396

Query  367     GCGGGACTTAACCCAACATTTCACAACACGAGCTGACGACAGCCATGCAGCACCTGTCTC  426
               ||||||||||||||||||||||||||||||||||||||||||||||||||||||||||||
Sbjct  171397  GCGGGACTTAACCCAACATTTCACAACACGAGCTGACGACAGCCATGCAGCACCTGTCTC  171456

Query  427     ACGGTTCCCGAAGGCACTAAGGCATCTCTGNNAAATTCCGTGGATGTCAAGAGTAGGTAA  486
               ||||||||||||||||||||||||||||||  ||||||||||||||||||||||||||||
Sbjct  171457  ACGGTTCCCGAAGGCACTAAGGCATCTCTGCCAAATTCCGTGGATGTCAAGAGTAGGTAA  171516

Query  487     GGTTCTTCGCGTTGCATCGAATTAAACCACATGCTCCACCGCTTGTGCGGGCCCCCGTCA  546
               ||||||||||||||||||||||||||||||||||||||||||||||||||||||||||||
Sbjct  171517  GGTTCTTCGCGTTGCATCGAATTAAACCACATGCTCCACCGCTTGTGCGGGCCCCCGTCA  171576

Query  547     ATTCATTTGAGTTTTAACCTTGCGGCCGTACTCCCCAGGCGGTCGACTTAACGCGTTAGC  606
               ||||||||||||||||||||||||||||||||||||||||||||||||||||||||||||
Sbjct  171577  ATTCATTTGAGTTTTAACCTTGCGGCCGTACTCCCCAGGCGGTCGACTTAACGCGTTAGC  171636

Query  607     TCCGGAAGCCACTCCTCAAGGGAACAACCTCCAAGTCGACATCGTTTACGGCGTGGACTA  666
               ||||||||||||||||||||||||||||||||||||||||||||||||||||||||||||
Sbjct  171637  TCCGGAAGCCACTCCTCAAGGGAACAACCTCCAAGTCGACATCGTTTACGGCGTGGACTA  171696

Query  667     CCAGGGTATCTAATCCTGTTTGCTCCCCACGCTTTCGCACCTGAGCGTCAGTCTTTGTCC  726
               ||||||||||||||||||||||||||||||||||||||||||||||||||||||||||||
Sbjct  171697  CCAGGGTATCTAATCCTGTTTGCTCCCCACGCTTTCGCACCTGAGCGTCAGTCTTTGTCC  171756

Query  727     AGGGGGCCGCCTTCGCCACCGGTATTCCTCCAGATCTCTACGCATTTCACCGCTACACCT  786
               ||||||||||||||||||||||||||||||||||||||||||||||||||||||||||||
Sbjct  171757  AGGGGGCCGCCTTCGCCACCGGTATTCCTCCAGATCTCTACGCATTTCACCGCTACACCT  171816

Query  787     GGAATTCTACCCCCCTCTACAAGACTCAAGCCTGCCAGTTTCAAATGCAGTTCCCAGGTT  846
               ||||||||||||||||||||||||||||||||||||||||||||||||||||||||||||
Sbjct  171817  GGAATTCTACCCCCCTCTACAAGACTCAAGCCTGCCAGTTTCAAATGCAGTTCCCAGGTT  171876

Query  847     AAGCCCGGGGATTTCACATCTGACTTAACAGACCGCCTGCGTGCGCTTTACGCCCAGTAA  906
               ||||||||||||||||||||||||||||||||||||||||||||||||||||||||||||
Sbjct  171877  AAGCCCGGGGATTTCACATCTGACTTAACAGACCGCCTGCGTGCGCTTTACGCCCAGTAA  171936

Query  907     TTCCGATTAACGCTTGCACCCTCCGTATTACCGCGGCTGCTGGCACGGAGTTAGCCGGTG  966
               ||||||||||||||||||||||||||||||||||||||||||||||||||||||||||||
Sbjct  171937  TTCCGATTAACGCTTGCACCCTCCGTATTACCGCGGCTGCTGGCACGGAGTTAGCCGGTG  171996

Query  967     CTTCTTCTGCGGGTAACGTCAATCG  991
               |||||||||||||||||||||||||
Sbjct  171997  CTTCTTCTGCGGGTAACGTCAATCG  172021
```

Range 3: 447691 to 448675GenBankGraphics

Next Match
Previous Match
First Match

Alignment statistics for match #3

| Score | Expect | Identities | Gaps | Strand | Frame |
| --- | --- | --- | --- | --- | --- |
| 1797 bits(973) | 0.0() | 979/985(99%) | 0/985(0%) | Plus/Minus |  |

Features:

```
Query  7       GCCCTCCCGAAGGTTANGCTACCTACTTCTTTTNNANCCCACTCCCATGGTGTGACGGGC  66
               |||||||||||||||| ||||||||||||||||  | |||||||||||||||||||||||
Sbjct  448675  GCCCTCCCGAAGGTTAAGCTACCTACTTCTTTTGCAACCCACTCCCATGGTGTGACGGGC  448616

Query  67      GGTGTGTACAAGGCCCGGGAACGTATTCACCGTGGCATTCTGATCCACGATTACTAGCGA  126
               ||||||||||||||||||||||||||||||||||||||||||||||||||||||||||||
Sbjct  448615  GGTGTGTACAAGGCCCGGGAACGTATTCACCGTGGCATTCTGATCCACGATTACTAGCGA  448556

Query  127     TTCCGACTTCACGGAGTCGAGTTGCAGACTCCGATCCGGACTACGACGCACTTTGTGAGG  186
               ||||||||||||||||||||||||||||||||||||||||||||||||||||||||||||
Sbjct  448555  TTCCGACTTCACGGAGTCGAGTTGCAGACTCCGATCCGGACTACGACGCACTTTGTGAGG  448496

Query  187     TCCGCTTGCTCTCGCGAGGTCGCTTCTCTTTGTATGCGCCATTGTAGCACGTGTGTAGCC  246
               ||||||||||||||||||||||||||||||||||||||||||||||||||||||||||||
Sbjct  448495  TCCGCTTGCTCTCGCGAGGTCGCTTCTCTTTGTATGCGCCATTGTAGCACGTGTGTAGCC  448436

Query  247     CTACTCGTAAGGGCCATGATGACTTGACGTCATCCCCACCTTCCTCCGGTTTATCACCGG  306
               ||||||||||||||||||||||||||||||||||||||||||||||||||||||||||||
Sbjct  448435  CTACTCGTAAGGGCCATGATGACTTGACGTCATCCCCACCTTCCTCCGGTTTATCACCGG  448376

Query  307     CAGTCTCCTTTGAGTTCCCGACCGAATCGCTGGCAACAAAGGATAAGGGTTGCGCTCGTT  366
               ||||||||||||||||||||||||||||||||||||||||||||||||||||||||||||
Sbjct  448375  CAGTCTCCTTTGAGTTCCCGACCGAATCGCTGGCAACAAAGGATAAGGGTTGCGCTCGTT  448316

Query  367     GCGGGACTTAACCCAACATTTCACAACACGAGCTGACGACAGCCATGCAGCACCTGTCTC  426
               ||||||||||||||||||||||||||||||||||||||||||||||||||||||||||||
Sbjct  448315  GCGGGACTTAACCCAACATTTCACAACACGAGCTGACGACAGCCATGCAGCACCTGTCTC  448256

Query  427     ACGGTTCCCGAAGGCACTAAGGCATCTCTGNNAAATTCCGTGGATGTCAAGAGTAGGTAA  486
               ||||||||||||||||||||||||||||||  ||||||||||||||||||||||||||||
Sbjct  448255  ACGGTTCCCGAAGGCACTAAGGCATCTCTGCCAAATTCCGTGGATGTCAAGAGTAGGTAA  448196

Query  487     GGTTCTTCGCGTTGCATCGAATTAAACCACATGCTCCACCGCTTGTGCGGGCCCCCGTCA  546
               ||||||||||||||||||||||||||||||||||||||||||||||||||||||||||||
Sbjct  448195  GGTTCTTCGCGTTGCATCGAATTAAACCACATGCTCCACCGCTTGTGCGGGCCCCCGTCA  448136

Query  547     ATTCATTTGAGTTTTAACCTTGCGGCCGTACTCCCCAGGCGGTCGACTTAACGCGTTAGC  606
               ||||||||||||||||||||||||||||||||||||||||||||||||||||||||||||
Sbjct  448135  ATTCATTTGAGTTTTAACCTTGCGGCCGTACTCCCCAGGCGGTCGACTTAACGCGTTAGC  448076

Query  607     TCCGGAAGCCACTCCTCAAGGGAACAACCTCCAAGTCGACATCGTTTACGGCGTGGACTA  666
               ||||||||||||||||||||||||||||||||||||||||||||||||||||||||||||
Sbjct  448075  TCCGGAAGCCACTCCTCAAGGGAACAACCTCCAAGTCGACATCGTTTACGGCGTGGACTA  448016

Query  667     CCAGGGTATCTAATCCTGTTTGCTCCCCACGCTTTCGCACCTGAGCGTCAGTCTTTGTCC  726
               ||||||||||||||||||||||||||||||||||||||||||||||||||||||||||||
Sbjct  448015  CCAGGGTATCTAATCCTGTTTGCTCCCCACGCTTTCGCACCTGAGCGTCAGTCTTTGTCC  447956

Query  727     AGGGGGCCGCCTTCGCCACCGGTATTCCTCCAGATCTCTACGCATTTCACCGCTACACCT  786
               ||||||||||||||||||||||||||||||||||||||||||||||||||||||||||||
Sbjct  447955  AGGGGGCCGCCTTCGCCACCGGTATTCCTCCAGATCTCTACGCATTTCACCGCTACACCT  447896

Query  787     GGAATTCTACCCCCCTCTACAAGACTCAAGCCTGCCAGTTTCAAATGCAGTTCCCAGGTT  846
               ||||||||||||||||||||||||||||||||||||||||||||||||||||||||||||
Sbjct  447895  GGAATTCTACCCCCCTCTACAAGACTCAAGCCTGCCAGTTTCAAATGCAGTTCCCAGGTT  447836

Query  847     AAGCCCGGGGATTTCACATCTGACTTAACAGACCGCCTGCGTGCGCTTTACGCCCAGTAA  906
               ||||||||||||||||||||||||||||||||||||||||||||||||||||||||||||
Sbjct  447835  AAGCCCGGGGATTTCACATCTGACTTAACAGACCGCCTGCGTGCGCTTTACGCCCAGTAA  447776

Query  907     TTCCGATTAACGCTTGCACCCTCCGTATTACCGCGGCTGCTGGCACGGAGTTAGCCGGTG  966
               ||||||||||||||||||||||||||||||||||||||||||||||||||||||||||||
Sbjct  447775  TTCCGATTAACGCTTGCACCCTCCGTATTACCGCGGCTGCTGGCACGGAGTTAGCCGGTG  447716

Query  967     CTTCTTCTGCGGGTAACGTCAATCG  991
               |||||||||||||||||||||||||
Sbjct  447715  CTTCTTCTGCGGGTAACGTCAATCG  447691
```

Range 4: 1038308 to 1039292GenBankGraphics

Next Match
Previous Match
First Match

Alignment statistics for match #4

| Score | Expect | Identities | Gaps | Strand | Frame |
| --- | --- | --- | --- | --- | --- |
| 1797 bits(973) | 0.0() | 979/985(99%) | 0/985(0%) | Plus/Minus |  |

Features:

```
Query  7        GCCCTCCCGAAGGTTANGCTACCTACTTCTTTTNNANCCCACTCCCATGGTGTGACGGGC  66
                |||||||||||||||| ||||||||||||||||  | |||||||||||||||||||||||
Sbjct  1039292  GCCCTCCCGAAGGTTAAGCTACCTACTTCTTTTGCAACCCACTCCCATGGTGTGACGGGC  1039233

Query  67       GGTGTGTACAAGGCCCGGGAACGTATTCACCGTGGCATTCTGATCCACGATTACTAGCGA  126
                ||||||||||||||||||||||||||||||||||||||||||||||||||||||||||||
Sbjct  1039232  GGTGTGTACAAGGCCCGGGAACGTATTCACCGTGGCATTCTGATCCACGATTACTAGCGA  1039173

Query  127      TTCCGACTTCACGGAGTCGAGTTGCAGACTCCGATCCGGACTACGACGCACTTTGTGAGG  186
                ||||||||||||||||||||||||||||||||||||||||||||||||||||||||||||
Sbjct  1039172  TTCCGACTTCACGGAGTCGAGTTGCAGACTCCGATCCGGACTACGACGCACTTTGTGAGG  1039113

Query  187      TCCGCTTGCTCTCGCGAGGTCGCTTCTCTTTGTATGCGCCATTGTAGCACGTGTGTAGCC  246
                ||||||||||||||||||||||||||||||||||||||||||||||||||||||||||||
Sbjct  1039112  TCCGCTTGCTCTCGCGAGGTCGCTTCTCTTTGTATGCGCCATTGTAGCACGTGTGTAGCC  1039053

Query  247      CTACTCGTAAGGGCCATGATGACTTGACGTCATCCCCACCTTCCTCCGGTTTATCACCGG  306
                ||||||||||||||||||||||||||||||||||||||||||||||||||||||||||||
Sbjct  1039052  CTACTCGTAAGGGCCATGATGACTTGACGTCATCCCCACCTTCCTCCGGTTTATCACCGG  1038993

Query  307      CAGTCTCCTTTGAGTTCCCGACCGAATCGCTGGCAACAAAGGATAAGGGTTGCGCTCGTT  366
                ||||||||||||||||||||||||||||||||||||||||||||||||||||||||||||
Sbjct  1038992  CAGTCTCCTTTGAGTTCCCGACCGAATCGCTGGCAACAAAGGATAAGGGTTGCGCTCGTT  1038933

Query  367      GCGGGACTTAACCCAACATTTCACAACACGAGCTGACGACAGCCATGCAGCACCTGTCTC  426
                ||||||||||||||||||||||||||||||||||||||||||||||||||||||||||||
Sbjct  1038932  GCGGGACTTAACCCAACATTTCACAACACGAGCTGACGACAGCCATGCAGCACCTGTCTC  1038873

Query  427      ACGGTTCCCGAAGGCACTAAGGCATCTCTGNNAAATTCCGTGGATGTCAAGAGTAGGTAA  486
                ||||||||||||||||||||||||||||||  ||||||||||||||||||||||||||||
Sbjct  1038872  ACGGTTCCCGAAGGCACTAAGGCATCTCTGCCAAATTCCGTGGATGTCAAGAGTAGGTAA  1038813

Query  487      GGTTCTTCGCGTTGCATCGAATTAAACCACATGCTCCACCGCTTGTGCGGGCCCCCGTCA  546
                ||||||||||||||||||||||||||||||||||||||||||||||||||||||||||||
Sbjct  1038812  GGTTCTTCGCGTTGCATCGAATTAAACCACATGCTCCACCGCTTGTGCGGGCCCCCGTCA  1038753

Query  547      ATTCATTTGAGTTTTAACCTTGCGGCCGTACTCCCCAGGCGGTCGACTTAACGCGTTAGC  606
                ||||||||||||||||||||||||||||||||||||||||||||||||||||||||||||
Sbjct  1038752  ATTCATTTGAGTTTTAACCTTGCGGCCGTACTCCCCAGGCGGTCGACTTAACGCGTTAGC  1038693

Query  607      TCCGGAAGCCACTCCTCAAGGGAACAACCTCCAAGTCGACATCGTTTACGGCGTGGACTA  666
                ||||||||||||||||||||||||||||||||||||||||||||||||||||||||||||
Sbjct  1038692  TCCGGAAGCCACTCCTCAAGGGAACAACCTCCAAGTCGACATCGTTTACGGCGTGGACTA  1038633

Query  667      CCAGGGTATCTAATCCTGTTTGCTCCCCACGCTTTCGCACCTGAGCGTCAGTCTTTGTCC  726
                ||||||||||||||||||||||||||||||||||||||||||||||||||||||||||||
Sbjct  1038632  CCAGGGTATCTAATCCTGTTTGCTCCCCACGCTTTCGCACCTGAGCGTCAGTCTTTGTCC  1038573

Query  727      AGGGGGCCGCCTTCGCCACCGGTATTCCTCCAGATCTCTACGCATTTCACCGCTACACCT  786
                ||||||||||||||||||||||||||||||||||||||||||||||||||||||||||||
Sbjct  1038572  AGGGGGCCGCCTTCGCCACCGGTATTCCTCCAGATCTCTACGCATTTCACCGCTACACCT  1038513

Query  787      GGAATTCTACCCCCCTCTACAAGACTCAAGCCTGCCAGTTTCAAATGCAGTTCCCAGGTT  846
                ||||||||||||||||||||||||||||||||||||||||||||||||||||||||||||
Sbjct  1038512  GGAATTCTACCCCCCTCTACAAGACTCAAGCCTGCCAGTTTCAAATGCAGTTCCCAGGTT  1038453

Query  847      AAGCCCGGGGATTTCACATCTGACTTAACAGACCGCCTGCGTGCGCTTTACGCCCAGTAA  906
                ||||||||||||||||||||||||||||||||||||||||||||||||||||||||||||
Sbjct  1038452  AAGCCCGGGGATTTCACATCTGACTTAACAGACCGCCTGCGTGCGCTTTACGCCCAGTAA  1038393

Query  907      TTCCGATTAACGCTTGCACCCTCCGTATTACCGCGGCTGCTGGCACGGAGTTAGCCGGTG  966
                ||||||||||||||||||||||||||||||||||||||||||||||||||||||||||||
Sbjct  1038392  TTCCGATTAACGCTTGCACCCTCCGTATTACCGCGGCTGCTGGCACGGAGTTAGCCGGTG  1038333

Query  967      CTTCTTCTGCGGGTAACGTCAATCG  991
                |||||||||||||||||||||||||
Sbjct  1038332  CTTCTTCTGCGGGTAACGTCAATCG  1038308
```

Range 5: 410982 to 411966GenBankGraphics

Next Match
Previous Match
First Match

Alignment statistics for match #5

| Score | Expect | Identities | Gaps | Strand | Frame |
| --- | --- | --- | --- | --- | --- |
| 1792 bits(970) | 0.0() | 978/985(99%) | 0/985(0%) | Plus/Minus |  |

Features:

```
Query  7       GCCCTCCCGAAGGTTANGCTACCTACTTCTTTTNNANCCCACTCCCATGGTGTGACGGGC  66
               |||||||||||||||| ||||||||||||||||  | |||||||||||||||||||||||
Sbjct  411966  GCCCTCCCGAAGGTTAAGCTACCTACTTCTTTTGCAACCCACTCCCATGGTGTGACGGGC  411907

Query  67      GGTGTGTACAAGGCCCGGGAACGTATTCACCGTGGCATTCTGATCCACGATTACTAGCGA  126
               ||||||||||||||||||||||||||||||||||||||||||||||||||||||||||||
Sbjct  411906  GGTGTGTACAAGGCCCGGGAACGTATTCACCGTGGCATTCTGATCCACGATTACTAGCGA  411847

Query  127     TTCCGACTTCACGGAGTCGAGTTGCAGACTCCGATCCGGACTACGACGCACTTTGTGAGG  186
               |||||||||||||||||||||||||||||||||||||||||||||| |||||||||||||
Sbjct  411846  TTCCGACTTCACGGAGTCGAGTTGCAGACTCCGATCCGGACTACGATGCACTTTGTGAGG  411787

Query  187     TCCGCTTGCTCTCGCGAGGTCGCTTCTCTTTGTATGCGCCATTGTAGCACGTGTGTAGCC  246
               ||||||||||||||||||||||||||||||||||||||||||||||||||||||||||||
Sbjct  411786  TCCGCTTGCTCTCGCGAGGTCGCTTCTCTTTGTATGCGCCATTGTAGCACGTGTGTAGCC  411727

Query  247     CTACTCGTAAGGGCCATGATGACTTGACGTCATCCCCACCTTCCTCCGGTTTATCACCGG  306
               ||||||||||||||||||||||||||||||||||||||||||||||||||||||||||||
Sbjct  411726  CTACTCGTAAGGGCCATGATGACTTGACGTCATCCCCACCTTCCTCCGGTTTATCACCGG  411667

Query  307     CAGTCTCCTTTGAGTTCCCGACCGAATCGCTGGCAACAAAGGATAAGGGTTGCGCTCGTT  366
               ||||||||||||||||||||||||||||||||||||||||||||||||||||||||||||
Sbjct  411666  CAGTCTCCTTTGAGTTCCCGACCGAATCGCTGGCAACAAAGGATAAGGGTTGCGCTCGTT  411607

Query  367     GCGGGACTTAACCCAACATTTCACAACACGAGCTGACGACAGCCATGCAGCACCTGTCTC  426
               ||||||||||||||||||||||||||||||||||||||||||||||||||||||||||||
Sbjct  411606  GCGGGACTTAACCCAACATTTCACAACACGAGCTGACGACAGCCATGCAGCACCTGTCTC  411547

Query  427     ACGGTTCCCGAAGGCACTAAGGCATCTCTGNNAAATTCCGTGGATGTCAAGAGTAGGTAA  486
               ||||||||||||||||||||||||||||||  ||||||||||||||||||||||||||||
Sbjct  411546  ACGGTTCCCGAAGGCACTAAGGCATCTCTGCCAAATTCCGTGGATGTCAAGAGTAGGTAA  411487

Query  487     GGTTCTTCGCGTTGCATCGAATTAAACCACATGCTCCACCGCTTGTGCGGGCCCCCGTCA  546
               ||||||||||||||||||||||||||||||||||||||||||||||||||||||||||||
Sbjct  411486  GGTTCTTCGCGTTGCATCGAATTAAACCACATGCTCCACCGCTTGTGCGGGCCCCCGTCA  411427

Query  547     ATTCATTTGAGTTTTAACCTTGCGGCCGTACTCCCCAGGCGGTCGACTTAACGCGTTAGC  606
               ||||||||||||||||||||||||||||||||||||||||||||||||||||||||||||
Sbjct  411426  ATTCATTTGAGTTTTAACCTTGCGGCCGTACTCCCCAGGCGGTCGACTTAACGCGTTAGC  411367

Query  607     TCCGGAAGCCACTCCTCAAGGGAACAACCTCCAAGTCGACATCGTTTACGGCGTGGACTA  666
               ||||||||||||||||||||||||||||||||||||||||||||||||||||||||||||
Sbjct  411366  TCCGGAAGCCACTCCTCAAGGGAACAACCTCCAAGTCGACATCGTTTACGGCGTGGACTA  411307

Query  667     CCAGGGTATCTAATCCTGTTTGCTCCCCACGCTTTCGCACCTGAGCGTCAGTCTTTGTCC  726
               ||||||||||||||||||||||||||||||||||||||||||||||||||||||||||||
Sbjct  411306  CCAGGGTATCTAATCCTGTTTGCTCCCCACGCTTTCGCACCTGAGCGTCAGTCTTTGTCC  411247

Query  727     AGGGGGCCGCCTTCGCCACCGGTATTCCTCCAGATCTCTACGCATTTCACCGCTACACCT  786
               ||||||||||||||||||||||||||||||||||||||||||||||||||||||||||||
Sbjct  411246  AGGGGGCCGCCTTCGCCACCGGTATTCCTCCAGATCTCTACGCATTTCACCGCTACACCT  411187

Query  787     GGAATTCTACCCCCCTCTACAAGACTCAAGCCTGCCAGTTTCAAATGCAGTTCCCAGGTT  846
               ||||||||||||||||||||||||||||||||||||||||||||||||||||||||||||
Sbjct  411186  GGAATTCTACCCCCCTCTACAAGACTCAAGCCTGCCAGTTTCAAATGCAGTTCCCAGGTT  411127

Query  847     AAGCCCGGGGATTTCACATCTGACTTAACAGACCGCCTGCGTGCGCTTTACGCCCAGTAA  906
               ||||||||||||||||||||||||||||||||||||||||||||||||||||||||||||
Sbjct  411126  AAGCCCGGGGATTTCACATCTGACTTAACAGACCGCCTGCGTGCGCTTTACGCCCAGTAA  411067

Query  907     TTCCGATTAACGCTTGCACCCTCCGTATTACCGCGGCTGCTGGCACGGAGTTAGCCGGTG  966
               ||||||||||||||||||||||||||||||||||||||||||||||||||||||||||||
Sbjct  411066  TTCCGATTAACGCTTGCACCCTCCGTATTACCGCGGCTGCTGGCACGGAGTTAGCCGGTG  411007

Query  967     CTTCTTCTGCGGGTAACGTCAATCG  991
               |||||||||||||||||||||||||
Sbjct  411006  CTTCTTCTGCGGGTAACGTCAATCG  410982
```

Range 6: 3350389 to 3351373GenBankGraphics

Next Match
Previous Match
First Match

Alignment statistics for match #6

| Score | Expect | Identities | Gaps | Strand | Frame |
| --- | --- | --- | --- | --- | --- |
| 1792 bits(970) | 0.0() | 978/985(99%) | 0/985(0%) | Plus/Plus |  |

Features:

```
Query  7        GCCCTCCCGAAGGTTANGCTACCTACTTCTTTTNNANCCCACTCCCATGGTGTGACGGGC  66
                |||||||||||||||| ||||||||||||||||  | |||||||||||||||||||||||
Sbjct  3350389  GCCCTCCCGAAGGTTAAGCTACCTACTTCTTTTGCAACCCACTCCCATGGTGTGACGGGC  3350448

Query  67       GGTGTGTACAAGGCCCGGGAACGTATTCACCGTGGCATTCTGATCCACGATTACTAGCGA  126
                ||||||||||||||||||||||||||||||||||||||||||||||||||||||||||||
Sbjct  3350449  GGTGTGTACAAGGCCCGGGAACGTATTCACCGTGGCATTCTGATCCACGATTACTAGCGA  3350508

Query  127      TTCCGACTTCACGGAGTCGAGTTGCAGACTCCGATCCGGACTACGACGCACTTTGTGAGG  186
                ||||||||||||||||||||||||||||||||||||||||||||||||||||||||||||
Sbjct  3350509  TTCCGACTTCACGGAGTCGAGTTGCAGACTCCGATCCGGACTACGACGCACTTTGTGAGG  3350568

Query  187      TCCGCTTGCTCTCGCGAGGTCGCTTCTCTTTGTATGCGCCATTGTAGCACGTGTGTAGCC  246
                ||||||||||||||||||||||||||||||||||||||||||||||||||||||||||||
Sbjct  3350569  TCCGCTTGCTCTCGCGAGGTCGCTTCTCTTTGTATGCGCCATTGTAGCACGTGTGTAGCC  3350628

Query  247      CTACTCGTAAGGGCCATGATGACTTGACGTCATCCCCACCTTCCTCCGGTTTATCACCGG  306
                ||||||||||||||||||||||||||||||||||||||||||||||||||||||||||||
Sbjct  3350629  CTACTCGTAAGGGCCATGATGACTTGACGTCATCCCCACCTTCCTCCGGTTTATCACCGG  3350688

Query  307      CAGTCTCCTTTGAGTTCCCGACCGAATCGCTGGCAACAAAGGATAAGGGTTGCGCTCGTT  366
                ||||||||||||||||||||||||||||||||||||||||||||||||||||||||||||
Sbjct  3350689  CAGTCTCCTTTGAGTTCCCGACCGAATCGCTGGCAACAAAGGATAAGGGTTGCGCTCGTT  3350748

Query  367      GCGGGACTTAACCCAACATTTCACAACACGAGCTGACGACAGCCATGCAGCACCTGTCTC  426
                ||||||||||||||||||||||||||||||||||||||||||||||||||||||||||||
Sbjct  3350749  GCGGGACTTAACCCAACATTTCACAACACGAGCTGACGACAGCCATGCAGCACCTGTCTC  3350808

Query  427      ACGGTTCCCGAAGGCACTAAGGCATCTCTGNNAAATTCCGTGGATGTCAAGAGTAGGTAA  486
                ||||||||||||||||||||||||||||||   |||||||||||||||||||||||||||
Sbjct  3350809  ACGGTTCCCGAAGGCACTAAGGCATCTCTGCCGAATTCCGTGGATGTCAAGAGTAGGTAA  3350868

Query  487      GGTTCTTCGCGTTGCATCGAATTAAACCACATGCTCCACCGCTTGTGCGGGCCCCCGTCA  546
                ||||||||||||||||||||||||||||||||||||||||||||||||||||||||||||
Sbjct  3350869  GGTTCTTCGCGTTGCATCGAATTAAACCACATGCTCCACCGCTTGTGCGGGCCCCCGTCA  3350928

Query  547      ATTCATTTGAGTTTTAACCTTGCGGCCGTACTCCCCAGGCGGTCGACTTAACGCGTTAGC  606
                ||||||||||||||||||||||||||||||||||||||||||||||||||||||||||||
Sbjct  3350929  ATTCATTTGAGTTTTAACCTTGCGGCCGTACTCCCCAGGCGGTCGACTTAACGCGTTAGC  3350988

Query  607      TCCGGAAGCCACTCCTCAAGGGAACAACCTCCAAGTCGACATCGTTTACGGCGTGGACTA  666
                ||||||||||||||||||||||||||||||||||||||||||||||||||||||||||||
Sbjct  3350989  TCCGGAAGCCACTCCTCAAGGGAACAACCTCCAAGTCGACATCGTTTACGGCGTGGACTA  3351048

Query  667      CCAGGGTATCTAATCCTGTTTGCTCCCCACGCTTTCGCACCTGAGCGTCAGTCTTTGTCC  726
                ||||||||||||||||||||||||||||||||||||||||||||||||||||||||||||
Sbjct  3351049  CCAGGGTATCTAATCCTGTTTGCTCCCCACGCTTTCGCACCTGAGCGTCAGTCTTTGTCC  3351108

Query  727      AGGGGGCCGCCTTCGCCACCGGTATTCCTCCAGATCTCTACGCATTTCACCGCTACACCT  786
                ||||||||||||||||||||||||||||||||||||||||||||||||||||||||||||
Sbjct  3351109  AGGGGGCCGCCTTCGCCACCGGTATTCCTCCAGATCTCTACGCATTTCACCGCTACACCT  3351168

Query  787      GGAATTCTACCCCCCTCTACAAGACTCAAGCCTGCCAGTTTCAAATGCAGTTCCCAGGTT  846
                ||||||||||||||||||||||||||||||||||||||||||||||||||||||||||||
Sbjct  3351169  GGAATTCTACCCCCCTCTACAAGACTCAAGCCTGCCAGTTTCAAATGCAGTTCCCAGGTT  3351228

Query  847      AAGCCCGGGGATTTCACATCTGACTTAACAGACCGCCTGCGTGCGCTTTACGCCCAGTAA  906
                ||||||||||||||||||||||||||||||||||||||||||||||||||||||||||||
Sbjct  3351229  AAGCCCGGGGATTTCACATCTGACTTAACAGACCGCCTGCGTGCGCTTTACGCCCAGTAA  3351288

Query  907      TTCCGATTAACGCTTGCACCCTCCGTATTACCGCGGCTGCTGGCACGGAGTTAGCCGGTG  966
                ||||||||||||||||||||||||||||||||||||||||||||||||||||||||||||
Sbjct  3351289  TTCCGATTAACGCTTGCACCCTCCGTATTACCGCGGCTGCTGGCACGGAGTTAGCCGGTG  3351348

Query  967      CTTCTTCTGCGGGTAACGTCAATCG  991
                |||||||||||||||||||||||||
Sbjct  3351349  CTTCTTCTGCGGGTAACGTCAATCG  3351373
```

Range 7: 3833146 to 3834130GenBankGraphics

Next Match
Previous Match
First Match

Alignment statistics for match #7

| Score | Expect | Identities | Gaps | Strand | Frame |
| --- | --- | --- | --- | --- | --- |
| 1792 bits(970) | 0.0() | 978/985(99%) | 0/985(0%) | Plus/Plus |  |

Features:

```
Query  7        GCCCTCCCGAAGGTTANGCTACCTACTTCTTTTNNANCCCACTCCCATGGTGTGACGGGC  66
                |||||||||||||||| ||||||||||||||||  | |||||||||||||||||||||||
Sbjct  3833146  GCCCTCCCGAAGGTTAAGCTACCTACTTCTTTTGCAACCCACTCCCATGGTGTGACGGGC  3833205

Query  67       GGTGTGTACAAGGCCCGGGAACGTATTCACCGTGGCATTCTGATCCACGATTACTAGCGA  126
                ||||||||||||||||||||||||||||||||||||||||||||||||||||||||||||
Sbjct  3833206  GGTGTGTACAAGGCCCGGGAACGTATTCACCGTGGCATTCTGATCCACGATTACTAGCGA  3833265

Query  127      TTCCGACTTCACGGAGTCGAGTTGCAGACTCCGATCCGGACTACGACGCACTTTGTGAGG  186
                ||||||||||||||||||||||||||||||||||||||||||||||||||||||||||||
Sbjct  3833266  TTCCGACTTCACGGAGTCGAGTTGCAGACTCCGATCCGGACTACGACGCACTTTGTGAGG  3833325

Query  187      TCCGCTTGCTCTCGCGAGGTCGCTTCTCTTTGTATGCGCCATTGTAGCACGTGTGTAGCC  246
                ||||||||||||||||||||||||||||||||||||||||||||||||||||||||||||
Sbjct  3833326  TCCGCTTGCTCTCGCGAGGTCGCTTCTCTTTGTATGCGCCATTGTAGCACGTGTGTAGCC  3833385

Query  247      CTACTCGTAAGGGCCATGATGACTTGACGTCATCCCCACCTTCCTCCGGTTTATCACCGG  306
                ||||||||||||||||||||||||||||||||||||||||||||||||||||||||||||
Sbjct  3833386  CTACTCGTAAGGGCCATGATGACTTGACGTCATCCCCACCTTCCTCCGGTTTATCACCGG  3833445

Query  307      CAGTCTCCTTTGAGTTCCCGACCGAATCGCTGGCAACAAAGGATAAGGGTTGCGCTCGTT  366
                ||||||||||||||||||||||||||||||||||||||||||||||||||||||||||||
Sbjct  3833446  CAGTCTCCTTTGAGTTCCCGACCGAATCGCTGGCAACAAAGGATAAGGGTTGCGCTCGTT  3833505

Query  367      GCGGGACTTAACCCAACATTTCACAACACGAGCTGACGACAGCCATGCAGCACCTGTCTC  426
                ||||||||||||||||||||||||||||||||||||||||||||||||||||||||||||
Sbjct  3833506  GCGGGACTTAACCCAACATTTCACAACACGAGCTGACGACAGCCATGCAGCACCTGTCTC  3833565

Query  427      ACGGTTCCCGAAGGCACTAAGGCATCTCTGNNAAATTCCGTGGATGTCAAGAGTAGGTAA  486
                ||||||||||||||||||||||||||||||   |||||||||||||||||||||||||||
Sbjct  3833566  ACGGTTCCCGAAGGCACTAAGGCATCTCTGCCGAATTCCGTGGATGTCAAGAGTAGGTAA  3833625

Query  487      GGTTCTTCGCGTTGCATCGAATTAAACCACATGCTCCACCGCTTGTGCGGGCCCCCGTCA  546
                ||||||||||||||||||||||||||||||||||||||||||||||||||||||||||||
Sbjct  3833626  GGTTCTTCGCGTTGCATCGAATTAAACCACATGCTCCACCGCTTGTGCGGGCCCCCGTCA  3833685

Query  547      ATTCATTTGAGTTTTAACCTTGCGGCCGTACTCCCCAGGCGGTCGACTTAACGCGTTAGC  606
                ||||||||||||||||||||||||||||||||||||||||||||||||||||||||||||
Sbjct  3833686  ATTCATTTGAGTTTTAACCTTGCGGCCGTACTCCCCAGGCGGTCGACTTAACGCGTTAGC  3833745

Query  607      TCCGGAAGCCACTCCTCAAGGGAACAACCTCCAAGTCGACATCGTTTACGGCGTGGACTA  666
                ||||||||||||||||||||||||||||||||||||||||||||||||||||||||||||
Sbjct  3833746  TCCGGAAGCCACTCCTCAAGGGAACAACCTCCAAGTCGACATCGTTTACGGCGTGGACTA  3833805

Query  667      CCAGGGTATCTAATCCTGTTTGCTCCCCACGCTTTCGCACCTGAGCGTCAGTCTTTGTCC  726
                ||||||||||||||||||||||||||||||||||||||||||||||||||||||||||||
Sbjct  3833806  CCAGGGTATCTAATCCTGTTTGCTCCCCACGCTTTCGCACCTGAGCGTCAGTCTTTGTCC  3833865

Query  727      AGGGGGCCGCCTTCGCCACCGGTATTCCTCCAGATCTCTACGCATTTCACCGCTACACCT  786
                ||||||||||||||||||||||||||||||||||||||||||||||||||||||||||||
Sbjct  3833866  AGGGGGCCGCCTTCGCCACCGGTATTCCTCCAGATCTCTACGCATTTCACCGCTACACCT  3833925

Query  787      GGAATTCTACCCCCCTCTACAAGACTCAAGCCTGCCAGTTTCAAATGCAGTTCCCAGGTT  846
                ||||||||||||||||||||||||||||||||||||||||||||||||||||||||||||
Sbjct  3833926  GGAATTCTACCCCCCTCTACAAGACTCAAGCCTGCCAGTTTCAAATGCAGTTCCCAGGTT  3833985

Query  847      AAGCCCGGGGATTTCACATCTGACTTAACAGACCGCCTGCGTGCGCTTTACGCCCAGTAA  906
                ||||||||||||||||||||||||||||||||||||||||||||||||||||||||||||
Sbjct  3833986  AAGCCCGGGGATTTCACATCTGACTTAACAGACCGCCTGCGTGCGCTTTACGCCCAGTAA  3834045

Query  907      TTCCGATTAACGCTTGCACCCTCCGTATTACCGCGGCTGCTGGCACGGAGTTAGCCGGTG  966
                ||||||||||||||||||||||||||||||||||||||||||||||||||||||||||||
Sbjct  3834046  TTCCGATTAACGCTTGCACCCTCCGTATTACCGCGGCTGCTGGCACGGAGTTAGCCGGTG  3834105

Query  967      CTTCTTCTGCGGGTAACGTCAATCG  991
                |||||||||||||||||||||||||
Sbjct  3834106  CTTCTTCTGCGGGTAACGTCAATCG  3834130
```

Download

FASTA (complete sequence)

FASTA (aligned sequences)

GenBank (complete sequence)

Continue
Cancel

GenBankGraphics

Next
Previous
Descriptions

Pantoea agglomerans strain AL14 16S ribosomal RNA gene, partial sequence

Sequence ID: MG819218.1Length: 1416Number of Matches: 1

Related Information

Range 1: 386 to 1371GenBankGraphics

Next Match
Previous Match
First Match

Alignment statistics for match #1

| Score | Expect | Identities | Gaps | Strand | Frame |
| --- | --- | --- | --- | --- | --- |
| 1799 bits(974) | 0.0() | 980/986(99%) | 0/986(0%) | Plus/Minus |  |

Features:

```
Query  7     GCCCTCCCGAAGGTTANGCTACCTACTTCTTTTNNANCCCACTCCCATGGTGTGACGGGC  66
             |||||||||||||||| ||||||||||||||||  | |||||||||||||||||||||||
Sbjct  1371  GCCCTCCCGAAGGTTAAGCTACCTACTTCTTTTGCAACCCACTCCCATGGTGTGACGGGC  1312

Query  67    GGTGTGTACAAGGCCCGGGAACGTATTCACCGTGGCATTCTGATCCACGATTACTAGCGA  126
             ||||||||||||||||||||||||||||||||||||||||||||||||||||||||||||
Sbjct  1311  GGTGTGTACAAGGCCCGGGAACGTATTCACCGTGGCATTCTGATCCACGATTACTAGCGA  1252

Query  127   TTCCGACTTCACGGAGTCGAGTTGCAGACTCCGATCCGGACTACGACGCACTTTGTGAGG  186
             ||||||||||||||||||||||||||||||||||||||||||||||||||||||||||||
Sbjct  1251  TTCCGACTTCACGGAGTCGAGTTGCAGACTCCGATCCGGACTACGACGCACTTTGTGAGG  1192

Query  187   TCCGCTTGCTCTCGCGAGGTCGCTTCTCTTTGTATGCGCCATTGTAGCACGTGTGTAGCC  246
             ||||||||||||||||||||||||||||||||||||||||||||||||||||||||||||
Sbjct  1191  TCCGCTTGCTCTCGCGAGGTCGCTTCTCTTTGTATGCGCCATTGTAGCACGTGTGTAGCC  1132

Query  247   CTACTCGTAAGGGCCATGATGACTTGACGTCATCCCCACCTTCCTCCGGTTTATCACCGG  306
             ||||||||||||||||||||||||||||||||||||||||||||||||||||||||||||
Sbjct  1131  CTACTCGTAAGGGCCATGATGACTTGACGTCATCCCCACCTTCCTCCGGTTTATCACCGG  1072

Query  307   CAGTCTCCTTTGAGTTCCCGACCGAATCGCTGGCAACAAAGGATAAGGGTTGCGCTCGTT  366
             ||||||||||||||||||||||||||||||||||||||||||||||||||||||||||||
Sbjct  1071  CAGTCTCCTTTGAGTTCCCGACCGAATCGCTGGCAACAAAGGATAAGGGTTGCGCTCGTT  1012

Query  367   GCGGGACTTAACCCAACATTTCACAACACGAGCTGACGACAGCCATGCAGCACCTGTCTC  426
             ||||||||||||||||||||||||||||||||||||||||||||||||||||||||||||
Sbjct  1011  GCGGGACTTAACCCAACATTTCACAACACGAGCTGACGACAGCCATGCAGCACCTGTCTC  952

Query  427   ACGGTTCCCGAAGGCACTAAGGCATCTCTGNNAAATTCCGTGGATGTCAAGAGTAGGTAA  486
             ||||||||||||||||||||||||||||||  ||||||||||||||||||||||||||||
Sbjct  951   ACGGTTCCCGAAGGCACTAAGGCATCTCTGCCAAATTCCGTGGATGTCAAGAGTAGGTAA  892

Query  487   GGTTCTTCGCGTTGCATCGAATTAAACCACATGCTCCACCGCTTGTGCGGGCCCCCGTCA  546
             ||||||||||||||||||||||||||||||||||||||||||||||||||||||||||||
Sbjct  891   GGTTCTTCGCGTTGCATCGAATTAAACCACATGCTCCACCGCTTGTGCGGGCCCCCGTCA  832

Query  547   ATTCATTTGAGTTTTAACCTTGCGGCCGTACTCCCCAGGCGGTCGACTTAACGCGTTAGC  606
             ||||||||||||||||||||||||||||||||||||||||||||||||||||||||||||
Sbjct  831   ATTCATTTGAGTTTTAACCTTGCGGCCGTACTCCCCAGGCGGTCGACTTAACGCGTTAGC  772

Query  607   TCCGGAAGCCACTCCTCAAGGGAACAACCTCCAAGTCGACATCGTTTACGGCGTGGACTA  666
             ||||||||||||||||||||||||||||||||||||||||||||||||||||||||||||
Sbjct  771   TCCGGAAGCCACTCCTCAAGGGAACAACCTCCAAGTCGACATCGTTTACGGCGTGGACTA  712

Query  667   CCAGGGTATCTAATCCTGTTTGCTCCCCACGCTTTCGCACCTGAGCGTCAGTCTTTGTCC  726
             ||||||||||||||||||||||||||||||||||||||||||||||||||||||||||||
Sbjct  711   CCAGGGTATCTAATCCTGTTTGCTCCCCACGCTTTCGCACCTGAGCGTCAGTCTTTGTCC  652

Query  727   AGGGGGCCGCCTTCGCCACCGGTATTCCTCCAGATCTCTACGCATTTCACCGCTACACCT  786
             ||||||||||||||||||||||||||||||||||||||||||||||||||||||||||||
Sbjct  651   AGGGGGCCGCCTTCGCCACCGGTATTCCTCCAGATCTCTACGCATTTCACCGCTACACCT  592

Query  787   GGAATTCTACCCCCCTCTACAAGACTCAAGCCTGCCAGTTTCAAATGCAGTTCCCAGGTT  846
             ||||||||||||||||||||||||||||||||||||||||||||||||||||||||||||
Sbjct  591   GGAATTCTACCCCCCTCTACAAGACTCAAGCCTGCCAGTTTCAAATGCAGTTCCCAGGTT  532

Query  847   AAGCCCGGGGATTTCACATCTGACTTAACAGACCGCCTGCGTGCGCTTTACGCCCAGTAA  906
             ||||||||||||||||||||||||||||||||||||||||||||||||||||||||||||
Sbjct  531   AAGCCCGGGGATTTCACATCTGACTTAACAGACCGCCTGCGTGCGCTTTACGCCCAGTAA  472

Query  907   TTCCGATTAACGCTTGCACCCTCCGTATTACCGCGGCTGCTGGCACGGAGTTAGCCGGTG  966
             ||||||||||||||||||||||||||||||||||||||||||||||||||||||||||||
Sbjct  471   TTCCGATTAACGCTTGCACCCTCCGTATTACCGCGGCTGCTGGCACGGAGTTAGCCGGTG  412

Query  967   CTTCTTCTGCGGGTAACGTCAATCGA  992
             ||||||||||||||||||||||||||
Sbjct  411   CTTCTTCTGCGGGTAACGTCAATCGA  386
```


BLAST is a registered trademark of the National Library of Medicine

Support center
Mailing list


YouTube

- National Library Of Medicine
- National Institutes Of Health
- U.S. Department of Health & Human Services
- USA.gov

### NCBI


National Center for Biotechnology Information,
 U.S. National Library of Medicine

8600 Rockville Pike,
Bethesda
 MD,
20894
USA

Policies and Guidelines
|
Contact


PreferencesTurn off

External link. Please review our privacy policy.
