# Supplementary material for: Isothermal microcalorimetry for thermal viable count of microorganisms in pure cultures and stabilized formulations
Source: BMC Microbiol. 2019 Mar 21;19:65. doi: 10.1186/s12866-019-1432-8 (PMC6429831; doi:10.1186/s12866-019-1432-8)
Supplement: Supplementary file 3 — 16S rRNA gene sequences’ BLASTN hits in zipped HTML format. (ZIP 15810 kb) [file 12866_2019_1432_MOESM3_ESM.zip › Best blastn hits/NCBI Blast_J pink F -- 16..1018 of sequence (1003.html]

NCBI Blast:J pink F -- 16..1018 of sequence (1003


- NCBI Home
- Sign in to NCBI
- Skip to Main Content
- Skip to Navigation
- About NCBI Accesskeys

U.S. National Library of Medicine

NCBI
National Center for Biotechnology Information

- My NCBI
- Sign in to NCBI
- Register
- Sign Out

BLAST ^®^ » blastn suite » RID-A4XV6EG2014


- Home
- Recent Results
- Saved Strategies
- Help

BLAST Results


Edit and Resubmit
Save Search Strategies
[Sign in above to save your search strategy]

Formatting options 


Download


How to read this page
Blast report description
Questions/comments


|  |  |
| --- | --- |
| Formatting options | |
| Show | Alignment as  HTML Plain text   Old View Reset form to defaults [?]  These options control formatting of alignments in results pages. The default is HTML, but other formats (including plain text) are available. PSSM and PssmWithParameters are representations of Position Specific Scoring Matrices and are only available for PSI-BLAST. The Advanced view option allows the database descriptions to be sorted by various indices in a table. |
| Alignment View | Pairwise Pairwise with dots for identities Query-anchored with dots for identities Query-anchored with letters for identities Flat query-anchored with dots for identities Flat query-anchored with letters for identities [?]  Choose how to view alignments. The default "pairwise" view shows how each subject sequence aligns individually to the query sequence. The "query-anchored" view shows how all subject sequences align to the query sequence. For each view type, you can choose to show "identities" (matching residues) as letters or dots. more... |
| Display | Graphical Overview   Linkout   Sequence Retrieval  NCBI-gi   CDS feature [?]  - Graphical Overview: Graphical Overview: Show graph of similar sequence regions aligned to query.   more... - NCBI-gi: Show NCBI gi identifiers. - CDS feature: Show annotated coding region and translation.   more... |
| Masking | Character:   X for protein, n for nucleotide Lower Case  Color:  Black Grey Red [?]  - Masking Character: Display masked (filtered) sequence regions as lower-case or as specific letters (N for nucleotide, P for protein). - Masking Color: Display masked sequence regions in the given color. |
| Limit results | Descriptions:  10 50 100 Graphical overview:  0 10 50 100  Alignments:  0 10 50 100 Line length:  60 90 120 150 [?]  - Descriptions: Show short descriptions for up to the given number of sequences. - Alignments: Show alignments for up to the given number of sequences, in order of statistical significance. - Line lenghth: Number of letters to show on one line in an alignment. |
|  | Organism Type common name, binomial, taxid, or group name. Only 20 top taxa will be shown.     Exclude   [?]  Show only sequences from the given organism. |
|  | Entrez query:  [?]  Show only those sequences that match the given Entrez query. more... |
|  | Expect Min:  Expect Max:  [?]  Show only sequences with expect values in the given range. more... |
|  | Percent Identity Min:  Percent Identity Max:  [?]  Show only sequences with percent identity values in the given range. |
| Format for | PSI-BLAST with inclusion threshold:  [?]  - Format for PSI-BLAST: The Position-Specific Iterated BLAST (PSI-BLAST) program performs iterative searches with a protein query,   in which sequences found in one round of search are used to build a custom score model for the next round.   more... - Inclusion Threshold: This sets the statistical significance threshold for including a sequence in the model used   by PSI-BLAST to create the PSSM on the next iteration. |

|  |  |  |  |  |  |
| --- | --- | --- | --- | --- | --- |
| Download | | | | | |
| Alignment  Text XML ASN.1 JSON Seq-align Hit Table(text) Hit Table(csv) Multiple-file XML2 Single-file XML2 Multiple-file JSON Single-file JSON SAM | Search Strategies  ASN.1 | PSSM to restart search  PSSM | [?] |  |  |

The Download link provides BLAST output that may be used as input to another program.
This includes parseable formats such as the tabular report or XML as well as the Search Strategy files read by the BLAST+ applications.
More details on the parseable (XML, tabular, and ASN.1) reports can be found at
https://www.ncbi.nlm.nih.gov/books/NBK153387/  
  

The following formats are offered under the Alignment section:  
1). "Text". Non-HTML standard BLAST report.  
2). "XML". XML report based upon the DTD at https://www.ncbi.nlm.nih.gov/data\_specs/dtd/NCBI\_BlastOutput.dtd  
3). "ASN.1". Alignment written out in Abstract Syntax Notation 1.  
4). "JSON Seq-align". Alignment written out in JSON.  
4). "Hit Table(text)". The tabular report as text.  
5). "Hit Table(csv)". The tabular report ready for import into spread-sheet programs like Excel.  
6). "XML2". New XML format described at ftp://ftp.ncbi.nlm.nih.gov/blast/documents/NEWXML/xml2.pdf.  
7). "JSON". New JSON format described at ftp://ftp.ncbi.nlm.nih.gov/blast/documents/NEWXML/xml2.pdf.  
8). "SAM". Sequence Alignment Map format.

XML2 and JSON can be downloaded either as one file per query (multiple-file) or one file for all queries (single-file). These formats are listed as Multiple-file XML2 (and JSON) or Single-file XML (and JSON).

The following report is offered under the Search Strategy section:  
1). "ASN.1" Search Strategy. A record of the parameters, query, and database used in the search. This file can be used to start a stand-alone BLAST search, see
https://www.ncbi.nlm.nih.gov/books/NBK1763/#CmdLineAppsManual.I455\_BLAST\_search\_stra


# Job title: J pink F -- 16..1018 of sequence (1003

Results for:

lcl|Query\_240781 J pink F -- 16..1018 of sequence(1003bp)
[?]

Your BLAST job specified more than one input sequence.
This box lets you choose which input sequence to show BLAST results for.

RID
:   A4XV6EG2014 (Expires on 03-10 19:00 pm)

Query ID
:   lcl|Query\_240781
:   lcl|Query\_240781

Description
:   J pink F -- 16..1018 of sequence

Molecule type
:   nucleic acid

Query Length
:   1003

Database Name
:   nr

Description
:   Nucleotide collection (nt) See details

Program
:   BLASTN 2.8.0+ Citation

  

Reference 

Zheng Zhang, Scott Schwartz, Lukas Wagner, and Webb Miller (2000), "A greedy algorithm for aligning DNA sequences", J Comput Biol 2000; 7(1-2):203-14.

Reference - database indexing

Aleksandr Morgulis, George Coulouris, Yan Raytselis, Thomas L. Madden, Richa Agarwala, Alejandro A. Schäffer (2008), "Database Indexing for Production MegaBLAST Searches", Bioinformatics 24:1757-1764.

Other reports:
Search Summary

[Taxonomy reports]
[Distance tree of results]
[MSA viewer]

Search Parameters

| Search parameter name | Search parameter value |
| --- | --- |
| Program | blastn |
| Word size | 28 |
| Expect value | 10 |
| Hitlist size | 100 |
| Match/Mismatch scores | 1,-2 |
| Gapcosts | 0,2.5 |
| Low Complexity Filter | Yes |
| Filter string | L;m; |
| Genetic Code | 1 |

Database

| Database parameter name | Database parameter value |
| --- | --- |
| Posted date | Mar 7, 2018 1:58 PM |
| Number of letters | 174,044,644,244 |
| Number of sequences | 46,882,714 |
| Entrez query | Includes:  Excludes:  None |

Karlin-Altschul statistics

| Params | Ungapped | Gapped |
| --- | --- | --- |
| Lambda | 1.33271 | 1.28 |
| K | 0.620991 | 0.46 |
| H | 1.12409 | 0.85 |

Results Statistics

| Results Statistics parameter name | Results Statistics parameter value |
| --- | --- |
| Length adjustment | 35 |
| Effective length of query | 968 |
| Effective length of database | 172403749254 |
| Effective search space | 166886829277872 |
| Effective search space used | 166886829277872 |


## Graphic Summary

### Distribution of the top 100 Blast Hits on 100 subject sequences [?]

The graphic is an overview of the database sequences aligned to the query sequence. These are represented horizontal bars colored coded by score and showing the extent
of the alignment on the query sequence. Separate aligned regions on the same database sequence are connected by a thin grey line.
Mousing over an alignment shows the database sequence title. Clicking an alignment displays a box with more details about the alignment and
link to the sequence alignment itself in the Alignments section of the report.

Mouse over to see the title, click to show alignments

Color key for alignment scores

<40

40-50

50-80

80-200

>=200

Query

1

200

400

600

800

1000

Paenibacillus sp. 3Cp1 16S ribosomal RNA gene, partial ..

Score:1812 Evalue:0

Accession:KP852522.1

Alignment

Uncultured bacterium clone 4L13 16S ribosomal RNA gene,..

Score:1812 Evalue:0

Accession:KP843054.1

Alignment

Paenibacillus sp. TA\_EU partial 16S rRNA gene, strain T..

Score:1812 Evalue:0

Accession:HG942127.1

Alignment

Bacterium 4R9 16S ribosomal RNA gene, partial sequence

Score:1807 Evalue:0

Accession:KP965575.1

Alignment

Paenibacillus sp. strain PS-K-17 16S ribosomal RNA gene..

Score:1801 Evalue:0

Accession:MF443394.1

Alignment

Paenibacillus sp. TI45-13ar 16S ribosomal RNA gene, par..

Score:1792 Evalue:0

Accession:KY419705.1

Alignment

Paenibacillus sp. Rs5 16S ribosomal RNA gene, partial s..

Score:1790 Evalue:0

Accession:JQ977193.1

Alignment

Paenibacillus sp. 9N4 16S ribosomal RNA gene, partial s..

Score:1777 Evalue:0

Accession:HQ284889.1

Alignment

Paenibacillus sp. strain BSW35 16S ribosomal RNA gene, ..

Score:1775 Evalue:0

Accession:KY446062.1

Alignment

Bacterium 4L18 16S ribosomal RNA gene, partial sequence

Score:1759 Evalue:0

Accession:KP965574.1

Alignment

Bacterium 4L13 16S ribosomal RNA gene, partial sequence

Score:1753 Evalue:0

Accession:KP965573.1

Alignment

Uncultured bacterium clone nbw208h09c1 16S ribosomal RN..

Score:1751 Evalue:0

Accession:GQ073426.1

Alignment

Paenibacillus sp. Q1-R10 16S ribosomal RNA gene, partia..

Score:1749 Evalue:0

Accession:JX994123.1

Alignment

Paenibacillus sp. HDDMM03 16S ribosomal RNA gene, parti..

Score:1746 Evalue:0

Accession:EU723825.1

Alignment

Paenibacillus sp. NCCB 100172 16S ribosomal RNA gene, p..

Score:1746 Evalue:0

Accession:EU003588.1

Alignment

Paenibacillus illinoisensis strain WR\_17 16S ribosomal ..

Score:1740 Evalue:0

Accession:HQ661869.1

Alignment

Uncultured bacterium clone 10-9BM 16S ribosomal RNA gen..

Score:1738 Evalue:0

Accession:HQ143281.1

Alignment

Bacterium 4R1 16S ribosomal RNA gene, partial sequence

Score:1735 Evalue:0

Accession:KP965572.1

Alignment

Uncultured bacterium clone ncd2164d05c1 16S ribosomal R..

Score:1735 Evalue:0

Accession:JF185937.1

Alignment

Uncultured bacterium clone ncd2162h02c1 16S ribosomal R..

Score:1729 Evalue:0

Accession:JF185833.1

Alignment

Uncultured bacterium clone ncd2163a03c1 16S ribosomal R..

Score:1724 Evalue:0

Accession:JF185844.1

Alignment

Paenibacillus nicotianae strain YIM h-19 16S ribosomal ..

Score:1712 Evalue:0

Accession:NR\_134783.2

Alignment

Paenibacillus nicotianae strain YIM h-19 16S ribosomal ..

Score:1712 Evalue:0

Accession:KJ792471.1

Alignment

Paenibacillus kyungheensis strain DCY88 16S ribosomal R..

Score:1705 Evalue:0

Accession:NR\_145628.1

Alignment

Paenibacillus kyungheensis strain DCY88 16S ribosomal R..

Score:1705 Evalue:0

Accession:KF793934.1

Alignment

Paenibacillus hordei strain RH-N24 16S ribosomal RNA ge..

Score:1703 Evalue:0

Accession:NR\_109318.1

Alignment

Paenibacillus sp. R1 16S ribosomal RNA gene, partial se..

Score:1701 Evalue:0

Accession:KR263165.1

Alignment

Paenibacillus illinoisensis genomic DNA containing 16S-..

Score:1676 Evalue:0

Accession:LM994777.1

Alignment

Paenibacillus illinoisensis partial 16S rRNA gene, isol..

Score:1663 Evalue:0

Accession:HE587985.1

Alignment

Paenibacillus sp. SC22 partial 16S rRNA gene, isolate S..

Score:1633 Evalue:0

Accession:HF566260.1

Alignment

Paenibacillus sp. UPUM12 16S ribosomal RNA gene, partia..

Score:1624 Evalue:0

Accession:KP889056.1

Alignment

Paenibacillus illinoisensis partial 16S rRNA gene, isol..

Score:1624 Evalue:0

Accession:HE587984.1

Alignment

Paenibacillus hordei strain poapink1 16S ribosomal RNA ..

Score:1622 Evalue:0

Accession:KJ528493.1

Alignment

Uncultured bacterium clone 56-9BM 16S ribosomal RNA gen..

Score:1596 Evalue:0

Accession:HQ143315.1

Alignment

Paenibacillus sp. WS26 16S ribosomal RNA gene, partial ..

Score:1580 Evalue:0

Accession:JN899576.1

Alignment

Paenibacillus sp. H316 16S ribosomal RNA gene, partial ..

Score:1578 Evalue:0

Accession:KT205397.1

Alignment

Paenibacillus sp. strain B1 16S ribosomal RNA gene, par..

Score:1574 Evalue:0

Accession:KY111475.1

Alignment

Paenibacillus sp. EN-26 16S ribosomal RNA gene, partial..

Score:1574 Evalue:0

Accession:KP702927.1

Alignment

Paenibacillus sp. WS28 16S ribosomal RNA gene, partial ..

Score:1574 Evalue:0

Accession:JN899577.1

Alignment

Paenibacillus sp. WS16 16S ribosomal RNA gene, partial ..

Score:1574 Evalue:0

Accession:JN899571.1

Alignment

Paenibacillus durus strain hswX78 16S ribosomal RNA gen..

Score:1574 Evalue:0

Accession:JQ236815.1

Alignment

Paenibacillus sp. BL13 16S ribosomal RNA gene, partial ..

Score:1574 Evalue:0

Accession:EU912463.1

Alignment

Paenibacillus illinoisensis strain Z002 16S ribosomal R..

Score:1572 Evalue:0

Accession:MG266466.1

Alignment

Paenibacillus sp. A10 16S ribosomal RNA gene, partial s..

Score:1568 Evalue:0

Accession:KP126817.1

Alignment

Paenibacillus illinoisensis strain SBANHCa16 16S riboso..

Score:1568 Evalue:0

Accession:KR152299.1

Alignment

Bacterium enrichment culture clone JYJ-11 16S ribosomal..

Score:1568 Evalue:0

Accession:KR996781.1

Alignment

Paenibacillus illinoisensis strain P9S 16S ribosomal RN..

Score:1568 Evalue:0

Accession:KF010797.1

Alignment

Paenibacillus sp. WS30 16S ribosomal RNA gene, partial ..

Score:1568 Evalue:0

Accession:JN899578.1

Alignment

Paenibacillus sp. MBEU37 gene for 16S rRNA, partial seq..

Score:1568 Evalue:0

Accession:AB733580.1

Alignment

Paenibacillus illinoisensis strain F24 16S ribosomal RN..

Score:1568 Evalue:0

Accession:JQ579623.1

Alignment

Paenibacillus illinoisensis strain 3728 16S ribosomal R..

Score:1568 Evalue:0

Accession:JN867752.1

Alignment

Paenibacillus durus strain YUPP-3 16S ribosomal RNA gen..

Score:1568 Evalue:0

Accession:FJ696708.1

Alignment

Paenibacillus illinoisensis partial 16S rRNA gene, stra..

Score:1568 Evalue:0

Accession:FN422001.1

Alignment

Paenibacillus xylanilyticus strain F2-2-22 16S ribosoma..

Score:1567 Evalue:0

Accession:KX350030.1

Alignment

Paenibacillus sp. CRRI-82\_WR13B 16S ribosomal RNA gene,..

Score:1567 Evalue:0

Accession:KF840379.1

Alignment

Paenibacillus illinoisensis strain JCM 9907 16S ribosom..

Score:1567 Evalue:0

Accession:NR\_040884.1

Alignment

## Descriptions

, Reading indexes 1-5, displaying indexes 1-5


Load next setPrevious Match

Sequences producing significant alignments:

Show all columns  of the table presenting sequences producing significant alignments 

Select:AllNone
Selected:0

Alignments
Download

FASTA (complete sequence)

FASTA (aligned sequences)

GenBank (complete sequence)

Hit Table (text)

Hit Table (CSV)

Text

XML

ASN.1

Continue
Cancel

GenBank 
Graphics
Distance tree of results
Multiple alignment
Show/hide columns of the table presenting sequences producing significant alignments 

Available columns

Description  
Max Score  
Total Score  
Coverage  
E-value  
IdentN  
Accession  
Restore Defaults
Ok
Cancel

Sequences producing significant alignments:

| Select for downloading or viewing reports | Description | Max score | Total score | Query cover | E value | Ident | Accession |
| --- | --- | --- | --- | --- | --- | --- | --- |
| 1Select seq KP852522.1 | Paenibacillus sp. 3Cp1 16S ribosomal RNA gene, partial sequence | 1812 | 1812 | 98% | 0.0 | 99% | KP852522.1 |
| 2Select seq KP843054.1 | Uncultured bacterium clone 4L13 16S ribosomal RNA gene, partial sequence | 1812 | 1812 | 98% | 0.0 | 99% | KP843054.1 |
| 3Select seq HG942127.1 | Paenibacillus sp. TA\_EU partial 16S rRNA gene, strain TA\_EU | 1812 | 1812 | 98% | 0.0 | 99% | HG942127.1 |
| 4Select seq KP965575.1 | Bacterium 4R9 16S ribosomal RNA gene, partial sequence | 1807 | 1807 | 98% | 0.0 | 99% | KP965575.1 |
| 5Select seq MF443394.1 | Paenibacillus sp. strain PS-K-17 16S ribosomal RNA gene, partial sequence | 1801 | 1801 | 98% | 0.0 | 99% | MF443394.1 |
| 6Select seq KY419705.1 | Paenibacillus sp. TI45-13ar 16S ribosomal RNA gene, partial sequence | 1792 | 1792 | 98% | 0.0 | 99% | KY419705.1 |
| 7Select seq JQ977193.1 | Paenibacillus sp. Rs5 16S ribosomal RNA gene, partial sequence | 1790 | 1790 | 98% | 0.0 | 99% | JQ977193.1 |
| 8Select seq HQ284889.1 | Paenibacillus sp. 9N4 16S ribosomal RNA gene, partial sequence | 1777 | 1777 | 98% | 0.0 | 99% | HQ284889.1 |
| 9Select seq KY446062.1 | Paenibacillus sp. strain BSW35 16S ribosomal RNA gene, partial sequence | 1775 | 1775 | 98% | 0.0 | 99% | KY446062.1 |
| 10Select seq KP965574.1 | Bacterium 4L18 16S ribosomal RNA gene, partial sequence | 1759 | 1759 | 98% | 0.0 | 98% | KP965574.1 |
| 11Select seq KP965573.1 | Bacterium 4L13 16S ribosomal RNA gene, partial sequence | 1753 | 1753 | 98% | 0.0 | 98% | KP965573.1 |
| 12Select seq GQ073426.1 | Uncultured bacterium clone nbw208h09c1 16S ribosomal RNA gene, partial sequence | 1751 | 1751 | 98% | 0.0 | 98% | GQ073426.1 |
| 13Select seq JX994123.1 | Paenibacillus sp. Q1-R10 16S ribosomal RNA gene, partial sequence | 1749 | 1749 | 98% | 0.0 | 98% | JX994123.1 |
| 14Select seq EU723825.1 | Paenibacillus sp. HDDMM03 16S ribosomal RNA gene, partial sequence | 1746 | 1746 | 98% | 0.0 | 98% | EU723825.1 |
| 15Select seq EU003588.1 | Paenibacillus sp. NCCB 100172 16S ribosomal RNA gene, partial sequence | 1746 | 1746 | 98% | 0.0 | 98% | EU003588.1 |
| 16Select seq HQ661869.1 | Paenibacillus illinoisensis strain WR\_17 16S ribosomal RNA gene, partial sequence | 1740 | 1740 | 95% | 0.0 | 99% | HQ661869.1 |
| 17Select seq HQ143281.1 | Uncultured bacterium clone 10-9BM 16S ribosomal RNA gene, partial sequence | 1738 | 1738 | 98% | 0.0 | 98% | HQ143281.1 |
| 18Select seq KP965572.1 | Bacterium 4R1 16S ribosomal RNA gene, partial sequence | 1735 | 1735 | 98% | 0.0 | 98% | KP965572.1 |
| 19Select seq JF185937.1 | Uncultured bacterium clone ncd2164d05c1 16S ribosomal RNA gene, partial sequence | 1735 | 1735 | 98% | 0.0 | 98% | JF185937.1 |
| 20Select seq JF185833.1 | Uncultured bacterium clone ncd2162h02c1 16S ribosomal RNA gene, partial sequence | 1729 | 1729 | 98% | 0.0 | 98% | JF185833.1 |
| 21Select seq JF185844.1 | Uncultured bacterium clone ncd2163a03c1 16S ribosomal RNA gene, partial sequence | 1724 | 1724 | 98% | 0.0 | 98% | JF185844.1 |
| 22Select seq NR\_134783.2 | Paenibacillus nicotianae strain YIM h-19 16S ribosomal RNA, complete sequence | 1712 | 1712 | 98% | 0.0 | 98% | NR\_134783.2 |
| 23Select seq KJ792471.1 | Paenibacillus nicotianae strain YIM h-19 16S ribosomal RNA gene, partial sequence | 1712 | 1712 | 98% | 0.0 | 98% | KJ792471.1 |
| 24Select seq NR\_145628.1 | Paenibacillus kyungheensis strain DCY88 16S ribosomal RNA, partial sequence | 1705 | 1705 | 98% | 0.0 | 97% | NR\_145628.1 |
| 25Select seq KF793934.1 | Paenibacillus kyungheensis strain DCY88 16S ribosomal RNA gene, partial sequence | 1705 | 1705 | 98% | 0.0 | 97% | KF793934.1 |
| 26Select seq NR\_109318.1 | Paenibacillus hordei strain RH-N24 16S ribosomal RNA gene, partial sequence | 1703 | 1703 | 98% | 0.0 | 98% | NR\_109318.1 |
| 27Select seq KR263165.1 | Paenibacillus sp. R1 16S ribosomal RNA gene, partial sequence | 1701 | 1701 | 98% | 0.0 | 97% | KR263165.1 |
| 28Select seq LM994777.1 | Paenibacillus illinoisensis genomic DNA containing 16S-23S intergenic spacer region, strain PuiB5.2 | 1676 | 1676 | 93% | 0.0 | 99% | LM994777.1 |
| 29Select seq HE587985.1 | Paenibacillus illinoisensis partial 16S rRNA gene, isolate C5 1197 | 1663 | 1663 | 93% | 0.0 | 98% | HE587985.1 |
| 30Select seq HF566260.1 | Paenibacillus sp. SC22 partial 16S rRNA gene, isolate SC22 | 1633 | 1633 | 94% | 0.0 | 97% | HF566260.1 |
| 31Select seq KP889056.1 | Paenibacillus sp. UPUM12 16S ribosomal RNA gene, partial sequence | 1624 | 1624 | 94% | 0.0 | 97% | KP889056.1 |
| 32Select seq HE587984.1 | Paenibacillus illinoisensis partial 16S rRNA gene, isolate C5 1196 | 1624 | 1624 | 95% | 0.0 | 97% | HE587984.1 |
| 33Select seq KJ528493.1 | Paenibacillus hordei strain poapink1 16S ribosomal RNA gene, partial sequence | 1622 | 1622 | 89% | 0.0 | 99% | KJ528493.1 |
| 34Select seq HQ143315.1 | Uncultured bacterium clone 56-9BM 16S ribosomal RNA gene, partial sequence | 1596 | 1596 | 90% | 0.0 | 98% | HQ143315.1 |
| 35Select seq JN899576.1 | Paenibacillus sp. WS26 16S ribosomal RNA gene, partial sequence | 1580 | 1580 | 98% | 0.0 | 95% | JN899576.1 |
| 36Select seq KT205397.1 | Paenibacillus sp. H316 16S ribosomal RNA gene, partial sequence | 1578 | 1578 | 98% | 0.0 | 95% | KT205397.1 |
| 37Select seq KY111475.1 | Paenibacillus sp. strain B1 16S ribosomal RNA gene, partial sequence | 1574 | 1574 | 98% | 0.0 | 95% | KY111475.1 |
| 38Select seq KP702927.1 | Paenibacillus sp. EN-26 16S ribosomal RNA gene, partial sequence | 1574 | 1574 | 98% | 0.0 | 95% | KP702927.1 |
| 39Select seq JN899577.1 | Paenibacillus sp. WS28 16S ribosomal RNA gene, partial sequence | 1574 | 1574 | 98% | 0.0 | 95% | JN899577.1 |
| 40Select seq JN899571.1 | Paenibacillus sp. WS16 16S ribosomal RNA gene, partial sequence | 1574 | 1574 | 98% | 0.0 | 95% | JN899571.1 |
| 41Select seq JQ236815.1 | Paenibacillus durus strain hswX78 16S ribosomal RNA gene, partial sequence | 1574 | 1574 | 98% | 0.0 | 95% | JQ236815.1 |
| 42Select seq EU912463.1 | Paenibacillus sp. BL13 16S ribosomal RNA gene, partial sequence | 1574 | 1574 | 98% | 0.0 | 95% | EU912463.1 |
| 43Select seq MG266466.1 | Paenibacillus illinoisensis strain Z002 16S ribosomal RNA gene, partial sequence | 1572 | 1572 | 98% | 0.0 | 95% | MG266466.1 |
| 44Select seq KP126817.1 | Paenibacillus sp. A10 16S ribosomal RNA gene, partial sequence | 1568 | 1568 | 98% | 0.0 | 95% | KP126817.1 |
| 45Select seq KR152299.1 | Paenibacillus illinoisensis strain SBANHCa16 16S ribosomal RNA gene, partial sequence | 1568 | 1568 | 98% | 0.0 | 95% | KR152299.1 |
| 46Select seq KR996781.1 | Bacterium enrichment culture clone JYJ-11 16S ribosomal RNA gene, partial sequence | 1568 | 1568 | 98% | 0.0 | 95% | KR996781.1 |
| 47Select seq KF010797.1 | Paenibacillus illinoisensis strain P9S 16S ribosomal RNA gene, partial sequence | 1568 | 1568 | 98% | 0.0 | 95% | KF010797.1 |
| 48Select seq JN899578.1 | Paenibacillus sp. WS30 16S ribosomal RNA gene, partial sequence | 1568 | 1568 | 98% | 0.0 | 95% | JN899578.1 |
| 49Select seq AB733580.1 | Paenibacillus sp. MBEU37 gene for 16S rRNA, partial sequence | 1568 | 1568 | 98% | 0.0 | 95% | AB733580.1 |
| 50Select seq JQ579623.1 | Paenibacillus illinoisensis strain F24 16S ribosomal RNA gene, partial sequence | 1568 | 1568 | 98% | 0.0 | 95% | JQ579623.1 |
| 51Select seq JN867752.1 | Paenibacillus illinoisensis strain 3728 16S ribosomal RNA gene, partial sequence | 1568 | 1568 | 98% | 0.0 | 95% | JN867752.1 |
| 52Select seq FJ696708.1 | Paenibacillus durus strain YUPP-3 16S ribosomal RNA gene, partial sequence | 1568 | 1568 | 98% | 0.0 | 95% | FJ696708.1 |
| 53Select seq FN422001.1 | Paenibacillus illinoisensis partial 16S rRNA gene, strain IB-1087 | 1568 | 1568 | 98% | 0.0 | 95% | FN422001.1 |
| 54Select seq KX350030.1 | Paenibacillus xylanilyticus strain F2-2-22 16S ribosomal RNA gene, partial sequence | 1567 | 1567 | 98% | 0.0 | 95% | KX350030.1 |
| 55Select seq KF840379.1 | Paenibacillus sp. CRRI-82\_WR13B 16S ribosomal RNA gene, partial sequence | 1567 | 1567 | 98% | 0.0 | 95% | KF840379.1 |
| 56Select seq NR\_040884.1 | Paenibacillus illinoisensis strain JCM 9907 16S ribosomal RNA gene, partial sequence | 1567 | 1567 | 98% | 0.0 | 95% | NR\_040884.1 |
| 57Select seq KJ577635.1 | Paenibacillus illinoisensis strain AAU M 17 16S ribosomal RNA gene, partial sequence | 1565 | 1565 | 98% | 0.0 | 95% | KJ577635.1 |
| 58Select seq NR\_113828.1 | Paenibacillus illinoisensis strain NBRC 15959 16S ribosomal RNA gene, partial sequence | 1565 | 1565 | 98% | 0.0 | 95% | NR\_113828.1 |
| 59Select seq KM817264.1 | Paenibacillus illinoisensis strain IHB B 15700 16S ribosomal RNA gene, partial sequence | 1563 | 1563 | 98% | 0.0 | 95% | KM817264.1 |
| 60Select seq KJ769171.1 | Paenibacillus sp. THK-CF25 16S ribosomal RNA gene, partial sequence | 1563 | 1563 | 98% | 0.0 | 95% | KJ769171.1 |
| 61Select seq KF054892.1 | Paenibacillus durus strain IARI-IIWP-40 16S ribosomal RNA gene, partial sequence | 1563 | 1563 | 98% | 0.0 | 95% | KF054892.1 |
| 62Select seq JQ063001.1 | Paenibacillus sp. XGL212 16S ribosomal RNA gene, partial sequence | 1563 | 1563 | 98% | 0.0 | 95% | JQ063001.1 |
| 63Select seq JF892727.1 | Paenibacillus sp. F37 16S ribosomal RNA gene, partial sequence | 1563 | 1563 | 98% | 0.0 | 95% | JF892727.1 |
| 64Select seq EU912464.1 | Paenibacillus sp. BL17-2 16S ribosomal RNA gene, partial sequence | 1563 | 1563 | 98% | 0.0 | 95% | EU912464.1 |
| 65Select seq GU080334.1 | Paenibacillus sp. XP13 16S ribosomal RNA gene, partial sequence | 1563 | 1563 | 98% | 0.0 | 95% | GU080334.1 |
| 66Select seq KR856190.1 | Paenibacillus illinoisensis strain C1 16S ribosomal RNA gene, partial sequence | 1561 | 1561 | 98% | 0.0 | 95% | KR856190.1 |
| 67Select seq KP670215.1 | Paenibacillus sp. DB14410 16S ribosomal RNA gene, partial sequence | 1561 | 1561 | 98% | 0.0 | 95% | KP670215.1 |
| 68Select seq KF535141.1 | Paenibacillus xylanilyticus strain BAB-1610 16S ribosomal RNA gene, partial sequence | 1561 | 1561 | 98% | 0.0 | 95% | KF535141.1 |
| 69Select seq AB680852.1 | Paenibacillus illinoisensis gene for 16S rRNA, partial sequence, strain: NBRC 15379 | 1561 | 1561 | 98% | 0.0 | 95% | AB680852.1 |
| 70Select seq KR856189.1 | Paenibacillus illinoisensis strain D4 16S ribosomal RNA gene, partial sequence | 1559 | 1559 | 98% | 0.0 | 95% | KR856189.1 |
| 71Select seq LC011515.1 | Paenibacillus sp. MTB37-3 gene for 16S ribosomal RNA, partial sequence | 1557 | 1557 | 98% | 0.0 | 95% | LC011515.1 |
| 72Select seq KJ951054.1 | Paenibacillus illinoisensis strain YZ29 16S ribosomal RNA gene, partial sequence | 1557 | 1557 | 98% | 0.0 | 95% | KJ951054.1 |
| 73Select seq KJ600908.1 | Uncultured bacterium clone 18B 16S ribosomal RNA gene, partial sequence | 1557 | 1557 | 98% | 0.0 | 95% | KJ600908.1 |
| 74Select seq KF475708.1 | Bacterium R-22 16S ribosomal RNA gene, partial sequence | 1557 | 1557 | 98% | 0.0 | 95% | KF475708.1 |
| 75Select seq FJ232954.1 | Paenibacillus sp. ZY-8 16S ribosomal RNA gene, partial sequence | 1557 | 1557 | 98% | 0.0 | 95% | FJ232954.1 |
| 76Select seq GQ202680.1 | Paenibacillus sp. U5 STR 16S ribosomal RNA gene, partial sequence | 1557 | 1557 | 98% | 0.0 | 95% | GQ202680.1 |
| 77Select seq DQ105965.1 | P-decomposing bacterium 1 16S ribosomal RNA gene, partial sequence | 1557 | 1557 | 98% | 0.0 | 95% | DQ105965.1 |
| 78Select seq KX825907.1 | Paenibacillus sp. strain I2746 16S ribosomal RNA gene, partial sequence | 1555 | 1555 | 98% | 0.0 | 95% | KX825907.1 |
| 79Select seq AB748941.1 | Paenibacillus xylanilyticus gene for 16S ribosomal RNA, partial sequence, strain: AECSP02 | 1554 | 1554 | 98% | 0.0 | 95% | AB748941.1 |
| 80Select seq KC000067.1 | Uncultured bacterium clone Water1 16S ribosomal RNA gene, partial sequence | 1554 | 1554 | 98% | 0.0 | 95% | KC000067.1 |
| 81Select seq FJ006887.1 | Paenibacillus sp. WPCB083 16S ribosomal RNA gene, partial sequence | 1554 | 1554 | 98% | 0.0 | 95% | FJ006887.1 |
| 82Select seq AB110989.1 | Paenibacillus sp. W-61 gene for 16S rRNA, partial sequence | 1554 | 1554 | 98% | 0.0 | 95% | AB110989.1 |
| 83Select seq MF948342.1 | Bacillus sp. strain FJAT-29882 16S ribosomal RNA gene, partial sequence | 1552 | 1552 | 98% | 0.0 | 95% | MF948342.1 |
| 84Select seq KX212148.1 | Uncultured bacterium clone HT3 16S ribosomal RNA gene, partial sequence | 1552 | 1552 | 98% | 0.0 | 95% | KX212148.1 |
| 85Select seq KP126829.1 | Paenibacillus sp. CF7 16S ribosomal RNA gene, partial sequence | 1552 | 1552 | 98% | 0.0 | 95% | KP126829.1 |
| 86Select seq LC057692.1 | Paenibacillus sp. TBTK040501B gene for 16S ribosomal RNA, partial sequence | 1552 | 1552 | 98% | 0.0 | 95% | LC057692.1 |
| 87Select seq KP172212.1 | Paenibacillus sp. EgN2201 16S ribosomal RNA gene, partial sequence | 1552 | 1552 | 98% | 0.0 | 95% | KP172212.1 |
| 88Select seq KM019819.1 | Paenibacillus taichungensis strain F9 16S ribosomal RNA gene, partial sequence | 1552 | 1552 | 98% | 0.0 | 95% | KM019819.1 |
| 89Select seq GQ284463.1 | Paenibacillus sp. TSWCW18 16S ribosomal RNA gene, partial sequence | 1552 | 1552 | 98% | 0.0 | 95% | GQ284463.1 |
| 90Select seq AB366401.1 | Paenibacillus pabuli gene for 16S ribosomal RNA, partial sequence, strain: SW12 | 1552 | 1552 | 98% | 0.0 | 95% | AB366401.1 |
| 91Select seq AB366400.1 | Paenibacillus pabuli gene for 16S ribosomal RNA, partial sequence, strain: SW10 | 1552 | 1552 | 98% | 0.0 | 95% | AB366400.1 |
| 92Select seq EU218535.1 | Paenibacillus sp. K1 16S ribosomal RNA gene, complete sequence | 1552 | 1552 | 98% | 0.0 | 95% | EU218535.1 |
| 93Select seq DQ288948.1 | Paenibacillus pabuli isolate TG12 16S ribosomal RNA gene, partial sequence | 1552 | 1552 | 98% | 0.0 | 95% | DQ288948.1 |
| 94Select seq KY622502.1 | Paenibacillus sp. strain LB033 16S ribosomal RNA gene, partial sequence | 1550 | 1550 | 98% | 0.0 | 95% | KY622502.1 |
| 95Select seq MG027626.1 | Paenibacillus illinoisensis strain VBE24 16S ribosomal RNA gene, partial sequence | 1550 | 1550 | 98% | 0.0 | 95% | MG027626.1 |
| 96Select seq FJ006870.1 | Paenibacillus sp. WPCB023 16S ribosomal RNA gene, partial sequence | 1550 | 1550 | 98% | 0.0 | 95% | FJ006870.1 |
| 97Select seq KX825908.1 | Paenibacillus sp. strain I2747 16S ribosomal RNA gene, partial sequence | 1548 | 1548 | 98% | 0.0 | 95% | KX825908.1 |
| 98Select seq KP670221.1 | Paenibacillus sp. DB14405 16S ribosomal RNA gene, partial sequence | 1548 | 1548 | 98% | 0.0 | 95% | KP670221.1 |
| 99Select seq MG266469.1 | Paenibacillus xylanilyticus strain Z007 16S ribosomal RNA gene, partial sequence | 1546 | 1546 | 98% | 0.0 | 95% | MG266469.1 |
| 100Select seq KJ544003.1 | Bacterium AM0258 16S ribosomal RNA gene, partial sequence | 1539 | 1539 | 97% | 0.0 | 95% | KJ544003.1 |


## Alignments

Loading alignment... for sequences gi|915515201,gi|915512955,gi|631782762,gi|846924131,gi|1214741488 Reading indexes 1-5

Download

FASTA (complete sequence)

FASTA (aligned sequences)

GenBank (complete sequence)

Continue
Cancel

GenBankGraphics

Next
Previous
Descriptions

Paenibacillus sp. 3Cp1 16S ribosomal RNA gene, partial sequence

Sequence ID: KP852522.1Length: 1434Number of Matches: 1

Related Information

Range 1: 13 to 1003GenBankGraphics

Next Match
Previous Match
First Match

Alignment statistics for match #1

| Score | Expect | Identities | Gaps | Strand | Frame |
| --- | --- | --- | --- | --- | --- |
| 1812 bits(981) | 0.0() | 986/991(99%) | 0/991(0%) | Plus/Plus |  |

Features:

```
Query  9     GTCGAGCGGAGTTGANGAGAAGCTTGCTTCTTTGATGCTTAGCGGCGGACGGGTGAGTAA  68
             ||||||||||||||| ||||||||||||||||||||||||||||||||||||||||||||
Sbjct  13    GTCGAGCGGAGTTGACGAGAAGCTTGCTTCTTTGATGCTTAGCGGCGGACGGGTGAGTAA  72

Query  69    CACGTAGGCAACCTGCCCTCAAGCTTGGGACAACTACCGGAAACGGTAGCTAATACCGAA  128
             ||||||||||||||||||||||||||||||||||||||||||||||||||||||||||||
Sbjct  73    CACGTAGGCAACCTGCCCTCAAGCTTGGGACAACTACCGGAAACGGTAGCTAATACCGAA  132

Query  129   TACATGATTTGTTCGCCTGAACGAATNTGGAAAGACGGAGCAATCTGTCACTTGAGGATG  188
             |||||||||||||||||||||||||| |||||||||||||||||||||||||||||||||
Sbjct  133   TACATGATTTGTTCGCCTGAACGAATTTGGAAAGACGGAGCAATCTGTCACTTGAGGATG  192

Query  189   GGCCTGCGGCGCATTAGCTAGTTGGTGAGGTAACGGCTCACCAAGGCGACGATGCGTAGC  248
             ||||||||||||||||||||||||||||||||||||||||||||||||||||||||||||
Sbjct  193   GGCCTGCGGCGCATTAGCTAGTTGGTGAGGTAACGGCTCACCAAGGCGACGATGCGTAGC  252

Query  249   CGACCTGAGAGGGTGATCGGCCACACTGGGACTGAGACACGGCCCAGACTCCTACGGGAG  308
             ||||||||||||||||||||||||||||||||||||||||||||||||||||||||||||
Sbjct  253   CGACCTGAGAGGGTGATCGGCCACACTGGGACTGAGACACGGCCCAGACTCCTACGGGAG  312

Query  309   GCAGCAGTAGGGAATCTTCCGCAATGGACGCAAGTCTGACGGAGCAACGCCGCGTGAGTG  368
             ||||||||||||||||||||||||||||||||||||||||||||||||||||||||||||
Sbjct  313   GCAGCAGTAGGGAATCTTCCGCAATGGACGCAAGTCTGACGGAGCAACGCCGCGTGAGTG  372

Query  369   ATGAAGGTTTTCGGATCGTAAAGCTCTGTTGCCAGGGAAGAACGTCNNATAGAGTAACTG  428
             ||||||||||||||||||||||||||||||||||||||||||||||  ||||||||||||
Sbjct  373   ATGAAGGTTTTCGGATCGTAAAGCTCTGTTGCCAGGGAAGAACGTCCGATAGAGTAACTG  432

Query  429   CTATCGGAGTGACGGTACCTGAGAAGAAAGCCCCGGCTAACTACGTGCCAGCAGCCGCGG  488
             ||||||||||||||||||||||||||||||||||||||||||||||||||||||||||||
Sbjct  433   CTATCGGAGTGACGGTACCTGAGAAGAAAGCCCCGGCTAACTACGTGCCAGCAGCCGCGG  492

Query  489   TAATACGTAGGGGGCAAGCGTTGTCCGGAATTATTGGGCGTAAAGCGCGCGCAGGCGGCT  548
             ||||||||||||||||||||||||||||||||||||||||||||||||||||||||||||
Sbjct  493   TAATACGTAGGGGGCAAGCGTTGTCCGGAATTATTGGGCGTAAAGCGCGCGCAGGCGGCT  552

Query  549   TTTTAAGTCCGGTGTCACAGCCCAAGGCTCAACCTTGGGTCGCACTGGAAACTGGAGAGC  608
             ||||||||||||||||||||||||||||||||||||||||||||||||||||||||||||
Sbjct  553   TTTTAAGTCCGGTGTCACAGCCCAAGGCTCAACCTTGGGTCGCACTGGAAACTGGAGAGC  612

Query  609   TTGAGTACAGAAGAGGAAAGTGGAATTCCACGTGTAGCGGTGAAATGCGTAGAGATGTGG  668
             ||||||||||||||||||||||||||||||||||||||||||||||||||||||||||||
Sbjct  613   TTGAGTACAGAAGAGGAAAGTGGAATTCCACGTGTAGCGGTGAAATGCGTAGAGATGTGG  672

Query  669   AGGAACACCAGTGGCGAAGGCGACTTTCTGGGCTGTAACTGACGCTGAGGCGCGAAAGCG  728
             ||||||||||||||||||||||||||||||||||||||||||||||||||||||||||||
Sbjct  673   AGGAACACCAGTGGCGAAGGCGACTTTCTGGGCTGTAACTGACGCTGAGGCGCGAAAGCG  732

Query  729   TGGGGAGCAAACAGGATTAGATACCCTGGTAGTCCACGCCGTAAACGATGAATGCTAGGT  788
             ||||||||||||||||||||||||||||||||||||||||||||||||||||||||||||
Sbjct  733   TGGGGAGCAAACAGGATTAGATACCCTGGTAGTCCACGCCGTAAACGATGAATGCTAGGT  792

Query  789   GTTAGGGGTTTCGATACCCTTGGTGCCGAAGTTAACACATTAAGCATTCCGCCTGGGGAG  848
             ||||||||||||||||||||||||||||||||||||||||||||||||||||||||||||
Sbjct  793   GTTAGGGGTTTCGATACCCTTGGTGCCGAAGTTAACACATTAAGCATTCCGCCTGGGGAG  852

Query  849   TACGGTCGCAAGACTGAAACTCAAAGGAATTGACGGGGACCCGCACAAGCAGTGGNGTAT  908
             ||||||||||||||||||||||||||||||||||||||||||||||||||||||| ||||
Sbjct  853   TACGGTCGCAAGACTGAAACTCAAAGGAATTGACGGGGACCCGCACAAGCAGTGGAGTAT  912

Query  909   GTGGTTTAATTCGAAGCAACGCGAAGAACCTTACCAAGTCTTGACATCCCTTTGACCGGA  968
             ||||||||||||||||||||||||||||||||||||||||||||||||||||||||||||
Sbjct  913   GTGGTTTAATTCGAAGCAACGCGAAGAACCTTACCAAGTCTTGACATCCCTTTGACCGGA  972

Query  969   CTAGAGATAGTCTTTTCCTTCGGGACAAAGG  999
             |||||||||||||||||||||||||||||||
Sbjct  973   CTAGAGATAGTCTTTTCCTTCGGGACAAAGG  1003
```

Download

FASTA (complete sequence)

FASTA (aligned sequences)

GenBank (complete sequence)

Continue
Cancel

GenBankGraphics

Next
Previous
Descriptions

Uncultured bacterium clone 4L13 16S ribosomal RNA gene, partial sequence

Sequence ID: KP843054.1Length: 1460Number of Matches: 1

Related Information

Range 1: 25 to 1015GenBankGraphics

Next Match
Previous Match
First Match

Alignment statistics for match #1

| Score | Expect | Identities | Gaps | Strand | Frame |
| --- | --- | --- | --- | --- | --- |
| 1812 bits(981) | 0.0() | 986/991(99%) | 0/991(0%) | Plus/Plus |  |

Features:

```
Query  9     GTCGAGCGGAGTTGANGAGAAGCTTGCTTCTTTGATGCTTAGCGGCGGACGGGTGAGTAA  68
             ||||||||||||||| ||||||||||||||||||||||||||||||||||||||||||||
Sbjct  25    GTCGAGCGGAGTTGACGAGAAGCTTGCTTCTTTGATGCTTAGCGGCGGACGGGTGAGTAA  84

Query  69    CACGTAGGCAACCTGCCCTCAAGCTTGGGACAACTACCGGAAACGGTAGCTAATACCGAA  128
             ||||||||||||||||||||||||||||||||||||||||||||||||||||||||||||
Sbjct  85    CACGTAGGCAACCTGCCCTCAAGCTTGGGACAACTACCGGAAACGGTAGCTAATACCGAA  144

Query  129   TACATGATTTGTTCGCCTGAACGAATNTGGAAAGACGGAGCAATCTGTCACTTGAGGATG  188
             |||||||||||||||||||||||||| |||||||||||||||||||||||||||||||||
Sbjct  145   TACATGATTTGTTCGCCTGAACGAATTTGGAAAGACGGAGCAATCTGTCACTTGAGGATG  204

Query  189   GGCCTGCGGCGCATTAGCTAGTTGGTGAGGTAACGGCTCACCAAGGCGACGATGCGTAGC  248
             ||||||||||||||||||||||||||||||||||||||||||||||||||||||||||||
Sbjct  205   GGCCTGCGGCGCATTAGCTAGTTGGTGAGGTAACGGCTCACCAAGGCGACGATGCGTAGC  264

Query  249   CGACCTGAGAGGGTGATCGGCCACACTGGGACTGAGACACGGCCCAGACTCCTACGGGAG  308
             ||||||||||||||||||||||||||||||||||||||||||||||||||||||||||||
Sbjct  265   CGACCTGAGAGGGTGATCGGCCACACTGGGACTGAGACACGGCCCAGACTCCTACGGGAG  324

Query  309   GCAGCAGTAGGGAATCTTCCGCAATGGACGCAAGTCTGACGGAGCAACGCCGCGTGAGTG  368
             ||||||||||||||||||||||||||||||||||||||||||||||||||||||||||||
Sbjct  325   GCAGCAGTAGGGAATCTTCCGCAATGGACGCAAGTCTGACGGAGCAACGCCGCGTGAGTG  384

Query  369   ATGAAGGTTTTCGGATCGTAAAGCTCTGTTGCCAGGGAAGAACGTCNNATAGAGTAACTG  428
             ||||||||||||||||||||||||||||||||||||||||||||||  ||||||||||||
Sbjct  385   ATGAAGGTTTTCGGATCGTAAAGCTCTGTTGCCAGGGAAGAACGTCCGATAGAGTAACTG  444

Query  429   CTATCGGAGTGACGGTACCTGAGAAGAAAGCCCCGGCTAACTACGTGCCAGCAGCCGCGG  488
             ||||||||||||||||||||||||||||||||||||||||||||||||||||||||||||
Sbjct  445   CTATCGGAGTGACGGTACCTGAGAAGAAAGCCCCGGCTAACTACGTGCCAGCAGCCGCGG  504

Query  489   TAATACGTAGGGGGCAAGCGTTGTCCGGAATTATTGGGCGTAAAGCGCGCGCAGGCGGCT  548
             ||||||||||||||||||||||||||||||||||||||||||||||||||||||||||||
Sbjct  505   TAATACGTAGGGGGCAAGCGTTGTCCGGAATTATTGGGCGTAAAGCGCGCGCAGGCGGCT  564

Query  549   TTTTAAGTCCGGTGTCACAGCCCAAGGCTCAACCTTGGGTCGCACTGGAAACTGGAGAGC  608
             ||||||||||||||||||||||||||||||||||||||||||||||||||||||||||||
Sbjct  565   TTTTAAGTCCGGTGTCACAGCCCAAGGCTCAACCTTGGGTCGCACTGGAAACTGGAGAGC  624

Query  609   TTGAGTACAGAAGAGGAAAGTGGAATTCCACGTGTAGCGGTGAAATGCGTAGAGATGTGG  668
             ||||||||||||||||||||||||||||||||||||||||||||||||||||||||||||
Sbjct  625   TTGAGTACAGAAGAGGAAAGTGGAATTCCACGTGTAGCGGTGAAATGCGTAGAGATGTGG  684

Query  669   AGGAACACCAGTGGCGAAGGCGACTTTCTGGGCTGTAACTGACGCTGAGGCGCGAAAGCG  728
             ||||||||||||||||||||||||||||||||||||||||||||||||||||||||||||
Sbjct  685   AGGAACACCAGTGGCGAAGGCGACTTTCTGGGCTGTAACTGACGCTGAGGCGCGAAAGCG  744

Query  729   TGGGGAGCAAACAGGATTAGATACCCTGGTAGTCCACGCCGTAAACGATGAATGCTAGGT  788
             ||||||||||||||||||||||||||||||||||||||||||||||||||||||||||||
Sbjct  745   TGGGGAGCAAACAGGATTAGATACCCTGGTAGTCCACGCCGTAAACGATGAATGCTAGGT  804

Query  789   GTTAGGGGTTTCGATACCCTTGGTGCCGAAGTTAACACATTAAGCATTCCGCCTGGGGAG  848
             ||||||||||||||||||||||||||||||||||||||||||||||||||||||||||||
Sbjct  805   GTTAGGGGTTTCGATACCCTTGGTGCCGAAGTTAACACATTAAGCATTCCGCCTGGGGAG  864

Query  849   TACGGTCGCAAGACTGAAACTCAAAGGAATTGACGGGGACCCGCACAAGCAGTGGNGTAT  908
             ||||||||||||||||||||||||||||||||||||||||||||||||||||||| ||||
Sbjct  865   TACGGTCGCAAGACTGAAACTCAAAGGAATTGACGGGGACCCGCACAAGCAGTGGAGTAT  924

Query  909   GTGGTTTAATTCGAAGCAACGCGAAGAACCTTACCAAGTCTTGACATCCCTTTGACCGGA  968
             ||||||||||||||||||||||||||||||||||||||||||||||||||||||||||||
Sbjct  925   GTGGTTTAATTCGAAGCAACGCGAAGAACCTTACCAAGTCTTGACATCCCTTTGACCGGA  984

Query  969   CTAGAGATAGTCTTTTCCTTCGGGACAAAGG  999
             |||||||||||||||||||||||||||||||
Sbjct  985   CTAGAGATAGTCTTTTCCTTCGGGACAAAGG  1015
```

Download

FASTA (complete sequence)

FASTA (aligned sequences)

GenBank (complete sequence)

Continue
Cancel

GenBankGraphics

Next
Previous
Descriptions

Paenibacillus sp. TA\_EU partial 16S rRNA gene, strain TA\_EU

Sequence ID: HG942127.1Length: 1310Number of Matches: 1

Related Information

Range 1: 4 to 994GenBankGraphics

Next Match
Previous Match
First Match

Alignment statistics for match #1

| Score | Expect | Identities | Gaps | Strand | Frame |
| --- | --- | --- | --- | --- | --- |
| 1812 bits(981) | 0.0() | 986/991(99%) | 0/991(0%) | Plus/Plus |  |

Features:

```
Query  9    GTCGAGCGGAGTTGANGAGAAGCTTGCTTCTTTGATGCTTAGCGGCGGACGGGTGAGTAA  68
            ||||||||||||||| ||||||||||||||||||||||||||||||||||||||||||||
Sbjct  4    GTCGAGCGGAGTTGACGAGAAGCTTGCTTCTTTGATGCTTAGCGGCGGACGGGTGAGTAA  63

Query  69   CACGTAGGCAACCTGCCCTCAAGCTTGGGACAACTACCGGAAACGGTAGCTAATACCGAA  128
            ||||||||||||||||||||||||||||||||||||||||||||||||||||||||||||
Sbjct  64   CACGTAGGCAACCTGCCCTCAAGCTTGGGACAACTACCGGAAACGGTAGCTAATACCGAA  123

Query  129  TACATGATTTGTTCGCCTGAACGAATNTGGAAAGACGGAGCAATCTGTCACTTGAGGATG  188
            |||||||||||||||||||||||||| |||||||||||||||||||||||||||||||||
Sbjct  124  TACATGATTTGTTCGCCTGAACGAATTTGGAAAGACGGAGCAATCTGTCACTTGAGGATG  183

Query  189  GGCCTGCGGCGCATTAGCTAGTTGGTGAGGTAACGGCTCACCAAGGCGACGATGCGTAGC  248
            ||||||||||||||||||||||||||||||||||||||||||||||||||||||||||||
Sbjct  184  GGCCTGCGGCGCATTAGCTAGTTGGTGAGGTAACGGCTCACCAAGGCGACGATGCGTAGC  243

Query  249  CGACCTGAGAGGGTGATCGGCCACACTGGGACTGAGACACGGCCCAGACTCCTACGGGAG  308
            ||||||||||||||||||||||||||||||||||||||||||||||||||||||||||||
Sbjct  244  CGACCTGAGAGGGTGATCGGCCACACTGGGACTGAGACACGGCCCAGACTCCTACGGGAG  303

Query  309  GCAGCAGTAGGGAATCTTCCGCAATGGACGCAAGTCTGACGGAGCAACGCCGCGTGAGTG  368
            ||||||||||||||||||||||||||||||||||||||||||||||||||||||||||||
Sbjct  304  GCAGCAGTAGGGAATCTTCCGCAATGGACGCAAGTCTGACGGAGCAACGCCGCGTGAGTG  363

Query  369  ATGAAGGTTTTCGGATCGTAAAGCTCTGTTGCCAGGGAAGAACGTCNNATAGAGTAACTG  428
            ||||||||||||||||||||||||||||||||||||||||||||||  ||||||||||||
Sbjct  364  ATGAAGGTTTTCGGATCGTAAAGCTCTGTTGCCAGGGAAGAACGTCCGATAGAGTAACTG  423

Query  429  CTATCGGAGTGACGGTACCTGAGAAGAAAGCCCCGGCTAACTACGTGCCAGCAGCCGCGG  488
            ||||||||||||||||||||||||||||||||||||||||||||||||||||||||||||
Sbjct  424  CTATCGGAGTGACGGTACCTGAGAAGAAAGCCCCGGCTAACTACGTGCCAGCAGCCGCGG  483

Query  489  TAATACGTAGGGGGCAAGCGTTGTCCGGAATTATTGGGCGTAAAGCGCGCGCAGGCGGCT  548
            ||||||||||||||||||||||||||||||||||||||||||||||||||||||||||||
Sbjct  484  TAATACGTAGGGGGCAAGCGTTGTCCGGAATTATTGGGCGTAAAGCGCGCGCAGGCGGCT  543

Query  549  TTTTAAGTCCGGTGTCACAGCCCAAGGCTCAACCTTGGGTCGCACTGGAAACTGGAGAGC  608
            ||||||||||||||||||||||||||||||||||||||||||||||||||||||||||||
Sbjct  544  TTTTAAGTCCGGTGTCACAGCCCAAGGCTCAACCTTGGGTCGCACTGGAAACTGGAGAGC  603

Query  609  TTGAGTACAGAAGAGGAAAGTGGAATTCCACGTGTAGCGGTGAAATGCGTAGAGATGTGG  668
            ||||||||||||||||||||||||||||||||||||||||||||||||||||||||||||
Sbjct  604  TTGAGTACAGAAGAGGAAAGTGGAATTCCACGTGTAGCGGTGAAATGCGTAGAGATGTGG  663

Query  669  AGGAACACCAGTGGCGAAGGCGACTTTCTGGGCTGTAACTGACGCTGAGGCGCGAAAGCG  728
            ||||||||||||||||||||||||||||||||||||||||||||||||||||||||||||
Sbjct  664  AGGAACACCAGTGGCGAAGGCGACTTTCTGGGCTGTAACTGACGCTGAGGCGCGAAAGCG  723

Query  729  TGGGGAGCAAACAGGATTAGATACCCTGGTAGTCCACGCCGTAAACGATGAATGCTAGGT  788
            ||||||||||||||||||||||||||||||||||||||||||||||||||||||||||||
Sbjct  724  TGGGGAGCAAACAGGATTAGATACCCTGGTAGTCCACGCCGTAAACGATGAATGCTAGGT  783

Query  789  GTTAGGGGTTTCGATACCCTTGGTGCCGAAGTTAACACATTAAGCATTCCGCCTGGGGAG  848
            ||||||||||||||||||||||||||||||||||||||||||||||||||||||||||||
Sbjct  784  GTTAGGGGTTTCGATACCCTTGGTGCCGAAGTTAACACATTAAGCATTCCGCCTGGGGAG  843

Query  849  TACGGTCGCAAGACTGAAACTCAAAGGAATTGACGGGGACCCGCACAAGCAGTGGNGTAT  908
            ||||||||||||||||||||||||||||||||||||||||||||||||||||||| ||||
Sbjct  844  TACGGTCGCAAGACTGAAACTCAAAGGAATTGACGGGGACCCGCACAAGCAGTGGAGTAT  903

Query  909  GTGGTTTAATTCGAAGCAACGCGAAGAACCTTACCAAGTCTTGACATCCCTTTGACCGGA  968
            ||||||||||||||||||||||||||||||||||||||||||||||||||||||||||||
Sbjct  904  GTGGTTTAATTCGAAGCAACGCGAAGAACCTTACCAAGTCTTGACATCCCTTTGACCGGA  963

Query  969  CTAGAGATAGTCTTTTCCTTCGGGACAAAGG  999
            |||||||||||||||||||||||||||||||
Sbjct  964  CTAGAGATAGTCTTTTCCTTCGGGACAAAGG  994
```

Download

FASTA (complete sequence)

FASTA (aligned sequences)

GenBank (complete sequence)

Continue
Cancel

GenBankGraphics

Next
Previous
Descriptions

Bacterium 4R9 16S ribosomal RNA gene, partial sequence

Sequence ID: KP965575.1Length: 1517Number of Matches: 1

Related Information

Range 1: 54 to 1045GenBankGraphics

Next Match
Previous Match
First Match

Alignment statistics for match #1

| Score | Expect | Identities | Gaps | Strand | Frame |
| --- | --- | --- | --- | --- | --- |
| 1807 bits(978) | 0.0() | 986/992(99%) | 1/992(0%) | Plus/Plus |  |

Features:

```
Query  9     GTCGAGCGGAGTTGANGAGAAGCTTGCTTCTTTGATGCTTAGCGGCGGACGGGTGAGTAA  68
             ||||||||||||||| ||||||||||||||||||||||||||||||||||||||||||||
Sbjct  54    GTCGAGCGGAGTTGACGAGAAGCTTGCTTCTTTGATGCTTAGCGGCGGACGGGTGAGTAA  113

Query  69    CACGTAGGCAACCTGCCCTCAAGCTTGGGACAACTACCGGAAACGGTAGCTAATACCGAA  128
             ||||||||||||||||||||||||||||||||||||||||||||||||||||||||||||
Sbjct  114   CACGTAGGCAACCTGCCCTCAAGCTTGGGACAACTACCGGAAACGGTAGCTAATACCGAA  173

Query  129   TACATGATTTGTTCGCCTGAACGAATNTGGAAAGACGGAGCAATCTGTCACTTGAGGATG  188
             |||||||||||||||||||||||||| |||||||||||||||||||||||||||||||||
Sbjct  174   TACATGATTTGTTCGCCTGAACGAATTTGGAAAGACGGAGCAATCTGTCACTTGAGGATG  233

Query  189   GGCCTGCGGCGCATTAGCTAGTTGGTGAGGTAACGGCTCACCAAGGCGACGATGCGTAGC  248
             ||||||||||||||||||||||||||||||||||||||||||||||||||||||||||||
Sbjct  234   GGCCTGCGGCGCATTAGCTAGTTGGTGAGGTAACGGCTCACCAAGGCGACGATGCGTAGC  293

Query  249   CGACCTGAGAGGGTGATCGGCCACACTGGGACTGAGACACGGCCCAGACTCCTACGGGAG  308
             ||||||||||||||||||||||||||||||||||||||||||||||||||||||||||||
Sbjct  294   CGACCTGAGAGGGTGATCGGCCACACTGGGACTGAGACACGGCCCAGACTCCTACGGGAG  353

Query  309   GCAGCAGTAGGGAATCTTCCGCAATGGACGCAAGTCTGACGGAGCAACGCCGCGTGAGTG  368
             ||||||||||||||||||||||||||||||||||||||||||||||||||||||||||||
Sbjct  354   GCAGCAGTAGGGAATCTTCCGCAATGGACGCAAGTCTGACGGAGCAACGCCGCGTGAGTG  413

Query  369   ATGAAGGTTTTCGGATCGTAAAGCTCTGTTGCCAGGGAAGAACGTCNNATAGAGTAACTG  428
             ||||||||||||||||||||||||||||||||||||||||||||||  ||||||||||||
Sbjct  414   ATGAAGGTTTTCGGATCGTAAAGCTCTGTTGCCAGGGAAGAACGTCCGATAGAGTAACTG  473

Query  429   CTATCGGAGTGACGGTACCTGAGAAGAAAGCCCCGGCTAACTACGTGCCAGCAGCCGCGG  488
             ||||||||||||||||||||||||||||||||||||||||||||||||||||||||||||
Sbjct  474   CTATCGGAGTGACGGTACCTGAGAAGAAAGCCCCGGCTAACTACGTGCCAGCAGCCGCGG  533

Query  489   TAATACGTAGGGGGCAAGCGTTGTCCGGAATTATTGGGCGTAAAGCGCGCGCAGGCGGCT  548
             ||||||||||||||||||||||||||||||||||||||||||||||||||||||||||||
Sbjct  534   TAATACGTAGGGGGCAAGCGTTGTCCGGAATTATTGGGCGTAAAGCGCGCGCAGGCGGCT  593

Query  549   TTTTAAGTCCGGTGTCACAGCCCAAGGCTCAA-CCTTGGGTCGCACTGGAAACTGGAGAG  607
             |||||||||||||||||||||||||||||||| |||||||||||||||||||||||||||
Sbjct  594   TTTTAAGTCCGGTGTCACAGCCCAAGGCTCAACCCTTGGGTCGCACTGGAAACTGGAGAG  653

Query  608   CTTGAGTACAGAAGAGGAAAGTGGAATTCCACGTGTAGCGGTGAAATGCGTAGAGATGTG  667
             ||||||||||||||||||||||||||||||||||||||||||||||||||||||||||||
Sbjct  654   CTTGAGTACAGAAGAGGAAAGTGGAATTCCACGTGTAGCGGTGAAATGCGTAGAGATGTG  713

Query  668   GAGGAACACCAGTGGCGAAGGCGACTTTCTGGGCTGTAACTGACGCTGAGGCGCGAAAGC  727
             ||||||||||||||||||||||||||||||||||||||||||||||||||||||||||||
Sbjct  714   GAGGAACACCAGTGGCGAAGGCGACTTTCTGGGCTGTAACTGACGCTGAGGCGCGAAAGC  773

Query  728   GTGGGGAGCAAACAGGATTAGATACCCTGGTAGTCCACGCCGTAAACGATGAATGCTAGG  787
             ||||||||||||||||||||||||||||||||||||||||||||||||||||||||||||
Sbjct  774   GTGGGGAGCAAACAGGATTAGATACCCTGGTAGTCCACGCCGTAAACGATGAATGCTAGG  833

Query  788   TGTTAGGGGTTTCGATACCCTTGGTGCCGAAGTTAACACATTAAGCATTCCGCCTGGGGA  847
             ||||||||||||||||||||||||||||||||||||||||||||||||||||||||||||
Sbjct  834   TGTTAGGGGTTTCGATACCCTTGGTGCCGAAGTTAACACATTAAGCATTCCGCCTGGGGA  893

Query  848   GTACGGTCGCAAGACTGAAACTCAAAGGAATTGACGGGGACCCGCACAAGCAGTGGNGTA  907
             |||||||||||||||||||||||||||||||||||||||||||||||||||||||| |||
Sbjct  894   GTACGGTCGCAAGACTGAAACTCAAAGGAATTGACGGGGACCCGCACAAGCAGTGGAGTA  953

Query  908   TGTGGTTTAATTCGAAGCAACGCGAAGAACCTTACCAAGTCTTGACATCCCTTTGACCGG  967
             ||||||||||||||||||||||||||||||||||||||||||||||||||||||||||||
Sbjct  954   TGTGGTTTAATTCGAAGCAACGCGAAGAACCTTACCAAGTCTTGACATCCCTTTGACCGG  1013

Query  968   ACTAGAGATAGTCTTTTCCTTCGGGACAAAGG  999
             ||||||||||||||||||||||||||||||||
Sbjct  1014  ACTAGAGATAGTCTTTTCCTTCGGGACAAAGG  1045
```

Download

FASTA (complete sequence)

FASTA (aligned sequences)

GenBank (complete sequence)

Continue
Cancel

GenBankGraphics

Next
Previous
Descriptions

Paenibacillus sp. strain PS-K-17 16S ribosomal RNA gene, partial sequence

Sequence ID: MF443394.1Length: 1462Number of Matches: 1

Related Information

Range 1: 6 to 996GenBankGraphics

Next Match
Previous Match
First Match

Alignment statistics for match #1

| Score | Expect | Identities | Gaps | Strand | Frame |
| --- | --- | --- | --- | --- | --- |
| 1801 bits(975) | 0.0() | 984/991(99%) | 0/991(0%) | Plus/Plus |  |

Features:

```
Query  9    GTCGAGCGGAGTTGANGAGAAGCTTGCTTCTTTGATGCTTAGCGGCGGACGGGTGAGTAA  68
            ||||||||||||||| |||||||||||||||  |||||||||||||||||||||||||||
Sbjct  6    GTCGAGCGGAGTTGAYGAGAAGCTTGCTTCTCGGATGCTTAGCGGCGGACGGGTGAGTAA  65

Query  69   CACGTAGGCAACCTGCCCTCAAGCTTGGGACAACTACCGGAAACGGTAGCTAATACCGAA  128
            ||||||||||||||||||||||||||||||||||||||||||||||||||||||||||||
Sbjct  66   CACGTAGGCAACCTGCCCTCAAGCTTGGGACAACTACCGGAAACGGTAGCTAATACCGAA  125

Query  129  TACATGATTTGTTCGCCTGAACGAATNTGGAAAGACGGAGCAATCTGTCACTTGAGGATG  188
            |||||||||||||||||||||||||| |||||||||||||||||||||||||||||||||
Sbjct  126  TACATGATTTGTTCGCCTGAACGAATTTGGAAAGACGGAGCAATCTGTCACTTGAGGATG  185

Query  189  GGCCTGCGGCGCATTAGCTAGTTGGTGAGGTAACGGCTCACCAAGGCGACGATGCGTAGC  248
            ||||||||||||||||||||||||||||||||||||||||||||||||||||||||||||
Sbjct  186  GGCCTGCGGCGCATTAGCTAGTTGGTGAGGTAACGGCTCACCAAGGCGACGATGCGTAGC  245

Query  249  CGACCTGAGAGGGTGATCGGCCACACTGGGACTGAGACACGGCCCAGACTCCTACGGGAG  308
            ||||||||||||||||||||||||||||||||||||||||||||||||||||||||||||
Sbjct  246  CGACCTGAGAGGGTGATCGGCCACACTGGGACTGAGACACGGCCCAGACTCCTACGGGAG  305

Query  309  GCAGCAGTAGGGAATCTTCCGCAATGGACGCAAGTCTGACGGAGCAACGCCGCGTGAGTG  368
            ||||||||||||||||||||||||||||||||||||||||||||||||||||||||||||
Sbjct  306  GCAGCAGTAGGGAATCTTCCGCAATGGACGCAAGTCTGACGGAGCAACGCCGCGTGAGTG  365

Query  369  ATGAAGGTTTTCGGATCGTAAAGCTCTGTTGCCAGGGAAGAACGTCNNATAGAGTAACTG  428
            ||||||||||||||||||||||||||||||||||||||||||||||  ||||||||||||
Sbjct  366  ATGAAGGTTTTCGGATCGTAAAGCTCTGTTGCCAGGGAAGAACGTCCGATAGAGTAACTG  425

Query  429  CTATCGGAGTGACGGTACCTGAGAAGAAAGCCCCGGCTAACTACGTGCCAGCAGCCGCGG  488
            ||||||||||||||||||||||||||||||||||||||||||||||||||||||||||||
Sbjct  426  CTATCGGAGTGACGGTACCTGAGAAGAAAGCCCCGGCTAACTACGTGCCAGCAGCCGCGG  485

Query  489  TAATACGTAGGGGGCAAGCGTTGTCCGGAATTATTGGGCGTAAAGCGCGCGCAGGCGGCT  548
            ||||||||||||||||||||||||||||||||||||||||||||||||||||||||||||
Sbjct  486  TAATACGTAGGGGGCAAGCGTTGTCCGGAATTATTGGGCGTAAAGCGCGCGCAGGCGGCT  545

Query  549  TTTTAAGTCCGGTGTCACAGCCCAAGGCTCAACCTTGGGTCGCACTGGAAACTGGAGAGC  608
            ||||||||||||||||||||||||||||||||||||||||||||||||||||||||||||
Sbjct  546  TTTTAAGTCCGGTGTCACAGCCCAAGGCTCAACCTTGGGTCGCACTGGAAACTGGAGAGC  605

Query  609  TTGAGTACAGAAGAGGAAAGTGGAATTCCACGTGTAGCGGTGAAATGCGTAGAGATGTGG  668
            ||||||||||||||||||||||||||||||||||||||||||||||||||||||||||||
Sbjct  606  TTGAGTACAGAAGAGGAAAGTGGAATTCCACGTGTAGCGGTGAAATGCGTAGAGATGTGG  665

Query  669  AGGAACACCAGTGGCGAAGGCGACTTTCTGGGCTGTAACTGACGCTGAGGCGCGAAAGCG  728
            ||||||||||||||||||||||||||||||||||||||||||||||||||||||||||||
Sbjct  666  AGGAACACCAGTGGCGAAGGCGACTTTCTGGGCTGTAACTGACGCTGAGGCGCGAAAGCG  725

Query  729  TGGGGAGCAAACAGGATTAGATACCCTGGTAGTCCACGCCGTAAACGATGAATGCTAGGT  788
            ||||||||||||||||||||||||||||||||||||||||||||||||||||||||||||
Sbjct  726  TGGGGAGCAAACAGGATTAGATACCCTGGTAGTCCACGCCGTAAACGATGAATGCTAGGT  785

Query  789  GTTAGGGGTTTCGATACCCTTGGTGCCGAAGTTAACACATTAAGCATTCCGCCTGGGGAG  848
            ||||||||||||||||||||||||||||||||||||||||||||||||||||||||||||
Sbjct  786  GTTAGGGGTTTCGATACCCTTGGTGCCGAAGTTAACACATTAAGCATTCCGCCTGGGGAG  845

Query  849  TACGGTCGCAAGACTGAAACTCAAAGGAATTGACGGGGACCCGCACAAGCAGTGGNGTAT  908
            ||||||||||||||||||||||||||||||||||||||||||||||||||||||| ||||
Sbjct  846  TACGGTCGCAAGACTGAAACTCAAAGGAATTGACGGGGACCCGCACAAGCAGTGGAGTAT  905

Query  909  GTGGTTTAATTCGAAGCAACGCGAAGAACCTTACCAAGTCTTGACATCCCTTTGACCGGA  968
            ||||||||||||||||||||||||||||||||||||||||||||||||||||||||||||
Sbjct  906  GTGGTTTAATTCGAAGCAACGCGAAGAACCTTACCAAGTCTTGACATCCCTTTGACCGGA  965

Query  969  CTAGAGATAGTCTTTTCCTTCGGGACAAAGG  999
            |||||||||||||||||||||||||||||||
Sbjct  966  CTAGAGATAGTCTTTTCCTTCGGGACAAAGG  996
```


BLAST is a registered trademark of the National Library of Medicine

Support center
Mailing list


YouTube

- National Library Of Medicine
- National Institutes Of Health
- U.S. Department of Health & Human Services
- USA.gov

### NCBI


National Center for Biotechnology Information,
 U.S. National Library of Medicine

8600 Rockville Pike,
Bethesda
 MD,
20894
USA

Policies and Guidelines
|
Contact


PreferencesTurn off

External link. Please review our privacy policy.
