# Supplementary material for: Isothermal microcalorimetry for thermal viable count of microorganisms in pure cultures and stabilized formulations
Source: BMC Microbiol. 2019 Mar 21;19:65. doi: 10.1186/s12866-019-1432-8 (PMC6429831; doi:10.1186/s12866-019-1432-8)
Supplement: Supplementary file 3 — 16S rRNA gene sequences’ BLASTN hits in zipped HTML format. (ZIP 15810 kb) [file 12866_2019_1432_MOESM3_ESM.zip › Best blastn hits/NCBI Blast_J pink R -- 32..1048 of sequence (1017.html]

NCBI Blast:J pink R -- 32..1048 of sequence (1017


- NCBI Home
- Sign in to NCBI
- Skip to Main Content
- Skip to Navigation
- About NCBI Accesskeys

U.S. National Library of Medicine

NCBI
National Center for Biotechnology Information

- My NCBI
- Sign in to NCBI
- Register
- Sign Out

BLAST ^®^ » blastn suite » RID-A4XVFYKX014


- Home
- Recent Results
- Saved Strategies
- Help

BLAST Results


Edit and Resubmit
Save Search Strategies
[Sign in above to save your search strategy]

Formatting options 


Download


How to read this page
Blast report description
Questions/comments


|  |  |
| --- | --- |
| Formatting options | |
| Show | Alignment as  HTML Plain text   Old View Reset form to defaults [?]  These options control formatting of alignments in results pages. The default is HTML, but other formats (including plain text) are available. PSSM and PssmWithParameters are representations of Position Specific Scoring Matrices and are only available for PSI-BLAST. The Advanced view option allows the database descriptions to be sorted by various indices in a table. |
| Alignment View | Pairwise Pairwise with dots for identities Query-anchored with dots for identities Query-anchored with letters for identities Flat query-anchored with dots for identities Flat query-anchored with letters for identities [?]  Choose how to view alignments. The default "pairwise" view shows how each subject sequence aligns individually to the query sequence. The "query-anchored" view shows how all subject sequences align to the query sequence. For each view type, you can choose to show "identities" (matching residues) as letters or dots. more... |
| Display | Graphical Overview   Linkout   Sequence Retrieval  NCBI-gi   CDS feature [?]  - Graphical Overview: Graphical Overview: Show graph of similar sequence regions aligned to query.   more... - NCBI-gi: Show NCBI gi identifiers. - CDS feature: Show annotated coding region and translation.   more... |
| Masking | Character:   X for protein, n for nucleotide Lower Case  Color:  Black Grey Red [?]  - Masking Character: Display masked (filtered) sequence regions as lower-case or as specific letters (N for nucleotide, P for protein). - Masking Color: Display masked sequence regions in the given color. |
| Limit results | Descriptions:  10 50 100 Graphical overview:  0 10 50 100  Alignments:  0 10 50 100 Line length:  60 90 120 150 [?]  - Descriptions: Show short descriptions for up to the given number of sequences. - Alignments: Show alignments for up to the given number of sequences, in order of statistical significance. - Line lenghth: Number of letters to show on one line in an alignment. |
|  | Organism Type common name, binomial, taxid, or group name. Only 20 top taxa will be shown.     Exclude   [?]  Show only sequences from the given organism. |
|  | Entrez query:  [?]  Show only those sequences that match the given Entrez query. more... |
|  | Expect Min:  Expect Max:  [?]  Show only sequences with expect values in the given range. more... |
|  | Percent Identity Min:  Percent Identity Max:  [?]  Show only sequences with percent identity values in the given range. |
| Format for | PSI-BLAST with inclusion threshold:  [?]  - Format for PSI-BLAST: The Position-Specific Iterated BLAST (PSI-BLAST) program performs iterative searches with a protein query,   in which sequences found in one round of search are used to build a custom score model for the next round.   more... - Inclusion Threshold: This sets the statistical significance threshold for including a sequence in the model used   by PSI-BLAST to create the PSSM on the next iteration. |

|  |  |  |  |  |  |
| --- | --- | --- | --- | --- | --- |
| Download | | | | | |
| Alignment  Text XML ASN.1 JSON Seq-align Hit Table(text) Hit Table(csv) Multiple-file XML2 Single-file XML2 Multiple-file JSON Single-file JSON SAM | Search Strategies  ASN.1 | PSSM to restart search  PSSM | [?] |  |  |

The Download link provides BLAST output that may be used as input to another program.
This includes parseable formats such as the tabular report or XML as well as the Search Strategy files read by the BLAST+ applications.
More details on the parseable (XML, tabular, and ASN.1) reports can be found at
https://www.ncbi.nlm.nih.gov/books/NBK153387/  
  

The following formats are offered under the Alignment section:  
1). "Text". Non-HTML standard BLAST report.  
2). "XML". XML report based upon the DTD at https://www.ncbi.nlm.nih.gov/data\_specs/dtd/NCBI\_BlastOutput.dtd  
3). "ASN.1". Alignment written out in Abstract Syntax Notation 1.  
4). "JSON Seq-align". Alignment written out in JSON.  
4). "Hit Table(text)". The tabular report as text.  
5). "Hit Table(csv)". The tabular report ready for import into spread-sheet programs like Excel.  
6). "XML2". New XML format described at ftp://ftp.ncbi.nlm.nih.gov/blast/documents/NEWXML/xml2.pdf.  
7). "JSON". New JSON format described at ftp://ftp.ncbi.nlm.nih.gov/blast/documents/NEWXML/xml2.pdf.  
8). "SAM". Sequence Alignment Map format.

XML2 and JSON can be downloaded either as one file per query (multiple-file) or one file for all queries (single-file). These formats are listed as Multiple-file XML2 (and JSON) or Single-file XML (and JSON).

The following report is offered under the Search Strategy section:  
1). "ASN.1" Search Strategy. A record of the parameters, query, and database used in the search. This file can be used to start a stand-alone BLAST search, see
https://www.ncbi.nlm.nih.gov/books/NBK1763/#CmdLineAppsManual.I455\_BLAST\_search\_stra


# Job title: J pink R -- 32..1048 of sequence (1017

Results for:

lcl|Query\_232325 J pink R -- 32..1048 of sequence(1017bp)
[?]

Your BLAST job specified more than one input sequence.
This box lets you choose which input sequence to show BLAST results for.

RID
:   A4XVFYKX014 (Expires on 03-10 19:00 pm)

Query ID
:   lcl|Query\_232325
:   lcl|Query\_232325

Description
:   J pink R -- 32..1048 of sequence

Molecule type
:   nucleic acid

Query Length
:   1017

Database Name
:   nr

Description
:   Nucleotide collection (nt) See details

Program
:   BLASTN 2.8.0+ Citation

  

Reference 

Zheng Zhang, Scott Schwartz, Lukas Wagner, and Webb Miller (2000), "A greedy algorithm for aligning DNA sequences", J Comput Biol 2000; 7(1-2):203-14.

Reference - database indexing

Aleksandr Morgulis, George Coulouris, Yan Raytselis, Thomas L. Madden, Richa Agarwala, Alejandro A. Schäffer (2008), "Database Indexing for Production MegaBLAST Searches", Bioinformatics 24:1757-1764.

Other reports:
Search Summary

[Taxonomy reports]
[Distance tree of results]
[MSA viewer]

Search Parameters

| Search parameter name | Search parameter value |
| --- | --- |
| Program | blastn |
| Word size | 28 |
| Expect value | 10 |
| Hitlist size | 100 |
| Match/Mismatch scores | 1,-2 |
| Gapcosts | 0,2.5 |
| Low Complexity Filter | Yes |
| Filter string | L;m; |
| Genetic Code | 1 |

Database

| Database parameter name | Database parameter value |
| --- | --- |
| Posted date | Mar 3, 2018 9:32 PM |
| Number of letters | 171,598,911,005 |
| Number of sequences | 46,870,701 |
| Entrez query | Includes:  Excludes:  None |

Karlin-Altschul statistics

| Params | Ungapped | Gapped |
| --- | --- | --- |
| Lambda | 1.33271 | 1.28 |
| K | 0.620991 | 0.46 |
| H | 1.12409 | 0.85 |

Results Statistics

| Results Statistics parameter name | Results Statistics parameter value |
| --- | --- |
| Length adjustment | 35 |
| Effective length of query | 982 |
| Effective length of database | 169958436470 |
| Effective search space | 166899184613540 |
| Effective search space used | 166899184613540 |


## Graphic Summary

### Distribution of the top 199 Blast Hits on 100 subject sequences [?]

The graphic is an overview of the database sequences aligned to the query sequence. These are represented horizontal bars colored coded by score and showing the extent
of the alignment on the query sequence. Separate aligned regions on the same database sequence are connected by a thin grey line.
Mousing over an alignment shows the database sequence title. Clicking an alignment displays a box with more details about the alignment and
link to the sequence alignment itself in the Alignments section of the report.

Mouse over to see the title, click to show alignments

Color key for alignment scores

<40

40-50

50-80

80-200

>=200

Query

1

200

400

600

800

1000

Paenibacillus sp. strain PS-K-17 16S ribosomal RNA gene..

Score:1855 Evalue:0

Accession:MF443394.1

Alignment

Paenibacillus sp. 3Cp1 16S ribosomal RNA gene, partial ..

Score:1855 Evalue:0

Accession:KP852522.1

Alignment

Uncultured bacterium clone 4L13 16S ribosomal RNA gene,..

Score:1855 Evalue:0

Accession:KP843054.1

Alignment

Paenibacillus sp. TI45-13ar 16S ribosomal RNA gene, par..

Score:1849 Evalue:0

Accession:KY419705.1

Alignment

Bacterium 4R9 16S ribosomal RNA gene, partial sequence

Score:1844 Evalue:0

Accession:KP965575.1

Alignment

Paenibacillus illinoisensis strain WR\_17 16S ribosomal ..

Score:1838 Evalue:0

Accession:HQ661869.1

Alignment

Paenibacillus sp. 9N4 16S ribosomal RNA gene, partial s..

Score:1831 Evalue:0

Accession:HQ284889.1

Alignment

Paenibacillus sp. strain BSW35 16S ribosomal RNA gene, ..

Score:1827 Evalue:0

Accession:KY446062.1

Alignment

Paenibacillus sp. Rs5 16S ribosomal RNA gene, partial s..

Score:1827 Evalue:0

Accession:JQ977193.1

Alignment

Paenibacillus sp. Q1-R10 16S ribosomal RNA gene, partia..

Score:1810 Evalue:0

Accession:JX994123.1

Alignment

Bacterium 4R1 16S ribosomal RNA gene, partial sequence

Score:1805 Evalue:0

Accession:KP965572.1

Alignment

Paenibacillus sp. HDDMM03 16S ribosomal RNA gene, parti..

Score:1805 Evalue:0

Accession:EU723825.1

Alignment

Uncultured bacterium clone 10-9BM 16S ribosomal RNA gen..

Score:1799 Evalue:0

Accession:HQ143281.1

Alignment

Bacterium 4L18 16S ribosomal RNA gene, partial sequence

Score:1796 Evalue:0

Accession:KP965574.1

Alignment

Bacterium 4L13 16S ribosomal RNA gene, partial sequence

Score:1796 Evalue:0

Accession:KP965573.1

Alignment

Paenibacillus sp. NCCB 100172 16S ribosomal RNA gene, p..

Score:1788 Evalue:0

Accession:EU003588.1

Alignment

Paenibacillus nicotianae strain YIM h-19 16S ribosomal ..

Score:1777 Evalue:0

Accession:NR\_134783.2

Alignment

Paenibacillus nicotianae strain YIM h-19 16S ribosomal ..

Score:1777 Evalue:0

Accession:KJ792471.1

Alignment

Paenibacillus sp. R1 16S ribosomal RNA gene, partial se..

Score:1773 Evalue:0

Accession:KR263165.1

Alignment

Paenibacillus kyungheensis strain DCY88 16S ribosomal R..

Score:1764 Evalue:0

Accession:NR\_145628.1

Alignment

Paenibacillus kyungheensis strain DCY88 16S ribosomal R..

Score:1764 Evalue:0

Accession:KF793934.1

Alignment

Paenibacillus hordei strain RH-N24 16S ribosomal RNA ge..

Score:1748 Evalue:0

Accession:NR\_109318.1

Alignment

Uncultured bacterium clone ncd2164d05c1 16S ribosomal R..

Score:1714 Evalue:0

Accession:JF185937.1

Alignment

Uncultured bacterium clone ncd2162h02c1 16S ribosomal R..

Score:1714 Evalue:0

Accession:JF185833.1

Alignment

Uncultured bacterium clone ncd2163a03c1 16S ribosomal R..

Score:1709 Evalue:0

Accession:JF185844.1

Alignment

Uncultured bacterium clone nbw208h09c1 16S ribosomal RN..

Score:1698 Evalue:0

Accession:GQ073426.1

Alignment

Paenibacillus kyungheensis strain R2abR 16S ribosomal R..

Score:1696 Evalue:0

Accession:MG607391.1

Alignment

Paenibacillus sp. TA\_EU partial 16S rRNA gene, strain T..

Score:1696 Evalue:0

Accession:HG942127.1

Alignment

Uncultured bacterium clone 56-9BM 16S ribosomal RNA gen..

Score:1690 Evalue:0

Accession:HQ143315.1

Alignment

Uncultured bacterium clone HR-R2W6B52 16S ribosomal RNA..

Score:1672 Evalue:0

Accession:KM507259.1

Alignment

Paenibacillus peoriae strain DSM 8320 16S ribosomal RNA..

Score:1655 Evalue:0

Accession:NR\_117740.1

Alignment

Paenibacillus polymyxa strain J, complete genome

Score:1650 Evalue:0

Accession:CP015423.1

Alignment

Paenibacillus polymyxa partial 16S rRNA gene, strain KC..

Score:1648 Evalue:0

Accession:HE981768.1

Alignment

Paenibacillus sp. strain Avl-2 16S ribosomal RNA gene, ..

Score:1644 Evalue:0

Accession:KX907738.1

Alignment

Paenibacillus peoriae strain DSM 8320 16S ribosomal RNA..

Score:1644 Evalue:0

Accession:NR\_117739.1

Alignment

Paenibacillus brasilensis strain 136 16S ribosomal RNA ..

Score:1644 Evalue:0

Accession:JQ860094.1

Alignment

Paenibacillus polymyxa strain DSM 36 16S ribosomal RNA,..

Score:1642 Evalue:0

Accession:NR\_117727.2

Alignment

Paenibacillus polymyxa strain DSM 36 16S ribosomal RNA,..

Score:1642 Evalue:0

Accession:NR\_117726.2

Alignment

Uncultured Paenibacillus sp. clone DDGJ01 16S ribosomal..

Score:1642 Evalue:0

Accession:KR095605.1

Alignment

Paenibacillus polymyxa partial 16S rRNA gene, type stra..

Score:1642 Evalue:0

Accession:HG324071.1

Alignment

Paenibacillus polymyxa partial 16S rRNA gene, type stra..

Score:1642 Evalue:0

Accession:HG324070.1

Alignment

Paenibacillus jamilae partial 16S rRNA gene, strain KCT..

Score:1642 Evalue:0

Accession:HE981796.1

Alignment

Paenibacillus polymyxa partial 16S rRNA gene, strain KC..

Score:1642 Evalue:0

Accession:HE981788.1

Alignment

Paenibacillus polymyxa strain HX-140 16S ribosomal RNA ..

Score:1639 Evalue:0

Accession:MF136611.1

Alignment

Paenibacillus sp. strain YN-3 16S ribosomal RNA gene, p..

Score:1639 Evalue:0

Accession:MF967298.1

Alignment

Paenibacillus sp. strain BJ-7 16S ribosomal RNA gene, p..

Score:1639 Evalue:0

Accession:MF967288.1

Alignment

Paenibacillus peoriae strain GQJK57 16S ribosomal RNA g..

Score:1639 Evalue:0

Accession:KY952711.1

Alignment

Paenibacillus polymyxa strain TS683 16S ribosomal RNA g..

Score:1639 Evalue:0

Accession:KU860093.1

Alignment

Paenibacillus polymyxa strain TS682 16S ribosomal RNA g..

Score:1639 Evalue:0

Accession:KU860092.1

Alignment

Paenibacillus polymyxa strain TS681 16S ribosomal RNA g..

Score:1639 Evalue:0

Accession:KU860091.1

Alignment

Paenibacillus polymyxa strain ATCC 15970, complete geno..

Score:1639 Evalue:0

Accession:CP011420.1

Alignment

Paenibacillus polymyxa strain JK1 16S ribosomal RNA gen..

Score:1639 Evalue:0

Accession:KU563771.1

Alignment

Paenibacillus polymyxa partial 16S rRNA gene, strain Ma..

Score:1639 Evalue:0

Accession:LT223607.1

Alignment

Paenibacillus polymyxa strain J 16S ribosomal RNA gene,..

Score:1639 Evalue:0

Accession:KT783525.1

Alignment

Paenibacillus polymyxa strain LY214 16S ribosomal RNA g..

Score:1639 Evalue:0

Accession:KR058350.1

Alignment

Paenibacillus peoriae strain IHB B 12520 16S ribosomal ..

Score:1639 Evalue:0

Accession:KJ767388.1

Alignment

## Descriptions

, Reading indexes 1-5, displaying indexes 1-5


Load next setPrevious Match

Sequences producing significant alignments:

Show all columns  of the table presenting sequences producing significant alignments 

Select:AllNone
Selected:0

Alignments
Download

FASTA (complete sequence)

FASTA (aligned sequences)

GenBank (complete sequence)

Hit Table (text)

Hit Table (CSV)

Text

XML

ASN.1

Continue
Cancel

GenBank 
Graphics
Distance tree of results
Multiple alignment
Show/hide columns of the table presenting sequences producing significant alignments 

Available columns

Description  
Max Score  
Total Score  
Coverage  
E-value  
IdentN  
Accession  
Restore Defaults
Ok
Cancel

Sequences producing significant alignments:

| Select for downloading or viewing reports | Description | Max score | Total score | Query cover | E value | Ident | Accession |
| --- | --- | --- | --- | --- | --- | --- | --- |
| 1Select seq MF443394.1 | Paenibacillus sp. strain PS-K-17 16S ribosomal RNA gene, partial sequence | 1855 | 1855 | 98% | 0.0 | 100% | MF443394.1 |
| 2Select seq KP852522.1 | Paenibacillus sp. 3Cp1 16S ribosomal RNA gene, partial sequence | 1855 | 1855 | 98% | 0.0 | 100% | KP852522.1 |
| 3Select seq KP843054.1 | Uncultured bacterium clone 4L13 16S ribosomal RNA gene, partial sequence | 1855 | 1855 | 98% | 0.0 | 100% | KP843054.1 |
| 4Select seq KY419705.1 | Paenibacillus sp. TI45-13ar 16S ribosomal RNA gene, partial sequence | 1849 | 1849 | 98% | 0.0 | 99% | KY419705.1 |
| 5Select seq KP965575.1 | Bacterium 4R9 16S ribosomal RNA gene, partial sequence | 1844 | 1844 | 98% | 0.0 | 99% | KP965575.1 |
| 6Select seq HQ661869.1 | Paenibacillus illinoisensis strain WR\_17 16S ribosomal RNA gene, partial sequence | 1838 | 1838 | 98% | 0.0 | 99% | HQ661869.1 |
| 7Select seq HQ284889.1 | Paenibacillus sp. 9N4 16S ribosomal RNA gene, partial sequence | 1831 | 1831 | 98% | 0.0 | 99% | HQ284889.1 |
| 8Select seq KY446062.1 | Paenibacillus sp. strain BSW35 16S ribosomal RNA gene, partial sequence | 1827 | 1827 | 98% | 0.0 | 99% | KY446062.1 |
| 9Select seq JQ977193.1 | Paenibacillus sp. Rs5 16S ribosomal RNA gene, partial sequence | 1827 | 1827 | 98% | 0.0 | 99% | JQ977193.1 |
| 10Select seq JX994123.1 | Paenibacillus sp. Q1-R10 16S ribosomal RNA gene, partial sequence | 1810 | 1810 | 98% | 0.0 | 99% | JX994123.1 |
| 11Select seq KP965572.1 | Bacterium 4R1 16S ribosomal RNA gene, partial sequence | 1805 | 1805 | 98% | 0.0 | 99% | KP965572.1 |
| 12Select seq EU723825.1 | Paenibacillus sp. HDDMM03 16S ribosomal RNA gene, partial sequence | 1805 | 1805 | 98% | 0.0 | 99% | EU723825.1 |
| 13Select seq HQ143281.1 | Uncultured bacterium clone 10-9BM 16S ribosomal RNA gene, partial sequence | 1799 | 1799 | 98% | 0.0 | 99% | HQ143281.1 |
| 14Select seq KP965574.1 | Bacterium 4L18 16S ribosomal RNA gene, partial sequence | 1796 | 1796 | 98% | 0.0 | 99% | KP965574.1 |
| 15Select seq KP965573.1 | Bacterium 4L13 16S ribosomal RNA gene, partial sequence | 1796 | 1796 | 98% | 0.0 | 99% | KP965573.1 |
| 16Select seq EU003588.1 | Paenibacillus sp. NCCB 100172 16S ribosomal RNA gene, partial sequence | 1788 | 1788 | 98% | 0.0 | 99% | EU003588.1 |
| 17Select seq NR\_134783.2 | Paenibacillus nicotianae strain YIM h-19 16S ribosomal RNA, complete sequence | 1777 | 1777 | 98% | 0.0 | 99% | NR\_134783.2 |
| 18Select seq KJ792471.1 | Paenibacillus nicotianae strain YIM h-19 16S ribosomal RNA gene, partial sequence | 1777 | 1777 | 98% | 0.0 | 99% | KJ792471.1 |
| 19Select seq KR263165.1 | Paenibacillus sp. R1 16S ribosomal RNA gene, partial sequence | 1773 | 1773 | 98% | 0.0 | 98% | KR263165.1 |
| 20Select seq NR\_145628.1 | Paenibacillus kyungheensis strain DCY88 16S ribosomal RNA, partial sequence | 1764 | 1764 | 98% | 0.0 | 98% | NR\_145628.1 |
| 21Select seq KF793934.1 | Paenibacillus kyungheensis strain DCY88 16S ribosomal RNA gene, partial sequence | 1764 | 1764 | 98% | 0.0 | 98% | KF793934.1 |
| 22Select seq NR\_109318.1 | Paenibacillus hordei strain RH-N24 16S ribosomal RNA gene, partial sequence | 1748 | 1748 | 98% | 0.0 | 98% | NR\_109318.1 |
| 23Select seq JF185937.1 | Uncultured bacterium clone ncd2164d05c1 16S ribosomal RNA gene, partial sequence | 1714 | 1714 | 93% | 0.0 | 99% | JF185937.1 |
| 24Select seq JF185833.1 | Uncultured bacterium clone ncd2162h02c1 16S ribosomal RNA gene, partial sequence | 1714 | 1714 | 93% | 0.0 | 99% | JF185833.1 |
| 25Select seq JF185844.1 | Uncultured bacterium clone ncd2163a03c1 16S ribosomal RNA gene, partial sequence | 1709 | 1709 | 93% | 0.0 | 99% | JF185844.1 |
| 26Select seq GQ073426.1 | Uncultured bacterium clone nbw208h09c1 16S ribosomal RNA gene, partial sequence | 1698 | 1698 | 93% | 0.0 | 99% | GQ073426.1 |
| 27Select seq MG607391.1 | Paenibacillus kyungheensis strain R2abR 16S ribosomal RNA gene, partial sequence | 1696 | 1696 | 90% | 0.0 | 100% | MG607391.1 |
| 28Select seq HG942127.1 | Paenibacillus sp. TA\_EU partial 16S rRNA gene, strain TA\_EU | 1696 | 1696 | 90% | 0.0 | 100% | HG942127.1 |
| 29Select seq HQ143315.1 | Uncultured bacterium clone 56-9BM 16S ribosomal RNA gene, partial sequence | 1690 | 1690 | 94% | 0.0 | 99% | HQ143315.1 |
| 30Select seq KM507259.1 | Uncultured bacterium clone HR-R2W6B52 16S ribosomal RNA gene, partial sequence | 1672 | 1672 | 89% | 0.0 | 99% | KM507259.1 |
| 31Select seq NR\_117740.1 | Paenibacillus peoriae strain DSM 8320 16S ribosomal RNA gene, partial sequence | 1655 | 1655 | 98% | 0.0 | 96% | NR\_117740.1 |
| 32Select seq CP015423.1 | Paenibacillus polymyxa strain J, complete genome | 1650 | 22936 | 98% | 0.0 | 96% | CP015423.1 |
| 33Select seq HE981768.1 | Paenibacillus polymyxa partial 16S rRNA gene, strain KCTC 3627, clone 3 | 1648 | 1648 | 98% | 0.0 | 96% | HE981768.1 |
| 34Select seq KX907738.1 | Paenibacillus sp. strain Avl-2 16S ribosomal RNA gene, partial sequence | 1644 | 1644 | 98% | 0.0 | 96% | KX907738.1 |
| 35Select seq NR\_117739.1 | Paenibacillus peoriae strain DSM 8320 16S ribosomal RNA gene, partial sequence | 1644 | 1644 | 98% | 0.0 | 96% | NR\_117739.1 |
| 36Select seq JQ860094.1 | Paenibacillus brasilensis strain 136 16S ribosomal RNA gene, partial sequence | 1644 | 1644 | 98% | 0.0 | 96% | JQ860094.1 |
| 37Select seq NR\_117727.2 | Paenibacillus polymyxa strain DSM 36 16S ribosomal RNA, partial sequence | 1642 | 1642 | 98% | 0.0 | 96% | NR\_117727.2 |
| 38Select seq NR\_117726.2 | Paenibacillus polymyxa strain DSM 36 16S ribosomal RNA, partial sequence | 1642 | 1642 | 98% | 0.0 | 96% | NR\_117726.2 |
| 39Select seq KR095605.1 | Uncultured Paenibacillus sp. clone DDGJ01 16S ribosomal RNA gene, partial sequence | 1642 | 1642 | 98% | 0.0 | 96% | KR095605.1 |
| 40Select seq HG324071.1 | Paenibacillus polymyxa partial 16S rRNA gene, type strain DSM 36T, clone 8 | 1642 | 1642 | 98% | 0.0 | 96% | HG324071.1 |
| 41Select seq HG324070.1 | Paenibacillus polymyxa partial 16S rRNA gene, type strain DSM 36T, clone 7 | 1642 | 1642 | 98% | 0.0 | 96% | HG324070.1 |
| 42Select seq HE981796.1 | Paenibacillus jamilae partial 16S rRNA gene, strain KCTC 13919, clone 4 | 1642 | 1642 | 98% | 0.0 | 96% | HE981796.1 |
| 43Select seq HE981788.1 | Paenibacillus polymyxa partial 16S rRNA gene, strain KCTC 3627, clone 23 | 1642 | 1642 | 98% | 0.0 | 96% | HE981788.1 |
| 44Select seq MF136611.1 | Paenibacillus polymyxa strain HX-140 16S ribosomal RNA gene, partial sequence | 1639 | 1639 | 98% | 0.0 | 96% | MF136611.1 |
| 45Select seq MF967298.1 | Paenibacillus sp. strain YN-3 16S ribosomal RNA gene, partial sequence | 1639 | 1639 | 98% | 0.0 | 96% | MF967298.1 |
| 46Select seq MF967288.1 | Paenibacillus sp. strain BJ-7 16S ribosomal RNA gene, partial sequence | 1639 | 1639 | 98% | 0.0 | 96% | MF967288.1 |
| 47Select seq KY952711.1 | Paenibacillus peoriae strain GQJK57 16S ribosomal RNA gene, partial sequence | 1639 | 1639 | 98% | 0.0 | 96% | KY952711.1 |
| 48Select seq KU860093.1 | Paenibacillus polymyxa strain TS683 16S ribosomal RNA gene, partial sequence | 1639 | 1639 | 98% | 0.0 | 96% | KU860093.1 |
| 49Select seq KU860092.1 | Paenibacillus polymyxa strain TS682 16S ribosomal RNA gene, partial sequence | 1639 | 1639 | 98% | 0.0 | 96% | KU860092.1 |
| 50Select seq KU860091.1 | Paenibacillus polymyxa strain TS681 16S ribosomal RNA gene, partial sequence | 1639 | 1639 | 98% | 0.0 | 96% | KU860091.1 |
| 51Select seq CP011420.1 | Paenibacillus polymyxa strain ATCC 15970, complete genome | 1639 | 21225 | 98% | 0.0 | 96% | CP011420.1 |
| 52Select seq KU563771.1 | Paenibacillus polymyxa strain JK1 16S ribosomal RNA gene, partial sequence | 1639 | 1639 | 98% | 0.0 | 96% | KU563771.1 |
| 53Select seq LT223607.1 | Paenibacillus polymyxa partial 16S rRNA gene, strain Marseille-AA00092 | 1639 | 1639 | 98% | 0.0 | 96% | LT223607.1 |
| 54Select seq KT783525.1 | Paenibacillus polymyxa strain J 16S ribosomal RNA gene, partial sequence | 1639 | 1639 | 98% | 0.0 | 96% | KT783525.1 |
| 55Select seq KR058350.1 | Paenibacillus polymyxa strain LY214 16S ribosomal RNA gene, partial sequence | 1639 | 1639 | 98% | 0.0 | 96% | KR058350.1 |
| 56Select seq KJ767388.1 | Paenibacillus peoriae strain IHB B 12520 16S ribosomal RNA gene, partial sequence | 1639 | 1639 | 98% | 0.0 | 96% | KJ767388.1 |
| 57Select seq KF441710.1 | Paenibacillus sp. 7B-896 16S ribosomal RNA gene, partial sequence | 1639 | 1639 | 98% | 0.0 | 96% | KF441710.1 |
| 58Select seq KC581713.1 | Paenibacillus sp. LXfos16 16S ribosomal RNA gene, partial sequence | 1639 | 1639 | 98% | 0.0 | 96% | KC581713.1 |
| 59Select seq KC236521.1 | Paenibacillus sp. 2083 16S ribosomal RNA gene, partial sequence | 1639 | 1639 | 98% | 0.0 | 96% | KC236521.1 |
| 60Select seq JX566596.1 | Paenibacillus sp. 4129 16S ribosomal RNA gene, partial sequence | 1639 | 1639 | 98% | 0.0 | 96% | JX566596.1 |
| 61Select seq JX517259.1 | Paenibacillus polymyxa strain A1-25c-13 16S ribosomal RNA gene, partial sequence | 1639 | 1639 | 98% | 0.0 | 96% | JX517259.1 |
| 62Select seq JN409465.1 | Paenibacillus polymyxa strain SP16 16S ribosomal RNA gene, partial sequence | 1639 | 1639 | 98% | 0.0 | 96% | JN409465.1 |
| 63Select seq HQ844466.1 | Paenibacillus peoriae strain HLJFQ25 16S ribosomal RNA gene, partial sequence | 1639 | 1639 | 98% | 0.0 | 96% | HQ844466.1 |
| 64Select seq JF747221.1 | Paenibacillus polymyxa strain S 16S ribosomal RNA gene, partial sequence | 1639 | 1639 | 98% | 0.0 | 96% | JF747221.1 |
| 65Select seq JF683620.1 | Paenibacillus polymyxa strain RS-10 16S ribosomal RNA gene, partial sequence | 1639 | 1639 | 98% | 0.0 | 96% | JF683620.1 |
| 66Select seq GU328691.1 | Paenibacillus sp. Sx52 16S ribosomal RNA gene, partial sequence | 1639 | 1639 | 98% | 0.0 | 96% | GU328691.1 |
| 67Select seq EU362611.1 | Paenibacillus polymyxa isolate TN99 16S ribosomal RNA gene, partial sequence | 1639 | 1639 | 98% | 0.0 | 96% | EU362611.1 |
| 68Select seq EU362610.1 | Paenibacillus polymyxa isolate TN98 16S ribosomal RNA gene, partial sequence | 1639 | 1639 | 98% | 0.0 | 96% | EU362610.1 |
| 69Select seq AM900509.1 | Paenibacillus sp. 14A-7 partial 16S rRNA gene, strain 14A-7 | 1639 | 1639 | 98% | 0.0 | 96% | AM900509.1 |
| 70Select seq EF634024.1 | Paenibacillus polymyxa strain ISSDS-851 16S ribosomal RNA gene, partial sequence | 1639 | 1639 | 98% | 0.0 | 96% | EF634024.1 |
| 71Select seq EF620468.1 | Paenibacillus polymyxa strain ISSDS-792 16S ribosomal RNA gene, partial sequence | 1639 | 1639 | 98% | 0.0 | 96% | EF620468.1 |
| 72Select seq DQ358734.1 | Paenibacillus sp. GA5 16S ribosomal RNA gene, partial sequence | 1639 | 1639 | 98% | 0.0 | 96% | DQ358734.1 |
| 73Select seq KY713250.1 | Paenibacillus sp. strain UFLA01-829 16S ribosomal RNA gene, partial sequence | 1637 | 1637 | 98% | 0.0 | 96% | KY713250.1 |
| 74Select seq NR\_117732.2 | Paenibacillus polymyxa strain DSM 36 16S ribosomal RNA, partial sequence | 1637 | 1637 | 98% | 0.0 | 96% | NR\_117732.2 |
| 75Select seq KR095625.1 | Uncultured Paenibacillus sp. clone DDEN02 16S ribosomal RNA gene, partial sequence | 1637 | 1637 | 98% | 0.0 | 96% | KR095625.1 |
| 76Select seq KR095614.1 | Uncultured Paenibacillus sp. clone DDGJ10 16S ribosomal RNA gene, partial sequence | 1637 | 1637 | 98% | 0.0 | 96% | KR095614.1 |
| 77Select seq KR095606.1 | Uncultured Paenibacillus sp. clone DDGJ02 16S ribosomal RNA gene, partial sequence | 1637 | 1637 | 98% | 0.0 | 96% | KR095606.1 |
| 78Select seq CP002213.2 | Paenibacillus polymyxa SC2, complete genome | 1637 | 21140 | 98% | 0.0 | 96% | CP002213.2 |
| 79Select seq CP010268.1 | Paenibacillus polymyxa strain Sb3-1, complete genome | 1637 | 22777 | 98% | 0.0 | 96% | CP010268.1 |
| 80Select seq CP009909.1 | Paenibacillus polymyxa strain CF05 genome | 1637 | 22801 | 98% | 0.0 | 96% | CP009909.1 |
| 81Select seq CP006872.1 | Paenibacillus polymyxa SQR-21, complete genome | 1637 | 20920 | 98% | 0.0 | 96% | CP006872.1 |
| 82Select seq HG324076.1 | Paenibacillus polymyxa partial 16S rRNA gene, type strain DSM 36T, clone 13 | 1637 | 1637 | 98% | 0.0 | 96% | HG324076.1 |
| 83Select seq JX498909.1 | Paenibacillus jamilae strain NBI 16S ribosomal RNA gene, partial sequence | 1637 | 1637 | 98% | 0.0 | 96% | JX498909.1 |
| 84Select seq HE981792.1 | Paenibacillus polymyxa partial 16S rRNA gene, strain KCTC 3627, clone 27 | 1637 | 1637 | 98% | 0.0 | 96% | HE981792.1 |
| 85Select seq HE981790.1 | Paenibacillus polymyxa partial 16S rRNA gene, strain KCTC 3627, clone 25 | 1637 | 1637 | 98% | 0.0 | 96% | HE981790.1 |
| 86Select seq HE981782.1 | Paenibacillus polymyxa partial 16S rRNA gene, strain KCTC 3627, clone 17 | 1637 | 1637 | 98% | 0.0 | 96% | HE981782.1 |
| 87Select seq HE981780.1 | Paenibacillus polymyxa partial 16S rRNA gene, strain KCTC 3627, clone 15 | 1637 | 1637 | 98% | 0.0 | 96% | HE981780.1 |
| 88Select seq HE981777.1 | Paenibacillus polymyxa partial 16S rRNA gene, strain KCTC 3627, clone 12 | 1637 | 1637 | 98% | 0.0 | 96% | HE981777.1 |
| 89Select seq HE981776.1 | Paenibacillus polymyxa partial 16S rRNA gene, strain KCTC 3627, clone 11 | 1637 | 1637 | 98% | 0.0 | 96% | HE981776.1 |
| 90Select seq AB689757.1 | Paenibacillus polymyxa gene for 16S ribosomal RNA, partial sequence | 1637 | 1637 | 98% | 0.0 | 96% | AB689757.1 |
| 91Select seq HE577054.1 | Paenibacillus polymyxa M1 main chromosome, complete genome | 1637 | 22794 | 98% | 0.0 | 96% | HE577054.1 |
| 92Select seq FR727737.1 | Paenibacillus polymyxa partial 16S rRNA gene, strain M1 | 1637 | 1637 | 98% | 0.0 | 96% | FR727737.1 |
| 93Select seq KU312055.1 | Bacillus sp. JZ212 16S ribosomal RNA gene, partial sequence | 1635 | 1635 | 98% | 0.0 | 96% | KU312055.1 |
| 94Select seq HQ844465.1 | Paenibacillus brasilensis strain HLJFQ23 16S ribosomal RNA gene, partial sequence | 1635 | 1635 | 98% | 0.0 | 96% | HQ844465.1 |
| 95Select seq EF620470.1 | Paenibacillus polymyxa strain ISSDS-796 16S ribosomal RNA gene, partial sequence | 1635 | 1635 | 98% | 0.0 | 96% | EF620470.1 |
| 96Select seq EF108320.1 | Paenibacillus polymyxa strain DY1 16S ribosomal RNA gene, partial sequence | 1635 | 1635 | 98% | 0.0 | 96% | EF108320.1 |
| 97Select seq CP017968.3 | Paenibacillus polymyxa strain YC0573 chromosome, complete genome | 1633 | 19569 | 98% | 0.0 | 96% | CP017968.3 |
| 98Select seq AB689755.1 | Paenibacillus polymyxa gene for 16S ribosomal RNA, partial sequence | 1633 | 1633 | 98% | 0.0 | 96% | AB689755.1 |
| 99Select seq JQ279510.1 | Paenibacillus polymyxa strain AB15 16S ribosomal RNA gene, partial sequence | 1631 | 1631 | 98% | 0.0 | 96% | JQ279510.1 |
| 100Select seq EU982498.1 | Paenibacillus polymyxa strain 1170 16S ribosomal RNA gene, partial sequence | 1629 | 1629 | 98% | 0.0 | 96% | EU982498.1 |


## Alignments

Loading alignment... for sequences gi|1214741488,gi|915515201,gi|915512955,gi|1127353198,gi|846924131 Reading indexes 1-5

Download

FASTA (complete sequence)

FASTA (aligned sequences)

GenBank (complete sequence)

Continue
Cancel

GenBankGraphics

Next
Previous
Descriptions

Paenibacillus sp. strain PS-K-17 16S ribosomal RNA gene, partial sequence

Sequence ID: MF443394.1Length: 1462Number of Matches: 1

Related Information

Range 1: 395 to 1398GenBankGraphics

Next Match
Previous Match
First Match

Alignment statistics for match #1

| Score | Expect | Identities | Gaps | Strand | Frame |
| --- | --- | --- | --- | --- | --- |
| 1855 bits(1004) | 0.0() | 1004/1004(100%) | 0/1004(0%) | Plus/Minus |  |

Features:

```
Query  10    CCACCGACTTCGGGTGTTGCAAACTCTCGTGGTGTGACGGGCGGTGTGTACAAGACCCGG  69
             ||||||||||||||||||||||||||||||||||||||||||||||||||||||||||||
Sbjct  1398  CCACCGACTTCGGGTGTTGCAAACTCTCGTGGTGTGACGGGCGGTGTGTACAAGACCCGG  1339

Query  70    GAACGTATTCACCGCGGCATGCTGATCCGCGATTACTAGCAATTCCGACTTCATGCAGGC  129
             ||||||||||||||||||||||||||||||||||||||||||||||||||||||||||||
Sbjct  1338  GAACGTATTCACCGCGGCATGCTGATCCGCGATTACTAGCAATTCCGACTTCATGCAGGC  1279

Query  130   GAGTTGCAGCCTGCAATCCGAACTGAGACCGGCTTTTAAGGATTGGCTCCATCTTGCGAT  189
             ||||||||||||||||||||||||||||||||||||||||||||||||||||||||||||
Sbjct  1278  GAGTTGCAGCCTGCAATCCGAACTGAGACCGGCTTTTAAGGATTGGCTCCATCTTGCGAT  1219

Query  190   ATTGCTTCCCGTTGTACCGGCCATTGTAGTACGTGTGTAGCCCAAGTCATAAGGGGCATG  249
             ||||||||||||||||||||||||||||||||||||||||||||||||||||||||||||
Sbjct  1218  ATTGCTTCCCGTTGTACCGGCCATTGTAGTACGTGTGTAGCCCAAGTCATAAGGGGCATG  1159

Query  250   ATGATTTGACGTCATCCCCACCTTCCTCCGGTTTGTCACCGGCAGTCTGCTTAGAGTGCC  309
             ||||||||||||||||||||||||||||||||||||||||||||||||||||||||||||
Sbjct  1158  ATGATTTGACGTCATCCCCACCTTCCTCCGGTTTGTCACCGGCAGTCTGCTTAGAGTGCC  1099

Query  310   CACCATGATGTGCTGGCAACTAAGCATAAGGGTTGCGCTCGTTGCGGGACTTAACCCAAC  369
             ||||||||||||||||||||||||||||||||||||||||||||||||||||||||||||
Sbjct  1098  CACCATGATGTGCTGGCAACTAAGCATAAGGGTTGCGCTCGTTGCGGGACTTAACCCAAC  1039

Query  370   ATCTCACGACACGAGCTGACGACAACCATGCACCACCTGTCTCCTTTGTCCCGAAGGAAA  429
             ||||||||||||||||||||||||||||||||||||||||||||||||||||||||||||
Sbjct  1038  ATCTCACGACACGAGCTGACGACAACCATGCACCACCTGTCTCCTTTGTCCCGAAGGAAA  979

Query  430   AGACTATCTCTAGTCCGGTCAAAGGGATGTCAAGACTTGGTAAGGTTCTTCGCGTTGCTT  489
             ||||||||||||||||||||||||||||||||||||||||||||||||||||||||||||
Sbjct  978   AGACTATCTCTAGTCCGGTCAAAGGGATGTCAAGACTTGGTAAGGTTCTTCGCGTTGCTT  919

Query  490   CGAATTAAACCACATACTCCACTGCTTGTGCGGGTCCCCGTCAATTCCTTTGAGTTTCAG  549
             ||||||||||||||||||||||||||||||||||||||||||||||||||||||||||||
Sbjct  918   CGAATTAAACCACATACTCCACTGCTTGTGCGGGTCCCCGTCAATTCCTTTGAGTTTCAG  859

Query  550   TCTTGCGACCGTACTCCCCAGGCGGAATGCTTAATGTGTTAACTTCGGCACCAAGGGTAT  609
             ||||||||||||||||||||||||||||||||||||||||||||||||||||||||||||
Sbjct  858   TCTTGCGACCGTACTCCCCAGGCGGAATGCTTAATGTGTTAACTTCGGCACCAAGGGTAT  799

Query  610   CGAAACCCCTAACACCTAGCATTCATCGTTTACGGCGTGGACTACCAGGGTATCTAATCC  669
             ||||||||||||||||||||||||||||||||||||||||||||||||||||||||||||
Sbjct  798   CGAAACCCCTAACACCTAGCATTCATCGTTTACGGCGTGGACTACCAGGGTATCTAATCC  739

Query  670   TGTTTGCTCCCCACGCTTTCGCGCCTCAGCGTCAGTTACAGCCCAGAAAGTCGCCTTCGC  729
             ||||||||||||||||||||||||||||||||||||||||||||||||||||||||||||
Sbjct  738   TGTTTGCTCCCCACGCTTTCGCGCCTCAGCGTCAGTTACAGCCCAGAAAGTCGCCTTCGC  679

Query  730   CACTGGTGTTCCTCCACATCTCTACGCATTTCACCGCTACACGTGGAATTCCACTTTCCT  789
             ||||||||||||||||||||||||||||||||||||||||||||||||||||||||||||
Sbjct  678   CACTGGTGTTCCTCCACATCTCTACGCATTTCACCGCTACACGTGGAATTCCACTTTCCT  619

Query  790   CTTCTGTACTCAAGCTCTCCAGTTTCCAGTGCGACCCAAGGTTGAGCCTTGGGCTGTGAC  849
             ||||||||||||||||||||||||||||||||||||||||||||||||||||||||||||
Sbjct  618   CTTCTGTACTCAAGCTCTCCAGTTTCCAGTGCGACCCAAGGTTGAGCCTTGGGCTGTGAC  559

Query  850   ACCGGACTTAAAAAGCCGCCTGCGCGCGCTTTACGCCCAATAATTCCGGACAACGCTTGC  909
             ||||||||||||||||||||||||||||||||||||||||||||||||||||||||||||
Sbjct  558   ACCGGACTTAAAAAGCCGCCTGCGCGCGCTTTACGCCCAATAATTCCGGACAACGCTTGC  499

Query  910   CCCCTACGTATTACCGCGGCTGCTGGCACGTAGTTAGCCGGGGCTTTCTTCTCAGGTACC  969
             ||||||||||||||||||||||||||||||||||||||||||||||||||||||||||||
Sbjct  498   CCCCTACGTATTACCGCGGCTGCTGGCACGTAGTTAGCCGGGGCTTTCTTCTCAGGTACC  439

Query  970   GTCACTCCGATAGCAGTTACTCTATCGGACGTTCTTCCCTGGCA  1013
             ||||||||||||||||||||||||||||||||||||||||||||
Sbjct  438   GTCACTCCGATAGCAGTTACTCTATCGGACGTTCTTCCCTGGCA  395
```

Download

FASTA (complete sequence)

FASTA (aligned sequences)

GenBank (complete sequence)

Continue
Cancel

GenBankGraphics

Next
Previous
Descriptions

Paenibacillus sp. 3Cp1 16S ribosomal RNA gene, partial sequence

Sequence ID: KP852522.1Length: 1434Number of Matches: 1

Related Information

Range 1: 402 to 1405GenBankGraphics

Next Match
Previous Match
First Match

Alignment statistics for match #1

| Score | Expect | Identities | Gaps | Strand | Frame |
| --- | --- | --- | --- | --- | --- |
| 1855 bits(1004) | 0.0() | 1004/1004(100%) | 0/1004(0%) | Plus/Minus |  |

Features:

```
Query  10    CCACCGACTTCGGGTGTTGCAAACTCTCGTGGTGTGACGGGCGGTGTGTACAAGACCCGG  69
             ||||||||||||||||||||||||||||||||||||||||||||||||||||||||||||
Sbjct  1405  CCACCGACTTCGGGTGTTGCAAACTCTCGTGGTGTGACGGGCGGTGTGTACAAGACCCGG  1346

Query  70    GAACGTATTCACCGCGGCATGCTGATCCGCGATTACTAGCAATTCCGACTTCATGCAGGC  129
             ||||||||||||||||||||||||||||||||||||||||||||||||||||||||||||
Sbjct  1345  GAACGTATTCACCGCGGCATGCTGATCCGCGATTACTAGCAATTCCGACTTCATGCAGGC  1286

Query  130   GAGTTGCAGCCTGCAATCCGAACTGAGACCGGCTTTTAAGGATTGGCTCCATCTTGCGAT  189
             ||||||||||||||||||||||||||||||||||||||||||||||||||||||||||||
Sbjct  1285  GAGTTGCAGCCTGCAATCCGAACTGAGACCGGCTTTTAAGGATTGGCTCCATCTTGCGAT  1226

Query  190   ATTGCTTCCCGTTGTACCGGCCATTGTAGTACGTGTGTAGCCCAAGTCATAAGGGGCATG  249
             ||||||||||||||||||||||||||||||||||||||||||||||||||||||||||||
Sbjct  1225  ATTGCTTCCCGTTGTACCGGCCATTGTAGTACGTGTGTAGCCCAAGTCATAAGGGGCATG  1166

Query  250   ATGATTTGACGTCATCCCCACCTTCCTCCGGTTTGTCACCGGCAGTCTGCTTAGAGTGCC  309
             ||||||||||||||||||||||||||||||||||||||||||||||||||||||||||||
Sbjct  1165  ATGATTTGACGTCATCCCCACCTTCCTCCGGTTTGTCACCGGCAGTCTGCTTAGAGTGCC  1106

Query  310   CACCATGATGTGCTGGCAACTAAGCATAAGGGTTGCGCTCGTTGCGGGACTTAACCCAAC  369
             ||||||||||||||||||||||||||||||||||||||||||||||||||||||||||||
Sbjct  1105  CACCATGATGTGCTGGCAACTAAGCATAAGGGTTGCGCTCGTTGCGGGACTTAACCCAAC  1046

Query  370   ATCTCACGACACGAGCTGACGACAACCATGCACCACCTGTCTCCTTTGTCCCGAAGGAAA  429
             ||||||||||||||||||||||||||||||||||||||||||||||||||||||||||||
Sbjct  1045  ATCTCACGACACGAGCTGACGACAACCATGCACCACCTGTCTCCTTTGTCCCGAAGGAAA  986

Query  430   AGACTATCTCTAGTCCGGTCAAAGGGATGTCAAGACTTGGTAAGGTTCTTCGCGTTGCTT  489
             ||||||||||||||||||||||||||||||||||||||||||||||||||||||||||||
Sbjct  985   AGACTATCTCTAGTCCGGTCAAAGGGATGTCAAGACTTGGTAAGGTTCTTCGCGTTGCTT  926

Query  490   CGAATTAAACCACATACTCCACTGCTTGTGCGGGTCCCCGTCAATTCCTTTGAGTTTCAG  549
             ||||||||||||||||||||||||||||||||||||||||||||||||||||||||||||
Sbjct  925   CGAATTAAACCACATACTCCACTGCTTGTGCGGGTCCCCGTCAATTCCTTTGAGTTTCAG  866

Query  550   TCTTGCGACCGTACTCCCCAGGCGGAATGCTTAATGTGTTAACTTCGGCACCAAGGGTAT  609
             ||||||||||||||||||||||||||||||||||||||||||||||||||||||||||||
Sbjct  865   TCTTGCGACCGTACTCCCCAGGCGGAATGCTTAATGTGTTAACTTCGGCACCAAGGGTAT  806

Query  610   CGAAACCCCTAACACCTAGCATTCATCGTTTACGGCGTGGACTACCAGGGTATCTAATCC  669
             ||||||||||||||||||||||||||||||||||||||||||||||||||||||||||||
Sbjct  805   CGAAACCCCTAACACCTAGCATTCATCGTTTACGGCGTGGACTACCAGGGTATCTAATCC  746

Query  670   TGTTTGCTCCCCACGCTTTCGCGCCTCAGCGTCAGTTACAGCCCAGAAAGTCGCCTTCGC  729
             ||||||||||||||||||||||||||||||||||||||||||||||||||||||||||||
Sbjct  745   TGTTTGCTCCCCACGCTTTCGCGCCTCAGCGTCAGTTACAGCCCAGAAAGTCGCCTTCGC  686

Query  730   CACTGGTGTTCCTCCACATCTCTACGCATTTCACCGCTACACGTGGAATTCCACTTTCCT  789
             ||||||||||||||||||||||||||||||||||||||||||||||||||||||||||||
Sbjct  685   CACTGGTGTTCCTCCACATCTCTACGCATTTCACCGCTACACGTGGAATTCCACTTTCCT  626

Query  790   CTTCTGTACTCAAGCTCTCCAGTTTCCAGTGCGACCCAAGGTTGAGCCTTGGGCTGTGAC  849
             ||||||||||||||||||||||||||||||||||||||||||||||||||||||||||||
Sbjct  625   CTTCTGTACTCAAGCTCTCCAGTTTCCAGTGCGACCCAAGGTTGAGCCTTGGGCTGTGAC  566

Query  850   ACCGGACTTAAAAAGCCGCCTGCGCGCGCTTTACGCCCAATAATTCCGGACAACGCTTGC  909
             ||||||||||||||||||||||||||||||||||||||||||||||||||||||||||||
Sbjct  565   ACCGGACTTAAAAAGCCGCCTGCGCGCGCTTTACGCCCAATAATTCCGGACAACGCTTGC  506

Query  910   CCCCTACGTATTACCGCGGCTGCTGGCACGTAGTTAGCCGGGGCTTTCTTCTCAGGTACC  969
             ||||||||||||||||||||||||||||||||||||||||||||||||||||||||||||
Sbjct  505   CCCCTACGTATTACCGCGGCTGCTGGCACGTAGTTAGCCGGGGCTTTCTTCTCAGGTACC  446

Query  970   GTCACTCCGATAGCAGTTACTCTATCGGACGTTCTTCCCTGGCA  1013
             ||||||||||||||||||||||||||||||||||||||||||||
Sbjct  445   GTCACTCCGATAGCAGTTACTCTATCGGACGTTCTTCCCTGGCA  402
```

Download

FASTA (complete sequence)

FASTA (aligned sequences)

GenBank (complete sequence)

Continue
Cancel

GenBankGraphics

Next
Previous
Descriptions

Uncultured bacterium clone 4L13 16S ribosomal RNA gene, partial sequence

Sequence ID: KP843054.1Length: 1460Number of Matches: 1

Related Information

Range 1: 414 to 1417GenBankGraphics

Next Match
Previous Match
First Match

Alignment statistics for match #1

| Score | Expect | Identities | Gaps | Strand | Frame |
| --- | --- | --- | --- | --- | --- |
| 1855 bits(1004) | 0.0() | 1004/1004(100%) | 0/1004(0%) | Plus/Minus |  |

Features:

```
Query  10    CCACCGACTTCGGGTGTTGCAAACTCTCGTGGTGTGACGGGCGGTGTGTACAAGACCCGG  69
             ||||||||||||||||||||||||||||||||||||||||||||||||||||||||||||
Sbjct  1417  CCACCGACTTCGGGTGTTGCAAACTCTCGTGGTGTGACGGGCGGTGTGTACAAGACCCGG  1358

Query  70    GAACGTATTCACCGCGGCATGCTGATCCGCGATTACTAGCAATTCCGACTTCATGCAGGC  129
             ||||||||||||||||||||||||||||||||||||||||||||||||||||||||||||
Sbjct  1357  GAACGTATTCACCGCGGCATGCTGATCCGCGATTACTAGCAATTCCGACTTCATGCAGGC  1298

Query  130   GAGTTGCAGCCTGCAATCCGAACTGAGACCGGCTTTTAAGGATTGGCTCCATCTTGCGAT  189
             ||||||||||||||||||||||||||||||||||||||||||||||||||||||||||||
Sbjct  1297  GAGTTGCAGCCTGCAATCCGAACTGAGACCGGCTTTTAAGGATTGGCTCCATCTTGCGAT  1238

Query  190   ATTGCTTCCCGTTGTACCGGCCATTGTAGTACGTGTGTAGCCCAAGTCATAAGGGGCATG  249
             ||||||||||||||||||||||||||||||||||||||||||||||||||||||||||||
Sbjct  1237  ATTGCTTCCCGTTGTACCGGCCATTGTAGTACGTGTGTAGCCCAAGTCATAAGGGGCATG  1178

Query  250   ATGATTTGACGTCATCCCCACCTTCCTCCGGTTTGTCACCGGCAGTCTGCTTAGAGTGCC  309
             ||||||||||||||||||||||||||||||||||||||||||||||||||||||||||||
Sbjct  1177  ATGATTTGACGTCATCCCCACCTTCCTCCGGTTTGTCACCGGCAGTCTGCTTAGAGTGCC  1118

Query  310   CACCATGATGTGCTGGCAACTAAGCATAAGGGTTGCGCTCGTTGCGGGACTTAACCCAAC  369
             ||||||||||||||||||||||||||||||||||||||||||||||||||||||||||||
Sbjct  1117  CACCATGATGTGCTGGCAACTAAGCATAAGGGTTGCGCTCGTTGCGGGACTTAACCCAAC  1058

Query  370   ATCTCACGACACGAGCTGACGACAACCATGCACCACCTGTCTCCTTTGTCCCGAAGGAAA  429
             ||||||||||||||||||||||||||||||||||||||||||||||||||||||||||||
Sbjct  1057  ATCTCACGACACGAGCTGACGACAACCATGCACCACCTGTCTCCTTTGTCCCGAAGGAAA  998

Query  430   AGACTATCTCTAGTCCGGTCAAAGGGATGTCAAGACTTGGTAAGGTTCTTCGCGTTGCTT  489
             ||||||||||||||||||||||||||||||||||||||||||||||||||||||||||||
Sbjct  997   AGACTATCTCTAGTCCGGTCAAAGGGATGTCAAGACTTGGTAAGGTTCTTCGCGTTGCTT  938

Query  490   CGAATTAAACCACATACTCCACTGCTTGTGCGGGTCCCCGTCAATTCCTTTGAGTTTCAG  549
             ||||||||||||||||||||||||||||||||||||||||||||||||||||||||||||
Sbjct  937   CGAATTAAACCACATACTCCACTGCTTGTGCGGGTCCCCGTCAATTCCTTTGAGTTTCAG  878

Query  550   TCTTGCGACCGTACTCCCCAGGCGGAATGCTTAATGTGTTAACTTCGGCACCAAGGGTAT  609
             ||||||||||||||||||||||||||||||||||||||||||||||||||||||||||||
Sbjct  877   TCTTGCGACCGTACTCCCCAGGCGGAATGCTTAATGTGTTAACTTCGGCACCAAGGGTAT  818

Query  610   CGAAACCCCTAACACCTAGCATTCATCGTTTACGGCGTGGACTACCAGGGTATCTAATCC  669
             ||||||||||||||||||||||||||||||||||||||||||||||||||||||||||||
Sbjct  817   CGAAACCCCTAACACCTAGCATTCATCGTTTACGGCGTGGACTACCAGGGTATCTAATCC  758

Query  670   TGTTTGCTCCCCACGCTTTCGCGCCTCAGCGTCAGTTACAGCCCAGAAAGTCGCCTTCGC  729
             ||||||||||||||||||||||||||||||||||||||||||||||||||||||||||||
Sbjct  757   TGTTTGCTCCCCACGCTTTCGCGCCTCAGCGTCAGTTACAGCCCAGAAAGTCGCCTTCGC  698

Query  730   CACTGGTGTTCCTCCACATCTCTACGCATTTCACCGCTACACGTGGAATTCCACTTTCCT  789
             ||||||||||||||||||||||||||||||||||||||||||||||||||||||||||||
Sbjct  697   CACTGGTGTTCCTCCACATCTCTACGCATTTCACCGCTACACGTGGAATTCCACTTTCCT  638

Query  790   CTTCTGTACTCAAGCTCTCCAGTTTCCAGTGCGACCCAAGGTTGAGCCTTGGGCTGTGAC  849
             ||||||||||||||||||||||||||||||||||||||||||||||||||||||||||||
Sbjct  637   CTTCTGTACTCAAGCTCTCCAGTTTCCAGTGCGACCCAAGGTTGAGCCTTGGGCTGTGAC  578

Query  850   ACCGGACTTAAAAAGCCGCCTGCGCGCGCTTTACGCCCAATAATTCCGGACAACGCTTGC  909
             ||||||||||||||||||||||||||||||||||||||||||||||||||||||||||||
Sbjct  577   ACCGGACTTAAAAAGCCGCCTGCGCGCGCTTTACGCCCAATAATTCCGGACAACGCTTGC  518

Query  910   CCCCTACGTATTACCGCGGCTGCTGGCACGTAGTTAGCCGGGGCTTTCTTCTCAGGTACC  969
             ||||||||||||||||||||||||||||||||||||||||||||||||||||||||||||
Sbjct  517   CCCCTACGTATTACCGCGGCTGCTGGCACGTAGTTAGCCGGGGCTTTCTTCTCAGGTACC  458

Query  970   GTCACTCCGATAGCAGTTACTCTATCGGACGTTCTTCCCTGGCA  1013
             ||||||||||||||||||||||||||||||||||||||||||||
Sbjct  457   GTCACTCCGATAGCAGTTACTCTATCGGACGTTCTTCCCTGGCA  414
```

Download

FASTA (complete sequence)

FASTA (aligned sequences)

GenBank (complete sequence)

Continue
Cancel

GenBankGraphics

Next
Previous
Descriptions

Paenibacillus sp. TI45-13ar 16S ribosomal RNA gene, partial sequence

Sequence ID: KY419705.1Length: 1472Number of Matches: 1

Related Information

Range 1: 431 to 1435GenBankGraphics

Next Match
Previous Match
First Match

Alignment statistics for match #1

| Score | Expect | Identities | Gaps | Strand | Frame |
| --- | --- | --- | --- | --- | --- |
| 1849 bits(1001) | 0.0() | 1004/1005(99%) | 1/1005(0%) | Plus/Minus |  |

Features:

```
Query  10    CCACCGACTTCGGGTGTTGCAAACTCTCGTGGTGTGACGGGCGGTGTGTACAAGACCCGG  69
             ||||||||||||||||||||||||||||||||||||||||||||||||||||||||||||
Sbjct  1435  CCACCGACTTCGGGTGTTGCAAACTCTCGTGGTGTGACGGGCGGTGTGTACAAGACCCGG  1376

Query  70    GAACGTATTCACCGCGGCATGCTGATCCGCGATTACTAGCAATTCCGACTTCATGCAGGC  129
             ||||||||||||||||||||||||||||||||||||||||||||||||||||||||||||
Sbjct  1375  GAACGTATTCACCGCGGCATGCTGATCCGCGATTACTAGCAATTCCGACTTCATGCAGGC  1316

Query  130   GAGTTGCAGCCTGCAATCCGAACTGAGACCGGCTTTTAAGGATTGGCTCCATCTTGCGAT  189
             ||||||||||||||||||||||||||||||||||||||||||||||||||||||||||||
Sbjct  1315  GAGTTGCAGCCTGCAATCCGAACTGAGACCGGCTTTTAAGGATTGGCTCCATCTTGCGAT  1256

Query  190   ATTGCTTCCCGTTGTACCGGCCATTGTAGTACGTGTGTAGCCCAAGTCATAAGGGGCATG  249
             ||||||||||||||||||||||||||||||||||||||||||||||||||||||||||||
Sbjct  1255  ATTGCTTCCCGTTGTACCGGCCATTGTAGTACGTGTGTAGCCCAAGTCATAAGGGGCATG  1196

Query  250   ATGATTTGACGTCATCCCCACCTTCCTCCGGTTTGTCACCGGCAGTCTGCTTAGAGTGCC  309
             ||||||||||||||||||||||||||||||||||||||||||||||||||||||||||||
Sbjct  1195  ATGATTTGACGTCATCCCCACCTTCCTCCGGTTTGTCACCGGCAGTCTGCTTAGAGTGCC  1136

Query  310   CACCATGATGTGCTGGCAACTAAGCATAAGGGTTGCGCTCGTTGCGGGACTTAACCCAAC  369
             ||||||||||||||||||||||||||||||||||||||||||||||||||||||||||||
Sbjct  1135  CACCATGATGTGCTGGCAACTAAGCATAAGGGTTGCGCTCGTTGCGGGACTTAACCCAAC  1076

Query  370   ATCTCACGACACGAGCTGACGACAACCATGCACCACCTGTCTCCTTTGTCCCGAAGGAAA  429
             ||||||||||||||||||||||||||||||||||||||||||||||||||||||||||||
Sbjct  1075  ATCTCACGACACGAGCTGACGACAACCATGCACCACCTGTCTCCTTTGTCCCGAAGGAAA  1016

Query  430   AGACTATCTCTAGTCCGGTCAAAGGGATGTCAAGACTTGGTAAGGTTCTTCGCGTTGCTT  489
             ||||||||||||||||||||||||||||||||||||||||||||||||||||||||||||
Sbjct  1015  AGACTATCTCTAGTCCGGTCAAAGGGATGTCAAGACTTGGTAAGGTTCTTCGCGTTGCTT  956

Query  490   CGAATTAAACCACATACTCCACTGCTTGTGCGGGTCCCCGTCAATTCCTTTGAGTTTCAG  549
             ||||||||||||||||||||||||||||||||||||||||||||||||||||||||||||
Sbjct  955   CGAATTAAACCACATACTCCACTGCTTGTGCGGGTCCCCGTCAATTCCTTTGAGTTTCAG  896

Query  550   TCTTGCGACCGTACTCCCCAGGCGGAATGCTTAATGTGTTAACTTCGGCACCAAGGGTAT  609
             ||||||||||||||||||||||||||||||||||||||||||||||||||||||||||||
Sbjct  895   TCTTGCGACCGTACTCCCCAGGCGGAATGCTTAATGTGTTAACTTCGGCACCAAGGGTAT  836

Query  610   CGAAACCCCTAACACCTAGCATTCATCGTTTACGGCGTGGACTACCAGGGTATCTAATCC  669
             ||||||||||||||||||||||||||||||||||||||||||||||||||||||||||||
Sbjct  835   CGAAACCCCTAACACCTAGCATTCATCGTTTACGGCGTGGACTACCAGGGTATCTAATCC  776

Query  670   TGTTTGCTCCCCACGCTTTCGCGCCTCAGCGTCAGTTACAGCCCAGAAAGTCGCCTTCGC  729
             ||||||||||||||||||||||||||||||||||||||||||||||||||||||||||||
Sbjct  775   TGTTTGCTCCCCACGCTTTCGCGCCTCAGCGTCAGTTACAGCCCAGAAAGTCGCCTTCGC  716

Query  730   CACTGGTGTTCCTCCACATCTCTACGCATTTCACCGCTACACGTGGAATTC-CACTTTCC  788
             ||||||||||||||||||||||||||||||||||||||||||||||||||| ||||||||
Sbjct  715   CACTGGTGTTCCTCCACATCTCTACGCATTTCACCGCTACACGTGGAATTCTCACTTTCC  656

Query  789   TCTTCTGTACTCAAGCTCTCCAGTTTCCAGTGCGACCCAAGGTTGAGCCTTGGGCTGTGA  848
             ||||||||||||||||||||||||||||||||||||||||||||||||||||||||||||
Sbjct  655   TCTTCTGTACTCAAGCTCTCCAGTTTCCAGTGCGACCCAAGGTTGAGCCTTGGGCTGTGA  596

Query  849   CACCGGACTTAAAAAGCCGCCTGCGCGCGCTTTACGCCCAATAATTCCGGACAACGCTTG  908
             ||||||||||||||||||||||||||||||||||||||||||||||||||||||||||||
Sbjct  595   CACCGGACTTAAAAAGCCGCCTGCGCGCGCTTTACGCCCAATAATTCCGGACAACGCTTG  536

Query  909   CCCCCTACGTATTACCGCGGCTGCTGGCACGTAGTTAGCCGGGGCTTTCTTCTCAGGTAC  968
             ||||||||||||||||||||||||||||||||||||||||||||||||||||||||||||
Sbjct  535   CCCCCTACGTATTACCGCGGCTGCTGGCACGTAGTTAGCCGGGGCTTTCTTCTCAGGTAC  476

Query  969   CGTCACTCCGATAGCAGTTACTCTATCGGACGTTCTTCCCTGGCA  1013
             |||||||||||||||||||||||||||||||||||||||||||||
Sbjct  475   CGTCACTCCGATAGCAGTTACTCTATCGGACGTTCTTCCCTGGCA  431
```

Download

FASTA (complete sequence)

FASTA (aligned sequences)

GenBank (complete sequence)

Continue
Cancel

GenBankGraphics

Next
Previous
Descriptions

Bacterium 4R9 16S ribosomal RNA gene, partial sequence

Sequence ID: KP965575.1Length: 1517Number of Matches: 1

Related Information

Range 1: 443 to 1447GenBankGraphics

Next Match
Previous Match
First Match

Alignment statistics for match #1

| Score | Expect | Identities | Gaps | Strand | Frame |
| --- | --- | --- | --- | --- | --- |
| 1844 bits(998) | 0.0() | 1003/1005(99%) | 1/1005(0%) | Plus/Minus |  |

Features:

```
Query  10    CCACCGACTTCGGGTGTTGCAAACTCTCGTGGTGTGACGGGCGGTGTGTACAAGACCCGG  69
             ||||||||||||||||||||||||||||||||||||||||||||||||||||||||||||
Sbjct  1447  CCACCGACTTCGGGTGTTGCAAACTCTCGTGGTGTGACGGGCGGTGTGTACAAGACCCGG  1388

Query  70    GAACGTATTCACCGCGGCATGCTGATCCGCGATTACTAGCAATTCCGACTTCATGCAGGC  129
             ||||||||||||||||||||||||||||||||||||||||||||||||||||||| ||||
Sbjct  1387  GAACGTATTCACCGCGGCATGCTGATCCGCGATTACTAGCAATTCCGACTTCATGTAGGC  1328

Query  130   GAGTTGCAGCCTGCAATCCGAACTGAGACCGGCTTTTAAGGATTGGCTCCATCTTGCGAT  189
             ||||||||||||||||||||||||||||||||||||||||||||||||||||||||||||
Sbjct  1327  GAGTTGCAGCCTGCAATCCGAACTGAGACCGGCTTTTAAGGATTGGCTCCATCTTGCGAT  1268

Query  190   ATTGCTTCCCGTTGTACCGGCCATTGTAGTACGTGTGTAGCCCAAGTCATAAGGGGCATG  249
             ||||||||||||||||||||||||||||||||||||||||||||||||||||||||||||
Sbjct  1267  ATTGCTTCCCGTTGTACCGGCCATTGTAGTACGTGTGTAGCCCAAGTCATAAGGGGCATG  1208

Query  250   ATGATTTGACGTCATCCCCACCTTCCTCCGGTTTGTCACCGGCAGTCTGCTTAGAGTGCC  309
             ||||||||||||||||||||||||||||||||||||||||||||||||||||||||||||
Sbjct  1207  ATGATTTGACGTCATCCCCACCTTCCTCCGGTTTGTCACCGGCAGTCTGCTTAGAGTGCC  1148

Query  310   CACCATGATGTGCTGGCAACTAAGCATAAGGGTTGCGCTCGTTGCGGGACTTAACCCAAC  369
             ||||||||||||||||||||||||||||||||||||||||||||||||||||||||||||
Sbjct  1147  CACCATGATGTGCTGGCAACTAAGCATAAGGGTTGCGCTCGTTGCGGGACTTAACCCAAC  1088

Query  370   ATCTCACGACACGAGCTGACGACAACCATGCACCACCTGTCTCCTTTGTCCCGAAGGAAA  429
             ||||||||||||||||||||||||||||||||||||||||||||||||||||||||||||
Sbjct  1087  ATCTCACGACACGAGCTGACGACAACCATGCACCACCTGTCTCCTTTGTCCCGAAGGAAA  1028

Query  430   AGACTATCTCTAGTCCGGTCAAAGGGATGTCAAGACTTGGTAAGGTTCTTCGCGTTGCTT  489
             ||||||||||||||||||||||||||||||||||||||||||||||||||||||||||||
Sbjct  1027  AGACTATCTCTAGTCCGGTCAAAGGGATGTCAAGACTTGGTAAGGTTCTTCGCGTTGCTT  968

Query  490   CGAATTAAACCACATACTCCACTGCTTGTGCGGGTCCCCGTCAATTCCTTTGAGTTTCAG  549
             ||||||||||||||||||||||||||||||||||||||||||||||||||||||||||||
Sbjct  967   CGAATTAAACCACATACTCCACTGCTTGTGCGGGTCCCCGTCAATTCCTTTGAGTTTCAG  908

Query  550   TCTTGCGACCGTACTCCCCAGGCGGAATGCTTAATGTGTTAACTTCGGCACCAAGGGTAT  609
             ||||||||||||||||||||||||||||||||||||||||||||||||||||||||||||
Sbjct  907   TCTTGCGACCGTACTCCCCAGGCGGAATGCTTAATGTGTTAACTTCGGCACCAAGGGTAT  848

Query  610   CGAAACCCCTAACACCTAGCATTCATCGTTTACGGCGTGGACTACCAGGGTATCTAATCC  669
             ||||||||||||||||||||||||||||||||||||||||||||||||||||||||||||
Sbjct  847   CGAAACCCCTAACACCTAGCATTCATCGTTTACGGCGTGGACTACCAGGGTATCTAATCC  788

Query  670   TGTTTGCTCCCCACGCTTTCGCGCCTCAGCGTCAGTTACAGCCCAGAAAGTCGCCTTCGC  729
             ||||||||||||||||||||||||||||||||||||||||||||||||||||||||||||
Sbjct  787   TGTTTGCTCCCCACGCTTTCGCGCCTCAGCGTCAGTTACAGCCCAGAAAGTCGCCTTCGC  728

Query  730   CACTGGTGTTCCTCCACATCTCTACGCATTTCACCGCTACACGTGGAATTCCACTTTCCT  789
             ||||||||||||||||||||||||||||||||||||||||||||||||||||||||||||
Sbjct  727   CACTGGTGTTCCTCCACATCTCTACGCATTTCACCGCTACACGTGGAATTCCACTTTCCT  668

Query  790   CTTCTGTACTCAAGCTCTCCAGTTTCCAGTGCGACCCAAGG-TTGAGCCTTGGGCTGTGA  848
             ||||||||||||||||||||||||||||||||||||||||| ||||||||||||||||||
Sbjct  667   CTTCTGTACTCAAGCTCTCCAGTTTCCAGTGCGACCCAAGGGTTGAGCCTTGGGCTGTGA  608

Query  849   CACCGGACTTAAAAAGCCGCCTGCGCGCGCTTTACGCCCAATAATTCCGGACAACGCTTG  908
             ||||||||||||||||||||||||||||||||||||||||||||||||||||||||||||
Sbjct  607   CACCGGACTTAAAAAGCCGCCTGCGCGCGCTTTACGCCCAATAATTCCGGACAACGCTTG  548

Query  909   CCCCCTACGTATTACCGCGGCTGCTGGCACGTAGTTAGCCGGGGCTTTCTTCTCAGGTAC  968
             ||||||||||||||||||||||||||||||||||||||||||||||||||||||||||||
Sbjct  547   CCCCCTACGTATTACCGCGGCTGCTGGCACGTAGTTAGCCGGGGCTTTCTTCTCAGGTAC  488

Query  969   CGTCACTCCGATAGCAGTTACTCTATCGGACGTTCTTCCCTGGCA  1013
             |||||||||||||||||||||||||||||||||||||||||||||
Sbjct  487   CGTCACTCCGATAGCAGTTACTCTATCGGACGTTCTTCCCTGGCA  443
```


BLAST is a registered trademark of the National Library of Medicine

Support center
Mailing list


YouTube

- National Library Of Medicine
- National Institutes Of Health
- U.S. Department of Health & Human Services
- USA.gov

### NCBI


National Center for Biotechnology Information,
 U.S. National Library of Medicine

8600 Rockville Pike,
Bethesda
 MD,
20894
USA

Policies and Guidelines
|
Contact


PreferencesTurn off

External link. Please review our privacy policy.
