# Supplementary material for: Isothermal microcalorimetry for thermal viable count of microorganisms in pure cultures and stabilized formulations
Source: BMC Microbiol. 2019 Mar 21;19:65. doi: 10.1186/s12866-019-1432-8 (PMC6429831; doi:10.1186/s12866-019-1432-8)
Supplement: Supplementary file 3 — 16S rRNA gene sequences’ BLASTN hits in zipped HTML format. (ZIP 15810 kb) [file 12866_2019_1432_MOESM3_ESM.zip › Best blastn hits/NCBI Blast_L pinkish SS F -- 13..1001 of sequence.html]

NCBI Blast:L pinkish SS F -- 13..1001 of sequence


- NCBI Home
- Sign in to NCBI
- Skip to Main Content
- Skip to Navigation
- About NCBI Accesskeys

U.S. National Library of Medicine

NCBI
National Center for Biotechnology Information

- My NCBI
- Sign in to NCBI
- Register
- Sign Out

BLAST ^®^ » blastn suite » RID-A4XVSRST014


- Home
- Recent Results
- Saved Strategies
- Help

BLAST Results


Edit and Resubmit
Save Search Strategies
[Sign in above to save your search strategy]

Formatting options 


Download


How to read this page
Blast report description
Questions/comments


|  |  |
| --- | --- |
| Formatting options | |
| Show | Alignment as  HTML Plain text   Old View Reset form to defaults [?]  These options control formatting of alignments in results pages. The default is HTML, but other formats (including plain text) are available. PSSM and PssmWithParameters are representations of Position Specific Scoring Matrices and are only available for PSI-BLAST. The Advanced view option allows the database descriptions to be sorted by various indices in a table. |
| Alignment View | Pairwise Pairwise with dots for identities Query-anchored with dots for identities Query-anchored with letters for identities Flat query-anchored with dots for identities Flat query-anchored with letters for identities [?]  Choose how to view alignments. The default "pairwise" view shows how each subject sequence aligns individually to the query sequence. The "query-anchored" view shows how all subject sequences align to the query sequence. For each view type, you can choose to show "identities" (matching residues) as letters or dots. more... |
| Display | Graphical Overview   Linkout   Sequence Retrieval  NCBI-gi   CDS feature [?]  - Graphical Overview: Graphical Overview: Show graph of similar sequence regions aligned to query.   more... - NCBI-gi: Show NCBI gi identifiers. - CDS feature: Show annotated coding region and translation.   more... |
| Masking | Character:   X for protein, n for nucleotide Lower Case  Color:  Black Grey Red [?]  - Masking Character: Display masked (filtered) sequence regions as lower-case or as specific letters (N for nucleotide, P for protein). - Masking Color: Display masked sequence regions in the given color. |
| Limit results | Descriptions:  10 50 100 Graphical overview:  0 10 50 100  Alignments:  0 10 50 100 Line length:  60 90 120 150 [?]  - Descriptions: Show short descriptions for up to the given number of sequences. - Alignments: Show alignments for up to the given number of sequences, in order of statistical significance. - Line lenghth: Number of letters to show on one line in an alignment. |
|  | Organism Type common name, binomial, taxid, or group name. Only 20 top taxa will be shown.     Exclude   [?]  Show only sequences from the given organism. |
|  | Entrez query:  [?]  Show only those sequences that match the given Entrez query. more... |
|  | Expect Min:  Expect Max:  [?]  Show only sequences with expect values in the given range. more... |
|  | Percent Identity Min:  Percent Identity Max:  [?]  Show only sequences with percent identity values in the given range. |
| Format for | PSI-BLAST with inclusion threshold:  [?]  - Format for PSI-BLAST: The Position-Specific Iterated BLAST (PSI-BLAST) program performs iterative searches with a protein query,   in which sequences found in one round of search are used to build a custom score model for the next round.   more... - Inclusion Threshold: This sets the statistical significance threshold for including a sequence in the model used   by PSI-BLAST to create the PSSM on the next iteration. |

|  |  |  |  |  |  |
| --- | --- | --- | --- | --- | --- |
| Download | | | | | |
| Alignment  Text XML ASN.1 JSON Seq-align Hit Table(text) Hit Table(csv) Multiple-file XML2 Single-file XML2 Multiple-file JSON Single-file JSON SAM | Search Strategies  ASN.1 | PSSM to restart search  PSSM | [?] |  |  |

The Download link provides BLAST output that may be used as input to another program.
This includes parseable formats such as the tabular report or XML as well as the Search Strategy files read by the BLAST+ applications.
More details on the parseable (XML, tabular, and ASN.1) reports can be found at
https://www.ncbi.nlm.nih.gov/books/NBK153387/  
  

The following formats are offered under the Alignment section:  
1). "Text". Non-HTML standard BLAST report.  
2). "XML". XML report based upon the DTD at https://www.ncbi.nlm.nih.gov/data\_specs/dtd/NCBI\_BlastOutput.dtd  
3). "ASN.1". Alignment written out in Abstract Syntax Notation 1.  
4). "JSON Seq-align". Alignment written out in JSON.  
4). "Hit Table(text)". The tabular report as text.  
5). "Hit Table(csv)". The tabular report ready for import into spread-sheet programs like Excel.  
6). "XML2". New XML format described at ftp://ftp.ncbi.nlm.nih.gov/blast/documents/NEWXML/xml2.pdf.  
7). "JSON". New JSON format described at ftp://ftp.ncbi.nlm.nih.gov/blast/documents/NEWXML/xml2.pdf.  
8). "SAM". Sequence Alignment Map format.

XML2 and JSON can be downloaded either as one file per query (multiple-file) or one file for all queries (single-file). These formats are listed as Multiple-file XML2 (and JSON) or Single-file XML (and JSON).

The following report is offered under the Search Strategy section:  
1). "ASN.1" Search Strategy. A record of the parameters, query, and database used in the search. This file can be used to start a stand-alone BLAST search, see
https://www.ncbi.nlm.nih.gov/books/NBK1763/#CmdLineAppsManual.I455\_BLAST\_search\_stra


# Job title: L pinkish SS F -- 13..1001 of sequence

Results for:

lcl|Query\_6549 L pinkish SS F -- 13..1001 of sequence(989bp)
[?]

Your BLAST job specified more than one input sequence.
This box lets you choose which input sequence to show BLAST results for.

RID
:   A4XVSRST014 (Expires on 03-10 19:00 pm)

Query ID
:   lcl|Query\_6549
:   lcl|Query\_6549

Description
:   L pinkish SS F -- 13..1001 of sequence

Molecule type
:   nucleic acid

Query Length
:   989

Database Name
:   nr

Description
:   Nucleotide collection (nt) See details

Program
:   BLASTN 2.8.0+ Citation

  

Reference 

Zheng Zhang, Scott Schwartz, Lukas Wagner, and Webb Miller (2000), "A greedy algorithm for aligning DNA sequences", J Comput Biol 2000; 7(1-2):203-14.

Reference - database indexing

Aleksandr Morgulis, George Coulouris, Yan Raytselis, Thomas L. Madden, Richa Agarwala, Alejandro A. Schäffer (2008), "Database Indexing for Production MegaBLAST Searches", Bioinformatics 24:1757-1764.

Other reports:
Search Summary

[Taxonomy reports]
[Distance tree of results]
[MSA viewer]

Search Parameters

| Search parameter name | Search parameter value |
| --- | --- |
| Program | blastn |
| Word size | 28 |
| Expect value | 10 |
| Hitlist size | 100 |
| Match/Mismatch scores | 1,-2 |
| Gapcosts | 0,2.5 |
| Low Complexity Filter | Yes |
| Filter string | L;m; |
| Genetic Code | 1 |

Database

| Database parameter name | Database parameter value |
| --- | --- |
| Posted date | Mar 3, 2018 9:32 PM |
| Number of letters | 171,598,911,005 |
| Number of sequences | 46,870,701 |
| Entrez query | Includes:  Excludes:  None |

Karlin-Altschul statistics

| Params | Ungapped | Gapped |
| --- | --- | --- |
| Lambda | 1.33271 | 1.28 |
| K | 0.620991 | 0.46 |
| H | 1.12409 | 0.85 |

Results Statistics

| Results Statistics parameter name | Results Statistics parameter value |
| --- | --- |
| Length adjustment | 35 |
| Effective length of query | 954 |
| Effective length of database | 169958436470 |
| Effective search space | 162140348392380 |
| Effective search space used | 162140348392380 |


## Graphic Summary

### Distribution of the top 136 Blast Hits on 100 subject sequences [?]

The graphic is an overview of the database sequences aligned to the query sequence. These are represented horizontal bars colored coded by score and showing the extent
of the alignment on the query sequence. Separate aligned regions on the same database sequence are connected by a thin grey line.
Mousing over an alignment shows the database sequence title. Clicking an alignment displays a box with more details about the alignment and
link to the sequence alignment itself in the Alignments section of the report.

Mouse over to see the title, click to show alignments

Color key for alignment scores

<40

40-50

50-80

80-200

>=200

Query

1

150

300

450

600

750

900

Pseudomonas syringae pv. syringae strain B728a 16S ribo..

Score:1786 Evalue:0

Accession:MG722805.1

Alignment

Pseudomonas syringae pv. malvacearum strain 480-2 16S r..

Score:1786 Evalue:0

Accession:MG230266.1

Alignment

Pseudomonas savastanoi strain XJPY11 16S ribosomal RNA ..

Score:1786 Evalue:0

Accession:KR708944.1

Alignment

Pseudomonas savastanoi strain XJPY13 16S ribosomal RNA ..

Score:1786 Evalue:0

Accession:KR708943.1

Alignment

Pseudomonas savastanoi strain XJGY3 16S ribosomal RNA g..

Score:1786 Evalue:0

Accession:KR708880.1

Alignment

Pseudomonas syringae strain 31R1 genome assembly, chrom..

Score:1786 Evalue:0

Accession:LT629769.1

Alignment

Pseudomonas sp. HP7P 16S ribosomal RNA gene, partial se..

Score:1786 Evalue:0

Accession:KM187525.1

Alignment

Pseudomonas amygdali pv. tabaci strain YWTG-1 16S ribos..

Score:1786 Evalue:0

Accession:KR476391.1

Alignment

Pseudomonas syringae pv. syringae B301D, complete genom..

Score:1786 Evalue:0

Accession:CP005969.1

Alignment

Pseudomonas syringae pv. tabaci gene for 16S ribosomal ..

Score:1786 Evalue:0

Accession:LC010244.1

Alignment

Pseudomonas syringae pv. tabaci gene for 16S ribosomal ..

Score:1786 Evalue:0

Accession:LC010243.1

Alignment

Pseudomonas syringae pv. tabaci strain SWTG-1 16S ribos..

Score:1786 Evalue:0

Accession:KJ756714.1

Alignment

Pseudomonas syringae pv. phaseolicola strain LDS9 16S r..

Score:1786 Evalue:0

Accession:KF888741.1

Alignment

Pseudomonas syringae pv. phaseolicola strain LDS8 16S r..

Score:1786 Evalue:0

Accession:KF888740.1

Alignment

Pseudomonas syringae pv. phaseolicola strain LDS7 16S r..

Score:1786 Evalue:0

Accession:KF888739.1

Alignment

Pseudomonas syringae gene for 16S rRNA, partial sequenc..

Score:1786 Evalue:0

Accession:AB680556.1

Alignment

Pseudomonas syringae gene for 16S rRNA, partial sequenc..

Score:1786 Evalue:0

Accession:AB680544.1

Alignment

Pseudomonas sp. Act34 partial 16S rRNA gene, strain Act..

Score:1786 Evalue:0

Accession:AM909659.1

Alignment

Pseudomonas sp. Ina1 partial 16S rRNA gene, strain Ina1

Score:1786 Evalue:0

Accession:AM909655.1

Alignment

Pseudomonas syringae pv. coryli 16S rRNA gene, strain I..

Score:1786 Evalue:0

Accession:AM399034.1

Alignment

Pseudomonas syringae pv. pisi gene for 16S ribosomal RN..

Score:1786 Evalue:0

Accession:AB109218.1

Alignment

Pseudomonas syringae pv. syringae B728a, complete genom..

Score:1786 Evalue:0

Accession:CP000075.1

Alignment

Pseudomonas savastanoi strain CGPY4 16S ribosomal RNA g..

Score:1783 Evalue:0

Accession:KR708885.1

Alignment

Pseudomonas syringae pv. syringae strain Pss9097 chromo..

Score:1781 Evalue:0

Accession:CP026568.1

Alignment

Pseudomonas syringae pv. syringae isolate CFBP2118 geno..

Score:1781 Evalue:0

Accession:LT962481.1

Alignment

Pseudomonas syringae pv. syringae isolate CFBP4215 geno..

Score:1781 Evalue:0

Accession:LT962480.1

Alignment

Pseudomonas syringae pv. malvacearum strain 480-5 16S r..

Score:1781 Evalue:0

Accession:MG230269.1

Alignment

Pseudomonas syringae pv. malvacearum strain 480-4 16S r..

Score:1781 Evalue:0

Accession:MG230268.1

Alignment

Pseudomonas syringae pv. malvacearum strain 480-3 16S r..

Score:1781 Evalue:0

Accession:MG230267.1

Alignment

Pseudomonas syringae pv. malvacearum strain 480-1 16S r..

Score:1781 Evalue:0

Accession:MG230265.1

Alignment

Pseudomonas coronafaciens pv. striafaciens strain NCPPB..

Score:1781 Evalue:0

Accession:MF693348.1

Alignment

Pseudomonas amygdali pv. lachrymans strain NM002, compl..

Score:1781 Evalue:0

Accession:CP020351.1

Alignment

Paenibacillus polymyxa strain HGPJ-61 16S ribosomal RNA..

Score:1781 Evalue:0

Accession:KR708873.1

Alignment

Pseudomonas tremae strain HGPY-131 16S ribosomal RNA ge..

Score:1781 Evalue:0

Accession:KR708870.1

Alignment

Pseudomonas syringae strain 7GJ-14 16S ribosomal RNA ge..

Score:1781 Evalue:0

Accession:KR708853.1

Alignment

Pseudomonas syringae strain HGGY-6 16S ribosomal RNA ge..

Score:1781 Evalue:0

Accession:KR708837.1

Alignment

Pseudomonas syringae gene for 16S ribosomal RNA, partia..

Score:1781 Evalue:0

Accession:LC031818.1

Alignment

Pseudomonas syringae gene for 16S ribosomal RNA, partia..

Score:1781 Evalue:0

Accession:LC031817.1

Alignment

Pseudomonas sp. FBF16 partial 16S rRNA gene, isolate FB..

Score:1781 Evalue:0

Accession:HG805694.1

Alignment

Pseudomonas syringae pv. lapsa strain ATCC 10859, compl..

Score:1781 Evalue:0

Accession:CP013183.1

Alignment

Pseudomonas syringae pv. syringae strain W-1 16S riboso..

Score:1781 Evalue:0

Accession:KT008095.1

Alignment

Pseudomonas syringae strain PDD-70b-1 16S ribosomal RNA..

Score:1781 Evalue:0

Accession:KR922304.1

Alignment

Pseudomonas sp. PDD-59b-7 16S ribosomal RNA gene, compl..

Score:1781 Evalue:0

Accession:KR922145.1

Alignment

Pseudomonas syringae strain PDD-50b-5 16S ribosomal RNA..

Score:1781 Evalue:0

Accession:KR922071.1

Alignment

Pseudomonas syringae strain PDD-48b-5 16S ribosomal RNA..

Score:1781 Evalue:0

Accession:KR922059.1

Alignment

Pseudomonas syringae UMAF0158, complete genome

Score:1781 Evalue:0

Accession:CP005970.1

Alignment

Pseudomonas amygdali pv. tabaci strain BRST-1 16S ribos..

Score:1781 Evalue:0

Accession:KR476390.1

Alignment

Pseudomonas syringae pv. pisi strain ATCC 11043 16S rib..

Score:1781 Evalue:0

Accession:KP211411.1

Alignment

Pseudomonas syringae pv. pisi strain pea seed 1314 16S ..

Score:1781 Evalue:0

Accession:KP211410.1

Alignment

Pseudomonas congelans partial 16S rRNA gene, isolate 11..

Score:1781 Evalue:0

Accession:LN774224.1

Alignment

Pseudomonas syringae pv. syringae strain IZB212 16S rib..

Score:1781 Evalue:0

Accession:KP099974.1

Alignment

Pseudomonas syringae pv. syringae strain IZB201 16S rib..

Score:1781 Evalue:0

Accession:KP099970.1

Alignment

Pseudomonas syringae pv. tabaci strain YC-1 16S ribosom..

Score:1781 Evalue:0

Accession:KJ756715.1

Alignment

Pseudomonas syringae pv. glycinea strain EM1 16S riboso..

Score:1781 Evalue:0

Accession:KJ569377.1

Alignment

Pseudomonas syringae pv. glycinea strain LSC13 16S ribo..

Score:1781 Evalue:0

Accession:KJ569375.1

Alignment

Pseudomonas syringae pv. glycinea strain VT2 16S riboso..

Score:1781 Evalue:0

Accession:KJ569374.1

Alignment

## Descriptions

, Reading indexes 1-5, displaying indexes 1-5


Load next setPrevious Match

Sequences producing significant alignments:

Show all columns  of the table presenting sequences producing significant alignments 

Select:AllNone
Selected:0

Alignments
Download

FASTA (complete sequence)

FASTA (aligned sequences)

GenBank (complete sequence)

Hit Table (text)

Hit Table (CSV)

Text

XML

ASN.1

Continue
Cancel

GenBank 
Graphics
Distance tree of results
Multiple alignment
Show/hide columns of the table presenting sequences producing significant alignments 

Available columns

Description  
Max Score  
Total Score  
Coverage  
E-value  
IdentN  
Accession  
Restore Defaults
Ok
Cancel

Sequences producing significant alignments:

| Select for downloading or viewing reports | Description | Max score | Total score | Query cover | E value | Ident | Accession |
| --- | --- | --- | --- | --- | --- | --- | --- |
| 1Select seq MG722805.1 | Pseudomonas syringae pv. syringae strain B728a 16S ribosomal RNA gene, partial sequence | 1786 | 1786 | 98% | 0.0 | 99% | MG722805.1 |
| 2Select seq MG230266.1 | Pseudomonas syringae pv. malvacearum strain 480-2 16S ribosomal RNA gene, partial sequence | 1786 | 1786 | 98% | 0.0 | 99% | MG230266.1 |
| 3Select seq KR708944.1 | Pseudomonas savastanoi strain XJPY11 16S ribosomal RNA gene, partial sequence | 1786 | 1786 | 98% | 0.0 | 99% | KR708944.1 |
| 4Select seq KR708943.1 | Pseudomonas savastanoi strain XJPY13 16S ribosomal RNA gene, partial sequence | 1786 | 1786 | 98% | 0.0 | 99% | KR708943.1 |
| 5Select seq KR708880.1 | Pseudomonas savastanoi strain XJGY3 16S ribosomal RNA gene, partial sequence | 1786 | 1786 | 98% | 0.0 | 99% | KR708880.1 |
| 6Select seq LT629769.1 | Pseudomonas syringae strain 31R1 genome assembly, chromosome: I | 1786 | 8878 | 98% | 0.0 | 99% | LT629769.1 |
| 7Select seq KM187525.1 | Pseudomonas sp. HP7P 16S ribosomal RNA gene, partial sequence | 1786 | 1786 | 98% | 0.0 | 99% | KM187525.1 |
| 8Select seq KR476391.1 | Pseudomonas amygdali pv. tabaci strain YWTG-1 16S ribosomal RNA gene, partial sequence | 1786 | 1786 | 98% | 0.0 | 99% | KR476391.1 |
| 9Select seq CP005969.1 | Pseudomonas syringae pv. syringae B301D, complete genome | 1786 | 8934 | 98% | 0.0 | 99% | CP005969.1 |
| 10Select seq LC010244.1 | Pseudomonas syringae pv. tabaci gene for 16S ribosomal RNA, partial sequence, strain: GWF-311 | 1786 | 1786 | 98% | 0.0 | 99% | LC010244.1 |
| 11Select seq LC010243.1 | Pseudomonas syringae pv. tabaci gene for 16S ribosomal RNA, partial sequence, strain: GWF-121 | 1786 | 1786 | 98% | 0.0 | 99% | LC010243.1 |
| 12Select seq KJ756714.1 | Pseudomonas syringae pv. tabaci strain SWTG-1 16S ribosomal RNA gene, partial sequence | 1786 | 1786 | 98% | 0.0 | 99% | KJ756714.1 |
| 13Select seq KF888741.1 | Pseudomonas syringae pv. phaseolicola strain LDS9 16S ribosomal RNA gene, partial sequence | 1786 | 1786 | 98% | 0.0 | 99% | KF888741.1 |
| 14Select seq KF888740.1 | Pseudomonas syringae pv. phaseolicola strain LDS8 16S ribosomal RNA gene, partial sequence | 1786 | 1786 | 98% | 0.0 | 99% | KF888740.1 |
| 15Select seq KF888739.1 | Pseudomonas syringae pv. phaseolicola strain LDS7 16S ribosomal RNA gene, partial sequence | 1786 | 1786 | 98% | 0.0 | 99% | KF888739.1 |
| 16Select seq AB680556.1 | Pseudomonas syringae gene for 16S rRNA, partial sequence, strain: NBRC 14084 | 1786 | 1786 | 98% | 0.0 | 99% | AB680556.1 |
| 17Select seq AB680544.1 | Pseudomonas syringae gene for 16S rRNA, partial sequence, strain: NBRC 14054 | 1786 | 1786 | 98% | 0.0 | 99% | AB680544.1 |
| 18Select seq AM909659.1 | Pseudomonas sp. Act34 partial 16S rRNA gene, strain Act34 | 1786 | 1786 | 98% | 0.0 | 99% | AM909659.1 |
| 19Select seq AM909655.1 | Pseudomonas sp. Ina1 partial 16S rRNA gene, strain Ina1 | 1786 | 1786 | 98% | 0.0 | 99% | AM909655.1 |
| 20Select seq AM399034.1 | Pseudomonas syringae pv. coryli 16S rRNA gene, strain ISPaVe 595 | 1786 | 1786 | 98% | 0.0 | 99% | AM399034.1 |
| 21Select seq AB109218.1 | Pseudomonas syringae pv. pisi gene for 16S ribosomal RNA | 1786 | 1786 | 98% | 0.0 | 99% | AB109218.1 |
| 22Select seq CP000075.1 | Pseudomonas syringae pv. syringae B728a, complete genome | 1786 | 8911 | 98% | 0.0 | 99% | CP000075.1 |
| 23Select seq KR708885.1 | Pseudomonas savastanoi strain CGPY4 16S ribosomal RNA gene, partial sequence | 1783 | 1783 | 98% | 0.0 | 99% | KR708885.1 |
| 24Select seq CP026568.1 | Pseudomonas syringae pv. syringae strain Pss9097 chromosome, complete genome | 1781 | 8900 | 98% | 0.0 | 99% | CP026568.1 |
| 25Select seq LT962481.1 | Pseudomonas syringae pv. syringae isolate CFBP2118 genome assembly, chromosome: 1 | 1781 | 8900 | 98% | 0.0 | 99% | LT962481.1 |
| 26Select seq LT962480.1 | Pseudomonas syringae pv. syringae isolate CFBP4215 genome assembly, chromosome: 1 | 1781 | 8900 | 98% | 0.0 | 99% | LT962480.1 |
| 27Select seq MG230269.1 | Pseudomonas syringae pv. malvacearum strain 480-5 16S ribosomal RNA gene, partial sequence | 1781 | 1781 | 98% | 0.0 | 99% | MG230269.1 |
| 28Select seq MG230268.1 | Pseudomonas syringae pv. malvacearum strain 480-4 16S ribosomal RNA gene, partial sequence | 1781 | 1781 | 98% | 0.0 | 99% | MG230268.1 |
| 29Select seq MG230267.1 | Pseudomonas syringae pv. malvacearum strain 480-3 16S ribosomal RNA gene, partial sequence | 1781 | 1781 | 98% | 0.0 | 99% | MG230267.1 |
| 30Select seq MG230265.1 | Pseudomonas syringae pv. malvacearum strain 480-1 16S ribosomal RNA gene, partial sequence | 1781 | 1781 | 98% | 0.0 | 99% | MG230265.1 |
| 31Select seq MF693348.1 | Pseudomonas coronafaciens pv. striafaciens strain NCPPB 1898 16S ribosomal RNA gene, partial sequence | 1781 | 1781 | 98% | 0.0 | 99% | MF693348.1 |
| 32Select seq CP020351.1 | Pseudomonas amygdali pv. lachrymans strain NM002, complete genome | 1781 | 8884 | 98% | 0.0 | 99% | CP020351.1 |
| 33Select seq KR708873.1 | Paenibacillus polymyxa strain HGPJ-61 16S ribosomal RNA gene, partial sequence | 1781 | 1781 | 98% | 0.0 | 99% | KR708873.1 |
| 34Select seq KR708870.1 | Pseudomonas tremae strain HGPY-131 16S ribosomal RNA gene, partial sequence | 1781 | 1781 | 98% | 0.0 | 99% | KR708870.1 |
| 35Select seq KR708853.1 | Pseudomonas syringae strain 7GJ-14 16S ribosomal RNA gene, partial sequence | 1781 | 1781 | 98% | 0.0 | 99% | KR708853.1 |
| 36Select seq KR708837.1 | Pseudomonas syringae strain HGGY-6 16S ribosomal RNA gene, partial sequence | 1781 | 1781 | 98% | 0.0 | 99% | KR708837.1 |
| 37Select seq LC031818.1 | Pseudomonas syringae gene for 16S ribosomal RNA, partial sequence, strain: KS2 | 1781 | 1781 | 98% | 0.0 | 99% | LC031818.1 |
| 38Select seq LC031817.1 | Pseudomonas syringae gene for 16S ribosomal RNA, partial sequence, strain: KS1 | 1781 | 1781 | 98% | 0.0 | 99% | LC031817.1 |
| 39Select seq HG805694.1 | Pseudomonas sp. FBF16 partial 16S rRNA gene, isolate FBF16 | 1781 | 1781 | 98% | 0.0 | 99% | HG805694.1 |
| 40Select seq CP013183.1 | Pseudomonas syringae pv. lapsa strain ATCC 10859, complete genome | 1781 | 8867 | 98% | 0.0 | 99% | CP013183.1 |
| 41Select seq KT008095.1 | Pseudomonas syringae pv. syringae strain W-1 16S ribosomal RNA gene, partial sequence | 1781 | 1781 | 98% | 0.0 | 99% | KT008095.1 |
| 42Select seq KR922304.1 | Pseudomonas syringae strain PDD-70b-1 16S ribosomal RNA gene, complete sequence | 1781 | 1781 | 98% | 0.0 | 99% | KR922304.1 |
| 43Select seq KR922145.1 | Pseudomonas sp. PDD-59b-7 16S ribosomal RNA gene, complete sequence | 1781 | 1781 | 98% | 0.0 | 99% | KR922145.1 |
| 44Select seq KR922071.1 | Pseudomonas syringae strain PDD-50b-5 16S ribosomal RNA gene, complete sequence | 1781 | 1781 | 98% | 0.0 | 99% | KR922071.1 |
| 45Select seq KR922059.1 | Pseudomonas syringae strain PDD-48b-5 16S ribosomal RNA gene, complete sequence | 1781 | 1781 | 98% | 0.0 | 99% | KR922059.1 |
| 46Select seq CP005970.1 | Pseudomonas syringae UMAF0158, complete genome | 1781 | 8906 | 98% | 0.0 | 99% | CP005970.1 |
| 47Select seq KR476390.1 | Pseudomonas amygdali pv. tabaci strain BRST-1 16S ribosomal RNA gene, partial sequence | 1781 | 1781 | 98% | 0.0 | 99% | KR476390.1 |
| 48Select seq KP211411.1 | Pseudomonas syringae pv. pisi strain ATCC 11043 16S ribosomal RNA gene, 16S-23S ribosomal RNA intergenic spacer, and 23S ribosomal RNA gene, complete sequence | 1781 | 1781 | 98% | 0.0 | 99% | KP211411.1 |
| 49Select seq KP211410.1 | Pseudomonas syringae pv. pisi strain pea seed 1314 16S ribosomal RNA gene, partial sequence; 16S-23S ribosomal RNA intergenic spacer, complete sequence; and 23S ribosomal RNA gene, partial sequence | 1781 | 1781 | 98% | 0.0 | 99% | KP211410.1 |
| 50Select seq LN774224.1 | Pseudomonas congelans partial 16S rRNA gene, isolate 1111ARD14N6 | 1781 | 1781 | 98% | 0.0 | 99% | LN774224.1 |
| 51Select seq KP099974.1 | Pseudomonas syringae pv. syringae strain IZB212 16S ribosomal RNA gene, partial sequence | 1781 | 1781 | 98% | 0.0 | 99% | KP099974.1 |
| 52Select seq KP099970.1 | Pseudomonas syringae pv. syringae strain IZB201 16S ribosomal RNA gene, partial sequence | 1781 | 1781 | 98% | 0.0 | 99% | KP099970.1 |
| 53Select seq KJ756715.1 | Pseudomonas syringae pv. tabaci strain YC-1 16S ribosomal RNA gene, partial sequence | 1781 | 1781 | 98% | 0.0 | 99% | KJ756715.1 |
| 54Select seq KJ569377.1 | Pseudomonas syringae pv. glycinea strain EM1 16S ribosomal RNA gene, partial sequence | 1781 | 1781 | 98% | 0.0 | 99% | KJ569377.1 |
| 55Select seq KJ569375.1 | Pseudomonas syringae pv. glycinea strain LSC13 16S ribosomal RNA gene, partial sequence | 1781 | 1781 | 98% | 0.0 | 99% | KJ569375.1 |
| 56Select seq KJ569374.1 | Pseudomonas syringae pv. glycinea strain VT2 16S ribosomal RNA gene, partial sequence | 1781 | 1781 | 98% | 0.0 | 99% | KJ569374.1 |
| 57Select seq KJ569372.1 | Pseudomonas syringae pv. glycinea strain Q 16S ribosomal RNA gene, partial sequence | 1781 | 1781 | 98% | 0.0 | 99% | KJ569372.1 |
| 58Select seq KC776127.1 | Pseudomonas syringae strain CL2 16S ribosomal RNA gene, partial sequence | 1781 | 1781 | 98% | 0.0 | 99% | KC776127.1 |
| 59Select seq KC776126.1 | Pseudomonas syringae strain CL1 16S ribosomal RNA gene, partial sequence | 1781 | 1781 | 98% | 0.0 | 99% | KC776126.1 |
| 60Select seq KF147003.1 | Pseudomonas sp. R5SsM3P3C4 16S ribosomal RNA gene, partial sequence | 1781 | 1781 | 98% | 0.0 | 99% | KF147003.1 |
| 61Select seq KF147002.1 | Pseudomonas sp. R1SsM3P3C9 16S ribosomal RNA gene, partial sequence | 1781 | 1781 | 98% | 0.0 | 99% | KF147002.1 |
| 62Select seq KF147001.1 | Pseudomonas sp. R3ScM3P1C11 16S ribosomal RNA gene, partial sequence | 1781 | 1781 | 98% | 0.0 | 99% | KF147001.1 |
| 63Select seq KF147000.1 | Pseudomonas sp. R1SsM3P1C7 16S ribosomal RNA gene, partial sequence | 1781 | 1781 | 98% | 0.0 | 99% | KF147000.1 |
| 64Select seq KC860047.1 | Pseudomonas syringae pv. lachrymans strain njl18 16S ribosomal RNA gene, partial sequence | 1781 | 1781 | 98% | 0.0 | 99% | KC860047.1 |
| 65Select seq KC816630.1 | Pseudomonas syringae pv. syringae strain XJLX-2-2 16S ribosomal RNA gene, partial sequence | 1781 | 1781 | 98% | 0.0 | 99% | KC816630.1 |
| 66Select seq KC816629.1 | Pseudomonas syringae pv. syringae strain XJLX-3-8 16S ribosomal RNA gene, partial sequence | 1781 | 1781 | 98% | 0.0 | 99% | KC816629.1 |
| 67Select seq KC816628.1 | Pseudomonas syringae pv. syringae strain XJLX-3-7 16S ribosomal RNA gene, partial sequence | 1781 | 1781 | 98% | 0.0 | 99% | KC816628.1 |
| 68Select seq KC816626.1 | Pseudomonas syringae pv. syringae strain XJLX-4-2 16S ribosomal RNA gene, partial sequence | 1781 | 1781 | 98% | 0.0 | 99% | KC816626.1 |
| 69Select seq KC311258.1 | Pseudomonas sp. GCh5Fc 16S ribosomal RNA gene, partial sequence | 1781 | 1781 | 98% | 0.0 | 99% | KC311258.1 |
| 70Select seq KC311257.1 | Pseudomonas sp. BXIN3 16S ribosomal RNA gene, partial sequence | 1781 | 1781 | 98% | 0.0 | 99% | KC311257.1 |
| 71Select seq KC311256.1 | Pseudomonas sp. HCh1a 16S ribosomal RNA gene, partial sequence | 1781 | 1781 | 98% | 0.0 | 99% | KC311256.1 |
| 72Select seq KC311255.1 | Pseudomonas sp. PCa2a 16S ribosomal RNA gene, partial sequence | 1781 | 1781 | 98% | 0.0 | 99% | KC311255.1 |
| 73Select seq KC311254.1 | Pseudomonas sp. BXIN4 16S ribosomal RNA gene, partial sequence | 1781 | 1781 | 98% | 0.0 | 99% | KC311254.1 |
| 74Select seq JX474752.1 | Pseudomonas syringae strain XJKEL-1 16S ribosomal RNA gene, partial sequence | 1781 | 1781 | 98% | 0.0 | 99% | JX474752.1 |
| 75Select seq JQ845902.1 | Pseudomonas syringae strain 61 16S ribosomal RNA gene, partial sequence | 1781 | 1781 | 98% | 0.0 | 99% | JQ845902.1 |
| 76Select seq JQ320090.1 | Pseudomonas congelans strain XjGEB-29 16S ribosomal RNA gene, partial sequence | 1781 | 1781 | 98% | 0.0 | 99% | JQ320090.1 |
| 77Select seq JQ071937.1 | Pseudomonas syringae pv. syringae strain KFB 93 16S ribosomal RNA gene, partial sequence | 1781 | 1781 | 98% | 0.0 | 99% | JQ071937.1 |
| 78Select seq JQ071934.1 | Pseudomonas syringae pv. syringae strain KFB 80 16S ribosomal RNA gene, partial sequence | 1781 | 1781 | 98% | 0.0 | 99% | JQ071934.1 |
| 79Select seq AB680543.1 | Pseudomonas syringae gene for 16S rRNA, partial sequence, strain: NBRC 14053 | 1781 | 1781 | 98% | 0.0 | 99% | AB680543.1 |
| 80Select seq AB680547.1 | Pseudomonas syringae gene for 16S rRNA, partial sequence, strain: NBRC 14075 | 1781 | 1781 | 98% | 0.0 | 99% | AB680547.1 |
| 81Select seq NR\_117820.1 | Pseudomonas syringae strain ICMP 3023 16S ribosomal RNA gene, partial sequence | 1781 | 1781 | 98% | 0.0 | 99% | NR\_117820.1 |
| 82Select seq GU997636.1 | Pseudomonas syringae pv. morsprunorum strain MAFF301436 16S ribosomal RNA gene, partial sequence | 1781 | 1781 | 98% | 0.0 | 99% | GU997636.1 |
| 83Select seq NR\_028985.1 | Pseudomonas congelans strain P 538/23 16S ribosomal RNA gene, partial sequence | 1781 | 1781 | 98% | 0.0 | 99% | NR\_028985.1 |
| 84Select seq FN421697.1 | Uncultured bacterium partial 16S rRNA gene, clone 3\_F04 | 1781 | 1781 | 98% | 0.0 | 99% | FN421697.1 |
| 85Select seq FN421655.1 | Uncultured bacterium partial 16S rRNA gene, clone 13\_H12 | 1781 | 1781 | 98% | 0.0 | 99% | FN421655.1 |
| 86Select seq FN421637.1 | Uncultured bacterium partial 16S rRNA gene, clone 13\_F04 | 1781 | 1781 | 98% | 0.0 | 99% | FN421637.1 |
| 87Select seq FN421630.1 | Uncultured bacterium partial 16S rRNA gene, clone 13\_E05 | 1781 | 1781 | 98% | 0.0 | 99% | FN421630.1 |
| 88Select seq FN421626.1 | Uncultured bacterium partial 16S rRNA gene, clone 13\_D11 | 1781 | 1781 | 98% | 0.0 | 99% | FN421626.1 |
| 89Select seq FN421615.1 | Uncultured bacterium partial 16S rRNA gene, clone 13\_C06 | 1781 | 1781 | 98% | 0.0 | 99% | FN421615.1 |
| 90Select seq FJ755788.1 | Pseudomonas syringae pv. tabaci strain BC2366 16S ribosomal RNA gene, partial sequence | 1781 | 1781 | 98% | 0.0 | 99% | FJ755788.1 |
| 91Select seq AM399036.1 | Pseudomonas syringae pv. syringae 16S rRNA gene, strain NCPPB 3869 | 1781 | 1781 | 98% | 0.0 | 99% | AM399036.1 |
| 92Select seq AY574913.1 | Pseudomonas syringae strain PNA29.1a 16S ribosomal RNA gene, partial sequence | 1781 | 1781 | 98% | 0.0 | 99% | AY574913.1 |
| 93Select seq AJ889841.1 | Pseudomonas syringae pv. coryli 16S rRNA gene, strain NCPPB 4273 | 1781 | 1781 | 98% | 0.0 | 99% | AJ889841.1 |
| 94Select seq AJ970169.1 | Pseudomonas sp. LPPA C152 partial 16S rRNA gene, strain LPPA C152 | 1781 | 1781 | 98% | 0.0 | 99% | AJ970169.1 |
| 95Select seq AJ970168.1 | Pseudomonas sp. LPPA C117 partial 16S rRNA gene, strain LPPA C117 | 1781 | 1781 | 98% | 0.0 | 99% | AJ970168.1 |
| 96Select seq AJ492828.1 | Pseudomonas congelans partial 16S rRNA gene, type strain DSM 14939T | 1781 | 1781 | 98% | 0.0 | 99% | AJ492828.1 |
| 97Select seq AB001443.1 | Pseudomonas syringae pv. glycinea gene for 16S rRNA | 1781 | 1781 | 98% | 0.0 | 99% | AB001443.1 |
| 98Select seq KP099972.1 | Pseudomonas syringae pv. syringae strain IZB208 16S ribosomal RNA gene, partial sequence | 1779 | 1779 | 98% | 0.0 | 99% | KP099972.1 |
| 99Select seq KP099969.1 | Pseudomonas syringae pv. syringae strain IZB200 16S ribosomal RNA gene, partial sequence | 1779 | 1779 | 98% | 0.0 | 99% | KP099969.1 |
| 100Select seq AB680305.1 | Pseudomonas syringae gene for 16S rRNA, partial sequence, strain: NBRC 12655 | 1777 | 1777 | 98% | 0.0 | 99% | AB680305.1 |


## Alignments

Loading alignment... for sequences gi|1317079578,gi|1268088842,gi|967512029,gi|967512028,gi|967511965 Reading indexes 1-5

Download

FASTA (complete sequence)

FASTA (aligned sequences)

GenBank (complete sequence)

Continue
Cancel

GenBankGraphics

Next
Previous
Descriptions

Pseudomonas syringae pv. syringae strain B728a 16S ribosomal RNA gene, partial sequence

Sequence ID: MG722805.1Length: 1538Number of Matches: 1

Related Information

Range 1: 59 to 1036GenBankGraphics

Next Match
Previous Match
First Match

Alignment statistics for match #1

| Score | Expect | Identities | Gaps | Strand | Frame |
| --- | --- | --- | --- | --- | --- |
| 1786 bits(967) | 0.0() | 974/979(99%) | 1/979(0%) | Plus/Plus |  |

Features:

```
Query  10    AGTCGAGCGGCAGCACGGGTACTTGTACCTGGTGGCGAGCGGCGGACGGGTGAGTAATGC  69
             ||||||||||||||||||||||||||||||||||||||||||||||||||||||||||||
Sbjct  59    AGTCGAGCGGCAGCACGGGTACTTGTACCTGGTGGCGAGCGGCGGACGGGTGAGTAATGC  118

Query  70    CTAGGAATCTGCCTGGTAGTGGGGGATAACGCTCGGAAACGGACGCTAATACCGCATACG  129
             ||||||||||||||||||||||||||||||||||||||||||||||||||||||||||||
Sbjct  119   CTAGGAATCTGCCTGGTAGTGGGGGATAACGCTCGGAAACGGACGCTAATACCGCATACG  178

Query  130   TCCTACGGGAGAAAGCAGGGGACCTTCGGGCCTTGCGCTATCAGATGAGCCTAGGTCGGA  189
             ||||||||||||||||||||||||||||||||||||||||||||||||||||||||||||
Sbjct  179   TCCTACGGGAGAAAGCAGGGGACCTTCGGGCCTTGCGCTATCAGATGAGCCTAGGTCGGA  238

Query  190   TTAGCTAGTTGGTGAGGTAATGGCTCACCAAGGCGACGATCCGTAACTGGTCTGAGAGGA  249
             ||||||||||||||||||||||||||||||||||||||||||||||||||||||||||||
Sbjct  239   TTAGCTAGTTGGTGAGGTAATGGCTCACCAAGGCGACGATCCGTAACTGGTCTGAGAGGA  298

Query  250   TGATCAGTCACACTGGAACTGAGACACGGTCCAGACTCCTACGGGAGGCAGCAGTGGGGA  309
             ||||||||||||||||||||||||||||||||||||||||||||||||||||||||||||
Sbjct  299   TGATCAGTCACACTGGAACTGAGACACGGTCCAGACTCCTACGGGAGGCAGCAGTGGGGA  358

Query  310   ATATTGGACAATGGGCGAAAGCCTGATCCAGCCATGCCGCGTGTGTGAAGAAGGTCTTCG  369
             ||||||||||||||||||||||||||||||||||||||||||||||||||||||||||||
Sbjct  359   ATATTGGACAATGGGCGAAAGCCTGATCCAGCCATGCCGCGTGTGTGAAGAAGGTCTTCG  418

Query  370   GATTGTAAAGCACTTTAAGTTGGGAGGAAGGGCAGTTACCTAATACGTGATTGTTTTGAC  429
             ||||||||||||||||||||||||||||||||||||||||||||||||||||||||||||
Sbjct  419   GATTGTAAAGCACTTTAAGTTGGGAGGAAGGGCAGTTACCTAATACGTGATTGTTTTGAC  478

Query  430   GTTACCGACAGAATAAGCACCGGCTAACTCTGTGCCAGCAGCCGCGGTAATACAGAGGGT  489
             ||||||||||||||||||||||||||||||||||||||||||||||||||||||||||||
Sbjct  479   GTTACCGACAGAATAAGCACCGGCTAACTCTGTGCCAGCAGCCGCGGTAATACAGAGGGT  538

Query  490   GCAAGCGTTAATCGGAATTACTGGGCGTAAAGCGCGCGTAGGTGGTTTGTTAAGTTGAAT  549
             ||||||||||||||||||||||||||||||||||||||||||||||||||||||||||||
Sbjct  539   GCAAGCGTTAATCGGAATTACTGGGCGTAAAGCGCGCGTAGGTGGTTTGTTAAGTTGAAT  598

Query  550   GTGAAATCCCCGGGCTCAACCTGGGAACTGCATCCAAAACTGGCAAGCTAGAGTATGGTA  609
             ||||||||||||||||||||||||||||||||||||||||||||||||||||||||||||
Sbjct  599   GTGAAATCCCCGGGCTCAACCTGGGAACTGCATCCAAAACTGGCAAGCTAGAGTATGGTA  658

Query  610   GAGGGTGGTGGAATTTCCTGTGTAGCGGTGAAATGCGTAGATATAGGAAGGAACACCAGT  669
             ||||||||||||||||||||||||||||||||||||||||||||||||||||||||||||
Sbjct  659   GAGGGTGGTGGAATTTCCTGTGTAGCGGTGAAATGCGTAGATATAGGAAGGAACACCAGT  718

Query  670   GGCGAAGGCGACCACCTGGACTGATACTGACACTGAGGTGCGAAAGCGTGGGGAGCAAAC  729
             ||||||||||||||||||||||||||||||||||||||||||||||||||||||||||||
Sbjct  719   GGCGAAGGCGACCACCTGGACTGATACTGACACTGAGGTGCGAAAGCGTGGGGAGCAAAC  778

Query  730   AGGATTAGATACCCTGGTAGTCCACGCCGTAAACGATGTCAACTAGCCGTTGGGAGCCTT  789
             ||||||||||||||||||||||||||||||||||||||||||||||||||||||||||||
Sbjct  779   AGGATTAGATACCCTGGTAGTCCACGCCGTAAACGATGTCAACTAGCCGTTGGGAGCCTT  838

Query  790   GAGCTCTTAGTGGCGCAGCTAACGCATTAAGTTGACCGCCTGGGGGAGTACGGCCGCAAG  849
             ||||||||||||||||||||||||||||||||||||||||| ||||||||||||||||||
Sbjct  839   GAGCTCTTAGTGGCGCAGCTAACGCATTAAGTTGACCGCCT-GGGGAGTACGGCCGCAAG  897

Query  850   GTTAAAACTCAAATGAATTGACGGGGGCCCGCACAAGCGGTGGAGCATGTGGTTTAATTC  909
             ||||||||||||||||||||||||||||||||||||||||||||||||||||||||||||
Sbjct  898   GTTAAAACTCAAATGAATTGACGGGGGCCCGCACAAGCGGTGGAGCATGTGGTTTAATTC  957

Query  910   GAAGCAACGCGAAGAACCTTACCAGGCCTTGACATCCAATGAACTTTCCNNAGATGGATT  969
             |||||||||||||||||||||||||||||||||||||||||||||||||  |||||||||
Sbjct  958   GAAGCAACGCGAAGAACCTTACCAGGCCTTGACATCCAATGAACTTTCCAGAGATGGATT  1017

Query  970   GGTGCCTTCGGGNGNATTG  988
             |||||||||||| | ||||
Sbjct  1018  GGTGCCTTCGGGAGCATTG  1036
```

Download

FASTA (complete sequence)

FASTA (aligned sequences)

GenBank (complete sequence)

Continue
Cancel

GenBankGraphics

Next
Previous
Descriptions

Pseudomonas syringae pv. malvacearum strain 480-2 16S ribosomal RNA gene, partial sequence

Sequence ID: MG230266.1Length: 1535Number of Matches: 1

Related Information

Range 1: 57 to 1034GenBankGraphics

Next Match
Previous Match
First Match

Alignment statistics for match #1

| Score | Expect | Identities | Gaps | Strand | Frame |
| --- | --- | --- | --- | --- | --- |
| 1786 bits(967) | 0.0() | 974/979(99%) | 1/979(0%) | Plus/Plus |  |

Features:

```
Query  10    AGTCGAGCGGCAGCACGGGTACTTGTACCTGGTGGCGAGCGGCGGACGGGTGAGTAATGC  69
             ||||||||||||||||||||||||||||||||||||||||||||||||||||||||||||
Sbjct  57    AGTCGAGCGGCAGCACGGGTACTTGTACCTGGTGGCGAGCGGCGGACGGGTGAGTAATGC  116

Query  70    CTAGGAATCTGCCTGGTAGTGGGGGATAACGCTCGGAAACGGACGCTAATACCGCATACG  129
             ||||||||||||||||||||||||||||||||||||||||||||||||||||||||||||
Sbjct  117   CTAGGAATCTGCCTGGTAGTGGGGGATAACGCTCGGAAACGGACGCTAATACCGCATACG  176

Query  130   TCCTACGGGAGAAAGCAGGGGACCTTCGGGCCTTGCGCTATCAGATGAGCCTAGGTCGGA  189
             ||||||||||||||||||||||||||||||||||||||||||||||||||||||||||||
Sbjct  177   TCCTACGGGAGAAAGCAGGGGACCTTCGGGCCTTGCGCTATCAGATGAGCCTAGGTCGGA  236

Query  190   TTAGCTAGTTGGTGAGGTAATGGCTCACCAAGGCGACGATCCGTAACTGGTCTGAGAGGA  249
             ||||||||||||||||||||||||||||||||||||||||||||||||||||||||||||
Sbjct  237   TTAGCTAGTTGGTGAGGTAATGGCTCACCAAGGCGACGATCCGTAACTGGTCTGAGAGGA  296

Query  250   TGATCAGTCACACTGGAACTGAGACACGGTCCAGACTCCTACGGGAGGCAGCAGTGGGGA  309
             ||||||||||||||||||||||||||||||||||||||||||||||||||||||||||||
Sbjct  297   TGATCAGTCACACTGGAACTGAGACACGGTCCAGACTCCTACGGGAGGCAGCAGTGGGGA  356

Query  310   ATATTGGACAATGGGCGAAAGCCTGATCCAGCCATGCCGCGTGTGTGAAGAAGGTCTTCG  369
             ||||||||||||||||||||||||||||||||||||||||||||||||||||||||||||
Sbjct  357   ATATTGGACAATGGGCGAAAGCCTGATCCAGCCATGCCGCGTGTGTGAAGAAGGTCTTCG  416

Query  370   GATTGTAAAGCACTTTAAGTTGGGAGGAAGGGCAGTTACCTAATACGTGATTGTTTTGAC  429
             ||||||||||||||||||||||||||||||||||||||||||||||||||||||||||||
Sbjct  417   GATTGTAAAGCACTTTAAGTTGGGAGGAAGGGCAGTTACCTAATACGTGATTGTTTTGAC  476

Query  430   GTTACCGACAGAATAAGCACCGGCTAACTCTGTGCCAGCAGCCGCGGTAATACAGAGGGT  489
             ||||||||||||||||||||||||||||||||||||||||||||||||||||||||||||
Sbjct  477   GTTACCGACAGAATAAGCACCGGCTAACTCTGTGCCAGCAGCCGCGGTAATACAGAGGGT  536

Query  490   GCAAGCGTTAATCGGAATTACTGGGCGTAAAGCGCGCGTAGGTGGTTTGTTAAGTTGAAT  549
             ||||||||||||||||||||||||||||||||||||||||||||||||||||||||||||
Sbjct  537   GCAAGCGTTAATCGGAATTACTGGGCGTAAAGCGCGCGTAGGTGGTTTGTTAAGTTGAAT  596

Query  550   GTGAAATCCCCGGGCTCAACCTGGGAACTGCATCCAAAACTGGCAAGCTAGAGTATGGTA  609
             ||||||||||||||||||||||||||||||||||||||||||||||||||||||||||||
Sbjct  597   GTGAAATCCCCGGGCTCAACCTGGGAACTGCATCCAAAACTGGCAAGCTAGAGTATGGTA  656

Query  610   GAGGGTGGTGGAATTTCCTGTGTAGCGGTGAAATGCGTAGATATAGGAAGGAACACCAGT  669
             ||||||||||||||||||||||||||||||||||||||||||||||||||||||||||||
Sbjct  657   GAGGGTGGTGGAATTTCCTGTGTAGCGGTGAAATGCGTAGATATAGGAAGGAACACCAGT  716

Query  670   GGCGAAGGCGACCACCTGGACTGATACTGACACTGAGGTGCGAAAGCGTGGGGAGCAAAC  729
             ||||||||||||||||||||||||||||||||||||||||||||||||||||||||||||
Sbjct  717   GGCGAAGGCGACCACCTGGACTGATACTGACACTGAGGTGCGAAAGCGTGGGGAGCAAAC  776

Query  730   AGGATTAGATACCCTGGTAGTCCACGCCGTAAACGATGTCAACTAGCCGTTGGGAGCCTT  789
             ||||||||||||||||||||||||||||||||||||||||||||||||||||||||||||
Sbjct  777   AGGATTAGATACCCTGGTAGTCCACGCCGTAAACGATGTCAACTAGCCGTTGGGAGCCTT  836

Query  790   GAGCTCTTAGTGGCGCAGCTAACGCATTAAGTTGACCGCCTGGGGGAGTACGGCCGCAAG  849
             ||||||||||||||||||||||||||||||||||||||||| ||||||||||||||||||
Sbjct  837   GAGCTCTTAGTGGCGCAGCTAACGCATTAAGTTGACCGCCT-GGGGAGTACGGCCGCAAG  895

Query  850   GTTAAAACTCAAATGAATTGACGGGGGCCCGCACAAGCGGTGGAGCATGTGGTTTAATTC  909
             ||||||||||||||||||||||||||||||||||||||||||||||||||||||||||||
Sbjct  896   GTTAAAACTCAAATGAATTGACGGGGGCCCGCACAAGCGGTGGAGCATGTGGTTTAATTC  955

Query  910   GAAGCAACGCGAAGAACCTTACCAGGCCTTGACATCCAATGAACTTTCCNNAGATGGATT  969
             |||||||||||||||||||||||||||||||||||||||||||||||||  |||||||||
Sbjct  956   GAAGCAACGCGAAGAACCTTACCAGGCCTTGACATCCAATGAACTTTCCAGAGATGGATT  1015

Query  970   GGTGCCTTCGGGNGNATTG  988
             |||||||||||| | ||||
Sbjct  1016  GGTGCCTTCGGGAGCATTG  1034
```

Download

FASTA (complete sequence)

FASTA (aligned sequences)

GenBank (complete sequence)

Continue
Cancel

GenBankGraphics

Next
Previous
Descriptions

Pseudomonas savastanoi strain XJPY11 16S ribosomal RNA gene, partial sequence

Sequence ID: KR708944.1Length: 1405Number of Matches: 1

Related Information

Range 1: 6 to 983GenBankGraphics

Next Match
Previous Match
First Match

Alignment statistics for match #1

| Score | Expect | Identities | Gaps | Strand | Frame |
| --- | --- | --- | --- | --- | --- |
| 1786 bits(967) | 0.0() | 974/979(99%) | 1/979(0%) | Plus/Plus |  |

Features:

```
Query  10   AGTCGAGCGGCAGCACGGGTACTTGTACCTGGTGGCGAGCGGCGGACGGGTGAGTAATGC  69
            ||||||||||||||||||||||||||||||||||||||||||||||||||||||||||||
Sbjct  6    AGTCGAGCGGCAGCACGGGTACTTGTACCTGGTGGCGAGCGGCGGACGGGTGAGTAATGC  65

Query  70   CTAGGAATCTGCCTGGTAGTGGGGGATAACGCTCGGAAACGGACGCTAATACCGCATACG  129
            ||||||||||||||||||||||||||||||||||||||||||||||||||||||||||||
Sbjct  66   CTAGGAATCTGCCTGGTAGTGGGGGATAACGCTCGGAAACGGACGCTAATACCGCATACG  125

Query  130  TCCTACGGGAGAAAGCAGGGGACCTTCGGGCCTTGCGCTATCAGATGAGCCTAGGTCGGA  189
            ||||||||||||||||||||||||||||||||||||||||||||||||||||||||||||
Sbjct  126  TCCTACGGGAGAAAGCAGGGGACCTTCGGGCCTTGCGCTATCAGATGAGCCTAGGTCGGA  185

Query  190  TTAGCTAGTTGGTGAGGTAATGGCTCACCAAGGCGACGATCCGTAACTGGTCTGAGAGGA  249
            ||||||||||||||||||||||||||||||||||||||||||||||||||||||||||||
Sbjct  186  TTAGCTAGTTGGTGAGGTAATGGCTCACCAAGGCGACGATCCGTAACTGGTCTGAGAGGA  245

Query  250  TGATCAGTCACACTGGAACTGAGACACGGTCCAGACTCCTACGGGAGGCAGCAGTGGGGA  309
            ||||||||||||||||||||||||||||||||||||||||||||||||||||||||||||
Sbjct  246  TGATCAGTCACACTGGAACTGAGACACGGTCCAGACTCCTACGGGAGGCAGCAGTGGGGA  305

Query  310  ATATTGGACAATGGGCGAAAGCCTGATCCAGCCATGCCGCGTGTGTGAAGAAGGTCTTCG  369
            ||||||||||||||||||||||||||||||||||||||||||||||||||||||||||||
Sbjct  306  ATATTGGACAATGGGCGAAAGCCTGATCCAGCCATGCCGCGTGTGTGAAGAAGGTCTTCG  365

Query  370  GATTGTAAAGCACTTTAAGTTGGGAGGAAGGGCAGTTACCTAATACGTGATTGTTTTGAC  429
            ||||||||||||||||||||||||||||||||||||||||||||||||||||||||||||
Sbjct  366  GATTGTAAAGCACTTTAAGTTGGGAGGAAGGGCAGTTACCTAATACGTGATTGTTTTGAC  425

Query  430  GTTACCGACAGAATAAGCACCGGCTAACTCTGTGCCAGCAGCCGCGGTAATACAGAGGGT  489
            ||||||||||||||||||||||||||||||||||||||||||||||||||||||||||||
Sbjct  426  GTTACCGACAGAATAAGCACCGGCTAACTCTGTGCCAGCAGCCGCGGTAATACAGAGGGT  485

Query  490  GCAAGCGTTAATCGGAATTACTGGGCGTAAAGCGCGCGTAGGTGGTTTGTTAAGTTGAAT  549
            ||||||||||||||||||||||||||||||||||||||||||||||||||||||||||||
Sbjct  486  GCAAGCGTTAATCGGAATTACTGGGCGTAAAGCGCGCGTAGGTGGTTTGTTAAGTTGAAT  545

Query  550  GTGAAATCCCCGGGCTCAACCTGGGAACTGCATCCAAAACTGGCAAGCTAGAGTATGGTA  609
            ||||||||||||||||||||||||||||||||||||||||||||||||||||||||||||
Sbjct  546  GTGAAATCCCCGGGCTCAACCTGGGAACTGCATCCAAAACTGGCAAGCTAGAGTATGGTA  605

Query  610  GAGGGTGGTGGAATTTCCTGTGTAGCGGTGAAATGCGTAGATATAGGAAGGAACACCAGT  669
            ||||||||||||||||||||||||||||||||||||||||||||||||||||||||||||
Sbjct  606  GAGGGTGGTGGAATTTCCTGTGTAGCGGTGAAATGCGTAGATATAGGAAGGAACACCAGT  665

Query  670  GGCGAAGGCGACCACCTGGACTGATACTGACACTGAGGTGCGAAAGCGTGGGGAGCAAAC  729
            ||||||||||||||||||||||||||||||||||||||||||||||||||||||||||||
Sbjct  666  GGCGAAGGCGACCACCTGGACTGATACTGACACTGAGGTGCGAAAGCGTGGGGAGCAAAC  725

Query  730  AGGATTAGATACCCTGGTAGTCCACGCCGTAAACGATGTCAACTAGCCGTTGGGAGCCTT  789
            ||||||||||||||||||||||||||||||||||||||||||||||||||||||||||||
Sbjct  726  AGGATTAGATACCCTGGTAGTCCACGCCGTAAACGATGTCAACTAGCCGTTGGGAGCCTT  785

Query  790  GAGCTCTTAGTGGCGCAGCTAACGCATTAAGTTGACCGCCTGGGGGAGTACGGCCGCAAG  849
            ||||||||||||||||||||||||||||||||||||||||| ||||||||||||||||||
Sbjct  786  GAGCTCTTAGTGGCGCAGCTAACGCATTAAGTTGACCGCCT-GGGGAGTACGGCCGCAAG  844

Query  850  GTTAAAACTCAAATGAATTGACGGGGGCCCGCACAAGCGGTGGAGCATGTGGTTTAATTC  909
            ||||||||||||||||||||||||||||||||||||||||||||||||||||||||||||
Sbjct  845  GTTAAAACTCAAATGAATTGACGGGGGCCCGCACAAGCGGTGGAGCATGTGGTTTAATTC  904

Query  910  GAAGCAACGCGAAGAACCTTACCAGGCCTTGACATCCAATGAACTTTCCNNAGATGGATT  969
            |||||||||||||||||||||||||||||||||||||||||||||||||  |||||||||
Sbjct  905  GAAGCAACGCGAAGAACCTTACCAGGCCTTGACATCCAATGAACTTTCCAGAGATGGATT  964

Query  970  GGTGCCTTCGGGNGNATTG  988
            |||||||||||| | ||||
Sbjct  965  GGTGCCTTCGGGAGCATTG  983
```

Download

FASTA (complete sequence)

FASTA (aligned sequences)

GenBank (complete sequence)

Continue
Cancel

GenBankGraphics

Next
Previous
Descriptions

Pseudomonas savastanoi strain XJPY13 16S ribosomal RNA gene, partial sequence

Sequence ID: KR708943.1Length: 1406Number of Matches: 1

#### See 1 more title(s)

Bacillus methylotrophicus strain XJPJ14 16S ribosomal RNA gene, partial sequence

Sequence ID: KR708947.1Length: 1406Number of Matches:

Related Information

Range 1: 7 to 984GenBankGraphics

Next Match
Previous Match
First Match

Alignment statistics for match #1

| Score | Expect | Identities | Gaps | Strand | Frame |
| --- | --- | --- | --- | --- | --- |
| 1786 bits(967) | 0.0() | 974/979(99%) | 1/979(0%) | Plus/Plus |  |

Features:

```
Query  10   AGTCGAGCGGCAGCACGGGTACTTGTACCTGGTGGCGAGCGGCGGACGGGTGAGTAATGC  69
            ||||||||||||||||||||||||||||||||||||||||||||||||||||||||||||
Sbjct  7    AGTCGAGCGGCAGCACGGGTACTTGTACCTGGTGGCGAGCGGCGGACGGGTGAGTAATGC  66

Query  70   CTAGGAATCTGCCTGGTAGTGGGGGATAACGCTCGGAAACGGACGCTAATACCGCATACG  129
            ||||||||||||||||||||||||||||||||||||||||||||||||||||||||||||
Sbjct  67   CTAGGAATCTGCCTGGTAGTGGGGGATAACGCTCGGAAACGGACGCTAATACCGCATACG  126

Query  130  TCCTACGGGAGAAAGCAGGGGACCTTCGGGCCTTGCGCTATCAGATGAGCCTAGGTCGGA  189
            ||||||||||||||||||||||||||||||||||||||||||||||||||||||||||||
Sbjct  127  TCCTACGGGAGAAAGCAGGGGACCTTCGGGCCTTGCGCTATCAGATGAGCCTAGGTCGGA  186

Query  190  TTAGCTAGTTGGTGAGGTAATGGCTCACCAAGGCGACGATCCGTAACTGGTCTGAGAGGA  249
            ||||||||||||||||||||||||||||||||||||||||||||||||||||||||||||
Sbjct  187  TTAGCTAGTTGGTGAGGTAATGGCTCACCAAGGCGACGATCCGTAACTGGTCTGAGAGGA  246

Query  250  TGATCAGTCACACTGGAACTGAGACACGGTCCAGACTCCTACGGGAGGCAGCAGTGGGGA  309
            ||||||||||||||||||||||||||||||||||||||||||||||||||||||||||||
Sbjct  247  TGATCAGTCACACTGGAACTGAGACACGGTCCAGACTCCTACGGGAGGCAGCAGTGGGGA  306

Query  310  ATATTGGACAATGGGCGAAAGCCTGATCCAGCCATGCCGCGTGTGTGAAGAAGGTCTTCG  369
            ||||||||||||||||||||||||||||||||||||||||||||||||||||||||||||
Sbjct  307  ATATTGGACAATGGGCGAAAGCCTGATCCAGCCATGCCGCGTGTGTGAAGAAGGTCTTCG  366

Query  370  GATTGTAAAGCACTTTAAGTTGGGAGGAAGGGCAGTTACCTAATACGTGATTGTTTTGAC  429
            ||||||||||||||||||||||||||||||||||||||||||||||||||||||||||||
Sbjct  367  GATTGTAAAGCACTTTAAGTTGGGAGGAAGGGCAGTTACCTAATACGTGATTGTTTTGAC  426

Query  430  GTTACCGACAGAATAAGCACCGGCTAACTCTGTGCCAGCAGCCGCGGTAATACAGAGGGT  489
            ||||||||||||||||||||||||||||||||||||||||||||||||||||||||||||
Sbjct  427  GTTACCGACAGAATAAGCACCGGCTAACTCTGTGCCAGCAGCCGCGGTAATACAGAGGGT  486

Query  490  GCAAGCGTTAATCGGAATTACTGGGCGTAAAGCGCGCGTAGGTGGTTTGTTAAGTTGAAT  549
            ||||||||||||||||||||||||||||||||||||||||||||||||||||||||||||
Sbjct  487  GCAAGCGTTAATCGGAATTACTGGGCGTAAAGCGCGCGTAGGTGGTTTGTTAAGTTGAAT  546

Query  550  GTGAAATCCCCGGGCTCAACCTGGGAACTGCATCCAAAACTGGCAAGCTAGAGTATGGTA  609
            ||||||||||||||||||||||||||||||||||||||||||||||||||||||||||||
Sbjct  547  GTGAAATCCCCGGGCTCAACCTGGGAACTGCATCCAAAACTGGCAAGCTAGAGTATGGTA  606

Query  610  GAGGGTGGTGGAATTTCCTGTGTAGCGGTGAAATGCGTAGATATAGGAAGGAACACCAGT  669
            ||||||||||||||||||||||||||||||||||||||||||||||||||||||||||||
Sbjct  607  GAGGGTGGTGGAATTTCCTGTGTAGCGGTGAAATGCGTAGATATAGGAAGGAACACCAGT  666

Query  670  GGCGAAGGCGACCACCTGGACTGATACTGACACTGAGGTGCGAAAGCGTGGGGAGCAAAC  729
            ||||||||||||||||||||||||||||||||||||||||||||||||||||||||||||
Sbjct  667  GGCGAAGGCGACCACCTGGACTGATACTGACACTGAGGTGCGAAAGCGTGGGGAGCAAAC  726

Query  730  AGGATTAGATACCCTGGTAGTCCACGCCGTAAACGATGTCAACTAGCCGTTGGGAGCCTT  789
            ||||||||||||||||||||||||||||||||||||||||||||||||||||||||||||
Sbjct  727  AGGATTAGATACCCTGGTAGTCCACGCCGTAAACGATGTCAACTAGCCGTTGGGAGCCTT  786

Query  790  GAGCTCTTAGTGGCGCAGCTAACGCATTAAGTTGACCGCCTGGGGGAGTACGGCCGCAAG  849
            ||||||||||||||||||||||||||||||||||||||||| ||||||||||||||||||
Sbjct  787  GAGCTCTTAGTGGCGCAGCTAACGCATTAAGTTGACCGCCT-GGGGAGTACGGCCGCAAG  845

Query  850  GTTAAAACTCAAATGAATTGACGGGGGCCCGCACAAGCGGTGGAGCATGTGGTTTAATTC  909
            ||||||||||||||||||||||||||||||||||||||||||||||||||||||||||||
Sbjct  846  GTTAAAACTCAAATGAATTGACGGGGGCCCGCACAAGCGGTGGAGCATGTGGTTTAATTC  905

Query  910  GAAGCAACGCGAAGAACCTTACCAGGCCTTGACATCCAATGAACTTTCCNNAGATGGATT  969
            |||||||||||||||||||||||||||||||||||||||||||||||||  |||||||||
Sbjct  906  GAAGCAACGCGAAGAACCTTACCAGGCCTTGACATCCAATGAACTTTCCAGAGATGGATT  965

Query  970  GGTGCCTTCGGGNGNATTG  988
            |||||||||||| | ||||
Sbjct  966  GGTGCCTTCGGGAGCATTG  984
```

Download

FASTA (complete sequence)

FASTA (aligned sequences)

GenBank (complete sequence)

Continue
Cancel

GenBankGraphics

Next
Previous
Descriptions

Pseudomonas savastanoi strain XJGY3 16S ribosomal RNA gene, partial sequence

Sequence ID: KR708880.1Length: 1405Number of Matches: 1

#### See 2 more title(s)

Pseudomonas savastanoi strain CGGJ22 16S ribosomal RNA gene, partial sequence

Sequence ID: KR708883.1Length: 1405Number of Matches:

Pseudomonas savastanoi strain XJPJ20 16S ribosomal RNA gene, partial sequence

Sequence ID: KR708948.1Length: 1405Number of Matches:

Related Information

Range 1: 7 to 984GenBankGraphics

Next Match
Previous Match
First Match

Alignment statistics for match #1

| Score | Expect | Identities | Gaps | Strand | Frame |
| --- | --- | --- | --- | --- | --- |
| 1786 bits(967) | 0.0() | 974/979(99%) | 1/979(0%) | Plus/Plus |  |

Features:

```
Query  10   AGTCGAGCGGCAGCACGGGTACTTGTACCTGGTGGCGAGCGGCGGACGGGTGAGTAATGC  69
            ||||||||||||||||||||||||||||||||||||||||||||||||||||||||||||
Sbjct  7    AGTCGAGCGGCAGCACGGGTACTTGTACCTGGTGGCGAGCGGCGGACGGGTGAGTAATGC  66

Query  70   CTAGGAATCTGCCTGGTAGTGGGGGATAACGCTCGGAAACGGACGCTAATACCGCATACG  129
            ||||||||||||||||||||||||||||||||||||||||||||||||||||||||||||
Sbjct  67   CTAGGAATCTGCCTGGTAGTGGGGGATAACGCTCGGAAACGGACGCTAATACCGCATACG  126

Query  130  TCCTACGGGAGAAAGCAGGGGACCTTCGGGCCTTGCGCTATCAGATGAGCCTAGGTCGGA  189
            ||||||||||||||||||||||||||||||||||||||||||||||||||||||||||||
Sbjct  127  TCCTACGGGAGAAAGCAGGGGACCTTCGGGCCTTGCGCTATCAGATGAGCCTAGGTCGGA  186

Query  190  TTAGCTAGTTGGTGAGGTAATGGCTCACCAAGGCGACGATCCGTAACTGGTCTGAGAGGA  249
            ||||||||||||||||||||||||||||||||||||||||||||||||||||||||||||
Sbjct  187  TTAGCTAGTTGGTGAGGTAATGGCTCACCAAGGCGACGATCCGTAACTGGTCTGAGAGGA  246

Query  250  TGATCAGTCACACTGGAACTGAGACACGGTCCAGACTCCTACGGGAGGCAGCAGTGGGGA  309
            ||||||||||||||||||||||||||||||||||||||||||||||||||||||||||||
Sbjct  247  TGATCAGTCACACTGGAACTGAGACACGGTCCAGACTCCTACGGGAGGCAGCAGTGGGGA  306

Query  310  ATATTGGACAATGGGCGAAAGCCTGATCCAGCCATGCCGCGTGTGTGAAGAAGGTCTTCG  369
            ||||||||||||||||||||||||||||||||||||||||||||||||||||||||||||
Sbjct  307  ATATTGGACAATGGGCGAAAGCCTGATCCAGCCATGCCGCGTGTGTGAAGAAGGTCTTCG  366

Query  370  GATTGTAAAGCACTTTAAGTTGGGAGGAAGGGCAGTTACCTAATACGTGATTGTTTTGAC  429
            ||||||||||||||||||||||||||||||||||||||||||||||||||||||||||||
Sbjct  367  GATTGTAAAGCACTTTAAGTTGGGAGGAAGGGCAGTTACCTAATACGTGATTGTTTTGAC  426

Query  430  GTTACCGACAGAATAAGCACCGGCTAACTCTGTGCCAGCAGCCGCGGTAATACAGAGGGT  489
            ||||||||||||||||||||||||||||||||||||||||||||||||||||||||||||
Sbjct  427  GTTACCGACAGAATAAGCACCGGCTAACTCTGTGCCAGCAGCCGCGGTAATACAGAGGGT  486

Query  490  GCAAGCGTTAATCGGAATTACTGGGCGTAAAGCGCGCGTAGGTGGTTTGTTAAGTTGAAT  549
            ||||||||||||||||||||||||||||||||||||||||||||||||||||||||||||
Sbjct  487  GCAAGCGTTAATCGGAATTACTGGGCGTAAAGCGCGCGTAGGTGGTTTGTTAAGTTGAAT  546

Query  550  GTGAAATCCCCGGGCTCAACCTGGGAACTGCATCCAAAACTGGCAAGCTAGAGTATGGTA  609
            ||||||||||||||||||||||||||||||||||||||||||||||||||||||||||||
Sbjct  547  GTGAAATCCCCGGGCTCAACCTGGGAACTGCATCCAAAACTGGCAAGCTAGAGTATGGTA  606

Query  610  GAGGGTGGTGGAATTTCCTGTGTAGCGGTGAAATGCGTAGATATAGGAAGGAACACCAGT  669
            ||||||||||||||||||||||||||||||||||||||||||||||||||||||||||||
Sbjct  607  GAGGGTGGTGGAATTTCCTGTGTAGCGGTGAAATGCGTAGATATAGGAAGGAACACCAGT  666

Query  670  GGCGAAGGCGACCACCTGGACTGATACTGACACTGAGGTGCGAAAGCGTGGGGAGCAAAC  729
            ||||||||||||||||||||||||||||||||||||||||||||||||||||||||||||
Sbjct  667  GGCGAAGGCGACCACCTGGACTGATACTGACACTGAGGTGCGAAAGCGTGGGGAGCAAAC  726

Query  730  AGGATTAGATACCCTGGTAGTCCACGCCGTAAACGATGTCAACTAGCCGTTGGGAGCCTT  789
            ||||||||||||||||||||||||||||||||||||||||||||||||||||||||||||
Sbjct  727  AGGATTAGATACCCTGGTAGTCCACGCCGTAAACGATGTCAACTAGCCGTTGGGAGCCTT  786

Query  790  GAGCTCTTAGTGGCGCAGCTAACGCATTAAGTTGACCGCCTGGGGGAGTACGGCCGCAAG  849
            ||||||||||||||||||||||||||||||||||||||||| ||||||||||||||||||
Sbjct  787  GAGCTCTTAGTGGCGCAGCTAACGCATTAAGTTGACCGCCT-GGGGAGTACGGCCGCAAG  845

Query  850  GTTAAAACTCAAATGAATTGACGGGGGCCCGCACAAGCGGTGGAGCATGTGGTTTAATTC  909
            ||||||||||||||||||||||||||||||||||||||||||||||||||||||||||||
Sbjct  846  GTTAAAACTCAAATGAATTGACGGGGGCCCGCACAAGCGGTGGAGCATGTGGTTTAATTC  905

Query  910  GAAGCAACGCGAAGAACCTTACCAGGCCTTGACATCCAATGAACTTTCCNNAGATGGATT  969
            |||||||||||||||||||||||||||||||||||||||||||||||||  |||||||||
Sbjct  906  GAAGCAACGCGAAGAACCTTACCAGGCCTTGACATCCAATGAACTTTCCAGAGATGGATT  965

Query  970  GGTGCCTTCGGGNGNATTG  988
            |||||||||||| | ||||
Sbjct  966  GGTGCCTTCGGGAGCATTG  984
```


BLAST is a registered trademark of the National Library of Medicine

Support center
Mailing list


YouTube

- National Library Of Medicine
- National Institutes Of Health
- U.S. Department of Health & Human Services
- USA.gov

### NCBI


National Center for Biotechnology Information,
 U.S. National Library of Medicine

8600 Rockville Pike,
Bethesda
 MD,
20894
USA

Policies and Guidelines
|
Contact


PreferencesTurn off

External link. Please review our privacy policy.
