# Supplementary material for: Isothermal microcalorimetry for thermal viable count of microorganisms in pure cultures and stabilized formulations
Source: BMC Microbiol. 2019 Mar 21;19:65. doi: 10.1186/s12866-019-1432-8 (PMC6429831; doi:10.1186/s12866-019-1432-8)
Supplement: Supplementary file 3 — 16S rRNA gene sequences’ BLASTN hits in zipped HTML format. (ZIP 15810 kb) [file 12866_2019_1432_MOESM3_ESM.zip › Best blastn hits/NCBI Blast_L pinkish SS R -- 12..1038 of sequence.html]

NCBI Blast:L pinkish SS R -- 12..1038 of sequence


- NCBI Home
- Sign in to NCBI
- Skip to Main Content
- Skip to Navigation
- About NCBI Accesskeys

U.S. National Library of Medicine

NCBI
National Center for Biotechnology Information

- My NCBI
- Sign in to NCBI
- Register
- Sign Out

BLAST ^®^ » blastn suite » RID-A4XVZRTH015


- Home
- Recent Results
- Saved Strategies
- Help

BLAST Results


Edit and Resubmit
Save Search Strategies
[Sign in above to save your search strategy]

Formatting options 


Download


How to read this page
Blast report description
Questions/comments


|  |  |
| --- | --- |
| Formatting options | |
| Show | Alignment as  HTML Plain text   Old View Reset form to defaults [?]  These options control formatting of alignments in results pages. The default is HTML, but other formats (including plain text) are available. PSSM and PssmWithParameters are representations of Position Specific Scoring Matrices and are only available for PSI-BLAST. The Advanced view option allows the database descriptions to be sorted by various indices in a table. |
| Alignment View | Pairwise Pairwise with dots for identities Query-anchored with dots for identities Query-anchored with letters for identities Flat query-anchored with dots for identities Flat query-anchored with letters for identities [?]  Choose how to view alignments. The default "pairwise" view shows how each subject sequence aligns individually to the query sequence. The "query-anchored" view shows how all subject sequences align to the query sequence. For each view type, you can choose to show "identities" (matching residues) as letters or dots. more... |
| Display | Graphical Overview   Linkout   Sequence Retrieval  NCBI-gi   CDS feature [?]  - Graphical Overview: Graphical Overview: Show graph of similar sequence regions aligned to query.   more... - NCBI-gi: Show NCBI gi identifiers. - CDS feature: Show annotated coding region and translation.   more... |
| Masking | Character:   X for protein, n for nucleotide Lower Case  Color:  Black Grey Red [?]  - Masking Character: Display masked (filtered) sequence regions as lower-case or as specific letters (N for nucleotide, P for protein). - Masking Color: Display masked sequence regions in the given color. |
| Limit results | Descriptions:  10 50 100 Graphical overview:  0 10 50 100  Alignments:  0 10 50 100 Line length:  60 90 120 150 [?]  - Descriptions: Show short descriptions for up to the given number of sequences. - Alignments: Show alignments for up to the given number of sequences, in order of statistical significance. - Line lenghth: Number of letters to show on one line in an alignment. |
|  | Organism Type common name, binomial, taxid, or group name. Only 20 top taxa will be shown.     Exclude   [?]  Show only sequences from the given organism. |
|  | Entrez query:  [?]  Show only those sequences that match the given Entrez query. more... |
|  | Expect Min:  Expect Max:  [?]  Show only sequences with expect values in the given range. more... |
|  | Percent Identity Min:  Percent Identity Max:  [?]  Show only sequences with percent identity values in the given range. |
| Format for | PSI-BLAST with inclusion threshold:  [?]  - Format for PSI-BLAST: The Position-Specific Iterated BLAST (PSI-BLAST) program performs iterative searches with a protein query,   in which sequences found in one round of search are used to build a custom score model for the next round.   more... - Inclusion Threshold: This sets the statistical significance threshold for including a sequence in the model used   by PSI-BLAST to create the PSSM on the next iteration. |

|  |  |  |  |  |  |
| --- | --- | --- | --- | --- | --- |
| Download | | | | | |
| Alignment  Text XML ASN.1 JSON Seq-align Hit Table(text) Hit Table(csv) Multiple-file XML2 Single-file XML2 Multiple-file JSON Single-file JSON SAM | Search Strategies  ASN.1 | PSSM to restart search  PSSM | [?] |  |  |

The Download link provides BLAST output that may be used as input to another program.
This includes parseable formats such as the tabular report or XML as well as the Search Strategy files read by the BLAST+ applications.
More details on the parseable (XML, tabular, and ASN.1) reports can be found at
https://www.ncbi.nlm.nih.gov/books/NBK153387/  
  

The following formats are offered under the Alignment section:  
1). "Text". Non-HTML standard BLAST report.  
2). "XML". XML report based upon the DTD at https://www.ncbi.nlm.nih.gov/data\_specs/dtd/NCBI\_BlastOutput.dtd  
3). "ASN.1". Alignment written out in Abstract Syntax Notation 1.  
4). "JSON Seq-align". Alignment written out in JSON.  
4). "Hit Table(text)". The tabular report as text.  
5). "Hit Table(csv)". The tabular report ready for import into spread-sheet programs like Excel.  
6). "XML2". New XML format described at ftp://ftp.ncbi.nlm.nih.gov/blast/documents/NEWXML/xml2.pdf.  
7). "JSON". New JSON format described at ftp://ftp.ncbi.nlm.nih.gov/blast/documents/NEWXML/xml2.pdf.  
8). "SAM". Sequence Alignment Map format.

XML2 and JSON can be downloaded either as one file per query (multiple-file) or one file for all queries (single-file). These formats are listed as Multiple-file XML2 (and JSON) or Single-file XML (and JSON).

The following report is offered under the Search Strategy section:  
1). "ASN.1" Search Strategy. A record of the parameters, query, and database used in the search. This file can be used to start a stand-alone BLAST search, see
https://www.ncbi.nlm.nih.gov/books/NBK1763/#CmdLineAppsManual.I455\_BLAST\_search\_stra


# Job title: L pinkish SS R -- 12..1038 of sequence

Results for:

lcl|Query\_238577 L pinkish SS R -- 12..1038 of sequence(1027bp)
[?]

Your BLAST job specified more than one input sequence.
This box lets you choose which input sequence to show BLAST results for.

RID
:   A4XVZRTH015 (Expires on 03-10 19:01 pm)

Query ID
:   lcl|Query\_238577
:   lcl|Query\_238577

Description
:   L pinkish SS R -- 12..1038 of sequence

Molecule type
:   nucleic acid

Query Length
:   1027

Database Name
:   nr

Description
:   Nucleotide collection (nt) See details

Program
:   BLASTN 2.8.0+ Citation

  

Reference 

Zheng Zhang, Scott Schwartz, Lukas Wagner, and Webb Miller (2000), "A greedy algorithm for aligning DNA sequences", J Comput Biol 2000; 7(1-2):203-14.

Reference - database indexing

Aleksandr Morgulis, George Coulouris, Yan Raytselis, Thomas L. Madden, Richa Agarwala, Alejandro A. Schäffer (2008), "Database Indexing for Production MegaBLAST Searches", Bioinformatics 24:1757-1764.

Other reports:
Search Summary

[Taxonomy reports]
[Distance tree of results]
[MSA viewer]

Search Parameters

| Search parameter name | Search parameter value |
| --- | --- |
| Program | blastn |
| Word size | 28 |
| Expect value | 10 |
| Hitlist size | 100 |
| Match/Mismatch scores | 1,-2 |
| Gapcosts | 0,2.5 |
| Low Complexity Filter | Yes |
| Filter string | L;m; |
| Genetic Code | 1 |

Database

| Database parameter name | Database parameter value |
| --- | --- |
| Posted date | Mar 7, 2018 1:58 PM |
| Number of letters | 174,044,644,244 |
| Number of sequences | 46,882,714 |
| Entrez query | Includes:  Excludes:  None |

Karlin-Altschul statistics

| Params | Ungapped | Gapped |
| --- | --- | --- |
| Lambda | 1.33271 | 1.28 |
| K | 0.620991 | 0.46 |
| H | 1.12409 | 0.85 |

Results Statistics

| Results Statistics parameter name | Results Statistics parameter value |
| --- | --- |
| Length adjustment | 35 |
| Effective length of query | 992 |
| Effective length of database | 172403749254 |
| Effective search space | 171024519259968 |
| Effective search space used | 171024519259968 |


## Graphic Summary

### Distribution of the top 139 Blast Hits on 95 subject sequences [?]

The graphic is an overview of the database sequences aligned to the query sequence. These are represented horizontal bars colored coded by score and showing the extent
of the alignment on the query sequence. Separate aligned regions on the same database sequence are connected by a thin grey line.
Mousing over an alignment shows the database sequence title. Clicking an alignment displays a box with more details about the alignment and
link to the sequence alignment itself in the Alignments section of the report.

Mouse over to see the title, click to show alignments

Color key for alignment scores

<40

40-50

50-80

80-200

>=200

Query

1

200

400

600

800

1000

Pseudomonas syringae pv. malvacearum strain 480-2 16S r..

Score:1827 Evalue:0

Accession:MG230266.1

Alignment

Uncultured bacterium clone 21-23 16S ribosomal RNA gene..

Score:1827 Evalue:0

Accession:KT029459.1

Alignment

Pseudomonas savastanoi pv. nerii strain IPPBC-O38 16S r..

Score:1827 Evalue:0

Accession:HQ840767.1

Alignment

Pseudomonas sp. Ina1 partial 16S rRNA gene, strain Ina1

Score:1827 Evalue:0

Accession:AM909655.1

Alignment

Pseudomonas sp. HP7P 16S ribosomal RNA gene, partial se..

Score:1825 Evalue:0

Accession:KM187525.1

Alignment

Pseudomonas cerasi isolate PL963 genome assembly, chrom..

Score:1821 Evalue:0

Accession:LT963395.1

Alignment

Pseudomonas sp. 58 isolate Sour cherry (Prunus cerasus)..

Score:1821 Evalue:0

Accession:LT222319.1

Alignment

Pseudomonas syringae strain PDD-70b-1 16S ribosomal RNA..

Score:1821 Evalue:0

Accession:KR922304.1

Alignment

Uncultured bacterium clone 21-19 16S ribosomal RNA gene..

Score:1821 Evalue:0

Accession:KT029454.1

Alignment

Pseudomonas amygdali pv. tabaci strain YWTG-1 16S ribos..

Score:1821 Evalue:0

Accession:KR476391.1

Alignment

Pseudomonas syringae pv. pisi strain ATCC 11043 16S rib..

Score:1821 Evalue:0

Accession:KP211411.1

Alignment

Pseudomonas syringae pv. pisi strain pea seed 1314 16S ..

Score:1821 Evalue:0

Accession:KP211410.1

Alignment

Pseudomonas syringae pv. syringae strain IZB221 16S rib..

Score:1821 Evalue:0

Accession:KP099978.1

Alignment

Pseudomonas syringae pv. syringae strain IZB212 16S rib..

Score:1821 Evalue:0

Accession:KP099974.1

Alignment

Pseudomonas syringae pv. syringae strain IZB208 16S rib..

Score:1821 Evalue:0

Accession:KP099972.1

Alignment

Pseudomonas syringae pv. syringae strain IZB201 16S rib..

Score:1821 Evalue:0

Accession:KP099970.1

Alignment

Pseudomonas syringae pv. tabaci strain SWTG-1 16S ribos..

Score:1821 Evalue:0

Accession:KJ756714.1

Alignment

Pseudomonas syringae pv. phaseolicola strain LDS8 16S r..

Score:1821 Evalue:0

Accession:KF888740.1

Alignment

Pseudomonas syringae pv. syringae strain XJLX-2-2 16S r..

Score:1821 Evalue:0

Accession:KC816630.1

Alignment

Pseudomonas syringae pv. syringae strain XJLX-3-8 16S r..

Score:1821 Evalue:0

Accession:KC816629.1

Alignment

Pseudomonas syringae pv. syringae strain XJLX-3-7 16S r..

Score:1821 Evalue:0

Accession:KC816628.1

Alignment

Pseudomonas syringae pv. syringae strain XJLX-4-2 16S r..

Score:1821 Evalue:0

Accession:KC816626.1

Alignment

Pseudomonas sp. GCh5Fc 16S ribosomal RNA gene, partial ..

Score:1821 Evalue:0

Accession:KC311258.1

Alignment

Pseudomonas sp. PCa2a 16S ribosomal RNA gene, partial s..

Score:1821 Evalue:0

Accession:KC311255.1

Alignment

Pseudomonas sp. BXIN4 16S ribosomal RNA gene, partial s..

Score:1821 Evalue:0

Accession:KC311254.1

Alignment

Pseudomonas syringae strain XJKEL-1 16S ribosomal RNA g..

Score:1821 Evalue:0

Accession:JX474752.1

Alignment

Pseudomonas syringae pv. syringae strain KFB 93 16S rib..

Score:1821 Evalue:0

Accession:JQ071937.1

Alignment

Pseudomonas syringae pv. syringae strain KFB 82 16S rib..

Score:1821 Evalue:0

Accession:JQ071935.1

Alignment

Pseudomonas syringae pv. syringae strain KFB 80 16S rib..

Score:1821 Evalue:0

Accession:JQ071934.1

Alignment

Pseudomonas syringae gene for 16S rRNA, partial sequenc..

Score:1821 Evalue:0

Accession:AB680547.1

Alignment

Pseudomonas syringae strain ICMP 3023 16S ribosomal RNA..

Score:1821 Evalue:0

Accession:NR\_117820.1

Alignment

Pseudomonas congelans strain P 538/23 16S ribosomal RNA..

Score:1821 Evalue:0

Accession:NR\_028985.1

Alignment

Pseudomonas syringae strain 17 16S ribosomal RNA gene, ..

Score:1821 Evalue:0

Accession:DQ318868.1

Alignment

Pseudomonas syringae pv. pisi gene for 16S ribosomal RN..

Score:1821 Evalue:0

Accession:AB109218.1

Alignment

Pseudomonas sp. LPPA C117 partial 16S rRNA gene, strain..

Score:1821 Evalue:0

Accession:AJ970168.1

Alignment

Pseudomonas congelans partial 16S rRNA gene, type strai..

Score:1821 Evalue:0

Accession:AJ492828.1

Alignment

Pseudomonas syringae pv. tabaci gene for 16S ribosomal ..

Score:1818 Evalue:0

Accession:LC010244.1

Alignment

Pseudomonas syringae pv. tabaci gene for 16S ribosomal ..

Score:1818 Evalue:0

Accession:LC010243.1

Alignment

Pseudomonas syringae pv. syringae strain XJLX-8-3 16S r..

Score:1818 Evalue:0

Accession:KC816627.1

Alignment

Pseudomonas syringae pv. syringae strain N1 16S ribosom..

Score:1818 Evalue:0

Accession:JX826634.1

Alignment

Pseudomonas syringae strain NCPPB 281 16S ribosomal RNA..

Score:1818 Evalue:0

Accession:NR\_043716.1

Alignment

Pseudomonas syringae pv. cerasicola isolate CFBP6109 ge..

Score:1816 Evalue:0

Accession:LT963391.1

Alignment

Pseudomonas syringae pv. syringae strain B728a 16S ribo..

Score:1816 Evalue:0

Accession:MG722805.1

Alignment

Pseudomonas syringae pv. malvacearum strain 480-4 16S r..

Score:1816 Evalue:0

Accession:MG230268.1

Alignment

Pseudomonas syringae pv. malvacearum strain 480-1 16S r..

Score:1816 Evalue:0

Accession:MG230265.1

Alignment

Pseudomonas amygdali pv. lachrymans strain NM002, compl..

Score:1816 Evalue:0

Accession:CP020351.1

Alignment

Pseudomonas savastanoi strain XJPY13 16S ribosomal RNA ..

Score:1816 Evalue:0

Accession:KR708943.1

Alignment

Pseudomonas syringae strain 7GJ-14 16S ribosomal RNA ge..

Score:1816 Evalue:0

Accession:KR708853.1

Alignment

Pseudomonas syringae pv. syringae strain AK1 16S riboso..

Score:1816 Evalue:0

Accession:KU321601.1

Alignment

Pseudomonas syringae strain 31R1 genome assembly, chrom..

Score:1816 Evalue:0

Accession:LT629769.1

Alignment

Pseudomonas sp. FBF16 partial 16S rRNA gene, isolate FB..

Score:1816 Evalue:0

Accession:HG805694.1

Alignment

Pseudomonas syringae pv. lapsa strain ATCC 10859, compl..

Score:1816 Evalue:0

Accession:CP013183.1

Alignment

Pseudomonas sp. PDD-59b-7 16S ribosomal RNA gene, compl..

Score:1816 Evalue:0

Accession:KR922145.1

Alignment

Pseudomonas amygdali pv. tabaci strain BRST-1 16S ribos..

Score:1816 Evalue:0

Accession:KR476390.1

Alignment

Pseudomonas syringae pv. syringae B301D, complete genom..

Score:1816 Evalue:0

Accession:CP005969.1

Alignment

Pseudomonas syringae pv. syringae strain IZB218 16S rib..

Score:1816 Evalue:0

Accession:KP099976.1

Alignment

## Descriptions

, Reading indexes 1-5, displaying indexes 1-5


Load next setPrevious Match

Sequences producing significant alignments:

Show all columns  of the table presenting sequences producing significant alignments 

Select:AllNone
Selected:0

Alignments
Download

FASTA (complete sequence)

FASTA (aligned sequences)

GenBank (complete sequence)

Hit Table (text)

Hit Table (CSV)

Text

XML

ASN.1

Continue
Cancel

GenBank 
Graphics
Distance tree of results
Multiple alignment
Show/hide columns of the table presenting sequences producing significant alignments 

Available columns

Description  
Max Score  
Total Score  
Coverage  
E-value  
IdentN  
Accession  
Restore Defaults
Ok
Cancel

Sequences producing significant alignments:

| Select for downloading or viewing reports | Description | Max score | Total score | Query cover | E value | Ident | Accession |
| --- | --- | --- | --- | --- | --- | --- | --- |
| 1Select seq MG230266.1 | Pseudomonas syringae pv. malvacearum strain 480-2 16S ribosomal RNA gene, partial sequence | 1827 | 1827 | 98% | 0.0 | 99% | MG230266.1 |
| 2Select seq KT029459.1 | Uncultured bacterium clone 21-23 16S ribosomal RNA gene, partial sequence | 1827 | 1827 | 98% | 0.0 | 99% | KT029459.1 |
| 3Select seq HQ840767.1 | Pseudomonas savastanoi pv. nerii strain IPPBC-O38 16S ribosomal RNA gene, partial sequence | 1827 | 1827 | 98% | 0.0 | 99% | HQ840767.1 |
| 4Select seq AM909655.1 | Pseudomonas sp. Ina1 partial 16S rRNA gene, strain Ina1 | 1827 | 1827 | 98% | 0.0 | 99% | AM909655.1 |
| 5Select seq KM187525.1 | Pseudomonas sp. HP7P 16S ribosomal RNA gene, partial sequence | 1825 | 1825 | 98% | 0.0 | 99% | KM187525.1 |
| 6Select seq LT963395.1 | Pseudomonas cerasi isolate PL963 genome assembly, chromosome: 1 | 1821 | 9087 | 98% | 0.0 | 99% | LT963395.1 |
| 7Select seq LT222319.1 | Pseudomonas sp. 58 isolate Sour cherry (Prunus cerasus) symptomatic leaf genome assembly, chromosome: 1 | 1821 | 9087 | 98% | 0.0 | 99% | LT222319.1 |
| 8Select seq KR922304.1 | Pseudomonas syringae strain PDD-70b-1 16S ribosomal RNA gene, complete sequence | 1821 | 1821 | 98% | 0.0 | 99% | KR922304.1 |
| 9Select seq KT029454.1 | Uncultured bacterium clone 21-19 16S ribosomal RNA gene, partial sequence | 1821 | 1821 | 98% | 0.0 | 99% | KT029454.1 |
| 10Select seq KR476391.1 | Pseudomonas amygdali pv. tabaci strain YWTG-1 16S ribosomal RNA gene, partial sequence | 1821 | 1821 | 98% | 0.0 | 99% | KR476391.1 |
| 11Select seq KP211411.1 | Pseudomonas syringae pv. pisi strain ATCC 11043 16S ribosomal RNA gene, 16S-23S ribosomal RNA intergenic spacer, and 23S ribosomal RNA gene, complete sequence | 1821 | 1821 | 98% | 0.0 | 99% | KP211411.1 |
| 12Select seq KP211410.1 | Pseudomonas syringae pv. pisi strain pea seed 1314 16S ribosomal RNA gene, partial sequence; 16S-23S ribosomal RNA intergenic spacer, complete sequence; and 23S ribosomal RNA gene, partial sequence | 1821 | 1821 | 98% | 0.0 | 99% | KP211410.1 |
| 13Select seq KP099978.1 | Pseudomonas syringae pv. syringae strain IZB221 16S ribosomal RNA gene, partial sequence | 1821 | 1821 | 98% | 0.0 | 99% | KP099978.1 |
| 14Select seq KP099974.1 | Pseudomonas syringae pv. syringae strain IZB212 16S ribosomal RNA gene, partial sequence | 1821 | 1821 | 98% | 0.0 | 99% | KP099974.1 |
| 15Select seq KP099972.1 | Pseudomonas syringae pv. syringae strain IZB208 16S ribosomal RNA gene, partial sequence | 1821 | 1821 | 98% | 0.0 | 99% | KP099972.1 |
| 16Select seq KP099970.1 | Pseudomonas syringae pv. syringae strain IZB201 16S ribosomal RNA gene, partial sequence | 1821 | 1821 | 98% | 0.0 | 99% | KP099970.1 |
| 17Select seq KJ756714.1 | Pseudomonas syringae pv. tabaci strain SWTG-1 16S ribosomal RNA gene, partial sequence | 1821 | 1821 | 98% | 0.0 | 99% | KJ756714.1 |
| 18Select seq KF888740.1 | Pseudomonas syringae pv. phaseolicola strain LDS8 16S ribosomal RNA gene, partial sequence | 1821 | 1821 | 98% | 0.0 | 99% | KF888740.1 |
| 19Select seq KC816630.1 | Pseudomonas syringae pv. syringae strain XJLX-2-2 16S ribosomal RNA gene, partial sequence | 1821 | 1821 | 98% | 0.0 | 99% | KC816630.1 |
| 20Select seq KC816629.1 | Pseudomonas syringae pv. syringae strain XJLX-3-8 16S ribosomal RNA gene, partial sequence | 1821 | 1821 | 98% | 0.0 | 99% | KC816629.1 |
| 21Select seq KC816628.1 | Pseudomonas syringae pv. syringae strain XJLX-3-7 16S ribosomal RNA gene, partial sequence | 1821 | 1821 | 98% | 0.0 | 99% | KC816628.1 |
| 22Select seq KC816626.1 | Pseudomonas syringae pv. syringae strain XJLX-4-2 16S ribosomal RNA gene, partial sequence | 1821 | 1821 | 98% | 0.0 | 99% | KC816626.1 |
| 23Select seq KC311258.1 | Pseudomonas sp. GCh5Fc 16S ribosomal RNA gene, partial sequence | 1821 | 1821 | 98% | 0.0 | 99% | KC311258.1 |
| 24Select seq KC311255.1 | Pseudomonas sp. PCa2a 16S ribosomal RNA gene, partial sequence | 1821 | 1821 | 98% | 0.0 | 99% | KC311255.1 |
| 25Select seq KC311254.1 | Pseudomonas sp. BXIN4 16S ribosomal RNA gene, partial sequence | 1821 | 1821 | 98% | 0.0 | 99% | KC311254.1 |
| 26Select seq JX474752.1 | Pseudomonas syringae strain XJKEL-1 16S ribosomal RNA gene, partial sequence | 1821 | 1821 | 98% | 0.0 | 99% | JX474752.1 |
| 27Select seq JQ071937.1 | Pseudomonas syringae pv. syringae strain KFB 93 16S ribosomal RNA gene, partial sequence | 1821 | 1821 | 98% | 0.0 | 99% | JQ071937.1 |
| 28Select seq JQ071935.1 | Pseudomonas syringae pv. syringae strain KFB 82 16S ribosomal RNA gene, partial sequence | 1821 | 1821 | 98% | 0.0 | 99% | JQ071935.1 |
| 29Select seq JQ071934.1 | Pseudomonas syringae pv. syringae strain KFB 80 16S ribosomal RNA gene, partial sequence | 1821 | 1821 | 98% | 0.0 | 99% | JQ071934.1 |
| 30Select seq AB680547.1 | Pseudomonas syringae gene for 16S rRNA, partial sequence, strain: NBRC 14075 | 1821 | 1821 | 98% | 0.0 | 99% | AB680547.1 |
| 31Select seq NR\_117820.1 | Pseudomonas syringae strain ICMP 3023 16S ribosomal RNA gene, partial sequence | 1821 | 1821 | 98% | 0.0 | 99% | NR\_117820.1 |
| 32Select seq NR\_028985.1 | Pseudomonas congelans strain P 538/23 16S ribosomal RNA gene, partial sequence | 1821 | 1821 | 98% | 0.0 | 99% | NR\_028985.1 |
| 33Select seq DQ318868.1 | Pseudomonas syringae strain 17 16S ribosomal RNA gene, partial sequence | 1821 | 1821 | 98% | 0.0 | 99% | DQ318868.1 |
| 34Select seq AB109218.1 | Pseudomonas syringae pv. pisi gene for 16S ribosomal RNA | 1821 | 1821 | 98% | 0.0 | 99% | AB109218.1 |
| 35Select seq AJ970168.1 | Pseudomonas sp. LPPA C117 partial 16S rRNA gene, strain LPPA C117 | 1821 | 1821 | 98% | 0.0 | 99% | AJ970168.1 |
| 36Select seq AJ492828.1 | Pseudomonas congelans partial 16S rRNA gene, type strain DSM 14939T | 1821 | 1821 | 98% | 0.0 | 99% | AJ492828.1 |
| 37Select seq LC010244.1 | Pseudomonas syringae pv. tabaci gene for 16S ribosomal RNA, partial sequence, strain: GWF-311 | 1818 | 1818 | 98% | 0.0 | 99% | LC010244.1 |
| 38Select seq LC010243.1 | Pseudomonas syringae pv. tabaci gene for 16S ribosomal RNA, partial sequence, strain: GWF-121 | 1818 | 1818 | 98% | 0.0 | 99% | LC010243.1 |
| 39Select seq KC816627.1 | Pseudomonas syringae pv. syringae strain XJLX-8-3 16S ribosomal RNA gene, partial sequence | 1818 | 1818 | 98% | 0.0 | 99% | KC816627.1 |
| 40Select seq JX826634.1 | Pseudomonas syringae pv. syringae strain N1 16S ribosomal RNA gene, partial sequence | 1818 | 1818 | 98% | 0.0 | 99% | JX826634.1 |
| 41Select seq NR\_043716.1 | Pseudomonas syringae strain NCPPB 281 16S ribosomal RNA gene, partial sequence | 1818 | 1818 | 98% | 0.0 | 99% | NR\_043716.1 |
| 42Select seq LT963391.1 | Pseudomonas syringae pv. cerasicola isolate CFBP6109 genome assembly, chromosome: 1 | 1816 | 9081 | 98% | 0.0 | 99% | LT963391.1 |
| 43Select seq MG722805.1 | Pseudomonas syringae pv. syringae strain B728a 16S ribosomal RNA gene, partial sequence | 1816 | 1816 | 98% | 0.0 | 99% | MG722805.1 |
| 44Select seq MG230268.1 | Pseudomonas syringae pv. malvacearum strain 480-4 16S ribosomal RNA gene, partial sequence | 1816 | 1816 | 98% | 0.0 | 99% | MG230268.1 |
| 45Select seq MG230265.1 | Pseudomonas syringae pv. malvacearum strain 480-1 16S ribosomal RNA gene, partial sequence | 1816 | 1816 | 98% | 0.0 | 99% | MG230265.1 |
| 46Select seq CP020351.1 | Pseudomonas amygdali pv. lachrymans strain NM002, complete genome | 1816 | 9059 | 98% | 0.0 | 99% | CP020351.1 |
| 47Select seq KR708943.1 | Pseudomonas savastanoi strain XJPY13 16S ribosomal RNA gene, partial sequence | 1816 | 1816 | 98% | 0.0 | 99% | KR708943.1 |
| 48Select seq KR708853.1 | Pseudomonas syringae strain 7GJ-14 16S ribosomal RNA gene, partial sequence | 1816 | 1816 | 98% | 0.0 | 99% | KR708853.1 |
| 49Select seq KU321601.1 | Pseudomonas syringae pv. syringae strain AK1 16S ribosomal RNA gene, partial sequence | 1816 | 1816 | 98% | 0.0 | 99% | KU321601.1 |
| 50Select seq LT629769.1 | Pseudomonas syringae strain 31R1 genome assembly, chromosome: I | 1816 | 9032 | 98% | 0.0 | 99% | LT629769.1 |
| 51Select seq HG805694.1 | Pseudomonas sp. FBF16 partial 16S rRNA gene, isolate FBF16 | 1816 | 1816 | 98% | 0.0 | 99% | HG805694.1 |
| 52Select seq CP013183.1 | Pseudomonas syringae pv. lapsa strain ATCC 10859, complete genome | 1816 | 9026 | 98% | 0.0 | 99% | CP013183.1 |
| 53Select seq KR922145.1 | Pseudomonas sp. PDD-59b-7 16S ribosomal RNA gene, complete sequence | 1816 | 1816 | 98% | 0.0 | 99% | KR922145.1 |
| 54Select seq KR476390.1 | Pseudomonas amygdali pv. tabaci strain BRST-1 16S ribosomal RNA gene, partial sequence | 1816 | 1816 | 98% | 0.0 | 99% | KR476390.1 |
| 55Select seq CP005969.1 | Pseudomonas syringae pv. syringae B301D, complete genome | 1816 | 9081 | 98% | 0.0 | 99% | CP005969.1 |
| 56Select seq KP099976.1 | Pseudomonas syringae pv. syringae strain IZB218 16S ribosomal RNA gene, partial sequence | 1816 | 1816 | 98% | 0.0 | 99% | KP099976.1 |
| 57Select seq KJ756715.1 | Pseudomonas syringae pv. tabaci strain YC-1 16S ribosomal RNA gene, partial sequence | 1816 | 1816 | 98% | 0.0 | 99% | KJ756715.1 |
| 58Select seq KJ569377.1 | Pseudomonas syringae pv. glycinea strain EM1 16S ribosomal RNA gene, partial sequence | 1816 | 1816 | 98% | 0.0 | 99% | KJ569377.1 |
| 59Select seq KF888738.1 | Pseudomonas syringae pv. tabaci strain LDST3 16S ribosomal RNA gene, partial sequence | 1816 | 1816 | 98% | 0.0 | 99% | KF888738.1 |
| 60Select seq KF888736.1 | Pseudomonas syringae pv. tabaci strain LDST1 16S ribosomal RNA gene, partial sequence | 1816 | 1816 | 98% | 0.0 | 99% | KF888736.1 |
| 61Select seq KC860048.1 | Pseudomonas syringae pv. lachrymans strain 361 16S ribosomal RNA gene, partial sequence | 1816 | 1816 | 98% | 0.0 | 99% | KC860048.1 |
| 62Select seq KC860047.1 | Pseudomonas syringae pv. lachrymans strain njl18 16S ribosomal RNA gene, partial sequence | 1816 | 1816 | 98% | 0.0 | 99% | KC860047.1 |
| 63Select seq KF019101.1 | Pseudomonas syringae isolate PS10.1 16S ribosomal RNA gene, partial sequence | 1816 | 1816 | 98% | 0.0 | 99% | KF019101.1 |
| 64Select seq KF003377.1 | Pseudomonas syringae strain ABAC10 16S ribosomal RNA gene, partial sequence | 1816 | 1816 | 98% | 0.0 | 99% | KF003377.1 |
| 65Select seq KF003372.1 | Pseudomonas syringae strain ABAC1 16S ribosomal RNA gene, partial sequence | 1816 | 1816 | 98% | 0.0 | 99% | KF003372.1 |
| 66Select seq KC139457.1 | Pseudomonas sp. CZ01 16S ribosomal RNA gene, partial sequence | 1816 | 1816 | 98% | 0.0 | 99% | KC139457.1 |
| 67Select seq JQ320090.1 | Pseudomonas congelans strain XjGEB-29 16S ribosomal RNA gene, partial sequence | 1816 | 1816 | 98% | 0.0 | 99% | JQ320090.1 |
| 68Select seq AB680544.1 | Pseudomonas syringae gene for 16S rRNA, partial sequence, strain: NBRC 14054 | 1816 | 1816 | 98% | 0.0 | 99% | AB680544.1 |
| 69Select seq AB680305.1 | Pseudomonas syringae gene for 16S rRNA, partial sequence, strain: NBRC 12655 | 1816 | 1816 | 98% | 0.0 | 99% | AB680305.1 |
| 70Select seq FJ607146.1 | Pseudomonas syringae pv. cerasicola strain ICMP 13927 16S ribosomal RNA gene, partial sequence | 1816 | 1816 | 98% | 0.0 | 99% | FJ607146.1 |
| 71Select seq FN421714.1 | Uncultured bacterium partial 16S rRNA gene, clone 7\_G10 | 1816 | 1816 | 98% | 0.0 | 99% | FN421714.1 |
| 72Select seq FN421699.1 | Uncultured bacterium partial 16S rRNA gene, clone 3\_F06 | 1816 | 1816 | 98% | 0.0 | 99% | FN421699.1 |
| 73Select seq FN421697.1 | Uncultured bacterium partial 16S rRNA gene, clone 3\_F04 | 1816 | 1816 | 98% | 0.0 | 99% | FN421697.1 |
| 74Select seq FN421615.1 | Uncultured bacterium partial 16S rRNA gene, clone 13\_C06 | 1816 | 1816 | 98% | 0.0 | 99% | FN421615.1 |
| 75Select seq FJ755788.1 | Pseudomonas syringae pv. tabaci strain BC2366 16S ribosomal RNA gene, partial sequence | 1816 | 1816 | 98% | 0.0 | 99% | FJ755788.1 |
| 76Select seq AM909659.1 | Pseudomonas sp. Act34 partial 16S rRNA gene, strain Act34 | 1816 | 1816 | 98% | 0.0 | 99% | AM909659.1 |
| 77Select seq AM399034.1 | Pseudomonas syringae pv. coryli 16S rRNA gene, strain ISPaVe 595 | 1816 | 1816 | 98% | 0.0 | 99% | AM399034.1 |
| 78Select seq DQ318865.1 | Pseudomonas syringae strain 8535 16S ribosomal RNA gene, partial sequence | 1816 | 1816 | 98% | 0.0 | 99% | DQ318865.1 |
| 79Select seq DQ318862.1 | Pseudomonas savastanoi strain 8531 16S ribosomal RNA gene, partial sequence | 1816 | 1816 | 98% | 0.0 | 99% | DQ318862.1 |
| 80Select seq CP000075.1 | Pseudomonas syringae pv. syringae B728a, complete genome | 1816 | 9043 | 98% | 0.0 | 99% | CP000075.1 |
| 81Select seq AB001443.1 | Pseudomonas syringae pv. glycinea gene for 16S rRNA | 1816 | 1816 | 98% | 0.0 | 99% | AB001443.1 |
| 82Select seq KR708880.1 | Pseudomonas savastanoi strain XJGY3 16S ribosomal RNA gene, partial sequence | 1814 | 1814 | 98% | 0.0 | 99% | KR708880.1 |
| 83Select seq HM209785.1 | Pseudomonas syringae strain GGA973 16S ribosomal RNA gene, partial sequence | 1812 | 1812 | 98% | 0.0 | 99% | HM209785.1 |
| 84Select seq FN421665.1 | Uncultured bacterium partial 16S rRNA gene, clone 14\_F07 | 1812 | 1812 | 98% | 0.0 | 99% | FN421665.1 |
| 85Select seq FN421655.1 | Uncultured bacterium partial 16S rRNA gene, clone 13\_H12 | 1812 | 1812 | 98% | 0.0 | 99% | FN421655.1 |
| 86Select seq FN421637.1 | Uncultured bacterium partial 16S rRNA gene, clone 13\_F04 | 1812 | 1812 | 98% | 0.0 | 99% | FN421637.1 |
| 87Select seq FN421630.1 | Uncultured bacterium partial 16S rRNA gene, clone 13\_E05 | 1812 | 1812 | 98% | 0.0 | 99% | FN421630.1 |
| 88Select seq FN421622.1 | Uncultured bacterium partial 16S rRNA gene, clone 13\_D07 | 1812 | 1812 | 98% | 0.0 | 99% | FN421622.1 |
| 89Select seq AJ308316.1 | Pseudomonas syringae partial 16S rRNA gene, type strain ICMP 3023T | 1812 | 1812 | 98% | 0.0 | 99% | AJ308316.1 |
| 90Select seq Z76669.1 | P.syringae 16S rRNA gene | 1812 | 1812 | 98% | 0.0 | 99% | Z76669.1 |
| 91Select seq CP026568.1 | Pseudomonas syringae pv. syringae strain Pss9097 chromosome, complete genome | 1810 | 9032 | 98% | 0.0 | 99% | CP026568.1 |
| 92Select seq LT962481.1 | Pseudomonas syringae pv. syringae isolate CFBP2118 genome assembly, chromosome: 1 | 1810 | 9048 | 98% | 0.0 | 99% | LT962481.1 |
| 93Select seq LT962480.1 | Pseudomonas syringae pv. syringae isolate CFBP4215 genome assembly, chromosome: 1 | 1810 | 9032 | 98% | 0.0 | 99% | LT962480.1 |
| 94Select seq MG230269.1 | Pseudomonas syringae pv. malvacearum strain 480-5 16S ribosomal RNA gene, partial sequence | 1810 | 1810 | 98% | 0.0 | 99% | MG230269.1 |
| 95Select seq KX782339.1 | Pseudomonas syringae pv. syringae strain pss1 16S ribosomal RNA gene, partial sequence | 1810 | 1810 | 98% | 0.0 | 99% | KX782339.1 |


## Alignments

Loading alignment... for sequences gi|1268088842,gi|901409292,gi|328879887,gi|160730404,gi|695102743 Reading indexes 1-5

Download

FASTA (complete sequence)

FASTA (aligned sequences)

GenBank (complete sequence)

Continue
Cancel

GenBankGraphics

Next
Previous
Descriptions

Pseudomonas syringae pv. malvacearum strain 480-2 16S ribosomal RNA gene, partial sequence

Sequence ID: MG230266.1Length: 1535Number of Matches: 1

Related Information

Range 1: 450 to 1456GenBankGraphics

Next Match
Previous Match
First Match

Alignment statistics for match #1

| Score | Expect | Identities | Gaps | Strand | Frame |
| --- | --- | --- | --- | --- | --- |
| 1827 bits(989) | 0.0() | 1002/1010(99%) | 3/1010(0%) | Plus/Minus |  |

Features:

```
Query  9     CCGTCCCCCCGAAGGTTAGACTAGCTACTTCTGGTNCAACCCACTCCCATGGTGTGACGG  68
             ||||||||||||||||||||||||||||||||||| ||||||||||||||||||||||||
Sbjct  1456  CCGTCCCCCCGAAGGTTAGACTAGCTACTTCTGGTGCAACCCACTCCCATGGTGTGACGG  1397

Query  69    GCGGTGTGTACAAGGCCCGGGAACGTATTCACCGCGACATTCTGATTCGCGATTACTAGC  128
             ||||||||||||||||||||||||||||||||||||||||||||||||||||||||||||
Sbjct  1396  GCGGTGTGTACAAGGCCCGGGAACGTATTCACCGCGACATTCTGATTCGCGATTACTAGC  1337

Query  129   GATTCCGACTTCACGCAGTCGAGTTGCAGACTGCGATCCGGACTACGATCGGTTTTGTGA  188
             ||||||||||||||||||||||||||||||||||||||||||||||||||||||||||||
Sbjct  1336  GATTCCGACTTCACGCAGTCGAGTTGCAGACTGCGATCCGGACTACGATCGGTTTTGTGA  1277

Query  189   GATTAGCTCCACCTCGCGGCTTGGCAACCCTCTGTACCGACCATTGTAGCACGTGTGTAG  248
             ||||||||||||||||||||||||||||||||||||||||||||||||||||||||||||
Sbjct  1276  GATTAGCTCCACCTCGCGGCTTGGCAACCCTCTGTACCGACCATTGTAGCACGTGTGTAG  1217

Query  249   CCCAGGCCGTAAGGGCCATGATGACTTGACGTCATCCCCACCTTCCTCCGGTTTGTCACC  308
             ||||||||||||||||||||||||||||||||||||||||||||||||||||||||||||
Sbjct  1216  CCCAGGCCGTAAGGGCCATGATGACTTGACGTCATCCCCACCTTCCTCCGGTTTGTCACC  1157

Query  309   GGCAGTCTCCTTAGAGTGCCCACCTTAACGTGCTGGTAACTAAGGACAAGGGTTGCGCTC  368
             ||||||||||||||||||||||||||||||||||||||||||||||||||||||||||||
Sbjct  1156  GGCAGTCTCCTTAGAGTGCCCACCTTAACGTGCTGGTAACTAAGGACAAGGGTTGCGCTC  1097

Query  369   GTTACGGGACTTAACCCAACATCTCACGACACGAGCTGACGACAGCCATGCAGCACCTGT  428
             ||||||||||||||||||||||||||||||||||||||||||||||||||||||||||||
Sbjct  1096  GTTACGGGACTTAACCCAACATCTCACGACACGAGCTGACGACAGCCATGCAGCACCTGT  1037

Query  429   CTCAATGCTCCCGAAGGCACCAATCCATCTCTGGAAAGTTCATTGGATGTCAAGGCCTGG  488
             ||||||||||||||||||||||||||||||||||||||||||||||||||||||||||||
Sbjct  1036  CTCAATGCTCCCGAAGGCACCAATCCATCTCTGGAAAGTTCATTGGATGTCAAGGCCTGG  977

Query  489   TAAGGTTCTTCGCGTTGCTTCGAATTAAACCACATGCTCCACCGCTTGTGCGGGCCCCCG  548
             ||||||||||||||||||||||||||||||||||||||||||||||||||||||||||||
Sbjct  976   TAAGGTTCTTCGCGTTGCTTCGAATTAAACCACATGCTCCACCGCTTGTGCGGGCCCCCG  917

Query  549   TCAATTCATTTGAGTTTTAACCTTGCGGCCGTACTCCCCAGGCGGTCAACTTAATGCGTT  608
             ||||||||||||||||||||||||||||||||||||||||||||||||||||||||||||
Sbjct  916   TCAATTCATTTGAGTTTTAACCTTGCGGCCGTACTCCCCAGGCGGTCAACTTAATGCGTT  857

Query  609   AGCTGCGCCACTAAGAGCTCAAGGCTCCCAACGGCTAGTTGACATCGTTTACGGCGTGGA  668
             ||||||||||||||||||||||||||||||||||||||||||||||||||||||||||||
Sbjct  856   AGCTGCGCCACTAAGAGCTCAAGGCTCCCAACGGCTAGTTGACATCGTTTACGGCGTGGA  797

Query  669   CTACCAGGGTATCTAATCCTGTTTGCTCCCCACGCTTTCGCACCTCAGTGTCAGTATCAG  728
             ||||||||||||||||||||||||||||||||||||||||||||||||||||||||||||
Sbjct  796   CTACCAGGGTATCTAATCCTGTTTGCTCCCCACGCTTTCGCACCTCAGTGTCAGTATCAG  737

Query  729   TCCAGGTGGTCGCCTTCGCCACTGGTGTTCCTTCCTATATCTACGCATTTCACCGCTACA  788
             ||||||||||||||||||||||||||||||||||||||||||||||||||||||||||||
Sbjct  736   TCCAGGTGGTCGCCTTCGCCACTGGTGTTCCTTCCTATATCTACGCATTTCACCGCTACA  677

Query  789   CAGGAAATTCCACCACCCTCTACCATACTCTAGCTTGCCAGTTTTGGATGCAGTTCCCAG  848
             ||||||||||||||||||||||||||||||||||||||||||||||||||||||||||||
Sbjct  676   CAGGAAATTCCACCACCCTCTACCATACTCTAGCTTGCCAGTTTTGGATGCAGTTCCCAG  617

Query  849   GTTGAGCCCGGGGGATTTCACATTCAACTTAACAAACCACCTACGCGNNNCTTTACGCCC  908
             ||||||||||||| |||||||||||||||||||||||||||||||||   ||||||||||
Sbjct  616   GTTGAGCCCGGGG-ATTTCACATTCAACTTAACAAACCACCTACGCGCG-CTTTACGCCC  559

Query  909   AGTAATTCCGATTAACGCTTGCACCCTCTGTATTACCGCGGCTGCTGGCACNNAGTTAGC  968
             |||||||||||||||||||||||||||||||||||||||||||||||||||  |||||||
Sbjct  558   AGTAATTCCGATTAACGCTTGCACCCTCTGTATTACCGCGGCTGCTGGCACAGAGTTAGC  499

Query  969   CNGGTGCTTATTCTGTCGGTAACGTCAAAACAATCACGTATTAGGTAACT  1018
             | ||||||||||||||||||||||||||||||||||||||||||||||||
Sbjct  498   C-GGTGCTTATTCTGTCGGTAACGTCAAAACAATCACGTATTAGGTAACT  450
```

Download

FASTA (complete sequence)

FASTA (aligned sequences)

GenBank (complete sequence)

Continue
Cancel

GenBankGraphics

Next
Previous
Descriptions

Uncultured bacterium clone 21-23 16S ribosomal RNA gene, partial sequence

Sequence ID: KT029459.1Length: 1461Number of Matches: 1

Related Information

Range 1: 426 to 1432GenBankGraphics

Next Match
Previous Match
First Match

Alignment statistics for match #1

| Score | Expect | Identities | Gaps | Strand | Frame |
| --- | --- | --- | --- | --- | --- |
| 1827 bits(989) | 0.0() | 1002/1010(99%) | 3/1010(0%) | Plus/Minus |  |

Features:

```
Query  9     CCGTCCCCCCGAAGGTTAGACTAGCTACTTCTGGTNCAACCCACTCCCATGGTGTGACGG  68
             ||||||||||||||||||||||||||||||||||| ||||||||||||||||||||||||
Sbjct  1432  CCGTCCCCCCGAAGGTTAGACTAGCTACTTCTGGTGCAACCCACTCCCATGGTGTGACGG  1373

Query  69    GCGGTGTGTACAAGGCCCGGGAACGTATTCACCGCGACATTCTGATTCGCGATTACTAGC  128
             ||||||||||||||||||||||||||||||||||||||||||||||||||||||||||||
Sbjct  1372  GCGGTGTGTACAAGGCCCGGGAACGTATTCACCGCGACATTCTGATTCGCGATTACTAGC  1313

Query  129   GATTCCGACTTCACGCAGTCGAGTTGCAGACTGCGATCCGGACTACGATCGGTTTTGTGA  188
             ||||||||||||||||||||||||||||||||||||||||||||||||||||||||||||
Sbjct  1312  GATTCCGACTTCACGCAGTCGAGTTGCAGACTGCGATCCGGACTACGATCGGTTTTGTGA  1253

Query  189   GATTAGCTCCACCTCGCGGCTTGGCAACCCTCTGTACCGACCATTGTAGCACGTGTGTAG  248
             ||||||||||||||||||||||||||||||||||||||||||||||||||||||||||||
Sbjct  1252  GATTAGCTCCACCTCGCGGCTTGGCAACCCTCTGTACCGACCATTGTAGCACGTGTGTAG  1193

Query  249   CCCAGGCCGTAAGGGCCATGATGACTTGACGTCATCCCCACCTTCCTCCGGTTTGTCACC  308
             ||||||||||||||||||||||||||||||||||||||||||||||||||||||||||||
Sbjct  1192  CCCAGGCCGTAAGGGCCATGATGACTTGACGTCATCCCCACCTTCCTCCGGTTTGTCACC  1133

Query  309   GGCAGTCTCCTTAGAGTGCCCACCTTAACGTGCTGGTAACTAAGGACAAGGGTTGCGCTC  368
             ||||||||||||||||||||||||||||||||||||||||||||||||||||||||||||
Sbjct  1132  GGCAGTCTCCTTAGAGTGCCCACCTTAACGTGCTGGTAACTAAGGACAAGGGTTGCGCTC  1073

Query  369   GTTACGGGACTTAACCCAACATCTCACGACACGAGCTGACGACAGCCATGCAGCACCTGT  428
             ||||||||||||||||||||||||||||||||||||||||||||||||||||||||||||
Sbjct  1072  GTTACGGGACTTAACCCAACATCTCACGACACGAGCTGACGACAGCCATGCAGCACCTGT  1013

Query  429   CTCAATGCTCCCGAAGGCACCAATCCATCTCTGGAAAGTTCATTGGATGTCAAGGCCTGG  488
             ||||||||||||||||||||||||||||||||||||||||||||||||||||||||||||
Sbjct  1012  CTCAATGCTCCCGAAGGCACCAATCCATCTCTGGAAAGTTCATTGGATGTCAAGGCCTGG  953

Query  489   TAAGGTTCTTCGCGTTGCTTCGAATTAAACCACATGCTCCACCGCTTGTGCGGGCCCCCG  548
             ||||||||||||||||||||||||||||||||||||||||||||||||||||||||||||
Sbjct  952   TAAGGTTCTTCGCGTTGCTTCGAATTAAACCACATGCTCCACCGCTTGTGCGGGCCCCCG  893

Query  549   TCAATTCATTTGAGTTTTAACCTTGCGGCCGTACTCCCCAGGCGGTCAACTTAATGCGTT  608
             ||||||||||||||||||||||||||||||||||||||||||||||||||||||||||||
Sbjct  892   TCAATTCATTTGAGTTTTAACCTTGCGGCCGTACTCCCCAGGCGGTCAACTTAATGCGTT  833

Query  609   AGCTGCGCCACTAAGAGCTCAAGGCTCCCAACGGCTAGTTGACATCGTTTACGGCGTGGA  668
             ||||||||||||||||||||||||||||||||||||||||||||||||||||||||||||
Sbjct  832   AGCTGCGCCACTAAGAGCTCAAGGCTCCCAACGGCTAGTTGACATCGTTTACGGCGTGGA  773

Query  669   CTACCAGGGTATCTAATCCTGTTTGCTCCCCACGCTTTCGCACCTCAGTGTCAGTATCAG  728
             ||||||||||||||||||||||||||||||||||||||||||||||||||||||||||||
Sbjct  772   CTACCAGGGTATCTAATCCTGTTTGCTCCCCACGCTTTCGCACCTCAGTGTCAGTATCAG  713

Query  729   TCCAGGTGGTCGCCTTCGCCACTGGTGTTCCTTCCTATATCTACGCATTTCACCGCTACA  788
             ||||||||||||||||||||||||||||||||||||||||||||||||||||||||||||
Sbjct  712   TCCAGGTGGTCGCCTTCGCCACTGGTGTTCCTTCCTATATCTACGCATTTCACCGCTACA  653

Query  789   CAGGAAATTCCACCACCCTCTACCATACTCTAGCTTGCCAGTTTTGGATGCAGTTCCCAG  848
             ||||||||||||||||||||||||||||||||||||||||||||||||||||||||||||
Sbjct  652   CAGGAAATTCCACCACCCTCTACCATACTCTAGCTTGCCAGTTTTGGATGCAGTTCCCAG  593

Query  849   GTTGAGCCCGGGGGATTTCACATTCAACTTAACAAACCACCTACGCGNNNCTTTACGCCC  908
             ||||||||||||| |||||||||||||||||||||||||||||||||   ||||||||||
Sbjct  592   GTTGAGCCCGGGG-ATTTCACATTCAACTTAACAAACCACCTACGCGCG-CTTTACGCCC  535

Query  909   AGTAATTCCGATTAACGCTTGCACCCTCTGTATTACCGCGGCTGCTGGCACNNAGTTAGC  968
             |||||||||||||||||||||||||||||||||||||||||||||||||||  |||||||
Sbjct  534   AGTAATTCCGATTAACGCTTGCACCCTCTGTATTACCGCGGCTGCTGGCACAGAGTTAGC  475

Query  969   CNGGTGCTTATTCTGTCGGTAACGTCAAAACAATCACGTATTAGGTAACT  1018
             | ||||||||||||||||||||||||||||||||||||||||||||||||
Sbjct  474   C-GGTGCTTATTCTGTCGGTAACGTCAAAACAATCACGTATTAGGTAACT  426
```

Download

FASTA (complete sequence)

FASTA (aligned sequences)

GenBank (complete sequence)

Continue
Cancel

GenBankGraphics

Next
Previous
Descriptions

Pseudomonas savastanoi pv. nerii strain IPPBC-O38 16S ribosomal RNA gene, partial sequence

Sequence ID: HQ840767.1Length: 1408Number of Matches: 1

Related Information

Range 1: 400 to 1406GenBankGraphics

Next Match
Previous Match
First Match

Alignment statistics for match #1

| Score | Expect | Identities | Gaps | Strand | Frame |
| --- | --- | --- | --- | --- | --- |
| 1827 bits(989) | 0.0() | 1002/1010(99%) | 3/1010(0%) | Plus/Minus |  |

Features:

```
Query  9     CCGTCCCCCCGAAGGTTAGACTAGCTACTTCTGGTNCAACCCACTCCCATGGTGTGACGG  68
             ||||||||||||||||||||||||||||||||||| ||||||||||||||||||||||||
Sbjct  1406  CCGTCCCCCCGAAGGTTAGACTAGCTACTTCTGGTGCAACCCACTCCCATGGTGTGACGG  1347

Query  69    GCGGTGTGTACAAGGCCCGGGAACGTATTCACCGCGACATTCTGATTCGCGATTACTAGC  128
             ||||||||||||||||||||||||||||||||||||||||||||||||||||||||||||
Sbjct  1346  GCGGTGTGTACAAGGCCCGGGAACGTATTCACCGCGACATTCTGATTCGCGATTACTAGC  1287

Query  129   GATTCCGACTTCACGCAGTCGAGTTGCAGACTGCGATCCGGACTACGATCGGTTTTGTGA  188
             ||||||||||||||||||||||||||||||||||||||||||||||||||||||||||||
Sbjct  1286  GATTCCGACTTCACGCAGTCGAGTTGCAGACTGCGATCCGGACTACGATCGGTTTTGTGA  1227

Query  189   GATTAGCTCCACCTCGCGGCTTGGCAACCCTCTGTACCGACCATTGTAGCACGTGTGTAG  248
             ||||||||||||||||||||||||||||||||||||||||||||||||||||||||||||
Sbjct  1226  GATTAGCTCCACCTCGCGGCTTGGCAACCCTCTGTACCGACCATTGTAGCACGTGTGTAG  1167

Query  249   CCCAGGCCGTAAGGGCCATGATGACTTGACGTCATCCCCACCTTCCTCCGGTTTGTCACC  308
             ||||||||||||||||||||||||||||||||||||||||||||||||||||||||||||
Sbjct  1166  CCCAGGCCGTAAGGGCCATGATGACTTGACGTCATCCCCACCTTCCTCCGGTTTGTCACC  1107

Query  309   GGCAGTCTCCTTAGAGTGCCCACCTTAACGTGCTGGTAACTAAGGACAAGGGTTGCGCTC  368
             ||||||||||||||||||||||||||||||||||||||||||||||||||||||||||||
Sbjct  1106  GGCAGTCTCCTTAGAGTGCCCACCTTAACGTGCTGGTAACTAAGGACAAGGGTTGCGCTC  1047

Query  369   GTTACGGGACTTAACCCAACATCTCACGACACGAGCTGACGACAGCCATGCAGCACCTGT  428
             ||||||||||||||||||||||||||||||||||||||||||||||||||||||||||||
Sbjct  1046  GTTACGGGACTTAACCCAACATCTCACGACACGAGCTGACGACAGCCATGCAGCACCTGT  987

Query  429   CTCAATGCTCCCGAAGGCACCAATCCATCTCTGGAAAGTTCATTGGATGTCAAGGCCTGG  488
             ||||||||||||||||||||||||||||||||||||||||||||||||||||||||||||
Sbjct  986   CTCAATGCTCCCGAAGGCACCAATCCATCTCTGGAAAGTTCATTGGATGTCAAGGCCTGG  927

Query  489   TAAGGTTCTTCGCGTTGCTTCGAATTAAACCACATGCTCCACCGCTTGTGCGGGCCCCCG  548
             ||||||||||||||||||||||||||||||||||||||||||||||||||||||||||||
Sbjct  926   TAAGGTTCTTCGCGTTGCTTCGAATTAAACCACATGCTCCACCGCTTGTGCGGGCCCCCG  867

Query  549   TCAATTCATTTGAGTTTTAACCTTGCGGCCGTACTCCCCAGGCGGTCAACTTAATGCGTT  608
             ||||||||||||||||||||||||||||||||||||||||||||||||||||||||||||
Sbjct  866   TCAATTCATTTGAGTTTTAACCTTGCGGCCGTACTCCCCAGGCGGTCAACTTAATGCGTT  807

Query  609   AGCTGCGCCACTAAGAGCTCAAGGCTCCCAACGGCTAGTTGACATCGTTTACGGCGTGGA  668
             ||||||||||||||||||||||||||||||||||||||||||||||||||||||||||||
Sbjct  806   AGCTGCGCCACTAAGAGCTCAAGGCTCCCAACGGCTAGTTGACATCGTTTACGGCGTGGA  747

Query  669   CTACCAGGGTATCTAATCCTGTTTGCTCCCCACGCTTTCGCACCTCAGTGTCAGTATCAG  728
             ||||||||||||||||||||||||||||||||||||||||||||||||||||||||||||
Sbjct  746   CTACCAGGGTATCTAATCCTGTTTGCTCCCCACGCTTTCGCACCTCAGTGTCAGTATCAG  687

Query  729   TCCAGGTGGTCGCCTTCGCCACTGGTGTTCCTTCCTATATCTACGCATTTCACCGCTACA  788
             ||||||||||||||||||||||||||||||||||||||||||||||||||||||||||||
Sbjct  686   TCCAGGTGGTCGCCTTCGCCACTGGTGTTCCTTCCTATATCTACGCATTTCACCGCTACA  627

Query  789   CAGGAAATTCCACCACCCTCTACCATACTCTAGCTTGCCAGTTTTGGATGCAGTTCCCAG  848
             ||||||||||||||||||||||||||||||||||||||||||||||||||||||||||||
Sbjct  626   CAGGAAATTCCACCACCCTCTACCATACTCTAGCTTGCCAGTTTTGGATGCAGTTCCCAG  567

Query  849   GTTGAGCCCGGGGGATTTCACATTCAACTTAACAAACCACCTACGCGNNNCTTTACGCCC  908
             ||||||||||||| |||||||||||||||||||||||||||||||||   ||||||||||
Sbjct  566   GTTGAGCCCGGGG-ATTTCACATTCAACTTAACAAACCACCTACGCGCG-CTTTACGCCC  509

Query  909   AGTAATTCCGATTAACGCTTGCACCCTCTGTATTACCGCGGCTGCTGGCACNNAGTTAGC  968
             |||||||||||||||||||||||||||||||||||||||||||||||||||  |||||||
Sbjct  508   AGTAATTCCGATTAACGCTTGCACCCTCTGTATTACCGCGGCTGCTGGCACAGAGTTAGC  449

Query  969   CNGGTGCTTATTCTGTCGGTAACGTCAAAACAATCACGTATTAGGTAACT  1018
             | ||||||||||||||||||||||||||||||||||||||||||||||||
Sbjct  448   C-GGTGCTTATTCTGTCGGTAACGTCAAAACAATCACGTATTAGGTAACT  400
```

Download

FASTA (complete sequence)

FASTA (aligned sequences)

GenBank (complete sequence)

Continue
Cancel

GenBankGraphics

Next
Previous
Descriptions

Pseudomonas sp. Ina1 partial 16S rRNA gene, strain Ina1

Sequence ID: AM909655.1Length: 1538Number of Matches: 1

Related Information

Range 1: 83 to 1089GenBankGraphics

Next Match
Previous Match
First Match

Alignment statistics for match #1

| Score | Expect | Identities | Gaps | Strand | Frame |
| --- | --- | --- | --- | --- | --- |
| 1827 bits(989) | 0.0() | 1002/1010(99%) | 3/1010(0%) | Plus/Plus |  |

Features:

```
Query  9     CCGTCCCCCCGAAGGTTAGACTAGCTACTTCTGGTNCAACCCACTCCCATGGTGTGACGG  68
             ||||||||||||||||||||||||||||||||||| ||||||||||||||||||||||||
Sbjct  83    CCGTCCCCCCGAAGGTTAGACTAGCTACTTCTGGTGCAACCCACTCCCATGGTGTGACGG  142

Query  69    GCGGTGTGTACAAGGCCCGGGAACGTATTCACCGCGACATTCTGATTCGCGATTACTAGC  128
             ||||||||||||||||||||||||||||||||||||||||||||||||||||||||||||
Sbjct  143   GCGGTGTGTACAAGGCCCGGGAACGTATTCACCGCGACATTCTGATTCGCGATTACTAGC  202

Query  129   GATTCCGACTTCACGCAGTCGAGTTGCAGACTGCGATCCGGACTACGATCGGTTTTGTGA  188
             ||||||||||||||||||||||||||||||||||||||||||||||||||||||||||||
Sbjct  203   GATTCCGACTTCACGCAGTCGAGTTGCAGACTGCGATCCGGACTACGATCGGTTTTGTGA  262

Query  189   GATTAGCTCCACCTCGCGGCTTGGCAACCCTCTGTACCGACCATTGTAGCACGTGTGTAG  248
             ||||||||||||||||||||||||||||||||||||||||||||||||||||||||||||
Sbjct  263   GATTAGCTCCACCTCGCGGCTTGGCAACCCTCTGTACCGACCATTGTAGCACGTGTGTAG  322

Query  249   CCCAGGCCGTAAGGGCCATGATGACTTGACGTCATCCCCACCTTCCTCCGGTTTGTCACC  308
             ||||||||||||||||||||||||||||||||||||||||||||||||||||||||||||
Sbjct  323   CCCAGGCCGTAAGGGCCATGATGACTTGACGTCATCCCCACCTTCCTCCGGTTTGTCACC  382

Query  309   GGCAGTCTCCTTAGAGTGCCCACCTTAACGTGCTGGTAACTAAGGACAAGGGTTGCGCTC  368
             ||||||||||||||||||||||||||||||||||||||||||||||||||||||||||||
Sbjct  383   GGCAGTCTCCTTAGAGTGCCCACCTTAACGTGCTGGTAACTAAGGACAAGGGTTGCGCTC  442

Query  369   GTTACGGGACTTAACCCAACATCTCACGACACGAGCTGACGACAGCCATGCAGCACCTGT  428
             ||||||||||||||||||||||||||||||||||||||||||||||||||||||||||||
Sbjct  443   GTTACGGGACTTAACCCAACATCTCACGACACGAGCTGACGACAGCCATGCAGCACCTGT  502

Query  429   CTCAATGCTCCCGAAGGCACCAATCCATCTCTGGAAAGTTCATTGGATGTCAAGGCCTGG  488
             ||||||||||||||||||||||||||||||||||||||||||||||||||||||||||||
Sbjct  503   CTCAATGCTCCCGAAGGCACCAATCCATCTCTGGAAAGTTCATTGGATGTCAAGGCCTGG  562

Query  489   TAAGGTTCTTCGCGTTGCTTCGAATTAAACCACATGCTCCACCGCTTGTGCGGGCCCCCG  548
             ||||||||||||||||||||||||||||||||||||||||||||||||||||||||||||
Sbjct  563   TAAGGTTCTTCGCGTTGCTTCGAATTAAACCACATGCTCCACCGCTTGTGCGGGCCCCCG  622

Query  549   TCAATTCATTTGAGTTTTAACCTTGCGGCCGTACTCCCCAGGCGGTCAACTTAATGCGTT  608
             ||||||||||||||||||||||||||||||||||||||||||||||||||||||||||||
Sbjct  623   TCAATTCATTTGAGTTTTAACCTTGCGGCCGTACTCCCCAGGCGGTCAACTTAATGCGTT  682

Query  609   AGCTGCGCCACTAAGAGCTCAAGGCTCCCAACGGCTAGTTGACATCGTTTACGGCGTGGA  668
             ||||||||||||||||||||||||||||||||||||||||||||||||||||||||||||
Sbjct  683   AGCTGCGCCACTAAGAGCTCAAGGCTCCCAACGGCTAGTTGACATCGTTTACGGCGTGGA  742

Query  669   CTACCAGGGTATCTAATCCTGTTTGCTCCCCACGCTTTCGCACCTCAGTGTCAGTATCAG  728
             ||||||||||||||||||||||||||||||||||||||||||||||||||||||||||||
Sbjct  743   CTACCAGGGTATCTAATCCTGTTTGCTCCCCACGCTTTCGCACCTCAGTGTCAGTATCAG  802

Query  729   TCCAGGTGGTCGCCTTCGCCACTGGTGTTCCTTCCTATATCTACGCATTTCACCGCTACA  788
             ||||||||||||||||||||||||||||||||||||||||||||||||||||||||||||
Sbjct  803   TCCAGGTGGTCGCCTTCGCCACTGGTGTTCCTTCCTATATCTACGCATTTCACCGCTACA  862

Query  789   CAGGAAATTCCACCACCCTCTACCATACTCTAGCTTGCCAGTTTTGGATGCAGTTCCCAG  848
             ||||||||||||||||||||||||||||||||||||||||||||||||||||||||||||
Sbjct  863   CAGGAAATTCCACCACCCTCTACCATACTCTAGCTTGCCAGTTTTGGATGCAGTTCCCAG  922

Query  849   GTTGAGCCCGGGGGATTTCACATTCAACTTAACAAACCACCTACGCGNNNCTTTACGCCC  908
             ||||||||| |||||||||||||||||||||||||||||||||||||   ||||||||||
Sbjct  923   GTTGAGCCC-GGGGATTTCACATTCAACTTAACAAACCACCTACGCGCG-CTTTACGCCC  980

Query  909   AGTAATTCCGATTAACGCTTGCACCCTCTGTATTACCGCGGCTGCTGGCACNNAGTTAGC  968
             |||||||||||||||||||||||||||||||||||||||||||||||||||  |||||||
Sbjct  981   AGTAATTCCGATTAACGCTTGCACCCTCTGTATTACCGCGGCTGCTGGCACAGAGTTAGC  1040

Query  969   CNGGTGCTTATTCTGTCGGTAACGTCAAAACAATCACGTATTAGGTAACT  1018
             | ||||||||||||||||||||||||||||||||||||||||||||||||
Sbjct  1041  C-GGTGCTTATTCTGTCGGTAACGTCAAAACAATCACGTATTAGGTAACT  1089
```

Download

FASTA (complete sequence)

FASTA (aligned sequences)

GenBank (complete sequence)

Continue
Cancel

GenBankGraphics

Next
Previous
Descriptions

Pseudomonas sp. HP7P 16S ribosomal RNA gene, partial sequence

Sequence ID: KM187525.1Length: 1402Number of Matches: 1

Related Information

Range 1: 397 to 1402GenBankGraphics

Next Match
Previous Match
First Match

Alignment statistics for match #1

| Score | Expect | Identities | Gaps | Strand | Frame |
| --- | --- | --- | --- | --- | --- |
| 1825 bits(988) | 0.0() | 1001/1009(99%) | 3/1009(0%) | Plus/Minus |  |

Features:

```
Query  10    CGTCCCCCCGAAGGTTAGACTAGCTACTTCTGGTNCAACCCACTCCCATGGTGTGACGGG  69
             |||||||||||||||||||||||||||||||||| |||||||||||||||||||||||||
Sbjct  1402  CGTCCCCCCGAAGGTTAGACTAGCTACTTCTGGTGCAACCCACTCCCATGGTGTGACGGG  1343

Query  70    CGGTGTGTACAAGGCCCGGGAACGTATTCACCGCGACATTCTGATTCGCGATTACTAGCG  129
             ||||||||||||||||||||||||||||||||||||||||||||||||||||||||||||
Sbjct  1342  CGGTGTGTACAAGGCCCGGGAACGTATTCACCGCGACATTCTGATTCGCGATTACTAGCG  1283

Query  130   ATTCCGACTTCACGCAGTCGAGTTGCAGACTGCGATCCGGACTACGATCGGTTTTGTGAG  189
             ||||||||||||||||||||||||||||||||||||||||||||||||||||||||||||
Sbjct  1282  ATTCCGACTTCACGCAGTCGAGTTGCAGACTGCGATCCGGACTACGATCGGTTTTGTGAG  1223

Query  190   ATTAGCTCCACCTCGCGGCTTGGCAACCCTCTGTACCGACCATTGTAGCACGTGTGTAGC  249
             ||||||||||||||||||||||||||||||||||||||||||||||||||||||||||||
Sbjct  1222  ATTAGCTCCACCTCGCGGCTTGGCAACCCTCTGTACCGACCATTGTAGCACGTGTGTAGC  1163

Query  250   CCAGGCCGTAAGGGCCATGATGACTTGACGTCATCCCCACCTTCCTCCGGTTTGTCACCG  309
             ||||||||||||||||||||||||||||||||||||||||||||||||||||||||||||
Sbjct  1162  CCAGGCCGTAAGGGCCATGATGACTTGACGTCATCCCCACCTTCCTCCGGTTTGTCACCG  1103

Query  310   GCAGTCTCCTTAGAGTGCCCACCTTAACGTGCTGGTAACTAAGGACAAGGGTTGCGCTCG  369
             ||||||||||||||||||||||||||||||||||||||||||||||||||||||||||||
Sbjct  1102  GCAGTCTCCTTAGAGTGCCCACCTTAACGTGCTGGTAACTAAGGACAAGGGTTGCGCTCG  1043

Query  370   TTACGGGACTTAACCCAACATCTCACGACACGAGCTGACGACAGCCATGCAGCACCTGTC  429
             ||||||||||||||||||||||||||||||||||||||||||||||||||||||||||||
Sbjct  1042  TTACGGGACTTAACCCAACATCTCACGACACGAGCTGACGACAGCCATGCAGCACCTGTC  983

Query  430   TCAATGCTCCCGAAGGCACCAATCCATCTCTGGAAAGTTCATTGGATGTCAAGGCCTGGT  489
             ||||||||||||||||||||||||||||||||||||||||||||||||||||||||||||
Sbjct  982   TCAATGCTCCCGAAGGCACCAATCCATCTCTGGAAAGTTCATTGGATGTCAAGGCCTGGT  923

Query  490   AAGGTTCTTCGCGTTGCTTCGAATTAAACCACATGCTCCACCGCTTGTGCGGGCCCCCGT  549
             ||||||||||||||||||||||||||||||||||||||||||||||||||||||||||||
Sbjct  922   AAGGTTCTTCGCGTTGCTTCGAATTAAACCACATGCTCCACCGCTTGTGCGGGCCCCCGT  863

Query  550   CAATTCATTTGAGTTTTAACCTTGCGGCCGTACTCCCCAGGCGGTCAACTTAATGCGTTA  609
             ||||||||||||||||||||||||||||||||||||||||||||||||||||||||||||
Sbjct  862   CAATTCATTTGAGTTTTAACCTTGCGGCCGTACTCCCCAGGCGGTCAACTTAATGCGTTA  803

Query  610   GCTGCGCCACTAAGAGCTCAAGGCTCCCAACGGCTAGTTGACATCGTTTACGGCGTGGAC  669
             ||||||||||||||||||||||||||||||||||||||||||||||||||||||||||||
Sbjct  802   GCTGCGCCACTAAGAGCTCAAGGCTCCCAACGGCTAGTTGACATCGTTTACGGCGTGGAC  743

Query  670   TACCAGGGTATCTAATCCTGTTTGCTCCCCACGCTTTCGCACCTCAGTGTCAGTATCAGT  729
             ||||||||||||||||||||||||||||||||||||||||||||||||||||||||||||
Sbjct  742   TACCAGGGTATCTAATCCTGTTTGCTCCCCACGCTTTCGCACCTCAGTGTCAGTATCAGT  683

Query  730   CCAGGTGGTCGCCTTCGCCACTGGTGTTCCTTCCTATATCTACGCATTTCACCGCTACAC  789
             ||||||||||||||||||||||||||||||||||||||||||||||||||||||||||||
Sbjct  682   CCAGGTGGTCGCCTTCGCCACTGGTGTTCCTTCCTATATCTACGCATTTCACCGCTACAC  623

Query  790   AGGAAATTCCACCACCCTCTACCATACTCTAGCTTGCCAGTTTTGGATGCAGTTCCCAGG  849
             ||||||||||||||||||||||||||||||||||||||||||||||||||||||||||||
Sbjct  622   AGGAAATTCCACCACCCTCTACCATACTCTAGCTTGCCAGTTTTGGATGCAGTTCCCAGG  563

Query  850   TTGAGCCCGGGGGATTTCACATTCAACTTAACAAACCACCTACGCGNNNCTTTACGCCCA  909
             |||||||||||| |||||||||||||||||||||||||||||||||   |||||||||||
Sbjct  562   TTGAGCCCGGGG-ATTTCACATTCAACTTAACAAACCACCTACGCGCG-CTTTACGCCCA  505

Query  910   GTAATTCCGATTAACGCTTGCACCCTCTGTATTACCGCGGCTGCTGGCACNNAGTTAGCC  969
             ||||||||||||||||||||||||||||||||||||||||||||||||||  ||||||||
Sbjct  504   GTAATTCCGATTAACGCTTGCACCCTCTGTATTACCGCGGCTGCTGGCACAGAGTTAGCC  445

Query  970   NGGTGCTTATTCTGTCGGTAACGTCAAAACAATCACGTATTAGGTAACT  1018
              ||||||||||||||||||||||||||||||||||||||||||||||||
Sbjct  444   -GGTGCTTATTCTGTCGGTAACGTCAAAACAATCACGTATTAGGTAACT  397
```


BLAST is a registered trademark of the National Library of Medicine

Support center
Mailing list


YouTube

- National Library Of Medicine
- National Institutes Of Health
- U.S. Department of Health & Human Services
- USA.gov

### NCBI


National Center for Biotechnology Information,
 U.S. National Library of Medicine

8600 Rockville Pike,
Bethesda
 MD,
20894
USA

Policies and Guidelines
|
Contact


PreferencesTurn off

External link. Please review our privacy policy.
