# Supplementary material for: Isothermal microcalorimetry for thermal viable count of microorganisms in pure cultures and stabilized formulations
Source: BMC Microbiol. 2019 Mar 21;19:65. doi: 10.1186/s12866-019-1432-8 (PMC6429831; doi:10.1186/s12866-019-1432-8)
Supplement: Supplementary file 3 — 16S rRNA gene sequences’ BLASTN hits in zipped HTML format. (ZIP 15810 kb) [file 12866_2019_1432_MOESM3_ESM.zip › Best blastn hits/NCBI Blast_M yellow SS F -- 20..1038 of sequence (1019.html]

NCBI Blast:M yellow SS F -- 20..1038 of sequence (1019


- NCBI Home
- Sign in to NCBI
- Skip to Main Content
- Skip to Navigation
- About NCBI Accesskeys

U.S. National Library of Medicine

NCBI
National Center for Biotechnology Information

- My NCBI
- Sign in to NCBI
- Register
- Sign Out

BLAST ^®^ » blastn suite » RID-A50HJ5NR015


- Home
- Recent Results
- Saved Strategies
- Help

BLAST Results


Edit and Resubmit
Save Search Strategies
[Sign in above to save your search strategy]

Formatting options 


Download


How to read this page
Blast report description
Questions/comments


|  |  |
| --- | --- |
| Formatting options | |
| Show | Alignment as  HTML Plain text   Old View Reset form to defaults [?]  These options control formatting of alignments in results pages. The default is HTML, but other formats (including plain text) are available. PSSM and PssmWithParameters are representations of Position Specific Scoring Matrices and are only available for PSI-BLAST. The Advanced view option allows the database descriptions to be sorted by various indices in a table. |
| Alignment View | Pairwise Pairwise with dots for identities Query-anchored with dots for identities Query-anchored with letters for identities Flat query-anchored with dots for identities Flat query-anchored with letters for identities [?]  Choose how to view alignments. The default "pairwise" view shows how each subject sequence aligns individually to the query sequence. The "query-anchored" view shows how all subject sequences align to the query sequence. For each view type, you can choose to show "identities" (matching residues) as letters or dots. more... |
| Display | Graphical Overview   Linkout   Sequence Retrieval  NCBI-gi   CDS feature [?]  - Graphical Overview: Graphical Overview: Show graph of similar sequence regions aligned to query.   more... - NCBI-gi: Show NCBI gi identifiers. - CDS feature: Show annotated coding region and translation.   more... |
| Masking | Character:   X for protein, n for nucleotide Lower Case  Color:  Black Grey Red [?]  - Masking Character: Display masked (filtered) sequence regions as lower-case or as specific letters (N for nucleotide, P for protein). - Masking Color: Display masked sequence regions in the given color. |
| Limit results | Descriptions:  10 50 100 Graphical overview:  0 10 50 100  Alignments:  0 10 50 100 Line length:  60 90 120 150 [?]  - Descriptions: Show short descriptions for up to the given number of sequences. - Alignments: Show alignments for up to the given number of sequences, in order of statistical significance. - Line lenghth: Number of letters to show on one line in an alignment. |
|  | Organism Type common name, binomial, taxid, or group name. Only 20 top taxa will be shown.     Exclude   [?]  Show only sequences from the given organism. |
|  | Entrez query:  [?]  Show only those sequences that match the given Entrez query. more... |
|  | Expect Min:  Expect Max:  [?]  Show only sequences with expect values in the given range. more... |
|  | Percent Identity Min:  Percent Identity Max:  [?]  Show only sequences with percent identity values in the given range. |
| Format for | PSI-BLAST with inclusion threshold:  [?]  - Format for PSI-BLAST: The Position-Specific Iterated BLAST (PSI-BLAST) program performs iterative searches with a protein query,   in which sequences found in one round of search are used to build a custom score model for the next round.   more... - Inclusion Threshold: This sets the statistical significance threshold for including a sequence in the model used   by PSI-BLAST to create the PSSM on the next iteration. |

|  |  |  |  |  |  |
| --- | --- | --- | --- | --- | --- |
| Download | | | | | |
| Alignment  Text XML ASN.1 JSON Seq-align Hit Table(text) Hit Table(csv) Multiple-file XML2 Single-file XML2 Multiple-file JSON Single-file JSON SAM | Search Strategies  ASN.1 | PSSM to restart search  PSSM | [?] |  |  |

The Download link provides BLAST output that may be used as input to another program.
This includes parseable formats such as the tabular report or XML as well as the Search Strategy files read by the BLAST+ applications.
More details on the parseable (XML, tabular, and ASN.1) reports can be found at
https://www.ncbi.nlm.nih.gov/books/NBK153387/  
  

The following formats are offered under the Alignment section:  
1). "Text". Non-HTML standard BLAST report.  
2). "XML". XML report based upon the DTD at https://www.ncbi.nlm.nih.gov/data\_specs/dtd/NCBI\_BlastOutput.dtd  
3). "ASN.1". Alignment written out in Abstract Syntax Notation 1.  
4). "JSON Seq-align". Alignment written out in JSON.  
4). "Hit Table(text)". The tabular report as text.  
5). "Hit Table(csv)". The tabular report ready for import into spread-sheet programs like Excel.  
6). "XML2". New XML format described at ftp://ftp.ncbi.nlm.nih.gov/blast/documents/NEWXML/xml2.pdf.  
7). "JSON". New JSON format described at ftp://ftp.ncbi.nlm.nih.gov/blast/documents/NEWXML/xml2.pdf.  
8). "SAM". Sequence Alignment Map format.

XML2 and JSON can be downloaded either as one file per query (multiple-file) or one file for all queries (single-file). These formats are listed as Multiple-file XML2 (and JSON) or Single-file XML (and JSON).

The following report is offered under the Search Strategy section:  
1). "ASN.1" Search Strategy. A record of the parameters, query, and database used in the search. This file can be used to start a stand-alone BLAST search, see
https://www.ncbi.nlm.nih.gov/books/NBK1763/#CmdLineAppsManual.I455\_BLAST\_search\_stra


# Job title: M yellow SS F -- 20..1038 of sequence (1019

Results for:

lcl|Query\_114267 M yellow SS F -- 20..1038 of sequence(1019bp)
[?]

Your BLAST job specified more than one input sequence.
This box lets you choose which input sequence to show BLAST results for.

RID
:   A50HJ5NR015 (Expires on 03-10 19:46 pm)

Query ID
:   lcl|Query\_114267
:   lcl|Query\_114267

Description
:   M yellow SS F -- 20..1038 of sequence

Molecule type
:   nucleic acid

Query Length
:   1019

Database Name
:   nr

Description
:   Nucleotide collection (nt) See details

Program
:   BLASTN 2.8.0+ Citation

  

Reference 

Zheng Zhang, Scott Schwartz, Lukas Wagner, and Webb Miller (2000), "A greedy algorithm for aligning DNA sequences", J Comput Biol 2000; 7(1-2):203-14.

Reference - database indexing

Aleksandr Morgulis, George Coulouris, Yan Raytselis, Thomas L. Madden, Richa Agarwala, Alejandro A. Schäffer (2008), "Database Indexing for Production MegaBLAST Searches", Bioinformatics 24:1757-1764.

Other reports:
Search Summary

[Taxonomy reports]
[Distance tree of results]
[MSA viewer]

Search Parameters

| Search parameter name | Search parameter value |
| --- | --- |
| Program | blastn |
| Word size | 28 |
| Expect value | 10 |
| Hitlist size | 100 |
| Match/Mismatch scores | 1,-2 |
| Gapcosts | 0,2.5 |
| Low Complexity Filter | Yes |
| Filter string | L;m; |
| Genetic Code | 1 |

Database

| Database parameter name | Database parameter value |
| --- | --- |
| Posted date | Mar 7, 2018 1:58 PM |
| Number of letters | 174,044,644,244 |
| Number of sequences | 46,882,714 |
| Entrez query | Includes:  Excludes:  None |

Karlin-Altschul statistics

| Params | Ungapped | Gapped |
| --- | --- | --- |
| Lambda | 1.33271 | 1.28 |
| K | 0.620991 | 0.46 |
| H | 1.12409 | 0.85 |

Results Statistics

| Results Statistics parameter name | Results Statistics parameter value |
| --- | --- |
| Length adjustment | 35 |
| Effective length of query | 984 |
| Effective length of database | 172403749254 |
| Effective search space | 169645289265936 |
| Effective search space used | 169645289265936 |


## Graphic Summary

### Distribution of the top 121 Blast Hits on 100 subject sequences [?]

The graphic is an overview of the database sequences aligned to the query sequence. These are represented horizontal bars colored coded by score and showing the extent
of the alignment on the query sequence. Separate aligned regions on the same database sequence are connected by a thin grey line.
Mousing over an alignment shows the database sequence title. Clicking an alignment displays a box with more details about the alignment and
link to the sequence alignment itself in the Alignments section of the report.

Mouse over to see the title, click to show alignments

Color key for alignment scores

<40

40-50

50-80

80-200

>=200

Query

1

200

400

600

800

1000

Pseudomonas trivialis strain BS3111 genome assembly, ch..

Score:1862 Evalue:0

Accession:LT629760.1

Alignment

Pseudomonas poae strain BS2776 genome assembly, chromos..

Score:1862 Evalue:0

Accession:LT629706.1

Alignment

Pseudomonas poae RE\*1-1-14, complete genome

Score:1862 Evalue:0

Accession:CP004045.1

Alignment

uncultured bacterium partial 16S rRNA gene, clone K15.8..

Score:1862 Evalue:0

Accession:HE576021.1

Alignment

Pseudomonas sp. PX7\_S1 16S ribosomal RNA gene, partial ..

Score:1862 Evalue:0

Accession:JF274937.1

Alignment

Uncultured bacterium clone ncd564g06c1 16S ribosomal RN..

Score:1862 Evalue:0

Accession:HM274624.1

Alignment

Pseudomonas trivialis strain P 513/19 16S ribosomal RNA..

Score:1862 Evalue:0

Accession:NR\_028987.1

Alignment

Pseudomonas poae strain P 527/13 16S ribosomal RNA gene..

Score:1862 Evalue:0

Accession:NR\_028986.1

Alignment

Pseudomonas sp. BBI9 16S ribosomal RNA gene, partial se..

Score:1862 Evalue:0

Accession:GQ868355.1

Alignment

Pseudomonas sp. BR3-39 16S ribosomal RNA gene, partial ..

Score:1862 Evalue:0

Accession:EU853186.1

Alignment

Pseudomonas sp. BR2-22 16S ribosomal RNA gene, partial ..

Score:1862 Evalue:0

Accession:EU853183.1

Alignment

Pseudomonas sp. BR2-02 16S ribosomal RNA gene, partial ..

Score:1862 Evalue:0

Accession:EU853182.1

Alignment

Pseudomonas poae strain T23 16S ribosomal RNA gene, com..

Score:1862 Evalue:0

Accession:EU111704.2

Alignment

Pseudomonas sp. LBSA1 16S ribosomal RNA gene, partial s..

Score:1862 Evalue:0

Accession:AY756059.1

Alignment

Pseudomonas trivialis partial 16S rRNA gene, type strai..

Score:1862 Evalue:0

Accession:AJ492831.1

Alignment

Pseudomonas poae partial 16S rRNA gene, type strain DSM..

Score:1862 Evalue:0

Accession:AJ492829.1

Alignment

Pseudomonas sp. BBI2 16S ribosomal RNA gene, partial se..

Score:1858 Evalue:0

Accession:GQ868350.1

Alignment

Pseudomonas sp. S09G 359 chromosome

Score:1857 Evalue:0

Accession:CP025263.1

Alignment

Pseudomonas sp. strain KBL2 16S ribosomal RNA gene, par..

Score:1857 Evalue:0

Accession:MG576155.1

Alignment

Pseudomonas poae strain A2B5 16S ribosomal RNA gene, pa..

Score:1857 Evalue:0

Accession:KY940313.1

Alignment

Pseudomonas poae strain AAS-144 16S ribosomal RNA gene,..

Score:1857 Evalue:0

Accession:KY810658.1

Alignment

Pseudomonas trivialis strain LMTK28 16S ribosomal RNA g..

Score:1857 Evalue:0

Accession:KY614180.1

Alignment

Pseudomonas poae strain ICE349 16S ribosomal RNA gene, ..

Score:1857 Evalue:0

Accession:KX588598.1

Alignment

Pseudomonas poae strain ICE259 16S ribosomal RNA gene, ..

Score:1857 Evalue:0

Accession:KX588597.1

Alignment

Pseudomonas sp. strain 3-10 16S ribosomal RNA gene, par..

Score:1857 Evalue:0

Accession:KX378948.1

Alignment

Pseudomonas sp. strain 1-29 16S ribosomal RNA gene, par..

Score:1857 Evalue:0

Accession:KX378900.1

Alignment

Pseudomonas sp. RZ110 16S ribosomal RNA gene, partial s..

Score:1857 Evalue:0

Accession:KT710812.1

Alignment

Pseudomonas sp. 8K8 16S ribosomal RNA gene, partial seq..

Score:1857 Evalue:0

Accession:KT825845.1

Alignment

Pseudomonas sp. 23K33a 16S ribosomal RNA gene, partial ..

Score:1857 Evalue:0

Accession:KT825765.1

Alignment

Pseudomonas sp. 17K8 16S ribosomal RNA gene, partial se..

Score:1857 Evalue:0

Accession:KT825736.1

Alignment

Pseudomonas sp. LB8 16S ribosomal RNA gene, partial seq..

Score:1857 Evalue:0

Accession:KT424973.1

Alignment

Pseudomonas sp. PDD-59b-10 16S ribosomal RNA gene, comp..

Score:1857 Evalue:0

Accession:KR922148.1

Alignment

Pseudomonas poae strain B42 16S ribosomal RNA gene, par..

Score:1857 Evalue:0

Accession:KT767705.1

Alignment

Uncultured Pseudomonas sp. partial 16S rRNA gene, clone..

Score:1857 Evalue:0

Accession:LN875096.1

Alignment

Pseudomonas poae strain IHBB 11151 16S ribosomal RNA ge..

Score:1857 Evalue:0

Accession:KR085862.1

Alignment

Pseudomonas poae strain IHBB 9211 16S ribosomal RNA gen..

Score:1857 Evalue:0

Accession:KR085822.1

Alignment

Uncultured bacterium clone OTU51 16S ribosomal RNA gene..

Score:1857 Evalue:0

Accession:KP975307.1

Alignment

Uncultured bacterium clone 19-16 16S ribosomal RNA gene..

Score:1857 Evalue:0

Accession:KT029380.1

Alignment

Uncultured bacterium clone 19-14 16S ribosomal RNA gene..

Score:1857 Evalue:0

Accession:KT029378.1

Alignment

Pseudomonas trivialis strain IHBB745, complete genome

Score:1857 Evalue:0

Accession:CP011507.1

Alignment

Bacterium MA\_26 16S ribosomal RNA gene, partial sequenc..

Score:1857 Evalue:0

Accession:KP204130.1

Alignment

Pseudomonas poae strain MA\_25 16S ribosomal RNA gene, p..

Score:1857 Evalue:0

Accession:KP204129.1

Alignment

Pseudomonas trivialis strain KXL-Y3 16S ribosomal RNA g..

Score:1857 Evalue:0

Accession:KF704111.1

Alignment

Pseudomonas sp. Bma14 16S ribosomal RNA gene, partial s..

Score:1857 Evalue:0

Accession:JQ977483.1

Alignment

Pseudomonas sp. Ms16 16S ribosomal RNA gene, partial se..

Score:1857 Evalue:0

Accession:JQ977324.1

Alignment

Pseudomonas sp. Ms14 16S ribosomal RNA gene, partial se..

Score:1857 Evalue:0

Accession:JQ977322.1

Alignment

Pseudomonas sp. P61-2 16S ribosomal RNA gene, partial s..

Score:1857 Evalue:0

Accession:JX569781.1

Alignment

Pseudomonas salomonii strain CCNWQLS112 16S ribosomal R..

Score:1857 Evalue:0

Accession:JX840381.1

Alignment

Pseudomonas poae strain EB20 16S ribosomal RNA gene, pa..

Score:1857 Evalue:0

Accession:JX515573.1

Alignment

Pseudomonas sp. SAP770.2 16S ribosomal RNA gene, partia..

Score:1857 Evalue:0

Accession:JX067683.1

Alignment

Pseudomonas poae strain zol-15 16S ribosomal RNA gene, ..

Score:1857 Evalue:0

Accession:JQ782898.1

Alignment

Pseudomonas sp. FI 1007 16S ribosomal RNA gene, partial..

Score:1857 Evalue:0

Accession:JQ691542.1

Alignment

Pseudomonas sp. ZBC1 16S ribosomal RNA gene, partial se..

Score:1857 Evalue:0

Accession:JQ638271.1

Alignment

Pseudomonas poae strain MML1950 16S ribosomal RNA gene,..

Score:1857 Evalue:0

Accession:JQ336974.1

Alignment

Pseudomonas poae strain ORC5 16S ribosomal RNA gene, pa..

Score:1857 Evalue:0

Accession:JQ236630.1

Alignment

Pseudomonas sp. SGb136 16S ribosomal RNA gene, partial ..

Score:1857 Evalue:0

Accession:HQ224624.1

Alignment

## Descriptions

, Reading indexes 1-5, displaying indexes 1-5


Load next setPrevious Match

Sequences producing significant alignments:

Show all columns  of the table presenting sequences producing significant alignments 

Select:AllNone
Selected:0

Alignments
Download

FASTA (complete sequence)

FASTA (aligned sequences)

GenBank (complete sequence)

Hit Table (text)

Hit Table (CSV)

Text

XML

ASN.1

Continue
Cancel

GenBank 
Graphics
Distance tree of results
Multiple alignment
Show/hide columns of the table presenting sequences producing significant alignments 

Available columns

Description  
Max Score  
Total Score  
Coverage  
E-value  
IdentN  
Accession  
Restore Defaults
Ok
Cancel

Sequences producing significant alignments:

| Select for downloading or viewing reports | Description | Max score | Total score | Query cover | E value | Ident | Accession |
| --- | --- | --- | --- | --- | --- | --- | --- |
| 1Select seq LT629760.1 | Pseudomonas trivialis strain BS3111 genome assembly, chromosome: I | 1862 | 9307 | 98% | 0.0 | 100% | LT629760.1 |
| 2Select seq LT629706.1 | Pseudomonas poae strain BS2776 genome assembly, chromosome: I | 1862 | 9312 | 98% | 0.0 | 100% | LT629706.1 |
| 3Select seq CP004045.1 | Pseudomonas poae RE\*1-1-14, complete genome | 1862 | 9307 | 98% | 0.0 | 100% | CP004045.1 |
| 4Select seq HE576021.1 | uncultured bacterium partial 16S rRNA gene, clone K15.87 KSS | 1862 | 1862 | 98% | 0.0 | 100% | HE576021.1 |
| 5Select seq JF274937.1 | Pseudomonas sp. PX7\_S1 16S ribosomal RNA gene, partial sequence | 1862 | 1862 | 98% | 0.0 | 100% | JF274937.1 |
| 6Select seq HM274624.1 | Uncultured bacterium clone ncd564g06c1 16S ribosomal RNA gene, partial sequence | 1862 | 1862 | 98% | 0.0 | 100% | HM274624.1 |
| 7Select seq NR\_028987.1 | Pseudomonas trivialis strain P 513/19 16S ribosomal RNA gene, partial sequence | 1862 | 1862 | 98% | 0.0 | 100% | NR\_028987.1 |
| 8Select seq NR\_028986.1 | Pseudomonas poae strain P 527/13 16S ribosomal RNA gene, partial sequence | 1862 | 1862 | 98% | 0.0 | 100% | NR\_028986.1 |
| 9Select seq GQ868355.1 | Pseudomonas sp. BBI9 16S ribosomal RNA gene, partial sequence | 1862 | 1862 | 98% | 0.0 | 100% | GQ868355.1 |
| 10Select seq EU853186.1 | Pseudomonas sp. BR3-39 16S ribosomal RNA gene, partial sequence | 1862 | 1862 | 98% | 0.0 | 100% | EU853186.1 |
| 11Select seq EU853183.1 | Pseudomonas sp. BR2-22 16S ribosomal RNA gene, partial sequence | 1862 | 1862 | 98% | 0.0 | 100% | EU853183.1 |
| 12Select seq EU853182.1 | Pseudomonas sp. BR2-02 16S ribosomal RNA gene, partial sequence | 1862 | 1862 | 98% | 0.0 | 100% | EU853182.1 |
| 13Select seq EU111704.2 | Pseudomonas poae strain T23 16S ribosomal RNA gene, complete sequence | 1862 | 1862 | 98% | 0.0 | 100% | EU111704.2 |
| 14Select seq AY756059.1 | Pseudomonas sp. LBSA1 16S ribosomal RNA gene, partial sequence; internal transcribed spacer 1, complete sequence; and 23S ribosomal RNA gene, partial sequence | 1862 | 1862 | 98% | 0.0 | 100% | AY756059.1 |
| 15Select seq AJ492831.1 | Pseudomonas trivialis partial 16S rRNA gene, type strain DSM 14937T | 1862 | 1862 | 98% | 0.0 | 100% | AJ492831.1 |
| 16Select seq AJ492829.1 | Pseudomonas poae partial 16S rRNA gene, type strain DSM 14936T | 1862 | 1862 | 98% | 0.0 | 100% | AJ492829.1 |
| 17Select seq GQ868350.1 | Pseudomonas sp. BBI2 16S ribosomal RNA gene, partial sequence | 1858 | 1858 | 98% | 0.0 | 100% | GQ868350.1 |
| 18Select seq CP025263.1 | Pseudomonas sp. S09G 359 chromosome | 1857 | 11136 | 98% | 0.0 | 99% | CP025263.1 |
| 19Select seq MG576155.1 | Pseudomonas sp. strain KBL2 16S ribosomal RNA gene, partial sequence | 1857 | 1857 | 98% | 0.0 | 99% | MG576155.1 |
| 20Select seq KY940313.1 | Pseudomonas poae strain A2B5 16S ribosomal RNA gene, partial sequence | 1857 | 1857 | 98% | 0.0 | 99% | KY940313.1 |
| 21Select seq KY810658.1 | Pseudomonas poae strain AAS-144 16S ribosomal RNA gene, partial sequence | 1857 | 1857 | 98% | 0.0 | 99% | KY810658.1 |
| 22Select seq KY614180.1 | Pseudomonas trivialis strain LMTK28 16S ribosomal RNA gene, partial sequence | 1857 | 1857 | 98% | 0.0 | 99% | KY614180.1 |
| 23Select seq KX588598.1 | Pseudomonas poae strain ICE349 16S ribosomal RNA gene, partial sequence | 1857 | 1857 | 98% | 0.0 | 99% | KX588598.1 |
| 24Select seq KX588597.1 | Pseudomonas poae strain ICE259 16S ribosomal RNA gene, partial sequence | 1857 | 1857 | 98% | 0.0 | 99% | KX588597.1 |
| 25Select seq KX378948.1 | Pseudomonas sp. strain 3-10 16S ribosomal RNA gene, partial sequence | 1857 | 1857 | 98% | 0.0 | 99% | KX378948.1 |
| 26Select seq KX378900.1 | Pseudomonas sp. strain 1-29 16S ribosomal RNA gene, partial sequence | 1857 | 1857 | 98% | 0.0 | 99% | KX378900.1 |
| 27Select seq KT710812.1 | Pseudomonas sp. RZ110 16S ribosomal RNA gene, partial sequence | 1857 | 1857 | 98% | 0.0 | 99% | KT710812.1 |
| 28Select seq KT825845.1 | Pseudomonas sp. 8K8 16S ribosomal RNA gene, partial sequence | 1857 | 1857 | 98% | 0.0 | 99% | KT825845.1 |
| 29Select seq KT825765.1 | Pseudomonas sp. 23K33a 16S ribosomal RNA gene, partial sequence | 1857 | 1857 | 98% | 0.0 | 99% | KT825765.1 |
| 30Select seq KT825736.1 | Pseudomonas sp. 17K8 16S ribosomal RNA gene, partial sequence | 1857 | 1857 | 98% | 0.0 | 99% | KT825736.1 |
| 31Select seq KT424973.1 | Pseudomonas sp. LB8 16S ribosomal RNA gene, partial sequence | 1857 | 1857 | 98% | 0.0 | 99% | KT424973.1 |
| 32Select seq KR922148.1 | Pseudomonas sp. PDD-59b-10 16S ribosomal RNA gene, complete sequence | 1857 | 1857 | 98% | 0.0 | 99% | KR922148.1 |
| 33Select seq KT767705.1 | Pseudomonas poae strain B42 16S ribosomal RNA gene, partial sequence | 1857 | 1857 | 98% | 0.0 | 99% | KT767705.1 |
| 34Select seq LN875096.1 | Uncultured Pseudomonas sp. partial 16S rRNA gene, clone 133X76C\_34 | 1857 | 1857 | 98% | 0.0 | 99% | LN875096.1 |
| 35Select seq KR085862.1 | Pseudomonas poae strain IHBB 11151 16S ribosomal RNA gene, partial sequence | 1857 | 1857 | 98% | 0.0 | 99% | KR085862.1 |
| 36Select seq KR085822.1 | Pseudomonas poae strain IHBB 9211 16S ribosomal RNA gene, partial sequence | 1857 | 1857 | 98% | 0.0 | 99% | KR085822.1 |
| 37Select seq KP975307.1 | Uncultured bacterium clone OTU51 16S ribosomal RNA gene, partial sequence | 1857 | 1857 | 98% | 0.0 | 99% | KP975307.1 |
| 38Select seq KT029380.1 | Uncultured bacterium clone 19-16 16S ribosomal RNA gene, partial sequence | 1857 | 1857 | 98% | 0.0 | 99% | KT029380.1 |
| 39Select seq KT029378.1 | Uncultured bacterium clone 19-14 16S ribosomal RNA gene, partial sequence | 1857 | 1857 | 98% | 0.0 | 99% | KT029378.1 |
| 40Select seq CP011507.1 | Pseudomonas trivialis strain IHBB745, complete genome | 1857 | 9279 | 98% | 0.0 | 99% | CP011507.1 |
| 41Select seq KP204130.1 | Bacterium MA\_26 16S ribosomal RNA gene, partial sequence | 1857 | 1857 | 98% | 0.0 | 99% | KP204130.1 |
| 42Select seq KP204129.1 | Pseudomonas poae strain MA\_25 16S ribosomal RNA gene, partial sequence | 1857 | 1857 | 98% | 0.0 | 99% | KP204129.1 |
| 43Select seq KF704111.1 | Pseudomonas trivialis strain KXL-Y3 16S ribosomal RNA gene, partial sequence | 1857 | 1857 | 98% | 0.0 | 99% | KF704111.1 |
| 44Select seq JQ977483.1 | Pseudomonas sp. Bma14 16S ribosomal RNA gene, partial sequence | 1857 | 1857 | 98% | 0.0 | 99% | JQ977483.1 |
| 45Select seq JQ977324.1 | Pseudomonas sp. Ms16 16S ribosomal RNA gene, partial sequence | 1857 | 1857 | 98% | 0.0 | 99% | JQ977324.1 |
| 46Select seq JQ977322.1 | Pseudomonas sp. Ms14 16S ribosomal RNA gene, partial sequence | 1857 | 1857 | 98% | 0.0 | 99% | JQ977322.1 |
| 47Select seq JX569781.1 | Pseudomonas sp. P61-2 16S ribosomal RNA gene, partial sequence | 1857 | 1857 | 98% | 0.0 | 99% | JX569781.1 |
| 48Select seq JX840381.1 | Pseudomonas salomonii strain CCNWQLS112 16S ribosomal RNA gene, partial sequence | 1857 | 1857 | 98% | 0.0 | 99% | JX840381.1 |
| 49Select seq JX515573.1 | Pseudomonas poae strain EB20 16S ribosomal RNA gene, partial sequence | 1857 | 1857 | 98% | 0.0 | 99% | JX515573.1 |
| 50Select seq JX067683.1 | Pseudomonas sp. SAP770.2 16S ribosomal RNA gene, partial sequence | 1857 | 1857 | 98% | 0.0 | 99% | JX067683.1 |
| 51Select seq JQ782898.1 | Pseudomonas poae strain zol-15 16S ribosomal RNA gene, partial sequence | 1857 | 1857 | 98% | 0.0 | 99% | JQ782898.1 |
| 52Select seq JQ691542.1 | Pseudomonas sp. FI 1007 16S ribosomal RNA gene, partial sequence | 1857 | 1857 | 98% | 0.0 | 99% | JQ691542.1 |
| 53Select seq JQ638271.1 | Pseudomonas sp. ZBC1 16S ribosomal RNA gene, partial sequence | 1857 | 1857 | 98% | 0.0 | 99% | JQ638271.1 |
| 54Select seq JQ336974.1 | Pseudomonas poae strain MML1950 16S ribosomal RNA gene, partial sequence | 1857 | 1857 | 98% | 0.0 | 99% | JQ336974.1 |
| 55Select seq JQ236630.1 | Pseudomonas poae strain ORC5 16S ribosomal RNA gene, partial sequence | 1857 | 1857 | 98% | 0.0 | 99% | JQ236630.1 |
| 56Select seq HQ224624.1 | Pseudomonas sp. SGb136 16S ribosomal RNA gene, partial sequence | 1857 | 1857 | 98% | 0.0 | 99% | HQ224624.1 |
| 57Select seq JF808098.1 | Pseudomonas sp. 5(2011) 16S ribosomal RNA gene, partial sequence | 1857 | 1857 | 98% | 0.0 | 99% | JF808098.1 |
| 58Select seq JN377670.1 | Pseudomonas trivialis strain B24 16S ribosomal RNA gene, partial sequence | 1857 | 1857 | 98% | 0.0 | 99% | JN377670.1 |
| 59Select seq JN033052.1 | Uncultured bacterium clone 3-42017 16S ribosomal RNA gene, partial sequence | 1857 | 1857 | 98% | 0.0 | 99% | JN033052.1 |
| 60Select seq JF820107.1 | Pseudomonas sp. PG-3-10 16S ribosomal RNA gene, partial sequence | 1857 | 1857 | 98% | 0.0 | 99% | JF820107.1 |
| 61Select seq HQ647258.1 | Pseudomonas poae strain P6 16S ribosomal RNA gene, partial sequence | 1857 | 1857 | 98% | 0.0 | 99% | HQ647258.1 |
| 62Select seq HQ443230.1 | Pseudomonas poae strain FBRh6 16S ribosomal RNA gene, partial sequence | 1857 | 1857 | 98% | 0.0 | 99% | HQ443230.1 |
| 63Select seq HM066261.1 | Uncultured bacterium clone EDW07B001\_111 16S ribosomal RNA gene, partial sequence | 1857 | 1857 | 98% | 0.0 | 99% | HM066261.1 |
| 64Select seq HQ256851.1 | Pseudomonas trivialis isolate PDD-32b-53 16S ribosomal RNA gene, partial sequence | 1857 | 1857 | 98% | 0.0 | 99% | HQ256851.1 |
| 65Select seq JF230879.1 | Uncultured bacterium clone ncd2648e11c1 16S ribosomal RNA gene, partial sequence | 1857 | 1857 | 98% | 0.0 | 99% | JF230879.1 |
| 66Select seq JF228956.1 | Uncultured bacterium clone ncd2619g02c1 16S ribosomal RNA gene, partial sequence | 1857 | 1857 | 98% | 0.0 | 99% | JF228956.1 |
| 67Select seq JF209277.1 | Uncultured bacterium clone ncd2405h01c1 16S ribosomal RNA gene, partial sequence | 1857 | 1857 | 98% | 0.0 | 99% | JF209277.1 |
| 68Select seq JF208644.1 | Uncultured bacterium clone ncd2396f05c1 16S ribosomal RNA gene, partial sequence | 1857 | 1857 | 98% | 0.0 | 99% | JF208644.1 |
| 69Select seq JF200837.1 | Uncultured bacterium clone ncd2408f01c1 16S ribosomal RNA gene, partial sequence | 1857 | 1857 | 98% | 0.0 | 99% | JF200837.1 |
| 70Select seq AB607148.1 | Marine bacterium B23 gene for 16S ribosomal RNA, partial sequence | 1857 | 1857 | 98% | 0.0 | 99% | AB607148.1 |
| 71Select seq GU188947.1 | Pseudomonas poae strain BCHCNZ253 16S ribosomal RNA gene, partial sequence | 1857 | 1857 | 98% | 0.0 | 99% | GU188947.1 |
| 72Select seq FJ662886.1 | Pseudomonas sp. VS05\_25 16S ribosomal RNA gene, partial sequence | 1857 | 1857 | 98% | 0.0 | 99% | FJ662886.1 |
| 73Select seq FJ937929.1 | Pseudomonas sp. LS227 16S ribosomal RNA gene, partial sequence | 1857 | 1857 | 98% | 0.0 | 99% | FJ937929.1 |
| 74Select seq FJ937922.1 | Pseudomonas poae strain LS172 16S ribosomal RNA gene, partial sequence | 1857 | 1857 | 98% | 0.0 | 99% | FJ937922.1 |
| 75Select seq FJ179366.1 | Pseudomonas trivialis strain BIHB 750 16S ribosomal RNA gene, partial sequence | 1857 | 1857 | 98% | 0.0 | 99% | FJ179366.1 |
| 76Select seq EU540146.1 | Uncultured bacterium clone nbt246b03 16S ribosomal RNA gene, partial sequence | 1857 | 1857 | 98% | 0.0 | 99% | EU540146.1 |
| 77Select seq EU360313.1 | Pseudomonas fluorescens strain 2312 16S ribosomal RNA gene and 16S-23S ribosomal RNA intergenic spacer, complete sequence; and 23S ribosomal RNA gene, partial sequence | 1857 | 1857 | 98% | 0.0 | 99% | EU360313.1 |
| 78Select seq EF028122.1 | Pseudomonas sp. Dui-7 16S ribosomal RNA gene, partial sequence | 1857 | 1857 | 98% | 0.0 | 99% | EF028122.1 |
| 79Select seq DQ885949.1 | Pseudomonas trivialis strain BIHB 749 16S ribosomal RNA gene, partial sequence | 1857 | 1857 | 98% | 0.0 | 99% | DQ885949.1 |
| 80Select seq DQ536516.1 | Pseudomonas trivialis strain BIHB 745 16S ribosomal RNA gene, partial sequence | 1857 | 1857 | 98% | 0.0 | 99% | DQ536516.1 |
| 81Select seq AY263477.1 | Pseudomonas sp. SE22#1a 16S ribosomal RNA gene, partial sequence | 1857 | 1857 | 98% | 0.0 | 99% | AY263477.1 |
| 82Select seq AY959122.1 | Uncultured bacterium clone rRNA349 16S ribosomal RNA gene, partial sequence | 1857 | 1857 | 98% | 0.0 | 99% | AY959122.1 |
| 83Select seq AY599721.1 | Pseudomonas sp. TB3-6-I 16S ribosomal RNA gene, partial sequence | 1857 | 1857 | 98% | 0.0 | 99% | AY599721.1 |
| 84Select seq AJ556801.1 | Pseudomonas sp. MWH1 partial 16S rRNA gene | 1857 | 1857 | 98% | 0.0 | 99% | AJ556801.1 |
| 85Select seq KU529202.1 | Pseudomonas sp. HD34 16S ribosomal RNA gene, partial sequence | 1855 | 1855 | 98% | 0.0 | 99% | KU529202.1 |
| 86Select seq KF020668.1 | Bacterium PGV023 16S ribosomal RNA gene, partial sequence | 1855 | 1855 | 98% | 0.0 | 99% | KF020668.1 |
| 87Select seq MG675589.1 | Pseudomonas poae strain W3AP8 16S ribosomal RNA gene, partial sequence | 1851 | 1851 | 98% | 0.0 | 99% | MG675589.1 |
| 88Select seq KY742759.1 | Pseudomonas sp. strain 8.12.1 16S ribosomal RNA gene, partial sequence | 1851 | 1851 | 98% | 0.0 | 99% | KY742759.1 |
| 89Select seq MF590113.1 | Pseudomonas trivialis strain MAF3 16S ribosomal RNA gene, partial sequence | 1851 | 1851 | 98% | 0.0 | 99% | MF590113.1 |
| 90Select seq KU529215.1 | Pseudomonas sp. LU5 16S ribosomal RNA gene, partial sequence | 1851 | 1851 | 98% | 0.0 | 99% | KU529215.1 |
| 91Select seq KU529210.1 | Pseudomonas sp. LD9 16S ribosomal RNA gene, partial sequence | 1851 | 1851 | 98% | 0.0 | 99% | KU529210.1 |
| 92Select seq KY681891.1 | Pseudomonas sp. strain 36 16S ribosomal RNA gene, partial sequence | 1851 | 1851 | 98% | 0.0 | 99% | KY681891.1 |
| 93Select seq KX509247.1 | Uncultured bacterium clone MTWL201306-19 16S ribosomal RNA gene, partial sequence | 1851 | 1851 | 98% | 0.0 | 99% | KX509247.1 |
| 94Select seq KX079774.1 | Pseudomonas sp. MYb117 16S ribosomal RNA gene, partial sequence | 1851 | 1851 | 98% | 0.0 | 99% | KX079774.1 |
| 95Select seq KX079768.1 | Pseudomonas sp. MYb114 16S ribosomal RNA gene, partial sequence | 1851 | 1851 | 98% | 0.0 | 99% | KX079768.1 |
| 96Select seq LC140795.1 | Uncultured Pseudomonas sp. gene for 16S ribosomal RNA, partial sequence, clone: 1d18 | 1851 | 1851 | 98% | 0.0 | 99% | LC140795.1 |
| 97Select seq KT825760.1 | Pseudomonas sp. 23K17 16S ribosomal RNA gene, partial sequence | 1851 | 1851 | 98% | 0.0 | 99% | KT825760.1 |
| 98Select seq HG805797.1 | Pseudomonas sp. FBF128 partial 16S rRNA gene, isolate FBF128 | 1851 | 1851 | 98% | 0.0 | 99% | HG805797.1 |
| 99Select seq HG805781.1 | Pseudomonas sp. FBF110 partial 16S rRNA gene, isolate FBF110 | 1851 | 1851 | 98% | 0.0 | 99% | HG805781.1 |
| 100Select seq JQ727438.1 | Bacterium enrichment culture clone SaA1 16S ribosomal RNA gene, partial sequence | 1851 | 1851 | 98% | 0.0 | 99% | JQ727438.1 |


## Alignments

Loading alignment... for sequences gi|1085875934,gi|1085717528,gi|445198867,gi|377550157,gi|332000891 Reading indexes 1-5

Download

FASTA (complete sequence)

FASTA (aligned sequences)

GenBank (complete sequence)

Continue
Cancel

GenBankGraphics

Sort by:

E value
 Score
Percent identity
Query start position
Subject start position

Next
Previous
Descriptions

Pseudomonas trivialis strain BS3111 genome assembly, chromosome: I

Sequence ID: LT629760.1Length: 5634667Number of Matches: 5

Related Information

Range 1: 453420 to 454427GenBankGraphics

Next Match
Previous Match
First Match

Alignment statistics for match #1

| Score | Expect | Identities | Gaps | Strand | Frame |
| --- | --- | --- | --- | --- | --- |
| 1862 bits(1008) | 0.0() | 1008/1008(100%) | 0/1008(0%) | Plus/Minus |  |

Features:

rRNA-16S ribosomal RNA . Bacterial SSU

```
Query  8       TCGAGCGGTAGAGAGAAGCTTGCTTCTCTTGAGAGCGGCGGACGGGTGAGTAATGCCTAG  67
               ||||||||||||||||||||||||||||||||||||||||||||||||||||||||||||
Sbjct  454427  TCGAGCGGTAGAGAGAAGCTTGCTTCTCTTGAGAGCGGCGGACGGGTGAGTAATGCCTAG  454368

Query  68      GAATCTGCCTGGTAGTGGGGGATAACGTTCGGAAACGGACGCTAATACCGCATACGTCCT  127
               ||||||||||||||||||||||||||||||||||||||||||||||||||||||||||||
Sbjct  454367  GAATCTGCCTGGTAGTGGGGGATAACGTTCGGAAACGGACGCTAATACCGCATACGTCCT  454308

Query  128     ACGGGAGAAAGCAGGGGACCTTCGGGCCTTGCGCTATCAGATGAGCCTAGGTCGGATTAG  187
               ||||||||||||||||||||||||||||||||||||||||||||||||||||||||||||
Sbjct  454307  ACGGGAGAAAGCAGGGGACCTTCGGGCCTTGCGCTATCAGATGAGCCTAGGTCGGATTAG  454248

Query  188     CTAGTTGGTGGGGTAATGGCTCACCAAGGCGACGATCCGTAACTGGTCTGAGAGGATGAT  247
               ||||||||||||||||||||||||||||||||||||||||||||||||||||||||||||
Sbjct  454247  CTAGTTGGTGGGGTAATGGCTCACCAAGGCGACGATCCGTAACTGGTCTGAGAGGATGAT  454188

Query  248     CAGTCACACTGGAACTGAGACACGGTCCAGACTCCTACGGGAGGCAGCAGTGGGGAATAT  307
               ||||||||||||||||||||||||||||||||||||||||||||||||||||||||||||
Sbjct  454187  CAGTCACACTGGAACTGAGACACGGTCCAGACTCCTACGGGAGGCAGCAGTGGGGAATAT  454128

Query  308     TGGACAATGGGCGAAAGCCTGATCCAGCCATGCCGCGTGTGTGAAGAAGGTCTTCGGATT  367
               ||||||||||||||||||||||||||||||||||||||||||||||||||||||||||||
Sbjct  454127  TGGACAATGGGCGAAAGCCTGATCCAGCCATGCCGCGTGTGTGAAGAAGGTCTTCGGATT  454068

Query  368     GTAAAGCACTTTAAGTTGGGAGGAAGGGCAGTTACCTAATACGTGATTGTTTTGACGTTA  427
               ||||||||||||||||||||||||||||||||||||||||||||||||||||||||||||
Sbjct  454067  GTAAAGCACTTTAAGTTGGGAGGAAGGGCAGTTACCTAATACGTGATTGTTTTGACGTTA  454008

Query  428     CCGACAGAATAAGCACCGGCTAACTCTGTGCCAGCAGCCGCGGTAATACAGAGGGTGCAA  487
               ||||||||||||||||||||||||||||||||||||||||||||||||||||||||||||
Sbjct  454007  CCGACAGAATAAGCACCGGCTAACTCTGTGCCAGCAGCCGCGGTAATACAGAGGGTGCAA  453948

Query  488     GCGTTAATCGGAATTACTGGGCGTAAAGCGCGCGTAGGTGGTTTGTTAAGTTGGATGTGA  547
               ||||||||||||||||||||||||||||||||||||||||||||||||||||||||||||
Sbjct  453947  GCGTTAATCGGAATTACTGGGCGTAAAGCGCGCGTAGGTGGTTTGTTAAGTTGGATGTGA  453888

Query  548     AATCCCCGGGCTCAACCTGGGAACTGCATTCAAAACTGACTGACTAGAGTATGGTAGAGG  607
               ||||||||||||||||||||||||||||||||||||||||||||||||||||||||||||
Sbjct  453887  AATCCCCGGGCTCAACCTGGGAACTGCATTCAAAACTGACTGACTAGAGTATGGTAGAGG  453828

Query  608     GTGGTGGAATTTCCTGTGTAGCGGTGAAATGCGTAGATATAGGAAGGAACACCAGTGGCG  667
               ||||||||||||||||||||||||||||||||||||||||||||||||||||||||||||
Sbjct  453827  GTGGTGGAATTTCCTGTGTAGCGGTGAAATGCGTAGATATAGGAAGGAACACCAGTGGCG  453768

Query  668     AAGGCGACCACCTGGACTAATACTGACACTGAGGTGCGAAAGCGTGGGGAGCAAACAGGA  727
               ||||||||||||||||||||||||||||||||||||||||||||||||||||||||||||
Sbjct  453767  AAGGCGACCACCTGGACTAATACTGACACTGAGGTGCGAAAGCGTGGGGAGCAAACAGGA  453708

Query  728     TTAGATACCCTGGTAGTCCACGCCGTAAACGATGTCAACTAGCCGTTGGAAGCCTTGAGC  787
               ||||||||||||||||||||||||||||||||||||||||||||||||||||||||||||
Sbjct  453707  TTAGATACCCTGGTAGTCCACGCCGTAAACGATGTCAACTAGCCGTTGGAAGCCTTGAGC  453648

Query  788     TTTTAGTGGCGCAGCTAACGCATTAAGTTGACCGCCTGGGGAGTACGGCCGCAAGGTTAA  847
               ||||||||||||||||||||||||||||||||||||||||||||||||||||||||||||
Sbjct  453647  TTTTAGTGGCGCAGCTAACGCATTAAGTTGACCGCCTGGGGAGTACGGCCGCAAGGTTAA  453588

Query  848     AACTCAAATGAATTGACGGGGGCCCGCACAAGCGGTGGAGCATGTGGTTTAATTCGAAGC  907
               ||||||||||||||||||||||||||||||||||||||||||||||||||||||||||||
Sbjct  453587  AACTCAAATGAATTGACGGGGGCCCGCACAAGCGGTGGAGCATGTGGTTTAATTCGAAGC  453528

Query  908     AACGCGAAGAACCTTACCAGGCCTTGACATCCAATGAACTTTCTAGAGATAGATTGGTGC  967
               ||||||||||||||||||||||||||||||||||||||||||||||||||||||||||||
Sbjct  453527  AACGCGAAGAACCTTACCAGGCCTTGACATCCAATGAACTTTCTAGAGATAGATTGGTGC  453468

Query  968     CTTCGGGAACATTGAGACAGGTGCTGCATGGCTGTCGTCAGCTCGTGT  1015
               ||||||||||||||||||||||||||||||||||||||||||||||||
Sbjct  453467  CTTCGGGAACATTGAGACAGGTGCTGCATGGCTGTCGTCAGCTCGTGT  453420
```

Range 2: 812071 to 813078GenBankGraphics

Next Match
Previous Match
First Match

Alignment statistics for match #2

| Score | Expect | Identities | Gaps | Strand | Frame |
| --- | --- | --- | --- | --- | --- |
| 1862 bits(1008) | 0.0() | 1008/1008(100%) | 0/1008(0%) | Plus/Minus |  |

Features:

rRNA-16S ribosomal RNA . Bacterial SSU

```
Query  8       TCGAGCGGTAGAGAGAAGCTTGCTTCTCTTGAGAGCGGCGGACGGGTGAGTAATGCCTAG  67
               ||||||||||||||||||||||||||||||||||||||||||||||||||||||||||||
Sbjct  813078  TCGAGCGGTAGAGAGAAGCTTGCTTCTCTTGAGAGCGGCGGACGGGTGAGTAATGCCTAG  813019

Query  68      GAATCTGCCTGGTAGTGGGGGATAACGTTCGGAAACGGACGCTAATACCGCATACGTCCT  127
               ||||||||||||||||||||||||||||||||||||||||||||||||||||||||||||
Sbjct  813018  GAATCTGCCTGGTAGTGGGGGATAACGTTCGGAAACGGACGCTAATACCGCATACGTCCT  812959

Query  128     ACGGGAGAAAGCAGGGGACCTTCGGGCCTTGCGCTATCAGATGAGCCTAGGTCGGATTAG  187
               ||||||||||||||||||||||||||||||||||||||||||||||||||||||||||||
Sbjct  812958  ACGGGAGAAAGCAGGGGACCTTCGGGCCTTGCGCTATCAGATGAGCCTAGGTCGGATTAG  812899

Query  188     CTAGTTGGTGGGGTAATGGCTCACCAAGGCGACGATCCGTAACTGGTCTGAGAGGATGAT  247
               ||||||||||||||||||||||||||||||||||||||||||||||||||||||||||||
Sbjct  812898  CTAGTTGGTGGGGTAATGGCTCACCAAGGCGACGATCCGTAACTGGTCTGAGAGGATGAT  812839

Query  248     CAGTCACACTGGAACTGAGACACGGTCCAGACTCCTACGGGAGGCAGCAGTGGGGAATAT  307
               ||||||||||||||||||||||||||||||||||||||||||||||||||||||||||||
Sbjct  812838  CAGTCACACTGGAACTGAGACACGGTCCAGACTCCTACGGGAGGCAGCAGTGGGGAATAT  812779

Query  308     TGGACAATGGGCGAAAGCCTGATCCAGCCATGCCGCGTGTGTGAAGAAGGTCTTCGGATT  367
               ||||||||||||||||||||||||||||||||||||||||||||||||||||||||||||
Sbjct  812778  TGGACAATGGGCGAAAGCCTGATCCAGCCATGCCGCGTGTGTGAAGAAGGTCTTCGGATT  812719

Query  368     GTAAAGCACTTTAAGTTGGGAGGAAGGGCAGTTACCTAATACGTGATTGTTTTGACGTTA  427
               ||||||||||||||||||||||||||||||||||||||||||||||||||||||||||||
Sbjct  812718  GTAAAGCACTTTAAGTTGGGAGGAAGGGCAGTTACCTAATACGTGATTGTTTTGACGTTA  812659

Query  428     CCGACAGAATAAGCACCGGCTAACTCTGTGCCAGCAGCCGCGGTAATACAGAGGGTGCAA  487
               ||||||||||||||||||||||||||||||||||||||||||||||||||||||||||||
Sbjct  812658  CCGACAGAATAAGCACCGGCTAACTCTGTGCCAGCAGCCGCGGTAATACAGAGGGTGCAA  812599

Query  488     GCGTTAATCGGAATTACTGGGCGTAAAGCGCGCGTAGGTGGTTTGTTAAGTTGGATGTGA  547
               ||||||||||||||||||||||||||||||||||||||||||||||||||||||||||||
Sbjct  812598  GCGTTAATCGGAATTACTGGGCGTAAAGCGCGCGTAGGTGGTTTGTTAAGTTGGATGTGA  812539

Query  548     AATCCCCGGGCTCAACCTGGGAACTGCATTCAAAACTGACTGACTAGAGTATGGTAGAGG  607
               ||||||||||||||||||||||||||||||||||||||||||||||||||||||||||||
Sbjct  812538  AATCCCCGGGCTCAACCTGGGAACTGCATTCAAAACTGACTGACTAGAGTATGGTAGAGG  812479

Query  608     GTGGTGGAATTTCCTGTGTAGCGGTGAAATGCGTAGATATAGGAAGGAACACCAGTGGCG  667
               ||||||||||||||||||||||||||||||||||||||||||||||||||||||||||||
Sbjct  812478  GTGGTGGAATTTCCTGTGTAGCGGTGAAATGCGTAGATATAGGAAGGAACACCAGTGGCG  812419

Query  668     AAGGCGACCACCTGGACTAATACTGACACTGAGGTGCGAAAGCGTGGGGAGCAAACAGGA  727
               ||||||||||||||||||||||||||||||||||||||||||||||||||||||||||||
Sbjct  812418  AAGGCGACCACCTGGACTAATACTGACACTGAGGTGCGAAAGCGTGGGGAGCAAACAGGA  812359

Query  728     TTAGATACCCTGGTAGTCCACGCCGTAAACGATGTCAACTAGCCGTTGGAAGCCTTGAGC  787
               ||||||||||||||||||||||||||||||||||||||||||||||||||||||||||||
Sbjct  812358  TTAGATACCCTGGTAGTCCACGCCGTAAACGATGTCAACTAGCCGTTGGAAGCCTTGAGC  812299

Query  788     TTTTAGTGGCGCAGCTAACGCATTAAGTTGACCGCCTGGGGAGTACGGCCGCAAGGTTAA  847
               ||||||||||||||||||||||||||||||||||||||||||||||||||||||||||||
Sbjct  812298  TTTTAGTGGCGCAGCTAACGCATTAAGTTGACCGCCTGGGGAGTACGGCCGCAAGGTTAA  812239

Query  848     AACTCAAATGAATTGACGGGGGCCCGCACAAGCGGTGGAGCATGTGGTTTAATTCGAAGC  907
               ||||||||||||||||||||||||||||||||||||||||||||||||||||||||||||
Sbjct  812238  AACTCAAATGAATTGACGGGGGCCCGCACAAGCGGTGGAGCATGTGGTTTAATTCGAAGC  812179

Query  908     AACGCGAAGAACCTTACCAGGCCTTGACATCCAATGAACTTTCTAGAGATAGATTGGTGC  967
               ||||||||||||||||||||||||||||||||||||||||||||||||||||||||||||
Sbjct  812178  AACGCGAAGAACCTTACCAGGCCTTGACATCCAATGAACTTTCTAGAGATAGATTGGTGC  812119

Query  968     CTTCGGGAACATTGAGACAGGTGCTGCATGGCTGTCGTCAGCTCGTGT  1015
               ||||||||||||||||||||||||||||||||||||||||||||||||
Sbjct  812118  CTTCGGGAACATTGAGACAGGTGCTGCATGGCTGTCGTCAGCTCGTGT  812071
```

Range 3: 1552453 to 1553460GenBankGraphics

Next Match
Previous Match
First Match

Alignment statistics for match #3

| Score | Expect | Identities | Gaps | Strand | Frame |
| --- | --- | --- | --- | --- | --- |
| 1862 bits(1008) | 0.0() | 1008/1008(100%) | 0/1008(0%) | Plus/Plus |  |

Features:

rRNA-16S ribosomal RNA . Bacterial SSU

```
Query  8        TCGAGCGGTAGAGAGAAGCTTGCTTCTCTTGAGAGCGGCGGACGGGTGAGTAATGCCTAG  67
                ||||||||||||||||||||||||||||||||||||||||||||||||||||||||||||
Sbjct  1552453  TCGAGCGGTAGAGAGAAGCTTGCTTCTCTTGAGAGCGGCGGACGGGTGAGTAATGCCTAG  1552512

Query  68       GAATCTGCCTGGTAGTGGGGGATAACGTTCGGAAACGGACGCTAATACCGCATACGTCCT  127
                ||||||||||||||||||||||||||||||||||||||||||||||||||||||||||||
Sbjct  1552513  GAATCTGCCTGGTAGTGGGGGATAACGTTCGGAAACGGACGCTAATACCGCATACGTCCT  1552572

Query  128      ACGGGAGAAAGCAGGGGACCTTCGGGCCTTGCGCTATCAGATGAGCCTAGGTCGGATTAG  187
                ||||||||||||||||||||||||||||||||||||||||||||||||||||||||||||
Sbjct  1552573  ACGGGAGAAAGCAGGGGACCTTCGGGCCTTGCGCTATCAGATGAGCCTAGGTCGGATTAG  1552632

Query  188      CTAGTTGGTGGGGTAATGGCTCACCAAGGCGACGATCCGTAACTGGTCTGAGAGGATGAT  247
                ||||||||||||||||||||||||||||||||||||||||||||||||||||||||||||
Sbjct  1552633  CTAGTTGGTGGGGTAATGGCTCACCAAGGCGACGATCCGTAACTGGTCTGAGAGGATGAT  1552692

Query  248      CAGTCACACTGGAACTGAGACACGGTCCAGACTCCTACGGGAGGCAGCAGTGGGGAATAT  307
                ||||||||||||||||||||||||||||||||||||||||||||||||||||||||||||
Sbjct  1552693  CAGTCACACTGGAACTGAGACACGGTCCAGACTCCTACGGGAGGCAGCAGTGGGGAATAT  1552752

Query  308      TGGACAATGGGCGAAAGCCTGATCCAGCCATGCCGCGTGTGTGAAGAAGGTCTTCGGATT  367
                ||||||||||||||||||||||||||||||||||||||||||||||||||||||||||||
Sbjct  1552753  TGGACAATGGGCGAAAGCCTGATCCAGCCATGCCGCGTGTGTGAAGAAGGTCTTCGGATT  1552812

Query  368      GTAAAGCACTTTAAGTTGGGAGGAAGGGCAGTTACCTAATACGTGATTGTTTTGACGTTA  427
                ||||||||||||||||||||||||||||||||||||||||||||||||||||||||||||
Sbjct  1552813  GTAAAGCACTTTAAGTTGGGAGGAAGGGCAGTTACCTAATACGTGATTGTTTTGACGTTA  1552872

Query  428      CCGACAGAATAAGCACCGGCTAACTCTGTGCCAGCAGCCGCGGTAATACAGAGGGTGCAA  487
                ||||||||||||||||||||||||||||||||||||||||||||||||||||||||||||
Sbjct  1552873  CCGACAGAATAAGCACCGGCTAACTCTGTGCCAGCAGCCGCGGTAATACAGAGGGTGCAA  1552932

Query  488      GCGTTAATCGGAATTACTGGGCGTAAAGCGCGCGTAGGTGGTTTGTTAAGTTGGATGTGA  547
                ||||||||||||||||||||||||||||||||||||||||||||||||||||||||||||
Sbjct  1552933  GCGTTAATCGGAATTACTGGGCGTAAAGCGCGCGTAGGTGGTTTGTTAAGTTGGATGTGA  1552992

Query  548      AATCCCCGGGCTCAACCTGGGAACTGCATTCAAAACTGACTGACTAGAGTATGGTAGAGG  607
                ||||||||||||||||||||||||||||||||||||||||||||||||||||||||||||
Sbjct  1552993  AATCCCCGGGCTCAACCTGGGAACTGCATTCAAAACTGACTGACTAGAGTATGGTAGAGG  1553052

Query  608      GTGGTGGAATTTCCTGTGTAGCGGTGAAATGCGTAGATATAGGAAGGAACACCAGTGGCG  667
                ||||||||||||||||||||||||||||||||||||||||||||||||||||||||||||
Sbjct  1553053  GTGGTGGAATTTCCTGTGTAGCGGTGAAATGCGTAGATATAGGAAGGAACACCAGTGGCG  1553112

Query  668      AAGGCGACCACCTGGACTAATACTGACACTGAGGTGCGAAAGCGTGGGGAGCAAACAGGA  727
                ||||||||||||||||||||||||||||||||||||||||||||||||||||||||||||
Sbjct  1553113  AAGGCGACCACCTGGACTAATACTGACACTGAGGTGCGAAAGCGTGGGGAGCAAACAGGA  1553172

Query  728      TTAGATACCCTGGTAGTCCACGCCGTAAACGATGTCAACTAGCCGTTGGAAGCCTTGAGC  787
                ||||||||||||||||||||||||||||||||||||||||||||||||||||||||||||
Sbjct  1553173  TTAGATACCCTGGTAGTCCACGCCGTAAACGATGTCAACTAGCCGTTGGAAGCCTTGAGC  1553232

Query  788      TTTTAGTGGCGCAGCTAACGCATTAAGTTGACCGCCTGGGGAGTACGGCCGCAAGGTTAA  847
                ||||||||||||||||||||||||||||||||||||||||||||||||||||||||||||
Sbjct  1553233  TTTTAGTGGCGCAGCTAACGCATTAAGTTGACCGCCTGGGGAGTACGGCCGCAAGGTTAA  1553292

Query  848      AACTCAAATGAATTGACGGGGGCCCGCACAAGCGGTGGAGCATGTGGTTTAATTCGAAGC  907
                ||||||||||||||||||||||||||||||||||||||||||||||||||||||||||||
Sbjct  1553293  AACTCAAATGAATTGACGGGGGCCCGCACAAGCGGTGGAGCATGTGGTTTAATTCGAAGC  1553352

Query  908      AACGCGAAGAACCTTACCAGGCCTTGACATCCAATGAACTTTCTAGAGATAGATTGGTGC  967
                ||||||||||||||||||||||||||||||||||||||||||||||||||||||||||||
Sbjct  1553353  AACGCGAAGAACCTTACCAGGCCTTGACATCCAATGAACTTTCTAGAGATAGATTGGTGC  1553412

Query  968      CTTCGGGAACATTGAGACAGGTGCTGCATGGCTGTCGTCAGCTCGTGT  1015
                ||||||||||||||||||||||||||||||||||||||||||||||||
Sbjct  1553413  CTTCGGGAACATTGAGACAGGTGCTGCATGGCTGTCGTCAGCTCGTGT  1553460
```

Range 4: 3431048 to 3432055GenBankGraphics

Next Match
Previous Match
First Match

Alignment statistics for match #4

| Score | Expect | Identities | Gaps | Strand | Frame |
| --- | --- | --- | --- | --- | --- |
| 1862 bits(1008) | 0.0() | 1008/1008(100%) | 0/1008(0%) | Plus/Plus |  |

Features:

rRNA-16S ribosomal RNA . Bacterial SSU

```
Query  8        TCGAGCGGTAGAGAGAAGCTTGCTTCTCTTGAGAGCGGCGGACGGGTGAGTAATGCCTAG  67
                ||||||||||||||||||||||||||||||||||||||||||||||||||||||||||||
Sbjct  3431048  TCGAGCGGTAGAGAGAAGCTTGCTTCTCTTGAGAGCGGCGGACGGGTGAGTAATGCCTAG  3431107

Query  68       GAATCTGCCTGGTAGTGGGGGATAACGTTCGGAAACGGACGCTAATACCGCATACGTCCT  127
                ||||||||||||||||||||||||||||||||||||||||||||||||||||||||||||
Sbjct  3431108  GAATCTGCCTGGTAGTGGGGGATAACGTTCGGAAACGGACGCTAATACCGCATACGTCCT  3431167

Query  128      ACGGGAGAAAGCAGGGGACCTTCGGGCCTTGCGCTATCAGATGAGCCTAGGTCGGATTAG  187
                ||||||||||||||||||||||||||||||||||||||||||||||||||||||||||||
Sbjct  3431168  ACGGGAGAAAGCAGGGGACCTTCGGGCCTTGCGCTATCAGATGAGCCTAGGTCGGATTAG  3431227

Query  188      CTAGTTGGTGGGGTAATGGCTCACCAAGGCGACGATCCGTAACTGGTCTGAGAGGATGAT  247
                ||||||||||||||||||||||||||||||||||||||||||||||||||||||||||||
Sbjct  3431228  CTAGTTGGTGGGGTAATGGCTCACCAAGGCGACGATCCGTAACTGGTCTGAGAGGATGAT  3431287

Query  248      CAGTCACACTGGAACTGAGACACGGTCCAGACTCCTACGGGAGGCAGCAGTGGGGAATAT  307
                ||||||||||||||||||||||||||||||||||||||||||||||||||||||||||||
Sbjct  3431288  CAGTCACACTGGAACTGAGACACGGTCCAGACTCCTACGGGAGGCAGCAGTGGGGAATAT  3431347

Query  308      TGGACAATGGGCGAAAGCCTGATCCAGCCATGCCGCGTGTGTGAAGAAGGTCTTCGGATT  367
                ||||||||||||||||||||||||||||||||||||||||||||||||||||||||||||
Sbjct  3431348  TGGACAATGGGCGAAAGCCTGATCCAGCCATGCCGCGTGTGTGAAGAAGGTCTTCGGATT  3431407

Query  368      GTAAAGCACTTTAAGTTGGGAGGAAGGGCAGTTACCTAATACGTGATTGTTTTGACGTTA  427
                ||||||||||||||||||||||||||||||||||||||||||||||||||||||||||||
Sbjct  3431408  GTAAAGCACTTTAAGTTGGGAGGAAGGGCAGTTACCTAATACGTGATTGTTTTGACGTTA  3431467

Query  428      CCGACAGAATAAGCACCGGCTAACTCTGTGCCAGCAGCCGCGGTAATACAGAGGGTGCAA  487
                ||||||||||||||||||||||||||||||||||||||||||||||||||||||||||||
Sbjct  3431468  CCGACAGAATAAGCACCGGCTAACTCTGTGCCAGCAGCCGCGGTAATACAGAGGGTGCAA  3431527

Query  488      GCGTTAATCGGAATTACTGGGCGTAAAGCGCGCGTAGGTGGTTTGTTAAGTTGGATGTGA  547
                ||||||||||||||||||||||||||||||||||||||||||||||||||||||||||||
Sbjct  3431528  GCGTTAATCGGAATTACTGGGCGTAAAGCGCGCGTAGGTGGTTTGTTAAGTTGGATGTGA  3431587

Query  548      AATCCCCGGGCTCAACCTGGGAACTGCATTCAAAACTGACTGACTAGAGTATGGTAGAGG  607
                ||||||||||||||||||||||||||||||||||||||||||||||||||||||||||||
Sbjct  3431588  AATCCCCGGGCTCAACCTGGGAACTGCATTCAAAACTGACTGACTAGAGTATGGTAGAGG  3431647

Query  608      GTGGTGGAATTTCCTGTGTAGCGGTGAAATGCGTAGATATAGGAAGGAACACCAGTGGCG  667
                ||||||||||||||||||||||||||||||||||||||||||||||||||||||||||||
Sbjct  3431648  GTGGTGGAATTTCCTGTGTAGCGGTGAAATGCGTAGATATAGGAAGGAACACCAGTGGCG  3431707

Query  668      AAGGCGACCACCTGGACTAATACTGACACTGAGGTGCGAAAGCGTGGGGAGCAAACAGGA  727
                ||||||||||||||||||||||||||||||||||||||||||||||||||||||||||||
Sbjct  3431708  AAGGCGACCACCTGGACTAATACTGACACTGAGGTGCGAAAGCGTGGGGAGCAAACAGGA  3431767

Query  728      TTAGATACCCTGGTAGTCCACGCCGTAAACGATGTCAACTAGCCGTTGGAAGCCTTGAGC  787
                ||||||||||||||||||||||||||||||||||||||||||||||||||||||||||||
Sbjct  3431768  TTAGATACCCTGGTAGTCCACGCCGTAAACGATGTCAACTAGCCGTTGGAAGCCTTGAGC  3431827

Query  788      TTTTAGTGGCGCAGCTAACGCATTAAGTTGACCGCCTGGGGAGTACGGCCGCAAGGTTAA  847
                ||||||||||||||||||||||||||||||||||||||||||||||||||||||||||||
Sbjct  3431828  TTTTAGTGGCGCAGCTAACGCATTAAGTTGACCGCCTGGGGAGTACGGCCGCAAGGTTAA  3431887

Query  848      AACTCAAATGAATTGACGGGGGCCCGCACAAGCGGTGGAGCATGTGGTTTAATTCGAAGC  907
                ||||||||||||||||||||||||||||||||||||||||||||||||||||||||||||
Sbjct  3431888  AACTCAAATGAATTGACGGGGGCCCGCACAAGCGGTGGAGCATGTGGTTTAATTCGAAGC  3431947

Query  908      AACGCGAAGAACCTTACCAGGCCTTGACATCCAATGAACTTTCTAGAGATAGATTGGTGC  967
                ||||||||||||||||||||||||||||||||||||||||||||||||||||||||||||
Sbjct  3431948  AACGCGAAGAACCTTACCAGGCCTTGACATCCAATGAACTTTCTAGAGATAGATTGGTGC  3432007

Query  968      CTTCGGGAACATTGAGACAGGTGCTGCATGGCTGTCGTCAGCTCGTGT  1015
                ||||||||||||||||||||||||||||||||||||||||||||||||
Sbjct  3432008  CTTCGGGAACATTGAGACAGGTGCTGCATGGCTGTCGTCAGCTCGTGT  3432055
```

Range 5: 2158687 to 2159694GenBankGraphics

Next Match
Previous Match
First Match

Alignment statistics for match #5

| Score | Expect | Identities | Gaps | Strand | Frame |
| --- | --- | --- | --- | --- | --- |
| 1857 bits(1005) | 0.0() | 1007/1008(99%) | 0/1008(0%) | Plus/Plus |  |

Features:

rRNA-16S ribosomal RNA . Bacterial SSU

```
Query  8        TCGAGCGGTAGAGAGAAGCTTGCTTCTCTTGAGAGCGGCGGACGGGTGAGTAATGCCTAG  67
                ||||||||||||||||||||||||||||||||||||||||||||||||||||||||||||
Sbjct  2158687  TCGAGCGGTAGAGAGAAGCTTGCTTCTCTTGAGAGCGGCGGACGGGTGAGTAATGCCTAG  2158746

Query  68       GAATCTGCCTGGTAGTGGGGGATAACGTTCGGAAACGGACGCTAATACCGCATACGTCCT  127
                ||||||||||||||||||||||||||||||||||||||||||||||||||||||||||||
Sbjct  2158747  GAATCTGCCTGGTAGTGGGGGATAACGTTCGGAAACGGACGCTAATACCGCATACGTCCT  2158806

Query  128      ACGGGAGAAAGCAGGGGACCTTCGGGCCTTGCGCTATCAGATGAGCCTAGGTCGGATTAG  187
                ||||||||||||||||||||||||||||||||||||||||||||||||||||||||||||
Sbjct  2158807  ACGGGAGAAAGCAGGGGACCTTCGGGCCTTGCGCTATCAGATGAGCCTAGGTCGGATTAG  2158866

Query  188      CTAGTTGGTGGGGTAATGGCTCACCAAGGCGACGATCCGTAACTGGTCTGAGAGGATGAT  247
                ||||||||||||||||||||||||||||||||||||||||||||||||||||||||||||
Sbjct  2158867  CTAGTTGGTGGGGTAATGGCTCACCAAGGCGACGATCCGTAACTGGTCTGAGAGGATGAT  2158926

Query  248      CAGTCACACTGGAACTGAGACACGGTCCAGACTCCTACGGGAGGCAGCAGTGGGGAATAT  307
                ||||||||||||||||||||||||||||||||||||||||||||||||||||||||||||
Sbjct  2158927  CAGTCACACTGGAACTGAGACACGGTCCAGACTCCTACGGGAGGCAGCAGTGGGGAATAT  2158986

Query  308      TGGACAATGGGCGAAAGCCTGATCCAGCCATGCCGCGTGTGTGAAGAAGGTCTTCGGATT  367
                ||||||||||||||||||||||||||||||||||||||||||||||||||||||||||||
Sbjct  2158987  TGGACAATGGGCGAAAGCCTGATCCAGCCATGCCGCGTGTGTGAAGAAGGTCTTCGGATT  2159046

Query  368      GTAAAGCACTTTAAGTTGGGAGGAAGGGCAGTTACCTAATACGTGATTGTTTTGACGTTA  427
                ||||||||||||||||||||||||||||||||||| ||||||||||||||||||||||||
Sbjct  2159047  GTAAAGCACTTTAAGTTGGGAGGAAGGGCAGTTACTTAATACGTGATTGTTTTGACGTTA  2159106

Query  428      CCGACAGAATAAGCACCGGCTAACTCTGTGCCAGCAGCCGCGGTAATACAGAGGGTGCAA  487
                ||||||||||||||||||||||||||||||||||||||||||||||||||||||||||||
Sbjct  2159107  CCGACAGAATAAGCACCGGCTAACTCTGTGCCAGCAGCCGCGGTAATACAGAGGGTGCAA  2159166

Query  488      GCGTTAATCGGAATTACTGGGCGTAAAGCGCGCGTAGGTGGTTTGTTAAGTTGGATGTGA  547
                ||||||||||||||||||||||||||||||||||||||||||||||||||||||||||||
Sbjct  2159167  GCGTTAATCGGAATTACTGGGCGTAAAGCGCGCGTAGGTGGTTTGTTAAGTTGGATGTGA  2159226

Query  548      AATCCCCGGGCTCAACCTGGGAACTGCATTCAAAACTGACTGACTAGAGTATGGTAGAGG  607
                ||||||||||||||||||||||||||||||||||||||||||||||||||||||||||||
Sbjct  2159227  AATCCCCGGGCTCAACCTGGGAACTGCATTCAAAACTGACTGACTAGAGTATGGTAGAGG  2159286

Query  608      GTGGTGGAATTTCCTGTGTAGCGGTGAAATGCGTAGATATAGGAAGGAACACCAGTGGCG  667
                ||||||||||||||||||||||||||||||||||||||||||||||||||||||||||||
Sbjct  2159287  GTGGTGGAATTTCCTGTGTAGCGGTGAAATGCGTAGATATAGGAAGGAACACCAGTGGCG  2159346

Query  668      AAGGCGACCACCTGGACTAATACTGACACTGAGGTGCGAAAGCGTGGGGAGCAAACAGGA  727
                ||||||||||||||||||||||||||||||||||||||||||||||||||||||||||||
Sbjct  2159347  AAGGCGACCACCTGGACTAATACTGACACTGAGGTGCGAAAGCGTGGGGAGCAAACAGGA  2159406

Query  728      TTAGATACCCTGGTAGTCCACGCCGTAAACGATGTCAACTAGCCGTTGGAAGCCTTGAGC  787
                ||||||||||||||||||||||||||||||||||||||||||||||||||||||||||||
Sbjct  2159407  TTAGATACCCTGGTAGTCCACGCCGTAAACGATGTCAACTAGCCGTTGGAAGCCTTGAGC  2159466

Query  788      TTTTAGTGGCGCAGCTAACGCATTAAGTTGACCGCCTGGGGAGTACGGCCGCAAGGTTAA  847
                ||||||||||||||||||||||||||||||||||||||||||||||||||||||||||||
Sbjct  2159467  TTTTAGTGGCGCAGCTAACGCATTAAGTTGACCGCCTGGGGAGTACGGCCGCAAGGTTAA  2159526

Query  848      AACTCAAATGAATTGACGGGGGCCCGCACAAGCGGTGGAGCATGTGGTTTAATTCGAAGC  907
                ||||||||||||||||||||||||||||||||||||||||||||||||||||||||||||
Sbjct  2159527  AACTCAAATGAATTGACGGGGGCCCGCACAAGCGGTGGAGCATGTGGTTTAATTCGAAGC  2159586

Query  908      AACGCGAAGAACCTTACCAGGCCTTGACATCCAATGAACTTTCTAGAGATAGATTGGTGC  967
                ||||||||||||||||||||||||||||||||||||||||||||||||||||||||||||
Sbjct  2159587  AACGCGAAGAACCTTACCAGGCCTTGACATCCAATGAACTTTCTAGAGATAGATTGGTGC  2159646

Query  968      CTTCGGGAACATTGAGACAGGTGCTGCATGGCTGTCGTCAGCTCGTGT  1015
                ||||||||||||||||||||||||||||||||||||||||||||||||
Sbjct  2159647  CTTCGGGAACATTGAGACAGGTGCTGCATGGCTGTCGTCAGCTCGTGT  2159694
```

Download

FASTA (complete sequence)

FASTA (aligned sequences)

GenBank (complete sequence)

Continue
Cancel

GenBankGraphics

Sort by:

E value
 Score
Percent identity
Query start position
Subject start position

Next
Previous
Descriptions

Pseudomonas poae strain BS2776 genome assembly, chromosome: I

Sequence ID: LT629706.1Length: 5950238Number of Matches: 5

Related Information

Range 1: 1521569 to 1522576GenBankGraphics

Next Match
Previous Match
First Match

Alignment statistics for match #1

| Score | Expect | Identities | Gaps | Strand | Frame |
| --- | --- | --- | --- | --- | --- |
| 1862 bits(1008) | 0.0() | 1008/1008(100%) | 0/1008(0%) | Plus/Minus |  |

Features:

rRNA-16S ribosomal RNA . Bacterial SSU

```
Query  8        TCGAGCGGTAGAGAGAAGCTTGCTTCTCTTGAGAGCGGCGGACGGGTGAGTAATGCCTAG  67
                ||||||||||||||||||||||||||||||||||||||||||||||||||||||||||||
Sbjct  1522576  TCGAGCGGTAGAGAGAAGCTTGCTTCTCTTGAGAGCGGCGGACGGGTGAGTAATGCCTAG  1522517

Query  68       GAATCTGCCTGGTAGTGGGGGATAACGTTCGGAAACGGACGCTAATACCGCATACGTCCT  127
                ||||||||||||||||||||||||||||||||||||||||||||||||||||||||||||
Sbjct  1522516  GAATCTGCCTGGTAGTGGGGGATAACGTTCGGAAACGGACGCTAATACCGCATACGTCCT  1522457

Query  128      ACGGGAGAAAGCAGGGGACCTTCGGGCCTTGCGCTATCAGATGAGCCTAGGTCGGATTAG  187
                ||||||||||||||||||||||||||||||||||||||||||||||||||||||||||||
Sbjct  1522456  ACGGGAGAAAGCAGGGGACCTTCGGGCCTTGCGCTATCAGATGAGCCTAGGTCGGATTAG  1522397

Query  188      CTAGTTGGTGGGGTAATGGCTCACCAAGGCGACGATCCGTAACTGGTCTGAGAGGATGAT  247
                ||||||||||||||||||||||||||||||||||||||||||||||||||||||||||||
Sbjct  1522396  CTAGTTGGTGGGGTAATGGCTCACCAAGGCGACGATCCGTAACTGGTCTGAGAGGATGAT  1522337

Query  248      CAGTCACACTGGAACTGAGACACGGTCCAGACTCCTACGGGAGGCAGCAGTGGGGAATAT  307
                ||||||||||||||||||||||||||||||||||||||||||||||||||||||||||||
Sbjct  1522336  CAGTCACACTGGAACTGAGACACGGTCCAGACTCCTACGGGAGGCAGCAGTGGGGAATAT  1522277

Query  308      TGGACAATGGGCGAAAGCCTGATCCAGCCATGCCGCGTGTGTGAAGAAGGTCTTCGGATT  367
                ||||||||||||||||||||||||||||||||||||||||||||||||||||||||||||
Sbjct  1522276  TGGACAATGGGCGAAAGCCTGATCCAGCCATGCCGCGTGTGTGAAGAAGGTCTTCGGATT  1522217

Query  368      GTAAAGCACTTTAAGTTGGGAGGAAGGGCAGTTACCTAATACGTGATTGTTTTGACGTTA  427
                ||||||||||||||||||||||||||||||||||||||||||||||||||||||||||||
Sbjct  1522216  GTAAAGCACTTTAAGTTGGGAGGAAGGGCAGTTACCTAATACGTGATTGTTTTGACGTTA  1522157

Query  428      CCGACAGAATAAGCACCGGCTAACTCTGTGCCAGCAGCCGCGGTAATACAGAGGGTGCAA  487
                ||||||||||||||||||||||||||||||||||||||||||||||||||||||||||||
Sbjct  1522156  CCGACAGAATAAGCACCGGCTAACTCTGTGCCAGCAGCCGCGGTAATACAGAGGGTGCAA  1522097

Query  488      GCGTTAATCGGAATTACTGGGCGTAAAGCGCGCGTAGGTGGTTTGTTAAGTTGGATGTGA  547
                ||||||||||||||||||||||||||||||||||||||||||||||||||||||||||||
Sbjct  1522096  GCGTTAATCGGAATTACTGGGCGTAAAGCGCGCGTAGGTGGTTTGTTAAGTTGGATGTGA  1522037

Query  548      AATCCCCGGGCTCAACCTGGGAACTGCATTCAAAACTGACTGACTAGAGTATGGTAGAGG  607
                ||||||||||||||||||||||||||||||||||||||||||||||||||||||||||||
Sbjct  1522036  AATCCCCGGGCTCAACCTGGGAACTGCATTCAAAACTGACTGACTAGAGTATGGTAGAGG  1521977

Query  608      GTGGTGGAATTTCCTGTGTAGCGGTGAAATGCGTAGATATAGGAAGGAACACCAGTGGCG  667
                ||||||||||||||||||||||||||||||||||||||||||||||||||||||||||||
Sbjct  1521976  GTGGTGGAATTTCCTGTGTAGCGGTGAAATGCGTAGATATAGGAAGGAACACCAGTGGCG  1521917

Query  668      AAGGCGACCACCTGGACTAATACTGACACTGAGGTGCGAAAGCGTGGGGAGCAAACAGGA  727
                ||||||||||||||||||||||||||||||||||||||||||||||||||||||||||||
Sbjct  1521916  AAGGCGACCACCTGGACTAATACTGACACTGAGGTGCGAAAGCGTGGGGAGCAAACAGGA  1521857

Query  728      TTAGATACCCTGGTAGTCCACGCCGTAAACGATGTCAACTAGCCGTTGGAAGCCTTGAGC  787
                ||||||||||||||||||||||||||||||||||||||||||||||||||||||||||||
Sbjct  1521856  TTAGATACCCTGGTAGTCCACGCCGTAAACGATGTCAACTAGCCGTTGGAAGCCTTGAGC  1521797

Query  788      TTTTAGTGGCGCAGCTAACGCATTAAGTTGACCGCCTGGGGAGTACGGCCGCAAGGTTAA  847
                ||||||||||||||||||||||||||||||||||||||||||||||||||||||||||||
Sbjct  1521796  TTTTAGTGGCGCAGCTAACGCATTAAGTTGACCGCCTGGGGAGTACGGCCGCAAGGTTAA  1521737

Query  848      AACTCAAATGAATTGACGGGGGCCCGCACAAGCGGTGGAGCATGTGGTTTAATTCGAAGC  907
                ||||||||||||||||||||||||||||||||||||||||||||||||||||||||||||
Sbjct  1521736  AACTCAAATGAATTGACGGGGGCCCGCACAAGCGGTGGAGCATGTGGTTTAATTCGAAGC  1521677

Query  908      AACGCGAAGAACCTTACCAGGCCTTGACATCCAATGAACTTTCTAGAGATAGATTGGTGC  967
                ||||||||||||||||||||||||||||||||||||||||||||||||||||||||||||
Sbjct  1521676  AACGCGAAGAACCTTACCAGGCCTTGACATCCAATGAACTTTCTAGAGATAGATTGGTGC  1521617

Query  968      CTTCGGGAACATTGAGACAGGTGCTGCATGGCTGTCGTCAGCTCGTGT  1015
                ||||||||||||||||||||||||||||||||||||||||||||||||
Sbjct  1521616  CTTCGGGAACATTGAGACAGGTGCTGCATGGCTGTCGTCAGCTCGTGT  1521569
```

Range 2: 2816453 to 2817460GenBankGraphics

Next Match
Previous Match
First Match

Alignment statistics for match #2

| Score | Expect | Identities | Gaps | Strand | Frame |
| --- | --- | --- | --- | --- | --- |
| 1862 bits(1008) | 0.0() | 1008/1008(100%) | 0/1008(0%) | Plus/Minus |  |

Features:

rRNA-16S ribosomal RNA . Bacterial SSU

```
Query  8        TCGAGCGGTAGAGAGAAGCTTGCTTCTCTTGAGAGCGGCGGACGGGTGAGTAATGCCTAG  67
                ||||||||||||||||||||||||||||||||||||||||||||||||||||||||||||
Sbjct  2817460  TCGAGCGGTAGAGAGAAGCTTGCTTCTCTTGAGAGCGGCGGACGGGTGAGTAATGCCTAG  2817401

Query  68       GAATCTGCCTGGTAGTGGGGGATAACGTTCGGAAACGGACGCTAATACCGCATACGTCCT  127
                ||||||||||||||||||||||||||||||||||||||||||||||||||||||||||||
Sbjct  2817400  GAATCTGCCTGGTAGTGGGGGATAACGTTCGGAAACGGACGCTAATACCGCATACGTCCT  2817341

Query  128      ACGGGAGAAAGCAGGGGACCTTCGGGCCTTGCGCTATCAGATGAGCCTAGGTCGGATTAG  187
                ||||||||||||||||||||||||||||||||||||||||||||||||||||||||||||
Sbjct  2817340  ACGGGAGAAAGCAGGGGACCTTCGGGCCTTGCGCTATCAGATGAGCCTAGGTCGGATTAG  2817281

Query  188      CTAGTTGGTGGGGTAATGGCTCACCAAGGCGACGATCCGTAACTGGTCTGAGAGGATGAT  247
                ||||||||||||||||||||||||||||||||||||||||||||||||||||||||||||
Sbjct  2817280  CTAGTTGGTGGGGTAATGGCTCACCAAGGCGACGATCCGTAACTGGTCTGAGAGGATGAT  2817221

Query  248      CAGTCACACTGGAACTGAGACACGGTCCAGACTCCTACGGGAGGCAGCAGTGGGGAATAT  307
                ||||||||||||||||||||||||||||||||||||||||||||||||||||||||||||
Sbjct  2817220  CAGTCACACTGGAACTGAGACACGGTCCAGACTCCTACGGGAGGCAGCAGTGGGGAATAT  2817161

Query  308      TGGACAATGGGCGAAAGCCTGATCCAGCCATGCCGCGTGTGTGAAGAAGGTCTTCGGATT  367
                ||||||||||||||||||||||||||||||||||||||||||||||||||||||||||||
Sbjct  2817160  TGGACAATGGGCGAAAGCCTGATCCAGCCATGCCGCGTGTGTGAAGAAGGTCTTCGGATT  2817101

Query  368      GTAAAGCACTTTAAGTTGGGAGGAAGGGCAGTTACCTAATACGTGATTGTTTTGACGTTA  427
                ||||||||||||||||||||||||||||||||||||||||||||||||||||||||||||
Sbjct  2817100  GTAAAGCACTTTAAGTTGGGAGGAAGGGCAGTTACCTAATACGTGATTGTTTTGACGTTA  2817041

Query  428      CCGACAGAATAAGCACCGGCTAACTCTGTGCCAGCAGCCGCGGTAATACAGAGGGTGCAA  487
                ||||||||||||||||||||||||||||||||||||||||||||||||||||||||||||
Sbjct  2817040  CCGACAGAATAAGCACCGGCTAACTCTGTGCCAGCAGCCGCGGTAATACAGAGGGTGCAA  2816981

Query  488      GCGTTAATCGGAATTACTGGGCGTAAAGCGCGCGTAGGTGGTTTGTTAAGTTGGATGTGA  547
                ||||||||||||||||||||||||||||||||||||||||||||||||||||||||||||
Sbjct  2816980  GCGTTAATCGGAATTACTGGGCGTAAAGCGCGCGTAGGTGGTTTGTTAAGTTGGATGTGA  2816921

Query  548      AATCCCCGGGCTCAACCTGGGAACTGCATTCAAAACTGACTGACTAGAGTATGGTAGAGG  607
                ||||||||||||||||||||||||||||||||||||||||||||||||||||||||||||
Sbjct  2816920  AATCCCCGGGCTCAACCTGGGAACTGCATTCAAAACTGACTGACTAGAGTATGGTAGAGG  2816861

Query  608      GTGGTGGAATTTCCTGTGTAGCGGTGAAATGCGTAGATATAGGAAGGAACACCAGTGGCG  667
                ||||||||||||||||||||||||||||||||||||||||||||||||||||||||||||
Sbjct  2816860  GTGGTGGAATTTCCTGTGTAGCGGTGAAATGCGTAGATATAGGAAGGAACACCAGTGGCG  2816801

Query  668      AAGGCGACCACCTGGACTAATACTGACACTGAGGTGCGAAAGCGTGGGGAGCAAACAGGA  727
                ||||||||||||||||||||||||||||||||||||||||||||||||||||||||||||
Sbjct  2816800  AAGGCGACCACCTGGACTAATACTGACACTGAGGTGCGAAAGCGTGGGGAGCAAACAGGA  2816741

Query  728      TTAGATACCCTGGTAGTCCACGCCGTAAACGATGTCAACTAGCCGTTGGAAGCCTTGAGC  787
                ||||||||||||||||||||||||||||||||||||||||||||||||||||||||||||
Sbjct  2816740  TTAGATACCCTGGTAGTCCACGCCGTAAACGATGTCAACTAGCCGTTGGAAGCCTTGAGC  2816681

Query  788      TTTTAGTGGCGCAGCTAACGCATTAAGTTGACCGCCTGGGGAGTACGGCCGCAAGGTTAA  847
                ||||||||||||||||||||||||||||||||||||||||||||||||||||||||||||
Sbjct  2816680  TTTTAGTGGCGCAGCTAACGCATTAAGTTGACCGCCTGGGGAGTACGGCCGCAAGGTTAA  2816621

Query  848      AACTCAAATGAATTGACGGGGGCCCGCACAAGCGGTGGAGCATGTGGTTTAATTCGAAGC  907
                ||||||||||||||||||||||||||||||||||||||||||||||||||||||||||||
Sbjct  2816620  AACTCAAATGAATTGACGGGGGCCCGCACAAGCGGTGGAGCATGTGGTTTAATTCGAAGC  2816561

Query  908      AACGCGAAGAACCTTACCAGGCCTTGACATCCAATGAACTTTCTAGAGATAGATTGGTGC  967
                ||||||||||||||||||||||||||||||||||||||||||||||||||||||||||||
Sbjct  2816560  AACGCGAAGAACCTTACCAGGCCTTGACATCCAATGAACTTTCTAGAGATAGATTGGTGC  2816501

Query  968      CTTCGGGAACATTGAGACAGGTGCTGCATGGCTGTCGTCAGCTCGTGT  1015
                ||||||||||||||||||||||||||||||||||||||||||||||||
Sbjct  2816500  CTTCGGGAACATTGAGACAGGTGCTGCATGGCTGTCGTCAGCTCGTGT  2816453
```

Range 3: 3457529 to 3458536GenBankGraphics

Next Match
Previous Match
First Match

Alignment statistics for match #3

| Score | Expect | Identities | Gaps | Strand | Frame |
| --- | --- | --- | --- | --- | --- |
| 1862 bits(1008) | 0.0() | 1008/1008(100%) | 0/1008(0%) | Plus/Minus |  |

Features:

rRNA-16S ribosomal RNA . Bacterial SSU

```
Query  8        TCGAGCGGTAGAGAGAAGCTTGCTTCTCTTGAGAGCGGCGGACGGGTGAGTAATGCCTAG  67
                ||||||||||||||||||||||||||||||||||||||||||||||||||||||||||||
Sbjct  3458536  TCGAGCGGTAGAGAGAAGCTTGCTTCTCTTGAGAGCGGCGGACGGGTGAGTAATGCCTAG  3458477

Query  68       GAATCTGCCTGGTAGTGGGGGATAACGTTCGGAAACGGACGCTAATACCGCATACGTCCT  127
                ||||||||||||||||||||||||||||||||||||||||||||||||||||||||||||
Sbjct  3458476  GAATCTGCCTGGTAGTGGGGGATAACGTTCGGAAACGGACGCTAATACCGCATACGTCCT  3458417

Query  128      ACGGGAGAAAGCAGGGGACCTTCGGGCCTTGCGCTATCAGATGAGCCTAGGTCGGATTAG  187
                ||||||||||||||||||||||||||||||||||||||||||||||||||||||||||||
Sbjct  3458416  ACGGGAGAAAGCAGGGGACCTTCGGGCCTTGCGCTATCAGATGAGCCTAGGTCGGATTAG  3458357

Query  188      CTAGTTGGTGGGGTAATGGCTCACCAAGGCGACGATCCGTAACTGGTCTGAGAGGATGAT  247
                ||||||||||||||||||||||||||||||||||||||||||||||||||||||||||||
Sbjct  3458356  CTAGTTGGTGGGGTAATGGCTCACCAAGGCGACGATCCGTAACTGGTCTGAGAGGATGAT  3458297

Query  248      CAGTCACACTGGAACTGAGACACGGTCCAGACTCCTACGGGAGGCAGCAGTGGGGAATAT  307
                ||||||||||||||||||||||||||||||||||||||||||||||||||||||||||||
Sbjct  3458296  CAGTCACACTGGAACTGAGACACGGTCCAGACTCCTACGGGAGGCAGCAGTGGGGAATAT  3458237

Query  308      TGGACAATGGGCGAAAGCCTGATCCAGCCATGCCGCGTGTGTGAAGAAGGTCTTCGGATT  367
                ||||||||||||||||||||||||||||||||||||||||||||||||||||||||||||
Sbjct  3458236  TGGACAATGGGCGAAAGCCTGATCCAGCCATGCCGCGTGTGTGAAGAAGGTCTTCGGATT  3458177

Query  368      GTAAAGCACTTTAAGTTGGGAGGAAGGGCAGTTACCTAATACGTGATTGTTTTGACGTTA  427
                ||||||||||||||||||||||||||||||||||||||||||||||||||||||||||||
Sbjct  3458176  GTAAAGCACTTTAAGTTGGGAGGAAGGGCAGTTACCTAATACGTGATTGTTTTGACGTTA  3458117

Query  428      CCGACAGAATAAGCACCGGCTAACTCTGTGCCAGCAGCCGCGGTAATACAGAGGGTGCAA  487
                ||||||||||||||||||||||||||||||||||||||||||||||||||||||||||||
Sbjct  3458116  CCGACAGAATAAGCACCGGCTAACTCTGTGCCAGCAGCCGCGGTAATACAGAGGGTGCAA  3458057

Query  488      GCGTTAATCGGAATTACTGGGCGTAAAGCGCGCGTAGGTGGTTTGTTAAGTTGGATGTGA  547
                ||||||||||||||||||||||||||||||||||||||||||||||||||||||||||||
Sbjct  3458056  GCGTTAATCGGAATTACTGGGCGTAAAGCGCGCGTAGGTGGTTTGTTAAGTTGGATGTGA  3457997

Query  548      AATCCCCGGGCTCAACCTGGGAACTGCATTCAAAACTGACTGACTAGAGTATGGTAGAGG  607
                ||||||||||||||||||||||||||||||||||||||||||||||||||||||||||||
Sbjct  3457996  AATCCCCGGGCTCAACCTGGGAACTGCATTCAAAACTGACTGACTAGAGTATGGTAGAGG  3457937

Query  608      GTGGTGGAATTTCCTGTGTAGCGGTGAAATGCGTAGATATAGGAAGGAACACCAGTGGCG  667
                ||||||||||||||||||||||||||||||||||||||||||||||||||||||||||||
Sbjct  3457936  GTGGTGGAATTTCCTGTGTAGCGGTGAAATGCGTAGATATAGGAAGGAACACCAGTGGCG  3457877

Query  668      AAGGCGACCACCTGGACTAATACTGACACTGAGGTGCGAAAGCGTGGGGAGCAAACAGGA  727
                ||||||||||||||||||||||||||||||||||||||||||||||||||||||||||||
Sbjct  3457876  AAGGCGACCACCTGGACTAATACTGACACTGAGGTGCGAAAGCGTGGGGAGCAAACAGGA  3457817

Query  728      TTAGATACCCTGGTAGTCCACGCCGTAAACGATGTCAACTAGCCGTTGGAAGCCTTGAGC  787
                ||||||||||||||||||||||||||||||||||||||||||||||||||||||||||||
Sbjct  3457816  TTAGATACCCTGGTAGTCCACGCCGTAAACGATGTCAACTAGCCGTTGGAAGCCTTGAGC  3457757

Query  788      TTTTAGTGGCGCAGCTAACGCATTAAGTTGACCGCCTGGGGAGTACGGCCGCAAGGTTAA  847
                ||||||||||||||||||||||||||||||||||||||||||||||||||||||||||||
Sbjct  3457756  TTTTAGTGGCGCAGCTAACGCATTAAGTTGACCGCCTGGGGAGTACGGCCGCAAGGTTAA  3457697

Query  848      AACTCAAATGAATTGACGGGGGCCCGCACAAGCGGTGGAGCATGTGGTTTAATTCGAAGC  907
                ||||||||||||||||||||||||||||||||||||||||||||||||||||||||||||
Sbjct  3457696  AACTCAAATGAATTGACGGGGGCCCGCACAAGCGGTGGAGCATGTGGTTTAATTCGAAGC  3457637

Query  908      AACGCGAAGAACCTTACCAGGCCTTGACATCCAATGAACTTTCTAGAGATAGATTGGTGC  967
                ||||||||||||||||||||||||||||||||||||||||||||||||||||||||||||
Sbjct  3457636  AACGCGAAGAACCTTACCAGGCCTTGACATCCAATGAACTTTCTAGAGATAGATTGGTGC  3457577

Query  968      CTTCGGGAACATTGAGACAGGTGCTGCATGGCTGTCGTCAGCTCGTGT  1015
                ||||||||||||||||||||||||||||||||||||||||||||||||
Sbjct  3457576  CTTCGGGAACATTGAGACAGGTGCTGCATGGCTGTCGTCAGCTCGTGT  3457529
```

Range 4: 4255118 to 4256125GenBankGraphics

Next Match
Previous Match
First Match

Alignment statistics for match #4

| Score | Expect | Identities | Gaps | Strand | Frame |
| --- | --- | --- | --- | --- | --- |
| 1862 bits(1008) | 0.0() | 1008/1008(100%) | 0/1008(0%) | Plus/Plus |  |

Features:

rRNA-16S ribosomal RNA . Bacterial SSU

```
Query  8        TCGAGCGGTAGAGAGAAGCTTGCTTCTCTTGAGAGCGGCGGACGGGTGAGTAATGCCTAG  67
                ||||||||||||||||||||||||||||||||||||||||||||||||||||||||||||
Sbjct  4255118  TCGAGCGGTAGAGAGAAGCTTGCTTCTCTTGAGAGCGGCGGACGGGTGAGTAATGCCTAG  4255177

Query  68       GAATCTGCCTGGTAGTGGGGGATAACGTTCGGAAACGGACGCTAATACCGCATACGTCCT  127
                ||||||||||||||||||||||||||||||||||||||||||||||||||||||||||||
Sbjct  4255178  GAATCTGCCTGGTAGTGGGGGATAACGTTCGGAAACGGACGCTAATACCGCATACGTCCT  4255237

Query  128      ACGGGAGAAAGCAGGGGACCTTCGGGCCTTGCGCTATCAGATGAGCCTAGGTCGGATTAG  187
                ||||||||||||||||||||||||||||||||||||||||||||||||||||||||||||
Sbjct  4255238  ACGGGAGAAAGCAGGGGACCTTCGGGCCTTGCGCTATCAGATGAGCCTAGGTCGGATTAG  4255297

Query  188      CTAGTTGGTGGGGTAATGGCTCACCAAGGCGACGATCCGTAACTGGTCTGAGAGGATGAT  247
                ||||||||||||||||||||||||||||||||||||||||||||||||||||||||||||
Sbjct  4255298  CTAGTTGGTGGGGTAATGGCTCACCAAGGCGACGATCCGTAACTGGTCTGAGAGGATGAT  4255357

Query  248      CAGTCACACTGGAACTGAGACACGGTCCAGACTCCTACGGGAGGCAGCAGTGGGGAATAT  307
                ||||||||||||||||||||||||||||||||||||||||||||||||||||||||||||
Sbjct  4255358  CAGTCACACTGGAACTGAGACACGGTCCAGACTCCTACGGGAGGCAGCAGTGGGGAATAT  4255417

Query  308      TGGACAATGGGCGAAAGCCTGATCCAGCCATGCCGCGTGTGTGAAGAAGGTCTTCGGATT  367
                ||||||||||||||||||||||||||||||||||||||||||||||||||||||||||||
Sbjct  4255418  TGGACAATGGGCGAAAGCCTGATCCAGCCATGCCGCGTGTGTGAAGAAGGTCTTCGGATT  4255477

Query  368      GTAAAGCACTTTAAGTTGGGAGGAAGGGCAGTTACCTAATACGTGATTGTTTTGACGTTA  427
                ||||||||||||||||||||||||||||||||||||||||||||||||||||||||||||
Sbjct  4255478  GTAAAGCACTTTAAGTTGGGAGGAAGGGCAGTTACCTAATACGTGATTGTTTTGACGTTA  4255537

Query  428      CCGACAGAATAAGCACCGGCTAACTCTGTGCCAGCAGCCGCGGTAATACAGAGGGTGCAA  487
                ||||||||||||||||||||||||||||||||||||||||||||||||||||||||||||
Sbjct  4255538  CCGACAGAATAAGCACCGGCTAACTCTGTGCCAGCAGCCGCGGTAATACAGAGGGTGCAA  4255597

Query  488      GCGTTAATCGGAATTACTGGGCGTAAAGCGCGCGTAGGTGGTTTGTTAAGTTGGATGTGA  547
                ||||||||||||||||||||||||||||||||||||||||||||||||||||||||||||
Sbjct  4255598  GCGTTAATCGGAATTACTGGGCGTAAAGCGCGCGTAGGTGGTTTGTTAAGTTGGATGTGA  4255657

Query  548      AATCCCCGGGCTCAACCTGGGAACTGCATTCAAAACTGACTGACTAGAGTATGGTAGAGG  607
                ||||||||||||||||||||||||||||||||||||||||||||||||||||||||||||
Sbjct  4255658  AATCCCCGGGCTCAACCTGGGAACTGCATTCAAAACTGACTGACTAGAGTATGGTAGAGG  4255717

Query  608      GTGGTGGAATTTCCTGTGTAGCGGTGAAATGCGTAGATATAGGAAGGAACACCAGTGGCG  667
                ||||||||||||||||||||||||||||||||||||||||||||||||||||||||||||
Sbjct  4255718  GTGGTGGAATTTCCTGTGTAGCGGTGAAATGCGTAGATATAGGAAGGAACACCAGTGGCG  4255777

Query  668      AAGGCGACCACCTGGACTAATACTGACACTGAGGTGCGAAAGCGTGGGGAGCAAACAGGA  727
                ||||||||||||||||||||||||||||||||||||||||||||||||||||||||||||
Sbjct  4255778  AAGGCGACCACCTGGACTAATACTGACACTGAGGTGCGAAAGCGTGGGGAGCAAACAGGA  4255837

Query  728      TTAGATACCCTGGTAGTCCACGCCGTAAACGATGTCAACTAGCCGTTGGAAGCCTTGAGC  787
                ||||||||||||||||||||||||||||||||||||||||||||||||||||||||||||
Sbjct  4255838  TTAGATACCCTGGTAGTCCACGCCGTAAACGATGTCAACTAGCCGTTGGAAGCCTTGAGC  4255897

Query  788      TTTTAGTGGCGCAGCTAACGCATTAAGTTGACCGCCTGGGGAGTACGGCCGCAAGGTTAA  847
                ||||||||||||||||||||||||||||||||||||||||||||||||||||||||||||
Sbjct  4255898  TTTTAGTGGCGCAGCTAACGCATTAAGTTGACCGCCTGGGGAGTACGGCCGCAAGGTTAA  4255957

Query  848      AACTCAAATGAATTGACGGGGGCCCGCACAAGCGGTGGAGCATGTGGTTTAATTCGAAGC  907
                ||||||||||||||||||||||||||||||||||||||||||||||||||||||||||||
Sbjct  4255958  AACTCAAATGAATTGACGGGGGCCCGCACAAGCGGTGGAGCATGTGGTTTAATTCGAAGC  4256017

Query  908      AACGCGAAGAACCTTACCAGGCCTTGACATCCAATGAACTTTCTAGAGATAGATTGGTGC  967
                ||||||||||||||||||||||||||||||||||||||||||||||||||||||||||||
Sbjct  4256018  AACGCGAAGAACCTTACCAGGCCTTGACATCCAATGAACTTTCTAGAGATAGATTGGTGC  4256077

Query  968      CTTCGGGAACATTGAGACAGGTGCTGCATGGCTGTCGTCAGCTCGTGT  1015
                ||||||||||||||||||||||||||||||||||||||||||||||||
Sbjct  4256078  CTTCGGGAACATTGAGACAGGTGCTGCATGGCTGTCGTCAGCTCGTGT  4256125
```

Range 5: 4574167 to 4575174GenBankGraphics

Next Match
Previous Match
First Match

Alignment statistics for match #5

| Score | Expect | Identities | Gaps | Strand | Frame |
| --- | --- | --- | --- | --- | --- |
| 1862 bits(1008) | 0.0() | 1008/1008(100%) | 0/1008(0%) | Plus/Plus |  |

Features:

rRNA-16S ribosomal RNA . Bacterial SSU

```
Query  8        TCGAGCGGTAGAGAGAAGCTTGCTTCTCTTGAGAGCGGCGGACGGGTGAGTAATGCCTAG  67
                ||||||||||||||||||||||||||||||||||||||||||||||||||||||||||||
Sbjct  4574167  TCGAGCGGTAGAGAGAAGCTTGCTTCTCTTGAGAGCGGCGGACGGGTGAGTAATGCCTAG  4574226

Query  68       GAATCTGCCTGGTAGTGGGGGATAACGTTCGGAAACGGACGCTAATACCGCATACGTCCT  127
                ||||||||||||||||||||||||||||||||||||||||||||||||||||||||||||
Sbjct  4574227  GAATCTGCCTGGTAGTGGGGGATAACGTTCGGAAACGGACGCTAATACCGCATACGTCCT  4574286

Query  128      ACGGGAGAAAGCAGGGGACCTTCGGGCCTTGCGCTATCAGATGAGCCTAGGTCGGATTAG  187
                ||||||||||||||||||||||||||||||||||||||||||||||||||||||||||||
Sbjct  4574287  ACGGGAGAAAGCAGGGGACCTTCGGGCCTTGCGCTATCAGATGAGCCTAGGTCGGATTAG  4574346

Query  188      CTAGTTGGTGGGGTAATGGCTCACCAAGGCGACGATCCGTAACTGGTCTGAGAGGATGAT  247
                ||||||||||||||||||||||||||||||||||||||||||||||||||||||||||||
Sbjct  4574347  CTAGTTGGTGGGGTAATGGCTCACCAAGGCGACGATCCGTAACTGGTCTGAGAGGATGAT  4574406

Query  248      CAGTCACACTGGAACTGAGACACGGTCCAGACTCCTACGGGAGGCAGCAGTGGGGAATAT  307
                ||||||||||||||||||||||||||||||||||||||||||||||||||||||||||||
Sbjct  4574407  CAGTCACACTGGAACTGAGACACGGTCCAGACTCCTACGGGAGGCAGCAGTGGGGAATAT  4574466

Query  308      TGGACAATGGGCGAAAGCCTGATCCAGCCATGCCGCGTGTGTGAAGAAGGTCTTCGGATT  367
                ||||||||||||||||||||||||||||||||||||||||||||||||||||||||||||
Sbjct  4574467  TGGACAATGGGCGAAAGCCTGATCCAGCCATGCCGCGTGTGTGAAGAAGGTCTTCGGATT  4574526

Query  368      GTAAAGCACTTTAAGTTGGGAGGAAGGGCAGTTACCTAATACGTGATTGTTTTGACGTTA  427
                ||||||||||||||||||||||||||||||||||||||||||||||||||||||||||||
Sbjct  4574527  GTAAAGCACTTTAAGTTGGGAGGAAGGGCAGTTACCTAATACGTGATTGTTTTGACGTTA  4574586

Query  428      CCGACAGAATAAGCACCGGCTAACTCTGTGCCAGCAGCCGCGGTAATACAGAGGGTGCAA  487
                ||||||||||||||||||||||||||||||||||||||||||||||||||||||||||||
Sbjct  4574587  CCGACAGAATAAGCACCGGCTAACTCTGTGCCAGCAGCCGCGGTAATACAGAGGGTGCAA  4574646

Query  488      GCGTTAATCGGAATTACTGGGCGTAAAGCGCGCGTAGGTGGTTTGTTAAGTTGGATGTGA  547
                ||||||||||||||||||||||||||||||||||||||||||||||||||||||||||||
Sbjct  4574647  GCGTTAATCGGAATTACTGGGCGTAAAGCGCGCGTAGGTGGTTTGTTAAGTTGGATGTGA  4574706

Query  548      AATCCCCGGGCTCAACCTGGGAACTGCATTCAAAACTGACTGACTAGAGTATGGTAGAGG  607
                ||||||||||||||||||||||||||||||||||||||||||||||||||||||||||||
Sbjct  4574707  AATCCCCGGGCTCAACCTGGGAACTGCATTCAAAACTGACTGACTAGAGTATGGTAGAGG  4574766

Query  608      GTGGTGGAATTTCCTGTGTAGCGGTGAAATGCGTAGATATAGGAAGGAACACCAGTGGCG  667
                ||||||||||||||||||||||||||||||||||||||||||||||||||||||||||||
Sbjct  4574767  GTGGTGGAATTTCCTGTGTAGCGGTGAAATGCGTAGATATAGGAAGGAACACCAGTGGCG  4574826

Query  668      AAGGCGACCACCTGGACTAATACTGACACTGAGGTGCGAAAGCGTGGGGAGCAAACAGGA  727
                ||||||||||||||||||||||||||||||||||||||||||||||||||||||||||||
Sbjct  4574827  AAGGCGACCACCTGGACTAATACTGACACTGAGGTGCGAAAGCGTGGGGAGCAAACAGGA  4574886

Query  728      TTAGATACCCTGGTAGTCCACGCCGTAAACGATGTCAACTAGCCGTTGGAAGCCTTGAGC  787
                ||||||||||||||||||||||||||||||||||||||||||||||||||||||||||||
Sbjct  4574887  TTAGATACCCTGGTAGTCCACGCCGTAAACGATGTCAACTAGCCGTTGGAAGCCTTGAGC  4574946

Query  788      TTTTAGTGGCGCAGCTAACGCATTAAGTTGACCGCCTGGGGAGTACGGCCGCAAGGTTAA  847
                ||||||||||||||||||||||||||||||||||||||||||||||||||||||||||||
Sbjct  4574947  TTTTAGTGGCGCAGCTAACGCATTAAGTTGACCGCCTGGGGAGTACGGCCGCAAGGTTAA  4575006

Query  848      AACTCAAATGAATTGACGGGGGCCCGCACAAGCGGTGGAGCATGTGGTTTAATTCGAAGC  907
                ||||||||||||||||||||||||||||||||||||||||||||||||||||||||||||
Sbjct  4575007  AACTCAAATGAATTGACGGGGGCCCGCACAAGCGGTGGAGCATGTGGTTTAATTCGAAGC  4575066

Query  908      AACGCGAAGAACCTTACCAGGCCTTGACATCCAATGAACTTTCTAGAGATAGATTGGTGC  967
                ||||||||||||||||||||||||||||||||||||||||||||||||||||||||||||
Sbjct  4575067  AACGCGAAGAACCTTACCAGGCCTTGACATCCAATGAACTTTCTAGAGATAGATTGGTGC  4575126

Query  968      CTTCGGGAACATTGAGACAGGTGCTGCATGGCTGTCGTCAGCTCGTGT  1015
                ||||||||||||||||||||||||||||||||||||||||||||||||
Sbjct  4575127  CTTCGGGAACATTGAGACAGGTGCTGCATGGCTGTCGTCAGCTCGTGT  4575174
```

Download

FASTA (complete sequence)

FASTA (aligned sequences)

GenBank (complete sequence)

Continue
Cancel

GenBankGraphics

Sort by:

E value
 Score
Percent identity
Query start position
Subject start position

Next
Previous
Descriptions

Pseudomonas poae RE\*1-1-14, complete genome

Sequence ID: CP004045.1Length: 5512241Number of Matches: 5

Related Information

Range 1: 1279346 to 1280353GenBankGraphics

Next Match
Previous Match
First Match

Alignment statistics for match #1

| Score | Expect | Identities | Gaps | Strand | Frame |
| --- | --- | --- | --- | --- | --- |
| 1862 bits(1008) | 0.0() | 1008/1008(100%) | 0/1008(0%) | Plus/Plus |  |

Features:

```
Query  8        TCGAGCGGTAGAGAGAAGCTTGCTTCTCTTGAGAGCGGCGGACGGGTGAGTAATGCCTAG  67
                ||||||||||||||||||||||||||||||||||||||||||||||||||||||||||||
Sbjct  1279346  TCGAGCGGTAGAGAGAAGCTTGCTTCTCTTGAGAGCGGCGGACGGGTGAGTAATGCCTAG  1279405

Query  68       GAATCTGCCTGGTAGTGGGGGATAACGTTCGGAAACGGACGCTAATACCGCATACGTCCT  127
                ||||||||||||||||||||||||||||||||||||||||||||||||||||||||||||
Sbjct  1279406  GAATCTGCCTGGTAGTGGGGGATAACGTTCGGAAACGGACGCTAATACCGCATACGTCCT  1279465

Query  128      ACGGGAGAAAGCAGGGGACCTTCGGGCCTTGCGCTATCAGATGAGCCTAGGTCGGATTAG  187
                ||||||||||||||||||||||||||||||||||||||||||||||||||||||||||||
Sbjct  1279466  ACGGGAGAAAGCAGGGGACCTTCGGGCCTTGCGCTATCAGATGAGCCTAGGTCGGATTAG  1279525

Query  188      CTAGTTGGTGGGGTAATGGCTCACCAAGGCGACGATCCGTAACTGGTCTGAGAGGATGAT  247
                ||||||||||||||||||||||||||||||||||||||||||||||||||||||||||||
Sbjct  1279526  CTAGTTGGTGGGGTAATGGCTCACCAAGGCGACGATCCGTAACTGGTCTGAGAGGATGAT  1279585

Query  248      CAGTCACACTGGAACTGAGACACGGTCCAGACTCCTACGGGAGGCAGCAGTGGGGAATAT  307
                ||||||||||||||||||||||||||||||||||||||||||||||||||||||||||||
Sbjct  1279586  CAGTCACACTGGAACTGAGACACGGTCCAGACTCCTACGGGAGGCAGCAGTGGGGAATAT  1279645

Query  308      TGGACAATGGGCGAAAGCCTGATCCAGCCATGCCGCGTGTGTGAAGAAGGTCTTCGGATT  367
                ||||||||||||||||||||||||||||||||||||||||||||||||||||||||||||
Sbjct  1279646  TGGACAATGGGCGAAAGCCTGATCCAGCCATGCCGCGTGTGTGAAGAAGGTCTTCGGATT  1279705

Query  368      GTAAAGCACTTTAAGTTGGGAGGAAGGGCAGTTACCTAATACGTGATTGTTTTGACGTTA  427
                ||||||||||||||||||||||||||||||||||||||||||||||||||||||||||||
Sbjct  1279706  GTAAAGCACTTTAAGTTGGGAGGAAGGGCAGTTACCTAATACGTGATTGTTTTGACGTTA  1279765

Query  428      CCGACAGAATAAGCACCGGCTAACTCTGTGCCAGCAGCCGCGGTAATACAGAGGGTGCAA  487
                ||||||||||||||||||||||||||||||||||||||||||||||||||||||||||||
Sbjct  1279766  CCGACAGAATAAGCACCGGCTAACTCTGTGCCAGCAGCCGCGGTAATACAGAGGGTGCAA  1279825

Query  488      GCGTTAATCGGAATTACTGGGCGTAAAGCGCGCGTAGGTGGTTTGTTAAGTTGGATGTGA  547
                ||||||||||||||||||||||||||||||||||||||||||||||||||||||||||||
Sbjct  1279826  GCGTTAATCGGAATTACTGGGCGTAAAGCGCGCGTAGGTGGTTTGTTAAGTTGGATGTGA  1279885

Query  548      AATCCCCGGGCTCAACCTGGGAACTGCATTCAAAACTGACTGACTAGAGTATGGTAGAGG  607
                ||||||||||||||||||||||||||||||||||||||||||||||||||||||||||||
Sbjct  1279886  AATCCCCGGGCTCAACCTGGGAACTGCATTCAAAACTGACTGACTAGAGTATGGTAGAGG  1279945

Query  608      GTGGTGGAATTTCCTGTGTAGCGGTGAAATGCGTAGATATAGGAAGGAACACCAGTGGCG  667
                ||||||||||||||||||||||||||||||||||||||||||||||||||||||||||||
Sbjct  1279946  GTGGTGGAATTTCCTGTGTAGCGGTGAAATGCGTAGATATAGGAAGGAACACCAGTGGCG  1280005

Query  668      AAGGCGACCACCTGGACTAATACTGACACTGAGGTGCGAAAGCGTGGGGAGCAAACAGGA  727
                ||||||||||||||||||||||||||||||||||||||||||||||||||||||||||||
Sbjct  1280006  AAGGCGACCACCTGGACTAATACTGACACTGAGGTGCGAAAGCGTGGGGAGCAAACAGGA  1280065

Query  728      TTAGATACCCTGGTAGTCCACGCCGTAAACGATGTCAACTAGCCGTTGGAAGCCTTGAGC  787
                ||||||||||||||||||||||||||||||||||||||||||||||||||||||||||||
Sbjct  1280066  TTAGATACCCTGGTAGTCCACGCCGTAAACGATGTCAACTAGCCGTTGGAAGCCTTGAGC  1280125

Query  788      TTTTAGTGGCGCAGCTAACGCATTAAGTTGACCGCCTGGGGAGTACGGCCGCAAGGTTAA  847
                ||||||||||||||||||||||||||||||||||||||||||||||||||||||||||||
Sbjct  1280126  TTTTAGTGGCGCAGCTAACGCATTAAGTTGACCGCCTGGGGAGTACGGCCGCAAGGTTAA  1280185

Query  848      AACTCAAATGAATTGACGGGGGCCCGCACAAGCGGTGGAGCATGTGGTTTAATTCGAAGC  907
                ||||||||||||||||||||||||||||||||||||||||||||||||||||||||||||
Sbjct  1280186  AACTCAAATGAATTGACGGGGGCCCGCACAAGCGGTGGAGCATGTGGTTTAATTCGAAGC  1280245

Query  908      AACGCGAAGAACCTTACCAGGCCTTGACATCCAATGAACTTTCTAGAGATAGATTGGTGC  967
                ||||||||||||||||||||||||||||||||||||||||||||||||||||||||||||
Sbjct  1280246  AACGCGAAGAACCTTACCAGGCCTTGACATCCAATGAACTTTCTAGAGATAGATTGGTGC  1280305

Query  968      CTTCGGGAACATTGAGACAGGTGCTGCATGGCTGTCGTCAGCTCGTGT  1015
                ||||||||||||||||||||||||||||||||||||||||||||||||
Sbjct  1280306  CTTCGGGAACATTGAGACAGGTGCTGCATGGCTGTCGTCAGCTCGTGT  1280353
```

Range 2: 3808774 to 3809781GenBankGraphics

Next Match
Previous Match
First Match

Alignment statistics for match #2

| Score | Expect | Identities | Gaps | Strand | Frame |
| --- | --- | --- | --- | --- | --- |
| 1862 bits(1008) | 0.0() | 1008/1008(100%) | 0/1008(0%) | Plus/Minus |  |

Features:

```
Query  8        TCGAGCGGTAGAGAGAAGCTTGCTTCTCTTGAGAGCGGCGGACGGGTGAGTAATGCCTAG  67
                ||||||||||||||||||||||||||||||||||||||||||||||||||||||||||||
Sbjct  3809781  TCGAGCGGTAGAGAGAAGCTTGCTTCTCTTGAGAGCGGCGGACGGGTGAGTAATGCCTAG  3809722

Query  68       GAATCTGCCTGGTAGTGGGGGATAACGTTCGGAAACGGACGCTAATACCGCATACGTCCT  127
                ||||||||||||||||||||||||||||||||||||||||||||||||||||||||||||
Sbjct  3809721  GAATCTGCCTGGTAGTGGGGGATAACGTTCGGAAACGGACGCTAATACCGCATACGTCCT  3809662

Query  128      ACGGGAGAAAGCAGGGGACCTTCGGGCCTTGCGCTATCAGATGAGCCTAGGTCGGATTAG  187
                ||||||||||||||||||||||||||||||||||||||||||||||||||||||||||||
Sbjct  3809661  ACGGGAGAAAGCAGGGGACCTTCGGGCCTTGCGCTATCAGATGAGCCTAGGTCGGATTAG  3809602

Query  188      CTAGTTGGTGGGGTAATGGCTCACCAAGGCGACGATCCGTAACTGGTCTGAGAGGATGAT  247
                ||||||||||||||||||||||||||||||||||||||||||||||||||||||||||||
Sbjct  3809601  CTAGTTGGTGGGGTAATGGCTCACCAAGGCGACGATCCGTAACTGGTCTGAGAGGATGAT  3809542

Query  248      CAGTCACACTGGAACTGAGACACGGTCCAGACTCCTACGGGAGGCAGCAGTGGGGAATAT  307
                ||||||||||||||||||||||||||||||||||||||||||||||||||||||||||||
Sbjct  3809541  CAGTCACACTGGAACTGAGACACGGTCCAGACTCCTACGGGAGGCAGCAGTGGGGAATAT  3809482

Query  308      TGGACAATGGGCGAAAGCCTGATCCAGCCATGCCGCGTGTGTGAAGAAGGTCTTCGGATT  367
                ||||||||||||||||||||||||||||||||||||||||||||||||||||||||||||
Sbjct  3809481  TGGACAATGGGCGAAAGCCTGATCCAGCCATGCCGCGTGTGTGAAGAAGGTCTTCGGATT  3809422

Query  368      GTAAAGCACTTTAAGTTGGGAGGAAGGGCAGTTACCTAATACGTGATTGTTTTGACGTTA  427
                ||||||||||||||||||||||||||||||||||||||||||||||||||||||||||||
Sbjct  3809421  GTAAAGCACTTTAAGTTGGGAGGAAGGGCAGTTACCTAATACGTGATTGTTTTGACGTTA  3809362

Query  428      CCGACAGAATAAGCACCGGCTAACTCTGTGCCAGCAGCCGCGGTAATACAGAGGGTGCAA  487
                ||||||||||||||||||||||||||||||||||||||||||||||||||||||||||||
Sbjct  3809361  CCGACAGAATAAGCACCGGCTAACTCTGTGCCAGCAGCCGCGGTAATACAGAGGGTGCAA  3809302

Query  488      GCGTTAATCGGAATTACTGGGCGTAAAGCGCGCGTAGGTGGTTTGTTAAGTTGGATGTGA  547
                ||||||||||||||||||||||||||||||||||||||||||||||||||||||||||||
Sbjct  3809301  GCGTTAATCGGAATTACTGGGCGTAAAGCGCGCGTAGGTGGTTTGTTAAGTTGGATGTGA  3809242

Query  548      AATCCCCGGGCTCAACCTGGGAACTGCATTCAAAACTGACTGACTAGAGTATGGTAGAGG  607
                ||||||||||||||||||||||||||||||||||||||||||||||||||||||||||||
Sbjct  3809241  AATCCCCGGGCTCAACCTGGGAACTGCATTCAAAACTGACTGACTAGAGTATGGTAGAGG  3809182

Query  608      GTGGTGGAATTTCCTGTGTAGCGGTGAAATGCGTAGATATAGGAAGGAACACCAGTGGCG  667
                ||||||||||||||||||||||||||||||||||||||||||||||||||||||||||||
Sbjct  3809181  GTGGTGGAATTTCCTGTGTAGCGGTGAAATGCGTAGATATAGGAAGGAACACCAGTGGCG  3809122

Query  668      AAGGCGACCACCTGGACTAATACTGACACTGAGGTGCGAAAGCGTGGGGAGCAAACAGGA  727
                ||||||||||||||||||||||||||||||||||||||||||||||||||||||||||||
Sbjct  3809121  AAGGCGACCACCTGGACTAATACTGACACTGAGGTGCGAAAGCGTGGGGAGCAAACAGGA  3809062

Query  728      TTAGATACCCTGGTAGTCCACGCCGTAAACGATGTCAACTAGCCGTTGGAAGCCTTGAGC  787
                ||||||||||||||||||||||||||||||||||||||||||||||||||||||||||||
Sbjct  3809061  TTAGATACCCTGGTAGTCCACGCCGTAAACGATGTCAACTAGCCGTTGGAAGCCTTGAGC  3809002

Query  788      TTTTAGTGGCGCAGCTAACGCATTAAGTTGACCGCCTGGGGAGTACGGCCGCAAGGTTAA  847
                ||||||||||||||||||||||||||||||||||||||||||||||||||||||||||||
Sbjct  3809001  TTTTAGTGGCGCAGCTAACGCATTAAGTTGACCGCCTGGGGAGTACGGCCGCAAGGTTAA  3808942

Query  848      AACTCAAATGAATTGACGGGGGCCCGCACAAGCGGTGGAGCATGTGGTTTAATTCGAAGC  907
                ||||||||||||||||||||||||||||||||||||||||||||||||||||||||||||
Sbjct  3808941  AACTCAAATGAATTGACGGGGGCCCGCACAAGCGGTGGAGCATGTGGTTTAATTCGAAGC  3808882

Query  908      AACGCGAAGAACCTTACCAGGCCTTGACATCCAATGAACTTTCTAGAGATAGATTGGTGC  967
                ||||||||||||||||||||||||||||||||||||||||||||||||||||||||||||
Sbjct  3808881  AACGCGAAGAACCTTACCAGGCCTTGACATCCAATGAACTTTCTAGAGATAGATTGGTGC  3808822

Query  968      CTTCGGGAACATTGAGACAGGTGCTGCATGGCTGTCGTCAGCTCGTGT  1015
                ||||||||||||||||||||||||||||||||||||||||||||||||
Sbjct  3808821  CTTCGGGAACATTGAGACAGGTGCTGCATGGCTGTCGTCAGCTCGTGT  3808774
```

Range 3: 4137182 to 4138189GenBankGraphics

Next Match
Previous Match
First Match

Alignment statistics for match #3

| Score | Expect | Identities | Gaps | Strand | Frame |
| --- | --- | --- | --- | --- | --- |
| 1862 bits(1008) | 0.0() | 1008/1008(100%) | 0/1008(0%) | Plus/Minus |  |

Features:

```
Query  8        TCGAGCGGTAGAGAGAAGCTTGCTTCTCTTGAGAGCGGCGGACGGGTGAGTAATGCCTAG  67
                ||||||||||||||||||||||||||||||||||||||||||||||||||||||||||||
Sbjct  4138189  TCGAGCGGTAGAGAGAAGCTTGCTTCTCTTGAGAGCGGCGGACGGGTGAGTAATGCCTAG  4138130

Query  68       GAATCTGCCTGGTAGTGGGGGATAACGTTCGGAAACGGACGCTAATACCGCATACGTCCT  127
                ||||||||||||||||||||||||||||||||||||||||||||||||||||||||||||
Sbjct  4138129  GAATCTGCCTGGTAGTGGGGGATAACGTTCGGAAACGGACGCTAATACCGCATACGTCCT  4138070

Query  128      ACGGGAGAAAGCAGGGGACCTTCGGGCCTTGCGCTATCAGATGAGCCTAGGTCGGATTAG  187
                ||||||||||||||||||||||||||||||||||||||||||||||||||||||||||||
Sbjct  4138069  ACGGGAGAAAGCAGGGGACCTTCGGGCCTTGCGCTATCAGATGAGCCTAGGTCGGATTAG  4138010

Query  188      CTAGTTGGTGGGGTAATGGCTCACCAAGGCGACGATCCGTAACTGGTCTGAGAGGATGAT  247
                ||||||||||||||||||||||||||||||||||||||||||||||||||||||||||||
Sbjct  4138009  CTAGTTGGTGGGGTAATGGCTCACCAAGGCGACGATCCGTAACTGGTCTGAGAGGATGAT  4137950

Query  248      CAGTCACACTGGAACTGAGACACGGTCCAGACTCCTACGGGAGGCAGCAGTGGGGAATAT  307
                ||||||||||||||||||||||||||||||||||||||||||||||||||||||||||||
Sbjct  4137949  CAGTCACACTGGAACTGAGACACGGTCCAGACTCCTACGGGAGGCAGCAGTGGGGAATAT  4137890

Query  308      TGGACAATGGGCGAAAGCCTGATCCAGCCATGCCGCGTGTGTGAAGAAGGTCTTCGGATT  367
                ||||||||||||||||||||||||||||||||||||||||||||||||||||||||||||
Sbjct  4137889  TGGACAATGGGCGAAAGCCTGATCCAGCCATGCCGCGTGTGTGAAGAAGGTCTTCGGATT  4137830

Query  368      GTAAAGCACTTTAAGTTGGGAGGAAGGGCAGTTACCTAATACGTGATTGTTTTGACGTTA  427
                ||||||||||||||||||||||||||||||||||||||||||||||||||||||||||||
Sbjct  4137829  GTAAAGCACTTTAAGTTGGGAGGAAGGGCAGTTACCTAATACGTGATTGTTTTGACGTTA  4137770

Query  428      CCGACAGAATAAGCACCGGCTAACTCTGTGCCAGCAGCCGCGGTAATACAGAGGGTGCAA  487
                ||||||||||||||||||||||||||||||||||||||||||||||||||||||||||||
Sbjct  4137769  CCGACAGAATAAGCACCGGCTAACTCTGTGCCAGCAGCCGCGGTAATACAGAGGGTGCAA  4137710

Query  488      GCGTTAATCGGAATTACTGGGCGTAAAGCGCGCGTAGGTGGTTTGTTAAGTTGGATGTGA  547
                ||||||||||||||||||||||||||||||||||||||||||||||||||||||||||||
Sbjct  4137709  GCGTTAATCGGAATTACTGGGCGTAAAGCGCGCGTAGGTGGTTTGTTAAGTTGGATGTGA  4137650

Query  548      AATCCCCGGGCTCAACCTGGGAACTGCATTCAAAACTGACTGACTAGAGTATGGTAGAGG  607
                ||||||||||||||||||||||||||||||||||||||||||||||||||||||||||||
Sbjct  4137649  AATCCCCGGGCTCAACCTGGGAACTGCATTCAAAACTGACTGACTAGAGTATGGTAGAGG  4137590

Query  608      GTGGTGGAATTTCCTGTGTAGCGGTGAAATGCGTAGATATAGGAAGGAACACCAGTGGCG  667
                ||||||||||||||||||||||||||||||||||||||||||||||||||||||||||||
Sbjct  4137589  GTGGTGGAATTTCCTGTGTAGCGGTGAAATGCGTAGATATAGGAAGGAACACCAGTGGCG  4137530

Query  668      AAGGCGACCACCTGGACTAATACTGACACTGAGGTGCGAAAGCGTGGGGAGCAAACAGGA  727
                ||||||||||||||||||||||||||||||||||||||||||||||||||||||||||||
Sbjct  4137529  AAGGCGACCACCTGGACTAATACTGACACTGAGGTGCGAAAGCGTGGGGAGCAAACAGGA  4137470

Query  728      TTAGATACCCTGGTAGTCCACGCCGTAAACGATGTCAACTAGCCGTTGGAAGCCTTGAGC  787
                ||||||||||||||||||||||||||||||||||||||||||||||||||||||||||||
Sbjct  4137469  TTAGATACCCTGGTAGTCCACGCCGTAAACGATGTCAACTAGCCGTTGGAAGCCTTGAGC  4137410

Query  788      TTTTAGTGGCGCAGCTAACGCATTAAGTTGACCGCCTGGGGAGTACGGCCGCAAGGTTAA  847
                ||||||||||||||||||||||||||||||||||||||||||||||||||||||||||||
Sbjct  4137409  TTTTAGTGGCGCAGCTAACGCATTAAGTTGACCGCCTGGGGAGTACGGCCGCAAGGTTAA  4137350

Query  848      AACTCAAATGAATTGACGGGGGCCCGCACAAGCGGTGGAGCATGTGGTTTAATTCGAAGC  907
                ||||||||||||||||||||||||||||||||||||||||||||||||||||||||||||
Sbjct  4137349  AACTCAAATGAATTGACGGGGGCCCGCACAAGCGGTGGAGCATGTGGTTTAATTCGAAGC  4137290

Query  908      AACGCGAAGAACCTTACCAGGCCTTGACATCCAATGAACTTTCTAGAGATAGATTGGTGC  967
                ||||||||||||||||||||||||||||||||||||||||||||||||||||||||||||
Sbjct  4137289  AACGCGAAGAACCTTACCAGGCCTTGACATCCAATGAACTTTCTAGAGATAGATTGGTGC  4137230

Query  968      CTTCGGGAACATTGAGACAGGTGCTGCATGGCTGTCGTCAGCTCGTGT  1015
                ||||||||||||||||||||||||||||||||||||||||||||||||
Sbjct  4137229  CTTCGGGAACATTGAGACAGGTGCTGCATGGCTGTCGTCAGCTCGTGT  4137182
```

Range 4: 5506949 to 5507956GenBankGraphics

Next Match
Previous Match
First Match

Alignment statistics for match #4

| Score | Expect | Identities | Gaps | Strand | Frame |
| --- | --- | --- | --- | --- | --- |
| 1862 bits(1008) | 0.0() | 1008/1008(100%) | 0/1008(0%) | Plus/Plus |  |

Features:

```
Query  8        TCGAGCGGTAGAGAGAAGCTTGCTTCTCTTGAGAGCGGCGGACGGGTGAGTAATGCCTAG  67
                ||||||||||||||||||||||||||||||||||||||||||||||||||||||||||||
Sbjct  5506949  TCGAGCGGTAGAGAGAAGCTTGCTTCTCTTGAGAGCGGCGGACGGGTGAGTAATGCCTAG  5507008

Query  68       GAATCTGCCTGGTAGTGGGGGATAACGTTCGGAAACGGACGCTAATACCGCATACGTCCT  127
                ||||||||||||||||||||||||||||||||||||||||||||||||||||||||||||
Sbjct  5507009  GAATCTGCCTGGTAGTGGGGGATAACGTTCGGAAACGGACGCTAATACCGCATACGTCCT  5507068

Query  128      ACGGGAGAAAGCAGGGGACCTTCGGGCCTTGCGCTATCAGATGAGCCTAGGTCGGATTAG  187
                ||||||||||||||||||||||||||||||||||||||||||||||||||||||||||||
Sbjct  5507069  ACGGGAGAAAGCAGGGGACCTTCGGGCCTTGCGCTATCAGATGAGCCTAGGTCGGATTAG  5507128

Query  188      CTAGTTGGTGGGGTAATGGCTCACCAAGGCGACGATCCGTAACTGGTCTGAGAGGATGAT  247
                ||||||||||||||||||||||||||||||||||||||||||||||||||||||||||||
Sbjct  5507129  CTAGTTGGTGGGGTAATGGCTCACCAAGGCGACGATCCGTAACTGGTCTGAGAGGATGAT  5507188

Query  248      CAGTCACACTGGAACTGAGACACGGTCCAGACTCCTACGGGAGGCAGCAGTGGGGAATAT  307
                ||||||||||||||||||||||||||||||||||||||||||||||||||||||||||||
Sbjct  5507189  CAGTCACACTGGAACTGAGACACGGTCCAGACTCCTACGGGAGGCAGCAGTGGGGAATAT  5507248

Query  308      TGGACAATGGGCGAAAGCCTGATCCAGCCATGCCGCGTGTGTGAAGAAGGTCTTCGGATT  367
                ||||||||||||||||||||||||||||||||||||||||||||||||||||||||||||
Sbjct  5507249  TGGACAATGGGCGAAAGCCTGATCCAGCCATGCCGCGTGTGTGAAGAAGGTCTTCGGATT  5507308

Query  368      GTAAAGCACTTTAAGTTGGGAGGAAGGGCAGTTACCTAATACGTGATTGTTTTGACGTTA  427
                ||||||||||||||||||||||||||||||||||||||||||||||||||||||||||||
Sbjct  5507309  GTAAAGCACTTTAAGTTGGGAGGAAGGGCAGTTACCTAATACGTGATTGTTTTGACGTTA  5507368

Query  428      CCGACAGAATAAGCACCGGCTAACTCTGTGCCAGCAGCCGCGGTAATACAGAGGGTGCAA  487
                ||||||||||||||||||||||||||||||||||||||||||||||||||||||||||||
Sbjct  5507369  CCGACAGAATAAGCACCGGCTAACTCTGTGCCAGCAGCCGCGGTAATACAGAGGGTGCAA  5507428

Query  488      GCGTTAATCGGAATTACTGGGCGTAAAGCGCGCGTAGGTGGTTTGTTAAGTTGGATGTGA  547
                ||||||||||||||||||||||||||||||||||||||||||||||||||||||||||||
Sbjct  5507429  GCGTTAATCGGAATTACTGGGCGTAAAGCGCGCGTAGGTGGTTTGTTAAGTTGGATGTGA  5507488

Query  548      AATCCCCGGGCTCAACCTGGGAACTGCATTCAAAACTGACTGACTAGAGTATGGTAGAGG  607
                ||||||||||||||||||||||||||||||||||||||||||||||||||||||||||||
Sbjct  5507489  AATCCCCGGGCTCAACCTGGGAACTGCATTCAAAACTGACTGACTAGAGTATGGTAGAGG  5507548

Query  608      GTGGTGGAATTTCCTGTGTAGCGGTGAAATGCGTAGATATAGGAAGGAACACCAGTGGCG  667
                ||||||||||||||||||||||||||||||||||||||||||||||||||||||||||||
Sbjct  5507549  GTGGTGGAATTTCCTGTGTAGCGGTGAAATGCGTAGATATAGGAAGGAACACCAGTGGCG  5507608

Query  668      AAGGCGACCACCTGGACTAATACTGACACTGAGGTGCGAAAGCGTGGGGAGCAAACAGGA  727
                ||||||||||||||||||||||||||||||||||||||||||||||||||||||||||||
Sbjct  5507609  AAGGCGACCACCTGGACTAATACTGACACTGAGGTGCGAAAGCGTGGGGAGCAAACAGGA  5507668

Query  728      TTAGATACCCTGGTAGTCCACGCCGTAAACGATGTCAACTAGCCGTTGGAAGCCTTGAGC  787
                ||||||||||||||||||||||||||||||||||||||||||||||||||||||||||||
Sbjct  5507669  TTAGATACCCTGGTAGTCCACGCCGTAAACGATGTCAACTAGCCGTTGGAAGCCTTGAGC  5507728

Query  788      TTTTAGTGGCGCAGCTAACGCATTAAGTTGACCGCCTGGGGAGTACGGCCGCAAGGTTAA  847
                ||||||||||||||||||||||||||||||||||||||||||||||||||||||||||||
Sbjct  5507729  TTTTAGTGGCGCAGCTAACGCATTAAGTTGACCGCCTGGGGAGTACGGCCGCAAGGTTAA  5507788

Query  848      AACTCAAATGAATTGACGGGGGCCCGCACAAGCGGTGGAGCATGTGGTTTAATTCGAAGC  907
                ||||||||||||||||||||||||||||||||||||||||||||||||||||||||||||
Sbjct  5507789  AACTCAAATGAATTGACGGGGGCCCGCACAAGCGGTGGAGCATGTGGTTTAATTCGAAGC  5507848

Query  908      AACGCGAAGAACCTTACCAGGCCTTGACATCCAATGAACTTTCTAGAGATAGATTGGTGC  967
                ||||||||||||||||||||||||||||||||||||||||||||||||||||||||||||
Sbjct  5507849  AACGCGAAGAACCTTACCAGGCCTTGACATCCAATGAACTTTCTAGAGATAGATTGGTGC  5507908

Query  968      CTTCGGGAACATTGAGACAGGTGCTGCATGGCTGTCGTCAGCTCGTGT  1015
                ||||||||||||||||||||||||||||||||||||||||||||||||
Sbjct  5507909  CTTCGGGAACATTGAGACAGGTGCTGCATGGCTGTCGTCAGCTCGTGT  5507956
```

Range 5: 4900351 to 4901358GenBankGraphics

Next Match
Previous Match
First Match

Alignment statistics for match #5

| Score | Expect | Identities | Gaps | Strand | Frame |
| --- | --- | --- | --- | --- | --- |
| 1857 bits(1005) | 0.0() | 1007/1008(99%) | 0/1008(0%) | Plus/Plus |  |

Features:

```
Query  8        TCGAGCGGTAGAGAGAAGCTTGCTTCTCTTGAGAGCGGCGGACGGGTGAGTAATGCCTAG  67
                |||||||||||||||||||| |||||||||||||||||||||||||||||||||||||||
Sbjct  4900351  TCGAGCGGTAGAGAGAAGCTCGCTTCTCTTGAGAGCGGCGGACGGGTGAGTAATGCCTAG  4900410

Query  68       GAATCTGCCTGGTAGTGGGGGATAACGTTCGGAAACGGACGCTAATACCGCATACGTCCT  127
                ||||||||||||||||||||||||||||||||||||||||||||||||||||||||||||
Sbjct  4900411  GAATCTGCCTGGTAGTGGGGGATAACGTTCGGAAACGGACGCTAATACCGCATACGTCCT  4900470

Query  128      ACGGGAGAAAGCAGGGGACCTTCGGGCCTTGCGCTATCAGATGAGCCTAGGTCGGATTAG  187
                ||||||||||||||||||||||||||||||||||||||||||||||||||||||||||||
Sbjct  4900471  ACGGGAGAAAGCAGGGGACCTTCGGGCCTTGCGCTATCAGATGAGCCTAGGTCGGATTAG  4900530

Query  188      CTAGTTGGTGGGGTAATGGCTCACCAAGGCGACGATCCGTAACTGGTCTGAGAGGATGAT  247
                ||||||||||||||||||||||||||||||||||||||||||||||||||||||||||||
Sbjct  4900531  CTAGTTGGTGGGGTAATGGCTCACCAAGGCGACGATCCGTAACTGGTCTGAGAGGATGAT  4900590

Query  248      CAGTCACACTGGAACTGAGACACGGTCCAGACTCCTACGGGAGGCAGCAGTGGGGAATAT  307
                ||||||||||||||||||||||||||||||||||||||||||||||||||||||||||||
Sbjct  4900591  CAGTCACACTGGAACTGAGACACGGTCCAGACTCCTACGGGAGGCAGCAGTGGGGAATAT  4900650

Query  308      TGGACAATGGGCGAAAGCCTGATCCAGCCATGCCGCGTGTGTGAAGAAGGTCTTCGGATT  367
                ||||||||||||||||||||||||||||||||||||||||||||||||||||||||||||
Sbjct  4900651  TGGACAATGGGCGAAAGCCTGATCCAGCCATGCCGCGTGTGTGAAGAAGGTCTTCGGATT  4900710

Query  368      GTAAAGCACTTTAAGTTGGGAGGAAGGGCAGTTACCTAATACGTGATTGTTTTGACGTTA  427
                ||||||||||||||||||||||||||||||||||||||||||||||||||||||||||||
Sbjct  4900711  GTAAAGCACTTTAAGTTGGGAGGAAGGGCAGTTACCTAATACGTGATTGTTTTGACGTTA  4900770

Query  428      CCGACAGAATAAGCACCGGCTAACTCTGTGCCAGCAGCCGCGGTAATACAGAGGGTGCAA  487
                ||||||||||||||||||||||||||||||||||||||||||||||||||||||||||||
Sbjct  4900771  CCGACAGAATAAGCACCGGCTAACTCTGTGCCAGCAGCCGCGGTAATACAGAGGGTGCAA  4900830

Query  488      GCGTTAATCGGAATTACTGGGCGTAAAGCGCGCGTAGGTGGTTTGTTAAGTTGGATGTGA  547
                ||||||||||||||||||||||||||||||||||||||||||||||||||||||||||||
Sbjct  4900831  GCGTTAATCGGAATTACTGGGCGTAAAGCGCGCGTAGGTGGTTTGTTAAGTTGGATGTGA  4900890

Query  548      AATCCCCGGGCTCAACCTGGGAACTGCATTCAAAACTGACTGACTAGAGTATGGTAGAGG  607
                ||||||||||||||||||||||||||||||||||||||||||||||||||||||||||||
Sbjct  4900891  AATCCCCGGGCTCAACCTGGGAACTGCATTCAAAACTGACTGACTAGAGTATGGTAGAGG  4900950

Query  608      GTGGTGGAATTTCCTGTGTAGCGGTGAAATGCGTAGATATAGGAAGGAACACCAGTGGCG  667
                ||||||||||||||||||||||||||||||||||||||||||||||||||||||||||||
Sbjct  4900951  GTGGTGGAATTTCCTGTGTAGCGGTGAAATGCGTAGATATAGGAAGGAACACCAGTGGCG  4901010

Query  668      AAGGCGACCACCTGGACTAATACTGACACTGAGGTGCGAAAGCGTGGGGAGCAAACAGGA  727
                ||||||||||||||||||||||||||||||||||||||||||||||||||||||||||||
Sbjct  4901011  AAGGCGACCACCTGGACTAATACTGACACTGAGGTGCGAAAGCGTGGGGAGCAAACAGGA  4901070

Query  728      TTAGATACCCTGGTAGTCCACGCCGTAAACGATGTCAACTAGCCGTTGGAAGCCTTGAGC  787
                ||||||||||||||||||||||||||||||||||||||||||||||||||||||||||||
Sbjct  4901071  TTAGATACCCTGGTAGTCCACGCCGTAAACGATGTCAACTAGCCGTTGGAAGCCTTGAGC  4901130

Query  788      TTTTAGTGGCGCAGCTAACGCATTAAGTTGACCGCCTGGGGAGTACGGCCGCAAGGTTAA  847
                ||||||||||||||||||||||||||||||||||||||||||||||||||||||||||||
Sbjct  4901131  TTTTAGTGGCGCAGCTAACGCATTAAGTTGACCGCCTGGGGAGTACGGCCGCAAGGTTAA  4901190

Query  848      AACTCAAATGAATTGACGGGGGCCCGCACAAGCGGTGGAGCATGTGGTTTAATTCGAAGC  907
                ||||||||||||||||||||||||||||||||||||||||||||||||||||||||||||
Sbjct  4901191  AACTCAAATGAATTGACGGGGGCCCGCACAAGCGGTGGAGCATGTGGTTTAATTCGAAGC  4901250

Query  908      AACGCGAAGAACCTTACCAGGCCTTGACATCCAATGAACTTTCTAGAGATAGATTGGTGC  967
                ||||||||||||||||||||||||||||||||||||||||||||||||||||||||||||
Sbjct  4901251  AACGCGAAGAACCTTACCAGGCCTTGACATCCAATGAACTTTCTAGAGATAGATTGGTGC  4901310

Query  968      CTTCGGGAACATTGAGACAGGTGCTGCATGGCTGTCGTCAGCTCGTGT  1015
                ||||||||||||||||||||||||||||||||||||||||||||||||
Sbjct  4901311  CTTCGGGAACATTGAGACAGGTGCTGCATGGCTGTCGTCAGCTCGTGT  4901358
```

Download

FASTA (complete sequence)

FASTA (aligned sequences)

GenBank (complete sequence)

Continue
Cancel

GenBankGraphics

Next
Previous
Descriptions

uncultured bacterium partial 16S rRNA gene, clone K15.87 KSS

Sequence ID: HE576021.1Length: 1459Number of Matches: 1

Related Information

Range 1: 35 to 1042GenBankGraphics

Next Match
Previous Match
First Match

Alignment statistics for match #1

| Score | Expect | Identities | Gaps | Strand | Frame |
| --- | --- | --- | --- | --- | --- |
| 1862 bits(1008) | 0.0() | 1008/1008(100%) | 0/1008(0%) | Plus/Plus |  |

Features:

```
Query  8     TCGAGCGGTAGAGAGAAGCTTGCTTCTCTTGAGAGCGGCGGACGGGTGAGTAATGCCTAG  67
             ||||||||||||||||||||||||||||||||||||||||||||||||||||||||||||
Sbjct  35    TCGAGCGGTAGAGAGAAGCTTGCTTCTCTTGAGAGCGGCGGACGGGTGAGTAATGCCTAG  94

Query  68    GAATCTGCCTGGTAGTGGGGGATAACGTTCGGAAACGGACGCTAATACCGCATACGTCCT  127
             ||||||||||||||||||||||||||||||||||||||||||||||||||||||||||||
Sbjct  95    GAATCTGCCTGGTAGTGGGGGATAACGTTCGGAAACGGACGCTAATACCGCATACGTCCT  154

Query  128   ACGGGAGAAAGCAGGGGACCTTCGGGCCTTGCGCTATCAGATGAGCCTAGGTCGGATTAG  187
             ||||||||||||||||||||||||||||||||||||||||||||||||||||||||||||
Sbjct  155   ACGGGAGAAAGCAGGGGACCTTCGGGCCTTGCGCTATCAGATGAGCCTAGGTCGGATTAG  214

Query  188   CTAGTTGGTGGGGTAATGGCTCACCAAGGCGACGATCCGTAACTGGTCTGAGAGGATGAT  247
             ||||||||||||||||||||||||||||||||||||||||||||||||||||||||||||
Sbjct  215   CTAGTTGGTGGGGTAATGGCTCACCAAGGCGACGATCCGTAACTGGTCTGAGAGGATGAT  274

Query  248   CAGTCACACTGGAACTGAGACACGGTCCAGACTCCTACGGGAGGCAGCAGTGGGGAATAT  307
             ||||||||||||||||||||||||||||||||||||||||||||||||||||||||||||
Sbjct  275   CAGTCACACTGGAACTGAGACACGGTCCAGACTCCTACGGGAGGCAGCAGTGGGGAATAT  334

Query  308   TGGACAATGGGCGAAAGCCTGATCCAGCCATGCCGCGTGTGTGAAGAAGGTCTTCGGATT  367
             ||||||||||||||||||||||||||||||||||||||||||||||||||||||||||||
Sbjct  335   TGGACAATGGGCGAAAGCCTGATCCAGCCATGCCGCGTGTGTGAAGAAGGTCTTCGGATT  394

Query  368   GTAAAGCACTTTAAGTTGGGAGGAAGGGCAGTTACCTAATACGTGATTGTTTTGACGTTA  427
             ||||||||||||||||||||||||||||||||||||||||||||||||||||||||||||
Sbjct  395   GTAAAGCACTTTAAGTTGGGAGGAAGGGCAGTTACCTAATACGTGATTGTTTTGACGTTA  454

Query  428   CCGACAGAATAAGCACCGGCTAACTCTGTGCCAGCAGCCGCGGTAATACAGAGGGTGCAA  487
             ||||||||||||||||||||||||||||||||||||||||||||||||||||||||||||
Sbjct  455   CCGACAGAATAAGCACCGGCTAACTCTGTGCCAGCAGCCGCGGTAATACAGAGGGTGCAA  514

Query  488   GCGTTAATCGGAATTACTGGGCGTAAAGCGCGCGTAGGTGGTTTGTTAAGTTGGATGTGA  547
             ||||||||||||||||||||||||||||||||||||||||||||||||||||||||||||
Sbjct  515   GCGTTAATCGGAATTACTGGGCGTAAAGCGCGCGTAGGTGGTTTGTTAAGTTGGATGTGA  574

Query  548   AATCCCCGGGCTCAACCTGGGAACTGCATTCAAAACTGACTGACTAGAGTATGGTAGAGG  607
             ||||||||||||||||||||||||||||||||||||||||||||||||||||||||||||
Sbjct  575   AATCCCCGGGCTCAACCTGGGAACTGCATTCAAAACTGACTGACTAGAGTATGGTAGAGG  634

Query  608   GTGGTGGAATTTCCTGTGTAGCGGTGAAATGCGTAGATATAGGAAGGAACACCAGTGGCG  667
             ||||||||||||||||||||||||||||||||||||||||||||||||||||||||||||
Sbjct  635   GTGGTGGAATTTCCTGTGTAGCGGTGAAATGCGTAGATATAGGAAGGAACACCAGTGGCG  694

Query  668   AAGGCGACCACCTGGACTAATACTGACACTGAGGTGCGAAAGCGTGGGGAGCAAACAGGA  727
             ||||||||||||||||||||||||||||||||||||||||||||||||||||||||||||
Sbjct  695   AAGGCGACCACCTGGACTAATACTGACACTGAGGTGCGAAAGCGTGGGGAGCAAACAGGA  754

Query  728   TTAGATACCCTGGTAGTCCACGCCGTAAACGATGTCAACTAGCCGTTGGAAGCCTTGAGC  787
             ||||||||||||||||||||||||||||||||||||||||||||||||||||||||||||
Sbjct  755   TTAGATACCCTGGTAGTCCACGCCGTAAACGATGTCAACTAGCCGTTGGAAGCCTTGAGC  814

Query  788   TTTTAGTGGCGCAGCTAACGCATTAAGTTGACCGCCTGGGGAGTACGGCCGCAAGGTTAA  847
             ||||||||||||||||||||||||||||||||||||||||||||||||||||||||||||
Sbjct  815   TTTTAGTGGCGCAGCTAACGCATTAAGTTGACCGCCTGGGGAGTACGGCCGCAAGGTTAA  874

Query  848   AACTCAAATGAATTGACGGGGGCCCGCACAAGCGGTGGAGCATGTGGTTTAATTCGAAGC  907
             ||||||||||||||||||||||||||||||||||||||||||||||||||||||||||||
Sbjct  875   AACTCAAATGAATTGACGGGGGCCCGCACAAGCGGTGGAGCATGTGGTTTAATTCGAAGC  934

Query  908   AACGCGAAGAACCTTACCAGGCCTTGACATCCAATGAACTTTCTAGAGATAGATTGGTGC  967
             ||||||||||||||||||||||||||||||||||||||||||||||||||||||||||||
Sbjct  935   AACGCGAAGAACCTTACCAGGCCTTGACATCCAATGAACTTTCTAGAGATAGATTGGTGC  994

Query  968   CTTCGGGAACATTGAGACAGGTGCTGCATGGCTGTCGTCAGCTCGTGT  1015
             ||||||||||||||||||||||||||||||||||||||||||||||||
Sbjct  995   CTTCGGGAACATTGAGACAGGTGCTGCATGGCTGTCGTCAGCTCGTGT  1042
```

Download

FASTA (complete sequence)

FASTA (aligned sequences)

GenBank (complete sequence)

Continue
Cancel

GenBankGraphics

Next
Previous
Descriptions

Pseudomonas sp. PX7\_S1 16S ribosomal RNA gene, partial sequence

Sequence ID: JF274937.1Length: 1516Number of Matches: 1

Related Information

Range 1: 56 to 1063GenBankGraphics

Next Match
Previous Match
First Match

Alignment statistics for match #1

| Score | Expect | Identities | Gaps | Strand | Frame |
| --- | --- | --- | --- | --- | --- |
| 1862 bits(1008) | 0.0() | 1008/1008(100%) | 0/1008(0%) | Plus/Plus |  |

Features:

```
Query  8     TCGAGCGGTAGAGAGAAGCTTGCTTCTCTTGAGAGCGGCGGACGGGTGAGTAATGCCTAG  67
             ||||||||||||||||||||||||||||||||||||||||||||||||||||||||||||
Sbjct  56    TCGAGCGGTAGAGAGAAGCTTGCTTCTCTTGAGAGCGGCGGACGGGTGAGTAATGCCTAG  115

Query  68    GAATCTGCCTGGTAGTGGGGGATAACGTTCGGAAACGGACGCTAATACCGCATACGTCCT  127
             ||||||||||||||||||||||||||||||||||||||||||||||||||||||||||||
Sbjct  116   GAATCTGCCTGGTAGTGGGGGATAACGTTCGGAAACGGACGCTAATACCGCATACGTCCT  175

Query  128   ACGGGAGAAAGCAGGGGACCTTCGGGCCTTGCGCTATCAGATGAGCCTAGGTCGGATTAG  187
             ||||||||||||||||||||||||||||||||||||||||||||||||||||||||||||
Sbjct  176   ACGGGAGAAAGCAGGGGACCTTCGGGCCTTGCGCTATCAGATGAGCCTAGGTCGGATTAG  235

Query  188   CTAGTTGGTGGGGTAATGGCTCACCAAGGCGACGATCCGTAACTGGTCTGAGAGGATGAT  247
             ||||||||||||||||||||||||||||||||||||||||||||||||||||||||||||
Sbjct  236   CTAGTTGGTGGGGTAATGGCTCACCAAGGCGACGATCCGTAACTGGTCTGAGAGGATGAT  295

Query  248   CAGTCACACTGGAACTGAGACACGGTCCAGACTCCTACGGGAGGCAGCAGTGGGGAATAT  307
             ||||||||||||||||||||||||||||||||||||||||||||||||||||||||||||
Sbjct  296   CAGTCACACTGGAACTGAGACACGGTCCAGACTCCTACGGGAGGCAGCAGTGGGGAATAT  355

Query  308   TGGACAATGGGCGAAAGCCTGATCCAGCCATGCCGCGTGTGTGAAGAAGGTCTTCGGATT  367
             ||||||||||||||||||||||||||||||||||||||||||||||||||||||||||||
Sbjct  356   TGGACAATGGGCGAAAGCCTGATCCAGCCATGCCGCGTGTGTGAAGAAGGTCTTCGGATT  415

Query  368   GTAAAGCACTTTAAGTTGGGAGGAAGGGCAGTTACCTAATACGTGATTGTTTTGACGTTA  427
             ||||||||||||||||||||||||||||||||||||||||||||||||||||||||||||
Sbjct  416   GTAAAGCACTTTAAGTTGGGAGGAAGGGCAGTTACCTAATACGTGATTGTTTTGACGTTA  475

Query  428   CCGACAGAATAAGCACCGGCTAACTCTGTGCCAGCAGCCGCGGTAATACAGAGGGTGCAA  487
             ||||||||||||||||||||||||||||||||||||||||||||||||||||||||||||
Sbjct  476   CCGACAGAATAAGCACCGGCTAACTCTGTGCCAGCAGCCGCGGTAATACAGAGGGTGCAA  535

Query  488   GCGTTAATCGGAATTACTGGGCGTAAAGCGCGCGTAGGTGGTTTGTTAAGTTGGATGTGA  547
             ||||||||||||||||||||||||||||||||||||||||||||||||||||||||||||
Sbjct  536   GCGTTAATCGGAATTACTGGGCGTAAAGCGCGCGTAGGTGGTTTGTTAAGTTGGATGTGA  595

Query  548   AATCCCCGGGCTCAACCTGGGAACTGCATTCAAAACTGACTGACTAGAGTATGGTAGAGG  607
             ||||||||||||||||||||||||||||||||||||||||||||||||||||||||||||
Sbjct  596   AATCCCCGGGCTCAACCTGGGAACTGCATTCAAAACTGACTGACTAGAGTATGGTAGAGG  655

Query  608   GTGGTGGAATTTCCTGTGTAGCGGTGAAATGCGTAGATATAGGAAGGAACACCAGTGGCG  667
             ||||||||||||||||||||||||||||||||||||||||||||||||||||||||||||
Sbjct  656   GTGGTGGAATTTCCTGTGTAGCGGTGAAATGCGTAGATATAGGAAGGAACACCAGTGGCG  715

Query  668   AAGGCGACCACCTGGACTAATACTGACACTGAGGTGCGAAAGCGTGGGGAGCAAACAGGA  727
             ||||||||||||||||||||||||||||||||||||||||||||||||||||||||||||
Sbjct  716   AAGGCGACCACCTGGACTAATACTGACACTGAGGTGCGAAAGCGTGGGGAGCAAACAGGA  775

Query  728   TTAGATACCCTGGTAGTCCACGCCGTAAACGATGTCAACTAGCCGTTGGAAGCCTTGAGC  787
             ||||||||||||||||||||||||||||||||||||||||||||||||||||||||||||
Sbjct  776   TTAGATACCCTGGTAGTCCACGCCGTAAACGATGTCAACTAGCCGTTGGAAGCCTTGAGC  835

Query  788   TTTTAGTGGCGCAGCTAACGCATTAAGTTGACCGCCTGGGGAGTACGGCCGCAAGGTTAA  847
             ||||||||||||||||||||||||||||||||||||||||||||||||||||||||||||
Sbjct  836   TTTTAGTGGCGCAGCTAACGCATTAAGTTGACCGCCTGGGGAGTACGGCCGCAAGGTTAA  895

Query  848   AACTCAAATGAATTGACGGGGGCCCGCACAAGCGGTGGAGCATGTGGTTTAATTCGAAGC  907
             ||||||||||||||||||||||||||||||||||||||||||||||||||||||||||||
Sbjct  896   AACTCAAATGAATTGACGGGGGCCCGCACAAGCGGTGGAGCATGTGGTTTAATTCGAAGC  955

Query  908   AACGCGAAGAACCTTACCAGGCCTTGACATCCAATGAACTTTCTAGAGATAGATTGGTGC  967
             ||||||||||||||||||||||||||||||||||||||||||||||||||||||||||||
Sbjct  956   AACGCGAAGAACCTTACCAGGCCTTGACATCCAATGAACTTTCTAGAGATAGATTGGTGC  1015

Query  968   CTTCGGGAACATTGAGACAGGTGCTGCATGGCTGTCGTCAGCTCGTGT  1015
             ||||||||||||||||||||||||||||||||||||||||||||||||
Sbjct  1016  CTTCGGGAACATTGAGACAGGTGCTGCATGGCTGTCGTCAGCTCGTGT  1063
```


BLAST is a registered trademark of the National Library of Medicine

Support center
Mailing list


YouTube

- National Library Of Medicine
- National Institutes Of Health
- U.S. Department of Health & Human Services
- USA.gov

### NCBI


National Center for Biotechnology Information,
 U.S. National Library of Medicine

8600 Rockville Pike,
Bethesda
 MD,
20894
USA

Policies and Guidelines
|
Contact


PreferencesTurn off

External link. Please review our privacy policy.
