# Supplementary material for: Isothermal microcalorimetry for thermal viable count of microorganisms in pure cultures and stabilized formulations
Source: BMC Microbiol. 2019 Mar 21;19:65. doi: 10.1186/s12866-019-1432-8 (PMC6429831; doi:10.1186/s12866-019-1432-8)
Supplement: Supplementary file 3 — 16S rRNA gene sequences’ BLASTN hits in zipped HTML format. (ZIP 15810 kb) [file 12866_2019_1432_MOESM3_ESM.zip › Best blastn hits/NCBI Blast_M yellow SS R -- 8..1070 of sequence (1063.html]

NCBI Blast:M yellow SS R -- 8..1070 of sequence (1063


- NCBI Home
- Sign in to NCBI
- Skip to Main Content
- Skip to Navigation
- About NCBI Accesskeys

U.S. National Library of Medicine

NCBI
National Center for Biotechnology Information

- My NCBI
- Sign in to NCBI
- Register
- Sign Out

BLAST ^®^ » blastn suite » RID-A50HVVM5014


- Home
- Recent Results
- Saved Strategies
- Help

BLAST Results


Edit and Resubmit
Save Search Strategies
[Sign in above to save your search strategy]

Formatting options 


Download


How to read this page
Blast report description
Questions/comments


|  |  |
| --- | --- |
| Formatting options | |
| Show | Alignment as  HTML Plain text   Old View Reset form to defaults [?]  These options control formatting of alignments in results pages. The default is HTML, but other formats (including plain text) are available. PSSM and PssmWithParameters are representations of Position Specific Scoring Matrices and are only available for PSI-BLAST. The Advanced view option allows the database descriptions to be sorted by various indices in a table. |
| Alignment View | Pairwise Pairwise with dots for identities Query-anchored with dots for identities Query-anchored with letters for identities Flat query-anchored with dots for identities Flat query-anchored with letters for identities [?]  Choose how to view alignments. The default "pairwise" view shows how each subject sequence aligns individually to the query sequence. The "query-anchored" view shows how all subject sequences align to the query sequence. For each view type, you can choose to show "identities" (matching residues) as letters or dots. more... |
| Display | Graphical Overview   Linkout   Sequence Retrieval  NCBI-gi   CDS feature [?]  - Graphical Overview: Graphical Overview: Show graph of similar sequence regions aligned to query.   more... - NCBI-gi: Show NCBI gi identifiers. - CDS feature: Show annotated coding region and translation.   more... |
| Masking | Character:   X for protein, n for nucleotide Lower Case  Color:  Black Grey Red [?]  - Masking Character: Display masked (filtered) sequence regions as lower-case or as specific letters (N for nucleotide, P for protein). - Masking Color: Display masked sequence regions in the given color. |
| Limit results | Descriptions:  10 50 100 Graphical overview:  0 10 50 100  Alignments:  0 10 50 100 Line length:  60 90 120 150 [?]  - Descriptions: Show short descriptions for up to the given number of sequences. - Alignments: Show alignments for up to the given number of sequences, in order of statistical significance. - Line lenghth: Number of letters to show on one line in an alignment. |
|  | Organism Type common name, binomial, taxid, or group name. Only 20 top taxa will be shown.     Exclude   [?]  Show only sequences from the given organism. |
|  | Entrez query:  [?]  Show only those sequences that match the given Entrez query. more... |
|  | Expect Min:  Expect Max:  [?]  Show only sequences with expect values in the given range. more... |
|  | Percent Identity Min:  Percent Identity Max:  [?]  Show only sequences with percent identity values in the given range. |
| Format for | PSI-BLAST with inclusion threshold:  [?]  - Format for PSI-BLAST: The Position-Specific Iterated BLAST (PSI-BLAST) program performs iterative searches with a protein query,   in which sequences found in one round of search are used to build a custom score model for the next round.   more... - Inclusion Threshold: This sets the statistical significance threshold for including a sequence in the model used   by PSI-BLAST to create the PSSM on the next iteration. |

|  |  |  |  |  |  |
| --- | --- | --- | --- | --- | --- |
| Download | | | | | |
| Alignment  Text XML ASN.1 JSON Seq-align Hit Table(text) Hit Table(csv) Multiple-file XML2 Single-file XML2 Multiple-file JSON Single-file JSON SAM | Search Strategies  ASN.1 | PSSM to restart search  PSSM | [?] |  |  |

The Download link provides BLAST output that may be used as input to another program.
This includes parseable formats such as the tabular report or XML as well as the Search Strategy files read by the BLAST+ applications.
More details on the parseable (XML, tabular, and ASN.1) reports can be found at
https://www.ncbi.nlm.nih.gov/books/NBK153387/  
  

The following formats are offered under the Alignment section:  
1). "Text". Non-HTML standard BLAST report.  
2). "XML". XML report based upon the DTD at https://www.ncbi.nlm.nih.gov/data\_specs/dtd/NCBI\_BlastOutput.dtd  
3). "ASN.1". Alignment written out in Abstract Syntax Notation 1.  
4). "JSON Seq-align". Alignment written out in JSON.  
4). "Hit Table(text)". The tabular report as text.  
5). "Hit Table(csv)". The tabular report ready for import into spread-sheet programs like Excel.  
6). "XML2". New XML format described at ftp://ftp.ncbi.nlm.nih.gov/blast/documents/NEWXML/xml2.pdf.  
7). "JSON". New JSON format described at ftp://ftp.ncbi.nlm.nih.gov/blast/documents/NEWXML/xml2.pdf.  
8). "SAM". Sequence Alignment Map format.

XML2 and JSON can be downloaded either as one file per query (multiple-file) or one file for all queries (single-file). These formats are listed as Multiple-file XML2 (and JSON) or Single-file XML (and JSON).

The following report is offered under the Search Strategy section:  
1). "ASN.1" Search Strategy. A record of the parameters, query, and database used in the search. This file can be used to start a stand-alone BLAST search, see
https://www.ncbi.nlm.nih.gov/books/NBK1763/#CmdLineAppsManual.I455\_BLAST\_search\_stra


# Job title: M yellow SS R -- 8..1070 of sequence (1063

Results for:

lcl|Query\_116721 M yellow SS R -- 8..1070 of sequence(1063bp)
[?]

Your BLAST job specified more than one input sequence.
This box lets you choose which input sequence to show BLAST results for.

RID
:   A50HVVM5014 (Expires on 03-10 19:46 pm)

Query ID
:   lcl|Query\_116721
:   lcl|Query\_116721

Description
:   M yellow SS R -- 8..1070 of sequence

Molecule type
:   nucleic acid

Query Length
:   1063

Database Name
:   nr

Description
:   Nucleotide collection (nt) See details

Program
:   BLASTN 2.8.0+ Citation

  

Reference 

Zheng Zhang, Scott Schwartz, Lukas Wagner, and Webb Miller (2000), "A greedy algorithm for aligning DNA sequences", J Comput Biol 2000; 7(1-2):203-14.

Reference - database indexing

Aleksandr Morgulis, George Coulouris, Yan Raytselis, Thomas L. Madden, Richa Agarwala, Alejandro A. Schäffer (2008), "Database Indexing for Production MegaBLAST Searches", Bioinformatics 24:1757-1764.

Other reports:
Search Summary

[Taxonomy reports]
[Distance tree of results]
[MSA viewer]

Search Parameters

| Search parameter name | Search parameter value |
| --- | --- |
| Program | blastn |
| Word size | 28 |
| Expect value | 10 |
| Hitlist size | 100 |
| Match/Mismatch scores | 1,-2 |
| Gapcosts | 0,2.5 |
| Low Complexity Filter | Yes |
| Filter string | L;m; |
| Genetic Code | 1 |

Database

| Database parameter name | Database parameter value |
| --- | --- |
| Posted date | Mar 7, 2018 1:58 PM |
| Number of letters | 174,044,644,244 |
| Number of sequences | 46,882,714 |
| Entrez query | Includes:  Excludes:  None |

Karlin-Altschul statistics

| Params | Ungapped | Gapped |
| --- | --- | --- |
| Lambda | 1.33271 | 1.28 |
| K | 0.620991 | 0.46 |
| H | 1.12409 | 0.85 |

Results Statistics

| Results Statistics parameter name | Results Statistics parameter value |
| --- | --- |
| Length adjustment | 35 |
| Effective length of query | 1028 |
| Effective length of database | 172403749254 |
| Effective search space | 177231054233112 |
| Effective search space used | 177231054233112 |


## Graphic Summary

### Distribution of the top 132 Blast Hits on 100 subject sequences [?]

The graphic is an overview of the database sequences aligned to the query sequence. These are represented horizontal bars colored coded by score and showing the extent
of the alignment on the query sequence. Separate aligned regions on the same database sequence are connected by a thin grey line.
Mousing over an alignment shows the database sequence title. Clicking an alignment displays a box with more details about the alignment and
link to the sequence alignment itself in the Alignments section of the report.

Mouse over to see the title, click to show alignments

Color key for alignment scores

<40

40-50

50-80

80-200

>=200

Query

1

200

400

600

800

1000

Pseudomonas poae strain T23 16S ribosomal RNA gene, com..

Score:1938 Evalue:0

Accession:EU111704.2

Alignment

Uncultured Pseudomonas sp. gene for 16S ribosomal RNA, ..

Score:1934 Evalue:0

Accession:LC140869.1

Alignment

Pseudomonas sp. 2-36(2010) 16S ribosomal RNA gene, part..

Score:1932 Evalue:0

Accession:HQ403088.2

Alignment

Pseudomonas fluorescens strain SWFU22 16S ribosomal RNA..

Score:1932 Evalue:0

Accession:KJ756337.1

Alignment

Pseudomonas putida partial 16S rRNA gene, strain CFBP 5..

Score:1932 Evalue:0

Accession:HF545843.1

Alignment

Pseudomonas sp. WH1 16S ribosomal RNA gene, partial seq..

Score:1932 Evalue:0

Accession:FJ262366.1

Alignment

Pseudomonas sp. S09G 359 chromosome

Score:1929 Evalue:0

Accession:CP025263.1

Alignment

Pseudomonas poae strain AAS-144 16S ribosomal RNA gene,..

Score:1929 Evalue:0

Accession:KY810658.1

Alignment

Pseudomonas poae strain BS2776 genome assembly, chromos..

Score:1929 Evalue:0

Accession:LT629706.1

Alignment

Uncultured bacterium partial 16S rRNA gene, clone clone..

Score:1929 Evalue:0

Accession:LT595870.1

Alignment

Uncultured bacterium clone MTWL201306-19 16S ribosomal ..

Score:1929 Evalue:0

Accession:KX509247.1

Alignment

Pseudomonas sp. 23K33a 16S ribosomal RNA gene, partial ..

Score:1929 Evalue:0

Accession:KT825765.1

Alignment

Pseudomonas sp. 17K8 16S ribosomal RNA gene, partial se..

Score:1929 Evalue:0

Accession:KT825736.1

Alignment

Pseudomonas poae strain B42 16S ribosomal RNA gene, par..

Score:1929 Evalue:0

Accession:KT767705.1

Alignment

Pseudomonas sp. HP10C 16S ribosomal RNA gene, partial s..

Score:1929 Evalue:0

Accession:KM187375.1

Alignment

Pseudomonas fluorescens PICF7, complete genome

Score:1929 Evalue:0

Accession:CP005975.1

Alignment

Pseudomonas fluorescens strain PCL1751, complete genome

Score:1929 Evalue:0

Accession:CP010896.1

Alignment

Pseudomonas simiae strain WCS417 genome

Score:1929 Evalue:0

Accession:CP007637.1

Alignment

Pseudomonas fluorescens strain PE8 16S ribosomal RNA ge..

Score:1929 Evalue:0

Accession:KJ420524.1

Alignment

Pseudomonas sp. isolate PS2.1 16S ribosomal RNA gene, p..

Score:1929 Evalue:0

Accession:KF019106.1

Alignment

Pseudomonas sp. ABAC21 16S ribosomal RNA gene, partial ..

Score:1929 Evalue:0

Accession:KF003385.1

Alignment

Pseudomonas fluorescens strain 20130311XA1 16S ribosoma..

Score:1929 Evalue:0

Accession:KC773764.1

Alignment

Pseudomonas poae RE\*1-1-14, complete genome

Score:1929 Evalue:0

Accession:CP004045.1

Alignment

Pseudomonas sp. SAP770.2 16S ribosomal RNA gene, partia..

Score:1929 Evalue:0

Accession:JX067683.1

Alignment

Pseudomonas poae strain zol-15 16S ribosomal RNA gene, ..

Score:1929 Evalue:0

Accession:JQ782898.1

Alignment

Pseudomonas sp. TK-3 gene for 16S rRNA, partial sequenc..

Score:1929 Evalue:0

Accession:AB705621.1

Alignment

uncultured bacterium partial 16S rRNA gene, clone K15.8..

Score:1929 Evalue:0

Accession:HE576021.1

Alignment

Pseudomonas poae strain ORC5 16S ribosomal RNA gene, pa..

Score:1929 Evalue:0

Accession:JQ236630.1

Alignment

Pseudomonas sp. PX7\_S1 16S ribosomal RNA gene, partial ..

Score:1929 Evalue:0

Accession:JF274937.1

Alignment

Pseudomonas sp. SGb192 16S ribosomal RNA gene, partial ..

Score:1929 Evalue:0

Accession:HQ224626.1

Alignment

Pseudomonas sp. SGb136 16S ribosomal RNA gene, partial ..

Score:1929 Evalue:0

Accession:HQ224624.1

Alignment

Pseudomonas sp. 5(2011) 16S ribosomal RNA gene, partial..

Score:1929 Evalue:0

Accession:JF808098.1

Alignment

Pseudomonas sp. YXE2-f 16S ribosomal RNA gene, partial ..

Score:1929 Evalue:0

Accession:JF701975.1

Alignment

Pseudomonas sp. PG-3-10 16S ribosomal RNA gene, partial..

Score:1929 Evalue:0

Accession:JF820107.1

Alignment

Uncultured bacterium clone EDW07B003\_41 16S ribosomal R..

Score:1929 Evalue:0

Accession:HM066455.1

Alignment

Uncultured Pseudomonas sp. clone CHINA4 16S ribosomal R..

Score:1929 Evalue:0

Accession:GU563752.1

Alignment

Pseudomonas sp. 231 16S ribosomal RNA gene, partial seq..

Score:1929 Evalue:0

Accession:GU213409.1

Alignment

Pseudomonas poae strain P 527/13 16S ribosomal RNA gene..

Score:1929 Evalue:0

Accession:NR\_028986.1

Alignment

Pseudomonas poae strain BIHB 808 16S ribosomal RNA gene..

Score:1929 Evalue:0

Accession:FJ179369.1

Alignment

Pseudomonas fluorescens strain 2312 16S ribosomal RNA g..

Score:1929 Evalue:0

Accession:EU360313.1

Alignment

Pseudomonas poae strain CN017 16S ribosomal RNA gene, p..

Score:1929 Evalue:0

Accession:EU364532.1

Alignment

Pseudomonas mandelii strain B61 16S ribosomal RNA gene,..

Score:1929 Evalue:0

Accession:EU169159.1

Alignment

Pseudomonas sp. CBCEN8 16S ribosomal RNA gene, partial ..

Score:1929 Evalue:0

Accession:EF427849.1

Alignment

Pseudomonas sp. CBMB39 16S ribosomal RNA gene, partial ..

Score:1929 Evalue:0

Accession:EF126756.1

Alignment

Pseudomonas sp. CBMB25 16S ribosomal RNA gene, partial ..

Score:1929 Evalue:0

Accession:EF126755.1

Alignment

Pseudomonas sp. CBMB18 16S ribosomal RNA gene, partial ..

Score:1929 Evalue:0

Accession:EF126753.1

Alignment

Pseudomonas poae strain BIHB 730 16S ribosomal RNA gene..

Score:1929 Evalue:0

Accession:DQ536513.1

Alignment

Pseudomonas poae partial 16S rRNA gene, type strain DSM..

Score:1929 Evalue:0

Accession:AJ492829.1

Alignment

Pseudomonas sp. strain KBL2 16S ribosomal RNA gene, par..

Score:1927 Evalue:0

Accession:MG576155.1

Alignment

Pseudomonas sp. BR2-02 16S ribosomal RNA gene, partial ..

Score:1927 Evalue:0

Accession:EU853182.1

Alignment

Pseudomonas sp. LBSA1 16S ribosomal RNA gene, partial s..

Score:1927 Evalue:0

Accession:AY756059.1

Alignment

Pseudomonas sp. strain 1-29 16S ribosomal RNA gene, par..

Score:1925 Evalue:0

Accession:KX378900.1

Alignment

Pseudomonas fluorescens strain SWFU21 16S ribosomal RNA..

Score:1925 Evalue:0

Accession:KJ756336.1

Alignment

Pseudomonas sp. BR2-22 16S ribosomal RNA gene, partial ..

Score:1925 Evalue:0

Accession:EU853183.1

Alignment

Pseudomonas sp. W15Feb3 16S ribosomal RNA gene, partial..

Score:1925 Evalue:0

Accession:EU680981.1

Alignment

Pseudomonas trivialis strain MAF3 16S ribosomal RNA gen..

Score:1923 Evalue:0

Accession:MF590113.1

Alignment

## Descriptions

, Reading indexes 1-5, displaying indexes 1-5


Load next setPrevious Match

Sequences producing significant alignments:

Show all columns  of the table presenting sequences producing significant alignments 

Select:AllNone
Selected:0

Alignments
Download

FASTA (complete sequence)

FASTA (aligned sequences)

GenBank (complete sequence)

Hit Table (text)

Hit Table (CSV)

Text

XML

ASN.1

Continue
Cancel

GenBank 
Graphics
Distance tree of results
Multiple alignment
Show/hide columns of the table presenting sequences producing significant alignments 

Available columns

Description  
Max Score  
Total Score  
Coverage  
E-value  
IdentN  
Accession  
Restore Defaults
Ok
Cancel

Sequences producing significant alignments:

| Select for downloading or viewing reports | Description | Max score | Total score | Query cover | E value | Ident | Accession |
| --- | --- | --- | --- | --- | --- | --- | --- |
| 1Select seq EU111704.2 | Pseudomonas poae strain T23 16S ribosomal RNA gene, complete sequence | 1938 | 1938 | 99% | 0.0 | 99% | EU111704.2 |
| 2Select seq LC140869.1 | Uncultured Pseudomonas sp. gene for 16S ribosomal RNA, partial sequence, clone: 89d159 | 1934 | 1934 | 98% | 0.0 | 99% | LC140869.1 |
| 3Select seq HQ403088.2 | Pseudomonas sp. 2-36(2010) 16S ribosomal RNA gene, partial sequence | 1932 | 1932 | 99% | 0.0 | 99% | HQ403088.2 |
| 4Select seq KJ756337.1 | Pseudomonas fluorescens strain SWFU22 16S ribosomal RNA gene, partial sequence | 1932 | 1932 | 99% | 0.0 | 99% | KJ756337.1 |
| 5Select seq HF545843.1 | Pseudomonas putida partial 16S rRNA gene, strain CFBP 5898 | 1932 | 1932 | 99% | 0.0 | 99% | HF545843.1 |
| 6Select seq FJ262366.1 | Pseudomonas sp. WH1 16S ribosomal RNA gene, partial sequence | 1932 | 1932 | 99% | 0.0 | 99% | FJ262366.1 |
| 7Select seq CP025263.1 | Pseudomonas sp. S09G 359 chromosome | 1929 | 11551 | 98% | 0.0 | 99% | CP025263.1 |
| 8Select seq KY810658.1 | Pseudomonas poae strain AAS-144 16S ribosomal RNA gene, partial sequence | 1929 | 1929 | 98% | 0.0 | 99% | KY810658.1 |
| 9Select seq LT629706.1 | Pseudomonas poae strain BS2776 genome assembly, chromosome: I | 1929 | 9645 | 98% | 0.0 | 99% | LT629706.1 |
| 10Select seq LT595870.1 | Uncultured bacterium partial 16S rRNA gene, clone clone AF-36051 | 1929 | 1929 | 98% | 0.0 | 99% | LT595870.1 |
| 11Select seq KX509247.1 | Uncultured bacterium clone MTWL201306-19 16S ribosomal RNA gene, partial sequence | 1929 | 1929 | 98% | 0.0 | 99% | KX509247.1 |
| 12Select seq KT825765.1 | Pseudomonas sp. 23K33a 16S ribosomal RNA gene, partial sequence | 1929 | 1929 | 98% | 0.0 | 99% | KT825765.1 |
| 13Select seq KT825736.1 | Pseudomonas sp. 17K8 16S ribosomal RNA gene, partial sequence | 1929 | 1929 | 98% | 0.0 | 99% | KT825736.1 |
| 14Select seq KT767705.1 | Pseudomonas poae strain B42 16S ribosomal RNA gene, partial sequence | 1929 | 1929 | 98% | 0.0 | 99% | KT767705.1 |
| 15Select seq KM187375.1 | Pseudomonas sp. HP10C 16S ribosomal RNA gene, partial sequence | 1929 | 1929 | 98% | 0.0 | 99% | KM187375.1 |
| 16Select seq CP005975.1 | Pseudomonas fluorescens PICF7, complete genome | 1929 | 11574 | 98% | 0.0 | 99% | CP005975.1 |
| 17Select seq CP010896.1 | Pseudomonas fluorescens strain PCL1751, complete genome | 1929 | 11557 | 98% | 0.0 | 99% | CP010896.1 |
| 18Select seq CP007637.1 | Pseudomonas simiae strain WCS417 genome | 1929 | 3858 | 98% | 0.0 | 99% | CP007637.1 |
| 19Select seq KJ420524.1 | Pseudomonas fluorescens strain PE8 16S ribosomal RNA gene, partial sequence | 1929 | 1929 | 98% | 0.0 | 99% | KJ420524.1 |
| 20Select seq KF019106.1 | Pseudomonas sp. isolate PS2.1 16S ribosomal RNA gene, partial sequence | 1929 | 1929 | 98% | 0.0 | 99% | KF019106.1 |
| 21Select seq KF003385.1 | Pseudomonas sp. ABAC21 16S ribosomal RNA gene, partial sequence | 1929 | 1929 | 98% | 0.0 | 99% | KF003385.1 |
| 22Select seq KC773764.1 | Pseudomonas fluorescens strain 20130311XA1 16S ribosomal RNA gene, partial sequence | 1929 | 1929 | 98% | 0.0 | 99% | KC773764.1 |
| 23Select seq CP004045.1 | Pseudomonas poae RE\*1-1-14, complete genome | 1929 | 9645 | 98% | 0.0 | 99% | CP004045.1 |
| 24Select seq JX067683.1 | Pseudomonas sp. SAP770.2 16S ribosomal RNA gene, partial sequence | 1929 | 1929 | 98% | 0.0 | 99% | JX067683.1 |
| 25Select seq JQ782898.1 | Pseudomonas poae strain zol-15 16S ribosomal RNA gene, partial sequence | 1929 | 1929 | 98% | 0.0 | 99% | JQ782898.1 |
| 26Select seq AB705621.1 | Pseudomonas sp. TK-3 gene for 16S rRNA, partial sequence | 1929 | 1929 | 98% | 0.0 | 99% | AB705621.1 |
| 27Select seq HE576021.1 | uncultured bacterium partial 16S rRNA gene, clone K15.87 KSS | 1929 | 1929 | 98% | 0.0 | 99% | HE576021.1 |
| 28Select seq JQ236630.1 | Pseudomonas poae strain ORC5 16S ribosomal RNA gene, partial sequence | 1929 | 1929 | 98% | 0.0 | 99% | JQ236630.1 |
| 29Select seq JF274937.1 | Pseudomonas sp. PX7\_S1 16S ribosomal RNA gene, partial sequence | 1929 | 1929 | 98% | 0.0 | 99% | JF274937.1 |
| 30Select seq HQ224626.1 | Pseudomonas sp. SGb192 16S ribosomal RNA gene, partial sequence | 1929 | 1929 | 98% | 0.0 | 99% | HQ224626.1 |
| 31Select seq HQ224624.1 | Pseudomonas sp. SGb136 16S ribosomal RNA gene, partial sequence | 1929 | 1929 | 98% | 0.0 | 99% | HQ224624.1 |
| 32Select seq JF808098.1 | Pseudomonas sp. 5(2011) 16S ribosomal RNA gene, partial sequence | 1929 | 1929 | 98% | 0.0 | 99% | JF808098.1 |
| 33Select seq JF701975.1 | Pseudomonas sp. YXE2-f 16S ribosomal RNA gene, partial sequence | 1929 | 1929 | 98% | 0.0 | 99% | JF701975.1 |
| 34Select seq JF820107.1 | Pseudomonas sp. PG-3-10 16S ribosomal RNA gene, partial sequence | 1929 | 1929 | 98% | 0.0 | 99% | JF820107.1 |
| 35Select seq HM066455.1 | Uncultured bacterium clone EDW07B003\_41 16S ribosomal RNA gene, partial sequence | 1929 | 1929 | 98% | 0.0 | 99% | HM066455.1 |
| 36Select seq GU563752.1 | Uncultured Pseudomonas sp. clone CHINA4 16S ribosomal RNA gene, partial sequence | 1929 | 1929 | 98% | 0.0 | 99% | GU563752.1 |
| 37Select seq GU213409.1 | Pseudomonas sp. 231 16S ribosomal RNA gene, partial sequence | 1929 | 1929 | 98% | 0.0 | 99% | GU213409.1 |
| 38Select seq NR\_028986.1 | Pseudomonas poae strain P 527/13 16S ribosomal RNA gene, partial sequence | 1929 | 1929 | 98% | 0.0 | 99% | NR\_028986.1 |
| 39Select seq FJ179369.1 | Pseudomonas poae strain BIHB 808 16S ribosomal RNA gene, partial sequence | 1929 | 1929 | 98% | 0.0 | 99% | FJ179369.1 |
| 40Select seq EU360313.1 | Pseudomonas fluorescens strain 2312 16S ribosomal RNA gene and 16S-23S ribosomal RNA intergenic spacer, complete sequence; and 23S ribosomal RNA gene, partial sequence | 1929 | 1929 | 98% | 0.0 | 99% | EU360313.1 |
| 41Select seq EU364532.1 | Pseudomonas poae strain CN017 16S ribosomal RNA gene, partial sequence | 1929 | 1929 | 98% | 0.0 | 99% | EU364532.1 |
| 42Select seq EU169159.1 | Pseudomonas mandelii strain B61 16S ribosomal RNA gene, partial sequence | 1929 | 1929 | 98% | 0.0 | 99% | EU169159.1 |
| 43Select seq EF427849.1 | Pseudomonas sp. CBCEN8 16S ribosomal RNA gene, partial sequence | 1929 | 1929 | 98% | 0.0 | 99% | EF427849.1 |
| 44Select seq EF126756.1 | Pseudomonas sp. CBMB39 16S ribosomal RNA gene, partial sequence | 1929 | 1929 | 98% | 0.0 | 99% | EF126756.1 |
| 45Select seq EF126755.1 | Pseudomonas sp. CBMB25 16S ribosomal RNA gene, partial sequence | 1929 | 1929 | 98% | 0.0 | 99% | EF126755.1 |
| 46Select seq EF126753.1 | Pseudomonas sp. CBMB18 16S ribosomal RNA gene, partial sequence | 1929 | 1929 | 98% | 0.0 | 99% | EF126753.1 |
| 47Select seq DQ536513.1 | Pseudomonas poae strain BIHB 730 16S ribosomal RNA gene, partial sequence | 1929 | 1929 | 98% | 0.0 | 99% | DQ536513.1 |
| 48Select seq AJ492829.1 | Pseudomonas poae partial 16S rRNA gene, type strain DSM 14936T | 1929 | 1929 | 98% | 0.0 | 99% | AJ492829.1 |
| 49Select seq MG576155.1 | Pseudomonas sp. strain KBL2 16S ribosomal RNA gene, partial sequence | 1927 | 1927 | 99% | 0.0 | 99% | MG576155.1 |
| 50Select seq EU853182.1 | Pseudomonas sp. BR2-02 16S ribosomal RNA gene, partial sequence | 1927 | 1927 | 98% | 0.0 | 99% | EU853182.1 |
| 51Select seq AY756059.1 | Pseudomonas sp. LBSA1 16S ribosomal RNA gene, partial sequence; internal transcribed spacer 1, complete sequence; and 23S ribosomal RNA gene, partial sequence | 1927 | 1927 | 98% | 0.0 | 99% | AY756059.1 |
| 52Select seq KX378900.1 | Pseudomonas sp. strain 1-29 16S ribosomal RNA gene, partial sequence | 1925 | 1925 | 98% | 0.0 | 99% | KX378900.1 |
| 53Select seq KJ756336.1 | Pseudomonas fluorescens strain SWFU21 16S ribosomal RNA gene, partial sequence | 1925 | 1925 | 98% | 0.0 | 99% | KJ756336.1 |
| 54Select seq EU853183.1 | Pseudomonas sp. BR2-22 16S ribosomal RNA gene, partial sequence | 1925 | 1925 | 98% | 0.0 | 99% | EU853183.1 |
| 55Select seq EU680981.1 | Pseudomonas sp. W15Feb3 16S ribosomal RNA gene, partial sequence | 1925 | 1925 | 98% | 0.0 | 99% | EU680981.1 |
| 56Select seq MF590113.1 | Pseudomonas trivialis strain MAF3 16S ribosomal RNA gene, partial sequence | 1923 | 1923 | 98% | 0.0 | 99% | MF590113.1 |
| 57Select seq KY940313.1 | Pseudomonas poae strain A2B5 16S ribosomal RNA gene, partial sequence | 1923 | 1923 | 98% | 0.0 | 99% | KY940313.1 |
| 58Select seq KX588598.1 | Pseudomonas poae strain ICE349 16S ribosomal RNA gene, partial sequence | 1923 | 1923 | 98% | 0.0 | 99% | KX588598.1 |
| 59Select seq KX588597.1 | Pseudomonas poae strain ICE259 16S ribosomal RNA gene, partial sequence | 1923 | 1923 | 98% | 0.0 | 99% | KX588597.1 |
| 60Select seq LT629760.1 | Pseudomonas trivialis strain BS3111 genome assembly, chromosome: I | 1923 | 9611 | 98% | 0.0 | 99% | LT629760.1 |
| 61Select seq KT710812.1 | Pseudomonas sp. RZ110 16S ribosomal RNA gene, partial sequence | 1923 | 1923 | 98% | 0.0 | 99% | KT710812.1 |
| 62Select seq KT825845.1 | Pseudomonas sp. 8K8 16S ribosomal RNA gene, partial sequence | 1923 | 1923 | 98% | 0.0 | 99% | KT825845.1 |
| 63Select seq KT825760.1 | Pseudomonas sp. 23K17 16S ribosomal RNA gene, partial sequence | 1923 | 1923 | 98% | 0.0 | 99% | KT825760.1 |
| 64Select seq KT424973.1 | Pseudomonas sp. LB8 16S ribosomal RNA gene, partial sequence | 1923 | 1923 | 98% | 0.0 | 99% | KT424973.1 |
| 65Select seq HG805781.1 | Pseudomonas sp. FBF110 partial 16S rRNA gene, isolate FBF110 | 1923 | 1923 | 98% | 0.0 | 99% | HG805781.1 |
| 66Select seq LN875096.1 | Uncultured Pseudomonas sp. partial 16S rRNA gene, clone 133X76C\_34 | 1923 | 1923 | 98% | 0.0 | 99% | LN875096.1 |
| 67Select seq KR085862.1 | Pseudomonas poae strain IHBB 11151 16S ribosomal RNA gene, partial sequence | 1923 | 1923 | 98% | 0.0 | 99% | KR085862.1 |
| 68Select seq KR085822.1 | Pseudomonas poae strain IHBB 9211 16S ribosomal RNA gene, partial sequence | 1923 | 1923 | 98% | 0.0 | 99% | KR085822.1 |
| 69Select seq KP975307.1 | Uncultured bacterium clone OTU51 16S ribosomal RNA gene, partial sequence | 1923 | 1923 | 98% | 0.0 | 99% | KP975307.1 |
| 70Select seq KT029390.1 | Uncultured bacterium clone 19-26 16S ribosomal RNA gene, partial sequence | 1923 | 1923 | 98% | 0.0 | 99% | KT029390.1 |
| 71Select seq KT029380.1 | Uncultured bacterium clone 19-16 16S ribosomal RNA gene, partial sequence | 1923 | 1923 | 98% | 0.0 | 99% | KT029380.1 |
| 72Select seq KT029378.1 | Uncultured bacterium clone 19-14 16S ribosomal RNA gene, partial sequence | 1923 | 1923 | 98% | 0.0 | 99% | KT029378.1 |
| 73Select seq CP011507.1 | Pseudomonas trivialis strain IHBB745, complete genome | 1923 | 9617 | 98% | 0.0 | 99% | CP011507.1 |
| 74Select seq KP204140.1 | Pseudomonas poae strain NA\_1 16S ribosomal RNA gene, partial sequence | 1923 | 1923 | 98% | 0.0 | 99% | KP204140.1 |
| 75Select seq KM589026.1 | Pseudomonas poae strain Pf9 16S ribosomal RNA gene, partial sequence | 1923 | 1923 | 98% | 0.0 | 99% | KM589026.1 |
| 76Select seq KM461107.1 | Pseudomonas sp. WHAS15 16S ribosomal RNA gene, partial sequence | 1923 | 1923 | 98% | 0.0 | 99% | KM461107.1 |
| 77Select seq KJ848593.1 | Pseudomonas poae strain BT 89 16S ribosomal RNA gene, partial sequence | 1923 | 1923 | 98% | 0.0 | 99% | KJ848593.1 |
| 78Select seq KJ420526.1 | Pseudomonas fluorescens strain PE16 16S ribosomal RNA gene, partial sequence | 1923 | 1923 | 98% | 0.0 | 99% | KJ420526.1 |
| 79Select seq KJ127239.1 | Pseudomonas fluorescens strain PE3 16S ribosomal RNA gene, partial sequence | 1923 | 1923 | 98% | 0.0 | 99% | KJ127239.1 |
| 80Select seq KC618443.1 | Pseudomonas mandelii 16S ribosomal RNA gene, partial sequence | 1923 | 1923 | 98% | 0.0 | 99% | KC618443.1 |
| 81Select seq JQ977483.1 | Pseudomonas sp. Bma14 16S ribosomal RNA gene, partial sequence | 1923 | 1923 | 98% | 0.0 | 99% | JQ977483.1 |
| 82Select seq JX840381.1 | Pseudomonas salomonii strain CCNWQLS112 16S ribosomal RNA gene, partial sequence | 1923 | 1923 | 98% | 0.0 | 99% | JX840381.1 |
| 83Select seq JX949554.1 | Pseudomonas sp. MDT1-21 16S ribosomal RNA gene, partial sequence | 1923 | 1923 | 98% | 0.0 | 99% | JX949554.1 |
| 84Select seq JX515573.1 | Pseudomonas poae strain EB20 16S ribosomal RNA gene, partial sequence | 1923 | 1923 | 98% | 0.0 | 99% | JX515573.1 |
| 85Select seq JQ782893.1 | Pseudomonas poae strain Car-06 16S ribosomal RNA gene, partial sequence | 1923 | 1923 | 98% | 0.0 | 99% | JQ782893.1 |
| 86Select seq JQ638271.1 | Pseudomonas sp. ZBC1 16S ribosomal RNA gene, partial sequence | 1923 | 1923 | 98% | 0.0 | 99% | JQ638271.1 |
| 87Select seq AB680977.1 | Pseudomonas fluorescens gene for 16S rRNA, partial sequence, strain: NBRC 15838 | 1923 | 1923 | 98% | 0.0 | 99% | AB680977.1 |
| 88Select seq JN377670.1 | Pseudomonas trivialis strain B24 16S ribosomal RNA gene, partial sequence | 1923 | 1923 | 98% | 0.0 | 99% | JN377670.1 |
| 89Select seq JN038283.1 | Uncultured Pseudomonas sp. clone EK\_An818 16S ribosomal RNA gene, partial sequence | 1923 | 1923 | 98% | 0.0 | 99% | JN038283.1 |
| 90Select seq JN033052.1 | Uncultured bacterium clone 3-42017 16S ribosomal RNA gene, partial sequence | 1923 | 1923 | 98% | 0.0 | 99% | JN033052.1 |
| 91Select seq HQ647258.1 | Pseudomonas poae strain P6 16S ribosomal RNA gene, partial sequence | 1923 | 1923 | 98% | 0.0 | 99% | HQ647258.1 |
| 92Select seq JF703597.1 | Uncultured bacterium clone GcG58 16S ribosomal RNA gene, partial sequence | 1923 | 1923 | 98% | 0.0 | 99% | JF703597.1 |
| 93Select seq HM448986.1 | Pseudomonas sp. DPs-8 16S ribosomal RNA gene, partial sequence | 1923 | 1923 | 98% | 0.0 | 99% | HM448986.1 |
| 94Select seq HM066471.1 | Uncultured bacterium clone EDW07B003\_70 16S ribosomal RNA gene, partial sequence | 1923 | 1923 | 98% | 0.0 | 99% | HM066471.1 |
| 95Select seq HM066261.1 | Uncultured bacterium clone EDW07B001\_111 16S ribosomal RNA gene, partial sequence | 1923 | 1923 | 98% | 0.0 | 99% | HM066261.1 |
| 96Select seq GU188947.1 | Pseudomonas poae strain BCHCNZ253 16S ribosomal RNA gene, partial sequence | 1923 | 1923 | 98% | 0.0 | 99% | GU188947.1 |
| 97Select seq GU198111.1 | Pseudomonas fluorescens strain LMG 5833 16S ribosomal RNA gene, partial sequence | 1923 | 1923 | 98% | 0.0 | 99% | GU198111.1 |
| 98Select seq NR\_028987.1 | Pseudomonas trivialis strain P 513/19 16S ribosomal RNA gene, partial sequence | 1923 | 1923 | 98% | 0.0 | 99% | NR\_028987.1 |
| 99Select seq GQ868355.1 | Pseudomonas sp. BBI9 16S ribosomal RNA gene, partial sequence | 1923 | 1923 | 98% | 0.0 | 99% | GQ868355.1 |
| 100Select seq FJ999933.1 | Pseudomonas trivialis strain EQH4 16S ribosomal RNA gene, partial sequence | 1923 | 1923 | 98% | 0.0 | 99% | FJ999933.1 |


## Alignments

Loading alignment... for sequences gi|223890418,gi|1024846463,gi|752303983,gi|647082308,gi|515782672 Reading indexes 1-5

Download

FASTA (complete sequence)

FASTA (aligned sequences)

GenBank (complete sequence)

Continue
Cancel

GenBankGraphics

Next
Previous
Descriptions

Pseudomonas poae strain T23 16S ribosomal RNA gene, complete sequence

Sequence ID: EU111704.2Length: 1423Number of Matches: 1

Related Information

Range 1: 366 to 1422GenBankGraphics

Next Match
Previous Match
First Match

Alignment statistics for match #1

| Score | Expect | Identities | Gaps | Strand | Frame |
| --- | --- | --- | --- | --- | --- |
| 1938 bits(1049) | 0.0() | 1053/1057(99%) | 0/1057(0%) | Plus/Minus |  |

Features:

```
Query  5     CGTGNNACCGTCCTCCCGAAGGTTAGACTAGCTACTTCTGGTGCAACCCACTCCCATGGT  64
             ||||  ||||||||||||||||||||||||||||||||||||||||||||||||||||||
Sbjct  1422  CGTGGTACCGTCCTCCCGAAGGTTAGACTAGCTACTTCTGGTGCAACCCACTCCCATGGT  1363

Query  65    GTGACGGGCGGTGTGTACAAGGCCCGGGAACGTATTCACCGCGACATTCTGATTCGCGAT  124
             ||||||||||||||||||||||||||||||||||||||||||||||||||||||||||||
Sbjct  1362  GTGACGGGCGGTGTGTACAAGGCCCGGGAACGTATTCACCGCGACATTCTGATTCGCGAT  1303

Query  125   TACTAGCGATTCCGACTTCACGCAGTCGAGTTGCAGACTGCGATCCGGACTACGATCGGT  184
             ||||||||||||||||||||||||||||||||||||||||||||||||||||||||||||
Sbjct  1302  TACTAGCGATTCCGACTTCACGCAGTCGAGTTGCAGACTGCGATCCGGACTACGATCGGT  1243

Query  185   TTTATGGGATTAGCTCCACCTCGCGGCTTGGCAACCCTCTGTACCGACCATTGTAGCACG  244
             ||||||||||||||||||||||||||||||||||||||||||||||||||||||||||||
Sbjct  1242  TTTATGGGATTAGCTCCACCTCGCGGCTTGGCAACCCTCTGTACCGACCATTGTAGCACG  1183

Query  245   TGTGTAGCCCAGGCCGTAAGGGCCATGATGACTTGACGTCATCCCCACCTTCCTCCGGTT  304
             ||||||||||||||||||||||||||||||||||||||||||||||||||||||||||||
Sbjct  1182  TGTGTAGCCCAGGCCGTAAGGGCCATGATGACTTGACGTCATCCCCACCTTCCTCCGGTT  1123

Query  305   TGTCACCGGCAGTCTCCTTAGAGTGCCCACCATCACGTGCTGGTAACTAAGGACAAGGGT  364
             ||||||||||||||||||||||||||||||||||||||||||||||||||||||||||||
Sbjct  1122  TGTCACCGGCAGTCTCCTTAGAGTGCCCACCATCACGTGCTGGTAACTAAGGACAAGGGT  1063

Query  365   TGCGCTCGTTACGGGACTTAACCCAACATCTCACGACACGAGCTGACGACAGCCATGCAG  424
             ||||||||||||||||||||||||||||||||||||||||||||||||||||||||||||
Sbjct  1062  TGCGCTCGTTACGGGACTTAACCCAACATCTCACGACACGAGCTGACGACAGCCATGCAG  1003

Query  425   CACCTGTCTCAATGTTCCCGAAGGCACCAATCTATCTCTAGAAAGTTCATTGGATGTCAA  484
             ||||||||||||||||||||||||||||||||||||||||||||||||||||||||||||
Sbjct  1002  CACCTGTCTCAATGTTCCCGAAGGCACCAATCTATCTCTAGAAAGTTCATTGGATGTCAA  943

Query  485   GGCCTGGTAAGGTTCTTCGCGTTGCTTCGAATTAAACCACATGCTCCACCGCTTGTGCGG  544
             ||||||||||||||||||||||||||||||||||||||||||||||||||||||||||||
Sbjct  942   GGCCTGGTAAGGTTCTTCGCGTTGCTTCGAATTAAACCACATGCTCCACCGCTTGTGCGG  883

Query  545   GCCCCCGTCAATTCATTTGAGTTTTAACCTTGCGGCCGTACTCCCCAGGCGGTCAACTTA  604
             ||||||||||||||||||||||||||||||||||||||||||||||||||||||||||||
Sbjct  882   GCCCCCGTCAATTCATTTGAGTTTTAACCTTGCGGCCGTACTCCCCAGGCGGTCAACTTA  823

Query  605   ATGCGTTAGCTGCGCCACTAAAAGCTCAAGGCTTCCAACGGCTAGTTGACATCGTTTACG  664
             ||||||||||||||||||||||||||||||||||||||||||||||||||||||||||||
Sbjct  822   ATGCGTTAGCTGCGCCACTAAAAGCTCAAGGCTTCCAACGGCTAGTTGACATCGTTTACG  763

Query  665   GCGTGGACTACCAGGGTATCTAATCCTGTTTGCTCCCCACGCTTTCGCACCTCAGTGTCA  724
             ||||||||||||||||||||||||||||||||||||||||||||||||||||||||||||
Sbjct  762   GCGTGGACTACCAGGGTATCTAATCCTGTTTGCTCCCCACGCTTTCGCACCTCAGTGTCA  703

Query  725   GTATTAGTCCAGGTGGTCGCCTTCGCCACTGGTGTTCCTTCCTATATCTACGCATTTCAC  784
             ||||||||||||||||||||||||||||||||||||||||||||||||||||||||||||
Sbjct  702   GTATTAGTCCAGGTGGTCGCCTTCGCCACTGGTGTTCCTTCCTATATCTACGCATTTCAC  643

Query  785   CGCTACACAGGAAATTCCACCACCCTCTACCATACTCTAGTCAGTCAGTTTTGAATGCAG  844
             ||||||||||||||||||||||||||||||||||||||||||||||||||||||||||||
Sbjct  642   CGCTACACAGGAAATTCCACCACCCTCTACCATACTCTAGTCAGTCAGTTTTGAATGCAG  583

Query  845   TTCCCAGGTTGAGCCCGGGGATTTCACATCCAACTTAACAAACCACCTACGCGCGCTTTA  904
             ||||||||||||||||||||||||||||||||||||||||||||||||||||||||||||
Sbjct  582   TTCCCAGGTTGAGCCCGGGGATTTCACATCCAACTTAACAAACCACCTACGCGCGCTTTA  523

Query  905   CGCCCAGTAATTCCGATTAACGCTTGCACCCTCTGTATTACCGCGGCTGCTGGCACAGAG  964
             ||||||||||||||||||||||||||||||||||||||||||||||||||||||||||||
Sbjct  522   CGCCCAGTAATTCCGATTAACGCTTGCACCCTCTGTATTACCGCGGCTGCTGGCACAGAG  463

Query  965   TTAGCCGGTGCTTATTCTGTCGGTAACGTCAAANCAATCACGTATTAGGTAACTGCCCTT  1024
             ||||||||||||||||||||||||||||||||| ||||||||||||||||||||||||||
Sbjct  462   TTAGCCGGTGCTTATTCTGTCGGTAACGTCAAAACAATCACGTATTAGGTAACTGCCCTT  403

Query  1025  CCTCCCAACTTAAAGTGCTTTACAATCCGAAGNCCTT  1061
             |||||||||||||||||||||||||||||||| ||||
Sbjct  402   CCTCCCAACTTAAAGTGCTTTACAATCCGAAGACCTT  366
```

Download

FASTA (complete sequence)

FASTA (aligned sequences)

GenBank (complete sequence)

Continue
Cancel

GenBankGraphics

Next
Previous
Descriptions

Uncultured Pseudomonas sp. gene for 16S ribosomal RNA, partial sequence, clone: 89d159

Sequence ID: LC140869.1Length: 1526Number of Matches: 1

Related Information

Range 1: 429 to 1479GenBankGraphics

Next Match
Previous Match
First Match

Alignment statistics for match #1

| Score | Expect | Identities | Gaps | Strand | Frame |
| --- | --- | --- | --- | --- | --- |
| 1934 bits(1047) | 0.0() | 1049/1051(99%) | 0/1051(0%) | Plus/Minus |  |

Features:

```
Query  11    ACCGTCCTCCCGAAGGTTAGACTAGCTACTTCTGGTGCAACCCACTCCCATGGTGTGACG  70
             ||||||||||||||||||||||||||||||||||||||||||||||||||||||||||||
Sbjct  1479  ACCGTCCTCCCGAAGGTTAGACTAGCTACTTCTGGTGCAACCCACTCCCATGGTGTGACG  1420

Query  71    GGCGGTGTGTACAAGGCCCGGGAACGTATTCACCGCGACATTCTGATTCGCGATTACTAG  130
             ||||||||||||||||||||||||||||||||||||||||||||||||||||||||||||
Sbjct  1419  GGCGGTGTGTACAAGGCCCGGGAACGTATTCACCGCGACATTCTGATTCGCGATTACTAG  1360

Query  131   CGATTCCGACTTCACGCAGTCGAGTTGCAGACTGCGATCCGGACTACGATCGGTTTTATG  190
             ||||||||||||||||||||||||||||||||||||||||||||||||||||||||||||
Sbjct  1359  CGATTCCGACTTCACGCAGTCGAGTTGCAGACTGCGATCCGGACTACGATCGGTTTTATG  1300

Query  191   GGATTAGCTCCACCTCGCGGCTTGGCAACCCTCTGTACCGACCATTGTAGCACGTGTGTA  250
             ||||||||||||||||||||||||||||||||||||||||||||||||||||||||||||
Sbjct  1299  GGATTAGCTCCACCTCGCGGCTTGGCAACCCTCTGTACCGACCATTGTAGCACGTGTGTA  1240

Query  251   GCCCAGGCCGTAAGGGCCATGATGACTTGACGTCATCCCCACCTTCCTCCGGTTTGTCAC  310
             ||||||||||||||||||||||||||||||||||||||||||||||||||||||||||||
Sbjct  1239  GCCCAGGCCGTAAGGGCCATGATGACTTGACGTCATCCCCACCTTCCTCCGGTTTGTCAC  1180

Query  311   CGGCAGTCTCCTTAGAGTGCCCACCATCACGTGCTGGTAACTAAGGACAAGGGTTGCGCT  370
             ||||||||||||||||||||||||||||||||||||||||||||||||||||||||||||
Sbjct  1179  CGGCAGTCTCCTTAGAGTGCCCACCATCACGTGCTGGTAACTAAGGACAAGGGTTGCGCT  1120

Query  371   CGTTACGGGACTTAACCCAACATCTCACGACACGAGCTGACGACAGCCATGCAGCACCTG  430
             ||||||||||||||||||||||||||||||||||||||||||||||||||||||||||||
Sbjct  1119  CGTTACGGGACTTAACCCAACATCTCACGACACGAGCTGACGACAGCCATGCAGCACCTG  1060

Query  431   TCTCAATGTTCCCGAAGGCACCAATCTATCTCTAGAAAGTTCATTGGATGTCAAGGCCTG  490
             ||||||||||||||||||||||||||||||||||||||||||||||||||||||||||||
Sbjct  1059  TCTCAATGTTCCCGAAGGCACCAATCTATCTCTAGAAAGTTCATTGGATGTCAAGGCCTG  1000

Query  491   GTAAGGTTCTTCGCGTTGCTTCGAATTAAACCACATGCTCCACCGCTTGTGCGGGCCCCC  550
             ||||||||||||||||||||||||||||||||||||||||||||||||||||||||||||
Sbjct  999   GTAAGGTTCTTCGCGTTGCTTCGAATTAAACCACATGCTCCACCGCTTGTGCGGGCCCCC  940

Query  551   GTCAATTCATTTGAGTTTTAACCTTGCGGCCGTACTCCCCAGGCGGTCAACTTAATGCGT  610
             ||||||||||||||||||||||||||||||||||||||||||||||||||||||||||||
Sbjct  939   GTCAATTCATTTGAGTTTTAACCTTGCGGCCGTACTCCCCAGGCGGTCAACTTAATGCGT  880

Query  611   TAGCTGCGCCACTAAAAGCTCAAGGCTTCCAACGGCTAGTTGACATCGTTTACGGCGTGG  670
             ||||||||||||||||||||||||||||||||||||||||||||||||||||||||||||
Sbjct  879   TAGCTGCGCCACTAAAAGCTCAAGGCTTCCAACGGCTAGTTGACATCGTTTACGGCGTGG  820

Query  671   ACTACCAGGGTATCTAATCCTGTTTGCTCCCCACGCTTTCGCACCTCAGTGTCAGTATTA  730
             ||||||||||||||||||||||||||||||||||||||||||||||||||||||||||||
Sbjct  819   ACTACCAGGGTATCTAATCCTGTTTGCTCCCCACGCTTTCGCACCTCAGTGTCAGTATTA  760

Query  731   GTCCAGGTGGTCGCCTTCGCCACTGGTGTTCCTTCCTATATCTACGCATTTCACCGCTAC  790
             ||||||||||||||||||||||||||||||||||||||||||||||||||||||||||||
Sbjct  759   GTCCAGGTGGTCGCCTTCGCCACTGGTGTTCCTTCCTATATCTACGCATTTCACCGCTAC  700

Query  791   ACAGGAAATTCCACCACCCTCTACCATACTCTAGTCAGTCAGTTTTGAATGCAGTTCCCA  850
             ||||||||||||||||||||||||||||||||||||||||||||||||||||||||||||
Sbjct  699   ACAGGAAATTCCACCACCCTCTACCATACTCTAGTCAGTCAGTTTTGAATGCAGTTCCCA  640

Query  851   GGTTGAGCCCGGGGATTTCACATCCAACTTAACAAACCACCTACGCGCGCTTTACGCCCA  910
             ||||||||||||||||||||||||||||||||||||||||||||||||||||||||||||
Sbjct  639   GGTTGAGCCCGGGGATTTCACATCCAACTTAACAAACCACCTACGCGCGCTTTACGCCCA  580

Query  911   GTAATTCCGATTAACGCTTGCACCCTCTGTATTACCGCGGCTGCTGGCACAGAGTTAGCC  970
             ||||||||||||||||||||||||||||||||||||||||||||||||||||||||||||
Sbjct  579   GTAATTCCGATTAACGCTTGCACCCTCTGTATTACCGCGGCTGCTGGCACAGAGTTAGCC  520

Query  971   GGTGCTTATTCTGTCGGTAACGTCAAANCAATCACGTATTAGGTAACTGCCCTTCCTCCC  1030
             ||||||||||||||||||||||||||| ||||||||||||||||||||||||||||||||
Sbjct  519   GGTGCTTATTCTGTCGGTAACGTCAAAACAATCACGTATTAGGTAACTGCCCTTCCTCCC  460

Query  1031  AACTTAAAGTGCTTTACAATCCGAAGNCCTT  1061
             |||||||||||||||||||||||||| ||||
Sbjct  459   AACTTAAAGTGCTTTACAATCCGAAGACCTT  429
```

Download

FASTA (complete sequence)

FASTA (aligned sequences)

GenBank (complete sequence)

Continue
Cancel

GenBankGraphics

Next
Previous
Descriptions

Pseudomonas sp. 2-36(2010) 16S ribosomal RNA gene, partial sequence

Sequence ID: HQ403088.2Length: 1410Number of Matches: 1

Related Information

Range 1: 352 to 1408GenBankGraphics

Next Match
Previous Match
First Match

Alignment statistics for match #1

| Score | Expect | Identities | Gaps | Strand | Frame |
| --- | --- | --- | --- | --- | --- |
| 1932 bits(1046) | 0.0() | 1052/1057(99%) | 0/1057(0%) | Plus/Minus |  |

Features:

```
Query  5     CGTGNNACCGTCCTCCCGAAGGTTAGACTAGCTACTTCTGGTGCAACCCACTCCCATGGT  64
             ||||  ||||||||||||||||||||||||||||||||||||||||||||||||||||||
Sbjct  1408  CGTGGTACCGTCCTCCCGAAGGTTAGACTAGCTACTTCTGGTGCAACCCACTCCCATGGT  1349

Query  65    GTGACGGGCGGTGTGTACAAGGCCCGGGAACGTATTCACCGCGACATTCTGATTCGCGAT  124
             ||||||||||||||||||||||||||||||||||||||||||||||||||||||||||||
Sbjct  1348  GTGACGGGCGGTGTGTACAAGGCCCGGGAACGTATTCACCGCGACATTCTGATTCGCGAT  1289

Query  125   TACTAGCGATTCCGACTTCACGCAGTCGAGTTGCAGACTGCGATCCGGACTACGATCGGT  184
             ||||||||||||||||||||||||||||||||||||||||||||||||||||||||||||
Sbjct  1288  TACTAGCGATTCCGACTTCACGCAGTCGAGTTGCAGACTGCGATCCGGACTACGATCGGT  1229

Query  185   TTTATGGGATTAGCTCCACCTCGCGGCTTGGCAACCCTCTGTACCGACCATTGTAGCACG  244
             ||||||||||||||||||||||||||||||||||||||||||||||||||||||||||||
Sbjct  1228  TTTATGGGATTAGCTCCACCTCGCGGCTTGGCAACCCTCTGTACCGACCATTGTAGCACG  1169

Query  245   TGTGTAGCCCAGGCCGTAAGGGCCATGATGACTTGACGTCATCCCCACCTTCCTCCGGTT  304
             ||||||||||||||||||||||||||||||||||||||||||||||||||||||||||||
Sbjct  1168  TGTGTAGCCCAGGCCGTAAGGGCCATGATGACTTGACGTCATCCCCACCTTCCTCCGGTT  1109

Query  305   TGTCACCGGCAGTCTCCTTAGAGTGCCCACCATCACGTGCTGGTAACTAAGGACAAGGGT  364
             ||||||||||||||||||||||||||||||||| ||||||||||||||||||||||||||
Sbjct  1108  TGTCACCGGCAGTCTCCTTAGAGTGCCCACCATTACGTGCTGGTAACTAAGGACAAGGGT  1049

Query  365   TGCGCTCGTTACGGGACTTAACCCAACATCTCACGACACGAGCTGACGACAGCCATGCAG  424
             ||||||||||||||||||||||||||||||||||||||||||||||||||||||||||||
Sbjct  1048  TGCGCTCGTTACGGGACTTAACCCAACATCTCACGACACGAGCTGACGACAGCCATGCAG  989

Query  425   CACCTGTCTCAATGTTCCCGAAGGCACCAATCTATCTCTAGAAAGTTCATTGGATGTCAA  484
             ||||||||||||||||||||||||||||||||||||||||||||||||||||||||||||
Sbjct  988   CACCTGTCTCAATGTTCCCGAAGGCACCAATCTATCTCTAGAAAGTTCATTGGATGTCAA  929

Query  485   GGCCTGGTAAGGTTCTTCGCGTTGCTTCGAATTAAACCACATGCTCCACCGCTTGTGCGG  544
             ||||||||||||||||||||||||||||||||||||||||||||||||||||||||||||
Sbjct  928   GGCCTGGTAAGGTTCTTCGCGTTGCTTCGAATTAAACCACATGCTCCACCGCTTGTGCGG  869

Query  545   GCCCCCGTCAATTCATTTGAGTTTTAACCTTGCGGCCGTACTCCCCAGGCGGTCAACTTA  604
             ||||||||||||||||||||||||||||||||||||||||||||||||||||||||||||
Sbjct  868   GCCCCCGTCAATTCATTTGAGTTTTAACCTTGCGGCCGTACTCCCCAGGCGGTCAACTTA  809

Query  605   ATGCGTTAGCTGCGCCACTAAAAGCTCAAGGCTTCCAACGGCTAGTTGACATCGTTTACG  664
             ||||||||||||||||||||||||||||||||||||||||||||||||||||||||||||
Sbjct  808   ATGCGTTAGCTGCGCCACTAAAAGCTCAAGGCTTCCAACGGCTAGTTGACATCGTTTACG  749

Query  665   GCGTGGACTACCAGGGTATCTAATCCTGTTTGCTCCCCACGCTTTCGCACCTCAGTGTCA  724
             ||||||||||||||||||||||||||||||||||||||||||||||||||||||||||||
Sbjct  748   GCGTGGACTACCAGGGTATCTAATCCTGTTTGCTCCCCACGCTTTCGCACCTCAGTGTCA  689

Query  725   GTATTAGTCCAGGTGGTCGCCTTCGCCACTGGTGTTCCTTCCTATATCTACGCATTTCAC  784
             ||||||||||||||||||||||||||||||||||||||||||||||||||||||||||||
Sbjct  688   GTATTAGTCCAGGTGGTCGCCTTCGCCACTGGTGTTCCTTCCTATATCTACGCATTTCAC  629

Query  785   CGCTACACAGGAAATTCCACCACCCTCTACCATACTCTAGTCAGTCAGTTTTGAATGCAG  844
             ||||||||||||||||||||||||||||||||||||||||||||||||||||||||||||
Sbjct  628   CGCTACACAGGAAATTCCACCACCCTCTACCATACTCTAGTCAGTCAGTTTTGAATGCAG  569

Query  845   TTCCCAGGTTGAGCCCGGGGATTTCACATCCAACTTAACAAACCACCTACGCGCGCTTTA  904
             ||||||||||||||||||||||||||||||||||||||||||||||||||||||||||||
Sbjct  568   TTCCCAGGTTGAGCCCGGGGATTTCACATCCAACTTAACAAACCACCTACGCGCGCTTTA  509

Query  905   CGCCCAGTAATTCCGATTAACGCTTGCACCCTCTGTATTACCGCGGCTGCTGGCACAGAG  964
             ||||||||||||||||||||||||||||||||||||||||||||||||||||||||||||
Sbjct  508   CGCCCAGTAATTCCGATTAACGCTTGCACCCTCTGTATTACCGCGGCTGCTGGCACAGAG  449

Query  965   TTAGCCGGTGCTTATTCTGTCGGTAACGTCAAANCAATCACGTATTAGGTAACTGCCCTT  1024
             ||||||||||||||||||||||||||||||||| ||||||||||||||||||||||||||
Sbjct  448   TTAGCCGGTGCTTATTCTGTCGGTAACGTCAAAACAATCACGTATTAGGTAACTGCCCTT  389

Query  1025  CCTCCCAACTTAAAGTGCTTTACAATCCGAAGNCCTT  1061
             |||||||||||||||||||||||||||||||| ||||
Sbjct  388   CCTCCCAACTTAAAGTGCTTTACAATCCGAAGACCTT  352
```

Download

FASTA (complete sequence)

FASTA (aligned sequences)

GenBank (complete sequence)

Continue
Cancel

GenBankGraphics

Next
Previous
Descriptions

Pseudomonas fluorescens strain SWFU22 16S ribosomal RNA gene, partial sequence

Sequence ID: KJ756337.1Length: 1435Number of Matches: 1

Related Information

Range 1: 369 to 1425GenBankGraphics

Next Match
Previous Match
First Match

Alignment statistics for match #1

| Score | Expect | Identities | Gaps | Strand | Frame |
| --- | --- | --- | --- | --- | --- |
| 1932 bits(1046) | 0.0() | 1052/1057(99%) | 0/1057(0%) | Plus/Minus |  |

Features:

```
Query  5     CGTGNNACCGTCCTCCCGAAGGTTAGACTAGCTACTTCTGGTGCAACCCACTCCCATGGT  64
             ||||  ||||||||||||||||||||||||||||||||||||||||||||||||||||||
Sbjct  1425  CGTGGTACCGTCCTCCCGAAGGTTAGACTAGCTACTTCTGGTGCAACCCACTCCCATGGT  1366

Query  65    GTGACGGGCGGTGTGTACAAGGCCCGGGAACGTATTCACCGCGACATTCTGATTCGCGAT  124
             ||||||||||||||||||||||||||||||||||||||||||||||||||||||||||||
Sbjct  1365  GTGACGGGCGGTGTGTACAAGGCCCGGGAACGTATTCACCGCGACATTCTGATTCGCGAT  1306

Query  125   TACTAGCGATTCCGACTTCACGCAGTCGAGTTGCAGACTGCGATCCGGACTACGATCGGT  184
             ||||||||||||||||||||||||||||||||||||||||||||||||||||||||||||
Sbjct  1305  TACTAGCGATTCCGACTTCACGCAGTCGAGTTGCAGACTGCGATCCGGACTACGATCGGT  1246

Query  185   TTTATGGGATTAGCTCCACCTCGCGGCTTGGCAACCCTCTGTACCGACCATTGTAGCACG  244
             ||||||||||||||||||||||||||||||||||||||||||||||||||||||||||||
Sbjct  1245  TTTATGGGATTAGCTCCACCTCGCGGCTTGGCAACCCTCTGTACCGACCATTGTAGCACG  1186

Query  245   TGTGTAGCCCAGGCCGTAAGGGCCATGATGACTTGACGTCATCCCCACCTTCCTCCGGTT  304
             ||||||||||||||||||||||||||||||||||||||||||||||||||||||||||||
Sbjct  1185  TGTGTAGCCCAGGCCGTAAGGGCCATGATGACTTGACGTCATCCCCACCTTCCTCCGGTT  1126

Query  305   TGTCACCGGCAGTCTCCTTAGAGTGCCCACCATCACGTGCTGGTAACTAAGGACAAGGGT  364
             ||||||||||||||||||||||||||||||||| ||||||||||||||||||||||||||
Sbjct  1125  TGTCACCGGCAGTCTCCTTAGAGTGCCCACCATTACGTGCTGGTAACTAAGGACAAGGGT  1066

Query  365   TGCGCTCGTTACGGGACTTAACCCAACATCTCACGACACGAGCTGACGACAGCCATGCAG  424
             ||||||||||||||||||||||||||||||||||||||||||||||||||||||||||||
Sbjct  1065  TGCGCTCGTTACGGGACTTAACCCAACATCTCACGACACGAGCTGACGACAGCCATGCAG  1006

Query  425   CACCTGTCTCAATGTTCCCGAAGGCACCAATCTATCTCTAGAAAGTTCATTGGATGTCAA  484
             ||||||||||||||||||||||||||||||||||||||||||||||||||||||||||||
Sbjct  1005  CACCTGTCTCAATGTTCCCGAAGGCACCAATCTATCTCTAGAAAGTTCATTGGATGTCAA  946

Query  485   GGCCTGGTAAGGTTCTTCGCGTTGCTTCGAATTAAACCACATGCTCCACCGCTTGTGCGG  544
             ||||||||||||||||||||||||||||||||||||||||||||||||||||||||||||
Sbjct  945   GGCCTGGTAAGGTTCTTCGCGTTGCTTCGAATTAAACCACATGCTCCACCGCTTGTGCGG  886

Query  545   GCCCCCGTCAATTCATTTGAGTTTTAACCTTGCGGCCGTACTCCCCAGGCGGTCAACTTA  604
             ||||||||||||||||||||||||||||||||||||||||||||||||||||||||||||
Sbjct  885   GCCCCCGTCAATTCATTTGAGTTTTAACCTTGCGGCCGTACTCCCCAGGCGGTCAACTTA  826

Query  605   ATGCGTTAGCTGCGCCACTAAAAGCTCAAGGCTTCCAACGGCTAGTTGACATCGTTTACG  664
             ||||||||||||||||||||||||||||||||||||||||||||||||||||||||||||
Sbjct  825   ATGCGTTAGCTGCGCCACTAAAAGCTCAAGGCTTCCAACGGCTAGTTGACATCGTTTACG  766

Query  665   GCGTGGACTACCAGGGTATCTAATCCTGTTTGCTCCCCACGCTTTCGCACCTCAGTGTCA  724
             ||||||||||||||||||||||||||||||||||||||||||||||||||||||||||||
Sbjct  765   GCGTGGACTACCAGGGTATCTAATCCTGTTTGCTCCCCACGCTTTCGCACCTCAGTGTCA  706

Query  725   GTATTAGTCCAGGTGGTCGCCTTCGCCACTGGTGTTCCTTCCTATATCTACGCATTTCAC  784
             ||||||||||||||||||||||||||||||||||||||||||||||||||||||||||||
Sbjct  705   GTATTAGTCCAGGTGGTCGCCTTCGCCACTGGTGTTCCTTCCTATATCTACGCATTTCAC  646

Query  785   CGCTACACAGGAAATTCCACCACCCTCTACCATACTCTAGTCAGTCAGTTTTGAATGCAG  844
             ||||||||||||||||||||||||||||||||||||||||||||||||||||||||||||
Sbjct  645   CGCTACACAGGAAATTCCACCACCCTCTACCATACTCTAGTCAGTCAGTTTTGAATGCAG  586

Query  845   TTCCCAGGTTGAGCCCGGGGATTTCACATCCAACTTAACAAACCACCTACGCGCGCTTTA  904
             ||||||||||||||||||||||||||||||||||||||||||||||||||||||||||||
Sbjct  585   TTCCCAGGTTGAGCCCGGGGATTTCACATCCAACTTAACAAACCACCTACGCGCGCTTTA  526

Query  905   CGCCCAGTAATTCCGATTAACGCTTGCACCCTCTGTATTACCGCGGCTGCTGGCACAGAG  964
             ||||||||||||||||||||||||||||||||||||||||||||||||||||||||||||
Sbjct  525   CGCCCAGTAATTCCGATTAACGCTTGCACCCTCTGTATTACCGCGGCTGCTGGCACAGAG  466

Query  965   TTAGCCGGTGCTTATTCTGTCGGTAACGTCAAANCAATCACGTATTAGGTAACTGCCCTT  1024
             ||||||||||||||||||||||||||||||||| ||||||||||||||||||||||||||
Sbjct  465   TTAGCCGGTGCTTATTCTGTCGGTAACGTCAAAACAATCACGTATTAGGTAACTGCCCTT  406

Query  1025  CCTCCCAACTTAAAGTGCTTTACAATCCGAAGNCCTT  1061
             |||||||||||||||||||||||||||||||| ||||
Sbjct  405   CCTCCCAACTTAAAGTGCTTTACAATCCGAAGACCTT  369
```

Download

FASTA (complete sequence)

FASTA (aligned sequences)

GenBank (complete sequence)

Continue
Cancel

GenBankGraphics

Next
Previous
Descriptions

Pseudomonas putida partial 16S rRNA gene, strain CFBP 5898

Sequence ID: HF545843.1Length: 1418Number of Matches: 1

Related Information

Range 1: 360 to 1416GenBankGraphics

Next Match
Previous Match
First Match

Alignment statistics for match #1

| Score | Expect | Identities | Gaps | Strand | Frame |
| --- | --- | --- | --- | --- | --- |
| 1932 bits(1046) | 0.0() | 1052/1057(99%) | 0/1057(0%) | Plus/Minus |  |

Features:

```
Query  5     CGTGNNACCGTCCTCCCGAAGGTTAGACTAGCTACTTCTGGTGCAACCCACTCCCATGGT  64
             ||||  ||||||||||||||||||||||||||||||||||||||||||||||||||||||
Sbjct  1416  CGTGGTACCGTCCTCCCGAAGGTTAGACTAGCTACTTCTGGTGCAACCCACTCCCATGGT  1357

Query  65    GTGACGGGCGGTGTGTACAAGGCCCGGGAACGTATTCACCGCGACATTCTGATTCGCGAT  124
             ||||||||||||||||||||||||||||||||||||||||||||||||||||||||||||
Sbjct  1356  GTGACGGGCGGTGTGTACAAGGCCCGGGAACGTATTCACCGCGACATTCTGATTCGCGAT  1297

Query  125   TACTAGCGATTCCGACTTCACGCAGTCGAGTTGCAGACTGCGATCCGGACTACGATCGGT  184
             ||||||||||||||||||||||||||||||||||||||||||||||||||||||||||||
Sbjct  1296  TACTAGCGATTCCGACTTCACGCAGTCGAGTTGCAGACTGCGATCCGGACTACGATCGGT  1237

Query  185   TTTATGGGATTAGCTCCACCTCGCGGCTTGGCAACCCTCTGTACCGACCATTGTAGCACG  244
             ||||||||||||||||||||||||||||||||||||||||||||||||||||||||||||
Sbjct  1236  TTTATGGGATTAGCTCCACCTCGCGGCTTGGCAACCCTCTGTACCGACCATTGTAGCACG  1177

Query  245   TGTGTAGCCCAGGCCGTAAGGGCCATGATGACTTGACGTCATCCCCACCTTCCTCCGGTT  304
             ||||||||||||||||||||||||||||||||||||||||||||||||||||||||||||
Sbjct  1176  TGTGTAGCCCAGGCCGTAAGGGCCATGATGACTTGACGTCATCCCCACCTTCCTCCGGTT  1117

Query  305   TGTCACCGGCAGTCTCCTTAGAGTGCCCACCATCACGTGCTGGTAACTAAGGACAAGGGT  364
             ||||||||||||||||||||||||||||||||| ||||||||||||||||||||||||||
Sbjct  1116  TGTCACCGGCAGTCTCCTTAGAGTGCCCACCATTACGTGCTGGTAACTAAGGACAAGGGT  1057

Query  365   TGCGCTCGTTACGGGACTTAACCCAACATCTCACGACACGAGCTGACGACAGCCATGCAG  424
             ||||||||||||||||||||||||||||||||||||||||||||||||||||||||||||
Sbjct  1056  TGCGCTCGTTACGGGACTTAACCCAACATCTCACGACACGAGCTGACGACAGCCATGCAG  997

Query  425   CACCTGTCTCAATGTTCCCGAAGGCACCAATCTATCTCTAGAAAGTTCATTGGATGTCAA  484
             ||||||||||||||||||||||||||||||||||||||||||||||||||||||||||||
Sbjct  996   CACCTGTCTCAATGTTCCCGAAGGCACCAATCTATCTCTAGAAAGTTCATTGGATGTCAA  937

Query  485   GGCCTGGTAAGGTTCTTCGCGTTGCTTCGAATTAAACCACATGCTCCACCGCTTGTGCGG  544
             ||||||||||||||||||||||||||||||||||||||||||||||||||||||||||||
Sbjct  936   GGCCTGGTAAGGTTCTTCGCGTTGCTTCGAATTAAACCACATGCTCCACCGCTTGTGCGG  877

Query  545   GCCCCCGTCAATTCATTTGAGTTTTAACCTTGCGGCCGTACTCCCCAGGCGGTCAACTTA  604
             ||||||||||||||||||||||||||||||||||||||||||||||||||||||||||||
Sbjct  876   GCCCCCGTCAATTCATTTGAGTTTTAACCTTGCGGCCGTACTCCCCAGGCGGTCAACTTA  817

Query  605   ATGCGTTAGCTGCGCCACTAAAAGCTCAAGGCTTCCAACGGCTAGTTGACATCGTTTACG  664
             ||||||||||||||||||||||||||||||||||||||||||||||||||||||||||||
Sbjct  816   ATGCGTTAGCTGCGCCACTAAAAGCTCAAGGCTTCCAACGGCTAGTTGACATCGTTTACG  757

Query  665   GCGTGGACTACCAGGGTATCTAATCCTGTTTGCTCCCCACGCTTTCGCACCTCAGTGTCA  724
             ||||||||||||||||||||||||||||||||||||||||||||||||||||||||||||
Sbjct  756   GCGTGGACTACCAGGGTATCTAATCCTGTTTGCTCCCCACGCTTTCGCACCTCAGTGTCA  697

Query  725   GTATTAGTCCAGGTGGTCGCCTTCGCCACTGGTGTTCCTTCCTATATCTACGCATTTCAC  784
             ||||||||||||||||||||||||||||||||||||||||||||||||||||||||||||
Sbjct  696   GTATTAGTCCAGGTGGTCGCCTTCGCCACTGGTGTTCCTTCCTATATCTACGCATTTCAC  637

Query  785   CGCTACACAGGAAATTCCACCACCCTCTACCATACTCTAGTCAGTCAGTTTTGAATGCAG  844
             ||||||||||||||||||||||||||||||||||||||||||||||||||||||||||||
Sbjct  636   CGCTACACAGGAAATTCCACCACCCTCTACCATACTCTAGTCAGTCAGTTTTGAATGCAG  577

Query  845   TTCCCAGGTTGAGCCCGGGGATTTCACATCCAACTTAACAAACCACCTACGCGCGCTTTA  904
             ||||||||||||||||||||||||||||||||||||||||||||||||||||||||||||
Sbjct  576   TTCCCAGGTTGAGCCCGGGGATTTCACATCCAACTTAACAAACCACCTACGCGCGCTTTA  517

Query  905   CGCCCAGTAATTCCGATTAACGCTTGCACCCTCTGTATTACCGCGGCTGCTGGCACAGAG  964
             ||||||||||||||||||||||||||||||||||||||||||||||||||||||||||||
Sbjct  516   CGCCCAGTAATTCCGATTAACGCTTGCACCCTCTGTATTACCGCGGCTGCTGGCACAGAG  457

Query  965   TTAGCCGGTGCTTATTCTGTCGGTAACGTCAAANCAATCACGTATTAGGTAACTGCCCTT  1024
             ||||||||||||||||||||||||||||||||| ||||||||||||||||||||||||||
Sbjct  456   TTAGCCGGTGCTTATTCTGTCGGTAACGTCAAAACAATCACGTATTAGGTAACTGCCCTT  397

Query  1025  CCTCCCAACTTAAAGTGCTTTACAATCCGAAGNCCTT  1061
             |||||||||||||||||||||||||||||||| ||||
Sbjct  396   CCTCCCAACTTAAAGTGCTTTACAATCCGAAGACCTT  360
```


BLAST is a registered trademark of the National Library of Medicine

Support center
Mailing list


YouTube

- National Library Of Medicine
- National Institutes Of Health
- U.S. Department of Health & Human Services
- USA.gov

### NCBI


National Center for Biotechnology Information,
 U.S. National Library of Medicine

8600 Rockville Pike,
Bethesda
 MD,
20894
USA

Policies and Guidelines
|
Contact


PreferencesTurn off

External link. Please review our privacy policy.
