# Supplementary material for: Isothermal microcalorimetry for thermal viable count of microorganisms in pure cultures and stabilized formulations
Source: BMC Microbiol. 2019 Mar 21;19:65. doi: 10.1186/s12866-019-1432-8 (PMC6429831; doi:10.1186/s12866-019-1432-8)
Supplement: Supplementary file 3 — 16S rRNA gene sequences’ BLASTN hits in zipped HTML format. (ZIP 15810 kb) [file 12866_2019_1432_MOESM3_ESM.zip › Best blastn hits/NCBI Blast_MA250 positive R -- 10..1065 of sequence.html]

NCBI Blast:MA250 positive R -- 10..1065 of sequence


- NCBI Home
- Sign in to NCBI
- Skip to Main Content
- Skip to Navigation
- About NCBI Accesskeys

U.S. National Library of Medicine

NCBI
National Center for Biotechnology Information

- My NCBI
- Sign in to NCBI
- Register
- Sign Out

BLAST ^®^ » blastn suite » RID-A50JM53B014


- Home
- Recent Results
- Saved Strategies
- Help

BLAST Results


Edit and Resubmit
Save Search Strategies
[Sign in above to save your search strategy]

Formatting options 


Download


How to read this page
Blast report description
Questions/comments


|  |  |
| --- | --- |
| Formatting options | |
| Show | Alignment as  HTML Plain text   Old View Reset form to defaults [?]  These options control formatting of alignments in results pages. The default is HTML, but other formats (including plain text) are available. PSSM and PssmWithParameters are representations of Position Specific Scoring Matrices and are only available for PSI-BLAST. The Advanced view option allows the database descriptions to be sorted by various indices in a table. |
| Alignment View | Pairwise Pairwise with dots for identities Query-anchored with dots for identities Query-anchored with letters for identities Flat query-anchored with dots for identities Flat query-anchored with letters for identities [?]  Choose how to view alignments. The default "pairwise" view shows how each subject sequence aligns individually to the query sequence. The "query-anchored" view shows how all subject sequences align to the query sequence. For each view type, you can choose to show "identities" (matching residues) as letters or dots. more... |
| Display | Graphical Overview   Linkout   Sequence Retrieval  NCBI-gi   CDS feature [?]  - Graphical Overview: Graphical Overview: Show graph of similar sequence regions aligned to query.   more... - NCBI-gi: Show NCBI gi identifiers. - CDS feature: Show annotated coding region and translation.   more... |
| Masking | Character:   X for protein, n for nucleotide Lower Case  Color:  Black Grey Red [?]  - Masking Character: Display masked (filtered) sequence regions as lower-case or as specific letters (N for nucleotide, P for protein). - Masking Color: Display masked sequence regions in the given color. |
| Limit results | Descriptions:  10 50 100 Graphical overview:  0 10 50 100  Alignments:  0 10 50 100 Line length:  60 90 120 150 [?]  - Descriptions: Show short descriptions for up to the given number of sequences. - Alignments: Show alignments for up to the given number of sequences, in order of statistical significance. - Line lenghth: Number of letters to show on one line in an alignment. |
|  | Organism Type common name, binomial, taxid, or group name. Only 20 top taxa will be shown.     Exclude   [?]  Show only sequences from the given organism. |
|  | Entrez query:  [?]  Show only those sequences that match the given Entrez query. more... |
|  | Expect Min:  Expect Max:  [?]  Show only sequences with expect values in the given range. more... |
|  | Percent Identity Min:  Percent Identity Max:  [?]  Show only sequences with percent identity values in the given range. |
| Format for | PSI-BLAST with inclusion threshold:  [?]  - Format for PSI-BLAST: The Position-Specific Iterated BLAST (PSI-BLAST) program performs iterative searches with a protein query,   in which sequences found in one round of search are used to build a custom score model for the next round.   more... - Inclusion Threshold: This sets the statistical significance threshold for including a sequence in the model used   by PSI-BLAST to create the PSSM on the next iteration. |

|  |  |  |  |  |  |
| --- | --- | --- | --- | --- | --- |
| Download | | | | | |
| Alignment  Text XML ASN.1 JSON Seq-align Hit Table(text) Hit Table(csv) Multiple-file XML2 Single-file XML2 Multiple-file JSON Single-file JSON SAM | Search Strategies  ASN.1 | PSSM to restart search  PSSM | [?] |  |  |

The Download link provides BLAST output that may be used as input to another program.
This includes parseable formats such as the tabular report or XML as well as the Search Strategy files read by the BLAST+ applications.
More details on the parseable (XML, tabular, and ASN.1) reports can be found at
https://www.ncbi.nlm.nih.gov/books/NBK153387/  
  

The following formats are offered under the Alignment section:  
1). "Text". Non-HTML standard BLAST report.  
2). "XML". XML report based upon the DTD at https://www.ncbi.nlm.nih.gov/data\_specs/dtd/NCBI\_BlastOutput.dtd  
3). "ASN.1". Alignment written out in Abstract Syntax Notation 1.  
4). "JSON Seq-align". Alignment written out in JSON.  
4). "Hit Table(text)". The tabular report as text.  
5). "Hit Table(csv)". The tabular report ready for import into spread-sheet programs like Excel.  
6). "XML2". New XML format described at ftp://ftp.ncbi.nlm.nih.gov/blast/documents/NEWXML/xml2.pdf.  
7). "JSON". New JSON format described at ftp://ftp.ncbi.nlm.nih.gov/blast/documents/NEWXML/xml2.pdf.  
8). "SAM". Sequence Alignment Map format.

XML2 and JSON can be downloaded either as one file per query (multiple-file) or one file for all queries (single-file). These formats are listed as Multiple-file XML2 (and JSON) or Single-file XML (and JSON).

The following report is offered under the Search Strategy section:  
1). "ASN.1" Search Strategy. A record of the parameters, query, and database used in the search. This file can be used to start a stand-alone BLAST search, see
https://www.ncbi.nlm.nih.gov/books/NBK1763/#CmdLineAppsManual.I455\_BLAST\_search\_stra


# Job title: MA250 positive R -- 10..1065 of sequence

Results for:

lcl|Query\_124487 MA250 positive R -- 10..1065 of sequence(1056bp)
[?]

Your BLAST job specified more than one input sequence.
This box lets you choose which input sequence to show BLAST results for.

RID
:   A50JM53B014 (Expires on 03-10 19:47 pm)

Query ID
:   lcl|Query\_124487
:   lcl|Query\_124487

Description
:   MA250 positive R -- 10..1065 of sequence

Molecule type
:   nucleic acid

Query Length
:   1056

Database Name
:   nr

Description
:   Nucleotide collection (nt) See details

Program
:   BLASTN 2.8.0+ Citation

  

Reference 

Zheng Zhang, Scott Schwartz, Lukas Wagner, and Webb Miller (2000), "A greedy algorithm for aligning DNA sequences", J Comput Biol 2000; 7(1-2):203-14.

Reference - database indexing

Aleksandr Morgulis, George Coulouris, Yan Raytselis, Thomas L. Madden, Richa Agarwala, Alejandro A. Schäffer (2008), "Database Indexing for Production MegaBLAST Searches", Bioinformatics 24:1757-1764.

Other reports:
Search Summary

[Taxonomy reports]
[Distance tree of results]
[MSA viewer]

Search Parameters

| Search parameter name | Search parameter value |
| --- | --- |
| Program | blastn |
| Word size | 28 |
| Expect value | 10 |
| Hitlist size | 100 |
| Match/Mismatch scores | 1,-2 |
| Gapcosts | 0,2.5 |
| Low Complexity Filter | Yes |
| Filter string | L;m; |
| Genetic Code | 1 |

Database

| Database parameter name | Database parameter value |
| --- | --- |
| Posted date | Mar 7, 2018 1:58 PM |
| Number of letters | 174,044,644,244 |
| Number of sequences | 46,882,714 |
| Entrez query | Includes:  Excludes:  None |

Karlin-Altschul statistics

| Params | Ungapped | Gapped |
| --- | --- | --- |
| Lambda | 1.33271 | 1.28 |
| K | 0.620991 | 0.46 |
| H | 1.12409 | 0.85 |

Results Statistics

| Results Statistics parameter name | Results Statistics parameter value |
| --- | --- |
| Length adjustment | 35 |
| Effective length of query | 1021 |
| Effective length of database | 172403749254 |
| Effective search space | 176024227988334 |
| Effective search space used | 176024227988334 |


## Graphic Summary

### Distribution of the top 134 Blast Hits on 100 subject sequences [?]

The graphic is an overview of the database sequences aligned to the query sequence. These are represented horizontal bars colored coded by score and showing the extent
of the alignment on the query sequence. Separate aligned regions on the same database sequence are connected by a thin grey line.
Mousing over an alignment shows the database sequence title. Clicking an alignment displays a box with more details about the alignment and
link to the sequence alignment itself in the Alignments section of the report.

Mouse over to see the title, click to show alignments

Color key for alignment scores

<40

40-50

50-80

80-200

>=200

Query

1

200

400

600

800

1000

Pseudomonas brassicacearum isolate MA250 16S ribosomal ..

Score:1916 Evalue:0

Accession:DQ886486.1

Alignment

Pseudomonas sp. strain 7.3 16S ribosomal RNA gene, part..

Score:1910 Evalue:0

Accession:KY542120.1

Alignment

Pseudomonas fluorescens strain SBR10 16S ribosomal RNA ..

Score:1910 Evalue:0

Accession:KX018310.1

Alignment

Pseudomonas sp. cpRA293 16S ribosomal RNA gene, partial..

Score:1910 Evalue:0

Accession:KJ510220.1

Alignment

Pseudomonas brassicacearum strain Kr21 16S ribosomal RN..

Score:1910 Evalue:0

Accession:KT215482.1

Alignment

Pseudomonas brassicacearum strain IHB B 13650 16S ribos..

Score:1910 Evalue:0

Accession:KP762561.1

Alignment

Pseudomonas brassicacearum subsp. neoaurantiaca strain ..

Score:1910 Evalue:0

Accession:KP762555.1

Alignment

Pseudomonas sp. 41(2015) 16S ribosomal RNA gene, partia..

Score:1910 Evalue:0

Accession:KR080568.1

Alignment

Pseudomonas sp. SAM1 16S ribosomal RNA gene, partial se..

Score:1910 Evalue:0

Accession:KM269192.1

Alignment

Pseudomonas brassicacearum strain WK-444s 16S ribosomal..

Score:1910 Evalue:0

Accession:KF580861.1

Alignment

Pseudomonas putida partial 16S rRNA gene, strain CFBP 4..

Score:1910 Evalue:0

Accession:HF545841.1

Alignment

Pseudomonas sp. S-3 16S ribosomal RNA gene, partial seq..

Score:1910 Evalue:0

Accession:KC207086.1

Alignment

Pseudomonas fluorescens gene for 16S rRNA, partial sequ..

Score:1910 Evalue:0

Accession:AB621593.1

Alignment

Pseudomonas thivervalensis 16S ribosomal RNA gene, part..

Score:1910 Evalue:0

Accession:JN628032.1

Alignment

Pseudomonas fluorescens 16S rRNA gene, strain C7R12

Score:1910 Evalue:0

Accession:AM229082.1

Alignment

Pseudomonas sp. HA-09 partial 16S rRNA gene, isolate HA..

Score:1906 Evalue:0

Accession:LT844660.1

Alignment

Pseudomonas brassicacearum subsp. brassicacearum strain..

Score:1906 Evalue:0

Accession:KP730603.1

Alignment

Uncultured bacterium clone Untreatedsoil-0day-94 16S ri..

Score:1905 Evalue:0

Accession:MF314815.2

Alignment

Uncultured bacterium clone Untreatedsoil-0day-1 16S rib..

Score:1905 Evalue:0

Accession:MF314725.2

Alignment

Pseudomonas fluorescens strain 2P24 chromosome, complet..

Score:1905 Evalue:0

Accession:CP025542.1

Alignment

Pseudomonas brassicacearum strain LBUM300 16S ribosomal..

Score:1905 Evalue:0

Accession:MG461459.1

Alignment

Pseudomonas putida strain 42R-P6 16S ribosomal RNA gene..

Score:1905 Evalue:0

Accession:MF062638.1

Alignment

Uncultured bacterium clone SPN400-90day-85 16S ribosoma..

Score:1905 Evalue:0

Accession:MF314628.1

Alignment

Uncultured bacterium clone SPN400-90day-76 16S ribosoma..

Score:1905 Evalue:0

Accession:MF314621.1

Alignment

Uncultured bacterium clone SPN400-90day-50 16S ribosoma..

Score:1905 Evalue:0

Accession:MF314595.1

Alignment

Uncultured bacterium clone SPN400-90day-46 16S ribosoma..

Score:1905 Evalue:0

Accession:MF314592.1

Alignment

Uncultured bacterium clone SPN400-90day-43 16S ribosoma..

Score:1905 Evalue:0

Accession:MF314589.1

Alignment

Uncultured bacterium clone SPN400-90day-20 16S ribosoma..

Score:1905 Evalue:0

Accession:MF314569.1

Alignment

Uncultured bacterium clone SPN400-90day-6 16S ribosomal..

Score:1905 Evalue:0

Accession:MF314558.1

Alignment

Pseudomonas brassicacearum strain Pv10 16S ribosomal RN..

Score:1905 Evalue:0

Accession:MF624719.1

Alignment

Pseudomonas jessenii strain AP3\_16 16S ribosomal RNA ge..

Score:1905 Evalue:0

Accession:MF498772.1

Alignment

Pseudomonas sp. strain A9 16S ribosomal RNA gene, parti..

Score:1905 Evalue:0

Accession:KX859150.1

Alignment

Pseudomonas brassicacearum strain YC-1 16S ribosomal RN..

Score:1905 Evalue:0

Accession:KY753310.1

Alignment

Pseudomonas brassicacearum strain FC-7 16S ribosomal RN..

Score:1905 Evalue:0

Accession:KY649379.1

Alignment

Pseudomonas fluorescens strain PFR1 16S ribosomal RNA g..

Score:1905 Evalue:0

Accession:MF000304.1

Alignment

Pseudomonas brassicacearum strain SKUAST-K21 16S riboso..

Score:1905 Evalue:0

Accession:KY612271.1

Alignment

Pseudomonas syringae strain yangyueP4 16S ribosomal RNA..

Score:1905 Evalue:0

Accession:KU977139.1

Alignment

Pseudomonas brassicacearum strain B04 16S ribosomal RNA..

Score:1905 Evalue:0

Accession:KU878092.1

Alignment

Pseudomonas synxantha strain LMG 2190 genome assembly, ..

Score:1905 Evalue:0

Accession:LT629786.1

Alignment

Pseudomonas brassicacearum strain BS3663 genome assembl..

Score:1905 Evalue:0

Accession:LT629713.1

Alignment

Pseudomonas brassicacearum strain TSDS1 16S ribosomal R..

Score:1905 Evalue:0

Accession:KX984045.1

Alignment

Pseudomonas brassicacearum strain Delaware 16S ribosoma..

Score:1905 Evalue:0

Accession:KT695846.1

Alignment

Pseudomonas brassicacearum strain Wood1 16S ribosomal R..

Score:1905 Evalue:0

Accession:KT695843.1

Alignment

Pseudomonas brassicacearum strain 93F8 16S ribosomal RN..

Score:1905 Evalue:0

Accession:KT695841.1

Alignment

Pseudomonas brassicacearum strain L13-6-12, complete ge..

Score:1905 Evalue:0

Accession:CP014693.1

Alignment

Pseudomonas brassicacearum strain KK 5 16S ribosomal RN..

Score:1905 Evalue:0

Accession:KP858915.1

Alignment

Pseudomonas fluorescens strain FW300-N2E2 genome

Score:1905 Evalue:0

Accession:CP015225.1

Alignment

Pseudomonas jessenii partial 16S rRNA gene, strain R-52..

Score:1905 Evalue:0

Accession:LN995697.1

Alignment

Pseudomonas brassicacearum strain LBUM300, complete gen..

Score:1905 Evalue:0

Accession:CP012680.1

Alignment

Pseudomonas corrugata strain G1 16S ribosomal RNA gene,..

Score:1905 Evalue:0

Accession:KT957303.1

Alignment

Pseudomonas brassicacearum strain SM27 16S ribosomal RN..

Score:1905 Evalue:0

Accession:KR855698.1

Alignment

Pseudomonas fluorescens strain FW300-N2C3, complete gen..

Score:1905 Evalue:0

Accession:CP012831.1

Alignment

Pseudomonas migulae strain DD290 16S ribosomal RNA gene..

Score:1905 Evalue:0

Accession:KR822273.1

Alignment

Pseudomonas fluorescens 16S ribosomal RNA gene, partial..

Score:1905 Evalue:0

Accession:KT321681.1

Alignment

Pseudomonas brassicacearum strain 13B-23 16S ribosomal ..

Score:1905 Evalue:0

Accession:KR083019.1

Alignment

Pseudomonas thivervalensis strain MAH1 16S ribosomal RN..

Score:1905 Evalue:0

Accession:KP742980.1

Alignment

## Descriptions

, Reading indexes 1-5, displaying indexes 1-5


Load next setPrevious Match

Sequences producing significant alignments:

Show all columns  of the table presenting sequences producing significant alignments 

Select:AllNone
Selected:0

Alignments
Download

FASTA (complete sequence)

FASTA (aligned sequences)

GenBank (complete sequence)

Hit Table (text)

Hit Table (CSV)

Text

XML

ASN.1

Continue
Cancel

GenBank 
Graphics
Distance tree of results
Multiple alignment
Show/hide columns of the table presenting sequences producing significant alignments 

Available columns

Description  
Max Score  
Total Score  
Coverage  
E-value  
IdentN  
Accession  
Restore Defaults
Ok
Cancel

Sequences producing significant alignments:

| Select for downloading or viewing reports | Description | Max score | Total score | Query cover | E value | Ident | Accession |
| --- | --- | --- | --- | --- | --- | --- | --- |
| 1Select seq DQ886486.1 | Pseudomonas brassicacearum isolate MA250 16S ribosomal RNA gene, partial sequence | 1916 | 1916 | 98% | 0.0 | 99% | DQ886486.1 |
| 2Select seq KY542120.1 | Pseudomonas sp. strain 7.3 16S ribosomal RNA gene, partial sequence | 1910 | 1910 | 98% | 0.0 | 99% | KY542120.1 |
| 3Select seq KX018310.1 | Pseudomonas fluorescens strain SBR10 16S ribosomal RNA gene, partial sequence | 1910 | 1910 | 98% | 0.0 | 99% | KX018310.1 |
| 4Select seq KJ510220.1 | Pseudomonas sp. cpRA293 16S ribosomal RNA gene, partial sequence | 1910 | 1910 | 98% | 0.0 | 99% | KJ510220.1 |
| 5Select seq KT215482.1 | Pseudomonas brassicacearum strain Kr21 16S ribosomal RNA gene, partial sequence | 1910 | 1910 | 98% | 0.0 | 99% | KT215482.1 |
| 6Select seq KP762561.1 | Pseudomonas brassicacearum strain IHB B 13650 16S ribosomal RNA gene, partial sequence | 1910 | 1910 | 98% | 0.0 | 99% | KP762561.1 |
| 7Select seq KP762555.1 | Pseudomonas brassicacearum subsp. neoaurantiaca strain IHB B 13645 16S ribosomal RNA gene, partial sequence | 1910 | 1910 | 98% | 0.0 | 99% | KP762555.1 |
| 8Select seq KR080568.1 | Pseudomonas sp. 41(2015) 16S ribosomal RNA gene, partial sequence | 1910 | 1910 | 98% | 0.0 | 99% | KR080568.1 |
| 9Select seq KM269192.1 | Pseudomonas sp. SAM1 16S ribosomal RNA gene, partial sequence | 1910 | 1910 | 98% | 0.0 | 99% | KM269192.1 |
| 10Select seq KF580861.1 | Pseudomonas brassicacearum strain WK-444s 16S ribosomal RNA gene, partial sequence | 1910 | 1910 | 98% | 0.0 | 99% | KF580861.1 |
| 11Select seq HF545841.1 | Pseudomonas putida partial 16S rRNA gene, strain CFBP 4629 | 1910 | 1910 | 98% | 0.0 | 99% | HF545841.1 |
| 12Select seq KC207086.1 | Pseudomonas sp. S-3 16S ribosomal RNA gene, partial sequence | 1910 | 1910 | 98% | 0.0 | 99% | KC207086.1 |
| 13Select seq AB621593.1 | Pseudomonas fluorescens gene for 16S rRNA, partial sequence, strain: MPF29 | 1910 | 1910 | 98% | 0.0 | 99% | AB621593.1 |
| 14Select seq JN628032.1 | Pseudomonas thivervalensis 16S ribosomal RNA gene, partial sequence | 1910 | 1910 | 98% | 0.0 | 99% | JN628032.1 |
| 15Select seq AM229082.1 | Pseudomonas fluorescens 16S rRNA gene, strain C7R12 | 1910 | 1910 | 98% | 0.0 | 99% | AM229082.1 |
| 16Select seq LT844660.1 | Pseudomonas sp. HA-09 partial 16S rRNA gene, isolate HA-09 | 1906 | 1906 | 98% | 0.0 | 99% | LT844660.1 |
| 17Select seq KP730603.1 | Pseudomonas brassicacearum subsp. brassicacearum strain BW0808 16S ribosomal RNA gene, partial sequence | 1906 | 1906 | 98% | 0.0 | 99% | KP730603.1 |
| 18Select seq MF314815.2 | Uncultured bacterium clone Untreatedsoil-0day-94 16S ribosomal RNA gene, partial sequence | 1905 | 1905 | 98% | 0.0 | 99% | MF314815.2 |
| 19Select seq MF314725.2 | Uncultured bacterium clone Untreatedsoil-0day-1 16S ribosomal RNA gene, partial sequence | 1905 | 1905 | 98% | 0.0 | 99% | MF314725.2 |
| 20Select seq CP025542.1 | Pseudomonas fluorescens strain 2P24 chromosome, complete genome | 1905 | 9514 | 98% | 0.0 | 99% | CP025542.1 |
| 21Select seq MG461459.1 | Pseudomonas brassicacearum strain LBUM300 16S ribosomal RNA gene, partial sequence | 1905 | 1905 | 98% | 0.0 | 99% | MG461459.1 |
| 22Select seq MF062638.1 | Pseudomonas putida strain 42R-P6 16S ribosomal RNA gene, partial sequence | 1905 | 1905 | 98% | 0.0 | 99% | MF062638.1 |
| 23Select seq MF314628.1 | Uncultured bacterium clone SPN400-90day-85 16S ribosomal RNA gene, partial sequence | 1905 | 1905 | 98% | 0.0 | 99% | MF314628.1 |
| 24Select seq MF314621.1 | Uncultured bacterium clone SPN400-90day-76 16S ribosomal RNA gene, partial sequence | 1905 | 1905 | 98% | 0.0 | 99% | MF314621.1 |
| 25Select seq MF314595.1 | Uncultured bacterium clone SPN400-90day-50 16S ribosomal RNA gene, partial sequence | 1905 | 1905 | 98% | 0.0 | 99% | MF314595.1 |
| 26Select seq MF314592.1 | Uncultured bacterium clone SPN400-90day-46 16S ribosomal RNA gene, partial sequence | 1905 | 1905 | 98% | 0.0 | 99% | MF314592.1 |
| 27Select seq MF314589.1 | Uncultured bacterium clone SPN400-90day-43 16S ribosomal RNA gene, partial sequence | 1905 | 1905 | 98% | 0.0 | 99% | MF314589.1 |
| 28Select seq MF314569.1 | Uncultured bacterium clone SPN400-90day-20 16S ribosomal RNA gene, partial sequence | 1905 | 1905 | 98% | 0.0 | 99% | MF314569.1 |
| 29Select seq MF314558.1 | Uncultured bacterium clone SPN400-90day-6 16S ribosomal RNA gene, partial sequence | 1905 | 1905 | 98% | 0.0 | 99% | MF314558.1 |
| 30Select seq MF624719.1 | Pseudomonas brassicacearum strain Pv10 16S ribosomal RNA gene, partial sequence | 1905 | 1905 | 98% | 0.0 | 99% | MF624719.1 |
| 31Select seq MF498772.1 | Pseudomonas jessenii strain AP3\_16 16S ribosomal RNA gene, partial sequence | 1905 | 1905 | 98% | 0.0 | 99% | MF498772.1 |
| 32Select seq KX859150.1 | Pseudomonas sp. strain A9 16S ribosomal RNA gene, partial sequence | 1905 | 1905 | 98% | 0.0 | 99% | KX859150.1 |
| 33Select seq KY753310.1 | Pseudomonas brassicacearum strain YC-1 16S ribosomal RNA gene, partial sequence | 1905 | 1905 | 98% | 0.0 | 99% | KY753310.1 |
| 34Select seq KY649379.1 | Pseudomonas brassicacearum strain FC-7 16S ribosomal RNA gene, partial sequence | 1905 | 1905 | 98% | 0.0 | 99% | KY649379.1 |
| 35Select seq MF000304.1 | Pseudomonas fluorescens strain PFR1 16S ribosomal RNA gene, partial sequence | 1905 | 1905 | 98% | 0.0 | 99% | MF000304.1 |
| 36Select seq KY612271.1 | Pseudomonas brassicacearum strain SKUAST-K21 16S ribosomal RNA gene, partial sequence | 1905 | 1905 | 98% | 0.0 | 99% | KY612271.1 |
| 37Select seq KU977139.1 | Pseudomonas syringae strain yangyueP4 16S ribosomal RNA gene, partial sequence | 1905 | 1905 | 98% | 0.0 | 99% | KU977139.1 |
| 38Select seq KU878092.1 | Pseudomonas brassicacearum strain B04 16S ribosomal RNA gene, partial sequence | 1905 | 1905 | 98% | 0.0 | 99% | KU878092.1 |
| 39Select seq LT629786.1 | Pseudomonas synxantha strain LMG 2190 genome assembly, chromosome: I | 1905 | 12681 | 98% | 0.0 | 99% | LT629786.1 |
| 40Select seq LT629713.1 | Pseudomonas brassicacearum strain BS3663 genome assembly, chromosome: I | 1905 | 9525 | 98% | 0.0 | 99% | LT629713.1 |
| 41Select seq KX984045.1 | Pseudomonas brassicacearum strain TSDS1 16S ribosomal RNA gene, partial sequence | 1905 | 1905 | 98% | 0.0 | 99% | KX984045.1 |
| 42Select seq KT695846.1 | Pseudomonas brassicacearum strain Delaware 16S ribosomal RNA gene, partial sequence | 1905 | 1905 | 98% | 0.0 | 99% | KT695846.1 |
| 43Select seq KT695843.1 | Pseudomonas brassicacearum strain Wood1 16S ribosomal RNA gene, partial sequence | 1905 | 1905 | 98% | 0.0 | 99% | KT695843.1 |
| 44Select seq KT695841.1 | Pseudomonas brassicacearum strain 93F8 16S ribosomal RNA gene, partial sequence | 1905 | 1905 | 98% | 0.0 | 99% | KT695841.1 |
| 45Select seq CP014693.1 | Pseudomonas brassicacearum strain L13-6-12, complete genome | 1905 | 9525 | 98% | 0.0 | 99% | CP014693.1 |
| 46Select seq KP858915.1 | Pseudomonas brassicacearum strain KK 5 16S ribosomal RNA gene, partial sequence | 1905 | 1905 | 98% | 0.0 | 99% | KP858915.1 |
| 47Select seq CP015225.1 | Pseudomonas fluorescens strain FW300-N2E2 genome | 1905 | 9517 | 98% | 0.0 | 99% | CP015225.1 |
| 48Select seq LN995697.1 | Pseudomonas jessenii partial 16S rRNA gene, strain R-52636 | 1905 | 1905 | 98% | 0.0 | 99% | LN995697.1 |
| 49Select seq CP012680.1 | Pseudomonas brassicacearum strain LBUM300, complete genome | 1905 | 9525 | 98% | 0.0 | 99% | CP012680.1 |
| 50Select seq KT957303.1 | Pseudomonas corrugata strain G1 16S ribosomal RNA gene, partial sequence | 1905 | 1905 | 98% | 0.0 | 99% | KT957303.1 |
| 51Select seq KR855698.1 | Pseudomonas brassicacearum strain SM27 16S ribosomal RNA gene, partial sequence | 1905 | 1905 | 98% | 0.0 | 99% | KR855698.1 |
| 52Select seq CP012831.1 | Pseudomonas fluorescens strain FW300-N2C3, complete genome | 1905 | 9525 | 98% | 0.0 | 99% | CP012831.1 |
| 53Select seq KR822273.1 | Pseudomonas migulae strain DD290 16S ribosomal RNA gene, partial sequence | 1905 | 1905 | 98% | 0.0 | 99% | KR822273.1 |
| 54Select seq KT321681.1 | Pseudomonas fluorescens 16S ribosomal RNA gene, partial sequence | 1905 | 1905 | 98% | 0.0 | 99% | KT321681.1 |
| 55Select seq KR083019.1 | Pseudomonas brassicacearum strain 13B-23 16S ribosomal RNA gene, partial sequence | 1905 | 1905 | 98% | 0.0 | 99% | KR083019.1 |
| 56Select seq KP742980.1 | Pseudomonas thivervalensis strain MAH1 16S ribosomal RNA gene, partial sequence | 1905 | 1905 | 98% | 0.0 | 99% | KP742980.1 |
| 57Select seq KR611048.1 | Pseudomonas brassicacearum strain 11B23 16S ribosomal RNA gene, partial sequence | 1905 | 1905 | 98% | 0.0 | 99% | KR611048.1 |
| 58Select seq KR611047.1 | Pseudomonas brassicacearum strain 3B1 16S ribosomal RNA gene, partial sequence | 1905 | 1905 | 98% | 0.0 | 99% | KR611047.1 |
| 59Select seq KR080563.1 | Pseudomonas sp. 15(2015) 16S ribosomal RNA gene, partial sequence | 1905 | 1905 | 98% | 0.0 | 99% | KR080563.1 |
| 60Select seq KR080562.1 | Pseudomonas sp. 12(2015) 16S ribosomal RNA gene, partial sequence | 1905 | 1905 | 98% | 0.0 | 99% | KR080562.1 |
| 61Select seq LC015570.1 | Pseudomonas brassicacearum subsp. brassicacearum gene for 16S ribosomal RNA, partial sequence, strain: AF129 | 1905 | 1905 | 98% | 0.0 | 99% | LC015570.1 |
| 62Select seq LC015569.1 | Pseudomonas brassicacearum subsp. brassicacearum gene for 16S ribosomal RNA, partial sequence, strain: AF5 | 1905 | 1905 | 98% | 0.0 | 99% | LC015569.1 |
| 63Select seq LC015567.1 | Pseudomonas brassicacearum subsp. neoaurantiaca gene for 16S ribosomal RNA, partial sequence, strain: AF82 | 1905 | 1905 | 98% | 0.0 | 99% | LC015567.1 |
| 64Select seq KM030057.1 | Pseudomonas sp. TY1210 16S ribosomal RNA gene, partial sequence | 1905 | 1905 | 98% | 0.0 | 99% | KM030057.1 |
| 65Select seq KJ420530.1 | Pseudomonas thivervalensis strain PE32 16S ribosomal RNA gene, partial sequence | 1905 | 1905 | 98% | 0.0 | 99% | KJ420530.1 |
| 66Select seq KF148637.1 | Pseudomonas fluorescens strain JK15 16S ribosomal RNA gene, partial sequence | 1905 | 1905 | 98% | 0.0 | 99% | KF148637.1 |
| 67Select seq KF840730.1 | Pseudomonas kilonensis strain JX22 16S ribosomal RNA gene, partial sequence | 1905 | 1905 | 98% | 0.0 | 99% | KF840730.1 |
| 68Select seq KF835389.1 | Pseudomonas sp. SDW-16 16S ribosomal RNA gene, partial sequence | 1905 | 1905 | 98% | 0.0 | 99% | KF835389.1 |
| 69Select seq KF475874.1 | Pseudomonas brassicacearum strain IHB B 821 16S ribosomal RNA gene, partial sequence | 1905 | 1905 | 98% | 0.0 | 99% | KF475874.1 |
| 70Select seq KF360067.1 | Pseudomonas sp. BSP5 16S ribosomal RNA gene, partial sequence | 1905 | 1905 | 98% | 0.0 | 99% | KF360067.1 |
| 71Select seq KF312468.1 | Pseudomonas sp. SJ7b 16S ribosomal RNA gene, partial sequence | 1905 | 1905 | 98% | 0.0 | 99% | KF312468.1 |
| 72Select seq KC428747.1 | Pseudomonas sp. X123 16S ribosomal RNA gene, partial sequence | 1905 | 1905 | 98% | 0.0 | 99% | KC428747.1 |
| 73Select seq KC246049.1 | Pseudomonas fluorescens strain IBFC2012-45 16S ribosomal RNA gene, partial sequence | 1905 | 1905 | 98% | 0.0 | 99% | KC246049.1 |
| 74Select seq HQ888871.1 | Pseudomonas fluorescens strain P-72-10 16S ribosomal RNA gene, partial sequence | 1905 | 1905 | 98% | 0.0 | 99% | HQ888871.1 |
| 75Select seq JN216880.1 | Pseudomonas sp. N-128 16S ribosomal RNA gene, partial sequence | 1905 | 1905 | 98% | 0.0 | 99% | JN216880.1 |
| 76Select seq JX077087.1 | Uncultured Pseudomonas sp. clone CGMCG 6071 16S ribosomal RNA gene, partial sequence | 1905 | 1905 | 98% | 0.0 | 99% | JX077087.1 |
| 77Select seq JF901350.1 | Endophytic bacterium 41P-2 16S ribosomal RNA gene, partial sequence | 1905 | 1905 | 98% | 0.0 | 99% | JF901350.1 |
| 78Select seq JN605747.1 | Pseudomonas brassicacearum strain J12 16S ribosomal RNA gene, partial sequence | 1905 | 1905 | 98% | 0.0 | 99% | JN605747.1 |
| 79Select seq JQ779065.1 | Bacterium NTL501 16S ribosomal RNA gene, partial sequence | 1905 | 1905 | 98% | 0.0 | 99% | JQ779065.1 |
| 80Select seq JQ779056.1 | Bacterium NTL264 16S ribosomal RNA gene, partial sequence | 1905 | 1905 | 98% | 0.0 | 99% | JQ779056.1 |
| 81Select seq JQ779041.1 | Bacterium NTL206 16S ribosomal RNA gene, partial sequence | 1905 | 1905 | 98% | 0.0 | 99% | JQ779041.1 |
| 82Select seq JN628030.1 | Pseudomonas brassicacearum subsp. neoaurantiaca 16S ribosomal RNA gene, partial sequence | 1905 | 1905 | 98% | 0.0 | 99% | JN628030.1 |
| 83Select seq JN033549.1 | Pseudomonas brassicacearum strain ME BHU2 16S ribosomal RNA gene, partial sequence | 1905 | 1905 | 98% | 0.0 | 99% | JN033549.1 |
| 84Select seq FR675976.1 | Pseudomonas sp. LB184 partial 16S rRNA gene, isolate LB184 | 1905 | 1905 | 98% | 0.0 | 99% | FR675976.1 |
| 85Select seq JF500978.1 | Uncultured Pseudomonas sp. clone 101 16S ribosomal RNA gene, partial sequence | 1905 | 1905 | 98% | 0.0 | 99% | JF500978.1 |
| 86Select seq JF500937.1 | Uncultured Pseudomonas sp. clone 57 16S ribosomal RNA gene, partial sequence | 1905 | 1905 | 98% | 0.0 | 99% | JF500937.1 |
| 87Select seq JF500932.1 | Uncultured Pseudomonas sp. clone 52 16S ribosomal RNA gene, partial sequence | 1905 | 1905 | 98% | 0.0 | 99% | JF500932.1 |
| 88Select seq JF500908.1 | Uncultured Pseudomonas sp. clone 24 16S ribosomal RNA gene, partial sequence | 1905 | 1905 | 98% | 0.0 | 99% | JF500908.1 |
| 89Select seq CP002585.1 | Pseudomonas brassicacearum subsp. brassicacearum NFM421, complete genome | 1905 | 9519 | 98% | 0.0 | 99% | CP002585.1 |
| 90Select seq HM854217.1 | Pseudomonas fluorescens strain STAD384 16S ribosomal RNA gene, partial sequence | 1905 | 1905 | 98% | 0.0 | 99% | HM854217.1 |
| 91Select seq HM579795.1 | Pseudomonas sp. HY4(2010) 16S ribosomal RNA gene, partial sequence | 1905 | 1905 | 98% | 0.0 | 99% | HM579795.1 |
| 92Select seq KT318824.1 | Pseudomonas sp. RhizorgN 16S ribosomal RNA gene, partial sequence | 1901 | 1901 | 97% | 0.0 | 99% | KT318824.1 |
| 93Select seq KT318813.1 | Pseudomonas sp. RhizorgB 16S ribosomal RNA gene, partial sequence | 1901 | 1901 | 97% | 0.0 | 99% | KT318813.1 |
| 94Select seq KJ191010.1 | Pseudomonas sp. B055-40 16S ribosomal RNA gene, partial sequence | 1901 | 1901 | 97% | 0.0 | 99% | KJ191010.1 |
| 95Select seq KR061416.1 | Pseudomonas brassicacearum strain 10C-30 16S ribosomal RNA gene, partial sequence | 1901 | 1901 | 97% | 0.0 | 99% | KR061416.1 |
| 96Select seq KR061398.1 | Pseudomonas brassicacearum strain 1Cg-22 16S ribosomal RNA gene, partial sequence | 1901 | 1901 | 97% | 0.0 | 99% | KR061398.1 |
| 97Select seq JN399995.1 | Pseudomonas fluorescens strain J2 16S ribosomal RNA gene, partial sequence | 1901 | 1901 | 97% | 0.0 | 99% | JN399995.1 |
| 98Select seq JX082293.1 | Pseudomonas sp. R25(2012) 16S ribosomal RNA gene, partial sequence | 1901 | 1901 | 97% | 0.0 | 99% | JX082293.1 |
| 99Select seq JQ237663.1 | Pseudomonas sp. hswx161 16S ribosomal RNA gene, partial sequence | 1901 | 1901 | 97% | 0.0 | 99% | JQ237663.1 |
| 100Select seq HQ143608.1 | Pseudomonas sp. strain MTQ15 16S ribosomal RNA gene, partial sequence | 1901 | 1901 | 97% | 0.0 | 99% | HQ143608.1 |


## Alignments

Loading alignment... for sequences gi|115315703,gi|1137473154,gi|1015918342,gi|673537586,gi|858972104 Reading indexes 1-5

Download

FASTA (complete sequence)

FASTA (aligned sequences)

GenBank (complete sequence)

Continue
Cancel

GenBankGraphics

Next
Previous
Descriptions

Pseudomonas brassicacearum isolate MA250 16S ribosomal RNA gene, partial sequence

Sequence ID: DQ886486.1Length: 1515Number of Matches: 1

Related Information

Range 1: 402 to 1444GenBankGraphics

Next Match
Previous Match
First Match

Alignment statistics for match #1

| Score | Expect | Identities | Gaps | Strand | Frame |
| --- | --- | --- | --- | --- | --- |
| 1916 bits(1037) | 0.0() | 1040/1043(99%) | 0/1043(0%) | Plus/Minus |  |

Features:

```
Query  9     CCGTCCTNNCGAAGGTTAGACTAGCTACTTCTGGTGCAACCCACTCCCATGGTGTGACGG  68
             |||||||  |||||||||||||||||||||||||||||||||||||||||||||||||||
Sbjct  1444  CCGTCCTCCCGAAGGTTAGACTAGCTACTTCTGGTGCAACCCACTCCCATGGTGTGACGG  1385

Query  69    GCGGTGTGTACAAGGCCCGGGAACGTATTCACCGCGACATTCTGATTCGCGATTACTAGC  128
             ||||||||||||||||||||||||||||||||||||||||||||||||||||||||||||
Sbjct  1384  GCGGTGTGTACAAGGCCCGGGAACGTATTCACCGCGACATTCTGATTCGCGATTACTAGC  1325

Query  129   GATTCCGACTTCACGCAGTCGAGTTGCAGACTGCGATCCGGACTACGATCGGTTTTGTGG  188
             ||||||||||||||||||||||||||||||||||||||||||||||||||||||||||||
Sbjct  1324  GATTCCGACTTCACGCAGTCGAGTTGCAGACTGCGATCCGGACTACGATCGGTTTTGTGG  1265

Query  189   GATTAGCTCCACCTCGCGGCTTGGCAACCCTCTGTACCGACCATTGTAGCACGTGTGTAG  248
             ||||||||||||||||||||||||||||||||||||||||||||||||||||||||||||
Sbjct  1264  GATTAGCTCCACCTCGCGGCTTGGCAACCCTCTGTACCGACCATTGTAGCACGTGTGTAG  1205

Query  249   CCCAGGCCGTAAGGGCCATGATGACTTGACGTCATCCCCACCTTCCTCCGGTTTGTCACC  308
             ||||||||||||||||||||||||||||||||||||||||||||||||||||||||||||
Sbjct  1204  CCCAGGCCGTAAGGGCCATGATGACTTGACGTCATCCCCACCTTCCTCCGGTTTGTCACC  1145

Query  309   GGCAGTCTCCTTAGAGTGCCCACCATGACGTGCTGGTAACTAAGGACAAGGGTTGCGCTC  368
             ||||||||||||||||||||||||||||||||||||||||||||||||||||||||||||
Sbjct  1144  GGCAGTCTCCTTAGAGTGCCCACCATGACGTGCTGGTAACTAAGGACAAGGGTTGCGCTC  1085

Query  369   GTTACGGGACTTAACCCAACATCTCACGACACGAGCTGACGACAGCCATGCAGCACCTGT  428
             ||||||||||||||||||||||||||||||||||||||||||||||||||||||||||||
Sbjct  1084  GTTACGGGACTTAACCCAACATCTCACGACACGAGCTGACGACAGCCATGCAGCACCTGT  1025

Query  429   CTCAATGNTCCCGAAGGCACCAATCCATCTCTGGAAAGTTCATTGGATGTCAAGGCCTGG  488
             ||||||| ||||||||||||||||||||||||||||||||||||||||||||||||||||
Sbjct  1024  CTCAATGTTCCCGAAGGCACCAATCCATCTCTGGAAAGTTCATTGGATGTCAAGGCCTGG  965

Query  489   TAAGGTTCTTCGCGTTGCTTCGAATTAAACCACATGCTCCACCGCTTGTGCGGGCCCCCG  548
             ||||||||||||||||||||||||||||||||||||||||||||||||||||||||||||
Sbjct  964   TAAGGTTCTTCGCGTTGCTTCGAATTAAACCACATGCTCCACCGCTTGTGCGGGCCCCCG  905

Query  549   TCAATTCATTTGAGTTTTAACCTTGCGGCCGTACTCCCCAGGCGGTCAACTTAATGCGTT  608
             ||||||||||||||||||||||||||||||||||||||||||||||||||||||||||||
Sbjct  904   TCAATTCATTTGAGTTTTAACCTTGCGGCCGTACTCCCCAGGCGGTCAACTTAATGCGTT  845

Query  609   AGCTGCGCCACTAAGAGCTCAAGGCTCCCAACGGCTAGTTGACATCGTTTACGGCGTGGA  668
             ||||||||||||||||||||||||||||||||||||||||||||||||||||||||||||
Sbjct  844   AGCTGCGCCACTAAGAGCTCAAGGCTCCCAACGGCTAGTTGACATCGTTTACGGCGTGGA  785

Query  669   CTACCAGGGTATCTAATCCTGTTTGCTCCCCACGCTTTCGCACCTCAGTGTCAGTATCAG  728
             ||||||||||||||||||||||||||||||||||||||||||||||||||||||||||||
Sbjct  784   CTACCAGGGTATCTAATCCTGTTTGCTCCCCACGCTTTCGCACCTCAGTGTCAGTATCAG  725

Query  729   TCCAGGTGGTCGCCTTCGCCACTGGTGTTCCTTCCTATATCTACGCATTTCACCGCTACA  788
             ||||||||||||||||||||||||||||||||||||||||||||||||||||||||||||
Sbjct  724   TCCAGGTGGTCGCCTTCGCCACTGGTGTTCCTTCCTATATCTACGCATTTCACCGCTACA  665

Query  789   CAGGAAATTCCACCACCCTCTACCATACTCTAGCTCGACAGTTTTGAATGCAGTTCCCAG  848
             ||||||||||||||||||||||||||||||||||||||||||||||||||||||||||||
Sbjct  664   CAGGAAATTCCACCACCCTCTACCATACTCTAGCTCGACAGTTTTGAATGCAGTTCCCAG  605

Query  849   GTTGAGCCCGGGGCTTTCACATCCAACTTAACGAACCACCTACGCGCGCTTTACGCCCAG  908
             ||||||||||||||||||||||||||||||||||||||||||||||||||||||||||||
Sbjct  604   GTTGAGCCCGGGGCTTTCACATCCAACTTAACGAACCACCTACGCGCGCTTTACGCCCAG  545

Query  909   TAATTCCGATTAACGCTTGCACCCTCTGTATTACCGCGGCTGCTGGCACAGAGTTAGCCG  968
             ||||||||||||||||||||||||||||||||||||||||||||||||||||||||||||
Sbjct  544   TAATTCCGATTAACGCTTGCACCCTCTGTATTACCGCGGCTGCTGGCACAGAGTTAGCCG  485

Query  969   GTGCTTATTCTGTCGGTAACGTCAAAACACTAACGTATTAGGTTAATGCCCTTCCTCCCA  1028
             ||||||||||||||||||||||||||||||||||||||||||||||||||||||||||||
Sbjct  484   GTGCTTATTCTGTCGGTAACGTCAAAACACTAACGTATTAGGTTAATGCCCTTCCTCCCA  425

Query  1029  ACTTAAAGTGCTTTACAATCCGA  1051
             |||||||||||||||||||||||
Sbjct  424   ACTTAAAGTGCTTTACAATCCGA  402
```

Download

FASTA (complete sequence)

FASTA (aligned sequences)

GenBank (complete sequence)

Continue
Cancel

GenBankGraphics

Next
Previous
Descriptions

Pseudomonas sp. strain 7.3 16S ribosomal RNA gene, partial sequence

Sequence ID: KY542120.1Length: 1546Number of Matches: 1

Related Information

Range 1: 415 to 1457GenBankGraphics

Next Match
Previous Match
First Match

Alignment statistics for match #1

| Score | Expect | Identities | Gaps | Strand | Frame |
| --- | --- | --- | --- | --- | --- |
| 1910 bits(1034) | 0.0() | 1039/1043(99%) | 0/1043(0%) | Plus/Minus |  |

Features:

```
Query  9     CCGTCCTNNCGAAGGTTAGACTAGCTACTTCTGGTGCAACCCACTCCCATGGTGTGACGG  68
             |||||||  |||||||||||||||||||||||||||||||||||||||||||||||||||
Sbjct  1457  CCGTCCTCCCGAAGGTTAGACTAGCTACTTCTGGTGCAACCCACTCCCATGGTGTGACGG  1398

Query  69    GCGGTGTGTACAAGGCCCGGGAACGTATTCACCGCGACATTCTGATTCGCGATTACTAGC  128
             ||||||||||||||||||||||||||||||||||||||||||||||||||||||||||||
Sbjct  1397  GCGGTGTGTACAAGGCCCGGGAACGTATTCACCGCGACATTCTGATTCGCGATTACTAGC  1338

Query  129   GATTCCGACTTCACGCAGTCGAGTTGCAGACTGCGATCCGGACTACGATCGGTTTTGTGG  188
             ||||||||||||||||||||||||||||||||||||||||||||||||||||||||||||
Sbjct  1337  GATTCCGACTTCACGCAGTCGAGTTGCAGACTGCGATCCGGACTACGATCGGTTTTGTGG  1278

Query  189   GATTAGCTCCACCTCGCGGCTTGGCAACCCTCTGTACCGACCATTGTAGCACGTGTGTAG  248
             ||||||||||||||||||||||||||||||||||||||||||||||||||||||||||||
Sbjct  1277  GATTAGCTCCACCTCGCGGCTTGGCAACCCTCTGTACCGACCATTGTAGCACGTGTGTAG  1218

Query  249   CCCAGGCCGTAAGGGCCATGATGACTTGACGTCATCCCCACCTTCCTCCGGTTTGTCACC  308
             ||||||||||||||||||||||||||||||||||||||||||||||||||||||||||||
Sbjct  1217  CCCAGGCCGTAAGGGCCATGATGACTTGACGTCATCCCCACCTTCCTCCGGTTTGTCACC  1158

Query  309   GGCAGTCTCCTTAGAGTGCCCACCATGACGTGCTGGTAACTAAGGACAAGGGTTGCGCTC  368
             |||||||||||||||||||||||||| |||||||||||||||||||||||||||||||||
Sbjct  1157  GGCAGTCTCCTTAGAGTGCCCACCATAACGTGCTGGTAACTAAGGACAAGGGTTGCGCTC  1098

Query  369   GTTACGGGACTTAACCCAACATCTCACGACACGAGCTGACGACAGCCATGCAGCACCTGT  428
             ||||||||||||||||||||||||||||||||||||||||||||||||||||||||||||
Sbjct  1097  GTTACGGGACTTAACCCAACATCTCACGACACGAGCTGACGACAGCCATGCAGCACCTGT  1038

Query  429   CTCAATGNTCCCGAAGGCACCAATCCATCTCTGGAAAGTTCATTGGATGTCAAGGCCTGG  488
             ||||||| ||||||||||||||||||||||||||||||||||||||||||||||||||||
Sbjct  1037  CTCAATGTTCCCGAAGGCACCAATCCATCTCTGGAAAGTTCATTGGATGTCAAGGCCTGG  978

Query  489   TAAGGTTCTTCGCGTTGCTTCGAATTAAACCACATGCTCCACCGCTTGTGCGGGCCCCCG  548
             ||||||||||||||||||||||||||||||||||||||||||||||||||||||||||||
Sbjct  977   TAAGGTTCTTCGCGTTGCTTCGAATTAAACCACATGCTCCACCGCTTGTGCGGGCCCCCG  918

Query  549   TCAATTCATTTGAGTTTTAACCTTGCGGCCGTACTCCCCAGGCGGTCAACTTAATGCGTT  608
             ||||||||||||||||||||||||||||||||||||||||||||||||||||||||||||
Sbjct  917   TCAATTCATTTGAGTTTTAACCTTGCGGCCGTACTCCCCAGGCGGTCAACTTAATGCGTT  858

Query  609   AGCTGCGCCACTAAGAGCTCAAGGCTCCCAACGGCTAGTTGACATCGTTTACGGCGTGGA  668
             ||||||||||||||||||||||||||||||||||||||||||||||||||||||||||||
Sbjct  857   AGCTGCGCCACTAAGAGCTCAAGGCTCCCAACGGCTAGTTGACATCGTTTACGGCGTGGA  798

Query  669   CTACCAGGGTATCTAATCCTGTTTGCTCCCCACGCTTTCGCACCTCAGTGTCAGTATCAG  728
             ||||||||||||||||||||||||||||||||||||||||||||||||||||||||||||
Sbjct  797   CTACCAGGGTATCTAATCCTGTTTGCTCCCCACGCTTTCGCACCTCAGTGTCAGTATCAG  738

Query  729   TCCAGGTGGTCGCCTTCGCCACTGGTGTTCCTTCCTATATCTACGCATTTCACCGCTACA  788
             ||||||||||||||||||||||||||||||||||||||||||||||||||||||||||||
Sbjct  737   TCCAGGTGGTCGCCTTCGCCACTGGTGTTCCTTCCTATATCTACGCATTTCACCGCTACA  678

Query  789   CAGGAAATTCCACCACCCTCTACCATACTCTAGCTCGACAGTTTTGAATGCAGTTCCCAG  848
             ||||||||||||||||||||||||||||||||||||||||||||||||||||||||||||
Sbjct  677   CAGGAAATTCCACCACCCTCTACCATACTCTAGCTCGACAGTTTTGAATGCAGTTCCCAG  618

Query  849   GTTGAGCCCGGGGCTTTCACATCCAACTTAACGAACCACCTACGCGCGCTTTACGCCCAG  908
             ||||||||||||||||||||||||||||||||||||||||||||||||||||||||||||
Sbjct  617   GTTGAGCCCGGGGCTTTCACATCCAACTTAACGAACCACCTACGCGCGCTTTACGCCCAG  558

Query  909   TAATTCCGATTAACGCTTGCACCCTCTGTATTACCGCGGCTGCTGGCACAGAGTTAGCCG  968
             ||||||||||||||||||||||||||||||||||||||||||||||||||||||||||||
Sbjct  557   TAATTCCGATTAACGCTTGCACCCTCTGTATTACCGCGGCTGCTGGCACAGAGTTAGCCG  498

Query  969   GTGCTTATTCTGTCGGTAACGTCAAAACACTAACGTATTAGGTTAATGCCCTTCCTCCCA  1028
             ||||||||||||||||||||||||||||||||||||||||||||||||||||||||||||
Sbjct  497   GTGCTTATTCTGTCGGTAACGTCAAAACACTAACGTATTAGGTTAATGCCCTTCCTCCCA  438

Query  1029  ACTTAAAGTGCTTTACAATCCGA  1051
             |||||||||||||||||||||||
Sbjct  437   ACTTAAAGTGCTTTACAATCCGA  415
```

Download

FASTA (complete sequence)

FASTA (aligned sequences)

GenBank (complete sequence)

Continue
Cancel

GenBankGraphics

Next
Previous
Descriptions

Pseudomonas fluorescens strain SBR10 16S ribosomal RNA gene, partial sequence

Sequence ID: KX018310.1Length: 1486Number of Matches: 1

Related Information

Range 1: 419 to 1461GenBankGraphics

Next Match
Previous Match
First Match

Alignment statistics for match #1

| Score | Expect | Identities | Gaps | Strand | Frame |
| --- | --- | --- | --- | --- | --- |
| 1910 bits(1034) | 0.0() | 1040/1043(99%) | 0/1043(0%) | Plus/Minus |  |

Features:

```
Query  9     CCGTCCTNNCGAAGGTTAGACTAGCTACTTCTGGTGCAACCCACTCCCATGGTGTGACGG  68
             |||||||  |||||||||||||||||||||||||||||||||||||||||||||||||||
Sbjct  1461  CCGTCCTCCCGAAGGTTAGACTAGCTACTTCTGGTGCAACCCACTCCCATGGTGTGACGG  1402

Query  69    GCGGTGTGTACAAGGCCCGGGAACGTATTCACCGCGACATTCTGATTCGCGATTACTAGC  128
             ||||||||||||||||||||||||||||||||||||||||||||||||||||||||||||
Sbjct  1401  GCGGTGTGTACAAGGCCCGGGAACGTATTCACCGCGACATTCTGATTCGCGATTACTAGC  1342

Query  129   GATTCCGACTTCACGCAGTCGAGTTGCAGACTGCGATCCGGACTACGATCGGTTTTGTGG  188
             ||||||||||||||||||||||||||||||||||||||||||||||||||||||||||||
Sbjct  1341  GATTCCGACTTCACGCAGTCGAGTTGCAGACTGCGATCCGGACTACGATCGGTTTTGTGG  1282

Query  189   GATTAGCTCCACCTCGCGGCTTGGCAACCCTCTGTACCGACCATTGTAGCACGTGTGTAG  248
             ||||||||||||||||||||||||||||||||||||||||||||||||||||||||||||
Sbjct  1281  GATTAGCTCCACCTCGCGGCTTGGCAACCCTCTGTACCGACCATTGTAGCACGTGTGTAG  1222

Query  249   CCCAGGCCGTAAGGGCCATGATGACTTGACGTCATCCCCACCTTCCTCCGGTTTGTCACC  308
             ||||||||||||||||||||||||||||||||||||||||||||||||||||||||||||
Sbjct  1221  CCCAGGCCGTAAGGGCCATGATGACTTGACGTCATCCCCACCTTCCTCCGGTTTGTCACC  1162

Query  309   GGCAGTCTCCTTAGAGTGCCCACCATGACGTGCTGGTAACTAAGGACAAGGGTTGCGCTC  368
             |||||||||||||||||||||||||| |||||||||||||||||||||||||||||||||
Sbjct  1161  GGCAGTCTCCTTAGAGTGCCCACCATAACGTGCTGGTAACTAAGGACAAGGGTTGCGCTC  1102

Query  369   GTTACGGGACTTAACCCAACATCTCACGACACGAGCTGACGACAGCCATGCAGCACCTGT  428
             ||||||||||||||||||||||||||||||||||||||||||||||||||||||||||||
Sbjct  1101  GTTACGGGACTTAACCCAACATCTCACGACACGAGCTGACGACAGCCATGCAGCACCTGT  1042

Query  429   CTCAATGNTCCCGAAGGCACCAATCCATCTCTGGAAAGTTCATTGGATGTCAAGGCCTGG  488
             ||||||||||||||||||||||||||||||||||||||||||||||||||||||||||||
Sbjct  1041  CTCAATGNTCCCGAAGGCACCAATCCATCTCTGGAAAGTTCATTGGATGTCAAGGCCTGG  982

Query  489   TAAGGTTCTTCGCGTTGCTTCGAATTAAACCACATGCTCCACCGCTTGTGCGGGCCCCCG  548
             ||||||||||||||||||||||||||||||||||||||||||||||||||||||||||||
Sbjct  981   TAAGGTTCTTCGCGTTGCTTCGAATTAAACCACATGCTCCACCGCTTGTGCGGGCCCCCG  922

Query  549   TCAATTCATTTGAGTTTTAACCTTGCGGCCGTACTCCCCAGGCGGTCAACTTAATGCGTT  608
             ||||||||||||||||||||||||||||||||||||||||||||||||||||||||||||
Sbjct  921   TCAATTCATTTGAGTTTTAACCTTGCGGCCGTACTCCCCAGGCGGTCAACTTAATGCGTT  862

Query  609   AGCTGCGCCACTAAGAGCTCAAGGCTCCCAACGGCTAGTTGACATCGTTTACGGCGTGGA  668
             ||||||||||||||||||||||||||||||||||||||||||||||||||||||||||||
Sbjct  861   AGCTGCGCCACTAAGAGCTCAAGGCTCCCAACGGCTAGTTGACATCGTTTACGGCGTGGA  802

Query  669   CTACCAGGGTATCTAATCCTGTTTGCTCCCCACGCTTTCGCACCTCAGTGTCAGTATCAG  728
             ||||||||||||||||||||||||||||||||||||||||||||||||||||||||||||
Sbjct  801   CTACCAGGGTATCTAATCCTGTTTGCTCCCCACGCTTTCGCACCTCAGTGTCAGTATCAG  742

Query  729   TCCAGGTGGTCGCCTTCGCCACTGGTGTTCCTTCCTATATCTACGCATTTCACCGCTACA  788
             ||||||||||||||||||||||||||||||||||||||||||||||||||||||||||||
Sbjct  741   TCCAGGTGGTCGCCTTCGCCACTGGTGTTCCTTCCTATATCTACGCATTTCACCGCTACA  682

Query  789   CAGGAAATTCCACCACCCTCTACCATACTCTAGCTCGACAGTTTTGAATGCAGTTCCCAG  848
             ||||||||||||||||||||||||||||||||||||||||||||||||||||||||||||
Sbjct  681   CAGGAAATTCCACCACCCTCTACCATACTCTAGCTCGACAGTTTTGAATGCAGTTCCCAG  622

Query  849   GTTGAGCCCGGGGCTTTCACATCCAACTTAACGAACCACCTACGCGCGCTTTACGCCCAG  908
             ||||||||||||||||||||||||||||||||||||||||||||||||||||||||||||
Sbjct  621   GTTGAGCCCGGGGCTTTCACATCCAACTTAACGAACCACCTACGCGCGCTTTACGCCCAG  562

Query  909   TAATTCCGATTAACGCTTGCACCCTCTGTATTACCGCGGCTGCTGGCACAGAGTTAGCCG  968
             ||||||||||||||||||||||||||||||||||||||||||||||||||||||||||||
Sbjct  561   TAATTCCGATTAACGCTTGCACCCTCTGTATTACCGCGGCTGCTGGCACAGAGTTAGCCG  502

Query  969   GTGCTTATTCTGTCGGTAACGTCAAAACACTAACGTATTAGGTTAATGCCCTTCCTCCCA  1028
             ||||||||||||||||||||||||||||||||||||||||||||||||||||||||||||
Sbjct  501   GTGCTTATTCTGTCGGTAACGTCAAAACACTAACGTATTAGGTTAATGCCCTTCCTCCCA  442

Query  1029  ACTTAAAGTGCTTTACAATCCGA  1051
             |||||||||||||||||||||||
Sbjct  441   ACTTAAAGTGCTTTACAATCCGA  419
```

Download

FASTA (complete sequence)

FASTA (aligned sequences)

GenBank (complete sequence)

Continue
Cancel

GenBankGraphics

Next
Previous
Descriptions

Pseudomonas sp. cpRA293 16S ribosomal RNA gene, partial sequence

Sequence ID: KJ510220.1Length: 1420Number of Matches: 1

Related Information

Range 1: 374 to 1416GenBankGraphics

Next Match
Previous Match
First Match

Alignment statistics for match #1

| Score | Expect | Identities | Gaps | Strand | Frame |
| --- | --- | --- | --- | --- | --- |
| 1910 bits(1034) | 0.0() | 1039/1043(99%) | 0/1043(0%) | Plus/Minus |  |

Features:

```
Query  9     CCGTCCTNNCGAAGGTTAGACTAGCTACTTCTGGTGCAACCCACTCCCATGGTGTGACGG  68
             |||||||  |||||||||||||||||||||||||||||||||||||||||||||||||||
Sbjct  1416  CCGTCCTCCCGAAGGTTAGACTAGCTACTTCTGGTGCAACCCACTCCCATGGTGTGACGG  1357

Query  69    GCGGTGTGTACAAGGCCCGGGAACGTATTCACCGCGACATTCTGATTCGCGATTACTAGC  128
             ||||||||||||||||||||||||||||||||||||||||||||||||||||||||||||
Sbjct  1356  GCGGTGTGTACAAGGCCCGGGAACGTATTCACCGCGACATTCTGATTCGCGATTACTAGC  1297

Query  129   GATTCCGACTTCACGCAGTCGAGTTGCAGACTGCGATCCGGACTACGATCGGTTTTGTGG  188
             ||||||||||||||||||||||||||||||||||||||||||||||||||||||||||||
Sbjct  1296  GATTCCGACTTCACGCAGTCGAGTTGCAGACTGCGATCCGGACTACGATCGGTTTTGTGG  1237

Query  189   GATTAGCTCCACCTCGCGGCTTGGCAACCCTCTGTACCGACCATTGTAGCACGTGTGTAG  248
             ||||||||||||||||||||||||||||||||||||||||||||||||||||||||||||
Sbjct  1236  GATTAGCTCCACCTCGCGGCTTGGCAACCCTCTGTACCGACCATTGTAGCACGTGTGTAG  1177

Query  249   CCCAGGCCGTAAGGGCCATGATGACTTGACGTCATCCCCACCTTCCTCCGGTTTGTCACC  308
             ||||||||||||||||||||||||||||||||||||||||||||||||||||||||||||
Sbjct  1176  CCCAGGCCGTAAGGGCCATGATGACTTGACGTCATCCCCACCTTCCTCCGGTTTGTCACC  1117

Query  309   GGCAGTCTCCTTAGAGTGCCCACCATGACGTGCTGGTAACTAAGGACAAGGGTTGCGCTC  368
             |||||||||||||||||||||||||| |||||||||||||||||||||||||||||||||
Sbjct  1116  GGCAGTCTCCTTAGAGTGCCCACCATAACGTGCTGGTAACTAAGGACAAGGGTTGCGCTC  1057

Query  369   GTTACGGGACTTAACCCAACATCTCACGACACGAGCTGACGACAGCCATGCAGCACCTGT  428
             ||||||||||||||||||||||||||||||||||||||||||||||||||||||||||||
Sbjct  1056  GTTACGGGACTTAACCCAACATCTCACGACACGAGCTGACGACAGCCATGCAGCACCTGT  997

Query  429   CTCAATGNTCCCGAAGGCACCAATCCATCTCTGGAAAGTTCATTGGATGTCAAGGCCTGG  488
             ||||||| ||||||||||||||||||||||||||||||||||||||||||||||||||||
Sbjct  996   CTCAATGTTCCCGAAGGCACCAATCCATCTCTGGAAAGTTCATTGGATGTCAAGGCCTGG  937

Query  489   TAAGGTTCTTCGCGTTGCTTCGAATTAAACCACATGCTCCACCGCTTGTGCGGGCCCCCG  548
             ||||||||||||||||||||||||||||||||||||||||||||||||||||||||||||
Sbjct  936   TAAGGTTCTTCGCGTTGCTTCGAATTAAACCACATGCTCCACCGCTTGTGCGGGCCCCCG  877

Query  549   TCAATTCATTTGAGTTTTAACCTTGCGGCCGTACTCCCCAGGCGGTCAACTTAATGCGTT  608
             ||||||||||||||||||||||||||||||||||||||||||||||||||||||||||||
Sbjct  876   TCAATTCATTTGAGTTTTAACCTTGCGGCCGTACTCCCCAGGCGGTCAACTTAATGCGTT  817

Query  609   AGCTGCGCCACTAAGAGCTCAAGGCTCCCAACGGCTAGTTGACATCGTTTACGGCGTGGA  668
             ||||||||||||||||||||||||||||||||||||||||||||||||||||||||||||
Sbjct  816   AGCTGCGCCACTAAGAGCTCAAGGCTCCCAACGGCTAGTTGACATCGTTTACGGCGTGGA  757

Query  669   CTACCAGGGTATCTAATCCTGTTTGCTCCCCACGCTTTCGCACCTCAGTGTCAGTATCAG  728
             ||||||||||||||||||||||||||||||||||||||||||||||||||||||||||||
Sbjct  756   CTACCAGGGTATCTAATCCTGTTTGCTCCCCACGCTTTCGCACCTCAGTGTCAGTATCAG  697

Query  729   TCCAGGTGGTCGCCTTCGCCACTGGTGTTCCTTCCTATATCTACGCATTTCACCGCTACA  788
             ||||||||||||||||||||||||||||||||||||||||||||||||||||||||||||
Sbjct  696   TCCAGGTGGTCGCCTTCGCCACTGGTGTTCCTTCCTATATCTACGCATTTCACCGCTACA  637

Query  789   CAGGAAATTCCACCACCCTCTACCATACTCTAGCTCGACAGTTTTGAATGCAGTTCCCAG  848
             ||||||||||||||||||||||||||||||||||||||||||||||||||||||||||||
Sbjct  636   CAGGAAATTCCACCACCCTCTACCATACTCTAGCTCGACAGTTTTGAATGCAGTTCCCAG  577

Query  849   GTTGAGCCCGGGGCTTTCACATCCAACTTAACGAACCACCTACGCGCGCTTTACGCCCAG  908
             ||||||||||||||||||||||||||||||||||||||||||||||||||||||||||||
Sbjct  576   GTTGAGCCCGGGGCTTTCACATCCAACTTAACGAACCACCTACGCGCGCTTTACGCCCAG  517

Query  909   TAATTCCGATTAACGCTTGCACCCTCTGTATTACCGCGGCTGCTGGCACAGAGTTAGCCG  968
             ||||||||||||||||||||||||||||||||||||||||||||||||||||||||||||
Sbjct  516   TAATTCCGATTAACGCTTGCACCCTCTGTATTACCGCGGCTGCTGGCACAGAGTTAGCCG  457

Query  969   GTGCTTATTCTGTCGGTAACGTCAAAACACTAACGTATTAGGTTAATGCCCTTCCTCCCA  1028
             ||||||||||||||||||||||||||||||||||||||||||||||||||||||||||||
Sbjct  456   GTGCTTATTCTGTCGGTAACGTCAAAACACTAACGTATTAGGTTAATGCCCTTCCTCCCA  397

Query  1029  ACTTAAAGTGCTTTACAATCCGA  1051
             |||||||||||||||||||||||
Sbjct  396   ACTTAAAGTGCTTTACAATCCGA  374
```

Download

FASTA (complete sequence)

FASTA (aligned sequences)

GenBank (complete sequence)

Continue
Cancel

GenBankGraphics

Next
Previous
Descriptions

Pseudomonas brassicacearum strain Kr21 16S ribosomal RNA gene, partial sequence

Sequence ID: KT215482.1Length: 1410Number of Matches: 1

Related Information

Range 1: 360 to 1402GenBankGraphics

Next Match
Previous Match
First Match

Alignment statistics for match #1

| Score | Expect | Identities | Gaps | Strand | Frame |
| --- | --- | --- | --- | --- | --- |
| 1910 bits(1034) | 0.0() | 1039/1043(99%) | 0/1043(0%) | Plus/Minus |  |

Features:

```
Query  9     CCGTCCTNNCGAAGGTTAGACTAGCTACTTCTGGTGCAACCCACTCCCATGGTGTGACGG  68
             |||||||  |||||||||||||||||||||||||||||||||||||||||||||||||||
Sbjct  1402  CCGTCCTCCCGAAGGTTAGACTAGCTACTTCTGGTGCAACCCACTCCCATGGTGTGACGG  1343

Query  69    GCGGTGTGTACAAGGCCCGGGAACGTATTCACCGCGACATTCTGATTCGCGATTACTAGC  128
             ||||||||||||||||||||||||||||||||||||||||||||||||||||||||||||
Sbjct  1342  GCGGTGTGTACAAGGCCCGGGAACGTATTCACCGCGACATTCTGATTCGCGATTACTAGC  1283

Query  129   GATTCCGACTTCACGCAGTCGAGTTGCAGACTGCGATCCGGACTACGATCGGTTTTGTGG  188
             ||||||||||||||||||||||||||||||||||||||||||||||||||||||||||||
Sbjct  1282  GATTCCGACTTCACGCAGTCGAGTTGCAGACTGCGATCCGGACTACGATCGGTTTTGTGG  1223

Query  189   GATTAGCTCCACCTCGCGGCTTGGCAACCCTCTGTACCGACCATTGTAGCACGTGTGTAG  248
             ||||||||||||||||||||||||||||||||||||||||||||||||||||||||||||
Sbjct  1222  GATTAGCTCCACCTCGCGGCTTGGCAACCCTCTGTACCGACCATTGTAGCACGTGTGTAG  1163

Query  249   CCCAGGCCGTAAGGGCCATGATGACTTGACGTCATCCCCACCTTCCTCCGGTTTGTCACC  308
             ||||||||||||||||||||||||||||||||||||||||||||||||||||||||||||
Sbjct  1162  CCCAGGCCGTAAGGGCCATGATGACTTGACGTCATCCCCACCTTCCTCCGGTTTGTCACC  1103

Query  309   GGCAGTCTCCTTAGAGTGCCCACCATGACGTGCTGGTAACTAAGGACAAGGGTTGCGCTC  368
             ||||||||||||||||||||||||||||||||||||||||||||||||||||||||||||
Sbjct  1102  GGCAGTCTCCTTAGAGTGCCCACCATGACGTGCTGGTAACTAAGGACAAGGGTTGCGCTC  1043

Query  369   GTTACGGGACTTAACCCAACATCTCACGACACGAGCTGACGACAGCCATGCAGCACCTGT  428
             ||||||||||||||||||||||||||||||||||||||||||||||||||||||||||||
Sbjct  1042  GTTACGGGACTTAACCCAACATCTCACGACACGAGCTGACGACAGCCATGCAGCACCTGT  983

Query  429   CTCAATGNTCCCGAAGGCACCAATCCATCTCTGGAAAGTTCATTGGATGTCAAGGCCTGG  488
             ||||||| ||||||||||||||||||||||||||||||||||||||||||||||||||||
Sbjct  982   CTCAATGTTCCCGAAGGCACCAATCCATCTCTGGAAAGTTCATTGGATGTCAAGGCCTGG  923

Query  489   TAAGGTTCTTCGCGTTGCTTCGAATTAAACCACATGCTCCACCGCTTGTGCGGGCCCCCG  548
             ||||||||||||||||||||||||||||||||||||||||||||||||||||||||||||
Sbjct  922   TAAGGTTCTTCGCGTTGCTTCGAATTAAACCACATGCTCCACCGCTTGTGCGGGCCCCCG  863

Query  549   TCAATTCATTTGAGTTTTAACCTTGCGGCCGTACTCCCCAGGCGGTCAACTTAATGCGTT  608
             ||||||||||||||||||||||||||||||||||||||||||||||||||||||||||||
Sbjct  862   TCAATTCATTTGAGTTTTAACCTTGCGGCCGTACTCCCCAGGCGGTCAACTTAATGCGTT  803

Query  609   AGCTGCGCCACTAAGAGCTCAAGGCTCCCAACGGCTAGTTGACATCGTTTACGGCGTGGA  668
             ||||||||||||||||||||||||||||||||||||||||||||||||||||||||||||
Sbjct  802   AGCTGCGCCACTAAGAGCTCAAGGCTCCCAACGGCTAGTTGACATCGTTTACGGCGTGGA  743

Query  669   CTACCAGGGTATCTAATCCTGTTTGCTCCCCACGCTTTCGCACCTCAGTGTCAGTATCAG  728
             ||||||||||||||||||||||||||||||||||||||||||||||||||||||||||||
Sbjct  742   CTACCAGGGTATCTAATCCTGTTTGCTCCCCACGCTTTCGCACCTCAGTGTCAGTATCAG  683

Query  729   TCCAGGTGGTCGCCTTCGCCACTGGTGTTCCTTCCTATATCTACGCATTTCACCGCTACA  788
             ||||||||||||||||||||||||||||||||||||||||||||||||||||||||||||
Sbjct  682   TCCAGGTGGTCGCCTTCGCCACTGGTGTTCCTTCCTATATCTACGCATTTCACCGCTACA  623

Query  789   CAGGAAATTCCACCACCCTCTACCATACTCTAGCTCGACAGTTTTGAATGCAGTTCCCAG  848
             ||||||||||||||||||||||||||||||||||||||||||||||||||||||||||||
Sbjct  622   CAGGAAATTCCACCACCCTCTACCATACTCTAGCTCGACAGTTTTGAATGCAGTTCCCAG  563

Query  849   GTTGAGCCCGGGGCTTTCACATCCAACTTAACGAACCACCTACGCGCGCTTTACGCCCAG  908
             ||||||||||||| ||||||||||||||||||||||||||||||||||||||||||||||
Sbjct  562   GTTGAGCCCGGGGATTTCACATCCAACTTAACGAACCACCTACGCGCGCTTTACGCCCAG  503

Query  909   TAATTCCGATTAACGCTTGCACCCTCTGTATTACCGCGGCTGCTGGCACAGAGTTAGCCG  968
             ||||||||||||||||||||||||||||||||||||||||||||||||||||||||||||
Sbjct  502   TAATTCCGATTAACGCTTGCACCCTCTGTATTACCGCGGCTGCTGGCACAGAGTTAGCCG  443

Query  969   GTGCTTATTCTGTCGGTAACGTCAAAACACTAACGTATTAGGTTAATGCCCTTCCTCCCA  1028
             ||||||||||||||||||||||||||||||||||||||||||||||||||||||||||||
Sbjct  442   GTGCTTATTCTGTCGGTAACGTCAAAACACTAACGTATTAGGTTAATGCCCTTCCTCCCA  383

Query  1029  ACTTAAAGTGCTTTACAATCCGA  1051
             |||||||||||||||||||||||
Sbjct  382   ACTTAAAGTGCTTTACAATCCGA  360
```


BLAST is a registered trademark of the National Library of Medicine

Support center
Mailing list


YouTube

- National Library Of Medicine
- National Institutes Of Health
- U.S. Department of Health & Human Services
- USA.gov

### NCBI


National Center for Biotechnology Information,
 U.S. National Library of Medicine

8600 Rockville Pike,
Bethesda
 MD,
20894
USA

Policies and Guidelines
|
Contact


PreferencesTurn off

External link. Please review our privacy policy.
