# Supplementary material for: Isothermal microcalorimetry for thermal viable count of microorganisms in pure cultures and stabilized formulations
Source: BMC Microbiol. 2019 Mar 21;19:65. doi: 10.1186/s12866-019-1432-8 (PMC6429831; doi:10.1186/s12866-019-1432-8)
Supplement: Supplementary file 3 — 16S rRNA gene sequences’ BLASTN hits in zipped HTML format. (ZIP 15810 kb) [file 12866_2019_1432_MOESM3_ESM.zip › Best blastn hits/NCBI Blast_P pinkish F -- 15..1036 of sequence (1022.html]

NCBI Blast:P pinkish F -- 15..1036 of sequence (1022


- NCBI Home
- Sign in to NCBI
- Skip to Main Content
- Skip to Navigation
- About NCBI Accesskeys

U.S. National Library of Medicine

NCBI
National Center for Biotechnology Information

- My NCBI
- Sign in to NCBI
- Register
- Sign Out

BLAST ^®^ » blastn suite » RID-A50KANSE014


- Home
- Recent Results
- Saved Strategies
- Help

BLAST Results


Edit and Resubmit
Save Search Strategies
[Sign in above to save your search strategy]

Formatting options 


Download


How to read this page
Blast report description
Questions/comments


|  |  |
| --- | --- |
| Formatting options | |
| Show | Alignment as  HTML Plain text   Old View Reset form to defaults [?]  These options control formatting of alignments in results pages. The default is HTML, but other formats (including plain text) are available. PSSM and PssmWithParameters are representations of Position Specific Scoring Matrices and are only available for PSI-BLAST. The Advanced view option allows the database descriptions to be sorted by various indices in a table. |
| Alignment View | Pairwise Pairwise with dots for identities Query-anchored with dots for identities Query-anchored with letters for identities Flat query-anchored with dots for identities Flat query-anchored with letters for identities [?]  Choose how to view alignments. The default "pairwise" view shows how each subject sequence aligns individually to the query sequence. The "query-anchored" view shows how all subject sequences align to the query sequence. For each view type, you can choose to show "identities" (matching residues) as letters or dots. more... |
| Display | Graphical Overview   Linkout   Sequence Retrieval  NCBI-gi   CDS feature [?]  - Graphical Overview: Graphical Overview: Show graph of similar sequence regions aligned to query.   more... - NCBI-gi: Show NCBI gi identifiers. - CDS feature: Show annotated coding region and translation.   more... |
| Masking | Character:   X for protein, n for nucleotide Lower Case  Color:  Black Grey Red [?]  - Masking Character: Display masked (filtered) sequence regions as lower-case or as specific letters (N for nucleotide, P for protein). - Masking Color: Display masked sequence regions in the given color. |
| Limit results | Descriptions:  10 50 100 Graphical overview:  0 10 50 100  Alignments:  0 10 50 100 Line length:  60 90 120 150 [?]  - Descriptions: Show short descriptions for up to the given number of sequences. - Alignments: Show alignments for up to the given number of sequences, in order of statistical significance. - Line lenghth: Number of letters to show on one line in an alignment. |
|  | Organism Type common name, binomial, taxid, or group name. Only 20 top taxa will be shown.     Exclude   [?]  Show only sequences from the given organism. |
|  | Entrez query:  [?]  Show only those sequences that match the given Entrez query. more... |
|  | Expect Min:  Expect Max:  [?]  Show only sequences with expect values in the given range. more... |
|  | Percent Identity Min:  Percent Identity Max:  [?]  Show only sequences with percent identity values in the given range. |
| Format for | PSI-BLAST with inclusion threshold:  [?]  - Format for PSI-BLAST: The Position-Specific Iterated BLAST (PSI-BLAST) program performs iterative searches with a protein query,   in which sequences found in one round of search are used to build a custom score model for the next round.   more... - Inclusion Threshold: This sets the statistical significance threshold for including a sequence in the model used   by PSI-BLAST to create the PSSM on the next iteration. |

|  |  |  |  |  |  |
| --- | --- | --- | --- | --- | --- |
| Download | | | | | |
| Alignment  Text XML ASN.1 JSON Seq-align Hit Table(text) Hit Table(csv) Multiple-file XML2 Single-file XML2 Multiple-file JSON Single-file JSON SAM | Search Strategies  ASN.1 | PSSM to restart search  PSSM | [?] |  |  |

The Download link provides BLAST output that may be used as input to another program.
This includes parseable formats such as the tabular report or XML as well as the Search Strategy files read by the BLAST+ applications.
More details on the parseable (XML, tabular, and ASN.1) reports can be found at
https://www.ncbi.nlm.nih.gov/books/NBK153387/  
  

The following formats are offered under the Alignment section:  
1). "Text". Non-HTML standard BLAST report.  
2). "XML". XML report based upon the DTD at https://www.ncbi.nlm.nih.gov/data\_specs/dtd/NCBI\_BlastOutput.dtd  
3). "ASN.1". Alignment written out in Abstract Syntax Notation 1.  
4). "JSON Seq-align". Alignment written out in JSON.  
4). "Hit Table(text)". The tabular report as text.  
5). "Hit Table(csv)". The tabular report ready for import into spread-sheet programs like Excel.  
6). "XML2". New XML format described at ftp://ftp.ncbi.nlm.nih.gov/blast/documents/NEWXML/xml2.pdf.  
7). "JSON". New JSON format described at ftp://ftp.ncbi.nlm.nih.gov/blast/documents/NEWXML/xml2.pdf.  
8). "SAM". Sequence Alignment Map format.

XML2 and JSON can be downloaded either as one file per query (multiple-file) or one file for all queries (single-file). These formats are listed as Multiple-file XML2 (and JSON) or Single-file XML (and JSON).

The following report is offered under the Search Strategy section:  
1). "ASN.1" Search Strategy. A record of the parameters, query, and database used in the search. This file can be used to start a stand-alone BLAST search, see
https://www.ncbi.nlm.nih.gov/books/NBK1763/#CmdLineAppsManual.I455\_BLAST\_search\_stra


# Job title: P pinkish F -- 15..1036 of sequence (1022

Results for:

lcl|Query\_11919 P pinkish F -- 15..1036 of sequence(1022bp)
[?]

Your BLAST job specified more than one input sequence.
This box lets you choose which input sequence to show BLAST results for.

RID
:   A50KANSE014 (Expires on 03-10 19:47 pm)

Query ID
:   lcl|Query\_11919
:   lcl|Query\_11919

Description
:   P pinkish F -- 15..1036 of sequence

Molecule type
:   nucleic acid

Query Length
:   1022

Database Name
:   nr

Description
:   Nucleotide collection (nt) See details

Program
:   BLASTN 2.8.0+ Citation

  

Reference 

Zheng Zhang, Scott Schwartz, Lukas Wagner, and Webb Miller (2000), "A greedy algorithm for aligning DNA sequences", J Comput Biol 2000; 7(1-2):203-14.

Reference - database indexing

Aleksandr Morgulis, George Coulouris, Yan Raytselis, Thomas L. Madden, Richa Agarwala, Alejandro A. Schäffer (2008), "Database Indexing for Production MegaBLAST Searches", Bioinformatics 24:1757-1764.

Other reports:
Search Summary

[Taxonomy reports]
[Distance tree of results]
[MSA viewer]

Search Parameters

| Search parameter name | Search parameter value |
| --- | --- |
| Program | blastn |
| Word size | 28 |
| Expect value | 10 |
| Hitlist size | 100 |
| Match/Mismatch scores | 1,-2 |
| Gapcosts | 0,2.5 |
| Low Complexity Filter | Yes |
| Filter string | L;m; |
| Genetic Code | 1 |

Database

| Database parameter name | Database parameter value |
| --- | --- |
| Posted date | Mar 7, 2018 1:58 PM |
| Number of letters | 174,044,644,244 |
| Number of sequences | 46,882,714 |
| Entrez query | Includes:  Excludes:  None |

Karlin-Altschul statistics

| Params | Ungapped | Gapped |
| --- | --- | --- |
| Lambda | 1.33271 | 1.28 |
| K | 0.620991 | 0.46 |
| H | 1.12409 | 0.85 |

Results Statistics

| Results Statistics parameter name | Results Statistics parameter value |
| --- | --- |
| Length adjustment | 35 |
| Effective length of query | 987 |
| Effective length of database | 172403749254 |
| Effective search space | 170162500513698 |
| Effective search space used | 170162500513698 |


## Graphic Summary

### Distribution of the top 121 Blast Hits on 100 subject sequences [?]

The graphic is an overview of the database sequences aligned to the query sequence. These are represented horizontal bars colored coded by score and showing the extent
of the alignment on the query sequence. Separate aligned regions on the same database sequence are connected by a thin grey line.
Mousing over an alignment shows the database sequence title. Clicking an alignment displays a box with more details about the alignment and
link to the sequence alignment itself in the Alignments section of the report.

Mouse over to see the title, click to show alignments

Color key for alignment scores

<40

40-50

50-80

80-200

>=200

Query

1

200

400

600

800

1000

Pseudomonas trivialis strain BS3111 genome assembly, ch..

Score:1866 Evalue:0

Accession:LT629760.1

Alignment

Pseudomonas poae strain BS2776 genome assembly, chromos..

Score:1866 Evalue:0

Accession:LT629706.1

Alignment

Pseudomonas poae RE\*1-1-14, complete genome

Score:1866 Evalue:0

Accession:CP004045.1

Alignment

uncultured bacterium partial 16S rRNA gene, clone K15.8..

Score:1866 Evalue:0

Accession:HE576021.1

Alignment

Pseudomonas sp. PX7\_S1 16S ribosomal RNA gene, partial ..

Score:1866 Evalue:0

Accession:JF274937.1

Alignment

Uncultured bacterium clone ncd564g06c1 16S ribosomal RN..

Score:1866 Evalue:0

Accession:HM274624.1

Alignment

Pseudomonas trivialis strain P 513/19 16S ribosomal RNA..

Score:1866 Evalue:0

Accession:NR\_028987.1

Alignment

Pseudomonas poae strain P 527/13 16S ribosomal RNA gene..

Score:1866 Evalue:0

Accession:NR\_028986.1

Alignment

Pseudomonas sp. BBI9 16S ribosomal RNA gene, partial se..

Score:1866 Evalue:0

Accession:GQ868355.1

Alignment

Pseudomonas sp. BR3-39 16S ribosomal RNA gene, partial ..

Score:1866 Evalue:0

Accession:EU853186.1

Alignment

Pseudomonas sp. BR2-22 16S ribosomal RNA gene, partial ..

Score:1866 Evalue:0

Accession:EU853183.1

Alignment

Pseudomonas sp. BR2-02 16S ribosomal RNA gene, partial ..

Score:1866 Evalue:0

Accession:EU853182.1

Alignment

Pseudomonas poae strain T23 16S ribosomal RNA gene, com..

Score:1866 Evalue:0

Accession:EU111704.2

Alignment

Pseudomonas sp. LBSA1 16S ribosomal RNA gene, partial s..

Score:1866 Evalue:0

Accession:AY756059.1

Alignment

Pseudomonas trivialis partial 16S rRNA gene, type strai..

Score:1866 Evalue:0

Accession:AJ492831.1

Alignment

Pseudomonas poae partial 16S rRNA gene, type strain DSM..

Score:1866 Evalue:0

Accession:AJ492829.1

Alignment

Pseudomonas sp. S09G 359 chromosome

Score:1860 Evalue:0

Accession:CP025263.1

Alignment

Pseudomonas sp. strain KBL2 16S ribosomal RNA gene, par..

Score:1860 Evalue:0

Accession:MG576155.1

Alignment

Pseudomonas poae strain A2B5 16S ribosomal RNA gene, pa..

Score:1860 Evalue:0

Accession:KY940313.1

Alignment

Pseudomonas poae strain AAS-144 16S ribosomal RNA gene,..

Score:1860 Evalue:0

Accession:KY810658.1

Alignment

Pseudomonas trivialis strain LMTK28 16S ribosomal RNA g..

Score:1860 Evalue:0

Accession:KY614180.1

Alignment

Pseudomonas poae strain ICE349 16S ribosomal RNA gene, ..

Score:1860 Evalue:0

Accession:KX588598.1

Alignment

Pseudomonas poae strain ICE259 16S ribosomal RNA gene, ..

Score:1860 Evalue:0

Accession:KX588597.1

Alignment

Pseudomonas sp. strain 1-29 16S ribosomal RNA gene, par..

Score:1860 Evalue:0

Accession:KX378900.1

Alignment

Pseudomonas sp. RZ110 16S ribosomal RNA gene, partial s..

Score:1860 Evalue:0

Accession:KT710812.1

Alignment

Pseudomonas sp. 8K8 16S ribosomal RNA gene, partial seq..

Score:1860 Evalue:0

Accession:KT825845.1

Alignment

Pseudomonas sp. 23K33a 16S ribosomal RNA gene, partial ..

Score:1860 Evalue:0

Accession:KT825765.1

Alignment

Pseudomonas sp. 17K8 16S ribosomal RNA gene, partial se..

Score:1860 Evalue:0

Accession:KT825736.1

Alignment

Pseudomonas sp. LB8 16S ribosomal RNA gene, partial seq..

Score:1860 Evalue:0

Accession:KT424973.1

Alignment

Pseudomonas sp. PDD-59b-10 16S ribosomal RNA gene, comp..

Score:1860 Evalue:0

Accession:KR922148.1

Alignment

Pseudomonas poae strain B42 16S ribosomal RNA gene, par..

Score:1860 Evalue:0

Accession:KT767705.1

Alignment

Uncultured Pseudomonas sp. partial 16S rRNA gene, clone..

Score:1860 Evalue:0

Accession:LN875096.1

Alignment

Pseudomonas poae strain IHBB 11151 16S ribosomal RNA ge..

Score:1860 Evalue:0

Accession:KR085862.1

Alignment

Pseudomonas poae strain IHBB 9211 16S ribosomal RNA gen..

Score:1860 Evalue:0

Accession:KR085822.1

Alignment

Uncultured bacterium clone OTU51 16S ribosomal RNA gene..

Score:1860 Evalue:0

Accession:KP975307.1

Alignment

Uncultured bacterium clone 19-16 16S ribosomal RNA gene..

Score:1860 Evalue:0

Accession:KT029380.1

Alignment

Uncultured bacterium clone 19-14 16S ribosomal RNA gene..

Score:1860 Evalue:0

Accession:KT029378.1

Alignment

Pseudomonas trivialis strain IHBB745, complete genome

Score:1860 Evalue:0

Accession:CP011507.1

Alignment

Bacterium MA\_26 16S ribosomal RNA gene, partial sequenc..

Score:1860 Evalue:0

Accession:KP204130.1

Alignment

Pseudomonas poae strain MA\_25 16S ribosomal RNA gene, p..

Score:1860 Evalue:0

Accession:KP204129.1

Alignment

Pseudomonas sp. Bma14 16S ribosomal RNA gene, partial s..

Score:1860 Evalue:0

Accession:JQ977483.1

Alignment

Pseudomonas sp. Ms16 16S ribosomal RNA gene, partial se..

Score:1860 Evalue:0

Accession:JQ977324.1

Alignment

Pseudomonas sp. Ms14 16S ribosomal RNA gene, partial se..

Score:1860 Evalue:0

Accession:JQ977322.1

Alignment

Pseudomonas salomonii strain CCNWQLS112 16S ribosomal R..

Score:1860 Evalue:0

Accession:JX840381.1

Alignment

Pseudomonas poae strain EB20 16S ribosomal RNA gene, pa..

Score:1860 Evalue:0

Accession:JX515573.1

Alignment

Pseudomonas sp. SAP770.2 16S ribosomal RNA gene, partia..

Score:1860 Evalue:0

Accession:JX067683.1

Alignment

Pseudomonas poae strain zol-15 16S ribosomal RNA gene, ..

Score:1860 Evalue:0

Accession:JQ782898.1

Alignment

Pseudomonas sp. FI 1007 16S ribosomal RNA gene, partial..

Score:1860 Evalue:0

Accession:JQ691542.1

Alignment

Pseudomonas sp. ZBC1 16S ribosomal RNA gene, partial se..

Score:1860 Evalue:0

Accession:JQ638271.1

Alignment

Pseudomonas poae strain MML1950 16S ribosomal RNA gene,..

Score:1860 Evalue:0

Accession:JQ336974.1

Alignment

Pseudomonas sp. SGb136 16S ribosomal RNA gene, partial ..

Score:1860 Evalue:0

Accession:HQ224624.1

Alignment

Pseudomonas sp. 5(2011) 16S ribosomal RNA gene, partial..

Score:1860 Evalue:0

Accession:JF808098.1

Alignment

Pseudomonas trivialis strain B24 16S ribosomal RNA gene..

Score:1860 Evalue:0

Accession:JN377670.1

Alignment

Uncultured bacterium clone 3-42017 16S ribosomal RNA ge..

Score:1860 Evalue:0

Accession:JN033052.1

Alignment

Pseudomonas sp. PG-3-10 16S ribosomal RNA gene, partial..

Score:1860 Evalue:0

Accession:JF820107.1

Alignment

Pseudomonas poae strain P6 16S ribosomal RNA gene, part..

Score:1860 Evalue:0

Accession:HQ647258.1

Alignment

## Descriptions

, Reading indexes 1-5, displaying indexes 1-5


Load next setPrevious Match

Sequences producing significant alignments:

Show all columns  of the table presenting sequences producing significant alignments 

Select:AllNone
Selected:0

Alignments
Download

FASTA (complete sequence)

FASTA (aligned sequences)

GenBank (complete sequence)

Hit Table (text)

Hit Table (CSV)

Text

XML

ASN.1

Continue
Cancel

GenBank 
Graphics
Distance tree of results
Multiple alignment
Show/hide columns of the table presenting sequences producing significant alignments 

Available columns

Description  
Max Score  
Total Score  
Coverage  
E-value  
IdentN  
Accession  
Restore Defaults
Ok
Cancel

Sequences producing significant alignments:

| Select for downloading or viewing reports | Description | Max score | Total score | Query cover | E value | Ident | Accession |
| --- | --- | --- | --- | --- | --- | --- | --- |
| 1Select seq LT629760.1 | Pseudomonas trivialis strain BS3111 genome assembly, chromosome: I | 1866 | 9325 | 98% | 0.0 | 100% | LT629760.1 |
| 2Select seq LT629706.1 | Pseudomonas poae strain BS2776 genome assembly, chromosome: I | 1866 | 9331 | 98% | 0.0 | 100% | LT629706.1 |
| 3Select seq CP004045.1 | Pseudomonas poae RE\*1-1-14, complete genome | 1866 | 9325 | 98% | 0.0 | 100% | CP004045.1 |
| 4Select seq HE576021.1 | uncultured bacterium partial 16S rRNA gene, clone K15.87 KSS | 1866 | 1866 | 98% | 0.0 | 100% | HE576021.1 |
| 5Select seq JF274937.1 | Pseudomonas sp. PX7\_S1 16S ribosomal RNA gene, partial sequence | 1866 | 1866 | 98% | 0.0 | 100% | JF274937.1 |
| 6Select seq HM274624.1 | Uncultured bacterium clone ncd564g06c1 16S ribosomal RNA gene, partial sequence | 1866 | 1866 | 98% | 0.0 | 100% | HM274624.1 |
| 7Select seq NR\_028987.1 | Pseudomonas trivialis strain P 513/19 16S ribosomal RNA gene, partial sequence | 1866 | 1866 | 98% | 0.0 | 100% | NR\_028987.1 |
| 8Select seq NR\_028986.1 | Pseudomonas poae strain P 527/13 16S ribosomal RNA gene, partial sequence | 1866 | 1866 | 98% | 0.0 | 100% | NR\_028986.1 |
| 9Select seq GQ868355.1 | Pseudomonas sp. BBI9 16S ribosomal RNA gene, partial sequence | 1866 | 1866 | 98% | 0.0 | 100% | GQ868355.1 |
| 10Select seq EU853186.1 | Pseudomonas sp. BR3-39 16S ribosomal RNA gene, partial sequence | 1866 | 1866 | 98% | 0.0 | 100% | EU853186.1 |
| 11Select seq EU853183.1 | Pseudomonas sp. BR2-22 16S ribosomal RNA gene, partial sequence | 1866 | 1866 | 98% | 0.0 | 100% | EU853183.1 |
| 12Select seq EU853182.1 | Pseudomonas sp. BR2-02 16S ribosomal RNA gene, partial sequence | 1866 | 1866 | 98% | 0.0 | 100% | EU853182.1 |
| 13Select seq EU111704.2 | Pseudomonas poae strain T23 16S ribosomal RNA gene, complete sequence | 1866 | 1866 | 98% | 0.0 | 100% | EU111704.2 |
| 14Select seq AY756059.1 | Pseudomonas sp. LBSA1 16S ribosomal RNA gene, partial sequence; internal transcribed spacer 1, complete sequence; and 23S ribosomal RNA gene, partial sequence | 1866 | 1866 | 98% | 0.0 | 100% | AY756059.1 |
| 15Select seq AJ492831.1 | Pseudomonas trivialis partial 16S rRNA gene, type strain DSM 14937T | 1866 | 1866 | 98% | 0.0 | 100% | AJ492831.1 |
| 16Select seq AJ492829.1 | Pseudomonas poae partial 16S rRNA gene, type strain DSM 14936T | 1866 | 1866 | 98% | 0.0 | 100% | AJ492829.1 |
| 17Select seq CP025263.1 | Pseudomonas sp. S09G 359 chromosome | 1860 | 11158 | 98% | 0.0 | 99% | CP025263.1 |
| 18Select seq MG576155.1 | Pseudomonas sp. strain KBL2 16S ribosomal RNA gene, partial sequence | 1860 | 1860 | 98% | 0.0 | 99% | MG576155.1 |
| 19Select seq KY940313.1 | Pseudomonas poae strain A2B5 16S ribosomal RNA gene, partial sequence | 1860 | 1860 | 98% | 0.0 | 99% | KY940313.1 |
| 20Select seq KY810658.1 | Pseudomonas poae strain AAS-144 16S ribosomal RNA gene, partial sequence | 1860 | 1860 | 98% | 0.0 | 99% | KY810658.1 |
| 21Select seq KY614180.1 | Pseudomonas trivialis strain LMTK28 16S ribosomal RNA gene, partial sequence | 1860 | 1860 | 98% | 0.0 | 99% | KY614180.1 |
| 22Select seq KX588598.1 | Pseudomonas poae strain ICE349 16S ribosomal RNA gene, partial sequence | 1860 | 1860 | 98% | 0.0 | 99% | KX588598.1 |
| 23Select seq KX588597.1 | Pseudomonas poae strain ICE259 16S ribosomal RNA gene, partial sequence | 1860 | 1860 | 98% | 0.0 | 99% | KX588597.1 |
| 24Select seq KX378900.1 | Pseudomonas sp. strain 1-29 16S ribosomal RNA gene, partial sequence | 1860 | 1860 | 98% | 0.0 | 99% | KX378900.1 |
| 25Select seq KT710812.1 | Pseudomonas sp. RZ110 16S ribosomal RNA gene, partial sequence | 1860 | 1860 | 98% | 0.0 | 99% | KT710812.1 |
| 26Select seq KT825845.1 | Pseudomonas sp. 8K8 16S ribosomal RNA gene, partial sequence | 1860 | 1860 | 98% | 0.0 | 99% | KT825845.1 |
| 27Select seq KT825765.1 | Pseudomonas sp. 23K33a 16S ribosomal RNA gene, partial sequence | 1860 | 1860 | 98% | 0.0 | 99% | KT825765.1 |
| 28Select seq KT825736.1 | Pseudomonas sp. 17K8 16S ribosomal RNA gene, partial sequence | 1860 | 1860 | 98% | 0.0 | 99% | KT825736.1 |
| 29Select seq KT424973.1 | Pseudomonas sp. LB8 16S ribosomal RNA gene, partial sequence | 1860 | 1860 | 98% | 0.0 | 99% | KT424973.1 |
| 30Select seq KR922148.1 | Pseudomonas sp. PDD-59b-10 16S ribosomal RNA gene, complete sequence | 1860 | 1860 | 98% | 0.0 | 99% | KR922148.1 |
| 31Select seq KT767705.1 | Pseudomonas poae strain B42 16S ribosomal RNA gene, partial sequence | 1860 | 1860 | 98% | 0.0 | 99% | KT767705.1 |
| 32Select seq LN875096.1 | Uncultured Pseudomonas sp. partial 16S rRNA gene, clone 133X76C\_34 | 1860 | 1860 | 98% | 0.0 | 99% | LN875096.1 |
| 33Select seq KR085862.1 | Pseudomonas poae strain IHBB 11151 16S ribosomal RNA gene, partial sequence | 1860 | 1860 | 98% | 0.0 | 99% | KR085862.1 |
| 34Select seq KR085822.1 | Pseudomonas poae strain IHBB 9211 16S ribosomal RNA gene, partial sequence | 1860 | 1860 | 98% | 0.0 | 99% | KR085822.1 |
| 35Select seq KP975307.1 | Uncultured bacterium clone OTU51 16S ribosomal RNA gene, partial sequence | 1860 | 1860 | 98% | 0.0 | 99% | KP975307.1 |
| 36Select seq KT029380.1 | Uncultured bacterium clone 19-16 16S ribosomal RNA gene, partial sequence | 1860 | 1860 | 98% | 0.0 | 99% | KT029380.1 |
| 37Select seq KT029378.1 | Uncultured bacterium clone 19-14 16S ribosomal RNA gene, partial sequence | 1860 | 1860 | 98% | 0.0 | 99% | KT029378.1 |
| 38Select seq CP011507.1 | Pseudomonas trivialis strain IHBB745, complete genome | 1860 | 9297 | 98% | 0.0 | 99% | CP011507.1 |
| 39Select seq KP204130.1 | Bacterium MA\_26 16S ribosomal RNA gene, partial sequence | 1860 | 1860 | 98% | 0.0 | 99% | KP204130.1 |
| 40Select seq KP204129.1 | Pseudomonas poae strain MA\_25 16S ribosomal RNA gene, partial sequence | 1860 | 1860 | 98% | 0.0 | 99% | KP204129.1 |
| 41Select seq JQ977483.1 | Pseudomonas sp. Bma14 16S ribosomal RNA gene, partial sequence | 1860 | 1860 | 98% | 0.0 | 99% | JQ977483.1 |
| 42Select seq JQ977324.1 | Pseudomonas sp. Ms16 16S ribosomal RNA gene, partial sequence | 1860 | 1860 | 98% | 0.0 | 99% | JQ977324.1 |
| 43Select seq JQ977322.1 | Pseudomonas sp. Ms14 16S ribosomal RNA gene, partial sequence | 1860 | 1860 | 98% | 0.0 | 99% | JQ977322.1 |
| 44Select seq JX840381.1 | Pseudomonas salomonii strain CCNWQLS112 16S ribosomal RNA gene, partial sequence | 1860 | 1860 | 98% | 0.0 | 99% | JX840381.1 |
| 45Select seq JX515573.1 | Pseudomonas poae strain EB20 16S ribosomal RNA gene, partial sequence | 1860 | 1860 | 98% | 0.0 | 99% | JX515573.1 |
| 46Select seq JX067683.1 | Pseudomonas sp. SAP770.2 16S ribosomal RNA gene, partial sequence | 1860 | 1860 | 98% | 0.0 | 99% | JX067683.1 |
| 47Select seq JQ782898.1 | Pseudomonas poae strain zol-15 16S ribosomal RNA gene, partial sequence | 1860 | 1860 | 98% | 0.0 | 99% | JQ782898.1 |
| 48Select seq JQ691542.1 | Pseudomonas sp. FI 1007 16S ribosomal RNA gene, partial sequence | 1860 | 1860 | 98% | 0.0 | 99% | JQ691542.1 |
| 49Select seq JQ638271.1 | Pseudomonas sp. ZBC1 16S ribosomal RNA gene, partial sequence | 1860 | 1860 | 98% | 0.0 | 99% | JQ638271.1 |
| 50Select seq JQ336974.1 | Pseudomonas poae strain MML1950 16S ribosomal RNA gene, partial sequence | 1860 | 1860 | 98% | 0.0 | 99% | JQ336974.1 |
| 51Select seq HQ224624.1 | Pseudomonas sp. SGb136 16S ribosomal RNA gene, partial sequence | 1860 | 1860 | 98% | 0.0 | 99% | HQ224624.1 |
| 52Select seq JF808098.1 | Pseudomonas sp. 5(2011) 16S ribosomal RNA gene, partial sequence | 1860 | 1860 | 98% | 0.0 | 99% | JF808098.1 |
| 53Select seq JN377670.1 | Pseudomonas trivialis strain B24 16S ribosomal RNA gene, partial sequence | 1860 | 1860 | 98% | 0.0 | 99% | JN377670.1 |
| 54Select seq JN033052.1 | Uncultured bacterium clone 3-42017 16S ribosomal RNA gene, partial sequence | 1860 | 1860 | 98% | 0.0 | 99% | JN033052.1 |
| 55Select seq JF820107.1 | Pseudomonas sp. PG-3-10 16S ribosomal RNA gene, partial sequence | 1860 | 1860 | 98% | 0.0 | 99% | JF820107.1 |
| 56Select seq HQ647258.1 | Pseudomonas poae strain P6 16S ribosomal RNA gene, partial sequence | 1860 | 1860 | 98% | 0.0 | 99% | HQ647258.1 |
| 57Select seq HQ443230.1 | Pseudomonas poae strain FBRh6 16S ribosomal RNA gene, partial sequence | 1860 | 1860 | 98% | 0.0 | 99% | HQ443230.1 |
| 58Select seq HM066261.1 | Uncultured bacterium clone EDW07B001\_111 16S ribosomal RNA gene, partial sequence | 1860 | 1860 | 98% | 0.0 | 99% | HM066261.1 |
| 59Select seq HQ256851.1 | Pseudomonas trivialis isolate PDD-32b-53 16S ribosomal RNA gene, partial sequence | 1860 | 1860 | 98% | 0.0 | 99% | HQ256851.1 |
| 60Select seq JF230879.1 | Uncultured bacterium clone ncd2648e11c1 16S ribosomal RNA gene, partial sequence | 1860 | 1860 | 98% | 0.0 | 99% | JF230879.1 |
| 61Select seq JF228956.1 | Uncultured bacterium clone ncd2619g02c1 16S ribosomal RNA gene, partial sequence | 1860 | 1860 | 98% | 0.0 | 99% | JF228956.1 |
| 62Select seq JF209277.1 | Uncultured bacterium clone ncd2405h01c1 16S ribosomal RNA gene, partial sequence | 1860 | 1860 | 98% | 0.0 | 99% | JF209277.1 |
| 63Select seq JF208644.1 | Uncultured bacterium clone ncd2396f05c1 16S ribosomal RNA gene, partial sequence | 1860 | 1860 | 98% | 0.0 | 99% | JF208644.1 |
| 64Select seq JF200837.1 | Uncultured bacterium clone ncd2408f01c1 16S ribosomal RNA gene, partial sequence | 1860 | 1860 | 98% | 0.0 | 99% | JF200837.1 |
| 65Select seq AB607148.1 | Marine bacterium B23 gene for 16S ribosomal RNA, partial sequence | 1860 | 1860 | 98% | 0.0 | 99% | AB607148.1 |
| 66Select seq GU188947.1 | Pseudomonas poae strain BCHCNZ253 16S ribosomal RNA gene, partial sequence | 1860 | 1860 | 98% | 0.0 | 99% | GU188947.1 |
| 67Select seq FJ662886.1 | Pseudomonas sp. VS05\_25 16S ribosomal RNA gene, partial sequence | 1860 | 1860 | 98% | 0.0 | 99% | FJ662886.1 |
| 68Select seq FJ937929.1 | Pseudomonas sp. LS227 16S ribosomal RNA gene, partial sequence | 1860 | 1860 | 98% | 0.0 | 99% | FJ937929.1 |
| 69Select seq FJ937922.1 | Pseudomonas poae strain LS172 16S ribosomal RNA gene, partial sequence | 1860 | 1860 | 98% | 0.0 | 99% | FJ937922.1 |
| 70Select seq FJ179366.1 | Pseudomonas trivialis strain BIHB 750 16S ribosomal RNA gene, partial sequence | 1860 | 1860 | 98% | 0.0 | 99% | FJ179366.1 |
| 71Select seq EU540146.1 | Uncultured bacterium clone nbt246b03 16S ribosomal RNA gene, partial sequence | 1860 | 1860 | 98% | 0.0 | 99% | EU540146.1 |
| 72Select seq EU360313.1 | Pseudomonas fluorescens strain 2312 16S ribosomal RNA gene and 16S-23S ribosomal RNA intergenic spacer, complete sequence; and 23S ribosomal RNA gene, partial sequence | 1860 | 1860 | 98% | 0.0 | 99% | EU360313.1 |
| 73Select seq EF028122.1 | Pseudomonas sp. Dui-7 16S ribosomal RNA gene, partial sequence | 1860 | 1860 | 98% | 0.0 | 99% | EF028122.1 |
| 74Select seq DQ885949.1 | Pseudomonas trivialis strain BIHB 749 16S ribosomal RNA gene, partial sequence | 1860 | 1860 | 98% | 0.0 | 99% | DQ885949.1 |
| 75Select seq DQ536516.1 | Pseudomonas trivialis strain BIHB 745 16S ribosomal RNA gene, partial sequence | 1860 | 1860 | 98% | 0.0 | 99% | DQ536516.1 |
| 76Select seq AY263477.1 | Pseudomonas sp. SE22#1a 16S ribosomal RNA gene, partial sequence | 1860 | 1860 | 98% | 0.0 | 99% | AY263477.1 |
| 77Select seq AY959122.1 | Uncultured bacterium clone rRNA349 16S ribosomal RNA gene, partial sequence | 1860 | 1860 | 98% | 0.0 | 99% | AY959122.1 |
| 78Select seq AY599721.1 | Pseudomonas sp. TB3-6-I 16S ribosomal RNA gene, partial sequence | 1860 | 1860 | 98% | 0.0 | 99% | AY599721.1 |
| 79Select seq AJ556801.1 | Pseudomonas sp. MWH1 partial 16S rRNA gene | 1860 | 1860 | 98% | 0.0 | 99% | AJ556801.1 |
| 80Select seq KX378948.1 | Pseudomonas sp. strain 3-10 16S ribosomal RNA gene, partial sequence | 1858 | 1858 | 98% | 0.0 | 99% | KX378948.1 |
| 81Select seq JQ236630.1 | Pseudomonas poae strain ORC5 16S ribosomal RNA gene, partial sequence | 1858 | 1858 | 98% | 0.0 | 99% | JQ236630.1 |
| 82Select seq GQ868350.1 | Pseudomonas sp. BBI2 16S ribosomal RNA gene, partial sequence | 1858 | 1858 | 98% | 0.0 | 100% | GQ868350.1 |
| 83Select seq KF704111.1 | Pseudomonas trivialis strain KXL-Y3 16S ribosomal RNA gene, partial sequence | 1857 | 1857 | 98% | 0.0 | 99% | KF704111.1 |
| 84Select seq JX569781.1 | Pseudomonas sp. P61-2 16S ribosomal RNA gene, partial sequence | 1857 | 1857 | 98% | 0.0 | 99% | JX569781.1 |
| 85Select seq MG675589.1 | Pseudomonas poae strain W3AP8 16S ribosomal RNA gene, partial sequence | 1855 | 1855 | 98% | 0.0 | 99% | MG675589.1 |
| 86Select seq KY742759.1 | Pseudomonas sp. strain 8.12.1 16S ribosomal RNA gene, partial sequence | 1855 | 1855 | 98% | 0.0 | 99% | KY742759.1 |
| 87Select seq KU529202.1 | Pseudomonas sp. HD34 16S ribosomal RNA gene, partial sequence | 1855 | 1855 | 98% | 0.0 | 99% | KU529202.1 |
| 88Select seq KY681891.1 | Pseudomonas sp. strain 36 16S ribosomal RNA gene, partial sequence | 1855 | 1855 | 98% | 0.0 | 99% | KY681891.1 |
| 89Select seq KY681890.1 | Pseudomonas sp. strain 3 16S ribosomal RNA gene, partial sequence | 1855 | 1855 | 98% | 0.0 | 99% | KY681890.1 |
| 90Select seq KX509247.1 | Uncultured bacterium clone MTWL201306-19 16S ribosomal RNA gene, partial sequence | 1855 | 1855 | 98% | 0.0 | 99% | KX509247.1 |
| 91Select seq KX079774.1 | Pseudomonas sp. MYb117 16S ribosomal RNA gene, partial sequence | 1855 | 1855 | 98% | 0.0 | 99% | KX079774.1 |
| 92Select seq KX079768.1 | Pseudomonas sp. MYb114 16S ribosomal RNA gene, partial sequence | 1855 | 1855 | 98% | 0.0 | 99% | KX079768.1 |
| 93Select seq LC140795.1 | Uncultured Pseudomonas sp. gene for 16S ribosomal RNA, partial sequence, clone: 1d18 | 1855 | 1855 | 98% | 0.0 | 99% | LC140795.1 |
| 94Select seq KT825760.1 | Pseudomonas sp. 23K17 16S ribosomal RNA gene, partial sequence | 1855 | 1855 | 98% | 0.0 | 99% | KT825760.1 |
| 95Select seq HG805797.1 | Pseudomonas sp. FBF128 partial 16S rRNA gene, isolate FBF128 | 1855 | 1855 | 98% | 0.0 | 99% | HG805797.1 |
| 96Select seq HG805781.1 | Pseudomonas sp. FBF110 partial 16S rRNA gene, isolate FBF110 | 1855 | 1855 | 98% | 0.0 | 99% | HG805781.1 |
| 97Select seq KT890311.1 | Pseudomonas sp. S35 16S ribosomal RNA gene, partial sequence | 1855 | 1855 | 98% | 0.0 | 99% | KT890311.1 |
| 98Select seq KR922293.1 | Pseudomonas sp. PDD-69b-25 16S ribosomal RNA gene, complete sequence | 1855 | 1855 | 98% | 0.0 | 99% | KR922293.1 |
| 99Select seq KT767829.1 | Pseudomonas fluorescens strain C40 16S ribosomal RNA gene, partial sequence | 1855 | 1855 | 98% | 0.0 | 99% | KT767829.1 |
| 100Select seq JQ727438.1 | Bacterium enrichment culture clone SaA1 16S ribosomal RNA gene, partial sequence | 1855 | 1855 | 98% | 0.0 | 99% | JQ727438.1 |


## Alignments

Loading alignment... for sequences gi|1085875934,gi|1085717528,gi|445198867,gi|377550157,gi|332000891 Reading indexes 1-5

Download

FASTA (complete sequence)

FASTA (aligned sequences)

GenBank (complete sequence)

Continue
Cancel

GenBankGraphics

Sort by:

E value
 Score
Percent identity
Query start position
Subject start position

Next
Previous
Descriptions

Pseudomonas trivialis strain BS3111 genome assembly, chromosome: I

Sequence ID: LT629760.1Length: 5634667Number of Matches: 5

Related Information

Range 1: 453420 to 454429GenBankGraphics

Next Match
Previous Match
First Match

Alignment statistics for match #1

| Score | Expect | Identities | Gaps | Strand | Frame |
| --- | --- | --- | --- | --- | --- |
| 1866 bits(1010) | 0.0() | 1010/1010(100%) | 0/1010(0%) | Plus/Minus |  |

Features:

rRNA-16S ribosomal RNA . Bacterial SSU

```
Query  9       AGTCGAGCGGTAGAGAGAAGCTTGCTTCTCTTGAGAGCGGCGGACGGGTGAGTAATGCCT  68
               ||||||||||||||||||||||||||||||||||||||||||||||||||||||||||||
Sbjct  454429  AGTCGAGCGGTAGAGAGAAGCTTGCTTCTCTTGAGAGCGGCGGACGGGTGAGTAATGCCT  454370

Query  69      AGGAATCTGCCTGGTAGTGGGGGATAACGTTCGGAAACGGACGCTAATACCGCATACGTC  128
               ||||||||||||||||||||||||||||||||||||||||||||||||||||||||||||
Sbjct  454369  AGGAATCTGCCTGGTAGTGGGGGATAACGTTCGGAAACGGACGCTAATACCGCATACGTC  454310

Query  129     CTACGGGAGAAAGCAGGGGACCTTCGGGCCTTGCGCTATCAGATGAGCCTAGGTCGGATT  188
               ||||||||||||||||||||||||||||||||||||||||||||||||||||||||||||
Sbjct  454309  CTACGGGAGAAAGCAGGGGACCTTCGGGCCTTGCGCTATCAGATGAGCCTAGGTCGGATT  454250

Query  189     AGCTAGTTGGTGGGGTAATGGCTCACCAAGGCGACGATCCGTAACTGGTCTGAGAGGATG  248
               ||||||||||||||||||||||||||||||||||||||||||||||||||||||||||||
Sbjct  454249  AGCTAGTTGGTGGGGTAATGGCTCACCAAGGCGACGATCCGTAACTGGTCTGAGAGGATG  454190

Query  249     ATCAGTCACACTGGAACTGAGACACGGTCCAGACTCCTACGGGAGGCAGCAGTGGGGAAT  308
               ||||||||||||||||||||||||||||||||||||||||||||||||||||||||||||
Sbjct  454189  ATCAGTCACACTGGAACTGAGACACGGTCCAGACTCCTACGGGAGGCAGCAGTGGGGAAT  454130

Query  309     ATTGGACAATGGGCGAAAGCCTGATCCAGCCATGCCGCGTGTGTGAAGAAGGTCTTCGGA  368
               ||||||||||||||||||||||||||||||||||||||||||||||||||||||||||||
Sbjct  454129  ATTGGACAATGGGCGAAAGCCTGATCCAGCCATGCCGCGTGTGTGAAGAAGGTCTTCGGA  454070

Query  369     TTGTAAAGCACTTTAAGTTGGGAGGAAGGGCAGTTACCTAATACGTGATTGTTTTGACGT  428
               ||||||||||||||||||||||||||||||||||||||||||||||||||||||||||||
Sbjct  454069  TTGTAAAGCACTTTAAGTTGGGAGGAAGGGCAGTTACCTAATACGTGATTGTTTTGACGT  454010

Query  429     TACCGACAGAATAAGCACCGGCTAACTCTGTGCCAGCAGCCGCGGTAATACAGAGGGTGC  488
               ||||||||||||||||||||||||||||||||||||||||||||||||||||||||||||
Sbjct  454009  TACCGACAGAATAAGCACCGGCTAACTCTGTGCCAGCAGCCGCGGTAATACAGAGGGTGC  453950

Query  489     AAGCGTTAATCGGAATTACTGGGCGTAAAGCGCGCGTAGGTGGTTTGTTAAGTTGGATGT  548
               ||||||||||||||||||||||||||||||||||||||||||||||||||||||||||||
Sbjct  453949  AAGCGTTAATCGGAATTACTGGGCGTAAAGCGCGCGTAGGTGGTTTGTTAAGTTGGATGT  453890

Query  549     GAAATCCCCGGGCTCAACCTGGGAACTGCATTCAAAACTGACTGACTAGAGTATGGTAGA  608
               ||||||||||||||||||||||||||||||||||||||||||||||||||||||||||||
Sbjct  453889  GAAATCCCCGGGCTCAACCTGGGAACTGCATTCAAAACTGACTGACTAGAGTATGGTAGA  453830

Query  609     GGGTGGTGGAATTTCCTGTGTAGCGGTGAAATGCGTAGATATAGGAAGGAACACCAGTGG  668
               ||||||||||||||||||||||||||||||||||||||||||||||||||||||||||||
Sbjct  453829  GGGTGGTGGAATTTCCTGTGTAGCGGTGAAATGCGTAGATATAGGAAGGAACACCAGTGG  453770

Query  669     CGAAGGCGACCACCTGGACTAATACTGACACTGAGGTGCGAAAGCGTGGGGAGCAAACAG  728
               ||||||||||||||||||||||||||||||||||||||||||||||||||||||||||||
Sbjct  453769  CGAAGGCGACCACCTGGACTAATACTGACACTGAGGTGCGAAAGCGTGGGGAGCAAACAG  453710

Query  729     GATTAGATACCCTGGTAGTCCACGCCGTAAACGATGTCAACTAGCCGTTGGAAGCCTTGA  788
               ||||||||||||||||||||||||||||||||||||||||||||||||||||||||||||
Sbjct  453709  GATTAGATACCCTGGTAGTCCACGCCGTAAACGATGTCAACTAGCCGTTGGAAGCCTTGA  453650

Query  789     GCTTTTAGTGGCGCAGCTAACGCATTAAGTTGACCGCCTGGGGAGTACGGCCGCAAGGTT  848
               ||||||||||||||||||||||||||||||||||||||||||||||||||||||||||||
Sbjct  453649  GCTTTTAGTGGCGCAGCTAACGCATTAAGTTGACCGCCTGGGGAGTACGGCCGCAAGGTT  453590

Query  849     AAAACTCAAATGAATTGACGGGGGCCCGCACAAGCGGTGGAGCATGTGGTTTAATTCGAA  908
               ||||||||||||||||||||||||||||||||||||||||||||||||||||||||||||
Sbjct  453589  AAAACTCAAATGAATTGACGGGGGCCCGCACAAGCGGTGGAGCATGTGGTTTAATTCGAA  453530

Query  909     GCAACGCGAAGAACCTTACCAGGCCTTGACATCCAATGAACTTTCTAGAGATAGATTGGT  968
               ||||||||||||||||||||||||||||||||||||||||||||||||||||||||||||
Sbjct  453529  GCAACGCGAAGAACCTTACCAGGCCTTGACATCCAATGAACTTTCTAGAGATAGATTGGT  453470

Query  969     GCCTTCGGGAACATTGAGACAGGTGCTGCATGGCTGTCGTCAGCTCGTGT  1018
               ||||||||||||||||||||||||||||||||||||||||||||||||||
Sbjct  453469  GCCTTCGGGAACATTGAGACAGGTGCTGCATGGCTGTCGTCAGCTCGTGT  453420
```

Range 2: 812071 to 813080GenBankGraphics

Next Match
Previous Match
First Match

Alignment statistics for match #2

| Score | Expect | Identities | Gaps | Strand | Frame |
| --- | --- | --- | --- | --- | --- |
| 1866 bits(1010) | 0.0() | 1010/1010(100%) | 0/1010(0%) | Plus/Minus |  |

Features:

rRNA-16S ribosomal RNA . Bacterial SSU

```
Query  9       AGTCGAGCGGTAGAGAGAAGCTTGCTTCTCTTGAGAGCGGCGGACGGGTGAGTAATGCCT  68
               ||||||||||||||||||||||||||||||||||||||||||||||||||||||||||||
Sbjct  813080  AGTCGAGCGGTAGAGAGAAGCTTGCTTCTCTTGAGAGCGGCGGACGGGTGAGTAATGCCT  813021

Query  69      AGGAATCTGCCTGGTAGTGGGGGATAACGTTCGGAAACGGACGCTAATACCGCATACGTC  128
               ||||||||||||||||||||||||||||||||||||||||||||||||||||||||||||
Sbjct  813020  AGGAATCTGCCTGGTAGTGGGGGATAACGTTCGGAAACGGACGCTAATACCGCATACGTC  812961

Query  129     CTACGGGAGAAAGCAGGGGACCTTCGGGCCTTGCGCTATCAGATGAGCCTAGGTCGGATT  188
               ||||||||||||||||||||||||||||||||||||||||||||||||||||||||||||
Sbjct  812960  CTACGGGAGAAAGCAGGGGACCTTCGGGCCTTGCGCTATCAGATGAGCCTAGGTCGGATT  812901

Query  189     AGCTAGTTGGTGGGGTAATGGCTCACCAAGGCGACGATCCGTAACTGGTCTGAGAGGATG  248
               ||||||||||||||||||||||||||||||||||||||||||||||||||||||||||||
Sbjct  812900  AGCTAGTTGGTGGGGTAATGGCTCACCAAGGCGACGATCCGTAACTGGTCTGAGAGGATG  812841

Query  249     ATCAGTCACACTGGAACTGAGACACGGTCCAGACTCCTACGGGAGGCAGCAGTGGGGAAT  308
               ||||||||||||||||||||||||||||||||||||||||||||||||||||||||||||
Sbjct  812840  ATCAGTCACACTGGAACTGAGACACGGTCCAGACTCCTACGGGAGGCAGCAGTGGGGAAT  812781

Query  309     ATTGGACAATGGGCGAAAGCCTGATCCAGCCATGCCGCGTGTGTGAAGAAGGTCTTCGGA  368
               ||||||||||||||||||||||||||||||||||||||||||||||||||||||||||||
Sbjct  812780  ATTGGACAATGGGCGAAAGCCTGATCCAGCCATGCCGCGTGTGTGAAGAAGGTCTTCGGA  812721

Query  369     TTGTAAAGCACTTTAAGTTGGGAGGAAGGGCAGTTACCTAATACGTGATTGTTTTGACGT  428
               ||||||||||||||||||||||||||||||||||||||||||||||||||||||||||||
Sbjct  812720  TTGTAAAGCACTTTAAGTTGGGAGGAAGGGCAGTTACCTAATACGTGATTGTTTTGACGT  812661

Query  429     TACCGACAGAATAAGCACCGGCTAACTCTGTGCCAGCAGCCGCGGTAATACAGAGGGTGC  488
               ||||||||||||||||||||||||||||||||||||||||||||||||||||||||||||
Sbjct  812660  TACCGACAGAATAAGCACCGGCTAACTCTGTGCCAGCAGCCGCGGTAATACAGAGGGTGC  812601

Query  489     AAGCGTTAATCGGAATTACTGGGCGTAAAGCGCGCGTAGGTGGTTTGTTAAGTTGGATGT  548
               ||||||||||||||||||||||||||||||||||||||||||||||||||||||||||||
Sbjct  812600  AAGCGTTAATCGGAATTACTGGGCGTAAAGCGCGCGTAGGTGGTTTGTTAAGTTGGATGT  812541

Query  549     GAAATCCCCGGGCTCAACCTGGGAACTGCATTCAAAACTGACTGACTAGAGTATGGTAGA  608
               ||||||||||||||||||||||||||||||||||||||||||||||||||||||||||||
Sbjct  812540  GAAATCCCCGGGCTCAACCTGGGAACTGCATTCAAAACTGACTGACTAGAGTATGGTAGA  812481

Query  609     GGGTGGTGGAATTTCCTGTGTAGCGGTGAAATGCGTAGATATAGGAAGGAACACCAGTGG  668
               ||||||||||||||||||||||||||||||||||||||||||||||||||||||||||||
Sbjct  812480  GGGTGGTGGAATTTCCTGTGTAGCGGTGAAATGCGTAGATATAGGAAGGAACACCAGTGG  812421

Query  669     CGAAGGCGACCACCTGGACTAATACTGACACTGAGGTGCGAAAGCGTGGGGAGCAAACAG  728
               ||||||||||||||||||||||||||||||||||||||||||||||||||||||||||||
Sbjct  812420  CGAAGGCGACCACCTGGACTAATACTGACACTGAGGTGCGAAAGCGTGGGGAGCAAACAG  812361

Query  729     GATTAGATACCCTGGTAGTCCACGCCGTAAACGATGTCAACTAGCCGTTGGAAGCCTTGA  788
               ||||||||||||||||||||||||||||||||||||||||||||||||||||||||||||
Sbjct  812360  GATTAGATACCCTGGTAGTCCACGCCGTAAACGATGTCAACTAGCCGTTGGAAGCCTTGA  812301

Query  789     GCTTTTAGTGGCGCAGCTAACGCATTAAGTTGACCGCCTGGGGAGTACGGCCGCAAGGTT  848
               ||||||||||||||||||||||||||||||||||||||||||||||||||||||||||||
Sbjct  812300  GCTTTTAGTGGCGCAGCTAACGCATTAAGTTGACCGCCTGGGGAGTACGGCCGCAAGGTT  812241

Query  849     AAAACTCAAATGAATTGACGGGGGCCCGCACAAGCGGTGGAGCATGTGGTTTAATTCGAA  908
               ||||||||||||||||||||||||||||||||||||||||||||||||||||||||||||
Sbjct  812240  AAAACTCAAATGAATTGACGGGGGCCCGCACAAGCGGTGGAGCATGTGGTTTAATTCGAA  812181

Query  909     GCAACGCGAAGAACCTTACCAGGCCTTGACATCCAATGAACTTTCTAGAGATAGATTGGT  968
               ||||||||||||||||||||||||||||||||||||||||||||||||||||||||||||
Sbjct  812180  GCAACGCGAAGAACCTTACCAGGCCTTGACATCCAATGAACTTTCTAGAGATAGATTGGT  812121

Query  969     GCCTTCGGGAACATTGAGACAGGTGCTGCATGGCTGTCGTCAGCTCGTGT  1018
               ||||||||||||||||||||||||||||||||||||||||||||||||||
Sbjct  812120  GCCTTCGGGAACATTGAGACAGGTGCTGCATGGCTGTCGTCAGCTCGTGT  812071
```

Range 3: 1552451 to 1553460GenBankGraphics

Next Match
Previous Match
First Match

Alignment statistics for match #3

| Score | Expect | Identities | Gaps | Strand | Frame |
| --- | --- | --- | --- | --- | --- |
| 1866 bits(1010) | 0.0() | 1010/1010(100%) | 0/1010(0%) | Plus/Plus |  |

Features:

rRNA-16S ribosomal RNA . Bacterial SSU

```
Query  9        AGTCGAGCGGTAGAGAGAAGCTTGCTTCTCTTGAGAGCGGCGGACGGGTGAGTAATGCCT  68
                ||||||||||||||||||||||||||||||||||||||||||||||||||||||||||||
Sbjct  1552451  AGTCGAGCGGTAGAGAGAAGCTTGCTTCTCTTGAGAGCGGCGGACGGGTGAGTAATGCCT  1552510

Query  69       AGGAATCTGCCTGGTAGTGGGGGATAACGTTCGGAAACGGACGCTAATACCGCATACGTC  128
                ||||||||||||||||||||||||||||||||||||||||||||||||||||||||||||
Sbjct  1552511  AGGAATCTGCCTGGTAGTGGGGGATAACGTTCGGAAACGGACGCTAATACCGCATACGTC  1552570

Query  129      CTACGGGAGAAAGCAGGGGACCTTCGGGCCTTGCGCTATCAGATGAGCCTAGGTCGGATT  188
                ||||||||||||||||||||||||||||||||||||||||||||||||||||||||||||
Sbjct  1552571  CTACGGGAGAAAGCAGGGGACCTTCGGGCCTTGCGCTATCAGATGAGCCTAGGTCGGATT  1552630

Query  189      AGCTAGTTGGTGGGGTAATGGCTCACCAAGGCGACGATCCGTAACTGGTCTGAGAGGATG  248
                ||||||||||||||||||||||||||||||||||||||||||||||||||||||||||||
Sbjct  1552631  AGCTAGTTGGTGGGGTAATGGCTCACCAAGGCGACGATCCGTAACTGGTCTGAGAGGATG  1552690

Query  249      ATCAGTCACACTGGAACTGAGACACGGTCCAGACTCCTACGGGAGGCAGCAGTGGGGAAT  308
                ||||||||||||||||||||||||||||||||||||||||||||||||||||||||||||
Sbjct  1552691  ATCAGTCACACTGGAACTGAGACACGGTCCAGACTCCTACGGGAGGCAGCAGTGGGGAAT  1552750

Query  309      ATTGGACAATGGGCGAAAGCCTGATCCAGCCATGCCGCGTGTGTGAAGAAGGTCTTCGGA  368
                ||||||||||||||||||||||||||||||||||||||||||||||||||||||||||||
Sbjct  1552751  ATTGGACAATGGGCGAAAGCCTGATCCAGCCATGCCGCGTGTGTGAAGAAGGTCTTCGGA  1552810

Query  369      TTGTAAAGCACTTTAAGTTGGGAGGAAGGGCAGTTACCTAATACGTGATTGTTTTGACGT  428
                ||||||||||||||||||||||||||||||||||||||||||||||||||||||||||||
Sbjct  1552811  TTGTAAAGCACTTTAAGTTGGGAGGAAGGGCAGTTACCTAATACGTGATTGTTTTGACGT  1552870

Query  429      TACCGACAGAATAAGCACCGGCTAACTCTGTGCCAGCAGCCGCGGTAATACAGAGGGTGC  488
                ||||||||||||||||||||||||||||||||||||||||||||||||||||||||||||
Sbjct  1552871  TACCGACAGAATAAGCACCGGCTAACTCTGTGCCAGCAGCCGCGGTAATACAGAGGGTGC  1552930

Query  489      AAGCGTTAATCGGAATTACTGGGCGTAAAGCGCGCGTAGGTGGTTTGTTAAGTTGGATGT  548
                ||||||||||||||||||||||||||||||||||||||||||||||||||||||||||||
Sbjct  1552931  AAGCGTTAATCGGAATTACTGGGCGTAAAGCGCGCGTAGGTGGTTTGTTAAGTTGGATGT  1552990

Query  549      GAAATCCCCGGGCTCAACCTGGGAACTGCATTCAAAACTGACTGACTAGAGTATGGTAGA  608
                ||||||||||||||||||||||||||||||||||||||||||||||||||||||||||||
Sbjct  1552991  GAAATCCCCGGGCTCAACCTGGGAACTGCATTCAAAACTGACTGACTAGAGTATGGTAGA  1553050

Query  609      GGGTGGTGGAATTTCCTGTGTAGCGGTGAAATGCGTAGATATAGGAAGGAACACCAGTGG  668
                ||||||||||||||||||||||||||||||||||||||||||||||||||||||||||||
Sbjct  1553051  GGGTGGTGGAATTTCCTGTGTAGCGGTGAAATGCGTAGATATAGGAAGGAACACCAGTGG  1553110

Query  669      CGAAGGCGACCACCTGGACTAATACTGACACTGAGGTGCGAAAGCGTGGGGAGCAAACAG  728
                ||||||||||||||||||||||||||||||||||||||||||||||||||||||||||||
Sbjct  1553111  CGAAGGCGACCACCTGGACTAATACTGACACTGAGGTGCGAAAGCGTGGGGAGCAAACAG  1553170

Query  729      GATTAGATACCCTGGTAGTCCACGCCGTAAACGATGTCAACTAGCCGTTGGAAGCCTTGA  788
                ||||||||||||||||||||||||||||||||||||||||||||||||||||||||||||
Sbjct  1553171  GATTAGATACCCTGGTAGTCCACGCCGTAAACGATGTCAACTAGCCGTTGGAAGCCTTGA  1553230

Query  789      GCTTTTAGTGGCGCAGCTAACGCATTAAGTTGACCGCCTGGGGAGTACGGCCGCAAGGTT  848
                ||||||||||||||||||||||||||||||||||||||||||||||||||||||||||||
Sbjct  1553231  GCTTTTAGTGGCGCAGCTAACGCATTAAGTTGACCGCCTGGGGAGTACGGCCGCAAGGTT  1553290

Query  849      AAAACTCAAATGAATTGACGGGGGCCCGCACAAGCGGTGGAGCATGTGGTTTAATTCGAA  908
                ||||||||||||||||||||||||||||||||||||||||||||||||||||||||||||
Sbjct  1553291  AAAACTCAAATGAATTGACGGGGGCCCGCACAAGCGGTGGAGCATGTGGTTTAATTCGAA  1553350

Query  909      GCAACGCGAAGAACCTTACCAGGCCTTGACATCCAATGAACTTTCTAGAGATAGATTGGT  968
                ||||||||||||||||||||||||||||||||||||||||||||||||||||||||||||
Sbjct  1553351  GCAACGCGAAGAACCTTACCAGGCCTTGACATCCAATGAACTTTCTAGAGATAGATTGGT  1553410

Query  969      GCCTTCGGGAACATTGAGACAGGTGCTGCATGGCTGTCGTCAGCTCGTGT  1018
                ||||||||||||||||||||||||||||||||||||||||||||||||||
Sbjct  1553411  GCCTTCGGGAACATTGAGACAGGTGCTGCATGGCTGTCGTCAGCTCGTGT  1553460
```

Range 4: 3431046 to 3432055GenBankGraphics

Next Match
Previous Match
First Match

Alignment statistics for match #4

| Score | Expect | Identities | Gaps | Strand | Frame |
| --- | --- | --- | --- | --- | --- |
| 1866 bits(1010) | 0.0() | 1010/1010(100%) | 0/1010(0%) | Plus/Plus |  |

Features:

rRNA-16S ribosomal RNA . Bacterial SSU

```
Query  9        AGTCGAGCGGTAGAGAGAAGCTTGCTTCTCTTGAGAGCGGCGGACGGGTGAGTAATGCCT  68
                ||||||||||||||||||||||||||||||||||||||||||||||||||||||||||||
Sbjct  3431046  AGTCGAGCGGTAGAGAGAAGCTTGCTTCTCTTGAGAGCGGCGGACGGGTGAGTAATGCCT  3431105

Query  69       AGGAATCTGCCTGGTAGTGGGGGATAACGTTCGGAAACGGACGCTAATACCGCATACGTC  128
                ||||||||||||||||||||||||||||||||||||||||||||||||||||||||||||
Sbjct  3431106  AGGAATCTGCCTGGTAGTGGGGGATAACGTTCGGAAACGGACGCTAATACCGCATACGTC  3431165

Query  129      CTACGGGAGAAAGCAGGGGACCTTCGGGCCTTGCGCTATCAGATGAGCCTAGGTCGGATT  188
                ||||||||||||||||||||||||||||||||||||||||||||||||||||||||||||
Sbjct  3431166  CTACGGGAGAAAGCAGGGGACCTTCGGGCCTTGCGCTATCAGATGAGCCTAGGTCGGATT  3431225

Query  189      AGCTAGTTGGTGGGGTAATGGCTCACCAAGGCGACGATCCGTAACTGGTCTGAGAGGATG  248
                ||||||||||||||||||||||||||||||||||||||||||||||||||||||||||||
Sbjct  3431226  AGCTAGTTGGTGGGGTAATGGCTCACCAAGGCGACGATCCGTAACTGGTCTGAGAGGATG  3431285

Query  249      ATCAGTCACACTGGAACTGAGACACGGTCCAGACTCCTACGGGAGGCAGCAGTGGGGAAT  308
                ||||||||||||||||||||||||||||||||||||||||||||||||||||||||||||
Sbjct  3431286  ATCAGTCACACTGGAACTGAGACACGGTCCAGACTCCTACGGGAGGCAGCAGTGGGGAAT  3431345

Query  309      ATTGGACAATGGGCGAAAGCCTGATCCAGCCATGCCGCGTGTGTGAAGAAGGTCTTCGGA  368
                ||||||||||||||||||||||||||||||||||||||||||||||||||||||||||||
Sbjct  3431346  ATTGGACAATGGGCGAAAGCCTGATCCAGCCATGCCGCGTGTGTGAAGAAGGTCTTCGGA  3431405

Query  369      TTGTAAAGCACTTTAAGTTGGGAGGAAGGGCAGTTACCTAATACGTGATTGTTTTGACGT  428
                ||||||||||||||||||||||||||||||||||||||||||||||||||||||||||||
Sbjct  3431406  TTGTAAAGCACTTTAAGTTGGGAGGAAGGGCAGTTACCTAATACGTGATTGTTTTGACGT  3431465

Query  429      TACCGACAGAATAAGCACCGGCTAACTCTGTGCCAGCAGCCGCGGTAATACAGAGGGTGC  488
                ||||||||||||||||||||||||||||||||||||||||||||||||||||||||||||
Sbjct  3431466  TACCGACAGAATAAGCACCGGCTAACTCTGTGCCAGCAGCCGCGGTAATACAGAGGGTGC  3431525

Query  489      AAGCGTTAATCGGAATTACTGGGCGTAAAGCGCGCGTAGGTGGTTTGTTAAGTTGGATGT  548
                ||||||||||||||||||||||||||||||||||||||||||||||||||||||||||||
Sbjct  3431526  AAGCGTTAATCGGAATTACTGGGCGTAAAGCGCGCGTAGGTGGTTTGTTAAGTTGGATGT  3431585

Query  549      GAAATCCCCGGGCTCAACCTGGGAACTGCATTCAAAACTGACTGACTAGAGTATGGTAGA  608
                ||||||||||||||||||||||||||||||||||||||||||||||||||||||||||||
Sbjct  3431586  GAAATCCCCGGGCTCAACCTGGGAACTGCATTCAAAACTGACTGACTAGAGTATGGTAGA  3431645

Query  609      GGGTGGTGGAATTTCCTGTGTAGCGGTGAAATGCGTAGATATAGGAAGGAACACCAGTGG  668
                ||||||||||||||||||||||||||||||||||||||||||||||||||||||||||||
Sbjct  3431646  GGGTGGTGGAATTTCCTGTGTAGCGGTGAAATGCGTAGATATAGGAAGGAACACCAGTGG  3431705

Query  669      CGAAGGCGACCACCTGGACTAATACTGACACTGAGGTGCGAAAGCGTGGGGAGCAAACAG  728
                ||||||||||||||||||||||||||||||||||||||||||||||||||||||||||||
Sbjct  3431706  CGAAGGCGACCACCTGGACTAATACTGACACTGAGGTGCGAAAGCGTGGGGAGCAAACAG  3431765

Query  729      GATTAGATACCCTGGTAGTCCACGCCGTAAACGATGTCAACTAGCCGTTGGAAGCCTTGA  788
                ||||||||||||||||||||||||||||||||||||||||||||||||||||||||||||
Sbjct  3431766  GATTAGATACCCTGGTAGTCCACGCCGTAAACGATGTCAACTAGCCGTTGGAAGCCTTGA  3431825

Query  789      GCTTTTAGTGGCGCAGCTAACGCATTAAGTTGACCGCCTGGGGAGTACGGCCGCAAGGTT  848
                ||||||||||||||||||||||||||||||||||||||||||||||||||||||||||||
Sbjct  3431826  GCTTTTAGTGGCGCAGCTAACGCATTAAGTTGACCGCCTGGGGAGTACGGCCGCAAGGTT  3431885

Query  849      AAAACTCAAATGAATTGACGGGGGCCCGCACAAGCGGTGGAGCATGTGGTTTAATTCGAA  908
                ||||||||||||||||||||||||||||||||||||||||||||||||||||||||||||
Sbjct  3431886  AAAACTCAAATGAATTGACGGGGGCCCGCACAAGCGGTGGAGCATGTGGTTTAATTCGAA  3431945

Query  909      GCAACGCGAAGAACCTTACCAGGCCTTGACATCCAATGAACTTTCTAGAGATAGATTGGT  968
                ||||||||||||||||||||||||||||||||||||||||||||||||||||||||||||
Sbjct  3431946  GCAACGCGAAGAACCTTACCAGGCCTTGACATCCAATGAACTTTCTAGAGATAGATTGGT  3432005

Query  969      GCCTTCGGGAACATTGAGACAGGTGCTGCATGGCTGTCGTCAGCTCGTGT  1018
                ||||||||||||||||||||||||||||||||||||||||||||||||||
Sbjct  3432006  GCCTTCGGGAACATTGAGACAGGTGCTGCATGGCTGTCGTCAGCTCGTGT  3432055
```

Range 5: 2158685 to 2159694GenBankGraphics

Next Match
Previous Match
First Match

Alignment statistics for match #5

| Score | Expect | Identities | Gaps | Strand | Frame |
| --- | --- | --- | --- | --- | --- |
| 1860 bits(1007) | 0.0() | 1009/1010(99%) | 0/1010(0%) | Plus/Plus |  |

Features:

rRNA-16S ribosomal RNA . Bacterial SSU

```
Query  9        AGTCGAGCGGTAGAGAGAAGCTTGCTTCTCTTGAGAGCGGCGGACGGGTGAGTAATGCCT  68
                ||||||||||||||||||||||||||||||||||||||||||||||||||||||||||||
Sbjct  2158685  AGTCGAGCGGTAGAGAGAAGCTTGCTTCTCTTGAGAGCGGCGGACGGGTGAGTAATGCCT  2158744

Query  69       AGGAATCTGCCTGGTAGTGGGGGATAACGTTCGGAAACGGACGCTAATACCGCATACGTC  128
                ||||||||||||||||||||||||||||||||||||||||||||||||||||||||||||
Sbjct  2158745  AGGAATCTGCCTGGTAGTGGGGGATAACGTTCGGAAACGGACGCTAATACCGCATACGTC  2158804

Query  129      CTACGGGAGAAAGCAGGGGACCTTCGGGCCTTGCGCTATCAGATGAGCCTAGGTCGGATT  188
                ||||||||||||||||||||||||||||||||||||||||||||||||||||||||||||
Sbjct  2158805  CTACGGGAGAAAGCAGGGGACCTTCGGGCCTTGCGCTATCAGATGAGCCTAGGTCGGATT  2158864

Query  189      AGCTAGTTGGTGGGGTAATGGCTCACCAAGGCGACGATCCGTAACTGGTCTGAGAGGATG  248
                ||||||||||||||||||||||||||||||||||||||||||||||||||||||||||||
Sbjct  2158865  AGCTAGTTGGTGGGGTAATGGCTCACCAAGGCGACGATCCGTAACTGGTCTGAGAGGATG  2158924

Query  249      ATCAGTCACACTGGAACTGAGACACGGTCCAGACTCCTACGGGAGGCAGCAGTGGGGAAT  308
                ||||||||||||||||||||||||||||||||||||||||||||||||||||||||||||
Sbjct  2158925  ATCAGTCACACTGGAACTGAGACACGGTCCAGACTCCTACGGGAGGCAGCAGTGGGGAAT  2158984

Query  309      ATTGGACAATGGGCGAAAGCCTGATCCAGCCATGCCGCGTGTGTGAAGAAGGTCTTCGGA  368
                ||||||||||||||||||||||||||||||||||||||||||||||||||||||||||||
Sbjct  2158985  ATTGGACAATGGGCGAAAGCCTGATCCAGCCATGCCGCGTGTGTGAAGAAGGTCTTCGGA  2159044

Query  369      TTGTAAAGCACTTTAAGTTGGGAGGAAGGGCAGTTACCTAATACGTGATTGTTTTGACGT  428
                ||||||||||||||||||||||||||||||||||||| ||||||||||||||||||||||
Sbjct  2159045  TTGTAAAGCACTTTAAGTTGGGAGGAAGGGCAGTTACTTAATACGTGATTGTTTTGACGT  2159104

Query  429      TACCGACAGAATAAGCACCGGCTAACTCTGTGCCAGCAGCCGCGGTAATACAGAGGGTGC  488
                ||||||||||||||||||||||||||||||||||||||||||||||||||||||||||||
Sbjct  2159105  TACCGACAGAATAAGCACCGGCTAACTCTGTGCCAGCAGCCGCGGTAATACAGAGGGTGC  2159164

Query  489      AAGCGTTAATCGGAATTACTGGGCGTAAAGCGCGCGTAGGTGGTTTGTTAAGTTGGATGT  548
                ||||||||||||||||||||||||||||||||||||||||||||||||||||||||||||
Sbjct  2159165  AAGCGTTAATCGGAATTACTGGGCGTAAAGCGCGCGTAGGTGGTTTGTTAAGTTGGATGT  2159224

Query  549      GAAATCCCCGGGCTCAACCTGGGAACTGCATTCAAAACTGACTGACTAGAGTATGGTAGA  608
                ||||||||||||||||||||||||||||||||||||||||||||||||||||||||||||
Sbjct  2159225  GAAATCCCCGGGCTCAACCTGGGAACTGCATTCAAAACTGACTGACTAGAGTATGGTAGA  2159284

Query  609      GGGTGGTGGAATTTCCTGTGTAGCGGTGAAATGCGTAGATATAGGAAGGAACACCAGTGG  668
                ||||||||||||||||||||||||||||||||||||||||||||||||||||||||||||
Sbjct  2159285  GGGTGGTGGAATTTCCTGTGTAGCGGTGAAATGCGTAGATATAGGAAGGAACACCAGTGG  2159344

Query  669      CGAAGGCGACCACCTGGACTAATACTGACACTGAGGTGCGAAAGCGTGGGGAGCAAACAG  728
                ||||||||||||||||||||||||||||||||||||||||||||||||||||||||||||
Sbjct  2159345  CGAAGGCGACCACCTGGACTAATACTGACACTGAGGTGCGAAAGCGTGGGGAGCAAACAG  2159404

Query  729      GATTAGATACCCTGGTAGTCCACGCCGTAAACGATGTCAACTAGCCGTTGGAAGCCTTGA  788
                ||||||||||||||||||||||||||||||||||||||||||||||||||||||||||||
Sbjct  2159405  GATTAGATACCCTGGTAGTCCACGCCGTAAACGATGTCAACTAGCCGTTGGAAGCCTTGA  2159464

Query  789      GCTTTTAGTGGCGCAGCTAACGCATTAAGTTGACCGCCTGGGGAGTACGGCCGCAAGGTT  848
                ||||||||||||||||||||||||||||||||||||||||||||||||||||||||||||
Sbjct  2159465  GCTTTTAGTGGCGCAGCTAACGCATTAAGTTGACCGCCTGGGGAGTACGGCCGCAAGGTT  2159524

Query  849      AAAACTCAAATGAATTGACGGGGGCCCGCACAAGCGGTGGAGCATGTGGTTTAATTCGAA  908
                ||||||||||||||||||||||||||||||||||||||||||||||||||||||||||||
Sbjct  2159525  AAAACTCAAATGAATTGACGGGGGCCCGCACAAGCGGTGGAGCATGTGGTTTAATTCGAA  2159584

Query  909      GCAACGCGAAGAACCTTACCAGGCCTTGACATCCAATGAACTTTCTAGAGATAGATTGGT  968
                ||||||||||||||||||||||||||||||||||||||||||||||||||||||||||||
Sbjct  2159585  GCAACGCGAAGAACCTTACCAGGCCTTGACATCCAATGAACTTTCTAGAGATAGATTGGT  2159644

Query  969      GCCTTCGGGAACATTGAGACAGGTGCTGCATGGCTGTCGTCAGCTCGTGT  1018
                ||||||||||||||||||||||||||||||||||||||||||||||||||
Sbjct  2159645  GCCTTCGGGAACATTGAGACAGGTGCTGCATGGCTGTCGTCAGCTCGTGT  2159694
```

Download

FASTA (complete sequence)

FASTA (aligned sequences)

GenBank (complete sequence)

Continue
Cancel

GenBankGraphics

Sort by:

E value
 Score
Percent identity
Query start position
Subject start position

Next
Previous
Descriptions

Pseudomonas poae strain BS2776 genome assembly, chromosome: I

Sequence ID: LT629706.1Length: 5950238Number of Matches: 5

Related Information

Range 1: 1521569 to 1522578GenBankGraphics

Next Match
Previous Match
First Match

Alignment statistics for match #1

| Score | Expect | Identities | Gaps | Strand | Frame |
| --- | --- | --- | --- | --- | --- |
| 1866 bits(1010) | 0.0() | 1010/1010(100%) | 0/1010(0%) | Plus/Minus |  |

Features:

rRNA-16S ribosomal RNA . Bacterial SSU

```
Query  9        AGTCGAGCGGTAGAGAGAAGCTTGCTTCTCTTGAGAGCGGCGGACGGGTGAGTAATGCCT  68
                ||||||||||||||||||||||||||||||||||||||||||||||||||||||||||||
Sbjct  1522578  AGTCGAGCGGTAGAGAGAAGCTTGCTTCTCTTGAGAGCGGCGGACGGGTGAGTAATGCCT  1522519

Query  69       AGGAATCTGCCTGGTAGTGGGGGATAACGTTCGGAAACGGACGCTAATACCGCATACGTC  128
                ||||||||||||||||||||||||||||||||||||||||||||||||||||||||||||
Sbjct  1522518  AGGAATCTGCCTGGTAGTGGGGGATAACGTTCGGAAACGGACGCTAATACCGCATACGTC  1522459

Query  129      CTACGGGAGAAAGCAGGGGACCTTCGGGCCTTGCGCTATCAGATGAGCCTAGGTCGGATT  188
                ||||||||||||||||||||||||||||||||||||||||||||||||||||||||||||
Sbjct  1522458  CTACGGGAGAAAGCAGGGGACCTTCGGGCCTTGCGCTATCAGATGAGCCTAGGTCGGATT  1522399

Query  189      AGCTAGTTGGTGGGGTAATGGCTCACCAAGGCGACGATCCGTAACTGGTCTGAGAGGATG  248
                ||||||||||||||||||||||||||||||||||||||||||||||||||||||||||||
Sbjct  1522398  AGCTAGTTGGTGGGGTAATGGCTCACCAAGGCGACGATCCGTAACTGGTCTGAGAGGATG  1522339

Query  249      ATCAGTCACACTGGAACTGAGACACGGTCCAGACTCCTACGGGAGGCAGCAGTGGGGAAT  308
                ||||||||||||||||||||||||||||||||||||||||||||||||||||||||||||
Sbjct  1522338  ATCAGTCACACTGGAACTGAGACACGGTCCAGACTCCTACGGGAGGCAGCAGTGGGGAAT  1522279

Query  309      ATTGGACAATGGGCGAAAGCCTGATCCAGCCATGCCGCGTGTGTGAAGAAGGTCTTCGGA  368
                ||||||||||||||||||||||||||||||||||||||||||||||||||||||||||||
Sbjct  1522278  ATTGGACAATGGGCGAAAGCCTGATCCAGCCATGCCGCGTGTGTGAAGAAGGTCTTCGGA  1522219

Query  369      TTGTAAAGCACTTTAAGTTGGGAGGAAGGGCAGTTACCTAATACGTGATTGTTTTGACGT  428
                ||||||||||||||||||||||||||||||||||||||||||||||||||||||||||||
Sbjct  1522218  TTGTAAAGCACTTTAAGTTGGGAGGAAGGGCAGTTACCTAATACGTGATTGTTTTGACGT  1522159

Query  429      TACCGACAGAATAAGCACCGGCTAACTCTGTGCCAGCAGCCGCGGTAATACAGAGGGTGC  488
                ||||||||||||||||||||||||||||||||||||||||||||||||||||||||||||
Sbjct  1522158  TACCGACAGAATAAGCACCGGCTAACTCTGTGCCAGCAGCCGCGGTAATACAGAGGGTGC  1522099

Query  489      AAGCGTTAATCGGAATTACTGGGCGTAAAGCGCGCGTAGGTGGTTTGTTAAGTTGGATGT  548
                ||||||||||||||||||||||||||||||||||||||||||||||||||||||||||||
Sbjct  1522098  AAGCGTTAATCGGAATTACTGGGCGTAAAGCGCGCGTAGGTGGTTTGTTAAGTTGGATGT  1522039

Query  549      GAAATCCCCGGGCTCAACCTGGGAACTGCATTCAAAACTGACTGACTAGAGTATGGTAGA  608
                ||||||||||||||||||||||||||||||||||||||||||||||||||||||||||||
Sbjct  1522038  GAAATCCCCGGGCTCAACCTGGGAACTGCATTCAAAACTGACTGACTAGAGTATGGTAGA  1521979

Query  609      GGGTGGTGGAATTTCCTGTGTAGCGGTGAAATGCGTAGATATAGGAAGGAACACCAGTGG  668
                ||||||||||||||||||||||||||||||||||||||||||||||||||||||||||||
Sbjct  1521978  GGGTGGTGGAATTTCCTGTGTAGCGGTGAAATGCGTAGATATAGGAAGGAACACCAGTGG  1521919

Query  669      CGAAGGCGACCACCTGGACTAATACTGACACTGAGGTGCGAAAGCGTGGGGAGCAAACAG  728
                ||||||||||||||||||||||||||||||||||||||||||||||||||||||||||||
Sbjct  1521918  CGAAGGCGACCACCTGGACTAATACTGACACTGAGGTGCGAAAGCGTGGGGAGCAAACAG  1521859

Query  729      GATTAGATACCCTGGTAGTCCACGCCGTAAACGATGTCAACTAGCCGTTGGAAGCCTTGA  788
                ||||||||||||||||||||||||||||||||||||||||||||||||||||||||||||
Sbjct  1521858  GATTAGATACCCTGGTAGTCCACGCCGTAAACGATGTCAACTAGCCGTTGGAAGCCTTGA  1521799

Query  789      GCTTTTAGTGGCGCAGCTAACGCATTAAGTTGACCGCCTGGGGAGTACGGCCGCAAGGTT  848
                ||||||||||||||||||||||||||||||||||||||||||||||||||||||||||||
Sbjct  1521798  GCTTTTAGTGGCGCAGCTAACGCATTAAGTTGACCGCCTGGGGAGTACGGCCGCAAGGTT  1521739

Query  849      AAAACTCAAATGAATTGACGGGGGCCCGCACAAGCGGTGGAGCATGTGGTTTAATTCGAA  908
                ||||||||||||||||||||||||||||||||||||||||||||||||||||||||||||
Sbjct  1521738  AAAACTCAAATGAATTGACGGGGGCCCGCACAAGCGGTGGAGCATGTGGTTTAATTCGAA  1521679

Query  909      GCAACGCGAAGAACCTTACCAGGCCTTGACATCCAATGAACTTTCTAGAGATAGATTGGT  968
                ||||||||||||||||||||||||||||||||||||||||||||||||||||||||||||
Sbjct  1521678  GCAACGCGAAGAACCTTACCAGGCCTTGACATCCAATGAACTTTCTAGAGATAGATTGGT  1521619

Query  969      GCCTTCGGGAACATTGAGACAGGTGCTGCATGGCTGTCGTCAGCTCGTGT  1018
                ||||||||||||||||||||||||||||||||||||||||||||||||||
Sbjct  1521618  GCCTTCGGGAACATTGAGACAGGTGCTGCATGGCTGTCGTCAGCTCGTGT  1521569
```

Range 2: 2816453 to 2817462GenBankGraphics

Next Match
Previous Match
First Match

Alignment statistics for match #2

| Score | Expect | Identities | Gaps | Strand | Frame |
| --- | --- | --- | --- | --- | --- |
| 1866 bits(1010) | 0.0() | 1010/1010(100%) | 0/1010(0%) | Plus/Minus |  |

Features:

rRNA-16S ribosomal RNA . Bacterial SSU

```
Query  9        AGTCGAGCGGTAGAGAGAAGCTTGCTTCTCTTGAGAGCGGCGGACGGGTGAGTAATGCCT  68
                ||||||||||||||||||||||||||||||||||||||||||||||||||||||||||||
Sbjct  2817462  AGTCGAGCGGTAGAGAGAAGCTTGCTTCTCTTGAGAGCGGCGGACGGGTGAGTAATGCCT  2817403

Query  69       AGGAATCTGCCTGGTAGTGGGGGATAACGTTCGGAAACGGACGCTAATACCGCATACGTC  128
                ||||||||||||||||||||||||||||||||||||||||||||||||||||||||||||
Sbjct  2817402  AGGAATCTGCCTGGTAGTGGGGGATAACGTTCGGAAACGGACGCTAATACCGCATACGTC  2817343

Query  129      CTACGGGAGAAAGCAGGGGACCTTCGGGCCTTGCGCTATCAGATGAGCCTAGGTCGGATT  188
                ||||||||||||||||||||||||||||||||||||||||||||||||||||||||||||
Sbjct  2817342  CTACGGGAGAAAGCAGGGGACCTTCGGGCCTTGCGCTATCAGATGAGCCTAGGTCGGATT  2817283

Query  189      AGCTAGTTGGTGGGGTAATGGCTCACCAAGGCGACGATCCGTAACTGGTCTGAGAGGATG  248
                ||||||||||||||||||||||||||||||||||||||||||||||||||||||||||||
Sbjct  2817282  AGCTAGTTGGTGGGGTAATGGCTCACCAAGGCGACGATCCGTAACTGGTCTGAGAGGATG  2817223

Query  249      ATCAGTCACACTGGAACTGAGACACGGTCCAGACTCCTACGGGAGGCAGCAGTGGGGAAT  308
                ||||||||||||||||||||||||||||||||||||||||||||||||||||||||||||
Sbjct  2817222  ATCAGTCACACTGGAACTGAGACACGGTCCAGACTCCTACGGGAGGCAGCAGTGGGGAAT  2817163

Query  309      ATTGGACAATGGGCGAAAGCCTGATCCAGCCATGCCGCGTGTGTGAAGAAGGTCTTCGGA  368
                ||||||||||||||||||||||||||||||||||||||||||||||||||||||||||||
Sbjct  2817162  ATTGGACAATGGGCGAAAGCCTGATCCAGCCATGCCGCGTGTGTGAAGAAGGTCTTCGGA  2817103

Query  369      TTGTAAAGCACTTTAAGTTGGGAGGAAGGGCAGTTACCTAATACGTGATTGTTTTGACGT  428
                ||||||||||||||||||||||||||||||||||||||||||||||||||||||||||||
Sbjct  2817102  TTGTAAAGCACTTTAAGTTGGGAGGAAGGGCAGTTACCTAATACGTGATTGTTTTGACGT  2817043

Query  429      TACCGACAGAATAAGCACCGGCTAACTCTGTGCCAGCAGCCGCGGTAATACAGAGGGTGC  488
                ||||||||||||||||||||||||||||||||||||||||||||||||||||||||||||
Sbjct  2817042  TACCGACAGAATAAGCACCGGCTAACTCTGTGCCAGCAGCCGCGGTAATACAGAGGGTGC  2816983

Query  489      AAGCGTTAATCGGAATTACTGGGCGTAAAGCGCGCGTAGGTGGTTTGTTAAGTTGGATGT  548
                ||||||||||||||||||||||||||||||||||||||||||||||||||||||||||||
Sbjct  2816982  AAGCGTTAATCGGAATTACTGGGCGTAAAGCGCGCGTAGGTGGTTTGTTAAGTTGGATGT  2816923

Query  549      GAAATCCCCGGGCTCAACCTGGGAACTGCATTCAAAACTGACTGACTAGAGTATGGTAGA  608
                ||||||||||||||||||||||||||||||||||||||||||||||||||||||||||||
Sbjct  2816922  GAAATCCCCGGGCTCAACCTGGGAACTGCATTCAAAACTGACTGACTAGAGTATGGTAGA  2816863

Query  609      GGGTGGTGGAATTTCCTGTGTAGCGGTGAAATGCGTAGATATAGGAAGGAACACCAGTGG  668
                ||||||||||||||||||||||||||||||||||||||||||||||||||||||||||||
Sbjct  2816862  GGGTGGTGGAATTTCCTGTGTAGCGGTGAAATGCGTAGATATAGGAAGGAACACCAGTGG  2816803

Query  669      CGAAGGCGACCACCTGGACTAATACTGACACTGAGGTGCGAAAGCGTGGGGAGCAAACAG  728
                ||||||||||||||||||||||||||||||||||||||||||||||||||||||||||||
Sbjct  2816802  CGAAGGCGACCACCTGGACTAATACTGACACTGAGGTGCGAAAGCGTGGGGAGCAAACAG  2816743

Query  729      GATTAGATACCCTGGTAGTCCACGCCGTAAACGATGTCAACTAGCCGTTGGAAGCCTTGA  788
                ||||||||||||||||||||||||||||||||||||||||||||||||||||||||||||
Sbjct  2816742  GATTAGATACCCTGGTAGTCCACGCCGTAAACGATGTCAACTAGCCGTTGGAAGCCTTGA  2816683

Query  789      GCTTTTAGTGGCGCAGCTAACGCATTAAGTTGACCGCCTGGGGAGTACGGCCGCAAGGTT  848
                ||||||||||||||||||||||||||||||||||||||||||||||||||||||||||||
Sbjct  2816682  GCTTTTAGTGGCGCAGCTAACGCATTAAGTTGACCGCCTGGGGAGTACGGCCGCAAGGTT  2816623

Query  849      AAAACTCAAATGAATTGACGGGGGCCCGCACAAGCGGTGGAGCATGTGGTTTAATTCGAA  908
                ||||||||||||||||||||||||||||||||||||||||||||||||||||||||||||
Sbjct  2816622  AAAACTCAAATGAATTGACGGGGGCCCGCACAAGCGGTGGAGCATGTGGTTTAATTCGAA  2816563

Query  909      GCAACGCGAAGAACCTTACCAGGCCTTGACATCCAATGAACTTTCTAGAGATAGATTGGT  968
                ||||||||||||||||||||||||||||||||||||||||||||||||||||||||||||
Sbjct  2816562  GCAACGCGAAGAACCTTACCAGGCCTTGACATCCAATGAACTTTCTAGAGATAGATTGGT  2816503

Query  969      GCCTTCGGGAACATTGAGACAGGTGCTGCATGGCTGTCGTCAGCTCGTGT  1018
                ||||||||||||||||||||||||||||||||||||||||||||||||||
Sbjct  2816502  GCCTTCGGGAACATTGAGACAGGTGCTGCATGGCTGTCGTCAGCTCGTGT  2816453
```

Range 3: 3457529 to 3458538GenBankGraphics

Next Match
Previous Match
First Match

Alignment statistics for match #3

| Score | Expect | Identities | Gaps | Strand | Frame |
| --- | --- | --- | --- | --- | --- |
| 1866 bits(1010) | 0.0() | 1010/1010(100%) | 0/1010(0%) | Plus/Minus |  |

Features:

rRNA-16S ribosomal RNA . Bacterial SSU

```
Query  9        AGTCGAGCGGTAGAGAGAAGCTTGCTTCTCTTGAGAGCGGCGGACGGGTGAGTAATGCCT  68
                ||||||||||||||||||||||||||||||||||||||||||||||||||||||||||||
Sbjct  3458538  AGTCGAGCGGTAGAGAGAAGCTTGCTTCTCTTGAGAGCGGCGGACGGGTGAGTAATGCCT  3458479

Query  69       AGGAATCTGCCTGGTAGTGGGGGATAACGTTCGGAAACGGACGCTAATACCGCATACGTC  128
                ||||||||||||||||||||||||||||||||||||||||||||||||||||||||||||
Sbjct  3458478  AGGAATCTGCCTGGTAGTGGGGGATAACGTTCGGAAACGGACGCTAATACCGCATACGTC  3458419

Query  129      CTACGGGAGAAAGCAGGGGACCTTCGGGCCTTGCGCTATCAGATGAGCCTAGGTCGGATT  188
                ||||||||||||||||||||||||||||||||||||||||||||||||||||||||||||
Sbjct  3458418  CTACGGGAGAAAGCAGGGGACCTTCGGGCCTTGCGCTATCAGATGAGCCTAGGTCGGATT  3458359

Query  189      AGCTAGTTGGTGGGGTAATGGCTCACCAAGGCGACGATCCGTAACTGGTCTGAGAGGATG  248
                ||||||||||||||||||||||||||||||||||||||||||||||||||||||||||||
Sbjct  3458358  AGCTAGTTGGTGGGGTAATGGCTCACCAAGGCGACGATCCGTAACTGGTCTGAGAGGATG  3458299

Query  249      ATCAGTCACACTGGAACTGAGACACGGTCCAGACTCCTACGGGAGGCAGCAGTGGGGAAT  308
                ||||||||||||||||||||||||||||||||||||||||||||||||||||||||||||
Sbjct  3458298  ATCAGTCACACTGGAACTGAGACACGGTCCAGACTCCTACGGGAGGCAGCAGTGGGGAAT  3458239

Query  309      ATTGGACAATGGGCGAAAGCCTGATCCAGCCATGCCGCGTGTGTGAAGAAGGTCTTCGGA  368
                ||||||||||||||||||||||||||||||||||||||||||||||||||||||||||||
Sbjct  3458238  ATTGGACAATGGGCGAAAGCCTGATCCAGCCATGCCGCGTGTGTGAAGAAGGTCTTCGGA  3458179

Query  369      TTGTAAAGCACTTTAAGTTGGGAGGAAGGGCAGTTACCTAATACGTGATTGTTTTGACGT  428
                ||||||||||||||||||||||||||||||||||||||||||||||||||||||||||||
Sbjct  3458178  TTGTAAAGCACTTTAAGTTGGGAGGAAGGGCAGTTACCTAATACGTGATTGTTTTGACGT  3458119

Query  429      TACCGACAGAATAAGCACCGGCTAACTCTGTGCCAGCAGCCGCGGTAATACAGAGGGTGC  488
                ||||||||||||||||||||||||||||||||||||||||||||||||||||||||||||
Sbjct  3458118  TACCGACAGAATAAGCACCGGCTAACTCTGTGCCAGCAGCCGCGGTAATACAGAGGGTGC  3458059

Query  489      AAGCGTTAATCGGAATTACTGGGCGTAAAGCGCGCGTAGGTGGTTTGTTAAGTTGGATGT  548
                ||||||||||||||||||||||||||||||||||||||||||||||||||||||||||||
Sbjct  3458058  AAGCGTTAATCGGAATTACTGGGCGTAAAGCGCGCGTAGGTGGTTTGTTAAGTTGGATGT  3457999

Query  549      GAAATCCCCGGGCTCAACCTGGGAACTGCATTCAAAACTGACTGACTAGAGTATGGTAGA  608
                ||||||||||||||||||||||||||||||||||||||||||||||||||||||||||||
Sbjct  3457998  GAAATCCCCGGGCTCAACCTGGGAACTGCATTCAAAACTGACTGACTAGAGTATGGTAGA  3457939

Query  609      GGGTGGTGGAATTTCCTGTGTAGCGGTGAAATGCGTAGATATAGGAAGGAACACCAGTGG  668
                ||||||||||||||||||||||||||||||||||||||||||||||||||||||||||||
Sbjct  3457938  GGGTGGTGGAATTTCCTGTGTAGCGGTGAAATGCGTAGATATAGGAAGGAACACCAGTGG  3457879

Query  669      CGAAGGCGACCACCTGGACTAATACTGACACTGAGGTGCGAAAGCGTGGGGAGCAAACAG  728
                ||||||||||||||||||||||||||||||||||||||||||||||||||||||||||||
Sbjct  3457878  CGAAGGCGACCACCTGGACTAATACTGACACTGAGGTGCGAAAGCGTGGGGAGCAAACAG  3457819

Query  729      GATTAGATACCCTGGTAGTCCACGCCGTAAACGATGTCAACTAGCCGTTGGAAGCCTTGA  788
                ||||||||||||||||||||||||||||||||||||||||||||||||||||||||||||
Sbjct  3457818  GATTAGATACCCTGGTAGTCCACGCCGTAAACGATGTCAACTAGCCGTTGGAAGCCTTGA  3457759

Query  789      GCTTTTAGTGGCGCAGCTAACGCATTAAGTTGACCGCCTGGGGAGTACGGCCGCAAGGTT  848
                ||||||||||||||||||||||||||||||||||||||||||||||||||||||||||||
Sbjct  3457758  GCTTTTAGTGGCGCAGCTAACGCATTAAGTTGACCGCCTGGGGAGTACGGCCGCAAGGTT  3457699

Query  849      AAAACTCAAATGAATTGACGGGGGCCCGCACAAGCGGTGGAGCATGTGGTTTAATTCGAA  908
                ||||||||||||||||||||||||||||||||||||||||||||||||||||||||||||
Sbjct  3457698  AAAACTCAAATGAATTGACGGGGGCCCGCACAAGCGGTGGAGCATGTGGTTTAATTCGAA  3457639

Query  909      GCAACGCGAAGAACCTTACCAGGCCTTGACATCCAATGAACTTTCTAGAGATAGATTGGT  968
                ||||||||||||||||||||||||||||||||||||||||||||||||||||||||||||
Sbjct  3457638  GCAACGCGAAGAACCTTACCAGGCCTTGACATCCAATGAACTTTCTAGAGATAGATTGGT  3457579

Query  969      GCCTTCGGGAACATTGAGACAGGTGCTGCATGGCTGTCGTCAGCTCGTGT  1018
                ||||||||||||||||||||||||||||||||||||||||||||||||||
Sbjct  3457578  GCCTTCGGGAACATTGAGACAGGTGCTGCATGGCTGTCGTCAGCTCGTGT  3457529
```

Range 4: 4255116 to 4256125GenBankGraphics

Next Match
Previous Match
First Match

Alignment statistics for match #4

| Score | Expect | Identities | Gaps | Strand | Frame |
| --- | --- | --- | --- | --- | --- |
| 1866 bits(1010) | 0.0() | 1010/1010(100%) | 0/1010(0%) | Plus/Plus |  |

Features:

rRNA-16S ribosomal RNA . Bacterial SSU

```
Query  9        AGTCGAGCGGTAGAGAGAAGCTTGCTTCTCTTGAGAGCGGCGGACGGGTGAGTAATGCCT  68
                ||||||||||||||||||||||||||||||||||||||||||||||||||||||||||||
Sbjct  4255116  AGTCGAGCGGTAGAGAGAAGCTTGCTTCTCTTGAGAGCGGCGGACGGGTGAGTAATGCCT  4255175

Query  69       AGGAATCTGCCTGGTAGTGGGGGATAACGTTCGGAAACGGACGCTAATACCGCATACGTC  128
                ||||||||||||||||||||||||||||||||||||||||||||||||||||||||||||
Sbjct  4255176  AGGAATCTGCCTGGTAGTGGGGGATAACGTTCGGAAACGGACGCTAATACCGCATACGTC  4255235

Query  129      CTACGGGAGAAAGCAGGGGACCTTCGGGCCTTGCGCTATCAGATGAGCCTAGGTCGGATT  188
                ||||||||||||||||||||||||||||||||||||||||||||||||||||||||||||
Sbjct  4255236  CTACGGGAGAAAGCAGGGGACCTTCGGGCCTTGCGCTATCAGATGAGCCTAGGTCGGATT  4255295

Query  189      AGCTAGTTGGTGGGGTAATGGCTCACCAAGGCGACGATCCGTAACTGGTCTGAGAGGATG  248
                ||||||||||||||||||||||||||||||||||||||||||||||||||||||||||||
Sbjct  4255296  AGCTAGTTGGTGGGGTAATGGCTCACCAAGGCGACGATCCGTAACTGGTCTGAGAGGATG  4255355

Query  249      ATCAGTCACACTGGAACTGAGACACGGTCCAGACTCCTACGGGAGGCAGCAGTGGGGAAT  308
                ||||||||||||||||||||||||||||||||||||||||||||||||||||||||||||
Sbjct  4255356  ATCAGTCACACTGGAACTGAGACACGGTCCAGACTCCTACGGGAGGCAGCAGTGGGGAAT  4255415

Query  309      ATTGGACAATGGGCGAAAGCCTGATCCAGCCATGCCGCGTGTGTGAAGAAGGTCTTCGGA  368
                ||||||||||||||||||||||||||||||||||||||||||||||||||||||||||||
Sbjct  4255416  ATTGGACAATGGGCGAAAGCCTGATCCAGCCATGCCGCGTGTGTGAAGAAGGTCTTCGGA  4255475

Query  369      TTGTAAAGCACTTTAAGTTGGGAGGAAGGGCAGTTACCTAATACGTGATTGTTTTGACGT  428
                ||||||||||||||||||||||||||||||||||||||||||||||||||||||||||||
Sbjct  4255476  TTGTAAAGCACTTTAAGTTGGGAGGAAGGGCAGTTACCTAATACGTGATTGTTTTGACGT  4255535

Query  429      TACCGACAGAATAAGCACCGGCTAACTCTGTGCCAGCAGCCGCGGTAATACAGAGGGTGC  488
                ||||||||||||||||||||||||||||||||||||||||||||||||||||||||||||
Sbjct  4255536  TACCGACAGAATAAGCACCGGCTAACTCTGTGCCAGCAGCCGCGGTAATACAGAGGGTGC  4255595

Query  489      AAGCGTTAATCGGAATTACTGGGCGTAAAGCGCGCGTAGGTGGTTTGTTAAGTTGGATGT  548
                ||||||||||||||||||||||||||||||||||||||||||||||||||||||||||||
Sbjct  4255596  AAGCGTTAATCGGAATTACTGGGCGTAAAGCGCGCGTAGGTGGTTTGTTAAGTTGGATGT  4255655

Query  549      GAAATCCCCGGGCTCAACCTGGGAACTGCATTCAAAACTGACTGACTAGAGTATGGTAGA  608
                ||||||||||||||||||||||||||||||||||||||||||||||||||||||||||||
Sbjct  4255656  GAAATCCCCGGGCTCAACCTGGGAACTGCATTCAAAACTGACTGACTAGAGTATGGTAGA  4255715

Query  609      GGGTGGTGGAATTTCCTGTGTAGCGGTGAAATGCGTAGATATAGGAAGGAACACCAGTGG  668
                ||||||||||||||||||||||||||||||||||||||||||||||||||||||||||||
Sbjct  4255716  GGGTGGTGGAATTTCCTGTGTAGCGGTGAAATGCGTAGATATAGGAAGGAACACCAGTGG  4255775

Query  669      CGAAGGCGACCACCTGGACTAATACTGACACTGAGGTGCGAAAGCGTGGGGAGCAAACAG  728
                ||||||||||||||||||||||||||||||||||||||||||||||||||||||||||||
Sbjct  4255776  CGAAGGCGACCACCTGGACTAATACTGACACTGAGGTGCGAAAGCGTGGGGAGCAAACAG  4255835

Query  729      GATTAGATACCCTGGTAGTCCACGCCGTAAACGATGTCAACTAGCCGTTGGAAGCCTTGA  788
                ||||||||||||||||||||||||||||||||||||||||||||||||||||||||||||
Sbjct  4255836  GATTAGATACCCTGGTAGTCCACGCCGTAAACGATGTCAACTAGCCGTTGGAAGCCTTGA  4255895

Query  789      GCTTTTAGTGGCGCAGCTAACGCATTAAGTTGACCGCCTGGGGAGTACGGCCGCAAGGTT  848
                ||||||||||||||||||||||||||||||||||||||||||||||||||||||||||||
Sbjct  4255896  GCTTTTAGTGGCGCAGCTAACGCATTAAGTTGACCGCCTGGGGAGTACGGCCGCAAGGTT  4255955

Query  849      AAAACTCAAATGAATTGACGGGGGCCCGCACAAGCGGTGGAGCATGTGGTTTAATTCGAA  908
                ||||||||||||||||||||||||||||||||||||||||||||||||||||||||||||
Sbjct  4255956  AAAACTCAAATGAATTGACGGGGGCCCGCACAAGCGGTGGAGCATGTGGTTTAATTCGAA  4256015

Query  909      GCAACGCGAAGAACCTTACCAGGCCTTGACATCCAATGAACTTTCTAGAGATAGATTGGT  968
                ||||||||||||||||||||||||||||||||||||||||||||||||||||||||||||
Sbjct  4256016  GCAACGCGAAGAACCTTACCAGGCCTTGACATCCAATGAACTTTCTAGAGATAGATTGGT  4256075

Query  969      GCCTTCGGGAACATTGAGACAGGTGCTGCATGGCTGTCGTCAGCTCGTGT  1018
                ||||||||||||||||||||||||||||||||||||||||||||||||||
Sbjct  4256076  GCCTTCGGGAACATTGAGACAGGTGCTGCATGGCTGTCGTCAGCTCGTGT  4256125
```

Range 5: 4574165 to 4575174GenBankGraphics

Next Match
Previous Match
First Match

Alignment statistics for match #5

| Score | Expect | Identities | Gaps | Strand | Frame |
| --- | --- | --- | --- | --- | --- |
| 1866 bits(1010) | 0.0() | 1010/1010(100%) | 0/1010(0%) | Plus/Plus |  |

Features:

rRNA-16S ribosomal RNA . Bacterial SSU

```
Query  9        AGTCGAGCGGTAGAGAGAAGCTTGCTTCTCTTGAGAGCGGCGGACGGGTGAGTAATGCCT  68
                ||||||||||||||||||||||||||||||||||||||||||||||||||||||||||||
Sbjct  4574165  AGTCGAGCGGTAGAGAGAAGCTTGCTTCTCTTGAGAGCGGCGGACGGGTGAGTAATGCCT  4574224

Query  69       AGGAATCTGCCTGGTAGTGGGGGATAACGTTCGGAAACGGACGCTAATACCGCATACGTC  128
                ||||||||||||||||||||||||||||||||||||||||||||||||||||||||||||
Sbjct  4574225  AGGAATCTGCCTGGTAGTGGGGGATAACGTTCGGAAACGGACGCTAATACCGCATACGTC  4574284

Query  129      CTACGGGAGAAAGCAGGGGACCTTCGGGCCTTGCGCTATCAGATGAGCCTAGGTCGGATT  188
                ||||||||||||||||||||||||||||||||||||||||||||||||||||||||||||
Sbjct  4574285  CTACGGGAGAAAGCAGGGGACCTTCGGGCCTTGCGCTATCAGATGAGCCTAGGTCGGATT  4574344

Query  189      AGCTAGTTGGTGGGGTAATGGCTCACCAAGGCGACGATCCGTAACTGGTCTGAGAGGATG  248
                ||||||||||||||||||||||||||||||||||||||||||||||||||||||||||||
Sbjct  4574345  AGCTAGTTGGTGGGGTAATGGCTCACCAAGGCGACGATCCGTAACTGGTCTGAGAGGATG  4574404

Query  249      ATCAGTCACACTGGAACTGAGACACGGTCCAGACTCCTACGGGAGGCAGCAGTGGGGAAT  308
                ||||||||||||||||||||||||||||||||||||||||||||||||||||||||||||
Sbjct  4574405  ATCAGTCACACTGGAACTGAGACACGGTCCAGACTCCTACGGGAGGCAGCAGTGGGGAAT  4574464

Query  309      ATTGGACAATGGGCGAAAGCCTGATCCAGCCATGCCGCGTGTGTGAAGAAGGTCTTCGGA  368
                ||||||||||||||||||||||||||||||||||||||||||||||||||||||||||||
Sbjct  4574465  ATTGGACAATGGGCGAAAGCCTGATCCAGCCATGCCGCGTGTGTGAAGAAGGTCTTCGGA  4574524

Query  369      TTGTAAAGCACTTTAAGTTGGGAGGAAGGGCAGTTACCTAATACGTGATTGTTTTGACGT  428
                ||||||||||||||||||||||||||||||||||||||||||||||||||||||||||||
Sbjct  4574525  TTGTAAAGCACTTTAAGTTGGGAGGAAGGGCAGTTACCTAATACGTGATTGTTTTGACGT  4574584

Query  429      TACCGACAGAATAAGCACCGGCTAACTCTGTGCCAGCAGCCGCGGTAATACAGAGGGTGC  488
                ||||||||||||||||||||||||||||||||||||||||||||||||||||||||||||
Sbjct  4574585  TACCGACAGAATAAGCACCGGCTAACTCTGTGCCAGCAGCCGCGGTAATACAGAGGGTGC  4574644

Query  489      AAGCGTTAATCGGAATTACTGGGCGTAAAGCGCGCGTAGGTGGTTTGTTAAGTTGGATGT  548
                ||||||||||||||||||||||||||||||||||||||||||||||||||||||||||||
Sbjct  4574645  AAGCGTTAATCGGAATTACTGGGCGTAAAGCGCGCGTAGGTGGTTTGTTAAGTTGGATGT  4574704

Query  549      GAAATCCCCGGGCTCAACCTGGGAACTGCATTCAAAACTGACTGACTAGAGTATGGTAGA  608
                ||||||||||||||||||||||||||||||||||||||||||||||||||||||||||||
Sbjct  4574705  GAAATCCCCGGGCTCAACCTGGGAACTGCATTCAAAACTGACTGACTAGAGTATGGTAGA  4574764

Query  609      GGGTGGTGGAATTTCCTGTGTAGCGGTGAAATGCGTAGATATAGGAAGGAACACCAGTGG  668
                ||||||||||||||||||||||||||||||||||||||||||||||||||||||||||||
Sbjct  4574765  GGGTGGTGGAATTTCCTGTGTAGCGGTGAAATGCGTAGATATAGGAAGGAACACCAGTGG  4574824

Query  669      CGAAGGCGACCACCTGGACTAATACTGACACTGAGGTGCGAAAGCGTGGGGAGCAAACAG  728
                ||||||||||||||||||||||||||||||||||||||||||||||||||||||||||||
Sbjct  4574825  CGAAGGCGACCACCTGGACTAATACTGACACTGAGGTGCGAAAGCGTGGGGAGCAAACAG  4574884

Query  729      GATTAGATACCCTGGTAGTCCACGCCGTAAACGATGTCAACTAGCCGTTGGAAGCCTTGA  788
                ||||||||||||||||||||||||||||||||||||||||||||||||||||||||||||
Sbjct  4574885  GATTAGATACCCTGGTAGTCCACGCCGTAAACGATGTCAACTAGCCGTTGGAAGCCTTGA  4574944

Query  789      GCTTTTAGTGGCGCAGCTAACGCATTAAGTTGACCGCCTGGGGAGTACGGCCGCAAGGTT  848
                ||||||||||||||||||||||||||||||||||||||||||||||||||||||||||||
Sbjct  4574945  GCTTTTAGTGGCGCAGCTAACGCATTAAGTTGACCGCCTGGGGAGTACGGCCGCAAGGTT  4575004

Query  849      AAAACTCAAATGAATTGACGGGGGCCCGCACAAGCGGTGGAGCATGTGGTTTAATTCGAA  908
                ||||||||||||||||||||||||||||||||||||||||||||||||||||||||||||
Sbjct  4575005  AAAACTCAAATGAATTGACGGGGGCCCGCACAAGCGGTGGAGCATGTGGTTTAATTCGAA  4575064

Query  909      GCAACGCGAAGAACCTTACCAGGCCTTGACATCCAATGAACTTTCTAGAGATAGATTGGT  968
                ||||||||||||||||||||||||||||||||||||||||||||||||||||||||||||
Sbjct  4575065  GCAACGCGAAGAACCTTACCAGGCCTTGACATCCAATGAACTTTCTAGAGATAGATTGGT  4575124

Query  969      GCCTTCGGGAACATTGAGACAGGTGCTGCATGGCTGTCGTCAGCTCGTGT  1018
                ||||||||||||||||||||||||||||||||||||||||||||||||||
Sbjct  4575125  GCCTTCGGGAACATTGAGACAGGTGCTGCATGGCTGTCGTCAGCTCGTGT  4575174
```

Download

FASTA (complete sequence)

FASTA (aligned sequences)

GenBank (complete sequence)

Continue
Cancel

GenBankGraphics

Sort by:

E value
 Score
Percent identity
Query start position
Subject start position

Next
Previous
Descriptions

Pseudomonas poae RE\*1-1-14, complete genome

Sequence ID: CP004045.1Length: 5512241Number of Matches: 5

Related Information

Range 1: 1279344 to 1280353GenBankGraphics

Next Match
Previous Match
First Match

Alignment statistics for match #1

| Score | Expect | Identities | Gaps | Strand | Frame |
| --- | --- | --- | --- | --- | --- |
| 1866 bits(1010) | 0.0() | 1010/1010(100%) | 0/1010(0%) | Plus/Plus |  |

Features:

```
Query  9        AGTCGAGCGGTAGAGAGAAGCTTGCTTCTCTTGAGAGCGGCGGACGGGTGAGTAATGCCT  68
                ||||||||||||||||||||||||||||||||||||||||||||||||||||||||||||
Sbjct  1279344  AGTCGAGCGGTAGAGAGAAGCTTGCTTCTCTTGAGAGCGGCGGACGGGTGAGTAATGCCT  1279403

Query  69       AGGAATCTGCCTGGTAGTGGGGGATAACGTTCGGAAACGGACGCTAATACCGCATACGTC  128
                ||||||||||||||||||||||||||||||||||||||||||||||||||||||||||||
Sbjct  1279404  AGGAATCTGCCTGGTAGTGGGGGATAACGTTCGGAAACGGACGCTAATACCGCATACGTC  1279463

Query  129      CTACGGGAGAAAGCAGGGGACCTTCGGGCCTTGCGCTATCAGATGAGCCTAGGTCGGATT  188
                ||||||||||||||||||||||||||||||||||||||||||||||||||||||||||||
Sbjct  1279464  CTACGGGAGAAAGCAGGGGACCTTCGGGCCTTGCGCTATCAGATGAGCCTAGGTCGGATT  1279523

Query  189      AGCTAGTTGGTGGGGTAATGGCTCACCAAGGCGACGATCCGTAACTGGTCTGAGAGGATG  248
                ||||||||||||||||||||||||||||||||||||||||||||||||||||||||||||
Sbjct  1279524  AGCTAGTTGGTGGGGTAATGGCTCACCAAGGCGACGATCCGTAACTGGTCTGAGAGGATG  1279583

Query  249      ATCAGTCACACTGGAACTGAGACACGGTCCAGACTCCTACGGGAGGCAGCAGTGGGGAAT  308
                ||||||||||||||||||||||||||||||||||||||||||||||||||||||||||||
Sbjct  1279584  ATCAGTCACACTGGAACTGAGACACGGTCCAGACTCCTACGGGAGGCAGCAGTGGGGAAT  1279643

Query  309      ATTGGACAATGGGCGAAAGCCTGATCCAGCCATGCCGCGTGTGTGAAGAAGGTCTTCGGA  368
                ||||||||||||||||||||||||||||||||||||||||||||||||||||||||||||
Sbjct  1279644  ATTGGACAATGGGCGAAAGCCTGATCCAGCCATGCCGCGTGTGTGAAGAAGGTCTTCGGA  1279703

Query  369      TTGTAAAGCACTTTAAGTTGGGAGGAAGGGCAGTTACCTAATACGTGATTGTTTTGACGT  428
                ||||||||||||||||||||||||||||||||||||||||||||||||||||||||||||
Sbjct  1279704  TTGTAAAGCACTTTAAGTTGGGAGGAAGGGCAGTTACCTAATACGTGATTGTTTTGACGT  1279763

Query  429      TACCGACAGAATAAGCACCGGCTAACTCTGTGCCAGCAGCCGCGGTAATACAGAGGGTGC  488
                ||||||||||||||||||||||||||||||||||||||||||||||||||||||||||||
Sbjct  1279764  TACCGACAGAATAAGCACCGGCTAACTCTGTGCCAGCAGCCGCGGTAATACAGAGGGTGC  1279823

Query  489      AAGCGTTAATCGGAATTACTGGGCGTAAAGCGCGCGTAGGTGGTTTGTTAAGTTGGATGT  548
                ||||||||||||||||||||||||||||||||||||||||||||||||||||||||||||
Sbjct  1279824  AAGCGTTAATCGGAATTACTGGGCGTAAAGCGCGCGTAGGTGGTTTGTTAAGTTGGATGT  1279883

Query  549      GAAATCCCCGGGCTCAACCTGGGAACTGCATTCAAAACTGACTGACTAGAGTATGGTAGA  608
                ||||||||||||||||||||||||||||||||||||||||||||||||||||||||||||
Sbjct  1279884  GAAATCCCCGGGCTCAACCTGGGAACTGCATTCAAAACTGACTGACTAGAGTATGGTAGA  1279943

Query  609      GGGTGGTGGAATTTCCTGTGTAGCGGTGAAATGCGTAGATATAGGAAGGAACACCAGTGG  668
                ||||||||||||||||||||||||||||||||||||||||||||||||||||||||||||
Sbjct  1279944  GGGTGGTGGAATTTCCTGTGTAGCGGTGAAATGCGTAGATATAGGAAGGAACACCAGTGG  1280003

Query  669      CGAAGGCGACCACCTGGACTAATACTGACACTGAGGTGCGAAAGCGTGGGGAGCAAACAG  728
                ||||||||||||||||||||||||||||||||||||||||||||||||||||||||||||
Sbjct  1280004  CGAAGGCGACCACCTGGACTAATACTGACACTGAGGTGCGAAAGCGTGGGGAGCAAACAG  1280063

Query  729      GATTAGATACCCTGGTAGTCCACGCCGTAAACGATGTCAACTAGCCGTTGGAAGCCTTGA  788
                ||||||||||||||||||||||||||||||||||||||||||||||||||||||||||||
Sbjct  1280064  GATTAGATACCCTGGTAGTCCACGCCGTAAACGATGTCAACTAGCCGTTGGAAGCCTTGA  1280123

Query  789      GCTTTTAGTGGCGCAGCTAACGCATTAAGTTGACCGCCTGGGGAGTACGGCCGCAAGGTT  848
                ||||||||||||||||||||||||||||||||||||||||||||||||||||||||||||
Sbjct  1280124  GCTTTTAGTGGCGCAGCTAACGCATTAAGTTGACCGCCTGGGGAGTACGGCCGCAAGGTT  1280183

Query  849      AAAACTCAAATGAATTGACGGGGGCCCGCACAAGCGGTGGAGCATGTGGTTTAATTCGAA  908
                ||||||||||||||||||||||||||||||||||||||||||||||||||||||||||||
Sbjct  1280184  AAAACTCAAATGAATTGACGGGGGCCCGCACAAGCGGTGGAGCATGTGGTTTAATTCGAA  1280243

Query  909      GCAACGCGAAGAACCTTACCAGGCCTTGACATCCAATGAACTTTCTAGAGATAGATTGGT  968
                ||||||||||||||||||||||||||||||||||||||||||||||||||||||||||||
Sbjct  1280244  GCAACGCGAAGAACCTTACCAGGCCTTGACATCCAATGAACTTTCTAGAGATAGATTGGT  1280303

Query  969      GCCTTCGGGAACATTGAGACAGGTGCTGCATGGCTGTCGTCAGCTCGTGT  1018
                ||||||||||||||||||||||||||||||||||||||||||||||||||
Sbjct  1280304  GCCTTCGGGAACATTGAGACAGGTGCTGCATGGCTGTCGTCAGCTCGTGT  1280353
```

Range 2: 3808774 to 3809783GenBankGraphics

Next Match
Previous Match
First Match

Alignment statistics for match #2

| Score | Expect | Identities | Gaps | Strand | Frame |
| --- | --- | --- | --- | --- | --- |
| 1866 bits(1010) | 0.0() | 1010/1010(100%) | 0/1010(0%) | Plus/Minus |  |

Features:

```
Query  9        AGTCGAGCGGTAGAGAGAAGCTTGCTTCTCTTGAGAGCGGCGGACGGGTGAGTAATGCCT  68
                ||||||||||||||||||||||||||||||||||||||||||||||||||||||||||||
Sbjct  3809783  AGTCGAGCGGTAGAGAGAAGCTTGCTTCTCTTGAGAGCGGCGGACGGGTGAGTAATGCCT  3809724

Query  69       AGGAATCTGCCTGGTAGTGGGGGATAACGTTCGGAAACGGACGCTAATACCGCATACGTC  128
                ||||||||||||||||||||||||||||||||||||||||||||||||||||||||||||
Sbjct  3809723  AGGAATCTGCCTGGTAGTGGGGGATAACGTTCGGAAACGGACGCTAATACCGCATACGTC  3809664

Query  129      CTACGGGAGAAAGCAGGGGACCTTCGGGCCTTGCGCTATCAGATGAGCCTAGGTCGGATT  188
                ||||||||||||||||||||||||||||||||||||||||||||||||||||||||||||
Sbjct  3809663  CTACGGGAGAAAGCAGGGGACCTTCGGGCCTTGCGCTATCAGATGAGCCTAGGTCGGATT  3809604

Query  189      AGCTAGTTGGTGGGGTAATGGCTCACCAAGGCGACGATCCGTAACTGGTCTGAGAGGATG  248
                ||||||||||||||||||||||||||||||||||||||||||||||||||||||||||||
Sbjct  3809603  AGCTAGTTGGTGGGGTAATGGCTCACCAAGGCGACGATCCGTAACTGGTCTGAGAGGATG  3809544

Query  249      ATCAGTCACACTGGAACTGAGACACGGTCCAGACTCCTACGGGAGGCAGCAGTGGGGAAT  308
                ||||||||||||||||||||||||||||||||||||||||||||||||||||||||||||
Sbjct  3809543  ATCAGTCACACTGGAACTGAGACACGGTCCAGACTCCTACGGGAGGCAGCAGTGGGGAAT  3809484

Query  309      ATTGGACAATGGGCGAAAGCCTGATCCAGCCATGCCGCGTGTGTGAAGAAGGTCTTCGGA  368
                ||||||||||||||||||||||||||||||||||||||||||||||||||||||||||||
Sbjct  3809483  ATTGGACAATGGGCGAAAGCCTGATCCAGCCATGCCGCGTGTGTGAAGAAGGTCTTCGGA  3809424

Query  369      TTGTAAAGCACTTTAAGTTGGGAGGAAGGGCAGTTACCTAATACGTGATTGTTTTGACGT  428
                ||||||||||||||||||||||||||||||||||||||||||||||||||||||||||||
Sbjct  3809423  TTGTAAAGCACTTTAAGTTGGGAGGAAGGGCAGTTACCTAATACGTGATTGTTTTGACGT  3809364

Query  429      TACCGACAGAATAAGCACCGGCTAACTCTGTGCCAGCAGCCGCGGTAATACAGAGGGTGC  488
                ||||||||||||||||||||||||||||||||||||||||||||||||||||||||||||
Sbjct  3809363  TACCGACAGAATAAGCACCGGCTAACTCTGTGCCAGCAGCCGCGGTAATACAGAGGGTGC  3809304

Query  489      AAGCGTTAATCGGAATTACTGGGCGTAAAGCGCGCGTAGGTGGTTTGTTAAGTTGGATGT  548
                ||||||||||||||||||||||||||||||||||||||||||||||||||||||||||||
Sbjct  3809303  AAGCGTTAATCGGAATTACTGGGCGTAAAGCGCGCGTAGGTGGTTTGTTAAGTTGGATGT  3809244

Query  549      GAAATCCCCGGGCTCAACCTGGGAACTGCATTCAAAACTGACTGACTAGAGTATGGTAGA  608
                ||||||||||||||||||||||||||||||||||||||||||||||||||||||||||||
Sbjct  3809243  GAAATCCCCGGGCTCAACCTGGGAACTGCATTCAAAACTGACTGACTAGAGTATGGTAGA  3809184

Query  609      GGGTGGTGGAATTTCCTGTGTAGCGGTGAAATGCGTAGATATAGGAAGGAACACCAGTGG  668
                ||||||||||||||||||||||||||||||||||||||||||||||||||||||||||||
Sbjct  3809183  GGGTGGTGGAATTTCCTGTGTAGCGGTGAAATGCGTAGATATAGGAAGGAACACCAGTGG  3809124

Query  669      CGAAGGCGACCACCTGGACTAATACTGACACTGAGGTGCGAAAGCGTGGGGAGCAAACAG  728
                ||||||||||||||||||||||||||||||||||||||||||||||||||||||||||||
Sbjct  3809123  CGAAGGCGACCACCTGGACTAATACTGACACTGAGGTGCGAAAGCGTGGGGAGCAAACAG  3809064

Query  729      GATTAGATACCCTGGTAGTCCACGCCGTAAACGATGTCAACTAGCCGTTGGAAGCCTTGA  788
                ||||||||||||||||||||||||||||||||||||||||||||||||||||||||||||
Sbjct  3809063  GATTAGATACCCTGGTAGTCCACGCCGTAAACGATGTCAACTAGCCGTTGGAAGCCTTGA  3809004

Query  789      GCTTTTAGTGGCGCAGCTAACGCATTAAGTTGACCGCCTGGGGAGTACGGCCGCAAGGTT  848
                ||||||||||||||||||||||||||||||||||||||||||||||||||||||||||||
Sbjct  3809003  GCTTTTAGTGGCGCAGCTAACGCATTAAGTTGACCGCCTGGGGAGTACGGCCGCAAGGTT  3808944

Query  849      AAAACTCAAATGAATTGACGGGGGCCCGCACAAGCGGTGGAGCATGTGGTTTAATTCGAA  908
                ||||||||||||||||||||||||||||||||||||||||||||||||||||||||||||
Sbjct  3808943  AAAACTCAAATGAATTGACGGGGGCCCGCACAAGCGGTGGAGCATGTGGTTTAATTCGAA  3808884

Query  909      GCAACGCGAAGAACCTTACCAGGCCTTGACATCCAATGAACTTTCTAGAGATAGATTGGT  968
                ||||||||||||||||||||||||||||||||||||||||||||||||||||||||||||
Sbjct  3808883  GCAACGCGAAGAACCTTACCAGGCCTTGACATCCAATGAACTTTCTAGAGATAGATTGGT  3808824

Query  969      GCCTTCGGGAACATTGAGACAGGTGCTGCATGGCTGTCGTCAGCTCGTGT  1018
                ||||||||||||||||||||||||||||||||||||||||||||||||||
Sbjct  3808823  GCCTTCGGGAACATTGAGACAGGTGCTGCATGGCTGTCGTCAGCTCGTGT  3808774
```

Range 3: 4137182 to 4138191GenBankGraphics

Next Match
Previous Match
First Match

Alignment statistics for match #3

| Score | Expect | Identities | Gaps | Strand | Frame |
| --- | --- | --- | --- | --- | --- |
| 1866 bits(1010) | 0.0() | 1010/1010(100%) | 0/1010(0%) | Plus/Minus |  |

Features:

```
Query  9        AGTCGAGCGGTAGAGAGAAGCTTGCTTCTCTTGAGAGCGGCGGACGGGTGAGTAATGCCT  68
                ||||||||||||||||||||||||||||||||||||||||||||||||||||||||||||
Sbjct  4138191  AGTCGAGCGGTAGAGAGAAGCTTGCTTCTCTTGAGAGCGGCGGACGGGTGAGTAATGCCT  4138132

Query  69       AGGAATCTGCCTGGTAGTGGGGGATAACGTTCGGAAACGGACGCTAATACCGCATACGTC  128
                ||||||||||||||||||||||||||||||||||||||||||||||||||||||||||||
Sbjct  4138131  AGGAATCTGCCTGGTAGTGGGGGATAACGTTCGGAAACGGACGCTAATACCGCATACGTC  4138072

Query  129      CTACGGGAGAAAGCAGGGGACCTTCGGGCCTTGCGCTATCAGATGAGCCTAGGTCGGATT  188
                ||||||||||||||||||||||||||||||||||||||||||||||||||||||||||||
Sbjct  4138071  CTACGGGAGAAAGCAGGGGACCTTCGGGCCTTGCGCTATCAGATGAGCCTAGGTCGGATT  4138012

Query  189      AGCTAGTTGGTGGGGTAATGGCTCACCAAGGCGACGATCCGTAACTGGTCTGAGAGGATG  248
                ||||||||||||||||||||||||||||||||||||||||||||||||||||||||||||
Sbjct  4138011  AGCTAGTTGGTGGGGTAATGGCTCACCAAGGCGACGATCCGTAACTGGTCTGAGAGGATG  4137952

Query  249      ATCAGTCACACTGGAACTGAGACACGGTCCAGACTCCTACGGGAGGCAGCAGTGGGGAAT  308
                ||||||||||||||||||||||||||||||||||||||||||||||||||||||||||||
Sbjct  4137951  ATCAGTCACACTGGAACTGAGACACGGTCCAGACTCCTACGGGAGGCAGCAGTGGGGAAT  4137892

Query  309      ATTGGACAATGGGCGAAAGCCTGATCCAGCCATGCCGCGTGTGTGAAGAAGGTCTTCGGA  368
                ||||||||||||||||||||||||||||||||||||||||||||||||||||||||||||
Sbjct  4137891  ATTGGACAATGGGCGAAAGCCTGATCCAGCCATGCCGCGTGTGTGAAGAAGGTCTTCGGA  4137832

Query  369      TTGTAAAGCACTTTAAGTTGGGAGGAAGGGCAGTTACCTAATACGTGATTGTTTTGACGT  428
                ||||||||||||||||||||||||||||||||||||||||||||||||||||||||||||
Sbjct  4137831  TTGTAAAGCACTTTAAGTTGGGAGGAAGGGCAGTTACCTAATACGTGATTGTTTTGACGT  4137772

Query  429      TACCGACAGAATAAGCACCGGCTAACTCTGTGCCAGCAGCCGCGGTAATACAGAGGGTGC  488
                ||||||||||||||||||||||||||||||||||||||||||||||||||||||||||||
Sbjct  4137771  TACCGACAGAATAAGCACCGGCTAACTCTGTGCCAGCAGCCGCGGTAATACAGAGGGTGC  4137712

Query  489      AAGCGTTAATCGGAATTACTGGGCGTAAAGCGCGCGTAGGTGGTTTGTTAAGTTGGATGT  548
                ||||||||||||||||||||||||||||||||||||||||||||||||||||||||||||
Sbjct  4137711  AAGCGTTAATCGGAATTACTGGGCGTAAAGCGCGCGTAGGTGGTTTGTTAAGTTGGATGT  4137652

Query  549      GAAATCCCCGGGCTCAACCTGGGAACTGCATTCAAAACTGACTGACTAGAGTATGGTAGA  608
                ||||||||||||||||||||||||||||||||||||||||||||||||||||||||||||
Sbjct  4137651  GAAATCCCCGGGCTCAACCTGGGAACTGCATTCAAAACTGACTGACTAGAGTATGGTAGA  4137592

Query  609      GGGTGGTGGAATTTCCTGTGTAGCGGTGAAATGCGTAGATATAGGAAGGAACACCAGTGG  668
                ||||||||||||||||||||||||||||||||||||||||||||||||||||||||||||
Sbjct  4137591  GGGTGGTGGAATTTCCTGTGTAGCGGTGAAATGCGTAGATATAGGAAGGAACACCAGTGG  4137532

Query  669      CGAAGGCGACCACCTGGACTAATACTGACACTGAGGTGCGAAAGCGTGGGGAGCAAACAG  728
                ||||||||||||||||||||||||||||||||||||||||||||||||||||||||||||
Sbjct  4137531  CGAAGGCGACCACCTGGACTAATACTGACACTGAGGTGCGAAAGCGTGGGGAGCAAACAG  4137472

Query  729      GATTAGATACCCTGGTAGTCCACGCCGTAAACGATGTCAACTAGCCGTTGGAAGCCTTGA  788
                ||||||||||||||||||||||||||||||||||||||||||||||||||||||||||||
Sbjct  4137471  GATTAGATACCCTGGTAGTCCACGCCGTAAACGATGTCAACTAGCCGTTGGAAGCCTTGA  4137412

Query  789      GCTTTTAGTGGCGCAGCTAACGCATTAAGTTGACCGCCTGGGGAGTACGGCCGCAAGGTT  848
                ||||||||||||||||||||||||||||||||||||||||||||||||||||||||||||
Sbjct  4137411  GCTTTTAGTGGCGCAGCTAACGCATTAAGTTGACCGCCTGGGGAGTACGGCCGCAAGGTT  4137352

Query  849      AAAACTCAAATGAATTGACGGGGGCCCGCACAAGCGGTGGAGCATGTGGTTTAATTCGAA  908
                ||||||||||||||||||||||||||||||||||||||||||||||||||||||||||||
Sbjct  4137351  AAAACTCAAATGAATTGACGGGGGCCCGCACAAGCGGTGGAGCATGTGGTTTAATTCGAA  4137292

Query  909      GCAACGCGAAGAACCTTACCAGGCCTTGACATCCAATGAACTTTCTAGAGATAGATTGGT  968
                ||||||||||||||||||||||||||||||||||||||||||||||||||||||||||||
Sbjct  4137291  GCAACGCGAAGAACCTTACCAGGCCTTGACATCCAATGAACTTTCTAGAGATAGATTGGT  4137232

Query  969      GCCTTCGGGAACATTGAGACAGGTGCTGCATGGCTGTCGTCAGCTCGTGT  1018
                ||||||||||||||||||||||||||||||||||||||||||||||||||
Sbjct  4137231  GCCTTCGGGAACATTGAGACAGGTGCTGCATGGCTGTCGTCAGCTCGTGT  4137182
```

Range 4: 5506947 to 5507956GenBankGraphics

Next Match
Previous Match
First Match

Alignment statistics for match #4

| Score | Expect | Identities | Gaps | Strand | Frame |
| --- | --- | --- | --- | --- | --- |
| 1866 bits(1010) | 0.0() | 1010/1010(100%) | 0/1010(0%) | Plus/Plus |  |

Features:

```
Query  9        AGTCGAGCGGTAGAGAGAAGCTTGCTTCTCTTGAGAGCGGCGGACGGGTGAGTAATGCCT  68
                ||||||||||||||||||||||||||||||||||||||||||||||||||||||||||||
Sbjct  5506947  AGTCGAGCGGTAGAGAGAAGCTTGCTTCTCTTGAGAGCGGCGGACGGGTGAGTAATGCCT  5507006

Query  69       AGGAATCTGCCTGGTAGTGGGGGATAACGTTCGGAAACGGACGCTAATACCGCATACGTC  128
                ||||||||||||||||||||||||||||||||||||||||||||||||||||||||||||
Sbjct  5507007  AGGAATCTGCCTGGTAGTGGGGGATAACGTTCGGAAACGGACGCTAATACCGCATACGTC  5507066

Query  129      CTACGGGAGAAAGCAGGGGACCTTCGGGCCTTGCGCTATCAGATGAGCCTAGGTCGGATT  188
                ||||||||||||||||||||||||||||||||||||||||||||||||||||||||||||
Sbjct  5507067  CTACGGGAGAAAGCAGGGGACCTTCGGGCCTTGCGCTATCAGATGAGCCTAGGTCGGATT  5507126

Query  189      AGCTAGTTGGTGGGGTAATGGCTCACCAAGGCGACGATCCGTAACTGGTCTGAGAGGATG  248
                ||||||||||||||||||||||||||||||||||||||||||||||||||||||||||||
Sbjct  5507127  AGCTAGTTGGTGGGGTAATGGCTCACCAAGGCGACGATCCGTAACTGGTCTGAGAGGATG  5507186

Query  249      ATCAGTCACACTGGAACTGAGACACGGTCCAGACTCCTACGGGAGGCAGCAGTGGGGAAT  308
                ||||||||||||||||||||||||||||||||||||||||||||||||||||||||||||
Sbjct  5507187  ATCAGTCACACTGGAACTGAGACACGGTCCAGACTCCTACGGGAGGCAGCAGTGGGGAAT  5507246

Query  309      ATTGGACAATGGGCGAAAGCCTGATCCAGCCATGCCGCGTGTGTGAAGAAGGTCTTCGGA  368
                ||||||||||||||||||||||||||||||||||||||||||||||||||||||||||||
Sbjct  5507247  ATTGGACAATGGGCGAAAGCCTGATCCAGCCATGCCGCGTGTGTGAAGAAGGTCTTCGGA  5507306

Query  369      TTGTAAAGCACTTTAAGTTGGGAGGAAGGGCAGTTACCTAATACGTGATTGTTTTGACGT  428
                ||||||||||||||||||||||||||||||||||||||||||||||||||||||||||||
Sbjct  5507307  TTGTAAAGCACTTTAAGTTGGGAGGAAGGGCAGTTACCTAATACGTGATTGTTTTGACGT  5507366

Query  429      TACCGACAGAATAAGCACCGGCTAACTCTGTGCCAGCAGCCGCGGTAATACAGAGGGTGC  488
                ||||||||||||||||||||||||||||||||||||||||||||||||||||||||||||
Sbjct  5507367  TACCGACAGAATAAGCACCGGCTAACTCTGTGCCAGCAGCCGCGGTAATACAGAGGGTGC  5507426

Query  489      AAGCGTTAATCGGAATTACTGGGCGTAAAGCGCGCGTAGGTGGTTTGTTAAGTTGGATGT  548
                ||||||||||||||||||||||||||||||||||||||||||||||||||||||||||||
Sbjct  5507427  AAGCGTTAATCGGAATTACTGGGCGTAAAGCGCGCGTAGGTGGTTTGTTAAGTTGGATGT  5507486

Query  549      GAAATCCCCGGGCTCAACCTGGGAACTGCATTCAAAACTGACTGACTAGAGTATGGTAGA  608
                ||||||||||||||||||||||||||||||||||||||||||||||||||||||||||||
Sbjct  5507487  GAAATCCCCGGGCTCAACCTGGGAACTGCATTCAAAACTGACTGACTAGAGTATGGTAGA  5507546

Query  609      GGGTGGTGGAATTTCCTGTGTAGCGGTGAAATGCGTAGATATAGGAAGGAACACCAGTGG  668
                ||||||||||||||||||||||||||||||||||||||||||||||||||||||||||||
Sbjct  5507547  GGGTGGTGGAATTTCCTGTGTAGCGGTGAAATGCGTAGATATAGGAAGGAACACCAGTGG  5507606

Query  669      CGAAGGCGACCACCTGGACTAATACTGACACTGAGGTGCGAAAGCGTGGGGAGCAAACAG  728
                ||||||||||||||||||||||||||||||||||||||||||||||||||||||||||||
Sbjct  5507607  CGAAGGCGACCACCTGGACTAATACTGACACTGAGGTGCGAAAGCGTGGGGAGCAAACAG  5507666

Query  729      GATTAGATACCCTGGTAGTCCACGCCGTAAACGATGTCAACTAGCCGTTGGAAGCCTTGA  788
                ||||||||||||||||||||||||||||||||||||||||||||||||||||||||||||
Sbjct  5507667  GATTAGATACCCTGGTAGTCCACGCCGTAAACGATGTCAACTAGCCGTTGGAAGCCTTGA  5507726

Query  789      GCTTTTAGTGGCGCAGCTAACGCATTAAGTTGACCGCCTGGGGAGTACGGCCGCAAGGTT  848
                ||||||||||||||||||||||||||||||||||||||||||||||||||||||||||||
Sbjct  5507727  GCTTTTAGTGGCGCAGCTAACGCATTAAGTTGACCGCCTGGGGAGTACGGCCGCAAGGTT  5507786

Query  849      AAAACTCAAATGAATTGACGGGGGCCCGCACAAGCGGTGGAGCATGTGGTTTAATTCGAA  908
                ||||||||||||||||||||||||||||||||||||||||||||||||||||||||||||
Sbjct  5507787  AAAACTCAAATGAATTGACGGGGGCCCGCACAAGCGGTGGAGCATGTGGTTTAATTCGAA  5507846

Query  909      GCAACGCGAAGAACCTTACCAGGCCTTGACATCCAATGAACTTTCTAGAGATAGATTGGT  968
                ||||||||||||||||||||||||||||||||||||||||||||||||||||||||||||
Sbjct  5507847  GCAACGCGAAGAACCTTACCAGGCCTTGACATCCAATGAACTTTCTAGAGATAGATTGGT  5507906

Query  969      GCCTTCGGGAACATTGAGACAGGTGCTGCATGGCTGTCGTCAGCTCGTGT  1018
                ||||||||||||||||||||||||||||||||||||||||||||||||||
Sbjct  5507907  GCCTTCGGGAACATTGAGACAGGTGCTGCATGGCTGTCGTCAGCTCGTGT  5507956
```

Range 5: 4900349 to 4901358GenBankGraphics

Next Match
Previous Match
First Match

Alignment statistics for match #5

| Score | Expect | Identities | Gaps | Strand | Frame |
| --- | --- | --- | --- | --- | --- |
| 1860 bits(1007) | 0.0() | 1009/1010(99%) | 0/1010(0%) | Plus/Plus |  |

Features:

```
Query  9        AGTCGAGCGGTAGAGAGAAGCTTGCTTCTCTTGAGAGCGGCGGACGGGTGAGTAATGCCT  68
                |||||||||||||||||||||| |||||||||||||||||||||||||||||||||||||
Sbjct  4900349  AGTCGAGCGGTAGAGAGAAGCTCGCTTCTCTTGAGAGCGGCGGACGGGTGAGTAATGCCT  4900408

Query  69       AGGAATCTGCCTGGTAGTGGGGGATAACGTTCGGAAACGGACGCTAATACCGCATACGTC  128
                ||||||||||||||||||||||||||||||||||||||||||||||||||||||||||||
Sbjct  4900409  AGGAATCTGCCTGGTAGTGGGGGATAACGTTCGGAAACGGACGCTAATACCGCATACGTC  4900468

Query  129      CTACGGGAGAAAGCAGGGGACCTTCGGGCCTTGCGCTATCAGATGAGCCTAGGTCGGATT  188
                ||||||||||||||||||||||||||||||||||||||||||||||||||||||||||||
Sbjct  4900469  CTACGGGAGAAAGCAGGGGACCTTCGGGCCTTGCGCTATCAGATGAGCCTAGGTCGGATT  4900528

Query  189      AGCTAGTTGGTGGGGTAATGGCTCACCAAGGCGACGATCCGTAACTGGTCTGAGAGGATG  248
                ||||||||||||||||||||||||||||||||||||||||||||||||||||||||||||
Sbjct  4900529  AGCTAGTTGGTGGGGTAATGGCTCACCAAGGCGACGATCCGTAACTGGTCTGAGAGGATG  4900588

Query  249      ATCAGTCACACTGGAACTGAGACACGGTCCAGACTCCTACGGGAGGCAGCAGTGGGGAAT  308
                ||||||||||||||||||||||||||||||||||||||||||||||||||||||||||||
Sbjct  4900589  ATCAGTCACACTGGAACTGAGACACGGTCCAGACTCCTACGGGAGGCAGCAGTGGGGAAT  4900648

Query  309      ATTGGACAATGGGCGAAAGCCTGATCCAGCCATGCCGCGTGTGTGAAGAAGGTCTTCGGA  368
                ||||||||||||||||||||||||||||||||||||||||||||||||||||||||||||
Sbjct  4900649  ATTGGACAATGGGCGAAAGCCTGATCCAGCCATGCCGCGTGTGTGAAGAAGGTCTTCGGA  4900708

Query  369      TTGTAAAGCACTTTAAGTTGGGAGGAAGGGCAGTTACCTAATACGTGATTGTTTTGACGT  428
                ||||||||||||||||||||||||||||||||||||||||||||||||||||||||||||
Sbjct  4900709  TTGTAAAGCACTTTAAGTTGGGAGGAAGGGCAGTTACCTAATACGTGATTGTTTTGACGT  4900768

Query  429      TACCGACAGAATAAGCACCGGCTAACTCTGTGCCAGCAGCCGCGGTAATACAGAGGGTGC  488
                ||||||||||||||||||||||||||||||||||||||||||||||||||||||||||||
Sbjct  4900769  TACCGACAGAATAAGCACCGGCTAACTCTGTGCCAGCAGCCGCGGTAATACAGAGGGTGC  4900828

Query  489      AAGCGTTAATCGGAATTACTGGGCGTAAAGCGCGCGTAGGTGGTTTGTTAAGTTGGATGT  548
                ||||||||||||||||||||||||||||||||||||||||||||||||||||||||||||
Sbjct  4900829  AAGCGTTAATCGGAATTACTGGGCGTAAAGCGCGCGTAGGTGGTTTGTTAAGTTGGATGT  4900888

Query  549      GAAATCCCCGGGCTCAACCTGGGAACTGCATTCAAAACTGACTGACTAGAGTATGGTAGA  608
                ||||||||||||||||||||||||||||||||||||||||||||||||||||||||||||
Sbjct  4900889  GAAATCCCCGGGCTCAACCTGGGAACTGCATTCAAAACTGACTGACTAGAGTATGGTAGA  4900948

Query  609      GGGTGGTGGAATTTCCTGTGTAGCGGTGAAATGCGTAGATATAGGAAGGAACACCAGTGG  668
                ||||||||||||||||||||||||||||||||||||||||||||||||||||||||||||
Sbjct  4900949  GGGTGGTGGAATTTCCTGTGTAGCGGTGAAATGCGTAGATATAGGAAGGAACACCAGTGG  4901008

Query  669      CGAAGGCGACCACCTGGACTAATACTGACACTGAGGTGCGAAAGCGTGGGGAGCAAACAG  728
                ||||||||||||||||||||||||||||||||||||||||||||||||||||||||||||
Sbjct  4901009  CGAAGGCGACCACCTGGACTAATACTGACACTGAGGTGCGAAAGCGTGGGGAGCAAACAG  4901068

Query  729      GATTAGATACCCTGGTAGTCCACGCCGTAAACGATGTCAACTAGCCGTTGGAAGCCTTGA  788
                ||||||||||||||||||||||||||||||||||||||||||||||||||||||||||||
Sbjct  4901069  GATTAGATACCCTGGTAGTCCACGCCGTAAACGATGTCAACTAGCCGTTGGAAGCCTTGA  4901128

Query  789      GCTTTTAGTGGCGCAGCTAACGCATTAAGTTGACCGCCTGGGGAGTACGGCCGCAAGGTT  848
                ||||||||||||||||||||||||||||||||||||||||||||||||||||||||||||
Sbjct  4901129  GCTTTTAGTGGCGCAGCTAACGCATTAAGTTGACCGCCTGGGGAGTACGGCCGCAAGGTT  4901188

Query  849      AAAACTCAAATGAATTGACGGGGGCCCGCACAAGCGGTGGAGCATGTGGTTTAATTCGAA  908
                ||||||||||||||||||||||||||||||||||||||||||||||||||||||||||||
Sbjct  4901189  AAAACTCAAATGAATTGACGGGGGCCCGCACAAGCGGTGGAGCATGTGGTTTAATTCGAA  4901248

Query  909      GCAACGCGAAGAACCTTACCAGGCCTTGACATCCAATGAACTTTCTAGAGATAGATTGGT  968
                ||||||||||||||||||||||||||||||||||||||||||||||||||||||||||||
Sbjct  4901249  GCAACGCGAAGAACCTTACCAGGCCTTGACATCCAATGAACTTTCTAGAGATAGATTGGT  4901308

Query  969      GCCTTCGGGAACATTGAGACAGGTGCTGCATGGCTGTCGTCAGCTCGTGT  1018
                ||||||||||||||||||||||||||||||||||||||||||||||||||
Sbjct  4901309  GCCTTCGGGAACATTGAGACAGGTGCTGCATGGCTGTCGTCAGCTCGTGT  4901358
```

Download

FASTA (complete sequence)

FASTA (aligned sequences)

GenBank (complete sequence)

Continue
Cancel

GenBankGraphics

Next
Previous
Descriptions

uncultured bacterium partial 16S rRNA gene, clone K15.87 KSS

Sequence ID: HE576021.1Length: 1459Number of Matches: 1

Related Information

Range 1: 33 to 1042GenBankGraphics

Next Match
Previous Match
First Match

Alignment statistics for match #1

| Score | Expect | Identities | Gaps | Strand | Frame |
| --- | --- | --- | --- | --- | --- |
| 1866 bits(1010) | 0.0() | 1010/1010(100%) | 0/1010(0%) | Plus/Plus |  |

Features:

```
Query  9     AGTCGAGCGGTAGAGAGAAGCTTGCTTCTCTTGAGAGCGGCGGACGGGTGAGTAATGCCT  68
             ||||||||||||||||||||||||||||||||||||||||||||||||||||||||||||
Sbjct  33    AGTCGAGCGGTAGAGAGAAGCTTGCTTCTCTTGAGAGCGGCGGACGGGTGAGTAATGCCT  92

Query  69    AGGAATCTGCCTGGTAGTGGGGGATAACGTTCGGAAACGGACGCTAATACCGCATACGTC  128
             ||||||||||||||||||||||||||||||||||||||||||||||||||||||||||||
Sbjct  93    AGGAATCTGCCTGGTAGTGGGGGATAACGTTCGGAAACGGACGCTAATACCGCATACGTC  152

Query  129   CTACGGGAGAAAGCAGGGGACCTTCGGGCCTTGCGCTATCAGATGAGCCTAGGTCGGATT  188
             ||||||||||||||||||||||||||||||||||||||||||||||||||||||||||||
Sbjct  153   CTACGGGAGAAAGCAGGGGACCTTCGGGCCTTGCGCTATCAGATGAGCCTAGGTCGGATT  212

Query  189   AGCTAGTTGGTGGGGTAATGGCTCACCAAGGCGACGATCCGTAACTGGTCTGAGAGGATG  248
             ||||||||||||||||||||||||||||||||||||||||||||||||||||||||||||
Sbjct  213   AGCTAGTTGGTGGGGTAATGGCTCACCAAGGCGACGATCCGTAACTGGTCTGAGAGGATG  272

Query  249   ATCAGTCACACTGGAACTGAGACACGGTCCAGACTCCTACGGGAGGCAGCAGTGGGGAAT  308
             ||||||||||||||||||||||||||||||||||||||||||||||||||||||||||||
Sbjct  273   ATCAGTCACACTGGAACTGAGACACGGTCCAGACTCCTACGGGAGGCAGCAGTGGGGAAT  332

Query  309   ATTGGACAATGGGCGAAAGCCTGATCCAGCCATGCCGCGTGTGTGAAGAAGGTCTTCGGA  368
             ||||||||||||||||||||||||||||||||||||||||||||||||||||||||||||
Sbjct  333   ATTGGACAATGGGCGAAAGCCTGATCCAGCCATGCCGCGTGTGTGAAGAAGGTCTTCGGA  392

Query  369   TTGTAAAGCACTTTAAGTTGGGAGGAAGGGCAGTTACCTAATACGTGATTGTTTTGACGT  428
             ||||||||||||||||||||||||||||||||||||||||||||||||||||||||||||
Sbjct  393   TTGTAAAGCACTTTAAGTTGGGAGGAAGGGCAGTTACCTAATACGTGATTGTTTTGACGT  452

Query  429   TACCGACAGAATAAGCACCGGCTAACTCTGTGCCAGCAGCCGCGGTAATACAGAGGGTGC  488
             ||||||||||||||||||||||||||||||||||||||||||||||||||||||||||||
Sbjct  453   TACCGACAGAATAAGCACCGGCTAACTCTGTGCCAGCAGCCGCGGTAATACAGAGGGTGC  512

Query  489   AAGCGTTAATCGGAATTACTGGGCGTAAAGCGCGCGTAGGTGGTTTGTTAAGTTGGATGT  548
             ||||||||||||||||||||||||||||||||||||||||||||||||||||||||||||
Sbjct  513   AAGCGTTAATCGGAATTACTGGGCGTAAAGCGCGCGTAGGTGGTTTGTTAAGTTGGATGT  572

Query  549   GAAATCCCCGGGCTCAACCTGGGAACTGCATTCAAAACTGACTGACTAGAGTATGGTAGA  608
             ||||||||||||||||||||||||||||||||||||||||||||||||||||||||||||
Sbjct  573   GAAATCCCCGGGCTCAACCTGGGAACTGCATTCAAAACTGACTGACTAGAGTATGGTAGA  632

Query  609   GGGTGGTGGAATTTCCTGTGTAGCGGTGAAATGCGTAGATATAGGAAGGAACACCAGTGG  668
             ||||||||||||||||||||||||||||||||||||||||||||||||||||||||||||
Sbjct  633   GGGTGGTGGAATTTCCTGTGTAGCGGTGAAATGCGTAGATATAGGAAGGAACACCAGTGG  692

Query  669   CGAAGGCGACCACCTGGACTAATACTGACACTGAGGTGCGAAAGCGTGGGGAGCAAACAG  728
             ||||||||||||||||||||||||||||||||||||||||||||||||||||||||||||
Sbjct  693   CGAAGGCGACCACCTGGACTAATACTGACACTGAGGTGCGAAAGCGTGGGGAGCAAACAG  752

Query  729   GATTAGATACCCTGGTAGTCCACGCCGTAAACGATGTCAACTAGCCGTTGGAAGCCTTGA  788
             ||||||||||||||||||||||||||||||||||||||||||||||||||||||||||||
Sbjct  753   GATTAGATACCCTGGTAGTCCACGCCGTAAACGATGTCAACTAGCCGTTGGAAGCCTTGA  812

Query  789   GCTTTTAGTGGCGCAGCTAACGCATTAAGTTGACCGCCTGGGGAGTACGGCCGCAAGGTT  848
             ||||||||||||||||||||||||||||||||||||||||||||||||||||||||||||
Sbjct  813   GCTTTTAGTGGCGCAGCTAACGCATTAAGTTGACCGCCTGGGGAGTACGGCCGCAAGGTT  872

Query  849   AAAACTCAAATGAATTGACGGGGGCCCGCACAAGCGGTGGAGCATGTGGTTTAATTCGAA  908
             ||||||||||||||||||||||||||||||||||||||||||||||||||||||||||||
Sbjct  873   AAAACTCAAATGAATTGACGGGGGCCCGCACAAGCGGTGGAGCATGTGGTTTAATTCGAA  932

Query  909   GCAACGCGAAGAACCTTACCAGGCCTTGACATCCAATGAACTTTCTAGAGATAGATTGGT  968
             ||||||||||||||||||||||||||||||||||||||||||||||||||||||||||||
Sbjct  933   GCAACGCGAAGAACCTTACCAGGCCTTGACATCCAATGAACTTTCTAGAGATAGATTGGT  992

Query  969   GCCTTCGGGAACATTGAGACAGGTGCTGCATGGCTGTCGTCAGCTCGTGT  1018
             ||||||||||||||||||||||||||||||||||||||||||||||||||
Sbjct  993   GCCTTCGGGAACATTGAGACAGGTGCTGCATGGCTGTCGTCAGCTCGTGT  1042
```

Download

FASTA (complete sequence)

FASTA (aligned sequences)

GenBank (complete sequence)

Continue
Cancel

GenBankGraphics

Next
Previous
Descriptions

Pseudomonas sp. PX7\_S1 16S ribosomal RNA gene, partial sequence

Sequence ID: JF274937.1Length: 1516Number of Matches: 1

Related Information

Range 1: 54 to 1063GenBankGraphics

Next Match
Previous Match
First Match

Alignment statistics for match #1

| Score | Expect | Identities | Gaps | Strand | Frame |
| --- | --- | --- | --- | --- | --- |
| 1866 bits(1010) | 0.0() | 1010/1010(100%) | 0/1010(0%) | Plus/Plus |  |

Features:

```
Query  9     AGTCGAGCGGTAGAGAGAAGCTTGCTTCTCTTGAGAGCGGCGGACGGGTGAGTAATGCCT  68
             ||||||||||||||||||||||||||||||||||||||||||||||||||||||||||||
Sbjct  54    AGTCGAGCGGTAGAGAGAAGCTTGCTTCTCTTGAGAGCGGCGGACGGGTGAGTAATGCCT  113

Query  69    AGGAATCTGCCTGGTAGTGGGGGATAACGTTCGGAAACGGACGCTAATACCGCATACGTC  128
             ||||||||||||||||||||||||||||||||||||||||||||||||||||||||||||
Sbjct  114   AGGAATCTGCCTGGTAGTGGGGGATAACGTTCGGAAACGGACGCTAATACCGCATACGTC  173

Query  129   CTACGGGAGAAAGCAGGGGACCTTCGGGCCTTGCGCTATCAGATGAGCCTAGGTCGGATT  188
             ||||||||||||||||||||||||||||||||||||||||||||||||||||||||||||
Sbjct  174   CTACGGGAGAAAGCAGGGGACCTTCGGGCCTTGCGCTATCAGATGAGCCTAGGTCGGATT  233

Query  189   AGCTAGTTGGTGGGGTAATGGCTCACCAAGGCGACGATCCGTAACTGGTCTGAGAGGATG  248
             ||||||||||||||||||||||||||||||||||||||||||||||||||||||||||||
Sbjct  234   AGCTAGTTGGTGGGGTAATGGCTCACCAAGGCGACGATCCGTAACTGGTCTGAGAGGATG  293

Query  249   ATCAGTCACACTGGAACTGAGACACGGTCCAGACTCCTACGGGAGGCAGCAGTGGGGAAT  308
             ||||||||||||||||||||||||||||||||||||||||||||||||||||||||||||
Sbjct  294   ATCAGTCACACTGGAACTGAGACACGGTCCAGACTCCTACGGGAGGCAGCAGTGGGGAAT  353

Query  309   ATTGGACAATGGGCGAAAGCCTGATCCAGCCATGCCGCGTGTGTGAAGAAGGTCTTCGGA  368
             ||||||||||||||||||||||||||||||||||||||||||||||||||||||||||||
Sbjct  354   ATTGGACAATGGGCGAAAGCCTGATCCAGCCATGCCGCGTGTGTGAAGAAGGTCTTCGGA  413

Query  369   TTGTAAAGCACTTTAAGTTGGGAGGAAGGGCAGTTACCTAATACGTGATTGTTTTGACGT  428
             ||||||||||||||||||||||||||||||||||||||||||||||||||||||||||||
Sbjct  414   TTGTAAAGCACTTTAAGTTGGGAGGAAGGGCAGTTACCTAATACGTGATTGTTTTGACGT  473

Query  429   TACCGACAGAATAAGCACCGGCTAACTCTGTGCCAGCAGCCGCGGTAATACAGAGGGTGC  488
             ||||||||||||||||||||||||||||||||||||||||||||||||||||||||||||
Sbjct  474   TACCGACAGAATAAGCACCGGCTAACTCTGTGCCAGCAGCCGCGGTAATACAGAGGGTGC  533

Query  489   AAGCGTTAATCGGAATTACTGGGCGTAAAGCGCGCGTAGGTGGTTTGTTAAGTTGGATGT  548
             ||||||||||||||||||||||||||||||||||||||||||||||||||||||||||||
Sbjct  534   AAGCGTTAATCGGAATTACTGGGCGTAAAGCGCGCGTAGGTGGTTTGTTAAGTTGGATGT  593

Query  549   GAAATCCCCGGGCTCAACCTGGGAACTGCATTCAAAACTGACTGACTAGAGTATGGTAGA  608
             ||||||||||||||||||||||||||||||||||||||||||||||||||||||||||||
Sbjct  594   GAAATCCCCGGGCTCAACCTGGGAACTGCATTCAAAACTGACTGACTAGAGTATGGTAGA  653

Query  609   GGGTGGTGGAATTTCCTGTGTAGCGGTGAAATGCGTAGATATAGGAAGGAACACCAGTGG  668
             ||||||||||||||||||||||||||||||||||||||||||||||||||||||||||||
Sbjct  654   GGGTGGTGGAATTTCCTGTGTAGCGGTGAAATGCGTAGATATAGGAAGGAACACCAGTGG  713

Query  669   CGAAGGCGACCACCTGGACTAATACTGACACTGAGGTGCGAAAGCGTGGGGAGCAAACAG  728
             ||||||||||||||||||||||||||||||||||||||||||||||||||||||||||||
Sbjct  714   CGAAGGCGACCACCTGGACTAATACTGACACTGAGGTGCGAAAGCGTGGGGAGCAAACAG  773

Query  729   GATTAGATACCCTGGTAGTCCACGCCGTAAACGATGTCAACTAGCCGTTGGAAGCCTTGA  788
             ||||||||||||||||||||||||||||||||||||||||||||||||||||||||||||
Sbjct  774   GATTAGATACCCTGGTAGTCCACGCCGTAAACGATGTCAACTAGCCGTTGGAAGCCTTGA  833

Query  789   GCTTTTAGTGGCGCAGCTAACGCATTAAGTTGACCGCCTGGGGAGTACGGCCGCAAGGTT  848
             ||||||||||||||||||||||||||||||||||||||||||||||||||||||||||||
Sbjct  834   GCTTTTAGTGGCGCAGCTAACGCATTAAGTTGACCGCCTGGGGAGTACGGCCGCAAGGTT  893

Query  849   AAAACTCAAATGAATTGACGGGGGCCCGCACAAGCGGTGGAGCATGTGGTTTAATTCGAA  908
             ||||||||||||||||||||||||||||||||||||||||||||||||||||||||||||
Sbjct  894   AAAACTCAAATGAATTGACGGGGGCCCGCACAAGCGGTGGAGCATGTGGTTTAATTCGAA  953

Query  909   GCAACGCGAAGAACCTTACCAGGCCTTGACATCCAATGAACTTTCTAGAGATAGATTGGT  968
             ||||||||||||||||||||||||||||||||||||||||||||||||||||||||||||
Sbjct  954   GCAACGCGAAGAACCTTACCAGGCCTTGACATCCAATGAACTTTCTAGAGATAGATTGGT  1013

Query  969   GCCTTCGGGAACATTGAGACAGGTGCTGCATGGCTGTCGTCAGCTCGTGT  1018
             ||||||||||||||||||||||||||||||||||||||||||||||||||
Sbjct  1014  GCCTTCGGGAACATTGAGACAGGTGCTGCATGGCTGTCGTCAGCTCGTGT  1063
```


BLAST is a registered trademark of the National Library of Medicine

Support center
Mailing list


YouTube

- National Library Of Medicine
- National Institutes Of Health
- U.S. Department of Health & Human Services
- USA.gov

### NCBI


National Center for Biotechnology Information,
 U.S. National Library of Medicine

8600 Rockville Pike,
Bethesda
 MD,
20894
USA

Policies and Guidelines
|
Contact


PreferencesTurn off

External link. Please review our privacy policy.
