# Supplementary material for: Isothermal microcalorimetry for thermal viable count of microorganisms in pure cultures and stabilized formulations
Source: BMC Microbiol. 2019 Mar 21;19:65. doi: 10.1186/s12866-019-1432-8 (PMC6429831; doi:10.1186/s12866-019-1432-8)
Supplement: Supplementary file 3 — 16S rRNA gene sequences’ BLASTN hits in zipped HTML format. (ZIP 15810 kb) [file 12866_2019_1432_MOESM3_ESM.zip › Best blastn hits/NCBI Blast_P pinkish R -- 11..1070 of sequence (1060.html]

NCBI Blast:P pinkish R -- 11..1070 of sequence (1060


- NCBI Home
- Sign in to NCBI
- Skip to Main Content
- Skip to Navigation
- About NCBI Accesskeys

U.S. National Library of Medicine

NCBI
National Center for Biotechnology Information

- My NCBI
- Sign in to NCBI
- Register
- Sign Out

BLAST ^®^ » blastn suite » RID-A50KTJ2B014


- Home
- Recent Results
- Saved Strategies
- Help

BLAST Results


Edit and Resubmit
Save Search Strategies
[Sign in above to save your search strategy]

Formatting options 


Download


How to read this page
Blast report description
Questions/comments


|  |  |
| --- | --- |
| Formatting options | |
| Show | Alignment as  HTML Plain text   Old View Reset form to defaults [?]  These options control formatting of alignments in results pages. The default is HTML, but other formats (including plain text) are available. PSSM and PssmWithParameters are representations of Position Specific Scoring Matrices and are only available for PSI-BLAST. The Advanced view option allows the database descriptions to be sorted by various indices in a table. |
| Alignment View | Pairwise Pairwise with dots for identities Query-anchored with dots for identities Query-anchored with letters for identities Flat query-anchored with dots for identities Flat query-anchored with letters for identities [?]  Choose how to view alignments. The default "pairwise" view shows how each subject sequence aligns individually to the query sequence. The "query-anchored" view shows how all subject sequences align to the query sequence. For each view type, you can choose to show "identities" (matching residues) as letters or dots. more... |
| Display | Graphical Overview   Linkout   Sequence Retrieval  NCBI-gi   CDS feature [?]  - Graphical Overview: Graphical Overview: Show graph of similar sequence regions aligned to query.   more... - NCBI-gi: Show NCBI gi identifiers. - CDS feature: Show annotated coding region and translation.   more... |
| Masking | Character:   X for protein, n for nucleotide Lower Case  Color:  Black Grey Red [?]  - Masking Character: Display masked (filtered) sequence regions as lower-case or as specific letters (N for nucleotide, P for protein). - Masking Color: Display masked sequence regions in the given color. |
| Limit results | Descriptions:  10 50 100 Graphical overview:  0 10 50 100  Alignments:  0 10 50 100 Line length:  60 90 120 150 [?]  - Descriptions: Show short descriptions for up to the given number of sequences. - Alignments: Show alignments for up to the given number of sequences, in order of statistical significance. - Line lenghth: Number of letters to show on one line in an alignment. |
|  | Organism Type common name, binomial, taxid, or group name. Only 20 top taxa will be shown.     Exclude   [?]  Show only sequences from the given organism. |
|  | Entrez query:  [?]  Show only those sequences that match the given Entrez query. more... |
|  | Expect Min:  Expect Max:  [?]  Show only sequences with expect values in the given range. more... |
|  | Percent Identity Min:  Percent Identity Max:  [?]  Show only sequences with percent identity values in the given range. |
| Format for | PSI-BLAST with inclusion threshold:  [?]  - Format for PSI-BLAST: The Position-Specific Iterated BLAST (PSI-BLAST) program performs iterative searches with a protein query,   in which sequences found in one round of search are used to build a custom score model for the next round.   more... - Inclusion Threshold: This sets the statistical significance threshold for including a sequence in the model used   by PSI-BLAST to create the PSSM on the next iteration. |

|  |  |  |  |  |  |
| --- | --- | --- | --- | --- | --- |
| Download | | | | | |
| Alignment  Text XML ASN.1 JSON Seq-align Hit Table(text) Hit Table(csv) Multiple-file XML2 Single-file XML2 Multiple-file JSON Single-file JSON SAM | Search Strategies  ASN.1 | PSSM to restart search  PSSM | [?] |  |  |

The Download link provides BLAST output that may be used as input to another program.
This includes parseable formats such as the tabular report or XML as well as the Search Strategy files read by the BLAST+ applications.
More details on the parseable (XML, tabular, and ASN.1) reports can be found at
https://www.ncbi.nlm.nih.gov/books/NBK153387/  
  

The following formats are offered under the Alignment section:  
1). "Text". Non-HTML standard BLAST report.  
2). "XML". XML report based upon the DTD at https://www.ncbi.nlm.nih.gov/data\_specs/dtd/NCBI\_BlastOutput.dtd  
3). "ASN.1". Alignment written out in Abstract Syntax Notation 1.  
4). "JSON Seq-align". Alignment written out in JSON.  
4). "Hit Table(text)". The tabular report as text.  
5). "Hit Table(csv)". The tabular report ready for import into spread-sheet programs like Excel.  
6). "XML2". New XML format described at ftp://ftp.ncbi.nlm.nih.gov/blast/documents/NEWXML/xml2.pdf.  
7). "JSON". New JSON format described at ftp://ftp.ncbi.nlm.nih.gov/blast/documents/NEWXML/xml2.pdf.  
8). "SAM". Sequence Alignment Map format.

XML2 and JSON can be downloaded either as one file per query (multiple-file) or one file for all queries (single-file). These formats are listed as Multiple-file XML2 (and JSON) or Single-file XML (and JSON).

The following report is offered under the Search Strategy section:  
1). "ASN.1" Search Strategy. A record of the parameters, query, and database used in the search. This file can be used to start a stand-alone BLAST search, see
https://www.ncbi.nlm.nih.gov/books/NBK1763/#CmdLineAppsManual.I455\_BLAST\_search\_stra


# Job title: P pinkish R -- 11..1070 of sequence (1060

Results for:

lcl|Query\_15701 P pinkish R -- 11..1070 of sequence(1060bp)
[?]

Your BLAST job specified more than one input sequence.
This box lets you choose which input sequence to show BLAST results for.

RID
:   A50KTJ2B014 (Expires on 03-10 19:47 pm)

Query ID
:   lcl|Query\_15701
:   lcl|Query\_15701

Description
:   P pinkish R -- 11..1070 of sequence

Molecule type
:   nucleic acid

Query Length
:   1060

Database Name
:   nr

Description
:   Nucleotide collection (nt) See details

Program
:   BLASTN 2.8.0+ Citation

  

Reference 

Zheng Zhang, Scott Schwartz, Lukas Wagner, and Webb Miller (2000), "A greedy algorithm for aligning DNA sequences", J Comput Biol 2000; 7(1-2):203-14.

Reference - database indexing

Aleksandr Morgulis, George Coulouris, Yan Raytselis, Thomas L. Madden, Richa Agarwala, Alejandro A. Schäffer (2008), "Database Indexing for Production MegaBLAST Searches", Bioinformatics 24:1757-1764.

Other reports:
Search Summary

[Taxonomy reports]
[Distance tree of results]
[MSA viewer]

Search Parameters

| Search parameter name | Search parameter value |
| --- | --- |
| Program | blastn |
| Word size | 28 |
| Expect value | 10 |
| Hitlist size | 100 |
| Match/Mismatch scores | 1,-2 |
| Gapcosts | 0,2.5 |
| Low Complexity Filter | Yes |
| Filter string | L;m; |
| Genetic Code | 1 |

Database

| Database parameter name | Database parameter value |
| --- | --- |
| Posted date | Mar 7, 2018 1:58 PM |
| Number of letters | 174,044,644,244 |
| Number of sequences | 46,882,714 |
| Entrez query | Includes:  Excludes:  None |

Karlin-Altschul statistics

| Params | Ungapped | Gapped |
| --- | --- | --- |
| Lambda | 1.33271 | 1.28 |
| K | 0.620991 | 0.46 |
| H | 1.12409 | 0.85 |

Results Statistics

| Results Statistics parameter name | Results Statistics parameter value |
| --- | --- |
| Length adjustment | 35 |
| Effective length of query | 1025 |
| Effective length of database | 172403749254 |
| Effective search space | 176713842985350 |
| Effective search space used | 176713842985350 |


## Graphic Summary

### Distribution of the top 137 Blast Hits on 100 subject sequences [?]

The graphic is an overview of the database sequences aligned to the query sequence. These are represented horizontal bars colored coded by score and showing the extent
of the alignment on the query sequence. Separate aligned regions on the same database sequence are connected by a thin grey line.
Mousing over an alignment shows the database sequence title. Clicking an alignment displays a box with more details about the alignment and
link to the sequence alignment itself in the Alignments section of the report.

Mouse over to see the title, click to show alignments

Color key for alignment scores

<40

40-50

50-80

80-200

>=200

Query

1

200

400

600

800

1000

Pseudomonas trivialis strain BS3111 genome assembly, ch..

Score:1927 Evalue:0

Accession:LT629760.1

Alignment

Uncultured bacterium clone 19-26 16S ribosomal RNA gene..

Score:1927 Evalue:0

Accession:KT029390.1

Alignment

Uncultured bacterium clone 19-14 16S ribosomal RNA gene..

Score:1927 Evalue:0

Accession:KT029378.1

Alignment

Pseudomonas trivialis strain P 513/19 16S ribosomal RNA..

Score:1927 Evalue:0

Accession:NR\_028987.1

Alignment

Pseudomonas trivialis partial 16S rRNA gene, type strai..

Score:1927 Evalue:0

Accession:AJ492831.1

Alignment

Pseudomonas sp. S09G 359 chromosome

Score:1921 Evalue:0

Accession:CP025263.1

Alignment

Pseudomonas sp. strain 36 16S ribosomal RNA gene, parti..

Score:1921 Evalue:0

Accession:KY681891.1

Alignment

Pseudomonas poae strain A2B5 16S ribosomal RNA gene, pa..

Score:1921 Evalue:0

Accession:KY940313.1

Alignment

Pseudomonas poae strain AAS-144 16S ribosomal RNA gene,..

Score:1921 Evalue:0

Accession:KY810658.1

Alignment

Pseudomonas poae strain BS2776 genome assembly, chromos..

Score:1921 Evalue:0

Accession:LT629706.1

Alignment

Uncultured bacterium partial 16S rRNA gene, clone clone..

Score:1921 Evalue:0

Accession:LT595870.1

Alignment

Uncultured bacterium clone MTWL201306-19 16S ribosomal ..

Score:1921 Evalue:0

Accession:KX509247.1

Alignment

Pseudomonas sp. RZ110 16S ribosomal RNA gene, partial s..

Score:1921 Evalue:0

Accession:KT710812.1

Alignment

Pseudomonas sp. 17K8 16S ribosomal RNA gene, partial se..

Score:1921 Evalue:0

Accession:KT825736.1

Alignment

Pseudomonas sp. LB8 16S ribosomal RNA gene, partial seq..

Score:1921 Evalue:0

Accession:KT424973.1

Alignment

Pseudomonas sp. PDD-69b-25 16S ribosomal RNA gene, comp..

Score:1921 Evalue:0

Accession:KR922293.1

Alignment

Pseudomonas poae strain IHBB 9211 16S ribosomal RNA gen..

Score:1921 Evalue:0

Accession:KR085822.1

Alignment

Uncultured bacterium clone 19-23 16S ribosomal RNA gene..

Score:1921 Evalue:0

Accession:KT029387.1

Alignment

Uncultured bacterium clone 19-18 16S ribosomal RNA gene..

Score:1921 Evalue:0

Accession:KT029382.1

Alignment

Pseudomonas trivialis strain IHBB745, complete genome

Score:1921 Evalue:0

Accession:CP011507.1

Alignment

Pseudomonas fluorescens PICF7, complete genome

Score:1921 Evalue:0

Accession:CP005975.1

Alignment

Pseudomonas fluorescens strain PCL1751, complete genome

Score:1921 Evalue:0

Accession:CP010896.1

Alignment

Pseudomonas simiae strain WCS417 genome

Score:1921 Evalue:0

Accession:CP007637.1

Alignment

Pseudomonas fluorescens strain PE8 16S ribosomal RNA ge..

Score:1921 Evalue:0

Accession:KJ420524.1

Alignment

Pseudomonas fluorescens strain 20130311XA1 16S ribosoma..

Score:1921 Evalue:0

Accession:KC773764.1

Alignment

Pseudomonas salomonii strain CCNWQLS112 16S ribosomal R..

Score:1921 Evalue:0

Accession:JX840381.1

Alignment

Pseudomonas poae strain zol-15 16S ribosomal RNA gene, ..

Score:1921 Evalue:0

Accession:JQ782898.1

Alignment

Pseudomonas sp. TK-3 gene for 16S rRNA, partial sequenc..

Score:1921 Evalue:0

Accession:AB705621.1

Alignment

Pseudomonas sp. SGb136 16S ribosomal RNA gene, partial ..

Score:1921 Evalue:0

Accession:HQ224624.1

Alignment

Pseudomonas trivialis strain B24 16S ribosomal RNA gene..

Score:1921 Evalue:0

Accession:JN377670.1

Alignment

Uncultured bacterium clone EDW07B003\_41 16S ribosomal R..

Score:1921 Evalue:0

Accession:HM066455.1

Alignment

Uncultured Pseudomonas sp. clone CHINA4 16S ribosomal R..

Score:1921 Evalue:0

Accession:GU563752.1

Alignment

Pseudomonas poae strain P 527/13 16S ribosomal RNA gene..

Score:1921 Evalue:0

Accession:NR\_028986.1

Alignment

Pseudomonas trivialis strain EQH4 16S ribosomal RNA gen..

Score:1921 Evalue:0

Accession:FJ999933.1

Alignment

Pseudomonas poae strain BIHB 808 16S ribosomal RNA gene..

Score:1921 Evalue:0

Accession:FJ179369.1

Alignment

Pseudomonas trivialis strain BIHB 750 16S ribosomal RNA..

Score:1921 Evalue:0

Accession:FJ179366.1

Alignment

Pseudomonas fluorescens strain 2312 16S ribosomal RNA g..

Score:1921 Evalue:0

Accession:EU360313.1

Alignment

Pseudomonas poae strain CN017 16S ribosomal RNA gene, p..

Score:1921 Evalue:0

Accession:EU364532.1

Alignment

Pseudomonas trivialis strain BIHB 749 16S ribosomal RNA..

Score:1921 Evalue:0

Accession:DQ885949.1

Alignment

Pseudomonas trivialis strain BIHB 759 16S ribosomal RNA..

Score:1921 Evalue:0

Accession:DQ536520.1

Alignment

Pseudomonas trivialis strain BIHB 757 16S ribosomal RNA..

Score:1921 Evalue:0

Accession:DQ536519.1

Alignment

Pseudomonas trivialis strain BIHB 745 16S ribosomal RNA..

Score:1921 Evalue:0

Accession:DQ536516.1

Alignment

Pseudomonas poae strain BIHB 730 16S ribosomal RNA gene..

Score:1921 Evalue:0

Accession:DQ536513.1

Alignment

Pseudomonas sp. TB3-6-I 16S ribosomal RNA gene, partial..

Score:1921 Evalue:0

Accession:AY599721.1

Alignment

Pseudomonas sp. CH8 partial 16S rRNA gene, isolate CH8

Score:1921 Evalue:0

Accession:AJ582401.1

Alignment

Pseudomonas poae partial 16S rRNA gene, type strain DSM..

Score:1921 Evalue:0

Accession:AJ492829.1

Alignment

Pseudomonas sp. strain KBL2 16S ribosomal RNA gene, par..

Score:1919 Evalue:0

Accession:MG576155.1

Alignment

Pseudomonas sp. 8K8 16S ribosomal RNA gene, partial seq..

Score:1919 Evalue:0

Accession:KT825845.1

Alignment

Pseudomonas poae strain B42 16S ribosomal RNA gene, par..

Score:1919 Evalue:0

Accession:KT767705.1

Alignment

Pseudomonas sp. HP10C 16S ribosomal RNA gene, partial s..

Score:1919 Evalue:0

Accession:KM187375.1

Alignment

Pseudomonas sp. 2-36(2010) 16S ribosomal RNA gene, part..

Score:1919 Evalue:0

Accession:HQ403088.2

Alignment

Pseudomonas fluorescens strain SWFU22 16S ribosomal RNA..

Score:1919 Evalue:0

Accession:KJ756337.1

Alignment

Pseudomonas mandelii 16S ribosomal RNA gene, partial se..

Score:1919 Evalue:0

Accession:KC618443.1

Alignment

Pseudomonas putida partial 16S rRNA gene, strain CFBP 5..

Score:1919 Evalue:0

Accession:HF545843.1

Alignment

Pseudomonas sp. isolate PS2.1 16S ribosomal RNA gene, p..

Score:1919 Evalue:0

Accession:KF019106.1

Alignment

Pseudomonas sp. Bma14 16S ribosomal RNA gene, partial s..

Score:1919 Evalue:0

Accession:JQ977483.1

Alignment

## Descriptions

, Reading indexes 1-5, displaying indexes 1-5


Load next setPrevious Match

Sequences producing significant alignments:

Show all columns  of the table presenting sequences producing significant alignments 

Select:AllNone
Selected:0

Alignments
Download

FASTA (complete sequence)

FASTA (aligned sequences)

GenBank (complete sequence)

Hit Table (text)

Hit Table (CSV)

Text

XML

ASN.1

Continue
Cancel

GenBank 
Graphics
Distance tree of results
Multiple alignment
Show/hide columns of the table presenting sequences producing significant alignments 

Available columns

Description  
Max Score  
Total Score  
Coverage  
E-value  
IdentN  
Accession  
Restore Defaults
Ok
Cancel

Sequences producing significant alignments:

| Select for downloading or viewing reports | Description | Max score | Total score | Query cover | E value | Ident | Accession |
| --- | --- | --- | --- | --- | --- | --- | --- |
| 1Select seq LT629760.1 | Pseudomonas trivialis strain BS3111 genome assembly, chromosome: I | 1927 | 9630 | 98% | 0.0 | 99% | LT629760.1 |
| 2Select seq KT029390.1 | Uncultured bacterium clone 19-26 16S ribosomal RNA gene, partial sequence | 1927 | 1927 | 98% | 0.0 | 99% | KT029390.1 |
| 3Select seq KT029378.1 | Uncultured bacterium clone 19-14 16S ribosomal RNA gene, partial sequence | 1927 | 1927 | 98% | 0.0 | 99% | KT029378.1 |
| 4Select seq NR\_028987.1 | Pseudomonas trivialis strain P 513/19 16S ribosomal RNA gene, partial sequence | 1927 | 1927 | 98% | 0.0 | 99% | NR\_028987.1 |
| 5Select seq AJ492831.1 | Pseudomonas trivialis partial 16S rRNA gene, type strain DSM 14937T | 1927 | 1927 | 98% | 0.0 | 99% | AJ492831.1 |
| 6Select seq CP025263.1 | Pseudomonas sp. S09G 359 chromosome | 1921 | 11507 | 98% | 0.0 | 99% | CP025263.1 |
| 7Select seq KY681891.1 | Pseudomonas sp. strain 36 16S ribosomal RNA gene, partial sequence | 1921 | 1921 | 98% | 0.0 | 99% | KY681891.1 |
| 8Select seq KY940313.1 | Pseudomonas poae strain A2B5 16S ribosomal RNA gene, partial sequence | 1921 | 1921 | 98% | 0.0 | 99% | KY940313.1 |
| 9Select seq KY810658.1 | Pseudomonas poae strain AAS-144 16S ribosomal RNA gene, partial sequence | 1921 | 1921 | 98% | 0.0 | 99% | KY810658.1 |
| 10Select seq LT629706.1 | Pseudomonas poae strain BS2776 genome assembly, chromosome: I | 1921 | 9608 | 98% | 0.0 | 99% | LT629706.1 |
| 11Select seq LT595870.1 | Uncultured bacterium partial 16S rRNA gene, clone clone AF-36051 | 1921 | 1921 | 98% | 0.0 | 99% | LT595870.1 |
| 12Select seq KX509247.1 | Uncultured bacterium clone MTWL201306-19 16S ribosomal RNA gene, partial sequence | 1921 | 1921 | 98% | 0.0 | 99% | KX509247.1 |
| 13Select seq KT710812.1 | Pseudomonas sp. RZ110 16S ribosomal RNA gene, partial sequence | 1921 | 1921 | 98% | 0.0 | 99% | KT710812.1 |
| 14Select seq KT825736.1 | Pseudomonas sp. 17K8 16S ribosomal RNA gene, partial sequence | 1921 | 1921 | 98% | 0.0 | 99% | KT825736.1 |
| 15Select seq KT424973.1 | Pseudomonas sp. LB8 16S ribosomal RNA gene, partial sequence | 1921 | 1921 | 98% | 0.0 | 99% | KT424973.1 |
| 16Select seq KR922293.1 | Pseudomonas sp. PDD-69b-25 16S ribosomal RNA gene, complete sequence | 1921 | 1921 | 98% | 0.0 | 99% | KR922293.1 |
| 17Select seq KR085822.1 | Pseudomonas poae strain IHBB 9211 16S ribosomal RNA gene, partial sequence | 1921 | 1921 | 98% | 0.0 | 99% | KR085822.1 |
| 18Select seq KT029387.1 | Uncultured bacterium clone 19-23 16S ribosomal RNA gene, partial sequence | 1921 | 1921 | 98% | 0.0 | 99% | KT029387.1 |
| 19Select seq KT029382.1 | Uncultured bacterium clone 19-18 16S ribosomal RNA gene, partial sequence | 1921 | 1921 | 98% | 0.0 | 99% | KT029382.1 |
| 20Select seq CP011507.1 | Pseudomonas trivialis strain IHBB745, complete genome | 1921 | 9608 | 98% | 0.0 | 99% | CP011507.1 |
| 21Select seq CP005975.1 | Pseudomonas fluorescens PICF7, complete genome | 1921 | 11529 | 98% | 0.0 | 99% | CP005975.1 |
| 22Select seq CP010896.1 | Pseudomonas fluorescens strain PCL1751, complete genome | 1921 | 11513 | 98% | 0.0 | 99% | CP010896.1 |
| 23Select seq CP007637.1 | Pseudomonas simiae strain WCS417 genome | 1921 | 3843 | 98% | 0.0 | 99% | CP007637.1 |
| 24Select seq KJ420524.1 | Pseudomonas fluorescens strain PE8 16S ribosomal RNA gene, partial sequence | 1921 | 1921 | 98% | 0.0 | 99% | KJ420524.1 |
| 25Select seq KC773764.1 | Pseudomonas fluorescens strain 20130311XA1 16S ribosomal RNA gene, partial sequence | 1921 | 1921 | 98% | 0.0 | 99% | KC773764.1 |
| 26Select seq JX840381.1 | Pseudomonas salomonii strain CCNWQLS112 16S ribosomal RNA gene, partial sequence | 1921 | 1921 | 98% | 0.0 | 99% | JX840381.1 |
| 27Select seq JQ782898.1 | Pseudomonas poae strain zol-15 16S ribosomal RNA gene, partial sequence | 1921 | 1921 | 98% | 0.0 | 99% | JQ782898.1 |
| 28Select seq AB705621.1 | Pseudomonas sp. TK-3 gene for 16S rRNA, partial sequence | 1921 | 1921 | 98% | 0.0 | 99% | AB705621.1 |
| 29Select seq HQ224624.1 | Pseudomonas sp. SGb136 16S ribosomal RNA gene, partial sequence | 1921 | 1921 | 98% | 0.0 | 99% | HQ224624.1 |
| 30Select seq JN377670.1 | Pseudomonas trivialis strain B24 16S ribosomal RNA gene, partial sequence | 1921 | 1921 | 98% | 0.0 | 99% | JN377670.1 |
| 31Select seq HM066455.1 | Uncultured bacterium clone EDW07B003\_41 16S ribosomal RNA gene, partial sequence | 1921 | 1921 | 98% | 0.0 | 99% | HM066455.1 |
| 32Select seq GU563752.1 | Uncultured Pseudomonas sp. clone CHINA4 16S ribosomal RNA gene, partial sequence | 1921 | 1921 | 98% | 0.0 | 99% | GU563752.1 |
| 33Select seq NR\_028986.1 | Pseudomonas poae strain P 527/13 16S ribosomal RNA gene, partial sequence | 1921 | 1921 | 98% | 0.0 | 99% | NR\_028986.1 |
| 34Select seq FJ999933.1 | Pseudomonas trivialis strain EQH4 16S ribosomal RNA gene, partial sequence | 1921 | 1921 | 98% | 0.0 | 99% | FJ999933.1 |
| 35Select seq FJ179369.1 | Pseudomonas poae strain BIHB 808 16S ribosomal RNA gene, partial sequence | 1921 | 1921 | 98% | 0.0 | 99% | FJ179369.1 |
| 36Select seq FJ179366.1 | Pseudomonas trivialis strain BIHB 750 16S ribosomal RNA gene, partial sequence | 1921 | 1921 | 98% | 0.0 | 99% | FJ179366.1 |
| 37Select seq EU360313.1 | Pseudomonas fluorescens strain 2312 16S ribosomal RNA gene and 16S-23S ribosomal RNA intergenic spacer, complete sequence; and 23S ribosomal RNA gene, partial sequence | 1921 | 1921 | 98% | 0.0 | 99% | EU360313.1 |
| 38Select seq EU364532.1 | Pseudomonas poae strain CN017 16S ribosomal RNA gene, partial sequence | 1921 | 1921 | 98% | 0.0 | 99% | EU364532.1 |
| 39Select seq DQ885949.1 | Pseudomonas trivialis strain BIHB 749 16S ribosomal RNA gene, partial sequence | 1921 | 1921 | 98% | 0.0 | 99% | DQ885949.1 |
| 40Select seq DQ536520.1 | Pseudomonas trivialis strain BIHB 759 16S ribosomal RNA gene, partial sequence | 1921 | 1921 | 98% | 0.0 | 99% | DQ536520.1 |
| 41Select seq DQ536519.1 | Pseudomonas trivialis strain BIHB 757 16S ribosomal RNA gene, partial sequence | 1921 | 1921 | 98% | 0.0 | 99% | DQ536519.1 |
| 42Select seq DQ536516.1 | Pseudomonas trivialis strain BIHB 745 16S ribosomal RNA gene, partial sequence | 1921 | 1921 | 98% | 0.0 | 99% | DQ536516.1 |
| 43Select seq DQ536513.1 | Pseudomonas poae strain BIHB 730 16S ribosomal RNA gene, partial sequence | 1921 | 1921 | 98% | 0.0 | 99% | DQ536513.1 |
| 44Select seq AY599721.1 | Pseudomonas sp. TB3-6-I 16S ribosomal RNA gene, partial sequence | 1921 | 1921 | 98% | 0.0 | 99% | AY599721.1 |
| 45Select seq AJ582401.1 | Pseudomonas sp. CH8 partial 16S rRNA gene, isolate CH8 | 1921 | 1921 | 98% | 0.0 | 99% | AJ582401.1 |
| 46Select seq AJ492829.1 | Pseudomonas poae partial 16S rRNA gene, type strain DSM 14936T | 1921 | 1921 | 98% | 0.0 | 99% | AJ492829.1 |
| 47Select seq MG576155.1 | Pseudomonas sp. strain KBL2 16S ribosomal RNA gene, partial sequence | 1919 | 1919 | 98% | 0.0 | 99% | MG576155.1 |
| 48Select seq KT825845.1 | Pseudomonas sp. 8K8 16S ribosomal RNA gene, partial sequence | 1919 | 1919 | 98% | 0.0 | 99% | KT825845.1 |
| 49Select seq KT767705.1 | Pseudomonas poae strain B42 16S ribosomal RNA gene, partial sequence | 1919 | 1919 | 98% | 0.0 | 99% | KT767705.1 |
| 50Select seq KM187375.1 | Pseudomonas sp. HP10C 16S ribosomal RNA gene, partial sequence | 1919 | 1919 | 98% | 0.0 | 99% | KM187375.1 |
| 51Select seq HQ403088.2 | Pseudomonas sp. 2-36(2010) 16S ribosomal RNA gene, partial sequence | 1919 | 1919 | 98% | 0.0 | 99% | HQ403088.2 |
| 52Select seq KJ756337.1 | Pseudomonas fluorescens strain SWFU22 16S ribosomal RNA gene, partial sequence | 1919 | 1919 | 98% | 0.0 | 99% | KJ756337.1 |
| 53Select seq KC618443.1 | Pseudomonas mandelii 16S ribosomal RNA gene, partial sequence | 1919 | 1919 | 98% | 0.0 | 99% | KC618443.1 |
| 54Select seq HF545843.1 | Pseudomonas putida partial 16S rRNA gene, strain CFBP 5898 | 1919 | 1919 | 98% | 0.0 | 99% | HF545843.1 |
| 55Select seq KF019106.1 | Pseudomonas sp. isolate PS2.1 16S ribosomal RNA gene, partial sequence | 1919 | 1919 | 98% | 0.0 | 99% | KF019106.1 |
| 56Select seq JQ977483.1 | Pseudomonas sp. Bma14 16S ribosomal RNA gene, partial sequence | 1919 | 1919 | 98% | 0.0 | 99% | JQ977483.1 |
| 57Select seq JQ236630.1 | Pseudomonas poae strain ORC5 16S ribosomal RNA gene, partial sequence | 1919 | 1919 | 98% | 0.0 | 99% | JQ236630.1 |
| 58Select seq HQ224626.1 | Pseudomonas sp. SGb192 16S ribosomal RNA gene, partial sequence | 1919 | 1919 | 98% | 0.0 | 99% | HQ224626.1 |
| 59Select seq JF808098.1 | Pseudomonas sp. 5(2011) 16S ribosomal RNA gene, partial sequence | 1919 | 1919 | 98% | 0.0 | 99% | JF808098.1 |
| 60Select seq JF701975.1 | Pseudomonas sp. YXE2-f 16S ribosomal RNA gene, partial sequence | 1919 | 1919 | 98% | 0.0 | 99% | JF701975.1 |
| 61Select seq JF820107.1 | Pseudomonas sp. PG-3-10 16S ribosomal RNA gene, partial sequence | 1919 | 1919 | 98% | 0.0 | 99% | JF820107.1 |
| 62Select seq FJ262366.1 | Pseudomonas sp. WH1 16S ribosomal RNA gene, partial sequence | 1919 | 1919 | 98% | 0.0 | 99% | FJ262366.1 |
| 63Select seq EU169159.1 | Pseudomonas mandelii strain B61 16S ribosomal RNA gene, partial sequence | 1919 | 1919 | 98% | 0.0 | 99% | EU169159.1 |
| 64Select seq EF427849.1 | Pseudomonas sp. CBCEN8 16S ribosomal RNA gene, partial sequence | 1919 | 1919 | 98% | 0.0 | 99% | EF427849.1 |
| 65Select seq EF126756.1 | Pseudomonas sp. CBMB39 16S ribosomal RNA gene, partial sequence | 1919 | 1919 | 98% | 0.0 | 99% | EF126756.1 |
| 66Select seq EF126755.1 | Pseudomonas sp. CBMB25 16S ribosomal RNA gene, partial sequence | 1919 | 1919 | 98% | 0.0 | 99% | EF126755.1 |
| 67Select seq EF126753.1 | Pseudomonas sp. CBMB18 16S ribosomal RNA gene, partial sequence | 1919 | 1919 | 98% | 0.0 | 99% | EF126753.1 |
| 68Select seq EU680981.1 | Pseudomonas sp. W15Feb3 16S ribosomal RNA gene, partial sequence | 1917 | 1917 | 98% | 0.0 | 99% | EU680981.1 |
| 69Select seq EU680980.1 | Pseudomonas sp. W15Feb2 16S ribosomal RNA gene, partial sequence | 1917 | 1917 | 98% | 0.0 | 99% | EU680980.1 |
| 70Select seq KY681890.1 | Pseudomonas sp. strain 3 16S ribosomal RNA gene, partial sequence | 1916 | 1916 | 98% | 0.0 | 99% | KY681890.1 |
| 71Select seq KX186936.1 | Pseudomonas antarctica culture DSM:15318 16S ribosomal RNA gene, partial sequence | 1916 | 1916 | 98% | 0.0 | 99% | KX186936.1 |
| 72Select seq LT629704.1 | Pseudomonas antarctica strain BS2772 genome assembly, chromosome: I | 1916 | 9838 | 98% | 0.0 | 99% | LT629704.1 |
| 73Select seq KX378900.1 | Pseudomonas sp. strain 1-29 16S ribosomal RNA gene, partial sequence | 1916 | 1916 | 98% | 0.0 | 99% | KX378900.1 |
| 74Select seq LC140869.1 | Uncultured Pseudomonas sp. gene for 16S ribosomal RNA, partial sequence, clone: 89d159 | 1916 | 1916 | 98% | 0.0 | 99% | LC140869.1 |
| 75Select seq KT825765.1 | Pseudomonas sp. 23K33a 16S ribosomal RNA gene, partial sequence | 1916 | 1916 | 98% | 0.0 | 99% | KT825765.1 |
| 76Select seq KT029445.1 | Uncultured bacterium clone 21-10 16S ribosomal RNA gene, partial sequence | 1916 | 1916 | 98% | 0.0 | 99% | KT029445.1 |
| 77Select seq KT029395.1 | Uncultured bacterium clone 19-6 16S ribosomal RNA gene, partial sequence | 1916 | 1916 | 98% | 0.0 | 99% | KT029395.1 |
| 78Select seq KT029380.1 | Uncultured bacterium clone 19-16 16S ribosomal RNA gene, partial sequence | 1916 | 1916 | 98% | 0.0 | 99% | KT029380.1 |
| 79Select seq KM461107.1 | Pseudomonas sp. WHAS15 16S ribosomal RNA gene, partial sequence | 1916 | 1916 | 98% | 0.0 | 99% | KM461107.1 |
| 80Select seq KJ848593.1 | Pseudomonas poae strain BT 89 16S ribosomal RNA gene, partial sequence | 1916 | 1916 | 98% | 0.0 | 99% | KJ848593.1 |
| 81Select seq KJ756336.1 | Pseudomonas fluorescens strain SWFU21 16S ribosomal RNA gene, partial sequence | 1916 | 1916 | 98% | 0.0 | 99% | KJ756336.1 |
| 82Select seq KJ420526.1 | Pseudomonas fluorescens strain PE16 16S ribosomal RNA gene, partial sequence | 1916 | 1916 | 98% | 0.0 | 99% | KJ420526.1 |
| 83Select seq KJ127239.1 | Pseudomonas fluorescens strain PE3 16S ribosomal RNA gene, partial sequence | 1916 | 1916 | 98% | 0.0 | 99% | KJ127239.1 |
| 84Select seq KF003385.1 | Pseudomonas sp. ABAC21 16S ribosomal RNA gene, partial sequence | 1916 | 1916 | 98% | 0.0 | 99% | KF003385.1 |
| 85Select seq CP004045.1 | Pseudomonas poae RE\*1-1-14, complete genome | 1916 | 9580 | 98% | 0.0 | 99% | CP004045.1 |
| 86Select seq JX067683.1 | Pseudomonas sp. SAP770.2 16S ribosomal RNA gene, partial sequence | 1916 | 1916 | 98% | 0.0 | 99% | JX067683.1 |
| 87Select seq JQ782893.1 | Pseudomonas poae strain Car-06 16S ribosomal RNA gene, partial sequence | 1916 | 1916 | 98% | 0.0 | 99% | JQ782893.1 |
| 88Select seq HE576021.1 | uncultured bacterium partial 16S rRNA gene, clone K15.87 KSS | 1916 | 1916 | 98% | 0.0 | 99% | HE576021.1 |
| 89Select seq JF274937.1 | Pseudomonas sp. PX7\_S1 16S ribosomal RNA gene, partial sequence | 1916 | 1916 | 98% | 0.0 | 99% | JF274937.1 |
| 90Select seq JF327472.1 | Pseudomonas sp. KB47 16S ribosomal RNA gene, partial sequence | 1916 | 1916 | 98% | 0.0 | 99% | JF327472.1 |
| 91Select seq AB680977.1 | Pseudomonas fluorescens gene for 16S rRNA, partial sequence, strain: NBRC 15838 | 1916 | 1916 | 98% | 0.0 | 99% | AB680977.1 |
| 92Select seq HE586386.1 | Pseudomonas antarctica partial 16S rRNA gene, strain LMG 23832 | 1916 | 1916 | 98% | 0.0 | 99% | HE586386.1 |
| 93Select seq JF703597.1 | Uncultured bacterium clone GcG58 16S ribosomal RNA gene, partial sequence | 1916 | 1916 | 98% | 0.0 | 99% | JF703597.1 |
| 94Select seq HM066471.1 | Uncultured bacterium clone EDW07B003\_70 16S ribosomal RNA gene, partial sequence | 1916 | 1916 | 98% | 0.0 | 99% | HM066471.1 |
| 95Select seq FJ849527.1 | Uncultured bacterium clone SedUMA10 16S ribosomal RNA gene, partial sequence | 1916 | 1916 | 98% | 0.0 | 99% | FJ849527.1 |
| 96Select seq GU198111.1 | Pseudomonas fluorescens strain LMG 5833 16S ribosomal RNA gene, partial sequence | 1916 | 1916 | 98% | 0.0 | 99% | GU198111.1 |
| 97Select seq EU681015.1 | Pseudomonas sp. W15Feb32 16S ribosomal RNA gene, partial sequence | 1916 | 1916 | 98% | 0.0 | 99% | EU681015.1 |
| 98Select seq EU360314.1 | Pseudomonas fluorescens 2313 16S ribosomal RNA gene, complete sequence; and 23S ribosomal RNA gene, partial sequence | 1916 | 1916 | 98% | 0.0 | 99% | EU360314.1 |
| 99Select seq DQ898312.2 | Pseudomonas sp. Pb1(2006) small subunit ribosomal RNA gene, partial sequence | 1916 | 1916 | 98% | 0.0 | 99% | DQ898312.2 |
| 100Select seq DQ536517.1 | Pseudomonas trivialis strain BIHB 747 16S ribosomal RNA gene, partial sequence | 1916 | 1916 | 98% | 0.0 | 99% | DQ536517.1 |


## Alignments

Loading alignment... for sequences gi|1085875934,gi|901409223,gi|901409211,gi|265678682,gi|21726939 Reading indexes 1-5

Download

FASTA (complete sequence)

FASTA (aligned sequences)

GenBank (complete sequence)

Continue
Cancel

GenBankGraphics

Sort by:

E value
 Score
Percent identity
Query start position
Subject start position

Next
Previous
Descriptions

Pseudomonas trivialis strain BS3111 genome assembly, chromosome: I

Sequence ID: LT629760.1Length: 5634667Number of Matches: 5

Related Information

Range 1: 453030 to 454074GenBankGraphics

Next Match
Previous Match
First Match

Alignment statistics for match #1

| Score | Expect | Identities | Gaps | Strand | Frame |
| --- | --- | --- | --- | --- | --- |
| 1927 bits(1043) | 0.0() | 1044/1045(99%) | 0/1045(0%) | Plus/Plus |  |

Features:

rRNA-16S ribosomal RNA . Bacterial SSU

```
Query  9       AACCGTCCTCCCGAAGGTTAGACTAGCTACTTCTGGTGCANCCCACTCCCATGGTGTGAC  68
               |||||||||||||||||||||||||||||||||||||||| |||||||||||||||||||
Sbjct  453030  AACCGTCCTCCCGAAGGTTAGACTAGCTACTTCTGGTGCAACCCACTCCCATGGTGTGAC  453089

Query  69      GGGCGGTGTGTACAAGGCCCGGGAACGTATTCACCGCGACATTCTGATTCGCGATTACTA  128
               ||||||||||||||||||||||||||||||||||||||||||||||||||||||||||||
Sbjct  453090  GGGCGGTGTGTACAAGGCCCGGGAACGTATTCACCGCGACATTCTGATTCGCGATTACTA  453149

Query  129     GCGATTCCGACTTCACGCAGTCGAGTTGCAGACTGCGATCCGGACTACGATCGGTTTTGT  188
               ||||||||||||||||||||||||||||||||||||||||||||||||||||||||||||
Sbjct  453150  GCGATTCCGACTTCACGCAGTCGAGTTGCAGACTGCGATCCGGACTACGATCGGTTTTGT  453209

Query  189     GGGATTAGCTCCACCTCGCGGCTTGGCAACCCTCTGTACCGACCATTGTAGCACGTGTGT  248
               ||||||||||||||||||||||||||||||||||||||||||||||||||||||||||||
Sbjct  453210  GGGATTAGCTCCACCTCGCGGCTTGGCAACCCTCTGTACCGACCATTGTAGCACGTGTGT  453269

Query  249     AGCCCAGGCCGTAAGGGCCATGATGACTTGACGTCATCCCCACCTTCCTCCGGTTTGTCA  308
               ||||||||||||||||||||||||||||||||||||||||||||||||||||||||||||
Sbjct  453270  AGCCCAGGCCGTAAGGGCCATGATGACTTGACGTCATCCCCACCTTCCTCCGGTTTGTCA  453329

Query  309     CCGGCAGTCTCCTTAGAGTGCCCACCATTACGTGCTGGTAACTAAGGACAAGGGTTGCGC  368
               ||||||||||||||||||||||||||||||||||||||||||||||||||||||||||||
Sbjct  453330  CCGGCAGTCTCCTTAGAGTGCCCACCATTACGTGCTGGTAACTAAGGACAAGGGTTGCGC  453389

Query  369     TCGTTACGGGACTTAACCCAACATCTCACGACACGAGCTGACGACAGCCATGCAGCACCT  428
               ||||||||||||||||||||||||||||||||||||||||||||||||||||||||||||
Sbjct  453390  TCGTTACGGGACTTAACCCAACATCTCACGACACGAGCTGACGACAGCCATGCAGCACCT  453449

Query  429     GTCTCAATGTTCCCGAAGGCACCAATCTATCTCTAGAAAGTTCATTGGATGTCAAGGCCT  488
               ||||||||||||||||||||||||||||||||||||||||||||||||||||||||||||
Sbjct  453450  GTCTCAATGTTCCCGAAGGCACCAATCTATCTCTAGAAAGTTCATTGGATGTCAAGGCCT  453509

Query  489     GGTAAGGTTCTTCGCGTTGCTTCGAATTAAACCACATGCTCCACCGCTTGTGCGGGCCCC  548
               ||||||||||||||||||||||||||||||||||||||||||||||||||||||||||||
Sbjct  453510  GGTAAGGTTCTTCGCGTTGCTTCGAATTAAACCACATGCTCCACCGCTTGTGCGGGCCCC  453569

Query  549     CGTCAATTCATTTGAGTTTTAACCTTGCGGCCGTACTCCCCAGGCGGTCAACTTAATGCG  608
               ||||||||||||||||||||||||||||||||||||||||||||||||||||||||||||
Sbjct  453570  CGTCAATTCATTTGAGTTTTAACCTTGCGGCCGTACTCCCCAGGCGGTCAACTTAATGCG  453629

Query  609     TTAGCTGCGCCACTAAAAGCTCAAGGCTTCCAACGGCTAGTTGACATCGTTTACGGCGTG  668
               ||||||||||||||||||||||||||||||||||||||||||||||||||||||||||||
Sbjct  453630  TTAGCTGCGCCACTAAAAGCTCAAGGCTTCCAACGGCTAGTTGACATCGTTTACGGCGTG  453689

Query  669     GACTACCAGGGTATCTAATCCTGTTTGCTCCCCACGCTTTCGCACCTCAGTGTCAGTATT  728
               ||||||||||||||||||||||||||||||||||||||||||||||||||||||||||||
Sbjct  453690  GACTACCAGGGTATCTAATCCTGTTTGCTCCCCACGCTTTCGCACCTCAGTGTCAGTATT  453749

Query  729     AGTCCAGGTGGTCGCCTTCGCCACTGGTGTTCCTTCCTATATCTACGCATTTCACCGCTA  788
               ||||||||||||||||||||||||||||||||||||||||||||||||||||||||||||
Sbjct  453750  AGTCCAGGTGGTCGCCTTCGCCACTGGTGTTCCTTCCTATATCTACGCATTTCACCGCTA  453809

Query  789     CACAGGAAATTCCACCACCCTCTACCATACTCTAGTCAGTCAGTTTTGAATGCAGTTCCC  848
               ||||||||||||||||||||||||||||||||||||||||||||||||||||||||||||
Sbjct  453810  CACAGGAAATTCCACCACCCTCTACCATACTCTAGTCAGTCAGTTTTGAATGCAGTTCCC  453869

Query  849     AGGTTGAGCCCGGGGATTTCACATCCAACTTAACAAACCACCTACGCGCGCTTTACGCCC  908
               ||||||||||||||||||||||||||||||||||||||||||||||||||||||||||||
Sbjct  453870  AGGTTGAGCCCGGGGATTTCACATCCAACTTAACAAACCACCTACGCGCGCTTTACGCCC  453929

Query  909     AGTAATTCCGATTAACGCTTGCACCCTCTGTATTACCGCGGCTGCTGGCACAGAGTTAGC  968
               ||||||||||||||||||||||||||||||||||||||||||||||||||||||||||||
Sbjct  453930  AGTAATTCCGATTAACGCTTGCACCCTCTGTATTACCGCGGCTGCTGGCACAGAGTTAGC  453989

Query  969     CGGTGCTTATTCTGTCGGTAACGTCAAAACAATCACGTATTAGGTAACTGCCCTTCCTCC  1028
               ||||||||||||||||||||||||||||||||||||||||||||||||||||||||||||
Sbjct  453990  CGGTGCTTATTCTGTCGGTAACGTCAAAACAATCACGTATTAGGTAACTGCCCTTCCTCC  454049

Query  1029    CAACTTAAAGTGCTTTACAATCCGA  1053
               |||||||||||||||||||||||||
Sbjct  454050  CAACTTAAAGTGCTTTACAATCCGA  454074
```

Range 2: 811681 to 812725GenBankGraphics

Next Match
Previous Match
First Match

Alignment statistics for match #2

| Score | Expect | Identities | Gaps | Strand | Frame |
| --- | --- | --- | --- | --- | --- |
| 1927 bits(1043) | 0.0() | 1044/1045(99%) | 0/1045(0%) | Plus/Plus |  |

Features:

rRNA-16S ribosomal RNA . Bacterial SSU

```
Query  9       AACCGTCCTCCCGAAGGTTAGACTAGCTACTTCTGGTGCANCCCACTCCCATGGTGTGAC  68
               |||||||||||||||||||||||||||||||||||||||| |||||||||||||||||||
Sbjct  811681  AACCGTCCTCCCGAAGGTTAGACTAGCTACTTCTGGTGCAACCCACTCCCATGGTGTGAC  811740

Query  69      GGGCGGTGTGTACAAGGCCCGGGAACGTATTCACCGCGACATTCTGATTCGCGATTACTA  128
               ||||||||||||||||||||||||||||||||||||||||||||||||||||||||||||
Sbjct  811741  GGGCGGTGTGTACAAGGCCCGGGAACGTATTCACCGCGACATTCTGATTCGCGATTACTA  811800

Query  129     GCGATTCCGACTTCACGCAGTCGAGTTGCAGACTGCGATCCGGACTACGATCGGTTTTGT  188
               ||||||||||||||||||||||||||||||||||||||||||||||||||||||||||||
Sbjct  811801  GCGATTCCGACTTCACGCAGTCGAGTTGCAGACTGCGATCCGGACTACGATCGGTTTTGT  811860

Query  189     GGGATTAGCTCCACCTCGCGGCTTGGCAACCCTCTGTACCGACCATTGTAGCACGTGTGT  248
               ||||||||||||||||||||||||||||||||||||||||||||||||||||||||||||
Sbjct  811861  GGGATTAGCTCCACCTCGCGGCTTGGCAACCCTCTGTACCGACCATTGTAGCACGTGTGT  811920

Query  249     AGCCCAGGCCGTAAGGGCCATGATGACTTGACGTCATCCCCACCTTCCTCCGGTTTGTCA  308
               ||||||||||||||||||||||||||||||||||||||||||||||||||||||||||||
Sbjct  811921  AGCCCAGGCCGTAAGGGCCATGATGACTTGACGTCATCCCCACCTTCCTCCGGTTTGTCA  811980

Query  309     CCGGCAGTCTCCTTAGAGTGCCCACCATTACGTGCTGGTAACTAAGGACAAGGGTTGCGC  368
               ||||||||||||||||||||||||||||||||||||||||||||||||||||||||||||
Sbjct  811981  CCGGCAGTCTCCTTAGAGTGCCCACCATTACGTGCTGGTAACTAAGGACAAGGGTTGCGC  812040

Query  369     TCGTTACGGGACTTAACCCAACATCTCACGACACGAGCTGACGACAGCCATGCAGCACCT  428
               ||||||||||||||||||||||||||||||||||||||||||||||||||||||||||||
Sbjct  812041  TCGTTACGGGACTTAACCCAACATCTCACGACACGAGCTGACGACAGCCATGCAGCACCT  812100

Query  429     GTCTCAATGTTCCCGAAGGCACCAATCTATCTCTAGAAAGTTCATTGGATGTCAAGGCCT  488
               ||||||||||||||||||||||||||||||||||||||||||||||||||||||||||||
Sbjct  812101  GTCTCAATGTTCCCGAAGGCACCAATCTATCTCTAGAAAGTTCATTGGATGTCAAGGCCT  812160

Query  489     GGTAAGGTTCTTCGCGTTGCTTCGAATTAAACCACATGCTCCACCGCTTGTGCGGGCCCC  548
               ||||||||||||||||||||||||||||||||||||||||||||||||||||||||||||
Sbjct  812161  GGTAAGGTTCTTCGCGTTGCTTCGAATTAAACCACATGCTCCACCGCTTGTGCGGGCCCC  812220

Query  549     CGTCAATTCATTTGAGTTTTAACCTTGCGGCCGTACTCCCCAGGCGGTCAACTTAATGCG  608
               ||||||||||||||||||||||||||||||||||||||||||||||||||||||||||||
Sbjct  812221  CGTCAATTCATTTGAGTTTTAACCTTGCGGCCGTACTCCCCAGGCGGTCAACTTAATGCG  812280

Query  609     TTAGCTGCGCCACTAAAAGCTCAAGGCTTCCAACGGCTAGTTGACATCGTTTACGGCGTG  668
               ||||||||||||||||||||||||||||||||||||||||||||||||||||||||||||
Sbjct  812281  TTAGCTGCGCCACTAAAAGCTCAAGGCTTCCAACGGCTAGTTGACATCGTTTACGGCGTG  812340

Query  669     GACTACCAGGGTATCTAATCCTGTTTGCTCCCCACGCTTTCGCACCTCAGTGTCAGTATT  728
               ||||||||||||||||||||||||||||||||||||||||||||||||||||||||||||
Sbjct  812341  GACTACCAGGGTATCTAATCCTGTTTGCTCCCCACGCTTTCGCACCTCAGTGTCAGTATT  812400

Query  729     AGTCCAGGTGGTCGCCTTCGCCACTGGTGTTCCTTCCTATATCTACGCATTTCACCGCTA  788
               ||||||||||||||||||||||||||||||||||||||||||||||||||||||||||||
Sbjct  812401  AGTCCAGGTGGTCGCCTTCGCCACTGGTGTTCCTTCCTATATCTACGCATTTCACCGCTA  812460

Query  789     CACAGGAAATTCCACCACCCTCTACCATACTCTAGTCAGTCAGTTTTGAATGCAGTTCCC  848
               ||||||||||||||||||||||||||||||||||||||||||||||||||||||||||||
Sbjct  812461  CACAGGAAATTCCACCACCCTCTACCATACTCTAGTCAGTCAGTTTTGAATGCAGTTCCC  812520

Query  849     AGGTTGAGCCCGGGGATTTCACATCCAACTTAACAAACCACCTACGCGCGCTTTACGCCC  908
               ||||||||||||||||||||||||||||||||||||||||||||||||||||||||||||
Sbjct  812521  AGGTTGAGCCCGGGGATTTCACATCCAACTTAACAAACCACCTACGCGCGCTTTACGCCC  812580

Query  909     AGTAATTCCGATTAACGCTTGCACCCTCTGTATTACCGCGGCTGCTGGCACAGAGTTAGC  968
               ||||||||||||||||||||||||||||||||||||||||||||||||||||||||||||
Sbjct  812581  AGTAATTCCGATTAACGCTTGCACCCTCTGTATTACCGCGGCTGCTGGCACAGAGTTAGC  812640

Query  969     CGGTGCTTATTCTGTCGGTAACGTCAAAACAATCACGTATTAGGTAACTGCCCTTCCTCC  1028
               ||||||||||||||||||||||||||||||||||||||||||||||||||||||||||||
Sbjct  812641  CGGTGCTTATTCTGTCGGTAACGTCAAAACAATCACGTATTAGGTAACTGCCCTTCCTCC  812700

Query  1029    CAACTTAAAGTGCTTTACAATCCGA  1053
               |||||||||||||||||||||||||
Sbjct  812701  CAACTTAAAGTGCTTTACAATCCGA  812725
```

Range 3: 1552806 to 1553850GenBankGraphics

Next Match
Previous Match
First Match

Alignment statistics for match #3

| Score | Expect | Identities | Gaps | Strand | Frame |
| --- | --- | --- | --- | --- | --- |
| 1927 bits(1043) | 0.0() | 1044/1045(99%) | 0/1045(0%) | Plus/Minus |  |

Features:

rRNA-16S ribosomal RNA . Bacterial SSU

```
Query  9        AACCGTCCTCCCGAAGGTTAGACTAGCTACTTCTGGTGCANCCCACTCCCATGGTGTGAC  68
                |||||||||||||||||||||||||||||||||||||||| |||||||||||||||||||
Sbjct  1553850  AACCGTCCTCCCGAAGGTTAGACTAGCTACTTCTGGTGCAACCCACTCCCATGGTGTGAC  1553791

Query  69       GGGCGGTGTGTACAAGGCCCGGGAACGTATTCACCGCGACATTCTGATTCGCGATTACTA  128
                ||||||||||||||||||||||||||||||||||||||||||||||||||||||||||||
Sbjct  1553790  GGGCGGTGTGTACAAGGCCCGGGAACGTATTCACCGCGACATTCTGATTCGCGATTACTA  1553731

Query  129      GCGATTCCGACTTCACGCAGTCGAGTTGCAGACTGCGATCCGGACTACGATCGGTTTTGT  188
                ||||||||||||||||||||||||||||||||||||||||||||||||||||||||||||
Sbjct  1553730  GCGATTCCGACTTCACGCAGTCGAGTTGCAGACTGCGATCCGGACTACGATCGGTTTTGT  1553671

Query  189      GGGATTAGCTCCACCTCGCGGCTTGGCAACCCTCTGTACCGACCATTGTAGCACGTGTGT  248
                ||||||||||||||||||||||||||||||||||||||||||||||||||||||||||||
Sbjct  1553670  GGGATTAGCTCCACCTCGCGGCTTGGCAACCCTCTGTACCGACCATTGTAGCACGTGTGT  1553611

Query  249      AGCCCAGGCCGTAAGGGCCATGATGACTTGACGTCATCCCCACCTTCCTCCGGTTTGTCA  308
                ||||||||||||||||||||||||||||||||||||||||||||||||||||||||||||
Sbjct  1553610  AGCCCAGGCCGTAAGGGCCATGATGACTTGACGTCATCCCCACCTTCCTCCGGTTTGTCA  1553551

Query  309      CCGGCAGTCTCCTTAGAGTGCCCACCATTACGTGCTGGTAACTAAGGACAAGGGTTGCGC  368
                ||||||||||||||||||||||||||||||||||||||||||||||||||||||||||||
Sbjct  1553550  CCGGCAGTCTCCTTAGAGTGCCCACCATTACGTGCTGGTAACTAAGGACAAGGGTTGCGC  1553491

Query  369      TCGTTACGGGACTTAACCCAACATCTCACGACACGAGCTGACGACAGCCATGCAGCACCT  428
                ||||||||||||||||||||||||||||||||||||||||||||||||||||||||||||
Sbjct  1553490  TCGTTACGGGACTTAACCCAACATCTCACGACACGAGCTGACGACAGCCATGCAGCACCT  1553431

Query  429      GTCTCAATGTTCCCGAAGGCACCAATCTATCTCTAGAAAGTTCATTGGATGTCAAGGCCT  488
                ||||||||||||||||||||||||||||||||||||||||||||||||||||||||||||
Sbjct  1553430  GTCTCAATGTTCCCGAAGGCACCAATCTATCTCTAGAAAGTTCATTGGATGTCAAGGCCT  1553371

Query  489      GGTAAGGTTCTTCGCGTTGCTTCGAATTAAACCACATGCTCCACCGCTTGTGCGGGCCCC  548
                ||||||||||||||||||||||||||||||||||||||||||||||||||||||||||||
Sbjct  1553370  GGTAAGGTTCTTCGCGTTGCTTCGAATTAAACCACATGCTCCACCGCTTGTGCGGGCCCC  1553311

Query  549      CGTCAATTCATTTGAGTTTTAACCTTGCGGCCGTACTCCCCAGGCGGTCAACTTAATGCG  608
                ||||||||||||||||||||||||||||||||||||||||||||||||||||||||||||
Sbjct  1553310  CGTCAATTCATTTGAGTTTTAACCTTGCGGCCGTACTCCCCAGGCGGTCAACTTAATGCG  1553251

Query  609      TTAGCTGCGCCACTAAAAGCTCAAGGCTTCCAACGGCTAGTTGACATCGTTTACGGCGTG  668
                ||||||||||||||||||||||||||||||||||||||||||||||||||||||||||||
Sbjct  1553250  TTAGCTGCGCCACTAAAAGCTCAAGGCTTCCAACGGCTAGTTGACATCGTTTACGGCGTG  1553191

Query  669      GACTACCAGGGTATCTAATCCTGTTTGCTCCCCACGCTTTCGCACCTCAGTGTCAGTATT  728
                ||||||||||||||||||||||||||||||||||||||||||||||||||||||||||||
Sbjct  1553190  GACTACCAGGGTATCTAATCCTGTTTGCTCCCCACGCTTTCGCACCTCAGTGTCAGTATT  1553131

Query  729      AGTCCAGGTGGTCGCCTTCGCCACTGGTGTTCCTTCCTATATCTACGCATTTCACCGCTA  788
                ||||||||||||||||||||||||||||||||||||||||||||||||||||||||||||
Sbjct  1553130  AGTCCAGGTGGTCGCCTTCGCCACTGGTGTTCCTTCCTATATCTACGCATTTCACCGCTA  1553071

Query  789      CACAGGAAATTCCACCACCCTCTACCATACTCTAGTCAGTCAGTTTTGAATGCAGTTCCC  848
                ||||||||||||||||||||||||||||||||||||||||||||||||||||||||||||
Sbjct  1553070  CACAGGAAATTCCACCACCCTCTACCATACTCTAGTCAGTCAGTTTTGAATGCAGTTCCC  1553011

Query  849      AGGTTGAGCCCGGGGATTTCACATCCAACTTAACAAACCACCTACGCGCGCTTTACGCCC  908
                ||||||||||||||||||||||||||||||||||||||||||||||||||||||||||||
Sbjct  1553010  AGGTTGAGCCCGGGGATTTCACATCCAACTTAACAAACCACCTACGCGCGCTTTACGCCC  1552951

Query  909      AGTAATTCCGATTAACGCTTGCACCCTCTGTATTACCGCGGCTGCTGGCACAGAGTTAGC  968
                ||||||||||||||||||||||||||||||||||||||||||||||||||||||||||||
Sbjct  1552950  AGTAATTCCGATTAACGCTTGCACCCTCTGTATTACCGCGGCTGCTGGCACAGAGTTAGC  1552891

Query  969      CGGTGCTTATTCTGTCGGTAACGTCAAAACAATCACGTATTAGGTAACTGCCCTTCCTCC  1028
                ||||||||||||||||||||||||||||||||||||||||||||||||||||||||||||
Sbjct  1552890  CGGTGCTTATTCTGTCGGTAACGTCAAAACAATCACGTATTAGGTAACTGCCCTTCCTCC  1552831

Query  1029     CAACTTAAAGTGCTTTACAATCCGA  1053
                |||||||||||||||||||||||||
Sbjct  1552830  CAACTTAAAGTGCTTTACAATCCGA  1552806
```

Range 4: 3431401 to 3432445GenBankGraphics

Next Match
Previous Match
First Match

Alignment statistics for match #4

| Score | Expect | Identities | Gaps | Strand | Frame |
| --- | --- | --- | --- | --- | --- |
| 1927 bits(1043) | 0.0() | 1044/1045(99%) | 0/1045(0%) | Plus/Minus |  |

Features:

rRNA-16S ribosomal RNA . Bacterial SSU

```
Query  9        AACCGTCCTCCCGAAGGTTAGACTAGCTACTTCTGGTGCANCCCACTCCCATGGTGTGAC  68
                |||||||||||||||||||||||||||||||||||||||| |||||||||||||||||||
Sbjct  3432445  AACCGTCCTCCCGAAGGTTAGACTAGCTACTTCTGGTGCAACCCACTCCCATGGTGTGAC  3432386

Query  69       GGGCGGTGTGTACAAGGCCCGGGAACGTATTCACCGCGACATTCTGATTCGCGATTACTA  128
                ||||||||||||||||||||||||||||||||||||||||||||||||||||||||||||
Sbjct  3432385  GGGCGGTGTGTACAAGGCCCGGGAACGTATTCACCGCGACATTCTGATTCGCGATTACTA  3432326

Query  129      GCGATTCCGACTTCACGCAGTCGAGTTGCAGACTGCGATCCGGACTACGATCGGTTTTGT  188
                ||||||||||||||||||||||||||||||||||||||||||||||||||||||||||||
Sbjct  3432325  GCGATTCCGACTTCACGCAGTCGAGTTGCAGACTGCGATCCGGACTACGATCGGTTTTGT  3432266

Query  189      GGGATTAGCTCCACCTCGCGGCTTGGCAACCCTCTGTACCGACCATTGTAGCACGTGTGT  248
                ||||||||||||||||||||||||||||||||||||||||||||||||||||||||||||
Sbjct  3432265  GGGATTAGCTCCACCTCGCGGCTTGGCAACCCTCTGTACCGACCATTGTAGCACGTGTGT  3432206

Query  249      AGCCCAGGCCGTAAGGGCCATGATGACTTGACGTCATCCCCACCTTCCTCCGGTTTGTCA  308
                ||||||||||||||||||||||||||||||||||||||||||||||||||||||||||||
Sbjct  3432205  AGCCCAGGCCGTAAGGGCCATGATGACTTGACGTCATCCCCACCTTCCTCCGGTTTGTCA  3432146

Query  309      CCGGCAGTCTCCTTAGAGTGCCCACCATTACGTGCTGGTAACTAAGGACAAGGGTTGCGC  368
                ||||||||||||||||||||||||||||||||||||||||||||||||||||||||||||
Sbjct  3432145  CCGGCAGTCTCCTTAGAGTGCCCACCATTACGTGCTGGTAACTAAGGACAAGGGTTGCGC  3432086

Query  369      TCGTTACGGGACTTAACCCAACATCTCACGACACGAGCTGACGACAGCCATGCAGCACCT  428
                ||||||||||||||||||||||||||||||||||||||||||||||||||||||||||||
Sbjct  3432085  TCGTTACGGGACTTAACCCAACATCTCACGACACGAGCTGACGACAGCCATGCAGCACCT  3432026

Query  429      GTCTCAATGTTCCCGAAGGCACCAATCTATCTCTAGAAAGTTCATTGGATGTCAAGGCCT  488
                ||||||||||||||||||||||||||||||||||||||||||||||||||||||||||||
Sbjct  3432025  GTCTCAATGTTCCCGAAGGCACCAATCTATCTCTAGAAAGTTCATTGGATGTCAAGGCCT  3431966

Query  489      GGTAAGGTTCTTCGCGTTGCTTCGAATTAAACCACATGCTCCACCGCTTGTGCGGGCCCC  548
                ||||||||||||||||||||||||||||||||||||||||||||||||||||||||||||
Sbjct  3431965  GGTAAGGTTCTTCGCGTTGCTTCGAATTAAACCACATGCTCCACCGCTTGTGCGGGCCCC  3431906

Query  549      CGTCAATTCATTTGAGTTTTAACCTTGCGGCCGTACTCCCCAGGCGGTCAACTTAATGCG  608
                ||||||||||||||||||||||||||||||||||||||||||||||||||||||||||||
Sbjct  3431905  CGTCAATTCATTTGAGTTTTAACCTTGCGGCCGTACTCCCCAGGCGGTCAACTTAATGCG  3431846

Query  609      TTAGCTGCGCCACTAAAAGCTCAAGGCTTCCAACGGCTAGTTGACATCGTTTACGGCGTG  668
                ||||||||||||||||||||||||||||||||||||||||||||||||||||||||||||
Sbjct  3431845  TTAGCTGCGCCACTAAAAGCTCAAGGCTTCCAACGGCTAGTTGACATCGTTTACGGCGTG  3431786

Query  669      GACTACCAGGGTATCTAATCCTGTTTGCTCCCCACGCTTTCGCACCTCAGTGTCAGTATT  728
                ||||||||||||||||||||||||||||||||||||||||||||||||||||||||||||
Sbjct  3431785  GACTACCAGGGTATCTAATCCTGTTTGCTCCCCACGCTTTCGCACCTCAGTGTCAGTATT  3431726

Query  729      AGTCCAGGTGGTCGCCTTCGCCACTGGTGTTCCTTCCTATATCTACGCATTTCACCGCTA  788
                ||||||||||||||||||||||||||||||||||||||||||||||||||||||||||||
Sbjct  3431725  AGTCCAGGTGGTCGCCTTCGCCACTGGTGTTCCTTCCTATATCTACGCATTTCACCGCTA  3431666

Query  789      CACAGGAAATTCCACCACCCTCTACCATACTCTAGTCAGTCAGTTTTGAATGCAGTTCCC  848
                ||||||||||||||||||||||||||||||||||||||||||||||||||||||||||||
Sbjct  3431665  CACAGGAAATTCCACCACCCTCTACCATACTCTAGTCAGTCAGTTTTGAATGCAGTTCCC  3431606

Query  849      AGGTTGAGCCCGGGGATTTCACATCCAACTTAACAAACCACCTACGCGCGCTTTACGCCC  908
                ||||||||||||||||||||||||||||||||||||||||||||||||||||||||||||
Sbjct  3431605  AGGTTGAGCCCGGGGATTTCACATCCAACTTAACAAACCACCTACGCGCGCTTTACGCCC  3431546

Query  909      AGTAATTCCGATTAACGCTTGCACCCTCTGTATTACCGCGGCTGCTGGCACAGAGTTAGC  968
                ||||||||||||||||||||||||||||||||||||||||||||||||||||||||||||
Sbjct  3431545  AGTAATTCCGATTAACGCTTGCACCCTCTGTATTACCGCGGCTGCTGGCACAGAGTTAGC  3431486

Query  969      CGGTGCTTATTCTGTCGGTAACGTCAAAACAATCACGTATTAGGTAACTGCCCTTCCTCC  1028
                ||||||||||||||||||||||||||||||||||||||||||||||||||||||||||||
Sbjct  3431485  CGGTGCTTATTCTGTCGGTAACGTCAAAACAATCACGTATTAGGTAACTGCCCTTCCTCC  3431426

Query  1029     CAACTTAAAGTGCTTTACAATCCGA  1053
                |||||||||||||||||||||||||
Sbjct  3431425  CAACTTAAAGTGCTTTACAATCCGA  3431401
```

Range 5: 2159040 to 2160084GenBankGraphics

Next Match
Previous Match
First Match

Alignment statistics for match #5

| Score | Expect | Identities | Gaps | Strand | Frame |
| --- | --- | --- | --- | --- | --- |
| 1921 bits(1040) | 0.0() | 1043/1045(99%) | 0/1045(0%) | Plus/Minus |  |

Features:

rRNA-16S ribosomal RNA . Bacterial SSU

```
Query  9        AACCGTCCTCCCGAAGGTTAGACTAGCTACTTCTGGTGCANCCCACTCCCATGGTGTGAC  68
                |||||||||||||||||||||||||||||||||||||||| |||||||||||||||||||
Sbjct  2160084  AACCGTCCTCCCGAAGGTTAGACTAGCTACTTCTGGTGCAACCCACTCCCATGGTGTGAC  2160025

Query  69       GGGCGGTGTGTACAAGGCCCGGGAACGTATTCACCGCGACATTCTGATTCGCGATTACTA  128
                ||||||||||||||||||||||||||||||||||||||||||||||||||||||||||||
Sbjct  2160024  GGGCGGTGTGTACAAGGCCCGGGAACGTATTCACCGCGACATTCTGATTCGCGATTACTA  2159965

Query  129      GCGATTCCGACTTCACGCAGTCGAGTTGCAGACTGCGATCCGGACTACGATCGGTTTTGT  188
                ||||||||||||||||||||||||||||||||||||||||||||||||||||||||||||
Sbjct  2159964  GCGATTCCGACTTCACGCAGTCGAGTTGCAGACTGCGATCCGGACTACGATCGGTTTTGT  2159905

Query  189      GGGATTAGCTCCACCTCGCGGCTTGGCAACCCTCTGTACCGACCATTGTAGCACGTGTGT  248
                ||||||||||||||||||||||||||||||||||||||||||||||||||||||||||||
Sbjct  2159904  GGGATTAGCTCCACCTCGCGGCTTGGCAACCCTCTGTACCGACCATTGTAGCACGTGTGT  2159845

Query  249      AGCCCAGGCCGTAAGGGCCATGATGACTTGACGTCATCCCCACCTTCCTCCGGTTTGTCA  308
                ||||||||||||||||||||||||||||||||||||||||||||||||||||||||||||
Sbjct  2159844  AGCCCAGGCCGTAAGGGCCATGATGACTTGACGTCATCCCCACCTTCCTCCGGTTTGTCA  2159785

Query  309      CCGGCAGTCTCCTTAGAGTGCCCACCATTACGTGCTGGTAACTAAGGACAAGGGTTGCGC  368
                ||||||||||||||||||||||||||||||||||||||||||||||||||||||||||||
Sbjct  2159784  CCGGCAGTCTCCTTAGAGTGCCCACCATTACGTGCTGGTAACTAAGGACAAGGGTTGCGC  2159725

Query  369      TCGTTACGGGACTTAACCCAACATCTCACGACACGAGCTGACGACAGCCATGCAGCACCT  428
                ||||||||||||||||||||||||||||||||||||||||||||||||||||||||||||
Sbjct  2159724  TCGTTACGGGACTTAACCCAACATCTCACGACACGAGCTGACGACAGCCATGCAGCACCT  2159665

Query  429      GTCTCAATGTTCCCGAAGGCACCAATCTATCTCTAGAAAGTTCATTGGATGTCAAGGCCT  488
                ||||||||||||||||||||||||||||||||||||||||||||||||||||||||||||
Sbjct  2159664  GTCTCAATGTTCCCGAAGGCACCAATCTATCTCTAGAAAGTTCATTGGATGTCAAGGCCT  2159605

Query  489      GGTAAGGTTCTTCGCGTTGCTTCGAATTAAACCACATGCTCCACCGCTTGTGCGGGCCCC  548
                ||||||||||||||||||||||||||||||||||||||||||||||||||||||||||||
Sbjct  2159604  GGTAAGGTTCTTCGCGTTGCTTCGAATTAAACCACATGCTCCACCGCTTGTGCGGGCCCC  2159545

Query  549      CGTCAATTCATTTGAGTTTTAACCTTGCGGCCGTACTCCCCAGGCGGTCAACTTAATGCG  608
                ||||||||||||||||||||||||||||||||||||||||||||||||||||||||||||
Sbjct  2159544  CGTCAATTCATTTGAGTTTTAACCTTGCGGCCGTACTCCCCAGGCGGTCAACTTAATGCG  2159485

Query  609      TTAGCTGCGCCACTAAAAGCTCAAGGCTTCCAACGGCTAGTTGACATCGTTTACGGCGTG  668
                ||||||||||||||||||||||||||||||||||||||||||||||||||||||||||||
Sbjct  2159484  TTAGCTGCGCCACTAAAAGCTCAAGGCTTCCAACGGCTAGTTGACATCGTTTACGGCGTG  2159425

Query  669      GACTACCAGGGTATCTAATCCTGTTTGCTCCCCACGCTTTCGCACCTCAGTGTCAGTATT  728
                ||||||||||||||||||||||||||||||||||||||||||||||||||||||||||||
Sbjct  2159424  GACTACCAGGGTATCTAATCCTGTTTGCTCCCCACGCTTTCGCACCTCAGTGTCAGTATT  2159365

Query  729      AGTCCAGGTGGTCGCCTTCGCCACTGGTGTTCCTTCCTATATCTACGCATTTCACCGCTA  788
                ||||||||||||||||||||||||||||||||||||||||||||||||||||||||||||
Sbjct  2159364  AGTCCAGGTGGTCGCCTTCGCCACTGGTGTTCCTTCCTATATCTACGCATTTCACCGCTA  2159305

Query  789      CACAGGAAATTCCACCACCCTCTACCATACTCTAGTCAGTCAGTTTTGAATGCAGTTCCC  848
                ||||||||||||||||||||||||||||||||||||||||||||||||||||||||||||
Sbjct  2159304  CACAGGAAATTCCACCACCCTCTACCATACTCTAGTCAGTCAGTTTTGAATGCAGTTCCC  2159245

Query  849      AGGTTGAGCCCGGGGATTTCACATCCAACTTAACAAACCACCTACGCGCGCTTTACGCCC  908
                ||||||||||||||||||||||||||||||||||||||||||||||||||||||||||||
Sbjct  2159244  AGGTTGAGCCCGGGGATTTCACATCCAACTTAACAAACCACCTACGCGCGCTTTACGCCC  2159185

Query  909      AGTAATTCCGATTAACGCTTGCACCCTCTGTATTACCGCGGCTGCTGGCACAGAGTTAGC  968
                ||||||||||||||||||||||||||||||||||||||||||||||||||||||||||||
Sbjct  2159184  AGTAATTCCGATTAACGCTTGCACCCTCTGTATTACCGCGGCTGCTGGCACAGAGTTAGC  2159125

Query  969      CGGTGCTTATTCTGTCGGTAACGTCAAAACAATCACGTATTAGGTAACTGCCCTTCCTCC  1028
                |||||||||||||||||||||||||||||||||||||||||| |||||||||||||||||
Sbjct  2159124  CGGTGCTTATTCTGTCGGTAACGTCAAAACAATCACGTATTAAGTAACTGCCCTTCCTCC  2159065

Query  1029     CAACTTAAAGTGCTTTACAATCCGA  1053
                |||||||||||||||||||||||||
Sbjct  2159064  CAACTTAAAGTGCTTTACAATCCGA  2159040
```

Download

FASTA (complete sequence)

FASTA (aligned sequences)

GenBank (complete sequence)

Continue
Cancel

GenBankGraphics

Next
Previous
Descriptions

Uncultured bacterium clone 19-26 16S ribosomal RNA gene, partial sequence

Sequence ID: KT029390.1Length: 1459Number of Matches: 1

Related Information

Range 1: 388 to 1432GenBankGraphics

Next Match
Previous Match
First Match

Alignment statistics for match #1

| Score | Expect | Identities | Gaps | Strand | Frame |
| --- | --- | --- | --- | --- | --- |
| 1927 bits(1043) | 0.0() | 1044/1045(99%) | 0/1045(0%) | Plus/Minus |  |

Features:

```
Query  9     AACCGTCCTCCCGAAGGTTAGACTAGCTACTTCTGGTGCANCCCACTCCCATGGTGTGAC  68
             |||||||||||||||||||||||||||||||||||||||| |||||||||||||||||||
Sbjct  1432  AACCGTCCTCCCGAAGGTTAGACTAGCTACTTCTGGTGCAACCCACTCCCATGGTGTGAC  1373

Query  69    GGGCGGTGTGTACAAGGCCCGGGAACGTATTCACCGCGACATTCTGATTCGCGATTACTA  128
             ||||||||||||||||||||||||||||||||||||||||||||||||||||||||||||
Sbjct  1372  GGGCGGTGTGTACAAGGCCCGGGAACGTATTCACCGCGACATTCTGATTCGCGATTACTA  1313

Query  129   GCGATTCCGACTTCACGCAGTCGAGTTGCAGACTGCGATCCGGACTACGATCGGTTTTGT  188
             ||||||||||||||||||||||||||||||||||||||||||||||||||||||||||||
Sbjct  1312  GCGATTCCGACTTCACGCAGTCGAGTTGCAGACTGCGATCCGGACTACGATCGGTTTTGT  1253

Query  189   GGGATTAGCTCCACCTCGCGGCTTGGCAACCCTCTGTACCGACCATTGTAGCACGTGTGT  248
             ||||||||||||||||||||||||||||||||||||||||||||||||||||||||||||
Sbjct  1252  GGGATTAGCTCCACCTCGCGGCTTGGCAACCCTCTGTACCGACCATTGTAGCACGTGTGT  1193

Query  249   AGCCCAGGCCGTAAGGGCCATGATGACTTGACGTCATCCCCACCTTCCTCCGGTTTGTCA  308
             ||||||||||||||||||||||||||||||||||||||||||||||||||||||||||||
Sbjct  1192  AGCCCAGGCCGTAAGGGCCATGATGACTTGACGTCATCCCCACCTTCCTCCGGTTTGTCA  1133

Query  309   CCGGCAGTCTCCTTAGAGTGCCCACCATTACGTGCTGGTAACTAAGGACAAGGGTTGCGC  368
             ||||||||||||||||||||||||||||||||||||||||||||||||||||||||||||
Sbjct  1132  CCGGCAGTCTCCTTAGAGTGCCCACCATTACGTGCTGGTAACTAAGGACAAGGGTTGCGC  1073

Query  369   TCGTTACGGGACTTAACCCAACATCTCACGACACGAGCTGACGACAGCCATGCAGCACCT  428
             ||||||||||||||||||||||||||||||||||||||||||||||||||||||||||||
Sbjct  1072  TCGTTACGGGACTTAACCCAACATCTCACGACACGAGCTGACGACAGCCATGCAGCACCT  1013

Query  429   GTCTCAATGTTCCCGAAGGCACCAATCTATCTCTAGAAAGTTCATTGGATGTCAAGGCCT  488
             ||||||||||||||||||||||||||||||||||||||||||||||||||||||||||||
Sbjct  1012  GTCTCAATGTTCCCGAAGGCACCAATCTATCTCTAGAAAGTTCATTGGATGTCAAGGCCT  953

Query  489   GGTAAGGTTCTTCGCGTTGCTTCGAATTAAACCACATGCTCCACCGCTTGTGCGGGCCCC  548
             ||||||||||||||||||||||||||||||||||||||||||||||||||||||||||||
Sbjct  952   GGTAAGGTTCTTCGCGTTGCTTCGAATTAAACCACATGCTCCACCGCTTGTGCGGGCCCC  893

Query  549   CGTCAATTCATTTGAGTTTTAACCTTGCGGCCGTACTCCCCAGGCGGTCAACTTAATGCG  608
             ||||||||||||||||||||||||||||||||||||||||||||||||||||||||||||
Sbjct  892   CGTCAATTCATTTGAGTTTTAACCTTGCGGCCGTACTCCCCAGGCGGTCAACTTAATGCG  833

Query  609   TTAGCTGCGCCACTAAAAGCTCAAGGCTTCCAACGGCTAGTTGACATCGTTTACGGCGTG  668
             ||||||||||||||||||||||||||||||||||||||||||||||||||||||||||||
Sbjct  832   TTAGCTGCGCCACTAAAAGCTCAAGGCTTCCAACGGCTAGTTGACATCGTTTACGGCGTG  773

Query  669   GACTACCAGGGTATCTAATCCTGTTTGCTCCCCACGCTTTCGCACCTCAGTGTCAGTATT  728
             ||||||||||||||||||||||||||||||||||||||||||||||||||||||||||||
Sbjct  772   GACTACCAGGGTATCTAATCCTGTTTGCTCCCCACGCTTTCGCACCTCAGTGTCAGTATT  713

Query  729   AGTCCAGGTGGTCGCCTTCGCCACTGGTGTTCCTTCCTATATCTACGCATTTCACCGCTA  788
             ||||||||||||||||||||||||||||||||||||||||||||||||||||||||||||
Sbjct  712   AGTCCAGGTGGTCGCCTTCGCCACTGGTGTTCCTTCCTATATCTACGCATTTCACCGCTA  653

Query  789   CACAGGAAATTCCACCACCCTCTACCATACTCTAGTCAGTCAGTTTTGAATGCAGTTCCC  848
             ||||||||||||||||||||||||||||||||||||||||||||||||||||||||||||
Sbjct  652   CACAGGAAATTCCACCACCCTCTACCATACTCTAGTCAGTCAGTTTTGAATGCAGTTCCC  593

Query  849   AGGTTGAGCCCGGGGATTTCACATCCAACTTAACAAACCACCTACGCGCGCTTTACGCCC  908
             ||||||||||||||||||||||||||||||||||||||||||||||||||||||||||||
Sbjct  592   AGGTTGAGCCCGGGGATTTCACATCCAACTTAACAAACCACCTACGCGCGCTTTACGCCC  533

Query  909   AGTAATTCCGATTAACGCTTGCACCCTCTGTATTACCGCGGCTGCTGGCACAGAGTTAGC  968
             ||||||||||||||||||||||||||||||||||||||||||||||||||||||||||||
Sbjct  532   AGTAATTCCGATTAACGCTTGCACCCTCTGTATTACCGCGGCTGCTGGCACAGAGTTAGC  473

Query  969   CGGTGCTTATTCTGTCGGTAACGTCAAAACAATCACGTATTAGGTAACTGCCCTTCCTCC  1028
             ||||||||||||||||||||||||||||||||||||||||||||||||||||||||||||
Sbjct  472   CGGTGCTTATTCTGTCGGTAACGTCAAAACAATCACGTATTAGGTAACTGCCCTTCCTCC  413

Query  1029  CAACTTAAAGTGCTTTACAATCCGA  1053
             |||||||||||||||||||||||||
Sbjct  412   CAACTTAAAGTGCTTTACAATCCGA  388
```

Download

FASTA (complete sequence)

FASTA (aligned sequences)

GenBank (complete sequence)

Continue
Cancel

GenBankGraphics

Next
Previous
Descriptions

Uncultured bacterium clone 19-14 16S ribosomal RNA gene, partial sequence

Sequence ID: KT029378.1Length: 1459Number of Matches: 1

Related Information

Range 1: 388 to 1432GenBankGraphics

Next Match
Previous Match
First Match

Alignment statistics for match #1

| Score | Expect | Identities | Gaps | Strand | Frame |
| --- | --- | --- | --- | --- | --- |
| 1927 bits(1043) | 0.0() | 1044/1045(99%) | 0/1045(0%) | Plus/Minus |  |

Features:

```
Query  9     AACCGTCCTCCCGAAGGTTAGACTAGCTACTTCTGGTGCANCCCACTCCCATGGTGTGAC  68
             |||||||||||||||||||||||||||||||||||||||| |||||||||||||||||||
Sbjct  1432  AACCGTCCTCCCGAAGGTTAGACTAGCTACTTCTGGTGCAACCCACTCCCATGGTGTGAC  1373

Query  69    GGGCGGTGTGTACAAGGCCCGGGAACGTATTCACCGCGACATTCTGATTCGCGATTACTA  128
             ||||||||||||||||||||||||||||||||||||||||||||||||||||||||||||
Sbjct  1372  GGGCGGTGTGTACAAGGCCCGGGAACGTATTCACCGCGACATTCTGATTCGCGATTACTA  1313

Query  129   GCGATTCCGACTTCACGCAGTCGAGTTGCAGACTGCGATCCGGACTACGATCGGTTTTGT  188
             ||||||||||||||||||||||||||||||||||||||||||||||||||||||||||||
Sbjct  1312  GCGATTCCGACTTCACGCAGTCGAGTTGCAGACTGCGATCCGGACTACGATCGGTTTTGT  1253

Query  189   GGGATTAGCTCCACCTCGCGGCTTGGCAACCCTCTGTACCGACCATTGTAGCACGTGTGT  248
             ||||||||||||||||||||||||||||||||||||||||||||||||||||||||||||
Sbjct  1252  GGGATTAGCTCCACCTCGCGGCTTGGCAACCCTCTGTACCGACCATTGTAGCACGTGTGT  1193

Query  249   AGCCCAGGCCGTAAGGGCCATGATGACTTGACGTCATCCCCACCTTCCTCCGGTTTGTCA  308
             ||||||||||||||||||||||||||||||||||||||||||||||||||||||||||||
Sbjct  1192  AGCCCAGGCCGTAAGGGCCATGATGACTTGACGTCATCCCCACCTTCCTCCGGTTTGTCA  1133

Query  309   CCGGCAGTCTCCTTAGAGTGCCCACCATTACGTGCTGGTAACTAAGGACAAGGGTTGCGC  368
             ||||||||||||||||||||||||||||||||||||||||||||||||||||||||||||
Sbjct  1132  CCGGCAGTCTCCTTAGAGTGCCCACCATTACGTGCTGGTAACTAAGGACAAGGGTTGCGC  1073

Query  369   TCGTTACGGGACTTAACCCAACATCTCACGACACGAGCTGACGACAGCCATGCAGCACCT  428
             ||||||||||||||||||||||||||||||||||||||||||||||||||||||||||||
Sbjct  1072  TCGTTACGGGACTTAACCCAACATCTCACGACACGAGCTGACGACAGCCATGCAGCACCT  1013

Query  429   GTCTCAATGTTCCCGAAGGCACCAATCTATCTCTAGAAAGTTCATTGGATGTCAAGGCCT  488
             ||||||||||||||||||||||||||||||||||||||||||||||||||||||||||||
Sbjct  1012  GTCTCAATGTTCCCGAAGGCACCAATCTATCTCTAGAAAGTTCATTGGATGTCAAGGCCT  953

Query  489   GGTAAGGTTCTTCGCGTTGCTTCGAATTAAACCACATGCTCCACCGCTTGTGCGGGCCCC  548
             ||||||||||||||||||||||||||||||||||||||||||||||||||||||||||||
Sbjct  952   GGTAAGGTTCTTCGCGTTGCTTCGAATTAAACCACATGCTCCACCGCTTGTGCGGGCCCC  893

Query  549   CGTCAATTCATTTGAGTTTTAACCTTGCGGCCGTACTCCCCAGGCGGTCAACTTAATGCG  608
             ||||||||||||||||||||||||||||||||||||||||||||||||||||||||||||
Sbjct  892   CGTCAATTCATTTGAGTTTTAACCTTGCGGCCGTACTCCCCAGGCGGTCAACTTAATGCG  833

Query  609   TTAGCTGCGCCACTAAAAGCTCAAGGCTTCCAACGGCTAGTTGACATCGTTTACGGCGTG  668
             ||||||||||||||||||||||||||||||||||||||||||||||||||||||||||||
Sbjct  832   TTAGCTGCGCCACTAAAAGCTCAAGGCTTCCAACGGCTAGTTGACATCGTTTACGGCGTG  773

Query  669   GACTACCAGGGTATCTAATCCTGTTTGCTCCCCACGCTTTCGCACCTCAGTGTCAGTATT  728
             ||||||||||||||||||||||||||||||||||||||||||||||||||||||||||||
Sbjct  772   GACTACCAGGGTATCTAATCCTGTTTGCTCCCCACGCTTTCGCACCTCAGTGTCAGTATT  713

Query  729   AGTCCAGGTGGTCGCCTTCGCCACTGGTGTTCCTTCCTATATCTACGCATTTCACCGCTA  788
             ||||||||||||||||||||||||||||||||||||||||||||||||||||||||||||
Sbjct  712   AGTCCAGGTGGTCGCCTTCGCCACTGGTGTTCCTTCCTATATCTACGCATTTCACCGCTA  653

Query  789   CACAGGAAATTCCACCACCCTCTACCATACTCTAGTCAGTCAGTTTTGAATGCAGTTCCC  848
             ||||||||||||||||||||||||||||||||||||||||||||||||||||||||||||
Sbjct  652   CACAGGAAATTCCACCACCCTCTACCATACTCTAGTCAGTCAGTTTTGAATGCAGTTCCC  593

Query  849   AGGTTGAGCCCGGGGATTTCACATCCAACTTAACAAACCACCTACGCGCGCTTTACGCCC  908
             ||||||||||||||||||||||||||||||||||||||||||||||||||||||||||||
Sbjct  592   AGGTTGAGCCCGGGGATTTCACATCCAACTTAACAAACCACCTACGCGCGCTTTACGCCC  533

Query  909   AGTAATTCCGATTAACGCTTGCACCCTCTGTATTACCGCGGCTGCTGGCACAGAGTTAGC  968
             ||||||||||||||||||||||||||||||||||||||||||||||||||||||||||||
Sbjct  532   AGTAATTCCGATTAACGCTTGCACCCTCTGTATTACCGCGGCTGCTGGCACAGAGTTAGC  473

Query  969   CGGTGCTTATTCTGTCGGTAACGTCAAAACAATCACGTATTAGGTAACTGCCCTTCCTCC  1028
             ||||||||||||||||||||||||||||||||||||||||||||||||||||||||||||
Sbjct  472   CGGTGCTTATTCTGTCGGTAACGTCAAAACAATCACGTATTAGGTAACTGCCCTTCCTCC  413

Query  1029  CAACTTAAAGTGCTTTACAATCCGA  1053
             |||||||||||||||||||||||||
Sbjct  412   CAACTTAAAGTGCTTTACAATCCGA  388
```

Download

FASTA (complete sequence)

FASTA (aligned sequences)

GenBank (complete sequence)

Continue
Cancel

GenBankGraphics

Next
Previous
Descriptions

Pseudomonas trivialis strain P 513/19 16S ribosomal RNA gene, partial sequence

Sequence ID: NR\_028987.1Length: 1492Number of Matches: 1

Related Information

Range 1: 388 to 1432GenBankGraphics

Next Match
Previous Match
First Match

Alignment statistics for match #1

| Score | Expect | Identities | Gaps | Strand | Frame |
| --- | --- | --- | --- | --- | --- |
| 1927 bits(1043) | 0.0() | 1044/1045(99%) | 0/1045(0%) | Plus/Minus |  |

Features:

```
Query  9     AACCGTCCTCCCGAAGGTTAGACTAGCTACTTCTGGTGCANCCCACTCCCATGGTGTGAC  68
             |||||||||||||||||||||||||||||||||||||||| |||||||||||||||||||
Sbjct  1432  AACCGTCCTCCCGAAGGTTAGACTAGCTACTTCTGGTGCAACCCACTCCCATGGTGTGAC  1373

Query  69    GGGCGGTGTGTACAAGGCCCGGGAACGTATTCACCGCGACATTCTGATTCGCGATTACTA  128
             ||||||||||||||||||||||||||||||||||||||||||||||||||||||||||||
Sbjct  1372  GGGCGGTGTGTACAAGGCCCGGGAACGTATTCACCGCGACATTCTGATTCGCGATTACTA  1313

Query  129   GCGATTCCGACTTCACGCAGTCGAGTTGCAGACTGCGATCCGGACTACGATCGGTTTTGT  188
             ||||||||||||||||||||||||||||||||||||||||||||||||||||||||||||
Sbjct  1312  GCGATTCCGACTTCACGCAGTCGAGTTGCAGACTGCGATCCGGACTACGATCGGTTTTGT  1253

Query  189   GGGATTAGCTCCACCTCGCGGCTTGGCAACCCTCTGTACCGACCATTGTAGCACGTGTGT  248
             ||||||||||||||||||||||||||||||||||||||||||||||||||||||||||||
Sbjct  1252  GGGATTAGCTCCACCTCGCGGCTTGGCAACCCTCTGTACCGACCATTGTAGCACGTGTGT  1193

Query  249   AGCCCAGGCCGTAAGGGCCATGATGACTTGACGTCATCCCCACCTTCCTCCGGTTTGTCA  308
             ||||||||||||||||||||||||||||||||||||||||||||||||||||||||||||
Sbjct  1192  AGCCCAGGCCGTAAGGGCCATGATGACTTGACGTCATCCCCACCTTCCTCCGGTTTGTCA  1133

Query  309   CCGGCAGTCTCCTTAGAGTGCCCACCATTACGTGCTGGTAACTAAGGACAAGGGTTGCGC  368
             ||||||||||||||||||||||||||||||||||||||||||||||||||||||||||||
Sbjct  1132  CCGGCAGTCTCCTTAGAGTGCCCACCATTACGTGCTGGTAACTAAGGACAAGGGTTGCGC  1073

Query  369   TCGTTACGGGACTTAACCCAACATCTCACGACACGAGCTGACGACAGCCATGCAGCACCT  428
             ||||||||||||||||||||||||||||||||||||||||||||||||||||||||||||
Sbjct  1072  TCGTTACGGGACTTAACCCAACATCTCACGACACGAGCTGACGACAGCCATGCAGCACCT  1013

Query  429   GTCTCAATGTTCCCGAAGGCACCAATCTATCTCTAGAAAGTTCATTGGATGTCAAGGCCT  488
             ||||||||||||||||||||||||||||||||||||||||||||||||||||||||||||
Sbjct  1012  GTCTCAATGTTCCCGAAGGCACCAATCTATCTCTAGAAAGTTCATTGGATGTCAAGGCCT  953

Query  489   GGTAAGGTTCTTCGCGTTGCTTCGAATTAAACCACATGCTCCACCGCTTGTGCGGGCCCC  548
             ||||||||||||||||||||||||||||||||||||||||||||||||||||||||||||
Sbjct  952   GGTAAGGTTCTTCGCGTTGCTTCGAATTAAACCACATGCTCCACCGCTTGTGCGGGCCCC  893

Query  549   CGTCAATTCATTTGAGTTTTAACCTTGCGGCCGTACTCCCCAGGCGGTCAACTTAATGCG  608
             ||||||||||||||||||||||||||||||||||||||||||||||||||||||||||||
Sbjct  892   CGTCAATTCATTTGAGTTTTAACCTTGCGGCCGTACTCCCCAGGCGGTCAACTTAATGCG  833

Query  609   TTAGCTGCGCCACTAAAAGCTCAAGGCTTCCAACGGCTAGTTGACATCGTTTACGGCGTG  668
             ||||||||||||||||||||||||||||||||||||||||||||||||||||||||||||
Sbjct  832   TTAGCTGCGCCACTAAAAGCTCAAGGCTTCCAACGGCTAGTTGACATCGTTTACGGCGTG  773

Query  669   GACTACCAGGGTATCTAATCCTGTTTGCTCCCCACGCTTTCGCACCTCAGTGTCAGTATT  728
             ||||||||||||||||||||||||||||||||||||||||||||||||||||||||||||
Sbjct  772   GACTACCAGGGTATCTAATCCTGTTTGCTCCCCACGCTTTCGCACCTCAGTGTCAGTATT  713

Query  729   AGTCCAGGTGGTCGCCTTCGCCACTGGTGTTCCTTCCTATATCTACGCATTTCACCGCTA  788
             ||||||||||||||||||||||||||||||||||||||||||||||||||||||||||||
Sbjct  712   AGTCCAGGTGGTCGCCTTCGCCACTGGTGTTCCTTCCTATATCTACGCATTTCACCGCTA  653

Query  789   CACAGGAAATTCCACCACCCTCTACCATACTCTAGTCAGTCAGTTTTGAATGCAGTTCCC  848
             ||||||||||||||||||||||||||||||||||||||||||||||||||||||||||||
Sbjct  652   CACAGGAAATTCCACCACCCTCTACCATACTCTAGTCAGTCAGTTTTGAATGCAGTTCCC  593

Query  849   AGGTTGAGCCCGGGGATTTCACATCCAACTTAACAAACCACCTACGCGCGCTTTACGCCC  908
             ||||||||||||||||||||||||||||||||||||||||||||||||||||||||||||
Sbjct  592   AGGTTGAGCCCGGGGATTTCACATCCAACTTAACAAACCACCTACGCGCGCTTTACGCCC  533

Query  909   AGTAATTCCGATTAACGCTTGCACCCTCTGTATTACCGCGGCTGCTGGCACAGAGTTAGC  968
             ||||||||||||||||||||||||||||||||||||||||||||||||||||||||||||
Sbjct  532   AGTAATTCCGATTAACGCTTGCACCCTCTGTATTACCGCGGCTGCTGGCACAGAGTTAGC  473

Query  969   CGGTGCTTATTCTGTCGGTAACGTCAAAACAATCACGTATTAGGTAACTGCCCTTCCTCC  1028
             ||||||||||||||||||||||||||||||||||||||||||||||||||||||||||||
Sbjct  472   CGGTGCTTATTCTGTCGGTAACGTCAAAACAATCACGTATTAGGTAACTGCCCTTCCTCC  413

Query  1029  CAACTTAAAGTGCTTTACAATCCGA  1053
             |||||||||||||||||||||||||
Sbjct  412   CAACTTAAAGTGCTTTACAATCCGA  388
```

Download

FASTA (complete sequence)

FASTA (aligned sequences)

GenBank (complete sequence)

Continue
Cancel

GenBankGraphics

Next
Previous
Descriptions

Pseudomonas trivialis partial 16S rRNA gene, type strain DSM 14937T

Sequence ID: AJ492831.1Length: 1529Number of Matches: 1

Related Information

Range 1: 408 to 1452GenBankGraphics

Next Match
Previous Match
First Match

Alignment statistics for match #1

| Score | Expect | Identities | Gaps | Strand | Frame |
| --- | --- | --- | --- | --- | --- |
| 1927 bits(1043) | 0.0() | 1044/1045(99%) | 0/1045(0%) | Plus/Minus |  |

Features:

```
Query  9     AACCGTCCTCCCGAAGGTTAGACTAGCTACTTCTGGTGCANCCCACTCCCATGGTGTGAC  68
             |||||||||||||||||||||||||||||||||||||||| |||||||||||||||||||
Sbjct  1452  AACCGTCCTCCCGAAGGTTAGACTAGCTACTTCTGGTGCAACCCACTCCCATGGTGTGAC  1393

Query  69    GGGCGGTGTGTACAAGGCCCGGGAACGTATTCACCGCGACATTCTGATTCGCGATTACTA  128
             ||||||||||||||||||||||||||||||||||||||||||||||||||||||||||||
Sbjct  1392  GGGCGGTGTGTACAAGGCCCGGGAACGTATTCACCGCGACATTCTGATTCGCGATTACTA  1333

Query  129   GCGATTCCGACTTCACGCAGTCGAGTTGCAGACTGCGATCCGGACTACGATCGGTTTTGT  188
             ||||||||||||||||||||||||||||||||||||||||||||||||||||||||||||
Sbjct  1332  GCGATTCCGACTTCACGCAGTCGAGTTGCAGACTGCGATCCGGACTACGATCGGTTTTGT  1273

Query  189   GGGATTAGCTCCACCTCGCGGCTTGGCAACCCTCTGTACCGACCATTGTAGCACGTGTGT  248
             ||||||||||||||||||||||||||||||||||||||||||||||||||||||||||||
Sbjct  1272  GGGATTAGCTCCACCTCGCGGCTTGGCAACCCTCTGTACCGACCATTGTAGCACGTGTGT  1213

Query  249   AGCCCAGGCCGTAAGGGCCATGATGACTTGACGTCATCCCCACCTTCCTCCGGTTTGTCA  308
             ||||||||||||||||||||||||||||||||||||||||||||||||||||||||||||
Sbjct  1212  AGCCCAGGCCGTAAGGGCCATGATGACTTGACGTCATCCCCACCTTCCTCCGGTTTGTCA  1153

Query  309   CCGGCAGTCTCCTTAGAGTGCCCACCATTACGTGCTGGTAACTAAGGACAAGGGTTGCGC  368
             ||||||||||||||||||||||||||||||||||||||||||||||||||||||||||||
Sbjct  1152  CCGGCAGTCTCCTTAGAGTGCCCACCATTACGTGCTGGTAACTAAGGACAAGGGTTGCGC  1093

Query  369   TCGTTACGGGACTTAACCCAACATCTCACGACACGAGCTGACGACAGCCATGCAGCACCT  428
             ||||||||||||||||||||||||||||||||||||||||||||||||||||||||||||
Sbjct  1092  TCGTTACGGGACTTAACCCAACATCTCACGACACGAGCTGACGACAGCCATGCAGCACCT  1033

Query  429   GTCTCAATGTTCCCGAAGGCACCAATCTATCTCTAGAAAGTTCATTGGATGTCAAGGCCT  488
             ||||||||||||||||||||||||||||||||||||||||||||||||||||||||||||
Sbjct  1032  GTCTCAATGTTCCCGAAGGCACCAATCTATCTCTAGAAAGTTCATTGGATGTCAAGGCCT  973

Query  489   GGTAAGGTTCTTCGCGTTGCTTCGAATTAAACCACATGCTCCACCGCTTGTGCGGGCCCC  548
             ||||||||||||||||||||||||||||||||||||||||||||||||||||||||||||
Sbjct  972   GGTAAGGTTCTTCGCGTTGCTTCGAATTAAACCACATGCTCCACCGCTTGTGCGGGCCCC  913

Query  549   CGTCAATTCATTTGAGTTTTAACCTTGCGGCCGTACTCCCCAGGCGGTCAACTTAATGCG  608
             ||||||||||||||||||||||||||||||||||||||||||||||||||||||||||||
Sbjct  912   CGTCAATTCATTTGAGTTTTAACCTTGCGGCCGTACTCCCCAGGCGGTCAACTTAATGCG  853

Query  609   TTAGCTGCGCCACTAAAAGCTCAAGGCTTCCAACGGCTAGTTGACATCGTTTACGGCGTG  668
             ||||||||||||||||||||||||||||||||||||||||||||||||||||||||||||
Sbjct  852   TTAGCTGCGCCACTAAAAGCTCAAGGCTTCCAACGGCTAGTTGACATCGTTTACGGCGTG  793

Query  669   GACTACCAGGGTATCTAATCCTGTTTGCTCCCCACGCTTTCGCACCTCAGTGTCAGTATT  728
             ||||||||||||||||||||||||||||||||||||||||||||||||||||||||||||
Sbjct  792   GACTACCAGGGTATCTAATCCTGTTTGCTCCCCACGCTTTCGCACCTCAGTGTCAGTATT  733

Query  729   AGTCCAGGTGGTCGCCTTCGCCACTGGTGTTCCTTCCTATATCTACGCATTTCACCGCTA  788
             ||||||||||||||||||||||||||||||||||||||||||||||||||||||||||||
Sbjct  732   AGTCCAGGTGGTCGCCTTCGCCACTGGTGTTCCTTCCTATATCTACGCATTTCACCGCTA  673

Query  789   CACAGGAAATTCCACCACCCTCTACCATACTCTAGTCAGTCAGTTTTGAATGCAGTTCCC  848
             ||||||||||||||||||||||||||||||||||||||||||||||||||||||||||||
Sbjct  672   CACAGGAAATTCCACCACCCTCTACCATACTCTAGTCAGTCAGTTTTGAATGCAGTTCCC  613

Query  849   AGGTTGAGCCCGGGGATTTCACATCCAACTTAACAAACCACCTACGCGCGCTTTACGCCC  908
             ||||||||||||||||||||||||||||||||||||||||||||||||||||||||||||
Sbjct  612   AGGTTGAGCCCGGGGATTTCACATCCAACTTAACAAACCACCTACGCGCGCTTTACGCCC  553

Query  909   AGTAATTCCGATTAACGCTTGCACCCTCTGTATTACCGCGGCTGCTGGCACAGAGTTAGC  968
             ||||||||||||||||||||||||||||||||||||||||||||||||||||||||||||
Sbjct  552   AGTAATTCCGATTAACGCTTGCACCCTCTGTATTACCGCGGCTGCTGGCACAGAGTTAGC  493

Query  969   CGGTGCTTATTCTGTCGGTAACGTCAAAACAATCACGTATTAGGTAACTGCCCTTCCTCC  1028
             ||||||||||||||||||||||||||||||||||||||||||||||||||||||||||||
Sbjct  492   CGGTGCTTATTCTGTCGGTAACGTCAAAACAATCACGTATTAGGTAACTGCCCTTCCTCC  433

Query  1029  CAACTTAAAGTGCTTTACAATCCGA  1053
             |||||||||||||||||||||||||
Sbjct  432   CAACTTAAAGTGCTTTACAATCCGA  408
```


BLAST is a registered trademark of the National Library of Medicine

Support center
Mailing list


YouTube

- National Library Of Medicine
- National Institutes Of Health
- U.S. Department of Health & Human Services
- USA.gov

### NCBI


National Center for Biotechnology Information,
 U.S. National Library of Medicine

8600 Rockville Pike,
Bethesda
 MD,
20894
USA

Policies and Guidelines
|
Contact


PreferencesTurn off

External link. Please review our privacy policy.
